# A gut microbiome signature for cirrhosis due to nonalcoholic fatty liver disease

**DOI:** 10.1038/s41467-019-09455-9

**Published:** 2019-03-29

**Authors:** Cyrielle Caussy, Anupriya Tripathi, Greg Humphrey, Shirin Bassirian, Seema Singh, Claire Faulkner, Ricki Bettencourt, Emily Rizo, Lisa Richards, Zhenjiang Z. Xu, Michael R. Downes, Ronald M. Evans, David A. Brenner, Claude B. Sirlin, Rob Knight, Rohit Loomba

**Affiliations:** 1 NAFLD Research Center, Department of Medicine, University of California San Diego, La Jolla, CA 92093 USA; 2 Université Lyon 1, Hospices Civils de Lyon, Lyon, 69002 France; 3 Center for Microbiome Innovation, University of California San Diego, La Jolla, CA 92093 USA; 4 Department of Pediatrics, University of California San Diego, La Jolla, CA 92093 USA; 5 Division of Biological Sciences, University of California San Diego, La Jolla, CA 92093 USA; 6 Skaggs School of Pharmacy and Pharmaceutical Sciences, University of California San Diego, La Jolla, CA 93093 USA; 7 Division of Epidemiology, Department of Family and Preventive Medicine, University of California San Diego, La Jolla, CA 92093 USA; 8 Salk Institute for Biological Studies, La Jolla, CA 92037 USA; 9 Division of Gastroenterology, Department of Medicine, University of California San Diego, La Jolla, CA 92093 USA; 10 Liver Imaging Group, Department of Radiology, University of California at San Diego, La Jolla, CA 92037 USA; 11 Department of Computer Science and Engineering, University of California San Diego, La Jolla, CA 92093 USA; 12 Department of BioEngineering, University of California San Diego, La Jolla, CA 92093 USA

## Abstract

The presence of cirrhosis in nonalcoholic-fatty-liver-disease (NAFLD) is the most important predictor of liver-related mortality. Limited data exist concerning the diagnostic accuracy of gut-microbiome-derived signatures for detecting NAFLD-cirrhosis. Here we report 16S gut-microbiome compositions of 203 uniquely well-characterized participants from a prospective twin and family cohort, including 98 probands encompassing the entire spectrum of NAFLD and 105 of their first-degree relatives, assessed by advanced magnetic-resonance-imaging. We show strong familial correlation of gut-microbiome profiles, driven by shared housing. We report a panel of 30 features, including 27 bacterial features with discriminatory ability to detect NAFLD-cirrhosis using a Random Forest classifier model. In a derivation cohort of probands, the model has a robust diagnostic accuracy (AUROC of 0.92) for detecting NAFLD-cirrhosis, confirmed in a validation cohort of relatives of proband with NAFLD-cirrhosis (AUROC of 0.87). This study provides evidence for a fecal-microbiome-derived signature to detect NAFLD-cirrhosis.

## Abstract (web summary)

Development of cirrhosis in individuals with non-alcoholic fatty liver disease can predict mortality. Here the authors used a unique twin and family cohort to identify a gut microbiome-derived 16sRNA signature that can detect cirrhosis in individuals with non-alcoholic fatty liver disease.

## Introduction

Nonalcoholic fatty liver disease (NAFLD) is the most prevalent cause of chronic liver disease worldwide^1,2^ and yet remains largely underdiagnosed even in individuals with advanced stage of the disease^3^. NAFLD-cirrhosis represents the most severe stage of the disease, carries a significant risk of hepatocellular carcinoma (HCC), and is consistently identified as the most important predictor of liver-related morbidity-mortality in NAFLD^4,5^. Hence, the most important clinical challenge for the field remains to identify the high-risk populations of advanced NAFLD and to determine the optimal strategy for their screening using accurate, non-invasive, widely available and easy-to-perform screening test applicable at the level of these high-risk populations.

Over the last decade, the gut-liver axis has emerged as a pivotal component of NAFLD^6–11^ and represents a potential source of non-invasive biomarkers for the detection and stage of liver disease^6,7,12,13^. Limited data are available regarding the diagnostic accuracy of a stool microbiome-derived signature for detecting NAFLD-cirrhosis especially among high-risk populations.

We previously demonstrated that first-degree relatives of probands with NAFLD-cirrhosis have a high risk of advanced-fibrosis (AF)^14^. However, factors associated with progression towards NAFLD-cirrhosis among families remain obscure. Although earlier studies reported familial aggregation of NAFLD and NAFLD-related cirrhosis^15–19^, and demonstrated that both liver steatosis and fibrosis are heritable^20,21^, known genetic risk only accounts for ~10–30% of the variance observed in NAFLD^22–25^. This suggests an additional role for environmental factors, which predominate over genetic factors in shaping the human gut-microbiome^26–28^. Heritability of gut-microbiome features has been reported in twins studies^26,27^, but limited data exist regarding the similarity of gut-microbiome composition among family members, and whether similar microbiomes associate with disease traits especially in the entire spectrum of NAFLD including NAFLD-cirrhosis. Finally, the ability of a gut-microbiome-derived signature for the non-invasive screening of advanced fibrosis among a high-risk population such as first-degree relatives of probands with NAFLD-cirrhosis is unknown.

Therefore we study the stool microbiome of a unique twin and family cohort including well-characterized and prospectively recruited participants with and without NAFLD including stool samples collection and assessed using MRI proton density fat fraction (MRI-PDFF) for quantifying hepatic steatosis^29^ and MR-elastography (MRE) for quantifying liver fibrosis^30–33^. Our aim is to examine the familial similarity of gut-microbiome composition and to test whether a non-invasive stool-microbiome-derived signature accurately detects NAFLD-cirrhosis.

## Results

### Baseline characteristics of study population

This cross-sectional analysis included 203 well-characterized, prospectively recruited participants, encompassing the entire spectrum of NAFLD divided into three groups (NAFLD-cirrhosis, NAFLD without AF, non-NAFLD controls) paired with their first-degree relatives. Subjects included 26 probands with NAFLD-cirrhosis and 37 of their first-degree relatives, 18 probands with NAFLD (MRI-PDDF ≥ 5%) without AF (MRE < 3.63 kPa) and 17 of their first-degree relatives, and 54 non-NAFLD normal controls (MRI-PDFF < 5%) and 44 of their first-degree relatives. The detailed derivation of the study cohort is shown in Supplementary Fig. 1. The detailed demographic, biochemical, imaging data of the different groups are provided in Supplementary Tables 1 and 2.

### Significant familial correlation of the gut-microbiome composition

**Fig. 1:**
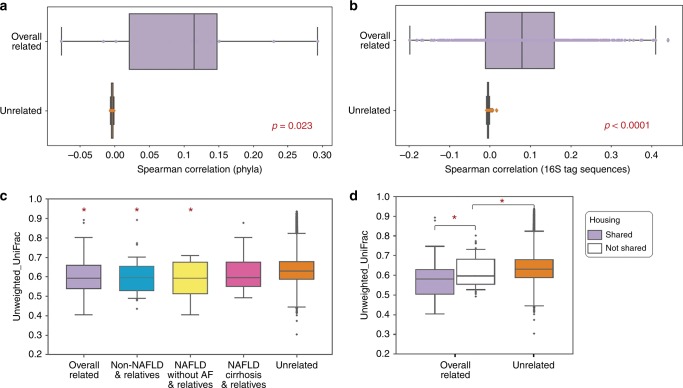
Familial association and shared microbiome among relatives is driven by shared housing. Distribution of spearman correlation coefficients between relatives in purple (*n* = 86) and unrelated pairs in orange (*n* = 18232) in the familial cohort, plotted for each phyla (**a**) and 16S tag sequences (**b**). The box plots show the quartiles and whiskers show the rest of the distribution (1.5 inter-quartile range) and the center line corresponds to the median. This analysis was done after filtering rare 16S sequences to avoid spurious correlations due to sparsity (total abundance <10E-6 across all samples in each disease group). The correlation among related individuals was significantly higher at both phylum (*p* = 0.023) and 16S tag sequences (*p* = 2.4E-41) levels. Similar plot showing the distribution of unweighted UniFrac distances between related in purple and unrelated pairs stratified by disease status (**c**). The beta-diversity was significantly lower among related individuals (*p* = 3.22E-05), non-NAFLD controls and relative in blue (*n* = 38 pairs) (*p* = 0.0011) and probands with NAFLD without AF and relatives in yellow (*n* = 15) (*p* = 0.0156) when compared to the same among unrelated pairs in orange, while the difference between NALFD-cirrhosis patients and relatives in pink (*n* = 33) and unrelated pairs in orange was not statistically significant (*p* > 0.1). When stratified by shared housing (**d**), beta-diversity was significantly lower among related individuals sharing a house in purple (*n* = 35 pairs) (*p* = 0.0455). Additionally, related individuals not sharing a house in white (*n* = 51 pairs) had significantly lower beta-diversity compared to unrelated pairs in orange (*p* = 0.028). All *p* value were determined by two-sided Kruskal-Wallis test. *p value < 0.05. Source data are provided as a Source Data file

The gut-microbiome profile showed significant correlation within biologically related pairs compared to random-unrelated pairs at the level of the phyla (*p* = 0.023) Kruskal-Wallis test. Fig. 1a and at the level of exact 16S sequences of bacterial strains in the gut microbiome (*p* = 2.4E-41), Kruskal-Wallis test Fig. 1b. In our analyses at the phylum level, this familial correlation was mainly driven by significant correlation of Bacteroidetes (*r* = 0.22, *p* = 0.01) and Actinobacteria (*r* = 0.29, *p* = 0.002), spearman correlation, between related individuals. Furthermore, phylogenetic dissimilarity assessed by unweighted UniFrac distances among biologically related pairs was significantly lower than in random-unrelated pairs (*p* = 3.0E-05) Kruskall–Wallis test. When stratified by the liver phenotype of the proband, the phylogenetic dissimilarity remained significantly lower among non-NAFLD controls and relatives (*p* = 0.001) and probands with NAFLD without AF and relatives (*p* = 0.015) compared to unrelated pairs, while no significant difference was observed among probands with NAFLD-cirrhosis and relatives (*p* = 0.107), Kruskal-Wallis test Fig. 1c. These results remained consistent when related pairs were stratified by monozygotic status. These results suggest that familial gut-microbiome similarities are independent of mild/moderate liver phenotype but are impacted by severe stage of liver disease. Finally, related individuals with shared-housing had a lower phylogenetic dissimilarity than those who did not share housing (*p* = 0.045), Kruskal-Wallis test Fig. 1d.

### The gut-microbiome profile across NAFLD features

**Fig. 2:**
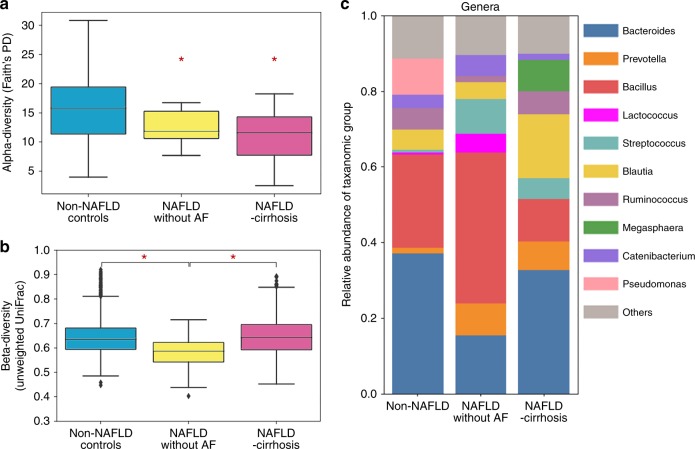
Gut microbiome alteration in NAFLD-cirrhosis. Comparison between non-NAFLD controls in blue (*n* = 51), NAFLD without advanced fibrosis in yellow (*n* = 17), and NAFLD-cirrhosis probands in pink (*n* = 25) with respect to **a** alpha-diversity using Faith’s Phylogenetic Diversity. Non-NAFLD controls have significantly higher alpha-diversity compared to probands with NAFLD without AF in yellow (*p* = 0.0163) and NAFLD-cirrhosis in pink (*p* = 0.0020). **b** Similar plot for beta-diversity using unweighted UniFrac distance metric. The beta-diversity among probands with NAFLD without AF in yellow was significantly lower than that among non-NAFLD controls in blue (*p* = 1.14E-18) and probands with NAFLD-cirrhosis in pink (*p* = 3.32E-15). The box plots show the quartiles and whiskers show the rest of the distribution (1.5 inter-quartile range) the center liner corresponds to the median. **c** Gut microbiome composition of non-NAFLD controls, NAFLD without AF and NALFD-cirrhosis probands shows differences at bacterial genus level. All *p* value were determined by two-sided Kruskal-Wallis test. *p value < 0.05. Source data are provided as a Source Data file

The gut-microbiome profile was first assessed in a derivation cohort including the 3 groups of probands encompassing the entire spectrum of NAFLD. As shown in previous studies^12,13^, α-diversity as measured by Faith’s phylogenetic diversity decreased with increase in liver damage severity Fig. 2a. The β-diversity (unweighted UniFrac distances) was lower among individuals with moderate liver damage (NAFLD without AF) compared to non-NAFLD controls (*p* = 1.1 E-18, Kruskal-Wallis test), whereas it was higher among individuals with severe liver damage (NAFLD-cirrhosis) compared to probands with moderate liver damage (NAFLD without AF) (*p* = 3.3E-15, Kruskal-Wallis test) Fig. 2b. This suggests an hourglass signature of disease severity in the gut-microbiome, with an initial decrease in phylogenetic diversity associated with a moderate stage of the disease that progress towards a phylogenetic dispersion in individuals with severe stages of disease such as NAFLD-cirrhosis.

### Gut-microbiome taxa alteration across the spectrum of NAFLD

At the genus level, both NAFLD-cirrhosis and NAFLD without AF group were enriched with *Streptococcus* but only the NAFLD-cirrhosis group was enriched with *Megasphaera*. In addition, *Bacillus* and *Lactococcus* were enriched in both non-NAFLD controls and NAFLD without AF whereas *Pseudomonas* was enriched only in the non-NAFLD controls Fig. 2c. Species belonging to the family Enterobacteriaceae and the genera *Streptococcus* and *Gallibacterium* were the most enriched in NAFLD-cirrhosis, while *Faecalibacterium prausnitzii* and species belonging to the genus, *Catenibacterium* and the families Rikenellaceae, Mogibacterium, Peptostreptococcaceae were enriched in non-NAFLD controls. These results are consistent with the study performed by Ponziani and colleagues in an Italian cohort showing higher Enterobacteriaceae and *Streptococcus* in NAFLD-cirrhosis with and without HCC^10^. In addition, it confirms a shift towards more Gram-negative microbes in advanced fibrosis stages, as previously reported in NAFLD^6,8,10^.

### A stool-microbiome signature accurately detects NAFLD-cirrhosis

**Fig. 3:**
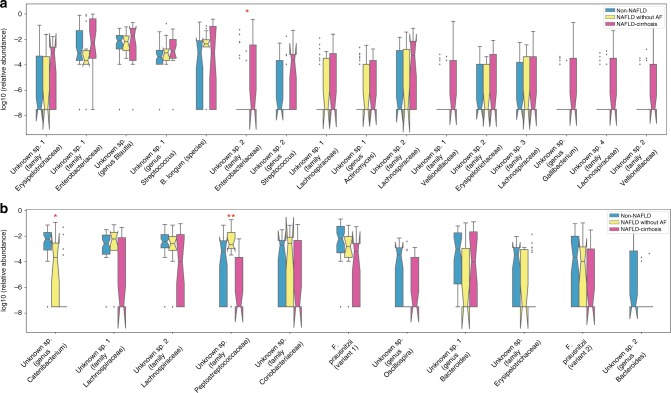
Relative abundance of predictive microbial features for the prediction of NAFLD-cirrhosis. The bacterial features most predictive of NAFLD-cirrhosis (*n* = 25) versus non-NAFLD controls (*n* = 51) sorted by decreasing importance score in Random Forest classification model. Features increased (**a**) and decreased (**b**) in NAFLD-cirrhosis probands are shown. Relative abundance is plotted for each subject group in the cohort (NAFLD-cirrhosis in pink (*n* = 25), NAFLD without advanced fibrosis in yellow (*n* = 17) and non-NAFLD controls in blue (*n* = 51)). The feature table is normalized to a total abundance of 1 per sample and relative abundances are plotted on a log10 scale. Each bacterial feature is a unique 16S tag sequence labeled to the highest possible taxonomic rank assigned using QIIME. The box plots show the quartiles and whiskers show the rest of the distribution (1.5 inter-quartile range). The notches show 95% confidence interval. Features that are differentially abundant in addition to being important predictors are marked by asterisk. Single asterisk (*) represents significant difference between non-NAFLD controls and NAFLD-cirrhosis probands *p* value < 0.05; Double asterisk (**) represents the same between NAFLD without advanced fibrosis and NAFLD cirrhosis probands *p* value < 0.05. Differential abundance was tested using permutation-based, ranked mean test, comparing mean difference between the two groups^63^. FDR (<0.1) was controlled using DS-FDR method^64^. Source data are provided as a Source Data file

**Fig. 4:**
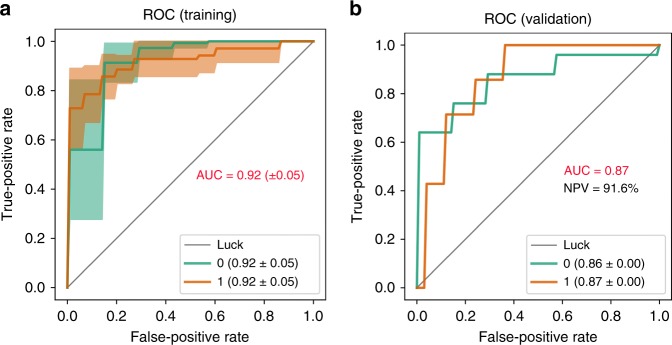
High diagnostic accuracy of a gut-microbiome signature for the detection of NAFLD-cirrhosis. Receiver operating characteristic (ROC) curves evaluating ability to predict advanced Fibrosis using Random Forest classification. Each curve represents the sensitivity and specificity to distinguish subjects with NAFLD-cirrhosis (1, brown line) from non-NALFD controls (0, green line). **a** Mean ROC curve from cross-validation within training data comprised of NAFLD-cirrhosis probands (*n* = 24) and non-NAFLD controls (*n* = 47). Cross-validation was performed by iteratively (10 times) training the Random Forest model with 70:30 train/test split on this training data. **b** ROC curve representing diagnostic accuracy of Random Forest classification model tested on first-degree relatives of NAFLD-cirrhosis probands (*n* = 32). The negative predictive value (NPV) of the model was 91.6% and the positive predictive value was 62.5%. Source data are provided as a Source Data file

A Random Forest model comprised of 30 features (including 27 bacterial features and age, sex and body mass index (BMI)) identifies probands with NAFLD-cirrhosis. The bacterial features most important for predicting NAFLD-cirrhosis and their relative abundances in non-NAFLD controls, NAFLD without AF and NAFLD-cirrhosis are shown in Fig. 3. In a derivation cohort of probands, the model had a robust diagnostic accuracy, with an AUROC of 0.92 after cross-validation for detecting NAFLD-cirrhosis Fig. 4a. The diagnostic accuracy of the model was then confirmed in a validation cohort of first-degree relatives of proband with NAFLD-cirrhosis with a good diagnostic accuracy, with an AUROC of 0.87 for the detection of advanced fibrosis with a high negative predictive value of 91.6% Fig. 4b. We performed sensitivity analyses by adjusting the prediction model for the presence of type 2 diabetes and the findings did not differ with an AUROC of 0.87. In addition, we performed sensitivity analyses in another validation group enriched with mild to moderate stage of NAFLD including probands with NAFLD without AF. The diagnostic accuracy of the model was confirmed and yielded a very good diagnostic accuracy with an AUROC of 86% Supplementary Fig. 2.

## Discussion

Here we report, using a unique twin and family study design, including well-characterized participants with and without NAFLD, a strong familial similarity of the gut-microbiome among first-degree relatives independent of mild/moderate liver phenotype and involving shared housing. These results confirm a strong impact of the environment in the familial similarity of the gut-microbiome^26–28^. This study builds on the seminal study by Song et al. demonstrating that couples sharing the same housing share microbiota with one another, providing additional evidence of a shared gut-microbiome profile among biologically related individuals^34^. In addition, these results confirm a strong impact of the environment in the familial similarity of the gut-microbiome^26–28^ independent of liver phenotype and demonstrate that shared-housing is a major determinant that should be controlled for in study designs assessing the microbiome in liver disease. In line with previous studies^12,13^, we observed an initial decrease in both alpha and beta diversity in the moderate stage of NAFLD. Interestingly, we observed a decrease in alpha-diversity and increase in beta-diversity in severe stage of disease (NAFLD-cirrhosis) compared to NAFLD without AF. This suggests that the extreme form of disease is characterized by a less-diverse but also significantly less stable microbiome. These findings are in line with the Anna Karenina principle and suggest that disease-linked changes in the microbiome are likely stochastic, leading to community instability^35–37^. However, further investigations with larger sample sizes are needed to determine whether this phylogenetic dispersion reflects a distinct profile among NAFLD-cirrhotic patients, and whether it is associated with specific NAFLD-cirrhosis related outcomes.

Finally, we identified a specific stool-microbiome-derived signature of NAFLD-cirrhosis that yielded a robust diagnostic accuracy for the detection of NAFLD-cirrhosis. Hence, this conveniently assessed microbial biomarker could present an adjunct tool to current invasive approaches to determine stage of liver disease.

We previously demonstrated that a microbial biomarker can detect AF in biopsy-proven NAFLD^6^. This study leverages from a unique and well-characterized cohort including participants with NAFLD-cirrhosis which allows studying the specific gut-microbiome signature of this extreme and well-defined clinical phenotype. The fundamental difference between the previous study and the present study is the clinical context of use of the gut-microbiome signature. In the present study, the clinical question is to accurately differentiate using a non-invasive gut-microbiome signature who among the first-degree relatives have advanced form of NAFLD and who are unaffected in a general setting as opposed to a liver clinic setting. We acknowledge that several non-invasive methods are currently available for the diagnosis of cirrhosis including MRE, ultrasound-based vibration controlled transient elastography (VCTE), laboratory tests and clinical prediction rules in the setting of liver clinics. However, the context of use is critical for biomarker development as suggested by the BEST Guidelines by FDA^38^. Indeed, recent reports suggest that the application of the current standard developed from NAFLD cohorts may perform poorly in other high-risk populations such as obese or type 2 diabetic individuals^39,40^. In order to address this clinically important question, this study leverage from 2 distinct levels of innovations. First, its study design as this innovative study leverages from a unique prospectively recruited case-control study design. This cross-sectional analysis included 203 well-characterized participants, encompassing the entire spectrum of NAFLD divided into three groups (NAFLD-cirrhosis, NAFLD without advanced fibrosis, non-NAFLD controls) paired with their first-degree relatives. Secondly, the discovery of shared housing effect: the familial cohort design enabled us to discover the effect of shared housing on the gut-microbiome signature related to NAFLD-cirrhosis. This unique familial cohort study design led us to a the discovery that shared housing had a dominant effect on microbiome. This effect would not be apparent from previous study in NAFLD among unrelated individuals.

We acknowledge the following limitations of this study. This is a single-center study performed in a center with expertise in clinical investigation of NAFLD with advanced MRI-based phenotyping, and the generalizability of the findings in other clinical settings remains to be established. 16S rRNA sequencing may not have capture additional insights associated with the disease status available at the species or strain level. In addition, due to the single-center design, the generalization of the gut-microbiome signature in population from other geographical locations is unknown^41^. Finally, the association does not suggest causality, and additional studies are needed to assess whether these microbial species impact gut permeability and/or induce NAFLD progression through cross-talk between serum metabolites and the liver^7,11,42,43^. However, the strengths of the study include a prospective study design, detailed phenotyping of participants using the most accurate non-invasive imaging modalities available, and assessment of accuracy using AUROC in both a *derivation* and *validation* cohort. Further multi-center studies including larger number of individuals from diverse geographical origin are needed to validate the clinical utility and applicability of the proposed microbiome-derived signature to detect NAFLD-cirrhosis in high-risk population for advanced stage of NAFLD.

## Methods

### Study design

This is a cross-sectional analysis of a prospective family cohort study of participants from the Familial Cirrhosis cohort and Twins and Family cohort who were participating in a biobank initiative and prospectively recruited at the University of California at San Diego (UCSD) NAFLD Research Center between December 2011 and December 2017. All participants underwent a standardized exhaustive clinical research visit including detailed medical history, physical examination, and testing to rule out other causes of chronic liver diseases (see inclusion and exclusion criteria for further details), fasting laboratory tests at the University of California at San Diego (UCSD) NAFLD Research Center^14,20,21,44^. Participants also underwent an advanced magnetic resonance examination including magnetic resonance imaging proton-density-fat-fraction (MRI-PDFF) and magnetic resonance elastography (MRE) at the UCSD MR3T Research Laboratory for the screening of NAFLD and advanced fibrosis^30–32^. Participants from the Familial Cirrhosis cohort also underwent an ultrasound-based vibration controlled transient elastography (VCTE) assessment using a FibroScan. At the time of each research visit, patients provided stool samples. These were collected and immediately stored in a −80 °C freezer. Written informed consent was obtained from every participant.

### Probands with NAFLD-cirrhosis and first-degree relatives

This study included 26 probands with NAFLD-cirrhosis and 37 of their first-degree relatives from the Familial Cirrhosis cohort prospectively recruited at the UCSD NAFLD Research Center^14^. Probands with NAFLD-cirrhosis had a documented evidence of NAFLD with either biopsy-proven or meeting imaging criteria for cirrhosis. Definition for NAFLD was based upon American Association for the Study of Liver Study (AASLD) Practice Guidelines^45^. The study complies with all relevant ethical regulations for work with human participants and was approved by the UCSD Institutional Review Board number 140084. Written informed consent was obtained from all participant.

Probands and first-degree relatives had to be at least 18 years old. Probands were required to have documented diagnosis of NAFLD-cirrhosis either by liver biopsy or by documented imaging evidence by a protocol-specified criterion. First-degree relatives (sibling, child, or parent) with written informed consent who did not meet any exclusion criteria were included in the study.

Exclusion criteria included the following: regular and excessive alcohol consumption within 2 years of recruitment (≥14 drinks/week for men or ≥7 drinks/week for women); use of hepatotoxic drugs or drugs known to cause hepatic steatosis; evidence of liver diseases other than NAFLD, including viral hepatitis (detected with positive serum hepatitis B surface antigen or hepatitis C viral RNA), Wilson’s disease, hemochromatosis, alpha-1 antitrypsin deficiency, autoimmune hepatitis, and cholestatic or vascular liver disease; clinical or laboratory evidence of chronic illnesses associated with hepatic steatosis, including human immunodeficiency virus infection (HIV), celiac disease, cystic fibrosis, lipodystrophy, dysbetalipoproteinemia, and glycogen storage diseases; evidence of active substance abuse, significant systemic illnesses, contraindication(s) to MRI, pregnant or trying to become pregnant, or any other condition which, in the investigator’s opinion, may affect the patient’s competence or compliance in completing the study.

### Control Proband and first-degree relatives

The study included 140 participants from the Twin and Family study corresponding to 100 twins (50 twin-pairs; 30 monozygotic twin-pairs, 20 dizygotic twin-pairs) and 40 siblings or parents-offspring. The non-NAFLD controls included 54 probands and 44 first-degree relatives and the group with NAFLD without AF included 18 probands and 17 first-degree relatives of community-dwelling controls either twin, sib-sib or parent-offspring pairs^14,20,44^. These twin, sib-sib, and parent-offspring pairs were prospectively recruited and they reside in southern California. Twins without evidence of NAFLD (MRI-PDFF < 5%) and advanced fibrosis (MRE < 3.63 kPa) were considered as non-NAFLD control and twins with evidence of NAFLD (MRI-PDFF ≥ 5%) without evidence of advanced fibrosis (MRE < 3.63 kPa) and their twin pair were randomly assigned as proband or first-degree relatives. The study complies with all relevant ethical regulations for work with human participants and was approved by the UCSD Institutional Review Board number 111282. Written informed consent was obtained from all participant.

Patients were included if they were twins, siblings or parent-offspring at least 18 years old, willing and able to complete all research procedures and observations. For each twin pair, a detailed assessment of twin-ship status (ie, monozygotic (MZ) or dizygotic (DZ)) was obtained. The majority of twin-pairs (34) were diagnosed by their physician as either MZ or DZ by genetic testing. Furthermore, twin-ship status was confirmed by using a previously published questionnaire^20,21^.

Participants were excluded from the study if they met any of the following criteria: significant alcohol intake (>10 g/day in females or >20 g/day in males) for at least 3 consecutive months over the previous 12 months or if the quantity of alcohol consumed could not be reliably ascertained; clinical or biochemical evidence of liver diseases other than NAFLD (eg, viral hepatitis, HIV, coeliac disease, cystic fibrosis, autoimmune hepatitis); metabolic and/or genetic liver disease (eg, Wilson’s disease, haemochromatosis, polycystic liver disease, alpha-1-antitrypsin deficiency, dysbetalipoproteinaemia); clinical or laboratory evidence of systemic infection or any other clinical evidence of liver disease associated with hepatic steatosis; use of drugs known to cause hepatic steatosis (eg amiodarone, glucocorticoids, methotrexate, L-asparaginase and valproic acid) for at least 3 months in the last past 6 months; history of bariatric surgery; presence of systemic infectious illnesses; females who were pregnant or nursing at the time of the study; contraindications to MRI (eg metal implants, severe claustrophobia, body circumference greater than the imaging chamber); any other condition(s) which, based on the principal investigator’s opinion, may significantly affect the participant’s compliance, competence, or ability to complete the study.

### Clinical assessments and laboratory test

All participants underwent a standardized clinical research visit at the UCSD NAFLD Research Center. A detailed history was obtained from all participants. A physical exam, which included vital signs, height, weight, and anthropometric measurements, was performed by a trained clinical investigator. Body mass index was defined as the body weight (in kilograms) divided by height (in meters) squared. Alcohol consumption was documented outside clinical visits and confirmed in the research clinic using the Alcohol Use Disorders Identifications Test and the Skinner questionnaire. A detailed history of medications was obtained and no patient took medications known or suspected to cause steatosis or steatohepatitis. Other causes of liver disease and secondary causes of hepatic steatosis were systemically ruled out using detailed history and laboratory data. After completion of the earlier described elements of the history and physical examination, participants had comprehensive fasting laboratory including metabolic and liver assessment^14,20,21,44,46^.

### MRI-PDFF assessment

MRI was performed at the UCSD MR3T Research Laboratory using the 3T research scanner (GE Signa EXCITE HDxt; GE Healthcare, Waukesha, WI) with all participants in the supine position. MRI-PDFF was used to measure hepatic fat content and MRE was used to measure liver fibrosis. It acquires multiple echo sequences at different times when fat and water signals are nominally in phase or out of phase with each other. Data from each echo time are passed into an algorithm that estimates and corrects T2* effects, models the fat signal as a superposition of multiple frequency components, and estimates fat and water proton densities from which the fat content is calculated. A magnitude‐based technique was applied to echo sequences to avoid phase errors, which can adversely affect fat quantification. This algorithm is applied to the source images using custom analysis software developed at the UCSD Liver Imaging Group to generate a PDFF parametric map depicting fat quantity and distribution throughout the liver^47,48^. The image analysts were blinded to all clinical and biochemical data including the study group of the participants.

### MRE assessment

A standard 60-Hz shear-wave was generated by an acoustic passive driver attached to the body wall anterior to the liver and coupled with an acoustic active driver outside the MR examination room. A 2-dimensional motionsensitized gradient-recalled echo MRE pulse sequence synchronized to the shear wave frequency was acquired to obtain 4 noncontiguous axial slices (10-mm thickness, 10-mm inter-slice gap), each during a 16-s breath hold, through the widest transverse section of the liver with short recovery times in between. The acquisition parameters were as follows: repetition time, 50 milliseconds; echo time, 20.2 milliseconds; flip angle, 30 degrees; matrix, 256 × 64; field of view, 48 × 48 cm; one-signal average; receiver bandwidth ±33 kHz; and parallel imaging accelerating factor, 2. The total acquisition time was approximately 2 min. The wave images from each slice location were automatically processed on the scanner computer using inversion algorithm to generate axial liver stiffness maps called elastograms. The elastograms were transferred and analyzed offline by a trained image analyst (at least 6 months of experience with MRE analysis) blinded to clinical and histologic data.

### Ultrasound-based VCTE assessment

VCTE was performed by a trained technician, using the FibroScan® 502 Touch model (M Probe; XL Probe; Echosens, Paris, France). VCTE measurement was obtained in the supine position with the right arm fully adducted by scanning the area of abdomen at the location of the right liver lobe during a 10 s breath hold. Participants were asked to fast at least 3 h prior to the exam. The procedure included a minimum of 10 measurements to determine the median valid liver stiffness measurements (LSM) in kilopascals (kPa) and the interquartile range (IQR). According to the manufacturer protocol, all patients were first scanned using the M probe (3.5 MHz) and when indicated by the equipment upon initial assessment, patients were re-scanned using the XL probe (2.5 MHz)^31,33^. The threshold used for the classification of cirrhosis (stage 4) was VCTE > 11.8 kPa as previously determined in reference^31^. Among the first-degree relatives of proband with NAFLD-cirrhosis, 11 did not have an MRE assessment due to contraindication and the presence of advanced fibrosis was determined using a VCTE threshold >11.8 kPa as previously determined in reference^31^.

### Justification for not using liver biopsy

Liver biopsy was not used for hepatic fat content and fibrosis assessment of controls and first-degree relatives as they were asymptomatic with no suspected liver disease and therefore performing a liver biopsy would have been unethical. A non-invasive, accurate quantitative imaging method was used to estimate liver fat and fibrosis. We have previously shown that MRI-PDFF is a highly precise, accurate, and reproducible non-invasive biomarker for the quantification of liver fat content^49,50^. In addition, MRE is the most accurate, currently available, non-invasive quantitative biomarker of liver fibrosis^30,31,51^. MRE has been shown to be have excellent diagnostic accuracy in differentiating between normal liver and mild fibrosis (stage 0–2) and between non-advanced fibrosis and advanced fibrosis (stage 3–4)^31,52,53^.

### Definition of NAFLD

Participants were considered to have NAFLD if they had hepatic steatosis (MRI-PDFF ≥ 5%) and no secondary causes of hepatic steatosis due to factors including the use of steatogenic medications, other liver diseases, and significant alcohol intake (see Exclusion Criteria above for details).

### Definition of cirrhosis and advanced fibrosis

Participants were considered to have NAFLD-related cirrhosis if they had NAFLD according to the definition above, and have biopsy-proven cirrhosis (histology fibrosis stage 4). We have previously validated that a liver stiffness cut point of >3.63 kPa on MRE provides an accuracy of 0.92 for the detection of advanced fibrosis, and it is the most accurate non-invasive test for the diagnosis of advanced fibrosis^30,31,54–56^. Advanced fibrosis among first-degree relatives was determined by either imaging evidence of nodularity and presence of intraabdominal varices or other evidence imaging evidence of portal hypertension or liver stiffness assessment with MRE threshold ≥ 3.63 kPa^30,31,51^ or if MRE were not performed using transient elastography assessment with VCTE threshold ≥ 11.8 kPa^31^.

### Microbiome composition by 16S rRNA gene amplicon analysis

DNA extraction and 16S rRNA amplicon sequencing were done using Earth Microbiome Project (EMP) standard protocols (http://www.earthmicrobiome.org/protocols-and-standards/16s)^57,58^. DNA was extracted using the Qiagen MagAttract PowerSoil DNA kit^59^. Amplicon PCR was performed on the V4 region of the 16S rRNA gene using the primer pair 515 f to 806r with Golay error-correcting barcodes on the reverse primer (Supplementary Data 1). Amplicons were barcoded and pooled in equal concentrations for sequencing. The amplicon pool was purified with the MO BIO UltraClean PCR cleanup kit and sequenced on the Illumina MiSeq sequencing platform. Sequence data were demultiplexed and minimally quality filtered using the QIIME 1.9.1 script split_libraries_fastq.py, with a Phred quality threshold of 3 and default parameters to generate per-study FASTA sequence files^60^.

### Statistical analysis

Development of a model utilizing stool derived 16S gut-microbiome profiles to predict NAFLD-cirrhosis. To build a model capable of distinguishing samples belonging to NAFLD-cirrhosis from those of non-NAFLD-controls, we developed a custom machine learning process that employed Random Forest (RF) analysis^61,62^. The set of input features for model building consisted of 16S sequences and patient metadata features. Features from stool microbiome data consisted of the number of 16S sequences (~5700 features) and the patient metadata consisted of age, gender and BMI. The first step in building an RF model consisted of training RF and then selecting features with the most importance score >0.005 (27 features) in a second step. The final random forest model included the 27 bacterial features and important patient metadata (age, sex, and BMI) for a total of 30 predictive features.

Patients’ demographic, anthropometric, clinical, and biochemical characteristics were summarized. Categorical variables were shown as counts and percentages, and associations were tested using a *Χ*^2^ test or Fisher’s exact test. Normally distributed continuous variables were shown as mean (± standard deviation), and differences between groups were analyzed using a two-independent samples t- test or Wilcoxon–Mann–Whitney test. Statistical analyses on cohort characteristics were performed using SPSS 25.0 (IBM, Chicago, IL). A two-sided *p* value < 0.05 was considered statistically significant.

### Sample size estimation

On the basis of our previous study, including 16 individuals with NASH-cirrhosis/advanced fibrosis and 33 controls, we could identify significant differences compared to 33 controls^6^. The patient data and species abundance had an AUROC of 0.88. Therefore, the study including 26 participants with NAFLD-cirrhosis and 72 controls would be adequate to detect clinically meaningful differences between the sub-groups with a power of at least 80% with a two-tailed *p* value of less than 0.01.

## Supplementary information

Supplementary Information

Description of Additional Supplementary Files

Supplementary Data 1

Source Data file

## Data Availability

The data sets generated during and/or analysed during the current study are available in the European Bioinformatics Institute (EMBL-EBI) repository, under the accession number ERP110543. The source data files underlying Figs. 1a–d, 2a–c, 3a–b, 4 are provided as a Source Data file.

## Code availability

the analysis was done using QIIME and in-house scripts. All analyses are documented in Jupyter notebooks available at https://github.com/knightlab-analyses/familial-cirrhosis.
