# Supplementary material for: A gut microbiome signature for cirrhosis due to nonalcoholic fatty liver disease
Source: Nat Commun. 2019 Mar 29;10:1406. doi: 10.1038/s41467-019-09455-9 (PMC6440960; doi:10.1038/s41467-019-09455-9)
Supplement: Supplementary file 1 — Supplementary Information [file 41467_2019_9455_MOESM1_ESM.pdf]

A gut microbiome signature for cirrhosis due to nonalcoholic fatty liver disease

Caussy and Tripathi et al.

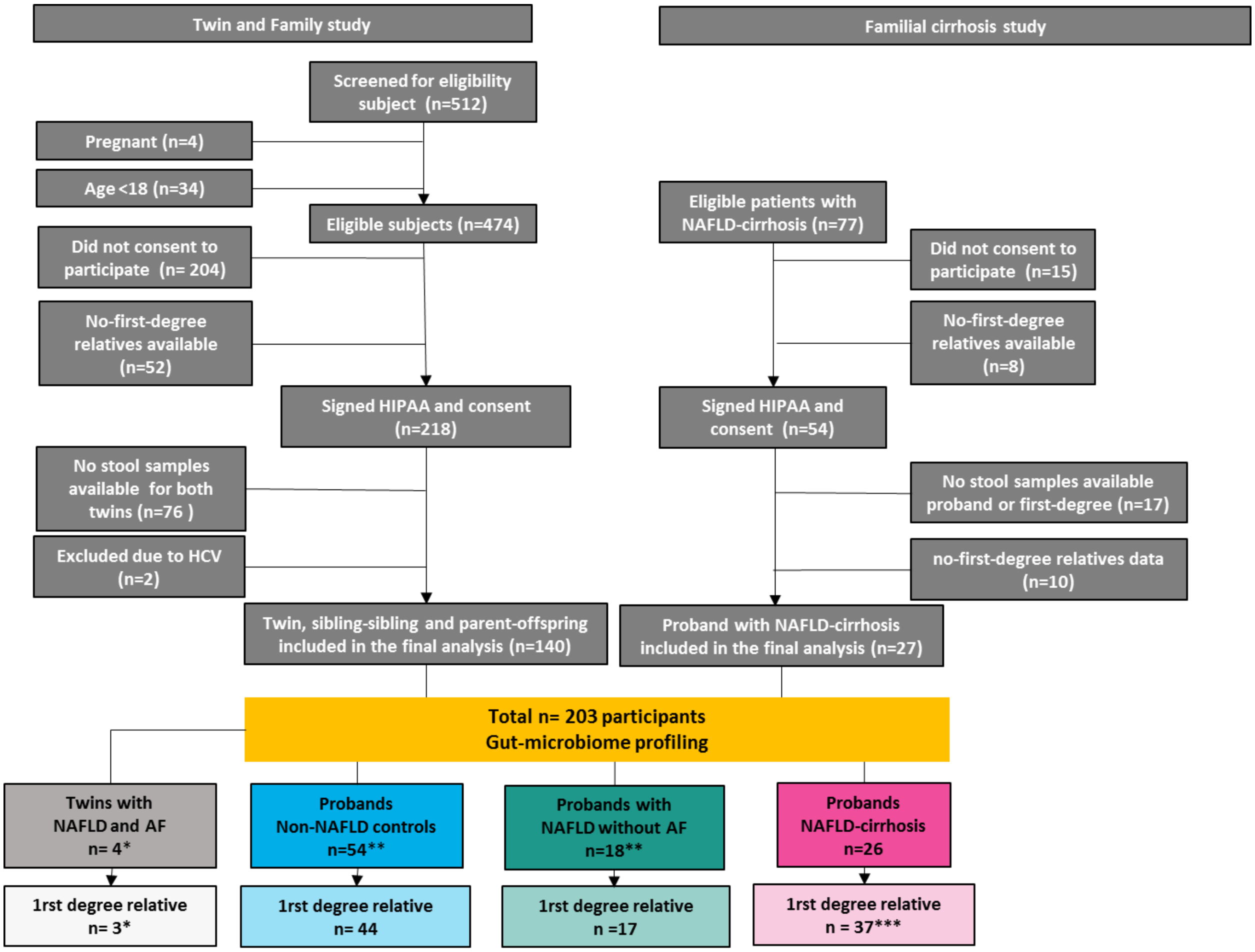

Supplementary Figure 1. Study flow-chart

A total of 203 participants from the Twin and Family study (n=140) and Familial cirrhosis study (n=63) with 16S gut-microbiome profiling were included in the study. *3 twin pairs were concordant for advanced fibrosis and 1 twin had NAFLD- cirrhosis were not assigned in a control group but was included in familial correlation analyses. ** stool samples were not available for the first-degree relative of 10 non-NAFLD controls and 1 proband with NAFLD without advanced fibrosis, the single probands were included in the gut-microbiome signature analysis. ***2 first degree relative did not have liver stiffness assessment (MRE of VCTE but were included in the familial correlation analysis), 11 first-degree relatives did not had an MRE assessment due to CI and were assessed using VCTE using a threshold of 11.8 kPa for the detection of cirrhosis.

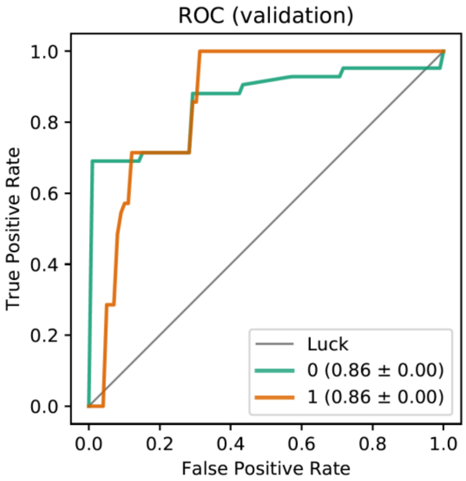

Supplementary Figure 2. Sensitivity analyses of the diagnostic accuracy of the gut-microbiome signature for the detection of advanced fibrosis

Receiver operating characteristic (ROC) curves evaluating ability to predict advanced Fibrosis using Random Forest classification. This curve represents the sensitivity and specificity to distinguish subjects with advanced fibrosis (1) from those without advanced fibrosis (0). The predictive model was trained on probands with NAFLD-cirrhosis (n=24) and non-NAFLD controls (n=47). We validated the prediction on a cohort comprising of NAFLD patients without advanced fibrosis (n=17) and first first-degree relatives of NAFLD-cirrhosis probands (n=32). The model predicted the presence of advanced fibrosis with an accuracy of 86%.

Supplementary Table 1: Baseline Characteristics of probands with NAFLD-cirrhosis, non-NAFLD controls and proband with NAFLD without advanced fibrosis

Characteristics	Non-NAFLD control (n=54)	Proband with NAFLD without Advanced fibrosis (n=18)	Probands with NAFLD-cirrhosis (n=26)	Overall p-value*
Demographics				
Age (years)	45.9 (19.9)	54.0 (14.9)	65.1 (9.8)	<0.001 β, δ
Female, n (%)	39 (72.2)	12 (66.7)	29 (76.3)	0.554
White, n (%)	43 (79.6)	14 (77.8)	11 (28.9)	<0.001 β, δ
Hispanic or Latino, n (%)	8 (14.8)	2 (11.1)	22 (57.8)	<0.001 β, δ
BMI (kg/m^2)	26.2 (6.8)	31.1 (6.6)	31.3 (6.1)	<0.001 α
Clinical				
Type 2 Diabetes, n (%)	1 (1.9)	2 (11.1)	22 (84.6)	<0.001 β, δ
Biological data				
AST (U/L)	22.3 (8.4)	22.2 (7.2)	47.1 (23.1)	<0.001 β, δ
ALT (U/L)	18.8 (8.8)	23.2 (11.2)	45.0 (37.4)	<0.001 β, δ
Alk P (U/L)	68.2 (19.0)	74.6 (24.1)	113.1 (40.1)	<0.001 β, δ
GGT (Ui/L)	17.6 (8.0)	29.1 (23.8)	100.2 (57.0)	<0.001 α, β, δ
Total Bilirubin (mg/dL)	0.51 (0.25)	0.37 (0.14)	2.4 (6.7)	0.055α
Direct Bilirubin (mg/dL)	0.12 (0.41)	0.10 (0.00)	2.4 (6.7)	0.021 α, β
Albumin (g/dL)	4.53 (0.325)	4.47 (0.23)	4.04 (0.62)	<0.001 β, δ
Glucose (mg/dl)	86.1 (9.0)	94.2 (18.8)	132.3 (63.1)	<0.001 α, β, δ
Hemoglobin A1c (%)	5.7 (0.5)	5.8 (0.3)	7.1 (1.9)	<0.001 β, δ
Insulin (U/ml)	8.8 (6.4)	15.0 (12.8)	43.5 (33.1)	<0.001 α, δ
Triglycerides (mg/dL)	81.5 (44.7)	112.2 (56.0)	147.4 (136.4)	0.004 α
Total cholesterol (mg/dL)	188.6 (41.0)	190.8 (34.4)	162.8 (64.2)	0.063 β
HDL-cholesterol (mg/dL)	69.9 (19.9)	56.8 (15.6)	49.2 (18.4)	<0.001 α, β
LDL-cholesterol (mg/dL)	102.4 (32.17)	112.6 (32.6)	79.3 (32.4)	0.003 β, δ
Platelet count ($10^3/\mu L$)	246.9 (48.5)	257.7 (44.0)	144.3 (69.6)	<0.001 β, δ
Prothrombin time	10.6 (0.69)	10.3 (0.7)	13.2 (3.7)	<0.001 β, δ

INR	1.02 (0.07)	1.00 (0.07)	1.23 (0.35)	0.001 β, δ
Ferritin (ng/mL)	103.9 (82.9)	112.6 (92.0)	132.8 (123.2)	0.474
Imaging data				
MRI-PDFF %	2.41 (0.86)	11.78 (6.77)	4.91 (3.8)	<0.001 α, β, δ
MRE kPa	2.13 (0.37)	2.47 (0.40)	5.88 (3.68)	<0.001 α, β, δ

Mean values are provided with standard deviation in parentheses, unless otherwise noted as n (%) BMI: body mass index, HbA1c: glycated hemoglobin, ALT: alanine aminotransferase, AST: aspartate aminotransferase, INR: International Normalized Ratio, APRI: AST to platelet ratio, HDL: High Density Lipoprotein, LDL: Low Density Lipoprotein, Alk P: Alkaline Phosphatase, MRI-PDFF: magnetic resonance imaging proton density fat fraction, MRE: magnetic resonance elastography. *P-value determined by chi-square or F-test from ANOVA. Superscripts indicate individual significant mean differences between α non-NAFLD control versus patients with NAFLD without advanced fibrosis, β non-NAFLD control versus proband with NAFLD-cirrhosis and δ patients with NAFLD without advanced fibrosis versus proband with NAFLD-cirrhosis.

Supplementary Table 2: Baseline Characteristics of first-degree relatives of NAFLD-cirrhosis, first-degree relatives of controls, first degree relative of Proband with NAFLD without advanced fibrosis

Characteristics	First Degree relatives of controls (n=44)	First Degree relatives of NAFLD without Advanced fibrosis (n=17)	First Degree relatives of NAFLD-cirrhosis (n=37)	Overall p-value*
Demographics				
Age (years)	43.9 (20.5)	54.3 (14.7)	47.2 (14.7)	0.121
Female, n (%)	32 (72.7)	10 (58.8)	21 (80.8)	0.430
White, n (%)	34 (77.3)	13 (76.5)	9 (34.6)	0.001Ώ, Δ
Hispanic or Latino, n (%)	7 (15.9)	2 (11.8)	15 (57.7)	0.001Ώ, Δ
BMI (kg/m^2)	25.3 (4.5)	29.1 (6.8)	34.4 (13.4)	<0.001Ύ, β
Clinical				
Type 2 Diabetes, n (%)	2 (4.5)	2 (11.8)	22 (84.6)	0.001Ώ, Δ
Biological data				
AST (U/L)	25.1 (9.0)	34.1 (16.4)	25.5 (10.8)	0.018Ύ
ALT (U/L)	23.3 (16.4)	37.2 (23.0)	25.9 (10.7)	0.012Ύ
Alk P (U/L)	72.1 (33.4)	90.8 (23.8)	73.8 (21.2)	0.057Ύ
GGT (Ui/L)	25.2 (25.8)	73.6 (95.7)	33.7 (25.4)	0.002Ύ
Total Bilirubin (mg/dL)	0.46 (0.21)	0.58 (0.37)	0.52 (0.20)	0.232
Direct Bilirubin (mg/dL)	0.11 (0.03)	0.17 (0.14)	0.52 (0.20)	<0.001Ύ, β
Albumin (g/dL)	4.5 (0.4)	4.4 (0.4)	4.44 (0.23)	0.431
Glucose (mg/dl)	88.1 (10.5)	91.8 (12.2)	95.5 (24.4)	0.170
Hemoglobin A1c (%)	5.7 (0.5)	5.9 (0.6)	5.7 (0.7)	0.462
Insulin (U/ml)	8.1 (4.9)	16.7 (13.8)	22.3 (21.2)	<0.001Ύ, β
Triglycerides (mg/dL)	81.1 (44.1)	163.5 (124.4)	141.9 (80.3)	<0.001Ύ, β
Total cholesterol (mg/dL)	178.4 (37.9)	205.2 (43.2)	184.2 (40.6)	0.069Ύ,
HDL-cholesterol (mg/dL)	66.0 (21.5)	59.2 (20.7)	49.2 (13.2)	0.001 β,
LDL-cholesterol (mg/dL)	96.3 (31.4)	114.9 (33.7)	104.0 (40.7)	0.201 β,
Platelet count (10^3/µL)	244.3 (52.1)	249.6 (86.0)	254.9 (68.0)	0.772
Prothrombin time	11.3 (2.6)	10.5 (1.2)	11.7 (4.3)	0.461

INR	1.1 (0.3)	1.0 (0.1)	1.1 (0.4)	0.650
Ferritin (ng/mL)	86.1 (61.2)	124.3 (89.9)	124.7 (126.4)	0.155
Imaging data				
MRI-PDFF %	2.22 (0.80)	8.38 (7.42)	8.00 (6.70)	<0.001Ύ, β
MRE kPa	2.19 (0.68)	2.88 (0.98)	4.10 (5.51)	0.008Ύ, β

Mean values are provided with standard deviation in parentheses, unless otherwise noted as n (%) BMI: body mass index, HbA1c: glycated hemoglobin, ALT: alanine aminotransferase, AST: aspartate aminotransferase, INR: International Normalized Ratio, APRI: AST to platelet ratio, HDL: High Density Lipoprotein, LDL: Low Density Lipoprotein, Alk P: Alkaline Phosphatase, MRI-PDFF: magnetic resonance imaging proton density fat fraction, MRE: magnetic resonance elastography. * Two sided p-value determined by chi-square or F-test from anova. Superscripts indicate individual significant mean differences between Ύ first degree relatives of non-NAFLD control versus first degree relatives of patients with NAFLD without advanced fibrosis, Ώ first degree relatives of non-NAFLD control versus first degree relatives of proband with NAFLD-cirrhosis and Δ first degree relatives of patients with NAFLD without advanced fibrosis versus first degree relatives of proband with NAFLD-cirrhosis.
