# Supplementary material for: A gut microbiome signature for cirrhosis due to nonalcoholic fatty liver disease
Source: Nat Commun. 2019 Mar 29;10:1406. doi: 10.1038/s41467-019-09455-9 (PMC6440960; doi:10.1038/s41467-019-09455-9)
Supplement: Supplementary file 2 — Description of Additional Supplementary Files [file 41467_2019_9455_MOESM2_ESM.pdf]

Description of Additional Supplementary Files

Supplementary Data 1: Primers sequences.
