# Supplementary material for: A gut microbiome signature for cirrhosis due to nonalcoholic fatty liver disease
Source: Nat Commun. 2019 Mar 29;10:1406. doi: 10.1038/s41467-019-09455-9 (PMC6440960; doi:10.1038/s41467-019-09455-9)
Supplement: Supplementary file 4 — Source Data file [file 41467_2019_9455_MOESM4_ESM.pdf]

predictors	11635.CIR42.(	11635.TW.BE.	11635.FS.CIR2	11635.CIR61.(	11635.TW.BV	11635.TW.CL.	11635.TW.AM
TACGGAGGGT	7886.0	66.0	6.0	8970.0	6.0	0.0	0.0
TACGTAGGTG	484.0	20.0	0.0	0.0	0.0	0.0	0.0
TACGGAGGGT	131.0	2.0	0.0	0.0	0.0	2.0	0.0
AACGTAGGTC	60.0	41.0	0.0	0.0	239.0	0.0	0.0
TACGGAGGAT	28.0	305.0	485.0	0.0	908.0	0.0	4.0
AACGTAGGTC	37.0	424.0	0.0	0.0	579.0	0.0	0.0
TACGGAAGGT	22.0	0.0	0.0	0.0	0.0	0.0	0.0
TACGTAGGTG	24.0	0.0	0.0	0.0	0.0	2.0	0.0
TACGTAGGTG	20.0	0.0	0.0	0.0	0.0	0.0	0.0
AACGTAGGGT	22.0	96.0	0.0	0.0	10.0	3.0	0.0
TACGGAGGAT	20.0	150.0	0.0	0.0	117.0	10.0	4.0
AACGTAGGTC	15.0	0.0	0.0	0.0	0.0	0.0	0.0
TACGGAGGAT	15.0	191.0	114.0	0.0	17.0	0.0	0.0
TACGTAGGGG	14.0	2.0	0.0	0.0	3.0	1008.0	0.0
TACGTAGGTG	16.0	16.0	0.0	0.0	28.0	0.0	0.0
TACGGAGGAT	12.0	495.0	2263.0	0.0	2746.0	2.0	3.0
TACGTAGGGG	10.0	33.0	0.0	0.0	6.0	0.0	0.0
TACGTAGGTG	8.0	0.0	0.0	0.0	3.0	61.0	5336.0
TACGTAGGGG	10.0	207.0	191.0	0.0	71.0	2.0	51.0
TACGTATGGT	8.0	524.0	181.0	1.0	25.0	0.0	0.0
AACGTAGGGT	9.0	83.0	57.0	0.0	58.0	0.0	0.0
TACGGAAGGT	8.0	0.0	0.0	0.0	0.0	0.0	0.0
TACGTAGGGG	8.0	34.0	0.0	0.0	0.0	0.0	0.0
TACGGAGGAT	10.0	0.0	0.0	0.0	0.0	0.0	0.0
TACGTAGGTG	6.0	49.0	0.0	0.0	0.0	0.0	0.0
TACGTAGGGG	4.0	34.0	0.0	0.0	24.0	0.0	0.0
AACGTAGGGT	3.0	0.0	0.0	0.0	0.0	0.0	0.0
TACGGAGGAT	6.0	62.0	0.0	0.0	0.0	0.0	0.0
TACGTAGGGG	5.0	0.0	0.0	0.0	0.0	0.0	0.0
TACGTAGGGG	5.0	12.0	0.0	0.0	110.0	0.0	0.0
TACGTAGGGG	6.0	15.0	0.0	0.0	6.0	4.0	0.0
TACGTATGGA	5.0	5.0	0.0	0.0	13.0	0.0	0.0
TACGTAGGGT	3.0	0.0	0.0	0.0	0.0	0.0	0.0
TACGTAGGGG	5.0	34.0	0.0	0.0	0.0	0.0	0.0
TACGTAGGGG	4.0	6.0	0.0	0.0	49.0	32.0	0.0
TACGTAGGTG	3.0	0.0	0.0	0.0	0.0	0.0	0.0
TACGTATGGT	4.0	53.0	0.0	0.0	0.0	0.0	0.0
TACGTAGGGG	2.0	30.0	0.0	0.0	22.0	0.0	0.0
TACGTATGGT	3.0	7.0	0.0	0.0	0.0	0.0	0.0
TACGTAGGGG	3.0	51.0	20.0	0.0	10.0	0.0	0.0
TACGGAGGAT	1.0	36.0	0.0	0.0	0.0	0.0	0.0
AACGTAGGTC	5.0	102.0	0.0	0.0	0.0	0.0	0.0
TACGTATGGA	3.0	9.0	0.0	0.0	1.0	0.0	0.0
TACGTAGGGG	3.0	208.0	0.0	0.0	11.0	3.0	0.0
AACGTAGGGT	1.0	1.0	0.0	0.0	7.0	0.0	0.0
TACGTAGGGG	3.0	71.0	2.0	0.0	2.0	3.0	0.0

TACGTAGGTC	2.0	5.0	4.0	0.0	19.0	0.0	362.0
TACAGAGGTC	3.0	119.0	0.0	0.0	0.0	0.0	0.0
TACGTATGGG	3.0	0.0	0.0	0.0	0.0	0.0	0.0
TACGTAGGGA	0.0	0.0	0.0	0.0	0.0	0.0	0.0
TACGTAGGGC	0.0	0.0	0.0	0.0	0.0	0.0	0.0
TACGTAGGTG	2.0	19.0	0.0	0.0	0.0	0.0	0.0
TACGTATGGT	2.0	94.0	0.0	0.0	66.0	0.0	0.0
TACGTAGGTG	1.0	0.0	0.0	0.0	0.0	0.0	0.0
TACGTAGGTG	0.0	0.0	0.0	0.0	0.0	0.0	0.0
TACGGAAGAT	2.0	0.0	0.0	0.0	0.0	0.0	0.0
TACGTAGGGA	2.0	8.0	0.0	0.0	0.0	0.0	0.0
TACGTAGGGG	0.0	0.0	0.0	0.0	1.0	0.0	0.0
TACGTAGGGG	1.0	0.0	0.0	0.0	0.0	0.0	0.0
TACGTAGGGG	1.0	0.0	0.0	0.0	0.0	0.0	0.0
TACGTAGGTG	1.0	17.0	0.0	0.0	251.0	0.0	0.0
TACGTAGGGG	2.0	0.0	0.0	0.0	0.0	0.0	0.0
TACGTAGGGG	1.0	0.0	0.0	0.0	0.0	0.0	0.0
TACGGAGGGT	1.0	0.0	0.0	0.0	0.0	0.0	0.0
TACGTATGGT	2.0	70.0	147.0	0.0	0.0	0.0	0.0
TACGTAGGGG	0.0	0.0	0.0	0.0	0.0	0.0	0.0
TACGTAGGTG	1.0	0.0	0.0	0.0	0.0	0.0	0.0
TACGTAGGGG	1.0	0.0	0.0	0.0	4.0	2.0	0.0
AACGTAGGGT	2.0	0.0	0.0	0.0	0.0	0.0	0.0
TACGTAGGGG	1.0	43.0	0.0	0.0	1.0	0.0	0.0
TACGTAGGGG	1.0	0.0	0.0	0.0	0.0	0.0	0.0
TACGTATGGA	1.0	26.0	0.0	0.0	0.0	0.0	0.0
TACGGAGGGT	1.0	0.0	0.0	0.0	0.0	0.0	0.0
TACGTAGGGG	1.0	0.0	0.0	0.0	0.0	0.0	0.0
TACGGAGGAT	1.0	0.0	0.0	0.0	98.0	0.0	0.0
TACGTAGGGG	1.0	0.0	0.0	0.0	0.0	0.0	0.0
TACGTAGGGG	1.0	0.0	0.0	0.0	0.0	0.0	0.0
TACGTAGGGG	1.0	0.0	0.0	0.0	0.0	0.0	0.0
TACGGAGGAT	1.0	112.0	0.0	0.0	0.0	0.0	0.0
TACGTAGGGG	0.0	604.0	0.0	0.0	0.0	0.0	0.0
TACGTAGGTG	0.0	299.0	0.0	0.0	89.0	35.0	0.0
TACGGAGGAT	0.0	201.0	0.0	0.0	0.0	0.0	0.0
TACGTAGGTG	0.0	199.0	0.0	0.0	0.0	0.0	0.0
TACGTATGTT	0.0	171.0	0.0	0.0	0.0	0.0	0.0
TACGTAGGGA	0.0	165.0	0.0	0.0	104.0	0.0	0.0
TACGGAGGAT	0.0	141.0	0.0	0.0	0.0	0.0	0.0
TACGTAGGTG	0.0	147.0	0.0	0.0	385.0	0.0	0.0
TACGTAGGTG	0.0	97.0	0.0	0.0	0.0	0.0	0.0
TACGGAGGAT	0.0	91.0	0.0	0.0	210.0	0.0	3.0
TACGTAGGGA	0.0	75.0	0.0	0.0	11.0	4.0	0.0
TACGTAGGGG	0.0	77.0	0.0	2.0	9.0	0.0	0.0
TACGGAAGGT	0.0	68.0	0.0	0.0	0.0	0.0	0.0
TACGTAGGGG	0.0	76.0	0.0	0.0	42.0	45.0	0.0

TACGTAGGG	0.0	58.0	7.0	0.0	35.0	2.0	33.0
TACGTATGGA	0.0	57.0	0.0	0.0	0.0	0.0	0.0
TACGTAGGGT	0.0	68.0	3601.0	0.0	81.0	157.0	0.0
TACGTAGGTG	0.0	68.0	0.0	0.0	0.0	0.0	0.0
TACGTAGGGT	0.0	66.0	0.0	0.0	8.0	0.0	0.0
TACGTAGGTG	0.0	60.0	0.0	0.0	0.0	0.0	0.0
TACGTATGGA	0.0	48.0	0.0	0.0	139.0	0.0	0.0
TACGGGGGT	0.0	47.0	0.0	0.0	0.0	0.0	0.0
TACGTATGGT	0.0	58.0	0.0	0.0	103.0	0.0	0.0
TACGTAGGGA	0.0	54.0	0.0	0.0	0.0	0.0	0.0
TACGTAGGTG	0.0	56.0	0.0	0.0	0.0	0.0	0.0
TACGGAGGGT	0.0	57.0	0.0	0.0	0.0	0.0	0.0
TACGTAGGTT	0.0	58.0	0.0	0.0	0.0	0.0	0.0
TACGTAGGGA	0.0	50.0	0.0	0.0	0.0	0.0	0.0
TACGGAGGAT	0.0	50.0	0.0	0.0	19.0	0.0	0.0
TACGTAGGG	0.0	49.0	0.0	0.0	0.0	0.0	0.0
TACGTAGGGA	0.0	40.0	0.0	0.0	0.0	0.0	0.0
TACGTATGGA	0.0	46.0	0.0	0.0	0.0	0.0	0.0
TACGTAGGG	0.0	34.0	0.0	0.0	0.0	0.0	0.0
TACGTAGGGA	0.0	46.0	0.0	0.0	0.0	0.0	0.0
TACGTATGGT	0.0	32.0	0.0	0.0	19.0	0.0	0.0
TACGTAGGG	0.0	31.0	0.0	0.0	1.0	0.0	0.0
TACGGAGGAT	0.0	31.0	0.0	0.0	53.0	0.0	0.0
TACGTAGGGA	0.0	35.0	0.0	0.0	0.0	0.0	0.0
TACGTAGGTG	0.0	30.0	0.0	0.0	0.0	0.0	0.0
TACATAGGG	0.0	27.0	0.0	0.0	0.0	0.0	0.0
TACGTAGGTG	0.0	27.0	0.0	0.0	374.0	0.0	0.0
TACGTAGGAT	0.0	28.0	0.0	0.0	0.0	0.0	0.0
TACGTAGGG	0.0	25.0	0.0	0.0	12.0	0.0	0.0
TACGTATGGT	0.0	25.0	0.0	0.0	0.0	2.0	2.0
TACGTAGGG	0.0	29.0	0.0	0.0	0.0	0.0	0.0
TACGTAGGGA	0.0	32.0	0.0	0.0	58.0	0.0	0.0
TACGTAGGTG	0.0	19.0	0.0	0.0	0.0	0.0	0.0
TACGTAGGTG	0.0	27.0	0.0	0.0	0.0	0.0	0.0
TACGAAGGG	0.0	24.0	0.0	0.0	0.0	0.0	0.0
TACGTAGGTG	0.0	22.0	0.0	0.0	0.0	0.0	0.0
TACGTAGGGT	0.0	22.0	0.0	0.0	0.0	0.0	0.0
TACGTATGGA	0.0	21.0	0.0	0.0	0.0	0.0	0.0
AACGTAGGTC	0.0	22.0	232.0	0.0	8.0	0.0	0.0
TACGTAGGTG	0.0	18.0	0.0	0.0	0.0	0.0	0.0
TACGTAGGG	0.0	21.0	0.0	0.0	0.0	0.0	0.0
TACGGAGGAT	0.0	11.0	0.0	0.0	0.0	0.0	0.0
TACGTAGGGA	0.0	14.0	1.0	0.0	0.0	0.0	0.0
TACGTAGGTG	0.0	19.0	0.0	0.0	0.0	0.0	0.0
TACGTATGGA	0.0	22.0	0.0	0.0	3.0	0.0	0.0
TACGTAGGTG	0.0	17.0	0.0	0.0	0.0	0.0	0.0
TACGGAGGGT	0.0	15.0	0.0	0.0	56.0	0.0	0.0

TACGGAAGGT	0.0	15.0	0.0	0.0	0.0	0.0	0.0
TACGTAGGTG	0.0	20.0	0.0	0.0	0.0	0.0	0.0
TACGTAGGTG	0.0	17.0	0.0	0.0	0.0	0.0	0.0
TACGGAGGAT	0.0	20.0	0.0	0.0	0.0	0.0	0.0
TACGTAGGGA	0.0	13.0	0.0	0.0	0.0	0.0	0.0
AACGTAGGGT	0.0	11.0	0.0	0.0	0.0	0.0	0.0
TACGTAGGGA	0.0	16.0	0.0	0.0	0.0	0.0	0.0
TACGTAGGGG	0.0	16.0	0.0	0.0	10.0	0.0	0.0
TACGTAGGGA	0.0	15.0	0.0	0.0	68.0	0.0	6.0
TACGGAGGAT	0.0	15.0	0.0	0.0	0.0	0.0	0.0
TACGTAGGGG	0.0	7.0	0.0	0.0	1.0	0.0	0.0
TACGTAGGTG	0.0	8.0	0.0	0.0	0.0	0.0	0.0
TACGTAGGGG	0.0	8.0	0.0	0.0	9.0	0.0	0.0
TACGTAGGGG	0.0	10.0	0.0	0.0	0.0	0.0	0.0
TACGTAGGTG	0.0	11.0	0.0	0.0	0.0	0.0	0.0
TACGTAGGGA	0.0	11.0	0.0	0.0	0.0	0.0	0.0
TACGTAGGGG	0.0	13.0	0.0	0.0	0.0	0.0	0.0
TACGTAGGGG	0.0	11.0	0.0	0.0	19.0	0.0	0.0
TACGTAGGGG	0.0	12.0	0.0	0.0	0.0	0.0	0.0
TACGTAGGGA	0.0	11.0	0.0	0.0	0.0	0.0	0.0
TACGTAGGTG	0.0	12.0	0.0	0.0	0.0	0.0	0.0
TACGTAGGGG	0.0	10.0	0.0	0.0	0.0	0.0	0.0
TACGTAGGGA	0.0	9.0	0.0	0.0	0.0	0.0	0.0
TACGTAGGGG	0.0	12.0	0.0	0.0	0.0	0.0	0.0
TACGTAGGTG	0.0	11.0	0.0	0.0	0.0	0.0	0.0
TACGTATGGT	0.0	7.0	0.0	0.0	38.0	0.0	0.0
TACGGAAGAT	0.0	10.0	0.0	0.0	0.0	0.0	0.0
TACGTAGGGG	0.0	9.0	0.0	0.0	0.0	0.0	0.0
TACGTAGGGG	0.0	10.0	0.0	0.0	0.0	0.0	0.0
TACGTATGGA	0.0	10.0	0.0	0.0	0.0	0.0	0.0
TACGTAGGTG	0.0	4.0	0.0	0.0	0.0	121.0	0.0
TACGTAGGGA	0.0	7.0	0.0	0.0	4.0	0.0	0.0
TACGTATGGT	0.0	10.0	0.0	0.0	5.0	2.0	0.0
TACGTAGGGG	0.0	6.0	0.0	0.0	0.0	0.0	0.0
TACGTAGGTG	0.0	7.0	0.0	0.0	1.0	0.0	0.0
TACGTAGGGG	0.0	6.0	0.0	0.0	0.0	0.0	0.0
TACGTAGGGG	0.0	10.0	0.0	0.0	0.0	0.0	391.0
TACGTAGGTG	0.0	6.0	0.0	0.0	50.0	0.0	0.0
TACGTAGGTG	0.0	7.0	0.0	0.0	6.0	0.0	0.0
TACGTATGGG	0.0	5.0	0.0	0.0	0.0	0.0	0.0
TACGTAGGTG	0.0	5.0	0.0	0.0	4.0	0.0	0.0
TACGTAGGGG	0.0	5.0	0.0	0.0	19.0	0.0	0.0
TACGTAGGGG	0.0	7.0	0.0	0.0	2.0	0.0	0.0
TACGTAGGTG	0.0	7.0	0.0	0.0	0.0	0.0	0.0
TACGTATGGT	0.0	5.0	0.0	0.0	4.0	0.0	12.0
TACGTAGGGG	0.0	6.0	0.0	0.0	1.0	0.0	0.0
TACGTAGGTG	0.0	4.0	0.0	0.0	0.0	0.0	0.0

TACGTATGGT	0.0	4.0	0.0	0.0	0.0	0.0	0.0
TACGTAGGGG	0.0	7.0	0.0	0.0	0.0	0.0	0.0
TACGTAGGGG	0.0	3.0	0.0	0.0	0.0	0.0	0.0
TACGTAGGTG	0.0	5.0	0.0	0.0	27.0	0.0	0.0
TACGTAGGTG	0.0	7.0	0.0	0.0	2.0	0.0	0.0
TACGTAGGGA	0.0	7.0	0.0	0.0	0.0	0.0	0.0
TACGTAGGGG	0.0	2.0	1.0	0.0	6.0	0.0	0.0
TACGTAGGTG	0.0	4.0	0.0	0.0	9.0	0.0	0.0
TACGTAGGTG	0.0	3.0	1.0	0.0	4.0	0.0	0.0
TACGTAGGGG	0.0	2.0	0.0	0.0	0.0	0.0	0.0
TACGGAGGAT	0.0	5.0	0.0	0.0	0.0	0.0	0.0
TACGTAGGGG	0.0	3.0	0.0	0.0	10.0	0.0	0.0
TACGTAGGGG	0.0	5.0	0.0	0.0	0.0	0.0	0.0
TACGTAGGGG	0.0	6.0	0.0	0.0	0.0	0.0	23.0
TACGTAGGGA	0.0	3.0	0.0	0.0	0.0	0.0	0.0
TACGTAGGGG	0.0	5.0	0.0	0.0	0.0	0.0	0.0
TACGTAGGGT	0.0	5.0	0.0	0.0	0.0	0.0	0.0
TACGTAGGAG	0.0	5.0	0.0	0.0	0.0	0.0	0.0
TACGTAGGGA	0.0	3.0	0.0	0.0	0.0	0.0	0.0
TACGTAGGGG	0.0	3.0	0.0	0.0	0.0	0.0	0.0
TACGTATGGA	0.0	6.0	0.0	0.0	1.0	0.0	0.0
TACGTAGGTG	0.0	3.0	310.0	0.0	0.0	0.0	0.0
TACGTAGGTG	0.0	5.0	0.0	0.0	146.0	0.0	0.0
TACGTAGGGA	0.0	3.0	2.0	0.0	0.0	0.0	0.0
AACGTAGGGT	0.0	1.0	0.0	0.0	0.0	0.0	0.0
TACGTAGGGG	0.0	5.0	0.0	0.0	0.0	0.0	0.0
TACGTATGGT	0.0	6.0	0.0	0.0	0.0	0.0	0.0
TACGTAGGGG	0.0	6.0	0.0	0.0	0.0	0.0	0.0
TACGTAGGTG	0.0	5.0	0.0	0.0	0.0	0.0	0.0
TACGTAGGTG	0.0	4.0	0.0	0.0	0.0	0.0	0.0
TACGGAAGGT	0.0	2.0	0.0	0.0	0.0	0.0	0.0
TACGTAGGGG	0.0	3.0	0.0	0.0	14.0	0.0	0.0
TACGTAGGTG	0.0	3.0	0.0	0.0	138.0	0.0	0.0
TACGTAGGGG	0.0	3.0	0.0	0.0	4.0	0.0	0.0
TACGTAGGGG	0.0	3.0	0.0	0.0	0.0	0.0	0.0
TACGTAGGTG	0.0	3.0	0.0	0.0	0.0	0.0	0.0
TACGTAGGTG	0.0	5.0	0.0	0.0	0.0	0.0	0.0
TACGTAGGGG	0.0	3.0	0.0	0.0	2.0	0.0	0.0
TACGTAGGGG	0.0	6.0	0.0	0.0	0.0	0.0	0.0
TACGTAGGGG	0.0	4.0	0.0	0.0	2.0	0.0	0.0
TACGTAGGGA	0.0	2.0	0.0	0.0	2.0	0.0	1.0
TACGTAGGGA	0.0	4.0	0.0	0.0	4.0	0.0	0.0
TACGTAGGGG	0.0	4.0	0.0	0.0	0.0	0.0	0.0
TACGTAGGTG	0.0	1.0	0.0	0.0	0.0	0.0	0.0
TACGTAGGGG	0.0	3.0	0.0	0.0	0.0	0.0	0.0
TACGGAAGGT	0.0	1.0	0.0	0.0	0.0	0.0	0.0
TACGTAGGGG	0.0	3.0	0.0	0.0	0.0	0.0	0.0

TACGTAGGGA	0.0	3.0	0.0	0.0	0.0	0.0	0.0
TACGTAGGGG	0.0	1.0	0.0	0.0	1.0	0.0	0.0
TACGTAGGTG	0.0	4.0	0.0	0.0	0.0	1.0	0.0
TACGTAGGTG	0.0	4.0	0.0	0.0	27.0	0.0	0.0
TACGTAGGGA	0.0	4.0	0.0	0.0	0.0	0.0	0.0
TACGTAGGTC	0.0	1.0	0.0	0.0	0.0	0.0	0.0
TACGTAGGGA	0.0	3.0	0.0	0.0	0.0	0.0	0.0
TACGTAGGGG	0.0	3.0	0.0	0.0	0.0	0.0	0.0
TACGTAGGGG	0.0	2.0	0.0	0.0	0.0	0.0	0.0
TACATAGGAT	0.0	2.0	0.0	0.0	0.0	0.0	0.0
TACGTAGGTG	0.0	2.0	0.0	0.0	0.0	0.0	0.0
TACGTAGGTG	0.0	3.0	0.0	0.0	0.0	0.0	0.0
TACGAAGGGG	0.0	3.0	0.0	0.0	171.0	0.0	0.0
TACGTAGGGG	0.0	2.0	0.0	0.0	0.0	0.0	0.0
TACGTAGGGG	0.0	3.0	0.0	0.0	2.0	0.0	0.0
TACGAAGGGG	0.0	4.0	0.0	0.0	0.0	0.0	0.0
TACGTAGGGG	0.0	2.0	0.0	0.0	0.0	0.0	0.0
TACGTAGGTG	0.0	4.0	0.0	0.0	0.0	0.0	0.0
TACGTAGGTG	0.0	2.0	0.0	0.0	0.0	0.0	0.0
TACGTAGGTG	0.0	1.0	0.0	0.0	0.0	0.0	0.0
TACGTAGGTG	0.0	0.0	0.0	0.0	0.0	0.0	0.0
TACGTAGGGG	0.0	3.0	0.0	0.0	0.0	0.0	0.0
TACGTAGGGA	0.0	2.0	0.0	0.0	0.0	0.0	0.0
TACGTAGGGA	0.0	3.0	0.0	0.0	0.0	0.0	0.0
TACGTAGGGG	0.0	2.0	0.0	0.0	2.0	0.0	0.0
TACGTAGGTG	0.0	1.0	0.0	0.0	0.0	0.0	0.0
TACGTAGGGG	0.0	1.0	0.0	0.0	0.0	0.0	0.0
TACGTAGGTG	0.0	2.0	0.0	0.0	0.0	0.0	0.0
TACGTAGGTG	0.0	3.0	0.0	0.0	3.0	0.0	0.0
TACGTAGGAG	0.0	2.0	0.0	0.0	0.0	0.0	0.0
TACGTAGGTG	0.0	3.0	0.0	0.0	0.0	0.0	0.0
TACGTAGGGA	0.0	3.0	0.0	0.0	0.0	0.0	0.0
TACGTAGGTG	0.0	3.0	0.0	0.0	0.0	0.0	0.0
TACGTAGGGG	0.0	3.0	0.0	0.0	1.0	0.0	0.0
TACGTAGGTG	0.0	2.0	0.0	0.0	0.0	0.0	0.0
TACGTAGGGG	0.0	1.0	0.0	0.0	0.0	0.0	0.0
TACGTAGGTG	0.0	2.0	0.0	0.0	0.0	0.0	0.0
TACGTAGGGG	0.0	1.0	0.0	0.0	0.0	0.0	0.0
TACGTAGGTG	0.0	2.0	0.0	0.0	0.0	0.0	0.0
TACGTAGGTG	0.0	2.0	0.0	0.0	0.0	0.0	0.0
TACGTAGGTG	0.0	2.0	0.0	0.0	0.0	0.0	0.0
TACGTAGGGG	0.0	1.0	0.0	0.0	0.0	0.0	32.0
TACGTAGGGG	0.0	2.0	0.0	0.0	0.0	0.0	0.0
TACGTAGGGG	0.0	1.0	0.0	0.0	1.0	0.0	0.0
TACGTAGGTG	0.0	2.0	0.0	0.0	0.0	0.0	0.0
TACGTAGGGG	0.0	2.0	0.0	0.0	0.0	0.0	0.0
TACGTAGGGG	0.0	1.0	0.0	0.0	0.0	0.0	0.0

TACGTAGGGG	0.0	2.0	0.0	0.0	0.0	0.0	0.0
TACGGAGGAT	0.0	2.0	0.0	0.0	0.0	0.0	0.0
TACGTAGGGA	0.0	2.0	0.0	0.0	0.0	0.0	0.0
TACGTAGGGG	0.0	0.0	0.0	0.0	0.0	0.0	0.0
TACGTAGGTG	0.0	0.0	0.0	0.0	4.0	0.0	0.0
TACGTAGGGG	0.0	2.0	0.0	0.0	0.0	0.0	0.0
TACGTAGGGG	0.0	2.0	0.0	0.0	0.0	0.0	0.0
TACGTAGGTG	0.0	1.0	0.0	0.0	0.0	0.0	0.0
TACGTAGGTG	0.0	1.0	0.0	0.0	0.0	0.0	0.0
TACGTAGGGG	0.0	1.0	0.0	0.0	0.0	0.0	0.0
TACGTAGGTG	0.0	2.0	0.0	0.0	0.0	3918.0	0.0
TACGTAGGGG	0.0	3.0	0.0	0.0	0.0	0.0	0.0
TACGTAGGGG	0.0	2.0	0.0	0.0	0.0	0.0	0.0
TACGTAGGTG	0.0	2.0	0.0	0.0	0.0	0.0	0.0
AACGTAGGGT	0.0	2.0	0.0	0.0	0.0	0.0	0.0
TACGTAGGGG	0.0	0.0	0.0	0.0	1.0	53.0	0.0
TACGTAGGGG	0.0	0.0	0.0	0.0	0.0	0.0	0.0
TACGTAGGGG	0.0	1.0	0.0	0.0	0.0	0.0	0.0
TACGTAGGGG	0.0	0.0	0.0	0.0	0.0	0.0	0.0
TACGTAGGGG	0.0	2.0	2.0	0.0	1.0	0.0	0.0
TACGTAGGGG	0.0	1.0	0.0	0.0	0.0	0.0	0.0
TACGTAGGGG	0.0	2.0	0.0	0.0	0.0	0.0	0.0
TACGTAGGGT	0.0	1.0	0.0	0.0	0.0	0.0	0.0
TACGTAGGGA	0.0	2.0	0.0	0.0	0.0	0.0	0.0
TACGTAGGGG	0.0	3.0	0.0	0.0	5.0	0.0	0.0
TACGTAGGGG	0.0	0.0	0.0	0.0	0.0	0.0	0.0
TACGTAGGTC	0.0	1.0	0.0	2.0	9.0	0.0	16.0
TACGTAGGTG	0.0	0.0	0.0	0.0	0.0	0.0	61.0
TACGTAGGGG	0.0	2.0	0.0	0.0	0.0	0.0	0.0
TACGTAGGTG	0.0	0.0	0.0	0.0	0.0	0.0	0.0
TACGTAGGGG	0.0	2.0	0.0	0.0	0.0	0.0	0.0
TACGTAGGGG	0.0	2.0	0.0	0.0	0.0	0.0	0.0
TACGTAGGTG	0.0	1.0	0.0	0.0	0.0	0.0	0.0
TACGTAGGTG	0.0	1.0	0.0	0.0	4.0	0.0	0.0
TACGTAGGGA	0.0	0.0	0.0	0.0	0.0	0.0	0.0
TACGGAGGAT	0.0	1.0	0.0	0.0	0.0	0.0	0.0
TACGTAGGTG	0.0	2.0	0.0	0.0	0.0	0.0	0.0
TACGTAGGTG	0.0	1.0	0.0	0.0	0.0	0.0	0.0
TACGTAGGTG	0.0	2.0	0.0	0.0	0.0	0.0	0.0
TACGTAGGGG	0.0	0.0	0.0	0.0	0.0	0.0	1.0
TACGTAGGGA	0.0	2.0	0.0	0.0	6.0	0.0	0.0
TACGTAGGGA	0.0	2.0	0.0	0.0	0.0	0.0	0.0
TACGTAGGTG	0.0	1.0	0.0	0.0	0.0	0.0	0.0
TACGTAGGGG	0.0	0.0	0.0	0.0	0.0	0.0	0.0
TACGTAGGTG	0.0	1.0	0.0	0.0	0.0	0.0	0.0
TACGTAGGGA	0.0	1.0	0.0	0.0	0.0	0.0	0.0
TACGTAGGGA	0.0	1.0	0.0	0.0	0.0	0.0	0.0

TACGTAGGTG	0.0	1.0	0.0	0.0	0.0	0.0	0.0
TACGTAGGGC	0.0	1.0	0.0	0.0	0.0	0.0	0.0
TACGTAGGGA	0.0	2.0	0.0	0.0	0.0	0.0	0.0
TACGTAGGTG	0.0	1.0	0.0	0.0	0.0	0.0	0.0
TACGTAAGGC	0.0	1.0	0.0	0.0	0.0	0.0	0.0
TACGTAGGAT	0.0	2.0	0.0	0.0	0.0	0.0	0.0
TACGTAGGGC	0.0	1.0	0.0	0.0	0.0	0.0	0.0
TACGTAGGGA	0.0	1.0	0.0	0.0	0.0	0.0	0.0
TACGTAGGTG	0.0	1.0	22.0	0.0	0.0	0.0	1196.0
TACGTAGGGC	0.0	2.0	0.0	0.0	0.0	0.0	0.0
TACGTAGGTG	0.0	0.0	0.0	0.0	0.0	0.0	0.0
TACGTAGGTG	0.0	1.0	0.0	0.0	1.0	0.0	0.0
TACGTAGGGA	0.0	1.0	0.0	0.0	0.0	0.0	0.0
TACGTAGGTG	0.0	2.0	0.0	0.0	0.0	0.0	0.0
TACGTAGGGA	0.0	0.0	0.0	0.0	0.0	0.0	0.0
TACGTAGGTG	0.0	0.0	0.0	0.0	0.0	0.0	0.0
TACGTAGGGA	0.0	2.0	0.0	0.0	1.0	0.0	0.0
TACGTAGGGC	0.0	1.0	0.0	0.0	0.0	0.0	0.0
TACGTAGGTG	0.0	1.0	0.0	0.0	0.0	0.0	0.0
TACGTAGGGC	0.0	1.0	0.0	0.0	0.0	0.0	0.0
TACGTAGGTG	0.0	0.0	0.0	0.0	0.0	0.0	0.0
TACGTAGGTG	0.0	1.0	0.0	0.0	0.0	0.0	0.0
TACGTAGGTG	0.0	1.0	0.0	0.0	0.0	0.0	0.0
TACGTAGGTG	0.0	1.0	0.0	0.0	0.0	0.0	2.0
TACGTAGGGC	0.0	1.0	0.0	0.0	0.0	0.0	0.0
TACGTAGGGC	0.0	1.0	0.0	0.0	0.0	0.0	0.0
TACGTAGGGA	0.0	1.0	0.0	0.0	0.0	0.0	0.0
TACGTAGGGC	0.0	1.0	0.0	0.0	0.0	0.0	0.0
TACGTAGGGA	0.0	0.0	0.0	0.0	0.0	0.0	0.0
TACGGAGGGT	0.0	2.0	0.0	0.0	0.0	0.0	0.0
TACGTAGGGC	0.0	0.0	0.0	0.0	0.0	0.0	0.0
TACGGAAGGT	0.0	2.0	0.0	0.0	0.0	0.0	0.0
TACGTAGGGC	0.0	1.0	0.0	0.0	0.0	0.0	0.0
TACGTAGGGC	0.0	0.0	0.0	0.0	2.0	0.0	0.0
TACGTAGGTG	0.0	1.0	0.0	0.0	0.0	0.0	0.0
TACGTAGGGA	0.0	1.0	0.0	0.0	0.0	0.0	0.0
TACGTAGGGC	0.0	1.0	0.0	0.0	0.0	0.0	0.0
TACGTAGGTG	0.0	1.0	0.0	0.0	0.0	0.0	0.0
TACGTAGGTG	0.0	1.0	0.0	0.0	0.0	0.0	0.0
TACGTAGGGA	0.0	0.0	0.0	0.0	0.0	0.0	0.0
TACGTATGGT	0.0	1.0	0.0	0.0	0.0	0.0	0.0
TACGTAGGGC	0.0	1.0	0.0	0.0	0.0	0.0	0.0
TACGTAGGTG	0.0	1.0	0.0	0.0	0.0	0.0	0.0
TACGTAGGTG	0.0	2.0	0.0	0.0	0.0	0.0	0.0
TACGTAGGGC	0.0	1.0	0.0	0.0	0.0	0.0	0.0
TACGTAGGTG	0.0	0.0	0.0	0.0	2.0	0.0	0.0
TACGTAGGGA	0.0	1.0	0.0	0.0	0.0	0.0	0.0

TACGTAGGGA	0.0	1.0	0.0	0.0	0.0	0.0	0.0
TACGTAGGGG	0.0	1.0	0.0	0.0	0.0	0.0	0.0
TACGTAGGGG	0.0	1.0	0.0	0.0	0.0	0.0	0.0
TACGTAGGTG	0.0	0.0	790.0	5.0	0.0	0.0	0.0
TACGTAGGTG	0.0	0.0	0.0	0.0	0.0	0.0	0.0
TACGGAGGAT	0.0	1.0	0.0	0.0	0.0	0.0	0.0
TACGTAGATG	0.0	1.0	0.0	0.0	0.0	0.0	0.0
TACGTAGGGA	0.0	2.0	0.0	0.0	2.0	0.0	0.0
TACGTAGGGG	0.0	0.0	0.0	0.0	0.0	0.0	0.0
TACGTAGGGG	0.0	0.0	0.0	0.0	0.0	0.0	0.0
TACGTAGGTG	0.0	2.0	0.0	0.0	0.0	0.0	0.0
AACGTAGGTG	0.0	1.0	0.0	0.0	0.0	0.0	0.0
TACGGAGGAT	0.0	0.0	0.0	0.0	185.0	0.0	0.0
TACGTAGGGG	0.0	0.0	0.0	0.0	0.0	0.0	0.0
TACGTAGGGA	0.0	0.0	0.0	0.0	0.0	0.0	0.0
TACGTAGGGG	0.0	0.0	0.0	0.0	0.0	0.0	0.0
TACGTAGGGG	0.0	0.0	0.0	0.0	0.0	0.0	0.0
TACGTAGGTG	0.0	0.0	0.0	0.0	0.0	0.0	0.0
TACGTAGGGG	0.0	0.0	0.0	0.0	0.0	0.0	0.0
TACGTAGGGG	0.0	1.0	0.0	0.0	0.0	0.0	0.0
TACGTAGGGA	0.0	1.0	0.0	0.0	0.0	0.0	0.0
TACGTATGGA	0.0	1.0	0.0	0.0	0.0	0.0	0.0
TACGTAGGTG	0.0	1.0	0.0	0.0	0.0	0.0	0.0
TACAGAGGTC	0.0	1.0	0.0	0.0	1.0	4.0	0.0
TACGTAGGTG	0.0	1.0	0.0	0.0	0.0	0.0	3.0
TACGAAGGGG	0.0	1.0	0.0	0.0	0.0	0.0	0.0
TACGTAGGGG	0.0	0.0	0.0	0.0	0.0	0.0	0.0
AACGTAGGTC	0.0	1.0	0.0	0.0	0.0	0.0	0.0
TACGTAGGGG	0.0	1.0	0.0	0.0	2.0	0.0	0.0
TACGTAGGGG	0.0	0.0	0.0	0.0	0.0	0.0	0.0
TACGTAGGGG	0.0	1.0	0.0	0.0	0.0	0.0	0.0
TACGTAGGGG	0.0	0.0	0.0	0.0	0.0	0.0	0.0
TACGTAGGTC	0.0	1.0	0.0	0.0	0.0	0.0	0.0
TACGTAGGGG	0.0	0.0	0.0	0.0	0.0	0.0	0.0
TACGTAGGTG	0.0	1.0	0.0	0.0	0.0	0.0	0.0
TACGTAGGTG	0.0	1.0	0.0	0.0	0.0	0.0	0.0
TACGTAGGGA	0.0	0.0	0.0	0.0	0.0	0.0	0.0
TACGTAGGGG	0.0	1.0	0.0	0.0	0.0	0.0	0.0
TACGTATGGA	0.0	0.0	0.0	0.0	0.0	0.0	0.0
TACGTAGGTG	0.0	1.0	0.0	0.0	0.0	0.0	0.0
TACGGAGGGT	0.0	0.0	261.0	0.0	0.0	0.0	0.0
TACGGAGGAT	0.0	0.0	172.0	0.0	0.0	0.0	0.0
TACGTATGTC	0.0	0.0	73.0	0.0	0.0	0.0	0.0
AACGTAGGTC	0.0	0.0	24.0	0.0	0.0	0.0	0.0
AACGTAGGTC	0.0	0.0	14.0	0.0	0.0	0.0	0.0
TACGTAGGTG	0.0	0.0	5.0	0.0	0.0	0.0	0.0
TACGTAGGTG	0.0	0.0	2.0	0.0	1.0	0.0	0.0

TACGTAGGG	0.0	0.0	1.0	0.0	0.0	0.0	0.0
TACGTAGGTG	0.0	0.0	0.0	0.0	0.0	0.0	0.0
TACGGAGGT	0.0	0.0	0.0	3.0	1.0	0.0	0.0
TACGTAGGTG	0.0	0.0	1.0	0.0	0.0	0.0	0.0
TACGGAGGA	0.0	0.0	1.0	0.0	0.0	0.0	0.0
TACGGAGGAT	0.0	0.0	2.0	0.0	0.0	0.0	0.0
TACGGAGGGT	0.0	0.0	1.0	0.0	0.0	0.0	0.0
TACGTAGGTC	0.0	0.0	2.0	0.0	0.0	0.0	0.0
TACGTAGGG	0.0	0.0	1.0	0.0	0.0	0.0	0.0
TACGTAGGTC	0.0	0.0	1.0	0.0	0.0	0.0	0.0
TACGTAGGTG	0.0	0.0	0.0	0.0	0.0	0.0	0.0
TACGTAGGTG	0.0	0.0	0.0	6.0	0.0	0.0	0.0
TACGTAGGG	0.0	0.0	0.0	5.0	5.0	0.0	0.0
TACGGAGGGT	0.0	0.0	0.0	0.0	1.0	2.0	0.0
TACTTCTTTTT	0.0	0.0	0.0	2.0	0.0	0.0	0.0
TACGTAGGGC	0.0	0.0	0.0	1.0	0.0	0.0	0.0
TTCTTCTTTTT	0.0	0.0	0.0	2.0	0.0	0.0	0.0
TACGGAGGGT	0.0	0.0	0.0	1.0	0.0	0.0	0.0
TACGTAGGTG	0.0	0.0	0.0	0.0	172.0	0.0	0.0
TACGTAGGGT	0.0	0.0	0.0	0.0	50.0	0.0	0.0
TACGGAGGAT	0.0	0.0	0.0	0.0	37.0	0.0	2.0
TACGGAGGAT	0.0	0.0	0.0	0.0	22.0	0.0	0.0
TACGTATGGA	0.0	0.0	0.0	0.0	18.0	0.0	0.0
TACGGAGGAT	0.0	0.0	0.0	0.0	18.0	0.0	0.0
TACGTAGGG	0.0	0.0	0.0	0.0	22.0	0.0	0.0
TACGGAGGAT	0.0	0.0	0.0	0.0	16.0	0.0	0.0
TACGTAGGGT	0.0	0.0	0.0	0.0	13.0	0.0	0.0
TACGTAGGTG	0.0	0.0	0.0	0.0	20.0	0.0	0.0
TACGTAGGG	0.0	0.0	0.0	0.0	13.0	0.0	0.0
TACGTAGGTG	0.0	0.0	0.0	0.0	10.0	0.0	0.0
TACGTAGGG	0.0	0.0	0.0	0.0	13.0	0.0	0.0
TACGTAGGGA	0.0	0.0	0.0	0.0	8.0	0.0	0.0
TACGTAGGG	0.0	0.0	0.0	0.0	12.0	0.0	0.0
TACGTAGGG	0.0	0.0	0.0	0.0	3.0	0.0	0.0
TACGGAGGAT	0.0	0.0	0.0	0.0	8.0	0.0	0.0
TACGTAGGTG	0.0	0.0	0.0	0.0	7.0	0.0	0.0
TACGTAGGG	0.0	0.0	0.0	0.0	8.0	0.0	0.0
TACGTAGGG	0.0	0.0	0.0	0.0	5.0	0.0	0.0
TACGTAGGTG	0.0	0.0	0.0	0.0	6.0	2.0	0.0
TACGTAGGG	0.0	0.0	0.0	0.0	5.0	0.0	0.0
TACGTAGGGT	0.0	0.0	0.0	0.0	5.0	0.0	0.0
TACGGAGGAT	0.0	0.0	0.0	0.0	5.0	0.0	0.0
TACGTAGGGA	0.0	0.0	0.0	0.0	3.0	0.0	0.0
TACGTAGGGT	0.0	0.0	0.0	0.0	3.0	0.0	0.0
TACGTAGGG	0.0	0.0	0.0	0.0	5.0	0.0	0.0
TACGTAGGG	0.0	0.0	0.0	0.0	7.0	0.0	0.0
TACGTAGGG	0.0	0.0	0.0	0.0	2.0	0.0	0.0

TACGTAGGG	0.0	0.0	0.0	0.0	5.0	0.0	0.0
TACGTAGGGA	0.0	0.0	0.0	0.0	2.0	0.0	0.0
TACGTAGGG	0.0	0.0	0.0	0.0	2.0	0.0	0.0
TACGTAGGTC	0.0	0.0	0.0	0.0	0.0	0.0	18.0
TACGTAGGG	0.0	0.0	0.0	0.0	4.0	0.0	0.0
TACGTAGGGA	0.0	0.0	0.0	0.0	3.0	0.0	0.0
TACGTAGGGA	0.0	0.0	0.0	0.0	2.0	0.0	0.0
TACGTAGGGA	0.0	0.0	0.0	0.0	2.0	0.0	0.0
TACGTAGGG	0.0	0.0	0.0	0.0	3.0	0.0	0.0
TACGTAGGTG	0.0	0.0	0.0	0.0	3.0	0.0	0.0
TACGTAGGG	0.0	0.0	0.0	0.0	2.0	0.0	0.0
TACGAAGGGT	0.0	0.0	0.0	0.0	2.0	3.0	0.0
TACGGAGGA	0.0	0.0	0.0	0.0	3.0	0.0	0.0
TACGTAGGTG	0.0	0.0	0.0	0.0	3.0	11.0	0.0
TACGTAGGTG	0.0	0.0	0.0	0.0	3.0	0.0	0.0
TACGTAGGGA	0.0	0.0	0.0	0.0	3.0	0.0	0.0
TACGTAGGTG	0.0	0.0	0.0	0.0	2.0	0.0	0.0
TACGTAGGTG	0.0	0.0	0.0	0.0	2.0	0.0	0.0
TACGTAGGG	0.0	0.0	0.0	0.0	3.0	0.0	0.0
TACGTATGGA	0.0	0.0	0.0	0.0	1.0	0.0	0.0
CACGTAGGG	0.0	0.0	0.0	0.0	1.0	0.0	0.0
TACGGAGGG	0.0	0.0	0.0	0.0	2.0	3.0	0.0
TACGTAGGTG	0.0	0.0	0.0	0.0	2.0	0.0	0.0
TACGTAGGG	0.0	0.0	0.0	0.0	3.0	0.0	0.0
TACGTAGGTG	0.0	0.0	0.0	0.0	0.0	0.0	0.0
TACGGAGGAT	0.0	0.0	0.0	0.0	1.0	0.0	0.0
TACGTAGGG	0.0	0.0	0.0	0.0	0.0	0.0	0.0
TACGTAGGG	0.0	0.0	0.0	0.0	1.0	0.0	0.0
TACGGAGGAT	0.0	0.0	0.0	0.0	2.0	0.0	0.0
TACGGAGGGT	0.0	0.0	0.0	0.0	1.0	0.0	0.0
TACGTAGGGA	0.0	0.0	0.0	0.0	2.0	0.0	0.0
TACGTAGGG	0.0	0.0	0.0	0.0	0.0	0.0	0.0
TACGGAGGGT	0.0	0.0	0.0	0.0	1.0	0.0	0.0
TACGTAGGTG	0.0	0.0	0.0	0.0	2.0	0.0	0.0
TACGGAGGGT	0.0	0.0	0.0	0.0	0.0	0.0	0.0
TACGTAGGGA	0.0	0.0	0.0	0.0	2.0	0.0	0.0
TACGTAGGG	0.0	0.0	0.0	0.0	2.0	0.0	0.0
TACGTAGGTG	0.0	0.0	0.0	0.0	1.0	0.0	0.0
TACGTAGGG	0.0	0.0	0.0	0.0	0.0	0.0	0.0
TACGTAGGGA	0.0	0.0	0.0	0.0	1.0	0.0	0.0
TACGTAGGGA	0.0	0.0	0.0	0.0	0.0	0.0	0.0
TACGTAGGGA	0.0	0.0	0.0	0.0	1.0	0.0	0.0
TACGTAGGG	0.0	0.0	0.0	0.0	1.0	0.0	0.0
TACGTAGGG	0.0	0.0	0.0	0.0	2.0	0.0	0.0
TACGTAGGG	0.0	0.0	0.0	0.0	0.0	0.0	0.0
TACGTAGGG	0.0	0.0	0.0	0.0	2.0	0.0	0.0
TACGTATGGA	0.0	0.0	0.0	0.0	1.0	0.0	0.0

TACGTATGGT	0.0	0.0	0.0	0.0	0.0	0.0	0.0
TACGGAGGAT	0.0	0.0	0.0	0.0	2.0	0.0	0.0
TACGTAGGGG	0.0	0.0	0.0	0.0	0.0	0.0	0.0
TACGTAGGGC	0.0	0.0	0.0	0.0	0.0	0.0	21.0
TACGTAGGTG	0.0	0.0	0.0	0.0	1.0	0.0	0.0
TACGTAGGGG	0.0	0.0	0.0	0.0	0.0	0.0	0.0
CACGTAGGGG	0.0	0.0	0.0	0.0	1.0	0.0	0.0
TACGTAGGTG	0.0	0.0	0.0	0.0	1.0	0.0	0.0
TACGTAGGGA	0.0	0.0	0.0	0.0	1.0	0.0	0.0
AACGTAGGTC	0.0	0.0	0.0	0.0	1.0	0.0	0.0
TACGGAGGGT	0.0	0.0	0.0	0.0	0.0	0.0	0.0
TACGGAGGAT	0.0	0.0	0.0	0.0	1.0	0.0	0.0
TACGTAGGTG	0.0	0.0	0.0	0.0	1.0	0.0	0.0
TACGGAGGGT	0.0	0.0	0.0	0.0	1.0	0.0	0.0
TACGTAGGGG	0.0	0.0	0.0	0.0	0.0	0.0	0.0
TACGTAGGGG	0.0	0.0	0.0	0.0	1.0	0.0	0.0
TACGTATGGT	0.0	0.0	0.0	0.0	1.0	0.0	0.0
TACGTAGGGT	0.0	0.0	0.0	0.0	1.0	0.0	0.0
TACGTAGGGA	0.0	0.0	0.0	0.0	1.0	0.0	0.0
TACGTAGGGG	0.0	0.0	0.0	0.0	0.0	0.0	0.0
AACGTAGGTC	0.0	0.0	0.0	0.0	1.0	0.0	0.0
TACGTAGGGA	0.0	0.0	0.0	0.0	1.0	0.0	0.0
TACGTAGGCG	0.0	0.0	0.0	0.0	1.0	0.0	0.0
TACGTAGGGG	0.0	0.0	0.0	0.0	1.0	0.0	0.0
TACGTAGGTG	0.0	0.0	0.0	0.0	1.0	0.0	0.0
TACGTAGGTG	0.0	0.0	0.0	0.0	0.0	2491.0	0.0
TACGTAGGGG	0.0	0.0	0.0	0.0	0.0	278.0	0.0
TACGTAGGTG	0.0	0.0	0.0	0.0	0.0	227.0	0.0
TACGTAGGTG	0.0	0.0	0.0	0.0	0.0	203.0	0.0
TACGTAGGGG	0.0	0.0	0.0	0.0	0.0	87.0	0.0
TACGTAGGTG	0.0	0.0	0.0	0.0	0.0	71.0	0.0
TACGTAGGTG	0.0	0.0	0.0	0.0	0.0	60.0	0.0
TACGTAGGGG	0.0	0.0	0.0	0.0	0.0	32.0	0.0
TACGTAGGGA	0.0	0.0	0.0	0.0	0.0	9.0	0.0
TACGTAGGGC	0.0	0.0	0.0	0.0	0.0	7.0	0.0
TACGTAGGGG	0.0	0.0	0.0	0.0	0.0	4.0	0.0
TACGTAGGTG	0.0	0.0	0.0	0.0	0.0	4.0	0.0
TACGTAGGGT	0.0	0.0	0.0	0.0	0.0	4.0	0.0
TACGTAGGGG	0.0	0.0	0.0	0.0	0.0	2.0	0.0
TACGGAGGGT	0.0	0.0	0.0	0.0	0.0	3.0	0.0
TACGTAGGTG	0.0	0.0	0.0	0.0	0.0	3.0	0.0
TACGTAGGGG	0.0	0.0	0.0	0.0	0.0	2.0	0.0
TACGTAGGGG	0.0	0.0	0.0	0.0	0.0	2.0	0.0
TACGTAGGTG	0.0	0.0	0.0	0.0	0.0	2.0	0.0
TACGTAGGGG	0.0	0.0	0.0	0.0	0.0	2.0	0.0
TACGTAGGGG	0.0	0.0	0.0	0.0	0.0	2.0	0.0
TACGGAGGGC	0.0	0.0	0.0	0.0	0.0	1.0	0.0

TACGAAGGGT	0.0	0.0	0.0	0.0	0.0	1.0	0.0
TACGTAGGTG	0.0	0.0	0.0	0.0	0.0	1.0	0.0
TACGTAGGTG	0.0	0.0	0.0	0.0	0.0	1.0	0.0
TACGTAGGTG	0.0	0.0	0.0	0.0	0.0	1.0	0.0
TACGTAGGGG	0.0	0.0	0.0	0.0	0.0	0.0	0.0
TACGTAGGGG	0.0	0.0	0.0	0.0	0.0	1.0	0.0
TACGTAGGGG	0.0	0.0	0.0	0.0	0.0	0.0	717.0
TACGTAGGGA	0.0	0.0	0.0	0.0	0.0	0.0	154.0
TACGTAGGGG	0.0	0.0	0.0	0.0	0.0	0.0	118.0
TACGTAGGTG	0.0	0.0	0.0	0.0	0.0	0.0	100.0
TACGTAAGGG	0.0	0.0	0.0	0.0	0.0	0.0	94.0
TACGTAGGGG	0.0	0.0	0.0	0.0	0.0	0.0	76.0
TACGTAGGGG	0.0	0.0	0.0	0.0	0.0	0.0	26.0
TACGTAGGGG	0.0	0.0	0.0	0.0	0.0	0.0	26.0
TACGTAGGGG	0.0	0.0	0.0	0.0	0.0	0.0	25.0
TACGTAGGGG	0.0	0.0	0.0	0.0	0.0	0.0	11.0
TACGTAGGTC	0.0	0.0	0.0	0.0	0.0	0.0	6.0
TACGGAGGGT	0.0	0.0	0.0	0.0	0.0	0.0	9.0
TACGTAGGGT	0.0	0.0	0.0	0.0	0.0	0.0	7.0
TACGTAGGTG	0.0	0.0	0.0	0.0	0.0	0.0	6.0
TACGTAGGTG	0.0	0.0	0.0	0.0	0.0	0.0	4.0
TACGTAGGGA	0.0	0.0	0.0	0.0	0.0	0.0	3.0
TACGTAAGGG	0.0	0.0	0.0	0.0	0.0	0.0	4.0
TACGTAGGGT	0.0	0.0	0.0	0.0	0.0	0.0	2.0
GACGGGGGG	0.0	0.0	0.0	0.0	0.0	0.0	3.0
TACGTAGGTG	0.0	0.0	0.0	0.0	0.0	0.0	2.0
TACGTAGGGA	0.0	0.0	0.0	0.0	0.0	0.0	3.0
TACGTATGGT	0.0	0.0	0.0	0.0	0.0	0.0	2.0
TACGGAGGGT	0.0	0.0	0.0	0.0	0.0	0.0	2.0
TACGTATGTC	0.0	0.0	0.0	0.0	0.0	0.0	2.0
TACGGAGGG	0.0	0.0	0.0	0.0	0.0	0.0	1.0
TACGTAGGGT	0.0	0.0	0.0	0.0	0.0	0.0	2.0
TACGTAGGGC	0.0	0.0	0.0	0.0	0.0	0.0	2.0
TACGGAGGGT	0.0	0.0	0.0	0.0	0.0	0.0	1.0
TACGTAGGTG	0.0	0.0	0.0	0.0	0.0	0.0	2.0
TACGTAGGGG	0.0	0.0	0.0	0.0	0.0	0.0	2.0
TACGTAGGGG	0.0	0.0	0.0	0.0	0.0	0.0	1.0
TACGGAGGAT	0.0	0.0	0.0	0.0	0.0	0.0	1.0
TACGGAGGAT	0.0	0.0	0.0	0.0	0.0	0.0	1.0
TACGTAGGGC	0.0	0.0	0.0	0.0	0.0	0.0	1.0
TACGTAGGGG	0.0	0.0	0.0	0.0	0.0	0.0	1.0
TACGTAGGTG	0.0	0.0	0.0	0.0	0.0	0.0	0.0
TACGTAGGGA	0.0	0.0	0.0	0.0	0.0	0.0	0.0
AACGTAGGTC	0.0	0.0	0.0	0.0	0.0	0.0	0.0
TACGGAGGAT	0.0	0.0	0.0	0.0	0.0	0.0	0.0
TACGTAGGGG	0.0	0.0	0.0	0.0	0.0	0.0	0.0
TACGTAGGGA	0.0	0.0	0.0	0.0	0.0	0.0	0.0

TACGTAGGTG	0.0	0.0	0.0	0.0	0.0	0.0	0.0
TACGGAGGAT	0.0	0.0	0.0	0.0	0.0	0.0	0.0
TACGTATGGA	0.0	0.0	0.0	0.0	0.0	0.0	0.0
TACGTAGGGG	0.0	0.0	0.0	0.0	0.0	0.0	0.0
TACGTAGGTG	0.0	0.0	0.0	0.0	0.0	0.0	0.0
TACGTAGGGG	0.0	0.0	0.0	0.0	0.0	0.0	0.0
TACGTAGGTG	0.0	0.0	0.0	0.0	0.0	0.0	0.0
TACGTAGGGG	0.0	0.0	0.0	0.0	0.0	0.0	0.0
TACGTAGGGA	0.0	0.0	0.0	0.0	0.0	0.0	0.0
TACGTAGGGA	0.0	0.0	0.0	0.0	0.0	0.0	0.0
TACGTATGGA	0.0	0.0	0.0	0.0	0.0	0.0	0.0
TACGTATGGA	0.0	0.0	0.0	0.0	0.0	0.0	0.0
TACGTAGGGG	0.0	0.0	0.0	0.0	0.0	0.0	0.0
TACGGAGGGT	0.0	0.0	0.0	0.0	0.0	0.0	0.0
TACGTAGGGA	0.0	0.0	0.0	0.0	0.0	0.0	0.0
TACGTAGGGG	0.0	0.0	0.0	0.0	0.0	0.0	0.0
TACGTAGGGG	0.0	0.0	0.0	0.0	0.0	0.0	0.0
TACGTAGGGG	0.0	0.0	0.0	0.0	0.0	0.0	0.0
TACGTAGGGA	0.0	0.0	0.0	0.0	0.0	0.0	0.0
TACGTAGGGA	0.0	0.0	0.0	0.0	0.0	0.0	0.0
TACGTAGGGG	0.0	0.0	0.0	0.0	0.0	0.0	0.0
TACGGAGGGT	0.0	0.0	0.0	0.0	0.0	0.0	0.0
TACGTATGGT	0.0	0.0	0.0	0.0	0.0	0.0	0.0
TACGGAGGAT	0.0	0.0	0.0	0.0	0.0	0.0	0.0
TACGTATGGT	0.0	0.0	0.0	0.0	0.0	0.0	0.0
TACGAAGGGG	0.0	0.0	0.0	0.0	0.0	0.0	0.0
TACGTAGGGT	0.0	0.0	0.0	0.0	0.0	0.0	0.0
TACGTAGGTG	0.0	0.0	0.0	0.0	0.0	0.0	0.0
TACGGAGGAT	0.0	0.0	0.0	0.0	0.0	0.0	0.0
TACGTAGGTG	0.0	0.0	0.0	0.0	0.0	0.0	0.0
TACGGAGGAT	0.0	0.0	0.0	0.0	0.0	0.0	0.0
TACGGAAGGT	0.0	0.0	0.0	0.0	0.0	0.0	0.0
TACGTATGGA	0.0	0.0	0.0	0.0	0.0	0.0	0.0
TACGGAGGGT	0.0	0.0	0.0	0.0	0.0	0.0	0.0
TACGTAGGGG	0.0	0.0	0.0	0.0	0.0	0.0	0.0
TACGTATGGT	0.0	0.0	0.0	0.0	0.0	0.0	0.0
TACGGAAGGT	0.0	0.0	0.0	0.0	0.0	0.0	0.0
TACGTAGGGG	0.0	0.0	0.0	0.0	0.0	0.0	0.0
TACGGAGGAT	0.0	0.0	0.0	0.0	0.0	0.0	0.0
TACGTAGGGG	0.0	0.0	0.0	0.0	0.0	0.0	0.0
TACGTAGGGG	0.0	0.0	0.0	0.0	0.0	0.0	0.0
TACGTAGGTG	0.0	0.0	0.0	0.0	0.0	0.0	0.0
TACGTAGGTG	0.0	0.0	0.0	0.0	0.0	0.0	0.0
TACGTAGGGG	0.0	0.0	0.0	0.0	0.0	0.0	0.0
TACGTATGGA	0.0	0.0	0.0	0.0	0.0	0.0	0.0
TACGTAGGGA	0.0	0.0	0.0	0.0	0.0	0.0	0.0
TACGTAGGTG	0.0	0.0	0.0	0.0	0.0	0.0	0.0

TACGGAGGGT	0.0	0.0	0.0	0.0	0.0	0.0	0.0
TACGTATGGG	0.0	0.0	0.0	0.0	0.0	0.0	0.0
TACGTAGGGA	0.0	0.0	0.0	0.0	0.0	0.0	0.0
TACGTAGGGA	0.0	0.0	0.0	0.0	0.0	0.0	0.0
TACGTAGGGA	0.0	0.0	0.0	0.0	0.0	0.0	0.0
TACGTAGGGG	0.0	0.0	0.0	0.0	0.0	0.0	0.0
TACGGAGGAT	0.0	0.0	0.0	0.0	0.0	0.0	0.0
TACGTAGGTG	0.0	0.0	0.0	0.0	0.0	0.0	0.0
TACGTAGGTG	0.0	0.0	0.0	0.0	0.0	0.0	0.0
TACGTAGGGG	0.0	0.0	0.0	0.0	0.0	0.0	0.0
TACGGAGGGT	0.0	0.0	0.0	0.0	0.0	0.0	0.0
TACGTAGGAT	0.0	0.0	0.0	0.0	0.0	0.0	0.0
TACGTAGGGG	0.0	0.0	0.0	0.0	0.0	0.0	0.0
TACGTAGGGG	0.0	0.0	0.0	0.0	0.0	0.0	0.0
TACGTATGGT	0.0	0.0	0.0	0.0	0.0	0.0	0.0
TACGTAGGGG	0.0	0.0	0.0	0.0	0.0	0.0	0.0
TACGTAGGTG	0.0	0.0	0.0	0.0	0.0	0.0	0.0
TACGTAGGGG	0.0	0.0	0.0	0.0	0.0	0.0	0.0
TACGTAGGTG	0.0	0.0	0.0	0.0	0.0	0.0	0.0
TACGTAGGGT	0.0	0.0	0.0	0.0	0.0	0.0	0.0
TACGTAGGGA	0.0	0.0	0.0	0.0	0.0	0.0	0.0
TACGTAGGTG	0.0	0.0	0.0	0.0	0.0	0.0	0.0
TACGTATGGA	0.0	0.0	0.0	0.0	0.0	0.0	0.0
TACGTAGGGG	0.0	0.0	0.0	0.0	0.0	0.0	0.0
TACGTAGGTG	0.0	0.0	0.0	0.0	0.0	0.0	0.0
TACGTAGGGT	0.0	0.0	0.0	0.0	0.0	0.0	0.0
TACGTAGGTG	0.0	0.0	0.0	0.0	0.0	0.0	0.0
TACGTAGGGG	0.0	0.0	0.0	0.0	0.0	0.0	0.0
TACGGAAGGT	0.0	0.0	0.0	0.0	0.0	0.0	0.0
TACGTAGGTG	0.0	0.0	0.0	0.0	0.0	0.0	0.0
TACGTAGGGG	0.0	0.0	0.0	0.0	0.0	0.0	0.0
TACGTAGGGG	0.0	0.0	0.0	0.0	0.0	0.0	0.0
TACGGAAGGT	0.0	0.0	0.0	0.0	0.0	0.0	0.0
TACGTAGGTG	0.0	0.0	0.0	0.0	0.0	0.0	0.0
TACGTAGGTG	0.0	0.0	0.0	0.0	0.0	0.0	0.0
TACGTATGGT	0.0	0.0	0.0	0.0	0.0	0.0	0.0
TACGTAGGGA	0.0	0.0	0.0	0.0	0.0	0.0	0.0
TACGTAGGGA	0.0	0.0	0.0	0.0	0.0	0.0	0.0
TACGTAGGGA	0.0	0.0	0.0	0.0	0.0	0.0	0.0
AACGTAGGTC	0.0	0.0	0.0	0.0	0.0	0.0	0.0
TACGTAGGGC	0.0	0.0	0.0	0.0	0.0	0.0	0.0
TACGTAGGTG	0.0	0.0	0.0	0.0	0.0	0.0	0.0
TACGTAGGGA	0.0	0.0	0.0	0.0	0.0	0.0	0.0
TACGTAAGGT	0.0	0.0	0.0	0.0	0.0	0.0	0.0
TACGTAGGTG	0.0	0.0	0.0	0.0	0.0	0.0	0.0
TACGTAGGTG	0.0	0.0	0.0	0.0	0.0	0.0	0.0
TACGTAGGGA	0.0	0.0	0.0	0.0	0.0	0.0	0.0

TACGTAGGGG	0.0	0.0	0.0	0.0	0.0	0.0	0.0
TACGGAGGAT	0.0	0.0	0.0	0.0	0.0	0.0	0.0
TACGGAGGAT	0.0	0.0	0.0	0.0	0.0	0.0	0.0
TACGGAAGGT	0.0	0.0	0.0	0.0	0.0	0.0	0.0
TACGTAGGGG	0.0	0.0	0.0	0.0	0.0	0.0	0.0
TACGTAGGAT	0.0	0.0	0.0	0.0	0.0	0.0	0.0
TACGTAGGGT	0.0	0.0	0.0	0.0	0.0	0.0	0.0
TACGTATGGT	0.0	0.0	0.0	0.0	0.0	0.0	0.0
TACGTAGGGG	0.0	0.0	0.0	0.0	0.0	0.0	0.0
TACGTAGGTG	0.0	0.0	0.0	0.0	0.0	0.0	0.0
TACGGAGGAT	0.0	0.0	0.0	0.0	0.0	0.0	0.0
TACGTAGGGG	0.0	0.0	0.0	0.0	0.0	0.0	0.0
TACGTAGGGG	0.0	0.0	0.0	0.0	0.0	0.0	0.0
TACGTATGGT	0.0	0.0	0.0	0.0	0.0	0.0	0.0
TACGTATGGA	0.0	0.0	0.0	0.0	0.0	0.0	0.0
TACGTATGGT	0.0	0.0	0.0	0.0	0.0	0.0	0.0
TACGGAAGGT	0.0	0.0	0.0	0.0	0.0	0.0	0.0
TACGTAGGGG	0.0	0.0	0.0	0.0	0.0	0.0	0.0
TACGTAGGGA	0.0	0.0	0.0	0.0	0.0	0.0	0.0
TACGTAGGGG	0.0	0.0	0.0	0.0	0.0	0.0	0.0
TACGTAGGGA	0.0	0.0	0.0	0.0	0.0	0.0	0.0
TACGTAGGGA	0.0	0.0	0.0	0.0	0.0	0.0	0.0
TACGGAGGAT	0.0	0.0	0.0	0.0	0.0	0.0	0.0
TACGTAGGGG	0.0	0.0	0.0	0.0	0.0	0.0	0.0
AACGTAGGTC	0.0	0.0	0.0	0.0	0.0	0.0	0.0
TACGTAGGTG	0.0	0.0	0.0	0.0	0.0	0.0	0.0
TACGTAGGTG	0.0	0.0	0.0	0.0	0.0	0.0	0.0
TACGTAGGGA	0.0	0.0	0.0	0.0	0.0	0.0	0.0
TACGTAGGTA	0.0	0.0	0.0	0.0	0.0	0.0	0.0
TACGTAGGGA	0.0	0.0	0.0	0.0	0.0	0.0	0.0
TACGGAGGAT	0.0	0.0	0.0	0.0	0.0	0.0	0.0
TACGTAGGGA	0.0	0.0	0.0	0.0	0.0	0.0	0.0
TACGTAGGTG	0.0	0.0	0.0	0.0	0.0	0.0	0.0
TACGTAGGTG	0.0	0.0	0.0	0.0	0.0	0.0	0.0
TACGGAAGAT	0.0	0.0	0.0	0.0	0.0	0.0	0.0
TACGTAGGGA	0.0	0.0	0.0	0.0	0.0	0.0	0.0
TACGGAGGAT	0.0	0.0	0.0	0.0	0.0	0.0	0.0
TACGTAGGGG	0.0	0.0	0.0	0.0	0.0	0.0	0.0
TACGGAGGAT	0.0	0.0	0.0	0.0	0.0	0.0	0.0
TACGTAGGGG	0.0	0.0	0.0	0.0	0.0	0.0	0.0
TACGTATGGA	0.0	0.0	0.0	0.0	0.0	0.0	0.0
TACGTAGGGA	0.0	0.0	0.0	0.0	0.0	0.0	0.0
TACGTAGGTG	0.0	0.0	0.0	0.0	0.0	0.0	0.0
TACGGAAGGT	0.0	0.0	0.0	0.0	0.0	0.0	0.0
TACGGAGGAT	0.0	0.0	0.0	0.0	0.0	0.0	0.0
TACGTAGGTG	0.0	0.0	0.0	0.0	0.0	0.0	0.0
TACGTATGGG	0.0	0.0	0.0	0.0	0.0	0.0	0.0

TACGTAGGGG	0.0	0.0	0.0	0.0	0.0	0.0	0.0
TACGTAGGGG	0.0	0.0	0.0	0.0	0.0	0.0	0.0
TACGTAGGGG	0.0	0.0	0.0	0.0	0.0	0.0	0.0
TACGTAGGTG	0.0	0.0	0.0	0.0	0.0	0.0	0.0
TACGTAGGTG	0.0	0.0	0.0	0.0	0.0	0.0	0.0
TACGGGGAT	0.0	0.0	0.0	0.0	0.0	0.0	0.0
TACGGAAGGT	0.0	0.0	0.0	0.0	0.0	0.0	0.0
TACGGAGGGT	0.0	0.0	0.0	0.0	0.0	0.0	0.0
TACGTAGGGA	0.0	0.0	0.0	0.0	0.0	0.0	0.0
TACGTAGGGA	0.0	0.0	0.0	0.0	0.0	0.0	0.0
TACGTAGGTG	0.0	0.0	0.0	0.0	0.0	0.0	0.0
TACGTAGGGG	0.0	0.0	0.0	0.0	0.0	0.0	0.0
TACGGAAGGT	0.0	0.0	0.0	0.0	0.0	0.0	0.0
TACGTAGGGT	0.0	0.0	0.0	0.0	0.0	0.0	0.0
TACGTAGGGG	0.0	0.0	0.0	0.0	0.0	0.0	0.0
TACGGAGGAT	0.0	0.0	0.0	0.0	0.0	0.0	0.0
TACGTAGGTG	0.0	0.0	0.0	0.0	0.0	0.0	0.0
AACGTAGGTC	0.0	0.0	0.0	0.0	0.0	0.0	0.0
TACGTAGGGG	0.0	0.0	0.0	0.0	0.0	0.0	0.0
TACGTAGGGA	0.0	0.0	0.0	0.0	0.0	0.0	0.0
TACGTAGGGA	0.0	0.0	0.0	0.0	0.0	0.0	0.0
TACGTAGGGA	0.0	0.0	0.0	0.0	0.0	0.0	0.0
TACGTAGGTG	0.0	0.0	0.0	0.0	0.0	0.0	0.0
TACGGAGGGT	0.0	0.0	0.0	0.0	0.0	0.0	0.0
TACGTAGGGA	0.0	0.0	0.0	0.0	0.0	0.0	0.0
AACGTAGGTG	0.0	0.0	0.0	0.0	0.0	0.0	0.0
TACGTAGGTG	0.0	0.0	0.0	0.0	0.0	0.0	0.0
AACGTAGGTG	0.0	0.0	0.0	0.0	0.0	0.0	0.0
TACGTAGGGT	0.0	0.0	0.0	0.0	0.0	0.0	0.0
TACGTAGGTG	0.0	0.0	0.0	0.0	0.0	0.0	0.0
TACGTATGGT	0.0	0.0	0.0	0.0	0.0	0.0	0.0
TACGTAGGGA	0.0	0.0	0.0	0.0	0.0	0.0	0.0
TACAGAGGGT	0.0	0.0	0.0	0.0	0.0	0.0	0.0
TACGTAGGGG	0.0	0.0	0.0	0.0	0.0	0.0	0.0
TACGTAGGGG	0.0	0.0	0.0	0.0	0.0	0.0	0.0
TACGTAGGGA	0.0	0.0	0.0	0.0	0.0	0.0	0.0
TACGTAGGGG	0.0	0.0	0.0	0.0	0.0	0.0	0.0
TACGTAGGGG	0.0	0.0	0.0	0.0	0.0	0.0	0.0
TACGTAGGGA	0.0	0.0	0.0	0.0	0.0	0.0	0.0
TACGTAGGTG	0.0	0.0	0.0	0.0	0.0	0.0	0.0
TACGTATGGT	0.0	0.0	0.0	0.0	0.0	0.0	0.0
TACGTAGGTG	0.0	0.0	0.0	0.0	0.0	0.0	0.0
TACGTAGGGA	0.0	0.0	0.0	0.0	0.0	0.0	0.0
TACGTAGGGA	0.0	0.0	0.0	0.0	0.0	0.0	0.0
TACGTAGGGA	0.0	0.0	0.0	0.0	0.0	0.0	0.0
TACGTAGGTG	0.0	0.0	0.0	0.0	0.0	0.0	0.0
TACGTAGGTG	0.0	0.0	0.0	0.0	0.0	0.0	0.0

TACGGAGGAT	0.0	0.0	0.0	0.0	0.0	0.0	0.0
TACGTAGGGA	0.0	0.0	0.0	0.0	0.0	0.0	0.0
TACGTATGGT	0.0	0.0	0.0	0.0	0.0	0.0	0.0
TACGTAGGTC	0.0	0.0	0.0	0.0	0.0	0.0	0.0
TACGTAGGTG	0.0	0.0	0.0	0.0	0.0	0.0	0.0
TACGTAGGGA	0.0	0.0	0.0	0.0	0.0	0.0	0.0
TACGGAGGAT	0.0	0.0	0.0	0.0	0.0	0.0	0.0
TACGTAGGGG	0.0	0.0	0.0	0.0	0.0	0.0	0.0
TACGTAGGTG	0.0	0.0	0.0	0.0	0.0	0.0	0.0
AACGTAGGTG	0.0	0.0	0.0	0.0	0.0	0.0	0.0
TACGTAGGGG	0.0	0.0	0.0	0.0	0.0	0.0	0.0
TACGTAGGGA	0.0	0.0	0.0	0.0	0.0	0.0	0.0
TACGTAGGTG	0.0	0.0	0.0	0.0	0.0	0.0	0.0
TACGTAGGTG	0.0	0.0	0.0	0.0	0.0	0.0	0.0
AACGTAGGTC	0.0	0.0	0.0	0.0	0.0	0.0	0.0
TACGTAGGGG	0.0	0.0	0.0	0.0	0.0	0.0	0.0
AACGTAGGTC	0.0	0.0	0.0	0.0	0.0	0.0	0.0
AACGTAGGGT	0.0	0.0	0.0	0.0	0.0	0.0	0.0
TACGTATGGT	0.0	0.0	0.0	0.0	0.0	0.0	0.0
TACGTAGGGG	0.0	0.0	0.0	0.0	0.0	0.0	0.0
TACGTAGGGG	0.0	0.0	0.0	0.0	0.0	0.0	0.0
AACGTAGGGT	0.0	0.0	0.0	0.0	0.0	0.0	0.0
TGCGTAGGGA	0.0	0.0	0.0	0.0	0.0	0.0	0.0
TACGTAGGGG	0.0	0.0	0.0	0.0	0.0	0.0	0.0
TACGTAGGTG	0.0	0.0	0.0	0.0	0.0	0.0	0.0
TACGTAGGTG	0.0	0.0	0.0	0.0	0.0	0.0	0.0
TACGTAGGTG	0.0	0.0	0.0	0.0	0.0	0.0	0.0
TACGTAGGGG	0.0	0.0	0.0	0.0	0.0	0.0	0.0
AACGTAGGTC	0.0	0.0	0.0	0.0	0.0	0.0	0.0
TACGTAGGGA	0.0	0.0	0.0	0.0	0.0	0.0	0.0
TACGTAGGGA	0.0	0.0	0.0	0.0	0.0	0.0	0.0
TACGGAGGGT	0.0	0.0	0.0	0.0	0.0	0.0	0.0
TACGGAAGAT	0.0	0.0	0.0	0.0	0.0	0.0	0.0
TACGTAGGGA	0.0	0.0	0.0	0.0	0.0	0.0	0.0
TACGTAGGGG	0.0	0.0	0.0	0.0	0.0	0.0	0.0
TACGGAGGGT	0.0	0.0	0.0	0.0	0.0	0.0	0.0
TACGGAGGAT	0.0	0.0	0.0	0.0	0.0	0.0	0.0
TACGTAGGGT	0.0	0.0	0.0	0.0	0.0	0.0	0.0
TACGTATGGG	0.0	0.0	0.0	0.0	0.0	0.0	0.0
TACGTAGGTG	0.0	0.0	0.0	0.0	0.0	0.0	0.0
TACGTATGGA	0.0	0.0	0.0	0.0	0.0	0.0	0.0
TACGTAGGGT	0.0	0.0	0.0	0.0	0.0	0.0	0.0
TACGTAGGGG	0.0	0.0	0.0	0.0	0.0	0.0	0.0
TACGTAGGTG	0.0	0.0	0.0	0.0	0.0	0.0	0.0
TACGTAGGGG	0.0	0.0	0.0	0.0	0.0	0.0	0.0
TACGTAGGGG	0.0	0.0	0.0	0.0	0.0	0.0	0.0
TACGTAGGGG	0.0	0.0	0.0	0.0	0.0	0.0	0.0

TACGGAAGAT	0.0	0.0	0.0	0.0	0.0	0.0	0.0
TACGTAGGTT	0.0	0.0	0.0	0.0	0.0	0.0	0.0
TACGTAGGTG	0.0	0.0	0.0	0.0	0.0	0.0	0.0
TACGTAGGTG	0.0	0.0	0.0	0.0	0.0	0.0	0.0
TACGGAGGGT	0.0	0.0	0.0	0.0	0.0	0.0	0.0
TACGTAGGGT	0.0	0.0	0.0	0.0	0.0	0.0	0.0
TACGTAGGGA	0.0	0.0	0.0	0.0	0.0	0.0	0.0
TACGTAGGGG	0.0	0.0	0.0	0.0	0.0	0.0	0.0
TACGTAGGGA	0.0	0.0	0.0	0.0	0.0	0.0	0.0
TACGTAGGGG	0.0	0.0	0.0	0.0	0.0	0.0	0.0
TACGGAGGAT	0.0	0.0	0.0	0.0	0.0	0.0	0.0
TACGTAGGGG	0.0	0.0	0.0	0.0	0.0	0.0	0.0
TACGTATGGA	0.0	0.0	0.0	0.0	0.0	0.0	0.0
TACGGAGGAT	0.0	0.0	0.0	0.0	0.0	0.0	0.0
TACGTAGGTG	0.0	0.0	0.0	0.0	0.0	0.0	0.0
TACGTAGGGG	0.0	0.0	0.0	0.0	0.0	0.0	0.0
TACGTAGGGG	0.0	0.0	0.0	0.0	0.0	0.0	0.0
TACGTAGGAT	0.0	0.0	0.0	0.0	0.0	0.0	0.0
TACGTAGGTG	0.0	0.0	0.0	0.0	0.0	0.0	0.0
TACGTAGGGG	0.0	0.0	0.0	0.0	0.0	0.0	0.0
TACGTAGGGA	0.0	0.0	0.0	0.0	0.0	0.0	0.0
TACGTAGGTG	0.0	0.0	0.0	0.0	0.0	0.0	0.0
TACGTAGGGG	0.0	0.0	0.0	0.0	0.0	0.0	0.0
TACGTAGGTG	0.0	0.0	0.0	0.0	0.0	0.0	0.0
TACGTAGGGG	0.0	0.0	0.0	0.0	0.0	0.0	0.0
TACGTAGGGG	0.0	0.0	0.0	0.0	0.0	0.0	0.0
TACGTAGGTG	0.0	0.0	0.0	0.0	0.0	0.0	0.0
TACGTAGGTG	0.0	0.0	0.0	0.0	0.0	0.0	0.0
TACGTAGGTG	0.0	0.0	0.0	0.0	0.0	0.0	0.0
TACGTAGGGG	0.0	0.0	0.0	0.0	0.0	0.0	0.0
TACGTAGGGG	0.0	0.0	0.0	0.0	0.0	0.0	0.0
TACGTAGGAG	0.0	0.0	0.0	0.0	0.0	0.0	0.0
TACGGAGGAT	0.0	0.0	0.0	0.0	0.0	0.0	0.0
TACGTAGGGG	0.0	0.0	0.0	0.0	0.0	0.0	0.0
AACGTAGGTG	0.0	0.0	0.0	0.0	0.0	0.0	0.0
TACGTAGGTT	0.0	0.0	0.0	0.0	0.0	0.0	0.0
TACGTAGGGG	0.0	0.0	0.0	0.0	0.0	0.0	0.0
TACGTAGGGC	0.0	0.0	0.0	0.0	0.0	0.0	0.0
AACGTAGGTC	0.0	0.0	0.0	0.0	0.0	0.0	0.0
TACGTAGGGG	0.0	0.0	0.0	0.0	0.0	0.0	0.0
TACGTAGGTG	0.0	0.0	0.0	0.0	0.0	0.0	0.0
TACGTAGGTG	0.0	0.0	0.0	0.0	0.0	0.0	0.0
TACGTAGGTG	0.0	0.0	0.0	0.0	0.0	0.0	0.0
TACGTAGGGG	0.0	0.0	0.0	0.0	0.0	0.0	0.0
TACGTAGGGT	0.0	0.0	0.0	0.0	0.0	0.0	0.0
TACGTAGGGG	0.0	0.0	0.0	0.0	0.0	0.0	0.0
TACGTAGGGA	0.0	0.0	0.0	0.0	0.0	0.0	0.0

TACGTAGGTG	0.0	0.0	0.0	0.0	0.0	0.0	0.0
TACGTAGGGA	0.0	0.0	0.0	0.0	0.0	0.0	0.0
AACGTAGGTC	0.0	0.0	0.0	0.0	0.0	0.0	0.0
TACAGAGGGT	0.0	0.0	0.0	0.0	0.0	0.0	0.0
TACGTAGGTG	0.0	0.0	0.0	0.0	0.0	0.0	0.0
TACGGAGGAT	0.0	0.0	0.0	0.0	0.0	0.0	0.0
AACGTAGGTC	0.0	0.0	0.0	0.0	0.0	0.0	0.0
TACGTAGGGG	0.0	0.0	0.0	0.0	0.0	0.0	0.0
TACGTAGGTG	0.0	0.0	0.0	0.0	0.0	0.0	0.0
TACGTAGGGA	0.0	0.0	0.0	0.0	0.0	0.0	0.0
TACGTAGGGA	0.0	0.0	0.0	0.0	0.0	0.0	0.0
TACGTAGGGG	0.0	0.0	0.0	0.0	0.0	0.0	0.0
TACGTAGGGG	0.0	0.0	0.0	0.0	0.0	0.0	0.0
AACGTAGGGT	0.0	0.0	0.0	0.0	0.0	0.0	0.0
TACGTAGGTG	0.0	0.0	0.0	0.0	0.0	0.0	0.0
TACGGAGGAT	0.0	0.0	0.0	0.0	0.0	0.0	0.0
TACGTAGGTG	0.0	0.0	0.0	0.0	0.0	0.0	0.0
AACGTAGGTC	0.0	0.0	0.0	0.0	0.0	0.0	0.0
TACGTAGGGA	0.0	0.0	0.0	0.0	0.0	0.0	0.0
TACGTAGGTG	0.0	0.0	0.0	0.0	0.0	0.0	0.0
TACGTAGGGG	0.0	0.0	0.0	0.0	0.0	0.0	0.0
TACGTAGGTG	0.0	0.0	0.0	0.0	0.0	0.0	0.0
TACGTAGGGG	0.0	0.0	0.0	0.0	0.0	0.0	0.0
TACGTAGGGA	0.0	0.0	0.0	0.0	0.0	0.0	0.0
TACGTAGGGG	0.0	0.0	0.0	0.0	0.0	0.0	0.0
TACGTAGGGG	0.0	0.0	0.0	0.0	0.0	0.0	0.0
TACGTAGGGG	0.0	0.0	0.0	0.0	0.0	0.0	0.0
TACGTAGGGG	0.0	0.0	0.0	0.0	0.0	0.0	0.0
TACGTAGGGG	0.0	0.0	0.0	0.0	0.0	0.0	0.0
TACGTATGGT	0.0	0.0	0.0	0.0	0.0	0.0	0.0
TACGTAGGTG	0.0	0.0	0.0	0.0	0.0	0.0	0.0
TACGTAGGGG	0.0	0.0	0.0	0.0	0.0	0.0	0.0
TACGTAGGTG	0.0	0.0	0.0	0.0	0.0	0.0	0.0
TACGTAGGGG	0.0	0.0	0.0	0.0	0.0	0.0	0.0
TACGTAGGGT	0.0	0.0	0.0	0.0	0.0	0.0	0.0
TACGTAGGGG	0.0	0.0	0.0	0.0	0.0	0.0	0.0
TACGTAGGGG	0.0	0.0	0.0	0.0	0.0	0.0	0.0
TACGTAGGTG	0.0	0.0	0.0	0.0	0.0	0.0	0.0
TACGTAGGGG	0.0	0.0	0.0	0.0	0.0	0.0	0.0
TACGTAGGTG	0.0	0.0	0.0	0.0	0.0	0.0	0.0
TACGTAGGGG	0.0	0.0	0.0	0.0	0.0	0.0	0.0
TACGAAGGGT	0.0	0.0	0.0	0.0	0.0	0.0	0.0
TACGTAGGTG	0.0	0.0	0.0	0.0	0.0	0.0	0.0
TACGTAGGGA	0.0	0.0	0.0	0.0	0.0	0.0	0.0
TACGTAGGGT	0.0	0.0	0.0	0.0	0.0	0.0	0.0
TACGTAGGTG	0.0	0.0	0.0	0.0	0.0	0.0	0.0
TACGTAGGTC	0.0	0.0	0.0	0.0	0.0	0.0	0.0

TACAGAGGAT	0.0	0.0	0.0	0.0	0.0	0.0	0.0
TACAGAGGTC	0.0	0.0	0.0	0.0	0.0	0.0	0.0
TACGTAAGGG	0.0	0.0	0.0	0.0	0.0	0.0	0.0
TACGTAGGGG	0.0	0.0	0.0	0.0	0.0	0.0	0.0
TACGTAGGGA	0.0	0.0	0.0	0.0	0.0	0.0	0.0
TACGGAGGAT	0.0	0.0	0.0	0.0	0.0	0.0	0.0
TACGTAGGGA	0.0	0.0	0.0	0.0	0.0	0.0	0.0
TACGTAGGGG	0.0	0.0	0.0	0.0	0.0	0.0	0.0
TACGTAGGGG	0.0	0.0	0.0	0.0	0.0	0.0	0.0
TACGTAGGGG	0.0	0.0	0.0	0.0	0.0	0.0	0.0
TACGGAGGAT	0.0	0.0	0.0	0.0	0.0	0.0	0.0
TACGGAGGAT	0.0	0.0	0.0	0.0	0.0	0.0	0.0
TACGGAGGAT	0.0	0.0	0.0	0.0	0.0	0.0	0.0
TACGTAGGGG	0.0	0.0	0.0	0.0	0.0	0.0	0.0
TACGTAGGTG	0.0	0.0	0.0	0.0	0.0	0.0	0.0
TACGTAGGGA	0.0	0.0	0.0	0.0	0.0	0.0	0.0
TACGTAGGGG	0.0	0.0	0.0	0.0	0.0	0.0	0.0
TACGGAGGAT	0.0	0.0	0.0	0.0	0.0	0.0	0.0
TACGTAGGTG	0.0	0.0	0.0	0.0	0.0	0.0	0.0
AACGTAGGTG	0.0	0.0	0.0	0.0	0.0	0.0	0.0
TACGTAGGGA	0.0	0.0	0.0	0.0	0.0	0.0	0.0
TACGGAGGAT	0.0	0.0	0.0	0.0	0.0	0.0	0.0
TACGTAGGGG	0.0	0.0	0.0	0.0	0.0	0.0	0.0
TACGTAGGGG	0.0	0.0	0.0	0.0	0.0	0.0	0.0
TACGTAGGTG	0.0	0.0	0.0	0.0	0.0	0.0	0.0
TACGTAGGGG	0.0	0.0	0.0	0.0	0.0	0.0	0.0
TACGTAGGTG	0.0	0.0	0.0	0.0	0.0	0.0	0.0
TACGTAGGGG	0.0	0.0	0.0	0.0	0.0	0.0	0.0
TACGTAGGGG	0.0	0.0	0.0	0.0	0.0	0.0	0.0
TACGTAGGGT	0.0	0.0	0.0	0.0	0.0	0.0	0.0
TACGTAGGTG	0.0	0.0	0.0	0.0	0.0	0.0	0.0
TACGTAGGGC	0.0	0.0	0.0	0.0	0.0	0.0	0.0
TACGTAGGGG	0.0	0.0	0.0	0.0	0.0	0.0	0.0
AACGTAGGGT	0.0	0.0	0.0	0.0	0.0	0.0	0.0
TACGTAGGGG	0.0	0.0	0.0	0.0	0.0	0.0	0.0
TACGTAGGGC	0.0	0.0	0.0	0.0	0.0	0.0	0.0
TACGTAGGGG	0.0	0.0	0.0	0.0	0.0	0.0	0.0
TACGTATGGT	0.0	0.0	0.0	0.0	0.0	0.0	0.0
TACGTAGGTG	0.0	0.0	0.0	0.0	0.0	0.0	0.0
TACGTAGGGC	0.0	0.0	0.0	0.0	0.0	0.0	0.0
TACGTAGGGT	0.0	0.0	0.0	0.0	0.0	0.0	0.0
TACGTATGGT	0.0	0.0	0.0	0.0	0.0	0.0	0.0
AACGTAGGGT	0.0	0.0	0.0	0.0	0.0	0.0	0.0
TACGTAGGTG	0.0	0.0	0.0	0.0	0.0	0.0	0.0
TACGTAGGGG	0.0	0.0	0.0	0.0	0.0	0.0	0.0
TACGTAGGTG	0.0	0.0	0.0	0.0	0.0	0.0	0.0
TACGTAGGGG	0.0	0.0	0.0	0.0	0.0	0.0	0.0

TACGTATGGT	0.0	0.0	0.0	0.0	0.0	0.0	0.0
TACGTAGGGG	0.0	0.0	0.0	0.0	0.0	0.0	0.0
TACGTAGGGA	0.0	0.0	0.0	0.0	0.0	0.0	0.0
TACGTAGGGG	0.0	0.0	0.0	0.0	0.0	0.0	0.0
TACGTAGGGG	0.0	0.0	0.0	0.0	0.0	0.0	0.0
TACGTAGGTG	0.0	0.0	0.0	0.0	0.0	0.0	0.0
TACGTAGGTG	0.0	0.0	0.0	0.0	0.0	0.0	0.0
TACGGAGGGT	0.0	0.0	0.0	0.0	0.0	0.0	0.0
TACGTATGGA	0.0	0.0	0.0	0.0	0.0	0.0	0.0
TACGTAGGGC	0.0	0.0	0.0	0.0	0.0	0.0	0.0
TACGTAGGGA	0.0	0.0	0.0	0.0	0.0	0.0	0.0
TACGTAGGGG	0.0	0.0	0.0	0.0	0.0	0.0	0.0
TACGTAGGGG	0.0	0.0	0.0	0.0	0.0	0.0	0.0
TACGTAGGGG	0.0	0.0	0.0	0.0	0.0	0.0	0.0
TACGGAAGGT	0.0	0.0	0.0	0.0	0.0	0.0	0.0
TACGTAGGTG	0.0	0.0	0.0	0.0	0.0	0.0	0.0
TACGTAGGGG	0.0	0.0	0.0	0.0	0.0	0.0	0.0
TACGTAGGTG	0.0	0.0	0.0	0.0	0.0	0.0	0.0
TACGTAGGTG	0.0	0.0	0.0	0.0	0.0	0.0	0.0
TACGTATGGA	0.0	0.0	0.0	0.0	0.0	0.0	0.0
TACGTAGGGG	0.0	0.0	0.0	0.0	0.0	0.0	0.0
TACGGAGGGT	0.0	0.0	0.0	0.0	0.0	0.0	0.0
AACGTAGGTG	0.0	0.0	0.0	0.0	0.0	0.0	0.0
TACGGAAGGT	0.0	0.0	0.0	0.0	0.0	0.0	0.0
TACGTAGGCG	0.0	0.0	0.0	0.0	0.0	0.0	0.0
TACGTAGGTG	0.0	0.0	0.0	0.0	0.0	0.0	0.0
TACGTAGGGG	0.0	0.0	0.0	0.0	0.0	0.0	0.0
TACGGAAGGT	0.0	0.0	0.0	0.0	0.0	0.0	0.0
TACGTAGGGA	0.0	0.0	0.0	0.0	0.0	0.0	0.0
AACGTAGGTG	0.0	0.0	0.0	0.0	0.0	0.0	0.0
TACGTATGGA	0.0	0.0	0.0	0.0	0.0	0.0	0.0
TACGGAGGGT	0.0	0.0	0.0	0.0	0.0	0.0	0.0
TACGTAGGGG	0.0	0.0	0.0	0.0	0.0	0.0	0.0
TACGTAGGGA	0.0	0.0	0.0	0.0	0.0	0.0	0.0
TACGTAGGGG	0.0	0.0	0.0	0.0	0.0	0.0	0.0
TACGTAGGGG	0.0	0.0	0.0	0.0	0.0	0.0	0.0
TACGTAGGTG	0.0	0.0	0.0	0.0	0.0	0.0	0.0
TACGTAGGTG	0.0	0.0	0.0	0.0	0.0	0.0	0.0
TACGTAGGGA	0.0	0.0	0.0	0.0	0.0	0.0	0.0
TACGTATGGA	0.0	0.0	0.0	0.0	0.0	0.0	0.0
TACGTAGGGA	0.0	0.0	0.0	0.0	0.0	0.0	0.0
TACGTAGGTG	0.0	0.0	0.0	0.0	0.0	0.0	0.0
TACGTAGGTG	0.0	0.0	0.0	0.0	0.0	0.0	0.0
TACGTAGGTC	0.0	0.0	0.0	0.0	0.0	0.0	0.0
TACGTAGGGG	0.0	0.0	0.0	0.0	0.0	0.0	0.0
TACGTAGGGA	0.0	0.0	0.0	0.0	0.0	0.0	0.0
TACGTAGGGC	0.0	0.0	0.0	0.0	0.0	0.0	0.0

TACGTAGGGA	0.0	0.0	0.0	0.0	0.0	0.0	0.0
TACGTAGGGA	0.0	0.0	0.0	0.0	0.0	0.0	0.0
TACGTAGGTG	0.0	0.0	0.0	0.0	0.0	0.0	0.0
TACGTAGGGG	0.0	0.0	0.0	0.0	0.0	0.0	0.0
TACGTAGGGT	0.0	0.0	0.0	0.0	0.0	0.0	0.0
TACGTAGGTC	0.0	0.0	0.0	0.0	0.0	0.0	0.0
TACGTATGGT	0.0	0.0	0.0	0.0	0.0	0.0	0.0
TACGTAGGTG	0.0	0.0	0.0	0.0	0.0	0.0	0.0
AACGTAGGGT	0.0	0.0	0.0	0.0	0.0	0.0	0.0
AACGTAGGGT	0.0	0.0	0.0	0.0	0.0	0.0	0.0
TACGTATGGT	0.0	0.0	0.0	0.0	0.0	0.0	0.0
TACGGAGGAT	0.0	0.0	0.0	0.0	0.0	0.0	0.0
TACGTAGGGG	0.0	0.0	0.0	0.0	0.0	0.0	0.0
TACGTAGGGG	0.0	0.0	0.0	0.0	0.0	0.0	0.0
TACGTAGGGC	0.0	0.0	0.0	0.0	0.0	0.0	0.0
TACGTAGGTG	0.0	0.0	0.0	0.0	0.0	0.0	0.0
TACGTAGGGG	0.0	0.0	0.0	0.0	0.0	0.0	0.0
TACGTATGGT	0.0	0.0	0.0	0.0	0.0	0.0	0.0
TACGTAGATG	0.0	0.0	0.0	0.0	0.0	0.0	0.0
TACGTAGGGG	0.0	0.0	0.0	0.0	0.0	0.0	0.0
TACGTAGGGG	0.0	0.0	0.0	0.0	0.0	0.0	0.0
TACGTAGGGG	0.0	0.0	0.0	0.0	0.0	0.0	0.0
TACGTATGGT	0.0	0.0	0.0	0.0	0.0	0.0	0.0
TACGTATGGA	0.0	0.0	0.0	0.0	0.0	0.0	0.0
AACGTAGGTC	0.0	0.0	0.0	0.0	0.0	0.0	0.0
TACGTATGGT	0.0	0.0	0.0	0.0	0.0	0.0	0.0
TACGTAGGGA	0.0	0.0	0.0	0.0	0.0	0.0	0.0
AACGTAGGGT	0.0	0.0	0.0	0.0	0.0	0.0	0.0
TACGTAGGGG	0.0	0.0	0.0	0.0	0.0	0.0	0.0
TACGTAGGGG	0.0	0.0	0.0	0.0	0.0	0.0	0.0
TACGTATGGA	0.0	0.0	0.0	0.0	0.0	0.0	0.0
TACGTAGGGG	0.0	0.0	0.0	0.0	0.0	0.0	0.0
AACGTAGGGC	0.0	0.0	0.0	0.0	0.0	0.0	0.0
TACGTAGGGG	0.0	0.0	0.0	0.0	0.0	0.0	0.0
TACGTAGGTC	0.0	0.0	0.0	0.0	0.0	0.0	0.0
TACGTATGGT	0.0	0.0	0.0	0.0	0.0	0.0	0.0
TACGTAGGTG	0.0	0.0	0.0	0.0	0.0	0.0	0.0
TACGTAGGGG	0.0	0.0	0.0	0.0	0.0	0.0	0.0
TACGTAGGGG	0.0	0.0	0.0	0.0	0.0	0.0	0.0
TACGTAGGTG	0.0	0.0	0.0	0.0	0.0	0.0	0.0
TACGTAGGTC	0.0	0.0	0.0	0.0	0.0	0.0	0.0
TACGTAGGGG	0.0	0.0	0.0	0.0	0.0	0.0	0.0
TACGTAGGGG	0.0	0.0	0.0	0.0	0.0	0.0	0.0
TACGTAGGTG	0.0	0.0	0.0	0.0	0.0	0.0	0.0
AACGTAGGTG	0.0	0.0	0.0	0.0	0.0	0.0	0.0
TACGTAGGTG	0.0	0.0	0.0	0.0	0.0	0.0	0.0
TACGTAGGGG	0.0	0.0	0.0	0.0	0.0	0.0	0.0

TACGTAGGG	0.0	0.0	0.0	0.0	0.0	0.0	0.0
TACGTAGGG	0.0	0.0	0.0	0.0	0.0	0.0	0.0
TACGTAGGTG	0.0	0.0	0.0	0.0	0.0	0.0	0.0
TACGTATGGA	0.0	0.0	0.0	0.0	0.0	0.0	0.0
TACGTAGGTC	0.0	0.0	0.0	0.0	0.0	0.0	0.0
TACGTATGGT	0.0	0.0	0.0	0.0	0.0	0.0	0.0
AACGTAGGG	0.0	0.0	0.0	0.0	0.0	0.0	0.0
TACGGAGGA	0.0	0.0	0.0	0.0	0.0	0.0	0.0
AACGTAGGT	0.0	0.0	0.0	0.0	0.0	0.0	0.0
TACGTAGGG	0.0	0.0	0.0	0.0	0.0	0.0	0.0
TACGTAGGG	0.0	0.0	0.0	0.0	0.0	0.0	0.0
TACGTAGGTG	0.0	0.0	0.0	0.0	0.0	0.0	0.0
AACGTAGGTC	0.0	0.0	0.0	0.0	0.0	0.0	0.0
TACGGAGGA	0.0	0.0	0.0	0.0	0.0	0.0	0.0
TACGTAGGTG	0.0	0.0	0.0	0.0	0.0	0.0	0.0
TACGTAGGAT	0.0	0.0	0.0	0.0	0.0	0.0	0.0
TACGTAGGTG	0.0	0.0	0.0	0.0	0.0	0.0	0.0
TACGTAGGTG	0.0	0.0	0.0	0.0	0.0	0.0	0.0
TACGTAGGTG	0.0	0.0	0.0	0.0	0.0	0.0	0.0
TACGTAGGG	0.0	0.0	0.0	0.0	0.0	0.0	0.0
TACGTAGGTG	0.0	0.0	0.0	0.0	0.0	0.0	0.0
TACGTATGGA	0.0	0.0	0.0	0.0	0.0	0.0	0.0
TACGTAGGTG	0.0	0.0	0.0	0.0	0.0	0.0	0.0
TACGGAGGA	0.0	0.0	0.0	0.0	0.0	0.0	0.0
TACGTAGGG	0.0	0.0	0.0	0.0	0.0	0.0	0.0
TACGGAGGA	0.0	0.0	0.0	0.0	0.0	0.0	0.0
TACGTAGGG	0.0	0.0	0.0	0.0	0.0	0.0	0.0
TACGTAGGG	0.0	0.0	0.0	0.0	0.0	0.0	0.0
TACGTAGGG	0.0	0.0	0.0	0.0	0.0	0.0	0.0
TACGGAGGA	0.0	0.0	0.0	0.0	0.0	0.0	0.0
TACGGAGGA	0.0	0.0	0.0	0.0	0.0	0.0	0.0
TACGTAGGG	0.0	0.0	0.0	0.0	0.0	0.0	0.0
TACGTAGGG	0.0	0.0	0.0	0.0	0.0	0.0	0.0
TACGTAGGG	0.0	0.0	0.0	0.0	0.0	0.0	0.0
TACGTAGAG	0.0	0.0	0.0	0.0	0.0	0.0	0.0
TACGTAGGG	0.0	0.0	0.0	0.0	0.0	0.0	0.0
TACCCGCGCC	0.0	0.0	0.0	0.0	0.0	0.0	0.0
TACGTAGGG	0.0	0.0	0.0	0.0	0.0	0.0	0.0
TACGGAGGG	0.0	0.0	0.0	0.0	0.0	0.0	0.0
TACGTAGGG	0.0	0.0	0.0	0.0	0.0	0.0	0.0
TACGTAGGG	0.0	0.0	0.0	0.0	0.0	0.0	0.0
TACGTAGGG	0.0	0.0	0.0	0.0	0.0	0.0	0.0
TACGTAGGG	0.0	0.0	0.0	0.0	0.0	0.0	0.0
TACGTATGGA	0.0	0.0	0.0	0.0	0.0	0.0	0.0
TACGTAGGTG	0.0	0.0	0.0	0.0	0.0	0.0	0.0
TACGTAGGTG	0.0	0.0	0.0	0.0	0.0	0.0	0.0
TACGTAGGG	0.0	0.0	0.0	0.0	0.0	0.0	0.0

TACGTAGGGA	0.0	0.0	0.0	0.0	0.0	0.0	0.0
TACGTAGGGA	0.0	0.0	0.0	0.0	0.0	0.0	0.0
TACGTAGGGG	0.0	0.0	0.0	0.0	0.0	0.0	0.0
TACGTAGGGA	0.0	0.0	0.0	0.0	0.0	0.0	0.0
TACGTAGGGG	0.0	0.0	0.0	0.0	0.0	0.0	0.0
TACGTAGGGG	0.0	0.0	0.0	0.0	0.0	0.0	0.0
TACGTAGGGG	0.0	0.0	0.0	0.0	0.0	0.0	0.0
TACGTAGGGG	0.0	0.0	0.0	0.0	0.0	0.0	0.0
TACGTAGGGG	0.0	0.0	0.0	0.0	0.0	0.0	0.0
TACGTAGGTG	0.0	0.0	0.0	0.0	0.0	0.0	0.0
TACGTAGGGG	0.0	0.0	0.0	0.0	0.0	0.0	0.0
TACGTAGGGG	0.0	0.0	0.0	0.0	0.0	0.0	0.0
TACGTAGGGG	0.0	0.0	0.0	0.0	0.0	0.0	0.0
TACGTATGGA	0.0	0.0	0.0	0.0	0.0	0.0	0.0
TACGTAGGAT	0.0	0.0	0.0	0.0	0.0	0.0	0.0
TACGTAGGTG	0.0	0.0	0.0	0.0	0.0	0.0	0.0
TACGTAGGTG	0.0	0.0	0.0	0.0	0.0	0.0	0.0
TACGTAGGGA	0.0	0.0	0.0	0.0	0.0	0.0	0.0
TACGTAGGGA	0.0	0.0	0.0	0.0	0.0	0.0	0.0
TACGTAGGGG	0.0	0.0	0.0	0.0	0.0	0.0	0.0
TACGTAGGTG	0.0	0.0	0.0	0.0	0.0	0.0	0.0
TACGTAGGGG	0.0	0.0	0.0	0.0	0.0	0.0	0.0
TACGTAGGGG	0.0	0.0	0.0	0.0	0.0	0.0	0.0
TACGTAGGGG	0.0	0.0	0.0	0.0	0.0	0.0	0.0
TACGTAGGGG	0.0	0.0	0.0	0.0	0.0	0.0	0.0
TACGTAGGTT	0.0	0.0	0.0	0.0	0.0	0.0	0.0
TACGTATGGA	0.0	0.0	0.0	0.0	0.0	0.0	0.0
TACGTAGGTG	0.0	0.0	0.0	0.0	0.0	0.0	0.0
TACGTAGGTA	0.0	0.0	0.0	0.0	0.0	0.0	0.0
TACGTAGGGA	0.0	0.0	0.0	0.0	0.0	0.0	0.0
TACGTAGGGG	0.0	0.0	0.0	0.0	0.0	0.0	0.0
TACGTAGGTG	0.0	0.0	0.0	0.0	0.0	0.0	0.0
TACGTAGGGG	0.0	0.0	0.0	0.0	0.0	0.0	0.0
TACGTAGGTG	0.0	0.0	0.0	0.0	0.0	0.0	0.0
TACGTAGGTG	0.0	0.0	0.0	0.0	0.0	0.0	0.0
TACGTAGGGG	0.0	0.0	0.0	0.0	0.0	0.0	0.0
TACGTAGGGG	0.0	0.0	0.0	0.0	0.0	0.0	0.0
TACGTAGGTG	0.0	0.0	0.0	0.0	0.0	0.0	0.0
AACGTAGGTC	0.0	0.0	0.0	0.0	0.0	0.0	0.0
TACGTAGGGG	0.0	0.0	0.0	0.0	0.0	0.0	0.0
TACGTATAGT	0.0	0.0	0.0	0.0	0.0	0.0	0.0
TACGTAGGTG	0.0	0.0	0.0	0.0	0.0	0.0	0.0
TACGTAGGTG	0.0	0.0	0.0	0.0	0.0	0.0	0.0
TACGTAGGGA	0.0	0.0	0.0	0.0	0.0	0.0	0.0
TACGTAGGGA	0.0	0.0	0.0	0.0	0.0	0.0	0.0
TACGTAGGTG	0.0	0.0	0.0	0.0	0.0	0.0	0.0
TACGTAGGTG	0.0	0.0	0.0	0.0	0.0	0.0	0.0

TACGTAGGGA	0.0	0.0	0.0	0.0	0.0	0.0	0.0
TACGTAGGGG	0.0	0.0	0.0	0.0	0.0	0.0	0.0
TACGGAGGAT	0.0	0.0	0.0	0.0	0.0	0.0	0.0
TACGTAGGTG	0.0	0.0	0.0	0.0	0.0	0.0	0.0
TACGTAGGGG	0.0	0.0	0.0	0.0	0.0	0.0	0.0
TACGTAGGGG	0.0	0.0	0.0	0.0	0.0	0.0	0.0
TACGGAGGAT	0.0	0.0	0.0	0.0	0.0	0.0	0.0
TACGTAGGGG	0.0	0.0	0.0	0.0	0.0	0.0	0.0
TACGTATGGT	0.0	0.0	0.0	0.0	0.0	0.0	0.0
TACGTATGGT	0.0	0.0	0.0	0.0	0.0	0.0	0.0
TACGTAGGGG	0.0	0.0	0.0	0.0	0.0	0.0	0.0
TACGTAGGGG	0.0	0.0	0.0	0.0	0.0	0.0	0.0
TACGTAGGTG	0.0	0.0	0.0	0.0	0.0	0.0	0.0
TACGTAGGGA	0.0	0.0	0.0	0.0	0.0	0.0	0.0
TACGTAGGGG	0.0	0.0	0.0	0.0	0.0	0.0	0.0
TACGTAGGTG	0.0	0.0	0.0	0.0	0.0	0.0	0.0
TACGTAGGGG	0.0	0.0	0.0	0.0	0.0	0.0	0.0
AACGTAGGGT	0.0	0.0	0.0	0.0	0.0	0.0	0.0
TACGTAGGGG	0.0	0.0	0.0	0.0	0.0	0.0	0.0
TACGTAGGGG	0.0	0.0	0.0	0.0	0.0	0.0	0.0
TACGTAGGGG	0.0	0.0	0.0	0.0	0.0	0.0	0.0
TACGTAGGGC	0.0	0.0	0.0	0.0	0.0	0.0	0.0
TACGTAGGGA	0.0	0.0	0.0	0.0	0.0	0.0	0.0
TACGTAGGGA	0.0	0.0	0.0	0.0	0.0	0.0	0.0
TACGGAGGAT	0.0	0.0	0.0	0.0	0.0	0.0	0.0
TACGTAGGTG	0.0	0.0	0.0	0.0	0.0	0.0	0.0
TACGTAGGGC	0.0	0.0	0.0	0.0	0.0	0.0	0.0
TACGTAGGTG	0.0	0.0	0.0	0.0	0.0	0.0	0.0
TACGTAGGGA	0.0	0.0	0.0	0.0	0.0	0.0	0.0
TACGTAGGGG	0.0	0.0	0.0	0.0	0.0	0.0	0.0
TACGTAGGTG	0.0	0.0	0.0	0.0	0.0	0.0	0.0
TACGTAGGGG	0.0	0.0	0.0	0.0	0.0	0.0	0.0
TACGTAGGGA	0.0	0.0	0.0	0.0	0.0	0.0	0.0
TACGGAGGGT	0.0	0.0	0.0	0.0	0.0	0.0	0.0
TACAGAGGGT	0.0	0.0	0.0	0.0	0.0	0.0	0.0
TACGTAGGGA	0.0	0.0	0.0	0.0	0.0	0.0	0.0
TACGTAGGGG	0.0	0.0	0.0	0.0	0.0	0.0	0.0
TACGGAGGGT	0.0	0.0	0.0	0.0	0.0	0.0	0.0
TACGTATGGA	0.0	0.0	0.0	0.0	0.0	0.0	0.0
TACGGAGGAT	0.0	0.0	0.0	0.0	0.0	0.0	0.0
TACGTAGGGG	0.0	0.0	0.0	0.0	0.0	0.0	0.0
TACGTAGGTG	0.0	0.0	0.0	0.0	0.0	0.0	0.0
TACGTATGGA	0.0	0.0	0.0	0.0	0.0	0.0	0.0
AACGTAGGTG	0.0	0.0	0.0	0.0	0.0	0.0	0.0
TACGTAGGGG	0.0	0.0	0.0	0.0	0.0	0.0	0.0
TACGTAGGGA	0.0	0.0	0.0	0.0	0.0	0.0	0.0
TACGTAGGTG	0.0	0.0	0.0	0.0	0.0	0.0	0.0

TACGTAGGGC	0.0	0.0	0.0	0.0	0.0	0.0	0.0
TACGTAGGGG	0.0	0.0	0.0	0.0	0.0	0.0	0.0
AACGTAGGTG	0.0	0.0	0.0	0.0	0.0	0.0	0.0
TACGTAGGAT	0.0	0.0	0.0	0.0	0.0	0.0	0.0
TACAGAGGGT	0.0	0.0	0.0	0.0	0.0	0.0	0.0
TACGTAGGTG	0.0	0.0	0.0	0.0	0.0	0.0	0.0
TACGTAGGTG	0.0	0.0	0.0	0.0	0.0	0.0	0.0
TACGTAGGTG	0.0	0.0	0.0	0.0	0.0	0.0	0.0
TACGGAGGGA	0.0	0.0	0.0	0.0	0.0	0.0	0.0
TACGGAGACT	0.0	0.0	0.0	0.0	0.0	0.0	0.0
TACGTAGGGG	0.0	0.0	0.0	0.0	0.0	0.0	0.0
TACGGAGGAT	0.0	0.0	0.0	0.0	0.0	0.0	0.0
TACGTAGGTG	0.0	0.0	0.0	0.0	0.0	0.0	0.0
TACGTAGGTG	0.0	0.0	0.0	0.0	0.0	0.0	0.0
TACGTAGGGA	0.0	0.0	0.0	0.0	0.0	0.0	0.0
TACGTAGGGG	0.0	0.0	0.0	0.0	0.0	0.0	0.0
AACGTAGGTC	0.0	0.0	0.0	0.0	0.0	0.0	0.0
TACGTAGGTG	0.0	0.0	0.0	0.0	0.0	0.0	0.0
TACGTATGGT	0.0	0.0	0.0	0.0	0.0	0.0	0.0
TACGTATGGT	0.0	0.0	0.0	0.0	0.0	0.0	0.0
TACGTAGGGA	0.0	0.0	0.0	0.0	0.0	0.0	0.0
TACGTAGGTG	0.0	0.0	0.0	0.0	0.0	0.0	0.0
TACGTAGGTG	0.0	0.0	0.0	0.0	0.0	0.0	0.0
TACGTAGGTG	0.0	0.0	0.0	0.0	0.0	0.0	0.0
TACGTATGGT	0.0	0.0	0.0	0.0	0.0	0.0	0.0
TACGTAGGTG	0.0	0.0	0.0	0.0	0.0	0.0	0.0
TACGGAGGGT	0.0	0.0	0.0	0.0	0.0	0.0	0.0
TACGTATGGT	0.0	0.0	0.0	0.0	0.0	0.0	0.0
TACGTAGGGG	0.0	0.0	0.0	0.0	0.0	0.0	0.0
TACGGAGGAT	0.0	0.0	0.0	0.0	0.0	0.0	0.0
TACGTAGGGA	0.0	0.0	0.0	0.0	0.0	0.0	0.0
TACGTAGGTG	0.0	0.0	0.0	0.0	0.0	0.0	0.0
TACGTATGGT	0.0	0.0	0.0	0.0	0.0	0.0	0.0
TACGTAGGGG	0.0	0.0	0.0	0.0	0.0	0.0	0.0
TACGTATGGT	0.0	0.0	0.0	0.0	0.0	0.0	0.0
TACGTAGGGA	0.0	0.0	0.0	0.0	0.0	0.0	0.0
TACGTAGGGG	0.0	0.0	0.0	0.0	0.0	0.0	0.0
TACGTAGGGA	0.0	0.0	0.0	0.0	0.0	0.0	0.0
TACGTAGGGG	0.0	0.0	0.0	0.0	0.0	0.0	0.0
TACGTATGGT	0.0	0.0	0.0	0.0	0.0	0.0	0.0
AACGTAGGGA	0.0	0.0	0.0	0.0	0.0	0.0	0.0
TACGTAGGGG	0.0	0.0	0.0	0.0	0.0	0.0	0.0
TACGTAGGGG	0.0	0.0	0.0	0.0	0.0	0.0	0.0
TACGTAGGGA	0.0	0.0	0.0	0.0	0.0	0.0	0.0
TACGTAGGTG	0.0	0.0	0.0	0.0	0.0	0.0	0.0
TACGTAGGGG	0.0	0.0	0.0	0.0	0.0	0.0	0.0
TACGTAGGGA	0.0	0.0	0.0	0.0	0.0	0.0	0.0

TACGTAGGGA	0.0	0.0	0.0	0.0	0.0	0.0	0.0
TACATAGGGG	0.0	0.0	0.0	0.0	0.0	0.0	0.0
TACGTAGGGA	0.0	0.0	0.0	0.0	0.0	0.0	0.0
TACGTAGGTG	0.0	0.0	0.0	0.0	0.0	0.0	0.0
TACGGAGGAT	0.0	0.0	0.0	0.0	0.0	0.0	0.0
TACGTAGGGG	0.0	0.0	0.0	0.0	0.0	0.0	0.0
TACGTAGGGG	0.0	0.0	0.0	0.0	0.0	0.0	0.0
TACGTAGGGG	0.0	0.0	0.0	0.0	0.0	0.0	0.0
TGCGTAGGGA	0.0	0.0	0.0	0.0	0.0	0.0	0.0
TACGTAGGGG	0.0	0.0	0.0	0.0	0.0	0.0	0.0
TACGGAGGAT	0.0	0.0	0.0	0.0	0.0	0.0	0.0
AACGTAGGTC	0.0	0.0	0.0	0.0	0.0	0.0	0.0
TACGTAGGTG	0.0	0.0	0.0	0.0	0.0	0.0	0.0
TACGTAGGGG	0.0	0.0	0.0	0.0	0.0	0.0	0.0
TACGTAGGGG	0.0	0.0	0.0	0.0	0.0	0.0	0.0
TACGTAGGTG	0.0	0.0	0.0	0.0	0.0	0.0	0.0
TACGTATGGT	0.0	0.0	0.0	0.0	0.0	0.0	0.0
TACGTAGGGG	0.0	0.0	0.0	0.0	0.0	0.0	0.0
TACGTAGGTG	0.0	0.0	0.0	0.0	0.0	0.0	0.0
TACGTAGGGA	0.0	0.0	0.0	0.0	0.0	0.0	0.0
TACGGAGGGT	0.0	0.0	0.0	0.0	0.0	0.0	0.0
TACGTATGGT	0.0	0.0	0.0	0.0	0.0	0.0	0.0
TACGTAGGGG	0.0	0.0	0.0	0.0	0.0	0.0	0.0
TACGTAGGGG	0.0	0.0	0.0	0.0	0.0	0.0	0.0
TACGTAGGGG	0.0	0.0	0.0	0.0	0.0	0.0	0.0
TACGTAGGGG	0.0	0.0	0.0	0.0	0.0	0.0	0.0
TACGTAGGGG	0.0	0.0	0.0	0.0	0.0	0.0	0.0
TACGGAGGAT	0.0	0.0	0.0	0.0	0.0	0.0	0.0
TACGTAGGGG	0.0	0.0	0.0	0.0	0.0	0.0	0.0
TACGTAGGGA	0.0	0.0	0.0	0.0	0.0	0.0	0.0
TACGTAGGTG	0.0	0.0	0.0	0.0	0.0	0.0	0.0
TACGTAGGGG	0.0	0.0	0.0	0.0	0.0	0.0	0.0
TACGTAGGGA	0.0	0.0	0.0	0.0	0.0	0.0	0.0
TACGTAGGGG	0.0	0.0	0.0	0.0	0.0	0.0	0.0
TACGAAGGGT	0.0	0.0	0.0	0.0	0.0	0.0	0.0
TACGTAGGGG	0.0	0.0	0.0	0.0	0.0	0.0	0.0
TACGTAGGGG	0.0	0.0	0.0	0.0	0.0	0.0	0.0
TACGGAGGGT	0.0	0.0	0.0	0.0	0.0	0.0	0.0
TACGTAGGGG	0.0	0.0	0.0	0.0	0.0	0.0	0.0
TACGGAGGAT	0.0	0.0	0.0	0.0	0.0	0.0	0.0
TACGTAGGGG	0.0	0.0	0.0	0.0	0.0	0.0	0.0
TACGTAGGTG	0.0	0.0	0.0	0.0	0.0	0.0	0.0
TACGGAGGAT	0.0	0.0	0.0	0.0	0.0	0.0	0.0
TACGTATGGA	0.0	0.0	0.0	0.0	0.0	0.0	0.0
TACGTAGGTG	0.0	0.0	0.0	0.0	0.0	0.0	0.0
TACGTAGGGG	0.0	0.0	0.0	0.0	0.0	0.0	0.0
TACGTATGGA	0.0	0.0	0.0	0.0	0.0	0.0	0.0

TACGTAGGGG	0.0	0.0	0.0	0.0	0.0	0.0	0.0
TACGTATGGT	0.0	0.0	0.0	0.0	0.0	0.0	0.0
TACATATGGA	0.0	0.0	0.0	0.0	0.0	0.0	0.0
TACGTATGGT	0.0	0.0	0.0	0.0	0.0	0.0	0.0
TACGTAGGGG	0.0	0.0	0.0	0.0	0.0	0.0	0.0
TACGTAGGGA	0.0	0.0	0.0	0.0	0.0	0.0	0.0
TACGTAGGGA	0.0	0.0	0.0	0.0	0.0	0.0	0.0
TACGTATGGA	0.0	0.0	0.0	0.0	0.0	0.0	0.0
TACGTAGGGG	0.0	0.0	0.0	0.0	0.0	0.0	0.0
TACGGAGGAT	0.0	0.0	0.0	0.0	0.0	0.0	0.0
AACGTAGGTG	0.0	0.0	0.0	0.0	0.0	0.0	0.0
TACGGAGGAT	0.0	0.0	0.0	0.0	0.0	0.0	0.0
TACGTAGGTG	0.0	0.0	0.0	0.0	0.0	0.0	0.0
TACGTAGGGG	0.0	0.0	0.0	0.0	0.0	0.0	0.0
TACGTAGGGG	0.0	0.0	0.0	0.0	0.0	0.0	0.0
TACGTAGGGG	0.0	0.0	0.0	0.0	0.0	0.0	0.0
TACGTAGGTG	0.0	0.0	0.0	0.0	0.0	0.0	0.0
TACGTAGGGG	0.0	0.0	0.0	0.0	0.0	0.0	0.0
TACGGAGGAT	0.0	0.0	0.0	0.0	0.0	0.0	0.0
TACGTAGGTG	0.0	0.0	0.0	0.0	0.0	0.0	0.0
TACGTAGGGA	0.0	0.0	0.0	0.0	0.0	0.0	0.0
TACGTAGGTG	0.0	0.0	0.0	0.0	0.0	0.0	0.0
TACGTAGGGG	0.0	0.0	0.0	0.0	0.0	0.0	0.0
TACGTAGGTG	0.0	0.0	0.0	0.0	0.0	0.0	0.0
TACGGAGGAT	0.0	0.0	0.0	0.0	0.0	0.0	0.0
TACGTAGGGG	0.0	0.0	0.0	0.0	0.0	0.0	0.0
TACGTAGGGA	0.0	0.0	0.0	0.0	0.0	0.0	0.0
TACGTAGGTG	0.0	0.0	0.0	0.0	0.0	0.0	0.0
TACGTAGGTG	0.0	0.0	0.0	0.0	0.0	0.0	0.0
TACGTAGGTG	0.0	0.0	0.0	0.0	0.0	0.0	0.0
TACGTATGGA	0.0	0.0	0.0	0.0	0.0	0.0	0.0
TACGTAGGTG	0.0	0.0	0.0	0.0	0.0	0.0	0.0
TACGGAGGAT	0.0	0.0	0.0	0.0	0.0	0.0	0.0
TACGTAGGGG	0.0	0.0	0.0	0.0	0.0	0.0	0.0
TACGTAGGGG	0.0	0.0	0.0	0.0	0.0	0.0	0.0
TACGGAAGGT	0.0	0.0	0.0	0.0	0.0	0.0	0.0
TACGTAGGTG	0.0	0.0	0.0	0.0	0.0	0.0	0.0
TACGTAGGGG	0.0	0.0	0.0	0.0	0.0	0.0	0.0
TACGTAGGAT	0.0	0.0	0.0	0.0	0.0	0.0	0.0
TACGTAGGTG	0.0	0.0	0.0	0.0	0.0	0.0	0.0
TACGTAGGTG	0.0	0.0	0.0	0.0	0.0	0.0	0.0
TACGTAGGGG	0.0	0.0	0.0	0.0	0.0	0.0	0.0
TACGTAGGTA	0.0	0.0	0.0	0.0	0.0	0.0	0.0
TACGTAGGGT	0.0	0.0	0.0	0.0	0.0	0.0	0.0
TACGTAGGGA	0.0	0.0	0.0	0.0	0.0	0.0	0.0
TACGTAGGGG	0.0	0.0	0.0	0.0	0.0	0.0	0.0
TACGTAGGGG	0.0	0.0	0.0	0.0	0.0	0.0	0.0

TACGTAGGTG	0.0	0.0	0.0	0.0	0.0	0.0	0.0
AACGTAGGTC	0.0	0.0	0.0	0.0	0.0	0.0	0.0
TACGTAGGGA	0.0	0.0	0.0	0.0	0.0	0.0	0.0
TACGTAGGTG	0.0	0.0	0.0	0.0	0.0	0.0	0.0
TACGTATGGG	0.0	0.0	0.0	0.0	0.0	0.0	0.0
TACGTAGGGC	0.0	0.0	0.0	0.0	0.0	0.0	0.0
TACGTAGGGC	0.0	0.0	0.0	0.0	0.0	0.0	0.0
TACGTAGGGC	0.0	0.0	0.0	0.0	0.0	0.0	0.0
TACGTAGGTG	0.0	0.0	0.0	0.0	0.0	0.0	0.0
CACGTAGGGC	0.0	0.0	0.0	0.0	0.0	0.0	0.0
TACGTAGGTG	0.0	0.0	0.0	0.0	0.0	0.0	0.0
TACGGAGGAT	0.0	0.0	0.0	0.0	0.0	0.0	0.0
AACGTAGGTC	0.0	0.0	0.0	0.0	0.0	0.0	0.0
TACGTAGGTG	0.0	0.0	0.0	0.0	0.0	0.0	0.0
TACGTAGGTG	0.0	0.0	0.0	0.0	0.0	0.0	0.0
TACGTAGGGC	0.0	0.0	0.0	0.0	0.0	0.0	0.0
TACGTAGGGC	0.0	0.0	0.0	0.0	0.0	0.0	0.0
AACGTAGGTC	0.0	0.0	0.0	0.0	0.0	0.0	0.0
TACGTAGGTG	0.0	0.0	0.0	0.0	0.0	0.0	0.0
TACGTAGGGC	0.0	0.0	0.0	0.0	0.0	0.0	0.0
TACGTAGGGT	0.0	0.0	0.0	0.0	0.0	0.0	0.0
TACGTAGGTG	0.0	0.0	0.0	0.0	0.0	0.0	0.0
TACGTAGGGA	0.0	0.0	0.0	0.0	0.0	0.0	0.0
TACGGAGGAT	0.0	0.0	0.0	0.0	0.0	0.0	0.0
TACGTAGGTG	0.0	0.0	0.0	0.0	0.0	0.0	0.0
TACGTAGGGC	0.0	0.0	0.0	0.0	0.0	0.0	0.0
TACGTATGGA	0.0	0.0	0.0	0.0	0.0	0.0	0.0
TACGTAGGGA	0.0	0.0	0.0	0.0	0.0	0.0	0.0
TACGTAGGGC	0.0	0.0	0.0	0.0	0.0	0.0	0.0
TACGTAGGGC	0.0	0.0	0.0	0.0	0.0	0.0	0.0
TACGTAGGGA	0.0	0.0	0.0	0.0	0.0	0.0	0.0
TACGTAGGGC	0.0	0.0	0.0	0.0	0.0	0.0	0.0
TACGTAGGTG	0.0	0.0	0.0	0.0	0.0	0.0	0.0
TACGTAGGTG	0.0	0.0	0.0	0.0	0.0	0.0	0.0
TACGTAGGGA	0.0	0.0	0.0	0.0	0.0	0.0	0.0
TACGTAGGGC	0.0	0.0	0.0	0.0	0.0	0.0	0.0
TACGTAGGTG	0.0	0.0	0.0	0.0	0.0	0.0	0.0
TACGTAGGGC	0.0	0.0	0.0	0.0	0.0	0.0	0.0
TACGGAGGAT	0.0	0.0	0.0	0.0	0.0	0.0	0.0
TACAGAGGGT	0.0	0.0	0.0	0.0	0.0	0.0	0.0
TACGTAGGTG	0.0	0.0	0.0	0.0	0.0	0.0	0.0
TACGTAGGTG	0.0	0.0	0.0	0.0	0.0	0.0	0.0
TACGGAGGGT	0.0	0.0	0.0	0.0	0.0	0.0	0.0
TACGTAGGTG	0.0	0.0	0.0	0.0	0.0	0.0	0.0
TACGTAGGGC	0.0	0.0	0.0	0.0	0.0	0.0	0.0
TACGTAGGTG	0.0	0.0	0.0	0.0	0.0	0.0	0.0
TACGTAGGGC	0.0	0.0	0.0	0.0	0.0	0.0	0.0

TACGTAGGTG	0.0	0.0	0.0	0.0	0.0	0.0	0.0
TACGTAGGTG	0.0	0.0	0.0	0.0	0.0	0.0	0.0
TACGTAGGGG	0.0	0.0	0.0	0.0	0.0	0.0	0.0
TACGTAGGGA	0.0	0.0	0.0	0.0	0.0	0.0	0.0
TACGTAGGGG	0.0	0.0	0.0	0.0	0.0	0.0	0.0
TACGTAGGGG	0.0	0.0	0.0	0.0	0.0	0.0	0.0
TACGTAGGTG	0.0	0.0	0.0	0.0	0.0	0.0	0.0
TACGTAGGGG	0.0	0.0	0.0	0.0	0.0	0.0	0.0
TACGTAAGGT	0.0	0.0	0.0	0.0	0.0	0.0	0.0
TACGTAGGTG	0.0	0.0	0.0	0.0	0.0	0.0	0.0
TACGTAGGGT	0.0	0.0	0.0	0.0	0.0	0.0	0.0
TACGTATGGA	0.0	0.0	0.0	0.0	0.0	0.0	0.0
TACGTAAGGT	0.0	0.0	0.0	0.0	0.0	0.0	0.0
TACGGAGGGT	0.0	0.0	0.0	0.0	0.0	0.0	0.0
TACGGAGGAT	0.0	0.0	0.0	0.0	0.0	0.0	0.0
TACGGAGGGT	0.0	0.0	0.0	0.0	0.0	0.0	0.0
TACAGAGGAT	0.0	0.0	0.0	0.0	0.0	0.0	0.0
TACGGAGGGT	0.0	0.0	0.0	0.0	0.0	0.0	0.0
TACGTAGGGA	0.0	0.0	0.0	0.0	0.0	0.0	0.0
TACGGAGGAT	0.0	0.0	0.0	0.0	0.0	0.0	0.0
TACGTAGGTG	0.0	0.0	0.0	0.0	0.0	0.0	0.0
TACGTATGGG	0.0	0.0	0.0	0.0	0.0	0.0	0.0
TACGTAGGTG	0.0	0.0	0.0	0.0	0.0	0.0	0.0
TACGTAGGGG	0.0	0.0	0.0	0.0	0.0	0.0	0.0
TACGTAGGGG	0.0	0.0	0.0	0.0	0.0	0.0	0.0
TACGTAGGGG	0.0	0.0	0.0	0.0	0.0	0.0	0.0
TACGTATGGT	0.0	0.0	0.0	0.0	0.0	0.0	0.0
TACGTAGGGA	0.0	0.0	0.0	0.0	0.0	0.0	0.0
TACGTAGGGG	0.0	0.0	0.0	0.0	0.0	0.0	0.0
TACGTAGGGA	0.0	0.0	0.0	0.0	0.0	0.0	0.0
TACGTAGGGA	0.0	0.0	0.0	0.0	0.0	0.0	0.0
TACGTAGGGG	0.0	0.0	0.0	0.0	0.0	0.0	0.0
TACGTAGGGA	0.0	0.0	0.0	0.0	0.0	0.0	0.0
TACGTAGGGA	0.0	0.0	0.0	0.0	0.0	0.0	0.0
TACGTATGGG	0.0	0.0	0.0	0.0	0.0	0.0	0.0
TACGTATGGT	0.0	0.0	0.0	0.0	0.0	0.0	0.0
TACGGAGGAT	0.0	0.0	0.0	0.0	0.0	0.0	0.0
TACGTAGGTG	0.0	0.0	0.0	0.0	0.0	0.0	0.0
AACGTAGGTC	0.0	0.0	0.0	0.0	0.0	0.0	0.0
TACGGAGGAT	0.0	0.0	0.0	0.0	0.0	0.0	0.0
TACGTAGGGG	0.0	0.0	0.0	0.0	0.0	0.0	0.0
TACGTAGGGA	0.0	0.0	0.0	0.0	0.0	0.0	0.0
AACGTAGGTC	0.0	0.0	0.0	0.0	0.0	0.0	0.0
TACGTAGGGA	0.0	0.0	0.0	0.0	0.0	0.0	0.0
TACGTAGGTG	0.0	0.0	0.0	0.0	0.0	0.0	0.0
TACGTAGGGA	0.0	0.0	0.0	0.0	0.0	0.0	0.0
TACGTAGGTG	0.0	0.0	0.0	0.0	0.0	0.0	0.0

TACGTAGGGG	0.0	0.0	0.0	0.0	0.0	0.0	0.0
TACGTAGGTG	0.0	0.0	0.0	0.0	0.0	0.0	0.0
TACGTAGGGG	0.0	0.0	0.0	0.0	0.0	0.0	0.0
TACGTAGGGG	0.0	0.0	0.0	0.0	0.0	0.0	0.0
AACGTAGGTC	0.0	0.0	0.0	0.0	0.0	0.0	0.0
TACGTAGGTG	0.0	0.0	0.0	0.0	0.0	0.0	0.0
TACGTAGGTG	0.0	0.0	0.0	0.0	0.0	0.0	0.0
TACGTAGGTG	0.0	0.0	0.0	0.0	0.0	0.0	0.0
TACGTATGGT	0.0	0.0	0.0	0.0	0.0	0.0	0.0
TACGTAGGGG	0.0	0.0	0.0	0.0	0.0	0.0	0.0
TACGTATGGA	0.0	0.0	0.0	0.0	0.0	0.0	0.0
TACGGAGGAT	0.0	0.0	0.0	0.0	0.0	0.0	0.0
TACGTAGGGG	0.0	0.0	0.0	0.0	0.0	0.0	0.0
TACGTATGGT	0.0	0.0	0.0	0.0	0.0	0.0	0.0
CACCGGCAGC	0.0	0.0	0.0	0.0	0.0	0.0	0.0
TACGTAGGTG	0.0	0.0	0.0	0.0	0.0	0.0	0.0
TACGTATGGG	0.0	0.0	0.0	0.0	0.0	0.0	0.0
TACGTATGGA	0.0	0.0	0.0	0.0	0.0	0.0	0.0
TACGTAGGGT	0.0	0.0	0.0	0.0	0.0	0.0	0.0
TACGTAGGGC	0.0	0.0	0.0	0.0	0.0	0.0	0.0
TACGGAGGAT	0.0	0.0	0.0	0.0	0.0	0.0	0.0
TACGTAGGGG	0.0	0.0	0.0	0.0	0.0	0.0	0.0
TACGGAGGAT	0.0	0.0	0.0	0.0	0.0	0.0	0.0
TACGTATGTC	0.0	0.0	0.0	0.0	0.0	0.0	0.0
TACGTAGGGA	0.0	0.0	0.0	0.0	0.0	0.0	0.0
TACGTAGGTG	0.0	0.0	0.0	0.0	0.0	0.0	0.0
TACGTAGGTG	0.0	0.0	0.0	0.0	0.0	0.0	0.0
TACGTAGGTG	0.0	0.0	0.0	0.0	0.0	0.0	0.0
TACGTAGGGG	0.0	0.0	0.0	0.0	0.0	0.0	0.0
TACGTAGGTG	0.0	0.0	0.0	0.0	0.0	0.0	0.0
TACGTAGGGG	0.0	0.0	0.0	0.0	0.0	0.0	0.0
TACGTATGGA	0.0	0.0	0.0	0.0	0.0	0.0	0.0
TACGTATGGA	0.0	0.0	0.0	0.0	0.0	0.0	0.0
TACGTAGGGG	0.0	0.0	0.0	0.0	0.0	0.0	0.0
TACGTAGGGA	0.0	0.0	0.0	0.0	0.0	0.0	0.0
TACGTAGGTG	0.0	0.0	0.0	0.0	0.0	0.0	0.0
TACGTAGGGT	0.0	0.0	0.0	0.0	0.0	0.0	0.0
TACGTATGTC	0.0	0.0	0.0	0.0	0.0	0.0	0.0
TACGTAGGGG	0.0	0.0	0.0	0.0	0.0	0.0	0.0
TACGTAGGGC	0.0	0.0	0.0	0.0	0.0	0.0	0.0
TACGGAGGGT	0.0	0.0	0.0	0.0	0.0	0.0	0.0
TACGTAGGGG	0.0	0.0	0.0	0.0	0.0	0.0	0.0
TACGGAAGGT	0.0	0.0	0.0	0.0	0.0	0.0	0.0
TACGTAGGGA	0.0	0.0	0.0	0.0	0.0	0.0	0.0
TACGTAGGTG	0.0	0.0	0.0	0.0	0.0	0.0	0.0
TACGTAGGGG	0.0	0.0	0.0	0.0	0.0	0.0	0.0
TACGTAGGGG	0.0	0.0	0.0	0.0	0.0	0.0	0.0

TACGTAGGGG	0.0	0.0	0.0	0.0	0.0	0.0	0.0
TACGTAGGGT	0.0	0.0	0.0	0.0	0.0	0.0	0.0
TACGTAGGGG	0.0	0.0	0.0	0.0	0.0	0.0	0.0
TACGGAGGAT	0.0	0.0	0.0	0.0	0.0	0.0	0.0
TACGGAGACT	0.0	0.0	0.0	0.0	0.0	0.0	0.0
TACGTAGGGA	0.0	0.0	0.0	0.0	0.0	0.0	0.0
TACGTAGGGG	0.0	0.0	0.0	0.0	0.0	0.0	0.0
TACGTAGGGA	0.0	0.0	0.0	0.0	0.0	0.0	0.0
TACGTAGGGC	0.0	0.0	0.0	0.0	0.0	0.0	0.0
TACGTAGGGG	0.0	0.0	0.0	0.0	0.0	0.0	0.0
TACGTAGGTG	0.0	0.0	0.0	0.0	0.0	0.0	0.0
TACGTAGGGG	0.0	0.0	0.0	0.0	0.0	0.0	0.0
TACGAAGGGA	0.0	0.0	0.0	0.0	0.0	0.0	0.0
TACGTAGGTG	0.0	0.0	0.0	0.0	0.0	0.0	0.0
TACGTAGGGC	0.0	0.0	0.0	0.0	0.0	0.0	0.0
TACGTAGGGT	0.0	0.0	0.0	0.0	0.0	0.0	0.0
TACGTAGGTG	0.0	0.0	0.0	0.0	0.0	0.0	0.0
TACGTAGGTG	0.0	0.0	0.0	0.0	0.0	0.0	0.0
TACGTAGGGG	0.0	0.0	0.0	0.0	0.0	0.0	0.0
TACGGAGGAT	0.0	0.0	0.0	0.0	0.0	0.0	0.0
TACGAAGGGC	0.0	0.0	0.0	0.0	0.0	0.0	0.0
TACGTAGGTG	0.0	0.0	0.0	0.0	0.0	0.0	0.0
TACGTAGGTG	0.0	0.0	0.0	0.0	0.0	0.0	0.0
TACGTAGGGG	0.0	0.0	0.0	0.0	0.0	0.0	0.0
TACGTAGGGG	0.0	0.0	0.0	0.0	0.0	0.0	0.0
TACGTAGGGG	0.0	0.0	0.0	0.0	0.0	0.0	0.0
TACGTAGGGG	0.0	0.0	0.0	0.0	0.0	0.0	0.0
TACGTAGGGA	0.0	0.0	0.0	0.0	0.0	0.0	0.0
TACGTAGGGG	0.0	0.0	0.0	0.0	0.0	0.0	0.0
TATTTCTTTG	0.0	0.0	0.0	0.0	0.0	0.0	0.0
TACGTAGGGG	0.0	0.0	0.0	0.0	0.0	0.0	0.0
AACGTAGGTC	0.0	0.0	0.0	0.0	0.0	0.0	0.0
TACGCAGGGA	0.0	0.0	0.0	0.0	0.0	0.0	0.0
TACGTAGGGG	0.0	0.0	0.0	0.0	0.0	0.0	0.0
TACGTAGGTG	0.0	0.0	0.0	0.0	0.0	0.0	0.0
TACGTAGGTG	0.0	0.0	0.0	0.0	0.0	0.0	0.0
TACGGAAGGT	0.0	0.0	0.0	0.0	0.0	0.0	0.0
TACGTAGGTG	0.0	0.0	0.0	0.0	0.0	0.0	0.0
TACGTATGGA	0.0	0.0	0.0	0.0	0.0	0.0	0.0
TACGTAGGGA	0.0	0.0	0.0	0.0	0.0	0.0	0.0
TACGTAGGGG	0.0	0.0	0.0	0.0	0.0	0.0	0.0
TACGTAGGGG	0.0	0.0	0.0	0.0	0.0	0.0	0.0
TACGGAAGGT	0.0	0.0	0.0	0.0	0.0	0.0	0.0
AACGTAGGGT	0.0	0.0	0.0	0.0	0.0	0.0	0.0
TACGTAGGTG	0.0	0.0	0.0	0.0	0.0	0.0	0.0
TACGGAAGGT	0.0	0.0	0.0	0.0	0.0	0.0	0.0
TACGTAGGGA	0.0	0.0	0.0	0.0	0.0	0.0	0.0

TACGTAGGGT	0.0	0.0	0.0	0.0	0.0	0.0	0.0
TACGGAAGGT	0.0	0.0	0.0	0.0	0.0	0.0	0.0
TACGTAGGTG	0.0	0.0	0.0	0.0	0.0	0.0	0.0
TACGGAAGGT	0.0	0.0	0.0	0.0	0.0	0.0	0.0
TACGTATGGG	0.0	0.0	0.0	0.0	0.0	0.0	0.0
TACGTAGGTG	0.0	0.0	0.0	0.0	0.0	0.0	0.0
TACGTAGGTT	0.0	0.0	0.0	0.0	0.0	0.0	0.0
TACGTAGGGG	0.0	0.0	0.0	0.0	0.0	0.0	0.0
TACGTAGGGG	0.0	0.0	0.0	0.0	0.0	0.0	0.0
TACGTAGGTG	0.0	0.0	0.0	0.0	0.0	0.0	0.0
TACGTATGGG	0.0	0.0	0.0	0.0	0.0	0.0	0.0
TACGTAGGGC	0.0	0.0	0.0	0.0	0.0	0.0	0.0
TACGTAGGGG	0.0	0.0	0.0	0.0	0.0	0.0	0.0
TACGTAGGAT	0.0	0.0	0.0	0.0	0.0	0.0	0.0
TACGGAAGGT	0.0	0.0	0.0	0.0	0.0	0.0	0.0
TACGTAGGGC	0.0	0.0	0.0	0.0	0.0	0.0	0.0
TACGTATGGA	0.0	0.0	0.0	0.0	0.0	0.0	0.0
TACGGAAGGT	0.0	0.0	0.0	0.0	0.0	0.0	0.0
TACGTAGGGT	0.0	0.0	0.0	0.0	0.0	0.0	0.0
TACGTAGGGG	0.0	0.0	0.0	0.0	0.0	0.0	0.0
TACGGAAGGT	0.0	0.0	0.0	0.0	0.0	0.0	0.0
TACGTAGGTC	0.0	0.0	0.0	0.0	0.0	0.0	0.0
TACGTAGGGT	0.0	0.0	0.0	0.0	0.0	0.0	0.0
GACAGAGGAT	0.0	0.0	0.0	0.0	0.0	0.0	0.0
TACGGAAGGT	0.0	0.0	0.0	0.0	0.0	0.0	0.0
TACGTAGGGG	0.0	0.0	0.0	0.0	0.0	0.0	0.0
TACGTAGGGT	0.0	0.0	0.0	0.0	0.0	0.0	0.0
TACGTAGGGC	0.0	0.0	0.0	0.0	0.0	0.0	0.0
TACGTAGGTC	0.0	0.0	0.0	0.0	0.0	0.0	0.0
TACGGAGGGC	0.0	0.0	0.0	0.0	0.0	0.0	0.0
TACGTAGGGG	0.0	0.0	0.0	0.0	0.0	0.0	0.0
TACGTAGGTC	0.0	0.0	0.0	0.0	0.0	0.0	0.0
TACGTAGGGT	0.0	0.0	0.0	0.0	0.0	0.0	0.0
TACGTAGGTG	0.0	0.0	0.0	0.0	0.0	0.0	0.0
TACGGAAGGT	0.0	0.0	0.0	0.0	0.0	0.0	0.0
TACGTAGGGG	0.0	0.0	0.0	0.0	0.0	0.0	0.0
TACGTAGGTC	0.0	0.0	0.0	0.0	0.0	0.0	0.0
TACGGAGGAT	0.0	0.0	0.0	0.0	0.0	0.0	0.0
TACGGAGGGT	0.0	0.0	0.0	0.0	0.0	0.0	0.0
TACGTAGGGG	0.0	0.0	0.0	0.0	0.0	0.0	0.0
TACGTAGGTG	0.0	0.0	0.0	0.0	0.0	0.0	0.0
TACGTATGGG	0.0	0.0	0.0	0.0	0.0	0.0	0.0
GACAGAGGAT	0.0	0.0	0.0	0.0	0.0	0.0	0.0
TACGTAGGTG	0.0	0.0	0.0	0.0	0.0	0.0	0.0
TACGTAGGTA	0.0	0.0	0.0	0.0	0.0	0.0	0.0
TACGTAGGGG	0.0	0.0	0.0	0.0	0.0	0.0	0.0
TACGGAAGGT	0.0	0.0	0.0	0.0	0.0	0.0	0.0

TACGTAGGGA	0.0	0.0	0.0	0.0	0.0	0.0	0.0
TACGGAAGGT	0.0	0.0	0.0	0.0	0.0	0.0	0.0
TACGTATGGT	0.0	0.0	0.0	0.0	0.0	0.0	0.0
TACGTAGGGG	0.0	0.0	0.0	0.0	0.0	0.0	0.0
TACGTAGGGG	0.0	0.0	0.0	0.0	0.0	0.0	0.0
TACGTAGGGG	0.0	0.0	0.0	0.0	0.0	0.0	0.0
TACGTAGGTG	0.0	0.0	0.0	0.0	0.0	0.0	0.0
TACGTAGGGC	0.0	0.0	0.0	0.0	0.0	0.0	0.0
TACGTAGGGA	0.0	0.0	0.0	0.0	0.0	0.0	0.0
TACGTAGGGA	0.0	0.0	0.0	0.0	0.0	0.0	0.0
TACGGAGGAT	0.0	0.0	0.0	0.0	0.0	0.0	0.0
TACGGAGGAT	0.0	0.0	0.0	0.0	0.0	0.0	0.0
TACGTAGGTG	0.0	0.0	0.0	0.0	0.0	0.0	0.0
TACGTAGGTG	0.0	0.0	0.0	0.0	0.0	0.0	0.0
TACGTATGGT	0.0	0.0	0.0	0.0	0.0	0.0	0.0
TACGGAGGAT	0.0	0.0	0.0	0.0	0.0	0.0	0.0
TACGTAGGTG	0.0	0.0	0.0	0.0	0.0	0.0	0.0
TACGGAGGGT	0.0	0.0	0.0	0.0	0.0	0.0	0.0
TACGTAGGTG	0.0	0.0	0.0	0.0	0.0	0.0	0.0
TACGGAGGAT	0.0	0.0	0.0	0.0	0.0	0.0	0.0
TACGTAGGGA	0.0	0.0	0.0	0.0	0.0	0.0	0.0
TACGTAGGGA	0.0	0.0	0.0	0.0	0.0	0.0	0.0
TACGTAGGTG	0.0	0.0	0.0	0.0	0.0	0.0	0.0
TACGTATGGT	0.0	0.0	0.0	0.0	0.0	0.0	0.0
TACGAAGGGC	0.0	0.0	0.0	0.0	0.0	0.0	0.0
TACGGAGGAT	0.0	0.0	0.0	0.0	0.0	0.0	0.0
TACGTATGGT	0.0	0.0	0.0	0.0	0.0	0.0	0.0
TACGTAGGGG	0.0	0.0	0.0	0.0	0.0	0.0	0.0
TACGTATGGG	0.0	0.0	0.0	0.0	0.0	0.0	0.0
TACGTAGGGG	0.0	0.0	0.0	0.0	0.0	0.0	0.0
TACGTAGGTG	0.0	0.0	0.0	0.0	0.0	0.0	0.0
TACGTAGGTG	0.0	0.0	0.0	0.0	0.0	0.0	0.0
TACGTAGGTG	0.0	0.0	0.0	0.0	0.0	0.0	0.0
TACGTAGGTG	0.0	0.0	0.0	0.0	0.0	0.0	0.0
TACGTATGGT	0.0	0.0	0.0	0.0	0.0	0.0	0.0
TACGTAGGGG	0.0	0.0	0.0	0.0	0.0	0.0	0.0
TACGTAGGAG	0.0	0.0	0.0	0.0	0.0	0.0	0.0
TACGTATGGG	0.0	0.0	0.0	0.0	0.0	0.0	0.0
TACGTAGGGG	0.0	0.0	0.0	0.0	0.0	0.0	0.0
TACGGAGGAT	0.0	0.0	0.0	0.0	0.0	0.0	0.0
TACGGAGGGT	0.0	0.0	0.0	0.0	0.0	0.0	0.0
TACGTAGGGG	0.0	0.0	0.0	0.0	0.0	0.0	0.0
TACGTAGGGG	0.0	0.0	0.0	0.0	0.0	0.0	0.0
TACGTAGGTG	0.0	0.0	0.0	0.0	0.0	0.0	0.0
TACGTAGGTG	0.0	0.0	0.0	0.0	0.0	0.0	0.0
TACGTATGGT	0.0	0.0	0.0	0.0	0.0	0.0	0.0
TACGTAGGGG	0.0	0.0	0.0	0.0	0.0	0.0	0.0

TACGTAGGTG	0.0	0.0	0.0	0.0	0.0	0.0	0.0
TACGTAGGGG	0.0	0.0	0.0	0.0	0.0	0.0	0.0
TACGTAGGTG	0.0	0.0	0.0	0.0	0.0	0.0	0.0
AACGTAGGTC	0.0	0.0	0.0	0.0	0.0	0.0	0.0
TACGTAGGGA	0.0	0.0	0.0	0.0	0.0	0.0	0.0
TACGTAGGTG	0.0	0.0	0.0	0.0	0.0	0.0	0.0
TACATAGGGG	0.0	0.0	0.0	0.0	0.0	0.0	0.0
TACGTAGGGG	0.0	0.0	0.0	0.0	0.0	0.0	0.0
TACGTATGGA	0.0	0.0	0.0	0.0	0.0	0.0	0.0
TACGTAGGTG	0.0	0.0	0.0	0.0	0.0	0.0	0.0
TACGGAGGAT	0.0	0.0	0.0	0.0	0.0	0.0	0.0
TACGTAGGTG	0.0	0.0	0.0	0.0	0.0	0.0	0.0
TACGTAGGTG	0.0	0.0	0.0	0.0	0.0	0.0	0.0
TACGTAGGTG	0.0	0.0	0.0	0.0	0.0	0.0	0.0
TACGGAGGGT	0.0	0.0	0.0	0.0	0.0	0.0	0.0
TACGTAGGTG	0.0	0.0	0.0	0.0	0.0	0.0	0.0
TACGTAGGGA	0.0	0.0	0.0	0.0	0.0	0.0	0.0
TACGGAGGAT	0.0	0.0	0.0	0.0	0.0	0.0	0.0
TACGTATGGA	0.0	0.0	0.0	0.0	0.0	0.0	0.0
TACGTATGGA	0.0	0.0	0.0	0.0	0.0	0.0	0.0
TACGTAGGTG	0.0	0.0	0.0	0.0	0.0	0.0	0.0
AACGTAGGTC	0.0	0.0	0.0	0.0	0.0	0.0	0.0
TACGTAGGGA	0.0	0.0	0.0	0.0	0.0	0.0	0.0
TACGTAGGTG	0.0	0.0	0.0	0.0	0.0	0.0	0.0
TACGGAAGGT	0.0	0.0	0.0	0.0	0.0	0.0	0.0
TACGTAGGGA	0.0	0.0	0.0	0.0	0.0	0.0	0.0
TACGTAGGGA	0.0	0.0	0.0	0.0	0.0	0.0	0.0
TACGTAGGTG	0.0	0.0	0.0	0.0	0.0	0.0	0.0
TACGTATGGA	0.0	0.0	0.0	0.0	0.0	0.0	0.0
TACGTAGGTG	0.0	0.0	0.0	0.0	0.0	0.0	0.0
TACGTAGAGG	0.0	0.0	0.0	0.0	0.0	0.0	0.0
AACGTAGGGT	0.0	0.0	0.0	0.0	0.0	0.0	0.0
CACGTAGGTC	0.0	0.0	0.0	0.0	0.0	0.0	0.0
TACGTAGGTG	0.0	0.0	0.0	0.0	0.0	0.0	0.0
TACGTAGGGG	0.0	0.0	0.0	0.0	0.0	0.0	0.0
TACGTAGGGG	0.0	0.0	0.0	0.0	0.0	0.0	0.0
TACGTAGGTG	0.0	0.0	0.0	0.0	0.0	0.0	0.0
TACGTAGGGA	0.0	0.0	0.0	0.0	0.0	0.0	0.0
TACGGAGGAT	0.0	0.0	0.0	0.0	0.0	0.0	0.0
TACGAAGGGG	0.0	0.0	0.0	0.0	0.0	0.0	0.0
TACGTAGGGG	0.0	0.0	0.0	0.0	0.0	0.0	0.0
TACGTAGGTG	0.0	0.0	0.0	0.0	0.0	0.0	0.0
TACGTAGGTG	0.0	0.0	0.0	0.0	0.0	0.0	0.0
TACGTAGGTG	0.0	0.0	0.0	0.0	0.0	0.0	0.0
TACGTAGGTG	0.0	0.0	0.0	0.0	0.0	0.0	0.0
TACGTAGGTG	0.0	0.0	0.0	0.0	0.0	0.0	0.0
TACGTAGGTG	0.0	0.0	0.0	0.0	0.0	0.0	0.0

TACGGAGGGT	0.0	0.0	0.0	0.0	0.0	0.0	0.0
TACGTAGGTG	0.0	0.0	0.0	0.0	0.0	0.0	0.0
TACGTAGGGG	0.0	0.0	0.0	0.0	0.0	0.0	0.0
TACGTAGGGG	0.0	0.0	0.0	0.0	0.0	0.0	0.0
TACGTAGGGG	0.0	0.0	0.0	0.0	0.0	0.0	0.0
TACGTAGGGG	0.0	0.0	0.0	0.0	0.0	0.0	0.0
TACGTAGGGG	0.0	0.0	0.0	0.0	0.0	0.0	0.0
TACGTAGGGA	0.0	0.0	0.0	0.0	0.0	0.0	0.0
TACGTAGGTG	0.0	0.0	0.0	0.0	0.0	0.0	0.0
TACGTAGGTG	0.0	0.0	0.0	0.0	0.0	0.0	0.0
TACGTAGGGG	0.0	0.0	0.0	0.0	0.0	0.0	0.0
TACGTAGGGA	0.0	0.0	0.0	0.0	0.0	0.0	0.0
TACGTAGGGA	0.0	0.0	0.0	0.0	0.0	0.0	0.0
AACGTAGGTC	0.0	0.0	0.0	0.0	0.0	0.0	0.0
TACATAGGTG	0.0	0.0	0.0	0.0	0.0	0.0	0.0
TACGTAGGGG	0.0	0.0	0.0	0.0	0.0	0.0	0.0
TACGTAGGGG	0.0	0.0	0.0	0.0	0.0	0.0	0.0
TACGTAGGTG	0.0	0.0	0.0	0.0	0.0	0.0	0.0
TACGTAGGGG	0.0	0.0	0.0	0.0	0.0	0.0	0.0
TACGTAGGTG	0.0	0.0	0.0	0.0	0.0	0.0	0.0
TACGTAGGGA	0.0	0.0	0.0	0.0	0.0	0.0	0.0
TACGTAGGTG	0.0	0.0	0.0	0.0	0.0	0.0	0.0
TACGTAGGTG	0.0	0.0	0.0	0.0	0.0	0.0	0.0
TACGCAGGGG	0.0	0.0	0.0	0.0	0.0	0.0	0.0
TACGTAGGGA	0.0	0.0	0.0	0.0	0.0	0.0	0.0
TACGTAGGGA	0.0	0.0	0.0	0.0	0.0	0.0	0.0
TACGTAGGGA	0.0	0.0	0.0	0.0	0.0	0.0	0.0
TACGTAGGTG	0.0	0.0	0.0	0.0	0.0	0.0	0.0
TACGTATGGT	0.0	0.0	0.0	0.0	0.0	0.0	0.0
TACGTAGGAG	0.0	0.0	0.0	0.0	0.0	0.0	0.0
TACGTAGGGG	0.0	0.0	0.0	0.0	0.0	0.0	0.0
TACGTAGGTG	0.0	0.0	0.0	0.0	0.0	0.0	0.0
TACGGAGGAT	0.0	0.0	0.0	0.0	0.0	0.0	0.0
TACGTAGGTG	0.0	0.0	0.0	0.0	0.0	0.0	0.0
TACGTAGGTG	0.0	0.0	0.0	0.0	0.0	0.0	0.0
TCCTTCTTCTC	0.0	0.0	0.0	0.0	0.0	0.0	0.0
TACGTAGGGG	0.0	0.0	0.0	0.0	0.0	0.0	0.0
TACGTAGGGG	0.0	0.0	0.0	0.0	0.0	0.0	0.0
TACGTAGGGG	0.0	0.0	0.0	0.0	0.0	0.0	0.0
TACGTAGGGA	0.0	0.0	0.0	0.0	0.0	0.0	0.0
TACGTAGGGG	0.0	0.0	0.0	0.0	0.0	0.0	0.0
TACGTAGGGA	0.0	0.0	0.0	0.0	0.0	0.0	0.0
TACGTAGGGG	0.0	0.0	0.0	0.0	0.0	0.0	0.0
TACGTAGGGG	0.0	0.0	0.0	0.0	0.0	0.0	0.0
TACGTAGGGG	0.0	0.0	0.0	0.0	0.0	0.0	0.0
TACGTAGAGG	0.0	0.0	0.0	0.0	0.0	0.0	0.0
TACGTAGGGG	0.0	0.0	0.0	0.0	0.0	0.0	0.0

TACGTATGGA	0.0	0.0	0.0	0.0	0.0	0.0	0.0
TACGTATGGT	0.0	0.0	0.0	0.0	0.0	0.0	0.0
TACGTAGGGG	0.0	0.0	0.0	0.0	0.0	0.0	0.0
TACGTAGGTG	0.0	0.0	0.0	0.0	0.0	0.0	0.0
TACGTATGGT	0.0	0.0	0.0	0.0	0.0	0.0	0.0
TACGTAGGGG	0.0	0.0	0.0	0.0	0.0	0.0	0.0
TACGTAGGGA	0.0	0.0	0.0	0.0	0.0	0.0	0.0
TACGTAGGGG	0.0	0.0	0.0	0.0	0.0	0.0	0.0
TACGTATGGT	0.0	0.0	0.0	0.0	0.0	0.0	0.0
TACGTAGGGG	0.0	0.0	0.0	0.0	0.0	0.0	0.0
TACGTAGGGG	0.0	0.0	0.0	0.0	0.0	0.0	0.0
TACGTAGGGG	0.0	0.0	0.0	0.0	0.0	0.0	0.0
TACGTAGGAT	0.0	0.0	0.0	0.0	0.0	0.0	0.0
TACGTAGGGG	0.0	0.0	0.0	0.0	0.0	0.0	0.0
TACAGAGGAT	0.0	0.0	0.0	0.0	0.0	0.0	0.0
TACGTAGGGC	0.0	0.0	0.0	0.0	0.0	0.0	0.0
TACGTAGGGT	0.0	0.0	0.0	0.0	0.0	0.0	0.0
TACGTATGTC	0.0	0.0	0.0	0.0	0.0	0.0	0.0
TACGTAGGGT	0.0	0.0	0.0	0.0	0.0	0.0	0.0
TACGTAGGTG	0.0	0.0	0.0	0.0	0.0	0.0	0.0
TACGTAGGTG	0.0	0.0	0.0	0.0	0.0	0.0	0.0
TACGTAGGTG	0.0	0.0	0.0	0.0	0.0	0.0	0.0
TACGTAGGGA	0.0	0.0	0.0	0.0	0.0	0.0	0.0
TACGTAGGTG	0.0	0.0	0.0	0.0	0.0	0.0	0.0
TACGTAGGTG	0.0	0.0	0.0	0.0	0.0	0.0	0.0
TACGTAGGTG	0.0	0.0	0.0	0.0	0.0	0.0	0.0
TACGTAGGGG	0.0	0.0	0.0	0.0	0.0	0.0	0.0
TACGTAGGGG	0.0	0.0	0.0	0.0	0.0	0.0	0.0
TACGTATGGT	0.0	0.0	0.0	0.0	0.0	0.0	0.0
TACGGAGGGT	0.0	0.0	0.0	0.0	0.0	0.0	0.0
TACGTAGGGG	0.0	0.0	0.0	0.0	0.0	0.0	0.0
TACGTAGGTG	0.0	0.0	0.0	0.0	0.0	0.0	0.0
TACGTAGGGA	0.0	0.0	0.0	0.0	0.0	0.0	0.0
TACGTAGGTG	0.0	0.0	0.0	0.0	0.0	0.0	0.0
TACGGAGGAT	0.0	0.0	0.0	0.0	0.0	0.0	0.0
TACGGAAGGT	0.0	0.0	0.0	0.0	0.0	0.0	0.0
TACGTAGGTG	0.0	0.0	0.0	0.0	0.0	0.0	0.0
TACGGAGGAT	0.0	0.0	0.0	0.0	0.0	0.0	0.0
TACGTAGGTG	0.0	0.0	0.0	0.0	0.0	0.0	0.0
TACGTAGGGA	0.0	0.0	0.0	0.0	0.0	0.0	0.0
TACGTAGGTG	0.0	0.0	0.0	0.0	0.0	0.0	0.0
TACGTAGGTG	0.0	0.0	0.0	0.0	0.0	0.0	0.0
TACGTAGGGG	0.0	0.0	0.0	0.0	0.0	0.0	0.0
TACGTAGGTG	0.0	0.0	0.0	0.0	0.0	0.0	0.0
TACGTAGGTT	0.0	0.0	0.0	0.0	0.0	0.0	0.0
TACGTAGGGG	0.0	0.0	0.0	0.0	0.0	0.0	0.0
TACGTAGGGG	0.0	0.0	0.0	0.0	0.0	0.0	0.0

TACGGAGGGT	0.0	0.0	0.0	0.0	0.0	0.0	0.0
TACGTAGGTG	0.0	0.0	0.0	0.0	0.0	0.0	0.0
TACGTAGGG	0.0	0.0	0.0	0.0	0.0	0.0	0.0
TACGTAGGTG	0.0	0.0	0.0	0.0	0.0	0.0	0.0
TACGTAGGGA	0.0	0.0	0.0	0.0	0.0	0.0	0.0
TACGGAAGAT	0.0	0.0	0.0	0.0	0.0	0.0	0.0
TACGTAGGGT	0.0	0.0	0.0	0.0	0.0	0.0	0.0
TACGTAGGTG	0.0	0.0	0.0	0.0	0.0	0.0	0.0
TACGGAGGA	0.0	0.0	0.0	0.0	0.0	0.0	0.0
TACGTAGGGA	0.0	0.0	0.0	0.0	0.0	0.0	0.0
TACGTAGGTG	0.0	0.0	0.0	0.0	0.0	0.0	0.0
TACGTAGGG	0.0	0.0	0.0	0.0	0.0	0.0	0.0
TACGTAGGGA	0.0	0.0	0.0	0.0	0.0	0.0	0.0
TACGTAGGG	0.0	0.0	0.0	0.0	0.0	0.0	0.0
TACGTAGGTG	0.0	0.0	0.0	0.0	0.0	0.0	0.0
TACGTAGGTA	0.0	0.0	0.0	0.0	0.0	0.0	0.0
TACGTAGGG	0.0	0.0	0.0	0.0	0.0	0.0	0.0
TACGTAGGGA	0.0	0.0	0.0	0.0	0.0	0.0	0.0
TACGTAGGTG	0.0	0.0	0.0	0.0	0.0	0.0	0.0
TACGTAGGTG	0.0	0.0	0.0	0.0	0.0	0.0	0.0
TACGTAGGTG	0.0	0.0	0.0	0.0	0.0	0.0	0.0
TACGTAGGG	0.0	0.0	0.0	0.0	0.0	0.0	0.0
TACGTAGGGA	0.0	0.0	0.0	0.0	0.0	0.0	0.0
TACGTAGGGA	0.0	0.0	0.0	0.0	0.0	0.0	0.0
TACGTAGGG	0.0	0.0	0.0	0.0	0.0	0.0	0.0
TACGTAGGTG	0.0	0.0	0.0	0.0	0.0	0.0	0.0
CACGTAGGG	0.0	0.0	0.0	0.0	0.0	0.0	0.0
AACGTAGGT	0.0	0.0	0.0	0.0	0.0	0.0	0.0
TACGTAGGTG	0.0	0.0	0.0	0.0	0.0	0.0	0.0
TACGTAGGGA	0.0	0.0	0.0	0.0	0.0	0.0	0.0
TACGTAGGTG	0.0	0.0	0.0	0.0	0.0	0.0	0.0
TACGTAGGGA	0.0	0.0	0.0	0.0	0.0	0.0	0.0
TACGTAGGG	0.0	0.0	0.0	0.0	0.0	0.0	0.0
TACGTAGGGA	0.0	0.0	0.0	0.0	0.0	0.0	0.0
TACGTAGGAT	0.0	0.0	0.0	0.0	0.0	0.0	0.0
TACGTAGGTG	0.0	0.0	0.0	0.0	0.0	0.0	0.0
TACGTAGGTG	0.0	0.0	0.0	0.0	0.0	0.0	0.0
TACGTATGGT	0.0	0.0	0.0	0.0	0.0	0.0	0.0
TACGTAGGGT	0.0	0.0	0.0	0.0	0.0	0.0	0.0
TACGTAGGG	0.0	0.0	0.0	0.0	0.0	0.0	0.0
TACGTAGGTG	0.0	0.0	0.0	0.0	0.0	0.0	0.0
TACGTAGGTG	0.0	0.0	0.0	0.0	0.0	0.0	0.0
TACGTAGGTG	0.0	0.0	0.0	0.0	0.0	0.0	0.0
TACGCAGGTG	0.0	0.0	0.0	0.0	0.0	0.0	0.0
TACGTAGGGA	0.0	0.0	0.0	0.0	0.0	0.0	0.0
TACGTAGGGT	0.0	0.0	0.0	0.0	0.0	0.0	0.0
TACGTAGGTG	0.0	0.0	0.0	0.0	0.0	0.0	0.0

TACGTAGGG	0.0	0.0	0.0	0.0	0.0	0.0	0.0
TACGTAGGTG	0.0	0.0	0.0	0.0	0.0	0.0	0.0
TACGTATGGA	0.0	0.0	0.0	0.0	0.0	0.0	0.0
TACGTAGGTG	0.0	0.0	0.0	0.0	0.0	0.0	0.0
TACGTAGGGA	0.0	0.0	0.0	0.0	0.0	0.0	0.0
TACGTAGGG	0.0	0.0	0.0	0.0	0.0	0.0	0.0
TACGTATGGT	0.0	0.0	0.0	0.0	0.0	0.0	0.0
TACGTAGGTG	0.0	0.0	0.0	0.0	0.0	0.0	0.0
TACGTAGGTG	0.0	0.0	0.0	0.0	0.0	0.0	0.0
TACGTAGGG	0.0	0.0	0.0	0.0	0.0	0.0	0.0
TACGTAGGTG	0.0	0.0	0.0	0.0	0.0	0.0	0.0
TACGTATGGT	0.0	0.0	0.0	0.0	0.0	0.0	0.0
TACGTATGGT	0.0	0.0	0.0	0.0	0.0	0.0	0.0
TACGTAGGG	0.0	0.0	0.0	0.0	0.0	0.0	0.0
TACGTAGGG	0.0	0.0	0.0	0.0	0.0	0.0	0.0
TACGTAGGG	0.0	0.0	0.0	0.0	0.0	0.0	0.0
TACGTAGGTG	0.0	0.0	0.0	0.0	0.0	0.0	0.0
TACGTAGGG	0.0	0.0	0.0	0.0	0.0	0.0	0.0
TACGTAGGG	0.0	0.0	0.0	0.0	0.0	0.0	0.0
TACGTAGGGA	0.0	0.0	0.0	0.0	0.0	0.0	0.0
TACGTAGGGA	0.0	0.0	0.0	0.0	0.0	0.0	0.0
TACGTAGGTG	0.0	0.0	0.0	0.0	0.0	0.0	0.0
TACGTAGGTG	0.0	0.0	0.0	0.0	0.0	0.0	0.0
TACGTAGGGA	0.0	0.0	0.0	0.0	0.0	0.0	0.0
TACGTAGGG	0.0	0.0	0.0	0.0	0.0	0.0	0.0
TACGTATGGA	0.0	0.0	0.0	0.0	0.0	0.0	0.0
TACGTAGGG	0.0	0.0	0.0	0.0	0.0	0.0	0.0
TACGTAGGGA	0.0	0.0	0.0	0.0	0.0	0.0	0.0
AACGTAGGGT	0.0	0.0	0.0	0.0	0.0	0.0	0.0
TACGTAGGG	0.0	0.0	0.0	0.0	0.0	0.0	0.0
TCCGGCCGGT	0.0	0.0	0.0	0.0	0.0	0.0	0.0
TACGTATGGT	0.0	0.0	0.0	0.0	0.0	0.0	0.0
TACGTAGGAT	0.0	0.0	0.0	0.0	0.0	0.0	0.0
AACGTAGGTC	0.0	0.0	0.0	0.0	0.0	0.0	0.0
TACGTAGGGA	0.0	0.0	0.0	0.0	0.0	0.0	0.0
TACGTAGGGA	0.0	0.0	0.0	0.0	0.0	0.0	0.0
TACGTAGGTG	0.0	0.0	0.0	0.0	0.0	0.0	0.0
TACGTAGGG	0.0	0.0	0.0	0.0	0.0	0.0	0.0
TACGTAGGG	0.0	0.0	0.0	0.0	0.0	0.0	0.0
TACGTAGGTG	0.0	0.0	0.0	0.0	0.0	0.0	0.0
TACGTAGGTG	0.0	0.0	0.0	0.0	0.0	0.0	0.0
TACGTAGGG	0.0	0.0	0.0	0.0	0.0	0.0	0.0
TACGTAGGTC	0.0	0.0	0.0	0.0	0.0	0.0	0.0
TACGGAGGAT	0.0	0.0	0.0	0.0	0.0	0.0	0.0
TACGTAGGGT	0.0	0.0	0.0	0.0	0.0	0.0	0.0
TACGTAGGG	0.0	0.0	0.0	0.0	0.0	0.0	0.0
TACGTATGGT	0.0	0.0	0.0	0.0	0.0	0.0	0.0

TACGTAGGG	0.0	0.0	0.0	0.0	0.0	0.0	0.0
TACGGAGGAT	0.0	0.0	0.0	0.0	0.0	0.0	0.0
TACGGAAGGT	0.0	0.0	0.0	0.0	0.0	0.0	0.0
TACGGAGGAT	0.0	0.0	0.0	0.0	0.0	0.0	0.0
TACGGAGGGT	0.0	0.0	0.0	0.0	0.0	0.0	0.0
TACGTAGGG	0.0	0.0	0.0	0.0	0.0	0.0	0.0
TACGTATGGT	0.0	0.0	0.0	0.0	0.0	0.0	0.0
TACGTAGGG	0.0	0.0	0.0	0.0	0.0	0.0	0.0
TACGTAGGG	0.0	0.0	0.0	0.0	0.0	0.0	0.0
TACGTAGGG	0.0	0.0	0.0	0.0	0.0	0.0	0.0
TACGTAGGGA	0.0	0.0	0.0	0.0	0.0	0.0	0.0
TACGTATGGG	0.0	0.0	0.0	0.0	0.0	0.0	0.0
TACGTATGGA	0.0	0.0	0.0	0.0	0.0	0.0	0.0
TACGTAGGG	0.0	0.0	0.0	0.0	0.0	0.0	0.0
TACGTAGGG	0.0	0.0	0.0	0.0	0.0	0.0	0.0
TACGTAGGG	0.0	0.0	0.0	0.0	0.0	0.0	0.0
TACGTAGGTG	0.0	0.0	0.0	0.0	0.0	0.0	0.0
TACGTATGGA	0.0	0.0	0.0	0.0	0.0	0.0	0.0
TACGTAGGTG	0.0	0.0	0.0	0.0	0.0	0.0	0.0
TACGTATGGT	0.0	0.0	0.0	0.0	0.0	0.0	0.0
TACGTAGGTG	0.0	0.0	0.0	0.0	0.0	0.0	0.0
TACGTAGGG	0.0	0.0	0.0	0.0	0.0	0.0	0.0
TACGTAGGG	0.0	0.0	0.0	0.0	0.0	0.0	0.0
TACGTAGGGA	0.0	0.0	0.0	0.0	0.0	0.0	0.0
TACGTAGGGA	0.0	0.0	0.0	0.0	0.0	0.0	0.0
AACGTAGGTC	0.0	0.0	0.0	0.0	0.0	0.0	0.0
TTCTTCTTTCT	0.0	0.0	0.0	0.0	0.0	0.0	0.0
TACGTAGATG	0.0	0.0	0.0	0.0	0.0	0.0	0.0
TACGTAGGTC	0.0	0.0	0.0	0.0	0.0	0.0	0.0
TACGTATGGT	0.0	0.0	0.0	0.0	0.0	0.0	0.0
TACGTAGGG	0.0	0.0	0.0	0.0	0.0	0.0	0.0
TACGTAGGG	0.0	0.0	0.0	0.0	0.0	0.0	0.0
TACGTAGGG	0.0	0.0	0.0	0.0	0.0	0.0	0.0
TACGTAGGGA	0.0	0.0	0.0	0.0	0.0	0.0	0.0
TACGTAGGGT	0.0	0.0	0.0	0.0	0.0	0.0	0.0
TACGTAGGG	0.0	0.0	0.0	0.0	0.0	0.0	0.0
TACGTATGGT	0.0	0.0	0.0	0.0	0.0	0.0	0.0
TACGTAGGGA	0.0	0.0	0.0	0.0	0.0	0.0	0.0
TACGTAGGGA	0.0	0.0	0.0	0.0	0.0	0.0	0.0
TACGTAGGTG	0.0	0.0	0.0	0.0	0.0	0.0	0.0
AACGTAGGTC	0.0	0.0	0.0	0.0	0.0	0.0	0.0
TACGTAGGG	0.0	0.0	0.0	0.0	0.0	0.0	0.0
AACGTAGGTC	0.0	0.0	0.0	0.0	0.0	0.0	0.0
TACGTAGGG	0.0	0.0	0.0	0.0	0.0	0.0	0.0
TACGTAGGTG	0.0	0.0	0.0	0.0	0.0	0.0	0.0
TACGTAGGTG	0.0	0.0	0.0	0.0	0.0	0.0	0.0
TACGTAGGG	0.0	0.0	0.0	0.0	0.0	0.0	0.0

TACGTAGGTG	0.0	0.0	0.0	0.0	0.0	0.0	0.0
TACGTAGGGG	0.0	0.0	0.0	0.0	0.0	0.0	0.0
TACGTAGGGG	0.0	0.0	0.0	0.0	0.0	0.0	0.0
TACGTAGGGG	0.0	0.0	0.0	0.0	0.0	0.0	0.0
TACGTAGGGG	0.0	0.0	0.0	0.0	0.0	0.0	0.0
TACGTAGGGG	0.0	0.0	0.0	0.0	0.0	0.0	0.0
TACGTAGGGG	0.0	0.0	0.0	0.0	0.0	0.0	0.0
TACGTAGGTG	0.0	0.0	0.0	0.0	0.0	0.0	0.0
AACGTAAGTC	0.0	0.0	0.0	0.0	0.0	0.0	0.0
AACGTAGGTC	0.0	0.0	0.0	0.0	0.0	0.0	0.0
TACGTAGGTG	0.0	0.0	0.0	0.0	0.0	0.0	0.0
TACGGAAGAT	0.0	0.0	0.0	0.0	0.0	0.0	0.0
TACGTAGGGG	0.0	0.0	0.0	0.0	0.0	0.0	0.0
TACGGAGGAT	0.0	0.0	0.0	0.0	0.0	0.0	0.0
TACGTAGGGA	0.0	0.0	0.0	0.0	0.0	0.0	0.0
TACGTAGGGA	0.0	0.0	0.0	0.0	0.0	0.0	0.0
TACGTAGGGA	0.0	0.0	0.0	0.0	0.0	0.0	0.0
TACGTAGGGA	0.0	0.0	0.0	0.0	0.0	0.0	0.0
AACGTAGGGT	0.0	0.0	0.0	0.0	0.0	0.0	0.0
TACGTATGGT	0.0	0.0	0.0	0.0	0.0	0.0	0.0
TACGTAGGGG	0.0	0.0	0.0	0.0	0.0	0.0	0.0
TACGTAGGGG	0.0	0.0	0.0	0.0	0.0	0.0	0.0
TACGTAGGGG	0.0	0.0	0.0	0.0	0.0	0.0	0.0
TACGTAGGGG	0.0	0.0	0.0	0.0	0.0	0.0	0.0
TACGTAGGGG	0.0	0.0	0.0	0.0	0.0	0.0	0.0
TACGTAGGGA	0.0	0.0	0.0	0.0	0.0	0.0	0.0
TACGTAGGAT	0.0	0.0	0.0	0.0	0.0	0.0	0.0
TACGTAGGGG	0.0	0.0	0.0	0.0	0.0	0.0	0.0
TACGTAGGTG	0.0	0.0	0.0	0.0	0.0	0.0	0.0
TACGTAGGTG	0.0	0.0	0.0	0.0	0.0	0.0	0.0
TACGTAGGGG	0.0	0.0	0.0	0.0	0.0	0.0	0.0
TACGTAGGGA	0.0	0.0	0.0	0.0	0.0	0.0	0.0
TACGTAGGTG	0.0	0.0	0.0	0.0	0.0	0.0	0.0
TACGTAGGTG	0.0	0.0	0.0	0.0	0.0	0.0	0.0
TACGTAAGGT	0.0	0.0	0.0	0.0	0.0	0.0	0.0
TACGTAGGTG	0.0	0.0	0.0	0.0	0.0	0.0	0.0
TACGTAGGTC	0.0	0.0	0.0	0.0	0.0	0.0	0.0
TACGTAGGGG	0.0	0.0	0.0	0.0	0.0	0.0	0.0
TACGTATGGA	0.0	0.0	0.0	0.0	0.0	0.0	0.0
TACGTATGGT	0.0	0.0	0.0	0.0	0.0	0.0	0.0
AACGTAGGTC	0.0	0.0	0.0	0.0	0.0	0.0	0.0
TACGTATGGT	0.0	0.0	0.0	0.0	0.0	0.0	0.0
CACGTATGGT	0.0	0.0	0.0	0.0	0.0	0.0	0.0
TACGTAGGGG	0.0	0.0	0.0	0.0	0.0	0.0	0.0
TACGTATGGA	0.0	0.0	0.0	0.0	0.0	0.0	0.0
TACGTAGGGG	0.0	0.0	0.0	0.0	0.0	0.0	0.0
TACGTATGGT	0.0	0.0	0.0	0.0	0.0	0.0	0.0

TACGTAGGGA	0.0	0.0	0.0	0.0	0.0	0.0	0.0
TACGTAGGGG	0.0	0.0	0.0	0.0	0.0	0.0	0.0
TACGGAGGAT	0.0	0.0	0.0	0.0	0.0	0.0	0.0
TACGTAGGGG	0.0	0.0	0.0	0.0	0.0	0.0	0.0
TACGGAGGGT	0.0	0.0	0.0	0.0	0.0	0.0	0.0
TACGAAGGGC	0.0	0.0	0.0	0.0	0.0	0.0	0.0
TACGTATGGT	0.0	0.0	0.0	0.0	0.0	0.0	0.0
AACGTAGGGT	0.0	0.0	0.0	0.0	0.0	0.0	0.0
TACGTAGGGG	0.0	0.0	0.0	0.0	0.0	0.0	0.0
TACGTAGGGG	0.0	0.0	0.0	0.0	0.0	0.0	0.0
TACGTAGGTG	0.0	0.0	0.0	0.0	0.0	0.0	0.0
TACGTAGGGC	0.0	0.0	0.0	0.0	0.0	0.0	0.0
TACGTAGGTG	0.0	0.0	0.0	0.0	0.0	0.0	0.0
TACGTATGGA	0.0	0.0	0.0	0.0	0.0	0.0	0.0
TACGTAGGTG	0.0	0.0	0.0	0.0	0.0	0.0	0.0
TACGTAGGGA	0.0	0.0	0.0	0.0	0.0	0.0	0.0
TACGTAGGGA	0.0	0.0	0.0	0.0	0.0	0.0	0.0
TACGTAGGGA	0.0	0.0	0.0	0.0	0.0	0.0	0.0
TACGTAGGGA	0.0	0.0	0.0	0.0	0.0	0.0	0.0
TACGTAGGGG	0.0	0.0	0.0	0.0	0.0	0.0	0.0
TACGTAGGTG	0.0	0.0	0.0	0.0	0.0	0.0	0.0
TACGTAGGGG	0.0	0.0	0.0	0.0	0.0	0.0	0.0
TACGTATGGA	0.0	0.0	0.0	0.0	0.0	0.0	0.0
TACGTAGGTG	0.0	0.0	0.0	0.0	0.0	0.0	0.0
AACGTAGGTG	0.0	0.0	0.0	0.0	0.0	0.0	0.0
TACGTAGGGG	0.0	0.0	0.0	0.0	0.0	0.0	0.0
TACGTAGGGA	0.0	0.0	0.0	0.0	0.0	0.0	0.0
TACGTAGGTG	0.0	0.0	0.0	0.0	0.0	0.0	0.0
TACGTAGGGG	0.0	0.0	0.0	0.0	0.0	0.0	0.0
TACGTAGGGA	0.0	0.0	0.0	0.0	0.0	0.0	0.0
TACGTAGGGG	0.0	0.0	0.0	0.0	0.0	0.0	0.0
TACGGAGGGT	0.0	0.0	0.0	0.0	0.0	0.0	0.0
AACGTAGGTG	0.0	0.0	0.0	0.0	0.0	0.0	0.0
TACGTATGGT	0.0	0.0	0.0	0.0	0.0	0.0	0.0
TACGTAGGGG	0.0	0.0	0.0	0.0	0.0	0.0	0.0
TACGTAGGTG	0.0	0.0	0.0	0.0	0.0	0.0	0.0
TACGTAGGGG	0.0	0.0	0.0	0.0	0.0	0.0	0.0
TACGGAGGAT	0.0	0.0	0.0	0.0	0.0	0.0	0.0
TACGTAGGGT	0.0	0.0	0.0	0.0	0.0	0.0	0.0
TACGTAGGGA	0.0	0.0	0.0	0.0	0.0	0.0	0.0
TACGTAGGTG	0.0	0.0	0.0	0.0	0.0	0.0	0.0
TACGTATGGA	0.0	0.0	0.0	0.0	0.0	0.0	0.0
TACGTATGGT	0.0	0.0	0.0	0.0	0.0	0.0	0.0
TACGGAGGGT	0.0	0.0	0.0	0.0	0.0	0.0	0.0
AACGTAGGTG	0.0	0.0	0.0	0.0	0.0	0.0	0.0
TACGCAGGGG	0.0	0.0	0.0	0.0	0.0	0.0	0.0
TACGGAGGAT	0.0	0.0	0.0	0.0	0.0	0.0	0.0

TACGTAGGTG	0.0	0.0	0.0	0.0	0.0	0.0	0.0
TACGTAGGGG	0.0	0.0	0.0	0.0	0.0	0.0	0.0
TACGTAGGGA	0.0	0.0	0.0	0.0	0.0	0.0	0.0
TACGTAGGGA	0.0	0.0	0.0	0.0	0.0	0.0	0.0
TACGTAGGTG	0.0	0.0	0.0	0.0	0.0	0.0	0.0
TACGTAGGGT	0.0	0.0	0.0	0.0	0.0	0.0	0.0
TACGTAGGGG	0.0	0.0	0.0	0.0	0.0	0.0	0.0
TACGTAGGGA	0.0	0.0	0.0	0.0	0.0	0.0	0.0
TACGTAGGGA	0.0	0.0	0.0	0.0	0.0	0.0	0.0
TACGTAGGGG	0.0	0.0	0.0	0.0	0.0	0.0	0.0
TACGTAGGTG	0.0	0.0	0.0	0.0	0.0	0.0	0.0
TACGTAGGAT	0.0	0.0	0.0	0.0	0.0	0.0	0.0
AACGTAGGGA	0.0	0.0	0.0	0.0	0.0	0.0	0.0
TACGTAGGGA	0.0	0.0	0.0	0.0	0.0	0.0	0.0
TACGGAGGAT	0.0	0.0	0.0	0.0	0.0	0.0	0.0
GACAGAGGAT	0.0	0.0	0.0	0.0	0.0	0.0	0.0
TACGTATGGT	0.0	0.0	0.0	0.0	0.0	0.0	0.0
TACGTAGGGG	0.0	0.0	0.0	0.0	0.0	0.0	0.0
TACGTATGGA	0.0	0.0	0.0	0.0	0.0	0.0	0.0
TACGTAGGGG	0.0	0.0	0.0	0.0	0.0	0.0	0.0
TACGTAGGGG	0.0	0.0	0.0	0.0	0.0	0.0	0.0
TACGTATGGA	0.0	0.0	0.0	0.0	0.0	0.0	0.0
TACGTAGGAT	0.0	0.0	0.0	0.0	0.0	0.0	0.0
TACGTAGGGG	0.0	0.0	0.0	0.0	0.0	0.0	0.0
TACGTAGGGA	0.0	0.0	0.0	0.0	0.0	0.0	0.0
TACGTAGGGG	0.0	0.0	0.0	0.0	0.0	0.0	0.0
TACGTATGTC	0.0	0.0	0.0	0.0	0.0	0.0	0.0
TACGTAGGGA	0.0	0.0	0.0	0.0	0.0	0.0	0.0
TACGGAGGAT	0.0	0.0	0.0	0.0	0.0	0.0	0.0
TACGTAGGGA	0.0	0.0	0.0	0.0	0.0	0.0	0.0
TACGTAGGGG	0.0	0.0	0.0	0.0	0.0	0.0	0.0
TACGTAGGGG	0.0	0.0	0.0	0.0	0.0	0.0	0.0
TACGTAGGGA	0.0	0.0	0.0	0.0	0.0	0.0	0.0
TACGTAGGGG	0.0	0.0	0.0	0.0	0.0	0.0	0.0
TACGTAGGGG	0.0	0.0	0.0	0.0	0.0	0.0	0.0
TACGTAGGTG	0.0	0.0	0.0	0.0	0.0	0.0	0.0
TACGTAGGGG	0.0	0.0	0.0	0.0	0.0	0.0	0.0
TACGTAGGGG	0.0	0.0	0.0	0.0	0.0	0.0	0.0
TACGTAGGGG	0.0	0.0	0.0	0.0	0.0	0.0	0.0
TACGTAGGGG	0.0	0.0	0.0	0.0	0.0	0.0	0.0
TACGTAGGTG	0.0	0.0	0.0	0.0	0.0	0.0	0.0
TACGTAGGGG	0.0	0.0	0.0	0.0	0.0	0.0	0.0
TACGTAGGTG	0.0	0.0	0.0	0.0	0.0	0.0	0.0
TCCTTCTTTTT	0.0	0.0	0.0	0.0	0.0	0.0	0.0
TACGTAGGTG	0.0	0.0	0.0	0.0	0.0	0.0	0.0
TCCTTCTTTTG	0.0	0.0	0.0	0.0	0.0	0.0	0.0
TACGTAGGGG	0.0	0.0	0.0	0.0	0.0	0.0	0.0

TACGTAGGTG	0.0	0.0	0.0	0.0	0.0	0.0	0.0
TACGTAGGGA	0.0	0.0	0.0	0.0	0.0	0.0	0.0
TACGTAGGGG	0.0	0.0	0.0	0.0	0.0	0.0	0.0
TACGTAGGTG	0.0	0.0	0.0	0.0	0.0	0.0	0.0
TACGTAGGGG	0.0	0.0	0.0	0.0	0.0	0.0	0.0
TACGTAGGTG	0.0	0.0	0.0	0.0	0.0	0.0	0.0
TACGTAGGGG	0.0	0.0	0.0	0.0	0.0	0.0	0.0
TACGTAGGGG	0.0	0.0	0.0	0.0	0.0	0.0	0.0
TACGTAGGGA	0.0	0.0	0.0	0.0	0.0	0.0	0.0
TACGTAGGGG	0.0	0.0	0.0	0.0	0.0	0.0	0.0
TACGTAGGGT	0.0	0.0	0.0	0.0	0.0	0.0	0.0
TACGTAGGGG	0.0	0.0	0.0	0.0	0.0	0.0	0.0
TACGTATGGA	0.0	0.0	0.0	0.0	0.0	0.0	0.0
TACGTAGGGG	0.0	0.0	0.0	0.0	0.0	0.0	0.0
TACGTAGGTG	0.0	0.0	0.0	0.0	0.0	0.0	0.0
TACGTAGGGG	0.0	0.0	0.0	0.0	0.0	0.0	0.0
TACGTAGGTG	0.0	0.0	0.0	0.0	0.0	0.0	0.0
TACGTAGGGG	0.0	0.0	0.0	0.0	0.0	0.0	0.0
TACGTAGGTG	0.0	0.0	0.0	0.0	0.0	0.0	0.0
TACGTAGGGG	0.0	0.0	0.0	0.0	0.0	0.0	0.0
TACGTAGGTG	0.0	0.0	0.0	0.0	0.0	0.0	0.0
TACGTAGGGG	0.0	0.0	0.0	0.0	0.0	0.0	0.0
TACGTAGGGG	0.0	0.0	0.0	0.0	0.0	0.0	0.0
TACGTAGGGG	0.0	0.0	0.0	0.0	0.0	0.0	0.0
TACGAAGGGC	0.0	0.0	0.0	0.0	0.0	0.0	0.0
TACGTAGGGG	0.0	0.0	0.0	0.0	0.0	0.0	0.0
TACGTAGGGG	0.0	0.0	0.0	0.0	0.0	0.0	0.0
CTACGTGCCA	0.0	0.0	0.0	0.0	0.0	0.0	0.0
TACGTAGGTG	0.0	0.0	0.0	0.0	0.0	0.0	0.0
TACGTAGGTG	0.0	0.0	0.0	0.0	0.0	0.0	0.0
TACGTAGGGA	0.0	0.0	0.0	0.0	0.0	0.0	0.0
TACGTAGGGG	0.0	0.0	0.0	0.0	0.0	0.0	0.0
TACGTAGGTG	0.0	0.0	0.0	0.0	0.0	0.0	0.0
TACGTAGGGG	0.0	0.0	0.0	0.0	0.0	0.0	0.0
TACGTATGGT	0.0	0.0	0.0	0.0	0.0	0.0	0.0
TACGTAGGTG	0.0	0.0	0.0	0.0	0.0	0.0	0.0
TACGTAGGGG	0.0	0.0	0.0	0.0	0.0	0.0	0.0
TACGTATGGT	0.0	0.0	0.0	0.0	0.0	0.0	0.0
TACGTAGGGA	0.0	0.0	0.0	0.0	0.0	0.0	0.0
TACGTAGGGG	0.0	0.0	0.0	0.0	0.0	0.0	0.0
TACGTAGGGG	0.0	0.0	0.0	0.0	0.0	0.0	0.0
TACGTAGGGG	0.0	0.0	0.0	0.0	0.0	0.0	0.0
TACGTATGGA	0.0	0.0	0.0	0.0	0.0	0.0	0.0
TACGTAGGGG	0.0	0.0	0.0	0.0	0.0	0.0	0.0
TACGTAGGTG	0.0	0.0	0.0	0.0	0.0	0.0	0.0
TACGTAGGGG	0.0	0.0	0.0	0.0	0.0	0.0	0.0
TACGTAGGGG	0.0	0.0	0.0	0.0	0.0	0.0	0.0

TACGTAGGG	0.0	0.0	0.0	0.0	0.0	0.0	0.0
TACGTATGGT	0.0	0.0	0.0	0.0	0.0	0.0	0.0
TACGTAGGGA	0.0	0.0	0.0	0.0	0.0	0.0	0.0
TACGTAGGGA	0.0	0.0	0.0	0.0	0.0	0.0	0.0
TACGTATGGA	0.0	0.0	0.0	0.0	0.0	0.0	0.0
TACGTAGGGA	0.0	0.0	0.0	0.0	0.0	0.0	0.0
TACGTAGGG	0.0	0.0	0.0	0.0	0.0	0.0	0.0
TACGTAGGTG	0.0	0.0	0.0	0.0	0.0	0.0	0.0
TACGGAGGAT	0.0	0.0	0.0	0.0	0.0	0.0	0.0
TACAGAGGTC	0.0	0.0	0.0	0.0	0.0	0.0	0.0
TACGTATGGT	0.0	0.0	0.0	0.0	0.0	0.0	0.0
TACGTAGGGT	0.0	0.0	0.0	0.0	0.0	0.0	0.0
TACGTATGGT	0.0	0.0	0.0	0.0	0.0	0.0	0.0
TACGGAGGAT	0.0	0.0	0.0	0.0	0.0	0.0	0.0
TACGTAGGG	0.0	0.0	0.0	0.0	0.0	0.0	0.0
TACGTATGGT	0.0	0.0	0.0	0.0	0.0	0.0	0.0
TACGTAGGGA	0.0	0.0	0.0	0.0	0.0	0.0	0.0
TACGTAGGTG	0.0	0.0	0.0	0.0	0.0	0.0	0.0
TACGTAGGG	0.0	0.0	0.0	0.0	0.0	0.0	0.0
TACGTAGGG	0.0	0.0	0.0	0.0	0.0	0.0	0.0
TACGTATGGT	0.0	0.0	0.0	0.0	0.0	0.0	0.0
TACGTAGGG	0.0	0.0	0.0	0.0	0.0	0.0	0.0
TACGTAGGGA	0.0	0.0	0.0	0.0	0.0	0.0	0.0
TACGGAGGAT	0.0	0.0	0.0	0.0	0.0	0.0	0.0
TACGTAGGG	0.0	0.0	0.0	0.0	0.0	0.0	0.0
TACGTAGGTG	0.0	0.0	0.0	0.0	0.0	0.0	0.0
TACGTAGGTG	0.0	0.0	0.0	0.0	0.0	0.0	0.0
TACGTAGGG	0.0	0.0	0.0	0.0	0.0	0.0	0.0
TACGTATGGT	0.0	0.0	0.0	0.0	0.0	0.0	0.0
TACGTATGTC	0.0	0.0	0.0	0.0	0.0	0.0	0.0
TACGGAGGGT	0.0	0.0	0.0	0.0	0.0	0.0	0.0
TACGTAGGG	0.0	0.0	0.0	0.0	0.0	0.0	0.0
GACGTAGGG	0.0	0.0	0.0	0.0	0.0	0.0	0.0
TACGTAGGTG	0.0	0.0	0.0	0.0	0.0	0.0	0.0
TACGTAGGG	0.0	0.0	0.0	0.0	0.0	0.0	0.0
TACGTAGGTG	0.0	0.0	0.0	0.0	0.0	0.0	0.0
TACGTAGGTG	0.0	0.0	0.0	0.0	0.0	0.0	0.0
TACGTAGGG	0.0	0.0	0.0	0.0	0.0	0.0	0.0
TACGGAGGG	0.0	0.0	0.0	0.0	0.0	0.0	0.0
TACGTAGGG	0.0	0.0	0.0	0.0	0.0	0.0	0.0
TACGTAGGGT	0.0	0.0	0.0	0.0	0.0	0.0	0.0
TACGTAGGTG	0.0	0.0	0.0	0.0	0.0	0.0	0.0
TACGTATGGT	0.0	0.0	0.0	0.0	0.0	0.0	0.0
TACGTATGGT	0.0	0.0	0.0	0.0	0.0	0.0	0.0
TACGTAGGG	0.0	0.0	0.0	0.0	0.0	0.0	0.0
TACGGAGGAT	0.0	0.0	0.0	0.0	0.0	0.0	0.0
TACGTAGGTG	0.0	0.0	0.0	0.0	0.0	0.0	0.0

TACGTAGGTG	0.0	0.0	0.0	0.0	0.0	0.0	0.0
TACGTAGGG	0.0	0.0	0.0	0.0	0.0	0.0	0.0
TACGTAGGG	0.0	0.0	0.0	0.0	0.0	0.0	0.0
TACGTAGGGT	0.0	0.0	0.0	0.0	0.0	0.0	0.0
TACGTAGGG	0.0	0.0	0.0	0.0	0.0	0.0	0.0
TACGTATGGT	0.0	0.0	0.0	0.0	0.0	0.0	0.0
TACGTATGGA	0.0	0.0	0.0	0.0	0.0	0.0	0.0
TACGTAGGTG	0.0	0.0	0.0	0.0	0.0	0.0	0.0
TACGAGGGGA	0.0	0.0	0.0	0.0	0.0	0.0	0.0
TACGTAGGG	0.0	0.0	0.0	0.0	0.0	0.0	0.0
TACGTAGGTG	0.0	0.0	0.0	0.0	0.0	0.0	0.0
TACGGAGGAT	0.0	0.0	0.0	0.0	0.0	0.0	0.0
TACGGAGGAT	0.0	0.0	0.0	0.0	0.0	0.0	0.0
TACGTAGGTG	0.0	0.0	0.0	0.0	0.0	0.0	0.0
TACGTAGGG	0.0	0.0	0.0	0.0	0.0	0.0	0.0
TACGGGGGAT	0.0	0.0	0.0	0.0	0.0	0.0	0.0
TACGTAGGTG	0.0	0.0	0.0	0.0	0.0	0.0	0.0
TACGTAGGTG	0.0	0.0	0.0	0.0	0.0	0.0	0.0
TACGTATGGA	0.0	0.0	0.0	0.0	0.0	0.0	0.0
TACGTAGGGA	0.0	0.0	0.0	0.0	0.0	0.0	0.0
TACGTAGGTC	0.0	0.0	0.0	0.0	0.0	0.0	0.0
TACGTAGGTG	0.0	0.0	0.0	0.0	0.0	0.0	0.0
TACGTAGGTG	0.0	0.0	0.0	0.0	0.0	0.0	0.0
TACGGAGGAT	0.0	0.0	0.0	0.0	0.0	0.0	0.0
AACGTAGGTC	0.0	0.0	0.0	0.0	0.0	0.0	0.0
TACGTAGGG	0.0	0.0	0.0	0.0	0.0	0.0	0.0
TACGTAGGGA	0.0	0.0	0.0	0.0	0.0	0.0	0.0
TACGTAGGTG	0.0	0.0	0.0	0.0	0.0	0.0	0.0
TACGTAGGG	0.0	0.0	0.0	0.0	0.0	0.0	0.0
TACGCAGGG	0.0	0.0	0.0	0.0	0.0	0.0	0.0
TACGTAGGG	0.0	0.0	0.0	0.0	0.0	0.0	0.0
TACGTATGGT	0.0	0.0	0.0	0.0	0.0	0.0	0.0
TACGTAGGTG	0.0	0.0	0.0	0.0	0.0	0.0	0.0
TACGGAGGAT	0.0	0.0	0.0	0.0	0.0	0.0	0.0
TACGTAGGG	0.0	0.0	0.0	0.0	0.0	0.0	0.0
TACGTAGGG	0.0	0.0	0.0	0.0	0.0	0.0	0.0
TACGGAGGAT	0.0	0.0	0.0	0.0	0.0	0.0	0.0
TACGGAGGAT	0.0	0.0	0.0	0.0	0.0	0.0	0.0
TACGTATGGT	0.0	0.0	0.0	0.0	0.0	0.0	0.0
TACGTAGGG	0.0	0.0	0.0	0.0	0.0	0.0	0.0
TACGTATGGT	0.0	0.0	0.0	0.0	0.0	0.0	0.0
TACGTAGGG	0.0	0.0	0.0	0.0	0.0	0.0	0.0
TACGTAGGTG	0.0	0.0	0.0	0.0	0.0	0.0	0.0
TACGTAGGGA	0.0	0.0	0.0	0.0	0.0	0.0	0.0
TACGTAGGG	0.0	0.0	0.0	0.0	0.0	0.0	0.0
TACGTATGGA	0.0	0.0	0.0	0.0	0.0	0.0	0.0
TACGGAGGAT	0.0	0.0	0.0	0.0	0.0	0.0	0.0

TACGTAGGGG	0.0	0.0	0.0	0.0	0.0	0.0	0.0
TACGTAGGGG	0.0	0.0	0.0	0.0	0.0	0.0	0.0
AACGTAGGTC	0.0	0.0	0.0	0.0	0.0	0.0	0.0
TACGTATGGT	0.0	0.0	0.0	0.0	0.0	0.0	0.0
TACGGAGGAT	0.0	0.0	0.0	0.0	0.0	0.0	0.0
TACGGAGGG	0.0	0.0	0.0	0.0	0.0	0.0	0.0
TACGTAGGTC	0.0	0.0	0.0	0.0	0.0	0.0	0.0
TACGTAGGGA	0.0	0.0	0.0	0.0	0.0	0.0	0.0
TACGTAGGGG	0.0	0.0	0.0	0.0	0.0	0.0	0.0
TACGTATGGG	0.0	0.0	0.0	0.0	0.0	0.0	0.0
TACGTAGGGA	0.0	0.0	0.0	0.0	0.0	0.0	0.0
TACGTAGGGG	0.0	0.0	0.0	0.0	0.0	0.0	0.0
TACGTAGGGG	0.0	0.0	0.0	0.0	0.0	0.0	0.0
AACGTAGGGT	0.0	0.0	0.0	0.0	0.0	0.0	0.0
TACGTAGGGG	0.0	0.0	0.0	0.0	0.0	0.0	0.0
TACGTAGGGG	0.0	0.0	0.0	0.0	0.0	0.0	0.0
TACGTAGGTG	0.0	0.0	0.0	0.0	0.0	0.0	0.0
TACGTATGGA	0.0	0.0	0.0	0.0	0.0	0.0	0.0
TACGTAGGGG	0.0	0.0	0.0	0.0	0.0	0.0	0.0
TACGTAGGTG	0.0	0.0	0.0	0.0	0.0	0.0	0.0
TACGTAGGGA	0.0	0.0	0.0	0.0	0.0	0.0	0.0
TACGTAGGGA	0.0	0.0	0.0	0.0	0.0	0.0	0.0
TACGTAGGGA	0.0	0.0	0.0	0.0	0.0	0.0	0.0
TACGTAGGGA	0.0	0.0	0.0	0.0	0.0	0.0	0.0
TACGTAGGGG	0.0	0.0	0.0	0.0	0.0	0.0	0.0
TACGTAGGGG	0.0	0.0	0.0	0.0	0.0	0.0	0.0
TACGTAGGTG	0.0	0.0	0.0	0.0	0.0	0.0	0.0
TACGTAGGTG	0.0	0.0	0.0	0.0	0.0	0.0	0.0
TACGTAGGGG	0.0	0.0	0.0	0.0	0.0	0.0	0.0
TACGTAGGGA	0.0	0.0	0.0	0.0	0.0	0.0	0.0
TACGTATGGT	0.0	0.0	0.0	0.0	0.0	0.0	0.0
TACGTAGGTG	0.0	0.0	0.0	0.0	0.0	0.0	0.0
TACGTAGGGA	0.0	0.0	0.0	0.0	0.0	0.0	0.0
TACGTAGGGA	0.0	0.0	0.0	0.0	0.0	0.0	0.0
AACGTAGGTC	0.0	0.0	0.0	0.0	0.0	0.0	0.0
TACGTATGAA	0.0	0.0	0.0	0.0	0.0	0.0	0.0
TACGTAGGGG	0.0	0.0	0.0	0.0	0.0	0.0	0.0
TACGTATGGG	0.0	0.0	0.0	0.0	0.0	0.0	0.0
TACGTAGGGG	0.0	0.0	0.0	0.0	0.0	0.0	0.0
TACGTAGGGA	0.0	0.0	0.0	0.0	0.0	0.0	0.0
TACGTATGGG	0.0	0.0	0.0	0.0	0.0	0.0	0.0
TACGTAGGGG	0.0	0.0	0.0	0.0	0.0	0.0	0.0
TACGTAGGGA	0.0	0.0	0.0	0.0	0.0	0.0	0.0
TACGGAAGAT	0.0	0.0	0.0	0.0	0.0	0.0	0.0
TACGTAGGTG	0.0	0.0	0.0	0.0	0.0	0.0	0.0
TACGTATGGG	0.0	0.0	0.0	0.0	0.0	0.0	0.0
TACGTAGGGA	0.0	0.0	0.0	0.0	0.0	0.0	0.0

TACGTAGGG	0.0	0.0	0.0	0.0	0.0	0.0	0.0
TACGTATGGA	0.0	0.0	0.0	0.0	0.0	0.0	0.0
TACGTAGGGT	0.0	0.0	0.0	0.0	0.0	0.0	0.0
TACGTAGGGA	0.0	0.0	0.0	0.0	0.0	0.0	0.0
TACGAAGGG	0.0	0.0	0.0	0.0	0.0	0.0	0.0
TACGGAGGAT	0.0	0.0	0.0	0.0	0.0	0.0	0.0
TACGTATGGT	0.0	0.0	0.0	0.0	0.0	0.0	0.0
TACGTATGGT	0.0	0.0	0.0	0.0	0.0	0.0	0.0
CACGTAGGG	0.0	0.0	0.0	0.0	0.0	0.0	0.0
TACGTAGGG	0.0	0.0	0.0	0.0	0.0	0.0	0.0
TACGTAGGTG	0.0	0.0	0.0	0.0	0.0	0.0	0.0
TACGTAGGG	0.0	0.0	0.0	0.0	0.0	0.0	0.0
TACGTAGGG	0.0	0.0	0.0	0.0	0.0	0.0	0.0
TACGTAGGG	0.0	0.0	0.0	0.0	0.0	0.0	0.0
TACGTAGGTG	0.0	0.0	0.0	0.0	0.0	0.0	0.0
AACGTAGGTC	0.0	0.0	0.0	0.0	0.0	0.0	0.0
TACGGAAGGT	0.0	0.0	0.0	0.0	0.0	0.0	0.0
TACGTAGGGA	0.0	0.0	0.0	0.0	0.0	0.0	0.0
TACGTAGGGT	0.0	0.0	0.0	0.0	0.0	0.0	0.0
AACGTAGGTC	0.0	0.0	0.0	0.0	0.0	0.0	0.0
TACGGAGGAT	0.0	0.0	0.0	0.0	0.0	0.0	0.0
TACGGAGGAT	0.0	0.0	0.0	0.0	0.0	0.0	0.0
TACGTAGGTG	0.0	0.0	0.0	0.0	0.0	0.0	0.0
TACGTATGGA	0.0	0.0	0.0	0.0	0.0	0.0	0.0
TACGTAGGTG	0.0	0.0	0.0	0.0	0.0	0.0	0.0
TACGTAGGTG	0.0	0.0	0.0	0.0	0.0	0.0	0.0
TACGTATGGA	0.0	0.0	0.0	0.0	0.0	0.0	0.0
TACGGAGGAT	0.0	0.0	0.0	0.0	0.0	0.0	0.0
TACGTAGGGA	0.0	0.0	0.0	0.0	0.0	0.0	0.0
TACGTAGGGA	0.0	0.0	0.0	0.0	0.0	0.0	0.0
TACGTAGGGA	0.0	0.0	0.0	0.0	0.0	0.0	0.0
AACGTAGGTC	0.0	0.0	0.0	0.0	0.0	0.0	0.0
TACGTATGGT	0.0	0.0	0.0	0.0	0.0	0.0	0.0
TACGTAGGG	0.0	0.0	0.0	0.0	0.0	0.0	0.0
TACGTAGGG	0.0	0.0	0.0	0.0	0.0	0.0	0.0
TACGTAGGTG	0.0	0.0	0.0	0.0	0.0	0.0	0.0
TACGTAGGG	0.0	0.0	0.0	0.0	0.0	0.0	0.0
TACGTAGGTG	0.0	0.0	0.0	0.0	0.0	0.0	0.0
TACGTAGGGA	0.0	0.0	0.0	0.0	0.0	0.0	0.0
TACGTAGGGA	0.0	0.0	0.0	0.0	0.0	0.0	0.0
TACGTAGGG	0.0	0.0	0.0	0.0	0.0	0.0	0.0
TACGTAGGCG	0.0	0.0	0.0	0.0	0.0	0.0	0.0
TACGTAGGTG	0.0	0.0	0.0	0.0	0.0	0.0	0.0
TACGTAGGGA	0.0	0.0	0.0	0.0	0.0	0.0	0.0
TACGTAGGG	0.0	0.0	0.0	0.0	0.0	0.0	0.0
TACGTAGGGA	0.0	0.0	0.0	0.0	0.0	0.0	0.0
AACGTAGGTC	0.0	0.0	0.0	0.0	0.0	0.0	0.0

AACGTAGGTC	0.0	0.0	0.0	0.0	0.0	0.0	0.0
AACGTAGGTC	0.0	0.0	0.0	0.0	0.0	0.0	0.0
TACGTAGGGG	0.0	0.0	0.0	0.0	0.0	0.0	0.0
TACGTAGGTG	0.0	0.0	0.0	0.0	0.0	0.0	0.0
TACGTAGGTG	0.0	0.0	0.0	0.0	0.0	0.0	0.0
TACGTAGGTG	0.0	0.0	0.0	0.0	0.0	0.0	0.0
AACGTAGGGT	0.0	0.0	0.0	0.0	0.0	0.0	0.0
TACGTAGGGG	0.0	0.0	0.0	0.0	0.0	0.0	0.0
TACGTAGGTG	0.0	0.0	0.0	0.0	0.0	0.0	0.0
TACGTAGGGG	0.0	0.0	0.0	0.0	0.0	0.0	0.0
TACGTAGGTG	0.0	0.0	0.0	0.0	0.0	0.0	0.0
TACGTAGGTG	0.0	0.0	0.0	0.0	0.0	0.0	0.0
AGCGTAGGTC	0.0	0.0	0.0	0.0	0.0	0.0	0.0
TACGTAGGGG	0.0	0.0	0.0	0.0	0.0	0.0	0.0
TACGTAGGGG	0.0	0.0	0.0	0.0	0.0	0.0	0.0
TACGTAGGGG	0.0	0.0	0.0	0.0	0.0	0.0	0.0
TACGTATGGT	0.0	0.0	0.0	0.0	0.0	0.0	0.0
TACGGAGGAT	0.0	0.0	0.0	0.0	0.0	0.0	0.0
TACGTATGGT	0.0	0.0	0.0	0.0	0.0	0.0	0.0
TACGTAGGTG	0.0	0.0	0.0	0.0	0.0	0.0	0.0
TACGTAGGTG	0.0	0.0	0.0	0.0	0.0	0.0	0.0
TACGTAGGGG	0.0	0.0	0.0	0.0	0.0	0.0	0.0
TACGTAGGGG	0.0	0.0	0.0	0.0	0.0	0.0	0.0
AACGTAGGGT	0.0	0.0	0.0	0.0	0.0	0.0	0.0
AACGTAGGTC	0.0	0.0	0.0	0.0	0.0	0.0	0.0
TACGTAGGTG	0.0	0.0	0.0	0.0	0.0	0.0	0.0
TACGTAGGGG	0.0	0.0	0.0	0.0	0.0	0.0	0.0
TACGTGTGGA	0.0	0.0	0.0	0.0	0.0	0.0	0.0
TACGTAGGTG	0.0	0.0	0.0	0.0	0.0	0.0	0.0
TACGTATGGT	0.0	0.0	0.0	0.0	0.0	0.0	0.0
TACGTAGGGG	0.0	0.0	0.0	0.0	0.0	0.0	0.0
TACGTAGGGG	0.0	0.0	0.0	0.0	0.0	0.0	0.0
TACAGAGGGG	0.0	0.0	0.0	0.0	0.0	0.0	0.0
AACGTAGGTC	0.0	0.0	0.0	0.0	0.0	0.0	0.0
TACGTAGGTG	0.0	0.0	0.0	0.0	0.0	0.0	0.0
TACGTATGGT	0.0	0.0	0.0	0.0	0.0	0.0	0.0
TACGGAGGAT	0.0	0.0	0.0	0.0	0.0	0.0	0.0
TACGGAGGGT	0.0	0.0	0.0	0.0	0.0	0.0	0.0
TACGTATGGG	0.0	0.0	0.0	0.0	0.0	0.0	0.0
TACGTAGGTG	0.0	0.0	0.0	0.0	0.0	0.0	0.0
TACGTATGGA	0.0	0.0	0.0	0.0	0.0	0.0	0.0
TACGTAGGGG	0.0	0.0	0.0	0.0	0.0	0.0	0.0
TACGTAGGGG	0.0	0.0	0.0	0.0	0.0	0.0	0.0
TACGTAGGGG	0.0	0.0	0.0	0.0	0.0	0.0	0.0
TACGTATGGA	0.0	0.0	0.0	0.0	0.0	0.0	0.0
TACGTATGGA	0.0	0.0	0.0	0.0	0.0	0.0	0.0
TACGTAGGGG	0.0	0.0	0.0	0.0	0.0	0.0	0.0

TACGTAGGTG	0.0	0.0	0.0	0.0	0.0	0.0	0.0
TACGTAGGGG	0.0	0.0	0.0	0.0	0.0	0.0	0.0
TACGTAGGGG	0.0	0.0	0.0	0.0	0.0	0.0	0.0
TACGTAGGGA	0.0	0.0	0.0	0.0	0.0	0.0	0.0
TACGTATGGA	0.0	0.0	0.0	0.0	0.0	0.0	0.0
TACGTAGGTG	0.0	0.0	0.0	0.0	0.0	0.0	0.0
TACGTAGGTG	0.0	0.0	0.0	0.0	0.0	0.0	0.0
TACGTAGGGA	0.0	0.0	0.0	0.0	0.0	0.0	0.0
TACGTAGGGA	0.0	0.0	0.0	0.0	0.0	0.0	0.0
TACGTAGGGG	0.0	0.0	0.0	0.0	0.0	0.0	0.0
TACGTATGGT	0.0	0.0	0.0	0.0	0.0	0.0	0.0
TACGTAGGTG	0.0	0.0	0.0	0.0	0.0	0.0	0.0
AACGTAGGTG	0.0	0.0	0.0	0.0	0.0	0.0	0.0
TACGTAGGGG	0.0	0.0	0.0	0.0	0.0	0.0	0.0
TACGTATGGT	0.0	0.0	0.0	0.0	0.0	0.0	0.0
TACGAAGGGA	0.0	0.0	0.0	0.0	0.0	0.0	0.0
TACGTAGGTG	0.0	0.0	0.0	0.0	0.0	0.0	0.0
TACGTAGGTG	0.0	0.0	0.0	0.0	0.0	0.0	0.0
TACGGAGGGT	0.0	0.0	0.0	0.0	0.0	0.0	0.0
TACGTAGGTG	0.0	0.0	0.0	0.0	0.0	0.0	0.0
TACGTAGGGG	0.0	0.0	0.0	0.0	0.0	0.0	0.0
TACGTAGGGG	0.0	0.0	0.0	0.0	0.0	0.0	0.0
GACAGAGGAT	0.0	0.0	0.0	0.0	0.0	0.0	0.0
TACGTAGGGA	0.0	0.0	0.0	0.0	0.0	0.0	0.0
TACGTAGGGG	0.0	0.0	0.0	0.0	0.0	0.0	0.0
TACGTATGGT	0.0	0.0	0.0	0.0	0.0	0.0	0.0
TACGTAGGGA	0.0	0.0	0.0	0.0	0.0	0.0	0.0
TACGTAGGTG	0.0	0.0	0.0	0.0	0.0	0.0	0.0
TACGTAGGGT	0.0	0.0	0.0	0.0	0.0	0.0	0.0
TACGGAAGGT	0.0	0.0	0.0	0.0	0.0	0.0	0.0
TACGGAAGGT	0.0	0.0	0.0	0.0	0.0	0.0	0.0
TACGGAAGGT	0.0	0.0	0.0	0.0	0.0	0.0	0.0
TACGTATGTT	0.0	0.0	0.0	0.0	0.0	0.0	0.0
TACGTAGGGG	0.0	0.0	0.0	0.0	0.0	0.0	0.0
TACGGGGGAT	0.0	0.0	0.0	0.0	0.0	0.0	0.0
TACGTAGGTG	0.0	0.0	0.0	0.0	0.0	0.0	0.0
TACGTAGGGG	0.0	0.0	0.0	0.0	0.0	0.0	0.0
TACGTAGGTT	0.0	0.0	0.0	0.0	0.0	0.0	0.0
TACGTAGGGG	0.0	0.0	0.0	0.0	0.0	0.0	0.0
TACGGGGGAT	0.0	0.0	0.0	0.0	0.0	0.0	0.0
TACGTAGGTG	0.0	0.0	0.0	0.0	0.0	0.0	0.0
TACGTATGGT	0.0	0.0	0.0	0.0	0.0	0.0	0.0
TACGTAGGGG	0.0	0.0	0.0	0.0	0.0	0.0	0.0
TACGTAGGGA	0.0	0.0	0.0	0.0	0.0	0.0	0.0
TACGTAGGGG	0.0	0.0	0.0	0.0	0.0	0.0	0.0
TACGTAGGTG	0.0	0.0	0.0	0.0	0.0	0.0	0.0
TACGTATGGT	0.0	0.0	0.0	0.0	0.0	0.0	0.0

TACGTAGGGA	0.0	0.0	0.0	0.0	0.0	0.0	0.0
TACGTAGGTG	0.0	0.0	0.0	0.0	0.0	0.0	0.0
TACGGAGGAT	0.0	0.0	0.0	0.0	0.0	0.0	0.0
TACGTATGGT	0.0	0.0	0.0	0.0	0.0	0.0	0.0
TACGGAGGGT	0.0	0.0	0.0	0.0	0.0	0.0	0.0
TACGTATGGT	0.0	0.0	0.0	0.0	0.0	0.0	0.0
TACGGAGGAT	0.0	0.0	0.0	0.0	0.0	0.0	0.0
TACGTAGGGA	0.0	0.0	0.0	0.0	0.0	0.0	0.0
TACGTATGGT	0.0	0.0	0.0	0.0	0.0	0.0	0.0
TACGTAGGTG	0.0	0.0	0.0	0.0	0.0	0.0	0.0
TACGTAGGTG	0.0	0.0	0.0	0.0	0.0	0.0	0.0
TACGGAAGGT	0.0	0.0	0.0	0.0	0.0	0.0	0.0
AACATAGGGG	0.0	0.0	0.0	0.0	0.0	0.0	0.0
TACGGAAGGT	0.0	0.0	0.0	0.0	0.0	0.0	0.0
TACGTAGGTG	0.0	0.0	0.0	0.0	0.0	0.0	0.0
TACGTAGGGG	0.0	0.0	0.0	0.0	0.0	0.0	0.0
TACGTAGGTG	0.0	0.0	0.0	0.0	0.0	0.0	0.0
TACGTATGGT	0.0	0.0	0.0	0.0	0.0	0.0	0.0
TACGTAGGGG	0.0	0.0	0.0	0.0	0.0	0.0	0.0
TACGTATGGG	0.0	0.0	0.0	0.0	0.0	0.0	0.0
TACGTAGGTG	0.0	0.0	0.0	0.0	0.0	0.0	0.0
TACGTATGGT	0.0	0.0	0.0	0.0	0.0	0.0	0.0
TACGTAGGTG	0.0	0.0	0.0	0.0	0.0	0.0	0.0
TACGTAGGGA	0.0	0.0	0.0	0.0	0.0	0.0	0.0
TACGTATGGT	0.0	0.0	0.0	0.0	0.0	0.0	0.0
TACGTAGGTG	0.0	0.0	0.0	0.0	0.0	0.0	0.0
TACGGAGGAT	0.0	0.0	0.0	0.0	0.0	0.0	0.0
TACGTAGGTG	0.0	0.0	0.0	0.0	0.0	0.0	0.0
TACGTAGGGG	0.0	0.0	0.0	0.0	0.0	0.0	0.0
TACGTAGGTG	0.0	0.0	0.0	0.0	0.0	0.0	0.0
AACGTAGGGT	0.0	0.0	0.0	0.0	0.0	0.0	0.0
TACGTATGGA	0.0	0.0	0.0	0.0	0.0	0.0	0.0
TACGTAGGGG	0.0	0.0	0.0	0.0	0.0	0.0	0.0
TGCGTAAGGG	0.0	0.0	0.0	0.0	0.0	0.0	0.0
TACGGAGGTG	0.0	0.0	0.0	0.0	0.0	0.0	0.0
GACATAGGGG	0.0	0.0	0.0	0.0	0.0	0.0	0.0
TACGTAGGGG	0.0	0.0	0.0	0.0	0.0	0.0	0.0
TACGTAGGGG	0.0	0.0	0.0	0.0	0.0	0.0	0.0
TACGTAGGGG	0.0	0.0	0.0	0.0	0.0	0.0	0.0
TACGTAGGGG	0.0	0.0	0.0	0.0	0.0	0.0	0.0
TACGGAGGAT	0.0	0.0	0.0	0.0	0.0	0.0	0.0
TACGGAGGGT	0.0	0.0	0.0	0.0	0.0	0.0	0.0
TACGTAGGGG	0.0	0.0	0.0	0.0	0.0	0.0	0.0
TACGTAGGGG	0.0	0.0	0.0	0.0	0.0	0.0	0.0
TACGTAGGGG	0.0	0.0	0.0	0.0	0.0	0.0	0.0
TACGTAGGTG	0.0	0.0	0.0	0.0	0.0	0.0	0.0
TACGTATGGT	0.0	0.0	0.0	0.0	0.0	0.0	0.0

TACGTAGGGG	0.0	0.0	0.0	0.0	0.0	0.0	0.0
TACGTAGGTG	0.0	0.0	0.0	0.0	0.0	0.0	0.0
TACGTAGGGA	0.0	0.0	0.0	0.0	0.0	0.0	0.0
TACGTAGGTG	0.0	0.0	0.0	0.0	0.0	0.0	0.0
TACGTAGGGT	0.0	0.0	0.0	0.0	0.0	0.0	0.0
TACGGAGGGT	0.0	0.0	0.0	0.0	0.0	0.0	0.0
TACGTAGGGA	0.0	0.0	0.0	0.0	0.0	0.0	0.0
TACGTAGGGC	0.0	0.0	0.0	0.0	0.0	0.0	0.0
TACAGAGGGT	0.0	0.0	0.0	0.0	0.0	0.0	0.0
TACGGAGGAT	0.0	0.0	0.0	0.0	0.0	0.0	0.0
TACGTAGGTG	0.0	0.0	0.0	0.0	0.0	0.0	0.0
TACGTAGGGT	0.0	0.0	0.0	0.0	0.0	0.0	0.0
TACGTAGGGG	0.0	0.0	0.0	0.0	0.0	0.0	0.0
TACGTAGGGG	0.0	0.0	0.0	0.0	0.0	0.0	0.0
TACGTAGGGG	0.0	0.0	0.0	0.0	0.0	0.0	0.0
TACGTAGGGG	0.0	0.0	0.0	0.0	0.0	0.0	0.0
TACGTAGGGG	0.0	0.0	0.0	0.0	0.0	0.0	0.0
TACGTAGGGG	0.0	0.0	0.0	0.0	0.0	0.0	0.0
TACGGAGGAT	0.0	0.0	0.0	0.0	0.0	0.0	0.0
TACGTATGGT	0.0	0.0	0.0	0.0	0.0	0.0	0.0
TACGGAGGAT	0.0	0.0	0.0	0.0	0.0	0.0	0.0
TACGTAGGGA	0.0	0.0	0.0	0.0	0.0	0.0	0.0
TACGTAGGGG	0.0	0.0	0.0	0.0	0.0	0.0	0.0
TACGTAGGGG	0.0	0.0	0.0	0.0	0.0	0.0	0.0
TACGTAGGGG	0.0	0.0	0.0	0.0	0.0	0.0	0.0
TACGTAGGGG	0.0	0.0	0.0	0.0	0.0	0.0	0.0
TACGTATGGT	0.0	0.0	0.0	0.0	0.0	0.0	0.0
TACGTAGGTG	0.0	0.0	0.0	0.0	0.0	0.0	0.0
TACGTAGGGG	0.0	0.0	0.0	0.0	0.0	0.0	0.0
TACGTAGGGG	0.0	0.0	0.0	0.0	0.0	0.0	0.0
TACGTAGGGG	0.0	0.0	0.0	0.0	0.0	0.0	0.0
TACGTAGGGA	0.0	0.0	0.0	0.0	0.0	0.0	0.0
TACGTAGGGA	0.0	0.0	0.0	0.0	0.0	0.0	0.0
TACGTAGGTG	0.0	0.0	0.0	0.0	0.0	0.0	0.0
TACGTAGGTG	0.0	0.0	0.0	0.0	0.0	0.0	0.0
TACGTAGGTG	0.0	0.0	0.0	0.0	0.0	0.0	0.0
TACGTAGGAG	0.0	0.0	0.0	0.0	0.0	0.0	0.0
TACGTAGGTG	0.0	0.0	0.0	0.0	0.0	0.0	0.0
TACGTAGGGA	0.0	0.0	0.0	0.0	0.0	0.0	0.0
TACGTAGGTG	0.0	0.0	0.0	0.0	0.0	0.0	0.0
TACGTAGGGG	0.0	0.0	0.0	0.0	0.0	0.0	0.0
TACGTAGGTG	0.0	0.0	0.0	0.0	0.0	0.0	0.0
TACGTAGGGG	0.0	0.0	0.0	0.0	0.0	0.0	0.0
TACGTAGGTG	0.0	0.0	0.0	0.0	0.0	0.0	0.0
TACGTAGGGA	0.0	0.0	0.0	0.0	0.0	0.0	0.0
TACGTAGGTG	0.0	0.0	0.0	0.0	0.0	0.0	0.0
TACGTAGGTG	0.0	0.0	0.0	0.0	0.0	0.0	0.0

TACGTAGGGC	0.0	0.0	0.0	0.0	0.0	0.0	0.0
TACGTATGGA	0.0	0.0	0.0	0.0	0.0	0.0	0.0
TACGTAGGGA	0.0	0.0	0.0	0.0	0.0	0.0	0.0
TACGTAGGTG	0.0	0.0	0.0	0.0	0.0	0.0	0.0
TACGTAGGGA	0.0	0.0	0.0	0.0	0.0	0.0	0.0
TACGTAGGGC	0.0	0.0	0.0	0.0	0.0	0.0	0.0
TACGTAGGGC	0.0	0.0	0.0	0.0	0.0	0.0	0.0
TACGTATGGT	0.0	0.0	0.0	0.0	0.0	0.0	0.0
TACGGAGGAT	0.0	0.0	0.0	0.0	0.0	0.0	0.0
TACGTAGGGT	0.0	0.0	0.0	0.0	0.0	0.0	0.0
AACGTAGGTC	0.0	0.0	0.0	0.0	0.0	0.0	0.0
TACGTAGGTG	0.0	0.0	0.0	0.0	0.0	0.0	0.0
TACGTAGGGA	0.0	0.0	0.0	0.0	0.0	0.0	0.0
TACGTAGGGC	0.0	0.0	0.0	0.0	0.0	0.0	0.0
TACGTAGGGA	0.0	0.0	0.0	0.0	0.0	0.0	0.0
TACGTAGGGA	0.0	0.0	0.0	0.0	0.0	0.0	0.0
TACGTAGGGC	0.0	0.0	0.0	0.0	0.0	0.0	0.0
TACGTAGGTG	0.0	0.0	0.0	0.0	0.0	0.0	0.0
TACGTAGGTC	0.0	0.0	0.0	0.0	0.0	0.0	0.0
TACGTAGGGC	0.0	0.0	0.0	0.0	0.0	0.0	0.0
TACGTAGGTG	0.0	0.0	0.0	0.0	0.0	0.0	0.0
TACGGAGGAT	0.0	0.0	0.0	0.0	0.0	0.0	0.0
TACGTATGGT	0.0	0.0	0.0	0.0	0.0	0.0	0.0
TACGGAGGAT	0.0	0.0	0.0	0.0	0.0	0.0	0.0
TACGTAGGGC	0.0	0.0	0.0	0.0	0.0	0.0	0.0
TACGTAGGGA	0.0	0.0	0.0	0.0	0.0	0.0	0.0
TACGGAGGAT	0.0	0.0	0.0	0.0	0.0	0.0	0.0
TACGGAGGGT	0.0	0.0	0.0	0.0	0.0	0.0	0.0
AACGTAGGTG	0.0	0.0	0.0	0.0	0.0	0.0	0.0
TACGTAGGGA	0.0	0.0	0.0	0.0	0.0	0.0	0.0
TACGTAGGGC	0.0	0.0	0.0	0.0	0.0	0.0	0.0
TACGTAGGGA	0.0	0.0	0.0	0.0	0.0	0.0	0.0
TACGTAGGGA	0.0	0.0	0.0	0.0	0.0	0.0	0.0
TACGTAGGTG	0.0	0.0	0.0	0.0	0.0	0.0	0.0
TACGTAGGGC	0.0	0.0	0.0	0.0	0.0	0.0	0.0
AACGTAGGTG	0.0	0.0	0.0	0.0	0.0	0.0	0.0
AACGTAGGTC	0.0	0.0	0.0	0.0	0.0	0.0	0.0
TACGTAGGGA	0.0	0.0	0.0	0.0	0.0	0.0	0.0
TACGGAGGGT	0.0	0.0	0.0	0.0	0.0	0.0	0.0
TACGTAGGGC	0.0	0.0	0.0	0.0	0.0	0.0	0.0
AACGTAGGTC	0.0	0.0	0.0	0.0	0.0	0.0	0.0
TACGTATGGA	0.0	0.0	0.0	0.0	0.0	0.0	0.0
TACGTATGGT	0.0	0.0	0.0	0.0	0.0	0.0	0.0
TACGTAGGGA	0.0	0.0	0.0	0.0	0.0	0.0	0.0
TACGTAGGTG	0.0	0.0	0.0	0.0	0.0	0.0	0.0
TACGTAGGGA	0.0	0.0	0.0	0.0	0.0	0.0	0.0
TACGTAGGGC	0.0	0.0	0.0	0.0	0.0	0.0	0.0

TACGTAGGGA	0.0	0.0	0.0	0.0	0.0	0.0	0.0
TACGAAGGGC	0.0	0.0	0.0	0.0	0.0	0.0	0.0
AACGTAGGGT	0.0	0.0	0.0	0.0	0.0	0.0	0.0
TACGTAGGTG	0.0	0.0	0.0	0.0	0.0	0.0	0.0
TACGTAGGGT	0.0	0.0	0.0	0.0	0.0	0.0	0.0
TACGTAGGGG	0.0	0.0	0.0	0.0	0.0	0.0	0.0
TACGTAGGTG	0.0	0.0	0.0	0.0	0.0	0.0	0.0
TACGTAGGGC	0.0	0.0	0.0	0.0	0.0	0.0	0.0
TACGTAGGTG	0.0	0.0	0.0	0.0	0.0	0.0	0.0
TACGTAGGTG	0.0	0.0	0.0	0.0	0.0	0.0	0.0
TCCTTCTTTTT	0.0	0.0	0.0	0.0	0.0	0.0	0.0
TACGTATGGT	0.0	0.0	0.0	0.0	0.0	0.0	0.0
TACGTAGGTG	0.0	0.0	0.0	0.0	0.0	0.0	0.0
TACGAAGGGT	0.0	0.0	0.0	0.0	0.0	0.0	0.0
TACGTAGGTG	0.0	0.0	0.0	0.0	0.0	0.0	0.0
TACGTAGGGG	0.0	0.0	0.0	0.0	0.0	0.0	0.0
TACGGAAGGT	0.0	0.0	0.0	0.0	0.0	0.0	0.0
TACGTAGGTG	0.0	0.0	0.0	0.0	0.0	0.0	0.0
TACGGAAGGT	0.0	0.0	0.0	0.0	0.0	0.0	0.0
TACGGAAGGT	0.0	0.0	0.0	0.0	0.0	0.0	0.0
TACGGAAGGT	0.0	0.0	0.0	0.0	0.0	0.0	0.0
TACGTAGGTG	0.0	0.0	0.0	0.0	0.0	0.0	0.0
TACGTAGGGG	0.0	0.0	0.0	0.0	0.0	0.0	0.0
TACGTAGGTG	0.0	0.0	0.0	0.0	0.0	0.0	0.0
TACGGAAGGT	0.0	0.0	0.0	0.0	0.0	0.0	0.0
TACGGAGGAT	0.0	0.0	0.0	0.0	0.0	0.0	0.0
TACGTAGGGG	0.0	0.0	0.0	0.0	0.0	0.0	0.0
TACGTAGGGG	0.0	0.0	0.0	0.0	0.0	0.0	0.0
TACGTAGGGG	0.0	0.0	0.0	0.0	0.0	0.0	0.0
TACGGAAGGT	0.0	0.0	0.0	0.0	0.0	0.0	0.0
TACGGAGGAT	0.0	0.0	0.0	0.0	0.0	0.0	0.0
TACGTAGGGG	0.0	0.0	0.0	0.0	0.0	0.0	0.0
TACGTAGGTG	0.0	0.0	0.0	0.0	0.0	0.0	0.0
TACGGAAGAT	0.0	0.0	0.0	0.0	0.0	0.0	0.0
TACGTAGGGA	0.0	0.0	0.0	0.0	0.0	0.0	0.0
TACGTAGGGG	0.0	0.0	0.0	0.0	0.0	0.0	0.0
TACGGAGGGT	0.0	0.0	0.0	0.0	0.0	0.0	0.0
TACGTAGGGG	0.0	0.0	0.0	0.0	0.0	0.0	0.0
TACGTAGGTG	0.0	0.0	0.0	0.0	0.0	0.0	0.0
TACGTAGGTG	0.0	0.0	0.0	0.0	0.0	0.0	0.0
TACGTAGGGA	0.0	0.0	0.0	0.0	0.0	0.0	0.0
TACGTAGGGG	0.0	0.0	0.0	0.0	0.0	0.0	0.0
TACGTAGGGG	0.0	0.0	0.0	0.0	0.0	0.0	0.0
TACGGAGGAT	0.0	0.0	0.0	0.0	0.0	0.0	0.0
TGCGTAGGTG	0.0	0.0	0.0	0.0	0.0	0.0	0.0
TACGTAGGGG	0.0	0.0	0.0	0.0	0.0	0.0	0.0
TACGGAAGGT	0.0	0.0	0.0	0.0	0.0	0.0	0.0

TACGTAGGG	0.0	0.0	0.0	0.0	0.0	0.0	0.0
TACGTAGGAT	0.0	0.0	0.0	0.0	0.0	0.0	0.0
TACGTAGGG	0.0	0.0	0.0	0.0	0.0	0.0	0.0
AACGTAGGTC	0.0	0.0	0.0	0.0	0.0	0.0	0.0
AACGTAGGTC	0.0	0.0	0.0	0.0	0.0	0.0	0.0
TACGTAGGTG	0.0	0.0	0.0	0.0	0.0	0.0	0.0
TACGTAGGCG	0.0	0.0	0.0	0.0	0.0	0.0	0.0
TACGTAGGG	0.0	0.0	0.0	0.0	0.0	0.0	0.0
TACGTAGGTG	0.0	0.0	0.0	0.0	0.0	0.0	0.0
TACGGAGGAT	0.0	0.0	0.0	0.0	0.0	0.0	0.0
TACGGAAGGT	0.0	0.0	0.0	0.0	0.0	0.0	0.0
TACGGAAGGT	0.0	0.0	0.0	0.0	0.0	0.0	0.0
TACGGAAGAT	0.0	0.0	0.0	0.0	0.0	0.0	0.0
TACGGAGGGT	0.0	0.0	0.0	0.0	0.0	0.0	0.0
TACGGAGGAT	0.0	0.0	0.0	0.0	0.0	0.0	0.0
TACGGAAGGT	0.0	0.0	0.0	0.0	0.0	0.0	0.0
AACGTAGGGT	0.0	0.0	0.0	0.0	0.0	0.0	0.0
TACGTATGGA	0.0	0.0	0.0	0.0	0.0	0.0	0.0
TACGTAGGG	0.0	0.0	0.0	0.0	0.0	0.0	0.0
TACGTATGGA	0.0	0.0	0.0	0.0	0.0	0.0	0.0
TACGTAGGTG	0.0	0.0	0.0	0.0	0.0	0.0	0.0
TACGTAGGG	0.0	0.0	0.0	0.0	0.0	0.0	0.0
TACGTAGGTG	0.0	0.0	0.0	0.0	0.0	0.0	0.0
TACGTAGGTG	0.0	0.0	0.0	0.0	0.0	0.0	0.0
TACGTAGGGA	0.0	0.0	0.0	0.0	0.0	0.0	0.0
TACGTAGGGA	0.0	0.0	0.0	0.0	0.0	0.0	0.0
TACGTAGGGA	0.0	0.0	0.0	0.0	0.0	0.0	0.0
AACGTAGGT	0.0	0.0	0.0	0.0	0.0	0.0	0.0
TACGGAAGGT	0.0	0.0	0.0	0.0	0.0	0.0	0.0
TACGTAGGTG	0.0	0.0	0.0	0.0	0.0	0.0	0.0
TACGGAAGGT	0.0	0.0	0.0	0.0	0.0	0.0	0.0
TACGTAGGG	0.0	0.0	0.0	0.0	0.0	0.0	0.0
TACGTAGGG	0.0	0.0	0.0	0.0	0.0	0.0	0.0
TACGTATGGA	0.0	0.0	0.0	0.0	0.0	0.0	0.0
TACGTATGGT	0.0	0.0	0.0	0.0	0.0	0.0	0.0
TACGTAGGGA	0.0	0.0	0.0	0.0	0.0	0.0	0.0
TACGTAGGTG	0.0	0.0	0.0	0.0	0.0	0.0	0.0
TACGTAGGTG	0.0	0.0	0.0	0.0	0.0	0.0	0.0
TACGTATGGA	0.0	0.0	0.0	0.0	0.0	0.0	0.0
TACGTAGGG	0.0	0.0	0.0	0.0	0.0	0.0	0.0
TACGTAGGTG	0.0	0.0	0.0	0.0	0.0	0.0	0.0
TACGTAGGG	0.0	0.0	0.0	0.0	0.0	0.0	0.0
TACGTAGGTG	0.0	0.0	0.0	0.0	0.0	0.0	0.0
TACGTAGGTC	0.0	0.0	0.0	0.0	0.0	0.0	0.0
TACGTAGGTG	0.0	0.0	0.0	0.0	0.0	0.0	0.0
TACGTAGGTG	0.0	0.0	0.0	0.0	0.0	0.0	0.0
TACGGAGGGT	0.0	0.0	0.0	0.0	0.0	0.0	0.0

TACGTAGGTG	0.0	0.0	0.0	0.0	0.0	0.0	0.0
AACGTAGGTC	0.0	0.0	0.0	0.0	0.0	0.0	0.0
TACGTATGGT	0.0	0.0	0.0	0.0	0.0	0.0	0.0
TACGTAGGGG	0.0	0.0	0.0	0.0	0.0	0.0	0.0
TACGTAGGGG	0.0	0.0	0.0	0.0	0.0	0.0	0.0
AACGTAGGTG	0.0	0.0	0.0	0.0	0.0	0.0	0.0
TACGTATGGA	0.0	0.0	0.0	0.0	0.0	0.0	0.0
TACATAGGTC	0.0	0.0	0.0	0.0	0.0	0.0	0.0
TACGTAGGGG	0.0	0.0	0.0	0.0	0.0	0.0	0.0
TACGGAGGGT	0.0	0.0	0.0	0.0	0.0	0.0	0.0
TACGTAGGGG	0.0	0.0	0.0	0.0	0.0	0.0	0.0
TACGTAGGGG	0.0	0.0	0.0	0.0	0.0	0.0	0.0
TACGTAGGGG	0.0	0.0	0.0	0.0	0.0	0.0	0.0
AACGTAGGTC	0.0	0.0	0.0	0.0	0.0	0.0	0.0
TACGGAAGGT	0.0	0.0	0.0	0.0	0.0	0.0	0.0
TACGTAGGGG	0.0	0.0	0.0	0.0	0.0	0.0	0.0
TACGTAGGGG	0.0	0.0	0.0	0.0	0.0	0.0	0.0
TACGTAGGGG	0.0	0.0	0.0	0.0	0.0	0.0	0.0
AACGTAGGTC	0.0	0.0	0.0	0.0	0.0	0.0	0.0
TACATAGGGG	0.0	0.0	0.0	0.0	0.0	0.0	0.0
TACGTAGGTG	0.0	0.0	0.0	0.0	0.0	0.0	0.0
TACGGAGGAT	0.0	0.0	0.0	0.0	0.0	0.0	0.0
TACGTAGGGA	0.0	0.0	0.0	0.0	0.0	0.0	0.0
TACGTAGGGG	0.0	0.0	0.0	0.0	0.0	0.0	0.0
TACGGGGGGT	0.0	0.0	0.0	0.0	0.0	0.0	0.0
TACGTAGGTG	0.0	0.0	0.0	0.0	0.0	0.0	0.0
TACATAGGGG	0.0	0.0	0.0	0.0	0.0	0.0	0.0
TACGTAGGGA	0.0	0.0	0.0	0.0	0.0	0.0	0.0
TACGTAGGTG	0.0	0.0	0.0	0.0	0.0	0.0	0.0
TACGTAGGGG	0.0	0.0	0.0	0.0	0.0	0.0	0.0
TACGGAGGGT	0.0	0.0	0.0	0.0	0.0	0.0	0.0
TACGTAGGTG	0.0	0.0	0.0	0.0	0.0	0.0	0.0
TACGTAGGGA	0.0	0.0	0.0	0.0	0.0	0.0	0.0
TACGTAGGTG	0.0	0.0	0.0	0.0	0.0	0.0	0.0
TACGTAGGTG	0.0	0.0	0.0	0.0	0.0	0.0	0.0
TACGTAGGAG	0.0	0.0	0.0	0.0	0.0	0.0	0.0
TACGTAGGTG	0.0	0.0	0.0	0.0	0.0	0.0	0.0
TACGTAGGGA	0.0	0.0	0.0	0.0	0.0	0.0	0.0
TACGTAGGGG	0.0	0.0	0.0	0.0	0.0	0.0	0.0
TACGTAGGGG	0.0	0.0	0.0	0.0	0.0	0.0	0.0
TACGTAGGTG	0.0	0.0	0.0	0.0	0.0	0.0	0.0
TACGTAGGTG	0.0	0.0	0.0	0.0	0.0	0.0	0.0
TACGTAGGGT	0.0	0.0	0.0	0.0	0.0	0.0	0.0
TACGTAGGTG	0.0	0.0	0.0	0.0	0.0	0.0	0.0
TACGTAGGGG	0.0	0.0	0.0	0.0	0.0	0.0	0.0
TACGTAGGGG	0.0	0.0	0.0	0.0	0.0	0.0	0.0
TACGTAGGGG	0.0	0.0	0.0	0.0	0.0	0.0	0.0

TACGTAGGGA	0.0	0.0	0.0	0.0	0.0	0.0	0.0
TACGTAGGTG	0.0	0.0	0.0	0.0	0.0	0.0	0.0
TACGTATGGA	0.0	0.0	0.0	0.0	0.0	0.0	0.0
TACGTAGGGG	0.0	0.0	0.0	0.0	0.0	0.0	0.0
TACGTAGGGG	0.0	0.0	0.0	0.0	0.0	0.0	0.0
TACGGAGGAT	0.0	0.0	0.0	0.0	0.0	0.0	0.0
TACGTAGGTG	0.0	0.0	0.0	0.0	0.0	0.0	0.0
AACGTAGGTC	0.0	0.0	0.0	0.0	0.0	0.0	0.0
TACGTAGGTG	0.0	0.0	0.0	0.0	0.0	0.0	0.0
TACGTAGGGG	0.0	0.0	0.0	0.0	0.0	0.0	0.0
TACGTAGGGG	0.0	0.0	0.0	0.0	0.0	0.0	0.0
TACGTAGGTG	0.0	0.0	0.0	0.0	0.0	0.0	0.0
TACGTAGGTG	0.0	0.0	0.0	0.0	0.0	0.0	0.0
TACGTAGGGG	0.0	0.0	0.0	0.0	0.0	0.0	0.0
TACGTAGGGG	0.0	0.0	0.0	0.0	0.0	0.0	0.0
TACGTAGGGA	0.0	0.0	0.0	0.0	0.0	0.0	0.0
TACGTAGGGG	0.0	0.0	0.0	0.0	0.0	0.0	0.0
TACGTAGGGA	0.0	0.0	0.0	0.0	0.0	0.0	0.0
TACGTAGGTG	0.0	0.0	0.0	0.0	0.0	0.0	0.0
AACGTAGGTG	0.0	0.0	0.0	0.0	0.0	0.0	0.0
TACGTAGGTG	0.0	0.0	0.0	0.0	0.0	0.0	0.0
TACGGAGGAT	0.0	0.0	0.0	0.0	0.0	0.0	0.0
TACGTAGGGG	0.0	0.0	0.0	0.0	0.0	0.0	0.0
TACGTAGGTG	0.0	0.0	0.0	0.0	0.0	0.0	0.0
TACGTAGGGG	0.0	0.0	0.0	0.0	0.0	0.0	0.0
TACGAAGGTG	0.0	0.0	0.0	0.0	0.0	0.0	0.0
TACGGAGGAT	0.0	0.0	0.0	0.0	0.0	0.0	0.0
TACGTAGGTG	0.0	0.0	0.0	0.0	0.0	0.0	0.0
AACGTAGGTC	0.0	0.0	0.0	0.0	0.0	0.0	0.0
TACGGAAGGT	0.0	0.0	0.0	0.0	0.0	0.0	0.0
TACGGAGGGT	0.0	0.0	0.0	0.0	0.0	0.0	0.0
TACGTAGGTG	0.0	0.0	0.0	0.0	0.0	0.0	0.0
TACGTAGGGG	0.0	0.0	0.0	0.0	0.0	0.0	0.0
GACGTAGGTC	0.0	0.0	0.0	0.0	0.0	0.0	0.0
TACGGAGGAT	0.0	0.0	0.0	0.0	0.0	0.0	0.0
TACGTAGGGG	0.0	0.0	0.0	0.0	0.0	0.0	0.0
TACGGAAGGT	0.0	0.0	0.0	0.0	0.0	0.0	0.0
TACGGAAGGT	0.0	0.0	0.0	0.0	0.0	0.0	0.0
AACGTAGGTC	0.0	0.0	0.0	0.0	0.0	0.0	0.0
TACGTAGGGG	0.0	0.0	0.0	0.0	0.0	0.0	0.0
TACGTATGGT	0.0	0.0	0.0	0.0	0.0	0.0	0.0
TCCTTCTTTTT	0.0	0.0	0.0	0.0	0.0	0.0	0.0
AACGTAGGTC	0.0	0.0	0.0	0.0	0.0	0.0	0.0
TACGTAGGGG	0.0	0.0	0.0	0.0	0.0	0.0	0.0
TACGTAGGGA	0.0	0.0	0.0	0.0	0.0	0.0	0.0
AACGTAGGTC	0.0	0.0	0.0	0.0	0.0	0.0	0.0
TACGTAGGTG	0.0	0.0	0.0	0.0	0.0	0.0	0.0

TACGTAGGG	0.0	0.0	0.0	0.0	0.0	0.0	0.0
TACGTAGGTG	0.0	0.0	0.0	0.0	0.0	0.0	0.0
TACGTAGGGA	0.0	0.0	0.0	0.0	0.0	0.0	0.0
TACGTAGGTG	0.0	0.0	0.0	0.0	0.0	0.0	0.0
TACGTAGGG	0.0	0.0	0.0	0.0	0.0	0.0	0.0
AACGTAGGTC	0.0	0.0	0.0	0.0	0.0	0.0	0.0
AACGTAGGTC	0.0	0.0	0.0	0.0	0.0	0.0	0.0
TACGGAGGAT	0.0	0.0	0.0	0.0	0.0	0.0	0.0
TACGTAGGTG	0.0	0.0	0.0	0.0	0.0	0.0	0.0
TACGGAGGAT	0.0	0.0	0.0	0.0	0.0	0.0	0.0
TACGTAGGG	0.0	0.0	0.0	0.0	0.0	0.0	0.0
TACGTAGGGA	0.0	0.0	0.0	0.0	0.0	0.0	0.0
TACGTAGGTG	0.0	0.0	0.0	0.0	0.0	0.0	0.0
TACGTAGGG	0.0	0.0	0.0	0.0	0.0	0.0	0.0
TACGTAGGG	0.0	0.0	0.0	0.0	0.0	0.0	0.0
TACGTAGGG	0.0	0.0	0.0	0.0	0.0	0.0	0.0
TACGGAGGAT	0.0	0.0	0.0	0.0	0.0	0.0	0.0
TACGTAGGG	0.0	0.0	0.0	0.0	0.0	0.0	0.0
AACGTAGGG	0.0	0.0	0.0	0.0	0.0	0.0	0.0
TACGTAGGG	0.0	0.0	0.0	0.0	0.0	0.0	0.0
TACGTAGGTG	0.0	0.0	0.0	0.0	0.0	0.0	0.0
AACGTAGGTC	0.0	0.0	0.0	0.0	0.0	0.0	0.0
TACGTAGGG	0.0	0.0	0.0	0.0	0.0	0.0	0.0
TACATAGGG	0.0	0.0	0.0	0.0	0.0	0.0	0.0
TACGTAGGG	0.0	0.0	0.0	0.0	0.0	0.0	0.0
TACGTAGGG	0.0	0.0	0.0	0.0	0.0	0.0	0.0
TACGTAGGG	0.0	0.0	0.0	0.0	0.0	0.0	0.0
TACGTAGGG	0.0	0.0	0.0	0.0	0.0	0.0	0.0
TACGTAGGTC	0.0	0.0	0.0	0.0	0.0	0.0	0.0
TACGTAGGG	0.0	0.0	0.0	0.0	0.0	0.0	0.0
TACGTAGGG	0.0	0.0	0.0	0.0	0.0	0.0	0.0
TACGTAGGG	0.0	0.0	0.0	0.0	0.0	0.0	0.0
TACGTAGGG	0.0	0.0	0.0	0.0	0.0	0.0	0.0
TACGTAGGGT	0.0	0.0	0.0	0.0	0.0	0.0	0.0
TACGTAGGTG	0.0	0.0	0.0	0.0	0.0	0.0	0.0
TACGGAGGG	0.0	0.0	0.0	0.0	0.0	0.0	0.0
TACGTAGGTG	0.0	0.0	0.0	0.0	0.0	0.0	0.0
GACGTAGGG	0.0	0.0	0.0	0.0	0.0	0.0	0.0
TACGTAGGTG	0.0	0.0	0.0	0.0	0.0	0.0	0.0
TACAGAGGGT	0.0	0.0	0.0	0.0	0.0	0.0	0.0
TACGGAGGG	0.0	0.0	0.0	0.0	0.0	0.0	0.0
TACGTAGGTG	0.0	0.0	0.0	0.0	0.0	0.0	0.0
TACGGAGGAT	0.0	0.0	0.0	0.0	0.0	0.0	0.0
TACGGAGGAT	0.0	0.0	0.0	0.0	0.0	0.0	0.0
TACGGAGGAT	0.0	0.0	0.0	0.0	0.0	0.0	0.0
TACGTAGGG	0.0	0.0	0.0	0.0	0.0	0.0	0.0
TACGGAGGAT	0.0	0.0	0.0	0.0	0.0	0.0	0.0

TACGGAGGAT	0.0	0.0	0.0	0.0	0.0	0.0	0.0
TACGGAGGAT	0.0	0.0	0.0	0.0	0.0	0.0	0.0
TACGTAGGGG	0.0	0.0	0.0	0.0	0.0	0.0	0.0
TACGGAGGAT	0.0	0.0	0.0	0.0	0.0	0.0	0.0
TACGGAGGAT	0.0	0.0	0.0	0.0	0.0	0.0	0.0
TACGTAGGGG	0.0	0.0	0.0	0.0	0.0	0.0	0.0
TACGGAGGAT	0.0	0.0	0.0	0.0	0.0	0.0	0.0
TACGTAGGGG	0.0	0.0	0.0	0.0	0.0	0.0	0.0
AACGTAGGTC	0.0	0.0	0.0	0.0	0.0	0.0	0.0
AACGTAGGTC	0.0	0.0	0.0	0.0	0.0	0.0	0.0
TACGTATGGT	0.0	0.0	0.0	0.0	0.0	0.0	0.0
AACGTAGGTC	0.0	0.0	0.0	0.0	0.0	0.0	0.0
TACGTAGGGG	0.0	0.0	0.0	0.0	0.0	0.0	0.0
TACGGAGGAT	0.0	0.0	0.0	0.0	0.0	0.0	0.0
TACGTAGGGG	0.0	0.0	0.0	0.0	0.0	0.0	0.0
TACGTATGGT	0.0	0.0	0.0	0.0	0.0	0.0	0.0
TACGTATGGA	0.0	0.0	0.0	0.0	0.0	0.0	0.0
TACGTAGGTG	0.0	0.0	0.0	0.0	0.0	0.0	0.0
TACGTAGGTG	0.0	0.0	0.0	0.0	0.0	0.0	0.0
TACGTAGGGA	0.0	0.0	0.0	0.0	0.0	0.0	0.0
AACGTAGGTC	0.0	0.0	0.0	0.0	0.0	0.0	0.0
TACGGAGGAT	0.0	0.0	0.0	0.0	0.0	0.0	0.0
AACGTATGGT	0.0	0.0	0.0	0.0	0.0	0.0	0.0
TACGTAGGTG	0.0	0.0	0.0	0.0	0.0	0.0	0.0
TACGTAGGTG	0.0	0.0	0.0	0.0	0.0	0.0	0.0
TACGTAGGGG	0.0	0.0	0.0	0.0	0.0	0.0	0.0
TACGTAGGGG	0.0	0.0	0.0	0.0	0.0	0.0	0.0
AACGTAGGTC	0.0	0.0	0.0	0.0	0.0	0.0	0.0
TACGTATGGT	0.0	0.0	0.0	0.0	0.0	0.0	0.0
TACGTAGGGG	0.0	0.0	0.0	0.0	0.0	0.0	0.0
TACGTATGGT	0.0	0.0	0.0	0.0	0.0	0.0	0.0
TACGTAGGGG	0.0	0.0	0.0	0.0	0.0	0.0	0.0
TACGTAGGGG	0.0	0.0	0.0	0.0	0.0	0.0	0.0
AACGTAGGTC	0.0	0.0	0.0	0.0	0.0	0.0	0.0
TACGTAGGGA	0.0	0.0	0.0	0.0	0.0	0.0	0.0
TACGTAGGGG	0.0	0.0	0.0	0.0	0.0	0.0	0.0
TACGTATGGT	0.0	0.0	0.0	0.0	0.0	0.0	0.0
TCCTTCTTTTT	0.0	0.0	0.0	0.0	0.0	0.0	0.0
TACGTAGGTG	0.0	0.0	0.0	0.0	0.0	0.0	0.0
TACAGAGGTC	0.0	0.0	0.0	0.0	0.0	0.0	0.0
TACGTAGGGA	0.0	0.0	0.0	0.0	0.0	0.0	0.0
TACGGAGGAT	0.0	0.0	0.0	0.0	0.0	0.0	0.0
TACGTAGGGG	0.0	0.0	0.0	0.0	0.0	0.0	0.0
TACGTAGGTG	0.0	0.0	0.0	0.0	0.0	0.0	0.0
TACGAAGGGG	0.0	0.0	0.0	0.0	0.0	0.0	0.0
TACGTAGGGA	0.0	0.0	0.0	0.0	0.0	0.0	0.0
TACGTAGGGG	0.0	0.0	0.0	0.0	0.0	0.0	0.0

TACGTATGGT	0.0	0.0	0.0	0.0	0.0	0.0	0.0
TACGTAGGGT	0.0	0.0	0.0	0.0	0.0	0.0	0.0
TACGTAGGG	0.0	0.0	0.0	0.0	0.0	0.0	0.0
TACGTAGGGA	0.0	0.0	0.0	0.0	0.0	0.0	0.0
TACGTAGGTG	0.0	0.0	0.0	0.0	0.0	0.0	0.0
TACGTATGGA	0.0	0.0	0.0	0.0	0.0	0.0	0.0
TACGTAGGG	0.0	0.0	0.0	0.0	0.0	0.0	0.0
TACGTAGGTG	0.0	0.0	0.0	0.0	0.0	0.0	0.0
TACGTAGGG	0.0	0.0	0.0	0.0	0.0	0.0	0.0
TACGTAGGGT	0.0	0.0	0.0	0.0	0.0	0.0	0.0
TACGTAGGTG	0.0	0.0	0.0	0.0	0.0	0.0	0.0
AACGTAGGGT	0.0	0.0	0.0	0.0	0.0	0.0	0.0
AACGTAGGT	0.0	0.0	0.0	0.0	0.0	0.0	0.0
TACGTAGGG	0.0	0.0	0.0	0.0	0.0	0.0	0.0
TACGTAGGTG	0.0	0.0	0.0	0.0	0.0	0.0	0.0
TACGTATGGT	0.0	0.0	0.0	0.0	0.0	0.0	0.0
TACGTATGGA	0.0	0.0	0.0	0.0	0.0	0.0	0.0
TACGTAGGG	0.0	0.0	0.0	0.0	0.0	0.0	0.0
TACGTATGGT	0.0	0.0	0.0	0.0	0.0	0.0	0.0
TACGTAGGG	0.0	0.0	0.0	0.0	0.0	0.0	0.0
TACGTAGGG	0.0	0.0	0.0	0.0	0.0	0.0	0.0
TACGTAGGG	0.0	0.0	0.0	0.0	0.0	0.0	0.0
TACGTAGGGT	0.0	0.0	0.0	0.0	0.0	0.0	0.0
TACGTAGGTG	0.0	0.0	0.0	0.0	0.0	0.0	0.0
TACGTAGGG	0.0	0.0	0.0	0.0	0.0	0.0	0.0
TACGTAGGG	0.0	0.0	0.0	0.0	0.0	0.0	0.0
TACGTAGGG	0.0	0.0	0.0	0.0	0.0	0.0	0.0
TACGGAAGGT	0.0	0.0	0.0	0.0	0.0	0.0	0.0
TACGTAGGG	0.0	0.0	0.0	0.0	0.0	0.0	0.0
TACGTAGGG	0.0	0.0	0.0	0.0	0.0	0.0	0.0
TACGTAGGG	0.0	0.0	0.0	0.0	0.0	0.0	0.0
AGCGGAGGAT	0.0	0.0	0.0	0.0	0.0	0.0	0.0
TACGTAAGGT	0.0	0.0	0.0	0.0	0.0	0.0	0.0
TACGTAGGTG	0.0	0.0	0.0	0.0	0.0	0.0	0.0
TACGTAGGTG	0.0	0.0	0.0	0.0	0.0	0.0	0.0
TACGTAGGG	0.0	0.0	0.0	0.0	0.0	0.0	0.0
TACGTATGGT	0.0	0.0	0.0	0.0	0.0	0.0	0.0
TACGTAGGTG	0.0	0.0	0.0	0.0	0.0	0.0	0.0
TACGTAGGGA	0.0	0.0	0.0	0.0	0.0	0.0	0.0
TACGTAGGTG	0.0	0.0	0.0	0.0	0.0	0.0	0.0
TACGTAGGG	0.0	0.0	0.0	0.0	0.0	0.0	0.0
TACGTAGGG	0.0	0.0	0.0	0.0	0.0	0.0	0.0
TACGGAGGAT	0.0	0.0	0.0	0.0	0.0	0.0	0.0
TACGTAGGTG	0.0	0.0	0.0	0.0	0.0	0.0	0.0
TACGTAGGG	0.0	0.0	0.0	0.0	0.0	0.0	0.0
TACGTAGGG	0.0	0.0	0.0	0.0	0.0	0.0	0.0
CACGTAGGG	0.0	0.0	0.0	0.0	0.0	0.0	0.0

TACGGAGGGT	0.0	0.0	0.0	0.0	0.0	0.0	0.0
TACGTAGGGT	0.0	0.0	0.0	0.0	0.0	0.0	0.0
TACGTAGGGG	0.0	0.0	0.0	0.0	0.0	0.0	0.0
TACGGAGGAT	0.0	0.0	0.0	0.0	0.0	0.0	0.0
TACGTATGTC	0.0	0.0	0.0	0.0	0.0	0.0	0.0
TACGGAGGAT	0.0	0.0	0.0	0.0	0.0	0.0	0.0
AACATAGGGG	0.0	0.0	0.0	0.0	0.0	0.0	0.0
TACGGGGGAT	0.0	0.0	0.0	0.0	0.0	0.0	0.0
TACGGAGGGT	0.0	0.0	0.0	0.0	0.0	0.0	0.0
TACGTAGGTG	0.0	0.0	0.0	0.0	0.0	0.0	0.0
TACGTAGGTG	0.0	0.0	0.0	0.0	0.0	0.0	0.0
TACGTAGGGG	0.0	0.0	0.0	0.0	0.0	0.0	0.0
TACGTAGGGG	0.0	0.0	0.0	0.0	0.0	0.0	0.0
TACGTATGGG	0.0	0.0	0.0	0.0	0.0	0.0	0.0
TACGTAGGTG	0.0	0.0	0.0	0.0	0.0	0.0	0.0
TACGTAGGGA	0.0	0.0	0.0	0.0	0.0	0.0	0.0
TACGTAGGTG	0.0	0.0	0.0	0.0	0.0	0.0	0.0
TACGTAGGGG	0.0	0.0	0.0	0.0	0.0	0.0	0.0
TACGTAGGGG	0.0	0.0	0.0	0.0	0.0	0.0	0.0
TACGTATGGT	0.0	0.0	0.0	0.0	0.0	0.0	0.0
TACGTAGGGG	0.0	0.0	0.0	0.0	0.0	0.0	0.0
TACGGAGGAT	0.0	0.0	0.0	0.0	0.0	0.0	0.0
TACGTAGGGG	0.0	0.0	0.0	0.0	0.0	0.0	0.0
TACGTAGGTG	0.0	0.0	0.0	0.0	0.0	0.0	0.0
TACGTATGGT	0.0	0.0	0.0	0.0	0.0	0.0	0.0
TACGTAGGGG	0.0	0.0	0.0	0.0	0.0	0.0	0.0
TACGTAGGGG	0.0	0.0	0.0	0.0	0.0	0.0	0.0
TACGTAGGGC	0.0	0.0	0.0	0.0	0.0	0.0	0.0
TACGTAGGGT	0.0	0.0	0.0	0.0	0.0	0.0	0.0
TACGTAGGTG	0.0	0.0	0.0	0.0	0.0	0.0	0.0
TACGTAGGTG	0.0	0.0	0.0	0.0	0.0	0.0	0.0
TACGTATGGA	0.0	0.0	0.0	0.0	0.0	0.0	0.0
TACGGAGGAT	0.0	0.0	0.0	0.0	0.0	0.0	0.0
TACGTAGGTG	0.0	0.0	0.0	0.0	0.0	0.0	0.0
TACGTAGGGG	0.0	0.0	0.0	0.0	0.0	0.0	0.0
TACGTAGGTG	0.0	0.0	0.0	0.0	0.0	0.0	0.0
TACGTAGGGT	0.0	0.0	0.0	0.0	0.0	0.0	0.0
TACGTAGGTG	0.0	0.0	0.0	0.0	0.0	0.0	0.0
AACGTAGGTC	0.0	0.0	0.0	0.0	0.0	0.0	0.0
TACGTAGGTG	0.0	0.0	0.0	0.0	0.0	0.0	0.0
TACGTAGGTC	0.0	0.0	0.0	0.0	0.0	0.0	0.0
TACGTAGGTG	0.0	0.0	0.0	0.0	0.0	0.0	0.0
TACGTAGGGG	0.0	0.0	0.0	0.0	0.0	0.0	0.0
TACGTAGGGG	0.0	0.0	0.0	0.0	0.0	0.0	0.0
TACGTAGGGT	0.0	0.0	0.0	0.0	0.0	0.0	0.0
TACGGAGGGT	0.0	0.0	0.0	0.0	0.0	0.0	0.0
TACGGAGGGT	0.0	0.0	0.0	0.0	0.0	0.0	0.0

TACGTATGGA	0.0	0.0	0.0	0.0	0.0	0.0	0.0
TACGGAGGGT	0.0	0.0	0.0	0.0	0.0	0.0	0.0
TACGTAGGTG	0.0	0.0	0.0	0.0	0.0	0.0	0.0
TACGTAGGGG	0.0	0.0	0.0	0.0	0.0	0.0	0.0
TACGGAGGGT	0.0	0.0	0.0	0.0	0.0	0.0	0.0
TACGTATGGA	0.0	0.0	0.0	0.0	0.0	0.0	0.0
TACGTAGGTG	0.0	0.0	0.0	0.0	0.0	0.0	0.0
TACGTATGGT	0.0	0.0	0.0	0.0	0.0	0.0	0.0
TACGTAGGTG	0.0	0.0	0.0	0.0	0.0	0.0	0.0
TACGTAGGGA	0.0	0.0	0.0	0.0	0.0	0.0	0.0
TACGTAGGGG	0.0	0.0	0.0	0.0	0.0	0.0	0.0
TACGTAGGTG	0.0	0.0	0.0	0.0	0.0	0.0	0.0
TACGTATGGA	0.0	0.0	0.0	0.0	0.0	0.0	0.0
TACGTAGGGA	0.0	0.0	0.0	0.0	0.0	0.0	0.0
TACGTAGGTG	0.0	0.0	0.0	0.0	0.0	0.0	0.0
TACGTAGGGG	0.0	0.0	0.0	0.0	0.0	0.0	0.0
TACGTAGGGG	0.0	0.0	0.0	0.0	0.0	0.0	0.0
TACGTAGGTG	0.0	0.0	0.0	0.0	0.0	0.0	0.0
TACGTAGGGG	0.0	0.0	0.0	0.0	0.0	0.0	0.0
TACGGAGGAT	0.0	0.0	0.0	0.0	0.0	0.0	0.0
TACGTAGGGA	0.0	0.0	0.0	0.0	0.0	0.0	0.0
TACGTATGGA	0.0	0.0	0.0	0.0	0.0	0.0	0.0
TACGTAGGGG	0.0	0.0	0.0	0.0	0.0	0.0	0.0
TACGTAGGGG	0.0	0.0	0.0	0.0	0.0	0.0	0.0
TACGTATGGA	0.0	0.0	0.0	0.0	0.0	0.0	0.0
TACGTAGGTG	0.0	0.0	0.0	0.0	0.0	0.0	0.0
TACGTAGGGA	0.0	0.0	0.0	0.0	0.0	0.0	0.0
TACGTAGGTG	0.0	0.0	0.0	0.0	0.0	0.0	0.0
TACGTAGGGG	0.0	0.0	0.0	0.0	0.0	0.0	0.0
TACGTAGGTG	0.0	0.0	0.0	0.0	0.0	0.0	0.0
TACGTAGGGG	0.0	0.0	0.0	0.0	0.0	0.0	0.0
TACGTAGGTG	0.0	0.0	0.0	0.0	0.0	0.0	0.0
TACGTAGGGG	0.0	0.0	0.0	0.0	0.0	0.0	0.0
TACGTATGGA	0.0	0.0	0.0	0.0	0.0	0.0	0.0
TACGTATGGT	0.0	0.0	0.0	0.0	0.0	0.0	0.0
TACGTATGGT	0.0	0.0	0.0	0.0	0.0	0.0	0.0
TACGTAGGGG	0.0	0.0	0.0	0.0	0.0	0.0	0.0
TACGTAGGTG	0.0	0.0	0.0	0.0	0.0	0.0	0.0
TACGGAGGAT	0.0	0.0	0.0	0.0	0.0	0.0	0.0
TACGTATGGT	0.0	0.0	0.0	0.0	0.0	0.0	0.0
TACGTAGGTG	0.0	0.0	0.0	0.0	0.0	0.0	0.0
TACGTAGGGG	0.0	0.0	0.0	0.0	0.0	0.0	0.0
TACGTAGGGG	0.0	0.0	0.0	0.0	0.0	0.0	0.0
TACGTAGGGA	0.0	0.0	0.0	0.0	0.0	0.0	0.0
TACGTAGGGG	0.0	0.0	0.0	0.0	0.0	0.0	0.0
TACGTAGGGA	0.0	0.0	0.0	0.0	0.0	0.0	0.0
TACGTAGGTG	0.0	0.0	0.0	0.0	0.0	0.0	0.0

TACGTAGGTG	0.0	0.0	0.0	0.0	0.0	0.0	0.0
TACGTAGGG	0.0	0.0	0.0	0.0	0.0	0.0	0.0
TACGGAGGAT	0.0	0.0	0.0	0.0	0.0	0.0	0.0
TACGGAGGGT	0.0	0.0	0.0	0.0	0.0	0.0	0.0
TACGTATGGT	0.0	0.0	0.0	0.0	0.0	0.0	0.0
TACGTAGGG	0.0	0.0	0.0	0.0	0.0	0.0	0.0
TACGTATGGT	0.0	0.0	0.0	0.0	0.0	0.0	0.0
TACGTATGGA	0.0	0.0	0.0	0.0	0.0	0.0	0.0
TACGGAGGA	0.0	0.0	0.0	0.0	0.0	0.0	0.0
TACGTAGGTA	0.0	0.0	0.0	0.0	0.0	0.0	0.0
TACGTAGGTG	0.0	0.0	0.0	0.0	0.0	0.0	0.0
TACGTATGGT	0.0	0.0	0.0	0.0	0.0	0.0	0.0
TACGTAGGGA	0.0	0.0	0.0	0.0	0.0	0.0	0.0
TACGTATGGT	0.0	0.0	0.0	0.0	0.0	0.0	0.0
TACGTAGGGA	0.0	0.0	0.0	0.0	0.0	0.0	0.0
AACGTGGGGT	0.0	0.0	0.0	0.0	0.0	0.0	0.0
TACGTAGGG	0.0	0.0	0.0	0.0	0.0	0.0	0.0
TACGTAGGG	0.0	0.0	0.0	0.0	0.0	0.0	0.0
TACGTAGGTG	0.0	0.0	0.0	0.0	0.0	0.0	0.0
TACGTAGGTG	0.0	0.0	0.0	0.0	0.0	0.0	0.0
TACGTAGGGA	0.0	0.0	0.0	0.0	0.0	0.0	0.0
TACGTATGGT	0.0	0.0	0.0	0.0	0.0	0.0	0.0
AACGTAGGTC	0.0	0.0	0.0	0.0	0.0	0.0	0.0
TACGTAGGG	0.0	0.0	0.0	0.0	0.0	0.0	0.0
TACGGAGGAT	0.0	0.0	0.0	0.0	0.0	0.0	0.0
TACGTATGGT	0.0	0.0	0.0	0.0	0.0	0.0	0.0
TACGTAGGG	0.0	0.0	0.0	0.0	0.0	0.0	0.0
TACGTATGGT	0.0	0.0	0.0	0.0	0.0	0.0	0.0
TACGTAGGG	0.0	0.0	0.0	0.0	0.0	0.0	0.0
TACGTAGGG	0.0	0.0	0.0	0.0	0.0	0.0	0.0
TACGTAGGG	0.0	0.0	0.0	0.0	0.0	0.0	0.0
TACGTAGGGA	0.0	0.0	0.0	0.0	0.0	0.0	0.0
AACGTAGGGT	0.0	0.0	0.0	0.0	0.0	0.0	0.0
TACGGAGGAT	0.0	0.0	0.0	0.0	0.0	0.0	0.0
TACGTATGGA	0.0	0.0	0.0	0.0	0.0	0.0	0.0
TACGTATGTC	0.0	0.0	0.0	0.0	0.0	0.0	0.0
TACGTAGGTG	0.0	0.0	0.0	0.0	0.0	0.0	0.0
AACGTAGGTC	0.0	0.0	0.0	0.0	0.0	0.0	0.0
AACGTAGGTC	0.0	0.0	0.0	0.0	0.0	0.0	0.0
TACGTATGGT	0.0	0.0	0.0	0.0	0.0	0.0	0.0
TACGGAGGGT	0.0	0.0	0.0	0.0	0.0	0.0	0.0
TACGTATGGA	0.0	0.0	0.0	0.0	0.0	0.0	0.0
TACGGAAGGT	0.0	0.0	0.0	0.0	0.0	0.0	0.0
TACGTATGGT	0.0	0.0	0.0	0.0	0.0	0.0	0.0
TACGTAGGG	0.0	0.0	0.0	0.0	0.0	0.0	0.0
TACGTAGGG	0.0	0.0	0.0	0.0	0.0	0.0	0.0
TACGTAGGG	0.0	0.0	0.0	0.0	0.0	0.0	0.0

TACGTAGGTG	0.0	0.0	0.0	0.0	0.0	0.0	0.0
AACGTAGGTC	0.0	0.0	0.0	0.0	0.0	0.0	0.0
TACGTAGGTG	0.0	0.0	0.0	0.0	0.0	0.0	0.0
TACGTATGGT	0.0	0.0	0.0	0.0	0.0	0.0	0.0
TACGTAGGGG	0.0	0.0	0.0	0.0	0.0	0.0	0.0
TACGTAGGTG	0.0	0.0	0.0	0.0	0.0	0.0	0.0
TACGTAGGTC	0.0	0.0	0.0	0.0	0.0	0.0	0.0
TACGTAGGGA	0.0	0.0	0.0	0.0	0.0	0.0	0.0
TACGTAGGGG	0.0	0.0	0.0	0.0	0.0	0.0	0.0
TACGTAGGGG	0.0	0.0	0.0	0.0	0.0	0.0	0.0
TACGGAGGAT	0.0	0.0	0.0	0.0	0.0	0.0	0.0
TACGTAGGGA	0.0	0.0	0.0	0.0	0.0	0.0	0.0
TACGGAGGAT	0.0	0.0	0.0	0.0	0.0	0.0	0.0
TACGTAGGGA	0.0	0.0	0.0	0.0	0.0	0.0	0.0
TACATAGGGG	0.0	0.0	0.0	0.0	0.0	0.0	0.0
TACGTAGGTG	0.0	0.0	0.0	0.0	0.0	0.0	0.0
AACGTAGGGT	0.0	0.0	0.0	0.0	0.0	0.0	0.0
TACGTAGGGA	0.0	0.0	0.0	0.0	0.0	0.0	0.0
TACGTAGGTG	0.0	0.0	0.0	0.0	0.0	0.0	0.0
TACAGAGGGT	0.0	0.0	0.0	0.0	0.0	0.0	0.0
TACGTAGGGG	0.0	0.0	0.0	0.0	0.0	0.0	0.0
TACGTAGGTG	0.0	0.0	0.0	0.0	0.0	0.0	0.0
TACGTAGGTG	0.0	0.0	0.0	0.0	0.0	0.0	0.0
TACGTAGGGG	0.0	0.0	0.0	0.0	0.0	0.0	0.0
TACGTATGGA	0.0	0.0	0.0	0.0	0.0	0.0	0.0
TACGTATGGA	0.0	0.0	0.0	0.0	0.0	0.0	0.0
TACGTAGGGG	0.0	0.0	0.0	0.0	0.0	0.0	0.0
TACGTATGGT	0.0	0.0	0.0	0.0	0.0	0.0	0.0
TACGTATGGT	0.0	0.0	0.0	0.0	0.0	0.0	0.0
TACGTAGGGG	0.0	0.0	0.0	0.0	0.0	0.0	0.0
TACGTAGGGT	0.0	0.0	0.0	0.0	0.0	0.0	0.0
TACGTAGGGG	0.0	0.0	0.0	0.0	0.0	0.0	0.0
TACGTAGGGT	0.0	0.0	0.0	0.0	0.0	0.0	0.0
TACGTATGGA	0.0	0.0	0.0	0.0	0.0	0.0	0.0
TACGGAGGTG	0.0	0.0	0.0	0.0	0.0	0.0	0.0
TACGTAGGGG	0.0	0.0	0.0	0.0	0.0	0.0	0.0
TACGTAGGGG	0.0	0.0	0.0	0.0	0.0	0.0	0.0
TACGTAGGGA	0.0	0.0	0.0	0.0	0.0	0.0	0.0
TACGTATGGA	0.0	0.0	0.0	0.0	0.0	0.0	0.0
TACGTAGGGG	0.0	0.0	0.0	0.0	0.0	0.0	0.0
TACGTAGGTG	0.0	0.0	0.0	0.0	0.0	0.0	0.0
TACGTAGGGG	0.0	0.0	0.0	0.0	0.0	0.0	0.0
TACGTAGGTG	0.0	0.0	0.0	0.0	0.0	0.0	0.0
TACGTAGGGT	0.0	0.0	0.0	0.0	0.0	0.0	0.0
TACGTATGGT	0.0	0.0	0.0	0.0	0.0	0.0	0.0
TACGGAGGGT	0.0	0.0	0.0	0.0	0.0	0.0	0.0
TACGTAGGGG	0.0	0.0	0.0	0.0	0.0	0.0	0.0

TACGGAGGAT	0.0	0.0	0.0	0.0	0.0	0.0	0.0
TACGTAGGGG	0.0	0.0	0.0	0.0	0.0	0.0	0.0
TACGTAGGGG	0.0	0.0	0.0	0.0	0.0	0.0	0.0
TACGTATGGT	0.0	0.0	0.0	0.0	0.0	0.0	0.0
TACGGAGGAT	0.0	0.0	0.0	0.0	0.0	0.0	0.0
TACGTATGGT	0.0	0.0	0.0	0.0	0.0	0.0	0.0
TACGTAGGGG	0.0	0.0	0.0	0.0	0.0	0.0	0.0
TACGTATGTC	0.0	0.0	0.0	0.0	0.0	0.0	0.0
TACGTAGGTG	0.0	0.0	0.0	0.0	0.0	0.0	0.0
TACGTATGGA	0.0	0.0	0.0	0.0	0.0	0.0	0.0
TACGTATGTC	0.0	0.0	0.0	0.0	0.0	0.0	0.0
TACGTAGGTG	0.0	0.0	0.0	0.0	0.0	0.0	0.0
TACGTAGGGG	0.0	0.0	0.0	0.0	0.0	0.0	0.0
TACGTAGGGG	0.0	0.0	0.0	0.0	0.0	0.0	0.0
TACGTATGTT	0.0	0.0	0.0	0.0	0.0	0.0	0.0
TACGGAGGAT	0.0	0.0	0.0	0.0	0.0	0.0	0.0
TACGGAGGAT	0.0	0.0	0.0	0.0	0.0	0.0	0.0
TACGTAGGGG	0.0	0.0	0.0	0.0	0.0	0.0	0.0
TACGTAGGTG	0.0	0.0	0.0	0.0	0.0	0.0	0.0
TACGGAGGGT	0.0	0.0	0.0	0.0	0.0	0.0	0.0
TACGTAGGGG	0.0	0.0	0.0	0.0	0.0	0.0	0.0
TACGTAGGGA	0.0	0.0	0.0	0.0	0.0	0.0	0.0
TACGGAGGAT	0.0	0.0	0.0	0.0	0.0	0.0	0.0
TACGTAGGTG	0.0	0.0	0.0	0.0	0.0	0.0	0.0
TACGTAGGTG	0.0	0.0	0.0	0.0	0.0	0.0	0.0
TACGTAGGTG	0.0	0.0	0.0	0.0	0.0	0.0	0.0
TACGTATGGT	0.0	0.0	0.0	0.0	0.0	0.0	0.0
TACGTATGGT	0.0	0.0	0.0	0.0	0.0	0.0	0.0
TACGTAGGTG	0.0	0.0	0.0	0.0	0.0	0.0	0.0
TACGTAGGGG	0.0	0.0	0.0	0.0	0.0	0.0	0.0
TACGTAGGTG	0.0	0.0	0.0	0.0	0.0	0.0	0.0
TACGTAGGGG	0.0	0.0	0.0	0.0	0.0	0.0	0.0
TACGTATGGT	0.0	0.0	0.0	0.0	0.0	0.0	0.0
TACGTAGGTG	0.0	0.0	0.0	0.0	0.0	0.0	0.0
AACGTAGGTC	0.0	0.0	0.0	0.0	0.0	0.0	0.0
TACGTATGGT	0.0	0.0	0.0	0.0	0.0	0.0	0.0
TACGTATGGT	0.0	0.0	0.0	0.0	0.0	0.0	0.0
TACGGAGGAT	0.0	0.0	0.0	0.0	0.0	0.0	0.0
TACGTATGGT	0.0	0.0	0.0	0.0	0.0	0.0	0.0
TACGTATGGT	0.0	0.0	0.0	0.0	0.0	0.0	0.0
TACGTATGGA	0.0	0.0	0.0	0.0	0.0	0.0	0.0
TGCGTAGGTG	0.0	0.0	0.0	0.0	0.0	0.0	0.0
TACGGAGGAT	0.0	0.0	0.0	0.0	0.0	0.0	0.0
TACGTAGGGG	0.0	0.0	0.0	0.0	0.0	0.0	0.0
TACGTAGGTG	0.0	0.0	0.0	0.0	0.0	0.0	0.0
TACGTAGGGG	0.0	0.0	0.0	0.0	0.0	0.0	0.0
TATGTAGGGG	0.0	0.0	0.0	0.0	0.0	0.0	0.0

TACGTATGGA	0.0	0.0	0.0	0.0	0.0	0.0	0.0
TACGTAGGGC	0.0	0.0	0.0	0.0	0.0	0.0	0.0
TACGTAGGGT	0.0	0.0	0.0	0.0	0.0	0.0	0.0
TACGGAGGAT	0.0	0.0	0.0	0.0	0.0	0.0	0.0
TACGTAGGGT	0.0	0.0	0.0	0.0	0.0	0.0	0.0
TACGTAGGTG	0.0	0.0	0.0	0.0	0.0	0.0	0.0
TACGTATGGA	0.0	0.0	0.0	0.0	0.0	0.0	0.0
TACGTAGGGG	0.0	0.0	0.0	0.0	0.0	0.0	0.0
TACGTAGGGG	0.0	0.0	0.0	0.0	0.0	0.0	0.0
TACGTAGGGG	0.0	0.0	0.0	0.0	0.0	0.0	0.0
TACGTATGGT	0.0	0.0	0.0	0.0	0.0	0.0	0.0
TACGTATGGT	0.0	0.0	0.0	0.0	0.0	0.0	0.0
TACGGAGGAT	0.0	0.0	0.0	0.0	0.0	0.0	0.0
TACATAGGGG	0.0	0.0	0.0	0.0	0.0	0.0	0.0
TACGTAGGGG	0.0	0.0	0.0	0.0	0.0	0.0	0.0
TACGTAGGGG	0.0	0.0	0.0	0.0	0.0	0.0	0.0
AACGTAGGTC	0.0	0.0	0.0	0.0	0.0	0.0	0.0
TACGTAGGGG	0.0	0.0	0.0	0.0	0.0	0.0	0.0
TACGTAGGGT	0.0	0.0	0.0	0.0	0.0	0.0	0.0
TACGTAGGGG	0.0	0.0	0.0	0.0	0.0	0.0	0.0
TACGTAGGGG	0.0	0.0	0.0	0.0	0.0	0.0	0.0
TACGTAGGTG	0.0	0.0	0.0	0.0	0.0	0.0	0.0
TACGTAGGGG	0.0	0.0	0.0	0.0	0.0	0.0	0.0
TACGTAGGGA	0.0	0.0	0.0	0.0	0.0	0.0	0.0
TACGTAGGGG	0.0	0.0	0.0	0.0	0.0	0.0	0.0
TACGAAGGGC	0.0	0.0	0.0	0.0	0.0	0.0	0.0
TACGTAGGGA	0.0	0.0	0.0	0.0	0.0	0.0	0.0
TACGTAGGTG	0.0	0.0	0.0	0.0	0.0	0.0	0.0
TACGTAGGTG	0.0	0.0	0.0	0.0	0.0	0.0	0.0
TACGTAGGGG	0.0	0.0	0.0	0.0	0.0	0.0	0.0
TACGTAGGGA	0.0	0.0	0.0	0.0	0.0	0.0	0.0
TACGTAGGTG	0.0	0.0	0.0	0.0	0.0	0.0	0.0
TACGTAGGGG	0.0	0.0	0.0	0.0	0.0	0.0	0.0
TACGTAGGGG	0.0	0.0	0.0	0.0	0.0	0.0	0.0
TACGTAGGTG	0.0	0.0	0.0	0.0	0.0	0.0	0.0
TACGTAGGTG	0.0	0.0	0.0	0.0	0.0	0.0	0.0
TACGTAGGTG	0.0	0.0	0.0	0.0	0.0	0.0	0.0
TACGTAGGTG	0.0	0.0	0.0	0.0	0.0	0.0	0.0
TACGTAGGGG	0.0	0.0	0.0	0.0	0.0	0.0	0.0
TACGTAGGTG	0.0	0.0	0.0	0.0	0.0	0.0	0.0
TACGTAGGGA	0.0	0.0	0.0	0.0	0.0	0.0	0.0
TACGTAGGTG	0.0	0.0	0.0	0.0	0.0	0.0	0.0
TACGTAGGGG	0.0	0.0	0.0	0.0	0.0	0.0	0.0
TACGTAGGGA	0.0	0.0	0.0	0.0	0.0	0.0	0.0
TACGTAGGGG	0.0	0.0	0.0	0.0	0.0	0.0	0.0
TACGGAGGAT	0.0	0.0	0.0	0.0	0.0	0.0	0.0
TACGTATGGA	0.0	0.0	0.0	0.0	0.0	0.0	0.0

TACGTATGGA	0.0	0.0	0.0	0.0	0.0	0.0	0.0
TACGTAGGGG	0.0	0.0	0.0	0.0	0.0	0.0	0.0
TACGGAGGAT	0.0	0.0	0.0	0.0	0.0	0.0	0.0
TACGTAGGGA	0.0	0.0	0.0	0.0	0.0	0.0	0.0
TACGTAGGTG	0.0	0.0	0.0	0.0	0.0	0.0	0.0
TACGTAGGGG	0.0	0.0	0.0	0.0	0.0	0.0	0.0
TACGTAGGTG	0.0	0.0	0.0	0.0	0.0	0.0	0.0
TACGTAGGGG	0.0	0.0	0.0	0.0	0.0	0.0	0.0
TACGTAGGGG	0.0	0.0	0.0	0.0	0.0	0.0	0.0
TACGTAGGTG	0.0	0.0	0.0	0.0	0.0	0.0	0.0
TACGTAGGGA	0.0	0.0	0.0	0.0	0.0	0.0	0.0
TACGTAGGTC	0.0	0.0	0.0	0.0	0.0	0.0	0.0
TACGTATGGT	0.0	0.0	0.0	0.0	0.0	0.0	0.0
AACGTAGGTG	0.0	0.0	0.0	0.0	0.0	0.0	0.0
TACGTATGGA	0.0	0.0	0.0	0.0	0.0	0.0	0.0
CACGTAGGTG	0.0	0.0	0.0	0.0	0.0	0.0	0.0
TACGTAGGGG	0.0	0.0	0.0	0.0	0.0	0.0	0.0
TACGTAGGTG	0.0	0.0	0.0	0.0	0.0	0.0	0.0
TACGTATGGA	0.0	0.0	0.0	0.0	0.0	0.0	0.0
TACGTAGGGG	0.0	0.0	0.0	0.0	0.0	0.0	0.0
TACGTAGGGG	0.0	0.0	0.0	0.0	0.0	0.0	0.0
TACGTAGGGA	0.0	0.0	0.0	0.0	0.0	0.0	0.0
TACGTAGGGG	0.0	0.0	0.0	0.0	0.0	0.0	0.0
TACGTAGGGG	0.0	0.0	0.0	0.0	0.0	0.0	0.0
TACGGAGGGT	0.0	0.0	0.0	0.0	0.0	0.0	0.0
TACGGAGGGT	0.0	0.0	0.0	0.0	0.0	0.0	0.0
TACGTAGGTG	0.0	0.0	0.0	0.0	0.0	0.0	0.0
TACGTAGGTC	0.0	0.0	0.0	0.0	0.0	0.0	0.0
TACGTAGGGG	0.0	0.0	0.0	0.0	0.0	0.0	0.0
TACGTAGGGG	0.0	0.0	0.0	0.0	0.0	0.0	0.0
TCCTTCTTTT	0.0	0.0	0.0	0.0	0.0	0.0	0.0
TACGGAGGGT	0.0	0.0	0.0	0.0	0.0	0.0	0.0
TACGTAGGTG	0.0	0.0	0.0	0.0	0.0	0.0	0.0
TACGGAGGGT	0.0	0.0	0.0	0.0	0.0	0.0	0.0
TACGTAGGTG	0.0	0.0	0.0	0.0	0.0	0.0	0.0
TACGTAGGTG	0.0	0.0	0.0	0.0	0.0	0.0	0.0
TACGTAGGTG	0.0	0.0	0.0	0.0	0.0	0.0	0.0
TACGTAGGTG	0.0	0.0	0.0	0.0	0.0	0.0	0.0
TACGTAGGGG	0.0	0.0	0.0	0.0	0.0	0.0	0.0
TACGGAAGAT	0.0	0.0	0.0	0.0	0.0	0.0	0.0
TACGGAGGAT	0.0	0.0	0.0	0.0	0.0	0.0	0.0
TACGTATGGT	0.0	0.0	0.0	0.0	0.0	0.0	0.0
TACGTATGGT	0.0	0.0	0.0	0.0	0.0	0.0	0.0
TACGTATGGT	0.0	0.0	0.0	0.0	0.0	0.0	0.0
TACGTAGGTG	0.0	0.0	0.0	0.0	0.0	0.0	0.0
TACGGAGGG	0.0	0.0	0.0	0.0	0.0	0.0	0.0
TACGTAGGTG	0.0	0.0	0.0	0.0	0.0	0.0	0.0

TACGTAGGGT	0.0	0.0	0.0	0.0	0.0	0.0	0.0
TACGTAGGGG	0.0	0.0	0.0	0.0	0.0	0.0	0.0
TACGTAGGGG	0.0	0.0	0.0	0.0	0.0	0.0	0.0
TACGTAGGTG	0.0	0.0	0.0	0.0	0.0	0.0	0.0
TACGTAGGGG	0.0	0.0	0.0	0.0	0.0	0.0	0.0
TACGTAGGGG	0.0	0.0	0.0	0.0	0.0	0.0	0.0
TACGTAGGGG	0.0	0.0	0.0	0.0	0.0	0.0	0.0
TACGTAGGGA	0.0	0.0	0.0	0.0	0.0	0.0	0.0
TACGTATGGA	0.0	0.0	0.0	0.0	0.0	0.0	0.0
AACGTAGGTC	0.0	0.0	0.0	0.0	0.0	0.0	0.0
TACGTAGGTG	0.0	0.0	0.0	0.0	0.0	0.0	0.0
TACGTAGGTG	0.0	0.0	0.0	0.0	0.0	0.0	0.0
TACGTAGGGG	0.0	0.0	0.0	0.0	0.0	0.0	0.0
TACGTAGGGA	0.0	0.0	0.0	0.0	0.0	0.0	0.0
TACGGAGGAT	0.0	0.0	0.0	0.0	0.0	0.0	0.0
TACGTAGGGG	0.0	0.0	0.0	0.0	0.0	0.0	0.0
AACGTGGGGT	0.0	0.0	0.0	0.0	0.0	0.0	0.0
AACGTAGGGT	0.0	0.0	0.0	0.0	0.0	0.0	0.0
TACGTAGGGG	0.0	0.0	0.0	0.0	0.0	0.0	0.0
TACGTAGGGA	0.0	0.0	0.0	0.0	0.0	0.0	0.0
TACGTAGGGG	0.0	0.0	0.0	0.0	0.0	0.0	0.0
TACGTATGGT	0.0	0.0	0.0	0.0	0.0	0.0	0.0
TACGGAGGGT	0.0	0.0	0.0	0.0	0.0	0.0	0.0
TACGTATGGT	0.0	0.0	0.0	0.0	0.0	0.0	0.0
TACGTAGGGG	0.0	0.0	0.0	0.0	0.0	0.0	0.0
TACGTATGGT	0.0	0.0	0.0	0.0	0.0	0.0	0.0
TACGTATGGT	0.0	0.0	0.0	0.0	0.0	0.0	0.0
TACGTAGGGA	0.0	0.0	0.0	0.0	0.0	0.0	0.0
TACGTAGGTG	0.0	0.0	0.0	0.0	0.0	0.0	0.0
TACGTAGGTG	0.0	0.0	0.0	0.0	0.0	0.0	0.0
TACGTAGGGA	0.0	0.0	0.0	0.0	0.0	0.0	0.0
TACGTAGGGA	0.0	0.0	0.0	0.0	0.0	0.0	0.0
TACGTAGGGA	0.0	0.0	0.0	0.0	0.0	0.0	0.0
TACGTAGGGA	0.0	0.0	0.0	0.0	0.0	0.0	0.0
TACGTAGGGT	0.0	0.0	0.0	0.0	0.0	0.0	0.0
AACGTAGGGT	0.0	0.0	0.0	0.0	0.0	0.0	0.0
TACGTAGGGG	0.0	0.0	0.0	0.0	0.0	0.0	0.0
AACGTAGGTG	0.0	0.0	0.0	0.0	0.0	0.0	0.0
TACGTAGGTG	0.0	0.0	0.0	0.0	0.0	0.0	0.0
TACAGAGGTC	0.0	0.0	0.0	0.0	0.0	0.0	0.0
TACGTAGGGA	0.0	0.0	0.0	0.0	0.0	0.0	0.0
TACGTAGGTG	0.0	0.0	0.0	0.0	0.0	0.0	0.0
TACAGAGGTC	0.0	0.0	0.0	0.0	0.0	0.0	0.0
TACGTAGGGA	0.0	0.0	0.0	0.0	0.0	0.0	0.0
TACGTAGGGG	0.0	0.0	0.0	0.0	0.0	0.0	0.0
AACGTAGGTG	0.0	0.0	0.0	0.0	0.0	0.0	0.0
TACGTAGGGA	0.0	0.0	0.0	0.0	0.0	0.0	0.0

TACGTATGGT	0.0	0.0	0.0	0.0	0.0	0.0	0.0
TACGTAGGGA	0.0	0.0	0.0	0.0	0.0	0.0	0.0
TACGTAGGGG	0.0	0.0	0.0	0.0	0.0	0.0	0.0
TACGTAGGGG	0.0	0.0	0.0	0.0	0.0	0.0	0.0
TACGTAGGGA	0.0	0.0	0.0	0.0	0.0	0.0	0.0
TACGGAGGAT	0.0	0.0	0.0	0.0	0.0	0.0	0.0
TACGTAGGGA	0.0	0.0	0.0	0.0	0.0	0.0	0.0
AACGTAGGGT	0.0	0.0	0.0	0.0	0.0	0.0	0.0
TACGTAGGGG	0.0	0.0	0.0	0.0	0.0	0.0	0.0
TACGTAGGGG	0.0	0.0	0.0	0.0	0.0	0.0	0.0
TACGTAGGGG	0.0	0.0	0.0	0.0	0.0	0.0	0.0
AACGTAGGGT	0.0	0.0	0.0	0.0	0.0	0.0	0.0
TACGTAGGGA	0.0	0.0	0.0	0.0	0.0	0.0	0.0
TACGTAGGGG	0.0	0.0	0.0	0.0	0.0	0.0	0.0
TACGTAGGTG	0.0	0.0	0.0	0.0	0.0	0.0	0.0
AACGTAGGGT	0.0	0.0	0.0	0.0	0.0	0.0	0.0
TACGGAGGAT	0.0	0.0	0.0	0.0	0.0	0.0	0.0
TACGTAGGGG	0.0	0.0	0.0	0.0	0.0	0.0	0.0
TACGTATGGT	0.0	0.0	0.0	0.0	0.0	0.0	0.0
TACGTATGGA	0.0	0.0	0.0	0.0	0.0	0.0	0.0
TACGTAGGGG	0.0	0.0	0.0	0.0	0.0	0.0	0.0
TACGTAGGGG	0.0	0.0	0.0	0.0	0.0	0.0	0.0
TACGTATGGT	0.0	0.0	0.0	0.0	0.0	0.0	0.0
TACGTAGGGG	0.0	0.0	0.0	0.0	0.0	0.0	0.0
AACGTAGGGT	0.0	0.0	0.0	0.0	0.0	0.0	0.0
TACGTAGGTG	0.0	0.0	0.0	0.0	0.0	0.0	0.0
TACGTAGGTG	0.0	0.0	0.0	0.0	0.0	0.0	0.0
TACGTAGGGG	0.0	0.0	0.0	0.0	0.0	0.0	0.0
TACGTATGGA	0.0	0.0	0.0	0.0	0.0	0.0	0.0
TACGTAGGTG	0.0	0.0	0.0	0.0	0.0	0.0	0.0
TACGTATGGT	0.0	0.0	0.0	0.0	0.0	0.0	0.0
TACGGAAGGT	0.0	0.0	0.0	0.0	0.0	0.0	0.0
TACGGAAGGT	0.0	0.0	0.0	0.0	0.0	0.0	0.0
TACGTAGGGG	0.0	0.0	0.0	0.0	0.0	0.0	0.0
TACGTAGGTA	0.0	0.0	0.0	0.0	0.0	0.0	0.0
TACGTAGGTG	0.0	0.0	0.0	0.0	0.0	0.0	0.0
TACGTATGGT	0.0	0.0	0.0	0.0	0.0	0.0	0.0
AACGTAGGGT	0.0	0.0	0.0	0.0	0.0	0.0	0.0
TACGTAGGGG	0.0	0.0	0.0	0.0	0.0	0.0	0.0
TACGTAGGGA	0.0	0.0	0.0	0.0	0.0	0.0	0.0
TACGGGGGAT	0.0	0.0	0.0	0.0	0.0	0.0	0.0
TACGTAGGGA	0.0	0.0	0.0	0.0	0.0	0.0	0.0
TACGTATGGT	0.0	0.0	0.0	0.0	0.0	0.0	0.0
TACGTAGGGA	0.0	0.0	0.0	0.0	0.0	0.0	0.0
TACGGAAGGT	0.0	0.0	0.0	0.0	0.0	0.0	0.0
TACGTAGGTG	0.0	0.0	0.0	0.0	0.0	0.0	0.0
TACGTAGGGG	0.0	0.0	0.0	0.0	0.0	0.0	0.0

TACGTAGGGG	0.0	0.0	0.0	0.0	0.0	0.0	0.0
TACGTATGGT	0.0	0.0	0.0	0.0	0.0	0.0	0.0
AACGTAGGGT	0.0	0.0	0.0	0.0	0.0	0.0	0.0
TACGTAGGGA	0.0	0.0	0.0	0.0	0.0	0.0	0.0
TACGTAGGTG	0.0	0.0	0.0	0.0	0.0	0.0	0.0
TACGTATGGT	0.0	0.0	0.0	0.0	0.0	0.0	0.0
TACGGAGGAT	0.0	0.0	0.0	0.0	0.0	0.0	0.0
TACGTAGGTG	0.0	0.0	0.0	0.0	0.0	0.0	0.0
TACGTAGGGA	0.0	0.0	0.0	0.0	0.0	0.0	0.0
AACGTAGGGT	0.0	0.0	0.0	0.0	0.0	0.0	0.0
AACGTAGGGT	0.0	0.0	0.0	0.0	0.0	0.0	0.0
TACGTAGGTG	0.0	0.0	0.0	0.0	0.0	0.0	0.0
TACGTAGGGA	0.0	0.0	0.0	0.0	0.0	0.0	0.0
TACGGAGGAT	0.0	0.0	0.0	0.0	0.0	0.0	0.0
TACGTATGGG	0.0	0.0	0.0	0.0	0.0	0.0	0.0
TACGTAGGGG	0.0	0.0	0.0	0.0	0.0	0.0	0.0
AACGTAGGGT	0.0	0.0	0.0	0.0	0.0	0.0	0.0
TACGTAGGGA	0.0	0.0	0.0	0.0	0.0	0.0	0.0
TACGTAGGGA	0.0	0.0	0.0	0.0	0.0	0.0	0.0
TACGGAGGAT	0.0	0.0	0.0	0.0	0.0	0.0	0.0
TACGTAGGTG	0.0	0.0	0.0	0.0	0.0	0.0	0.0
TACGTATGGA	0.0	0.0	0.0	0.0	0.0	0.0	0.0
TACGGAAGGT	0.0	0.0	0.0	0.0	0.0	0.0	0.0
TACGGAAGGT	0.0	0.0	0.0	0.0	0.0	0.0	0.0
TACGTAGGGA	0.0	0.0	0.0	0.0	0.0	0.0	0.0
TACGTATGGT	0.0	0.0	0.0	0.0	0.0	0.0	0.0
TACGAAGGGT	0.0	0.0	0.0	0.0	0.0	0.0	0.0
TACGTAGGGG	0.0	0.0	0.0	0.0	0.0	0.0	0.0
TACATAGGGG	0.0	0.0	0.0	0.0	0.0	0.0	0.0
TACGGAGGAT	0.0	0.0	0.0	0.0	0.0	0.0	0.0
AACGTAGGTC	0.0	0.0	0.0	0.0	0.0	0.0	0.0
TACGTATGGT	0.0	0.0	0.0	0.0	0.0	0.0	0.0
AACGTAGGTC	0.0	0.0	0.0	0.0	0.0	0.0	0.0
TACGTAGGAT	0.0	0.0	0.0	0.0	0.0	0.0	0.0
TACGTAGGGG	0.0	0.0	0.0	0.0	0.0	0.0	0.0
TACGTATGGT	0.0	0.0	0.0	0.0	0.0	0.0	0.0
TACGTAGGGG	0.0	0.0	0.0	0.0	0.0	0.0	0.0
TACGTAGGTG	0.0	0.0	0.0	0.0	0.0	0.0	0.0
TACGTAGGGG	0.0	0.0	0.0	0.0	0.0	0.0	0.0
TACGTAGGGA	0.0	0.0	0.0	0.0	0.0	0.0	0.0
TACGTAGGGG	0.0	0.0	0.0	0.0	0.0	0.0	0.0
TACGTAGGGA	0.0	0.0	0.0	0.0	0.0	0.0	0.0
TACGTAGGGA	0.0	0.0	0.0	0.0	0.0	0.0	0.0
TACGTATGGT	0.0	0.0	0.0	0.0	0.0	0.0	0.0
TACGTAGGGG	0.0	0.0	0.0	0.0	0.0	0.0	0.0
TACGTAGGGT	0.0	0.0	0.0	0.0	0.0	0.0	0.0
TACGGAAGAT	0.0	0.0	0.0	0.0	0.0	0.0	0.0

TACATAGGTG	0.0	0.0	0.0	0.0	0.0	0.0	0.0
TACGTAGGGG	0.0	0.0	0.0	0.0	0.0	0.0	0.0
TACGTAGGGA	0.0	0.0	0.0	0.0	0.0	0.0	0.0
TACGGAAGAT	0.0	0.0	0.0	0.0	0.0	0.0	0.0
TACGTAGGTG	0.0	0.0	0.0	0.0	0.0	0.0	0.0
TACGTAGGTG	0.0	0.0	0.0	0.0	0.0	0.0	0.0
TACGTAGGTG	0.0	0.0	0.0	0.0	0.0	0.0	0.0
TACGTAGGGA	0.0	0.0	0.0	0.0	0.0	0.0	0.0
TACGTAGGTG	0.0	0.0	0.0	0.0	0.0	0.0	0.0
TACGTAGGTG	0.0	0.0	0.0	0.0	0.0	0.0	0.0
TACGTAGGGG	0.0	0.0	0.0	0.0	0.0	0.0	0.0
TACGGAGGAT	0.0	0.0	0.0	0.0	0.0	0.0	0.0
TACGTATGGA	0.0	0.0	0.0	0.0	0.0	0.0	0.0
TACGTATGGA	0.0	0.0	0.0	0.0	0.0	0.0	0.0
TACGTAGGTG	0.0	0.0	0.0	0.0	0.0	0.0	0.0
TACGTAGGTG	0.0	0.0	0.0	0.0	0.0	0.0	0.0
TACGTAGGTG	0.0	0.0	0.0	0.0	0.0	0.0	0.0
TACGTAGGGA	0.0	0.0	0.0	0.0	0.0	0.0	0.0
TACGTAGGGG	0.0	0.0	0.0	0.0	0.0	0.0	0.0
TACGTAGGGG	0.0	0.0	0.0	0.0	0.0	0.0	0.0
GACGTAGGGC	0.0	0.0	0.0	0.0	0.0	0.0	0.0
AACATAGGGG	0.0	0.0	0.0	0.0	0.0	0.0	0.0
TACGTAGGGG	0.0	0.0	0.0	0.0	0.0	0.0	0.0
AACGTAGGGC	0.0	0.0	0.0	0.0	0.0	0.0	0.0
TACGTAGGTG	0.0	0.0	0.0	0.0	0.0	0.0	0.0
TACGTAGGTG	0.0	0.0	0.0	0.0	0.0	0.0	0.0
TACGGAGGAT	0.0	0.0	0.0	0.0	0.0	0.0	0.0
TACGTATGGT	0.0	0.0	0.0	0.0	0.0	0.0	0.0
TACGTAGGGG	0.0	0.0	0.0	0.0	0.0	0.0	0.0
TACGTAGGGA	0.0	0.0	0.0	0.0	0.0	0.0	0.0
TACGTAGGGT	0.0	0.0	0.0	0.0	0.0	0.0	0.0
TACGTATGGT	0.0	0.0	0.0	0.0	0.0	0.0	0.0
TACGTAGGTG	0.0	0.0	0.0	0.0	0.0	0.0	0.0
TACGTAGGTG	0.0	0.0	0.0	0.0	0.0	0.0	0.0
TACGTAGGGA	0.0	0.0	0.0	0.0	0.0	0.0	0.0
TACGTAGGGA	0.0	0.0	0.0	0.0	0.0	0.0	0.0
TACGTAGGTG	0.0	0.0	0.0	0.0	0.0	0.0	0.0
TACGTAGGGT	0.0	0.0	0.0	0.0	0.0	0.0	0.0
TACGTAGGGA	0.0	0.0	0.0	0.0	0.0	0.0	0.0
TACGTAGGTG	0.0	0.0	0.0	0.0	0.0	0.0	0.0
AACGTAGGTC	0.0	0.0	0.0	0.0	0.0	0.0	0.0
TACGTAGGGG	0.0	0.0	0.0	0.0	0.0	0.0	0.0
TACGTAGGGA	0.0	0.0	0.0	0.0	0.0	0.0	0.0
TACGTAGGGG	0.0	0.0	0.0	0.0	0.0	0.0	0.0
TACGTAGGAT	0.0	0.0	0.0	0.0	0.0	0.0	0.0
GACGTAGGTG	0.0	0.0	0.0	0.0	0.0	0.0	0.0
TACGTAGGGG	0.0	0.0	0.0	0.0	0.0	0.0	0.0

TACGTAGGGC	0.0	0.0	0.0	0.0	0.0	0.0	0.0
TACGTAGGGC	0.0	0.0	0.0	0.0	0.0	0.0	0.0
TACGTAGGGA	0.0	0.0	0.0	0.0	0.0	0.0	0.0
TACGTAGGGC	0.0	0.0	0.0	0.0	0.0	0.0	0.0
TACGTAGGGA	0.0	0.0	0.0	0.0	0.0	0.0	0.0
TACGTAGGGC	0.0	0.0	0.0	0.0	0.0	0.0	0.0
TACGTAGGGC	0.0	0.0	0.0	0.0	0.0	0.0	0.0
TACGTAGGGC	0.0	0.0	0.0	0.0	0.0	0.0	0.0
TACGTAGGTG	0.0	0.0	0.0	0.0	0.0	0.0	0.0
AACGTAGGGT	0.0	0.0	0.0	0.0	0.0	0.0	0.0
TACGTAGGGC	0.0	0.0	0.0	0.0	0.0	0.0	0.0
TACGTAGGGA	0.0	0.0	0.0	0.0	0.0	0.0	0.0
TACGTAGGTC	0.0	0.0	0.0	0.0	0.0	0.0	0.0
TACGTAGGGC	0.0	0.0	0.0	0.0	0.0	0.0	0.0
TACGTAGGTC	0.0	0.0	0.0	0.0	0.0	0.0	0.0
TACGTAGGGC	0.0	0.0	0.0	0.0	0.0	0.0	0.0
TACGTAGGTT	0.0	0.0	0.0	0.0	0.0	0.0	0.0
TACGTAGGGA	0.0	0.0	0.0	0.0	0.0	0.0	0.0
TACGTAGGGC	0.0	0.0	0.0	0.0	0.0	0.0	0.0
TACGTAGGGC	0.0	0.0	0.0	0.0	0.0	0.0	0.0
TACGTAGGGC	0.0	0.0	0.0	0.0	0.0	0.0	0.0
TACGTAGGGT	0.0	0.0	0.0	0.0	0.0	0.0	0.0
TACGTAGGGC	0.0	0.0	0.0	0.0	0.0	0.0	0.0
TACGTAGGGA	0.0	0.0	0.0	0.0	0.0	0.0	0.0
TACGTATGGA	0.0	0.0	0.0	0.0	0.0	0.0	0.0
TACGGAGGGT	0.0	0.0	0.0	0.0	0.0	0.0	0.0
TACGGAGGAT	0.0	0.0	0.0	0.0	0.0	0.0	0.0
TACGGAGGAT	0.0	0.0	0.0	0.0	0.0	0.0	0.0
TACGTATGGA	0.0	0.0	0.0	0.0	0.0	0.0	0.0
TACGTAGGTG	0.0	0.0	0.0	0.0	0.0	0.0	0.0
TACGTAGGGC	0.0	0.0	0.0	0.0	0.0	0.0	0.0
TACGTAGGGT	0.0	0.0	0.0	0.0	0.0	0.0	0.0
TACGTAGGGT	0.0	0.0	0.0	0.0	0.0	0.0	0.0
TACGTAGGTG	0.0	0.0	0.0	0.0	0.0	0.0	0.0
TACGTAGGGC	0.0	0.0	0.0	0.0	0.0	0.0	0.0
TACGTAGGGC	0.0	0.0	0.0	0.0	0.0	0.0	0.0
TACGTAGGGC	0.0	0.0	0.0	0.0	0.0	0.0	0.0
TACGTAGGGA	0.0	0.0	0.0	0.0	0.0	0.0	0.0
TACGTAGGTG	0.0	0.0	0.0	0.0	0.0	0.0	0.0
TACGTAGGGC	0.0	0.0	0.0	0.0	0.0	0.0	0.0
TACGTAGGGC	0.0	0.0	0.0	0.0	0.0	0.0	0.0
TACGGAGGAT	0.0	0.0	0.0	0.0	0.0	0.0	0.0
AACGTAGGTC	0.0	0.0	0.0	0.0	0.0	0.0	0.0
TACGGAGGAT	0.0	0.0	0.0	0.0	0.0	0.0	0.0
TACGTATGGT	0.0	0.0	0.0	0.0	0.0	0.0	0.0
TACGGAGGAT	0.0	0.0	0.0	0.0	0.0	0.0	0.0
TACGGAGGGT	0.0	0.0	0.0	0.0	0.0	0.0	0.0

TACGTATAGT	0.0	0.0	0.0	0.0	0.0	0.0	0.0
TACGTAGGTG	0.0	0.0	0.0	0.0	0.0	0.0	0.0
TACGTATGGT	0.0	0.0	0.0	0.0	0.0	0.0	0.0
TACGTAGGTG	0.0	0.0	0.0	0.0	0.0	0.0	0.0
TACGTATGGA	0.0	0.0	0.0	0.0	0.0	0.0	0.0
TACGTAGGG	0.0	0.0	0.0	0.0	0.0	0.0	0.0
TACGTAGGGT	0.0	0.0	0.0	0.0	0.0	0.0	0.0
TACGGGGGAT	0.0	0.0	0.0	0.0	0.0	0.0	0.0
TACGGAGGG	0.0	0.0	0.0	0.0	0.0	0.0	0.0
TACGGAGGAT	0.0	0.0	0.0	0.0	0.0	0.0	0.0
TACGTAGGG	0.0	0.0	0.0	0.0	0.0	0.0	0.0
TACGTAGGTG	0.0	0.0	0.0	0.0	0.0	0.0	0.0
TACGTAGGTG	0.0	0.0	0.0	0.0	0.0	0.0	0.0
TACGGAAGGT	0.0	0.0	0.0	0.0	0.0	0.0	0.0
TACGTAGGTG	0.0	0.0	0.0	0.0	0.0	0.0	0.0
TACGGAGGAT	0.0	0.0	0.0	0.0	0.0	0.0	0.0
TACGTAGGG	0.0	0.0	0.0	0.0	0.0	0.0	0.0
TACGTAGGTG	0.0	0.0	0.0	0.0	0.0	0.0	0.0
TACGTAGGG	0.0	0.0	0.0	0.0	0.0	0.0	0.0
TACGTATGGT	0.0	0.0	0.0	0.0	0.0	0.0	0.0
TACGTAGGTG	0.0	0.0	0.0	0.0	0.0	0.0	0.0
AACGTAGGTC	0.0	0.0	0.0	0.0	0.0	0.0	0.0
TACGTAGGTG	0.0	0.0	0.0	0.0	0.0	0.0	0.0
TACGTAGGTG	0.0	0.0	0.0	0.0	0.0	0.0	0.0
TACGTAGGG	0.0	0.0	0.0	0.0	0.0	0.0	0.0
TACGTAGGTG	0.0	0.0	0.0	0.0	0.0	0.0	0.0
TACGTAGGG	0.0	0.0	0.0	0.0	0.0	0.0	0.0
TACGGAAGGT	0.0	0.0	0.0	0.0	0.0	0.0	0.0
TACGTAGGTG	0.0	0.0	0.0	0.0	0.0	0.0	0.0
AACGTAGGTC	0.0	0.0	0.0	0.0	0.0	0.0	0.0
TACGTAGGTG	0.0	0.0	0.0	0.0	0.0	0.0	0.0
TACGGAAGGT	0.0	0.0	0.0	0.0	0.0	0.0	0.0
TACGTAGGGA	0.0	0.0	0.0	0.0	0.0	0.0	0.0
TACGTATGGT	0.0	0.0	0.0	0.0	0.0	0.0	0.0
TACGTATGGT	0.0	0.0	0.0	0.0	0.0	0.0	0.0
TACGTATGGT	0.0	0.0	0.0	0.0	0.0	0.0	0.0
TACGTAGGGT	0.0	0.0	0.0	0.0	0.0	0.0	0.0
TACGTAGGGA	0.0	0.0	0.0	0.0	0.0	0.0	0.0
TACGGAAGGT	0.0	0.0	0.0	0.0	0.0	0.0	0.0
AACGTAGGGT	0.0	0.0	0.0	0.0	0.0	0.0	0.0
TACGTAGGTG	0.0	0.0	0.0	0.0	0.0	0.0	0.0
TACGTAGGGC	0.0	0.0	0.0	0.0	0.0	0.0	0.0
TACGTAGGG	0.0	0.0	0.0	0.0	0.0	0.0	0.0
AACGTAGGTG	0.0	0.0	0.0	0.0	0.0	0.0	0.0
TACGTAGGGA	0.0	0.0	0.0	0.0	0.0	0.0	0.0
TACGTAGGG	0.0	0.0	0.0	0.0	0.0	0.0	0.0
TACGTAGGG	0.0	0.0	0.0	0.0	0.0	0.0	0.0

TACGTAGGGA	0.0	0.0	0.0	0.0	0.0	0.0	0.0
TACGTAGGGA	0.0	0.0	0.0	0.0	0.0	0.0	0.0
TACGTAGGGG	0.0	0.0	0.0	0.0	0.0	0.0	0.0
TACGGAGGAT	0.0	0.0	0.0	0.0	0.0	0.0	0.0
TACGTATGGA	0.0	0.0	0.0	0.0	0.0	0.0	0.0
TACTTCTTCTC	0.0	0.0	0.0	0.0	0.0	0.0	0.0
TACGTAGGGG	0.0	0.0	0.0	0.0	0.0	0.0	0.0
TACGTAGGTG	0.0	0.0	0.0	0.0	0.0	0.0	0.0
AACGTAGGTC	0.0	0.0	0.0	0.0	0.0	0.0	0.0
TACGTATGGT	0.0	0.0	0.0	0.0	0.0	0.0	0.0
TACGGAGGAT	0.0	0.0	0.0	0.0	0.0	0.0	0.0
TACGTAGGTG	0.0	0.0	0.0	0.0	0.0	0.0	0.0
TACGTATGGA	0.0	0.0	0.0	0.0	0.0	0.0	0.0
TACGTATGGT	0.0	0.0	0.0	0.0	0.0	0.0	0.0
AACGTATGGT	0.0	0.0	0.0	0.0	0.0	0.0	0.0
TACGGAGGAT	0.0	0.0	0.0	0.0	0.0	0.0	0.0
TACGTAGGGG	0.0	0.0	0.0	0.0	0.0	0.0	0.0
TACGGAGGAT	0.0	0.0	0.0	0.0	0.0	0.0	0.0
AACGTAGGTC	0.0	0.0	0.0	0.0	0.0	0.0	0.0
TACGTAGGTG	0.0	0.0	0.0	0.0	0.0	0.0	0.0
TACGTAGGTG	0.0	0.0	0.0	0.0	0.0	0.0	0.0
TACGTAGGGA	0.0	0.0	0.0	0.0	0.0	0.0	0.0
TACGTAGGGG	0.0	0.0	0.0	0.0	0.0	0.0	0.0
TACGTAGGGG	0.0	0.0	0.0	0.0	0.0	0.0	0.0
TACGTAGGTG	0.0	0.0	0.0	0.0	0.0	0.0	0.0
TACGTATGGT	0.0	0.0	0.0	0.0	0.0	0.0	0.0
TACGTAGGTG	0.0	0.0	0.0	0.0	0.0	0.0	0.0
TACGTAGGGG	0.0	0.0	0.0	0.0	0.0	0.0	0.0
TACGAAGGGG	0.0	0.0	0.0	0.0	0.0	0.0	0.0
TACGTAGGTT	0.0	0.0	0.0	0.0	0.0	0.0	0.0
AACGTAGGGT	0.0	0.0	0.0	0.0	0.0	0.0	0.0
TACGTAGGGG	0.0	0.0	0.0	0.0	0.0	0.0	0.0
TACGAAGGGT	0.0	0.0	0.0	0.0	0.0	0.0	0.0
TACGTAGGGG	0.0	0.0	0.0	0.0	0.0	0.0	0.0
TACGTAGGGG	0.0	0.0	0.0	0.0	0.0	0.0	0.0
TACGTAGGGG	0.0	0.0	0.0	0.0	0.0	0.0	0.0
CACGTAGGGG	0.0	0.0	0.0	0.0	0.0	0.0	0.0
TACGTGGGTC	0.0	0.0	0.0	0.0	0.0	0.0	0.0
TACGTAGGGA	0.0	0.0	0.0	0.0	0.0	0.0	0.0
TACGTAGGTG	0.0	0.0	0.0	0.0	0.0	0.0	0.0
TACGTAGGTG	0.0	0.0	0.0	0.0	0.0	0.0	0.0
TACGTAGGTG	0.0	0.0	0.0	0.0	0.0	0.0	0.0
TACGTATGGA	0.0	0.0	0.0	0.0	0.0	0.0	0.0
TACGGAGGAT	0.0	0.0	0.0	0.0	0.0	0.0	0.0
TACAGAGGTC	0.0	0.0	0.0	0.0	0.0	0.0	0.0
TACGGAGGAT	0.0	0.0	0.0	0.0	0.0	0.0	0.0
TACGTAGGTG	0.0	0.0	0.0	0.0	0.0	0.0	0.0

TCCTTCTTTTT	0.0	0.0	0.0	0.0	0.0	0.0	0.0
TACGGGGGGT	0.0	0.0	0.0	0.0	0.0	0.0	0.0
TACGTAGGTG	0.0	0.0	0.0	0.0	0.0	0.0	0.0
TACGTATGTC	0.0	0.0	0.0	0.0	0.0	0.0	0.0
TACGTAGGGG	0.0	0.0	0.0	0.0	0.0	0.0	0.0
TACGTAGGTG	0.0	0.0	0.0	0.0	0.0	0.0	0.0
TACGTATGGA	0.0	0.0	0.0	0.0	0.0	0.0	0.0
TACGGAGGAT	0.0	0.0	0.0	0.0	0.0	0.0	0.0
TACGTAGGGG	0.0	0.0	0.0	0.0	0.0	0.0	0.0
TACGTAGGGG	0.0	0.0	0.0	0.0	0.0	0.0	0.0
TACGGAGGAT	0.0	0.0	0.0	0.0	0.0	0.0	0.0
TACGTAGGTG	0.0	0.0	0.0	0.0	0.0	0.0	0.0
TACGTAGGGG	0.0	0.0	0.0	0.0	0.0	0.0	0.0
TACGTAGGGG	0.0	0.0	0.0	0.0	0.0	0.0	0.0
TACGTATGGA	0.0	0.0	0.0	0.0	0.0	0.0	0.0
TACGTAGGTG	0.0	0.0	0.0	0.0	0.0	0.0	0.0
TACGTAGGTG	0.0	0.0	0.0	0.0	0.0	0.0	0.0
TACGTAGGTG	0.0	0.0	0.0	0.0	0.0	0.0	0.0
TACGTAGGTG	0.0	0.0	0.0	0.0	0.0	0.0	0.0
TACGTAGGTG	0.0	0.0	0.0	0.0	0.0	0.0	0.0
TACTTCCTTTC	0.0	0.0	0.0	0.0	0.0	0.0	0.0
TACGTAGGTG	0.0	0.0	0.0	0.0	0.0	0.0	0.0
TACGGAGGAT	0.0	0.0	0.0	0.0	0.0	0.0	0.0
TACGTAGGGG	0.0	0.0	0.0	0.0	0.0	0.0	0.0
TACGTATGGT	0.0	0.0	0.0	0.0	0.0	0.0	0.0
TACGTAGGGT	0.0	0.0	0.0	0.0	0.0	0.0	0.0
TACTTCTTTTT	0.0	0.0	0.0	0.0	0.0	0.0	0.0
TACGTAGGGG	0.0	0.0	0.0	0.0	0.0	0.0	0.0
TACGTAGGTG	0.0	0.0	0.0	0.0	0.0	0.0	0.0
AACGTAGGTC	0.0	0.0	0.0	0.0	0.0	0.0	0.0
TACGTAGGTG	0.0	0.0	0.0	0.0	0.0	0.0	0.0
AACGTAGGGT	0.0	0.0	0.0	0.0	0.0	0.0	0.0
TACGTATGGA	0.0	0.0	0.0	0.0	0.0	0.0	0.0
TACGTAGGGC	0.0	0.0	0.0	0.0	0.0	0.0	0.0
TACGTATGGT	0.0	0.0	0.0	0.0	0.0	0.0	0.0
TACGTAGGGG	0.0	0.0	0.0	0.0	0.0	0.0	0.0
TACGTAGGGA	0.0	0.0	0.0	0.0	0.0	0.0	0.0
TACGGAGGAT	0.0	0.0	0.0	0.0	0.0	0.0	0.0
TACGTAGGGA	0.0	0.0	0.0	0.0	0.0	0.0	0.0
TACGTAGGTG	0.0	0.0	0.0	0.0	0.0	0.0	0.0
TACGGAGGGT	0.0	0.0	0.0	0.0	0.0	0.0	0.0
TACGTAGGGG	0.0	0.0	0.0	0.0	0.0	0.0	0.0
AACGTAGGGT	0.0	0.0	0.0	0.0	0.0	0.0	0.0
TACGTATGGC	0.0	0.0	0.0	0.0	0.0	0.0	0.0
TACGTAGGTG	0.0	0.0	0.0	0.0	0.0	0.0	0.0
TACGGAGGAT	0.0	0.0	0.0	0.0	0.0	0.0	0.0
TACGTAGGGA	0.0	0.0	0.0	0.0	0.0	0.0	0.0

TACGTAGGG(	0.0	0.0	0.0	0.0	0.0	0.0	0.0
TACGTAGGG(	0.0	0.0	0.0	0.0	0.0	0.0	0.0
TACGTAGGG(	0.0	0.0	0.0	0.0	0.0	0.0	0.0
TACGGAGGA1	0.0	0.0	0.0	0.0	0.0	0.0	0.0
TACGGAGGA1	0.0	0.0	0.0	0.0	0.0	0.0	0.0
TACGTAGGGA	0.0	0.0	0.0	0.0	0.0	0.0	0.0
TACGTAGGTG	0.0	0.0	0.0	0.0	0.0	0.0	0.0
TACGTAGGTG	0.0	0.0	0.0	0.0	0.0	0.0	0.0
TACGGAAGG1	0.0	0.0	0.0	0.0	0.0	0.0	0.0
TACGTAGGG(	0.0	0.0	0.0	0.0	0.0	0.0	0.0
TACGTAGGTG	0.0	0.0	0.0	0.0	0.0	0.0	0.0
TACGTAGGG(	0.0	0.0	0.0	0.0	0.0	0.0	0.0
TACGTAGGTG	0.0	0.0	0.0	0.0	0.0	0.0	0.0
TACGGAGGG1	0.0	0.0	0.0	0.0	0.0	0.0	0.0
TACGTAGGTG	0.0	0.0	0.0	0.0	0.0	0.0	0.0
AACATAGGG(	0.0	0.0	0.0	0.0	0.0	0.0	0.0
TACGTATGGA	0.0	0.0	0.0	0.0	0.0	0.0	0.0
TACGTAGGGA	0.0	0.0	0.0	0.0	0.0	0.0	0.0
TACGGAGGG1	0.0	0.0	0.0	0.0	0.0	0.0	0.0
TACGGAGGA1	0.0	0.0	0.0	0.0	0.0	0.0	0.0
TACGTAGGTG	0.0	0.0	0.0	0.0	0.0	0.0	0.0
TACGTAGGTG	0.0	0.0	0.0	0.0	0.0	0.0	0.0
TACGTAGGG(	0.0	0.0	0.0	0.0	0.0	0.0	0.0
TACGTAGGTG	0.0	0.0	0.0	0.0	0.0	0.0	0.0
TACGTAGGGA	0.0	0.0	0.0	0.0	0.0	0.0	0.0
TACGTAGGG(	0.0	0.0	0.0	0.0	0.0	0.0	0.0
TACGTAGGTG	0.0	0.0	0.0	0.0	0.0	0.0	0.0
TACGTAGGTG	0.0	0.0	0.0	0.0	0.0	0.0	0.0
TACGTAGGTG	0.0	0.0	0.0	0.0	0.0	0.0	0.0
TACGTAGGG(	0.0	0.0	0.0	0.0	0.0	0.0	0.0
TACGGAGGG1	0.0	0.0	0.0	0.0	0.0	0.0	0.0
TACGTAGGG(	0.0	0.0	0.0	0.0	0.0	0.0	0.0
TACGTAGGTG	0.0	0.0	0.0	0.0	0.0	0.0	0.0
TACGTAGGTG	0.0	0.0	0.0	0.0	0.0	0.0	0.0
TACGGAGGG1	0.0	0.0	0.0	0.0	0.0	0.0	0.0
TACGGAGGG1	0.0	0.0	0.0	0.0	0.0	0.0	0.0
TACGTAGGG(	0.0	0.0	0.0	0.0	0.0	0.0	0.0
TACGGGGGA1	0.0	0.0	0.0	0.0	0.0	0.0	0.0
TACGTAGGG(	0.0	0.0	0.0	0.0	0.0	0.0	0.0
TACGTAGGTG	0.0	0.0	0.0	0.0	0.0	0.0	0.0
TACGTATGGG	0.0	0.0	0.0	0.0	0.0	0.0	0.0
TACGTATGGT	0.0	0.0	0.0	0.0	0.0	0.0	0.0
TACGTAGGGA	0.0	0.0	0.0	0.0	0.0	0.0	0.0
TACGTAGGG(	0.0	0.0	0.0	0.0	0.0	0.0	0.0
TACGTAGGG(	0.0	0.0	0.0	0.0	0.0	0.0	0.0
TACGTAGGG(	0.0	0.0	0.0	0.0	0.0	0.0	0.0
TACGTAGGG(	0.0	0.0	0.0	0.0	0.0	0.0	0.0

TACGTAGGGA	0.0	0.0	0.0	0.0	0.0	0.0	0.0
TACGGAGGGT	0.0	0.0	0.0	0.0	0.0	0.0	0.0
TACGTAGGGG	0.0	0.0	0.0	0.0	0.0	0.0	0.0
TACGGAGGGT	0.0	0.0	0.0	0.0	0.0	0.0	0.0
TACGTAGGTG	0.0	0.0	0.0	0.0	0.0	0.0	0.0
TACGTAGGGG	0.0	0.0	0.0	0.0	0.0	0.0	0.0
TACGTAGGTG	0.0	0.0	0.0	0.0	0.0	0.0	0.0
TACGGAGGGT	0.0	0.0	0.0	0.0	0.0	0.0	0.0
AACGTAGGTC	0.0	0.0	0.0	0.0	0.0	0.0	0.0
TACGTATGGA	0.0	0.0	0.0	0.0	0.0	0.0	0.0
TACGTATGGT	0.0	0.0	0.0	0.0	0.0	0.0	0.0
AACGTAGGTC	0.0	0.0	0.0	0.0	0.0	0.0	0.0
TACGTAGGGA	0.0	0.0	0.0	0.0	0.0	0.0	0.0
TACGGAGGAT	0.0	0.0	0.0	0.0	0.0	0.0	0.0
TACGTAGGGG	0.0	0.0	0.0	0.0	0.0	0.0	0.0
TACGTAGGGG	0.0	0.0	0.0	0.0	0.0	0.0	0.0
TACGTAGGGG	0.0	0.0	0.0	0.0	0.0	0.0	0.0
TACGTAGGGG	0.0	0.0	0.0	0.0	0.0	0.0	0.0
TACGTAGGGG	0.0	0.0	0.0	0.0	0.0	0.0	0.0
TACGTATGGA	0.0	0.0	0.0	0.0	0.0	0.0	0.0
TACGTAGGGG	0.0	0.0	0.0	0.0	0.0	0.0	0.0
TACGTAGGGG	0.0	0.0	0.0	0.0	0.0	0.0	0.0
TACGTAGGTG	0.0	0.0	0.0	0.0	0.0	0.0	0.0
TACGTAGGGG	0.0	0.0	0.0	0.0	0.0	0.0	0.0
TACGTAGGGT	0.0	0.0	0.0	0.0	0.0	0.0	0.0
TACGTAGGGA	0.0	0.0	0.0	0.0	0.0	0.0	0.0
TACGTAGGTG	0.0	0.0	0.0	0.0	0.0	0.0	0.0
TACGGAGGGC	0.0	0.0	0.0	0.0	0.0	0.0	0.0
TACGTAGGTT	0.0	0.0	0.0	0.0	0.0	0.0	0.0
TACGGAGGAT	0.0	0.0	0.0	0.0	0.0	0.0	0.0
TACGGAGGAT	0.0	0.0	0.0	0.0	0.0	0.0	0.0
TACGTAGGGG	0.0	0.0	0.0	0.0	0.0	0.0	0.0
TACGTAGGGG	0.0	0.0	0.0	0.0	0.0	0.0	0.0
TCCGTCTGGC	0.0	0.0	0.0	0.0	0.0	0.0	0.0
TACGTAGGGG	0.0	0.0	0.0	0.0	0.0	0.0	0.0
TACGTAGGGG	0.0	0.0	0.0	0.0	0.0	0.0	0.0
TACGTAGGGG	0.0	0.0	0.0	0.0	0.0	0.0	0.0
TACGGAGGAT	0.0	0.0	0.0	0.0	0.0	0.0	0.0
TACGTAGGGG	0.0	0.0	0.0	0.0	0.0	0.0	0.0
TACGTAGGGG	0.0	0.0	0.0	0.0	0.0	0.0	0.0
TACGTAGGGG	0.0	0.0	0.0	0.0	0.0	0.0	0.0
TACGTAGGTG	0.0	0.0	0.0	0.0	0.0	0.0	0.0
TACGTAGGGG	0.0	0.0	0.0	0.0	0.0	0.0	0.0
AACGTAGGTG	0.0	0.0	0.0	0.0	0.0	0.0	0.0
TACGTAGGTG	0.0	0.0	0.0	0.0	0.0	0.0	0.0
TACGGAGGAT	0.0	0.0	0.0	0.0	0.0	0.0	0.0
TACGGAGGAT	0.0	0.0	0.0	0.0	0.0	0.0	0.0

TACGTAGGTG	0.0	0.0	0.0	0.0	0.0	0.0	0.0
TACGGAGGAT	0.0	0.0	0.0	0.0	0.0	0.0	0.0
TACGGAGGGT	0.0	0.0	0.0	0.0	0.0	0.0	0.0
TACGTAGGTG	0.0	0.0	0.0	0.0	0.0	0.0	0.0
TACGTAGGGG	0.0	0.0	0.0	0.0	0.0	0.0	0.0
TACGTAGGGG	0.0	0.0	0.0	0.0	0.0	0.0	0.0
AACGTAGGTG	0.0	0.0	0.0	0.0	0.0	0.0	0.0
TACGTAGGTG	0.0	0.0	0.0	0.0	0.0	0.0	0.0
TACGTAGGGG	0.0	0.0	0.0	0.0	0.0	0.0	0.0
TACGTAGGGG	0.0	0.0	0.0	0.0	0.0	0.0	0.0
TACGTAGGTG	0.0	0.0	0.0	0.0	0.0	0.0	0.0
TACGTAGGGG	0.0	0.0	0.0	0.0	0.0	0.0	0.0
TACGTAGGTG	0.0	0.0	0.0	0.0	0.0	0.0	0.0
TACTTCTTCTC	0.0	0.0	0.0	0.0	0.0	0.0	0.0
TACGTAGGTG	0.0	0.0	0.0	0.0	0.0	0.0	0.0
TACGTAGGGG	0.0	0.0	0.0	0.0	0.0	0.0	0.0
TACTTCTTCTC	0.0	0.0	0.0	0.0	0.0	0.0	0.0
TACGTAGGGA	0.0	0.0	0.0	0.0	0.0	0.0	0.0
TACGTAGGGA	0.0	0.0	0.0	0.0	0.0	0.0	0.0
TACGTAGGGG	0.0	0.0	0.0	0.0	0.0	0.0	0.0
TACGTAGGGG	0.0	0.0	0.0	0.0	0.0	0.0	0.0
TACGTAGGGG	0.0	0.0	0.0	0.0	0.0	0.0	0.0
TACGTAGGGG	0.0	0.0	0.0	0.0	0.0	0.0	0.0
TACGTAGGGG	0.0	0.0	0.0	0.0	0.0	0.0	0.0
TACGGAGGAT	0.0	0.0	0.0	0.0	0.0	0.0	0.0
AACGTAGGGT	0.0	0.0	0.0	0.0	0.0	0.0	0.0
TACGTAGGGG	0.0	0.0	0.0	0.0	0.0	0.0	0.0
TACGTAGGGG	0.0	0.0	0.0	0.0	0.0	0.0	0.0
TACGTAGGTG	0.0	0.0	0.0	0.0	0.0	0.0	0.0
TACGTAGGGG	0.0	0.0	0.0	0.0	0.0	0.0	0.0
TACGTATGGT	0.0	0.0	0.0	0.0	0.0	0.0	0.0
TACGTAGGGA	0.0	0.0	0.0	0.0	0.0	0.0	0.0
TACGTAGGGG	0.0	0.0	0.0	0.0	0.0	0.0	0.0
TACGTAGGTG	0.0	0.0	0.0	0.0	0.0	0.0	0.0
TACGTAGGGA	0.0	0.0	0.0	0.0	0.0	0.0	0.0
TACGTAGGGG	0.0	0.0	0.0	0.0	0.0	0.0	0.0
TACGTAGGTG	0.0	0.0	0.0	0.0	0.0	0.0	0.0
TACGTAGGGA	0.0	0.0	0.0	0.0	0.0	0.0	0.0
TACGTAGGGG	0.0	0.0	0.0	0.0	0.0	0.0	0.0
TACGTAGGTG	0.0	0.0	0.0	0.0	0.0	0.0	0.0
TACGTAGGGA	0.0	0.0	0.0	0.0	0.0	0.0	0.0
TACGTAGGGG	0.0	0.0	0.0	0.0	0.0	0.0	0.0
TACGTAGGGG	0.0	0.0	0.0	0.0	0.0	0.0	0.0
TACGTAGGAT	0.0	0.0	0.0	0.0	0.0	0.0	0.0
TACGGAGGAT	0.0	0.0	0.0	0.0	0.0	0.0	0.0
TACGTAGGTG	0.0	0.0	0.0	0.0	0.0	0.0	0.0
TACGGAGGAT	0.0	0.0	0.0	0.0	0.0	0.0	0.0

TACGGAGGAT	0.0	0.0	0.0	0.0	0.0	0.0	0.0
TACGGAGGAT	0.0	0.0	0.0	0.0	0.0	0.0	0.0
TACGTAGGTG	0.0	0.0	0.0	0.0	0.0	0.0	0.0
AACGTAGGGT	0.0	0.0	0.0	0.0	0.0	0.0	0.0
CTCTTCTTTT	0.0	0.0	0.0	0.0	0.0	0.0	0.0
TACGTAGGGA	0.0	0.0	0.0	0.0	0.0	0.0	0.0
TACGTAGGTG	0.0	0.0	0.0	0.0	0.0	0.0	0.0
TACAGAGGTC	0.0	0.0	0.0	0.0	0.0	0.0	0.0
TACGGAGGAT	0.0	0.0	0.0	0.0	0.0	0.0	0.0
TACGGAGGAT	0.0	0.0	0.0	0.0	0.0	0.0	0.0
TACGTAGGGG	0.0	0.0	0.0	0.0	0.0	0.0	0.0
TACGTATGGA	0.0	0.0	0.0	0.0	0.0	0.0	0.0
TACGTAGGGG	0.0	0.0	0.0	0.0	0.0	0.0	0.0
TACAGAGGGT	0.0	0.0	0.0	0.0	0.0	0.0	0.0
TACGTAGGGG	0.0	0.0	0.0	0.0	0.0	0.0	0.0
TACGTAGGGG	0.0	0.0	0.0	0.0	0.0	0.0	0.0
TACGGAGGAT	0.0	0.0	0.0	0.0	0.0	0.0	0.0
TACGTATGGT	0.0	0.0	0.0	0.0	0.0	0.0	0.0
TACGTATGGA	0.0	0.0	0.0	0.0	0.0	0.0	0.0
TACGTAGGGG	0.0	0.0	0.0	0.0	0.0	0.0	0.0
AACGTAGGGT	0.0	0.0	0.0	0.0	0.0	0.0	0.0
TACGTATGGT	0.0	0.0	0.0	0.0	0.0	0.0	0.0
TACGTAGGGG	0.0	0.0	0.0	0.0	0.0	0.0	0.0
TACGTAGGGG	0.0	0.0	0.0	0.0	0.0	0.0	0.0
TACGTATGGT	0.0	0.0	0.0	0.0	0.0	0.0	0.0
TACGGAGGAT	0.0	0.0	0.0	0.0	0.0	0.0	0.0
TACGTAGGGG	0.0	0.0	0.0	0.0	0.0	0.0	0.0
TACGTAGGGG	0.0	0.0	0.0	0.0	0.0	0.0	0.0
TACGTAGGGG	0.0	0.0	0.0	0.0	0.0	0.0	0.0
TACGTAGGGG	0.0	0.0	0.0	0.0	0.0	0.0	0.0
TACGTAGGGG	0.0	0.0	0.0	0.0	0.0	0.0	0.0
TACGTATGGT	0.0	0.0	0.0	0.0	0.0	0.0	0.0
AACGTAGGTG	0.0	0.0	0.0	0.0	0.0	0.0	0.0
TACGTATGGT	0.0	0.0	0.0	0.0	0.0	0.0	0.0
TACGTAGGTG	0.0	0.0	0.0	0.0	0.0	0.0	0.0
TACGTATGGT	0.0	0.0	0.0	0.0	0.0	0.0	0.0
TACGTAGGGG	0.0	0.0	0.0	0.0	0.0	0.0	0.0
TACGTAGGTG	0.0	0.0	0.0	0.0	0.0	0.0	0.0
TACAGAGGAT	0.0	0.0	0.0	0.0	0.0	0.0	0.0
TACGTAGGGG	0.0	0.0	0.0	0.0	0.0	0.0	0.0
TACGTATGGT	0.0	0.0	0.0	0.0	0.0	0.0	0.0
TACGTAGGGG	0.0	0.0	0.0	0.0	0.0	0.0	0.0
TACGTAGGGG	0.0	0.0	0.0	0.0	0.0	0.0	0.0
AACGTAGGGT	0.0	0.0	0.0	0.0	0.0	0.0	0.0
TACGTAGGGG	0.0	0.0	0.0	0.0	0.0	0.0	0.0
TACGTATGGA	0.0	0.0	0.0	0.0	0.0	0.0	0.0
TACGTATGGA	0.0	0.0	0.0	0.0	0.0	0.0	0.0

TACGTAGGG	0.0	0.0	0.0	0.0	0.0	0.0	0.0
TACGTAGGTG	0.0	0.0	0.0	0.0	0.0	0.0	0.0
TACGTAGGTG	0.0	0.0	0.0	0.0	0.0	0.0	0.0
TACGTAGGTG	0.0	0.0	0.0	0.0	0.0	0.0	0.0
TACGTAGAG	0.0	0.0	0.0	0.0	0.0	0.0	0.0
TACGTAGGG	0.0	0.0	0.0	0.0	0.0	0.0	0.0
TACGTAGGG	0.0	0.0	0.0	0.0	0.0	0.0	0.0
TACGTAGGTG	0.0	0.0	0.0	0.0	0.0	0.0	0.0
TACGTAGGGA	0.0	0.0	0.0	0.0	0.0	0.0	0.0
TACGTAGGGA	0.0	0.0	0.0	0.0	0.0	0.0	0.0
TACGTATGGT	0.0	0.0	0.0	0.0	0.0	0.0	0.0
TACGTAGGTG	0.0	0.0	0.0	0.0	0.0	0.0	0.0
TACGTAGGTG	0.0	0.0	0.0	0.0	0.0	0.0	0.0
TACGTAGGTG	0.0	0.0	0.0	0.0	0.0	0.0	0.0
TACGTAGGGA	0.0	0.0	0.0	0.0	0.0	0.0	0.0
TACGTAGGGA	0.0	0.0	0.0	0.0	0.0	0.0	0.0
TACGTAGGG	0.0	0.0	0.0	0.0	0.0	0.0	0.0
AACATAGGG	0.0	0.0	0.0	0.0	0.0	0.0	0.0
TACGTAGGGA	0.0	0.0	0.0	0.0	0.0	0.0	0.0
TACGTAGGGA	0.0	0.0	0.0	0.0	0.0	0.0	0.0
TACGTATGGT	0.0	0.0	0.0	0.0	0.0	0.0	0.0
TACGTAGGTG	0.0	0.0	0.0	0.0	0.0	0.0	0.0
TACGTAGGTG	0.0	0.0	0.0	0.0	0.0	0.0	0.0
TACGTAGGG	0.0	0.0	0.0	0.0	0.0	0.0	0.0
TACGTAGGG	0.0	0.0	0.0	0.0	0.0	0.0	0.0
TACGTAGGGA	0.0	0.0	0.0	0.0	0.0	0.0	0.0
TACGTAGGG	0.0	0.0	0.0	0.0	0.0	0.0	0.0
TACGTAGGTG	0.0	0.0	0.0	0.0	0.0	0.0	0.0
TACGTAGGTG	0.0	0.0	0.0	0.0	0.0	0.0	0.0
TACGTAGGG	0.0	0.0	0.0	0.0	0.0	0.0	0.0
TACGTAGGGA	0.0	0.0	0.0	0.0	0.0	0.0	0.0
TACGTAGGTG	0.0	0.0	0.0	0.0	0.0	0.0	0.0
TACGTAGGG	0.0	0.0	0.0	0.0	0.0	0.0	0.0
TACGTAGGTG	0.0	0.0	0.0	0.0	0.0	0.0	0.0
TACGTAGGTG	0.0	0.0	0.0	0.0	0.0	0.0	0.0
TACGGAAGGT	0.0	0.0	0.0	0.0	0.0	0.0	0.0
TACGTAGGTG	0.0	0.0	0.0	0.0	0.0	0.0	0.0
TACGTAGGGA	0.0	0.0	0.0	0.0	0.0	0.0	0.0
TACGTAGGTG	0.0	0.0	0.0	0.0	0.0	0.0	0.0
TACGTAGGGA	0.0	0.0	0.0	0.0	0.0	0.0	0.0
TACGTAGGG	0.0	0.0	0.0	0.0	0.0	0.0	0.0
TACGTAGGGA	0.0	0.0	0.0	0.0	0.0	0.0	0.0
TACGTAGGG	0.0	0.0	0.0	0.0	0.0	0.0	0.0
TACGTAGGG	0.0	0.0	0.0	0.0	0.0	0.0	0.0
TACGTAGGG	0.0	0.0	0.0	0.0	0.0	0.0	0.0
TACGTAGGTG	0.0	0.0	0.0	0.0	0.0	0.0	0.0
TACGGAGGGT	0.0	0.0	0.0	0.0	0.0	0.0	0.0

TACGTAGGGG	0.0	0.0	0.0	0.0	0.0	0.0	0.0
TACGTAGGGG	0.0	0.0	0.0	0.0	0.0	0.0	0.0
TACGTAGGGG	0.0	0.0	0.0	0.0	0.0	0.0	0.0
TACGTAGGGG	0.0	0.0	0.0	0.0	0.0	0.0	0.0
TACGTAGGGG	0.0	0.0	0.0	0.0	0.0	0.0	0.0
TACGGAGGAT	0.0	0.0	0.0	0.0	0.0	0.0	0.0
TACGTAGGGG	0.0	0.0	0.0	0.0	0.0	0.0	0.0
TACGTAGGTG	0.0	0.0	0.0	0.0	0.0	0.0	0.0
TACGTAGGGG	0.0	0.0	0.0	0.0	0.0	0.0	0.0
TACGTAGGGT	0.0	0.0	0.0	0.0	0.0	0.0	0.0
TACGTAGGGA	0.0	0.0	0.0	0.0	0.0	0.0	0.0
TACGTAGGTG	0.0	0.0	0.0	0.0	0.0	0.0	0.0
TACGTAGGGG	0.0	0.0	0.0	0.0	0.0	0.0	0.0
TACGTAGGGG	0.0	0.0	0.0	0.0	0.0	0.0	0.0
TACGTAGGGG	0.0	0.0	0.0	0.0	0.0	0.0	0.0
TACGTAGGTG	0.0	0.0	0.0	0.0	0.0	0.0	0.0
TACGTAGGGG	0.0	0.0	0.0	0.0	0.0	0.0	0.0
TACGTAGGGG	0.0	0.0	0.0	0.0	0.0	0.0	0.0
TACGTAGGTG	0.0	0.0	0.0	0.0	0.0	0.0	0.0
TACGTAGGGG	0.0	0.0	0.0	0.0	0.0	0.0	0.0
TACGTATGGT	0.0	0.0	0.0	0.0	0.0	0.0	0.0
TACGTATGGT	0.0	0.0	0.0	0.0	0.0	0.0	0.0
TACGGGGGAT	0.0	0.0	0.0	0.0	0.0	0.0	0.0
TACGTAGGGA	0.0	0.0	0.0	0.0	0.0	0.0	0.0
TACGGAGGGT	0.0	0.0	0.0	0.0	0.0	0.0	0.0
TACGTAGGTG	0.0	0.0	0.0	0.0	0.0	0.0	0.0
TACGTAGGGA	0.0	0.0	0.0	0.0	0.0	0.0	0.0
TACGTAGGTG	0.0	0.0	0.0	0.0	0.0	0.0	0.0
TACGTAGGTG	0.0	0.0	0.0	0.0	0.0	0.0	0.0
TACGTAGGTG	0.0	0.0	0.0	0.0	0.0	0.0	0.0
TACGTAGGGA	0.0	0.0	0.0	0.0	0.0	0.0	0.0
TACGTAGGGA	0.0	0.0	0.0	0.0	0.0	0.0	0.0
TACGTAGGGG	0.0	0.0	0.0	0.0	0.0	0.0	0.0
TACGTAGGGG	0.0	0.0	0.0	0.0	0.0	0.0	0.0
TACGTAGGGA	0.0	0.0	0.0	0.0	0.0	0.0	0.0
TACGTAGGGG	0.0	0.0	0.0	0.0	0.0	0.0	0.0
TACGTAGGGG	0.0	0.0	0.0	0.0	0.0	0.0	0.0
TACGTAGGGG	0.0	0.0	0.0	0.0	0.0	0.0	0.0
TACGTAGGGG	0.0	0.0	0.0	0.0	0.0	0.0	0.0
TACGTAGGGA	0.0	0.0	0.0	0.0	0.0	0.0	0.0
TACGTAGGTG	0.0	0.0	0.0	0.0	0.0	0.0	0.0
TACGTAGGGG	0.0	0.0	0.0	0.0	0.0	0.0	0.0
TACGTAGGGG	0.0	0.0	0.0	0.0	0.0	0.0	0.0
TACATAGGGA	0.0	0.0	0.0	0.0	0.0	0.0	0.0
TACGTAGGTG	0.0	0.0	0.0	0.0	0.0	0.0	0.0
TACGTAGGTG	0.0	0.0	0.0	0.0	0.0	0.0	0.0
TACGTAGGTG	0.0	0.0	0.0	0.0	0.0	0.0	0.0

TACGTAGGGG	0.0	0.0	0.0	0.0	0.0	0.0	0.0
TACGTAGGTG	0.0	0.0	0.0	0.0	0.0	0.0	0.0
TACGTAGGTG	0.0	0.0	0.0	0.0	0.0	0.0	0.0
TACGTAGGGA	0.0	0.0	0.0	0.0	0.0	0.0	0.0
TACGTAGGTG	0.0	0.0	0.0	0.0	0.0	0.0	0.0
TACGTAGGGG	0.0	0.0	0.0	0.0	0.0	0.0	0.0
TACGTAGGTG	0.0	0.0	0.0	0.0	0.0	0.0	0.0
TACGTAGGTG	0.0	0.0	0.0	0.0	0.0	0.0	0.0
TACGTAGGGG	0.0	0.0	0.0	0.0	0.0	0.0	0.0
AACGTAGGTG	0.0	0.0	0.0	0.0	0.0	0.0	0.0
TACGTATGGT	0.0	0.0	0.0	0.0	0.0	0.0	0.0
TACGTAGGTG	0.0	0.0	0.0	0.0	0.0	0.0	0.0
TACGTAGGTC	0.0	0.0	0.0	0.0	0.0	0.0	0.0
TACGGAAGGT	0.0	0.0	0.0	0.0	0.0	0.0	0.0
TACGTAGGGG	0.0	0.0	0.0	0.0	0.0	0.0	0.0
TACGTAGGTG	0.0	0.0	0.0	0.0	0.0	0.0	0.0
TACGTAGGGA	0.0	0.0	0.0	0.0	0.0	0.0	0.0
TACGTATGGA	0.0	0.0	0.0	0.0	0.0	0.0	0.0
TACGTAGGGG	0.0	0.0	0.0	0.0	0.0	0.0	0.0
TACGTAGGTG	0.0	0.0	0.0	0.0	0.0	0.0	0.0
TACGTAGGGG	0.0	0.0	0.0	0.0	0.0	0.0	0.0
TACGTAGGGA	0.0	0.0	0.0	0.0	0.0	0.0	0.0
TACGTAGGTG	0.0	0.0	0.0	0.0	0.0	0.0	0.0
TACAGAGGAT	0.0	0.0	0.0	0.0	0.0	0.0	0.0
TACTTCTTCTC	0.0	0.0	0.0	0.0	0.0	0.0	0.0
TACGTAGGTG	0.0	0.0	0.0	0.0	0.0	0.0	0.0
TACGTAGGGG	0.0	0.0	0.0	0.0	0.0	0.0	0.0
TACGGAGGAT	0.0	0.0	0.0	0.0	0.0	0.0	0.0
TACGTAGGGG	0.0	0.0	0.0	0.0	0.0	0.0	0.0
TACGTATGGT	0.0	0.0	0.0	0.0	0.0	0.0	0.0
TACGTAGGGA	0.0	0.0	0.0	0.0	0.0	0.0	0.0
TACGTAGGGA	0.0	0.0	0.0	0.0	0.0	0.0	0.0
TACGTAGGGG	0.0	0.0	0.0	0.0	0.0	0.0	0.0
CACGTAGGGG	0.0	0.0	0.0	0.0	0.0	0.0	0.0
TACGTAGGGG	0.0	0.0	0.0	0.0	0.0	0.0	0.0
TACGTAGGTG	0.0	0.0	0.0	0.0	0.0	0.0	0.0
TACGTAGGGG	0.0	0.0	0.0	0.0	0.0	0.0	0.0
TACGTATGGG	0.0	0.0	0.0	0.0	0.0	0.0	0.0
TACGTAAGGT	0.0	0.0	0.0	0.0	0.0	0.0	0.0
TCCTTCTTTTT	0.0	0.0	0.0	0.0	0.0	0.0	0.0
TACGTAGGTG	0.0	0.0	0.0	0.0	0.0	0.0	0.0
TACGTAGGGG	0.0	0.0	0.0	0.0	0.0	0.0	0.0
TACGTAGGTG	0.0	0.0	0.0	0.0	0.0	0.0	0.0
TACGTAGGTG	0.0	0.0	0.0	0.0	0.0	0.0	0.0
TACGTAGGGA	0.0	0.0	0.0	0.0	0.0	0.0	0.0
TACGTAGGGA	0.0	0.0	0.0	0.0	0.0	0.0	0.0
TACGTAGGTG	0.0	0.0	0.0	0.0	0.0	0.0	0.0

TACGTAGGGC	0.0	0.0	0.0	0.0	0.0	0.0	0.0
TACGTAGGTG	0.0	0.0	0.0	0.0	0.0	0.0	0.0
TACGTAGGTG	0.0	0.0	0.0	0.0	0.0	0.0	0.0
TACGTAGGGA	0.0	0.0	0.0	0.0	0.0	0.0	0.0
TACGTAGGGG	0.0	0.0	0.0	0.0	0.0	0.0	0.0
TACGTAGGGA	0.0	0.0	0.0	0.0	0.0	0.0	0.0
TACGTAGGTG	0.0	0.0	0.0	0.0	0.0	0.0	0.0
TACGTAGGTG	0.0	0.0	0.0	0.0	0.0	0.0	0.0
TACGTAGGGG	0.0	0.0	0.0	0.0	0.0	0.0	0.0
TACGTAGGGT	0.0	0.0	0.0	0.0	0.0	0.0	0.0
TACGTAGGGG	0.0	0.0	0.0	0.0	0.0	0.0	0.0
TACGTAGGGC	0.0	0.0	0.0	0.0	0.0	0.0	0.0
TACGGAAGGT	0.0	0.0	0.0	0.0	0.0	0.0	0.0
TACGTAGGGA	0.0	0.0	0.0	0.0	0.0	0.0	0.0
TACGTAGGGG	0.0	0.0	0.0	0.0	0.0	0.0	0.0
TACGGAGGAT	0.0	0.0	0.0	0.0	0.0	0.0	0.0
TACGTAGGGT	0.0	0.0	0.0	0.0	0.0	0.0	0.0
TACGTAGGTG	0.0	0.0	0.0	0.0	0.0	0.0	0.0
TACGTAGGGG	0.0	0.0	0.0	0.0	0.0	0.0	0.0
TACGTAGGGG	0.0	0.0	0.0	0.0	0.0	0.0	0.0
TACGTAGGGT	0.0	0.0	0.0	0.0	0.0	0.0	0.0
TACGTAGGGG	0.0	0.0	0.0	0.0	0.0	0.0	0.0
TACGGAAGGT	0.0	0.0	0.0	0.0	0.0	0.0	0.0
TACGTAGGGG	0.0	0.0	0.0	0.0	0.0	0.0	0.0
TACGTAGGTG	0.0	0.0	0.0	0.0	0.0	0.0	0.0
TACGTAGGGG	0.0	0.0	0.0	0.0	0.0	0.0	0.0
TACGTAGGGG	0.0	0.0	0.0	0.0	0.0	0.0	0.0
TACGTAGGGG	0.0	0.0	0.0	0.0	0.0	0.0	0.0
TACGGAGGAT	0.0	0.0	0.0	0.0	0.0	0.0	0.0
TACGGAGGAT	0.0	0.0	0.0	0.0	0.0	0.0	0.0
TACGGAAGGT	0.0	0.0	0.0	0.0	0.0	0.0	0.0
TACGTATGGT	0.0	0.0	0.0	0.0	0.0	0.0	0.0
TACGTAGGGA	0.0	0.0	0.0	0.0	0.0	0.0	0.0
TACGTAGGTG	0.0	0.0	0.0	0.0	0.0	0.0	0.0
TACGTAGGTG	0.0	0.0	0.0	0.0	0.0	0.0	0.0
TACGTAGGGA	0.0	0.0	0.0	0.0	0.0	0.0	0.0
TACGTAGGAG	0.0	0.0	0.0	0.0	0.0	0.0	0.0
TACGTAGGTG	0.0	0.0	0.0	0.0	0.0	0.0	0.0
TACGTAGGTG	0.0	0.0	0.0	0.0	0.0	0.0	0.0
TACGTAGGGG	0.0	0.0	0.0	0.0	0.0	0.0	0.0
TACGTAGGGA	0.0	0.0	0.0	0.0	0.0	0.0	0.0
TACGTAGGTG	0.0	0.0	0.0	0.0	0.0	0.0	0.0
TACGTAGGGG	0.0	0.0	0.0	0.0	0.0	0.0	0.0
TACGTAGGGT	0.0	0.0	0.0	0.0	0.0	0.0	0.0
TACGTAGGGT	0.0	0.0	0.0	0.0	0.0	0.0	0.0
TACGTAGGGA	0.0	0.0	0.0	0.0	0.0	0.0	0.0
TACGTATGGA	0.0	0.0	0.0	0.0	0.0	0.0	0.0

TACGTAGGTG	0.0	0.0	0.0	0.0	0.0	0.0	0.0
TACGTAGGTG	0.0	0.0	0.0	0.0	0.0	0.0	0.0
TACGTAGGGG	0.0	0.0	0.0	0.0	0.0	0.0	0.0
TACGTAGGGA	0.0	0.0	0.0	0.0	0.0	0.0	0.0
TACGTAGGTG	0.0	0.0	0.0	0.0	0.0	0.0	0.0
TACGTAGGGG	0.0	0.0	0.0	0.0	0.0	0.0	0.0
TACGTAGGGG	0.0	0.0	0.0	0.0	0.0	0.0	0.0
TACGTAGGGG	0.0	0.0	0.0	0.0	0.0	0.0	0.0
TACGTAGGTG	0.0	0.0	0.0	0.0	0.0	0.0	0.0
TACGTAGGGG	0.0	0.0	0.0	0.0	0.0	0.0	0.0
TACGTAGGGG	0.0	0.0	0.0	0.0	0.0	0.0	0.0
TACGTAGGGG	0.0	0.0	0.0	0.0	0.0	0.0	0.0
TACGTAGGGG	0.0	0.0	0.0	0.0	0.0	0.0	0.0
TACGTAGGGG	0.0	0.0	0.0	0.0	0.0	0.0	0.0
TACGTAGGTG	0.0	0.0	0.0	0.0	0.0	0.0	0.0
TACGTAGGGA	0.0	0.0	0.0	0.0	0.0	0.0	0.0
TACGTAGGGG	0.0	0.0	0.0	0.0	0.0	0.0	0.0
TACGTAGGTG	0.0	0.0	0.0	0.0	0.0	0.0	0.0
AACGTAGGGA	0.0	0.0	0.0	0.0	0.0	0.0	0.0
TACGTAGGTG	0.0	0.0	0.0	0.0	0.0	0.0	0.0
TACGTAGGGT	0.0	0.0	0.0	0.0	0.0	0.0	0.0
TACGTAGGGG	0.0	0.0	0.0	0.0	0.0	0.0	0.0
TACGTAGGTG	0.0	0.0	0.0	0.0	0.0	0.0	0.0
TACGTAGGGG	0.0	0.0	0.0	0.0	0.0	0.0	0.0
TACGTAGGGG	0.0	0.0	0.0	0.0	0.0	0.0	0.0
TACGTAGGGA	0.0	0.0	0.0	0.0	0.0	0.0	0.0
TACGTAGGTC	0.0	0.0	0.0	0.0	0.0	0.0	0.0
TACGTAGGGG	0.0	0.0	0.0	0.0	0.0	0.0	0.0
TACGTAGGGG	0.0	0.0	0.0	0.0	0.0	0.0	0.0
TACGTAGGTG	0.0	0.0	0.0	0.0	0.0	0.0	0.0
TACGTAGGTG	0.0	0.0	0.0	0.0	0.0	0.0	0.0
TACGTAGGGG	0.0	0.0	0.0	0.0	0.0	0.0	0.0
TACGGAGGAT	0.0	0.0	0.0	0.0	0.0	0.0	0.0
TACGTAGGGG	0.0	0.0	0.0	0.0	0.0	0.0	0.0
TACGTATGGA	0.0	0.0	0.0	0.0	0.0	0.0	0.0
TACGTAGGTG	0.0	0.0	0.0	0.0	0.0	0.0	0.0
TACGTAGGGA	0.0	0.0	0.0	0.0	0.0	0.0	0.0
TACGTAGGTG	0.0	0.0	0.0	0.0	0.0	0.0	0.0
TACGTAGGTG	0.0	0.0	0.0	0.0	0.0	0.0	0.0
TACGTATGGA	0.0	0.0	0.0	0.0	0.0	0.0	0.0
TACGTATGGT	0.0	0.0	0.0	0.0	0.0	0.0	0.0
TACGTAGGTG	0.0	0.0	0.0	0.0	0.0	0.0	0.0
TACGTAGGGG	0.0	0.0	0.0	0.0	0.0	0.0	0.0
TACGTAGGTG	0.0	0.0	0.0	0.0	0.0	0.0	0.0
TACGTAGGGG	0.0	0.0	0.0	0.0	0.0	0.0	0.0
TACGTAGGGG	0.0	0.0	0.0	0.0	0.0	0.0	0.0
TACGTAGGGG	0.0	0.0	0.0	0.0	0.0	0.0	0.0

TACGGAGGGT	0.0	0.0	0.0	0.0	0.0	0.0	0.0
TACGTATGGT	0.0	0.0	0.0	0.0	0.0	0.0	0.0
TACGTATGGA	0.0	0.0	0.0	0.0	0.0	0.0	0.0
TACGTATGGT	0.0	0.0	0.0	0.0	0.0	0.0	0.0
TACGTAGGGG	0.0	0.0	0.0	0.0	0.0	0.0	0.0
TACGTATGGA	0.0	0.0	0.0	0.0	0.0	0.0	0.0
TACGTAGGGG	0.0	0.0	0.0	0.0	0.0	0.0	0.0
TACGTATGGA	0.0	0.0	0.0	0.0	0.0	0.0	0.0
TACGTATGGT	0.0	0.0	0.0	0.0	0.0	0.0	0.0
TACGTAGGGA	0.0	0.0	0.0	0.0	0.0	0.0	0.0
TACAGAGGTC	0.0	0.0	0.0	0.0	0.0	0.0	0.0
TACGTAGGGA	0.0	0.0	0.0	0.0	0.0	0.0	0.0
TACGTAGGTG	0.0	0.0	0.0	0.0	0.0	0.0	0.0
TACGTAGGGG	0.0	0.0	0.0	0.0	0.0	0.0	0.0
TACGGAGGAT	0.0	0.0	0.0	0.0	0.0	0.0	0.0
TACGTAGGGG	0.0	0.0	0.0	0.0	0.0	0.0	0.0
TACGGAGGGT	0.0	0.0	0.0	0.0	0.0	0.0	0.0
TACGTAGGGG	0.0	0.0	0.0	0.0	0.0	0.0	0.0
TACGTAGGGG	0.0	0.0	0.0	0.0	0.0	0.0	0.0
AACGTAGGTC	0.0	0.0	0.0	0.0	0.0	0.0	0.0
TACGTAGGGT	0.0	0.0	0.0	0.0	0.0	0.0	0.0
TACGGAGGAT	0.0	0.0	0.0	0.0	0.0	0.0	0.0
TACGTAGGGA	0.0	0.0	0.0	0.0	0.0	0.0	0.0
TACGTAGGTG	0.0	0.0	0.0	0.0	0.0	0.0	0.0
TACGTAGGGG	0.0	0.0	0.0	0.0	0.0	0.0	0.0
TACGTAAGGG	0.0	0.0	0.0	0.0	0.0	0.0	0.0
TACGTAGGGG	0.0	0.0	0.0	0.0	0.0	0.0	0.0
TACGTAGGGG	0.0	0.0	0.0	0.0	0.0	0.0	0.0
TACGTAGGTG	0.0	0.0	0.0	0.0	0.0	0.0	0.0
TACGTAGGTG	0.0	0.0	0.0	0.0	0.0	0.0	0.0
AACGTAGGTC	0.0	0.0	0.0	0.0	0.0	0.0	0.0
TACGTAGGGG	0.0	0.0	0.0	0.0	0.0	0.0	0.0
TACGTAGATG	0.0	0.0	0.0	0.0	0.0	0.0	0.0
TACGTAGGGG	0.0	0.0	0.0	0.0	0.0	0.0	0.0
TACGTAGGGC	0.0	0.0	0.0	0.0	0.0	0.0	0.0
TACGTAGGGG	0.0	0.0	0.0	0.0	0.0	0.0	0.0
TACGTAGGTG	0.0	0.0	0.0	0.0	0.0	0.0	0.0
TACGTAGGGG	0.0	0.0	0.0	0.0	0.0	0.0	0.0
AACGTAGGTG	0.0	0.0	0.0	0.0	0.0	0.0	0.0
TACGTAGGTG	0.0	0.0	0.0	0.0	0.0	0.0	0.0
TACGTAGGGG	0.0	0.0	0.0	0.0	0.0	0.0	0.0
TACGGAGGAT	0.0	0.0	0.0	0.0	0.0	0.0	0.0
TACGGAGGAT	0.0	0.0	0.0	0.0	0.0	0.0	0.0
TACGTAGGGG	0.0	0.0	0.0	0.0	0.0	0.0	0.0
TACGTAGGGT	0.0	0.0	0.0	0.0	0.0	0.0	0.0
TACGGAGGAT	0.0	0.0	0.0	0.0	0.0	0.0	0.0
TACGGAGGAT	0.0	0.0	0.0	0.0	0.0	0.0	0.0

TACGTAGGTG	0.0	0.0	0.0	0.0	0.0	0.0	0.0
TACGTAGGGG	0.0	0.0	0.0	0.0	0.0	0.0	0.0
TACGTAGGGG	0.0	0.0	0.0	0.0	0.0	0.0	0.0
TACGTAGGGG	0.0	0.0	0.0	0.0	0.0	0.0	0.0
TACGTAGGGG	0.0	0.0	0.0	0.0	0.0	0.0	0.0
TACGTAGGGG	0.0	0.0	0.0	0.0	0.0	0.0	0.0
TACGTAGGTG	0.0	0.0	0.0	0.0	0.0	0.0	0.0
TACGGAGGGT	0.0	0.0	0.0	0.0	0.0	0.0	0.0
TACGTAGGGA	0.0	0.0	0.0	0.0	0.0	0.0	0.0
TACGTATGGT	0.0	0.0	0.0	0.0	0.0	0.0	0.0
TACGTAGGGG	0.0	0.0	0.0	0.0	0.0	0.0	0.0
TACGGAAGGT	0.0	0.0	0.0	0.0	0.0	0.0	0.0
TACGTATGGA	0.0	0.0	0.0	0.0	0.0	0.0	0.0
TACGTAGGTG	0.0	0.0	0.0	0.0	0.0	0.0	0.0
TACGTAGGTG	0.0	0.0	0.0	0.0	0.0	0.0	0.0
TACGGAGGAT	0.0	0.0	0.0	0.0	0.0	0.0	0.0
TACGTAGGGA	0.0	0.0	0.0	0.0	0.0	0.0	0.0
TACGTAGGGG	0.0	0.0	0.0	0.0	0.0	0.0	0.0
TACGTAGGGG	0.0	0.0	0.0	0.0	0.0	0.0	0.0
TACGTAGGGG	0.0	0.0	0.0	0.0	0.0	0.0	0.0
TACGTATGGA	0.0	0.0	0.0	0.0	0.0	0.0	0.0
TACGGAGGGT	0.0	0.0	0.0	0.0	0.0	0.0	0.0
AACGTAGGGT	0.0	0.0	0.0	0.0	0.0	0.0	0.0
TACGTAGGTG	0.0	0.0	0.0	0.0	0.0	0.0	0.0
TACGTAGGGG	0.0	0.0	0.0	0.0	0.0	0.0	0.0
TACGTAGGGA	0.0	0.0	0.0	0.0	0.0	0.0	0.0
TACGGAGGGT	0.0	0.0	0.0	0.0	0.0	0.0	0.0
TACGTAGGGT	0.0	0.0	0.0	0.0	0.0	0.0	0.0
TACGGAGGAT	0.0	0.0	0.0	0.0	0.0	0.0	0.0
TACGTAGGGG	0.0	0.0	0.0	0.0	0.0	0.0	0.0
TACAGAGGGT	0.0	0.0	0.0	0.0	0.0	0.0	0.0
AACGTAGGGT	0.0	0.0	0.0	0.0	0.0	0.0	0.0
TACGTAGGTG	0.0	0.0	0.0	0.0	0.0	0.0	0.0
TACGTAGGTG	0.0	0.0	0.0	0.0	0.0	0.0	0.0
TACGTATGGT	0.0	0.0	0.0	0.0	0.0	0.0	0.0
AACGTAGGGT	0.0	0.0	0.0	0.0	0.0	0.0	0.0
TACGTAGGGG	0.0	0.0	0.0	0.0	0.0	0.0	0.0
TACGTAGGGA	0.0	0.0	0.0	0.0	0.0	0.0	0.0
TACGTAGGGG	0.0	0.0	0.0	0.0	0.0	0.0	0.0
TACGTAGGTG	0.0	0.0	0.0	0.0	0.0	0.0	0.0
TACGTAGGGA	0.0	0.0	0.0	0.0	0.0	0.0	0.0
TACGTAGGTG	0.0	0.0	0.0	0.0	0.0	0.0	0.0
TACGTAGGGG	0.0	0.0	0.0	0.0	0.0	0.0	0.0
TACGTAGGGG	0.0	0.0	0.0	0.0	0.0	0.0	0.0
TACGTATGGT	0.0	0.0	0.0	0.0	0.0	0.0	0.0
TACGTAGGGG	0.0	0.0	0.0	0.0	0.0	0.0	0.0
TACGCAGGGG	0.0	0.0	0.0	0.0	0.0	0.0	0.0

TACGTAGGGG	0.0	0.0	0.0	0.0	0.0	0.0	0.0
AACGTAGGTC	0.0	0.0	0.0	0.0	0.0	0.0	0.0
TACGTATGGT	0.0	0.0	0.0	0.0	0.0	0.0	0.0
TACGTAGGGA	0.0	0.0	0.0	0.0	0.0	0.0	0.0
TACGTATGGA	0.0	0.0	0.0	0.0	0.0	0.0	0.0
TACGTATGGA	0.0	0.0	0.0	0.0	0.0	0.0	0.0
TACGTATGGT	0.0	0.0	0.0	0.0	0.0	0.0	0.0
TACGTAGGTG	0.0	0.0	0.0	0.0	0.0	0.0	0.0
TACGTAGGGA	0.0	0.0	0.0	0.0	0.0	0.0	0.0
TACGTAGGGG	0.0	0.0	0.0	0.0	0.0	0.0	0.0
TACGTAGGGA	0.0	0.0	0.0	0.0	0.0	0.0	0.0
TACGTATGGA	0.0	0.0	0.0	0.0	0.0	0.0	0.0
TACGTAGGTG	0.0	0.0	0.0	0.0	0.0	0.0	0.0
TACGTATGGA	0.0	0.0	0.0	0.0	0.0	0.0	0.0
AACGTAGGGT	0.0	0.0	0.0	0.0	0.0	0.0	0.0
TACGTAGGTG	0.0	0.0	0.0	0.0	0.0	0.0	0.0
TACGTAGGGG	0.0	0.0	0.0	0.0	0.0	0.0	0.0
AACGTAGGTC	0.0	0.0	0.0	0.0	0.0	0.0	0.0
TGCGTAGGTG	0.0	0.0	0.0	0.0	0.0	0.0	0.0
TACGTAGGTG	0.0	0.0	0.0	0.0	0.0	0.0	0.0
TACGTAGGTG	0.0	0.0	0.0	0.0	0.0	0.0	0.0
TACGGAGGGT	0.0	0.0	0.0	0.0	0.0	0.0	0.0
TACGTAGGTG	0.0	0.0	0.0	0.0	0.0	0.0	0.0
TACGTAGGTG	0.0	0.0	0.0	0.0	0.0	0.0	0.0
TACGAAGGTC	0.0	0.0	0.0	0.0	0.0	0.0	0.0
TACGGAGGGT	0.0	0.0	0.0	0.0	0.0	0.0	0.0
TACGGAGGGT	0.0	0.0	0.0	0.0	0.0	0.0	0.0
TACGGAAGGT	0.0	0.0	0.0	0.0	0.0	0.0	0.0
TACGGAAGAT	0.0	0.0	0.0	0.0	0.0	0.0	0.0
TACGTAGGGG	0.0	0.0	0.0	0.0	0.0	0.0	0.0
TACGTATGGT	0.0	0.0	0.0	0.0	0.0	0.0	0.0
TACGTAAGGG	0.0	0.0	0.0	0.0	0.0	0.0	0.0
TACGTAGGGG	0.0	0.0	0.0	0.0	0.0	0.0	0.0
TACGTAGGTG	0.0	0.0	0.0	0.0	0.0	0.0	0.0
TACGGAGGGT	0.0	0.0	0.0	0.0	0.0	0.0	0.0
TACGTAGGTG	0.0	0.0	0.0	0.0	0.0	0.0	0.0
TACGTAGGGG	0.0	0.0	0.0	0.0	0.0	0.0	0.0
TACGTAGGGA	0.0	0.0	0.0	0.0	0.0	0.0	0.0
TACGTAGGTG	0.0	0.0	0.0	0.0	0.0	0.0	0.0
TACGTATGGT	0.0	0.0	0.0	0.0	0.0	0.0	0.0
TACGGAGGGT	0.0	0.0	0.0	0.0	0.0	0.0	0.0
TACGGAGGAT	0.0	0.0	0.0	0.0	0.0	0.0	0.0
TACGGAGGGT	0.0	0.0	0.0	0.0	0.0	0.0	0.0
TACGTAGGTG	0.0	0.0	0.0	0.0	0.0	0.0	0.0
TACGTATGGA	0.0	0.0	0.0	0.0	0.0	0.0	0.0
TACGGAGGAT	0.0	0.0	0.0	0.0	0.0	0.0	0.0
TACGTAGGGA	0.0	0.0	0.0	0.0	0.0	0.0	0.0

TACGGAGGGT	0.0	0.0	0.0	0.0	0.0	0.0	0.0
TACGTAAGGG	0.0	0.0	0.0	0.0	0.0	0.0	0.0
AACGTAGGGT	0.0	0.0	0.0	0.0	0.0	0.0	0.0
TACGTAGGTG	0.0	0.0	0.0	0.0	0.0	0.0	0.0
TACGTAGGGA	0.0	0.0	0.0	0.0	0.0	0.0	0.0
TACGTAGGGG	0.0	0.0	0.0	0.0	0.0	0.0	0.0
TACGTAGGGG	0.0	0.0	0.0	0.0	0.0	0.0	0.0
TACGTAGGGG	0.0	0.0	0.0	0.0	0.0	0.0	0.0
TACGGAGGGT	0.0	0.0	0.0	0.0	0.0	0.0	0.0
TACGTAGGTG	0.0	0.0	0.0	0.0	0.0	0.0	0.0
TACGTAGGGG	0.0	0.0	0.0	0.0	0.0	0.0	0.0
TACGTAGGTG	0.0	0.0	0.0	0.0	0.0	0.0	0.0
TACGTAGGTG	0.0	0.0	0.0	0.0	0.0	0.0	0.0
TACGGAGGAC	0.0	0.0	0.0	0.0	0.0	0.0	0.0
TACGTATGGT	0.0	0.0	0.0	0.0	0.0	0.0	0.0
TACGTAGGTG	0.0	0.0	0.0	0.0	0.0	0.0	0.0
TACGTAGGGA	0.0	0.0	0.0	0.0	0.0	0.0	0.0
TACGTAGGGG	0.0	0.0	0.0	0.0	0.0	0.0	0.0
TACGTAGGGG	0.0	0.0	0.0	0.0	0.0	0.0	0.0
TACGTATGTC	0.0	0.0	0.0	0.0	0.0	0.0	0.0
TACGTAGGGG	0.0	0.0	0.0	0.0	0.0	0.0	0.0
TACGTATGGT	0.0	0.0	0.0	0.0	0.0	0.0	0.0
TACGTAGGTG	0.0	0.0	0.0	0.0	0.0	0.0	0.0
CACGTAGGGG	0.0	0.0	0.0	0.0	0.0	0.0	0.0
TACGTAGGTG	0.0	0.0	0.0	0.0	0.0	0.0	0.0
TACGTAGGTG	0.0	0.0	0.0	0.0	0.0	0.0	0.0
AACGTAGGTC	0.0	0.0	0.0	0.0	0.0	0.0	0.0
TACGTAGGGG	0.0	0.0	0.0	0.0	0.0	0.0	0.0
TACGTAGGTG	0.0	0.0	0.0	0.0	0.0	0.0	0.0
TACGTATGGT	0.0	0.0	0.0	0.0	0.0	0.0	0.0
TACGTAGGTG	0.0	0.0	0.0	0.0	0.0	0.0	0.0
TACGTAGGTG	0.0	0.0	0.0	0.0	0.0	0.0	0.0
TACGTAGGTG	0.0	0.0	0.0	0.0	0.0	0.0	0.0
TACGGAGGAT	0.0	0.0	0.0	0.0	0.0	0.0	0.0
TACGTAGGTG	0.0	0.0	0.0	0.0	0.0	0.0	0.0
TACGTAGGGG	0.0	0.0	0.0	0.0	0.0	0.0	0.0
TACGTAGGTG	0.0	0.0	0.0	0.0	0.0	0.0	0.0
TACGTAGGTG	0.0	0.0	0.0	0.0	0.0	0.0	0.0
TACGTAGGTA	0.0	0.0	0.0	0.0	0.0	0.0	0.0
TACGTAGGTG	0.0	0.0	0.0	0.0	0.0	0.0	0.0
TACGTAGGGG	0.0	0.0	0.0	0.0	0.0	0.0	0.0
TACGTAGGGG	0.0	0.0	0.0	0.0	0.0	0.0	0.0
TACGGAGGGT	0.0	0.0	0.0	0.0	0.0	0.0	0.0
TACGTAGGGG	0.0	0.0	0.0	0.0	0.0	0.0	0.0
TACGTAGGGG	0.0	0.0	0.0	0.0	0.0	0.0	0.0
TACGGAGGAT	0.0	0.0	0.0	0.0	0.0	0.0	0.0
TACGGAGGGT	0.0	0.0	0.0	0.0	0.0	0.0	0.0

TACGTAGGGG	0.0	0.0	0.0	0.0	0.0	0.0	0.0
TACGTATGGT	0.0	0.0	0.0	0.0	0.0	0.0	0.0
TACGTAGGTG	0.0	0.0	0.0	0.0	0.0	0.0	0.0
TACGTATGGG	0.0	0.0	0.0	0.0	0.0	0.0	0.0
TACGGAAGGT	0.0	0.0	0.0	0.0	0.0	0.0	0.0
AACGTAGGTG	0.0	0.0	0.0	0.0	0.0	0.0	0.0
TACGTAGGAT	0.0	0.0	0.0	0.0	0.0	0.0	0.0
TACGTAGGGG	0.0	0.0	0.0	0.0	0.0	0.0	0.0
TACGTAGGTG	0.0	0.0	0.0	0.0	0.0	0.0	0.0
TACGTAGGGA	0.0	0.0	0.0	0.0	0.0	0.0	0.0
TACGTAGGTG	0.0	0.0	0.0	0.0	0.0	0.0	0.0
TACGTAGGTG	0.0	0.0	0.0	0.0	0.0	0.0	0.0
TACGTAGGGA	0.0	0.0	0.0	0.0	0.0	0.0	0.0
TACGTAGGTG	0.0	0.0	0.0	0.0	0.0	0.0	0.0
TACGTAGGGG	0.0	0.0	0.0	0.0	0.0	0.0	0.0
TACGTAGGGG	0.0	0.0	0.0	0.0	0.0	0.0	0.0
TACGTATGGT	0.0	0.0	0.0	0.0	0.0	0.0	0.0
TACGTAGGGA	0.0	0.0	0.0	0.0	0.0	0.0	0.0
TACGTAGGGA	0.0	0.0	0.0	0.0	0.0	0.0	0.0
TACGTAGGAT	0.0	0.0	0.0	0.0	0.0	0.0	0.0
TACGTAGGGG	0.0	0.0	0.0	0.0	0.0	0.0	0.0
TACGTAGGGG	0.0	0.0	0.0	0.0	0.0	0.0	0.0
TACGGAGGAT	0.0	0.0	0.0	0.0	0.0	0.0	0.0
TACGTATGGA	0.0	0.0	0.0	0.0	0.0	0.0	0.0
AACGTAGGTC	0.0	0.0	0.0	0.0	0.0	0.0	0.0
AACGTAGGTC	0.0	0.0	0.0	0.0	0.0	0.0	0.0
TACGGAAGAT	0.0	0.0	0.0	0.0	0.0	0.0	0.0
TACGTATGGT	0.0	0.0	0.0	0.0	0.0	0.0	0.0
TACGTAGGTG	0.0	0.0	0.0	0.0	0.0	0.0	0.0
TACGGAAGGT	0.0	0.0	0.0	0.0	0.0	0.0	0.0
TACGTAGGGG	0.0	0.0	0.0	0.0	0.0	0.0	0.0
TACGTAGGGG	0.0	0.0	0.0	0.0	0.0	0.0	0.0
TACGTAGGGA	0.0	0.0	0.0	0.0	0.0	0.0	0.0
TACGTAGGGG	0.0	0.0	0.0	0.0	0.0	0.0	0.0
TACGTAGGTG	0.0	0.0	0.0	0.0	0.0	0.0	0.0
TACGGAGGAT	0.0	0.0	0.0	0.0	0.0	0.0	0.0
TACGTAGGGG	0.0	0.0	0.0	0.0	0.0	0.0	0.0
TACGTATGGT	0.0	0.0	0.0	0.0	0.0	0.0	0.0
TCCTTCTTCTC	0.0	0.0	0.0	0.0	0.0	0.0	0.0
TACGTATGGT	0.0	0.0	0.0	0.0	0.0	0.0	0.0
TACGTAGGGG	0.0	0.0	0.0	0.0	0.0	0.0	0.0
TACGTAGGGA	0.0	0.0	0.0	0.0	0.0	0.0	0.0
TACGGAAGGT	0.0	0.0	0.0	0.0	0.0	0.0	0.0
TACGTAGGGA	0.0	0.0	0.0	0.0	0.0	0.0	0.0
TACGGAAGGT	0.0	0.0	0.0	0.0	0.0	0.0	0.0
TACGTATGGT	0.0	0.0	0.0	0.0	0.0	0.0	0.0
TACGTAGGGG	0.0	0.0	0.0	0.0	0.0	0.0	0.0

TACGTATGGT	0.0	0.0	0.0	0.0	0.0	0.0	0.0
TACGTAGGGA	0.0	0.0	0.0	0.0	0.0	0.0	0.0
TACGTAGGGG	0.0	0.0	0.0	0.0	0.0	0.0	0.0
TACGTAGGTG	0.0	0.0	0.0	0.0	0.0	0.0	0.0
TACGTAGGTG	0.0	0.0	0.0	0.0	0.0	0.0	0.0
TACGTAGGTG	0.0	0.0	0.0	0.0	0.0	0.0	0.0
TACGTAGGGG	0.0	0.0	0.0	0.0	0.0	0.0	0.0
TACGTAGGGA	0.0	0.0	0.0	0.0	0.0	0.0	0.0
TACGTAGGTG	0.0	0.0	0.0	0.0	0.0	0.0	0.0
AACGTAGGGT	0.0	0.0	0.0	0.0	0.0	0.0	0.0
TACGTAGGGG	0.0	0.0	0.0	0.0	0.0	0.0	0.0
TACGTAGGGG	0.0	0.0	0.0	0.0	0.0	0.0	0.0
TACGGAGGAT	0.0	0.0	0.0	0.0	0.0	0.0	0.0
TACGTAGGGG	0.0	0.0	0.0	0.0	0.0	0.0	0.0
TACGTAGGTG	0.0	0.0	0.0	0.0	0.0	0.0	0.0
TACGTAGGGG	0.0	0.0	0.0	0.0	0.0	0.0	0.0
TACGTATGGT	0.0	0.0	0.0	0.0	0.0	0.0	0.0
TACGTAGGTG	0.0	0.0	0.0	0.0	0.0	0.0	0.0
TACGTAGGGG	0.0	0.0	0.0	0.0	0.0	0.0	0.0
TACGTAGGGT	0.0	0.0	0.0	0.0	0.0	0.0	0.0
TACGTAGGGA	0.0	0.0	0.0	0.0	0.0	0.0	0.0
TACGGAGGG	0.0	0.0	0.0	0.0	0.0	0.0	0.0
TACGTAGGGG	0.0	0.0	0.0	0.0	0.0	0.0	0.0
TACGTAGGGT	0.0	0.0	0.0	0.0	0.0	0.0	0.0
TACGTAGGGT	0.0	0.0	0.0	0.0	0.0	0.0	0.0
TACGTAGGTG	0.0	0.0	0.0	0.0	0.0	0.0	0.0
TACGTATGGT	0.0	0.0	0.0	0.0	0.0	0.0	0.0
TACGTAGGGA	0.0	0.0	0.0	0.0	0.0	0.0	0.0
TACGTAGGGA	0.0	0.0	0.0	0.0	0.0	0.0	0.0
TACGTAGGGG	0.0	0.0	0.0	0.0	0.0	0.0	0.0
TACGTAGGGG	0.0	0.0	0.0	0.0	0.0	0.0	0.0
TACGTAGGGG	0.0	0.0	0.0	0.0	0.0	0.0	0.0
TACGGAGGAT	0.0	0.0	0.0	0.0	0.0	0.0	0.0
TACGTAGGGG	0.0	0.0	0.0	0.0	0.0	0.0	0.0
TACGTAGGGG	0.0	0.0	0.0	0.0	0.0	0.0	0.0
TACGGAGGAT	0.0	0.0	0.0	0.0	0.0	0.0	0.0
TACGTAGGGG	0.0	0.0	0.0	0.0	0.0	0.0	0.0
TACGTATGGA	0.0	0.0	0.0	0.0	0.0	0.0	0.0
TACGTAGGGG	0.0	0.0	0.0	0.0	0.0	0.0	0.0
TACGTAGGGG	0.0	0.0	0.0	0.0	0.0	0.0	0.0
TACGGAGGAT	0.0	0.0	0.0	0.0	0.0	0.0	0.0
TACGTAGAGG	0.0	0.0	0.0	0.0	0.0	0.0	0.0
TACGTATGGT	0.0	0.0	0.0	0.0	0.0	0.0	0.0
TACGGAGGGT	0.0	0.0	0.0	0.0	0.0	0.0	0.0
TACGTATGGT	0.0	0.0	0.0	0.0	0.0	0.0	0.0
TACGTAGGGG	0.0	0.0	0.0	0.0	0.0	0.0	0.0
TACGTATGGT	0.0	0.0	0.0	0.0	0.0	0.0	0.0

TACGTAGGTG	0.0	0.0	0.0	0.0	0.0	0.0	0.0
TACGTAGGGG	0.0	0.0	0.0	0.0	0.0	0.0	0.0
TACGTAAGTG	0.0	0.0	0.0	0.0	0.0	0.0	0.0
TACGTAGGGG	0.0	0.0	0.0	0.0	0.0	0.0	0.0
AACGTAGGTC	0.0	0.0	0.0	0.0	0.0	0.0	0.0
TACGTAGGTG	0.0	0.0	0.0	0.0	0.0	0.0	0.0
AACGTAGGTG	0.0	0.0	0.0	0.0	0.0	0.0	0.0
TACGTAGGGG	0.0	0.0	0.0	0.0	0.0	0.0	0.0
TACGTAGGGG	0.0	0.0	0.0	0.0	0.0	0.0	0.0
TACGTAGGTG	0.0	0.0	0.0	0.0	0.0	0.0	0.0
TACGTAGGTG	0.0	0.0	0.0	0.0	0.0	0.0	0.0
TACGGAGGAT	0.0	0.0	0.0	0.0	0.0	0.0	0.0
TACGTAGGGG	0.0	0.0	0.0	0.0	0.0	0.0	0.0
TACGTAGGAT	0.0	0.0	0.0	0.0	0.0	0.0	0.0
TACGTAGGGG	0.0	0.0	0.0	0.0	0.0	0.0	0.0
TACGTAGGTG	0.0	0.0	0.0	0.0	0.0	0.0	0.0
TACGTAGGTG	0.0	0.0	0.0	0.0	0.0	0.0	0.0
TACGTAGGTG	0.0	0.0	0.0	0.0	0.0	0.0	0.0
TACGGAGGGT	0.0	0.0	0.0	0.0	0.0	0.0	0.0
TACGTAGGGG	0.0	0.0	0.0	0.0	0.0	0.0	0.0
TACGTAGGTG	0.0	0.0	0.0	0.0	0.0	0.0	0.0
TACGTAGGGA	0.0	0.0	0.0	0.0	0.0	0.0	0.0
TACGTAGGGG	0.0	0.0	0.0	0.0	0.0	0.0	0.0
TACGTAGGTG	0.0	0.0	0.0	0.0	0.0	0.0	0.0
TACGTAGGGG	0.0	0.0	0.0	0.0	0.0	0.0	0.0
TACGTAGGTG	0.0	0.0	0.0	0.0	0.0	0.0	0.0
TACGTAGGTG	0.0	0.0	0.0	0.0	0.0	0.0	0.0
TACGTAGGTG	0.0	0.0	0.0	0.0	0.0	0.0	0.0
TACGTAGGGG	0.0	0.0	0.0	0.0	0.0	0.0	0.0
AACGTAGGTG	0.0	0.0	0.0	0.0	0.0	0.0	0.0
TACGTAGGTG	0.0	0.0	0.0	0.0	0.0	0.0	0.0
TACGTAGGTG	0.0	0.0	0.0	0.0	0.0	0.0	0.0
TACGTAGGGG	0.0	0.0	0.0	0.0	0.0	0.0	0.0
TACGTAGGGG	0.0	0.0	0.0	0.0	0.0	0.0	0.0
TACGCAGGGC	0.0	0.0	0.0	0.0	0.0	0.0	0.0
TACGTAGGGG	0.0	0.0	0.0	0.0	0.0	0.0	0.0
TACGTAGGGC	0.0	0.0	0.0	0.0	0.0	0.0	0.0
TACGTAGGTG	0.0	0.0	0.0	0.0	0.0	0.0	0.0
TACGTAGGTG	0.0	0.0	0.0	0.0	0.0	0.0	0.0
TACGTAGGGG	0.0	0.0	0.0	0.0	0.0	0.0	0.0
TACGTAGGGG	0.0	0.0	0.0	0.0	0.0	0.0	0.0
TACGTAGGGG	0.0	0.0	0.0	0.0	0.0	0.0	0.0
TACGTAGGTG	0.0	0.0	0.0	0.0	0.0	0.0	0.0
TACGTAGGGG	0.0	0.0	0.0	0.0	0.0	0.0	0.0
TACGGAGGGT	0.0	0.0	0.0	0.0	0.0	0.0	0.0
TACGTAGGGG	0.0	0.0	0.0	0.0	0.0	0.0	0.0
TACGGAGGAT	0.0	0.0	0.0	0.0	0.0	0.0	0.0

TACGTAGGGG	0.0	0.0	0.0	0.0	0.0	0.0	0.0
TACGTAGGGG	0.0	0.0	0.0	0.0	0.0	0.0	0.0
TACGTAGGGG	0.0	0.0	0.0	0.0	0.0	0.0	0.0
TACGTAGGGA	0.0	0.0	0.0	0.0	0.0	0.0	0.0
TACGTAGGGG	0.0	0.0	0.0	0.0	0.0	0.0	0.0
TACGGAGGGT	0.0	0.0	0.0	0.0	0.0	0.0	0.0
TACGGAGGGT	0.0	0.0	0.0	0.0	0.0	0.0	0.0
TACGGAGGAT	0.0	0.0	0.0	0.0	0.0	0.0	0.0
TACGTAGGTG	0.0	0.0	0.0	0.0	0.0	0.0	0.0
TACGTAGGTC	0.0	0.0	0.0	0.0	0.0	0.0	0.0
TACGTAGGTG	0.0	0.0	0.0	0.0	0.0	0.0	0.0
TACGTAGGGT	0.0	0.0	0.0	0.0	0.0	0.0	0.0
TACGTAGGGG	0.0	0.0	0.0	0.0	0.0	0.0	0.0
AGCGTAGGGT	0.0	0.0	0.0	0.0	0.0	0.0	0.0
TACGTAGGGG	0.0	0.0	0.0	0.0	0.0	0.0	0.0
TACGGAGGAT	0.0	0.0	0.0	0.0	0.0	0.0	0.0
TACGGAGGGT	0.0	0.0	0.0	0.0	0.0	0.0	0.0
TACGTAGGGG	0.0	0.0	0.0	0.0	0.0	0.0	0.0
TACGTAGGTG	0.0	0.0	0.0	0.0	0.0	0.0	0.0
TACGTAGGGG	0.0	0.0	0.0	0.0	0.0	0.0	0.0
TACGTAGGGG	0.0	0.0	0.0	0.0	0.0	0.0	0.0
TACGTATGTT	0.0	0.0	0.0	0.0	0.0	0.0	0.0
TACGTATGGT	0.0	0.0	0.0	0.0	0.0	0.0	0.0
TACGTAGGTG	0.0	0.0	0.0	0.0	0.0	0.0	0.0
TACGTAGGTC	0.0	0.0	0.0	0.0	0.0	0.0	0.0
TACGTAGGTG	0.0	0.0	0.0	0.0	0.0	0.0	0.0
TACGGAGGAT	0.0	0.0	0.0	0.0	0.0	0.0	0.0
TACGTATGGA	0.0	0.0	0.0	0.0	0.0	0.0	0.0
TACGTAGGGG	0.0	0.0	0.0	0.0	0.0	0.0	0.0
TACGGAGGAT	0.0	0.0	0.0	0.0	0.0	0.0	0.0
TACGTATGGA	0.0	0.0	0.0	0.0	0.0	0.0	0.0
TACGTAGGGG	0.0	0.0	0.0	0.0	0.0	0.0	0.0
TACGTAGGTG	0.0	0.0	0.0	0.0	0.0	0.0	0.0
TACGTAGGGG	0.0	0.0	0.0	0.0	0.0	0.0	0.0
AACGTAGGTC	0.0	0.0	0.0	0.0	0.0	0.0	0.0
TACGTAGGGG	0.0	0.0	0.0	0.0	0.0	0.0	0.0
TACGGAGGTG	0.0	0.0	0.0	0.0	0.0	0.0	0.0
TACGTAGGGG	0.0	0.0	0.0	0.0	0.0	0.0	0.0
TACGTAGGGA	0.0	0.0	0.0	0.0	0.0	0.0	0.0
TACGTAGGGG	0.0	0.0	0.0	0.0	0.0	0.0	0.0
TACGTAGGGG	0.0	0.0	0.0	0.0	0.0	0.0	0.0
TACGTAGGGG	0.0	0.0	0.0	0.0	0.0	0.0	0.0
TACGTAGGGT	0.0	0.0	0.0	0.0	0.0	0.0	0.0
TACGTAGGGG	0.0	0.0	0.0	0.0	0.0	0.0	0.0
TACGTAGGGG	0.0	0.0	0.0	0.0	0.0	0.0	0.0
TCCTTCTTTTT	0.0	0.0	0.0	0.0	0.0	0.0	0.0
TACGTATGGT	0.0	0.0	0.0	0.0	0.0	0.0	0.0

TACGGAGGG	0.0	0.0	0.0	0.0	0.0	0.0	0.0
TACGGAGGGT	0.0	0.0	0.0	0.0	0.0	0.0	0.0
TACGTATGGT	0.0	0.0	0.0	0.0	0.0	0.0	0.0
TACGTAGGGA	0.0	0.0	0.0	0.0	0.0	0.0	0.0
TACGTAGGTG	0.0	0.0	0.0	0.0	0.0	0.0	0.0
TACGGAGGGT	0.0	0.0	0.0	0.0	0.0	0.0	0.0
AACGTAGGTC	0.0	0.0	0.0	0.0	0.0	0.0	0.0
AACGTAGGTG	0.0	0.0	0.0	0.0	0.0	0.0	0.0
TACGTAGGTG	0.0	0.0	0.0	0.0	0.0	0.0	0.0
TACGTAGGGG	0.0	0.0	0.0	0.0	0.0	0.0	0.0
TACGTAGGTG	0.0	0.0	0.0	0.0	0.0	0.0	0.0
TACGTAGGGG	0.0	0.0	0.0	0.0	0.0	0.0	0.0
TACGTAGGGA	0.0	0.0	0.0	0.0	0.0	0.0	0.0
TACGGAGGG	0.0	0.0	0.0	0.0	0.0	0.0	0.0
TACGTAGGTG	0.0	0.0	0.0	0.0	0.0	0.0	0.0
TACGTAGGTG	0.0	0.0	0.0	0.0	0.0	0.0	0.0
TACGTAGGTG	0.0	0.0	0.0	0.0	0.0	0.0	0.0
TACGTAGGTG	0.0	0.0	0.0	0.0	0.0	0.0	0.0
TACGTAGGGG	0.0	0.0	0.0	0.0	0.0	0.0	0.0
TACGTAGGTG	0.0	0.0	0.0	0.0	0.0	0.0	0.0
TACGTAGGGG	0.0	0.0	0.0	0.0	0.0	0.0	0.0
TACGTAGGGC	0.0	0.0	0.0	0.0	0.0	0.0	0.0
TACGTAGGTG	0.0	0.0	0.0	0.0	0.0	0.0	0.0
TGCGTATGGT	0.0	0.0	0.0	0.0	0.0	0.0	0.0
TACGTAGGGG	0.0	0.0	0.0	0.0	0.0	0.0	0.0
TACGTAGGTG	0.0	0.0	0.0	0.0	0.0	0.0	0.0
TACGTAGGGG	0.0	0.0	0.0	0.0	0.0	0.0	0.0
TACGTAGGGG	0.0	0.0	0.0	0.0	0.0	0.0	0.0
TACGTATGGA	0.0	0.0	0.0	0.0	0.0	0.0	0.0
TACGTAGGTC	0.0	0.0	0.0	0.0	0.0	0.0	0.0
TACGTATGGA	0.0	0.0	0.0	0.0	0.0	0.0	0.0
TACGTAGGGT	0.0	0.0	0.0	0.0	0.0	0.0	0.0
TACGGAGGGT	0.0	0.0	0.0	0.0	0.0	0.0	0.0
TACGTATGGA	0.0	0.0	0.0	0.0	0.0	0.0	0.0
TACGTAGGGG	0.0	0.0	0.0	0.0	0.0	0.0	0.0
AACGTAGGGT	0.0	0.0	0.0	0.0	0.0	0.0	0.0
TACGTAGGCC	0.0	0.0	0.0	0.0	0.0	0.0	0.0
TACGTAGGGG	0.0	0.0	0.0	0.0	0.0	0.0	0.0
TACGGAGGAT	0.0	0.0	0.0	0.0	0.0	0.0	0.0
TACGTAGGTG	0.0	0.0	0.0	0.0	0.0	0.0	0.0
TACGTAGGTG	0.0	0.0	0.0	0.0	0.0	0.0	0.0
TACGTAGGGG	0.0	0.0	0.0	0.0	0.0	0.0	0.0
TACGTAGGGG	0.0	0.0	0.0	0.0	0.0	0.0	0.0
TACGTAGGGG	0.0	0.0	0.0	0.0	0.0	0.0	0.0
TACGTAGGGG	0.0	0.0	0.0	0.0	0.0	0.0	0.0
TACGTAGGGG	0.0	0.0	0.0	0.0	0.0	0.0	0.0
TACTTCCTTTC	0.0	0.0	0.0	0.0	0.0	0.0	0.0

TCCTTCCTTTC	0.0	0.0	0.0	0.0	0.0	0.0	0.0
TACGGAAGGT	0.0	0.0	0.0	0.0	0.0	0.0	0.0
TACGTAGGGG	0.0	0.0	0.0	0.0	0.0	0.0	0.0
TACGTAGGGA	0.0	0.0	0.0	0.0	0.0	0.0	0.0
TACGGAGGAT	0.0	0.0	0.0	0.0	0.0	0.0	0.0
AACGTAGGTC	0.0	0.0	0.0	0.0	0.0	0.0	0.0
TACGTAGGGG	0.0	0.0	0.0	0.0	0.0	0.0	0.0
TACGTAGGTG	0.0	0.0	0.0	0.0	0.0	0.0	0.0
TACGTAGGGG	0.0	0.0	0.0	0.0	0.0	0.0	0.0
TACGTAGGGG	0.0	0.0	0.0	0.0	0.0	0.0	0.0
TACGTAGGTG	0.0	0.0	0.0	0.0	0.0	0.0	0.0
TACGTAGGTG	0.0	0.0	0.0	0.0	0.0	0.0	0.0
TACGTAGGTG	0.0	0.0	0.0	0.0	0.0	0.0	0.0
TACGTAGGGT	0.0	0.0	0.0	0.0	0.0	0.0	0.0
TACGTAGGGT	0.0	0.0	0.0	0.0	0.0	0.0	0.0
TACGTAGGTG	0.0	0.0	0.0	0.0	0.0	0.0	0.0
TACGTAGGTG	0.0	0.0	0.0	0.0	0.0	0.0	0.0
TACGTAGGTG	0.0	0.0	0.0	0.0	0.0	0.0	0.0
TACGTAGGTG	0.0	0.0	0.0	0.0	0.0	0.0	0.0
TACGTAGGGT	0.0	0.0	0.0	0.0	0.0	0.0	0.0
TACGTAGGGG	0.0	0.0	0.0	0.0	0.0	0.0	0.0
TACGTAGGGG	0.0	0.0	0.0	0.0	0.0	0.0	0.0
TACGTAGGGG	0.0	0.0	0.0	0.0	0.0	0.0	0.0
TACGTAGGGA	0.0	0.0	0.0	0.0	0.0	0.0	0.0
TACGGAGGAT	0.0	0.0	0.0	0.0	0.0	0.0	0.0
TACGTATGGT	0.0	0.0	0.0	0.0	0.0	0.0	0.0
TACGGAGGAT	0.0	0.0	0.0	0.0	0.0	0.0	0.0
TACGTAGGGG	0.0	0.0	0.0	0.0	0.0	0.0	0.0
TACGGAGGAT	0.0	0.0	0.0	0.0	0.0	0.0	0.0
TACGGAGGAT	0.0	0.0	0.0	0.0	0.0	0.0	0.0
TACGTAGGAG	0.0	0.0	0.0	0.0	0.0	0.0	0.0
TACGTAGGTG	0.0	0.0	0.0	0.0	0.0	0.0	0.0
TACGTAGGGG	0.0	0.0	0.0	0.0	0.0	0.0	0.0
TACGGAGGGT	0.0	0.0	0.0	0.0	0.0	0.0	0.0
TACGTAGGGG	0.0	0.0	0.0	0.0	0.0	0.0	0.0
TACGTAGGTG	0.0	0.0	0.0	0.0	0.0	0.0	0.0
TACGTATGGT	0.0	0.0	0.0	0.0	0.0	0.0	0.0
TACGTAGGGG	0.0	0.0	0.0	0.0	0.0	0.0	0.0
TACGTAGGGG	0.0	0.0	0.0	0.0	0.0	0.0	0.0
AACGTAGGGT	0.0	0.0	0.0	0.0	0.0	0.0	0.0
TACGGAGGGT	0.0	0.0	0.0	0.0	0.0	0.0	0.0
TACGTAGGGG	0.0	0.0	0.0	0.0	0.0	0.0	0.0
TACGTAGATG	0.0	0.0	0.0	0.0	0.0	0.0	0.0
TACGTAGGGA	0.0	0.0	0.0	0.0	0.0	0.0	0.0
TACGTAGGGG	0.0	0.0	0.0	0.0	0.0	0.0	0.0
TACGTAGGGA	0.0	0.0	0.0	0.0	0.0	0.0	0.0
TACGTAGGGG	0.0	0.0	0.0	0.0	0.0	0.0	0.0

TACGTATGGA	0.0	0.0	0.0	0.0	0.0	0.0	0.0
TACGTATGGG	0.0	0.0	0.0	0.0	0.0	0.0	0.0
TACGTAGGGG	0.0	0.0	0.0	0.0	0.0	0.0	0.0
TACGTAGGGG	0.0	0.0	0.0	0.0	0.0	0.0	0.0
TACGTAGGGA	0.0	0.0	0.0	0.0	0.0	0.0	0.0
TACGGAGGAT	0.0	0.0	0.0	0.0	0.0	0.0	0.0
TACGTATGGA	0.0	0.0	0.0	0.0	0.0	0.0	0.0
TACGTATGGA	0.0	0.0	0.0	0.0	0.0	0.0	0.0
TACGTATGGT	0.0	0.0	0.0	0.0	0.0	0.0	0.0
TACGTATGGT	0.0	0.0	0.0	0.0	0.0	0.0	0.0
TACGTAGGGA	0.0	0.0	0.0	0.0	0.0	0.0	0.0
AACGTAGGTG	0.0	0.0	0.0	0.0	0.0	0.0	0.0
TACGTAGGTG	0.0	0.0	0.0	0.0	0.0	0.0	0.0
AACGTAGGGT	0.0	0.0	0.0	0.0	0.0	0.0	0.0
TACGTAGGTG	0.0	0.0	0.0	0.0	0.0	0.0	0.0
TACGTAGGGG	0.0	0.0	0.0	0.0	0.0	0.0	0.0
TACGTAGGTG	0.0	0.0	0.0	0.0	0.0	0.0	0.0
TACGTATGGA	0.0	0.0	0.0	0.0	0.0	0.0	0.0
TACGTAGGGG	0.0	0.0	0.0	0.0	0.0	0.0	0.0
TACAGAGGGT	0.0	0.0	0.0	0.0	0.0	0.0	0.0
TACGTATGGT	0.0	0.0	0.0	0.0	0.0	0.0	0.0
TACAGAGGGT	0.0	0.0	0.0	0.0	0.0	0.0	0.0
TACGTAGGTG	0.0	0.0	0.0	0.0	0.0	0.0	0.0
TACGTAGGGG	0.0	0.0	0.0	0.0	0.0	0.0	0.0
TACGTAGGGG	0.0	0.0	0.0	0.0	0.0	0.0	0.0
TACGGAGGGT	0.0	0.0	0.0	0.0	0.0	0.0	0.0
TACGTAGGTG	0.0	0.0	0.0	0.0	0.0	0.0	0.0
TACGTAGGTG	0.0	0.0	0.0	0.0	0.0	0.0	0.0
TACGTAGGGG	0.0	0.0	0.0	0.0	0.0	0.0	0.0
TACGGAGGAT	0.0	0.0	0.0	0.0	0.0	0.0	0.0
TACGTTGGGG	0.0	0.0	0.0	0.0	0.0	0.0	0.0
TACGGAGGGG	0.0	0.0	0.0	0.0	0.0	0.0	0.0
TACGTATGGA	0.0	0.0	0.0	0.0	0.0	0.0	0.0
TACGTATGGG	0.0	0.0	0.0	0.0	0.0	0.0	0.0
TACGGAAGGT	0.0	0.0	0.0	0.0	0.0	0.0	0.0
TACGGAAGGT	0.0	0.0	0.0	0.0	0.0	0.0	0.0
TACGGGGGGT	0.0	0.0	0.0	0.0	0.0	0.0	0.0
TACGTAGGGG	0.0	0.0	0.0	0.0	0.0	0.0	0.0
TACGGAAGGT	0.0	0.0	0.0	0.0	0.0	0.0	0.0
TACGTAGGTG	0.0	0.0	0.0	0.0	0.0	0.0	0.0
TACGTAGGGG	0.0	0.0	0.0	0.0	0.0	0.0	0.0
TACGTAGGTG	0.0	0.0	0.0	0.0	0.0	0.0	0.0
TACGTAGGGG	0.0	0.0	0.0	0.0	0.0	0.0	0.0
TACGTAGGTG	0.0	0.0	0.0	0.0	0.0	0.0	0.0
TACGTAGGGG	0.0	0.0	0.0	0.0	0.0	0.0	0.0
TACGTAGGGG	0.0	0.0	0.0	0.0	0.0	0.0	0.0
TACGGAGGAT	0.0	0.0	0.0	0.0	0.0	0.0	0.0

TACGTATGGG	0.0	0.0	0.0	0.0	0.0	0.0	0.0
TACGTAGGGA	0.0	0.0	0.0	0.0	0.0	0.0	0.0
TACGTAGGGA	0.0	0.0	0.0	0.0	0.0	0.0	0.0
TACGTAGGTG	0.0	0.0	0.0	0.0	0.0	0.0	0.0
TACGTAGGGG	0.0	0.0	0.0	0.0	0.0	0.0	0.0
TACGTAGGTG	0.0	0.0	0.0	0.0	0.0	0.0	0.0
AACGTAGGGT	0.0	0.0	0.0	0.0	0.0	0.0	0.0
TACGTAGGGG	0.0	0.0	0.0	0.0	0.0	0.0	0.0
TACTTCTTCTC	0.0	0.0	0.0	0.0	0.0	0.0	0.0
TACGTAGGGC	0.0	0.0	0.0	0.0	0.0	0.0	0.0
TACGTAGGTC	0.0	0.0	0.0	0.0	0.0	0.0	0.0
TACGTAGGGG	0.0	0.0	0.0	0.0	0.0	0.0	0.0
TACGTATGGA	0.0	0.0	0.0	0.0	0.0	0.0	0.0
TACGTAGGGG	0.0	0.0	0.0	0.0	0.0	0.0	0.0
TACGTAGGGG	0.0	0.0	0.0	0.0	0.0	0.0	0.0
TACGTAGGGG	0.0	0.0	0.0	0.0	0.0	0.0	0.0
TACGGAGGAT	0.0	0.0	0.0	0.0	0.0	0.0	0.0
TACGTAGGTG	0.0	0.0	0.0	0.0	0.0	0.0	0.0
TACGTAGGGG	0.0	0.0	0.0	0.0	0.0	0.0	0.0
TACGTATGGT	0.0	0.0	0.0	0.0	0.0	0.0	0.0
TACGTAGGTG	0.0	0.0	0.0	0.0	0.0	0.0	0.0
TACGTAGGGA	0.0	0.0	0.0	0.0	0.0	0.0	0.0
CACGTAGGTG	0.0	0.0	0.0	0.0	0.0	0.0	0.0
TACGTAGGTG	0.0	0.0	0.0	0.0	0.0	0.0	0.0
TACGTATGGA	0.0	0.0	0.0	0.0	0.0	0.0	0.0
TACGTGTGGA	0.0	0.0	0.0	0.0	0.0	0.0	0.0
TACGTAGGGG	0.0	0.0	0.0	0.0	0.0	0.0	0.0
TACGTAGGGG	0.0	0.0	0.0	0.0	0.0	0.0	0.0
TACGTAGGGG	0.0	0.0	0.0	0.0	0.0	0.0	0.0
TACGTATGGG	0.0	0.0	0.0	0.0	0.0	0.0	0.0
TACGTAGGGG	0.0	0.0	0.0	0.0	0.0	0.0	0.0
TACGGAGGGT	0.0	0.0	0.0	0.0	0.0	0.0	0.0
TACGGAGGAT	0.0	0.0	0.0	0.0	0.0	0.0	0.0
TACGTAGGGG	0.0	0.0	0.0	0.0	0.0	0.0	0.0
TACGTAGGGG	0.0	0.0	0.0	0.0	0.0	0.0	0.0
TACGTAGGTG	0.0	0.0	0.0	0.0	0.0	0.0	0.0
TACGTATGGT	0.0	0.0	0.0	0.0	0.0	0.0	0.0
TACGTAGGGA	0.0	0.0	0.0	0.0	0.0	0.0	0.0
TACGTAGGGA	0.0	0.0	0.0	0.0	0.0	0.0	0.0
TACGTATGGA	0.0	0.0	0.0	0.0	0.0	0.0	0.0
TACGTAGGGG	0.0	0.0	0.0	0.0	0.0	0.0	0.0
TACGGAGGAT	0.0	0.0	0.0	0.0	0.0	0.0	0.0
TACGTAGGGG	0.0	0.0	0.0	0.0	0.0	0.0	0.0
TACGTAGGTG	0.0	0.0	0.0	0.0	0.0	0.0	0.0
TACGTAGGGG	0.0	0.0	0.0	0.0	0.0	0.0	0.0
TACGTATGGT	0.0	0.0	0.0	0.0	0.0	0.0	0.0
TACGTAGGTG	0.0	0.0	0.0	0.0	0.0	0.0	0.0

TACGTAGGGG	0.0	0.0	0.0	0.0	0.0	0.0	0.0
TACGGAGGGT	0.0	0.0	0.0	0.0	0.0	0.0	0.0
AACGTAGGGT	0.0	0.0	0.0	0.0	0.0	0.0	0.0
AACGTAGGTG	0.0	0.0	0.0	0.0	0.0	0.0	0.0
TACGTAGGGG	0.0	0.0	0.0	0.0	0.0	0.0	0.0
TACGTATGGT	0.0	0.0	0.0	0.0	0.0	0.0	0.0
TACGTAGGGG	0.0	0.0	0.0	0.0	0.0	0.0	0.0
TACGTAGGGT	0.0	0.0	0.0	0.0	0.0	0.0	0.0
TACGTAGGGT	0.0	0.0	0.0	0.0	0.0	0.0	0.0
TACGTAGGGA	0.0	0.0	0.0	0.0	0.0	0.0	0.0
TACGTAGGGG	0.0	0.0	0.0	0.0	0.0	0.0	0.0
TACGTAGGTG	0.0	0.0	0.0	0.0	0.0	0.0	0.0
TACGTAGGTG	0.0	0.0	0.0	0.0	0.0	0.0	0.0
TACGTAGGTG	0.0	0.0	0.0	0.0	0.0	0.0	0.0
TACGTAGGGA	0.0	0.0	0.0	0.0	0.0	0.0	0.0
TACGTGGGGA	0.0	0.0	0.0	0.0	0.0	0.0	0.0
TACGTAGGAA	0.0	0.0	0.0	0.0	0.0	0.0	0.0
TACGTAGGTG	0.0	0.0	0.0	0.0	0.0	0.0	0.0
TACGTAGGGG	0.0	0.0	0.0	0.0	0.0	0.0	0.0
TACGTAGGGA	0.0	0.0	0.0	0.0	0.0	0.0	0.0
TACGTAGGGA	0.0	0.0	0.0	0.0	0.0	0.0	0.0
TACGTAGGTG	0.0	0.0	0.0	0.0	0.0	0.0	0.0
TACGTAGGTG	0.0	0.0	0.0	0.0	0.0	0.0	0.0
TACGTAGGTG	0.0	0.0	0.0	0.0	0.0	0.0	0.0
TACGTAGGTG	0.0	0.0	0.0	0.0	0.0	0.0	0.0
AACGTAGGTC	0.0	0.0	0.0	0.0	0.0	0.0	0.0
TACGTAGGGG	0.0	0.0	0.0	0.0	0.0	0.0	0.0
TACGTAGGGA	0.0	0.0	0.0	0.0	0.0	0.0	0.0
TACGTAGGGG	0.0	0.0	0.0	0.0	0.0	0.0	0.0
TACGTAGGTG	0.0	0.0	0.0	0.0	0.0	0.0	0.0
TACGTAGGGA	0.0	0.0	0.0	0.0	0.0	0.0	0.0
TACGTAGGGG	0.0	0.0	0.0	0.0	0.0	0.0	0.0
TACGGAGGAT	0.0	0.0	0.0	0.0	0.0	0.0	0.0
TACGTAGGTG	0.0	0.0	0.0	0.0	0.0	0.0	0.0
TACGTAGGGG	0.0	0.0	0.0	0.0	0.0	0.0	0.0
TACGTATGGG	0.0	0.0	0.0	0.0	0.0	0.0	0.0
TACGTAGGGG	0.0	0.0	0.0	0.0	0.0	0.0	0.0
AACGTAGGTC	0.0	0.0	0.0	0.0	0.0	0.0	0.0
TACGTAGGGG	0.0	0.0	0.0	0.0	0.0	0.0	0.0
TACGTAGGGA	0.0	0.0	0.0	0.0	0.0	0.0	0.0
TACGTAGGTG	0.0	0.0	0.0	0.0	0.0	0.0	0.0
AACGTAGGTC	0.0	0.0	0.0	0.0	0.0	0.0	0.0
TACGTATGGT	0.0	0.0	0.0	0.0	0.0	0.0	0.0
TACGTAGGGG	0.0	0.0	0.0	0.0	0.0	0.0	0.0
TACGTAGGTG	0.0	0.0	0.0	0.0	0.0	0.0	0.0
TACGTAGGTG	0.0	0.0	0.0	0.0	0.0	0.0	0.0
TACGTAGGTG	0.0	0.0	0.0	0.0	0.0	0.0	0.0

AACGTAGGGA	0.0	0.0	0.0	0.0	0.0	0.0	0.0
TACGTAGGTG	0.0	0.0	0.0	0.0	0.0	0.0	0.0
TACGTAGGGA	0.0	0.0	0.0	0.0	0.0	0.0	0.0
TACGTAGGG	0.0	0.0	0.0	0.0	0.0	0.0	0.0
TACGTAGGGA	0.0	0.0	0.0	0.0	0.0	0.0	0.0
TACGTAGGTG	0.0	0.0	0.0	0.0	0.0	0.0	0.0
TACGGAGGAT	0.0	0.0	0.0	0.0	0.0	0.0	0.0
TACGTAGGTG	0.0	0.0	0.0	0.0	0.0	0.0	0.0
TACGTAGGGA	0.0	0.0	0.0	0.0	0.0	0.0	0.0
TACGTAGGG	0.0	0.0	0.0	0.0	0.0	0.0	0.0
TACGTAGGTG	0.0	0.0	0.0	0.0	0.0	0.0	0.0
TACGTACGGA	0.0	0.0	0.0	0.0	0.0	0.0	0.0
TACGTAGGG	0.0	0.0	0.0	0.0	0.0	0.0	0.0
TACGTATGGA	0.0	0.0	0.0	0.0	0.0	0.0	0.0
TACGTATGGT	0.0	0.0	0.0	0.0	0.0	0.0	0.0
TACGTAGGGA	0.0	0.0	0.0	0.0	0.0	0.0	0.0
TACGTAGGTG	0.0	0.0	0.0	0.0	0.0	0.0	0.0
TACGTATGGG	0.0	0.0	0.0	0.0	0.0	0.0	0.0
TACGTAGGGA	0.0	0.0	0.0	0.0	0.0	0.0	0.0
TACGTAGGGA	0.0	0.0	0.0	0.0	0.0	0.0	0.0
TACGTATGGA	0.0	0.0	0.0	0.0	0.0	0.0	0.0
TACGTAGGGA	0.0	0.0	0.0	0.0	0.0	0.0	0.0
TACGTAGGGA	0.0	0.0	0.0	0.0	0.0	0.0	0.0
TACGTAGGTG	0.0	0.0	0.0	0.0	0.0	0.0	0.0
TACGTAGGGA	0.0	0.0	0.0	0.0	0.0	0.0	0.0
TACGTAGGA	0.0	0.0	0.0	0.0	0.0	0.0	0.0
TACGTATGGA	0.0	0.0	0.0	0.0	0.0	0.0	0.0
TACGTAGGG	0.0	0.0	0.0	0.0	0.0	0.0	0.0
TACGTAGGG	0.0	0.0	0.0	0.0	0.0	0.0	0.0
TACGTAGGG	0.0	0.0	0.0	0.0	0.0	0.0	0.0
TACGTAGGGA	0.0	0.0	0.0	0.0	0.0	0.0	0.0
TACGGAGGGT	0.0	0.0	0.0	0.0	0.0	0.0	0.0
TACGTAGGTA	0.0	0.0	0.0	0.0	0.0	0.0	0.0
TACGTAGGGA	0.0	0.0	0.0	0.0	0.0	0.0	0.0
TACGTAGGGA	0.0	0.0	0.0	0.0	0.0	0.0	0.0
TACGTAGGTG	0.0	0.0	0.0	0.0	0.0	0.0	0.0
TACGTAGGGA	0.0	0.0	0.0	0.0	0.0	0.0	0.0
TACGTAGGG	0.0	0.0	0.0	0.0	0.0	0.0	0.0
TACGTAGGGA	0.0	0.0	0.0	0.0	0.0	0.0	0.0
AACGTAGGT	0.0	0.0	0.0	0.0	0.0	0.0	0.0
TACGTAGGG	0.0	0.0	0.0	0.0	0.0	0.0	0.0
TACGTAGGGA	0.0	0.0	0.0	0.0	0.0	0.0	0.0
TACGTAGGGA	0.0	0.0	0.0	0.0	0.0	0.0	0.0
TACGTAGGTG	0.0	0.0	0.0	0.0	0.0	0.0	0.0
TACGTAGGGT	0.0	0.0	0.0	0.0	0.0	0.0	0.0
TCCTTCTTTTT	0.0	0.0	0.0	0.0	0.0	0.0	0.0
TACGTAGGTC	0.0	0.0	0.0	0.0	0.0	0.0	0.0

TACGTAGGGA	0.0	0.0	0.0	0.0	0.0	0.0	0.0
TACGTATGGT	0.0	0.0	0.0	0.0	0.0	0.0	0.0
TACGTAGGGA	0.0	0.0	0.0	0.0	0.0	0.0	0.0
TACGTAGGGG	0.0	0.0	0.0	0.0	0.0	0.0	0.0
TAAGTAGGTG	0.0	0.0	0.0	0.0	0.0	0.0	0.0
TACGTAGGGA	0.0	0.0	0.0	0.0	0.0	0.0	0.0
TACGTAGGAT	0.0	0.0	0.0	0.0	0.0	0.0	0.0
TACGTATGGT	0.0	0.0	0.0	0.0	0.0	0.0	0.0
TACGTAGGGG	0.0	0.0	0.0	0.0	0.0	0.0	0.0
TACGTAGGGG	0.0	0.0	0.0	0.0	0.0	0.0	0.0
TACGTAGGTC	0.0	0.0	0.0	0.0	0.0	0.0	0.0
AACGTAGGTC	0.0	0.0	0.0	0.0	0.0	0.0	0.0
TACGTAGGGG	0.0	0.0	0.0	0.0	0.0	0.0	0.0
TACGTAGGGG	0.0	0.0	0.0	0.0	0.0	0.0	0.0
TACGTAGGGG	0.0	0.0	0.0	0.0	0.0	0.0	0.0
TACTTCTTTTT	0.0	0.0	0.0	0.0	0.0	0.0	0.0
TACGTATGGT	0.0	0.0	0.0	0.0	0.0	0.0	0.0
TACGTAGGTG	0.0	0.0	0.0	0.0	0.0	0.0	0.0
TACGTAGGGG	0.0	0.0	0.0	0.0	0.0	0.0	0.0
TACGTAGGTG	0.0	0.0	0.0	0.0	0.0	0.0	0.0
TACGTAGGGT	0.0	0.0	0.0	0.0	0.0	0.0	0.0
TACGTAGGGA	0.0	0.0	0.0	0.0	0.0	0.0	0.0
TACGTATGGT	0.0	0.0	0.0	0.0	0.0	0.0	0.0
TACGTATGGA	0.0	0.0	0.0	0.0	0.0	0.0	0.0
TACGTATGGC	0.0	0.0	0.0	0.0	0.0	0.0	0.0
TACGTAGGGA	0.0	0.0	0.0	0.0	0.0	0.0	0.0
TACGGAGGAT	0.0	0.0	0.0	0.0	0.0	0.0	0.0
AACGTAGGTC	0.0	0.0	0.0	0.0	0.0	0.0	0.0
TACGTAGGTG	0.0	0.0	0.0	0.0	0.0	0.0	0.0
TACGTAGGTG	0.0	0.0	0.0	0.0	0.0	0.0	0.0
TACGTAGGGT	0.0	0.0	0.0	0.0	0.0	0.0	0.0
TACGTATGGT	0.0	0.0	0.0	0.0	0.0	0.0	0.0
TACGTATGGT	0.0	0.0	0.0	0.0	0.0	0.0	0.0
TACGTAGGTG	0.0	0.0	0.0	0.0	0.0	0.0	0.0
TACGTATGGT	0.0	0.0	0.0	0.0	0.0	0.0	0.0
TACGTATGGC	0.0	0.0	0.0	0.0	0.0	0.0	0.0
TACGTAGGGG	0.0	0.0	0.0	0.0	0.0	0.0	0.0
TACGTATGGA	0.0	0.0	0.0	0.0	0.0	0.0	0.0
TACGTAGGGG	0.0	0.0	0.0	0.0	0.0	0.0	0.0
TACGTATGGT	0.0	0.0	0.0	0.0	0.0	0.0	0.0
TACGTAGGGG	0.0	0.0	0.0	0.0	0.0	0.0	0.0
TACGTAGGTG	0.0	0.0	0.0	0.0	0.0	0.0	0.0
TACGTAGGGG	0.0	0.0	0.0	0.0	0.0	0.0	0.0
TACGTAGGGG	0.0	0.0	0.0	0.0	0.0	0.0	0.0
TACGTAGGTG	0.0	0.0	0.0	0.0	0.0	0.0	0.0
TACGTAGGGG	0.0	0.0	0.0	0.0	0.0	0.0	0.0
TACGTAGGTG	0.0	0.0	0.0	0.0	0.0	0.0	0.0

TACGGAGGAT	0.0	0.0	0.0	0.0	0.0	0.0	0.0
TACGTAGGGG	0.0	0.0	0.0	0.0	0.0	0.0	0.0
TACGTAGGGA	0.0	0.0	0.0	0.0	0.0	0.0	0.0
TACGTAGGTG	0.0	0.0	0.0	0.0	0.0	0.0	0.0
TACGTAGGGA	0.0	0.0	0.0	0.0	0.0	0.0	0.0
TACGTAGGGG	0.0	0.0	0.0	0.0	0.0	0.0	0.0
TACGTAGGGA	0.0	0.0	0.0	0.0	0.0	0.0	0.0
TACGTAGGGG	0.0	0.0	0.0	0.0	0.0	0.0	0.0
TACGGAGGGT	0.0	0.0	0.0	0.0	0.0	0.0	0.0
TACGGAGGAT	0.0	0.0	0.0	0.0	0.0	0.0	0.0
TACGTATGTC	0.0	0.0	0.0	0.0	0.0	0.0	0.0
TACGGAAGGT	0.0	0.0	0.0	0.0	0.0	0.0	0.0
TACGTATGGT	0.0	0.0	0.0	0.0	0.0	0.0	0.0
TACGTAGGGG	0.0	0.0	0.0	0.0	0.0	0.0	0.0
TACGTAGGTG	0.0	0.0	0.0	0.0	0.0	0.0	0.0
TACGTAGGGG	0.0	0.0	0.0	0.0	0.0	0.0	0.0
TACGTAGGTC	0.0	0.0	0.0	0.0	0.0	0.0	0.0
AACGTAGGTG	0.0	0.0	0.0	0.0	0.0	0.0	0.0
TACGTAGGGG	0.0	0.0	0.0	0.0	0.0	0.0	0.0
AACGTAGGTC	0.0	0.0	0.0	0.0	0.0	0.0	0.0
TACGTAGGGG	0.0	0.0	0.0	0.0	0.0	0.0	0.0
TACGTAGGGG	0.0	0.0	0.0	0.0	0.0	0.0	0.0
TACGGAGGAT	0.0	0.0	0.0	0.0	0.0	0.0	0.0
TACGTAGGGA	0.0	0.0	0.0	0.0	0.0	0.0	0.0
TACGTATGGT	0.0	0.0	0.0	0.0	0.0	0.0	0.0
TACGTATGGT	0.0	0.0	0.0	0.0	0.0	0.0	0.0
TACGTAGGGG	0.0	0.0	0.0	0.0	0.0	0.0	0.0
TACGGAGGAT	0.0	0.0	0.0	0.0	0.0	0.0	0.0
TACGGAGGAT	0.0	0.0	0.0	0.0	0.0	0.0	0.0
TACGGAGGAT	0.0	0.0	0.0	0.0	0.0	0.0	0.0
TACGTAGGTG	0.0	0.0	0.0	0.0	0.0	0.0	0.0
TACGTATGGA	0.0	0.0	0.0	0.0	0.0	0.0	0.0
TACGTATGGA	0.0	0.0	0.0	0.0	0.0	0.0	0.0
TACGTATGGA	0.0	0.0	0.0	0.0	0.0	0.0	0.0
TACGTATGGT	0.0	0.0	0.0	0.0	0.0	0.0	0.0
TACGTAGGGG	0.0	0.0	0.0	0.0	0.0	0.0	0.0
TACGGAGGAT	0.0	0.0	0.0	0.0	0.0	0.0	0.0
TACGTATGGA	0.0	0.0	0.0	0.0	0.0	0.0	0.0
TACGGAGGAT	0.0	0.0	0.0	0.0	0.0	0.0	0.0
TACGTATGTT	0.0	0.0	0.0	0.0	0.0	0.0	0.0
TACGTAGGGG	0.0	0.0	0.0	0.0	0.0	0.0	0.0
TACGTAGGGG	0.0	0.0	0.0	0.0	0.0	0.0	0.0
TACGTAGGGA	0.0	0.0	0.0	0.0	0.0	0.0	0.0
TACGTATGGT	0.0	0.0	0.0	0.0	0.0	0.0	0.0
TCCGTCTTTTT	0.0	0.0	0.0	0.0	0.0	0.0	0.0
TACGTAGGGA	0.0	0.0	0.0	0.0	0.0	0.0	0.0
TACGTAGGTG	0.0	0.0	0.0	0.0	0.0	0.0	0.0

TACGTAGGG	0.0	0.0	0.0	0.0	0.0	0.0	0.0
TACGTAGGG	0.0	0.0	0.0	0.0	0.0	0.0	0.0
TACGTAGGTG	0.0	0.0	0.0	0.0	0.0	0.0	0.0
TACGTAGGTG	0.0	0.0	0.0	0.0	0.0	0.0	0.0
TACGTAGGTG	0.0	0.0	0.0	0.0	0.0	0.0	0.0
TACGTAGGG	0.0	0.0	0.0	0.0	0.0	0.0	0.0
TACGTAGGG	0.0	0.0	0.0	0.0	0.0	0.0	0.0
TACGTAGGTG	0.0	0.0	0.0	0.0	0.0	0.0	0.0
TACGGAGGAT	0.0	0.0	0.0	0.0	0.0	0.0	0.0
TACGTATGGT	0.0	0.0	0.0	0.0	0.0	0.0	0.0
TACGTAGGG	0.0	0.0	0.0	0.0	0.0	0.0	0.0
TACGGAGGGT	0.0	0.0	0.0	0.0	0.0	0.0	0.0
TACGTAGGGA	0.0	0.0	0.0	0.0	0.0	0.0	0.0
TACGGAGGGT	0.0	0.0	0.0	0.0	0.0	0.0	0.0
TACGTAGGG	0.0	0.0	0.0	0.0	0.0	0.0	0.0
TACGTAGGTG	0.0	0.0	0.0	0.0	0.0	0.0	0.0
TACGTAGGG	0.0	0.0	0.0	0.0	0.0	0.0	0.0
TACGTAGGG	0.0	0.0	0.0	0.0	0.0	0.0	0.0
TACGTAGGG	0.0	0.0	0.0	0.0	0.0	0.0	0.0
TACGTAGGG	0.0	0.0	0.0	0.0	0.0	0.0	0.0
TACGGAGGAT	0.0	0.0	0.0	0.0	0.0	0.0	0.0
TACGTATGGA	0.0	0.0	0.0	0.0	0.0	0.0	0.0
TACGTAGGG	0.0	0.0	0.0	0.0	0.0	0.0	0.0
TACGGAGGAT	0.0	0.0	0.0	0.0	0.0	0.0	0.0
TACGTAGGTG	0.0	0.0	0.0	0.0	0.0	0.0	0.0
TACGTATGGT	0.0	0.0	0.0	0.0	0.0	0.0	0.0
TACGGAGGAT	0.0	0.0	0.0	0.0	0.0	0.0	0.0
TACGTAGGG	0.0	0.0	0.0	0.0	0.0	0.0	0.0
TACGTAGGGA	0.0	0.0	0.0	0.0	0.0	0.0	0.0
TACGTAGGTG	0.0	0.0	0.0	0.0	0.0	0.0	0.0
TACGTATGGA	0.0	0.0	0.0	0.0	0.0	0.0	0.0
TACGTAGGG	0.0	0.0	0.0	0.0	0.0	0.0	0.0
TACGTAGGG	0.0	0.0	0.0	0.0	0.0	0.0	0.0
TACGGAGGAT	0.0	0.0	0.0	0.0	0.0	0.0	0.0
TACGTAGGTG	0.0	0.0	0.0	0.0	0.0	0.0	0.0
TACGTAGGG	0.0	0.0	0.0	0.0	0.0	0.0	0.0
TACGTATGGA	0.0	0.0	0.0	0.0	0.0	0.0	0.0
TACGGAGGAT	0.0	0.0	0.0	0.0	0.0	0.0	0.0
AACGTAGGGT	0.0	0.0	0.0	0.0	0.0	0.0	0.0
TACGTATGGA	0.0	0.0	0.0	0.0	0.0	0.0	0.0
TACGTAGGG	0.0	0.0	0.0	0.0	0.0	0.0	0.0
TACGTAGGG	0.0	0.0	0.0	0.0	0.0	0.0	0.0
TACGTATGGT	0.0	0.0	0.0	0.0	0.0	0.0	0.0
TACGGAGGAT	0.0	0.0	0.0	0.0	0.0	0.0	0.0
TACGTATGGT	0.0	0.0	0.0	0.0	0.0	0.0	0.0
TACGTAGGG	0.0	0.0	0.0	0.0	0.0	0.0	0.0
TACGGAGGGT	0.0	0.0	0.0	0.0	0.0	0.0	0.0

AACGGGGGG	0.0	0.0	0.0	0.0	0.0	0.0	0.0
TACGTAGGGA	0.0	0.0	0.0	0.0	0.0	0.0	0.0
TACGTAGGGG	0.0	0.0	0.0	0.0	0.0	0.0	0.0
TACGTAGGGC	0.0	0.0	0.0	0.0	0.0	0.0	0.0
TACGTAGGTC	0.0	0.0	0.0	0.0	0.0	0.0	0.0
TACGTATGGA	0.0	0.0	0.0	0.0	0.0	0.0	0.0
TACGGAGGAT	0.0	0.0	0.0	0.0	0.0	0.0	0.0
TACGTAGGTC	0.0	0.0	0.0	0.0	0.0	0.0	0.0
TACGTAGGTG	0.0	0.0	0.0	0.0	0.0	0.0	0.0
TACGTATGGT	0.0	0.0	0.0	0.0	0.0	0.0	0.0
TACGTAGGTG	0.0	0.0	0.0	0.0	0.0	0.0	0.0
TACGTATGGT	0.0	0.0	0.0	0.0	0.0	0.0	0.0
TACGTAGGTG	0.0	0.0	0.0	0.0	0.0	0.0	0.0
TACGTAGGTG	0.0	0.0	0.0	0.0	0.0	0.0	0.0
TACGTAGGGA	0.0	0.0	0.0	0.0	0.0	0.0	0.0
TACGTAGGTG	0.0	0.0	0.0	0.0	0.0	0.0	0.0
TACGTAGGGG	0.0	0.0	0.0	0.0	0.0	0.0	0.0
TACGTAGGTG	0.0	0.0	0.0	0.0	0.0	0.0	0.0
TACGTATGGT	0.0	0.0	0.0	0.0	0.0	0.0	0.0
TACGTATGGT	0.0	0.0	0.0	0.0	0.0	0.0	0.0
TACGTAGGGA	0.0	0.0	0.0	0.0	0.0	0.0	0.0
TACGTAGGTG	0.0	0.0	0.0	0.0	0.0	0.0	0.0
TACGTAGGGA	0.0	0.0	0.0	0.0	0.0	0.0	0.0
TACGTAGGTG	0.0	0.0	0.0	0.0	0.0	0.0	0.0
TACGTAGGGG	0.0	0.0	0.0	0.0	0.0	0.0	0.0
TACGTAGGTG	0.0	0.0	0.0	0.0	0.0	0.0	0.0
TACGTAGGTG	0.0	0.0	0.0	0.0	0.0	0.0	0.0
TACGTAGGGA	0.0	0.0	0.0	0.0	0.0	0.0	0.0
TACGTAGGGA	0.0	0.0	0.0	0.0	0.0	0.0	0.0
TACGTAGGGA	0.0	0.0	0.0	0.0	0.0	0.0	0.0
TACGTAGGGG	0.0	0.0	0.0	0.0	0.0	0.0	0.0
TACGTAGGTG	0.0	0.0	0.0	0.0	0.0	0.0	0.0
TACGTAGGTG	0.0	0.0	0.0	0.0	0.0	0.0	0.0
TACGTAGGGA	0.0	0.0	0.0	0.0	0.0	0.0	0.0
TACGTAGGGA	0.0	0.0	0.0	0.0	0.0	0.0	0.0
TACGTAGAGG	0.0	0.0	0.0	0.0	0.0	0.0	0.0
TACGTATGGA	0.0	0.0	0.0	0.0	0.0	0.0	0.0
TACGTATGGA	0.0	0.0	0.0	0.0	0.0	0.0	0.0
TACGTAGGGA	0.0	0.0	0.0	0.0	0.0	0.0	0.0
TACGTAGGTG	0.0	0.0	0.0	0.0	0.0	0.0	0.0
TACGTAGGTG	0.0	0.0	0.0	0.0	0.0	0.0	0.0
AACGTAGGTG	0.0	0.0	0.0	0.0	0.0	0.0	0.0
TACGGAGGAT	0.0	0.0	0.0	0.0	0.0	0.0	0.0
TACGTATGGA	0.0	0.0	0.0	0.0	0.0	0.0	0.0
TACGTATGGA	0.0	0.0	0.0	0.0	0.0	0.0	0.0
TACATAGGGA	0.0	0.0	0.0	0.0	0.0	0.0	0.0
TACGTATGGT	0.0	0.0	0.0	0.0	0.0	0.0	0.0

TACGTAGGTG	0.0	0.0	0.0	0.0	0.0	0.0	0.0
TACGTAGGG	0.0	0.0	0.0	0.0	0.0	0.0	0.0
TACGTATGGT	0.0	0.0	0.0	0.0	0.0	0.0	0.0
TACGTAGGTG	0.0	0.0	0.0	0.0	0.0	0.0	0.0
TACGTAGGG	0.0	0.0	0.0	0.0	0.0	0.0	0.0
TACGGAAGGT	0.0	0.0	0.0	0.0	0.0	0.0	0.0
TACGTAGGG	0.0	0.0	0.0	0.0	0.0	0.0	0.0
TACGTAGGGA	0.0	0.0	0.0	0.0	0.0	0.0	0.0
TACGTAGGGA	0.0	0.0	0.0	0.0	0.0	0.0	0.0
TACGTAGGGA	0.0	0.0	0.0	0.0	0.0	0.0	0.0
TACGTAGGG	0.0	0.0	0.0	0.0	0.0	0.0	0.0
TACGTAGGTA	0.0	0.0	0.0	0.0	0.0	0.0	0.0
TACGTAGGG	0.0	0.0	0.0	0.0	0.0	0.0	0.0
AACGTAGGTC	0.0	0.0	0.0	0.0	0.0	0.0	0.0
TACGTAGGG	0.0	0.0	0.0	0.0	0.0	0.0	0.0
TACGTAGGG	0.0	0.0	0.0	0.0	0.0	0.0	0.0
AACGTAGGTC	0.0	0.0	0.0	0.0	0.0	0.0	0.0
TACGTAGGG	0.0	0.0	0.0	0.0	0.0	0.0	0.0
TACGTAGGG	0.0	0.0	0.0	0.0	0.0	0.0	0.0
TACGTATGGG	0.0	0.0	0.0	0.0	0.0	0.0	0.0
TACGTAGGGT	0.0	0.0	0.0	0.0	0.0	0.0	0.0
TTCGTATGGT	0.0	0.0	0.0	0.0	0.0	0.0	0.0
TACGAAGGG	0.0	0.0	0.0	0.0	0.0	0.0	0.0
TACGTAGGG	0.0	0.0	0.0	0.0	0.0	0.0	0.0
TACGTAGGGA	0.0	0.0	0.0	0.0	0.0	0.0	0.0
AACGTAGGGT	0.0	0.0	0.0	0.0	0.0	0.0	0.0
TACGTAGGG	0.0	0.0	0.0	0.0	0.0	0.0	0.0
TACGTAGGGT	0.0	0.0	0.0	0.0	0.0	0.0	0.0
TACGTAGGGA	0.0	0.0	0.0	0.0	0.0	0.0	0.0
TACGTAGGG	0.0	0.0	0.0	0.0	0.0	0.0	0.0
TACGTAGGTC	0.0	0.0	0.0	0.0	0.0	0.0	0.0
TACGGAGGAT	0.0	0.0	0.0	0.0	0.0	0.0	0.0
TACGTAGGGT	0.0	0.0	0.0	0.0	0.0	0.0	0.0
TACGTAGGTG	0.0	0.0	0.0	0.0	0.0	0.0	0.0
TACGTAGGG	0.0	0.0	0.0	0.0	0.0	0.0	0.0
TACGTAGGGA	0.0	0.0	0.0	0.0	0.0	0.0	0.0
TACGGAGGGT	0.0	0.0	0.0	0.0	0.0	0.0	0.0
TACGTAGGGA	0.0	0.0	0.0	0.0	0.0	0.0	0.0
TACGTAGGGT	0.0	0.0	0.0	0.0	0.0	0.0	0.0
TACGTAGGG	0.0	0.0	0.0	0.0	0.0	0.0	0.0
TACGGAGGGT	0.0	0.0	0.0	0.0	0.0	0.0	0.0
TACGTAGGG	0.0	0.0	0.0	0.0	0.0	0.0	0.0
TACGTAGGG	0.0	0.0	0.0	0.0	0.0	0.0	0.0
TACGGAGGGT	0.0	0.0	0.0	0.0	0.0	0.0	0.0
TACGGAGGAT	0.0	0.0	0.0	0.0	0.0	0.0	0.0
TACGTAGGTG	0.0	0.0	0.0	0.0	0.0	0.0	0.0
TACGTAGGG	0.0	0.0	0.0	0.0	0.0	0.0	0.0

TACGTAGGGT	0.0	0.0	0.0	0.0	0.0	0.0	0.0
CACGTAGGTC	0.0	0.0	0.0	0.0	0.0	0.0	0.0
TACGTAGGGA	0.0	0.0	0.0	0.0	0.0	0.0	0.0
TACGTAGGGG	0.0	0.0	0.0	0.0	0.0	0.0	0.0
TACGTAGGGC	0.0	0.0	0.0	0.0	0.0	0.0	0.0
AACGTAGGGT	0.0	0.0	0.0	0.0	0.0	0.0	0.0
TACGGAGGAT	0.0	0.0	0.0	0.0	0.0	0.0	0.0
TACGTATGGA	0.0	0.0	0.0	0.0	0.0	0.0	0.0
TACGTAGGTC	0.0	0.0	0.0	0.0	0.0	0.0	0.0
TACGTAGGGG	0.0	0.0	0.0	0.0	0.0	0.0	0.0
TACGTAGGGA	0.0	0.0	0.0	0.0	0.0	0.0	0.0
TACGTAGGGG	0.0	0.0	0.0	0.0	0.0	0.0	0.0
TACATAGGGA	0.0	0.0	0.0	0.0	0.0	0.0	0.0
TACGTAGGGG	0.0	0.0	0.0	0.0	0.0	0.0	0.0
TACGTAGGTG	0.0	0.0	0.0	0.0	0.0	0.0	0.0
TACGTATGGT	0.0	0.0	0.0	0.0	0.0	0.0	0.0
TACGTAGGGA	0.0	0.0	0.0	0.0	0.0	0.0	0.0
TACGGAAGGT	0.0	0.0	0.0	0.0	0.0	0.0	0.0
TACGTAGGGA	0.0	0.0	0.0	0.0	0.0	0.0	0.0
TACGTAGAGG	0.0	0.0	0.0	0.0	0.0	0.0	0.0
TACGTAGGTG	0.0	0.0	0.0	0.0	0.0	0.0	0.0
TACGTAGGGA	0.0	0.0	0.0	0.0	0.0	0.0	0.0
TACGTAGGGA	0.0	0.0	0.0	0.0	0.0	0.0	0.0
TACGTAGGGA	0.0	0.0	0.0	0.0	0.0	0.0	0.0
TACGTAGGTA	0.0	0.0	0.0	0.0	0.0	0.0	0.0
TACGTAGGTG	0.0	0.0	0.0	0.0	0.0	0.0	0.0
TACGTAGGTG	0.0	0.0	0.0	0.0	0.0	0.0	0.0
TACGTAGGGG	0.0	0.0	0.0	0.0	0.0	0.0	0.0
TACGTAGGGG	0.0	0.0	0.0	0.0	0.0	0.0	0.0
TACGTAGGGT	0.0	0.0	0.0	0.0	0.0	0.0	0.0
TACGTAGGGG	0.0	0.0	0.0	0.0	0.0	0.0	0.0
TACGTAGGGG	0.0	0.0	0.0	0.0	0.0	0.0	0.0
TACGTAGGGG	0.0	0.0	0.0	0.0	0.0	0.0	0.0
TACGTAGGTG	0.0	0.0	0.0	0.0	0.0	0.0	0.0
TACGTAGGGG	0.0	0.0	0.0	0.0	0.0	0.0	0.0
TACGTATGGT	0.0	0.0	0.0	0.0	0.0	0.0	0.0
TACGTAGGGG	0.0	0.0	0.0	0.0	0.0	0.0	0.0
TACGTAGGGG	0.0	0.0	0.0	0.0	0.0	0.0	0.0
TACGTAGGGA	0.0	0.0	0.0	0.0	0.0	0.0	0.0
TACGGAGGG	0.0	0.0	0.0	0.0	0.0	0.0	0.0
TACGTAGGTG	0.0	0.0	0.0	0.0	0.0	0.0	0.0
TACGTAGGTT	0.0	0.0	0.0	0.0	0.0	0.0	0.0
TACGTAGGTG	0.0	0.0	0.0	0.0	0.0	0.0	0.0
TACGTAGGTG	0.0	0.0	0.0	0.0	0.0	0.0	0.0
TACGAAGGGT	0.0	0.0	0.0	0.0	0.0	0.0	0.0
TACGTAGGTG	0.0	0.0	0.0	0.0	0.0	0.0	0.0
TACGTAGGTG	0.0	0.0	0.0	0.0	0.0	0.0	0.0

TACGTAGGGG	0.0	0.0	0.0	0.0	0.0	0.0	0.0
TACGTAGGTG	0.0	0.0	0.0	0.0	0.0	0.0	0.0
TACGTAGGTG	0.0	0.0	0.0	0.0	0.0	0.0	0.0
TACGTATGGG	0.0	0.0	0.0	0.0	0.0	0.0	0.0
TACGTATGGT	0.0	0.0	0.0	0.0	0.0	0.0	0.0
TACGTATGGT	0.0	0.0	0.0	0.0	0.0	0.0	0.0
TACGTAGGGG	0.0	0.0	0.0	0.0	0.0	0.0	0.0
TACGTATGGG	0.0	0.0	0.0	0.0	0.0	0.0	0.0
TACAGAGGTC	0.0	0.0	0.0	0.0	0.0	0.0	0.0
TACGTAGGGG	0.0	0.0	0.0	0.0	0.0	0.0	0.0
AACGGAGGGT	0.0	0.0	0.0	0.0	0.0	0.0	0.0
TACGTAGGGA	0.0	0.0	0.0	0.0	0.0	0.0	0.0
TACGTAGGGG	0.0	0.0	0.0	0.0	0.0	0.0	0.0
TACGTAGGGG	0.0	0.0	0.0	0.0	0.0	0.0	0.0
TACGTAGGGG	0.0	0.0	0.0	0.0	0.0	0.0	0.0
TACGTAGGGG	0.0	0.0	0.0	0.0	0.0	0.0	0.0
TACGTAGGGA	0.0	0.0	0.0	0.0	0.0	0.0	0.0
TACGTAGGGG	0.0	0.0	0.0	0.0	0.0	0.0	0.0
TACGTAGGGG	0.0	0.0	0.0	0.0	0.0	0.0	0.0
TACGTAGGTG	0.0	0.0	0.0	0.0	0.0	0.0	0.0
TACGTAGGGG	0.0	0.0	0.0	0.0	0.0	0.0	0.0
TACGGAGGGT	0.0	0.0	0.0	0.0	0.0	0.0	0.0
TACGTAGGGA	0.0	0.0	0.0	0.0	0.0	0.0	0.0
TACGTAGGGG	0.0	0.0	0.0	0.0	0.0	0.0	0.0
TACGTAGGGT	0.0	0.0	0.0	0.0	0.0	0.0	0.0
TACGTATGGA	0.0	0.0	0.0	0.0	0.0	0.0	0.0
TACGTAGGGG	0.0	0.0	0.0	0.0	0.0	0.0	0.0
TACGTAGGGT	0.0	0.0	0.0	0.0	0.0	0.0	0.0
TACGTATGGT	0.0	0.0	0.0	0.0	0.0	0.0	0.0
TACGTAGGGG	0.0	0.0	0.0	0.0	0.0	0.0	0.0
TACGTAGGTC	0.0	0.0	0.0	0.0	0.0	0.0	0.0
TACGTAGGTG	0.0	0.0	0.0	0.0	0.0	0.0	0.0
TACGGAGGAT	0.0	0.0	0.0	0.0	0.0	0.0	0.0
TACGGAGGAT	0.0	0.0	0.0	0.0	0.0	0.0	0.0
TACGTAGGGG	0.0	0.0	0.0	0.0	0.0	0.0	0.0
TACGTAGGGG	0.0	0.0	0.0	0.0	0.0	0.0	0.0
AACGTAGGGT	0.0	0.0	0.0	0.0	0.0	0.0	0.0
TACGTAGGGG	0.0	0.0	0.0	0.0	0.0	0.0	0.0
TACGTAGGGA	0.0	0.0	0.0	0.0	0.0	0.0	0.0
AACGTAGGGT	0.0	0.0	0.0	0.0	0.0	0.0	0.0
TACATAGGGA	0.0	0.0	0.0	0.0	0.0	0.0	0.0
TACGTAGGGA	0.0	0.0	0.0	0.0	0.0	0.0	0.0
TACGTAGGGA	0.0	0.0	0.0	0.0	0.0	0.0	0.0
TACGTAGGGA	0.0	0.0	0.0	0.0	0.0	0.0	0.0
TACGGAGGAT	0.0	0.0	0.0	0.0	0.0	0.0	0.0
TACGTAGGGG	0.0	0.0	0.0	0.0	0.0	0.0	0.0
TACGAAGGGT	0.0	0.0	0.0	0.0	0.0	0.0	0.0

TACGTAGGGG	0.0	0.0	0.0	0.0	0.0	0.0	0.0
TACGTAGGGA	0.0	0.0	0.0	0.0	0.0	0.0	0.0
TACGTAGGGG	0.0	0.0	0.0	0.0	0.0	0.0	0.0
TACGTAGGGG	0.0	0.0	0.0	0.0	0.0	0.0	0.0
TACGTAGGGG	0.0	0.0	0.0	0.0	0.0	0.0	0.0
TACGTAGGGG	0.0	0.0	0.0	0.0	0.0	0.0	0.0
TACGTAGGTG	0.0	0.0	0.0	0.0	0.0	0.0	0.0
TACGTATGGT	0.0	0.0	0.0	0.0	0.0	0.0	0.0
TACGTAGGGG	0.0	0.0	0.0	0.0	0.0	0.0	0.0
TACATAGGGT	0.0	0.0	0.0	0.0	0.0	0.0	0.0
TACGTAGGTG	0.0	0.0	0.0	0.0	0.0	0.0	0.0
TACGTATGGT	0.0	0.0	0.0	0.0	0.0	0.0	0.0
TACGTATGGA	0.0	0.0	0.0	0.0	0.0	0.0	0.0
TACGTAGGTG	0.0	0.0	0.0	0.0	0.0	0.0	0.0
TACGTAGGTG	0.0	0.0	0.0	0.0	0.0	0.0	0.0
TACGTAGGTG	0.0	0.0	0.0	0.0	0.0	0.0	0.0
TACGTAGGGG	0.0	0.0	0.0	0.0	0.0	0.0	0.0
TACGTATGGT	0.0	0.0	0.0	0.0	0.0	0.0	0.0
TACGTATGGT	0.0	0.0	0.0	0.0	0.0	0.0	0.0
TACGTATGGA	0.0	0.0	0.0	0.0	0.0	0.0	0.0
TACGTAGGGG	0.0	0.0	0.0	0.0	0.0	0.0	0.0
TACGTATGGA	0.0	0.0	0.0	0.0	0.0	0.0	0.0
TACGTAGGTG	0.0	0.0	0.0	0.0	0.0	0.0	0.0
TACGTATGGA	0.0	0.0	0.0	0.0	0.0	0.0	0.0
TACGTAGGGG	0.0	0.0	0.0	0.0	0.0	0.0	0.0
TACGTAGGGC	0.0	0.0	0.0	0.0	0.0	0.0	0.0
TACGTAGGTG	0.0	0.0	0.0	0.0	0.0	0.0	0.0
TACGTACGTG	0.0	0.0	0.0	0.0	0.0	0.0	0.0
TACTTCTTCTC	0.0	0.0	0.0	0.0	0.0	0.0	0.0
TACGTAGGGT	0.0	0.0	0.0	0.0	0.0	0.0	0.0
TACGTATGGT	0.0	0.0	0.0	0.0	0.0	0.0	0.0
TACGTAGGGT	0.0	0.0	0.0	0.0	0.0	0.0	0.0
TACGTAGGCG	0.0	0.0	0.0	0.0	0.0	0.0	0.0
TACGGAGGAT	0.0	0.0	0.0	0.0	0.0	0.0	0.0
TACGTAGGGG	0.0	0.0	0.0	0.0	0.0	0.0	0.0
TACGTAGGGG	0.0	0.0	0.0	0.0	0.0	0.0	0.0
TACGTAGGGG	0.0	0.0	0.0	0.0	0.0	0.0	0.0
AACGTAGGTC	0.0	0.0	0.0	0.0	0.0	0.0	0.0
TACGTAGGGG	0.0	0.0	0.0	0.0	0.0	0.0	0.0
TACGTAGGGG	0.0	0.0	0.0	0.0	0.0	0.0	0.0
TACGTAGGGG	0.0	0.0	0.0	0.0	0.0	0.0	0.0
TACGTAGGGG	0.0	0.0	0.0	0.0	0.0	0.0	0.0
TACGTAGGGG	0.0	0.0	0.0	0.0	0.0	0.0	0.0
TACGTAGGGG	0.0	0.0	0.0	0.0	0.0	0.0	0.0
TACGTAGGTG	0.0	0.0	0.0	0.0	0.0	0.0	0.0
TACGTAGGGG	0.0	0.0	0.0	0.0	0.0	0.0	0.0
TACGGAGGGT	0.0	0.0	0.0	0.0	0.0	0.0	0.0

TACGTAGGGA	0.0	0.0	0.0	0.0	0.0	0.0	0.0
TACGTAGGGG	0.0	0.0	0.0	0.0	0.0	0.0	0.0
TACGTAGGTG	0.0	0.0	0.0	0.0	0.0	0.0	0.0
TACGGAGGGT	0.0	0.0	0.0	0.0	0.0	0.0	0.0
TACGGAGGGT	0.0	0.0	0.0	0.0	0.0	0.0	0.0
TACGTAGGTG	0.0	0.0	0.0	0.0	0.0	0.0	0.0
TACGTAGGGA	0.0	0.0	0.0	0.0	0.0	0.0	0.0
TACGTAGGTG	0.0	0.0	0.0	0.0	0.0	0.0	0.0
TACGTAGGGG	0.0	0.0	0.0	0.0	0.0	0.0	0.0
TACGTAGGGG	0.0	0.0	0.0	0.0	0.0	0.0	0.0
TACGTAGGGG	0.0	0.0	0.0	0.0	0.0	0.0	0.0
TACGTATGGT	0.0	0.0	0.0	0.0	0.0	0.0	0.0
TACGTAGGGG	0.0	0.0	0.0	0.0	0.0	0.0	0.0
TACGTAGGGG	0.0	0.0	0.0	0.0	0.0	0.0	0.0
TACGTATGGT	0.0	0.0	0.0	0.0	0.0	0.0	0.0
TACGTATGGT	0.0	0.0	0.0	0.0	0.0	0.0	0.0
TACGTAGGGG	0.0	0.0	0.0	0.0	0.0	0.0	0.0
TACGTAGGTG	0.0	0.0	0.0	0.0	0.0	0.0	0.0
TACGTAGGGA	0.0	0.0	0.0	0.0	0.0	0.0	0.0
TACGTAGGGG	0.0	0.0	0.0	0.0	0.0	0.0	0.0
TACGTAGGGG	0.0	0.0	0.0	0.0	0.0	0.0	0.0
TACGTAGGGG	0.0	0.0	0.0	0.0	0.0	0.0	0.0
TACGTAGGTG	0.0	0.0	0.0	0.0	0.0	0.0	0.0
TACGTATGGA	0.0	0.0	0.0	0.0	0.0	0.0	0.0
TACGTAGGGG	0.0	0.0	0.0	0.0	0.0	0.0	0.0
TACGTAGGGA	0.0	0.0	0.0	0.0	0.0	0.0	0.0
TACGTATGGT	0.0	0.0	0.0	0.0	0.0	0.0	0.0
TACGGAGGAT	0.0	0.0	0.0	0.0	0.0	0.0	0.0
TACGTAGGTG	0.0	0.0	0.0	0.0	0.0	0.0	0.0
TACGGAGGGT	0.0	0.0	0.0	0.0	0.0	0.0	0.0
TACGTAGGGC	0.0	0.0	0.0	0.0	0.0	0.0	0.0
TACTTCTTTCT	0.0	0.0	0.0	0.0	0.0	0.0	0.0
TACGTAGGGG	0.0	0.0	0.0	0.0	0.0	0.0	0.0
TACGTAGGGG	0.0	0.0	0.0	0.0	0.0	0.0	0.0
TACGTAGGGG	0.0	0.0	0.0	0.0	0.0	0.0	0.0
TACGGAGGAT	0.0	0.0	0.0	0.0	0.0	0.0	0.0
TACGTAGATG	0.0	0.0	0.0	0.0	0.0	0.0	0.0
TACGTATGGT	0.0	0.0	0.0	0.0	0.0	0.0	0.0
TACGTAGGGG	0.0	0.0	0.0	0.0	0.0	0.0	0.0
TACGTAGGTG	0.0	0.0	0.0	0.0	0.0	0.0	0.0
TACGTAGGTG	0.0	0.0	0.0	0.0	0.0	0.0	0.0
TACGTAGGGT	0.0	0.0	0.0	0.0	0.0	0.0	0.0
TACGTAGGTG	0.0	0.0	0.0	0.0	0.0	0.0	0.0
TACGTATGGA	0.0	0.0	0.0	0.0	0.0	0.0	0.0
TACGTAGGTG	0.0	0.0	0.0	0.0	0.0	0.0	0.0
TACGTAGGGG	0.0	0.0	0.0	0.0	0.0	0.0	0.0
TACGTAGGGG	0.0	0.0	0.0	0.0	0.0	0.0	0.0

TACGTATGGT	0.0	0.0	0.0	0.0	0.0	0.0	0.0
TACGTAGGTG	0.0	0.0	0.0	0.0	0.0	0.0	0.0
TACGTAGGGG	0.0	0.0	0.0	0.0	0.0	0.0	0.0
TACGTAGGGG	0.0	0.0	0.0	0.0	0.0	0.0	0.0
TACGTAGGTG	0.0	0.0	0.0	0.0	0.0	0.0	0.0
TACGTAGGGG	0.0	0.0	0.0	0.0	0.0	0.0	0.0
TACGTAGGTG	0.0	0.0	0.0	0.0	0.0	0.0	0.0
TACGTATGGT	0.0	0.0	0.0	0.0	0.0	0.0	0.0
TACGTAGGGT	0.0	0.0	0.0	0.0	0.0	0.0	0.0
AACGTAGGTC	0.0	0.0	0.0	0.0	0.0	0.0	0.0
TACGTAGGTG	0.0	0.0	0.0	0.0	0.0	0.0	0.0
AACGTAGGGT	0.0	0.0	0.0	0.0	0.0	0.0	0.0
AACTTCTTTCC	0.0	0.0	0.0	0.0	0.0	0.0	0.0
TACGTAGGGA	0.0	0.0	0.0	0.0	0.0	0.0	0.0
TACGTAGGGG	0.0	0.0	0.0	0.0	0.0	0.0	0.0
AACGTAGGTC	0.0	0.0	0.0	0.0	0.0	0.0	0.0
TACGTAGGGA	0.0	0.0	0.0	0.0	0.0	0.0	0.0
TACGTAGGGG	0.0	0.0	0.0	0.0	0.0	0.0	0.0
TACATAGGGG	0.0	0.0	0.0	0.0	0.0	0.0	0.0
TACGTAGGGA	0.0	0.0	0.0	0.0	0.0	0.0	0.0
TACGTAGGTG	0.0	0.0	0.0	0.0	0.0	0.0	0.0
TACGTAGGAT	0.0	0.0	0.0	0.0	0.0	0.0	0.0
TACGTAGGGG	0.0	0.0	0.0	0.0	0.0	0.0	0.0
TACGTAGGGA	0.0	0.0	0.0	0.0	0.0	0.0	0.0
TACGTAGGGG	0.0	0.0	0.0	0.0	0.0	0.0	0.0
TACATAGGGA	0.0	0.0	0.0	0.0	0.0	0.0	0.0
TACGTAGGGG	0.0	0.0	0.0	0.0	0.0	0.0	0.0
TACGGAGGAT	0.0	0.0	0.0	0.0	0.0	0.0	0.0
TACGTAGGTG	0.0	0.0	0.0	0.0	0.0	0.0	0.0
TACGTAGGGA	0.0	0.0	0.0	0.0	0.0	0.0	0.0
TACGTAGGGG	0.0	0.0	0.0	0.0	0.0	0.0	0.0
TACGTAGGGA	0.0	0.0	0.0	0.0	0.0	0.0	0.0
TACGTAGGTG	0.0	0.0	0.0	0.0	0.0	0.0	0.0
TACGTAGGGG	0.0	0.0	0.0	0.0	0.0	0.0	0.0
AACGTAGGGT	0.0	0.0	0.0	0.0	0.0	0.0	0.0
TACGTAGGGG	0.0	0.0	0.0	0.0	0.0	0.0	0.0
TACGTAGGGG	0.0	0.0	0.0	0.0	0.0	0.0	0.0
TACGTAGGGA	0.0	0.0	0.0	0.0	0.0	0.0	0.0
TACGTAGGTG	0.0	0.0	0.0	0.0	0.0	0.0	0.0
TACGTAGGGG	0.0	0.0	0.0	0.0	0.0	0.0	0.0
TACGTAGGTG	0.0	0.0	0.0	0.0	0.0	0.0	0.0
TACATAGGGG	0.0	0.0	0.0	0.0	0.0	0.0	0.0
TACGTAGGGG	0.0	0.0	0.0	0.0	0.0	0.0	0.0
TACGTAGGGA	0.0	0.0	0.0	0.0	0.0	0.0	0.0
TACGTAGGGG	0.0	0.0	0.0	0.0	0.0	0.0	0.0
TACGTAGGGG	0.0	0.0	0.0	0.0	0.0	0.0	0.0
TACGTATGGT	0.0	0.0	0.0	0.0	0.0	0.0	0.0

TACGTATGGA	0.0	0.0	0.0	0.0	0.0	0.0	0.0
TACGTAGGGG	0.0	0.0	0.0	0.0	0.0	0.0	0.0
TACGTAGGGA	0.0	0.0	0.0	0.0	0.0	0.0	0.0
TACGTAAGGG	0.0	0.0	0.0	0.0	0.0	0.0	0.0
TACGTAGGTG	0.0	0.0	0.0	0.0	0.0	0.0	0.0
TACGTAGGGG	0.0	0.0	0.0	0.0	0.0	0.0	0.0
TACGTAGGTG	0.0	0.0	0.0	0.0	0.0	0.0	0.0
TACATAGGGG	0.0	0.0	0.0	0.0	0.0	0.0	0.0
TACGTAGGTG	0.0	0.0	0.0	0.0	0.0	0.0	0.0
TACGTAGGGG	0.0	0.0	0.0	0.0	0.0	0.0	0.0
TACGGAGGAT	0.0	0.0	0.0	0.0	0.0	0.0	0.0
TACGTAGGAT	0.0	0.0	0.0	0.0	0.0	0.0	0.0
AACGTAGGTC	0.0	0.0	0.0	0.0	0.0	0.0	0.0
TACGGAGGAT	0.0	0.0	0.0	0.0	0.0	0.0	0.0
TACGTAGGGA	0.0	0.0	0.0	0.0	0.0	0.0	0.0
TACGTAGGGG	0.0	0.0	0.0	0.0	0.0	0.0	0.0
TACGTAGGGT	0.0	0.0	0.0	0.0	0.0	0.0	0.0
TACGTATGAT	0.0	0.0	0.0	0.0	0.0	0.0	0.0
TACGTATGGT	0.0	0.0	0.0	0.0	0.0	0.0	0.0
TACGTAGGGG	0.0	0.0	0.0	0.0	0.0	0.0	0.0
TACGTAGGTG	0.0	0.0	0.0	0.0	0.0	0.0	0.0
TACGTAGGGG	0.0	0.0	0.0	0.0	0.0	0.0	0.0
TACGTATGGT	0.0	0.0	0.0	0.0	0.0	0.0	0.0
TACGTAGGGG	0.0	0.0	0.0	0.0	0.0	0.0	0.0
TACGTAGGGG	0.0	0.0	0.0	0.0	0.0	0.0	0.0
TACGTAGGGA	0.0	0.0	0.0	0.0	0.0	0.0	0.0
TACGTAGGGA	0.0	0.0	0.0	0.0	0.0	0.0	0.0
TACGTAGGGG	0.0	0.0	0.0	0.0	0.0	0.0	0.0
TACGTATGGT	0.0	0.0	0.0	0.0	0.0	0.0	0.0
TACGTAGGGA	0.0	0.0	0.0	0.0	0.0	0.0	0.0
TACGTAGGGG	0.0	0.0	0.0	0.0	0.0	0.0	0.0
TACGTAGGGG	0.0	0.0	0.0	0.0	0.0	0.0	0.0
TACGTAGGGT	0.0	0.0	0.0	0.0	0.0	0.0	0.0
TACGTATGGT	0.0	0.0	0.0	0.0	0.0	0.0	0.0
AACGTAGGTG	0.0	0.0	0.0	0.0	0.0	0.0	0.0
TACGTAGGTG	0.0	0.0	0.0	0.0	0.0	0.0	0.0
TACGTAGGGA	0.0	0.0	0.0	0.0	0.0	0.0	0.0
TACGTAGGGA	0.0	0.0	0.0	0.0	0.0	0.0	0.0
TACGTAGGGA	0.0	0.0	0.0	0.0	0.0	0.0	0.0
TACGGAAGGT	0.0	0.0	0.0	0.0	0.0	0.0	0.0
TACGGAAGGT	0.0	0.0	0.0	0.0	0.0	0.0	0.0
TACGTATGGA	0.0	0.0	0.0	0.0	0.0	0.0	0.0
TACGTAGGGG	0.0	0.0	0.0	0.0	0.0	0.0	0.0
TACGTAGGGG	0.0	0.0	0.0	0.0	0.0	0.0	0.0
TACGTATGGT	0.0	0.0	0.0	0.0	0.0	0.0	0.0
TACGTATGGT	0.0	0.0	0.0	0.0	0.0	0.0	0.0
TACGTATGGT	0.0	0.0	0.0	0.0	0.0	0.0	0.0

TACGTAGGGG	0.0	0.0	0.0	0.0	0.0	0.0	0.0
TACGGAGGAT	0.0	0.0	0.0	0.0	0.0	0.0	0.0
TACGTATGGA	0.0	0.0	0.0	0.0	0.0	0.0	0.0
TACGTATGGT	0.0	0.0	0.0	0.0	0.0	0.0	0.0
TACGTAGGTG	0.0	0.0	0.0	0.0	0.0	0.0	0.0
TACGTAGGGG	0.0	0.0	0.0	0.0	0.0	0.0	0.0
TACGTATGGT	0.0	0.0	0.0	0.0	0.0	0.0	0.0
TACGTAGGGG	0.0	0.0	0.0	0.0	0.0	0.0	0.0
TACGTAGGTG	0.0	0.0	0.0	0.0	0.0	0.0	0.0
AACGTAGGTC	0.0	0.0	0.0	0.0	0.0	0.0	0.0
TACGTAGGGG	0.0	0.0	0.0	0.0	0.0	0.0	0.0
TACGTATGGA	0.0	0.0	0.0	0.0	0.0	0.0	0.0
TACGTAGGTG	0.0	0.0	0.0	0.0	0.0	0.0	0.0
TACGTAGGGG	0.0	0.0	0.0	0.0	0.0	0.0	0.0
TACGGAGGAT	0.0	0.0	0.0	0.0	0.0	0.0	0.0
TACGTATGGA	0.0	0.0	0.0	0.0	0.0	0.0	0.0
TACGTATGGC	0.0	0.0	0.0	0.0	0.0	0.0	0.0
TACGTAGGGG	0.0	0.0	0.0	0.0	0.0	0.0	0.0
TACGTAGGTG	0.0	0.0	0.0	0.0	0.0	0.0	0.0
TACGGAGGAT	0.0	0.0	0.0	0.0	0.0	0.0	0.0
AACGTAGGTC	0.0	0.0	0.0	0.0	0.0	0.0	0.0
TACGTATGGA	0.0	0.0	0.0	0.0	0.0	0.0	0.0
TACGTAGGGG	0.0	0.0	0.0	0.0	0.0	0.0	0.0
TACGTAGGGG	0.0	0.0	0.0	0.0	0.0	0.0	0.0
TACGTATGGT	0.0	0.0	0.0	0.0	0.0	0.0	0.0
TACGTAGGGA	0.0	0.0	0.0	0.0	0.0	0.0	0.0
TACGTATGGT	0.0	0.0	0.0	0.0	0.0	0.0	0.0
AACGTAGGGT	0.0	0.0	0.0	0.0	0.0	0.0	0.0
TACGTAGGGG	0.0	0.0	0.0	0.0	0.0	0.0	0.0
TACGTAGGTG	0.0	0.0	0.0	0.0	0.0	0.0	0.0
TACGGAGGGT	0.0	0.0	0.0	0.0	0.0	0.0	0.0
TACGTAGGTG	0.0	0.0	0.0	0.0	0.0	0.0	0.0
TACGTAGGGG	0.0	0.0	0.0	0.0	0.0	0.0	0.0
TACGTAGGGG	0.0	0.0	0.0	0.0	0.0	0.0	0.0
TACGTAGGGG	0.0	0.0	0.0	0.0	0.0	0.0	0.0
TACGTAGGGA	0.0	0.0	0.0	0.0	0.0	0.0	0.0
TACGTAGGGG	0.0	0.0	0.0	0.0	0.0	0.0	0.0
AACGTAGGGC	0.0	0.0	0.0	0.0	0.0	0.0	0.0
TACGTAGGGA	0.0	0.0	0.0	0.0	0.0	0.0	0.0
TACGTATGGA	0.0	0.0	0.0	0.0	0.0	0.0	0.0
TACGTAGTGG	0.0	0.0	0.0	0.0	0.0	0.0	0.0
TACGTAGGGT	0.0	0.0	0.0	0.0	0.0	0.0	0.0
TACGTAGGGC	0.0	0.0	0.0	0.0	0.0	0.0	0.0
AACGTAGGGT	0.0	0.0	0.0	0.0	0.0	0.0	0.0
TACGTATGGT	0.0	0.0	0.0	0.0	0.0	0.0	0.0
TACGTAGGGG	0.0	0.0	0.0	0.0	0.0	0.0	0.0
TACGTAGGGG	0.0	0.0	0.0	0.0	0.0	0.0	0.0

TACGTAGGG	0.0	0.0	0.0	0.0	0.0	0.0	0.0
TACGTAGGG	0.0	0.0	0.0	0.0	0.0	0.0	0.0
TACGTATGGA	0.0	0.0	0.0	0.0	0.0	0.0	0.0
TACGGAGGAT	0.0	0.0	0.0	0.0	0.0	0.0	0.0
TACGTAGGTG	0.0	0.0	0.0	0.0	0.0	0.0	0.0
TACGTATGGA	0.0	0.0	0.0	0.0	0.0	0.0	0.0
TACGTAGGG	0.0	0.0	0.0	0.0	0.0	0.0	0.0
TACGTATGGT	0.0	0.0	0.0	0.0	0.0	0.0	0.0
TACGTATGGT	0.0	0.0	0.0	0.0	0.0	0.0	0.0
TACGTAGGG	0.0	0.0	0.0	0.0	0.0	0.0	0.0
TACGTAGGG	0.0	0.0	0.0	0.0	0.0	0.0	0.0
TACATATGGT	0.0	0.0	0.0	0.0	0.0	0.0	0.0
TACGTAGGTG	0.0	0.0	0.0	0.0	0.0	0.0	0.0
TATTTCTTTG	0.0	0.0	0.0	0.0	0.0	0.0	0.0
TACGTATAGG	0.0	0.0	0.0	0.0	0.0	0.0	0.0
TACGGAGGAT	0.0	0.0	0.0	0.0	0.0	0.0	0.0
TACGTAGGG	0.0	0.0	0.0	0.0	0.0	0.0	0.0
TACGTAGGGA	0.0	0.0	0.0	0.0	0.0	0.0	0.0
TACGTAGGG	0.0	0.0	0.0	0.0	0.0	0.0	0.0
TACGTAGGG	0.0	0.0	0.0	0.0	0.0	0.0	0.0
TACGTAGGTG	0.0	0.0	0.0	0.0	0.0	0.0	0.0
TACGTAGGG	0.0	0.0	0.0	0.0	0.0	0.0	0.0
TACGTAGGG	0.0	0.0	0.0	0.0	0.0	0.0	0.0
TACGTAGGG	0.0	0.0	0.0	0.0	0.0	0.0	0.0
TACGTAGGG	0.0	0.0	0.0	0.0	0.0	0.0	0.0
TACAGAGGGT	0.0	0.0	0.0	0.0	0.0	0.0	0.0
TACGTAGGG	0.0	0.0	0.0	0.0	0.0	0.0	0.0
TACGTAGGG	0.0	0.0	0.0	0.0	0.0	0.0	0.0
TACGTAGGTG	0.0	0.0	0.0	0.0	0.0	0.0	0.0
TACGTAGGG	0.0	0.0	0.0	0.0	0.0	0.0	0.0
TACGTAGGTG	0.0	0.0	0.0	0.0	0.0	0.0	0.0
CACGTAGGG	0.0	0.0	0.0	0.0	0.0	0.0	0.0
TACGTAGGGA	0.0	0.0	0.0	0.0	0.0	0.0	0.0
TACAGAGGGT	0.0	0.0	0.0	0.0	0.0	0.0	0.0
TACGTAGGG	0.0	0.0	0.0	0.0	0.0	0.0	0.0
TACGTAGGTG	0.0	0.0	0.0	0.0	0.0	0.0	0.0
TACGTATGTT	0.0	0.0	0.0	0.0	0.0	0.0	0.0
TACGTAGGG	0.0	0.0	0.0	0.0	0.0	0.0	0.0
TACGTAGGG	0.0	0.0	0.0	0.0	0.0	0.0	0.0
TACGTATGGT	0.0	0.0	0.0	0.0	0.0	0.0	0.0
TACGTAGGG	0.0	0.0	0.0	0.0	0.0	0.0	0.0
TACGTAGGG	0.0	0.0	0.0	0.0	0.0	0.0	0.0
TACGTATGGT	0.0	0.0	0.0	0.0	0.0	0.0	0.0
TACGTAGGTG	0.0	0.0	0.0	0.0	0.0	0.0	0.0
TACGTAGGG	0.0	0.0	0.0	0.0	0.0	0.0	0.0
TACGTATGGA	0.0	0.0	0.0	0.0	0.0	0.0	0.0
TACGTAGGGA	0.0	0.0	0.0	0.0	0.0	0.0	0.0

TACGTATGGA	0.0	0.0	0.0	0.0	0.0	0.0	0.0
TACGTAGGTG	0.0	0.0	0.0	0.0	0.0	0.0	0.0
TACGTAGGGG	0.0	0.0	0.0	0.0	0.0	0.0	0.0
TACGTAGGGG	0.0	0.0	0.0	0.0	0.0	0.0	0.0
TACGTAGGGG	0.0	0.0	0.0	0.0	0.0	0.0	0.0
TACGTATGGA	0.0	0.0	0.0	0.0	0.0	0.0	0.0
TACGTAGGGG	0.0	0.0	0.0	0.0	0.0	0.0	0.0
TACATAGGTG	0.0	0.0	0.0	0.0	0.0	0.0	0.0
TACGTAGGTG	0.0	0.0	0.0	0.0	0.0	0.0	0.0
TACGTATGGT	0.0	0.0	0.0	0.0	0.0	0.0	0.0
TACGGAGGAT	0.0	0.0	0.0	0.0	0.0	0.0	0.0
TACGGAGGAT	0.0	0.0	0.0	0.0	0.0	0.0	0.0
TACGTAGGGG	0.0	0.0	0.0	0.0	0.0	0.0	0.0
TACGTAGGTG	0.0	0.0	0.0	0.0	0.0	0.0	0.0
TACGTAGGGG	0.0	0.0	0.0	0.0	0.0	0.0	0.0
TACGTAGGGA	0.0	0.0	0.0	0.0	0.0	0.0	0.0
TACGTAGGTG	0.0	0.0	0.0	0.0	0.0	0.0	0.0
TACGGAGGAT	0.0	0.0	0.0	0.0	0.0	0.0	0.0
TACGTATGGT	0.0	0.0	0.0	0.0	0.0	0.0	0.0
TACGTAGGTG	0.0	0.0	0.0	0.0	0.0	0.0	0.0
TACGGAGGAT	0.0	0.0	0.0	0.0	0.0	0.0	0.0
TACGTAGGGA	0.0	0.0	0.0	0.0	0.0	0.0	0.0
TACGTATGGT	0.0	0.0	0.0	0.0	0.0	0.0	0.0
TACGTATGGT	0.0	0.0	0.0	0.0	0.0	0.0	0.0
TACGTATGGA	0.0	0.0	0.0	0.0	0.0	0.0	0.0
TACGGAGGAT	0.0	0.0	0.0	0.0	0.0	0.0	0.0
TACATAGGGG	0.0	0.0	0.0	0.0	0.0	0.0	0.0
TACGTAGGGG	0.0	0.0	0.0	0.0	0.0	0.0	0.0
TACGTAGGTG	0.0	0.0	0.0	0.0	0.0	0.0	0.0
TACGTAGGTG	0.0	0.0	0.0	0.0	0.0	0.0	0.0
TACGTATGGT	0.0	0.0	0.0	0.0	0.0	0.0	0.0
TACGGAGGAT	0.0	0.0	0.0	0.0	0.0	0.0	0.0
TACGGAGGAT	0.0	0.0	0.0	0.0	0.0	0.0	0.0
AACGTAGGTC	0.0	0.0	0.0	0.0	0.0	0.0	0.0
TACGTATGGT	0.0	0.0	0.0	0.0	0.0	0.0	0.0
TACGTAGGGG	0.0	0.0	0.0	0.0	0.0	0.0	0.0
TACGTAGGGG	0.0	0.0	0.0	0.0	0.0	0.0	0.0
TACGTAGGGG	0.0	0.0	0.0	0.0	0.0	0.0	0.0
TACGTAGGTG	0.0	0.0	0.0	0.0	0.0	0.0	0.0
TACGTAGGGG	0.0	0.0	0.0	0.0	0.0	0.0	0.0
TACGTAGGTG	0.0	0.0	0.0	0.0	0.0	0.0	0.0
TACGTAGGGG	0.0	0.0	0.0	0.0	0.0	0.0	0.0
TACGTAGGTG	0.0	0.0	0.0	0.0	0.0	0.0	0.0
TACGTAGGGG	0.0	0.0	0.0	0.0	0.0	0.0	0.0
TACGTAGGTG	0.0	0.0	0.0	0.0	0.0	0.0	0.0
TACGTAGGGG	0.0	0.0	0.0	0.0	0.0	0.0	0.0
TACGTAGGGT	0.0	0.0	0.0	0.0	0.0	0.0	0.0

TACGTAGGGG	0.0	0.0	0.0	0.0	0.0	0.0	0.0
TACGTAGGAT	0.0	0.0	0.0	0.0	0.0	0.0	0.0
TACGTATGGT	0.0	0.0	0.0	0.0	0.0	0.0	0.0
TACGTAGGGG	0.0	0.0	0.0	0.0	0.0	0.0	0.0
TACGTAGGGT	0.0	0.0	0.0	0.0	0.0	0.0	0.0
TACGTATGTC	0.0	0.0	0.0	0.0	0.0	0.0	0.0
TACGGAGGGT	0.0	0.0	0.0	0.0	0.0	0.0	0.0
TACGTAGGTG	0.0	0.0	0.0	0.0	0.0	0.0	0.0
TACGTAGGGC	0.0	0.0	0.0	0.0	0.0	0.0	0.0
TACGTAGGTG	0.0	0.0	0.0	0.0	0.0	0.0	0.0
TACGTAGGTG	0.0	0.0	0.0	0.0	0.0	0.0	0.0
TTCTTCTTTT	0.0	0.0	0.0	0.0	0.0	0.0	0.0
TACGTAGGGC	0.0	0.0	0.0	0.0	0.0	0.0	0.0
TACGTAGGTG	0.0	0.0	0.0	0.0	0.0	0.0	0.0
TACGTAGGGG	0.0	0.0	0.0	0.0	0.0	0.0	0.0
TACGTAGGGG	0.0	0.0	0.0	0.0	0.0	0.0	0.0
TACGTATGGA	0.0	0.0	0.0	0.0	0.0	0.0	0.0
TACGTAGGGG	0.0	0.0	0.0	0.0	0.0	0.0	0.0
TACGTAGGGG	0.0	0.0	0.0	0.0	0.0	0.0	0.0
TACGTAGGGG	0.0	0.0	0.0	0.0	0.0	0.0	0.0
TACGTAGGGG	0.0	0.0	0.0	0.0	0.0	0.0	0.0
TACGTAGGGC	0.0	0.0	0.0	0.0	0.0	0.0	0.0
TACGTAGGTG	0.0	0.0	0.0	0.0	0.0	0.0	0.0
TACGTAGGGT	0.0	0.0	0.0	0.0	0.0	0.0	0.0
TACGTAGGTG	0.0	0.0	0.0	0.0	0.0	0.0	0.0
TACGTAGGGG	0.0	0.0	0.0	0.0	0.0	0.0	0.0
TACGTAGGGG	0.0	0.0	0.0	0.0	0.0	0.0	0.0
TACGTATGGT	0.0	0.0	0.0	0.0	0.0	0.0	0.0
TACGTAGGTG	0.0	0.0	0.0	0.0	0.0	0.0	0.0
AACGTAGGTC	0.0	0.0	0.0	0.0	0.0	0.0	0.0
TACAGAGGTC	0.0	0.0	0.0	0.0	0.0	0.0	0.0
TACGTAGGGG	0.0	0.0	0.0	0.0	0.0	0.0	0.0
AACGTAGGGT	0.0	0.0	0.0	0.0	0.0	0.0	0.0
AACGTAGGGT	0.0	0.0	0.0	0.0	0.0	0.0	0.0
TACGTAGGGG	0.0	0.0	0.0	0.0	0.0	0.0	0.0
TACGGAGGGA	0.0	0.0	0.0	0.0	0.0	0.0	0.0
TACGTATGGT	0.0	0.0	0.0	0.0	0.0	0.0	0.0
TACGTAGGGA	0.0	0.0	0.0	0.0	0.0	0.0	0.0
TACGTATGGT	0.0	0.0	0.0	0.0	0.0	0.0	0.0
TACGTAGGGG	0.0	0.0	0.0	0.0	0.0	0.0	0.0
TACGGAAGAT	0.0	0.0	0.0	0.0	0.0	0.0	0.0
AACGTAGGTG	0.0	0.0	0.0	0.0	0.0	0.0	0.0
TACGTACGGA	0.0	0.0	0.0	0.0	0.0	0.0	0.0
TACGTAGGGA	0.0	0.0	0.0	0.0	0.0	0.0	0.0
TACGTAGGTG	0.0	0.0	0.0	0.0	0.0	0.0	0.0
TACGTATGGA	0.0	0.0	0.0	0.0	0.0	0.0	0.0
TACGTAGGTG	0.0	0.0	0.0	0.0	0.0	0.0	0.0

TACGTAGGGT	0.0	0.0	0.0	0.0	0.0	0.0	0.0
TACGTAGGTG	0.0	0.0	0.0	0.0	0.0	0.0	0.0
TACGTAGGGA	0.0	0.0	0.0	0.0	0.0	0.0	0.0
TACGTAGGGA	0.0	0.0	0.0	0.0	0.0	0.0	0.0
TACGGAGGAT	0.0	0.0	0.0	0.0	0.0	0.0	0.0
TACGTAGGTG	0.0	0.0	0.0	0.0	0.0	0.0	0.0
TACGTATGGT	0.0	0.0	0.0	0.0	0.0	0.0	0.0
TACGTAGGGA	0.0	0.0	0.0	0.0	0.0	0.0	0.0
TACGTAGGTG	0.0	0.0	0.0	0.0	0.0	0.0	0.0
TACGTAGGGT	0.0	0.0	0.0	0.0	0.0	0.0	0.0
TACGTAGGGG	0.0	0.0	0.0	0.0	0.0	0.0	0.0
TACGTAGGGG	0.0	0.0	0.0	0.0	0.0	0.0	0.0
TACGTAGGGG	0.0	0.0	0.0	0.0	0.0	0.0	0.0
TACGGAGGAT	0.0	0.0	0.0	0.0	0.0	0.0	0.0
TACGTAGGGG	0.0	0.0	0.0	0.0	0.0	0.0	0.0
TACGTAGGGG	0.0	0.0	0.0	0.0	0.0	0.0	0.0
TACGTAGGGG	0.0	0.0	0.0	0.0	0.0	0.0	0.0
TACGTAGGTG	0.0	0.0	0.0	0.0	0.0	0.0	0.0
TACGTATGGT	0.0	0.0	0.0	0.0	0.0	0.0	0.0
TACGTATGGT	0.0	0.0	0.0	0.0	0.0	0.0	0.0
TACGTAGGTG	0.0	0.0	0.0	0.0	0.0	0.0	0.0
TACGTAGGGG	0.0	0.0	0.0	0.0	0.0	0.0	0.0
AACGTAGGGT	0.0	0.0	0.0	0.0	0.0	0.0	0.0
TACGTAGGGG	0.0	0.0	0.0	0.0	0.0	0.0	0.0
TACGGAGGAT	0.0	0.0	0.0	0.0	0.0	0.0	0.0
TACGTAGGGG	0.0	0.0	0.0	0.0	0.0	0.0	0.0
TCCTTCTTTTT	0.0	0.0	0.0	0.0	0.0	0.0	0.0
TACGTATGGA	0.0	0.0	0.0	0.0	0.0	0.0	0.0
AACGTAGGGT	0.0	0.0	0.0	0.0	0.0	0.0	0.0
TACAGAGGAT	0.0	0.0	0.0	0.0	0.0	0.0	0.0
TACGTAGGGG	0.0	0.0	0.0	0.0	0.0	0.0	0.0
TACGTAGGGG	0.0	0.0	0.0	0.0	0.0	0.0	0.0
TACGTAGGGG	0.0	0.0	0.0	0.0	0.0	0.0	0.0
TACGTAGGGG	0.0	0.0	0.0	0.0	0.0	0.0	0.0
TACGTAGGGG	0.0	0.0	0.0	0.0	0.0	0.0	0.0
TACGTAGGGG	0.0	0.0	0.0	0.0	0.0	0.0	0.0
AACGCAGGGT	0.0	0.0	0.0	0.0	0.0	0.0	0.0
TACTTAGGTG	0.0	0.0	0.0	0.0	0.0	0.0	0.0
AACGTAGGGT	0.0	0.0	0.0	0.0	0.0	0.0	0.0
TACGTAGGGG	0.0	0.0	0.0	0.0	0.0	0.0	0.0
TACGTAGGTG	0.0	0.0	0.0	0.0	0.0	0.0	0.0
TACGTAGGGG	0.0	0.0	0.0	0.0	0.0	0.0	0.0
AACGTAGGTC	0.0	0.0	0.0	0.0	0.0	0.0	0.0
TACGGAGGGG	0.0	0.0	0.0	0.0	0.0	0.0	0.0
TACGTAGGGG	0.0	0.0	0.0	0.0	0.0	0.0	0.0
TACGTAGGGG	0.0	0.0	0.0	0.0	0.0	0.0	0.0
TCCTTCTTTTT	0.0	0.0	0.0	0.0	0.0	0.0	0.0

TACGTAGGTG	0.0	0.0	0.0	0.0	0.0	0.0	0.0
TACGTAGGTG	0.0	0.0	0.0	0.0	0.0	0.0	0.0
TACGTAGGGG	0.0	0.0	0.0	0.0	0.0	0.0	0.0
TACGTAGTGA	0.0	0.0	0.0	0.0	0.0	0.0	0.0
TACGTAGGTG	0.0	0.0	0.0	0.0	0.0	0.0	0.0
TACGTAGGGG	0.0	0.0	0.0	0.0	0.0	0.0	0.0
TACGTAGGTG	0.0	0.0	0.0	0.0	0.0	0.0	0.0
TACGTATGGG	0.0	0.0	0.0	0.0	0.0	0.0	0.0
AACGTAGGGT	0.0	0.0	0.0	0.0	0.0	0.0	0.0
TACGTAGGTG	0.0	0.0	0.0	0.0	0.0	0.0	0.0
TACGTATGGA	0.0	0.0	0.0	0.0	0.0	0.0	0.0
TACGTAGGGG	0.0	0.0	0.0	0.0	0.0	0.0	0.0
AACGTAGGTG	0.0	0.0	0.0	0.0	0.0	0.0	0.0
TACGTAGGTG	0.0	0.0	0.0	0.0	0.0	0.0	0.0
TACGTAGATG	0.0	0.0	0.0	0.0	0.0	0.0	0.0
TACGTAGGGG	0.0	0.0	0.0	0.0	0.0	0.0	0.0
TACGTAGGGG	0.0	0.0	0.0	0.0	0.0	0.0	0.0
AACGTAGGTG	0.0	0.0	0.0	0.0	0.0	0.0	0.0
TACGTAGGGG	0.0	0.0	0.0	0.0	0.0	0.0	0.0
AACGTAGGGT	0.0	0.0	0.0	0.0	0.0	0.0	0.0
TACGTAGGGG	0.0	0.0	0.0	0.0	0.0	0.0	0.0
TACGTATGGG	0.0	0.0	0.0	0.0	0.0	0.0	0.0
TACGGAGGAT	0.0	0.0	0.0	0.0	0.0	0.0	0.0
TACGGAGGAT	0.0	0.0	0.0	0.0	0.0	0.0	0.0
TACGGAGACT	0.0	0.0	0.0	0.0	0.0	0.0	0.0
TACGTAGGTG	0.0	0.0	0.0	0.0	0.0	0.0	0.0
TACGTAGGGA	0.0	0.0	0.0	0.0	0.0	0.0	0.0
AACGTAGGGT	0.0	0.0	0.0	0.0	0.0	0.0	0.0
AACGTAGGTC	0.0	0.0	0.0	0.0	0.0	0.0	0.0
TACGTAGGTG	0.0	0.0	0.0	0.0	0.0	0.0	0.0
TACGTAGGGG	0.0	0.0	0.0	0.0	0.0	0.0	0.0
TACGTAGGTG	0.0	0.0	0.0	0.0	0.0	0.0	0.0
TACGTAGGGG	0.0	0.0	0.0	0.0	0.0	0.0	0.0
TACGGAGGAT	0.0	0.0	0.0	0.0	0.0	0.0	0.0
TACGTAGGGG	0.0	0.0	0.0	0.0	0.0	0.0	0.0
TACGTAGGGA	0.0	0.0	0.0	0.0	0.0	0.0	0.0
AACGTAGGGT	0.0	0.0	0.0	0.0	0.0	0.0	0.0
TACGGAGGAT	0.0	0.0	0.0	0.0	0.0	0.0	0.0
TACGTAGGGA	0.0	0.0	0.0	0.0	0.0	0.0	0.0
TACGGAAGAT	0.0	0.0	0.0	0.0	0.0	0.0	0.0
TACGTAGGGA	0.0	0.0	0.0	0.0	0.0	0.0	0.0
TACGTATGGT	0.0	0.0	0.0	0.0	0.0	0.0	0.0
TACGTAGGGG	0.0	0.0	0.0	0.0	0.0	0.0	0.0
AACGTAGGTC	0.0	0.0	0.0	0.0	0.0	0.0	0.0
TACGTAGGGG	0.0	0.0	0.0	0.0	0.0	0.0	0.0
AACGTAGGTC	0.0	0.0	0.0	0.0	0.0	0.0	0.0
TACGTATGGT	0.0	0.0	0.0	0.0	0.0	0.0	0.0

TACGTAGGGT	0.0	0.0	0.0	0.0	0.0	0.0	0.0
TACGTAGGGG	0.0	0.0	0.0	0.0	0.0	0.0	0.0
TACGTATGGT	0.0	0.0	0.0	0.0	0.0	0.0	0.0
TACGTAGGGG	0.0	0.0	0.0	0.0	0.0	0.0	0.0
TACGTAGGGG	0.0	0.0	0.0	0.0	0.0	0.0	0.0
TACTTCTTTTT	0.0	0.0	0.0	0.0	0.0	0.0	0.0
TACGTAGGGA	0.0	0.0	0.0	0.0	0.0	0.0	0.0
TACGTAGGTG	0.0	0.0	0.0	0.0	0.0	0.0	0.0
TACGTAGGTT	0.0	0.0	0.0	0.0	0.0	0.0	0.0
TACGTAGGGA	0.0	0.0	0.0	0.0	0.0	0.0	0.0
TACGTATGGT	0.0	0.0	0.0	0.0	0.0	0.0	0.0
TACGTAGGGA	0.0	0.0	0.0	0.0	0.0	0.0	0.0
TACGTAGGGG	0.0	0.0	0.0	0.0	0.0	0.0	0.0
TACGGAGGAT	0.0	0.0	0.0	0.0	0.0	0.0	0.0
TACGTAGGGA	0.0	0.0	0.0	0.0	0.0	0.0	0.0
TACGTAGGTG	0.0	0.0	0.0	0.0	0.0	0.0	0.0
TACGTAGGTG	0.0	0.0	0.0	0.0	0.0	0.0	0.0
TACGGAGGAT	0.0	0.0	0.0	0.0	0.0	0.0	0.0
TACGTAGGTG	0.0	0.0	0.0	0.0	0.0	0.0	0.0
TACGGAGGGT	0.0	0.0	0.0	0.0	0.0	0.0	0.0
TACGTAGGTG	0.0	0.0	0.0	0.0	0.0	0.0	0.0
GACGGGGGG	0.0	0.0	0.0	0.0	0.0	0.0	0.0
GACAGAGGAT	0.0	0.0	0.0	0.0	0.0	0.0	0.0
TACAGAGGGT	0.0	0.0	0.0	0.0	0.0	0.0	0.0
GACGGGGGG	0.0	0.0	0.0	0.0	0.0	0.0	0.0
GACAGAGGAT	0.0	0.0	0.0	0.0	0.0	0.0	0.0
TCCGTCTTTTT	0.0	0.0	0.0	0.0	0.0	0.0	0.0
GACGGGGGG	0.0	0.0	0.0	0.0	0.0	0.0	0.0
TACGTAGGTG	0.0	0.0	0.0	0.0	0.0	0.0	0.0
AACGTAGGGT	0.0	0.0	0.0	0.0	0.0	0.0	0.0
TACGTAGGGG	0.0	0.0	0.0	0.0	0.0	0.0	0.0
TACGTAGGGG	0.0	0.0	0.0	0.0	0.0	0.0	0.0
TACGTAGGGC	0.0	0.0	0.0	0.0	0.0	0.0	0.0
TACGTAGGTC	0.0	0.0	0.0	0.0	0.0	0.0	0.0
TACGTATGGA	0.0	0.0	0.0	0.0	0.0	0.0	0.0
TACGTAGGTG	0.0	0.0	0.0	0.0	0.0	0.0	0.0
TACGTAGGTG	0.0	0.0	0.0	0.0	0.0	0.0	0.0
TACGTAGGGG	0.0	0.0	0.0	0.0	0.0	0.0	0.0
TGCGTAGGTC	0.0	0.0	0.0	0.0	0.0	0.0	0.0
TACGTAGGGA	0.0	0.0	0.0	0.0	0.0	0.0	0.0
TACGTAGGGG	0.0	0.0	0.0	0.0	0.0	0.0	0.0
TACGGAGGGT	0.0	0.0	0.0	0.0	0.0	0.0	0.0
TACGTAGGGG	0.0	0.0	0.0	0.0	0.0	0.0	0.0
TACGGAGGAT	0.0	0.0	0.0	0.0	0.0	0.0	0.0
TACGTAGGGG	0.0	0.0	0.0	0.0	0.0	0.0	0.0
TACGTAGGTG	0.0	0.0	0.0	0.0	0.0	0.0	0.0
TACGTAGGGA	0.0	0.0	0.0	0.0	0.0	0.0	0.0

TACGTAGGGG	0.0	0.0	0.0	0.0	0.0	0.0	0.0
TACGTAGGGA	0.0	0.0	0.0	0.0	0.0	0.0	0.0
TACGTAGGGG	0.0	0.0	0.0	0.0	0.0	0.0	0.0
TACGTAGGTG	0.0	0.0	0.0	0.0	0.0	0.0	0.0
TACGTAGGTG	0.0	0.0	0.0	0.0	0.0	0.0	0.0
TACGTAGGGA	0.0	0.0	0.0	0.0	0.0	0.0	0.0
CACGTAGGTC	0.0	0.0	0.0	0.0	0.0	0.0	0.0
TACGTAGGGG	0.0	0.0	0.0	0.0	0.0	0.0	0.0
TACGTATGGA	0.0	0.0	0.0	0.0	0.0	0.0	0.0
TACGTAGGGG	0.0	0.0	0.0	0.0	0.0	0.0	0.0
TACATATGGA	0.0	0.0	0.0	0.0	0.0	0.0	0.0
TACGTAGGTG	0.0	0.0	0.0	0.0	0.0	0.0	0.0
TACGTAGGTG	0.0	0.0	0.0	0.0	0.0	0.0	0.0
TACGTAGGGG	0.0	0.0	0.0	0.0	0.0	0.0	0.0
TACGTAGGGT	0.0	0.0	0.0	0.0	0.0	0.0	0.0
TACGTAGGGA	0.0	0.0	0.0	0.0	0.0	0.0	0.0
TGCGTAGGTG	0.0	0.0	0.0	0.0	0.0	0.0	0.0
TACGTAGGGC	0.0	0.0	0.0	0.0	0.0	0.0	0.0
TACGTAGGGG	0.0	0.0	0.0	0.0	0.0	0.0	0.0
TACGGAGGAT	0.0	0.0	0.0	0.0	0.0	0.0	0.0
TACGGAAGGT	0.0	0.0	0.0	0.0	0.0	0.0	0.0
TACGGAGGAT	0.0	0.0	0.0	0.0	0.0	0.0	0.0
TACGGAAGGT	0.0	0.0	0.0	0.0	0.0	0.0	0.0
TACGTAGGGG	0.0	0.0	0.0	0.0	0.0	0.0	0.0
AACGTAGGGA	0.0	0.0	0.0	0.0	0.0	0.0	0.0
TACGTAGGGG	0.0	0.0	0.0	0.0	0.0	0.0	0.0
TACGTAGGTG	0.0	0.0	0.0	0.0	0.0	0.0	0.0
TACGTAGGTG	0.0	0.0	0.0	0.0	0.0	0.0	0.0
TACGTAGGGG	0.0	0.0	0.0	0.0	0.0	0.0	0.0
TACGTAGGGG	0.0	0.0	0.0	0.0	0.0	0.0	0.0
TACGTATGGG	0.0	0.0	0.0	0.0	0.0	0.0	0.0
AACGTAGGGT	0.0	0.0	0.0	0.0	0.0	0.0	0.0
TACGTAGGTG	0.0	0.0	0.0	0.0	0.0	0.0	0.0
AACGTAGGGC	0.0	0.0	0.0	0.0	0.0	0.0	0.0
TACGTAGGTG	0.0	0.0	0.0	0.0	0.0	0.0	0.0
TACGTATGGT	0.0	0.0	0.0	0.0	0.0	0.0	0.0
TACGGAAGAT	0.0	0.0	0.0	0.0	0.0	0.0	0.0
TACGTAGGTG	0.0	0.0	0.0	0.0	0.0	0.0	0.0
TACGTATGGA	0.0	0.0	0.0	0.0	0.0	0.0	0.0
TACGTAGGGA	0.0	0.0	0.0	0.0	0.0	0.0	0.0
TACGTAGGTG	0.0	0.0	0.0	0.0	0.0	0.0	0.0
TACGTAGGTA	0.0	0.0	0.0	0.0	0.0	0.0	0.0
TACGTAGGGG	0.0	0.0	0.0	0.0	0.0	0.0	0.0
TACGTAGGTG	0.0	0.0	0.0	0.0	0.0	0.0	0.0
TACGTAGGGA	0.0	0.0	0.0	0.0	0.0	0.0	0.0
TACGTAGGGA	0.0	0.0	0.0	0.0	0.0	0.0	0.0
TACGTAGGGA	0.0	0.0	0.0	0.0	0.0	0.0	0.0

TACGTAGGTG	0.0	0.0	0.0	0.0	0.0	0.0	0.0
TACGTAGGGA	0.0	0.0	0.0	0.0	0.0	0.0	0.0
TACGTATGGA	0.0	0.0	0.0	0.0	0.0	0.0	0.0
TACGTAGGGG	0.0	0.0	0.0	0.0	0.0	0.0	0.0
TACGGAGGAT	0.0	0.0	0.0	0.0	0.0	0.0	0.0
CACCGGCAGC	0.0	0.0	0.0	0.0	0.0	0.0	0.0
TACGTAGGGG	0.0	0.0	0.0	0.0	0.0	0.0	0.0
TACGTAGGGG	0.0	0.0	0.0	0.0	0.0	0.0	0.0
TACGTAGGGA	0.0	0.0	0.0	0.0	0.0	0.0	0.0
TACGTAGGGG	0.0	0.0	0.0	0.0	0.0	0.0	0.0
TACGTAGGGG	0.0	0.0	0.0	0.0	0.0	0.0	0.0
TACGTAGGGG	0.0	0.0	0.0	0.0	0.0	0.0	0.0
TACGGAGGAT	0.0	0.0	0.0	0.0	0.0	0.0	0.0
TACGTAGGTG	0.0	0.0	0.0	0.0	0.0	0.0	0.0
TACGGAGGAT	0.0	0.0	0.0	0.0	0.0	0.0	0.0
TACGTAGGTG	0.0	0.0	0.0	0.0	0.0	0.0	0.0
TACGTAGGGA	0.0	0.0	0.0	0.0	0.0	0.0	0.0
TACGTAGGGG	0.0	0.0	0.0	0.0	0.0	0.0	0.0
TACGGAGGAT	0.0	0.0	0.0	0.0	0.0	0.0	0.0
TACGTAGGGG	0.0	0.0	0.0	0.0	0.0	0.0	0.0
TACGGAGGAT	0.0	0.0	0.0	0.0	0.0	0.0	0.0
TACGTAGGTG	0.0	0.0	0.0	0.0	0.0	0.0	0.0
TACGTAGGTG	0.0	0.0	0.0	0.0	0.0	0.0	0.0
TACGTAGGGG	0.0	0.0	0.0	0.0	0.0	0.0	0.0
TACGTAGGTG	0.0	0.0	0.0	0.0	0.0	0.0	0.0
TACGTAGGGA	0.0	0.0	0.0	0.0	0.0	0.0	0.0
TACGTAGGGA	0.0	0.0	0.0	0.0	0.0	0.0	0.0
TACGTAGGGA	0.0	0.0	0.0	0.0	0.0	0.0	0.0
TACGTAGGTG	0.0	0.0	0.0	0.0	0.0	0.0	0.0
TACGTAGGGA	0.0	0.0	0.0	0.0	0.0	0.0	0.0
TACGTAGGGG	0.0	0.0	0.0	0.0	0.0	0.0	0.0
TACGTAGGGA	0.0	0.0	0.0	0.0	0.0	0.0	0.0
TACGTAGGGG	0.0	0.0	0.0	0.0	0.0	0.0	0.0
TACGTAGGGA	0.0	0.0	0.0	0.0	0.0	0.0	0.0
TACGTATGGT	0.0	0.0	0.0	0.0	0.0	0.0	0.0
TACGTAGGGT	0.0	0.0	0.0	0.0	0.0	0.0	0.0
TACGTAGGGG	0.0	0.0	0.0	0.0	0.0	0.0	0.0
TACGTAGGGG	0.0	0.0	0.0	0.0	0.0	0.0	0.0
TACGTAGGTG	0.0	0.0	0.0	0.0	0.0	0.0	0.0
TACGTATGGT	0.0	0.0	0.0	0.0	0.0	0.0	0.0
TACGTAGGGG	0.0	0.0	0.0	0.0	0.0	0.0	0.0
TACGTAGGGA	0.0	0.0	0.0	0.0	0.0	0.0	0.0
TACGTATGGT	0.0	0.0	0.0	0.0	0.0	0.0	0.0
TACGTATGGT	0.0	0.0	0.0	0.0	0.0	0.0	0.0
TACGTAGGGG	0.0	0.0	0.0	0.0	0.0	0.0	0.0
TACGTAGGTG	0.0	0.0	0.0	0.0	0.0	0.0	0.0
AACGTAGGTC	0.0	0.0	0.0	0.0	0.0	0.0	0.0

GACAGAGGAT	0.0	0.0	0.0	0.0	0.0	0.0	0.0
AACGTAGGTC	0.0	0.0	0.0	0.0	0.0	0.0	0.0
TACGTATGGT	0.0	0.0	0.0	0.0	0.0	0.0	0.0
TACGTAGGAA	0.0	0.0	0.0	0.0	0.0	0.0	0.0
TACGTAGGTG	0.0	0.0	0.0	0.0	0.0	0.0	0.0
TACGTAGGGG	0.0	0.0	0.0	0.0	0.0	0.0	0.0
TACGTAGGGA	0.0	0.0	0.0	0.0	0.0	0.0	0.0
TACGTATGGT	0.0	0.0	0.0	0.0	0.0	0.0	0.0
AACGTAGGGT	0.0	0.0	0.0	0.0	0.0	0.0	0.0
TACGTAGGGG	0.0	0.0	0.0	0.0	0.0	0.0	0.0
AACGTAGGGT	0.0	0.0	0.0	0.0	0.0	0.0	0.0
TACGTATGGA	0.0	0.0	0.0	0.0	0.0	0.0	0.0
TACGTAGGGG	0.0	0.0	0.0	0.0	0.0	0.0	0.0
TACGTAGGGG	0.0	0.0	0.0	0.0	0.0	0.0	0.0
TACGTAGGGA	0.0	0.0	0.0	0.0	0.0	0.0	0.0
TACGTAGGGG	0.0	0.0	0.0	0.0	0.0	0.0	0.0
TACGTATGGT	0.0	0.0	0.0	0.0	0.0	0.0	0.0
TACGGAGGAT	0.0	0.0	0.0	0.0	0.0	0.0	0.0
TACGTATGGT	0.0	0.0	0.0	0.0	0.0	0.0	0.0
TACGTAGGTG	0.0	0.0	0.0	0.0	0.0	0.0	0.0
TACGTAGGTG	0.0	0.0	0.0	0.0	0.0	0.0	0.0
TACGTAGGGT	0.0	0.0	0.0	0.0	0.0	0.0	0.0
TACGTAGGTG	0.0	0.0	0.0	0.0	0.0	0.0	0.0
TACGTATGGG	0.0	0.0	0.0	0.0	0.0	0.0	0.0
TACGTAGGGA	0.0	0.0	0.0	0.0	0.0	0.0	0.0
TACGGAGGGT	0.0	0.0	0.0	0.0	0.0	0.0	0.0
TACGTAGGTG	0.0	0.0	0.0	0.0	0.0	0.0	0.0
TACGTAGGGG	0.0	0.0	0.0	0.0	0.0	0.0	0.0
TACGTAGGTG	0.0	0.0	0.0	0.0	0.0	0.0	0.0
TACGTAGGGA	0.0	0.0	0.0	0.0	0.0	0.0	0.0
TACGTAAGGG	0.0	0.0	0.0	0.0	0.0	0.0	0.0
TACGTAGGTG	0.0	0.0	0.0	0.0	0.0	0.0	0.0
TACGTAGGGA	0.0	0.0	0.0	0.0	0.0	0.0	0.0
TACGTAGGTG	0.0	0.0	0.0	0.0	0.0	0.0	0.0
TACGTAGGGA	0.0	0.0	0.0	0.0	0.0	0.0	0.0
TACGTAGGGG	0.0	0.0	0.0	0.0	0.0	0.0	0.0
TACGTAGGGA	0.0	0.0	0.0	0.0	0.0	0.0	0.0
TACGTAGGTG	0.0	0.0	0.0	0.0	0.0	0.0	0.0
AACGTAGGGT	0.0	0.0	0.0	0.0	0.0	0.0	0.0
TACGTAGGTG	0.0	0.0	0.0	0.0	0.0	0.0	0.0
TACGTATGGT	0.0	0.0	0.0	0.0	0.0	0.0	0.0
TACGTAAGGG	0.0	0.0	0.0	0.0	0.0	0.0	0.0
TACGTAGGGG	0.0	0.0	0.0	0.0	0.0	0.0	0.0
TACGAAGGG	0.0	0.0	0.0	0.0	0.0	0.0	0.0
AACGTAGGTC	0.0	0.0	0.0	0.0	0.0	0.0	0.0
AACGTAGGTC	0.0	0.0	0.0	0.0	0.0	0.0	0.0
TACGTAGGGA	0.0	0.0	0.0	0.0	0.0	0.0	0.0

AACGTAGGGT	0.0	0.0	0.0	0.0	0.0	0.0	0.0
AACGTAGGTC	0.0	0.0	0.0	0.0	0.0	0.0	0.0
TACGTAGGGG	0.0	0.0	0.0	0.0	0.0	0.0	0.0
TACGTAGGGG	0.0	0.0	0.0	0.0	0.0	0.0	0.0
TACGTAGATG	0.0	0.0	0.0	0.0	0.0	0.0	0.0
TACGTATGGT	0.0	0.0	0.0	0.0	0.0	0.0	0.0
TACGTAGGGG	0.0	0.0	0.0	0.0	0.0	0.0	0.0
TACGTAGGGG	0.0	0.0	0.0	0.0	0.0	0.0	0.0
TACGTATGGT	0.0	0.0	0.0	0.0	0.0	0.0	0.0
TACGTAGGGA	0.0	0.0	0.0	0.0	0.0	0.0	0.0
TACGTAGGGG	0.0	0.0	0.0	0.0	0.0	0.0	0.0
TACGTAGGGG	0.0	0.0	0.0	0.0	0.0	0.0	0.0
TACGTAGGTG	0.0	0.0	0.0	0.0	0.0	0.0	0.0
TACGTAGGTG	0.0	0.0	0.0	0.0	0.0	0.0	0.0
CACCGGCAGC	0.0	0.0	0.0	0.0	0.0	0.0	0.0
TACGTAGGTG	0.0	0.0	0.0	0.0	0.0	0.0	0.0
TACGTAGGGA	0.0	0.0	0.0	0.0	0.0	0.0	0.0
TACGGAGGAC	0.0	0.0	0.0	0.0	0.0	0.0	0.0
TACGTATGTG	0.0	0.0	0.0	0.0	0.0	0.0	0.0
AACGTAGGTC	0.0	0.0	0.0	0.0	0.0	0.0	0.0
AACGTAGGTG	0.0	0.0	0.0	0.0	0.0	0.0	0.0
TACGTAGGGG	0.0	0.0	0.0	0.0	0.0	0.0	0.0
TACGGAGGGT	0.0	0.0	0.0	0.0	0.0	0.0	0.0
TACGTAGGGG	0.0	0.0	0.0	0.0	0.0	0.0	0.0
TACGTAGGGG	0.0	0.0	0.0	0.0	0.0	0.0	0.0
TACGTAGGGG	0.0	0.0	0.0	0.0	0.0	0.0	0.0
TACGTAGGTG	0.0	0.0	0.0	0.0	0.0	0.0	0.0
TACGTAGGTG	0.0	0.0	0.0	0.0	0.0	0.0	0.0
TACGTAGGGA	0.0	0.0	0.0	0.0	0.0	0.0	0.0
TACGTAGGGT	0.0	0.0	0.0	0.0	0.0	0.0	0.0
TCCTTCTTTTT	0.0	0.0	0.0	0.0	0.0	0.0	0.0
AACGTAGGGT	0.0	0.0	0.0	0.0	0.0	0.0	0.0
TACGTAGGGG	0.0	0.0	0.0	0.0	0.0	0.0	0.0
TACGGAAGGT	0.0	0.0	0.0	0.0	0.0	0.0	0.0
TACGTAGGGG	0.0	0.0	0.0	0.0	0.0	0.0	0.0
TACGTAGGTG	0.0	0.0	0.0	0.0	0.0	0.0	0.0
TACGTAGGGT	0.0	0.0	0.0	0.0	0.0	0.0	0.0
TACGTAGGGG	0.0	0.0	0.0	0.0	0.0	0.0	0.0
AACGTAGGTC	0.0	0.0	0.0	0.0	0.0	0.0	0.0
TACGTATGGT	0.0	0.0	0.0	0.0	0.0	0.0	0.0
TACGTATGGT	0.0	0.0	0.0	0.0	0.0	0.0	0.0
TACGTAGGTG	0.0	0.0	0.0	0.0	0.0	0.0	0.0
TACGTATGGA	0.0	0.0	0.0	0.0	0.0	0.0	0.0
TACGTAGGTG	0.0	0.0	0.0	0.0	0.0	0.0	0.0
AACGTAGGTC	0.0	0.0	0.0	0.0	0.0	0.0	0.0
TACGTAGGTG	0.0	0.0	0.0	0.0	0.0	0.0	0.0
TACGTAGGTG	0.0	0.0	0.0	0.0	0.0	0.0	0.0

TACGTAGGGT	0.0	0.0	0.0	0.0	0.0	0.0	0.0
AACGTAGGTC	0.0	0.0	0.0	0.0	0.0	0.0	0.0
TAGGTAGGTG	0.0	0.0	0.0	0.0	0.0	0.0	0.0
TACGTATGGT	0.0	0.0	0.0	0.0	0.0	0.0	0.0
TACGTAGGGG	0.0	0.0	0.0	0.0	0.0	0.0	0.0
TACGGAGGGC	0.0	0.0	0.0	0.0	0.0	0.0	0.0
TACGGAGGAT	0.0	0.0	0.0	0.0	0.0	0.0	0.0
TACGTATGGT	0.0	0.0	0.0	0.0	0.0	0.0	0.0
TACGTATGGT	0.0	0.0	0.0	0.0	0.0	0.0	0.0
TACGTAGGGA	0.0	0.0	0.0	0.0	0.0	0.0	0.0
TACATAGGGG	0.0	0.0	0.0	0.0	0.0	0.0	0.0
TACGTAGGGG	0.0	0.0	0.0	0.0	0.0	0.0	0.0
TACGGAGGAT	0.0	0.0	0.0	0.0	0.0	0.0	0.0
TACGTAGGTG	0.0	0.0	0.0	0.0	0.0	0.0	0.0
TACGTAGGTG	0.0	0.0	0.0	0.0	0.0	0.0	0.0
TACGGAGGAT	0.0	0.0	0.0	0.0	0.0	0.0	0.0
TACGGAGGAT	0.0	0.0	0.0	0.0	0.0	0.0	0.0
TACGTATGGT	0.0	0.0	0.0	0.0	0.0	0.0	0.0
TACGGAGGAT	0.0	0.0	0.0	0.0	0.0	0.0	0.0
TACGTAGGGG	0.0	0.0	0.0	0.0	0.0	0.0	0.0
TACGTAGGGG	0.0	0.0	0.0	0.0	0.0	0.0	0.0
TACGTATGGT	0.0	0.0	0.0	0.0	0.0	0.0	0.0
TACGTAGGGG	0.0	0.0	0.0	0.0	0.0	0.0	0.0
age	45	25	69	58	30	78	64
sex	0	1	1	1	0	0	1
bmi	26.53667355	22.2	25.09422734	45.4667576	21.4	36.1	23.4

11635.TW.BG	11635.CIR63.(	11635.TW.BT.	11635.TW.CN	11635.TW.AF.	11635.CIR21.(	11635.TW.DF.	11635.TW.BL.
34.0	25.0	0.0	2.0	3.0	183.0	2.0	12.0
0.0	0.0	0.0	0.0	0.0	12.0	0.0	2.0
0.0	0.0	0.0	2.0	0.0	5.0	5.0	2.0
509.0	235.0	198.0	0.0	212.0	24.0	0.0	1.0
345.0	874.0	136.0	0.0	56.0	3.0	1.0	21.0
79.0	717.0	647.0	0.0	15.0	116.0	19.0	16.0
0.0	0.0	0.0	0.0	0.0	24.0	0.0	0.0
0.0	0.0	0.0	0.0	0.0	0.0	0.0	0.0
0.0	51.0	0.0	0.0	0.0	140.0	0.0	0.0
0.0	320.0	25.0	1.0	244.0	1077.0	2623.0	0.0
62.0	34.0	41.0	0.0	9.0	2.0	3.0	28.0
0.0	0.0	0.0	0.0	0.0	0.0	1.0	0.0
100.0	35.0	51.0	0.0	3.0	0.0	0.0	0.0
2.0	0.0	0.0	0.0	45.0	175.0	21.0	303.0
48.0	180.0	25.0	1.0	0.0	7.0	1.0	2.0
1803.0	64.0	381.0	0.0	64.0	7.0	8.0	30.0
0.0	32.0	46.0	0.0	1.0	0.0	1.0	0.0
3.0	0.0	0.0	0.0	2.0	66.0	0.0	55.0
91.0	240.0	83.0	3.0	397.0	635.0	1634.0	12.0
390.0	95.0	2201.0	0.0	456.0	256.0	147.0	6.0
249.0	8.0	120.0	0.0	96.0	0.0	109.0	0.0
0.0	0.0	0.0	0.0	0.0	17.0	0.0	0.0
16.0	22.0	52.0	0.0	67.0	0.0	1.0	0.0
0.0	0.0	3.0	0.0	0.0	0.0	0.0	0.0
0.0	279.0	0.0	0.0	0.0	0.0	1.0	0.0
30.0	41.0	6.0	0.0	139.0	507.0	158.0	10.0
0.0	6.0	0.0	0.0	0.0	0.0	0.0	0.0
160.0	33.0	6.0	0.0	0.0	3.0	0.0	0.0
0.0	1.0	0.0	0.0	0.0	57.0	4.0	0.0
0.0	12.0	54.0	0.0	7.0	67.0	4.0	0.0
48.0	68.0	5.0	0.0	558.0	63.0	142.0	67.0
11.0	23.0	21.0	0.0	107.0	296.0	324.0	19.0
128.0	0.0	8.0	0.0	1035.0	9.0	741.0	0.0
0.0	40.0	23.0	0.0	0.0	0.0	0.0	0.0
18.0	38.0	25.0	0.0	57.0	183.0	0.0	0.0
4.0	0.0	0.0	0.0	0.0	0.0	0.0	0.0
0.0	9.0	0.0	0.0	0.0	47.0	0.0	0.0
51.0	101.0	11.0	0.0	632.0	353.0	515.0	7.0
0.0	0.0	0.0	0.0	0.0	6.0	0.0	0.0
24.0	19.0	10.0	0.0	66.0	67.0	2.0	1.0
205.0	46.0	40.0	0.0	4.0	2.0	0.0	2.0
0.0	26.0	28.0	0.0	0.0	0.0	2.0	0.0
6.0	7.0	0.0	0.0	49.0	88.0	122.0	38.0
242.0	42.0	116.0	1.0	821.0	160.0	398.0	26.0
4.0	6.0	1.0	0.0	5.0	18.0	1.0	1.0
13.0	85.0	36.0	0.0	104.0	181.0	327.0	13.0

1.0	5.0	28.0	1.0	155.0	103.0	21.0	10.0
12.0	0.0	9.0	0.0	0.0	112.0	0.0	0.0
0.0	8.0	0.0	0.0	0.0	9.0	2.0	0.0
0.0	0.0	0.0	0.0	0.0	0.0	0.0	0.0
0.0	0.0	0.0	0.0	0.0	0.0	0.0	0.0
37.0	2.0	21.0	0.0	0.0	16.0	12.0	1.0
37.0	10.0	87.0	0.0	5.0	11.0	0.0	1.0
0.0	0.0	0.0	0.0	0.0	0.0	0.0	0.0
86.0	0.0	0.0	0.0	0.0	0.0	1.0	21.0
0.0	170.0	0.0	0.0	0.0	2.0	0.0	0.0
0.0	4.0	0.0	0.0	7.0	0.0	5.0	0.0
1.0	5.0	0.0	0.0	15.0	20.0	8.0	6.0
0.0	1.0	0.0	0.0	0.0	0.0	0.0	0.0
0.0	0.0	0.0	0.0	0.0	0.0	0.0	0.0
77.0	6.0	78.0	0.0	51.0	22.0	0.0	0.0
0.0	0.0	0.0	0.0	0.0	0.0	0.0	0.0
0.0	0.0	0.0	0.0	0.0	0.0	0.0	0.0
0.0	0.0	0.0	0.0	0.0	0.0	0.0	0.0
30.0	21.0	6.0	0.0	139.0	37.0	13.0	0.0
0.0	0.0	0.0	0.0	35.0	0.0	0.0	0.0
0.0	0.0	0.0	0.0	0.0	0.0	0.0	0.0
0.0	0.0	0.0	0.0	2.0	25.0	7.0	0.0
0.0	16.0	0.0	0.0	0.0	0.0	0.0	0.0
15.0	33.0	94.0	0.0	0.0	113.0	77.0	15.0
5.0	334.0	155.0	0.0	38.0	0.0	0.0	0.0
0.0	0.0	34.0	0.0	18.0	2.0	0.0	1.0
0.0	0.0	0.0	0.0	0.0	0.0	0.0	0.0
0.0	0.0	0.0	0.0	0.0	0.0	0.0	0.0
5.0	0.0	0.0	0.0	0.0	0.0	0.0	0.0
0.0	0.0	0.0	0.0	0.0	0.0	0.0	0.0
0.0	0.0	0.0	0.0	0.0	0.0	0.0	0.0
0.0	0.0	0.0	0.0	0.0	0.0	0.0	0.0
0.0	0.0	36.0	0.0	0.0	0.0	1.0	0.0
0.0	0.0	0.0	0.0	0.0	0.0	0.0	0.0
191.0	0.0	163.0	0.0	568.0	8.0	12.0	25.0
0.0	0.0	0.0	2.0	0.0	0.0	0.0	0.0
2.0	3.0	48.0	0.0	3.0	0.0	6.0	0.0
0.0	0.0	0.0	0.0	0.0	0.0	0.0	0.0
130.0	7.0	231.0	0.0	0.0	0.0	477.0	160.0
37.0	14.0	0.0	0.0	3.0	0.0	1.0	7.0
25.0	159.0	25.0	0.0	101.0	115.0	0.0	0.0
0.0	0.0	0.0	0.0	0.0	0.0	0.0	0.0
129.0	0.0	61.0	0.0	0.0	0.0	2.0	0.0
101.0	0.0	40.0	0.0	83.0	16.0	8.0	6.0
3.0	0.0	18.0	0.0	34.0	0.0	0.0	0.0
0.0	0.0	0.0	0.0	0.0	0.0	0.0	0.0
1.0	25.0	0.0	0.0	4.0	21.0	1.0	0.0

38.0	43.0	53.0	1.0	106.0	35.0	123.0	1.0
2.0	0.0	0.0	0.0	14.0	0.0	0.0	1.0
192.0	3.0	85.0	0.0	625.0	223.0	111.0	84.0
0.0	0.0	0.0	0.0	0.0	0.0	0.0	0.0
3.0	0.0	0.0	0.0	0.0	0.0	1.0	2.0
0.0	0.0	0.0	0.0	0.0	0.0	0.0	0.0
34.0	2.0	18.0	0.0	1.0	0.0	2.0	3.0
0.0	0.0	0.0	0.0	0.0	0.0	0.0	0.0
52.0	13.0	134.0	0.0	97.0	1.0	0.0	0.0
0.0	0.0	0.0	0.0	0.0	0.0	0.0	0.0
8.0	51.0	7.0	0.0	5.0	6.0	0.0	0.0
0.0	0.0	0.0	0.0	0.0	0.0	0.0	0.0
0.0	6.0	8.0	0.0	0.0	0.0	1.0	0.0
0.0	0.0	0.0	0.0	0.0	543.0	1.0	0.0
0.0	74.0	16.0	0.0	1.0	1.0	0.0	3.0
0.0	0.0	0.0	0.0	0.0	0.0	0.0	0.0
0.0	0.0	0.0	0.0	0.0	0.0	0.0	0.0
0.0	0.0	0.0	0.0	0.0	0.0	0.0	0.0
0.0	247.0	70.0	0.0	0.0	57.0	0.0	0.0
0.0	0.0	0.0	0.0	0.0	0.0	0.0	0.0
34.0	14.0	58.0	1.0	48.0	9.0	21.0	0.0
0.0	0.0	0.0	0.0	0.0	0.0	0.0	2.0
20.0	7.0	33.0	0.0	0.0	2.0	0.0	0.0
0.0	0.0	0.0	0.0	0.0	0.0	0.0	0.0
0.0	0.0	0.0	0.0	0.0	0.0	0.0	0.0
0.0	0.0	10.0	0.0	0.0	0.0	0.0	0.0
12.0	0.0	10.0	0.0	12.0	116.0	3.0	0.0
0.0	0.0	127.0	0.0	0.0	225.0	0.0	1.0
0.0	1.0	23.0	0.0	0.0	7.0	0.0	0.0
0.0	0.0	0.0	0.0	0.0	0.0	6.0	0.0
0.0	3.0	3.0	0.0	0.0	0.0	0.0	0.0
0.0	56.0	0.0	0.0	0.0	0.0	0.0	0.0
15.0	6.0	0.0	0.0	0.0	0.0	3.0	0.0
0.0	0.0	0.0	0.0	0.0	0.0	0.0	0.0
0.0	0.0	0.0	0.0	0.0	0.0	0.0	0.0
0.0	7.0	14.0	0.0	1.0	7.0	0.0	0.0
0.0	0.0	0.0	0.0	0.0	0.0	0.0	0.0
23.0	11.0	0.0	0.0	20.0	0.0	1.0	0.0
392.0	169.0	80.0	0.0	4.0	10.0	0.0	2.0
0.0	0.0	0.0	0.0	0.0	0.0	0.0	0.0
0.0	0.0	0.0	0.0	0.0	0.0	0.0	0.0
650.0	0.0	348.0	2.0	0.0	2.0	4.0	0.0
0.0	0.0	0.0	0.0	0.0	0.0	0.0	0.0
0.0	2.0	23.0	0.0	0.0	0.0	0.0	0.0
45.0	0.0	3.0	0.0	2.0	0.0	0.0	4.0
0.0	0.0	0.0	0.0	0.0	0.0	1.0	0.0
37.0	6.0	0.0	0.0	0.0	0.0	1.0	1.0

0.0	0.0	0.0	0.0	0.0	0.0	0.0	0.0
0.0	31.0	0.0	0.0	2.0	0.0	0.0	0.0
4.0	0.0	0.0	0.0	0.0	0.0	0.0	0.0
0.0	0.0	0.0	0.0	0.0	0.0	0.0	0.0
7.0	10.0	45.0	0.0	97.0	0.0	38.0	0.0
0.0	0.0	0.0	0.0	0.0	0.0	0.0	0.0
0.0	0.0	0.0	0.0	0.0	0.0	0.0	0.0
5.0	0.0	0.0	0.0	0.0	0.0	8.0	0.0
1.0	5.0	6.0	0.0	4.0	2.0	0.0	3.0
0.0	0.0	0.0	0.0	0.0	0.0	2.0	0.0
15.0	2.0	0.0	0.0	1.0	0.0	0.0	3.0
7.0	0.0	2.0	0.0	0.0	0.0	0.0	0.0
0.0	7.0	0.0	0.0	11.0	0.0	8.0	0.0
10.0	0.0	4.0	0.0	0.0	0.0	0.0	0.0
0.0	0.0	0.0	0.0	0.0	0.0	0.0	0.0
46.0	0.0	0.0	0.0	0.0	0.0	0.0	17.0
0.0	0.0	0.0	0.0	0.0	0.0	0.0	0.0
0.0	0.0	2.0	0.0	44.0	0.0	0.0	0.0
7.0	0.0	19.0	2.0	269.0	3.0	0.0	0.0
0.0	1.0	0.0	0.0	0.0	0.0	4.0	0.0
0.0	3.0	5.0	1.0	0.0	0.0	0.0	0.0
9.0	0.0	9.0	0.0	0.0	0.0	0.0	0.0
3.0	0.0	0.0	0.0	0.0	8.0	0.0	0.0
0.0	0.0	1.0	0.0	0.0	2.0	0.0	0.0
5.0	3.0	8.0	0.0	0.0	0.0	0.0	0.0
0.0	92.0	0.0	0.0	0.0	70.0	0.0	0.0
0.0	0.0	0.0	0.0	0.0	0.0	0.0	0.0
7.0	0.0	4.0	0.0	0.0	0.0	0.0	0.0
4.0	0.0	0.0	0.0	0.0	0.0	0.0	0.0
0.0	0.0	0.0	0.0	0.0	0.0	0.0	0.0
0.0	0.0	6.0	1.0	26.0	44.0	0.0	26.0
0.0	0.0	6.0	0.0	0.0	9.0	0.0	0.0
0.0	0.0	0.0	0.0	0.0	7.0	0.0	0.0
0.0	0.0	0.0	0.0	0.0	0.0	0.0	0.0
10.0	2.0	1.0	0.0	0.0	0.0	1.0	0.0
0.0	14.0	3.0	0.0	0.0	13.0	3.0	0.0
0.0	0.0	0.0	0.0	0.0	0.0	2.0	0.0
16.0	0.0	0.0	0.0	3.0	3.0	0.0	2.0
5.0	1.0	4.0	0.0	4.0	0.0	0.0	0.0
0.0	2.0	0.0	0.0	0.0	0.0	8.0	0.0
12.0	0.0	5.0	0.0	33.0	0.0	0.0	0.0
17.0	3.0	9.0	0.0	3.0	1.0	0.0	0.0
8.0	0.0	0.0	0.0	0.0	0.0	0.0	0.0
0.0	0.0	0.0	0.0	0.0	0.0	0.0	0.0
3.0	16.0	24.0	0.0	0.0	7.0	3.0	3.0
1.0	0.0	0.0	0.0	0.0	17.0	18.0	0.0
0.0	0.0	0.0	0.0	0.0	0.0	0.0	0.0

0.0	0.0	0.0	0.0	0.0	0.0	0.0	0.0
1.0	0.0	6.0	0.0	0.0	1.0	0.0	0.0
0.0	0.0	2.0	0.0	0.0	0.0	0.0	0.0
60.0	18.0	6.0	0.0	3.0	0.0	0.0	0.0
0.0	0.0	1.0	0.0	0.0	0.0	0.0	0.0
1.0	1.0	0.0	0.0	0.0	0.0	0.0	0.0
0.0	0.0	2.0	0.0	17.0	51.0	8.0	1.0
0.0	2.0	0.0	0.0	0.0	0.0	0.0	0.0
3.0	0.0	5.0	0.0	4.0	0.0	0.0	0.0
0.0	2.0	0.0	0.0	0.0	0.0	0.0	0.0
0.0	0.0	0.0	0.0	0.0	0.0	0.0	0.0
0.0	1.0	4.0	0.0	0.0	0.0	0.0	0.0
0.0	0.0	2.0	0.0	0.0	0.0	0.0	0.0
0.0	0.0	1.0	3.0	27.0	0.0	8.0	10.0
0.0	0.0	1.0	0.0	6.0	1.0	0.0	0.0
1.0	1.0	0.0	0.0	7.0	11.0	7.0	3.0
0.0	0.0	0.0	0.0	0.0	0.0	0.0	0.0
0.0	0.0	0.0	0.0	0.0	0.0	0.0	0.0
0.0	0.0	0.0	0.0	0.0	0.0	0.0	0.0
0.0	0.0	1.0	0.0	0.0	0.0	0.0	0.0
0.0	24.0	1.0	0.0	23.0	0.0	0.0	23.0
0.0	0.0	0.0	0.0	0.0	0.0	0.0	0.0
0.0	0.0	0.0	0.0	0.0	0.0	0.0	0.0
3.0	9.0	3.0	0.0	2.0	20.0	20.0	0.0
0.0	0.0	0.0	0.0	0.0	0.0	0.0	0.0
0.0	0.0	0.0	0.0	0.0	0.0	0.0	0.0
0.0	94.0	0.0	0.0	0.0	7.0	0.0	0.0
0.0	0.0	0.0	0.0	0.0	0.0	0.0	0.0
0.0	9.0	0.0	0.0	0.0	0.0	0.0	0.0
0.0	0.0	0.0	0.0	0.0	0.0	0.0	0.0
0.0	0.0	0.0	0.0	0.0	0.0	0.0	0.0
0.0	1.0	0.0	0.0	0.0	3.0	0.0	0.0
57.0	60.0	14.0	0.0	0.0	0.0	0.0	0.0
1.0	0.0	1.0	0.0	1.0	0.0	0.0	0.0
0.0	0.0	0.0	0.0	0.0	0.0	0.0	0.0
0.0	2.0	1.0	0.0	0.0	0.0	0.0	0.0
0.0	0.0	0.0	0.0	0.0	0.0	0.0	0.0
0.0	0.0	0.0	0.0	0.0	0.0	0.0	0.0
0.0	12.0	8.0	0.0	0.0	0.0	0.0	0.0
0.0	7.0	0.0	0.0	0.0	20.0	10.0	0.0
6.0	0.0	0.0	0.0	1.0	0.0	0.0	0.0
0.0	0.0	3.0	0.0	0.0	1.0	0.0	0.0
0.0	0.0	0.0	0.0	0.0	0.0	0.0	0.0
0.0	0.0	0.0	0.0	0.0	0.0	0.0	0.0
0.0	0.0	3.0	0.0	0.0	0.0	0.0	0.0
0.0	0.0	0.0	0.0	0.0	0.0	0.0	0.0
0.0	13.0	12.0	0.0	0.0	0.0	0.0	0.0

23.0	0.0	0.0	0.0	0.0	0.0	0.0	0.0
5.0	1.0	0.0	0.0	8.0	5.0	0.0	0.0
0.0	0.0	2.0	0.0	1.0	0.0	0.0	0.0
0.0	0.0	0.0	0.0	0.0	0.0	0.0	0.0
0.0	0.0	0.0	0.0	0.0	0.0	0.0	0.0
2.0	0.0	0.0	0.0	0.0	0.0	0.0	0.0
4.0	2.0	1.0	0.0	0.0	0.0	0.0	0.0
0.0	0.0	1.0	0.0	0.0	0.0	0.0	0.0
0.0	0.0	2.0	0.0	0.0	0.0	0.0	0.0
0.0	0.0	0.0	0.0	0.0	0.0	0.0	0.0
0.0	0.0	0.0	0.0	0.0	0.0	0.0	0.0
0.0	0.0	0.0	0.0	0.0	0.0	0.0	0.0
0.0	0.0	0.0	0.0	0.0	0.0	0.0	0.0
0.0	0.0	11.0	0.0	0.0	107.0	0.0	0.0
1.0	2.0	0.0	0.0	0.0	0.0	0.0	0.0
0.0	0.0	0.0	0.0	0.0	0.0	0.0	0.0
0.0	0.0	0.0	0.0	0.0	0.0	0.0	0.0
0.0	0.0	0.0	0.0	0.0	0.0	0.0	0.0
2.0	0.0	0.0	0.0	0.0	0.0	0.0	0.0
0.0	0.0	2.0	0.0	0.0	0.0	0.0	0.0
0.0	0.0	0.0	0.0	2.0	0.0	0.0	0.0
0.0	54.0	0.0	0.0	0.0	3.0	0.0	0.0
0.0	0.0	0.0	0.0	0.0	0.0	0.0	0.0
0.0	0.0	0.0	0.0	0.0	0.0	0.0	0.0
0.0	0.0	1.0	0.0	0.0	0.0	0.0	0.0
0.0	0.0	0.0	0.0	0.0	0.0	0.0	0.0
1.0	0.0	0.0	0.0	0.0	0.0	1.0	3.0
0.0	0.0	0.0	0.0	0.0	0.0	0.0	0.0
0.0	0.0	1.0	0.0	0.0	0.0	0.0	0.0
0.0	0.0	0.0	0.0	0.0	0.0	0.0	0.0
0.0	0.0	0.0	0.0	1.0	0.0	0.0	0.0
0.0	1.0	0.0	0.0	5.0	0.0	0.0	0.0
0.0	0.0	0.0	0.0	0.0	0.0	0.0	0.0
0.0	0.0	0.0	0.0	0.0	0.0	0.0	0.0
0.0	0.0	0.0	0.0	0.0	0.0	0.0	0.0
0.0	0.0	2.0	0.0	0.0	0.0	0.0	0.0
0.0	0.0	0.0	0.0	0.0	0.0	0.0	0.0
2.0	1.0	2.0	0.0	0.0	0.0	0.0	5.0
0.0	0.0	0.0	0.0	0.0	0.0	0.0	0.0
0.0	0.0	0.0	0.0	0.0	0.0	0.0	0.0
0.0	0.0	0.0	0.0	0.0	0.0	0.0	0.0
0.0	0.0	0.0	0.0	0.0	0.0	0.0	0.0
0.0	0.0	2.0	0.0	0.0	0.0	0.0	0.0
2.0	0.0	2.0	0.0	0.0	0.0	0.0	0.0
0.0	0.0	0.0	0.0	0.0	0.0	0.0	0.0
0.0	0.0	0.0	0.0	0.0	0.0	0.0	0.0
0.0	0.0	0.0	0.0	0.0	0.0	0.0	0.0

0.0	0.0	0.0	0.0	0.0	5.0	0.0	7.0
0.0	0.0	0.0	0.0	0.0	0.0	0.0	0.0
0.0	0.0	0.0	0.0	0.0	0.0	0.0	0.0
0.0	0.0	0.0	0.0	0.0	0.0	0.0	0.0
0.0	0.0	0.0	0.0	0.0	0.0	0.0	0.0
0.0	0.0	1.0	0.0	0.0	0.0	0.0	0.0
0.0	0.0	0.0	0.0	2.0	0.0	0.0	5.0
0.0	0.0	0.0	0.0	0.0	0.0	0.0	0.0
0.0	0.0	0.0	0.0	0.0	0.0	0.0	0.0
0.0	0.0	0.0	0.0	0.0	0.0	0.0	0.0
0.0	0.0	0.0	0.0	0.0	0.0	12.0	6988.0
0.0	5.0	0.0	0.0	1.0	1.0	9.0	0.0
0.0	0.0	0.0	0.0	0.0	0.0	0.0	0.0
0.0	0.0	0.0	0.0	0.0	0.0	0.0	0.0
0.0	0.0	0.0	0.0	0.0	0.0	0.0	0.0
0.0	0.0	7.0	25.0	7.0	3.0	7.0	44.0
0.0	0.0	0.0	0.0	0.0	0.0	0.0	0.0
0.0	0.0	0.0	0.0	0.0	0.0	0.0	0.0
0.0	0.0	0.0	0.0	0.0	0.0	0.0	0.0
0.0	0.0	0.0	0.0	0.0	0.0	0.0	0.0
0.0	0.0	0.0	0.0	0.0	0.0	0.0	0.0
0.0	0.0	0.0	0.0	0.0	0.0	0.0	0.0
0.0	0.0	0.0	0.0	0.0	0.0	0.0	0.0
0.0	0.0	0.0	0.0	0.0	0.0	0.0	0.0
3.0	4.0	0.0	0.0	14.0	28.0	8.0	0.0
0.0	0.0	0.0	0.0	0.0	0.0	0.0	0.0
4.0	2.0	8.0	0.0	21.0	25.0	7.0	0.0
3.0	0.0	0.0	0.0	0.0	0.0	0.0	0.0
0.0	0.0	0.0	0.0	0.0	0.0	0.0	0.0
0.0	0.0	0.0	0.0	0.0	0.0	0.0	0.0
0.0	0.0	1.0	0.0	0.0	0.0	0.0	0.0
0.0	0.0	0.0	0.0	0.0	0.0	0.0	0.0
0.0	0.0	0.0	0.0	0.0	0.0	0.0	0.0
1.0	0.0	0.0	0.0	0.0	0.0	0.0	0.0
2.0	0.0	0.0	0.0	0.0	0.0	0.0	0.0
0.0	0.0	0.0	0.0	0.0	0.0	0.0	0.0
0.0	0.0	0.0	0.0	0.0	0.0	0.0	0.0
0.0	0.0	0.0	0.0	0.0	0.0	0.0	0.0
1.0	0.0	0.0	0.0	0.0	0.0	0.0	0.0
0.0	0.0	0.0	0.0	0.0	0.0	0.0	0.0
0.0	0.0	2.0	0.0	0.0	0.0	0.0	0.0
0.0	0.0	0.0	0.0	0.0	0.0	0.0	0.0
0.0	0.0	0.0	0.0	0.0	0.0	0.0	0.0
3.0	0.0	1.0	0.0	0.0	3.0	1.0	0.0
0.0	0.0	0.0	0.0	0.0	0.0	0.0	0.0
0.0	0.0	0.0	0.0	0.0	0.0	0.0	0.0
0.0	0.0	0.0	0.0	0.0	0.0	0.0	0.0

0.0	0.0	0.0	0.0	0.0	0.0	0.0	0.0
0.0	0.0	0.0	0.0	0.0	0.0	0.0	0.0
0.0	0.0	0.0	0.0	0.0	0.0	0.0	0.0
0.0	0.0	0.0	0.0	0.0	0.0	0.0	0.0
1.0	0.0	0.0	0.0	1.0	0.0	0.0	0.0
0.0	0.0	2.0	0.0	0.0	0.0	0.0	0.0
0.0	0.0	1.0	0.0	0.0	0.0	0.0	0.0
0.0	0.0	0.0	0.0	0.0	0.0	0.0	0.0
0.0	0.0	0.0	3.0	6.0	1.0	2.0	0.0
0.0	0.0	0.0	0.0	0.0	0.0	0.0	0.0
0.0	0.0	0.0	0.0	0.0	0.0	0.0	0.0
0.0	0.0	0.0	0.0	0.0	0.0	0.0	0.0
0.0	0.0	0.0	0.0	1.0	0.0	0.0	0.0
0.0	0.0	0.0	0.0	0.0	0.0	0.0	0.0
0.0	0.0	0.0	0.0	0.0	0.0	0.0	0.0
0.0	0.0	0.0	0.0	0.0	0.0	0.0	0.0
1.0	5.0	0.0	0.0	5.0	0.0	0.0	0.0
0.0	0.0	0.0	0.0	0.0	0.0	0.0	0.0
8.0	0.0	0.0	0.0	0.0	0.0	0.0	0.0
0.0	0.0	0.0	0.0	0.0	0.0	0.0	0.0
0.0	0.0	0.0	0.0	0.0	0.0	0.0	0.0
0.0	0.0	0.0	0.0	0.0	0.0	0.0	0.0
0.0	0.0	0.0	0.0	0.0	0.0	0.0	0.0
0.0	0.0	0.0	0.0	0.0	0.0	0.0	0.0
0.0	0.0	0.0	0.0	0.0	0.0	0.0	0.0
0.0	0.0	0.0	0.0	0.0	0.0	0.0	0.0
0.0	0.0	0.0	0.0	0.0	0.0	0.0	0.0
4.0	0.0	0.0	0.0	0.0	0.0	0.0	0.0
0.0	0.0	0.0	0.0	0.0	0.0	0.0	0.0
0.0	65.0	0.0	0.0	0.0	0.0	0.0	0.0
0.0	0.0	0.0	0.0	0.0	0.0	0.0	0.0
0.0	0.0	0.0	0.0	0.0	0.0	0.0	0.0
0.0	0.0	0.0	0.0	0.0	0.0	0.0	0.0
0.0	0.0	0.0	0.0	0.0	3.0	0.0	0.0
0.0	0.0	0.0	0.0	0.0	0.0	0.0	0.0
0.0	0.0	0.0	0.0	0.0	0.0	0.0	0.0
0.0	0.0	0.0	0.0	0.0	0.0	0.0	0.0
0.0	0.0	0.0	0.0	0.0	0.0	0.0	2.0
2.0	0.0	0.0	0.0	17.0	3.0	0.0	0.0
0.0	2.0	0.0	0.0	0.0	0.0	0.0	0.0
0.0	0.0	0.0	0.0	0.0	0.0	0.0	0.0
0.0	0.0	0.0	0.0	0.0	0.0	0.0	0.0
0.0	0.0	0.0	0.0	0.0	0.0	0.0	0.0
0.0	0.0	0.0	0.0	0.0	0.0	0.0	0.0
0.0	0.0	0.0	0.0	0.0	2.0	1.0	2.0
0.0	0.0	0.0	0.0	3.0	0.0	0.0	0.0
0.0	0.0	0.0	0.0	0.0	1.0	0.0	0.0

0.0	0.0	0.0	0.0	0.0	0.0	0.0	0.0
65.0	23.0	0.0	0.0	23.0	13.0	0.0	0.0
0.0	0.0	0.0	0.0	0.0	0.0	0.0	0.0
0.0	0.0	0.0	0.0	0.0	0.0	0.0	0.0
0.0	0.0	2.0	0.0	0.0	0.0	0.0	0.0
0.0	0.0	0.0	0.0	3.0	0.0	0.0	0.0
0.0	0.0	0.0	0.0	0.0	0.0	0.0	0.0
1.0	0.0	0.0	0.0	0.0	0.0	0.0	0.0
0.0	0.0	0.0	0.0	0.0	0.0	0.0	1.0
1.0	0.0	1.0	0.0	0.0	2.0	0.0	5.0
0.0	0.0	1.0	0.0	0.0	0.0	0.0	0.0
0.0	0.0	0.0	0.0	1.0	9.0	0.0	13.0
0.0	0.0	0.0	0.0	29.0	0.0	0.0	0.0
2.0	0.0	0.0	0.0	0.0	0.0	0.0	0.0
0.0	0.0	1.0	0.0	0.0	0.0	0.0	5.0
0.0	0.0	1.0	0.0	0.0	0.0	0.0	0.0
0.0	0.0	0.0	0.0	0.0	0.0	0.0	0.0
0.0	3.0	0.0	0.0	0.0	0.0	0.0	0.0
0.0	0.0	0.0	0.0	0.0	0.0	0.0	0.0
0.0	0.0	0.0	0.0	0.0	0.0	0.0	0.0
0.0	0.0	0.0	0.0	0.0	0.0	0.0	0.0
0.0	0.0	0.0	0.0	0.0	0.0	0.0	0.0
0.0	0.0	0.0	0.0	0.0	0.0	0.0	0.0
0.0	519.0	0.0	1.0	161.0	0.0	402.0	108.0
0.0	0.0	0.0	0.0	0.0	1.0	0.0	0.0
0.0	0.0	0.0	0.0	0.0	0.0	0.0	0.0
0.0	0.0	0.0	0.0	0.0	0.0	0.0	0.0
0.0	0.0	0.0	0.0	0.0	0.0	0.0	0.0
0.0	0.0	0.0	0.0	0.0	0.0	0.0	0.0
2.0	0.0	3.0	0.0	0.0	0.0	0.0	0.0
0.0	0.0	0.0	0.0	0.0	0.0	0.0	0.0
0.0	0.0	0.0	0.0	0.0	0.0	0.0	0.0
0.0	0.0	0.0	0.0	0.0	0.0	4.0	0.0
0.0	0.0	1.0	0.0	0.0	0.0	0.0	0.0
0.0	0.0	0.0	0.0	0.0	0.0	0.0	0.0
0.0	0.0	1.0	0.0	0.0	0.0	0.0	0.0
0.0	0.0	0.0	0.0	0.0	0.0	1.0	0.0
0.0	0.0	0.0	0.0	0.0	0.0	0.0	0.0
0.0	0.0	9.0	0.0	0.0	6.0	3.0	0.0
0.0	0.0	0.0	0.0	0.0	0.0	0.0	0.0
0.0	0.0	378.0	0.0	0.0	3.0	0.0	0.0
601.0	16.0	808.0	0.0	20.0	2.0	3.0	10.0
0.0	0.0	0.0	0.0	0.0	0.0	0.0	0.0
0.0	0.0	0.0	0.0	0.0	0.0	0.0	0.0
0.0	0.0	0.0	0.0	0.0	0.0	0.0	0.0
0.0	0.0	0.0	0.0	0.0	0.0	0.0	0.0
1.0	0.0	0.0	0.0	4.0	1.0	2.0	1.0

0.0	0.0	0.0	0.0	0.0	0.0	0.0	0.0
0.0	0.0	0.0	0.0	0.0	0.0	0.0	0.0
2.0	2.0	7.0	0.0	0.0	5.0	0.0	0.0
7.0	0.0	0.0	1.0	0.0	0.0	0.0	0.0
0.0	0.0	0.0	0.0	0.0	0.0	0.0	0.0
0.0	0.0	0.0	0.0	0.0	0.0	0.0	0.0
0.0	0.0	0.0	0.0	0.0	0.0	0.0	0.0
0.0	0.0	0.0	0.0	0.0	0.0	0.0	0.0
0.0	0.0	0.0	0.0	0.0	0.0	0.0	0.0
0.0	0.0	0.0	0.0	0.0	0.0	0.0	0.0
1.0	0.0	0.0	0.0	0.0	1.0	0.0	0.0
0.0	0.0	0.0	0.0	0.0	0.0	0.0	0.0
0.0	0.0	0.0	0.0	0.0	3.0	0.0	0.0
0.0	0.0	0.0	0.0	0.0	0.0	0.0	0.0
0.0	0.0	0.0	0.0	0.0	0.0	0.0	0.0
0.0	0.0	1.0	0.0	0.0	0.0	0.0	0.0
0.0	0.0	0.0	0.0	0.0	0.0	0.0	0.0
0.0	0.0	0.0	0.0	0.0	0.0	0.0	0.0
0.0	0.0	0.0	0.0	0.0	2.0	0.0	0.0
0.0	0.0	0.0	0.0	0.0	0.0	0.0	0.0
0.0	0.0	1.0	0.0	6.0	0.0	0.0	4.0
0.0	0.0	0.0	0.0	0.0	0.0	0.0	1.0
0.0	0.0	0.0	0.0	2.0	2.0	0.0	0.0
0.0	0.0	0.0	0.0	6.0	0.0	0.0	0.0
0.0	0.0	0.0	0.0	0.0	0.0	0.0	0.0
0.0	0.0	0.0	0.0	0.0	0.0	0.0	0.0
0.0	0.0	0.0	0.0	0.0	0.0	0.0	0.0
0.0	0.0	0.0	0.0	0.0	0.0	0.0	0.0
0.0	0.0	3.0	0.0	0.0	71.0	0.0	0.0
0.0	0.0	0.0	0.0	0.0	0.0	0.0	0.0
0.0	0.0	0.0	0.0	0.0	0.0	0.0	0.0
8.0	0.0	3.0	0.0	0.0	0.0	0.0	0.0
0.0	0.0	0.0	0.0	0.0	0.0	0.0	0.0
0.0	0.0	0.0	0.0	0.0	0.0	0.0	0.0
0.0	0.0	2.0	0.0	0.0	0.0	0.0	0.0
0.0	0.0	0.0	0.0	0.0	0.0	0.0	0.0
0.0	4.0	4.0	0.0	0.0	16.0	1.0	0.0
0.0	0.0	0.0	0.0	5.0	0.0	1.0	0.0
1.0	0.0	0.0	2.0	0.0	1.0	0.0	38.0
2.0	0.0	0.0	0.0	0.0	0.0	0.0	0.0
0.0	0.0	0.0	0.0	0.0	0.0	0.0	0.0
0.0	0.0	0.0	0.0	0.0	0.0	0.0	0.0
0.0	0.0	0.0	0.0	0.0	0.0	0.0	0.0
0.0	0.0	0.0	0.0	0.0	0.0	0.0	0.0
23.0	24.0	10.0	0.0	0.0	9.0	0.0	0.0
1.0	0.0	1.0	0.0	0.0	0.0	0.0	0.0
7.0	14.0	0.0	0.0	0.0	29.0	0.0	0.0

0.0	0.0	0.0	0.0	0.0	0.0	0.0	0.0
2.0	0.0	0.0	0.0	0.0	0.0	0.0	0.0
0.0	0.0	0.0	0.0	5.0	0.0	0.0	0.0
0.0	1.0	2.0	5.0	0.0	1.0	3.0	2.0
0.0	0.0	0.0	0.0	0.0	1.0	0.0	0.0
0.0	0.0	0.0	0.0	0.0	5.0	0.0	0.0
3.0	0.0	0.0	0.0	0.0	0.0	0.0	0.0
0.0	0.0	0.0	0.0	0.0	0.0	0.0	0.0
6.0	0.0	0.0	0.0	0.0	0.0	0.0	0.0
0.0	0.0	0.0	0.0	0.0	0.0	0.0	0.0
0.0	0.0	0.0	0.0	0.0	0.0	0.0	0.0
0.0	0.0	0.0	0.0	0.0	0.0	0.0	0.0
0.0	0.0	0.0	0.0	0.0	0.0	0.0	0.0
2.0	0.0	0.0	0.0	3.0	4.0	16.0	12.0
2.0	0.0	0.0	434.0	0.0	0.0	0.0	2.0
0.0	0.0	0.0	0.0	0.0	0.0	0.0	0.0
0.0	0.0	0.0	0.0	0.0	0.0	0.0	0.0
2.0	0.0	0.0	0.0	0.0	0.0	0.0	0.0
1.0	0.0	0.0	0.0	0.0	0.0	0.0	0.0
0.0	0.0	0.0	0.0	0.0	0.0	0.0	0.0
0.0	0.0	0.0	0.0	0.0	0.0	0.0	0.0
0.0	0.0	0.0	2.0	0.0	1.0	0.0	0.0
0.0	0.0	0.0	0.0	0.0	0.0	0.0	0.0
0.0	0.0	0.0	0.0	2.0	0.0	0.0	0.0
0.0	0.0	1.0	0.0	0.0	0.0	0.0	0.0
0.0	0.0	0.0	0.0	0.0	0.0	0.0	0.0
0.0	0.0	0.0	0.0	0.0	0.0	0.0	0.0
0.0	0.0	0.0	0.0	0.0	0.0	0.0	0.0
0.0	0.0	0.0	0.0	0.0	0.0	0.0	0.0
0.0	0.0	0.0	0.0	0.0	0.0	0.0	0.0
0.0	0.0	0.0	0.0	0.0	0.0	0.0	0.0
0.0	0.0	0.0	0.0	0.0	0.0	0.0	0.0
0.0	0.0	0.0	0.0	0.0	0.0	0.0	0.0
0.0	0.0	0.0	0.0	0.0	0.0	0.0	0.0
0.0	0.0	0.0	0.0	0.0	0.0	0.0	0.0
0.0	0.0	0.0	0.0	0.0	0.0	0.0	0.0
0.0	0.0	0.0	0.0	0.0	0.0	0.0	0.0
0.0	0.0	0.0	0.0	0.0	0.0	0.0	0.0
0.0	0.0	0.0	0.0	0.0	5.0	0.0	0.0
0.0	0.0	0.0	0.0	0.0	0.0	0.0	0.0
0.0	0.0	0.0	0.0	0.0	0.0	0.0	0.0
0.0	0.0	0.0	0.0	0.0	0.0	0.0	0.0
0.0	0.0	0.0	0.0	0.0	0.0	0.0	0.0
0.0	0.0	0.0	0.0	0.0	0.0	0.0	0.0
0.0	0.0	0.0	0.0	0.0	0.0	0.0	0.0
0.0	0.0	0.0	0.0	0.0	0.0	0.0	0.0
0.0	0.0	0.0	0.0	0.0	0.0	0.0	0.0

0.0	0.0	0.0	0.0	0.0	0.0	0.0	4.0
0.0	0.0	0.0	0.0	0.0	0.0	0.0	0.0
0.0	0.0	0.0	0.0	0.0	0.0	0.0	0.0
0.0	0.0	0.0	0.0	6.0	1.0	1.0	0.0
0.0	2.0	1.0	0.0	0.0	0.0	0.0	0.0
0.0	0.0	0.0	0.0	0.0	0.0	0.0	0.0
0.0	0.0	0.0	0.0	2.0	3.0	0.0	2.0
0.0	0.0	2.0	0.0	0.0	0.0	0.0	0.0
0.0	0.0	0.0	0.0	0.0	0.0	0.0	0.0
0.0	0.0	0.0	0.0	0.0	0.0	0.0	0.0
0.0	0.0	0.0	0.0	0.0	0.0	0.0	0.0
0.0	0.0	0.0	0.0	0.0	0.0	0.0	0.0
0.0	0.0	0.0	0.0	0.0	0.0	0.0	0.0
0.0	0.0	0.0	0.0	0.0	0.0	0.0	0.0
0.0	0.0	0.0	0.0	0.0	0.0	0.0	0.0
0.0	0.0	0.0	0.0	0.0	0.0	0.0	0.0
0.0	0.0	0.0	0.0	0.0	0.0	0.0	0.0
0.0	0.0	0.0	0.0	0.0	0.0	0.0	0.0
0.0	0.0	0.0	0.0	0.0	0.0	0.0	0.0
0.0	0.0	0.0	0.0	2.0	0.0	0.0	0.0
0.0	0.0	0.0	0.0	0.0	0.0	0.0	0.0
0.0	0.0	0.0	0.0	0.0	0.0	0.0	0.0
0.0	0.0	0.0	0.0	0.0	0.0	0.0	0.0
0.0	0.0	0.0	0.0	0.0	0.0	0.0	0.0
0.0	0.0	0.0	0.0	0.0	0.0	0.0	0.0
0.0	0.0	0.0	2.0	0.0	0.0	0.0	0.0
0.0	0.0	0.0	286.0	0.0	0.0	0.0	2.0
0.0	0.0	0.0	0.0	0.0	0.0	0.0	0.0
0.0	0.0	0.0	0.0	0.0	0.0	0.0	0.0
0.0	0.0	0.0	0.0	0.0	0.0	0.0	0.0
0.0	0.0	0.0	54.0	0.0	0.0	0.0	10.0
0.0	0.0	0.0	0.0	0.0	0.0	0.0	42.0
0.0	0.0	0.0	0.0	0.0	0.0	0.0	0.0
0.0	0.0	0.0	0.0	0.0	0.0	0.0	0.0
0.0	0.0	0.0	0.0	0.0	0.0	0.0	0.0
0.0	0.0	0.0	0.0	0.0	0.0	0.0	0.0
0.0	0.0	0.0	0.0	0.0	0.0	0.0	0.0
0.0	0.0	0.0	0.0	0.0	0.0	0.0	0.0
0.0	0.0	0.0	0.0	0.0	0.0	0.0	0.0
0.0	0.0	2.0	0.0	0.0	0.0	0.0	0.0
0.0	0.0	0.0	0.0	0.0	0.0	0.0	0.0
0.0	0.0	0.0	0.0	0.0	0.0	0.0	0.0
0.0	0.0	0.0	0.0	1.0	0.0	0.0	0.0
0.0	0.0	0.0	0.0	0.0	0.0	0.0	0.0
0.0	0.0	0.0	0.0	0.0	0.0	0.0	0.0
0.0	0.0	0.0	0.0	0.0	0.0	0.0	0.0
0.0	0.0	0.0	0.0	0.0	0.0	0.0	0.0

0.0	0.0	0.0	0.0	0.0	0.0	0.0	0.0
0.0	0.0	0.0	0.0	0.0	0.0	0.0	0.0
0.0	0.0	0.0	0.0	0.0	0.0	0.0	0.0
0.0	0.0	0.0	0.0	0.0	0.0	0.0	0.0
0.0	0.0	0.0	860.0	0.0	0.0	0.0	22.0
0.0	0.0	0.0	0.0	0.0	0.0	0.0	0.0
0.0	0.0	0.0	179.0	9.0	0.0	0.0	0.0
2.0	0.0	0.0	8.0	0.0	1.0	0.0	2.0
0.0	0.0	0.0	0.0	0.0	0.0	0.0	3.0
0.0	0.0	0.0	0.0	0.0	0.0	0.0	0.0
0.0	0.0	0.0	0.0	0.0	0.0	0.0	0.0
0.0	0.0	0.0	0.0	0.0	0.0	0.0	0.0
0.0	0.0	0.0	11.0	0.0	0.0	0.0	0.0
2.0	0.0	0.0	5.0	1.0	0.0	0.0	6.0
0.0	0.0	0.0	2.0	0.0	0.0	0.0	0.0
0.0	0.0	0.0	0.0	3.0	3.0	0.0	4.0
0.0	0.0	0.0	0.0	0.0	1.0	1.0	0.0
0.0	0.0	0.0	0.0	0.0	0.0	0.0	0.0
0.0	0.0	0.0	0.0	0.0	1.0	0.0	0.0
0.0	0.0	0.0	0.0	0.0	0.0	0.0	0.0
0.0	0.0	0.0	0.0	0.0	0.0	0.0	0.0
0.0	0.0	0.0	0.0	0.0	0.0	0.0	0.0
0.0	0.0	0.0	0.0	0.0	0.0	0.0	1.0
0.0	0.0	0.0	0.0	1.0	1.0	0.0	0.0
0.0	0.0	0.0	0.0	0.0	0.0	0.0	0.0
0.0	0.0	1.0	0.0	4.0	1.0	0.0	0.0
0.0	0.0	0.0	0.0	0.0	0.0	0.0	0.0
0.0	0.0	0.0	0.0	0.0	0.0	0.0	0.0
0.0	0.0	0.0	0.0	0.0	0.0	0.0	0.0
0.0	0.0	0.0	0.0	0.0	0.0	0.0	0.0
0.0	0.0	0.0	0.0	0.0	0.0	0.0	0.0
0.0	0.0	0.0	0.0	0.0	0.0	0.0	0.0
0.0	0.0	0.0	0.0	0.0	0.0	0.0	0.0
0.0	0.0	0.0	0.0	0.0	0.0	0.0	0.0
0.0	0.0	0.0	0.0	0.0	0.0	0.0	0.0
0.0	0.0	0.0	0.0	3.0	0.0	0.0	0.0
0.0	0.0	0.0	0.0	0.0	0.0	0.0	0.0
0.0	0.0	0.0	0.0	0.0	0.0	0.0	0.0
0.0	0.0	0.0	0.0	0.0	0.0	0.0	0.0
0.0	0.0	0.0	0.0	0.0	0.0	0.0	0.0
0.0	0.0	0.0	0.0	0.0	0.0	0.0	0.0
0.0	0.0	0.0	0.0	0.0	0.0	0.0	0.0
0.0	0.0	0.0	0.0	0.0	0.0	0.0	0.0
0.0	0.0	0.0	0.0	0.0	0.0	0.0	0.0
0.0	0.0	0.0	0.0	0.0	0.0	0.0	0.0
4.0	3.0	0.0	0.0	0.0	0.0	0.0	0.0
0.0	0.0	0.0	0.0	0.0	0.0	0.0	0.0

0.0	0.0	0.0	0.0	0.0	0.0	0.0	0.0
0.0	0.0	0.0	0.0	0.0	0.0	0.0	0.0
0.0	0.0	0.0	0.0	0.0	0.0	0.0	0.0
3.0	0.0	0.0	0.0	0.0	0.0	0.0	0.0
0.0	0.0	0.0	0.0	0.0	0.0	0.0	0.0
0.0	0.0	0.0	0.0	0.0	0.0	0.0	0.0
0.0	0.0	0.0	0.0	0.0	0.0	0.0	0.0
0.0	0.0	1.0	0.0	0.0	0.0	0.0	0.0
0.0	0.0	0.0	0.0	0.0	0.0	0.0	0.0
0.0	0.0	1.0	0.0	0.0	0.0	0.0	0.0
0.0	0.0	0.0	0.0	0.0	0.0	0.0	0.0
0.0	0.0	5.0	0.0	0.0	12.0	0.0	7.0
0.0	0.0	1.0	0.0	0.0	0.0	0.0	0.0
0.0	0.0	0.0	0.0	0.0	0.0	0.0	0.0
0.0	0.0	0.0	0.0	0.0	0.0	0.0	0.0
0.0	0.0	0.0	0.0	0.0	0.0	0.0	0.0
0.0	0.0	0.0	0.0	0.0	0.0	0.0	0.0
0.0	0.0	0.0	0.0	0.0	0.0	0.0	0.0
0.0	0.0	0.0	0.0	0.0	0.0	0.0	0.0
0.0	0.0	0.0	0.0	0.0	0.0	0.0	0.0
0.0	0.0	0.0	0.0	0.0	0.0	0.0	0.0
173.0	0.0	0.0	0.0	0.0	0.0	0.0	0.0
119.0	62.0	0.0	0.0	0.0	0.0	0.0	1.0
69.0	0.0	0.0	0.0	0.0	0.0	0.0	0.0
61.0	0.0	0.0	0.0	0.0	0.0	0.0	0.0
59.0	0.0	12.0	0.0	0.0	0.0	0.0	0.0
30.0	0.0	0.0	0.0	0.0	0.0	0.0	0.0
14.0	0.0	0.0	0.0	0.0	59.0	0.0	0.0
15.0	19.0	9.0	0.0	0.0	0.0	0.0	0.0
10.0	0.0	0.0	0.0	0.0	58.0	0.0	0.0
13.0	13.0	0.0	0.0	0.0	0.0	0.0	0.0
6.0	0.0	0.0	0.0	0.0	0.0	0.0	0.0
6.0	0.0	0.0	0.0	0.0	0.0	0.0	0.0
7.0	0.0	0.0	0.0	0.0	0.0	0.0	0.0
3.0	0.0	0.0	0.0	0.0	0.0	0.0	0.0
4.0	0.0	0.0	0.0	0.0	0.0	0.0	0.0
3.0	0.0	0.0	0.0	0.0	0.0	0.0	0.0
3.0	0.0	0.0	0.0	0.0	0.0	0.0	0.0
3.0	0.0	0.0	0.0	0.0	0.0	0.0	0.0
2.0	0.0	1.0	0.0	0.0	0.0	2.0	0.0
2.0	0.0	0.0	0.0	0.0	0.0	0.0	0.0
1.0	0.0	2.0	0.0	0.0	0.0	0.0	0.0
4.0	0.0	1.0	0.0	0.0	0.0	0.0	0.0
2.0	0.0	0.0	0.0	0.0	0.0	0.0	0.0
4.0	0.0	0.0	0.0	0.0	0.0	0.0	0.0
0.0	3.0	0.0	0.0	0.0	0.0	0.0	0.0
1.0	0.0	0.0	0.0	0.0	0.0	0.0	0.0

2.0	0.0	0.0	0.0	0.0	0.0	0.0	0.0
3.0	0.0	1.0	0.0	0.0	0.0	0.0	0.0
2.0	0.0	0.0	0.0	1.0	0.0	0.0	0.0
3.0	0.0	0.0	0.0	0.0	1.0	0.0	0.0
3.0	0.0	0.0	0.0	0.0	0.0	0.0	0.0
3.0	1.0	0.0	0.0	0.0	0.0	0.0	0.0
1.0	0.0	0.0	0.0	0.0	0.0	0.0	0.0
1.0	0.0	0.0	0.0	0.0	0.0	0.0	0.0
1.0	0.0	4.0	0.0	0.0	1.0	0.0	0.0
2.0	0.0	0.0	0.0	0.0	0.0	0.0	0.0
0.0	0.0	0.0	0.0	0.0	0.0	0.0	0.0
4.0	0.0	1.0	0.0	0.0	0.0	0.0	0.0
2.0	0.0	0.0	0.0	0.0	0.0	0.0	0.0
2.0	0.0	0.0	0.0	0.0	0.0	0.0	0.0
3.0	0.0	0.0	0.0	0.0	0.0	0.0	0.0
2.0	0.0	0.0	0.0	0.0	0.0	0.0	0.0
0.0	0.0	0.0	0.0	0.0	0.0	0.0	0.0
1.0	0.0	0.0	0.0	0.0	0.0	0.0	0.0
0.0	0.0	1.0	0.0	0.0	0.0	0.0	0.0
0.0	0.0	0.0	0.0	0.0	0.0	0.0	0.0
2.0	0.0	0.0	0.0	0.0	0.0	0.0	0.0
1.0	0.0	0.0	0.0	0.0	0.0	2.0	0.0
1.0	0.0	0.0	0.0	0.0	0.0	0.0	0.0
0.0	0.0	0.0	0.0	0.0	0.0	0.0	0.0
1.0	0.0	0.0	0.0	0.0	0.0	0.0	0.0
1.0	0.0	0.0	0.0	0.0	0.0	0.0	0.0
0.0	0.0	0.0	0.0	0.0	0.0	0.0	0.0
0.0	0.0	0.0	0.0	0.0	0.0	0.0	0.0
0.0	0.0	0.0	0.0	0.0	0.0	0.0	0.0
0.0	0.0	0.0	0.0	0.0	0.0	0.0	0.0
1.0	0.0	0.0	0.0	0.0	0.0	0.0	0.0
1.0	0.0	0.0	0.0	0.0	0.0	0.0	0.0
0.0	0.0	0.0	0.0	1.0	0.0	0.0	0.0
1.0	0.0	0.0	0.0	0.0	0.0	0.0	0.0
0.0	20.0	0.0	0.0	0.0	0.0	0.0	0.0
1.0	0.0	0.0	0.0	0.0	0.0	0.0	0.0
0.0	0.0	0.0	0.0	0.0	0.0	0.0	0.0
0.0	0.0	0.0	0.0	0.0	0.0	0.0	0.0
1.0	0.0	0.0	0.0	0.0	0.0	0.0	0.0
1.0	0.0	0.0	0.0	0.0	0.0	0.0	0.0
0.0	0.0	0.0	0.0	0.0	0.0	0.0	0.0
1.0	0.0	0.0	0.0	1.0	0.0	0.0	0.0
2.0	0.0	0.0	0.0	0.0	0.0	0.0	0.0
1.0	0.0	0.0	0.0	0.0	0.0	0.0	0.0
1.0	0.0	0.0	0.0	0.0	0.0	0.0	0.0
1.0	0.0	0.0	0.0	0.0	0.0	0.0	0.0
0.0	0.0	0.0	0.0	0.0	0.0	0.0	0.0

2.0	0.0	0.0	0.0	0.0	0.0	0.0	0.0
1.0	0.0	0.0	0.0	0.0	0.0	0.0	0.0
1.0	0.0	0.0	0.0	0.0	0.0	0.0	0.0
0.0	0.0	0.0	0.0	0.0	0.0	0.0	0.0
1.0	0.0	0.0	0.0	0.0	0.0	0.0	0.0
1.0	0.0	0.0	0.0	0.0	0.0	0.0	0.0
0.0	0.0	0.0	0.0	0.0	0.0	0.0	0.0
0.0	0.0	0.0	0.0	0.0	0.0	0.0	1.0
1.0	0.0	0.0	0.0	0.0	0.0	0.0	0.0
1.0	0.0	0.0	0.0	0.0	0.0	0.0	0.0
1.0	0.0	0.0	0.0	0.0	0.0	0.0	0.0
1.0	0.0	0.0	0.0	0.0	0.0	0.0	0.0
1.0	0.0	0.0	0.0	0.0	0.0	0.0	0.0
0.0	0.0	0.0	0.0	0.0	0.0	0.0	0.0
1.0	0.0	0.0	0.0	0.0	0.0	0.0	0.0
0.0	0.0	0.0	0.0	0.0	0.0	0.0	0.0
0.0	332.0	0.0	0.0	0.0	0.0	0.0	0.0
0.0	259.0	0.0	0.0	0.0	1.0	0.0	0.0
0.0	207.0	79.0	0.0	0.0	0.0	0.0	0.0
0.0	183.0	9.0	0.0	0.0	0.0	0.0	0.0
0.0	130.0	0.0	0.0	0.0	0.0	0.0	0.0
0.0	113.0	0.0	0.0	0.0	0.0	0.0	0.0
0.0	117.0	0.0	0.0	0.0	0.0	0.0	0.0
0.0	95.0	0.0	0.0	0.0	0.0	0.0	0.0
0.0	74.0	0.0	0.0	0.0	0.0	0.0	0.0
0.0	73.0	0.0	1.0	0.0	297.0	0.0	0.0
0.0	59.0	0.0	0.0	0.0	0.0	0.0	0.0
0.0	56.0	0.0	0.0	0.0	0.0	0.0	0.0
0.0	50.0	0.0	0.0	0.0	0.0	0.0	0.0
0.0	37.0	0.0	0.0	0.0	0.0	0.0	0.0
0.0	39.0	0.0	0.0	0.0	0.0	0.0	0.0
0.0	34.0	0.0	0.0	0.0	0.0	0.0	0.0
0.0	27.0	0.0	0.0	0.0	0.0	0.0	0.0
0.0	33.0	0.0	0.0	0.0	0.0	0.0	2.0
0.0	29.0	37.0	0.0	0.0	0.0	0.0	0.0
0.0	28.0	0.0	0.0	0.0	0.0	2.0	0.0
0.0	25.0	0.0	0.0	0.0	0.0	0.0	0.0
0.0	12.0	0.0	0.0	0.0	0.0	0.0	0.0
0.0	16.0	0.0	0.0	0.0	0.0	0.0	0.0
0.0	16.0	63.0	0.0	0.0	0.0	0.0	0.0
0.0	14.0	0.0	0.0	0.0	0.0	0.0	0.0
0.0	14.0	0.0	0.0	0.0	0.0	0.0	0.0
0.0	16.0	0.0	0.0	0.0	0.0	0.0	0.0
0.0	13.0	0.0	0.0	0.0	5.0	0.0	0.0
0.0	13.0	0.0	0.0	0.0	2.0	0.0	0.0
0.0	8.0	0.0	0.0	0.0	0.0	0.0	0.0
0.0	10.0	0.0	0.0	13.0	0.0	15.0	0.0

0.0	9.0	0.0	0.0	0.0	0.0	0.0	0.0
0.0	9.0	0.0	0.0	0.0	0.0	0.0	0.0
0.0	9.0	0.0	0.0	0.0	0.0	2.0	0.0
0.0	6.0	0.0	0.0	196.0	91.0	0.0	0.0
0.0	6.0	0.0	0.0	0.0	0.0	0.0	0.0
0.0	5.0	0.0	0.0	0.0	0.0	0.0	0.0
0.0	2.0	0.0	0.0	0.0	0.0	0.0	0.0
0.0	4.0	0.0	0.0	0.0	0.0	0.0	0.0
0.0	7.0	0.0	0.0	0.0	0.0	0.0	0.0
0.0	4.0	0.0	0.0	0.0	0.0	0.0	0.0
0.0	6.0	0.0	0.0	0.0	0.0	0.0	0.0
0.0	3.0	0.0	0.0	2.0	0.0	0.0	0.0
0.0	3.0	0.0	0.0	0.0	0.0	0.0	0.0
0.0	6.0	2.0	0.0	0.0	1.0	0.0	0.0
0.0	4.0	0.0	0.0	0.0	0.0	0.0	0.0
0.0	3.0	0.0	0.0	0.0	0.0	0.0	0.0
0.0	4.0	0.0	0.0	0.0	0.0	0.0	0.0
0.0	3.0	0.0	0.0	0.0	0.0	0.0	0.0
0.0	3.0	0.0	0.0	0.0	0.0	0.0	0.0
0.0	4.0	0.0	0.0	0.0	0.0	0.0	0.0
0.0	3.0	0.0	0.0	0.0	0.0	0.0	0.0
0.0	3.0	0.0	0.0	0.0	0.0	0.0	0.0
0.0	3.0	0.0	0.0	0.0	0.0	0.0	0.0
0.0	3.0	1.0	0.0	0.0	0.0	0.0	0.0
0.0	0.0	0.0	0.0	0.0	0.0	0.0	0.0
0.0	3.0	0.0	0.0	0.0	0.0	0.0	0.0
0.0	2.0	0.0	0.0	0.0	0.0	0.0	0.0
0.0	3.0	0.0	0.0	0.0	0.0	0.0	0.0
0.0	3.0	0.0	0.0	0.0	0.0	0.0	0.0
0.0	2.0	0.0	0.0	0.0	0.0	0.0	0.0
0.0	3.0	0.0	0.0	0.0	0.0	0.0	0.0
0.0	3.0	0.0	0.0	0.0	0.0	0.0	0.0
0.0	1.0	0.0	0.0	0.0	0.0	0.0	0.0
0.0	0.0	0.0	0.0	0.0	0.0	0.0	0.0
0.0	1.0	0.0	0.0	0.0	0.0	0.0	0.0
0.0	1.0	1.0	0.0	0.0	0.0	0.0	0.0
0.0	2.0	0.0	0.0	0.0	0.0	0.0	0.0
0.0	2.0	0.0	0.0	0.0	0.0	0.0	0.0
0.0	2.0	0.0	0.0	0.0	0.0	0.0	0.0
0.0	1.0	0.0	0.0	0.0	0.0	0.0	0.0
0.0	1.0	0.0	0.0	0.0	0.0	0.0	0.0
0.0	2.0	0.0	0.0	0.0	34.0	0.0	128.0
0.0	1.0	0.0	0.0	0.0	0.0	0.0	0.0
0.0	1.0	0.0	0.0	0.0	0.0	0.0	0.0
0.0	2.0	0.0	0.0	0.0	0.0	0.0	0.0
0.0	2.0	0.0	0.0	0.0	0.0	0.0	0.0
0.0	1.0	0.0	0.0	0.0	0.0	0.0	0.0

0.0	1.0	0.0	0.0	0.0	0.0	0.0	0.0
0.0	1.0	0.0	0.0	0.0	0.0	0.0	0.0
0.0	1.0	0.0	0.0	0.0	0.0	0.0	0.0
0.0	1.0	0.0	0.0	0.0	0.0	0.0	0.0
0.0	2.0	0.0	0.0	0.0	0.0	0.0	0.0
0.0	1.0	0.0	0.0	0.0	0.0	0.0	0.0
0.0	2.0	0.0	0.0	0.0	0.0	0.0	0.0
0.0	2.0	0.0	0.0	0.0	0.0	0.0	0.0
0.0	2.0	0.0	0.0	0.0	0.0	0.0	0.0
0.0	2.0	0.0	0.0	0.0	0.0	0.0	0.0
0.0	2.0	0.0	0.0	0.0	0.0	15.0	0.0
0.0	2.0	0.0	0.0	0.0	0.0	0.0	0.0
0.0	1.0	0.0	0.0	0.0	0.0	0.0	0.0
0.0	2.0	0.0	0.0	0.0	0.0	0.0	0.0
0.0	1.0	0.0	0.0	0.0	0.0	0.0	0.0
0.0	2.0	0.0	0.0	0.0	3.0	0.0	0.0
0.0	1.0	0.0	0.0	0.0	0.0	0.0	0.0
0.0	1.0	0.0	0.0	0.0	0.0	0.0	0.0
0.0	1.0	0.0	0.0	0.0	0.0	0.0	0.0
0.0	1.0	0.0	0.0	0.0	0.0	0.0	0.0
0.0	1.0	0.0	0.0	0.0	0.0	0.0	0.0
0.0	1.0	0.0	0.0	0.0	0.0	0.0	0.0
0.0	1.0	0.0	0.0	0.0	0.0	0.0	0.0
0.0	1.0	0.0	0.0	0.0	0.0	0.0	0.0
0.0	1.0	0.0	0.0	0.0	0.0	0.0	0.0
0.0	1.0	0.0	0.0	0.0	0.0	0.0	0.0
0.0	1.0	0.0	0.0	0.0	0.0	0.0	0.0
0.0	1.0	0.0	0.0	0.0	0.0	0.0	0.0
0.0	1.0	0.0	0.0	0.0	0.0	0.0	0.0
0.0	0.0	136.0	0.0	0.0	0.0	0.0	0.0
0.0	0.0	67.0	0.0	0.0	0.0	0.0	0.0
0.0	0.0	39.0	0.0	0.0	0.0	0.0	0.0
0.0	0.0	37.0	0.0	0.0	0.0	0.0	0.0
0.0	0.0	37.0	0.0	0.0	0.0	0.0	0.0
0.0	0.0	20.0	0.0	0.0	0.0	0.0	0.0
0.0	0.0	10.0	0.0	0.0	0.0	0.0	0.0
0.0	0.0	12.0	0.0	0.0	0.0	0.0	0.0
0.0	0.0	24.0	0.0	0.0	0.0	0.0	0.0
0.0	0.0	19.0	0.0	0.0	0.0	0.0	0.0
0.0	0.0	12.0	0.0	0.0	0.0	0.0	0.0
0.0	0.0	12.0	0.0	0.0	0.0	1.0	0.0
0.0	0.0	14.0	0.0	0.0	0.0	0.0	0.0
0.0	0.0	8.0	0.0	0.0	0.0	0.0	0.0
0.0	0.0	12.0	0.0	0.0	0.0	0.0	0.0
0.0	0.0	6.0	0.0	0.0	0.0	0.0	0.0
0.0	0.0	6.0	0.0	0.0	0.0	0.0	0.0
0.0	0.0	14.0	0.0	0.0	0.0	0.0	0.0

0.0	0.0	8.0	0.0	0.0	0.0	0.0	0.0
0.0	0.0	5.0	0.0	0.0	0.0	0.0	0.0
0.0	0.0	6.0	0.0	0.0	2.0	0.0	0.0
0.0	0.0	3.0	0.0	0.0	0.0	0.0	0.0
0.0	0.0	2.0	0.0	0.0	0.0	0.0	0.0
0.0	0.0	1.0	0.0	0.0	0.0	0.0	0.0
0.0	0.0	3.0	0.0	0.0	0.0	0.0	0.0
0.0	0.0	2.0	0.0	0.0	0.0	0.0	0.0
0.0	0.0	2.0	0.0	0.0	0.0	0.0	0.0
0.0	0.0	1.0	0.0	0.0	0.0	0.0	0.0
0.0	0.0	3.0	0.0	0.0	0.0	0.0	0.0
0.0	0.0	2.0	0.0	0.0	0.0	0.0	0.0
0.0	0.0	2.0	0.0	0.0	0.0	0.0	0.0
0.0	0.0	1.0	0.0	139.0	0.0	0.0	0.0
0.0	0.0	2.0	0.0	0.0	0.0	0.0	0.0
0.0	0.0	1.0	0.0	0.0	0.0	2.0	0.0
0.0	0.0	1.0	0.0	0.0	0.0	0.0	0.0
0.0	0.0	2.0	0.0	0.0	0.0	0.0	0.0
0.0	0.0	2.0	0.0	0.0	0.0	0.0	0.0
0.0	0.0	1.0	0.0	0.0	0.0	0.0	0.0
0.0	0.0	1.0	0.0	0.0	0.0	0.0	0.0
0.0	0.0	1.0	0.0	0.0	0.0	0.0	0.0
0.0	0.0	1.0	0.0	0.0	0.0	0.0	0.0
0.0	0.0	1.0	0.0	0.0	0.0	0.0	0.0
0.0	0.0	0.0	0.0	0.0	0.0	0.0	0.0
0.0	0.0	1.0	0.0	0.0	0.0	0.0	0.0
0.0	0.0	0.0	0.0	0.0	0.0	0.0	0.0
0.0	0.0	0.0	0.0	0.0	0.0	0.0	0.0
0.0	0.0	1.0	0.0	0.0	0.0	0.0	0.0
0.0	0.0	1.0	0.0	0.0	0.0	0.0	0.0
0.0	0.0	2.0	0.0	0.0	0.0	0.0	0.0
0.0	0.0	2.0	0.0	0.0	0.0	0.0	0.0
0.0	0.0	1.0	0.0	0.0	0.0	0.0	0.0
0.0	0.0	0.0	0.0	0.0	0.0	0.0	0.0
0.0	0.0	1.0	0.0	0.0	0.0	0.0	0.0
0.0	0.0	0.0	0.0	0.0	0.0	0.0	0.0
0.0	0.0	0.0	0.0	0.0	0.0	0.0	0.0
0.0	0.0	1.0	0.0	0.0	0.0	0.0	0.0
0.0	0.0	0.0	0.0	0.0	0.0	0.0	0.0
0.0	0.0	0.0	0.0	0.0	0.0	0.0	0.0
0.0	0.0	1.0	0.0	0.0	0.0	0.0	0.0
0.0	0.0	0.0	0.0	0.0	0.0	0.0	0.0
0.0	0.0	1.0	0.0	0.0	0.0	0.0	0.0
0.0	0.0	1.0	0.0	0.0	1.0	14.0	2.0
0.0	0.0	2.0	0.0	1.0	0.0	0.0	0.0
0.0	0.0	1.0	0.0	0.0	0.0	0.0	0.0
0.0	0.0	0.0	0.0	0.0	0.0	0.0	0.0

0.0	0.0	1.0	0.0	0.0	0.0	0.0	0.0
0.0	0.0	1.0	0.0	0.0	0.0	0.0	0.0
0.0	0.0	1.0	0.0	0.0	0.0	0.0	0.0
0.0	0.0	1.0	0.0	0.0	0.0	0.0	0.0
0.0	0.0	0.0	0.0	0.0	0.0	0.0	0.0
0.0	0.0	1.0	0.0	0.0	0.0	0.0	0.0
0.0	0.0	0.0	0.0	0.0	0.0	0.0	0.0
0.0	0.0	0.0	0.0	0.0	0.0	0.0	0.0
0.0	0.0	0.0	0.0	0.0	0.0	0.0	0.0
0.0	0.0	1.0	0.0	0.0	0.0	0.0	0.0
0.0	0.0	0.0	0.0	0.0	0.0	0.0	0.0
0.0	0.0	0.0	0.0	0.0	0.0	0.0	0.0
0.0	0.0	0.0	0.0	0.0	0.0	0.0	138.0
0.0	0.0	2.0	0.0	0.0	0.0	0.0	0.0
0.0	0.0	0.0	0.0	0.0	0.0	0.0	0.0
0.0	0.0	1.0	0.0	0.0	0.0	0.0	0.0
0.0	0.0	1.0	0.0	0.0	0.0	0.0	0.0
0.0	0.0	0.0	0.0	0.0	0.0	0.0	0.0
0.0	0.0	0.0	0.0	0.0	2.0	0.0	0.0
0.0	0.0	1.0	0.0	0.0	0.0	0.0	0.0
0.0	0.0	1.0	0.0	0.0	0.0	0.0	0.0
0.0	0.0	0.0	0.0	0.0	0.0	0.0	0.0
0.0	0.0	0.0	0.0	0.0	0.0	0.0	0.0
0.0	0.0	1.0	0.0	0.0	0.0	0.0	0.0
0.0	0.0	1.0	0.0	0.0	0.0	0.0	0.0
0.0	0.0	1.0	0.0	0.0	0.0	0.0	0.0
0.0	0.0	0.0	0.0	0.0	0.0	0.0	0.0
0.0	0.0	1.0	0.0	0.0	0.0	0.0	0.0
0.0	0.0	1.0	0.0	0.0	0.0	0.0	0.0
0.0	0.0	1.0	0.0	0.0	0.0	0.0	0.0
0.0	0.0	0.0	5139.0	0.0	0.0	0.0	0.0
0.0	0.0	0.0	1023.0	0.0	0.0	0.0	0.0
0.0	0.0	0.0	489.0	0.0	0.0	0.0	0.0
0.0	0.0	0.0	190.0	0.0	0.0	0.0	0.0
0.0	0.0	0.0	105.0	0.0	0.0	0.0	0.0
0.0	0.0	0.0	57.0	0.0	0.0	0.0	0.0
0.0	0.0	0.0	30.0	0.0	0.0	0.0	0.0
0.0	0.0	0.0	21.0	0.0	0.0	0.0	0.0
0.0	0.0	0.0	14.0	0.0	0.0	0.0	0.0
0.0	0.0	0.0	8.0	0.0	0.0	0.0	0.0
0.0	0.0	0.0	3.0	0.0	0.0	0.0	0.0
0.0	0.0	0.0	2.0	0.0	0.0	0.0	0.0
0.0	0.0	0.0	2.0	0.0	0.0	0.0	0.0
0.0	0.0	0.0	2.0	0.0	0.0	0.0	0.0
0.0	0.0	0.0	2.0	0.0	0.0	0.0	0.0
0.0	0.0	0.0	1.0	0.0	0.0	0.0	0.0
0.0	0.0	0.0	1.0	0.0	0.0	0.0	0.0

0.0	0.0	0.0	1.0	0.0	0.0	0.0	0.0
0.0	0.0	0.0	1.0	0.0	0.0	0.0	0.0
0.0	0.0	0.0	2.0	0.0	0.0	0.0	0.0
0.0	0.0	0.0	1.0	0.0	0.0	0.0	0.0
0.0	0.0	0.0	1.0	0.0	0.0	0.0	0.0
0.0	0.0	0.0	0.0	96.0	0.0	0.0	0.0
0.0	0.0	0.0	0.0	12.0	0.0	0.0	0.0
0.0	0.0	0.0	0.0	11.0	0.0	0.0	0.0
0.0	0.0	0.0	0.0	13.0	0.0	0.0	0.0
0.0	0.0	0.0	0.0	6.0	0.0	0.0	0.0
0.0	0.0	0.0	0.0	8.0	0.0	0.0	0.0
0.0	0.0	0.0	0.0	8.0	0.0	0.0	0.0
0.0	0.0	0.0	0.0	9.0	0.0	0.0	0.0
0.0	0.0	0.0	0.0	5.0	0.0	0.0	0.0
0.0	0.0	0.0	0.0	1.0	0.0	0.0	0.0
0.0	0.0	0.0	0.0	2.0	0.0	0.0	0.0
0.0	0.0	0.0	0.0	3.0	0.0	0.0	0.0
0.0	0.0	0.0	0.0	3.0	0.0	0.0	0.0
0.0	0.0	0.0	0.0	1.0	0.0	0.0	0.0
0.0	0.0	0.0	0.0	3.0	0.0	0.0	0.0
0.0	0.0	0.0	0.0	2.0	0.0	0.0	0.0
0.0	0.0	0.0	0.0	1.0	0.0	0.0	0.0
0.0	0.0	0.0	0.0	1.0	0.0	0.0	0.0
0.0	0.0	0.0	0.0	0.0	0.0	0.0	0.0
0.0	0.0	0.0	0.0	2.0	0.0	0.0	0.0
0.0	0.0	0.0	0.0	0.0	0.0	0.0	0.0
0.0	0.0	0.0	0.0	1.0	0.0	0.0	0.0
0.0	0.0	0.0	0.0	0.0	0.0	0.0	0.0
0.0	0.0	0.0	0.0	1.0	0.0	0.0	0.0
0.0	0.0	0.0	0.0	0.0	0.0	0.0	0.0
0.0	0.0	0.0	0.0	0.0	0.0	0.0	0.0
0.0	0.0	0.0	0.0	1.0	7.0	0.0	0.0
0.0	0.0	0.0	0.0	1.0	0.0	0.0	0.0
0.0	0.0	0.0	0.0	1.0	0.0	0.0	0.0
0.0	0.0	0.0	0.0	0.0	0.0	0.0	0.0
0.0	0.0	0.0	0.0	1.0	0.0	0.0	0.0
0.0	0.0	0.0	0.0	2.0	0.0	0.0	0.0
0.0	0.0	0.0	0.0	2.0	0.0	0.0	0.0
0.0	0.0	0.0	0.0	0.0	0.0	0.0	0.0
0.0	0.0	0.0	0.0	1.0	0.0	0.0	0.0
0.0	0.0	0.0	0.0	2.0	0.0	0.0	0.0
0.0	0.0	0.0	0.0	1.0	0.0	0.0	0.0
0.0	0.0	0.0	0.0	1.0	0.0	0.0	0.0
0.0	0.0	0.0	0.0	1.0	0.0	0.0	0.0
0.0	0.0	0.0	0.0	1.0	0.0	0.0	0.0
0.0	0.0	0.0	0.0	0.0	0.0	0.0	0.0
0.0	0.0	0.0	0.0	1.0	0.0	0.0	0.0

0.0	0.0	0.0	0.0	0.0	0.0	0.0	0.0
0.0	0.0	0.0	0.0	1.0	0.0	0.0	0.0
0.0	0.0	0.0	0.0	1.0	0.0	0.0	0.0
0.0	0.0	0.0	0.0	1.0	0.0	0.0	0.0
0.0	0.0	0.0	0.0	1.0	0.0	0.0	0.0
0.0	0.0	0.0	0.0	1.0	0.0	0.0	0.0
0.0	0.0	0.0	0.0	0.0	0.0	0.0	0.0
0.0	0.0	0.0	0.0	0.0	740.0	0.0	0.0
0.0	0.0	0.0	0.0	0.0	53.0	0.0	0.0
0.0	0.0	0.0	0.0	0.0	29.0	0.0	0.0
0.0	0.0	0.0	0.0	0.0	32.0	0.0	0.0
0.0	0.0	0.0	0.0	0.0	23.0	1.0	0.0
0.0	0.0	0.0	0.0	0.0	15.0	0.0	0.0
0.0	0.0	0.0	0.0	0.0	17.0	0.0	0.0
0.0	0.0	0.0	0.0	0.0	11.0	0.0	0.0
0.0	0.0	0.0	0.0	0.0	9.0	0.0	0.0
0.0	0.0	0.0	0.0	0.0	3.0	0.0	0.0
0.0	0.0	0.0	0.0	0.0	5.0	0.0	0.0
0.0	0.0	0.0	0.0	0.0	3.0	0.0	0.0
0.0	0.0	0.0	0.0	0.0	4.0	0.0	0.0
0.0	0.0	0.0	0.0	0.0	5.0	0.0	0.0
0.0	0.0	0.0	0.0	0.0	3.0	0.0	0.0
0.0	0.0	0.0	0.0	0.0	3.0	0.0	0.0
0.0	0.0	0.0	0.0	0.0	1.0	0.0	0.0
0.0	0.0	0.0	0.0	0.0	2.0	0.0	0.0
0.0	0.0	0.0	0.0	0.0	1.0	0.0	0.0
0.0	0.0	0.0	0.0	0.0	0.0	0.0	0.0
0.0	0.0	0.0	0.0	0.0	1.0	0.0	0.0
0.0	0.0	0.0	0.0	0.0	2.0	0.0	0.0
0.0	0.0	0.0	0.0	0.0	1.0	0.0	0.0
0.0	0.0	0.0	0.0	0.0	1.0	0.0	0.0
0.0	0.0	0.0	0.0	0.0	3.0	0.0	0.0
0.0	0.0	0.0	0.0	0.0	1.0	0.0	1.0
0.0	0.0	0.0	0.0	0.0	1.0	0.0	0.0
0.0	0.0	0.0	0.0	0.0	1.0	0.0	0.0
0.0	0.0	0.0	0.0	0.0	0.0	0.0	0.0
0.0	0.0	0.0	0.0	0.0	1.0	0.0	0.0
0.0	0.0	0.0	0.0	0.0	2.0	0.0	0.0
0.0	0.0	0.0	0.0	0.0	1.0	0.0	0.0
0.0	0.0	0.0	0.0	0.0	2.0	0.0	0.0
0.0	0.0	0.0	0.0	0.0	1.0	0.0	0.0
0.0	0.0	0.0	0.0	0.0	2.0	0.0	0.0
0.0	0.0	0.0	0.0	0.0	1.0	0.0	0.0
0.0	0.0	0.0	0.0	0.0	0.0	0.0	0.0
0.0	0.0	0.0	0.0	0.0	1.0	0.0	0.0
0.0	0.0	0.0	0.0	0.0	0.0	0.0	2.0
0.0	0.0	0.0	0.0	0.0	1.0	0.0	0.0

0.0	0.0	0.0	0.0	0.0	1.0	0.0	0.0
0.0	0.0	0.0	0.0	0.0	0.0	0.0	0.0
0.0	0.0	0.0	0.0	0.0	1.0	0.0	0.0
0.0	0.0	0.0	0.0	0.0	1.0	0.0	2.0
0.0	0.0	0.0	0.0	0.0	2.0	0.0	0.0
0.0	0.0	0.0	0.0	0.0	1.0	0.0	2.0
0.0	0.0	0.0	0.0	0.0	1.0	0.0	0.0
0.0	0.0	0.0	0.0	0.0	1.0	0.0	0.0
0.0	0.0	0.0	0.0	0.0	1.0	0.0	0.0
0.0	0.0	0.0	0.0	0.0	1.0	0.0	0.0
0.0	0.0	0.0	0.0	0.0	1.0	0.0	0.0
0.0	0.0	0.0	0.0	0.0	1.0	0.0	0.0
0.0	0.0	0.0	0.0	0.0	1.0	0.0	0.0
0.0	0.0	0.0	0.0	0.0	0.0	27.0	0.0
0.0	0.0	0.0	0.0	0.0	0.0	1.0	0.0
0.0	0.0	0.0	0.0	0.0	0.0	2.0	0.0
0.0	0.0	0.0	0.0	0.0	0.0	2.0	0.0
0.0	0.0	0.0	0.0	0.0	0.0	1.0	0.0
0.0	0.0	0.0	0.0	0.0	0.0	1.0	0.0
0.0	0.0	0.0	0.0	0.0	0.0	1.0	0.0
0.0	0.0	0.0	0.0	0.0	0.0	1.0	0.0
0.0	0.0	0.0	0.0	0.0	0.0	3.0	0.0
0.0	0.0	0.0	0.0	0.0	0.0	1.0	0.0
0.0	0.0	0.0	0.0	0.0	0.0	1.0	0.0
0.0	0.0	0.0	0.0	0.0	0.0	1.0	0.0
0.0	0.0	0.0	0.0	0.0	0.0	1.0	0.0
0.0	0.0	0.0	0.0	0.0	0.0	2.0	0.0
0.0	0.0	0.0	0.0	0.0	0.0	2.0	0.0
0.0	0.0	0.0	0.0	0.0	0.0	1.0	0.0
0.0	0.0	0.0	0.0	0.0	0.0	1.0	0.0
0.0	0.0	0.0	0.0	0.0	0.0	1.0	0.0
0.0	0.0	0.0	0.0	0.0	0.0	1.0	0.0
0.0	0.0	0.0	0.0	0.0	0.0	1.0	0.0
0.0	0.0	0.0	0.0	0.0	0.0	1.0	0.0
0.0	0.0	0.0	0.0	0.0	0.0	1.0	0.0
0.0	0.0	0.0	0.0	0.0	0.0	1.0	0.0
0.0	0.0	0.0	0.0	0.0	0.0	1.0	0.0
0.0	0.0	0.0	0.0	0.0	0.0	2.0	0.0
0.0	0.0	0.0	0.0	0.0	0.0	0.0	0.0
0.0	0.0	0.0	0.0	0.0	0.0	1.0	0.0
0.0	0.0	0.0	0.0	0.0	0.0	0.0	0.0
0.0	0.0	0.0	0.0	0.0	0.0	1.0	0.0
0.0	0.0	0.0	0.0	0.0	0.0	0.0	144.0
0.0	0.0	0.0	0.0	0.0	0.0	0.0	24.0
0.0	0.0	0.0	0.0	0.0	0.0	0.0	11.0
0.0	0.0	0.0	0.0	0.0	0.0	0.0	9.0
0.0	0.0	0.0	0.0	0.0	0.0	0.0	5.0

0.0	0.0	0.0	0.0	0.0	0.0	0.0	7.0
0.0	0.0	0.0	0.0	0.0	0.0	0.0	6.0
0.0	0.0	0.0	0.0	0.0	0.0	0.0	4.0
0.0	0.0	0.0	0.0	0.0	0.0	0.0	3.0
0.0	0.0	0.0	0.0	0.0	0.0	0.0	2.0
0.0	0.0	0.0	0.0	0.0	0.0	0.0	1.0
0.0	0.0	0.0	0.0	0.0	0.0	0.0	1.0
0.0	0.0	0.0	0.0	0.0	0.0	0.0	2.0
0.0	0.0	0.0	0.0	0.0	0.0	0.0	2.0
0.0	0.0	0.0	0.0	0.0	0.0	0.0	2.0
0.0	0.0	0.0	0.0	0.0	0.0	0.0	1.0
0.0	0.0	0.0	0.0	0.0	0.0	0.0	1.0
0.0	0.0	0.0	0.0	0.0	0.0	0.0	2.0
0.0	0.0	0.0	0.0	0.0	0.0	0.0	1.0
0.0	0.0	0.0	0.0	0.0	0.0	0.0	2.0
0.0	0.0	0.0	0.0	0.0	0.0	0.0	1.0
0.0	0.0	0.0	0.0	0.0	0.0	0.0	1.0
0.0	0.0	0.0	0.0	0.0	0.0	0.0	1.0
0.0	0.0	0.0	0.0	0.0	0.0	0.0	1.0
0.0	0.0	0.0	0.0	0.0	0.0	0.0	0.0
0.0	0.0	0.0	0.0	0.0	0.0	0.0	1.0
0.0	0.0	0.0	0.0	0.0	0.0	0.0	1.0
0.0	0.0	0.0	0.0	0.0	0.0	0.0	0.0
0.0	0.0	0.0	0.0	0.0	0.0	0.0	0.0
0.0	0.0	0.0	0.0	0.0	0.0	0.0	0.0
0.0	0.0	0.0	0.0	0.0	0.0	0.0	0.0
0.0	0.0	0.0	0.0	0.0	0.0	0.0	0.0
0.0	0.0	0.0	0.0	0.0	0.0	0.0	0.0
0.0	0.0	0.0	0.0	0.0	0.0	0.0	0.0
0.0	0.0	0.0	0.0	0.0	0.0	0.0	0.0
0.0	0.0	0.0	0.0	0.0	0.0	0.0	0.0
0.0	0.0	0.0	0.0	0.0	0.0	0.0	0.0
0.0	0.0	0.0	0.0	0.0	0.0	0.0	0.0
0.0	0.0	0.0	0.0	0.0	0.0	0.0	0.0
0.0	0.0	0.0	0.0	0.0	0.0	0.0	0.0
0.0	0.0	0.0	0.0	0.0	0.0	0.0	0.0
0.0	0.0	0.0	0.0	0.0	0.0	0.0	0.0
0.0	0.0	0.0	0.0	0.0	0.0	0.0	0.0
0.0	0.0	0.0	0.0	0.0	0.0	0.0	0.0
0.0	0.0	0.0	0.0	0.0	0.0	0.0	0.0
0.0	0.0	0.0	0.0	0.0	0.0	0.0	0.0
0.0	0.0	0.0	0.0	0.0	0.0	0.0	0.0
0.0	0.0	0.0	0.0	0.0	0.0	0.0	0.0
0.0	0.0	0.0	0.0	0.0	0.0	0.0	0.0
0.0	0.0	0.0	0.0	0.0	0.0	0.0	0.0
0.0	0.0	0.0	0.0	0.0	0.0	0.0	0.0
0.0	0.0	0.0	0.0	0.0	0.0	0.0	0.0

0.0	0.0	0.0	0.0	0.0	0.0	0.0	0.0
0.0	0.0	0.0	0.0	0.0	0.0	0.0	0.0
0.0	0.0	0.0	0.0	0.0	0.0	0.0	0.0
0.0	0.0	0.0	0.0	0.0	0.0	0.0	0.0
0.0	0.0	0.0	0.0	0.0	0.0	0.0	0.0
0.0	0.0	0.0	0.0	0.0	0.0	0.0	0.0
0.0	0.0	0.0	0.0	0.0	0.0	0.0	0.0
0.0	0.0	0.0	0.0	0.0	0.0	0.0	0.0
0.0	0.0	0.0	0.0	0.0	0.0	0.0	0.0
0.0	0.0	0.0	0.0	0.0	0.0	0.0	0.0
0.0	0.0	0.0	0.0	0.0	0.0	0.0	0.0
0.0	0.0	0.0	0.0	0.0	0.0	0.0	0.0
0.0	0.0	0.0	0.0	0.0	0.0	0.0	0.0
0.0	0.0	0.0	0.0	0.0	0.0	0.0	0.0
0.0	0.0	0.0	0.0	0.0	0.0	0.0	0.0
0.0	0.0	0.0	0.0	0.0	0.0	0.0	0.0
0.0	0.0	0.0	0.0	0.0	0.0	0.0	0.0
0.0	0.0	0.0	0.0	0.0	0.0	0.0	0.0
0.0	0.0	0.0	0.0	0.0	0.0	0.0	0.0
0.0	0.0	0.0	0.0	0.0	0.0	0.0	0.0
0.0	0.0	0.0	0.0	0.0	0.0	0.0	0.0
0.0	0.0	0.0	0.0	0.0	0.0	0.0	0.0
0.0	0.0	0.0	0.0	0.0	0.0	0.0	0.0
0.0	0.0	0.0	0.0	0.0	0.0	0.0	0.0
0.0	0.0	0.0	0.0	0.0	0.0	0.0	0.0
0.0	0.0	0.0	0.0	0.0	0.0	0.0	0.0
0.0	0.0	0.0	0.0	0.0	0.0	0.0	0.0
0.0	0.0	0.0	0.0	0.0	0.0	0.0	0.0
0.0	0.0	0.0	0.0	0.0	0.0	0.0	0.0
0.0	0.0	0.0	0.0	0.0	0.0	0.0	0.0
0.0	0.0	0.0	0.0	0.0	0.0	0.0	0.0
0.0	0.0	0.0	0.0	0.0	0.0	0.0	0.0
0.0	0.0	0.0	0.0	0.0	0.0	0.0	0.0
0.0	0.0	0.0	0.0	0.0	0.0	0.0	0.0
0.0	0.0	0.0	0.0	0.0	0.0	0.0	0.0
0.0	0.0	0.0	0.0	0.0	0.0	0.0	0.0
0.0	0.0	0.0	0.0	0.0	0.0	0.0	0.0
0.0	0.0	0.0	0.0	0.0	0.0	0.0	0.0
0.0	0.0	0.0	0.0	0.0	0.0	0.0	0.0
0.0	0.0	0.0	0.0	0.0	0.0	0.0	0.0
0.0	0.0	0.0	0.0	0.0	0.0	0.0	0.0
0.0	0.0	0.0	0.0	0.0	0.0	0.0	0.0
0.0	0.0	0.0	0.0	0.0	0.0	0.0	0.0
0.0	0.0	0.0	0.0	0.0	0.0	0.0	0.0
0.0	0.0	0.0	0.0	0.0	0.0	0.0	0.0
0.0	0.0	0.0	0.0	0.0	0.0	0.0	0.0
0.0	0.0	0.0	0.0	0.0	0.0	0.0	0.0
0.0	0.0	0.0	0.0	0.0	0.0	0.0	0.0

0.0	0.0	0.0	0.0	0.0	0.0	0.0	0.0
0.0	0.0	0.0	0.0	0.0	0.0	0.0	0.0
0.0	0.0	0.0	0.0	0.0	0.0	0.0	0.0
0.0	0.0	0.0	0.0	0.0	0.0	0.0	0.0
0.0	0.0	0.0	0.0	0.0	0.0	0.0	0.0
0.0	0.0	0.0	0.0	0.0	0.0	0.0	0.0
0.0	0.0	0.0	0.0	0.0	0.0	0.0	0.0
0.0	0.0	0.0	0.0	0.0	0.0	0.0	0.0
0.0	0.0	0.0	0.0	0.0	0.0	0.0	0.0
0.0	0.0	0.0	0.0	0.0	0.0	0.0	0.0
0.0	0.0	0.0	0.0	0.0	0.0	0.0	0.0
0.0	0.0	0.0	0.0	0.0	0.0	0.0	0.0
0.0	0.0	0.0	0.0	0.0	0.0	0.0	0.0
0.0	0.0	0.0	0.0	0.0	0.0	0.0	0.0
0.0	0.0	0.0	0.0	0.0	0.0	0.0	0.0
0.0	0.0	0.0	0.0	0.0	0.0	0.0	0.0
0.0	0.0	0.0	0.0	0.0	0.0	0.0	0.0
0.0	0.0	0.0	0.0	0.0	0.0	0.0	0.0
0.0	0.0	0.0	0.0	0.0	0.0	0.0	0.0
0.0	0.0	0.0	0.0	0.0	0.0	0.0	0.0
0.0	0.0	0.0	0.0	0.0	0.0	0.0	0.0
0.0	0.0	0.0	0.0	0.0	0.0	0.0	0.0
0.0	0.0	0.0	0.0	0.0	0.0	0.0	0.0
0.0	0.0	0.0	0.0	0.0	0.0	0.0	0.0
0.0	0.0	0.0	0.0	0.0	0.0	0.0	0.0
0.0	0.0	0.0	0.0	0.0	0.0	0.0	0.0
0.0	0.0	0.0	0.0	0.0	0.0	0.0	0.0
0.0	0.0	0.0	0.0	0.0	0.0	0.0	0.0
0.0	0.0	0.0	0.0	0.0	0.0	0.0	0.0
0.0	0.0	0.0	0.0	0.0	0.0	0.0	0.0
0.0	0.0	0.0	0.0	0.0	0.0	0.0	0.0
0.0	0.0	0.0	0.0	0.0	0.0	0.0	0.0
0.0	0.0	0.0	0.0	0.0	0.0	0.0	0.0
0.0	0.0	0.0	0.0	0.0	0.0	0.0	0.0
0.0	0.0	0.0	0.0	0.0	0.0	0.0	0.0
0.0	0.0	0.0	0.0	0.0	0.0	0.0	0.0
0.0	0.0	0.0	0.0	0.0	0.0	0.0	0.0
0.0	0.0	0.0	0.0	0.0	0.0	0.0	0.0
0.0	0.0	0.0	0.0	0.0	0.0	0.0	0.0
0.0	0.0	0.0	0.0	0.0	0.0	0.0	0.0
0.0	0.0	0.0	0.0	0.0	0.0	0.0	0.0
0.0	0.0	0.0	0.0	0.0	0.0	0.0	0.0
0.0	0.0	0.0	0.0	0.0	0.0	0.0	0.0
0.0	0.0	0.0	0.0	0.0	0.0	0.0	0.0
0.0	0.0	0.0	0.0	0.0	0.0	0.0	0.0
0.0	0.0	0.0	0.0	0.0	0.0	0.0	0.0
0.0	0.0	0.0	0.0	0.0	0.0	0.0	0.0

0.0	0.0	0.0	0.0	0.0	0.0	0.0	0.0
0.0	0.0	0.0	0.0	0.0	0.0	0.0	0.0
0.0	0.0	0.0	0.0	0.0	0.0	0.0	0.0
0.0	0.0	0.0	0.0	0.0	0.0	0.0	0.0
0.0	0.0	0.0	0.0	0.0	0.0	0.0	0.0
0.0	0.0	0.0	0.0	0.0	0.0	0.0	0.0
0.0	0.0	0.0	0.0	0.0	0.0	0.0	0.0
0.0	0.0	0.0	0.0	0.0	0.0	0.0	0.0
0.0	0.0	0.0	0.0	0.0	0.0	0.0	0.0
0.0	0.0	0.0	0.0	0.0	0.0	0.0	0.0
0.0	0.0	0.0	0.0	0.0	0.0	0.0	0.0
0.0	0.0	0.0	0.0	0.0	0.0	0.0	0.0
0.0	0.0	0.0	0.0	0.0	0.0	0.0	0.0
0.0	0.0	0.0	0.0	0.0	0.0	0.0	0.0
0.0	0.0	0.0	0.0	0.0	0.0	0.0	0.0
0.0	0.0	0.0	0.0	0.0	0.0	0.0	0.0
0.0	0.0	0.0	0.0	0.0	0.0	0.0	0.0
0.0	0.0	0.0	0.0	0.0	0.0	0.0	0.0
0.0	0.0	0.0	0.0	0.0	0.0	0.0	0.0
0.0	0.0	0.0	0.0	0.0	0.0	0.0	0.0
0.0	0.0	0.0	0.0	0.0	0.0	0.0	0.0
0.0	0.0	0.0	0.0	0.0	0.0	0.0	0.0
0.0	0.0	0.0	0.0	0.0	0.0	0.0	0.0
0.0	0.0	0.0	0.0	0.0	0.0	0.0	0.0
0.0	0.0	0.0	0.0	0.0	0.0	0.0	0.0
0.0	0.0	0.0	0.0	0.0	0.0	0.0	0.0
0.0	0.0	0.0	0.0	0.0	0.0	0.0	0.0
0.0	0.0	0.0	0.0	0.0	0.0	0.0	0.0
0.0	0.0	0.0	0.0	0.0	0.0	0.0	0.0
0.0	0.0	0.0	0.0	0.0	0.0	0.0	0.0
0.0	0.0	0.0	0.0	0.0	0.0	0.0	0.0
0.0	0.0	0.0	0.0	0.0	0.0	0.0	0.0
0.0	0.0	0.0	0.0	0.0	0.0	0.0	0.0
0.0	0.0	0.0	0.0	0.0	0.0	0.0	0.0
0.0	0.0	0.0	0.0	0.0	0.0	0.0	0.0
0.0	0.0	0.0	0.0	0.0	0.0	0.0	0.0
0.0	0.0	0.0	0.0	0.0	0.0	0.0	0.0
0.0	0.0	0.0	0.0	0.0	0.0	0.0	0.0
0.0	0.0	0.0	0.0	0.0	0.0	0.0	0.0
0.0	0.0	0.0	0.0	0.0	0.0	0.0	0.0
0.0	0.0	0.0	0.0	0.0	0.0	0.0	0.0
0.0	0.0	0.0	0.0	0.0	0.0	0.0	0.0
0.0	0.0	0.0	0.0	0.0	0.0	0.0	0.0
0.0	0.0	0.0	0.0	0.0	0.0	0.0	0.0
0.0	0.0	0.0	0.0	0.0	0.0	0.0	0.0
0.0	0.0	0.0	0.0	0.0	0.0	0.0	0.0
0.0	0.0	0.0	0.0	0.0	0.0	0.0	0.0
0.0	0.0	0.0	0.0	0.0	0.0	0.0	0.0

0.0	0.0	0.0	0.0	0.0	0.0	0.0	0.0
0.0	0.0	0.0	0.0	0.0	0.0	0.0	0.0
0.0	0.0	0.0	0.0	0.0	0.0	0.0	0.0
0.0	0.0	0.0	0.0	0.0	0.0	0.0	0.0
0.0	0.0	0.0	0.0	0.0	0.0	0.0	0.0
0.0	0.0	0.0	0.0	0.0	0.0	0.0	0.0
0.0	0.0	0.0	0.0	0.0	0.0	0.0	0.0
0.0	0.0	0.0	0.0	0.0	0.0	0.0	0.0
0.0	0.0	0.0	0.0	0.0	0.0	0.0	0.0
0.0	0.0	0.0	0.0	0.0	0.0	0.0	0.0
0.0	0.0	0.0	0.0	0.0	0.0	0.0	0.0
0.0	0.0	0.0	0.0	0.0	0.0	0.0	0.0
0.0	0.0	0.0	0.0	0.0	0.0	0.0	0.0
0.0	0.0	0.0	0.0	0.0	0.0	0.0	0.0
0.0	0.0	0.0	0.0	0.0	0.0	0.0	0.0
0.0	0.0	0.0	0.0	0.0	0.0	0.0	0.0
0.0	0.0	0.0	0.0	0.0	0.0	0.0	0.0
0.0	0.0	0.0	0.0	0.0	0.0	0.0	0.0
0.0	0.0	0.0	0.0	0.0	0.0	0.0	0.0
0.0	0.0	0.0	0.0	0.0	0.0	0.0	0.0
0.0	0.0	0.0	0.0	0.0	0.0	0.0	0.0
0.0	0.0	0.0	0.0	0.0	0.0	0.0	0.0
0.0	0.0	0.0	0.0	0.0	0.0	0.0	0.0
0.0	0.0	0.0	0.0	0.0	0.0	0.0	0.0
0.0	0.0	0.0	0.0	0.0	0.0	0.0	0.0
0.0	0.0	0.0	0.0	0.0	0.0	0.0	0.0
0.0	0.0	0.0	0.0	0.0	0.0	0.0	0.0
0.0	0.0	0.0	0.0	0.0	0.0	0.0	0.0
0.0	0.0	0.0	0.0	0.0	0.0	0.0	0.0
0.0	0.0	0.0	0.0	0.0	0.0	0.0	0.0
0.0	0.0	0.0	0.0	0.0	0.0	0.0	0.0
0.0	0.0	0.0	0.0	0.0	0.0	0.0	0.0
0.0	0.0	0.0	0.0	0.0	0.0	0.0	0.0
0.0	0.0	0.0	0.0	0.0	0.0	0.0	0.0
0.0	0.0	0.0	0.0	0.0	0.0	0.0	0.0
0.0	0.0	0.0	0.0	0.0	0.0	0.0	0.0
0.0	0.0	0.0	0.0	0.0	0.0	0.0	0.0
0.0	0.0	0.0	0.0	0.0	0.0	0.0	0.0
0.0	0.0	0.0	0.0	0.0	0.0	0.0	0.0
0.0	0.0	0.0	0.0	0.0	0.0	0.0	0.0
0.0	0.0	0.0	0.0	0.0	0.0	0.0	0.0
0.0	0.0	0.0	0.0	0.0	0.0	0.0	0.0
0.0	0.0	0.0	0.0	0.0	0.0	0.0	0.0
0.0	0.0	0.0	0.0	0.0	0.0	0.0	0.0
0.0	0.0	0.0	0.0	0.0	0.0	0.0	0.0
0.0	0.0	0.0	0.0	0.0	0.0	0.0	0.0
0.0	0.0	0.0	0.0	0.0	0.0	0.0	0.0

0.0	0.0	0.0	0.0	0.0	0.0	0.0	0.0
0.0	0.0	0.0	0.0	0.0	0.0	0.0	0.0
0.0	0.0	0.0	0.0	0.0	0.0	0.0	0.0
0.0	0.0	0.0	0.0	0.0	0.0	0.0	0.0
0.0	0.0	0.0	0.0	0.0	0.0	0.0	0.0
0.0	0.0	0.0	0.0	0.0	0.0	0.0	0.0
0.0	0.0	0.0	0.0	0.0	0.0	0.0	0.0
0.0	0.0	0.0	0.0	0.0	0.0	0.0	0.0
0.0	0.0	0.0	0.0	0.0	0.0	0.0	0.0
0.0	0.0	0.0	0.0	0.0	0.0	0.0	0.0
0.0	0.0	0.0	0.0	0.0	0.0	0.0	0.0
0.0	0.0	0.0	0.0	0.0	0.0	0.0	0.0
0.0	0.0	0.0	0.0	0.0	0.0	0.0	0.0
0.0	0.0	0.0	0.0	0.0	0.0	0.0	0.0
0.0	0.0	0.0	0.0	0.0	0.0	0.0	0.0
0.0	0.0	0.0	0.0	0.0	0.0	0.0	0.0
0.0	0.0	0.0	0.0	0.0	0.0	0.0	0.0
0.0	0.0	0.0	0.0	0.0	0.0	0.0	0.0
0.0	0.0	0.0	0.0	0.0	0.0	0.0	0.0
0.0	0.0	0.0	0.0	0.0	0.0	0.0	0.0
0.0	0.0	0.0	0.0	0.0	0.0	0.0	0.0
0.0	0.0	0.0	0.0	0.0	0.0	0.0	0.0
0.0	0.0	0.0	0.0	0.0	0.0	0.0	0.0
0.0	0.0	0.0	0.0	0.0	0.0	0.0	0.0
0.0	0.0	0.0	0.0	0.0	0.0	0.0	0.0
0.0	0.0	0.0	0.0	0.0	0.0	0.0	0.0
0.0	0.0	0.0	0.0	0.0	0.0	0.0	0.0
0.0	0.0	0.0	0.0	0.0	0.0	0.0	0.0
0.0	0.0	0.0	0.0	0.0	0.0	0.0	0.0
0.0	0.0	0.0	0.0	0.0	0.0	0.0	0.0
0.0	0.0	0.0	0.0	0.0	0.0	0.0	0.0
0.0	0.0	0.0	0.0	0.0	0.0	0.0	0.0
0.0	0.0	0.0	0.0	0.0	0.0	0.0	0.0
0.0	0.0	0.0	0.0	0.0	0.0	0.0	0.0
0.0	0.0	0.0	0.0	0.0	0.0	0.0	0.0
0.0	0.0	0.0	0.0	0.0	0.0	0.0	0.0
0.0	0.0	0.0	0.0	0.0	0.0	0.0	0.0
0.0	0.0	0.0	0.0	0.0	0.0	0.0	0.0
0.0	0.0	0.0	0.0	0.0	0.0	0.0	0.0
0.0	0.0	0.0	0.0	0.0	0.0	0.0	0.0
0.0	0.0	0.0	0.0	0.0	0.0	0.0	0.0
0.0	0.0	0.0	0.0	0.0	0.0	0.0	0.0
0.0	0.0	0.0	0.0	0.0	0.0	0.0	0.0
0.0	0.0	0.0	0.0	0.0	0.0	0.0	0.0
0.0	0.0	0.0	0.0	0.0	0.0	0.0	0.0
0.0	0.0	0.0	0.0	0.0	0.0	0.0	0.0
0.0	0.0	0.0	0.0	0.0	0.0	0.0	0.0

0.0	0.0	0.0	0.0	0.0	0.0	0.0	0.0
0.0	0.0	0.0	0.0	0.0	0.0	0.0	0.0
0.0	0.0	0.0	0.0	0.0	0.0	0.0	0.0
0.0	0.0	0.0	0.0	0.0	0.0	0.0	0.0
0.0	0.0	0.0	0.0	0.0	0.0	0.0	0.0
0.0	0.0	0.0	0.0	0.0	0.0	0.0	0.0
0.0	0.0	0.0	0.0	0.0	0.0	0.0	0.0
0.0	0.0	0.0	0.0	0.0	0.0	0.0	0.0
0.0	0.0	0.0	0.0	0.0	0.0	0.0	0.0
0.0	0.0	0.0	0.0	0.0	0.0	0.0	0.0
0.0	0.0	0.0	0.0	0.0	0.0	0.0	0.0
0.0	0.0	0.0	0.0	0.0	0.0	0.0	0.0
0.0	0.0	0.0	0.0	0.0	0.0	0.0	0.0
0.0	0.0	0.0	0.0	0.0	0.0	0.0	0.0
0.0	0.0	0.0	0.0	0.0	0.0	0.0	0.0
0.0	0.0	0.0	0.0	0.0	0.0	0.0	0.0
0.0	0.0	0.0	0.0	0.0	0.0	0.0	0.0
0.0	0.0	0.0	0.0	0.0	0.0	0.0	0.0
0.0	0.0	0.0	0.0	0.0	0.0	0.0	0.0
0.0	0.0	0.0	0.0	0.0	0.0	0.0	0.0
0.0	0.0	0.0	0.0	0.0	0.0	0.0	0.0
0.0	0.0	0.0	0.0	0.0	0.0	0.0	0.0
0.0	0.0	0.0	0.0	0.0	0.0	0.0	0.0
0.0	0.0	0.0	0.0	0.0	0.0	0.0	0.0
0.0	0.0	0.0	0.0	0.0	0.0	0.0	0.0
0.0	0.0	0.0	0.0	0.0	0.0	0.0	0.0
0.0	0.0	0.0	0.0	0.0	0.0	0.0	0.0
0.0	0.0	0.0	0.0	0.0	0.0	0.0	0.0
0.0	0.0	0.0	0.0	0.0	0.0	0.0	0.0
0.0	0.0	0.0	0.0	0.0	0.0	0.0	0.0
0.0	0.0	0.0	0.0	0.0	0.0	0.0	0.0
0.0	0.0	0.0	0.0	0.0	0.0	0.0	0.0
0.0	0.0	0.0	0.0	0.0	0.0	0.0	0.0
0.0	0.0	0.0	0.0	0.0	0.0	0.0	0.0
0.0	0.0	0.0	0.0	0.0	0.0	0.0	0.0
0.0	0.0	0.0	0.0	0.0	0.0	0.0	0.0
0.0	0.0	0.0	0.0	0.0	0.0	0.0	0.0
0.0	0.0	0.0	0.0	0.0	0.0	0.0	0.0
0.0	0.0	0.0	0.0	0.0	0.0	0.0	0.0
0.0	0.0	0.0	0.0	0.0	0.0	0.0	0.0
0.0	0.0	0.0	0.0	0.0	0.0	0.0	0.0
0.0	0.0	0.0	0.0	0.0	0.0	0.0	0.0
0.0	0.0	0.0	0.0	0.0	0.0	0.0	0.0
0.0	0.0	0.0	0.0	0.0	0.0	0.0	0.0
0.0	0.0	0.0	0.0	0.0	0.0	0.0	0.0
0.0	0.0	0.0	0.0	0.0	0.0	0.0	0.0
0.0	0.0	0.0	0.0	0.0	0.0	0.0	0.0
0.0	0.0	0.0	0.0	0.0	0.0	0.0	0.0

0.0	0.0	0.0	0.0	0.0	0.0	0.0	0.0
0.0	0.0	0.0	0.0	0.0	0.0	0.0	0.0
0.0	0.0	0.0	0.0	0.0	0.0	0.0	0.0
0.0	0.0	0.0	0.0	0.0	0.0	0.0	0.0
0.0	0.0	0.0	0.0	0.0	0.0	0.0	0.0
0.0	0.0	0.0	0.0	0.0	0.0	0.0	0.0
0.0	0.0	0.0	0.0	0.0	0.0	0.0	0.0
0.0	0.0	0.0	0.0	0.0	0.0	0.0	0.0
0.0	0.0	0.0	0.0	0.0	0.0	0.0	0.0
0.0	0.0	0.0	0.0	0.0	0.0	0.0	0.0
0.0	0.0	0.0	0.0	0.0	0.0	0.0	0.0
0.0	0.0	0.0	0.0	0.0	0.0	0.0	0.0
0.0	0.0	0.0	0.0	0.0	0.0	0.0	0.0
0.0	0.0	0.0	0.0	0.0	0.0	0.0	0.0
0.0	0.0	0.0	0.0	0.0	0.0	0.0	0.0
0.0	0.0	0.0	0.0	0.0	0.0	0.0	0.0
0.0	0.0	0.0	0.0	0.0	0.0	0.0	0.0
0.0	0.0	0.0	0.0	0.0	0.0	0.0	0.0
0.0	0.0	0.0	0.0	0.0	0.0	0.0	0.0
0.0	0.0	0.0	0.0	0.0	0.0	0.0	0.0
0.0	0.0	0.0	0.0	0.0	0.0	0.0	0.0
0.0	0.0	0.0	0.0	0.0	0.0	0.0	0.0
0.0	0.0	0.0	0.0	0.0	0.0	0.0	0.0
0.0	0.0	0.0	0.0	0.0	0.0	0.0	0.0
0.0	0.0	0.0	0.0	0.0	0.0	0.0	0.0
0.0	0.0	0.0	0.0	0.0	0.0	0.0	0.0
0.0	0.0	0.0	0.0	0.0	0.0	0.0	0.0
0.0	0.0	0.0	0.0	0.0	0.0	0.0	0.0
0.0	0.0	0.0	0.0	0.0	0.0	0.0	0.0
0.0	0.0	0.0	0.0	0.0	0.0	0.0	0.0
0.0	0.0	0.0	0.0	0.0	0.0	0.0	0.0
0.0	0.0	0.0	0.0	0.0	0.0	0.0	0.0
0.0	0.0	0.0	0.0	0.0	0.0	0.0	0.0
0.0	0.0	0.0	0.0	0.0	0.0	0.0	0.0
0.0	0.0	0.0	0.0	0.0	0.0	0.0	0.0
0.0	0.0	0.0	0.0	0.0	0.0	0.0	0.0
0.0	0.0	0.0	0.0	0.0	0.0	0.0	0.0
0.0	0.0	0.0	0.0	0.0	0.0	0.0	0.0
0.0	0.0	0.0	0.0	0.0	0.0	0.0	0.0
0.0	0.0	0.0	0.0	0.0	0.0	0.0	0.0
0.0	0.0	0.0	0.0	0.0	0.0	0.0	0.0
0.0	0.0	0.0	0.0	0.0	0.0	0.0	0.0
0.0	0.0	0.0	0.0	0.0	0.0	0.0	0.0
0.0	0.0	0.0	0.0	0.0	0.0	0.0	0.0
0.0	0.0	0.0	0.0	0.0	0.0	0.0	0.0
0.0	0.0	0.0	0.0	0.0	0.0	0.0	0.0
0.0	0.0	0.0	0.0	0.0	0.0	0.0	0.0

0.0	0.0	0.0	0.0	0.0	0.0	0.0	0.0
0.0	0.0	0.0	0.0	0.0	0.0	0.0	0.0
0.0	0.0	0.0	0.0	0.0	0.0	0.0	0.0
0.0	0.0	0.0	0.0	0.0	0.0	0.0	0.0
0.0	0.0	0.0	0.0	0.0	0.0	0.0	0.0
0.0	0.0	0.0	0.0	0.0	0.0	0.0	0.0
0.0	0.0	0.0	0.0	0.0	0.0	0.0	0.0
0.0	0.0	0.0	0.0	0.0	0.0	0.0	0.0
0.0	0.0	0.0	0.0	0.0	0.0	0.0	0.0
0.0	0.0	0.0	0.0	0.0	0.0	0.0	0.0
0.0	0.0	0.0	0.0	0.0	0.0	0.0	0.0
0.0	0.0	0.0	0.0	0.0	0.0	0.0	0.0
0.0	0.0	0.0	0.0	0.0	0.0	0.0	0.0
0.0	0.0	0.0	0.0	0.0	0.0	0.0	0.0
0.0	0.0	0.0	0.0	0.0	0.0	0.0	0.0
0.0	0.0	0.0	0.0	0.0	0.0	0.0	0.0
0.0	0.0	0.0	0.0	0.0	0.0	0.0	0.0
0.0	0.0	0.0	0.0	0.0	0.0	0.0	0.0
0.0	0.0	0.0	0.0	0.0	0.0	0.0	0.0
0.0	0.0	0.0	0.0	0.0	0.0	0.0	0.0
0.0	0.0	0.0	0.0	0.0	0.0	0.0	0.0
0.0	0.0	0.0	0.0	0.0	0.0	0.0	0.0
0.0	0.0	0.0	0.0	0.0	0.0	0.0	0.0
0.0	0.0	0.0	0.0	0.0	0.0	0.0	0.0
0.0	0.0	0.0	0.0	0.0	0.0	0.0	0.0
0.0	0.0	0.0	0.0	0.0	0.0	0.0	0.0
0.0	0.0	0.0	0.0	0.0	0.0	0.0	0.0
0.0	0.0	0.0	0.0	0.0	0.0	0.0	0.0
0.0	0.0	0.0	0.0	0.0	0.0	0.0	0.0
0.0	0.0	0.0	0.0	0.0	0.0	0.0	0.0
0.0	0.0	0.0	0.0	0.0	0.0	0.0	0.0
0.0	0.0	0.0	0.0	0.0	0.0	0.0	0.0
0.0	0.0	0.0	0.0	0.0	0.0	0.0	0.0
0.0	0.0	0.0	0.0	0.0	0.0	0.0	0.0
0.0	0.0	0.0	0.0	0.0	0.0	0.0	0.0
0.0	0.0	0.0	0.0	0.0	0.0	0.0	0.0
0.0	0.0	0.0	0.0	0.0	0.0	0.0	0.0
0.0	0.0	0.0	0.0	0.0	0.0	0.0	0.0
0.0	0.0	0.0	0.0	0.0	0.0	0.0	0.0
0.0	0.0	0.0	0.0	0.0	0.0	0.0	0.0
0.0	0.0	0.0	0.0	0.0	0.0	0.0	0.0
0.0	0.0	0.0	0.0	0.0	0.0	0.0	0.0
0.0	0.0	0.0	0.0	0.0	0.0	0.0	0.0
0.0	0.0	0.0	0.0	0.0	0.0	0.0	0.0
0.0	0.0	0.0	0.0	0.0	0.0	0.0	0.0
0.0	0.0	0.0	0.0	0.0	0.0	0.0	0.0
0.0	0.0	0.0	0.0	0.0	0.0	0.0	0.0

0.0	0.0	0.0	0.0	0.0	0.0	0.0	0.0
0.0	0.0	0.0	0.0	0.0	0.0	0.0	0.0
0.0	0.0	0.0	0.0	0.0	0.0	0.0	0.0
0.0	0.0	0.0	0.0	0.0	0.0	0.0	0.0
0.0	0.0	0.0	0.0	0.0	0.0	0.0	0.0
0.0	0.0	0.0	0.0	0.0	0.0	0.0	0.0
0.0	0.0	0.0	0.0	0.0	0.0	0.0	0.0
0.0	0.0	0.0	0.0	0.0	0.0	0.0	0.0
0.0	0.0	0.0	0.0	0.0	0.0	0.0	0.0
0.0	0.0	0.0	0.0	0.0	0.0	0.0	0.0
0.0	0.0	0.0	0.0	0.0	0.0	0.0	0.0
0.0	0.0	0.0	0.0	0.0	0.0	0.0	0.0
0.0	0.0	0.0	0.0	0.0	0.0	0.0	0.0
0.0	0.0	0.0	0.0	0.0	0.0	0.0	0.0
0.0	0.0	0.0	0.0	0.0	0.0	0.0	0.0
0.0	0.0	0.0	0.0	0.0	0.0	0.0	0.0
0.0	0.0	0.0	0.0	0.0	0.0	0.0	0.0
0.0	0.0	0.0	0.0	0.0	0.0	0.0	0.0
0.0	0.0	0.0	0.0	0.0	0.0	0.0	0.0
0.0	0.0	0.0	0.0	0.0	0.0	0.0	0.0
0.0	0.0	0.0	0.0	0.0	0.0	0.0	0.0
0.0	0.0	0.0	0.0	0.0	0.0	0.0	0.0
0.0	0.0	0.0	0.0	0.0	0.0	0.0	0.0
0.0	0.0	0.0	0.0	0.0	0.0	0.0	0.0
0.0	0.0	0.0	0.0	0.0	0.0	0.0	0.0
0.0	0.0	0.0	0.0	0.0	0.0	0.0	0.0
0.0	0.0	0.0	0.0	0.0	0.0	0.0	0.0
0.0	0.0	0.0	0.0	0.0	0.0	0.0	0.0
0.0	0.0	0.0	0.0	0.0	0.0	0.0	0.0
0.0	0.0	0.0	0.0	0.0	0.0	0.0	0.0
0.0	0.0	0.0	0.0	0.0	0.0	0.0	0.0
0.0	0.0	0.0	0.0	0.0	0.0	0.0	0.0
0.0	0.0	0.0	0.0	0.0	0.0	0.0	0.0
0.0	0.0	0.0	0.0	0.0	0.0	0.0	0.0
0.0	0.0	0.0	0.0	0.0	0.0	0.0	0.0
0.0	0.0	0.0	0.0	0.0	0.0	0.0	0.0
0.0	0.0	0.0	0.0	0.0	0.0	0.0	0.0
0.0	0.0	0.0	0.0	0.0	0.0	0.0	0.0
0.0	0.0	0.0	0.0	0.0	0.0	0.0	0.0
0.0	0.0	0.0	0.0	0.0	0.0	0.0	0.0
0.0	0.0	0.0	0.0	0.0	0.0	0.0	0.0
0.0	0.0	0.0	0.0	0.0	0.0	0.0	0.0
0.0	0.0	0.0	0.0	0.0	0.0	0.0	0.0
0.0	0.0	0.0	0.0	0.0	0.0	0.0	0.0
0.0	0.0	0.0	0.0	0.0	0.0	0.0	0.0
0.0	0.0	0.0	0.0	0.0	0.0	0.0	0.0
0.0	0.0	0.0	0.0	0.0	0.0	0.0	0.0

0.0	0.0	0.0	0.0	0.0	0.0	0.0	0.0
0.0	0.0	0.0	0.0	0.0	0.0	0.0	0.0
0.0	0.0	0.0	0.0	0.0	0.0	0.0	0.0
0.0	0.0	0.0	0.0	0.0	0.0	0.0	0.0
0.0	0.0	0.0	0.0	0.0	0.0	0.0	0.0
0.0	0.0	0.0	0.0	0.0	0.0	0.0	0.0
0.0	0.0	0.0	0.0	0.0	0.0	0.0	0.0
0.0	0.0	0.0	0.0	0.0	0.0	0.0	0.0
0.0	0.0	0.0	0.0	0.0	0.0	0.0	0.0
0.0	0.0	0.0	0.0	0.0	0.0	0.0	0.0
0.0	0.0	0.0	0.0	0.0	0.0	0.0	0.0
0.0	0.0	0.0	0.0	0.0	0.0	0.0	0.0
0.0	0.0	0.0	0.0	0.0	0.0	0.0	0.0
0.0	0.0	0.0	0.0	0.0	0.0	0.0	0.0
0.0	0.0	0.0	0.0	0.0	0.0	0.0	0.0
0.0	0.0	0.0	0.0	0.0	0.0	0.0	0.0
0.0	0.0	0.0	0.0	0.0	0.0	0.0	0.0
0.0	0.0	0.0	0.0	0.0	0.0	0.0	0.0
0.0	0.0	0.0	0.0	0.0	0.0	0.0	0.0
0.0	0.0	0.0	0.0	0.0	0.0	0.0	0.0
0.0	0.0	0.0	0.0	0.0	0.0	0.0	0.0
0.0	0.0	0.0	0.0	0.0	0.0	0.0	0.0
0.0	0.0	0.0	0.0	0.0	0.0	0.0	0.0
0.0	0.0	0.0	0.0	0.0	0.0	0.0	0.0
0.0	0.0	0.0	0.0	0.0	0.0	0.0	0.0
0.0	0.0	0.0	0.0	0.0	0.0	0.0	0.0
0.0	0.0	0.0	0.0	0.0	0.0	0.0	0.0
0.0	0.0	0.0	0.0	0.0	0.0	0.0	0.0
0.0	0.0	0.0	0.0	0.0	0.0	0.0	0.0
0.0	0.0	0.0	0.0	0.0	0.0	0.0	0.0
0.0	0.0	0.0	0.0	0.0	0.0	0.0	0.0
0.0	0.0	0.0	0.0	0.0	0.0	0.0	0.0
0.0	0.0	0.0	0.0	0.0	0.0	0.0	0.0
0.0	0.0	0.0	0.0	0.0	0.0	0.0	0.0
0.0	0.0	0.0	0.0	0.0	0.0	0.0	0.0
0.0	0.0	0.0	0.0	0.0	0.0	0.0	0.0
0.0	0.0	0.0	0.0	0.0	0.0	0.0	0.0
0.0	0.0	0.0	0.0	0.0	0.0	0.0	0.0
0.0	0.0	0.0	0.0	0.0	0.0	0.0	0.0
0.0	0.0	0.0	0.0	0.0	0.0	0.0	0.0
0.0	0.0	0.0	0.0	0.0	0.0	0.0	0.0
0.0	0.0	0.0	0.0	0.0	0.0	0.0	0.0
0.0	0.0	0.0	0.0	0.0	0.0	0.0	0.0
0.0	0.0	0.0	0.0	0.0	0.0	0.0	0.0
0.0	0.0	0.0	0.0	0.0	0.0	0.0	0.0
0.0	0.0	0.0	0.0	0.0	0.0	0.0	0.0
0.0	0.0	0.0	0.0	0.0	0.0	0.0	0.0

0.0	0.0	0.0	0.0	0.0	0.0	0.0	0.0
0.0	0.0	0.0	0.0	0.0	0.0	0.0	0.0
0.0	0.0	0.0	0.0	0.0	0.0	0.0	0.0
0.0	0.0	0.0	0.0	0.0	0.0	0.0	0.0
0.0	0.0	0.0	0.0	0.0	0.0	0.0	0.0
0.0	0.0	0.0	0.0	0.0	0.0	0.0	0.0
0.0	0.0	0.0	0.0	0.0	0.0	0.0	0.0
0.0	0.0	0.0	0.0	0.0	0.0	0.0	0.0
0.0	0.0	0.0	0.0	0.0	0.0	0.0	0.0
0.0	0.0	0.0	0.0	0.0	0.0	0.0	0.0
0.0	0.0	0.0	0.0	0.0	0.0	0.0	0.0
0.0	0.0	0.0	0.0	0.0	0.0	0.0	0.0
0.0	0.0	0.0	0.0	0.0	0.0	0.0	0.0
0.0	0.0	0.0	0.0	0.0	0.0	0.0	0.0
0.0	0.0	0.0	0.0	0.0	0.0	0.0	0.0
0.0	0.0	0.0	0.0	0.0	0.0	0.0	0.0
0.0	0.0	0.0	0.0	0.0	0.0	0.0	0.0
0.0	0.0	0.0	0.0	0.0	0.0	0.0	0.0
0.0	0.0	0.0	0.0	0.0	0.0	0.0	0.0
0.0	0.0	0.0	0.0	0.0	0.0	0.0	0.0
0.0	0.0	0.0	0.0	0.0	0.0	0.0	0.0
0.0	0.0	0.0	0.0	0.0	0.0	0.0	0.0
0.0	0.0	0.0	0.0	0.0	0.0	0.0	0.0
0.0	0.0	0.0	0.0	0.0	0.0	0.0	0.0
0.0	0.0	0.0	0.0	0.0	0.0	0.0	0.0
0.0	0.0	0.0	0.0	0.0	0.0	0.0	0.0
0.0	0.0	0.0	0.0	0.0	0.0	0.0	0.0
0.0	0.0	0.0	0.0	0.0	0.0	0.0	0.0
0.0	0.0	0.0	0.0	0.0	0.0	0.0	0.0
0.0	0.0	0.0	0.0	0.0	0.0	0.0	0.0
0.0	0.0	0.0	0.0	0.0	0.0	0.0	0.0
0.0	0.0	0.0	0.0	0.0	0.0	0.0	0.0
0.0	0.0	0.0	0.0	0.0	0.0	0.0	0.0
0.0	0.0	0.0	0.0	0.0	0.0	0.0	0.0
0.0	0.0	0.0	0.0	0.0	0.0	0.0	0.0
0.0	0.0	0.0	0.0	0.0	0.0	0.0	0.0
0.0	0.0	0.0	0.0	0.0	0.0	0.0	0.0
0.0	0.0	0.0	0.0	0.0	0.0	0.0	0.0
0.0	0.0	0.0	0.0	0.0	0.0	0.0	0.0
0.0	0.0	0.0	0.0	0.0	0.0	0.0	0.0
0.0	0.0	0.0	0.0	0.0	0.0	0.0	0.0
0.0	0.0	0.0	0.0	0.0	0.0	0.0	0.0
0.0	0.0	0.0	0.0	0.0	0.0	0.0	0.0
0.0	0.0	0.0	0.0	0.0	0.0	0.0	0.0
0.0	0.0	0.0	0.0	0.0	0.0	0.0	0.0
0.0	0.0	0.0	0.0	0.0	0.0	0.0	0.0
0.0	0.0	0.0	0.0	0.0	0.0	0.0	0.0
0.0	0.0	0.0	0.0	0.0	0.0	0.0	0.0

0.0	0.0	0.0	0.0	0.0	0.0	0.0	0.0
0.0	0.0	0.0	0.0	0.0	0.0	0.0	0.0
0.0	0.0	0.0	0.0	0.0	0.0	0.0	0.0
0.0	0.0	0.0	0.0	0.0	0.0	0.0	0.0
0.0	0.0	0.0	0.0	0.0	0.0	0.0	0.0
0.0	0.0	0.0	0.0	0.0	0.0	0.0	0.0
0.0	0.0	0.0	0.0	0.0	0.0	0.0	0.0
0.0	0.0	0.0	0.0	0.0	0.0	0.0	0.0
0.0	0.0	0.0	0.0	0.0	0.0	0.0	0.0
0.0	0.0	0.0	0.0	0.0	0.0	0.0	0.0
0.0	0.0	0.0	0.0	0.0	0.0	0.0	0.0
0.0	0.0	0.0	0.0	0.0	0.0	0.0	0.0
0.0	0.0	0.0	0.0	0.0	0.0	0.0	0.0
0.0	0.0	0.0	0.0	0.0	0.0	0.0	0.0
0.0	0.0	0.0	0.0	0.0	0.0	0.0	0.0
0.0	0.0	0.0	0.0	0.0	0.0	0.0	0.0
0.0	0.0	0.0	0.0	0.0	0.0	0.0	0.0
0.0	0.0	0.0	0.0	0.0	0.0	0.0	0.0
0.0	0.0	0.0	0.0	0.0	0.0	0.0	0.0
0.0	0.0	0.0	0.0	0.0	0.0	0.0	0.0
0.0	0.0	0.0	0.0	0.0	0.0	0.0	0.0
0.0	0.0	0.0	0.0	0.0	0.0	0.0	0.0
0.0	0.0	0.0	0.0	0.0	0.0	0.0	0.0
0.0	0.0	0.0	0.0	0.0	0.0	0.0	0.0
0.0	0.0	0.0	0.0	0.0	0.0	0.0	0.0
0.0	0.0	0.0	0.0	0.0	0.0	0.0	0.0
0.0	0.0	0.0	0.0	0.0	0.0	0.0	0.0
0.0	0.0	0.0	0.0	0.0	0.0	0.0	0.0
0.0	0.0	0.0	0.0	0.0	0.0	0.0	0.0
0.0	0.0	0.0	0.0	0.0	0.0	0.0	0.0
0.0	0.0	0.0	0.0	0.0	0.0	0.0	0.0
0.0	0.0	0.0	0.0	0.0	0.0	0.0	0.0
0.0	0.0	0.0	0.0	0.0	0.0	0.0	0.0
0.0	0.0	0.0	0.0	0.0	0.0	0.0	0.0
0.0	0.0	0.0	0.0	0.0	0.0	0.0	0.0
0.0	0.0	0.0	0.0	0.0	0.0	0.0	0.0
0.0	0.0	0.0	0.0	0.0	0.0	0.0	0.0
0.0	0.0	0.0	0.0	0.0	0.0	0.0	0.0
0.0	0.0	0.0	0.0	0.0	0.0	0.0	0.0
0.0	0.0	0.0	0.0	0.0	0.0	0.0	0.0
0.0	0.0	0.0	0.0	0.0	0.0	0.0	0.0
0.0	0.0	0.0	0.0	0.0	0.0	0.0	0.0
0.0	0.0	0.0	0.0	0.0	0.0	0.0	0.0
0.0	0.0	0.0	0.0	0.0	0.0	0.0	0.0
0.0	0.0	0.0	0.0	0.0	0.0	0.0	0.0
0.0	0.0	0.0	0.0	0.0	0.0	0.0	0.0
0.0	0.0	0.0	0.0	0.0	0.0	0.0	0.0
0.0	0.0	0.0	0.0	0.0	0.0	0.0	0.0

0.0	0.0	0.0	0.0	0.0	0.0	0.0	0.0
0.0	0.0	0.0	0.0	0.0	0.0	0.0	0.0
0.0	0.0	0.0	0.0	0.0	0.0	0.0	0.0
0.0	0.0	0.0	0.0	0.0	0.0	0.0	0.0
0.0	0.0	0.0	0.0	0.0	0.0	0.0	0.0
0.0	0.0	0.0	0.0	0.0	0.0	0.0	0.0
0.0	0.0	0.0	0.0	0.0	0.0	0.0	0.0
0.0	0.0	0.0	0.0	0.0	0.0	0.0	0.0
0.0	0.0	0.0	0.0	0.0	0.0	0.0	0.0
0.0	0.0	0.0	0.0	0.0	0.0	0.0	0.0
0.0	0.0	0.0	0.0	0.0	0.0	0.0	0.0
0.0	0.0	0.0	0.0	0.0	0.0	0.0	0.0
0.0	0.0	0.0	0.0	0.0	0.0	0.0	0.0
0.0	0.0	0.0	0.0	0.0	0.0	0.0	0.0
0.0	0.0	0.0	0.0	0.0	0.0	0.0	0.0
0.0	0.0	0.0	0.0	0.0	0.0	0.0	0.0
0.0	0.0	0.0	0.0	0.0	0.0	0.0	0.0
0.0	0.0	0.0	0.0	0.0	0.0	0.0	0.0
0.0	0.0	0.0	0.0	0.0	0.0	0.0	0.0
0.0	0.0	0.0	0.0	0.0	0.0	0.0	0.0
0.0	0.0	0.0	0.0	0.0	0.0	0.0	0.0
0.0	0.0	0.0	0.0	0.0	0.0	0.0	0.0
0.0	0.0	0.0	0.0	0.0	0.0	0.0	0.0
0.0	0.0	0.0	0.0	0.0	0.0	0.0	0.0
0.0	0.0	0.0	0.0	0.0	0.0	0.0	0.0
0.0	0.0	0.0	0.0	0.0	0.0	0.0	0.0
0.0	0.0	0.0	0.0	0.0	0.0	0.0	0.0
0.0	0.0	0.0	0.0	0.0	0.0	0.0	0.0
0.0	0.0	0.0	0.0	0.0	0.0	0.0	0.0
0.0	0.0	0.0	0.0	0.0	0.0	0.0	0.0
0.0	0.0	0.0	0.0	0.0	0.0	0.0	0.0
0.0	0.0	0.0	0.0	0.0	0.0	0.0	0.0
0.0	0.0	0.0	0.0	0.0	0.0	0.0	0.0
0.0	0.0	0.0	0.0	0.0	0.0	0.0	0.0
0.0	0.0	0.0	0.0	0.0	0.0	0.0	0.0
0.0	0.0	0.0	0.0	0.0	0.0	0.0	0.0
0.0	0.0	0.0	0.0	0.0	0.0	0.0	0.0
0.0	0.0	0.0	0.0	0.0	0.0	0.0	0.0
0.0	0.0	0.0	0.0	0.0	0.0	0.0	0.0
0.0	0.0	0.0	0.0	0.0	0.0	0.0	0.0
0.0	0.0	0.0	0.0	0.0	0.0	0.0	0.0
0.0	0.0	0.0	0.0	0.0	0.0	0.0	0.0
0.0	0.0	0.0	0.0	0.0	0.0	0.0	0.0
0.0	0.0	0.0	0.0	0.0	0.0	0.0	0.0
0.0	0.0	0.0	0.0	0.0	0.0	0.0	0.0
0.0	0.0	0.0	0.0	0.0	0.0	0.0	0.0
0.0	0.0	0.0	0.0	0.0	0.0	0.0	0.0

0.0	0.0	0.0	0.0	0.0	0.0	0.0	0.0
0.0	0.0	0.0	0.0	0.0	0.0	0.0	0.0
0.0	0.0	0.0	0.0	0.0	0.0	0.0	0.0
0.0	0.0	0.0	0.0	0.0	0.0	0.0	0.0
0.0	0.0	0.0	0.0	0.0	0.0	0.0	0.0
0.0	0.0	0.0	0.0	0.0	0.0	0.0	0.0
0.0	0.0	0.0	0.0	0.0	0.0	0.0	0.0
0.0	0.0	0.0	0.0	0.0	0.0	0.0	0.0
0.0	0.0	0.0	0.0	0.0	0.0	0.0	0.0
0.0	0.0	0.0	0.0	0.0	0.0	0.0	0.0
0.0	0.0	0.0	0.0	0.0	0.0	0.0	0.0
0.0	0.0	0.0	0.0	0.0	0.0	0.0	0.0
0.0	0.0	0.0	0.0	0.0	0.0	0.0	0.0
0.0	0.0	0.0	0.0	0.0	0.0	0.0	0.0
0.0	0.0	0.0	0.0	0.0	0.0	0.0	0.0
0.0	0.0	0.0	0.0	0.0	0.0	0.0	0.0
0.0	0.0	0.0	0.0	0.0	0.0	0.0	0.0
0.0	0.0	0.0	0.0	0.0	0.0	0.0	0.0
0.0	0.0	0.0	0.0	0.0	0.0	0.0	0.0
0.0	0.0	0.0	0.0	0.0	0.0	0.0	0.0
0.0	0.0	0.0	0.0	0.0	0.0	0.0	0.0
0.0	0.0	0.0	0.0	0.0	0.0	0.0	0.0
0.0	0.0	0.0	0.0	0.0	0.0	0.0	0.0
0.0	0.0	0.0	0.0	0.0	0.0	0.0	0.0
0.0	0.0	0.0	0.0	0.0	0.0	0.0	0.0
0.0	0.0	0.0	0.0	0.0	0.0	0.0	0.0
0.0	0.0	0.0	0.0	0.0	0.0	0.0	0.0
0.0	0.0	0.0	0.0	0.0	0.0	0.0	0.0
0.0	0.0	0.0	0.0	0.0	0.0	0.0	0.0
0.0	0.0	0.0	0.0	0.0	0.0	0.0	0.0
0.0	0.0	0.0	0.0	0.0	0.0	0.0	0.0
0.0	0.0	0.0	0.0	0.0	0.0	0.0	0.0
0.0	0.0	0.0	0.0	0.0	0.0	0.0	0.0
0.0	0.0	0.0	0.0	0.0	0.0	0.0	0.0
0.0	0.0	0.0	0.0	0.0	0.0	0.0	0.0
0.0	0.0	0.0	0.0	0.0	0.0	0.0	0.0
0.0	0.0	0.0	0.0	0.0	0.0	0.0	0.0
0.0	0.0	0.0	0.0	0.0	0.0	0.0	0.0
0.0	0.0	0.0	0.0	0.0	0.0	0.0	0.0
0.0	0.0	0.0	0.0	0.0	0.0	0.0	0.0
0.0	0.0	0.0	0.0	0.0	0.0	0.0	0.0
0.0	0.0	0.0	0.0	0.0	0.0	0.0	0.0
0.0	0.0	0.0	0.0	0.0	0.0	0.0	0.0
0.0	0.0	0.0	0.0	0.0	0.0	0.0	0.0
0.0	0.0	0.0	0.0	0.0	0.0	0.0	0.0
0.0	0.0	0.0	0.0	0.0	0.0	0.0	0.0
0.0	0.0	0.0	0.0	0.0	0.0	0.0	0.0
0.0	0.0	0.0	0.0	0.0	0.0	0.0	0.0

0.0	0.0	0.0	0.0	0.0	0.0	0.0	0.0
0.0	0.0	0.0	0.0	0.0	0.0	0.0	0.0
0.0	0.0	0.0	0.0	0.0	0.0	0.0	0.0
0.0	0.0	0.0	0.0	0.0	0.0	0.0	0.0
0.0	0.0	0.0	0.0	0.0	0.0	0.0	0.0
0.0	0.0	0.0	0.0	0.0	0.0	0.0	0.0
0.0	0.0	0.0	0.0	0.0	0.0	0.0	0.0
0.0	0.0	0.0	0.0	0.0	0.0	0.0	0.0
0.0	0.0	0.0	0.0	0.0	0.0	0.0	0.0
0.0	0.0	0.0	0.0	0.0	0.0	0.0	0.0
0.0	0.0	0.0	0.0	0.0	0.0	0.0	0.0
0.0	0.0	0.0	0.0	0.0	0.0	0.0	0.0
0.0	0.0	0.0	0.0	0.0	0.0	0.0	0.0
0.0	0.0	0.0	0.0	0.0	0.0	0.0	0.0
0.0	0.0	0.0	0.0	0.0	0.0	0.0	0.0
0.0	0.0	0.0	0.0	0.0	0.0	0.0	0.0
0.0	0.0	0.0	0.0	0.0	0.0	0.0	0.0
0.0	0.0	0.0	0.0	0.0	0.0	0.0	0.0
0.0	0.0	0.0	0.0	0.0	0.0	0.0	0.0
0.0	0.0	0.0	0.0	0.0	0.0	0.0	0.0
0.0	0.0	0.0	0.0	0.0	0.0	0.0	0.0
0.0	0.0	0.0	0.0	0.0	0.0	0.0	0.0
0.0	0.0	0.0	0.0	0.0	0.0	0.0	0.0
0.0	0.0	0.0	0.0	0.0	0.0	0.0	0.0
0.0	0.0	0.0	0.0	0.0	0.0	0.0	0.0
0.0	0.0	0.0	0.0	0.0	0.0	0.0	0.0
0.0	0.0	0.0	0.0	0.0	0.0	0.0	0.0
0.0	0.0	0.0	0.0	0.0	0.0	0.0	0.0
0.0	0.0	0.0	0.0	0.0	0.0	0.0	0.0
0.0	0.0	0.0	0.0	0.0	0.0	0.0	0.0
0.0	0.0	0.0	0.0	0.0	0.0	0.0	0.0
0.0	0.0	0.0	0.0	0.0	0.0	0.0	0.0
0.0	0.0	0.0	0.0	0.0	0.0	0.0	0.0
0.0	0.0	0.0	0.0	0.0	0.0	0.0	0.0
0.0	0.0	0.0	0.0	0.0	0.0	0.0	0.0
0.0	0.0	0.0	0.0	0.0	0.0	0.0	0.0
0.0	0.0	0.0	0.0	0.0	0.0	0.0	0.0
0.0	0.0	0.0	0.0	0.0	0.0	0.0	0.0
0.0	0.0	0.0	0.0	0.0	0.0	0.0	0.0
0.0	0.0	0.0	0.0	0.0	0.0	0.0	0.0
0.0	0.0	0.0	0.0	0.0	0.0	0.0	0.0
0.0	0.0	0.0	0.0	0.0	0.0	0.0	0.0
0.0	0.0	0.0	0.0	0.0	0.0	0.0	0.0
0.0	0.0	0.0	0.0	0.0	0.0	0.0	0.0
0.0	0.0	0.0	0.0	0.0	0.0	0.0	0.0
0.0	0.0	0.0	0.0	0.0	0.0	0.0	0.0
0.0	0.0	0.0	0.0	0.0	0.0	0.0	0.0
0.0	0.0	0.0	0.0	0.0	0.0	0.0	0.0

0.0	0.0	0.0	0.0	0.0	0.0	0.0	0.0
0.0	0.0	0.0	0.0	0.0	0.0	0.0	0.0
0.0	0.0	0.0	0.0	0.0	0.0	0.0	0.0
0.0	0.0	0.0	0.0	0.0	0.0	0.0	0.0
0.0	0.0	0.0	0.0	0.0	0.0	0.0	0.0
0.0	0.0	0.0	0.0	0.0	0.0	0.0	0.0
0.0	0.0	0.0	0.0	0.0	0.0	0.0	0.0
0.0	0.0	0.0	0.0	0.0	0.0	0.0	0.0
0.0	0.0	0.0	0.0	0.0	0.0	0.0	0.0
0.0	0.0	0.0	0.0	0.0	0.0	0.0	0.0
0.0	0.0	0.0	0.0	0.0	0.0	0.0	0.0
0.0	0.0	0.0	0.0	0.0	0.0	0.0	0.0
0.0	0.0	0.0	0.0	0.0	0.0	0.0	0.0
0.0	0.0	0.0	0.0	0.0	0.0	0.0	0.0
0.0	0.0	0.0	0.0	0.0	0.0	0.0	0.0
0.0	0.0	0.0	0.0	0.0	0.0	0.0	0.0
0.0	0.0	0.0	0.0	0.0	0.0	0.0	0.0
0.0	0.0	0.0	0.0	0.0	0.0	0.0	0.0
0.0	0.0	0.0	0.0	0.0	0.0	0.0	0.0
0.0	0.0	0.0	0.0	0.0	0.0	0.0	0.0
0.0	0.0	0.0	0.0	0.0	0.0	0.0	0.0
0.0	0.0	0.0	0.0	0.0	0.0	0.0	0.0
0.0	0.0	0.0	0.0	0.0	0.0	0.0	0.0
0.0	0.0	0.0	0.0	0.0	0.0	0.0	0.0
0.0	0.0	0.0	0.0	0.0	0.0	0.0	0.0
0.0	0.0	0.0	0.0	0.0	0.0	0.0	0.0
0.0	0.0	0.0	0.0	0.0	0.0	0.0	0.0
0.0	0.0	0.0	0.0	0.0	0.0	0.0	0.0
0.0	0.0	0.0	0.0	0.0	0.0	0.0	0.0
0.0	0.0	0.0	0.0	0.0	0.0	0.0	0.0
0.0	0.0	0.0	0.0	0.0	0.0	0.0	0.0
0.0	0.0	0.0	0.0	0.0	0.0	0.0	0.0
0.0	0.0	0.0	0.0	0.0	0.0	0.0	0.0
0.0	0.0	0.0	0.0	0.0	0.0	0.0	0.0
0.0	0.0	0.0	0.0	0.0	0.0	0.0	0.0
0.0	0.0	0.0	0.0	0.0	0.0	0.0	0.0
0.0	0.0	0.0	0.0	0.0	0.0	0.0	0.0
0.0	0.0	0.0	0.0	0.0	0.0	0.0	0.0
0.0	0.0	0.0	0.0	0.0	0.0	0.0	0.0
0.0	0.0	0.0	0.0	0.0	0.0	0.0	0.0
0.0	0.0	0.0	0.0	0.0	0.0	0.0	0.0
0.0	0.0	0.0	0.0	0.0	0.0	0.0	0.0
0.0	0.0	0.0	0.0	0.0	0.0	0.0	0.0
0.0	0.0	0.0	0.0	0.0	0.0	0.0	0.0
0.0	0.0	0.0	0.0	0.0	0.0	0.0	0.0
0.0	0.0	0.0	0.0	0.0	0.0	0.0	0.0
0.0	0.0	0.0	0.0	0.0	0.0	0.0	0.0

0.0	0.0	0.0	0.0	0.0	0.0	0.0	0.0
0.0	0.0	0.0	0.0	0.0	0.0	0.0	0.0
0.0	0.0	0.0	0.0	0.0	0.0	0.0	0.0
0.0	0.0	0.0	0.0	0.0	0.0	0.0	0.0
0.0	0.0	0.0	0.0	0.0	0.0	0.0	0.0
0.0	0.0	0.0	0.0	0.0	0.0	0.0	0.0
0.0	0.0	0.0	0.0	0.0	0.0	0.0	0.0
0.0	0.0	0.0	0.0	0.0	0.0	0.0	0.0
0.0	0.0	0.0	0.0	0.0	0.0	0.0	0.0
0.0	0.0	0.0	0.0	0.0	0.0	0.0	0.0
0.0	0.0	0.0	0.0	0.0	0.0	0.0	0.0
0.0	0.0	0.0	0.0	0.0	0.0	0.0	0.0
0.0	0.0	0.0	0.0	0.0	0.0	0.0	0.0
0.0	0.0	0.0	0.0	0.0	0.0	0.0	0.0
0.0	0.0	0.0	0.0	0.0	0.0	0.0	0.0
0.0	0.0	0.0	0.0	0.0	0.0	0.0	0.0
0.0	0.0	0.0	0.0	0.0	0.0	0.0	0.0
0.0	0.0	0.0	0.0	0.0	0.0	0.0	0.0
0.0	0.0	0.0	0.0	0.0	0.0	0.0	0.0
0.0	0.0	0.0	0.0	0.0	0.0	0.0	0.0
0.0	0.0	0.0	0.0	0.0	0.0	0.0	0.0
0.0	0.0	0.0	0.0	0.0	0.0	0.0	0.0
0.0	0.0	0.0	0.0	0.0	0.0	0.0	0.0
0.0	0.0	0.0	0.0	0.0	0.0	0.0	0.0
0.0	0.0	0.0	0.0	0.0	0.0	0.0	0.0
0.0	0.0	0.0	0.0	0.0	0.0	0.0	0.0
0.0	0.0	0.0	0.0	0.0	0.0	0.0	0.0
0.0	0.0	0.0	0.0	0.0	0.0	0.0	0.0
0.0	0.0	0.0	0.0	0.0	0.0	0.0	0.0
0.0	0.0	0.0	0.0	0.0	0.0	0.0	0.0
0.0	0.0	0.0	0.0	0.0	0.0	0.0	0.0
0.0	0.0	0.0	0.0	0.0	0.0	0.0	0.0
0.0	0.0	0.0	0.0	0.0	0.0	0.0	0.0
0.0	0.0	0.0	0.0	0.0	0.0	0.0	0.0
0.0	0.0	0.0	0.0	0.0	0.0	0.0	0.0
0.0	0.0	0.0	0.0	0.0	0.0	0.0	0.0
0.0	0.0	0.0	0.0	0.0	0.0	0.0	0.0
0.0	0.0	0.0	0.0	0.0	0.0	0.0	0.0
0.0	0.0	0.0	0.0	0.0	0.0	0.0	0.0
0.0	0.0	0.0	0.0	0.0	0.0	0.0	0.0
0.0	0.0	0.0	0.0	0.0	0.0	0.0	0.0
0.0	0.0	0.0	0.0	0.0	0.0	0.0	0.0
0.0	0.0	0.0	0.0	0.0	0.0	0.0	0.0
0.0	0.0	0.0	0.0	0.0	0.0	0.0	0.0
0.0	0.0	0.0	0.0	0.0	0.0	0.0	0.0
0.0	0.0	0.0	0.0	0.0	0.0	0.0	0.0
0.0	0.0	0.0	0.0	0.0	0.0	0.0	0.0

0.0	0.0	0.0	0.0	0.0	0.0	0.0	0.0
0.0	0.0	0.0	0.0	0.0	0.0	0.0	0.0
0.0	0.0	0.0	0.0	0.0	0.0	0.0	0.0
0.0	0.0	0.0	0.0	0.0	0.0	0.0	0.0
0.0	0.0	0.0	0.0	0.0	0.0	0.0	0.0
0.0	0.0	0.0	0.0	0.0	0.0	0.0	0.0
0.0	0.0	0.0	0.0	0.0	0.0	0.0	0.0
0.0	0.0	0.0	0.0	0.0	0.0	0.0	0.0
0.0	0.0	0.0	0.0	0.0	0.0	0.0	0.0
0.0	0.0	0.0	0.0	0.0	0.0	0.0	0.0
0.0	0.0	0.0	0.0	0.0	0.0	0.0	0.0
0.0	0.0	0.0	0.0	0.0	0.0	0.0	0.0
0.0	0.0	0.0	0.0	0.0	0.0	0.0	0.0
0.0	0.0	0.0	0.0	0.0	0.0	0.0	0.0
0.0	0.0	0.0	0.0	0.0	0.0	0.0	0.0
0.0	0.0	0.0	0.0	0.0	0.0	0.0	0.0
0.0	0.0	0.0	0.0	0.0	0.0	0.0	0.0
0.0	0.0	0.0	0.0	0.0	0.0	0.0	0.0
0.0	0.0	0.0	0.0	0.0	0.0	0.0	0.0
0.0	0.0	0.0	0.0	0.0	0.0	0.0	0.0
0.0	0.0	0.0	0.0	0.0	0.0	0.0	0.0
0.0	0.0	0.0	0.0	0.0	0.0	0.0	0.0
0.0	0.0	0.0	0.0	0.0	0.0	0.0	0.0
0.0	0.0	0.0	0.0	0.0	0.0	0.0	0.0
0.0	0.0	0.0	0.0	0.0	0.0	0.0	0.0
0.0	0.0	0.0	0.0	0.0	0.0	0.0	0.0
0.0	0.0	0.0	0.0	0.0	0.0	0.0	0.0
0.0	0.0	0.0	0.0	0.0	0.0	0.0	0.0
0.0	0.0	0.0	0.0	0.0	0.0	0.0	0.0
0.0	0.0	0.0	0.0	0.0	0.0	0.0	0.0
0.0	0.0	0.0	0.0	0.0	0.0	0.0	0.0
0.0	0.0	0.0	0.0	0.0	0.0	0.0	0.0
0.0	0.0	0.0	0.0	0.0	0.0	0.0	0.0
0.0	0.0	0.0	0.0	0.0	0.0	0.0	0.0
0.0	0.0	0.0	0.0	0.0	0.0	0.0	0.0
0.0	0.0	0.0	0.0	0.0	0.0	0.0	0.0
0.0	0.0	0.0	0.0	0.0	0.0	0.0	0.0
0.0	0.0	0.0	0.0	0.0	0.0	0.0	0.0
0.0	0.0	0.0	0.0	0.0	0.0	0.0	0.0
0.0	0.0	0.0	0.0	0.0	0.0	0.0	0.0
0.0	0.0	0.0	0.0	0.0	0.0	0.0	0.0
0.0	0.0	0.0	0.0	0.0	0.0	0.0	0.0
0.0	0.0	0.0	0.0	0.0	0.0	0.0	0.0
0.0	0.0	0.0	0.0	0.0	0.0	0.0	0.0
0.0	0.0	0.0	0.0	0.0	0.0	0.0	0.0
0.0	0.0	0.0	0.0	0.0	0.0	0.0	0.0
0.0	0.0	0.0	0.0	0.0	0.0	0.0	0.0
0.0	0.0	0.0	0.0	0.0	0.0	0.0	0.0

0.0	0.0	0.0	0.0	0.0	0.0	0.0	0.0
0.0	0.0	0.0	0.0	0.0	0.0	0.0	0.0
0.0	0.0	0.0	0.0	0.0	0.0	0.0	0.0
0.0	0.0	0.0	0.0	0.0	0.0	0.0	0.0
0.0	0.0	0.0	0.0	0.0	0.0	0.0	0.0
0.0	0.0	0.0	0.0	0.0	0.0	0.0	0.0
0.0	0.0	0.0	0.0	0.0	0.0	0.0	0.0
0.0	0.0	0.0	0.0	0.0	0.0	0.0	0.0
0.0	0.0	0.0	0.0	0.0	0.0	0.0	0.0
0.0	0.0	0.0	0.0	0.0	0.0	0.0	0.0
0.0	0.0	0.0	0.0	0.0	0.0	0.0	0.0
0.0	0.0	0.0	0.0	0.0	0.0	0.0	0.0
0.0	0.0	0.0	0.0	0.0	0.0	0.0	0.0
0.0	0.0	0.0	0.0	0.0	0.0	0.0	0.0
0.0	0.0	0.0	0.0	0.0	0.0	0.0	0.0
0.0	0.0	0.0	0.0	0.0	0.0	0.0	0.0
0.0	0.0	0.0	0.0	0.0	0.0	0.0	0.0
0.0	0.0	0.0	0.0	0.0	0.0	0.0	0.0
0.0	0.0	0.0	0.0	0.0	0.0	0.0	0.0
0.0	0.0	0.0	0.0	0.0	0.0	0.0	0.0
0.0	0.0	0.0	0.0	0.0	0.0	0.0	0.0
0.0	0.0	0.0	0.0	0.0	0.0	0.0	0.0
0.0	0.0	0.0	0.0	0.0	0.0	0.0	0.0
0.0	0.0	0.0	0.0	0.0	0.0	0.0	0.0
0.0	0.0	0.0	0.0	0.0	0.0	0.0	0.0
0.0	0.0	0.0	0.0	0.0	0.0	0.0	0.0
0.0	0.0	0.0	0.0	0.0	0.0	0.0	0.0
0.0	0.0	0.0	0.0	0.0	0.0	0.0	0.0
0.0	0.0	0.0	0.0	0.0	0.0	0.0	0.0
0.0	0.0	0.0	0.0	0.0	0.0	0.0	0.0
0.0	0.0	0.0	0.0	0.0	0.0	0.0	0.0
0.0	0.0	0.0	0.0	0.0	0.0	0.0	0.0
0.0	0.0	0.0	0.0	0.0	0.0	0.0	0.0
0.0	0.0	0.0	0.0	0.0	0.0	0.0	0.0
0.0	0.0	0.0	0.0	0.0	0.0	0.0	0.0
0.0	0.0	0.0	0.0	0.0	0.0	0.0	0.0
0.0	0.0	0.0	0.0	0.0	0.0	0.0	0.0
0.0	0.0	0.0	0.0	0.0	0.0	0.0	0.0
0.0	0.0	0.0	0.0	0.0	0.0	0.0	0.0
0.0	0.0	0.0	0.0	0.0	0.0	0.0	0.0
0.0	0.0	0.0	0.0	0.0	0.0	0.0	0.0
0.0	0.0	0.0	0.0	0.0	0.0	0.0	0.0
0.0	0.0	0.0	0.0	0.0	0.0	0.0	0.0
0.0	0.0	0.0	0.0	0.0	0.0	0.0	0.0
0.0	0.0	0.0	0.0	0.0	0.0	0.0	0.0
0.0	0.0	0.0	0.0	0.0	0.0	0.0	0.0
0.0	0.0	0.0	0.0	0.0	0.0	0.0	0.0

0.0	0.0	0.0	0.0	0.0	0.0	0.0	0.0
0.0	0.0	0.0	0.0	0.0	0.0	0.0	0.0
0.0	0.0	0.0	0.0	0.0	0.0	0.0	0.0
0.0	0.0	0.0	0.0	0.0	0.0	0.0	0.0
0.0	0.0	0.0	0.0	0.0	0.0	0.0	0.0
0.0	0.0	0.0	0.0	0.0	0.0	0.0	0.0
0.0	0.0	0.0	0.0	0.0	0.0	0.0	0.0
0.0	0.0	0.0	0.0	0.0	0.0	0.0	0.0
0.0	0.0	0.0	0.0	0.0	0.0	0.0	0.0
0.0	0.0	0.0	0.0	0.0	0.0	0.0	0.0
0.0	0.0	0.0	0.0	0.0	0.0	0.0	0.0
0.0	0.0	0.0	0.0	0.0	0.0	0.0	0.0
0.0	0.0	0.0	0.0	0.0	0.0	0.0	0.0
0.0	0.0	0.0	0.0	0.0	0.0	0.0	0.0
0.0	0.0	0.0	0.0	0.0	0.0	0.0	0.0
0.0	0.0	0.0	0.0	0.0	0.0	0.0	0.0
0.0	0.0	0.0	0.0	0.0	0.0	0.0	0.0
0.0	0.0	0.0	0.0	0.0	0.0	0.0	0.0
0.0	0.0	0.0	0.0	0.0	0.0	0.0	0.0
0.0	0.0	0.0	0.0	0.0	0.0	0.0	0.0
0.0	0.0	0.0	0.0	0.0	0.0	0.0	0.0
0.0	0.0	0.0	0.0	0.0	0.0	0.0	0.0
0.0	0.0	0.0	0.0	0.0	0.0	0.0	0.0
0.0	0.0	0.0	0.0	0.0	0.0	0.0	0.0
0.0	0.0	0.0	0.0	0.0	0.0	0.0	0.0
0.0	0.0	0.0	0.0	0.0	0.0	0.0	0.0
0.0	0.0	0.0	0.0	0.0	0.0	0.0	0.0
0.0	0.0	0.0	0.0	0.0	0.0	0.0	0.0
0.0	0.0	0.0	0.0	0.0	0.0	0.0	0.0
0.0	0.0	0.0	0.0	0.0	0.0	0.0	0.0
0.0	0.0	0.0	0.0	0.0	0.0	0.0	0.0
0.0	0.0	0.0	0.0	0.0	0.0	0.0	0.0
0.0	0.0	0.0	0.0	0.0	0.0	0.0	0.0
0.0	0.0	0.0	0.0	0.0	0.0	0.0	0.0
0.0	0.0	0.0	0.0	0.0	0.0	0.0	0.0
0.0	0.0	0.0	0.0	0.0	0.0	0.0	0.0
0.0	0.0	0.0	0.0	0.0	0.0	0.0	0.0
0.0	0.0	0.0	0.0	0.0	0.0	0.0	0.0
0.0	0.0	0.0	0.0	0.0	0.0	0.0	0.0
0.0	0.0	0.0	0.0	0.0	0.0	0.0	0.0
0.0	0.0	0.0	0.0	0.0	0.0	0.0	0.0
0.0	0.0	0.0	0.0	0.0	0.0	0.0	0.0
0.0	0.0	0.0	0.0	0.0	0.0	0.0	0.0
0.0	0.0	0.0	0.0	0.0	0.0	0.0	0.0
0.0	0.0	0.0	0.0	0.0	0.0	0.0	0.0
0.0	0.0	0.0	0.0	0.0	0.0	0.0	0.0
0.0	0.0	0.0	0.0	0.0	0.0	0.0	0.0
0.0	0.0	0.0	0.0	0.0	0.0	0.0	0.0

0.0	0.0	0.0	0.0	0.0	0.0	0.0	0.0
0.0	0.0	0.0	0.0	0.0	0.0	0.0	0.0
0.0	0.0	0.0	0.0	0.0	0.0	0.0	0.0
0.0	0.0	0.0	0.0	0.0	0.0	0.0	0.0
0.0	0.0	0.0	0.0	0.0	0.0	0.0	0.0
0.0	0.0	0.0	0.0	0.0	0.0	0.0	0.0
0.0	0.0	0.0	0.0	0.0	0.0	0.0	0.0
0.0	0.0	0.0	0.0	0.0	0.0	0.0	0.0
0.0	0.0	0.0	0.0	0.0	0.0	0.0	0.0
0.0	0.0	0.0	0.0	0.0	0.0	0.0	0.0
0.0	0.0	0.0	0.0	0.0	0.0	0.0	0.0
0.0	0.0	0.0	0.0	0.0	0.0	0.0	0.0
0.0	0.0	0.0	0.0	0.0	0.0	0.0	0.0
0.0	0.0	0.0	0.0	0.0	0.0	0.0	0.0
0.0	0.0	0.0	0.0	0.0	0.0	0.0	0.0
0.0	0.0	0.0	0.0	0.0	0.0	0.0	0.0
0.0	0.0	0.0	0.0	0.0	0.0	0.0	0.0
0.0	0.0	0.0	0.0	0.0	0.0	0.0	0.0
0.0	0.0	0.0	0.0	0.0	0.0	0.0	0.0
0.0	0.0	0.0	0.0	0.0	0.0	0.0	0.0
0.0	0.0	0.0	0.0	0.0	0.0	0.0	0.0
0.0	0.0	0.0	0.0	0.0	0.0	0.0	0.0
0.0	0.0	0.0	0.0	0.0	0.0	0.0	0.0
0.0	0.0	0.0	0.0	0.0	0.0	0.0	0.0
0.0	0.0	0.0	0.0	0.0	0.0	0.0	0.0
0.0	0.0	0.0	0.0	0.0	0.0	0.0	0.0
0.0	0.0	0.0	0.0	0.0	0.0	0.0	0.0
0.0	0.0	0.0	0.0	0.0	0.0	0.0	0.0
0.0	0.0	0.0	0.0	0.0	0.0	0.0	0.0
0.0	0.0	0.0	0.0	0.0	0.0	0.0	0.0
0.0	0.0	0.0	0.0	0.0	0.0	0.0	0.0
0.0	0.0	0.0	0.0	0.0	0.0	0.0	0.0
0.0	0.0	0.0	0.0	0.0	0.0	0.0	0.0
0.0	0.0	0.0	0.0	0.0	0.0	0.0	0.0
0.0	0.0	0.0	0.0	0.0	0.0	0.0	0.0
0.0	0.0	0.0	0.0	0.0	0.0	0.0	0.0
0.0	0.0	0.0	0.0	0.0	0.0	0.0	0.0
0.0	0.0	0.0	0.0	0.0	0.0	0.0	0.0
0.0	0.0	0.0	0.0	0.0	0.0	0.0	0.0
0.0	0.0	0.0	0.0	0.0	0.0	0.0	0.0
0.0	0.0	0.0	0.0	0.0	0.0	0.0	0.0
0.0	0.0	0.0	0.0	0.0	0.0	0.0	0.0
0.0	0.0	0.0	0.0	0.0	0.0	0.0	0.0
0.0	0.0	0.0	0.0	0.0	0.0	0.0	0.0
0.0	0.0	0.0	0.0	0.0	0.0	0.0	0.0
0.0	0.0	0.0	0.0	0.0	0.0	0.0	0.0
0.0	0.0	0.0	0.0	0.0	0.0	0.0	0.0
0.0	0.0	0.0	0.0	0.0	0.0	0.0	0.0

0.0	0.0	0.0	0.0	0.0	0.0	0.0	0.0
0.0	0.0	0.0	0.0	0.0	0.0	0.0	0.0
0.0	0.0	0.0	0.0	0.0	0.0	0.0	0.0
0.0	0.0	0.0	0.0	0.0	0.0	0.0	0.0
0.0	0.0	0.0	0.0	0.0	0.0	0.0	0.0
0.0	0.0	0.0	0.0	0.0	0.0	0.0	0.0
0.0	0.0	0.0	0.0	0.0	0.0	0.0	0.0
0.0	0.0	0.0	0.0	0.0	0.0	0.0	0.0
0.0	0.0	0.0	0.0	0.0	0.0	0.0	0.0
0.0	0.0	0.0	0.0	0.0	0.0	0.0	0.0
0.0	0.0	0.0	0.0	0.0	0.0	0.0	0.0
0.0	0.0	0.0	0.0	0.0	0.0	0.0	0.0
0.0	0.0	0.0	0.0	0.0	0.0	0.0	0.0
0.0	0.0	0.0	0.0	0.0	0.0	0.0	0.0
0.0	0.0	0.0	0.0	0.0	0.0	0.0	0.0
0.0	0.0	0.0	0.0	0.0	0.0	0.0	0.0
0.0	0.0	0.0	0.0	0.0	0.0	0.0	0.0
0.0	0.0	0.0	0.0	0.0	0.0	0.0	0.0
0.0	0.0	0.0	0.0	0.0	0.0	0.0	0.0
0.0	0.0	0.0	0.0	0.0	0.0	0.0	0.0
0.0	0.0	0.0	0.0	0.0	0.0	0.0	0.0
0.0	0.0	0.0	0.0	0.0	0.0	0.0	0.0
0.0	0.0	0.0	0.0	0.0	0.0	0.0	0.0
0.0	0.0	0.0	0.0	0.0	0.0	0.0	0.0
0.0	0.0	0.0	0.0	0.0	0.0	0.0	0.0
0.0	0.0	0.0	0.0	0.0	0.0	0.0	0.0
0.0	0.0	0.0	0.0	0.0	0.0	0.0	0.0
0.0	0.0	0.0	0.0	0.0	0.0	0.0	0.0
0.0	0.0	0.0	0.0	0.0	0.0	0.0	0.0
0.0	0.0	0.0	0.0	0.0	0.0	0.0	0.0
0.0	0.0	0.0	0.0	0.0	0.0	0.0	0.0
0.0	0.0	0.0	0.0	0.0	0.0	0.0	0.0
0.0	0.0	0.0	0.0	0.0	0.0	0.0	0.0
0.0	0.0	0.0	0.0	0.0	0.0	0.0	0.0
0.0	0.0	0.0	0.0	0.0	0.0	0.0	0.0
0.0	0.0	0.0	0.0	0.0	0.0	0.0	0.0
0.0	0.0	0.0	0.0	0.0	0.0	0.0	0.0
0.0	0.0	0.0	0.0	0.0	0.0	0.0	0.0
0.0	0.0	0.0	0.0	0.0	0.0	0.0	0.0
0.0	0.0	0.0	0.0	0.0	0.0	0.0	0.0
0.0	0.0	0.0	0.0	0.0	0.0	0.0	0.0
0.0	0.0	0.0	0.0	0.0	0.0	0.0	0.0
0.0	0.0	0.0	0.0	0.0	0.0	0.0	0.0
0.0	0.0	0.0	0.0	0.0	0.0	0.0	0.0
0.0	0.0	0.0	0.0	0.0	0.0	0.0	0.0
0.0	0.0	0.0	0.0	0.0	0.0	0.0	0.0
0.0	0.0	0.0	0.0	0.0	0.0	0.0	0.0

0.0	0.0	0.0	0.0	0.0	0.0	0.0	0.0
0.0	0.0	0.0	0.0	0.0	0.0	0.0	0.0
0.0	0.0	0.0	0.0	0.0	0.0	0.0	0.0
0.0	0.0	0.0	0.0	0.0	0.0	0.0	0.0
0.0	0.0	0.0	0.0	0.0	0.0	0.0	0.0
0.0	0.0	0.0	0.0	0.0	0.0	0.0	0.0
0.0	0.0	0.0	0.0	0.0	0.0	0.0	0.0
0.0	0.0	0.0	0.0	0.0	0.0	0.0	0.0
0.0	0.0	0.0	0.0	0.0	0.0	0.0	0.0
0.0	0.0	0.0	0.0	0.0	0.0	0.0	0.0
0.0	0.0	0.0	0.0	0.0	0.0	0.0	0.0
0.0	0.0	0.0	0.0	0.0	0.0	0.0	0.0
0.0	0.0	0.0	0.0	0.0	0.0	0.0	0.0
0.0	0.0	0.0	0.0	0.0	0.0	0.0	0.0
0.0	0.0	0.0	0.0	0.0	0.0	0.0	0.0
0.0	0.0	0.0	0.0	0.0	0.0	0.0	0.0
0.0	0.0	0.0	0.0	0.0	0.0	0.0	0.0
0.0	0.0	0.0	0.0	0.0	0.0	0.0	0.0
0.0	0.0	0.0	0.0	0.0	0.0	0.0	0.0
0.0	0.0	0.0	0.0	0.0	0.0	0.0	0.0
0.0	0.0	0.0	0.0	0.0	0.0	0.0	0.0
0.0	0.0	0.0	0.0	0.0	0.0	0.0	0.0
0.0	0.0	0.0	0.0	0.0	0.0	0.0	0.0
0.0	0.0	0.0	0.0	0.0	0.0	0.0	0.0
0.0	0.0	0.0	0.0	0.0	0.0	0.0	0.0
0.0	0.0	0.0	0.0	0.0	0.0	0.0	0.0
0.0	0.0	0.0	0.0	0.0	0.0	0.0	0.0
0.0	0.0	0.0	0.0	0.0	0.0	0.0	0.0
0.0	0.0	0.0	0.0	0.0	0.0	0.0	0.0
0.0	0.0	0.0	0.0	0.0	0.0	0.0	0.0
0.0	0.0	0.0	0.0	0.0	0.0	0.0	0.0
0.0	0.0	0.0	0.0	0.0	0.0	0.0	0.0
0.0	0.0	0.0	0.0	0.0	0.0	0.0	0.0
0.0	0.0	0.0	0.0	0.0	0.0	0.0	0.0
0.0	0.0	0.0	0.0	0.0	0.0	0.0	0.0
0.0	0.0	0.0	0.0	0.0	0.0	0.0	0.0
0.0	0.0	0.0	0.0	0.0	0.0	0.0	0.0
0.0	0.0	0.0	0.0	0.0	0.0	0.0	0.0
0.0	0.0	0.0	0.0	0.0	0.0	0.0	0.0
0.0	0.0	0.0	0.0	0.0	0.0	0.0	0.0
0.0	0.0	0.0	0.0	0.0	0.0	0.0	0.0
0.0	0.0	0.0	0.0	0.0	0.0	0.0	0.0
0.0	0.0	0.0	0.0	0.0	0.0	0.0	0.0
0.0	0.0	0.0	0.0	0.0	0.0	0.0	0.0
0.0	0.0	0.0	0.0	0.0	0.0	0.0	0.0
0.0	0.0	0.0	0.0	0.0	0.0	0.0	0.0
0.0	0.0	0.0	0.0	0.0	0.0	0.0	0.0
0.0	0.0	0.0	0.0	0.0	0.0	0.0	0.0

0.0	0.0	0.0	0.0	0.0	0.0	0.0	0.0
0.0	0.0	0.0	0.0	0.0	0.0	0.0	0.0
0.0	0.0	0.0	0.0	0.0	0.0	0.0	0.0
0.0	0.0	0.0	0.0	0.0	0.0	0.0	0.0
0.0	0.0	0.0	0.0	0.0	0.0	0.0	0.0
0.0	0.0	0.0	0.0	0.0	0.0	0.0	0.0
0.0	0.0	0.0	0.0	0.0	0.0	0.0	0.0
0.0	0.0	0.0	0.0	0.0	0.0	0.0	0.0
0.0	0.0	0.0	0.0	0.0	0.0	0.0	0.0
0.0	0.0	0.0	0.0	0.0	0.0	0.0	0.0
0.0	0.0	0.0	0.0	0.0	0.0	0.0	0.0
0.0	0.0	0.0	0.0	0.0	0.0	0.0	0.0
0.0	0.0	0.0	0.0	0.0	0.0	0.0	0.0
0.0	0.0	0.0	0.0	0.0	0.0	0.0	0.0
0.0	0.0	0.0	0.0	0.0	0.0	0.0	0.0
0.0	0.0	0.0	0.0	0.0	0.0	0.0	0.0
0.0	0.0	0.0	0.0	0.0	0.0	0.0	0.0
0.0	0.0	0.0	0.0	0.0	0.0	0.0	0.0
0.0	0.0	0.0	0.0	0.0	0.0	0.0	0.0
0.0	0.0	0.0	0.0	0.0	0.0	0.0	0.0
0.0	0.0	0.0	0.0	0.0	0.0	0.0	0.0
0.0	0.0	0.0	0.0	0.0	0.0	0.0	0.0
0.0	0.0	0.0	0.0	0.0	0.0	0.0	0.0
0.0	0.0	0.0	0.0	0.0	0.0	0.0	0.0
0.0	0.0	0.0	0.0	0.0	0.0	0.0	0.0
0.0	0.0	0.0	0.0	0.0	0.0	0.0	0.0
0.0	0.0	0.0	0.0	0.0	0.0	0.0	0.0
0.0	0.0	0.0	0.0	0.0	0.0	0.0	0.0
0.0	0.0	0.0	0.0	0.0	0.0	0.0	0.0
0.0	0.0	0.0	0.0	0.0	0.0	0.0	0.0
0.0	0.0	0.0	0.0	0.0	0.0	0.0	0.0
0.0	0.0	0.0	0.0	0.0	0.0	0.0	0.0
0.0	0.0	0.0	0.0	0.0	0.0	0.0	0.0
0.0	0.0	0.0	0.0	0.0	0.0	0.0	0.0
0.0	0.0	0.0	0.0	0.0	0.0	0.0	0.0
0.0	0.0	0.0	0.0	0.0	0.0	0.0	0.0
0.0	0.0	0.0	0.0	0.0	0.0	0.0	0.0
0.0	0.0	0.0	0.0	0.0	0.0	0.0	0.0
0.0	0.0	0.0	0.0	0.0	0.0	0.0	0.0
0.0	0.0	0.0	0.0	0.0	0.0	0.0	0.0
0.0	0.0	0.0	0.0	0.0	0.0	0.0	0.0
0.0	0.0	0.0	0.0	0.0	0.0	0.0	0.0
0.0	0.0	0.0	0.0	0.0	0.0	0.0	0.0
0.0	0.0	0.0	0.0	0.0	0.0	0.0	0.0
0.0	0.0	0.0	0.0	0.0	0.0	0.0	0.0
0.0	0.0	0.0	0.0	0.0	0.0	0.0	0.0
0.0	0.0	0.0	0.0	0.0	0.0	0.0	0.0

0.0	0.0	0.0	0.0	0.0	0.0	0.0	0.0
0.0	0.0	0.0	0.0	0.0	0.0	0.0	0.0
0.0	0.0	0.0	0.0	0.0	0.0	0.0	0.0
0.0	0.0	0.0	0.0	0.0	0.0	0.0	0.0
0.0	0.0	0.0	0.0	0.0	0.0	0.0	0.0
0.0	0.0	0.0	0.0	0.0	0.0	0.0	0.0
0.0	0.0	0.0	0.0	0.0	0.0	0.0	0.0
0.0	0.0	0.0	0.0	0.0	0.0	0.0	0.0
0.0	0.0	0.0	0.0	0.0	0.0	0.0	0.0
0.0	0.0	0.0	0.0	0.0	0.0	0.0	0.0
0.0	0.0	0.0	0.0	0.0	0.0	0.0	0.0
0.0	0.0	0.0	0.0	0.0	0.0	0.0	0.0
0.0	0.0	0.0	0.0	0.0	0.0	0.0	0.0
0.0	0.0	0.0	0.0	0.0	0.0	0.0	0.0
0.0	0.0	0.0	0.0	0.0	0.0	0.0	0.0
0.0	0.0	0.0	0.0	0.0	0.0	0.0	0.0
0.0	0.0	0.0	0.0	0.0	0.0	0.0	0.0
0.0	0.0	0.0	0.0	0.0	0.0	0.0	0.0
0.0	0.0	0.0	0.0	0.0	0.0	0.0	0.0
0.0	0.0	0.0	0.0	0.0	0.0	0.0	0.0
0.0	0.0	0.0	0.0	0.0	0.0	0.0	0.0
0.0	0.0	0.0	0.0	0.0	0.0	0.0	0.0
0.0	0.0	0.0	0.0	0.0	0.0	0.0	0.0
0.0	0.0	0.0	0.0	0.0	0.0	0.0	0.0
0.0	0.0	0.0	0.0	0.0	0.0	0.0	0.0
0.0	0.0	0.0	0.0	0.0	0.0	0.0	0.0
0.0	0.0	0.0	0.0	0.0	0.0	0.0	0.0
0.0	0.0	0.0	0.0	0.0	0.0	0.0	0.0
0.0	0.0	0.0	0.0	0.0	0.0	0.0	0.0
0.0	0.0	0.0	0.0	0.0	0.0	0.0	0.0
0.0	0.0	0.0	0.0	0.0	0.0	0.0	0.0
0.0	0.0	0.0	0.0	0.0	0.0	0.0	0.0
0.0	0.0	0.0	0.0	0.0	0.0	0.0	0.0
0.0	0.0	0.0	0.0	0.0	0.0	0.0	0.0
0.0	0.0	0.0	0.0	0.0	0.0	0.0	0.0
0.0	0.0	0.0	0.0	0.0	0.0	0.0	0.0
0.0	0.0	0.0	0.0	0.0	0.0	0.0	0.0
0.0	0.0	0.0	0.0	0.0	0.0	0.0	0.0
0.0	0.0	0.0	0.0	0.0	0.0	0.0	0.0
0.0	0.0	0.0	0.0	0.0	0.0	0.0	0.0
0.0	0.0	0.0	0.0	0.0	0.0	0.0	0.0
0.0	0.0	0.0	0.0	0.0	0.0	0.0	0.0
0.0	0.0	0.0	0.0	0.0	0.0	0.0	0.0
0.0	0.0	0.0	0.0	0.0	0.0	0.0	0.0
0.0	0.0	0.0	0.0	0.0	0.0	0.0	0.0
0.0	0.0	0.0	0.0	0.0	0.0	0.0	0.0
0.0	0.0	0.0	0.0	0.0	0.0	0.0	0.0
0.0	0.0	0.0	0.0	0.0	0.0	0.0	0.0

0.0	0.0	0.0	0.0	0.0	0.0	0.0	0.0
0.0	0.0	0.0	0.0	0.0	0.0	0.0	0.0
0.0	0.0	0.0	0.0	0.0	0.0	0.0	0.0
0.0	0.0	0.0	0.0	0.0	0.0	0.0	0.0
0.0	0.0	0.0	0.0	0.0	0.0	0.0	0.0
0.0	0.0	0.0	0.0	0.0	0.0	0.0	0.0
0.0	0.0	0.0	0.0	0.0	0.0	0.0	0.0
0.0	0.0	0.0	0.0	0.0	0.0	0.0	0.0
0.0	0.0	0.0	0.0	0.0	0.0	0.0	0.0
0.0	0.0	0.0	0.0	0.0	0.0	0.0	0.0
0.0	0.0	0.0	0.0	0.0	0.0	0.0	0.0
0.0	0.0	0.0	0.0	0.0	0.0	0.0	0.0
0.0	0.0	0.0	0.0	0.0	0.0	0.0	0.0
0.0	0.0	0.0	0.0	0.0	0.0	0.0	0.0
0.0	0.0	0.0	0.0	0.0	0.0	0.0	0.0
0.0	0.0	0.0	0.0	0.0	0.0	0.0	0.0
0.0	0.0	0.0	0.0	0.0	0.0	0.0	0.0
0.0	0.0	0.0	0.0	0.0	0.0	0.0	0.0
0.0	0.0	0.0	0.0	0.0	0.0	0.0	0.0
0.0	0.0	0.0	0.0	0.0	0.0	0.0	0.0
0.0	0.0	0.0	0.0	0.0	0.0	0.0	0.0
0.0	0.0	0.0	0.0	0.0	0.0	0.0	0.0
0.0	0.0	0.0	0.0	0.0	0.0	0.0	0.0
0.0	0.0	0.0	0.0	0.0	0.0	0.0	0.0
0.0	0.0	0.0	0.0	0.0	0.0	0.0	0.0
0.0	0.0	0.0	0.0	0.0	0.0	0.0	0.0
0.0	0.0	0.0	0.0	0.0	0.0	0.0	0.0
0.0	0.0	0.0	0.0	0.0	0.0	0.0	0.0
0.0	0.0	0.0	0.0	0.0	0.0	0.0	0.0
0.0	0.0	0.0	0.0	0.0	0.0	0.0	0.0
0.0	0.0	0.0	0.0	0.0	0.0	0.0	0.0
0.0	0.0	0.0	0.0	0.0	0.0	0.0	0.0
0.0	0.0	0.0	0.0	0.0	0.0	0.0	0.0
0.0	0.0	0.0	0.0	0.0	0.0	0.0	0.0
0.0	0.0	0.0	0.0	0.0	0.0	0.0	0.0
0.0	0.0	0.0	0.0	0.0	0.0	0.0	0.0
0.0	0.0	0.0	0.0	0.0	0.0	0.0	0.0
0.0	0.0	0.0	0.0	0.0	0.0	0.0	0.0
0.0	0.0	0.0	0.0	0.0	0.0	0.0	0.0
0.0	0.0	0.0	0.0	0.0	0.0	0.0	0.0
0.0	0.0	0.0	0.0	0.0	0.0	0.0	0.0
0.0	0.0	0.0	0.0	0.0	0.0	0.0	0.0
0.0	0.0	0.0	0.0	0.0	0.0	0.0	0.0
0.0	0.0	0.0	0.0	0.0	0.0	0.0	0.0
0.0	0.0	0.0	0.0	0.0	0.0	0.0	0.0
0.0	0.0	0.0	0.0	0.0	0.0	0.0	0.0
0.0	0.0	0.0	0.0	0.0	0.0	0.0	0.0

0.0	0.0	0.0	0.0	0.0	0.0	0.0	0.0
0.0	0.0	0.0	0.0	0.0	0.0	0.0	0.0
0.0	0.0	0.0	0.0	0.0	0.0	0.0	0.0
0.0	0.0	0.0	0.0	0.0	0.0	0.0	0.0
0.0	0.0	0.0	0.0	0.0	0.0	0.0	0.0
0.0	0.0	0.0	0.0	0.0	0.0	0.0	0.0
0.0	0.0	0.0	0.0	0.0	0.0	0.0	0.0
0.0	0.0	0.0	0.0	0.0	0.0	0.0	0.0
0.0	0.0	0.0	0.0	0.0	0.0	0.0	0.0
0.0	0.0	0.0	0.0	0.0	0.0	0.0	0.0
0.0	0.0	0.0	0.0	0.0	0.0	0.0	0.0
0.0	0.0	0.0	0.0	0.0	0.0	0.0	0.0
0.0	0.0	0.0	0.0	0.0	0.0	0.0	0.0
0.0	0.0	0.0	0.0	0.0	0.0	0.0	0.0
0.0	0.0	0.0	0.0	0.0	0.0	0.0	0.0
0.0	0.0	0.0	0.0	0.0	0.0	0.0	0.0
0.0	0.0	0.0	0.0	0.0	0.0	0.0	0.0
0.0	0.0	0.0	0.0	0.0	0.0	0.0	0.0
0.0	0.0	0.0	0.0	0.0	0.0	0.0	0.0
0.0	0.0	0.0	0.0	0.0	0.0	0.0	0.0
0.0	0.0	0.0	0.0	0.0	0.0	0.0	0.0
0.0	0.0	0.0	0.0	0.0	0.0	0.0	0.0
0.0	0.0	0.0	0.0	0.0	0.0	0.0	0.0
0.0	0.0	0.0	0.0	0.0	0.0	0.0	0.0
0.0	0.0	0.0	0.0	0.0	0.0	0.0	0.0
0.0	0.0	0.0	0.0	0.0	0.0	0.0	0.0
0.0	0.0	0.0	0.0	0.0	0.0	0.0	0.0
0.0	0.0	0.0	0.0	0.0	0.0	0.0	0.0
0.0	0.0	0.0	0.0	0.0	0.0	0.0	0.0
0.0	0.0	0.0	0.0	0.0	0.0	0.0	0.0
0.0	0.0	0.0	0.0	0.0	0.0	0.0	0.0
0.0	0.0	0.0	0.0	0.0	0.0	0.0	0.0
0.0	0.0	0.0	0.0	0.0	0.0	0.0	0.0
0.0	0.0	0.0	0.0	0.0	0.0	0.0	0.0
0.0	0.0	0.0	0.0	0.0	0.0	0.0	0.0
0.0	0.0	0.0	0.0	0.0	0.0	0.0	0.0
0.0	0.0	0.0	0.0	0.0	0.0	0.0	0.0
0.0	0.0	0.0	0.0	0.0	0.0	0.0	0.0
0.0	0.0	0.0	0.0	0.0	0.0	0.0	0.0
0.0	0.0	0.0	0.0	0.0	0.0	0.0	0.0
0.0	0.0	0.0	0.0	0.0	0.0	0.0	0.0
0.0	0.0	0.0	0.0	0.0	0.0	0.0	0.0
0.0	0.0	0.0	0.0	0.0	0.0	0.0	0.0
0.0	0.0	0.0	0.0	0.0	0.0	0.0	0.0
0.0	0.0	0.0	0.0	0.0	0.0	0.0	0.0
0.0	0.0	0.0	0.0	0.0	0.0	0.0	0.0
0.0	0.0	0.0	0.0	0.0	0.0	0.0	0.0

0.0	0.0	0.0	0.0	0.0	0.0	0.0	0.0
0.0	0.0	0.0	0.0	0.0	0.0	0.0	0.0
0.0	0.0	0.0	0.0	0.0	0.0	0.0	0.0
0.0	0.0	0.0	0.0	0.0	0.0	0.0	0.0
0.0	0.0	0.0	0.0	0.0	0.0	0.0	0.0
0.0	0.0	0.0	0.0	0.0	0.0	0.0	0.0
0.0	0.0	0.0	0.0	0.0	0.0	0.0	0.0
0.0	0.0	0.0	0.0	0.0	0.0	0.0	0.0
0.0	0.0	0.0	0.0	0.0	0.0	0.0	0.0
0.0	0.0	0.0	0.0	0.0	0.0	0.0	0.0
0.0	0.0	0.0	0.0	0.0	0.0	0.0	0.0
0.0	0.0	0.0	0.0	0.0	0.0	0.0	0.0
0.0	0.0	0.0	0.0	0.0	0.0	0.0	0.0
0.0	0.0	0.0	0.0	0.0	0.0	0.0	0.0
0.0	0.0	0.0	0.0	0.0	0.0	0.0	0.0
0.0	0.0	0.0	0.0	0.0	0.0	0.0	0.0
0.0	0.0	0.0	0.0	0.0	0.0	0.0	0.0
0.0	0.0	0.0	0.0	0.0	0.0	0.0	0.0
0.0	0.0	0.0	0.0	0.0	0.0	0.0	0.0
0.0	0.0	0.0	0.0	0.0	0.0	0.0	0.0
0.0	0.0	0.0	0.0	0.0	0.0	0.0	0.0
0.0	0.0	0.0	0.0	0.0	0.0	0.0	0.0
0.0	0.0	0.0	0.0	0.0	0.0	0.0	0.0
0.0	0.0	0.0	0.0	0.0	0.0	0.0	0.0
0.0	0.0	0.0	0.0	0.0	0.0	0.0	0.0
0.0	0.0	0.0	0.0	0.0	0.0	0.0	0.0
0.0	0.0	0.0	0.0	0.0	0.0	0.0	0.0
0.0	0.0	0.0	0.0	0.0	0.0	0.0	0.0
0.0	0.0	0.0	0.0	0.0	0.0	0.0	0.0
0.0	0.0	0.0	0.0	0.0	0.0	0.0	0.0
0.0	0.0	0.0	0.0	0.0	0.0	0.0	0.0
0.0	0.0	0.0	0.0	0.0	0.0	0.0	0.0
0.0	0.0	0.0	0.0	0.0	0.0	0.0	0.0
0.0	0.0	0.0	0.0	0.0	0.0	0.0	0.0
0.0	0.0	0.0	0.0	0.0	0.0	0.0	0.0
0.0	0.0	0.0	0.0	0.0	0.0	0.0	0.0
0.0	0.0	0.0	0.0	0.0	0.0	0.0	0.0
0.0	0.0	0.0	0.0	0.0	0.0	0.0	0.0
0.0	0.0	0.0	0.0	0.0	0.0	0.0	0.0
0.0	0.0	0.0	0.0	0.0	0.0	0.0	0.0
0.0	0.0	0.0	0.0	0.0	0.0	0.0	0.0
0.0	0.0	0.0	0.0	0.0	0.0	0.0	0.0
0.0	0.0	0.0	0.0	0.0	0.0	0.0	0.0
0.0	0.0	0.0	0.0	0.0	0.0	0.0	0.0
0.0	0.0	0.0	0.0	0.0	0.0	0.0	0.0
0.0	0.0	0.0	0.0	0.0	0.0	0.0	0.0
0.0	0.0	0.0	0.0	0.0	0.0	0.0	0.0
0.0	0.0	0.0	0.0	0.0	0.0	0.0	0.0

0.0	0.0	0.0	0.0	0.0	0.0	0.0	0.0
0.0	0.0	0.0	0.0	0.0	0.0	0.0	0.0
0.0	0.0	0.0	0.0	0.0	0.0	0.0	0.0
0.0	0.0	0.0	0.0	0.0	0.0	0.0	0.0
0.0	0.0	0.0	0.0	0.0	0.0	0.0	0.0
0.0	0.0	0.0	0.0	0.0	0.0	0.0	0.0
0.0	0.0	0.0	0.0	0.0	0.0	0.0	0.0
0.0	0.0	0.0	0.0	0.0	0.0	0.0	0.0
0.0	0.0	0.0	0.0	0.0	0.0	0.0	0.0
0.0	0.0	0.0	0.0	0.0	0.0	0.0	0.0
0.0	0.0	0.0	0.0	0.0	0.0	0.0	0.0
0.0	0.0	0.0	0.0	0.0	0.0	0.0	0.0
0.0	0.0	0.0	0.0	0.0	0.0	0.0	0.0
0.0	0.0	0.0	0.0	0.0	0.0	0.0	0.0
0.0	0.0	0.0	0.0	0.0	0.0	0.0	0.0
0.0	0.0	0.0	0.0	0.0	0.0	0.0	0.0
0.0	0.0	0.0	0.0	0.0	0.0	0.0	0.0
0.0	0.0	0.0	0.0	0.0	0.0	0.0	0.0
0.0	0.0	0.0	0.0	0.0	0.0	0.0	0.0
0.0	0.0	0.0	0.0	0.0	0.0	0.0	0.0
0.0	0.0	0.0	0.0	0.0	0.0	0.0	0.0
0.0	0.0	0.0	0.0	0.0	0.0	0.0	0.0
0.0	0.0	0.0	0.0	0.0	0.0	0.0	0.0
0.0	0.0	0.0	0.0	0.0	0.0	0.0	0.0
0.0	0.0	0.0	0.0	0.0	0.0	0.0	0.0
0.0	0.0	0.0	0.0	0.0	0.0	0.0	0.0
0.0	0.0	0.0	0.0	0.0	0.0	0.0	0.0
0.0	0.0	0.0	0.0	0.0	0.0	0.0	0.0
0.0	0.0	0.0	0.0	0.0	0.0	0.0	0.0
0.0	0.0	0.0	0.0	0.0	0.0	0.0	0.0
0.0	0.0	0.0	0.0	0.0	0.0	0.0	0.0
0.0	0.0	0.0	0.0	0.0	0.0	0.0	0.0
0.0	0.0	0.0	0.0	0.0	0.0	0.0	0.0
0.0	0.0	0.0	0.0	0.0	0.0	0.0	0.0
0.0	0.0	0.0	0.0	0.0	0.0	0.0	0.0
0.0	0.0	0.0	0.0	0.0	0.0	0.0	0.0
0.0	0.0	0.0	0.0	0.0	0.0	0.0	0.0
0.0	0.0	0.0	0.0	0.0	0.0	0.0	0.0
0.0	0.0	0.0	0.0	0.0	0.0	0.0	0.0
0.0	0.0	0.0	0.0	0.0	0.0	0.0	0.0
0.0	0.0	0.0	0.0	0.0	0.0	0.0	0.0
0.0	0.0	0.0	0.0	0.0	0.0	0.0	0.0
0.0	0.0	0.0	0.0	0.0	0.0	0.0	0.0
0.0	0.0	0.0	0.0	0.0	0.0	0.0	0.0
0.0	0.0	0.0	0.0	0.0	0.0	0.0	0.0
0.0	0.0	0.0	0.0	0.0	0.0	0.0	0.0
0.0	0.0	0.0	0.0	0.0	0.0	0.0	0.0

0.0	0.0	0.0	0.0	0.0	0.0	0.0	0.0
0.0	0.0	0.0	0.0	0.0	0.0	0.0	0.0
0.0	0.0	0.0	0.0	0.0	0.0	0.0	0.0
0.0	0.0	0.0	0.0	0.0	0.0	0.0	0.0
0.0	0.0	0.0	0.0	0.0	0.0	0.0	0.0
0.0	0.0	0.0	0.0	0.0	0.0	0.0	0.0
0.0	0.0	0.0	0.0	0.0	0.0	0.0	0.0
0.0	0.0	0.0	0.0	0.0	0.0	0.0	0.0
0.0	0.0	0.0	0.0	0.0	0.0	0.0	0.0
0.0	0.0	0.0	0.0	0.0	0.0	0.0	0.0
0.0	0.0	0.0	0.0	0.0	0.0	0.0	0.0
0.0	0.0	0.0	0.0	0.0	0.0	0.0	0.0
0.0	0.0	0.0	0.0	0.0	0.0	0.0	0.0
0.0	0.0	0.0	0.0	0.0	0.0	0.0	0.0
0.0	0.0	0.0	0.0	0.0	0.0	0.0	0.0
0.0	0.0	0.0	0.0	0.0	0.0	0.0	0.0
0.0	0.0	0.0	0.0	0.0	0.0	0.0	0.0
0.0	0.0	0.0	0.0	0.0	0.0	0.0	0.0
0.0	0.0	0.0	0.0	0.0	0.0	0.0	0.0
0.0	0.0	0.0	0.0	0.0	0.0	0.0	0.0
0.0	0.0	0.0	0.0	0.0	0.0	0.0	0.0
0.0	0.0	0.0	0.0	0.0	0.0	0.0	0.0
0.0	0.0	0.0	0.0	0.0	0.0	0.0	0.0
0.0	0.0	0.0	0.0	0.0	0.0	0.0	0.0
0.0	0.0	0.0	0.0	0.0	0.0	0.0	0.0
0.0	0.0	0.0	0.0	0.0	0.0	0.0	0.0
0.0	0.0	0.0	0.0	0.0	0.0	0.0	0.0
0.0	0.0	0.0	0.0	0.0	0.0	0.0	0.0
0.0	0.0	0.0	0.0	0.0	0.0	0.0	0.0
0.0	0.0	0.0	0.0	0.0	0.0	0.0	0.0
0.0	0.0	0.0	0.0	0.0	0.0	0.0	0.0
0.0	0.0	0.0	0.0	0.0	0.0	0.0	0.0
0.0	0.0	0.0	0.0	0.0	0.0	0.0	0.0
0.0	0.0	0.0	0.0	0.0	0.0	0.0	0.0
0.0	0.0	0.0	0.0	0.0	0.0	0.0	0.0
0.0	0.0	0.0	0.0	0.0	0.0	0.0	0.0
0.0	0.0	0.0	0.0	0.0	0.0	0.0	0.0
0.0	0.0	0.0	0.0	0.0	0.0	0.0	0.0
0.0	0.0	0.0	0.0	0.0	0.0	0.0	0.0
0.0	0.0	0.0	0.0	0.0	0.0	0.0	0.0
0.0	0.0	0.0	0.0	0.0	0.0	0.0	0.0
0.0	0.0	0.0	0.0	0.0	0.0	0.0	0.0
0.0	0.0	0.0	0.0	0.0	0.0	0.0	0.0
0.0	0.0	0.0	0.0	0.0	0.0	0.0	0.0
0.0	0.0	0.0	0.0	0.0	0.0	0.0	0.0
0.0	0.0	0.0	0.0	0.0	0.0	0.0	0.0
0.0	0.0	0.0	0.0	0.0	0.0	0.0	0.0

0.0	0.0	0.0	0.0	0.0	0.0	0.0	0.0
0.0	0.0	0.0	0.0	0.0	0.0	0.0	0.0
0.0	0.0	0.0	0.0	0.0	0.0	0.0	0.0
0.0	0.0	0.0	0.0	0.0	0.0	0.0	0.0
0.0	0.0	0.0	0.0	0.0	0.0	0.0	0.0
0.0	0.0	0.0	0.0	0.0	0.0	0.0	0.0
0.0	0.0	0.0	0.0	0.0	0.0	0.0	0.0
0.0	0.0	0.0	0.0	0.0	0.0	0.0	0.0
0.0	0.0	0.0	0.0	0.0	0.0	0.0	0.0
0.0	0.0	0.0	0.0	0.0	0.0	0.0	0.0
0.0	0.0	0.0	0.0	0.0	0.0	0.0	0.0
0.0	0.0	0.0	0.0	0.0	0.0	0.0	0.0
0.0	0.0	0.0	0.0	0.0	0.0	0.0	0.0
0.0	0.0	0.0	0.0	0.0	0.0	0.0	0.0
0.0	0.0	0.0	0.0	0.0	0.0	0.0	0.0
0.0	0.0	0.0	0.0	0.0	0.0	0.0	0.0
0.0	0.0	0.0	0.0	0.0	0.0	0.0	0.0
0.0	0.0	0.0	0.0	0.0	0.0	0.0	0.0
0.0	0.0	0.0	0.0	0.0	0.0	0.0	0.0
0.0	0.0	0.0	0.0	0.0	0.0	0.0	0.0
0.0	0.0	0.0	0.0	0.0	0.0	0.0	0.0
0.0	0.0	0.0	0.0	0.0	0.0	0.0	0.0
0.0	0.0	0.0	0.0	0.0	0.0	0.0	0.0
0.0	0.0	0.0	0.0	0.0	0.0	0.0	0.0
0.0	0.0	0.0	0.0	0.0	0.0	0.0	0.0
0.0	0.0	0.0	0.0	0.0	0.0	0.0	0.0
0.0	0.0	0.0	0.0	0.0	0.0	0.0	0.0
0.0	0.0	0.0	0.0	0.0	0.0	0.0	0.0
0.0	0.0	0.0	0.0	0.0	0.0	0.0	0.0
0.0	0.0	0.0	0.0	0.0	0.0	0.0	0.0
0.0	0.0	0.0	0.0	0.0	0.0	0.0	0.0
0.0	0.0	0.0	0.0	0.0	0.0	0.0	0.0
0.0	0.0	0.0	0.0	0.0	0.0	0.0	0.0
0.0	0.0	0.0	0.0	0.0	0.0	0.0	0.0
0.0	0.0	0.0	0.0	0.0	0.0	0.0	0.0
0.0	0.0	0.0	0.0	0.0	0.0	0.0	0.0
0.0	0.0	0.0	0.0	0.0	0.0	0.0	0.0
0.0	0.0	0.0	0.0	0.0	0.0	0.0	0.0
0.0	0.0	0.0	0.0	0.0	0.0	0.0	0.0
0.0	0.0	0.0	0.0	0.0	0.0	0.0	0.0
0.0	0.0	0.0	0.0	0.0	0.0	0.0	0.0
0.0	0.0	0.0	0.0	0.0	0.0	0.0	0.0
0.0	0.0	0.0	0.0	0.0	0.0	0.0	0.0
0.0	0.0	0.0	0.0	0.0	0.0	0.0	0.0
0.0	0.0	0.0	0.0	0.0	0.0	0.0	0.0
0.0	0.0	0.0	0.0	0.0	0.0	0.0	0.0
0.0	0.0	0.0	0.0	0.0	0.0	0.0	0.0

0.0	0.0	0.0	0.0	0.0	0.0	0.0	0.0
0.0	0.0	0.0	0.0	0.0	0.0	0.0	0.0
0.0	0.0	0.0	0.0	0.0	0.0	0.0	0.0
0.0	0.0	0.0	0.0	0.0	0.0	0.0	0.0
0.0	0.0	0.0	0.0	0.0	0.0	0.0	0.0
0.0	0.0	0.0	0.0	0.0	0.0	0.0	0.0
0.0	0.0	0.0	0.0	0.0	0.0	0.0	0.0
0.0	0.0	0.0	0.0	0.0	0.0	0.0	0.0
0.0	0.0	0.0	0.0	0.0	0.0	0.0	0.0
0.0	0.0	0.0	0.0	0.0	0.0	0.0	0.0
0.0	0.0	0.0	0.0	0.0	0.0	0.0	0.0
0.0	0.0	0.0	0.0	0.0	0.0	0.0	0.0
0.0	0.0	0.0	0.0	0.0	0.0	0.0	0.0
0.0	0.0	0.0	0.0	0.0	0.0	0.0	0.0
0.0	0.0	0.0	0.0	0.0	0.0	0.0	0.0
0.0	0.0	0.0	0.0	0.0	0.0	0.0	0.0
0.0	0.0	0.0	0.0	0.0	0.0	0.0	0.0
0.0	0.0	0.0	0.0	0.0	0.0	0.0	0.0
0.0	0.0	0.0	0.0	0.0	0.0	0.0	0.0
0.0	0.0	0.0	0.0	0.0	0.0	0.0	0.0
0.0	0.0	0.0	0.0	0.0	0.0	0.0	0.0
0.0	0.0	0.0	0.0	0.0	0.0	0.0	0.0
0.0	0.0	0.0	0.0	0.0	0.0	0.0	0.0
0.0	0.0	0.0	0.0	0.0	0.0	0.0	0.0
0.0	0.0	0.0	0.0	0.0	0.0	0.0	0.0
0.0	0.0	0.0	0.0	0.0	0.0	0.0	0.0
0.0	0.0	0.0	0.0	0.0	0.0	0.0	0.0
0.0	0.0	0.0	0.0	0.0	0.0	0.0	0.0
0.0	0.0	0.0	0.0	0.0	0.0	0.0	0.0
0.0	0.0	0.0	0.0	0.0	0.0	0.0	0.0
0.0	0.0	0.0	0.0	0.0	0.0	0.0	0.0
0.0	0.0	0.0	0.0	0.0	0.0	0.0	0.0
0.0	0.0	0.0	0.0	0.0	0.0	0.0	0.0
0.0	0.0	0.0	0.0	0.0	0.0	0.0	0.0
0.0	0.0	0.0	0.0	0.0	0.0	0.0	0.0
0.0	0.0	0.0	0.0	0.0	0.0	0.0	0.0
0.0	0.0	0.0	0.0	0.0	0.0	0.0	0.0
0.0	0.0	0.0	0.0	0.0	0.0	0.0	0.0
0.0	0.0	0.0	0.0	0.0	0.0	0.0	0.0
0.0	0.0	0.0	0.0	0.0	0.0	0.0	0.0
0.0	0.0	0.0	0.0	0.0	0.0	0.0	0.0
0.0	0.0	0.0	0.0	0.0	0.0	0.0	0.0
0.0	0.0	0.0	0.0	0.0	0.0	0.0	0.0
0.0	0.0	0.0	0.0	0.0	0.0	0.0	0.0
0.0	0.0	0.0	0.0	0.0	0.0	0.0	0.0
0.0	0.0	0.0	0.0	0.0	0.0	0.0	0.0
0.0	0.0	0.0	0.0	0.0	0.0	0.0	0.0

0.0	0.0	0.0	0.0	0.0	0.0	0.0	0.0
0.0	0.0	0.0	0.0	0.0	0.0	0.0	0.0
0.0	0.0	0.0	0.0	0.0	0.0	0.0	0.0
0.0	0.0	0.0	0.0	0.0	0.0	0.0	0.0
0.0	0.0	0.0	0.0	0.0	0.0	0.0	0.0
0.0	0.0	0.0	0.0	0.0	0.0	0.0	0.0
0.0	0.0	0.0	0.0	0.0	0.0	0.0	0.0
0.0	0.0	0.0	0.0	0.0	0.0	0.0	0.0
0.0	0.0	0.0	0.0	0.0	0.0	0.0	0.0
0.0	0.0	0.0	0.0	0.0	0.0	0.0	0.0
0.0	0.0	0.0	0.0	0.0	0.0	0.0	0.0
0.0	0.0	0.0	0.0	0.0	0.0	0.0	0.0
0.0	0.0	0.0	0.0	0.0	0.0	0.0	0.0
0.0	0.0	0.0	0.0	0.0	0.0	0.0	0.0
0.0	0.0	0.0	0.0	0.0	0.0	0.0	0.0
0.0	0.0	0.0	0.0	0.0	0.0	0.0	0.0
0.0	0.0	0.0	0.0	0.0	0.0	0.0	0.0
0.0	0.0	0.0	0.0	0.0	0.0	0.0	0.0
0.0	0.0	0.0	0.0	0.0	0.0	0.0	0.0
0.0	0.0	0.0	0.0	0.0	0.0	0.0	0.0
0.0	0.0	0.0	0.0	0.0	0.0	0.0	0.0
0.0	0.0	0.0	0.0	0.0	0.0	0.0	0.0
0.0	0.0	0.0	0.0	0.0	0.0	0.0	0.0
0.0	0.0	0.0	0.0	0.0	0.0	0.0	0.0
0.0	0.0	0.0	0.0	0.0	0.0	0.0	0.0
0.0	0.0	0.0	0.0	0.0	0.0	0.0	0.0
0.0	0.0	0.0	0.0	0.0	0.0	0.0	0.0
0.0	0.0	0.0	0.0	0.0	0.0	0.0	0.0
0.0	0.0	0.0	0.0	0.0	0.0	0.0	0.0
0.0	0.0	0.0	0.0	0.0	0.0	0.0	0.0
0.0	0.0	0.0	0.0	0.0	0.0	0.0	0.0
0.0	0.0	0.0	0.0	0.0	0.0	0.0	0.0
0.0	0.0	0.0	0.0	0.0	0.0	0.0	0.0
0.0	0.0	0.0	0.0	0.0	0.0	0.0	0.0
0.0	0.0	0.0	0.0	0.0	0.0	0.0	0.0
0.0	0.0	0.0	0.0	0.0	0.0	0.0	0.0
0.0	0.0	0.0	0.0	0.0	0.0	0.0	0.0
0.0	0.0	0.0	0.0	0.0	0.0	0.0	0.0
0.0	0.0	0.0	0.0	0.0	0.0	0.0	0.0
0.0	0.0	0.0	0.0	0.0	0.0	0.0	0.0
0.0	0.0	0.0	0.0	0.0	0.0	0.0	0.0
0.0	0.0	0.0	0.0	0.0	0.0	0.0	0.0
0.0	0.0	0.0	0.0	0.0	0.0	0.0	0.0
0.0	0.0	0.0	0.0	0.0	0.0	0.0	0.0
0.0	0.0	0.0	0.0	0.0	0.0	0.0	0.0
0.0	0.0	0.0	0.0	0.0	0.0	0.0	0.0
0.0	0.0	0.0	0.0	0.0	0.0	0.0	0.0
0.0	0.0	0.0	0.0	0.0	0.0	0.0	0.0

0.0	0.0	0.0	0.0	0.0	0.0	0.0	0.0
0.0	0.0	0.0	0.0	0.0	0.0	0.0	0.0
0.0	0.0	0.0	0.0	0.0	0.0	0.0	0.0
0.0	0.0	0.0	0.0	0.0	0.0	0.0	0.0
0.0	0.0	0.0	0.0	0.0	0.0	0.0	0.0
0.0	0.0	0.0	0.0	0.0	0.0	0.0	0.0
0.0	0.0	0.0	0.0	0.0	0.0	0.0	0.0
0.0	0.0	0.0	0.0	0.0	0.0	0.0	0.0
0.0	0.0	0.0	0.0	0.0	0.0	0.0	0.0
0.0	0.0	0.0	0.0	0.0	0.0	0.0	0.0
0.0	0.0	0.0	0.0	0.0	0.0	0.0	0.0
0.0	0.0	0.0	0.0	0.0	0.0	0.0	0.0
0.0	0.0	0.0	0.0	0.0	0.0	0.0	0.0
0.0	0.0	0.0	0.0	0.0	0.0	0.0	0.0
0.0	0.0	0.0	0.0	0.0	0.0	0.0	0.0
0.0	0.0	0.0	0.0	0.0	0.0	0.0	0.0
0.0	0.0	0.0	0.0	0.0	0.0	0.0	0.0
0.0	0.0	0.0	0.0	0.0	0.0	0.0	0.0
0.0	0.0	0.0	0.0	0.0	0.0	0.0	0.0
0.0	0.0	0.0	0.0	0.0	0.0	0.0	0.0
0.0	0.0	0.0	0.0	0.0	0.0	0.0	0.0
0.0	0.0	0.0	0.0	0.0	0.0	0.0	0.0
0.0	0.0	0.0	0.0	0.0	0.0	0.0	0.0
0.0	0.0	0.0	0.0	0.0	0.0	0.0	0.0
0.0	0.0	0.0	0.0	0.0	0.0	0.0	0.0
0.0	0.0	0.0	0.0	0.0	0.0	0.0	0.0
0.0	0.0	0.0	0.0	0.0	0.0	0.0	0.0
0.0	0.0	0.0	0.0	0.0	0.0	0.0	0.0
0.0	0.0	0.0	0.0	0.0	0.0	0.0	0.0
0.0	0.0	0.0	0.0	0.0	0.0	0.0	0.0
0.0	0.0	0.0	0.0	0.0	0.0	0.0	0.0
0.0	0.0	0.0	0.0	0.0	0.0	0.0	0.0
0.0	0.0	0.0	0.0	0.0	0.0	0.0	0.0
0.0	0.0	0.0	0.0	0.0	0.0	0.0	0.0
0.0	0.0	0.0	0.0	0.0	0.0	0.0	0.0
0.0	0.0	0.0	0.0	0.0	0.0	0.0	0.0
0.0	0.0	0.0	0.0	0.0	0.0	0.0	0.0
0.0	0.0	0.0	0.0	0.0	0.0	0.0	0.0
0.0	0.0	0.0	0.0	0.0	0.0	0.0	0.0
0.0	0.0	0.0	0.0	0.0	0.0	0.0	0.0
0.0	0.0	0.0	0.0	0.0	0.0	0.0	0.0
0.0	0.0	0.0	0.0	0.0	0.0	0.0	0.0
0.0	0.0	0.0	0.0	0.0	0.0	0.0	0.0
0.0	0.0	0.0	0.0	0.0	0.0	0.0	0.0
0.0	0.0	0.0	0.0	0.0	0.0	0.0	0.0
0.0	0.0	0.0	0.0	0.0	0.0	0.0	0.0
0.0	0.0	0.0	0.0	0.0	0.0	0.0	0.0

0.0	0.0	0.0	0.0	0.0	0.0	0.0	0.0
0.0	0.0	0.0	0.0	0.0	0.0	0.0	0.0
0.0	0.0	0.0	0.0	0.0	0.0	0.0	0.0
0.0	0.0	0.0	0.0	0.0	0.0	0.0	0.0
0.0	0.0	0.0	0.0	0.0	0.0	0.0	0.0
0.0	0.0	0.0	0.0	0.0	0.0	0.0	0.0
0.0	0.0	0.0	0.0	0.0	0.0	0.0	0.0
0.0	0.0	0.0	0.0	0.0	0.0	0.0	0.0
0.0	0.0	0.0	0.0	0.0	0.0	0.0	0.0
0.0	0.0	0.0	0.0	0.0	0.0	0.0	0.0
0.0	0.0	0.0	0.0	0.0	0.0	0.0	0.0
0.0	0.0	0.0	0.0	0.0	0.0	0.0	0.0
0.0	0.0	0.0	0.0	0.0	0.0	0.0	0.0
0.0	0.0	0.0	0.0	0.0	0.0	0.0	0.0
0.0	0.0	0.0	0.0	0.0	0.0	0.0	0.0
0.0	0.0	0.0	0.0	0.0	0.0	0.0	0.0
0.0	0.0	0.0	0.0	0.0	0.0	0.0	0.0
0.0	0.0	0.0	0.0	0.0	0.0	0.0	0.0
0.0	0.0	0.0	0.0	0.0	0.0	0.0	0.0
0.0	0.0	0.0	0.0	0.0	0.0	0.0	0.0
0.0	0.0	0.0	0.0	0.0	0.0	0.0	0.0
0.0	0.0	0.0	0.0	0.0	0.0	0.0	0.0
0.0	0.0	0.0	0.0	0.0	0.0	0.0	0.0
0.0	0.0	0.0	0.0	0.0	0.0	0.0	0.0
0.0	0.0	0.0	0.0	0.0	0.0	0.0	0.0
0.0	0.0	0.0	0.0	0.0	0.0	0.0	0.0
0.0	0.0	0.0	0.0	0.0	0.0	0.0	0.0
0.0	0.0	0.0	0.0	0.0	0.0	0.0	0.0
0.0	0.0	0.0	0.0	0.0	0.0	0.0	0.0
0.0	0.0	0.0	0.0	0.0	0.0	0.0	0.0
0.0	0.0	0.0	0.0	0.0	0.0	0.0	0.0
0.0	0.0	0.0	0.0	0.0	0.0	0.0	0.0
0.0	0.0	0.0	0.0	0.0	0.0	0.0	0.0
0.0	0.0	0.0	0.0	0.0	0.0	0.0	0.0
0.0	0.0	0.0	0.0	0.0	0.0	0.0	0.0
0.0	0.0	0.0	0.0	0.0	0.0	0.0	0.0
0.0	0.0	0.0	0.0	0.0	0.0	0.0	0.0
0.0	0.0	0.0	0.0	0.0	0.0	0.0	0.0
0.0	0.0	0.0	0.0	0.0	0.0	0.0	0.0
0.0	0.0	0.0	0.0	0.0	0.0	0.0	0.0
0.0	0.0	0.0	0.0	0.0	0.0	0.0	0.0
0.0	0.0	0.0	0.0	0.0	0.0	0.0	0.0
0.0	0.0	0.0	0.0	0.0	0.0	0.0	0.0
0.0	0.0	0.0	0.0	0.0	0.0	0.0	0.0
0.0	0.0	0.0	0.0	0.0	0.0	0.0	0.0
0.0	0.0	0.0	0.0	0.0	0.0	0.0	0.0
0.0	0.0	0.0	0.0	0.0	0.0	0.0	0.0
0.0	0.0	0.0	0.0	0.0	0.0	0.0	0.0

0.0	0.0	0.0	0.0	0.0	0.0	0.0	0.0
0.0	0.0	0.0	0.0	0.0	0.0	0.0	0.0
0.0	0.0	0.0	0.0	0.0	0.0	0.0	0.0
0.0	0.0	0.0	0.0	0.0	0.0	0.0	0.0
0.0	0.0	0.0	0.0	0.0	0.0	0.0	0.0
0.0	0.0	0.0	0.0	0.0	0.0	0.0	0.0
0.0	0.0	0.0	0.0	0.0	0.0	0.0	0.0
0.0	0.0	0.0	0.0	0.0	0.0	0.0	0.0
0.0	0.0	0.0	0.0	0.0	0.0	0.0	0.0
0.0	0.0	0.0	0.0	0.0	0.0	0.0	0.0
0.0	0.0	0.0	0.0	0.0	0.0	0.0	0.0
0.0	0.0	0.0	0.0	0.0	0.0	0.0	0.0
0.0	0.0	0.0	0.0	0.0	0.0	0.0	0.0
0.0	0.0	0.0	0.0	0.0	0.0	0.0	0.0
0.0	0.0	0.0	0.0	0.0	0.0	0.0	0.0
0.0	0.0	0.0	0.0	0.0	0.0	0.0	0.0
0.0	0.0	0.0	0.0	0.0	0.0	0.0	0.0
0.0	0.0	0.0	0.0	0.0	0.0	0.0	0.0
0.0	0.0	0.0	0.0	0.0	0.0	0.0	0.0
0.0	0.0	0.0	0.0	0.0	0.0	0.0	0.0
0.0	0.0	0.0	0.0	0.0	0.0	0.0	0.0
0.0	0.0	0.0	0.0	0.0	0.0	0.0	0.0
0.0	0.0	0.0	0.0	0.0	0.0	0.0	0.0
0.0	0.0	0.0	0.0	0.0	0.0	0.0	0.0
0.0	0.0	0.0	0.0	0.0	0.0	0.0	0.0
0.0	0.0	0.0	0.0	0.0	0.0	0.0	0.0
0.0	0.0	0.0	0.0	0.0	0.0	0.0	0.0
0.0	0.0	0.0	0.0	0.0	0.0	0.0	0.0
0.0	0.0	0.0	0.0	0.0	0.0	0.0	0.0
0.0	0.0	0.0	0.0	0.0	0.0	0.0	0.0
0.0	0.0	0.0	0.0	0.0	0.0	0.0	0.0
0.0	0.0	0.0	0.0	0.0	0.0	0.0	0.0
0.0	0.0	0.0	0.0	0.0	0.0	0.0	0.0
0.0	0.0	0.0	0.0	0.0	0.0	0.0	0.0
0.0	0.0	0.0	0.0	0.0	0.0	0.0	0.0
0.0	0.0	0.0	0.0	0.0	0.0	0.0	0.0
0.0	0.0	0.0	0.0	0.0	0.0	0.0	0.0
0.0	0.0	0.0	0.0	0.0	0.0	0.0	0.0
0.0	0.0	0.0	0.0	0.0	0.0	0.0	0.0
0.0	0.0	0.0	0.0	0.0	0.0	0.0	0.0
0.0	0.0	0.0	0.0	0.0	0.0	0.0	0.0
0.0	0.0	0.0	0.0	0.0	0.0	0.0	0.0
0.0	0.0	0.0	0.0	0.0	0.0	0.0	0.0
0.0	0.0	0.0	0.0	0.0	0.0	0.0	0.0
0.0	0.0	0.0	0.0	0.0	0.0	0.0	0.0
0.0	0.0	0.0	0.0	0.0	0.0	0.0	0.0
0.0	0.0	0.0	0.0	0.0	0.0	0.0	0.0

0.0	0.0	0.0	0.0	0.0	0.0	0.0	0.0
0.0	0.0	0.0	0.0	0.0	0.0	0.0	0.0
0.0	0.0	0.0	0.0	0.0	0.0	0.0	0.0
0.0	0.0	0.0	0.0	0.0	0.0	0.0	0.0
0.0	0.0	0.0	0.0	0.0	0.0	0.0	0.0
0.0	0.0	0.0	0.0	0.0	0.0	0.0	0.0
0.0	0.0	0.0	0.0	0.0	0.0	0.0	0.0
0.0	0.0	0.0	0.0	0.0	0.0	0.0	0.0
0.0	0.0	0.0	0.0	0.0	0.0	0.0	0.0
0.0	0.0	0.0	0.0	0.0	0.0	0.0	0.0
0.0	0.0	0.0	0.0	0.0	0.0	0.0	0.0
0.0	0.0	0.0	0.0	0.0	0.0	0.0	0.0
0.0	0.0	0.0	0.0	0.0	0.0	0.0	0.0
0.0	0.0	0.0	0.0	0.0	0.0	0.0	0.0
0.0	0.0	0.0	0.0	0.0	0.0	0.0	0.0
0.0	0.0	0.0	0.0	0.0	0.0	0.0	0.0
0.0	0.0	0.0	0.0	0.0	0.0	0.0	0.0
0.0	0.0	0.0	0.0	0.0	0.0	0.0	0.0
0.0	0.0	0.0	0.0	0.0	0.0	0.0	0.0
0.0	0.0	0.0	0.0	0.0	0.0	0.0	0.0
0.0	0.0	0.0	0.0	0.0	0.0	0.0	0.0
0.0	0.0	0.0	0.0	0.0	0.0	0.0	0.0
0.0	0.0	0.0	0.0	0.0	0.0	0.0	0.0
0.0	0.0	0.0	0.0	0.0	0.0	0.0	0.0
0.0	0.0	0.0	0.0	0.0	0.0	0.0	0.0
0.0	0.0	0.0	0.0	0.0	0.0	0.0	0.0
0.0	0.0	0.0	0.0	0.0	0.0	0.0	0.0
0.0	0.0	0.0	0.0	0.0	0.0	0.0	0.0
0.0	0.0	0.0	0.0	0.0	0.0	0.0	0.0
0.0	0.0	0.0	0.0	0.0	0.0	0.0	0.0
0.0	0.0	0.0	0.0	0.0	0.0	0.0	0.0
0.0	0.0	0.0	0.0	0.0	0.0	0.0	0.0
0.0	0.0	0.0	0.0	0.0	0.0	0.0	0.0
0.0	0.0	0.0	0.0	0.0	0.0	0.0	0.0
0.0	0.0	0.0	0.0	0.0	0.0	0.0	0.0
0.0	0.0	0.0	0.0	0.0	0.0	0.0	0.0
0.0	0.0	0.0	0.0	0.0	0.0	0.0	0.0
0.0	0.0	0.0	0.0	0.0	0.0	0.0	0.0
0.0	0.0	0.0	0.0	0.0	0.0	0.0	0.0
0.0	0.0	0.0	0.0	0.0	0.0	0.0	0.0
0.0	0.0	0.0	0.0	0.0	0.0	0.0	0.0
0.0	0.0	0.0	0.0	0.0	0.0	0.0	0.0
0.0	0.0	0.0	0.0	0.0	0.0	0.0	0.0
0.0	0.0	0.0	0.0	0.0	0.0	0.0	0.0
0.0	0.0	0.0	0.0	0.0	0.0	0.0	0.0
0.0	0.0	0.0	0.0	0.0	0.0	0.0	0.0
0.0	0.0	0.0	0.0	0.0	0.0	0.0	0.0
0.0	0.0	0.0	0.0	0.0	0.0	0.0	0.0

0.0	0.0	0.0	0.0	0.0	0.0	0.0	0.0
0.0	0.0	0.0	0.0	0.0	0.0	0.0	0.0
0.0	0.0	0.0	0.0	0.0	0.0	0.0	0.0
0.0	0.0	0.0	0.0	0.0	0.0	0.0	0.0
0.0	0.0	0.0	0.0	0.0	0.0	0.0	0.0
0.0	0.0	0.0	0.0	0.0	0.0	0.0	0.0
0.0	0.0	0.0	0.0	0.0	0.0	0.0	0.0
0.0	0.0	0.0	0.0	0.0	0.0	0.0	0.0
0.0	0.0	0.0	0.0	0.0	0.0	0.0	0.0
0.0	0.0	0.0	0.0	0.0	0.0	0.0	0.0
0.0	0.0	0.0	0.0	0.0	0.0	0.0	0.0
0.0	0.0	0.0	0.0	0.0	0.0	0.0	0.0
0.0	0.0	0.0	0.0	0.0	0.0	0.0	0.0
0.0	0.0	0.0	0.0	0.0	0.0	0.0	0.0
0.0	0.0	0.0	0.0	0.0	0.0	0.0	0.0
0.0	0.0	0.0	0.0	0.0	0.0	0.0	0.0
0.0	0.0	0.0	0.0	0.0	0.0	0.0	0.0
0.0	0.0	0.0	0.0	0.0	0.0	0.0	0.0
0.0	0.0	0.0	0.0	0.0	0.0	0.0	0.0
0.0	0.0	0.0	0.0	0.0	0.0	0.0	0.0
0.0	0.0	0.0	0.0	0.0	0.0	0.0	0.0
0.0	0.0	0.0	0.0	0.0	0.0	0.0	0.0
0.0	0.0	0.0	0.0	0.0	0.0	0.0	0.0
0.0	0.0	0.0	0.0	0.0	0.0	0.0	0.0
0.0	0.0	0.0	0.0	0.0	0.0	0.0	0.0
0.0	0.0	0.0	0.0	0.0	0.0	0.0	0.0
0.0	0.0	0.0	0.0	0.0	0.0	0.0	0.0
0.0	0.0	0.0	0.0	0.0	0.0	0.0	0.0
0.0	0.0	0.0	0.0	0.0	0.0	0.0	0.0
0.0	0.0	0.0	0.0	0.0	0.0	0.0	0.0
0.0	0.0	0.0	0.0	0.0	0.0	0.0	0.0
0.0	0.0	0.0	0.0	0.0	0.0	0.0	0.0
0.0	0.0	0.0	0.0	0.0	0.0	0.0	0.0
0.0	0.0	0.0	0.0	0.0	0.0	0.0	0.0
0.0	0.0	0.0	0.0	0.0	0.0	0.0	0.0
0.0	0.0	0.0	0.0	0.0	0.0	0.0	0.0
0.0	0.0	0.0	0.0	0.0	0.0	0.0	0.0
0.0	0.0	0.0	0.0	0.0	0.0	0.0	0.0
0.0	0.0	0.0	0.0	0.0	0.0	0.0	0.0
0.0	0.0	0.0	0.0	0.0	0.0	0.0	0.0
0.0	0.0	0.0	0.0	0.0	0.0	0.0	0.0
0.0	0.0	0.0	0.0	0.0	0.0	0.0	0.0
0.0	0.0	0.0	0.0	0.0	0.0	0.0	0.0
0.0	0.0	0.0	0.0	0.0	0.0	0.0	0.0
0.0	0.0	0.0	0.0	0.0	0.0	0.0	0.0
0.0	0.0	0.0	0.0	0.0	0.0	0.0	0.0
0.0	0.0	0.0	0.0	0.0	0.0	0.0	0.0

0.0	0.0	0.0	0.0	0.0	0.0	0.0	0.0
0.0	0.0	0.0	0.0	0.0	0.0	0.0	0.0
0.0	0.0	0.0	0.0	0.0	0.0	0.0	0.0
0.0	0.0	0.0	0.0	0.0	0.0	0.0	0.0
0.0	0.0	0.0	0.0	0.0	0.0	0.0	0.0
0.0	0.0	0.0	0.0	0.0	0.0	0.0	0.0
0.0	0.0	0.0	0.0	0.0	0.0	0.0	0.0
0.0	0.0	0.0	0.0	0.0	0.0	0.0	0.0
0.0	0.0	0.0	0.0	0.0	0.0	0.0	0.0
0.0	0.0	0.0	0.0	0.0	0.0	0.0	0.0
0.0	0.0	0.0	0.0	0.0	0.0	0.0	0.0
0.0	0.0	0.0	0.0	0.0	0.0	0.0	0.0
0.0	0.0	0.0	0.0	0.0	0.0	0.0	0.0
0.0	0.0	0.0	0.0	0.0	0.0	0.0	0.0
0.0	0.0	0.0	0.0	0.0	0.0	0.0	0.0
0.0	0.0	0.0	0.0	0.0	0.0	0.0	0.0
0.0	0.0	0.0	0.0	0.0	0.0	0.0	0.0
0.0	0.0	0.0	0.0	0.0	0.0	0.0	0.0
0.0	0.0	0.0	0.0	0.0	0.0	0.0	0.0
0.0	0.0	0.0	0.0	0.0	0.0	0.0	0.0
0.0	0.0	0.0	0.0	0.0	0.0	0.0	0.0
0.0	0.0	0.0	0.0	0.0	0.0	0.0	0.0
0.0	0.0	0.0	0.0	0.0	0.0	0.0	0.0
0.0	0.0	0.0	0.0	0.0	0.0	0.0	0.0
0.0	0.0	0.0	0.0	0.0	0.0	0.0	0.0
0.0	0.0	0.0	0.0	0.0	0.0	0.0	0.0
0.0	0.0	0.0	0.0	0.0	0.0	0.0	0.0
0.0	0.0	0.0	0.0	0.0	0.0	0.0	0.0
0.0	0.0	0.0	0.0	0.0	0.0	0.0	0.0
0.0	0.0	0.0	0.0	0.0	0.0	0.0	0.0
0.0	0.0	0.0	0.0	0.0	0.0	0.0	0.0
0.0	0.0	0.0	0.0	0.0	0.0	0.0	0.0
0.0	0.0	0.0	0.0	0.0	0.0	0.0	0.0
0.0	0.0	0.0	0.0	0.0	0.0	0.0	0.0
0.0	0.0	0.0	0.0	0.0	0.0	0.0	0.0
0.0	0.0	0.0	0.0	0.0	0.0	0.0	0.0
0.0	0.0	0.0	0.0	0.0	0.0	0.0	0.0
0.0	0.0	0.0	0.0	0.0	0.0	0.0	0.0
0.0	0.0	0.0	0.0	0.0	0.0	0.0	0.0
0.0	0.0	0.0	0.0	0.0	0.0	0.0	0.0
0.0	0.0	0.0	0.0	0.0	0.0	0.0	0.0
0.0	0.0	0.0	0.0	0.0	0.0	0.0	0.0
0.0	0.0	0.0	0.0	0.0	0.0	0.0	0.0
0.0	0.0	0.0	0.0	0.0	0.0	0.0	0.0
0.0	0.0	0.0	0.0	0.0	0.0	0.0	0.0
0.0	0.0	0.0	0.0	0.0	0.0	0.0	0.0
0.0	0.0	0.0	0.0	0.0	0.0	0.0	0.0

0.0	0.0	0.0	0.0	0.0	0.0	0.0	0.0
0.0	0.0	0.0	0.0	0.0	0.0	0.0	0.0
0.0	0.0	0.0	0.0	0.0	0.0	0.0	0.0
0.0	0.0	0.0	0.0	0.0	0.0	0.0	0.0
0.0	0.0	0.0	0.0	0.0	0.0	0.0	0.0
0.0	0.0	0.0	0.0	0.0	0.0	0.0	0.0
0.0	0.0	0.0	0.0	0.0	0.0	0.0	0.0
0.0	0.0	0.0	0.0	0.0	0.0	0.0	0.0
0.0	0.0	0.0	0.0	0.0	0.0	0.0	0.0
0.0	0.0	0.0	0.0	0.0	0.0	0.0	0.0
0.0	0.0	0.0	0.0	0.0	0.0	0.0	0.0
0.0	0.0	0.0	0.0	0.0	0.0	0.0	0.0
0.0	0.0	0.0	0.0	0.0	0.0	0.0	0.0
0.0	0.0	0.0	0.0	0.0	0.0	0.0	0.0
0.0	0.0	0.0	0.0	0.0	0.0	0.0	0.0
0.0	0.0	0.0	0.0	0.0	0.0	0.0	0.0
0.0	0.0	0.0	0.0	0.0	0.0	0.0	0.0
0.0	0.0	0.0	0.0	0.0	0.0	0.0	0.0
0.0	0.0	0.0	0.0	0.0	0.0	0.0	0.0
0.0	0.0	0.0	0.0	0.0	0.0	0.0	0.0
0.0	0.0	0.0	0.0	0.0	0.0	0.0	0.0
0.0	0.0	0.0	0.0	0.0	0.0	0.0	0.0
0.0	0.0	0.0	0.0	0.0	0.0	0.0	0.0
0.0	0.0	0.0	0.0	0.0	0.0	0.0	0.0
0.0	0.0	0.0	0.0	0.0	0.0	0.0	0.0
0.0	0.0	0.0	0.0	0.0	0.0	0.0	0.0
0.0	0.0	0.0	0.0	0.0	0.0	0.0	0.0
0.0	0.0	0.0	0.0	0.0	0.0	0.0	0.0
0.0	0.0	0.0	0.0	0.0	0.0	0.0	0.0
0.0	0.0	0.0	0.0	0.0	0.0	0.0	0.0
0.0	0.0	0.0	0.0	0.0	0.0	0.0	0.0
0.0	0.0	0.0	0.0	0.0	0.0	0.0	0.0
0.0	0.0	0.0	0.0	0.0	0.0	0.0	0.0
0.0	0.0	0.0	0.0	0.0	0.0	0.0	0.0
0.0	0.0	0.0	0.0	0.0	0.0	0.0	0.0
0.0	0.0	0.0	0.0	0.0	0.0	0.0	0.0
0.0	0.0	0.0	0.0	0.0	0.0	0.0	0.0
0.0	0.0	0.0	0.0	0.0	0.0	0.0	0.0
0.0	0.0	0.0	0.0	0.0	0.0	0.0	0.0
0.0	0.0	0.0	0.0	0.0	0.0	0.0	0.0
0.0	0.0	0.0	0.0	0.0	0.0	0.0	0.0
0.0	0.0	0.0	0.0	0.0	0.0	0.0	0.0
0.0	0.0	0.0	0.0	0.0	0.0	0.0	0.0
0.0	0.0	0.0	0.0	0.0	0.0	0.0	0.0
0.0	0.0	0.0	0.0	0.0	0.0	0.0	0.0
0.0	0.0	0.0	0.0	0.0	0.0	0.0	0.0
0.0	0.0	0.0	0.0	0.0	0.0	0.0	0.0

0.0	0.0	0.0	0.0	0.0	0.0	0.0	0.0
0.0	0.0	0.0	0.0	0.0	0.0	0.0	0.0
0.0	0.0	0.0	0.0	0.0	0.0	0.0	0.0
0.0	0.0	0.0	0.0	0.0	0.0	0.0	0.0
0.0	0.0	0.0	0.0	0.0	0.0	0.0	0.0
0.0	0.0	0.0	0.0	0.0	0.0	0.0	0.0
0.0	0.0	0.0	0.0	0.0	0.0	0.0	0.0
0.0	0.0	0.0	0.0	0.0	0.0	0.0	0.0
0.0	0.0	0.0	0.0	0.0	0.0	0.0	0.0
0.0	0.0	0.0	0.0	0.0	0.0	0.0	0.0
0.0	0.0	0.0	0.0	0.0	0.0	0.0	0.0
0.0	0.0	0.0	0.0	0.0	0.0	0.0	0.0
0.0	0.0	0.0	0.0	0.0	0.0	0.0	0.0
0.0	0.0	0.0	0.0	0.0	0.0	0.0	0.0
0.0	0.0	0.0	0.0	0.0	0.0	0.0	0.0
0.0	0.0	0.0	0.0	0.0	0.0	0.0	0.0
0.0	0.0	0.0	0.0	0.0	0.0	0.0	0.0
0.0	0.0	0.0	0.0	0.0	0.0	0.0	0.0
0.0	0.0	0.0	0.0	0.0	0.0	0.0	0.0
0.0	0.0	0.0	0.0	0.0	0.0	0.0	0.0
0.0	0.0	0.0	0.0	0.0	0.0	0.0	0.0
0.0	0.0	0.0	0.0	0.0	0.0	0.0	0.0
0.0	0.0	0.0	0.0	0.0	0.0	0.0	0.0
0.0	0.0	0.0	0.0	0.0	0.0	0.0	0.0
0.0	0.0	0.0	0.0	0.0	0.0	0.0	0.0
0.0	0.0	0.0	0.0	0.0	0.0	0.0	0.0
0.0	0.0	0.0	0.0	0.0	0.0	0.0	0.0
0.0	0.0	0.0	0.0	0.0	0.0	0.0	0.0
0.0	0.0	0.0	0.0	0.0	0.0	0.0	0.0
0.0	0.0	0.0	0.0	0.0	0.0	0.0	0.0
0.0	0.0	0.0	0.0	0.0	0.0	0.0	0.0
0.0	0.0	0.0	0.0	0.0	0.0	0.0	0.0
0.0	0.0	0.0	0.0	0.0	0.0	0.0	0.0
0.0	0.0	0.0	0.0	0.0	0.0	0.0	0.0
0.0	0.0	0.0	0.0	0.0	0.0	0.0	0.0
0.0	0.0	0.0	0.0	0.0	0.0	0.0	0.0
0.0	0.0	0.0	0.0	0.0	0.0	0.0	0.0
0.0	0.0	0.0	0.0	0.0	0.0	0.0	0.0
0.0	0.0	0.0	0.0	0.0	0.0	0.0	0.0
0.0	0.0	0.0	0.0	0.0	0.0	0.0	0.0
0.0	0.0	0.0	0.0	0.0	0.0	0.0	0.0
0.0	0.0	0.0	0.0	0.0	0.0	0.0	0.0
0.0	0.0	0.0	0.0	0.0	0.0	0.0	0.0
0.0	0.0	0.0	0.0	0.0	0.0	0.0	0.0
0.0	0.0	0.0	0.0	0.0	0.0	0.0	0.0
0.0	0.0	0.0	0.0	0.0	0.0	0.0	0.0
0.0	0.0	0.0	0.0	0.0	0.0	0.0	0.0

0.0	0.0	0.0	0.0	0.0	0.0	0.0	0.0
0.0	0.0	0.0	0.0	0.0	0.0	0.0	0.0
0.0	0.0	0.0	0.0	0.0	0.0	0.0	0.0
0.0	0.0	0.0	0.0	0.0	0.0	0.0	0.0
0.0	0.0	0.0	0.0	0.0	0.0	0.0	0.0
0.0	0.0	0.0	0.0	0.0	0.0	0.0	0.0
0.0	0.0	0.0	0.0	0.0	0.0	0.0	0.0
0.0	0.0	0.0	0.0	0.0	0.0	0.0	0.0
0.0	0.0	0.0	0.0	0.0	0.0	0.0	0.0
0.0	0.0	0.0	0.0	0.0	0.0	0.0	0.0
0.0	0.0	0.0	0.0	0.0	0.0	0.0	0.0
0.0	0.0	0.0	0.0	0.0	0.0	0.0	0.0
0.0	0.0	0.0	0.0	0.0	0.0	0.0	0.0
0.0	0.0	0.0	0.0	0.0	0.0	0.0	0.0
0.0	0.0	0.0	0.0	0.0	0.0	0.0	0.0
0.0	0.0	0.0	0.0	0.0	0.0	0.0	0.0
0.0	0.0	0.0	0.0	0.0	0.0	0.0	0.0
0.0	0.0	0.0	0.0	0.0	0.0	0.0	0.0
0.0	0.0	0.0	0.0	0.0	0.0	0.0	0.0
0.0	0.0	0.0	0.0	0.0	0.0	0.0	0.0
0.0	0.0	0.0	0.0	0.0	0.0	0.0	0.0
0.0	0.0	0.0	0.0	0.0	0.0	0.0	0.0
0.0	0.0	0.0	0.0	0.0	0.0	0.0	0.0
0.0	0.0	0.0	0.0	0.0	0.0	0.0	0.0
0.0	0.0	0.0	0.0	0.0	0.0	0.0	0.0
0.0	0.0	0.0	0.0	0.0	0.0	0.0	0.0
0.0	0.0	0.0	0.0	0.0	0.0	0.0	0.0
0.0	0.0	0.0	0.0	0.0	0.0	0.0	0.0
0.0	0.0	0.0	0.0	0.0	0.0	0.0	0.0
0.0	0.0	0.0	0.0	0.0	0.0	0.0	0.0
0.0	0.0	0.0	0.0	0.0	0.0	0.0	0.0
0.0	0.0	0.0	0.0	0.0	0.0	0.0	0.0
0.0	0.0	0.0	0.0	0.0	0.0	0.0	0.0
0.0	0.0	0.0	0.0	0.0	0.0	0.0	0.0
0.0	0.0	0.0	0.0	0.0	0.0	0.0	0.0
0.0	0.0	0.0	0.0	0.0	0.0	0.0	0.0
0.0	0.0	0.0	0.0	0.0	0.0	0.0	0.0
0.0	0.0	0.0	0.0	0.0	0.0	0.0	0.0
0.0	0.0	0.0	0.0	0.0	0.0	0.0	0.0
0.0	0.0	0.0	0.0	0.0	0.0	0.0	0.0
0.0	0.0	0.0	0.0	0.0	0.0	0.0	0.0
0.0	0.0	0.0	0.0	0.0	0.0	0.0	0.0
0.0	0.0	0.0	0.0	0.0	0.0	0.0	0.0
0.0	0.0	0.0	0.0	0.0	0.0	0.0	0.0
0.0	0.0	0.0	0.0	0.0	0.0	0.0	0.0
0.0	0.0	0.0	0.0	0.0	0.0	0.0	0.0
0.0	0.0	0.0	0.0	0.0	0.0	0.0	0.0

0.0	0.0	0.0	0.0	0.0	0.0	0.0	0.0
0.0	0.0	0.0	0.0	0.0	0.0	0.0	0.0
0.0	0.0	0.0	0.0	0.0	0.0	0.0	0.0
0.0	0.0	0.0	0.0	0.0	0.0	0.0	0.0
0.0	0.0	0.0	0.0	0.0	0.0	0.0	0.0
0.0	0.0	0.0	0.0	0.0	0.0	0.0	0.0
0.0	0.0	0.0	0.0	0.0	0.0	0.0	0.0
0.0	0.0	0.0	0.0	0.0	0.0	0.0	0.0
0.0	0.0	0.0	0.0	0.0	0.0	0.0	0.0
0.0	0.0	0.0	0.0	0.0	0.0	0.0	0.0
0.0	0.0	0.0	0.0	0.0	0.0	0.0	0.0
0.0	0.0	0.0	0.0	0.0	0.0	0.0	0.0
0.0	0.0	0.0	0.0	0.0	0.0	0.0	0.0
0.0	0.0	0.0	0.0	0.0	0.0	0.0	0.0
0.0	0.0	0.0	0.0	0.0	0.0	0.0	0.0
0.0	0.0	0.0	0.0	0.0	0.0	0.0	0.0
0.0	0.0	0.0	0.0	0.0	0.0	0.0	0.0
0.0	0.0	0.0	0.0	0.0	0.0	0.0	0.0
0.0	0.0	0.0	0.0	0.0	0.0	0.0	0.0
0.0	0.0	0.0	0.0	0.0	0.0	0.0	0.0
0.0	0.0	0.0	0.0	0.0	0.0	0.0	0.0
0.0	0.0	0.0	0.0	0.0	0.0	0.0	0.0
0.0	0.0	0.0	0.0	0.0	0.0	0.0	0.0
0.0	0.0	0.0	0.0	0.0	0.0	0.0	0.0
0.0	0.0	0.0	0.0	0.0	0.0	0.0	0.0
0.0	0.0	0.0	0.0	0.0	0.0	0.0	0.0
0.0	0.0	0.0	0.0	0.0	0.0	0.0	0.0
0.0	0.0	0.0	0.0	0.0	0.0	0.0	0.0
0.0	0.0	0.0	0.0	0.0	0.0	0.0	0.0
0.0	0.0	0.0	0.0	0.0	0.0	0.0	0.0
0.0	0.0	0.0	0.0	0.0	0.0	0.0	0.0
0.0	0.0	0.0	0.0	0.0	0.0	0.0	0.0
0.0	0.0	0.0	0.0	0.0	0.0	0.0	0.0
0.0	0.0	0.0	0.0	0.0	0.0	0.0	0.0
0.0	0.0	0.0	0.0	0.0	0.0	0.0	0.0
0.0	0.0	0.0	0.0	0.0	0.0	0.0	0.0
0.0	0.0	0.0	0.0	0.0	0.0	0.0	0.0
0.0	0.0	0.0	0.0	0.0	0.0	0.0	0.0
0.0	0.0	0.0	0.0	0.0	0.0	0.0	0.0
0.0	0.0	0.0	0.0	0.0	0.0	0.0	0.0
0.0	0.0	0.0	0.0	0.0	0.0	0.0	0.0
0.0	0.0	0.0	0.0	0.0	0.0	0.0	0.0
0.0	0.0	0.0	0.0	0.0	0.0	0.0	0.0
0.0	0.0	0.0	0.0	0.0	0.0	0.0	0.0
0.0	0.0	0.0	0.0	0.0	0.0	0.0	0.0
0.0	0.0	0.0	0.0	0.0	0.0	0.0	0.0
0.0	0.0	0.0	0.0	0.0	0.0	0.0	0.0

0.0	0.0	0.0	0.0	0.0	0.0	0.0	0.0
0.0	0.0	0.0	0.0	0.0	0.0	0.0	0.0
0.0	0.0	0.0	0.0	0.0	0.0	0.0	0.0
0.0	0.0	0.0	0.0	0.0	0.0	0.0	0.0
0.0	0.0	0.0	0.0	0.0	0.0	0.0	0.0
0.0	0.0	0.0	0.0	0.0	0.0	0.0	0.0
0.0	0.0	0.0	0.0	0.0	0.0	0.0	0.0
0.0	0.0	0.0	0.0	0.0	0.0	0.0	0.0
0.0	0.0	0.0	0.0	0.0	0.0	0.0	0.0
0.0	0.0	0.0	0.0	0.0	0.0	0.0	0.0
0.0	0.0	0.0	0.0	0.0	0.0	0.0	0.0
0.0	0.0	0.0	0.0	0.0	0.0	0.0	0.0
0.0	0.0	0.0	0.0	0.0	0.0	0.0	0.0
0.0	0.0	0.0	0.0	0.0	0.0	0.0	0.0
0.0	0.0	0.0	0.0	0.0	0.0	0.0	0.0
0.0	0.0	0.0	0.0	0.0	0.0	0.0	0.0
0.0	0.0	0.0	0.0	0.0	0.0	0.0	0.0
0.0	0.0	0.0	0.0	0.0	0.0	0.0	0.0
0.0	0.0	0.0	0.0	0.0	0.0	0.0	0.0
0.0	0.0	0.0	0.0	0.0	0.0	0.0	0.0
0.0	0.0	0.0	0.0	0.0	0.0	0.0	0.0
0.0	0.0	0.0	0.0	0.0	0.0	0.0	0.0
0.0	0.0	0.0	0.0	0.0	0.0	0.0	0.0
0.0	0.0	0.0	0.0	0.0	0.0	0.0	0.0
0.0	0.0	0.0	0.0	0.0	0.0	0.0	0.0
0.0	0.0	0.0	0.0	0.0	0.0	0.0	0.0
0.0	0.0	0.0	0.0	0.0	0.0	0.0	0.0
0.0	0.0	0.0	0.0	0.0	0.0	0.0	0.0
0.0	0.0	0.0	0.0	0.0	0.0	0.0	0.0
0.0	0.0	0.0	0.0	0.0	0.0	0.0	0.0
0.0	0.0	0.0	0.0	0.0	0.0	0.0	0.0
0.0	0.0	0.0	0.0	0.0	0.0	0.0	0.0
0.0	0.0	0.0	0.0	0.0	0.0	0.0	0.0
0.0	0.0	0.0	0.0	0.0	0.0	0.0	0.0
0.0	0.0	0.0	0.0	0.0	0.0	0.0	0.0
0.0	0.0	0.0	0.0	0.0	0.0	0.0	0.0
0.0	0.0	0.0	0.0	0.0	0.0	0.0	0.0
0.0	0.0	0.0	0.0	0.0	0.0	0.0	0.0
0.0	0.0	0.0	0.0	0.0	0.0	0.0	0.0
0.0	0.0	0.0	0.0	0.0	0.0	0.0	0.0
0.0	0.0	0.0	0.0	0.0	0.0	0.0	0.0
0.0	0.0	0.0	0.0	0.0	0.0	0.0	0.0
0.0	0.0	0.0	0.0	0.0	0.0	0.0	0.0
0.0	0.0	0.0	0.0	0.0	0.0	0.0	0.0
0.0	0.0	0.0	0.0	0.0	0.0	0.0	0.0
0.0	0.0	0.0	0.0	0.0	0.0	0.0	0.0
0.0	0.0	0.0	0.0	0.0	0.0	0.0	0.0

0.0	0.0	0.0	0.0	0.0	0.0	0.0	0.0
0.0	0.0	0.0	0.0	0.0	0.0	0.0	0.0
0.0	0.0	0.0	0.0	0.0	0.0	0.0	0.0
0.0	0.0	0.0	0.0	0.0	0.0	0.0	0.0
0.0	0.0	0.0	0.0	0.0	0.0	0.0	0.0
0.0	0.0	0.0	0.0	0.0	0.0	0.0	0.0
0.0	0.0	0.0	0.0	0.0	0.0	0.0	0.0
0.0	0.0	0.0	0.0	0.0	0.0	0.0	0.0
0.0	0.0	0.0	0.0	0.0	0.0	0.0	0.0
0.0	0.0	0.0	0.0	0.0	0.0	0.0	0.0
0.0	0.0	0.0	0.0	0.0	0.0	0.0	0.0
0.0	0.0	0.0	0.0	0.0	0.0	0.0	0.0
0.0	0.0	0.0	0.0	0.0	0.0	0.0	0.0
0.0	0.0	0.0	0.0	0.0	0.0	0.0	0.0
0.0	0.0	0.0	0.0	0.0	0.0	0.0	0.0
0.0	0.0	0.0	0.0	0.0	0.0	0.0	0.0
0.0	0.0	0.0	0.0	0.0	0.0	0.0	0.0
0.0	0.0	0.0	0.0	0.0	0.0	0.0	0.0
0.0	0.0	0.0	0.0	0.0	0.0	0.0	0.0
0.0	0.0	0.0	0.0	0.0	0.0	0.0	0.0
0.0	0.0	0.0	0.0	0.0	0.0	0.0	0.0
0.0	0.0	0.0	0.0	0.0	0.0	0.0	0.0
0.0	0.0	0.0	0.0	0.0	0.0	0.0	0.0
0.0	0.0	0.0	0.0	0.0	0.0	0.0	0.0
0.0	0.0	0.0	0.0	0.0	0.0	0.0	0.0
0.0	0.0	0.0	0.0	0.0	0.0	0.0	0.0
0.0	0.0	0.0	0.0	0.0	0.0	0.0	0.0
0.0	0.0	0.0	0.0	0.0	0.0	0.0	0.0
0.0	0.0	0.0	0.0	0.0	0.0	0.0	0.0
0.0	0.0	0.0	0.0	0.0	0.0	0.0	0.0
0.0	0.0	0.0	0.0	0.0	0.0	0.0	0.0
0.0	0.0	0.0	0.0	0.0	0.0	0.0	0.0
0.0	0.0	0.0	0.0	0.0	0.0	0.0	0.0
0.0	0.0	0.0	0.0	0.0	0.0	0.0	0.0
0.0	0.0	0.0	0.0	0.0	0.0	0.0	0.0
0.0	0.0	0.0	0.0	0.0	0.0	0.0	0.0
0.0	0.0	0.0	0.0	0.0	0.0	0.0	0.0
0.0	0.0	0.0	0.0	0.0	0.0	0.0	0.0
0.0	0.0	0.0	0.0	0.0	0.0	0.0	0.0
0.0	0.0	0.0	0.0	0.0	0.0	0.0	0.0
0.0	0.0	0.0	0.0	0.0	0.0	0.0	0.0
0.0	0.0	0.0	0.0	0.0	0.0	0.0	0.0
0.0	0.0	0.0	0.0	0.0	0.0	0.0	0.0
0.0	0.0	0.0	0.0	0.0	0.0	0.0	0.0
0.0	0.0	0.0	0.0	0.0	0.0	0.0	0.0
0.0	0.0	0.0	0.0	0.0	0.0	0.0	0.0
0.0	0.0	0.0	0.0	0.0	0.0	0.0	0.0

0.0	0.0	0.0	0.0	0.0	0.0	0.0	0.0
0.0	0.0	0.0	0.0	0.0	0.0	0.0	0.0
0.0	0.0	0.0	0.0	0.0	0.0	0.0	0.0
0.0	0.0	0.0	0.0	0.0	0.0	0.0	0.0
0.0	0.0	0.0	0.0	0.0	0.0	0.0	0.0
0.0	0.0	0.0	0.0	0.0	0.0	0.0	0.0
0.0	0.0	0.0	0.0	0.0	0.0	0.0	0.0
0.0	0.0	0.0	0.0	0.0	0.0	0.0	0.0
0.0	0.0	0.0	0.0	0.0	0.0	0.0	0.0
0.0	0.0	0.0	0.0	0.0	0.0	0.0	0.0
0.0	0.0	0.0	0.0	0.0	0.0	0.0	0.0
0.0	0.0	0.0	0.0	0.0	0.0	0.0	0.0
0.0	0.0	0.0	0.0	0.0	0.0	0.0	0.0
0.0	0.0	0.0	0.0	0.0	0.0	0.0	0.0
0.0	0.0	0.0	0.0	0.0	0.0	0.0	0.0
0.0	0.0	0.0	0.0	0.0	0.0	0.0	0.0
0.0	0.0	0.0	0.0	0.0	0.0	0.0	0.0
0.0	0.0	0.0	0.0	0.0	0.0	0.0	0.0
0.0	0.0	0.0	0.0	0.0	0.0	0.0	0.0
0.0	0.0	0.0	0.0	0.0	0.0	0.0	0.0
0.0	0.0	0.0	0.0	0.0	0.0	0.0	0.0
0.0	0.0	0.0	0.0	0.0	0.0	0.0	0.0
0.0	0.0	0.0	0.0	0.0	0.0	0.0	0.0
0.0	0.0	0.0	0.0	0.0	0.0	0.0	0.0
0.0	0.0	0.0	0.0	0.0	0.0	0.0	0.0
0.0	0.0	0.0	0.0	0.0	0.0	0.0	0.0
0.0	0.0	0.0	0.0	0.0	0.0	0.0	0.0
0.0	0.0	0.0	0.0	0.0	0.0	0.0	0.0
0.0	0.0	0.0	0.0	0.0	0.0	0.0	0.0
0.0	0.0	0.0	0.0	0.0	0.0	0.0	0.0
0.0	0.0	0.0	0.0	0.0	0.0	0.0	0.0
0.0	0.0	0.0	0.0	0.0	0.0	0.0	0.0
0.0	0.0	0.0	0.0	0.0	0.0	0.0	0.0
0.0	0.0	0.0	0.0	0.0	0.0	0.0	0.0
0.0	0.0	0.0	0.0	0.0	0.0	0.0	0.0
0.0	0.0	0.0	0.0	0.0	0.0	0.0	0.0
0.0	0.0	0.0	0.0	0.0	0.0	0.0	0.0
0.0	0.0	0.0	0.0	0.0	0.0	0.0	0.0
0.0	0.0	0.0	0.0	0.0	0.0	0.0	0.0
0.0	0.0	0.0	0.0	0.0	0.0	0.0	0.0
0.0	0.0	0.0	0.0	0.0	0.0	0.0	0.0
0.0	0.0	0.0	0.0	0.0	0.0	0.0	0.0
0.0	0.0	0.0	0.0	0.0	0.0	0.0	0.0
0.0	0.0	0.0	0.0	0.0	0.0	0.0	0.0
0.0	0.0	0.0	0.0	0.0	0.0	0.0	0.0
0.0	0.0	0.0	0.0	0.0	0.0	0.0	0.0
0.0	0.0	0.0	0.0	0.0	0.0	0.0	0.0
0.0	0.0	0.0	0.0	0.0	0.0	0.0	0.0

0.0	0.0	0.0	0.0	0.0	0.0	0.0	0.0
0.0	0.0	0.0	0.0	0.0	0.0	0.0	0.0
0.0	0.0	0.0	0.0	0.0	0.0	0.0	0.0
0.0	0.0	0.0	0.0	0.0	0.0	0.0	0.0
0.0	0.0	0.0	0.0	0.0	0.0	0.0	0.0
0.0	0.0	0.0	0.0	0.0	0.0	0.0	0.0
0.0	0.0	0.0	0.0	0.0	0.0	0.0	0.0
0.0	0.0	0.0	0.0	0.0	0.0	0.0	0.0
0.0	0.0	0.0	0.0	0.0	0.0	0.0	0.0
0.0	0.0	0.0	0.0	0.0	0.0	0.0	0.0
0.0	0.0	0.0	0.0	0.0	0.0	0.0	0.0
0.0	0.0	0.0	0.0	0.0	0.0	0.0	0.0
0.0	0.0	0.0	0.0	0.0	0.0	0.0	0.0
0.0	0.0	0.0	0.0	0.0	0.0	0.0	0.0
0.0	0.0	0.0	0.0	0.0	0.0	0.0	0.0
0.0	0.0	0.0	0.0	0.0	0.0	0.0	0.0
0.0	0.0	0.0	0.0	0.0	0.0	0.0	0.0
0.0	0.0	0.0	0.0	0.0	0.0	0.0	0.0
0.0	0.0	0.0	0.0	0.0	0.0	0.0	0.0
0.0	0.0	0.0	0.0	0.0	0.0	0.0	0.0
0.0	0.0	0.0	0.0	0.0	0.0	0.0	0.0
0.0	0.0	0.0	0.0	0.0	0.0	0.0	0.0
0.0	0.0	0.0	0.0	0.0	0.0	0.0	0.0
0.0	0.0	0.0	0.0	0.0	0.0	0.0	0.0
0.0	0.0	0.0	0.0	0.0	0.0	0.0	0.0
0.0	0.0	0.0	0.0	0.0	0.0	0.0	0.0
0.0	0.0	0.0	0.0	0.0	0.0	0.0	0.0
0.0	0.0	0.0	0.0	0.0	0.0	0.0	0.0
0.0	0.0	0.0	0.0	0.0	0.0	0.0	0.0
0.0	0.0	0.0	0.0	0.0	0.0	0.0	0.0
0.0	0.0	0.0	0.0	0.0	0.0	0.0	0.0
0.0	0.0	0.0	0.0	0.0	0.0	0.0	0.0
0.0	0.0	0.0	0.0	0.0	0.0	0.0	0.0
0.0	0.0	0.0	0.0	0.0	0.0	0.0	0.0
0.0	0.0	0.0	0.0	0.0	0.0	0.0	0.0
0.0	0.0	0.0	0.0	0.0	0.0	0.0	0.0
0.0	0.0	0.0	0.0	0.0	0.0	0.0	0.0
0.0	0.0	0.0	0.0	0.0	0.0	0.0	0.0
0.0	0.0	0.0	0.0	0.0	0.0	0.0	0.0
0.0	0.0	0.0	0.0	0.0	0.0	0.0	0.0
0.0	0.0	0.0	0.0	0.0	0.0	0.0	0.0
0.0	0.0	0.0	0.0	0.0	0.0	0.0	0.0
0.0	0.0	0.0	0.0	0.0	0.0	0.0	0.0
0.0	0.0	0.0	0.0	0.0	0.0	0.0	0.0
0.0	0.0	0.0	0.0	0.0	0.0	0.0	0.0
0.0	0.0	0.0	0.0	0.0	0.0	0.0	0.0
0.0	0.0	0.0	0.0	0.0	0.0	0.0	0.0

0.0	0.0	0.0	0.0	0.0	0.0	0.0	0.0
0.0	0.0	0.0	0.0	0.0	0.0	0.0	0.0
0.0	0.0	0.0	0.0	0.0	0.0	0.0	0.0
0.0	0.0	0.0	0.0	0.0	0.0	0.0	0.0
0.0	0.0	0.0	0.0	0.0	0.0	0.0	0.0
0.0	0.0	0.0	0.0	0.0	0.0	0.0	0.0
0.0	0.0	0.0	0.0	0.0	0.0	0.0	0.0
0.0	0.0	0.0	0.0	0.0	0.0	0.0	0.0
0.0	0.0	0.0	0.0	0.0	0.0	0.0	0.0
0.0	0.0	0.0	0.0	0.0	0.0	0.0	0.0
0.0	0.0	0.0	0.0	0.0	0.0	0.0	0.0
0.0	0.0	0.0	0.0	0.0	0.0	0.0	0.0
0.0	0.0	0.0	0.0	0.0	0.0	0.0	0.0
0.0	0.0	0.0	0.0	0.0	0.0	0.0	0.0
0.0	0.0	0.0	0.0	0.0	0.0	0.0	0.0
0.0	0.0	0.0	0.0	0.0	0.0	0.0	0.0
0.0	0.0	0.0	0.0	0.0	0.0	0.0	0.0
0.0	0.0	0.0	0.0	0.0	0.0	0.0	0.0
0.0	0.0	0.0	0.0	0.0	0.0	0.0	0.0
0.0	0.0	0.0	0.0	0.0	0.0	0.0	0.0
0.0	0.0	0.0	0.0	0.0	0.0	0.0	0.0
0.0	0.0	0.0	0.0	0.0	0.0	0.0	0.0
0.0	0.0	0.0	0.0	0.0	0.0	0.0	0.0
0.0	0.0	0.0	0.0	0.0	0.0	0.0	0.0
0.0	0.0	0.0	0.0	0.0	0.0	0.0	0.0
0.0	0.0	0.0	0.0	0.0	0.0	0.0	0.0
0.0	0.0	0.0	0.0	0.0	0.0	0.0	0.0
0.0	0.0	0.0	0.0	0.0	0.0	0.0	0.0
0.0	0.0	0.0	0.0	0.0	0.0	0.0	0.0
0.0	0.0	0.0	0.0	0.0	0.0	0.0	0.0
0.0	0.0	0.0	0.0	0.0	0.0	0.0	0.0
0.0	0.0	0.0	0.0	0.0	0.0	0.0	0.0
0.0	0.0	0.0	0.0	0.0	0.0	0.0	0.0
0.0	0.0	0.0	0.0	0.0	0.0	0.0	0.0
0.0	0.0	0.0	0.0	0.0	0.0	0.0	0.0
0.0	0.0	0.0	0.0	0.0	0.0	0.0	0.0
0.0	0.0	0.0	0.0	0.0	0.0	0.0	0.0
0.0	0.0	0.0	0.0	0.0	0.0	0.0	0.0
0.0	0.0	0.0	0.0	0.0	0.0	0.0	0.0
0.0	0.0	0.0	0.0	0.0	0.0	0.0	0.0
0.0	0.0	0.0	0.0	0.0	0.0	0.0	0.0
0.0	0.0	0.0	0.0	0.0	0.0	0.0	0.0
0.0	0.0	0.0	0.0	0.0	0.0	0.0	0.0
0.0	0.0	0.0	0.0	0.0	0.0	0.0	0.0
0.0	0.0	0.0	0.0	0.0	0.0	0.0	0.0
0.0	0.0	0.0	0.0	0.0	0.0	0.0	0.0
0.0	0.0	0.0	0.0	0.0	0.0	0.0	0.0
0.0	0.0	0.0	0.0	0.0	0.0	0.0	0.0

0.0	0.0	0.0	0.0	0.0	0.0	0.0	0.0
0.0	0.0	0.0	0.0	0.0	0.0	0.0	0.0
0.0	0.0	0.0	0.0	0.0	0.0	0.0	0.0
0.0	0.0	0.0	0.0	0.0	0.0	0.0	0.0
0.0	0.0	0.0	0.0	0.0	0.0	0.0	0.0
0.0	0.0	0.0	0.0	0.0	0.0	0.0	0.0
0.0	0.0	0.0	0.0	0.0	0.0	0.0	0.0
0.0	0.0	0.0	0.0	0.0	0.0	0.0	0.0
0.0	0.0	0.0	0.0	0.0	0.0	0.0	0.0
0.0	0.0	0.0	0.0	0.0	0.0	0.0	0.0
0.0	0.0	0.0	0.0	0.0	0.0	0.0	0.0
0.0	0.0	0.0	0.0	0.0	0.0	0.0	0.0
0.0	0.0	0.0	0.0	0.0	0.0	0.0	0.0
0.0	0.0	0.0	0.0	0.0	0.0	0.0	0.0
0.0	0.0	0.0	0.0	0.0	0.0	0.0	0.0
0.0	0.0	0.0	0.0	0.0	0.0	0.0	0.0
0.0	0.0	0.0	0.0	0.0	0.0	0.0	0.0
0.0	0.0	0.0	0.0	0.0	0.0	0.0	0.0
0.0	0.0	0.0	0.0	0.0	0.0	0.0	0.0
0.0	0.0	0.0	0.0	0.0	0.0	0.0	0.0
0.0	0.0	0.0	0.0	0.0	0.0	0.0	0.0
0.0	0.0	0.0	0.0	0.0	0.0	0.0	0.0
0.0	0.0	0.0	0.0	0.0	0.0	0.0	0.0
0.0	0.0	0.0	0.0	0.0	0.0	0.0	0.0
0.0	0.0	0.0	0.0	0.0	0.0	0.0	0.0
0.0	0.0	0.0	0.0	0.0	0.0	0.0	0.0
0.0	0.0	0.0	0.0	0.0	0.0	0.0	0.0
0.0	0.0	0.0	0.0	0.0	0.0	0.0	0.0
0.0	0.0	0.0	0.0	0.0	0.0	0.0	0.0
0.0	0.0	0.0	0.0	0.0	0.0	0.0	0.0
0.0	0.0	0.0	0.0	0.0	0.0	0.0	0.0
0.0	0.0	0.0	0.0	0.0	0.0	0.0	0.0
0.0	0.0	0.0	0.0	0.0	0.0	0.0	0.0
0.0	0.0	0.0	0.0	0.0	0.0	0.0	0.0
0.0	0.0	0.0	0.0	0.0	0.0	0.0	0.0
0.0	0.0	0.0	0.0	0.0	0.0	0.0	0.0
0.0	0.0	0.0	0.0	0.0	0.0	0.0	0.0
0.0	0.0	0.0	0.0	0.0	0.0	0.0	0.0
0.0	0.0	0.0	0.0	0.0	0.0	0.0	0.0
0.0	0.0	0.0	0.0	0.0	0.0	0.0	0.0
0.0	0.0	0.0	0.0	0.0	0.0	0.0	0.0
0.0	0.0	0.0	0.0	0.0	0.0	0.0	0.0
0.0	0.0	0.0	0.0	0.0	0.0	0.0	0.0
0.0	0.0	0.0	0.0	0.0	0.0	0.0	0.0
0.0	0.0	0.0	0.0	0.0	0.0	0.0	0.0
0.0	0.0	0.0	0.0	0.0	0.0	0.0	0.0
0.0	0.0	0.0	0.0	0.0	0.0	0.0	0.0
0.0	0.0	0.0	0.0	0.0	0.0	0.0	0.0

0.0	0.0	0.0	0.0	0.0	0.0	0.0	0.0
0.0	0.0	0.0	0.0	0.0	0.0	0.0	0.0
0.0	0.0	0.0	0.0	0.0	0.0	0.0	0.0
0.0	0.0	0.0	0.0	0.0	0.0	0.0	0.0
0.0	0.0	0.0	0.0	0.0	0.0	0.0	0.0
0.0	0.0	0.0	0.0	0.0	0.0	0.0	0.0
0.0	0.0	0.0	0.0	0.0	0.0	0.0	0.0
0.0	0.0	0.0	0.0	0.0	0.0	0.0	0.0
0.0	0.0	0.0	0.0	0.0	0.0	0.0	0.0
0.0	0.0	0.0	0.0	0.0	0.0	0.0	0.0
0.0	0.0	0.0	0.0	0.0	0.0	0.0	0.0
0.0	0.0	0.0	0.0	0.0	0.0	0.0	0.0
0.0	0.0	0.0	0.0	0.0	0.0	0.0	0.0
0.0	0.0	0.0	0.0	0.0	0.0	0.0	0.0
0.0	0.0	0.0	0.0	0.0	0.0	0.0	0.0
0.0	0.0	0.0	0.0	0.0	0.0	0.0	0.0
0.0	0.0	0.0	0.0	0.0	0.0	0.0	0.0
0.0	0.0	0.0	0.0	0.0	0.0	0.0	0.0
0.0	0.0	0.0	0.0	0.0	0.0	0.0	0.0
0.0	0.0	0.0	0.0	0.0	0.0	0.0	0.0
0.0	0.0	0.0	0.0	0.0	0.0	0.0	0.0
0.0	0.0	0.0	0.0	0.0	0.0	0.0	0.0
0.0	0.0	0.0	0.0	0.0	0.0	0.0	0.0
0.0	0.0	0.0	0.0	0.0	0.0	0.0	0.0
0.0	0.0	0.0	0.0	0.0	0.0	0.0	0.0
0.0	0.0	0.0	0.0	0.0	0.0	0.0	0.0
0.0	0.0	0.0	0.0	0.0	0.0	0.0	0.0
0.0	0.0	0.0	0.0	0.0	0.0	0.0	0.0
0.0	0.0	0.0	0.0	0.0	0.0	0.0	0.0
0.0	0.0	0.0	0.0	0.0	0.0	0.0	0.0
0.0	0.0	0.0	0.0	0.0	0.0	0.0	0.0
0.0	0.0	0.0	0.0	0.0	0.0	0.0	0.0
0.0	0.0	0.0	0.0	0.0	0.0	0.0	0.0
0.0	0.0	0.0	0.0	0.0	0.0	0.0	0.0
0.0	0.0	0.0	0.0	0.0	0.0	0.0	0.0
0.0	0.0	0.0	0.0	0.0	0.0	0.0	0.0
0.0	0.0	0.0	0.0	0.0	0.0	0.0	0.0
0.0	0.0	0.0	0.0	0.0	0.0	0.0	0.0
0.0	0.0	0.0	0.0	0.0	0.0	0.0	0.0
0.0	0.0	0.0	0.0	0.0	0.0	0.0	0.0
0.0	0.0	0.0	0.0	0.0	0.0	0.0	0.0
0.0	0.0	0.0	0.0	0.0	0.0	0.0	0.0
0.0	0.0	0.0	0.0	0.0	0.0	0.0	0.0
0.0	0.0	0.0	0.0	0.0	0.0	0.0	0.0
0.0	0.0	0.0	0.0	0.0	0.0	0.0	0.0
0.0	0.0	0.0	0.0	0.0	0.0	0.0	0.0
0.0	0.0	0.0	0.0	0.0	0.0	0.0	0.0

0.0	0.0	0.0	0.0	0.0	0.0	0.0	0.0
0.0	0.0	0.0	0.0	0.0	0.0	0.0	0.0
0.0	0.0	0.0	0.0	0.0	0.0	0.0	0.0
0.0	0.0	0.0	0.0	0.0	0.0	0.0	0.0
0.0	0.0	0.0	0.0	0.0	0.0	0.0	0.0
0.0	0.0	0.0	0.0	0.0	0.0	0.0	0.0
0.0	0.0	0.0	0.0	0.0	0.0	0.0	0.0
0.0	0.0	0.0	0.0	0.0	0.0	0.0	0.0
0.0	0.0	0.0	0.0	0.0	0.0	0.0	0.0
0.0	0.0	0.0	0.0	0.0	0.0	0.0	0.0
0.0	0.0	0.0	0.0	0.0	0.0	0.0	0.0
0.0	0.0	0.0	0.0	0.0	0.0	0.0	0.0
0.0	0.0	0.0	0.0	0.0	0.0	0.0	0.0
0.0	0.0	0.0	0.0	0.0	0.0	0.0	0.0
0.0	0.0	0.0	0.0	0.0	0.0	0.0	0.0
0.0	0.0	0.0	0.0	0.0	0.0	0.0	0.0
0.0	0.0	0.0	0.0	0.0	0.0	0.0	0.0
0.0	0.0	0.0	0.0	0.0	0.0	0.0	0.0
0.0	0.0	0.0	0.0	0.0	0.0	0.0	0.0
0.0	0.0	0.0	0.0	0.0	0.0	0.0	0.0
0.0	0.0	0.0	0.0	0.0	0.0	0.0	0.0
0.0	0.0	0.0	0.0	0.0	0.0	0.0	0.0
0.0	0.0	0.0	0.0	0.0	0.0	0.0	0.0
0.0	0.0	0.0	0.0	0.0	0.0	0.0	0.0
0.0	0.0	0.0	0.0	0.0	0.0	0.0	0.0
0.0	0.0	0.0	0.0	0.0	0.0	0.0	0.0
0.0	0.0	0.0	0.0	0.0	0.0	0.0	0.0
0.0	0.0	0.0	0.0	0.0	0.0	0.0	0.0
0.0	0.0	0.0	0.0	0.0	0.0	0.0	0.0
0.0	0.0	0.0	0.0	0.0	0.0	0.0	0.0
0.0	0.0	0.0	0.0	0.0	0.0	0.0	0.0
0.0	0.0	0.0	0.0	0.0	0.0	0.0	0.0
0.0	0.0	0.0	0.0	0.0	0.0	0.0	0.0
0.0	0.0	0.0	0.0	0.0	0.0	0.0	0.0
0.0	0.0	0.0	0.0	0.0	0.0	0.0	0.0
0.0	0.0	0.0	0.0	0.0	0.0	0.0	0.0
0.0	0.0	0.0	0.0	0.0	0.0	0.0	0.0
0.0	0.0	0.0	0.0	0.0	0.0	0.0	0.0
0.0	0.0	0.0	0.0	0.0	0.0	0.0	0.0
0.0	0.0	0.0	0.0	0.0	0.0	0.0	0.0
0.0	0.0	0.0	0.0	0.0	0.0	0.0	0.0
0.0	0.0	0.0	0.0	0.0	0.0	0.0	0.0
0.0	0.0	0.0	0.0	0.0	0.0	0.0	0.0
0.0	0.0	0.0	0.0	0.0	0.0	0.0	0.0
0.0	0.0	0.0	0.0	0.0	0.0	0.0	0.0
0.0	0.0	0.0	0.0	0.0	0.0	0.0	0.0
0.0	0.0	0.0	0.0	0.0	0.0	0.0	0.0
0.0	0.0	0.0	0.0	0.0	0.0	0.0	0.0

0.0	0.0	0.0	0.0	0.0	0.0	0.0	0.0
0.0	0.0	0.0	0.0	0.0	0.0	0.0	0.0
0.0	0.0	0.0	0.0	0.0	0.0	0.0	0.0
0.0	0.0	0.0	0.0	0.0	0.0	0.0	0.0
0.0	0.0	0.0	0.0	0.0	0.0	0.0	0.0
0.0	0.0	0.0	0.0	0.0	0.0	0.0	0.0
0.0	0.0	0.0	0.0	0.0	0.0	0.0	0.0
0.0	0.0	0.0	0.0	0.0	0.0	0.0	0.0
0.0	0.0	0.0	0.0	0.0	0.0	0.0	0.0
0.0	0.0	0.0	0.0	0.0	0.0	0.0	0.0
0.0	0.0	0.0	0.0	0.0	0.0	0.0	0.0
0.0	0.0	0.0	0.0	0.0	0.0	0.0	0.0
0.0	0.0	0.0	0.0	0.0	0.0	0.0	0.0
0.0	0.0	0.0	0.0	0.0	0.0	0.0	0.0
0.0	0.0	0.0	0.0	0.0	0.0	0.0	0.0
0.0	0.0	0.0	0.0	0.0	0.0	0.0	0.0
0.0	0.0	0.0	0.0	0.0	0.0	0.0	0.0
0.0	0.0	0.0	0.0	0.0	0.0	0.0	0.0
0.0	0.0	0.0	0.0	0.0	0.0	0.0	0.0
0.0	0.0	0.0	0.0	0.0	0.0	0.0	0.0
0.0	0.0	0.0	0.0	0.0	0.0	0.0	0.0
0.0	0.0	0.0	0.0	0.0	0.0	0.0	0.0
0.0	0.0	0.0	0.0	0.0	0.0	0.0	0.0
0.0	0.0	0.0	0.0	0.0	0.0	0.0	0.0
0.0	0.0	0.0	0.0	0.0	0.0	0.0	0.0
0.0	0.0	0.0	0.0	0.0	0.0	0.0	0.0
0.0	0.0	0.0	0.0	0.0	0.0	0.0	0.0
0.0	0.0	0.0	0.0	0.0	0.0	0.0	0.0
0.0	0.0	0.0	0.0	0.0	0.0	0.0	0.0
0.0	0.0	0.0	0.0	0.0	0.0	0.0	0.0
0.0	0.0	0.0	0.0	0.0	0.0	0.0	0.0
0.0	0.0	0.0	0.0	0.0	0.0	0.0	0.0
0.0	0.0	0.0	0.0	0.0	0.0	0.0	0.0
0.0	0.0	0.0	0.0	0.0	0.0	0.0	0.0
0.0	0.0	0.0	0.0	0.0	0.0	0.0	0.0
0.0	0.0	0.0	0.0	0.0	0.0	0.0	0.0
0.0	0.0	0.0	0.0	0.0	0.0	0.0	0.0
0.0	0.0	0.0	0.0	0.0	0.0	0.0	0.0
0.0	0.0	0.0	0.0	0.0	0.0	0.0	0.0
0.0	0.0	0.0	0.0	0.0	0.0	0.0	0.0
0.0	0.0	0.0	0.0	0.0	0.0	0.0	0.0
0.0	0.0	0.0	0.0	0.0	0.0	0.0	0.0
0.0	0.0	0.0	0.0	0.0	0.0	0.0	0.0
0.0	0.0	0.0	0.0	0.0	0.0	0.0	0.0
0.0	0.0	0.0	0.0	0.0	0.0	0.0	0.0
0.0	0.0	0.0	0.0	0.0	0.0	0.0	0.0
0.0	0.0	0.0	0.0	0.0	0.0	0.0	0.0

0.0	0.0	0.0	0.0	0.0	0.0	0.0	0.0
0.0	0.0	0.0	0.0	0.0	0.0	0.0	0.0
0.0	0.0	0.0	0.0	0.0	0.0	0.0	0.0
0.0	0.0	0.0	0.0	0.0	0.0	0.0	0.0
0.0	0.0	0.0	0.0	0.0	0.0	0.0	0.0
0.0	0.0	0.0	0.0	0.0	0.0	0.0	0.0
0.0	0.0	0.0	0.0	0.0	0.0	0.0	0.0
0.0	0.0	0.0	0.0	0.0	0.0	0.0	0.0
0.0	0.0	0.0	0.0	0.0	0.0	0.0	0.0
0.0	0.0	0.0	0.0	0.0	0.0	0.0	0.0
0.0	0.0	0.0	0.0	0.0	0.0	0.0	0.0
0.0	0.0	0.0	0.0	0.0	0.0	0.0	0.0
0.0	0.0	0.0	0.0	0.0	0.0	0.0	0.0
0.0	0.0	0.0	0.0	0.0	0.0	0.0	0.0
0.0	0.0	0.0	0.0	0.0	0.0	0.0	0.0
0.0	0.0	0.0	0.0	0.0	0.0	0.0	0.0
0.0	0.0	0.0	0.0	0.0	0.0	0.0	0.0
0.0	0.0	0.0	0.0	0.0	0.0	0.0	0.0
0.0	0.0	0.0	0.0	0.0	0.0	0.0	0.0
0.0	0.0	0.0	0.0	0.0	0.0	0.0	0.0
0.0	0.0	0.0	0.0	0.0	0.0	0.0	0.0
0.0	0.0	0.0	0.0	0.0	0.0	0.0	0.0
0.0	0.0	0.0	0.0	0.0	0.0	0.0	0.0
0.0	0.0	0.0	0.0	0.0	0.0	0.0	0.0
0.0	0.0	0.0	0.0	0.0	0.0	0.0	0.0
0.0	0.0	0.0	0.0	0.0	0.0	0.0	0.0
0.0	0.0	0.0	0.0	0.0	0.0	0.0	0.0
0.0	0.0	0.0	0.0	0.0	0.0	0.0	0.0
0.0	0.0	0.0	0.0	0.0	0.0	0.0	0.0
0.0	0.0	0.0	0.0	0.0	0.0	0.0	0.0
0.0	0.0	0.0	0.0	0.0	0.0	0.0	0.0
0.0	0.0	0.0	0.0	0.0	0.0	0.0	0.0
0.0	0.0	0.0	0.0	0.0	0.0	0.0	0.0
0.0	0.0	0.0	0.0	0.0	0.0	0.0	0.0
0.0	0.0	0.0	0.0	0.0	0.0	0.0	0.0
0.0	0.0	0.0	0.0	0.0	0.0	0.0	0.0
0.0	0.0	0.0	0.0	0.0	0.0	0.0	0.0
0.0	0.0	0.0	0.0	0.0	0.0	0.0	0.0
0.0	0.0	0.0	0.0	0.0	0.0	0.0	0.0
0.0	0.0	0.0	0.0	0.0	0.0	0.0	0.0
0.0	0.0	0.0	0.0	0.0	0.0	0.0	0.0
0.0	0.0	0.0	0.0	0.0	0.0	0.0	0.0
0.0	0.0	0.0	0.0	0.0	0.0	0.0	0.0
0.0	0.0	0.0	0.0	0.0	0.0	0.0	0.0
0.0	0.0	0.0	0.0	0.0	0.0	0.0	0.0
0.0	0.0	0.0	0.0	0.0	0.0	0.0	0.0
0.0	0.0	0.0	0.0	0.0	0.0	0.0	0.0

0.0	0.0	0.0	0.0	0.0	0.0	0.0	0.0
0.0	0.0	0.0	0.0	0.0	0.0	0.0	0.0
0.0	0.0	0.0	0.0	0.0	0.0	0.0	0.0
0.0	0.0	0.0	0.0	0.0	0.0	0.0	0.0
0.0	0.0	0.0	0.0	0.0	0.0	0.0	0.0
0.0	0.0	0.0	0.0	0.0	0.0	0.0	0.0
0.0	0.0	0.0	0.0	0.0	0.0	0.0	0.0
0.0	0.0	0.0	0.0	0.0	0.0	0.0	0.0
0.0	0.0	0.0	0.0	0.0	0.0	0.0	0.0
0.0	0.0	0.0	0.0	0.0	0.0	0.0	0.0
0.0	0.0	0.0	0.0	0.0	0.0	0.0	0.0
0.0	0.0	0.0	0.0	0.0	0.0	0.0	0.0
0.0	0.0	0.0	0.0	0.0	0.0	0.0	0.0
0.0	0.0	0.0	0.0	0.0	0.0	0.0	0.0
0.0	0.0	0.0	0.0	0.0	0.0	0.0	0.0
0.0	0.0	0.0	0.0	0.0	0.0	0.0	0.0
0.0	0.0	0.0	0.0	0.0	0.0	0.0	0.0
0.0	0.0	0.0	0.0	0.0	0.0	0.0	0.0
0.0	0.0	0.0	0.0	0.0	0.0	0.0	0.0
0.0	0.0	0.0	0.0	0.0	0.0	0.0	0.0
0.0	0.0	0.0	0.0	0.0	0.0	0.0	0.0
0.0	0.0	0.0	0.0	0.0	0.0	0.0	0.0
0.0	0.0	0.0	0.0	0.0	0.0	0.0	0.0
0.0	0.0	0.0	0.0	0.0	0.0	0.0	0.0
0.0	0.0	0.0	0.0	0.0	0.0	0.0	0.0
0.0	0.0	0.0	0.0	0.0	0.0	0.0	0.0
0.0	0.0	0.0	0.0	0.0	0.0	0.0	0.0
0.0	0.0	0.0	0.0	0.0	0.0	0.0	0.0
0.0	0.0	0.0	0.0	0.0	0.0	0.0	0.0
0.0	0.0	0.0	0.0	0.0	0.0	0.0	0.0
0.0	0.0	0.0	0.0	0.0	0.0	0.0	0.0
0.0	0.0	0.0	0.0	0.0	0.0	0.0	0.0
0.0	0.0	0.0	0.0	0.0	0.0	0.0	0.0
0.0	0.0	0.0	0.0	0.0	0.0	0.0	0.0
0.0	0.0	0.0	0.0	0.0	0.0	0.0	0.0
0.0	0.0	0.0	0.0	0.0	0.0	0.0	0.0
0.0	0.0	0.0	0.0	0.0	0.0	0.0	0.0
0.0	0.0	0.0	0.0	0.0	0.0	0.0	0.0
0.0	0.0	0.0	0.0	0.0	0.0	0.0	0.0
0.0	0.0	0.0	0.0	0.0	0.0	0.0	0.0
0.0	0.0	0.0	0.0	0.0	0.0	0.0	0.0
0.0	0.0	0.0	0.0	0.0	0.0	0.0	0.0
0.0	0.0	0.0	0.0	0.0	0.0	0.0	0.0
0.0	0.0	0.0	0.0	0.0	0.0	0.0	0.0
0.0	0.0	0.0	0.0	0.0	0.0	0.0	0.0
0.0	0.0	0.0	0.0	0.0	0.0	0.0	0.0
0.0	0.0	0.0	0.0	0.0	0.0	0.0	0.0

0.0	0.0	0.0	0.0	0.0	0.0	0.0	0.0
0.0	0.0	0.0	0.0	0.0	0.0	0.0	0.0
0.0	0.0	0.0	0.0	0.0	0.0	0.0	0.0
0.0	0.0	0.0	0.0	0.0	0.0	0.0	0.0
0.0	0.0	0.0	0.0	0.0	0.0	0.0	0.0
0.0	0.0	0.0	0.0	0.0	0.0	0.0	0.0
0.0	0.0	0.0	0.0	0.0	0.0	0.0	0.0
0.0	0.0	0.0	0.0	0.0	0.0	0.0	0.0
0.0	0.0	0.0	0.0	0.0	0.0	0.0	0.0
0.0	0.0	0.0	0.0	0.0	0.0	0.0	0.0
0.0	0.0	0.0	0.0	0.0	0.0	0.0	0.0
0.0	0.0	0.0	0.0	0.0	0.0	0.0	0.0
0.0	0.0	0.0	0.0	0.0	0.0	0.0	0.0
0.0	0.0	0.0	0.0	0.0	0.0	0.0	0.0
0.0	0.0	0.0	0.0	0.0	0.0	0.0	0.0
0.0	0.0	0.0	0.0	0.0	0.0	0.0	0.0
0.0	0.0	0.0	0.0	0.0	0.0	0.0	0.0
0.0	0.0	0.0	0.0	0.0	0.0	0.0	0.0
0.0	0.0	0.0	0.0	0.0	0.0	0.0	0.0
0.0	0.0	0.0	0.0	0.0	0.0	0.0	0.0
0.0	0.0	0.0	0.0	0.0	0.0	0.0	0.0
0.0	0.0	0.0	0.0	0.0	0.0	0.0	0.0
0.0	0.0	0.0	0.0	0.0	0.0	0.0	0.0
0.0	0.0	0.0	0.0	0.0	0.0	0.0	0.0
0.0	0.0	0.0	0.0	0.0	0.0	0.0	0.0
0.0	0.0	0.0	0.0	0.0	0.0	0.0	0.0
0.0	0.0	0.0	0.0	0.0	0.0	0.0	0.0
0.0	0.0	0.0	0.0	0.0	0.0	0.0	0.0
0.0	0.0	0.0	0.0	0.0	0.0	0.0	0.0
0.0	0.0	0.0	0.0	0.0	0.0	0.0	0.0
0.0	0.0	0.0	0.0	0.0	0.0	0.0	0.0
0.0	0.0	0.0	0.0	0.0	0.0	0.0	0.0
0.0	0.0	0.0	0.0	0.0	0.0	0.0	0.0
0.0	0.0	0.0	0.0	0.0	0.0	0.0	0.0
0.0	0.0	0.0	0.0	0.0	0.0	0.0	0.0
0.0	0.0	0.0	0.0	0.0	0.0	0.0	0.0
0.0	0.0	0.0	0.0	0.0	0.0	0.0	0.0
0.0	0.0	0.0	0.0	0.0	0.0	0.0	0.0
0.0	0.0	0.0	0.0	0.0	0.0	0.0	0.0
0.0	0.0	0.0	0.0	0.0	0.0	0.0	0.0
0.0	0.0	0.0	0.0	0.0	0.0	0.0	0.0
0.0	0.0	0.0	0.0	0.0	0.0	0.0	0.0
0.0	0.0	0.0	0.0	0.0	0.0	0.0	0.0
0.0	0.0	0.0	0.0	0.0	0.0	0.0	0.0
0.0	0.0	0.0	0.0	0.0	0.0	0.0	0.0
0.0	0.0	0.0	0.0	0.0	0.0	0.0	0.0
0.0	0.0	0.0	0.0	0.0	0.0	0.0	0.0

0.0	0.0	0.0	0.0	0.0	0.0	0.0	0.0
0.0	0.0	0.0	0.0	0.0	0.0	0.0	0.0
0.0	0.0	0.0	0.0	0.0	0.0	0.0	0.0
0.0	0.0	0.0	0.0	0.0	0.0	0.0	0.0
0.0	0.0	0.0	0.0	0.0	0.0	0.0	0.0
0.0	0.0	0.0	0.0	0.0	0.0	0.0	0.0
0.0	0.0	0.0	0.0	0.0	0.0	0.0	0.0
0.0	0.0	0.0	0.0	0.0	0.0	0.0	0.0
0.0	0.0	0.0	0.0	0.0	0.0	0.0	0.0
0.0	0.0	0.0	0.0	0.0	0.0	0.0	0.0
0.0	0.0	0.0	0.0	0.0	0.0	0.0	0.0
0.0	0.0	0.0	0.0	0.0	0.0	0.0	0.0
0.0	0.0	0.0	0.0	0.0	0.0	0.0	0.0
0.0	0.0	0.0	0.0	0.0	0.0	0.0	0.0
0.0	0.0	0.0	0.0	0.0	0.0	0.0	0.0
0.0	0.0	0.0	0.0	0.0	0.0	0.0	0.0
0.0	0.0	0.0	0.0	0.0	0.0	0.0	0.0
0.0	0.0	0.0	0.0	0.0	0.0	0.0	0.0
0.0	0.0	0.0	0.0	0.0	0.0	0.0	0.0
0.0	0.0	0.0	0.0	0.0	0.0	0.0	0.0
0.0	0.0	0.0	0.0	0.0	0.0	0.0	0.0
0.0	0.0	0.0	0.0	0.0	0.0	0.0	0.0
0.0	0.0	0.0	0.0	0.0	0.0	0.0	0.0
0.0	0.0	0.0	0.0	0.0	0.0	0.0	0.0
0.0	0.0	0.0	0.0	0.0	0.0	0.0	0.0
0.0	0.0	0.0	0.0	0.0	0.0	0.0	0.0
0.0	0.0	0.0	0.0	0.0	0.0	0.0	0.0
0.0	0.0	0.0	0.0	0.0	0.0	0.0	0.0
0.0	0.0	0.0	0.0	0.0	0.0	0.0	0.0
0.0	0.0	0.0	0.0	0.0	0.0	0.0	0.0
0.0	0.0	0.0	0.0	0.0	0.0	0.0	0.0
0.0	0.0	0.0	0.0	0.0	0.0	0.0	0.0
0.0	0.0	0.0	0.0	0.0	0.0	0.0	0.0
0.0	0.0	0.0	0.0	0.0	0.0	0.0	0.0
0.0	0.0	0.0	0.0	0.0	0.0	0.0	0.0
0.0	0.0	0.0	0.0	0.0	0.0	0.0	0.0
0.0	0.0	0.0	0.0	0.0	0.0	0.0	0.0
0.0	0.0	0.0	0.0	0.0	0.0	0.0	0.0
0.0	0.0	0.0	0.0	0.0	0.0	0.0	0.0
0.0	0.0	0.0	0.0	0.0	0.0	0.0	0.0
0.0	0.0	0.0	0.0	0.0	0.0	0.0	0.0
0.0	0.0	0.0	0.0	0.0	0.0	0.0	0.0
0.0	0.0	0.0	0.0	0.0	0.0	0.0	0.0
0.0	0.0	0.0	0.0	0.0	0.0	0.0	0.0
0.0	0.0	0.0	0.0	0.0	0.0	0.0	0.0
0.0	0.0	0.0	0.0	0.0	0.0	0.0	0.0
0.0	0.0	0.0	0.0	0.0	0.0	0.0	0.0

0.0	0.0	0.0	0.0	0.0	0.0	0.0	0.0
0.0	0.0	0.0	0.0	0.0	0.0	0.0	0.0
0.0	0.0	0.0	0.0	0.0	0.0	0.0	0.0
0.0	0.0	0.0	0.0	0.0	0.0	0.0	0.0
0.0	0.0	0.0	0.0	0.0	0.0	0.0	0.0
0.0	0.0	0.0	0.0	0.0	0.0	0.0	0.0
0.0	0.0	0.0	0.0	0.0	0.0	0.0	0.0
0.0	0.0	0.0	0.0	0.0	0.0	0.0	0.0
0.0	0.0	0.0	0.0	0.0	0.0	0.0	0.0
0.0	0.0	0.0	0.0	0.0	0.0	0.0	0.0
0.0	0.0	0.0	0.0	0.0	0.0	0.0	0.0
0.0	0.0	0.0	0.0	0.0	0.0	0.0	0.0
0.0	0.0	0.0	0.0	0.0	0.0	0.0	0.0
0.0	0.0	0.0	0.0	0.0	0.0	0.0	0.0
0.0	0.0	0.0	0.0	0.0	0.0	0.0	0.0
0.0	0.0	0.0	0.0	0.0	0.0	0.0	0.0
0.0	0.0	0.0	0.0	0.0	0.0	0.0	0.0
0.0	0.0	0.0	0.0	0.0	0.0	0.0	0.0
0.0	0.0	0.0	0.0	0.0	0.0	0.0	0.0
0.0	0.0	0.0	0.0	0.0	0.0	0.0	0.0
0.0	0.0	0.0	0.0	0.0	0.0	0.0	0.0
0.0	0.0	0.0	0.0	0.0	0.0	0.0	0.0
0.0	0.0	0.0	0.0	0.0	0.0	0.0	0.0
0.0	0.0	0.0	0.0	0.0	0.0	0.0	0.0
0.0	0.0	0.0	0.0	0.0	0.0	0.0	0.0
0.0	0.0	0.0	0.0	0.0	0.0	0.0	0.0
0.0	0.0	0.0	0.0	0.0	0.0	0.0	0.0
0.0	0.0	0.0	0.0	0.0	0.0	0.0	0.0
0.0	0.0	0.0	0.0	0.0	0.0	0.0	0.0
0.0	0.0	0.0	0.0	0.0	0.0	0.0	0.0
0.0	0.0	0.0	0.0	0.0	0.0	0.0	0.0
0.0	0.0	0.0	0.0	0.0	0.0	0.0	0.0
0.0	0.0	0.0	0.0	0.0	0.0	0.0	0.0
0.0	0.0	0.0	0.0	0.0	0.0	0.0	0.0
0.0	0.0	0.0	0.0	0.0	0.0	0.0	0.0
0.0	0.0	0.0	0.0	0.0	0.0	0.0	0.0
0.0	0.0	0.0	0.0	0.0	0.0	0.0	0.0
0.0	0.0	0.0	0.0	0.0	0.0	0.0	0.0
0.0	0.0	0.0	0.0	0.0	0.0	0.0	0.0
0.0	0.0	0.0	0.0	0.0	0.0	0.0	0.0
0.0	0.0	0.0	0.0	0.0	0.0	0.0	0.0
0.0	0.0	0.0	0.0	0.0	0.0	0.0	0.0
0.0	0.0	0.0	0.0	0.0	0.0	0.0	0.0
0.0	0.0	0.0	0.0	0.0	0.0	0.0	0.0
0.0	0.0	0.0	0.0	0.0	0.0	0.0	0.0
0.0	0.0	0.0	0.0	0.0	0.0	0.0	0.0
0.0	0.0	0.0	0.0	0.0	0.0	0.0	0.0

0.0	0.0	0.0	0.0	0.0	0.0	0.0	0.0
0.0	0.0	0.0	0.0	0.0	0.0	0.0	0.0
0.0	0.0	0.0	0.0	0.0	0.0	0.0	0.0
0.0	0.0	0.0	0.0	0.0	0.0	0.0	0.0
0.0	0.0	0.0	0.0	0.0	0.0	0.0	0.0
0.0	0.0	0.0	0.0	0.0	0.0	0.0	0.0
0.0	0.0	0.0	0.0	0.0	0.0	0.0	0.0
0.0	0.0	0.0	0.0	0.0	0.0	0.0	0.0
0.0	0.0	0.0	0.0	0.0	0.0	0.0	0.0
0.0	0.0	0.0	0.0	0.0	0.0	0.0	0.0
0.0	0.0	0.0	0.0	0.0	0.0	0.0	0.0
0.0	0.0	0.0	0.0	0.0	0.0	0.0	0.0
0.0	0.0	0.0	0.0	0.0	0.0	0.0	0.0
0.0	0.0	0.0	0.0	0.0	0.0	0.0	0.0
0.0	0.0	0.0	0.0	0.0	0.0	0.0	0.0
0.0	0.0	0.0	0.0	0.0	0.0	0.0	0.0
0.0	0.0	0.0	0.0	0.0	0.0	0.0	0.0
0.0	0.0	0.0	0.0	0.0	0.0	0.0	0.0
0.0	0.0	0.0	0.0	0.0	0.0	0.0	0.0
0.0	0.0	0.0	0.0	0.0	0.0	0.0	0.0
0.0	0.0	0.0	0.0	0.0	0.0	0.0	0.0
0.0	0.0	0.0	0.0	0.0	0.0	0.0	0.0
0.0	0.0	0.0	0.0	0.0	0.0	0.0	0.0
0.0	0.0	0.0	0.0	0.0	0.0	0.0	0.0
0.0	0.0	0.0	0.0	0.0	0.0	0.0	0.0
0.0	0.0	0.0	0.0	0.0	0.0	0.0	0.0
0.0	0.0	0.0	0.0	0.0	0.0	0.0	0.0
0.0	0.0	0.0	0.0	0.0	0.0	0.0	0.0
0.0	0.0	0.0	0.0	0.0	0.0	0.0	0.0
0.0	0.0	0.0	0.0	0.0	0.0	0.0	0.0
0.0	0.0	0.0	0.0	0.0	0.0	0.0	0.0
0.0	0.0	0.0	0.0	0.0	0.0	0.0	0.0
0.0	0.0	0.0	0.0	0.0	0.0	0.0	0.0
0.0	0.0	0.0	0.0	0.0	0.0	0.0	0.0
0.0	0.0	0.0	0.0	0.0	0.0	0.0	0.0
0.0	0.0	0.0	0.0	0.0	0.0	0.0	0.0
0.0	0.0	0.0	0.0	0.0	0.0	0.0	0.0
0.0	0.0	0.0	0.0	0.0	0.0	0.0	0.0
0.0	0.0	0.0	0.0	0.0	0.0	0.0	0.0
0.0	0.0	0.0	0.0	0.0	0.0	0.0	0.0
0.0	0.0	0.0	0.0	0.0	0.0	0.0	0.0
0.0	0.0	0.0	0.0	0.0	0.0	0.0	0.0
0.0	0.0	0.0	0.0	0.0	0.0	0.0	0.0
0.0	0.0	0.0	0.0	0.0	0.0	0.0	0.0
0.0	0.0	0.0	0.0	0.0	0.0	0.0	0.0
0.0	0.0	0.0	0.0	0.0	0.0	0.0	0.0
0.0	0.0	0.0	0.0	0.0	0.0	0.0	0.0

0.0	0.0	0.0	0.0	0.0	0.0	0.0	0.0
0.0	0.0	0.0	0.0	0.0	0.0	0.0	0.0
0.0	0.0	0.0	0.0	0.0	0.0	0.0	0.0
0.0	0.0	0.0	0.0	0.0	0.0	0.0	0.0
0.0	0.0	0.0	0.0	0.0	0.0	0.0	0.0
0.0	0.0	0.0	0.0	0.0	0.0	0.0	0.0
0.0	0.0	0.0	0.0	0.0	0.0	0.0	0.0
0.0	0.0	0.0	0.0	0.0	0.0	0.0	0.0
0.0	0.0	0.0	0.0	0.0	0.0	0.0	0.0
0.0	0.0	0.0	0.0	0.0	0.0	0.0	0.0
0.0	0.0	0.0	0.0	0.0	0.0	0.0	0.0
0.0	0.0	0.0	0.0	0.0	0.0	0.0	0.0
0.0	0.0	0.0	0.0	0.0	0.0	0.0	0.0
0.0	0.0	0.0	0.0	0.0	0.0	0.0	0.0
0.0	0.0	0.0	0.0	0.0	0.0	0.0	0.0
0.0	0.0	0.0	0.0	0.0	0.0	0.0	0.0
0.0	0.0	0.0	0.0	0.0	0.0	0.0	0.0
0.0	0.0	0.0	0.0	0.0	0.0	0.0	0.0
0.0	0.0	0.0	0.0	0.0	0.0	0.0	0.0
0.0	0.0	0.0	0.0	0.0	0.0	0.0	0.0
0.0	0.0	0.0	0.0	0.0	0.0	0.0	0.0
0.0	0.0	0.0	0.0	0.0	0.0	0.0	0.0
0.0	0.0	0.0	0.0	0.0	0.0	0.0	0.0
0.0	0.0	0.0	0.0	0.0	0.0	0.0	0.0
0.0	0.0	0.0	0.0	0.0	0.0	0.0	0.0
0.0	0.0	0.0	0.0	0.0	0.0	0.0	0.0
0.0	0.0	0.0	0.0	0.0	0.0	0.0	0.0
0.0	0.0	0.0	0.0	0.0	0.0	0.0	0.0
0.0	0.0	0.0	0.0	0.0	0.0	0.0	0.0
0.0	0.0	0.0	0.0	0.0	0.0	0.0	0.0
0.0	0.0	0.0	0.0	0.0	0.0	0.0	0.0
0.0	0.0	0.0	0.0	0.0	0.0	0.0	0.0
0.0	0.0	0.0	0.0	0.0	0.0	0.0	0.0
0.0	0.0	0.0	0.0	0.0	0.0	0.0	0.0
0.0	0.0	0.0	0.0	0.0	0.0	0.0	0.0
0.0	0.0	0.0	0.0	0.0	0.0	0.0	0.0
0.0	0.0	0.0	0.0	0.0	0.0	0.0	0.0
0.0	0.0	0.0	0.0	0.0	0.0	0.0	0.0
0.0	0.0	0.0	0.0	0.0	0.0	0.0	0.0
0.0	0.0	0.0	0.0	0.0	0.0	0.0	0.0
0.0	0.0	0.0	0.0	0.0	0.0	0.0	0.0
0.0	0.0	0.0	0.0	0.0	0.0	0.0	0.0
0.0	0.0	0.0	0.0	0.0	0.0	0.0	0.0
0.0	0.0	0.0	0.0	0.0	0.0	0.0	0.0
0.0	0.0	0.0	0.0	0.0	0.0	0.0	0.0
0.0	0.0	0.0	0.0	0.0	0.0	0.0	0.0
0.0	0.0	0.0	0.0	0.0	0.0	0.0	0.0

0.0	0.0	0.0	0.0	0.0	0.0	0.0	0.0
0.0	0.0	0.0	0.0	0.0	0.0	0.0	0.0
0.0	0.0	0.0	0.0	0.0	0.0	0.0	0.0
0.0	0.0	0.0	0.0	0.0	0.0	0.0	0.0
0.0	0.0	0.0	0.0	0.0	0.0	0.0	0.0
0.0	0.0	0.0	0.0	0.0	0.0	0.0	0.0
0.0	0.0	0.0	0.0	0.0	0.0	0.0	0.0
0.0	0.0	0.0	0.0	0.0	0.0	0.0	0.0
0.0	0.0	0.0	0.0	0.0	0.0	0.0	0.0
0.0	0.0	0.0	0.0	0.0	0.0	0.0	0.0
0.0	0.0	0.0	0.0	0.0	0.0	0.0	0.0
0.0	0.0	0.0	0.0	0.0	0.0	0.0	0.0
0.0	0.0	0.0	0.0	0.0	0.0	0.0	0.0
0.0	0.0	0.0	0.0	0.0	0.0	0.0	0.0
0.0	0.0	0.0	0.0	0.0	0.0	0.0	0.0
0.0	0.0	0.0	0.0	0.0	0.0	0.0	0.0
0.0	0.0	0.0	0.0	0.0	0.0	0.0	0.0
0.0	0.0	0.0	0.0	0.0	0.0	0.0	0.0
0.0	0.0	0.0	0.0	0.0	0.0	0.0	0.0
0.0	0.0	0.0	0.0	0.0	0.0	0.0	0.0
0.0	0.0	0.0	0.0	0.0	0.0	0.0	0.0
0.0	0.0	0.0	0.0	0.0	0.0	0.0	0.0
0.0	0.0	0.0	0.0	0.0	0.0	0.0	0.0
0.0	0.0	0.0	0.0	0.0	0.0	0.0	0.0
0.0	0.0	0.0	0.0	0.0	0.0	0.0	0.0
0.0	0.0	0.0	0.0	0.0	0.0	0.0	0.0
0.0	0.0	0.0	0.0	0.0	0.0	0.0	0.0
0.0	0.0	0.0	0.0	0.0	0.0	0.0	0.0
0.0	0.0	0.0	0.0	0.0	0.0	0.0	0.0
0.0	0.0	0.0	0.0	0.0	0.0	0.0	0.0
0.0	0.0	0.0	0.0	0.0	0.0	0.0	0.0
0.0	0.0	0.0	0.0	0.0	0.0	0.0	0.0
0.0	0.0	0.0	0.0	0.0	0.0	0.0	0.0
0.0	0.0	0.0	0.0	0.0	0.0	0.0	0.0
0.0	0.0	0.0	0.0	0.0	0.0	0.0	0.0
0.0	0.0	0.0	0.0	0.0	0.0	0.0	0.0
0.0	0.0	0.0	0.0	0.0	0.0	0.0	0.0
0.0	0.0	0.0	0.0	0.0	0.0	0.0	0.0
0.0	0.0	0.0	0.0	0.0	0.0	0.0	0.0
0.0	0.0	0.0	0.0	0.0	0.0	0.0	0.0
0.0	0.0	0.0	0.0	0.0	0.0	0.0	0.0
0.0	0.0	0.0	0.0	0.0	0.0	0.0	0.0
0.0	0.0	0.0	0.0	0.0	0.0	0.0	0.0
0.0	0.0	0.0	0.0	0.0	0.0	0.0	0.0
0.0	0.0	0.0	0.0	0.0	0.0	0.0	0.0
0.0	0.0	0.0	0.0	0.0	0.0	0.0	0.0
0.0	0.0	0.0	0.0	0.0	0.0	0.0	0.0

0.0	0.0	0.0	0.0	0.0	0.0	0.0	0.0
0.0	0.0	0.0	0.0	0.0	0.0	0.0	0.0
0.0	0.0	0.0	0.0	0.0	0.0	0.0	0.0
0.0	0.0	0.0	0.0	0.0	0.0	0.0	0.0
0.0	0.0	0.0	0.0	0.0	0.0	0.0	0.0
0.0	0.0	0.0	0.0	0.0	0.0	0.0	0.0
0.0	0.0	0.0	0.0	0.0	0.0	0.0	0.0
0.0	0.0	0.0	0.0	0.0	0.0	0.0	0.0
0.0	0.0	0.0	0.0	0.0	0.0	0.0	0.0
0.0	0.0	0.0	0.0	0.0	0.0	0.0	0.0
0.0	0.0	0.0	0.0	0.0	0.0	0.0	0.0
0.0	0.0	0.0	0.0	0.0	0.0	0.0	0.0
0.0	0.0	0.0	0.0	0.0	0.0	0.0	0.0
0.0	0.0	0.0	0.0	0.0	0.0	0.0	0.0
0.0	0.0	0.0	0.0	0.0	0.0	0.0	0.0
0.0	0.0	0.0	0.0	0.0	0.0	0.0	0.0
0.0	0.0	0.0	0.0	0.0	0.0	0.0	0.0
0.0	0.0	0.0	0.0	0.0	0.0	0.0	0.0
0.0	0.0	0.0	0.0	0.0	0.0	0.0	0.0
0.0	0.0	0.0	0.0	0.0	0.0	0.0	0.0
0.0	0.0	0.0	0.0	0.0	0.0	0.0	0.0
0.0	0.0	0.0	0.0	0.0	0.0	0.0	0.0
0.0	0.0	0.0	0.0	0.0	0.0	0.0	0.0
0.0	0.0	0.0	0.0	0.0	0.0	0.0	0.0
0.0	0.0	0.0	0.0	0.0	0.0	0.0	0.0
0.0	0.0	0.0	0.0	0.0	0.0	0.0	0.0
0.0	0.0	0.0	0.0	0.0	0.0	0.0	0.0
0.0	0.0	0.0	0.0	0.0	0.0	0.0	0.0
0.0	0.0	0.0	0.0	0.0	0.0	0.0	0.0
0.0	0.0	0.0	0.0	0.0	0.0	0.0	0.0
0.0	0.0	0.0	0.0	0.0	0.0	0.0	0.0
0.0	0.0	0.0	0.0	0.0	0.0	0.0	0.0
0.0	0.0	0.0	0.0	0.0	0.0	0.0	0.0
0.0	0.0	0.0	0.0	0.0	0.0	0.0	0.0
0.0	0.0	0.0	0.0	0.0	0.0	0.0	0.0
0.0	0.0	0.0	0.0	0.0	0.0	0.0	0.0
0.0	0.0	0.0	0.0	0.0	0.0	0.0	0.0
0.0	0.0	0.0	0.0	0.0	0.0	0.0	0.0
0.0	0.0	0.0	0.0	0.0	0.0	0.0	0.0
0.0	0.0	0.0	0.0	0.0	0.0	0.0	0.0
0.0	0.0	0.0	0.0	0.0	0.0	0.0	0.0
0.0	0.0	0.0	0.0	0.0	0.0	0.0	0.0
0.0	0.0	0.0	0.0	0.0	0.0	0.0	0.0
0.0	0.0	0.0	0.0	0.0	0.0	0.0	0.0
0.0	0.0	0.0	0.0	0.0	0.0	0.0	0.0
0.0	0.0	0.0	0.0	0.0	0.0	0.0	0.0
0.0	0.0	0.0	0.0	0.0	0.0	0.0	0.0

0.0	0.0	0.0	0.0	0.0	0.0	0.0	0.0
0.0	0.0	0.0	0.0	0.0	0.0	0.0	0.0
0.0	0.0	0.0	0.0	0.0	0.0	0.0	0.0
0.0	0.0	0.0	0.0	0.0	0.0	0.0	0.0
0.0	0.0	0.0	0.0	0.0	0.0	0.0	0.0
0.0	0.0	0.0	0.0	0.0	0.0	0.0	0.0
0.0	0.0	0.0	0.0	0.0	0.0	0.0	0.0
0.0	0.0	0.0	0.0	0.0	0.0	0.0	0.0
0.0	0.0	0.0	0.0	0.0	0.0	0.0	0.0
0.0	0.0	0.0	0.0	0.0	0.0	0.0	0.0
0.0	0.0	0.0	0.0	0.0	0.0	0.0	0.0
0.0	0.0	0.0	0.0	0.0	0.0	0.0	0.0
0.0	0.0	0.0	0.0	0.0	0.0	0.0	0.0
0.0	0.0	0.0	0.0	0.0	0.0	0.0	0.0
0.0	0.0	0.0	0.0	0.0	0.0	0.0	0.0
0.0	0.0	0.0	0.0	0.0	0.0	0.0	0.0
0.0	0.0	0.0	0.0	0.0	0.0	0.0	0.0
0.0	0.0	0.0	0.0	0.0	0.0	0.0	0.0
0.0	0.0	0.0	0.0	0.0	0.0	0.0	0.0
0.0	0.0	0.0	0.0	0.0	0.0	0.0	0.0
0.0	0.0	0.0	0.0	0.0	0.0	0.0	0.0
0.0	0.0	0.0	0.0	0.0	0.0	0.0	0.0
0.0	0.0	0.0	0.0	0.0	0.0	0.0	0.0
0.0	0.0	0.0	0.0	0.0	0.0	0.0	0.0
0.0	0.0	0.0	0.0	0.0	0.0	0.0	0.0
0.0	0.0	0.0	0.0	0.0	0.0	0.0	0.0
0.0	0.0	0.0	0.0	0.0	0.0	0.0	0.0
0.0	0.0	0.0	0.0	0.0	0.0	0.0	0.0
0.0	0.0	0.0	0.0	0.0	0.0	0.0	0.0
0.0	0.0	0.0	0.0	0.0	0.0	0.0	0.0
0.0	0.0	0.0	0.0	0.0	0.0	0.0	0.0
0.0	0.0	0.0	0.0	0.0	0.0	0.0	0.0
0.0	0.0	0.0	0.0	0.0	0.0	0.0	0.0
0.0	0.0	0.0	0.0	0.0	0.0	0.0	0.0
0.0	0.0	0.0	0.0	0.0	0.0	0.0	0.0
0.0	0.0	0.0	0.0	0.0	0.0	0.0	0.0
0.0	0.0	0.0	0.0	0.0	0.0	0.0	0.0
0.0	0.0	0.0	0.0	0.0	0.0	0.0	0.0
0.0	0.0	0.0	0.0	0.0	0.0	0.0	0.0
0.0	0.0	0.0	0.0	0.0	0.0	0.0	0.0
0.0	0.0	0.0	0.0	0.0	0.0	0.0	0.0
0.0	0.0	0.0	0.0	0.0	0.0	0.0	0.0
0.0	0.0	0.0	0.0	0.0	0.0	0.0	0.0
0.0	0.0	0.0	0.0	0.0	0.0	0.0	0.0
0.0	0.0	0.0	0.0	0.0	0.0	0.0	0.0
0.0	0.0	0.0	0.0	0.0	0.0	0.0	0.0
0.0	0.0	0.0	0.0	0.0	0.0	0.0	0.0

0.0	0.0	0.0	0.0	0.0	0.0	0.0	0.0
0.0	0.0	0.0	0.0	0.0	0.0	0.0	0.0
0.0	0.0	0.0	0.0	0.0	0.0	0.0	0.0
0.0	0.0	0.0	0.0	0.0	0.0	0.0	0.0
0.0	0.0	0.0	0.0	0.0	0.0	0.0	0.0
0.0	0.0	0.0	0.0	0.0	0.0	0.0	0.0
0.0	0.0	0.0	0.0	0.0	0.0	0.0	0.0
0.0	0.0	0.0	0.0	0.0	0.0	0.0	0.0
0.0	0.0	0.0	0.0	0.0	0.0	0.0	0.0
0.0	0.0	0.0	0.0	0.0	0.0	0.0	0.0
0.0	0.0	0.0	0.0	0.0	0.0	0.0	0.0
0.0	0.0	0.0	0.0	0.0	0.0	0.0	0.0
0.0	0.0	0.0	0.0	0.0	0.0	0.0	0.0
0.0	0.0	0.0	0.0	0.0	0.0	0.0	0.0
0.0	0.0	0.0	0.0	0.0	0.0	0.0	0.0
0.0	0.0	0.0	0.0	0.0	0.0	0.0	0.0
0.0	0.0	0.0	0.0	0.0	0.0	0.0	0.0
0.0	0.0	0.0	0.0	0.0	0.0	0.0	0.0
0.0	0.0	0.0	0.0	0.0	0.0	0.0	0.0
0.0	0.0	0.0	0.0	0.0	0.0	0.0	0.0
0.0	0.0	0.0	0.0	0.0	0.0	0.0	0.0
0.0	0.0	0.0	0.0	0.0	0.0	0.0	0.0
0.0	0.0	0.0	0.0	0.0	0.0	0.0	0.0
0.0	0.0	0.0	0.0	0.0	0.0	0.0	0.0
0.0	0.0	0.0	0.0	0.0	0.0	0.0	0.0
0.0	0.0	0.0	0.0	0.0	0.0	0.0	0.0
0.0	0.0	0.0	0.0	0.0	0.0	0.0	0.0
0.0	0.0	0.0	0.0	0.0	0.0	0.0	0.0
0.0	0.0	0.0	0.0	0.0	0.0	0.0	0.0
0.0	0.0	0.0	0.0	0.0	0.0	0.0	0.0
0.0	0.0	0.0	0.0	0.0	0.0	0.0	0.0
0.0	0.0	0.0	0.0	0.0	0.0	0.0	0.0
0.0	0.0	0.0	0.0	0.0	0.0	0.0	0.0
0.0	0.0	0.0	0.0	0.0	0.0	0.0	0.0
0.0	0.0	0.0	0.0	0.0	0.0	0.0	0.0
0.0	0.0	0.0	0.0	0.0	0.0	0.0	0.0
0.0	0.0	0.0	0.0	0.0	0.0	0.0	0.0
0.0	0.0	0.0	0.0	0.0	0.0	0.0	0.0
0.0	0.0	0.0	0.0	0.0	0.0	0.0	0.0
0.0	0.0	0.0	0.0	0.0	0.0	0.0	0.0
0.0	0.0	0.0	0.0	0.0	0.0	0.0	0.0
0.0	0.0	0.0	0.0	0.0	0.0	0.0	0.0
0.0	0.0	0.0	0.0	0.0	0.0	0.0	0.0
0.0	0.0	0.0	0.0	0.0	0.0	0.0	0.0
0.0	0.0	0.0	0.0	0.0	0.0	0.0	0.0
0.0	0.0	0.0	0.0	0.0	0.0	0.0	0.0
0.0	0.0	0.0	0.0	0.0	0.0	0.0	0.0

0.0	0.0	0.0	0.0	0.0	0.0	0.0	0.0
0.0	0.0	0.0	0.0	0.0	0.0	0.0	0.0
0.0	0.0	0.0	0.0	0.0	0.0	0.0	0.0
0.0	0.0	0.0	0.0	0.0	0.0	0.0	0.0
0.0	0.0	0.0	0.0	0.0	0.0	0.0	0.0
0.0	0.0	0.0	0.0	0.0	0.0	0.0	0.0
0.0	0.0	0.0	0.0	0.0	0.0	0.0	0.0
0.0	0.0	0.0	0.0	0.0	0.0	0.0	0.0
0.0	0.0	0.0	0.0	0.0	0.0	0.0	0.0
0.0	0.0	0.0	0.0	0.0	0.0	0.0	0.0
0.0	0.0	0.0	0.0	0.0	0.0	0.0	0.0
0.0	0.0	0.0	0.0	0.0	0.0	0.0	0.0
0.0	0.0	0.0	0.0	0.0	0.0	0.0	0.0
0.0	0.0	0.0	0.0	0.0	0.0	0.0	0.0
0.0	0.0	0.0	0.0	0.0	0.0	0.0	0.0
0.0	0.0	0.0	0.0	0.0	0.0	0.0	0.0
0.0	0.0	0.0	0.0	0.0	0.0	0.0	0.0
0.0	0.0	0.0	0.0	0.0	0.0	0.0	0.0
0.0	0.0	0.0	0.0	0.0	0.0	0.0	0.0
0.0	0.0	0.0	0.0	0.0	0.0	0.0	0.0
0.0	0.0	0.0	0.0	0.0	0.0	0.0	0.0
0.0	0.0	0.0	0.0	0.0	0.0	0.0	0.0
0.0	0.0	0.0	0.0	0.0	0.0	0.0	0.0
0.0	0.0	0.0	0.0	0.0	0.0	0.0	0.0
0.0	0.0	0.0	0.0	0.0	0.0	0.0	0.0
0.0	0.0	0.0	0.0	0.0	0.0	0.0	0.0
0.0	0.0	0.0	0.0	0.0	0.0	0.0	0.0
0.0	0.0	0.0	0.0	0.0	0.0	0.0	0.0
0.0	0.0	0.0	0.0	0.0	0.0	0.0	0.0
0.0	0.0	0.0	0.0	0.0	0.0	0.0	0.0
0.0	0.0	0.0	0.0	0.0	0.0	0.0	0.0
0.0	0.0	0.0	0.0	0.0	0.0	0.0	0.0
0.0	0.0	0.0	0.0	0.0	0.0	0.0	0.0
0.0	0.0	0.0	0.0	0.0	0.0	0.0	0.0
0.0	0.0	0.0	0.0	0.0	0.0	0.0	0.0
0.0	0.0	0.0	0.0	0.0	0.0	0.0	0.0
0.0	0.0	0.0	0.0	0.0	0.0	0.0	0.0
0.0	0.0	0.0	0.0	0.0	0.0	0.0	0.0
0.0	0.0	0.0	0.0	0.0	0.0	0.0	0.0
0.0	0.0	0.0	0.0	0.0	0.0	0.0	0.0
0.0	0.0	0.0	0.0	0.0	0.0	0.0	0.0
0.0	0.0	0.0	0.0	0.0	0.0	0.0	0.0
0.0	0.0	0.0	0.0	0.0	0.0	0.0	0.0
0.0	0.0	0.0	0.0	0.0	0.0	0.0	0.0
0.0	0.0	0.0	0.0	0.0	0.0	0.0	0.0
0.0	0.0	0.0	0.0	0.0	0.0	0.0	0.0
0.0	0.0	0.0	0.0	0.0	0.0	0.0	0.0
0.0	0.0	0.0	0.0	0.0	0.0	0.0	0.0

0.0	0.0	0.0	0.0	0.0	0.0	0.0	0.0
0.0	0.0	0.0	0.0	0.0	0.0	0.0	0.0
0.0	0.0	0.0	0.0	0.0	0.0	0.0	0.0
0.0	0.0	0.0	0.0	0.0	0.0	0.0	0.0
0.0	0.0	0.0	0.0	0.0	0.0	0.0	0.0
0.0	0.0	0.0	0.0	0.0	0.0	0.0	0.0
0.0	0.0	0.0	0.0	0.0	0.0	0.0	0.0
0.0	0.0	0.0	0.0	0.0	0.0	0.0	0.0
0.0	0.0	0.0	0.0	0.0	0.0	0.0	0.0
0.0	0.0	0.0	0.0	0.0	0.0	0.0	0.0
0.0	0.0	0.0	0.0	0.0	0.0	0.0	0.0
0.0	0.0	0.0	0.0	0.0	0.0	0.0	0.0
0.0	0.0	0.0	0.0	0.0	0.0	0.0	0.0
0.0	0.0	0.0	0.0	0.0	0.0	0.0	0.0
0.0	0.0	0.0	0.0	0.0	0.0	0.0	0.0
0.0	0.0	0.0	0.0	0.0	0.0	0.0	0.0
0.0	0.0	0.0	0.0	0.0	0.0	0.0	0.0
0.0	0.0	0.0	0.0	0.0	0.0	0.0	0.0
0.0	0.0	0.0	0.0	0.0	0.0	0.0	0.0
0.0	0.0	0.0	0.0	0.0	0.0	0.0	0.0
0.0	0.0	0.0	0.0	0.0	0.0	0.0	0.0
0.0	0.0	0.0	0.0	0.0	0.0	0.0	0.0
0.0	0.0	0.0	0.0	0.0	0.0	0.0	0.0
0.0	0.0	0.0	0.0	0.0	0.0	0.0	0.0
0.0	0.0	0.0	0.0	0.0	0.0	0.0	0.0
0.0	0.0	0.0	0.0	0.0	0.0	0.0	0.0
0.0	0.0	0.0	0.0	0.0	0.0	0.0	0.0
0.0	0.0	0.0	0.0	0.0	0.0	0.0	0.0
0.0	0.0	0.0	0.0	0.0	0.0	0.0	0.0
0.0	0.0	0.0	0.0	0.0	0.0	0.0	0.0
0.0	0.0	0.0	0.0	0.0	0.0	0.0	0.0
0.0	0.0	0.0	0.0	0.0	0.0	0.0	0.0
0.0	0.0	0.0	0.0	0.0	0.0	0.0	0.0
0.0	0.0	0.0	0.0	0.0	0.0	0.0	0.0
0.0	0.0	0.0	0.0	0.0	0.0	0.0	0.0
0.0	0.0	0.0	0.0	0.0	0.0	0.0	0.0
0.0	0.0	0.0	0.0	0.0	0.0	0.0	0.0
0.0	0.0	0.0	0.0	0.0	0.0	0.0	0.0
0.0	0.0	0.0	0.0	0.0	0.0	0.0	0.0
0.0	0.0	0.0	0.0	0.0	0.0	0.0	0.0
0.0	0.0	0.0	0.0	0.0	0.0	0.0	0.0
0.0	0.0	0.0	0.0	0.0	0.0	0.0	0.0
0.0	0.0	0.0	0.0	0.0	0.0	0.0	0.0
0.0	0.0	0.0	0.0	0.0	0.0	0.0	0.0
0.0	0.0	0.0	0.0	0.0	0.0	0.0	0.0
0.0	0.0	0.0	0.0	0.0	0.0	0.0	0.0
0.0	0.0	0.0	0.0	0.0	0.0	0.0	0.0

0.0	0.0	0.0	0.0	0.0	0.0	0.0	0.0
0.0	0.0	0.0	0.0	0.0	0.0	0.0	0.0
0.0	0.0	0.0	0.0	0.0	0.0	0.0	0.0
0.0	0.0	0.0	0.0	0.0	0.0	0.0	0.0
0.0	0.0	0.0	0.0	0.0	0.0	0.0	0.0
0.0	0.0	0.0	0.0	0.0	0.0	0.0	0.0
0.0	0.0	0.0	0.0	0.0	0.0	0.0	0.0
0.0	0.0	0.0	0.0	0.0	0.0	0.0	0.0
0.0	0.0	0.0	0.0	0.0	0.0	0.0	0.0
0.0	0.0	0.0	0.0	0.0	0.0	0.0	0.0
0.0	0.0	0.0	0.0	0.0	0.0	0.0	0.0
0.0	0.0	0.0	0.0	0.0	0.0	0.0	0.0
0.0	0.0	0.0	0.0	0.0	0.0	0.0	0.0
0.0	0.0	0.0	0.0	0.0	0.0	0.0	0.0
0.0	0.0	0.0	0.0	0.0	0.0	0.0	0.0
0.0	0.0	0.0	0.0	0.0	0.0	0.0	0.0
0.0	0.0	0.0	0.0	0.0	0.0	0.0	0.0
0.0	0.0	0.0	0.0	0.0	0.0	0.0	0.0
0.0	0.0	0.0	0.0	0.0	0.0	0.0	0.0
0.0	0.0	0.0	0.0	0.0	0.0	0.0	0.0
0.0	0.0	0.0	0.0	0.0	0.0	0.0	0.0
0.0	0.0	0.0	0.0	0.0	0.0	0.0	0.0
0.0	0.0	0.0	0.0	0.0	0.0	0.0	0.0
0.0	0.0	0.0	0.0	0.0	0.0	0.0	0.0
0.0	0.0	0.0	0.0	0.0	0.0	0.0	0.0
0.0	0.0	0.0	0.0	0.0	0.0	0.0	0.0
0.0	0.0	0.0	0.0	0.0	0.0	0.0	0.0
0.0	0.0	0.0	0.0	0.0	0.0	0.0	0.0
0.0	0.0	0.0	0.0	0.0	0.0	0.0	0.0
0.0	0.0	0.0	0.0	0.0	0.0	0.0	0.0
0.0	0.0	0.0	0.0	0.0	0.0	0.0	0.0
0.0	0.0	0.0	0.0	0.0	0.0	0.0	0.0
0.0	0.0	0.0	0.0	0.0	0.0	0.0	0.0
0.0	0.0	0.0	0.0	0.0	0.0	0.0	0.0
0.0	0.0	0.0	0.0	0.0	0.0	0.0	0.0
0.0	0.0	0.0	0.0	0.0	0.0	0.0	0.0
0.0	0.0	0.0	0.0	0.0	0.0	0.0	0.0
0.0	0.0	0.0	0.0	0.0	0.0	0.0	0.0
0.0	0.0	0.0	0.0	0.0	0.0	0.0	0.0
0.0	0.0	0.0	0.0	0.0	0.0	0.0	0.0
0.0	0.0	0.0	0.0	0.0	0.0	0.0	0.0
0.0	0.0	0.0	0.0	0.0	0.0	0.0	0.0
0.0	0.0	0.0	0.0	0.0	0.0	0.0	0.0
0.0	0.0	0.0	0.0	0.0	0.0	0.0	0.0
0.0	0.0	0.0	0.0	0.0	0.0	0.0	0.0
0.0	0.0	0.0	0.0	0.0	0.0	0.0	0.0
0.0	0.0	0.0	0.0	0.0	0.0	0.0	0.0

0.0	0.0	0.0	0.0	0.0	0.0	0.0	0.0
0.0	0.0	0.0	0.0	0.0	0.0	0.0	0.0
0.0	0.0	0.0	0.0	0.0	0.0	0.0	0.0
0.0	0.0	0.0	0.0	0.0	0.0	0.0	0.0
0.0	0.0	0.0	0.0	0.0	0.0	0.0	0.0
0.0	0.0	0.0	0.0	0.0	0.0	0.0	0.0
0.0	0.0	0.0	0.0	0.0	0.0	0.0	0.0
0.0	0.0	0.0	0.0	0.0	0.0	0.0	0.0
0.0	0.0	0.0	0.0	0.0	0.0	0.0	0.0
0.0	0.0	0.0	0.0	0.0	0.0	0.0	0.0
0.0	0.0	0.0	0.0	0.0	0.0	0.0	0.0
0.0	0.0	0.0	0.0	0.0	0.0	0.0	0.0
0.0	0.0	0.0	0.0	0.0	0.0	0.0	0.0
0.0	0.0	0.0	0.0	0.0	0.0	0.0	0.0
0.0	0.0	0.0	0.0	0.0	0.0	0.0	0.0
0.0	0.0	0.0	0.0	0.0	0.0	0.0	0.0
0.0	0.0	0.0	0.0	0.0	0.0	0.0	0.0
0.0	0.0	0.0	0.0	0.0	0.0	0.0	0.0
0.0	0.0	0.0	0.0	0.0	0.0	0.0	0.0
0.0	0.0	0.0	0.0	0.0	0.0	0.0	0.0
0.0	0.0	0.0	0.0	0.0	0.0	0.0	0.0
0.0	0.0	0.0	0.0	0.0	0.0	0.0	0.0
0.0	0.0	0.0	0.0	0.0	0.0	0.0	0.0
0.0	0.0	0.0	0.0	0.0	0.0	0.0	0.0
0.0	0.0	0.0	0.0	0.0	0.0	0.0	0.0
0.0	0.0	0.0	0.0	0.0	0.0	0.0	0.0
0.0	0.0	0.0	0.0	0.0	0.0	0.0	0.0
0.0	0.0	0.0	0.0	0.0	0.0	0.0	0.0
0.0	0.0	0.0	0.0	0.0	0.0	0.0	0.0
0.0	0.0	0.0	0.0	0.0	0.0	0.0	0.0
0.0	0.0	0.0	0.0	0.0	0.0	0.0	0.0
0.0	0.0	0.0	0.0	0.0	0.0	0.0	0.0
0.0	0.0	0.0	0.0	0.0	0.0	0.0	0.0
0.0	0.0	0.0	0.0	0.0	0.0	0.0	0.0
0.0	0.0	0.0	0.0	0.0	0.0	0.0	0.0
0.0	0.0	0.0	0.0	0.0	0.0	0.0	0.0
0.0	0.0	0.0	0.0	0.0	0.0	0.0	0.0
0.0	0.0	0.0	0.0	0.0	0.0	0.0	0.0
0.0	0.0	0.0	0.0	0.0	0.0	0.0	0.0
0.0	0.0	0.0	0.0	0.0	0.0	0.0	0.0
0.0	0.0	0.0	0.0	0.0	0.0	0.0	0.0
0.0	0.0	0.0	0.0	0.0	0.0	0.0	0.0
0.0	0.0	0.0	0.0	0.0	0.0	0.0	0.0
0.0	0.0	0.0	0.0	0.0	0.0	0.0	0.0
0.0	0.0	0.0	0.0	0.0	0.0	0.0	0.0
0.0	0.0	0.0	0.0	0.0	0.0	0.0	0.0
0.0	0.0	0.0	0.0	0.0	0.0	0.0	0.0

0.0	0.0	0.0	0.0	0.0	0.0	0.0	0.0
0.0	0.0	0.0	0.0	0.0	0.0	0.0	0.0
0.0	0.0	0.0	0.0	0.0	0.0	0.0	0.0
0.0	0.0	0.0	0.0	0.0	0.0	0.0	0.0
0.0	0.0	0.0	0.0	0.0	0.0	0.0	0.0
0.0	0.0	0.0	0.0	0.0	0.0	0.0	0.0
0.0	0.0	0.0	0.0	0.0	0.0	0.0	0.0
0.0	0.0	0.0	0.0	0.0	0.0	0.0	0.0
0.0	0.0	0.0	0.0	0.0	0.0	0.0	0.0
0.0	0.0	0.0	0.0	0.0	0.0	0.0	0.0
0.0	0.0	0.0	0.0	0.0	0.0	0.0	0.0
0.0	0.0	0.0	0.0	0.0	0.0	0.0	0.0
0.0	0.0	0.0	0.0	0.0	0.0	0.0	0.0
0.0	0.0	0.0	0.0	0.0	0.0	0.0	0.0
0.0	0.0	0.0	0.0	0.0	0.0	0.0	0.0
0.0	0.0	0.0	0.0	0.0	0.0	0.0	0.0
0.0	0.0	0.0	0.0	0.0	0.0	0.0	0.0
0.0	0.0	0.0	0.0	0.0	0.0	0.0	0.0
0.0	0.0	0.0	0.0	0.0	0.0	0.0	0.0
0.0	0.0	0.0	0.0	0.0	0.0	0.0	0.0
0.0	0.0	0.0	0.0	0.0	0.0	0.0	0.0
0.0	0.0	0.0	0.0	0.0	0.0	0.0	0.0
0.0	0.0	0.0	0.0	0.0	0.0	0.0	0.0
0.0	0.0	0.0	0.0	0.0	0.0	0.0	0.0
0.0	0.0	0.0	0.0	0.0	0.0	0.0	0.0
0.0	0.0	0.0	0.0	0.0	0.0	0.0	0.0
0.0	0.0	0.0	0.0	0.0	0.0	0.0	0.0
0.0	0.0	0.0	0.0	0.0	0.0	0.0	0.0
0.0	0.0	0.0	0.0	0.0	0.0	0.0	0.0
0.0	0.0	0.0	0.0	0.0	0.0	0.0	0.0
0.0	0.0	0.0	0.0	0.0	0.0	0.0	0.0
0.0	0.0	0.0	0.0	0.0	0.0	0.0	0.0
0.0	0.0	0.0	0.0	0.0	0.0	0.0	0.0
0.0	0.0	0.0	0.0	0.0	0.0	0.0	0.0
0.0	0.0	0.0	0.0	0.0	0.0	0.0	0.0
0.0	0.0	0.0	0.0	0.0	0.0	0.0	0.0
0.0	0.0	0.0	0.0	0.0	0.0	0.0	0.0
0.0	0.0	0.0	0.0	0.0	0.0	0.0	0.0
0.0	0.0	0.0	0.0	0.0	0.0	0.0	0.0
0.0	0.0	0.0	0.0	0.0	0.0	0.0	0.0
0.0	0.0	0.0	0.0	0.0	0.0	0.0	0.0
0.0	0.0	0.0	0.0	0.0	0.0	0.0	0.0
0.0	0.0	0.0	0.0	0.0	0.0	0.0	0.0
0.0	0.0	0.0	0.0	0.0	0.0	0.0	0.0
0.0	0.0	0.0	0.0	0.0	0.0	0.0	0.0
0.0	0.0	0.0	0.0	0.0	0.0	0.0	0.0
0.0	0.0	0.0	0.0	0.0	0.0	0.0	0.0
0.0	0.0	0.0	0.0	0.0	0.0	0.0	0.0

0.0	0.0	0.0	0.0	0.0	0.0	0.0	0.0
0.0	0.0	0.0	0.0	0.0	0.0	0.0	0.0
0.0	0.0	0.0	0.0	0.0	0.0	0.0	0.0
0.0	0.0	0.0	0.0	0.0	0.0	0.0	0.0
0.0	0.0	0.0	0.0	0.0	0.0	0.0	0.0
0.0	0.0	0.0	0.0	0.0	0.0	0.0	0.0
0.0	0.0	0.0	0.0	0.0	0.0	0.0	0.0
0.0	0.0	0.0	0.0	0.0	0.0	0.0	0.0
0.0	0.0	0.0	0.0	0.0	0.0	0.0	0.0
0.0	0.0	0.0	0.0	0.0	0.0	0.0	0.0
0.0	0.0	0.0	0.0	0.0	0.0	0.0	0.0
0.0	0.0	0.0	0.0	0.0	0.0	0.0	0.0
0.0	0.0	0.0	0.0	0.0	0.0	0.0	0.0
0.0	0.0	0.0	0.0	0.0	0.0	0.0	0.0
0.0	0.0	0.0	0.0	0.0	0.0	0.0	0.0
0.0	0.0	0.0	0.0	0.0	0.0	0.0	0.0
0.0	0.0	0.0	0.0	0.0	0.0	0.0	0.0
0.0	0.0	0.0	0.0	0.0	0.0	0.0	0.0
0.0	0.0	0.0	0.0	0.0	0.0	0.0	0.0
0.0	0.0	0.0	0.0	0.0	0.0	0.0	0.0
0.0	0.0	0.0	0.0	0.0	0.0	0.0	0.0
0.0	0.0	0.0	0.0	0.0	0.0	0.0	0.0
0.0	0.0	0.0	0.0	0.0	0.0	0.0	0.0
0.0	0.0	0.0	0.0	0.0	0.0	0.0	0.0
0.0	0.0	0.0	0.0	0.0	0.0	0.0	0.0
0.0	0.0	0.0	0.0	0.0	0.0	0.0	0.0
0.0	0.0	0.0	0.0	0.0	0.0	0.0	0.0
0.0	0.0	0.0	0.0	0.0	0.0	0.0	0.0
0.0	0.0	0.0	0.0	0.0	0.0	0.0	0.0
0.0	0.0	0.0	0.0	0.0	0.0	0.0	0.0
0.0	0.0	0.0	0.0	0.0	0.0	0.0	0.0
0.0	0.0	0.0	0.0	0.0	0.0	0.0	0.0
0.0	0.0	0.0	0.0	0.0	0.0	0.0	0.0
0.0	0.0	0.0	0.0	0.0	0.0	0.0	0.0
0.0	0.0	0.0	0.0	0.0	0.0	0.0	0.0
0.0	0.0	0.0	0.0	0.0	0.0	0.0	0.0
0.0	0.0	0.0	0.0	0.0	0.0	0.0	0.0
0.0	0.0	0.0	0.0	0.0	0.0	0.0	0.0
0.0	0.0	0.0	0.0	0.0	0.0	0.0	0.0
0.0	0.0	0.0	0.0	0.0	0.0	0.0	0.0
0.0	0.0	0.0	0.0	0.0	0.0	0.0	0.0
0.0	0.0	0.0	0.0	0.0	0.0	0.0	0.0
0.0	0.0	0.0	0.0	0.0	0.0	0.0	0.0
0.0	0.0	0.0	0.0	0.0	0.0	0.0	0.0
0.0	0.0	0.0	0.0	0.0	0.0	0.0	0.0
0.0	0.0	0.0	0.0	0.0	0.0	0.0	0.0
0.0	0.0	0.0	0.0	0.0	0.0	0.0	0.0

0.0	0.0	0.0	0.0	0.0	0.0	0.0	0.0
0.0	0.0	0.0	0.0	0.0	0.0	0.0	0.0
0.0	0.0	0.0	0.0	0.0	0.0	0.0	0.0
0.0	0.0	0.0	0.0	0.0	0.0	0.0	0.0
0.0	0.0	0.0	0.0	0.0	0.0	0.0	0.0
0.0	0.0	0.0	0.0	0.0	0.0	0.0	0.0
0.0	0.0	0.0	0.0	0.0	0.0	0.0	0.0
0.0	0.0	0.0	0.0	0.0	0.0	0.0	0.0
0.0	0.0	0.0	0.0	0.0	0.0	0.0	0.0
0.0	0.0	0.0	0.0	0.0	0.0	0.0	0.0
0.0	0.0	0.0	0.0	0.0	0.0	0.0	0.0
0.0	0.0	0.0	0.0	0.0	0.0	0.0	0.0
0.0	0.0	0.0	0.0	0.0	0.0	0.0	0.0
0.0	0.0	0.0	0.0	0.0	0.0	0.0	0.0
0.0	0.0	0.0	0.0	0.0	0.0	0.0	0.0
0.0	0.0	0.0	0.0	0.0	0.0	0.0	0.0
0.0	0.0	0.0	0.0	0.0	0.0	0.0	0.0
0.0	0.0	0.0	0.0	0.0	0.0	0.0	0.0
0.0	0.0	0.0	0.0	0.0	0.0	0.0	0.0
0.0	0.0	0.0	0.0	0.0	0.0	0.0	0.0
0.0	0.0	0.0	0.0	0.0	0.0	0.0	0.0
0.0	0.0	0.0	0.0	0.0	0.0	0.0	0.0
0.0	0.0	0.0	0.0	0.0	0.0	0.0	0.0
0.0	0.0	0.0	0.0	0.0	0.0	0.0	0.0
0.0	0.0	0.0	0.0	0.0	0.0	0.0	0.0
0.0	0.0	0.0	0.0	0.0	0.0	0.0	0.0
0.0	0.0	0.0	0.0	0.0	0.0	0.0	0.0
0.0	0.0	0.0	0.0	0.0	0.0	0.0	0.0
0.0	0.0	0.0	0.0	0.0	0.0	0.0	0.0
0.0	0.0	0.0	0.0	0.0	0.0	0.0	0.0
0.0	0.0	0.0	0.0	0.0	0.0	0.0	0.0
0.0	0.0	0.0	0.0	0.0	0.0	0.0	0.0
0.0	0.0	0.0	0.0	0.0	0.0	0.0	0.0
0.0	0.0	0.0	0.0	0.0	0.0	0.0	0.0
0.0	0.0	0.0	0.0	0.0	0.0	0.0	0.0
0.0	0.0	0.0	0.0	0.0	0.0	0.0	0.0
0.0	0.0	0.0	0.0	0.0	0.0	0.0	0.0
0.0	0.0	0.0	0.0	0.0	0.0	0.0	0.0
0.0	0.0	0.0	0.0	0.0	0.0	0.0	0.0
0.0	0.0	0.0	0.0	0.0	0.0	0.0	0.0
0.0	0.0	0.0	0.0	0.0	0.0	0.0	0.0
0.0	0.0	0.0	0.0	0.0	0.0	0.0	0.0
0.0	0.0	0.0	0.0	0.0	0.0	0.0	0.0
0.0	0.0	0.0	0.0	0.0	0.0	0.0	0.0
0.0	0.0	0.0	0.0	0.0	0.0	0.0	0.0
0.0	0.0	0.0	0.0	0.0	0.0	0.0	0.0
0.0	0.0	0.0	0.0	0.0	0.0	0.0	0.0

0.0	0.0	0.0	0.0	0.0	0.0	0.0	0.0
0.0	0.0	0.0	0.0	0.0	0.0	0.0	0.0
0.0	0.0	0.0	0.0	0.0	0.0	0.0	0.0
0.0	0.0	0.0	0.0	0.0	0.0	0.0	0.0
0.0	0.0	0.0	0.0	0.0	0.0	0.0	0.0
0.0	0.0	0.0	0.0	0.0	0.0	0.0	0.0
0.0	0.0	0.0	0.0	0.0	0.0	0.0	0.0
0.0	0.0	0.0	0.0	0.0	0.0	0.0	0.0
0.0	0.0	0.0	0.0	0.0	0.0	0.0	0.0
0.0	0.0	0.0	0.0	0.0	0.0	0.0	0.0
0.0	0.0	0.0	0.0	0.0	0.0	0.0	0.0
0.0	0.0	0.0	0.0	0.0	0.0	0.0	0.0
0.0	0.0	0.0	0.0	0.0	0.0	0.0	0.0
0.0	0.0	0.0	0.0	0.0	0.0	0.0	0.0
0.0	0.0	0.0	0.0	0.0	0.0	0.0	0.0
0.0	0.0	0.0	0.0	0.0	0.0	0.0	0.0
0.0	0.0	0.0	0.0	0.0	0.0	0.0	0.0
0.0	0.0	0.0	0.0	0.0	0.0	0.0	0.0
0.0	0.0	0.0	0.0	0.0	0.0	0.0	0.0
0.0	0.0	0.0	0.0	0.0	0.0	0.0	0.0
0.0	0.0	0.0	0.0	0.0	0.0	0.0	0.0
0.0	0.0	0.0	0.0	0.0	0.0	0.0	0.0
0.0	0.0	0.0	0.0	0.0	0.0	0.0	0.0
0.0	0.0	0.0	0.0	0.0	0.0	0.0	0.0
0.0	0.0	0.0	0.0	0.0	0.0	0.0	0.0
0.0	0.0	0.0	0.0	0.0	0.0	0.0	0.0
0.0	0.0	0.0	0.0	0.0	0.0	0.0	0.0
0.0	0.0	0.0	0.0	0.0	0.0	0.0	0.0
0.0	0.0	0.0	0.0	0.0	0.0	0.0	0.0
0.0	0.0	0.0	0.0	0.0	0.0	0.0	0.0
0.0	0.0	0.0	0.0	0.0	0.0	0.0	0.0
0.0	0.0	0.0	0.0	0.0	0.0	0.0	0.0
0.0	0.0	0.0	0.0	0.0	0.0	0.0	0.0
0.0	0.0	0.0	0.0	0.0	0.0	0.0	0.0
0.0	0.0	0.0	0.0	0.0	0.0	0.0	0.0
0.0	0.0	0.0	0.0	0.0	0.0	0.0	0.0
0.0	0.0	0.0	0.0	0.0	0.0	0.0	0.0
0.0	0.0	0.0	0.0	0.0	0.0	0.0	0.0
0.0	0.0	0.0	0.0	0.0	0.0	0.0	0.0
0.0	0.0	0.0	0.0	0.0	0.0	0.0	0.0
0.0	0.0	0.0	0.0	0.0	0.0	0.0	0.0
0.0	0.0	0.0	0.0	0.0	0.0	0.0	0.0
0.0	0.0	0.0	0.0	0.0	0.0	0.0	0.0
0.0	0.0	0.0	0.0	0.0	0.0	0.0	0.0
0.0	0.0	0.0	0.0	0.0	0.0	0.0	0.0
0.0	0.0	0.0	0.0	0.0	0.0	0.0	0.0
0.0	0.0	0.0	0.0	0.0	0.0	0.0	0.0

0.0	0.0	0.0	0.0	0.0	0.0	0.0	0.0
0.0	0.0	0.0	0.0	0.0	0.0	0.0	0.0
0.0	0.0	0.0	0.0	0.0	0.0	0.0	0.0
0.0	0.0	0.0	0.0	0.0	0.0	0.0	0.0
0.0	0.0	0.0	0.0	0.0	0.0	0.0	0.0
0.0	0.0	0.0	0.0	0.0	0.0	0.0	0.0
0.0	0.0	0.0	0.0	0.0	0.0	0.0	0.0
0.0	0.0	0.0	0.0	0.0	0.0	0.0	0.0
0.0	0.0	0.0	0.0	0.0	0.0	0.0	0.0
0.0	0.0	0.0	0.0	0.0	0.0	0.0	0.0
0.0	0.0	0.0	0.0	0.0	0.0	0.0	0.0
0.0	0.0	0.0	0.0	0.0	0.0	0.0	0.0
0.0	0.0	0.0	0.0	0.0	0.0	0.0	0.0
0.0	0.0	0.0	0.0	0.0	0.0	0.0	0.0
0.0	0.0	0.0	0.0	0.0	0.0	0.0	0.0
0.0	0.0	0.0	0.0	0.0	0.0	0.0	0.0
0.0	0.0	0.0	0.0	0.0	0.0	0.0	0.0
0.0	0.0	0.0	0.0	0.0	0.0	0.0	0.0
0.0	0.0	0.0	0.0	0.0	0.0	0.0	0.0
0.0	0.0	0.0	0.0	0.0	0.0	0.0	0.0
0.0	0.0	0.0	0.0	0.0	0.0	0.0	0.0
0.0	0.0	0.0	0.0	0.0	0.0	0.0	0.0
0.0	0.0	0.0	0.0	0.0	0.0	0.0	0.0
0.0	0.0	0.0	0.0	0.0	0.0	0.0	0.0
0.0	0.0	0.0	0.0	0.0	0.0	0.0	0.0
0.0	0.0	0.0	0.0	0.0	0.0	0.0	0.0
0.0	0.0	0.0	0.0	0.0	0.0	0.0	0.0
0.0	0.0	0.0	0.0	0.0	0.0	0.0	0.0
0.0	0.0	0.0	0.0	0.0	0.0	0.0	0.0
0.0	0.0	0.0	0.0	0.0	0.0	0.0	0.0
0.0	0.0	0.0	0.0	0.0	0.0	0.0	0.0
0.0	0.0	0.0	0.0	0.0	0.0	0.0	0.0
0.0	0.0	0.0	0.0	0.0	0.0	0.0	0.0
0.0	0.0	0.0	0.0	0.0	0.0	0.0	0.0
0.0	0.0	0.0	0.0	0.0	0.0	0.0	0.0
0.0	0.0	0.0	0.0	0.0	0.0	0.0	0.0
0.0	0.0	0.0	0.0	0.0	0.0	0.0	0.0
0.0	0.0	0.0	0.0	0.0	0.0	0.0	0.0
0.0	0.0	0.0	0.0	0.0	0.0	0.0	0.0
0.0	0.0	0.0	0.0	0.0	0.0	0.0	0.0
0.0	0.0	0.0	0.0	0.0	0.0	0.0	0.0
0.0	0.0	0.0	0.0	0.0	0.0	0.0	0.0
0.0	0.0	0.0	0.0	0.0	0.0	0.0	0.0
0.0	0.0	0.0	0.0	0.0	0.0	0.0	0.0
0.0	0.0	0.0	0.0	0.0	0.0	0.0	0.0
0.0	0.0	0.0	0.0	0.0	0.0	0.0	0.0
0.0	0.0	0.0	0.0	0.0	0.0	0.0	0.0

0.0	0.0	0.0	0.0	0.0	0.0	0.0	0.0
0.0	0.0	0.0	0.0	0.0	0.0	0.0	0.0
0.0	0.0	0.0	0.0	0.0	0.0	0.0	0.0
0.0	0.0	0.0	0.0	0.0	0.0	0.0	0.0
0.0	0.0	0.0	0.0	0.0	0.0	0.0	0.0
0.0	0.0	0.0	0.0	0.0	0.0	0.0	0.0
0.0	0.0	0.0	0.0	0.0	0.0	0.0	0.0
0.0	0.0	0.0	0.0	0.0	0.0	0.0	0.0
0.0	0.0	0.0	0.0	0.0	0.0	0.0	0.0
0.0	0.0	0.0	0.0	0.0	0.0	0.0	0.0
0.0	0.0	0.0	0.0	0.0	0.0	0.0	0.0
0.0	0.0	0.0	0.0	0.0	0.0	0.0	0.0
0.0	0.0	0.0	0.0	0.0	0.0	0.0	0.0
0.0	0.0	0.0	0.0	0.0	0.0	0.0	0.0
0.0	0.0	0.0	0.0	0.0	0.0	0.0	0.0
0.0	0.0	0.0	0.0	0.0	0.0	0.0	0.0
0.0	0.0	0.0	0.0	0.0	0.0	0.0	0.0
0.0	0.0	0.0	0.0	0.0	0.0	0.0	0.0
0.0	0.0	0.0	0.0	0.0	0.0	0.0	0.0
0.0	0.0	0.0	0.0	0.0	0.0	0.0	0.0
0.0	0.0	0.0	0.0	0.0	0.0	0.0	0.0
0.0	0.0	0.0	0.0	0.0	0.0	0.0	0.0
0.0	0.0	0.0	0.0	0.0	0.0	0.0	0.0
0.0	0.0	0.0	0.0	0.0	0.0	0.0	0.0
0.0	0.0	0.0	0.0	0.0	0.0	0.0	0.0
0.0	0.0	0.0	0.0	0.0	0.0	0.0	0.0
0.0	0.0	0.0	0.0	0.0	0.0	0.0	0.0
0.0	0.0	0.0	0.0	0.0	0.0	0.0	0.0
0.0	0.0	0.0	0.0	0.0	0.0	0.0	0.0
0.0	0.0	0.0	0.0	0.0	0.0	0.0	0.0
0.0	0.0	0.0	0.0	0.0	0.0	0.0	0.0
0.0	0.0	0.0	0.0	0.0	0.0	0.0	0.0
0.0	0.0	0.0	0.0	0.0	0.0	0.0	0.0
0.0	0.0	0.0	0.0	0.0	0.0	0.0	0.0
0.0	0.0	0.0	0.0	0.0	0.0	0.0	0.0
0.0	0.0	0.0	0.0	0.0	0.0	0.0	0.0
0.0	0.0	0.0	0.0	0.0	0.0	0.0	0.0
0.0	0.0	0.0	0.0	0.0	0.0	0.0	0.0
0.0	0.0	0.0	0.0	0.0	0.0	0.0	0.0
0.0	0.0	0.0	0.0	0.0	0.0	0.0	0.0
0.0	0.0	0.0	0.0	0.0	0.0	0.0	0.0
0.0	0.0	0.0	0.0	0.0	0.0	0.0	0.0
0.0	0.0	0.0	0.0	0.0	0.0	0.0	0.0
0.0	0.0	0.0	0.0	0.0	0.0	0.0	0.0
0.0	0.0	0.0	0.0	0.0	0.0	0.0	0.0
0.0	0.0	0.0	0.0	0.0	0.0	0.0	0.0
0.0	0.0	0.0	0.0	0.0	0.0	0.0	0.0

0.0	0.0	0.0	0.0	0.0	0.0	0.0	0.0
0.0	0.0	0.0	0.0	0.0	0.0	0.0	0.0
0.0	0.0	0.0	0.0	0.0	0.0	0.0	0.0
0.0	0.0	0.0	0.0	0.0	0.0	0.0	0.0
0.0	0.0	0.0	0.0	0.0	0.0	0.0	0.0
0.0	0.0	0.0	0.0	0.0	0.0	0.0	0.0
0.0	0.0	0.0	0.0	0.0	0.0	0.0	0.0
0.0	0.0	0.0	0.0	0.0	0.0	0.0	0.0
0.0	0.0	0.0	0.0	0.0	0.0	0.0	0.0
0.0	0.0	0.0	0.0	0.0	0.0	0.0	0.0
0.0	0.0	0.0	0.0	0.0	0.0	0.0	0.0
0.0	0.0	0.0	0.0	0.0	0.0	0.0	0.0
0.0	0.0	0.0	0.0	0.0	0.0	0.0	0.0
0.0	0.0	0.0	0.0	0.0	0.0	0.0	0.0
0.0	0.0	0.0	0.0	0.0	0.0	0.0	0.0
0.0	0.0	0.0	0.0	0.0	0.0	0.0	0.0
0.0	0.0	0.0	0.0	0.0	0.0	0.0	0.0
0.0	0.0	0.0	0.0	0.0	0.0	0.0	0.0
0.0	0.0	0.0	0.0	0.0	0.0	0.0	0.0
0.0	0.0	0.0	0.0	0.0	0.0	0.0	0.0
0.0	0.0	0.0	0.0	0.0	0.0	0.0	0.0
0.0	0.0	0.0	0.0	0.0	0.0	0.0	0.0
0.0	0.0	0.0	0.0	0.0	0.0	0.0	0.0
0.0	0.0	0.0	0.0	0.0	0.0	0.0	0.0
0.0	0.0	0.0	0.0	0.0	0.0	0.0	0.0
0.0	0.0	0.0	0.0	0.0	0.0	0.0	0.0
0.0	0.0	0.0	0.0	0.0	0.0	0.0	0.0
0.0	0.0	0.0	0.0	0.0	0.0	0.0	0.0
0.0	0.0	0.0	0.0	0.0	0.0	0.0	0.0
0.0	0.0	0.0	0.0	0.0	0.0	0.0	0.0
0.0	0.0	0.0	0.0	0.0	0.0	0.0	0.0
0.0	0.0	0.0	0.0	0.0	0.0	0.0	0.0
0.0	0.0	0.0	0.0	0.0	0.0	0.0	0.0
0.0	0.0	0.0	0.0	0.0	0.0	0.0	0.0
0.0	0.0	0.0	0.0	0.0	0.0	0.0	0.0
0.0	0.0	0.0	0.0	0.0	0.0	0.0	0.0
0.0	0.0	0.0	0.0	0.0	0.0	0.0	0.0
0.0	0.0	0.0	0.0	0.0	0.0	0.0	0.0
0.0	0.0	0.0	0.0	0.0	0.0	0.0	0.0
0.0	0.0	0.0	0.0	0.0	0.0	0.0	0.0
0.0	0.0	0.0	0.0	0.0	0.0	0.0	0.0
0.0	0.0	0.0	0.0	0.0	0.0	0.0	0.0
0.0	0.0	0.0	0.0	0.0	0.0	0.0	0.0
0.0	0.0	0.0	0.0	0.0	0.0	0.0	0.0
0.0	0.0	0.0	0.0	0.0	0.0	0.0	0.0
0.0	0.0	0.0	0.0	0.0	0.0	0.0	0.0
0.0	0.0	0.0	0.0	0.0	0.0	0.0	0.0

0.0	0.0	0.0	0.0	0.0	0.0	0.0	0.0
0.0	0.0	0.0	0.0	0.0	0.0	0.0	0.0
0.0	0.0	0.0	0.0	0.0	0.0	0.0	0.0
0.0	0.0	0.0	0.0	0.0	0.0	0.0	0.0
0.0	0.0	0.0	0.0	0.0	0.0	0.0	0.0
0.0	0.0	0.0	0.0	0.0	0.0	0.0	0.0
0.0	0.0	0.0	0.0	0.0	0.0	0.0	0.0
0.0	0.0	0.0	0.0	0.0	0.0	0.0	0.0
0.0	0.0	0.0	0.0	0.0	0.0	0.0	0.0
0.0	0.0	0.0	0.0	0.0	0.0	0.0	0.0
0.0	0.0	0.0	0.0	0.0	0.0	0.0	0.0
0.0	0.0	0.0	0.0	0.0	0.0	0.0	0.0
0.0	0.0	0.0	0.0	0.0	0.0	0.0	0.0
0.0	0.0	0.0	0.0	0.0	0.0	0.0	0.0
0.0	0.0	0.0	0.0	0.0	0.0	0.0	0.0
0.0	0.0	0.0	0.0	0.0	0.0	0.0	0.0
0.0	0.0	0.0	0.0	0.0	0.0	0.0	0.0
0.0	0.0	0.0	0.0	0.0	0.0	0.0	0.0
0.0	0.0	0.0	0.0	0.0	0.0	0.0	0.0
0.0	0.0	0.0	0.0	0.0	0.0	0.0	0.0
0.0	0.0	0.0	0.0	0.0	0.0	0.0	0.0
0.0	0.0	0.0	0.0	0.0	0.0	0.0	0.0
0.0	0.0	0.0	0.0	0.0	0.0	0.0	0.0
0.0	0.0	0.0	0.0	0.0	0.0	0.0	0.0
0.0	0.0	0.0	0.0	0.0	0.0	0.0	0.0
0.0	0.0	0.0	0.0	0.0	0.0	0.0	0.0
0.0	0.0	0.0	0.0	0.0	0.0	0.0	0.0
0.0	0.0	0.0	0.0	0.0	0.0	0.0	0.0
0.0	0.0	0.0	0.0	0.0	0.0	0.0	0.0
0.0	0.0	0.0	0.0	0.0	0.0	0.0	0.0
0.0	0.0	0.0	0.0	0.0	0.0	0.0	0.0
0.0	0.0	0.0	0.0	0.0	0.0	0.0	0.0
0.0	0.0	0.0	0.0	0.0	0.0	0.0	0.0
0.0	0.0	0.0	0.0	0.0	0.0	0.0	0.0
0.0	0.0	0.0	0.0	0.0	0.0	0.0	0.0
0.0	0.0	0.0	0.0	0.0	0.0	0.0	0.0
0.0	0.0	0.0	0.0	0.0	0.0	0.0	0.0
0.0	0.0	0.0	0.0	0.0	0.0	0.0	0.0
0.0	0.0	0.0	0.0	0.0	0.0	0.0	0.0
0.0	0.0	0.0	0.0	0.0	0.0	0.0	0.0
0.0	0.0	0.0	0.0	0.0	0.0	0.0	0.0
0.0	0.0	0.0	0.0	0.0	0.0	0.0	0.0
0.0	0.0	0.0	0.0	0.0	0.0	0.0	0.0
0.0	0.0	0.0	0.0	0.0	0.0	0.0	0.0
0.0	0.0	0.0	0.0	0.0	0.0	0.0	0.0
0.0	0.0	0.0	0.0	0.0	0.0	0.0	0.0
0.0	0.0	0.0	0.0	0.0	0.0	0.0	0.0

0.0	0.0	0.0	0.0	0.0	0.0	0.0	0.0
0.0	0.0	0.0	0.0	0.0	0.0	0.0	0.0
0.0	0.0	0.0	0.0	0.0	0.0	0.0	0.0
0.0	0.0	0.0	0.0	0.0	0.0	0.0	0.0
0.0	0.0	0.0	0.0	0.0	0.0	0.0	0.0
0.0	0.0	0.0	0.0	0.0	0.0	0.0	0.0
0.0	0.0	0.0	0.0	0.0	0.0	0.0	0.0
0.0	0.0	0.0	0.0	0.0	0.0	0.0	0.0
0.0	0.0	0.0	0.0	0.0	0.0	0.0	0.0
0.0	0.0	0.0	0.0	0.0	0.0	0.0	0.0
0.0	0.0	0.0	0.0	0.0	0.0	0.0	0.0
0.0	0.0	0.0	0.0	0.0	0.0	0.0	0.0
0.0	0.0	0.0	0.0	0.0	0.0	0.0	0.0
0.0	0.0	0.0	0.0	0.0	0.0	0.0	0.0
0.0	0.0	0.0	0.0	0.0	0.0	0.0	0.0
0.0	0.0	0.0	0.0	0.0	0.0	0.0	0.0
0.0	0.0	0.0	0.0	0.0	0.0	0.0	0.0
0.0	0.0	0.0	0.0	0.0	0.0	0.0	0.0
0.0	0.0	0.0	0.0	0.0	0.0	0.0	0.0
0.0	0.0	0.0	0.0	0.0	0.0	0.0	0.0
0.0	0.0	0.0	0.0	0.0	0.0	0.0	0.0
0.0	0.0	0.0	0.0	0.0	0.0	0.0	0.0
0.0	0.0	0.0	0.0	0.0	0.0	0.0	0.0
0.0	0.0	0.0	0.0	0.0	0.0	0.0	0.0
0.0	0.0	0.0	0.0	0.0	0.0	0.0	0.0
0.0	0.0	0.0	0.0	0.0	0.0	0.0	0.0
0.0	0.0	0.0	0.0	0.0	0.0	0.0	0.0
0.0	0.0	0.0	0.0	0.0	0.0	0.0	0.0
0.0	0.0	0.0	0.0	0.0	0.0	0.0	0.0
0.0	0.0	0.0	0.0	0.0	0.0	0.0	0.0
0.0	0.0	0.0	0.0	0.0	0.0	0.0	0.0
0.0	0.0	0.0	0.0	0.0	0.0	0.0	0.0
0.0	0.0	0.0	0.0	0.0	0.0	0.0	0.0
0.0	0.0	0.0	0.0	0.0	0.0	0.0	0.0
0.0	0.0	0.0	0.0	0.0	0.0	0.0	0.0
0.0	0.0	0.0	0.0	0.0	0.0	0.0	0.0
0.0	0.0	0.0	0.0	0.0	0.0	0.0	0.0
0.0	0.0	0.0	0.0	0.0	0.0	0.0	0.0
0.0	0.0	0.0	0.0	0.0	0.0	0.0	0.0
0.0	0.0	0.0	0.0	0.0	0.0	0.0	0.0
0.0	0.0	0.0	0.0	0.0	0.0	0.0	0.0
0.0	0.0	0.0	0.0	0.0	0.0	0.0	0.0
0.0	0.0	0.0	0.0	0.0	0.0	0.0	0.0
0.0	0.0	0.0	0.0	0.0	0.0	0.0	0.0
0.0	0.0	0.0	0.0	0.0	0.0	0.0	0.0
0.0	0.0	0.0	0.0	0.0	0.0	0.0	0.0
0.0	0.0	0.0	0.0	0.0	0.0	0.0	0.0

0.0	0.0	0.0	0.0	0.0	0.0	0.0	0.0
0.0	0.0	0.0	0.0	0.0	0.0	0.0	0.0
0.0	0.0	0.0	0.0	0.0	0.0	0.0	0.0
0.0	0.0	0.0	0.0	0.0	0.0	0.0	0.0
0.0	0.0	0.0	0.0	0.0	0.0	0.0	0.0
0.0	0.0	0.0	0.0	0.0	0.0	0.0	0.0
0.0	0.0	0.0	0.0	0.0	0.0	0.0	0.0
0.0	0.0	0.0	0.0	0.0	0.0	0.0	0.0
0.0	0.0	0.0	0.0	0.0	0.0	0.0	0.0
0.0	0.0	0.0	0.0	0.0	0.0	0.0	0.0
0.0	0.0	0.0	0.0	0.0	0.0	0.0	0.0
0.0	0.0	0.0	0.0	0.0	0.0	0.0	0.0
0.0	0.0	0.0	0.0	0.0	0.0	0.0	0.0
0.0	0.0	0.0	0.0	0.0	0.0	0.0	0.0
0.0	0.0	0.0	0.0	0.0	0.0	0.0	0.0
0.0	0.0	0.0	0.0	0.0	0.0	0.0	0.0
0.0	0.0	0.0	0.0	0.0	0.0	0.0	0.0
0.0	0.0	0.0	0.0	0.0	0.0	0.0	0.0
0.0	0.0	0.0	0.0	0.0	0.0	0.0	0.0
0.0	0.0	0.0	0.0	0.0	0.0	0.0	0.0
0.0	0.0	0.0	0.0	0.0	0.0	0.0	0.0
0.0	0.0	0.0	0.0	0.0	0.0	0.0	0.0
0.0	0.0	0.0	0.0	0.0	0.0	0.0	0.0
0.0	0.0	0.0	0.0	0.0	0.0	0.0	0.0
0.0	0.0	0.0	0.0	0.0	0.0	0.0	0.0
0.0	0.0	0.0	0.0	0.0	0.0	0.0	0.0
0.0	0.0	0.0	0.0	0.0	0.0	0.0	0.0
0.0	0.0	0.0	0.0	0.0	0.0	0.0	0.0
0.0	0.0	0.0	0.0	0.0	0.0	0.0	0.0
0.0	0.0	0.0	0.0	0.0	0.0	0.0	0.0
0.0	0.0	0.0	0.0	0.0	0.0	0.0	0.0
0.0	0.0	0.0	0.0	0.0	0.0	0.0	0.0
0.0	0.0	0.0	0.0	0.0	0.0	0.0	0.0
0.0	0.0	0.0	0.0	0.0	0.0	0.0	0.0
0.0	0.0	0.0	0.0	0.0	0.0	0.0	0.0
0.0	0.0	0.0	0.0	0.0	0.0	0.0	0.0
0.0	0.0	0.0	0.0	0.0	0.0	0.0	0.0
0.0	0.0	0.0	0.0	0.0	0.0	0.0	0.0
0.0	0.0	0.0	0.0	0.0	0.0	0.0	0.0
0.0	0.0	0.0	0.0	0.0	0.0	0.0	0.0
0.0	0.0	0.0	0.0	0.0	0.0	0.0	0.0
0.0	0.0	0.0	0.0	0.0	0.0	0.0	0.0
0.0	0.0	0.0	0.0	0.0	0.0	0.0	0.0
0.0	0.0	0.0	0.0	0.0	0.0	0.0	0.0
0.0	0.0	0.0	0.0	0.0	0.0	0.0	0.0
0.0	0.0	0.0	0.0	0.0	0.0	0.0	0.0
0.0	0.0	0.0	0.0	0.0	0.0	0.0	0.0
0.0	0.0	0.0	0.0	0.0	0.0	0.0	0.0

0.0	0.0	0.0	0.0	0.0	0.0	0.0	0.0
0.0	0.0	0.0	0.0	0.0	0.0	0.0	0.0
0.0	0.0	0.0	0.0	0.0	0.0	0.0	0.0
0.0	0.0	0.0	0.0	0.0	0.0	0.0	0.0
0.0	0.0	0.0	0.0	0.0	0.0	0.0	0.0
0.0	0.0	0.0	0.0	0.0	0.0	0.0	0.0
0.0	0.0	0.0	0.0	0.0	0.0	0.0	0.0
0.0	0.0	0.0	0.0	0.0	0.0	0.0	0.0
0.0	0.0	0.0	0.0	0.0	0.0	0.0	0.0
0.0	0.0	0.0	0.0	0.0	0.0	0.0	0.0
0.0	0.0	0.0	0.0	0.0	0.0	0.0	0.0
0.0	0.0	0.0	0.0	0.0	0.0	0.0	0.0
0.0	0.0	0.0	0.0	0.0	0.0	0.0	0.0
0.0	0.0	0.0	0.0	0.0	0.0	0.0	0.0
0.0	0.0	0.0	0.0	0.0	0.0	0.0	0.0
0.0	0.0	0.0	0.0	0.0	0.0	0.0	0.0
0.0	0.0	0.0	0.0	0.0	0.0	0.0	0.0
0.0	0.0	0.0	0.0	0.0	0.0	0.0	0.0
0.0	0.0	0.0	0.0	0.0	0.0	0.0	0.0
0.0	0.0	0.0	0.0	0.0	0.0	0.0	0.0
0.0	0.0	0.0	0.0	0.0	0.0	0.0	0.0
0.0	0.0	0.0	0.0	0.0	0.0	0.0	0.0
0.0	0.0	0.0	0.0	0.0	0.0	0.0	0.0
0.0	0.0	0.0	0.0	0.0	0.0	0.0	0.0
0.0	0.0	0.0	0.0	0.0	0.0	0.0	0.0
0.0	0.0	0.0	0.0	0.0	0.0	0.0	0.0
0.0	0.0	0.0	0.0	0.0	0.0	0.0	0.0
0.0	0.0	0.0	0.0	0.0	0.0	0.0	0.0
0.0	0.0	0.0	0.0	0.0	0.0	0.0	0.0
0.0	0.0	0.0	0.0	0.0	0.0	0.0	0.0
0.0	0.0	0.0	0.0	0.0	0.0	0.0	0.0
0.0	0.0	0.0	0.0	0.0	0.0	0.0	0.0
0.0	0.0	0.0	0.0	0.0	0.0	0.0	0.0
0.0	0.0	0.0	0.0	0.0	0.0	0.0	0.0
0.0	0.0	0.0	0.0	0.0	0.0	0.0	0.0
0.0	0.0	0.0	0.0	0.0	0.0	0.0	0.0
0.0	0.0	0.0	0.0	0.0	0.0	0.0	0.0
0.0	0.0	0.0	0.0	0.0	0.0	0.0	0.0
0.0	0.0	0.0	0.0	0.0	0.0	0.0	0.0
0.0	0.0	0.0	0.0	0.0	0.0	0.0	0.0
0.0	0.0	0.0	0.0	0.0	0.0	0.0	0.0
0.0	0.0	0.0	0.0	0.0	0.0	0.0	0.0
0.0	0.0	0.0	0.0	0.0	0.0	0.0	0.0
0.0	0.0	0.0	0.0	0.0	0.0	0.0	0.0
0.0	0.0	0.0	0.0	0.0	0.0	0.0	0.0
0.0	0.0	0.0	0.0	0.0	0.0	0.0	0.0
0.0	0.0	0.0	0.0	0.0	0.0	0.0	0.0

0.0	0.0	0.0	0.0	0.0	0.0	0.0	0.0
0.0	0.0	0.0	0.0	0.0	0.0	0.0	0.0
0.0	0.0	0.0	0.0	0.0	0.0	0.0	0.0
0.0	0.0	0.0	0.0	0.0	0.0	0.0	0.0
0.0	0.0	0.0	0.0	0.0	0.0	0.0	0.0
0.0	0.0	0.0	0.0	0.0	0.0	0.0	0.0
0.0	0.0	0.0	0.0	0.0	0.0	0.0	0.0
0.0	0.0	0.0	0.0	0.0	0.0	0.0	0.0
0.0	0.0	0.0	0.0	0.0	0.0	0.0	0.0
0.0	0.0	0.0	0.0	0.0	0.0	0.0	0.0
0.0	0.0	0.0	0.0	0.0	0.0	0.0	0.0
0.0	0.0	0.0	0.0	0.0	0.0	0.0	0.0
0.0	0.0	0.0	0.0	0.0	0.0	0.0	0.0
0.0	0.0	0.0	0.0	0.0	0.0	0.0	0.0
0.0	0.0	0.0	0.0	0.0	0.0	0.0	0.0
0.0	0.0	0.0	0.0	0.0	0.0	0.0	0.0
0.0	0.0	0.0	0.0	0.0	0.0	0.0	0.0
0.0	0.0	0.0	0.0	0.0	0.0	0.0	0.0
0.0	0.0	0.0	0.0	0.0	0.0	0.0	0.0
0.0	0.0	0.0	0.0	0.0	0.0	0.0	0.0
0.0	0.0	0.0	0.0	0.0	0.0	0.0	0.0
0.0	0.0	0.0	0.0	0.0	0.0	0.0	0.0
0.0	0.0	0.0	0.0	0.0	0.0	0.0	0.0
0.0	0.0	0.0	0.0	0.0	0.0	0.0	0.0
0.0	0.0	0.0	0.0	0.0	0.0	0.0	0.0
0.0	0.0	0.0	0.0	0.0	0.0	0.0	0.0
0.0	0.0	0.0	0.0	0.0	0.0	0.0	0.0
0.0	0.0	0.0	0.0	0.0	0.0	0.0	0.0
0.0	0.0	0.0	0.0	0.0	0.0	0.0	0.0
0.0	0.0	0.0	0.0	0.0	0.0	0.0	0.0
0.0	0.0	0.0	0.0	0.0	0.0	0.0	0.0
0.0	0.0	0.0	0.0	0.0	0.0	0.0	0.0
0.0	0.0	0.0	0.0	0.0	0.0	0.0	0.0
0.0	0.0	0.0	0.0	0.0	0.0	0.0	0.0
0.0	0.0	0.0	0.0	0.0	0.0	0.0	0.0
0.0	0.0	0.0	0.0	0.0	0.0	0.0	0.0
0.0	0.0	0.0	0.0	0.0	0.0	0.0	0.0
0.0	0.0	0.0	0.0	0.0	0.0	0.0	0.0
0.0	0.0	0.0	0.0	0.0	0.0	0.0	0.0
0.0	0.0	0.0	0.0	0.0	0.0	0.0	0.0
0.0	0.0	0.0	0.0	0.0	0.0	0.0	0.0
0.0	0.0	0.0	0.0	0.0	0.0	0.0	0.0
0.0	0.0	0.0	0.0	0.0	0.0	0.0	0.0
0.0	0.0	0.0	0.0	0.0	0.0	0.0	0.0
0.0	0.0	0.0	0.0	0.0	0.0	0.0	0.0
0.0	0.0	0.0	0.0	0.0	0.0	0.0	0.0
0.0	0.0	0.0	0.0	0.0	0.0	0.0	0.0
0.0	0.0	0.0	0.0	0.0	0.0	0.0	0.0

0.0	0.0	0.0	0.0	0.0	0.0	0.0	0.0
0.0	0.0	0.0	0.0	0.0	0.0	0.0	0.0
0.0	0.0	0.0	0.0	0.0	0.0	0.0	0.0
0.0	0.0	0.0	0.0	0.0	0.0	0.0	0.0
0.0	0.0	0.0	0.0	0.0	0.0	0.0	0.0
0.0	0.0	0.0	0.0	0.0	0.0	0.0	0.0
0.0	0.0	0.0	0.0	0.0	0.0	0.0	0.0
0.0	0.0	0.0	0.0	0.0	0.0	0.0	0.0
0.0	0.0	0.0	0.0	0.0	0.0	0.0	0.0
0.0	0.0	0.0	0.0	0.0	0.0	0.0	0.0
0.0	0.0	0.0	0.0	0.0	0.0	0.0	0.0
0.0	0.0	0.0	0.0	0.0	0.0	0.0	0.0
0.0	0.0	0.0	0.0	0.0	0.0	0.0	0.0
0.0	0.0	0.0	0.0	0.0	0.0	0.0	0.0
0.0	0.0	0.0	0.0	0.0	0.0	0.0	0.0
0.0	0.0	0.0	0.0	0.0	0.0	0.0	0.0
0.0	0.0	0.0	0.0	0.0	0.0	0.0	0.0
0.0	0.0	0.0	0.0	0.0	0.0	0.0	0.0
0.0	0.0	0.0	0.0	0.0	0.0	0.0	0.0
0.0	0.0	0.0	0.0	0.0	0.0	0.0	0.0
0.0	0.0	0.0	0.0	0.0	0.0	0.0	0.0
0.0	0.0	0.0	0.0	0.0	0.0	0.0	0.0
0.0	0.0	0.0	0.0	0.0	0.0	0.0	0.0
0.0	0.0	0.0	0.0	0.0	0.0	0.0	0.0
0.0	0.0	0.0	0.0	0.0	0.0	0.0	0.0
0.0	0.0	0.0	0.0	0.0	0.0	0.0	0.0
0.0	0.0	0.0	0.0	0.0	0.0	0.0	0.0
0.0	0.0	0.0	0.0	0.0	0.0	0.0	0.0
0.0	0.0	0.0	0.0	0.0	0.0	0.0	0.0
0.0	0.0	0.0	0.0	0.0	0.0	0.0	0.0
0.0	0.0	0.0	0.0	0.0	0.0	0.0	0.0
0.0	0.0	0.0	0.0	0.0	0.0	0.0	0.0
0.0	0.0	0.0	0.0	0.0	0.0	0.0	0.0
0.0	0.0	0.0	0.0	0.0	0.0	0.0	0.0
0.0	0.0	0.0	0.0	0.0	0.0	0.0	0.0
0.0	0.0	0.0	0.0	0.0	0.0	0.0	0.0
0.0	0.0	0.0	0.0	0.0	0.0	0.0	0.0
0.0	0.0	0.0	0.0	0.0	0.0	0.0	0.0
0.0	0.0	0.0	0.0	0.0	0.0	0.0	0.0
0.0	0.0	0.0	0.0	0.0	0.0	0.0	0.0
0.0	0.0	0.0	0.0	0.0	0.0	0.0	0.0
0.0	0.0	0.0	0.0	0.0	0.0	0.0	0.0
0.0	0.0	0.0	0.0	0.0	0.0	0.0	0.0
0.0	0.0	0.0	0.0	0.0	0.0	0.0	0.0
0.0	0.0	0.0	0.0	0.0	0.0	0.0	0.0
0.0	0.0	0.0	0.0	0.0	0.0	0.0	0.0
0.0	0.0	0.0	0.0	0.0	0.0	0.0	0.0

0.0	0.0	0.0	0.0	0.0	0.0	0.0	0.0
0.0	0.0	0.0	0.0	0.0	0.0	0.0	0.0
0.0	0.0	0.0	0.0	0.0	0.0	0.0	0.0
0.0	0.0	0.0	0.0	0.0	0.0	0.0	0.0
0.0	0.0	0.0	0.0	0.0	0.0	0.0	0.0
0.0	0.0	0.0	0.0	0.0	0.0	0.0	0.0
0.0	0.0	0.0	0.0	0.0	0.0	0.0	0.0
0.0	0.0	0.0	0.0	0.0	0.0	0.0	0.0
0.0	0.0	0.0	0.0	0.0	0.0	0.0	0.0
0.0	0.0	0.0	0.0	0.0	0.0	0.0	0.0
0.0	0.0	0.0	0.0	0.0	0.0	0.0	0.0
0.0	0.0	0.0	0.0	0.0	0.0	0.0	0.0
0.0	0.0	0.0	0.0	0.0	0.0	0.0	0.0
0.0	0.0	0.0	0.0	0.0	0.0	0.0	0.0
0.0	0.0	0.0	0.0	0.0	0.0	0.0	0.0
0.0	0.0	0.0	0.0	0.0	0.0	0.0	0.0
0.0	0.0	0.0	0.0	0.0	0.0	0.0	0.0
0.0	0.0	0.0	0.0	0.0	0.0	0.0	0.0
0.0	0.0	0.0	0.0	0.0	0.0	0.0	0.0
0.0	0.0	0.0	0.0	0.0	0.0	0.0	0.0
0.0	0.0	0.0	0.0	0.0	0.0	0.0	0.0
0.0	0.0	0.0	0.0	0.0	0.0	0.0	0.0
0.0	0.0	0.0	0.0	0.0	0.0	0.0	0.0
0.0	0.0	0.0	0.0	0.0	0.0	0.0	0.0
0.0	0.0	0.0	0.0	0.0	0.0	0.0	0.0
0.0	0.0	0.0	0.0	0.0	0.0	0.0	0.0
0.0	0.0	0.0	0.0	0.0	0.0	0.0	0.0
0.0	0.0	0.0	0.0	0.0	0.0	0.0	0.0
0.0	0.0	0.0	0.0	0.0	0.0	0.0	0.0
0.0	0.0	0.0	0.0	0.0	0.0	0.0	0.0
0.0	0.0	0.0	0.0	0.0	0.0	0.0	0.0
0.0	0.0	0.0	0.0	0.0	0.0	0.0	0.0
0.0	0.0	0.0	0.0	0.0	0.0	0.0	0.0
0.0	0.0	0.0	0.0	0.0	0.0	0.0	0.0
0.0	0.0	0.0	0.0	0.0	0.0	0.0	0.0
0.0	0.0	0.0	0.0	0.0	0.0	0.0	0.0
0.0	0.0	0.0	0.0	0.0	0.0	0.0	0.0
0.0	0.0	0.0	0.0	0.0	0.0	0.0	0.0
0.0	0.0	0.0	0.0	0.0	0.0	0.0	0.0
0.0	0.0	0.0	0.0	0.0	0.0	0.0	0.0
0.0	0.0	0.0	0.0	0.0	0.0	0.0	0.0
0.0	0.0	0.0	0.0	0.0	0.0	0.0	0.0
0.0	0.0	0.0	0.0	0.0	0.0	0.0	0.0
0.0	0.0	0.0	0.0	0.0	0.0	0.0	0.0
0.0	0.0	0.0	0.0	0.0	0.0	0.0	0.0
0.0	0.0	0.0	0.0	0.0	0.0	0.0	0.0
0.0	0.0	0.0	0.0	0.0	0.0	0.0	0.0
0.0	0.0	0.0	0.0	0.0	0.0	0.0	0.0

0.0	0.0	0.0	0.0	0.0	0.0	0.0	0.0
0.0	0.0	0.0	0.0	0.0	0.0	0.0	0.0
0.0	0.0	0.0	0.0	0.0	0.0	0.0	0.0
0.0	0.0	0.0	0.0	0.0	0.0	0.0	0.0
0.0	0.0	0.0	0.0	0.0	0.0	0.0	0.0
0.0	0.0	0.0	0.0	0.0	0.0	0.0	0.0
0.0	0.0	0.0	0.0	0.0	0.0	0.0	0.0
0.0	0.0	0.0	0.0	0.0	0.0	0.0	0.0
0.0	0.0	0.0	0.0	0.0	0.0	0.0	0.0
0.0	0.0	0.0	0.0	0.0	0.0	0.0	0.0
0.0	0.0	0.0	0.0	0.0	0.0	0.0	0.0
0.0	0.0	0.0	0.0	0.0	0.0	0.0	0.0
0.0	0.0	0.0	0.0	0.0	0.0	0.0	0.0
0.0	0.0	0.0	0.0	0.0	0.0	0.0	0.0
0.0	0.0	0.0	0.0	0.0	0.0	0.0	0.0
0.0	0.0	0.0	0.0	0.0	0.0	0.0	0.0
0.0	0.0	0.0	0.0	0.0	0.0	0.0	0.0
0.0	0.0	0.0	0.0	0.0	0.0	0.0	0.0
0.0	0.0	0.0	0.0	0.0	0.0	0.0	0.0
0.0	0.0	0.0	0.0	0.0	0.0	0.0	0.0
0.0	0.0	0.0	0.0	0.0	0.0	0.0	0.0
0.0	0.0	0.0	0.0	0.0	0.0	0.0	0.0
0.0	0.0	0.0	0.0	0.0	0.0	0.0	0.0
0.0	0.0	0.0	0.0	0.0	0.0	0.0	0.0
0.0	0.0	0.0	0.0	0.0	0.0	0.0	0.0
0.0	0.0	0.0	0.0	0.0	0.0	0.0	0.0
0.0	0.0	0.0	0.0	0.0	0.0	0.0	0.0
0.0	0.0	0.0	0.0	0.0	0.0	0.0	0.0
0.0	0.0	0.0	0.0	0.0	0.0	0.0	0.0
0.0	0.0	0.0	0.0	0.0	0.0	0.0	0.0
0.0	0.0	0.0	0.0	0.0	0.0	0.0	0.0
0.0	0.0	0.0	0.0	0.0	0.0	0.0	0.0
0.0	0.0	0.0	0.0	0.0	0.0	0.0	0.0
0.0	0.0	0.0	0.0	0.0	0.0	0.0	0.0
0.0	0.0	0.0	0.0	0.0	0.0	0.0	0.0
0.0	0.0	0.0	0.0	0.0	0.0	0.0	0.0
0.0	0.0	0.0	0.0	0.0	0.0	0.0	0.0
0.0	0.0	0.0	0.0	0.0	0.0	0.0	0.0
0.0	0.0	0.0	0.0	0.0	0.0	0.0	0.0
0.0	0.0	0.0	0.0	0.0	0.0	0.0	0.0
0.0	0.0	0.0	0.0	0.0	0.0	0.0	0.0
0.0	0.0	0.0	0.0	0.0	0.0	0.0	0.0
0.0	0.0	0.0	0.0	0.0	0.0	0.0	0.0
0.0	0.0	0.0	0.0	0.0	0.0	0.0	0.0
0.0	0.0	0.0	0.0	0.0	0.0	0.0	0.0
0.0	0.0	0.0	0.0	0.0	0.0	0.0	0.0
0.0	0.0	0.0	0.0	0.0	0.0	0.0	0.0
0.0	0.0	0.0	0.0	0.0	0.0	0.0	0.0

0.0	0.0	0.0	0.0	0.0	0.0	0.0	0.0
0.0	0.0	0.0	0.0	0.0	0.0	0.0	0.0
0.0	0.0	0.0	0.0	0.0	0.0	0.0	0.0
0.0	0.0	0.0	0.0	0.0	0.0	0.0	0.0
0.0	0.0	0.0	0.0	0.0	0.0	0.0	0.0
0.0	0.0	0.0	0.0	0.0	0.0	0.0	0.0
0.0	0.0	0.0	0.0	0.0	0.0	0.0	0.0
0.0	0.0	0.0	0.0	0.0	0.0	0.0	0.0
0.0	0.0	0.0	0.0	0.0	0.0	0.0	0.0
0.0	0.0	0.0	0.0	0.0	0.0	0.0	0.0
0.0	0.0	0.0	0.0	0.0	0.0	0.0	0.0
0.0	0.0	0.0	0.0	0.0	0.0	0.0	0.0
0.0	0.0	0.0	0.0	0.0	0.0	0.0	0.0
0.0	0.0	0.0	0.0	0.0	0.0	0.0	0.0
0.0	0.0	0.0	0.0	0.0	0.0	0.0	0.0
0.0	0.0	0.0	0.0	0.0	0.0	0.0	0.0
0.0	0.0	0.0	0.0	0.0	0.0	0.0	0.0
0.0	0.0	0.0	0.0	0.0	0.0	0.0	0.0
0.0	0.0	0.0	0.0	0.0	0.0	0.0	0.0
0.0	0.0	0.0	0.0	0.0	0.0	0.0	0.0
0.0	0.0	0.0	0.0	0.0	0.0	0.0	0.0
0.0	0.0	0.0	0.0	0.0	0.0	0.0	0.0
0.0	0.0	0.0	0.0	0.0	0.0	0.0	0.0
0.0	0.0	0.0	0.0	0.0	0.0	0.0	0.0
0.0	0.0	0.0	0.0	0.0	0.0	0.0	0.0
0.0	0.0	0.0	0.0	0.0	0.0	0.0	0.0
0.0	0.0	0.0	0.0	0.0	0.0	0.0	0.0
0.0	0.0	0.0	0.0	0.0	0.0	0.0	0.0
0.0	0.0	0.0	0.0	0.0	0.0	0.0	0.0
0.0	0.0	0.0	0.0	0.0	0.0	0.0	0.0
0.0	0.0	0.0	0.0	0.0	0.0	0.0	0.0
0.0	0.0	0.0	0.0	0.0	0.0	0.0	0.0
0.0	0.0	0.0	0.0	0.0	0.0	0.0	0.0
0.0	0.0	0.0	0.0	0.0	0.0	0.0	0.0
0.0	0.0	0.0	0.0	0.0	0.0	0.0	0.0
0.0	0.0	0.0	0.0	0.0	0.0	0.0	0.0
0.0	0.0	0.0	0.0	0.0	0.0	0.0	0.0
0.0	0.0	0.0	0.0	0.0	0.0	0.0	0.0
0.0	0.0	0.0	0.0	0.0	0.0	0.0	0.0
0.0	0.0	0.0	0.0	0.0	0.0	0.0	0.0
0.0	0.0	0.0	0.0	0.0	0.0	0.0	0.0
0.0	0.0	0.0	0.0	0.0	0.0	0.0	0.0
0.0	0.0	0.0	0.0	0.0	0.0	0.0	0.0
0.0	0.0	0.0	0.0	0.0	0.0	0.0	0.0
0.0	0.0	0.0	0.0	0.0	0.0	0.0	0.0
0.0	0.0	0.0	0.0	0.0	0.0	0.0	0.0
0.0	0.0	0.0	0.0	0.0	0.0	0.0	0.0
0.0	0.0	0.0	0.0	0.0	0.0	0.0	0.0

0.0	0.0	0.0	0.0	0.0	0.0	0.0	0.0
0.0	0.0	0.0	0.0	0.0	0.0	0.0	0.0
0.0	0.0	0.0	0.0	0.0	0.0	0.0	0.0
0.0	0.0	0.0	0.0	0.0	0.0	0.0	0.0
0.0	0.0	0.0	0.0	0.0	0.0	0.0	0.0
0.0	0.0	0.0	0.0	0.0	0.0	0.0	0.0
0.0	0.0	0.0	0.0	0.0	0.0	0.0	0.0
0.0	0.0	0.0	0.0	0.0	0.0	0.0	0.0
0.0	0.0	0.0	0.0	0.0	0.0	0.0	0.0
0.0	0.0	0.0	0.0	0.0	0.0	0.0	0.0
0.0	0.0	0.0	0.0	0.0	0.0	0.0	0.0
0.0	0.0	0.0	0.0	0.0	0.0	0.0	0.0
0.0	0.0	0.0	0.0	0.0	0.0	0.0	0.0
0.0	0.0	0.0	0.0	0.0	0.0	0.0	0.0
0.0	0.0	0.0	0.0	0.0	0.0	0.0	0.0
0.0	0.0	0.0	0.0	0.0	0.0	0.0	0.0
0.0	0.0	0.0	0.0	0.0	0.0	0.0	0.0
0.0	0.0	0.0	0.0	0.0	0.0	0.0	0.0
0.0	0.0	0.0	0.0	0.0	0.0	0.0	0.0
0.0	0.0	0.0	0.0	0.0	0.0	0.0	0.0
0.0	0.0	0.0	0.0	0.0	0.0	0.0	0.0
0.0	0.0	0.0	0.0	0.0	0.0	0.0	0.0
0.0	0.0	0.0	0.0	0.0	0.0	0.0	0.0
0.0	0.0	0.0	0.0	0.0	0.0	0.0	0.0
0.0	0.0	0.0	0.0	0.0	0.0	0.0	0.0
0.0	0.0	0.0	0.0	0.0	0.0	0.0	0.0
0.0	0.0	0.0	0.0	0.0	0.0	0.0	0.0
0.0	0.0	0.0	0.0	0.0	0.0	0.0	0.0
0.0	0.0	0.0	0.0	0.0	0.0	0.0	0.0
0.0	0.0	0.0	0.0	0.0	0.0	0.0	0.0
0.0	0.0	0.0	0.0	0.0	0.0	0.0	0.0
0.0	0.0	0.0	0.0	0.0	0.0	0.0	0.0
0.0	0.0	0.0	0.0	0.0	0.0	0.0	0.0
0.0	0.0	0.0	0.0	0.0	0.0	0.0	0.0
0.0	0.0	0.0	0.0	0.0	0.0	0.0	0.0
0.0	0.0	0.0	0.0	0.0	0.0	0.0	0.0
0.0	0.0	0.0	0.0	0.0	0.0	0.0	0.0
0.0	0.0	0.0	0.0	0.0	0.0	0.0	0.0
0.0	0.0	0.0	0.0	0.0	0.0	0.0	0.0
0.0	0.0	0.0	0.0	0.0	0.0	0.0	0.0
0.0	0.0	0.0	0.0	0.0	0.0	0.0	0.0
0.0	0.0	0.0	0.0	0.0	0.0	0.0	0.0
0.0	0.0	0.0	0.0	0.0	0.0	0.0	0.0
0.0	0.0	0.0	0.0	0.0	0.0	0.0	0.0
0.0	0.0	0.0	0.0	0.0	0.0	0.0	0.0
0.0	0.0	0.0	0.0	0.0	0.0	0.0	0.0
0.0	0.0	0.0	0.0	0.0	0.0	0.0	0.0

0.0	0.0	0.0	0.0	0.0	0.0	0.0	0.0
0.0	0.0	0.0	0.0	0.0	0.0	0.0	0.0
0.0	0.0	0.0	0.0	0.0	0.0	0.0	0.0
0.0	0.0	0.0	0.0	0.0	0.0	0.0	0.0
0.0	0.0	0.0	0.0	0.0	0.0	0.0	0.0
0.0	0.0	0.0	0.0	0.0	0.0	0.0	0.0
0.0	0.0	0.0	0.0	0.0	0.0	0.0	0.0
0.0	0.0	0.0	0.0	0.0	0.0	0.0	0.0
0.0	0.0	0.0	0.0	0.0	0.0	0.0	0.0
0.0	0.0	0.0	0.0	0.0	0.0	0.0	0.0
0.0	0.0	0.0	0.0	0.0	0.0	0.0	0.0
0.0	0.0	0.0	0.0	0.0	0.0	0.0	0.0
0.0	0.0	0.0	0.0	0.0	0.0	0.0	0.0
0.0	0.0	0.0	0.0	0.0	0.0	0.0	0.0
0.0	0.0	0.0	0.0	0.0	0.0	0.0	0.0
0.0	0.0	0.0	0.0	0.0	0.0	0.0	0.0
0.0	0.0	0.0	0.0	0.0	0.0	0.0	0.0
0.0	0.0	0.0	0.0	0.0	0.0	0.0	0.0
0.0	0.0	0.0	0.0	0.0	0.0	0.0	0.0
0.0	0.0	0.0	0.0	0.0	0.0	0.0	0.0
0.0	0.0	0.0	0.0	0.0	0.0	0.0	0.0
0.0	0.0	0.0	0.0	0.0	0.0	0.0	0.0
0.0	0.0	0.0	0.0	0.0	0.0	0.0	0.0
0.0	0.0	0.0	0.0	0.0	0.0	0.0	0.0
0.0	0.0	0.0	0.0	0.0	0.0	0.0	0.0
0.0	0.0	0.0	0.0	0.0	0.0	0.0	0.0
0.0	0.0	0.0	0.0	0.0	0.0	0.0	0.0
0.0	0.0	0.0	0.0	0.0	0.0	0.0	0.0
0.0	0.0	0.0	0.0	0.0	0.0	0.0	0.0
0.0	0.0	0.0	0.0	0.0	0.0	0.0	0.0
0.0	0.0	0.0	0.0	0.0	0.0	0.0	0.0
0.0	0.0	0.0	0.0	0.0	0.0	0.0	0.0
0.0	0.0	0.0	0.0	0.0	0.0	0.0	0.0
0.0	0.0	0.0	0.0	0.0	0.0	0.0	0.0
0.0	0.0	0.0	0.0	0.0	0.0	0.0	0.0
0.0	0.0	0.0	0.0	0.0	0.0	0.0	0.0
0.0	0.0	0.0	0.0	0.0	0.0	0.0	0.0
0.0	0.0	0.0	0.0	0.0	0.0	0.0	0.0
0.0	0.0	0.0	0.0	0.0	0.0	0.0	0.0
0.0	0.0	0.0	0.0	0.0	0.0	0.0	0.0
0.0	0.0	0.0	0.0	0.0	0.0	0.0	0.0
0.0	0.0	0.0	0.0	0.0	0.0	0.0	0.0
0.0	0.0	0.0	0.0	0.0	0.0	0.0	0.0
0.0	0.0	0.0	0.0	0.0	0.0	0.0	0.0
0.0	0.0	0.0	0.0	0.0	0.0	0.0	0.0
0.0	0.0	0.0	0.0	0.0	0.0	0.0	0.0
0.0	0.0	0.0	0.0	0.0	0.0	0.0	0.0
0.0	0.0	0.0	0.0	0.0	0.0	0.0	0.0

0.0	0.0	0.0	0.0	0.0	0.0	0.0	0.0
0.0	0.0	0.0	0.0	0.0	0.0	0.0	0.0
0.0	0.0	0.0	0.0	0.0	0.0	0.0	0.0
0.0	0.0	0.0	0.0	0.0	0.0	0.0	0.0
0.0	0.0	0.0	0.0	0.0	0.0	0.0	0.0
0.0	0.0	0.0	0.0	0.0	0.0	0.0	0.0
0.0	0.0	0.0	0.0	0.0	0.0	0.0	0.0
0.0	0.0	0.0	0.0	0.0	0.0	0.0	0.0
0.0	0.0	0.0	0.0	0.0	0.0	0.0	0.0
0.0	0.0	0.0	0.0	0.0	0.0	0.0	0.0
0.0	0.0	0.0	0.0	0.0	0.0	0.0	0.0
0.0	0.0	0.0	0.0	0.0	0.0	0.0	0.0
0.0	0.0	0.0	0.0	0.0	0.0	0.0	0.0
0.0	0.0	0.0	0.0	0.0	0.0	0.0	0.0
0.0	0.0	0.0	0.0	0.0	0.0	0.0	0.0
0.0	0.0	0.0	0.0	0.0	0.0	0.0	0.0
0.0	0.0	0.0	0.0	0.0	0.0	0.0	0.0
0.0	0.0	0.0	0.0	0.0	0.0	0.0	0.0
0.0	0.0	0.0	0.0	0.0	0.0	0.0	0.0
0.0	0.0	0.0	0.0	0.0	0.0	0.0	0.0
0.0	0.0	0.0	0.0	0.0	0.0	0.0	0.0
0.0	0.0	0.0	0.0	0.0	0.0	0.0	0.0
0.0	0.0	0.0	0.0	0.0	0.0	0.0	0.0
0.0	0.0	0.0	0.0	0.0	0.0	0.0	0.0
0.0	0.0	0.0	0.0	0.0	0.0	0.0	0.0
0.0	0.0	0.0	0.0	0.0	0.0	0.0	0.0
0.0	0.0	0.0	0.0	0.0	0.0	0.0	0.0
0.0	0.0	0.0	0.0	0.0	0.0	0.0	0.0
0.0	0.0	0.0	0.0	0.0	0.0	0.0	0.0
0.0	0.0	0.0	0.0	0.0	0.0	0.0	0.0
0.0	0.0	0.0	0.0	0.0	0.0	0.0	0.0
0.0	0.0	0.0	0.0	0.0	0.0	0.0	0.0
0.0	0.0	0.0	0.0	0.0	0.0	0.0	0.0
0.0	0.0	0.0	0.0	0.0	0.0	0.0	0.0
0.0	0.0	0.0	0.0	0.0	0.0	0.0	0.0
0.0	0.0	0.0	0.0	0.0	0.0	0.0	0.0
0.0	0.0	0.0	0.0	0.0	0.0	0.0	0.0
0.0	0.0	0.0	0.0	0.0	0.0	0.0	0.0
0.0	0.0	0.0	0.0	0.0	0.0	0.0	0.0
0.0	0.0	0.0	0.0	0.0	0.0	0.0	0.0
0.0	0.0	0.0	0.0	0.0	0.0	0.0	0.0
0.0	0.0	0.0	0.0	0.0	0.0	0.0	0.0
0.0	0.0	0.0	0.0	0.0	0.0	0.0	0.0
0.0	0.0	0.0	0.0	0.0	0.0	0.0	0.0
0.0	0.0	0.0	0.0	0.0	0.0	0.0	0.0
0.0	0.0	0.0	0.0	0.0	0.0	0.0	0.0
0.0	0.0	0.0	0.0	0.0	0.0	0.0	0.0

0.0	0.0	0.0	0.0	0.0	0.0	0.0	0.0
0.0	0.0	0.0	0.0	0.0	0.0	0.0	0.0
0.0	0.0	0.0	0.0	0.0	0.0	0.0	0.0
0.0	0.0	0.0	0.0	0.0	0.0	0.0	0.0
0.0	0.0	0.0	0.0	0.0	0.0	0.0	0.0
0.0	0.0	0.0	0.0	0.0	0.0	0.0	0.0
0.0	0.0	0.0	0.0	0.0	0.0	0.0	0.0
0.0	0.0	0.0	0.0	0.0	0.0	0.0	0.0
0.0	0.0	0.0	0.0	0.0	0.0	0.0	0.0
0.0	0.0	0.0	0.0	0.0	0.0	0.0	0.0
0.0	0.0	0.0	0.0	0.0	0.0	0.0	0.0
0.0	0.0	0.0	0.0	0.0	0.0	0.0	0.0
0.0	0.0	0.0	0.0	0.0	0.0	0.0	0.0
0.0	0.0	0.0	0.0	0.0	0.0	0.0	0.0
0.0	0.0	0.0	0.0	0.0	0.0	0.0	0.0
0.0	0.0	0.0	0.0	0.0	0.0	0.0	0.0
0.0	0.0	0.0	0.0	0.0	0.0	0.0	0.0
0.0	0.0	0.0	0.0	0.0	0.0	0.0	0.0
0.0	0.0	0.0	0.0	0.0	0.0	0.0	0.0
0.0	0.0	0.0	0.0	0.0	0.0	0.0	0.0
0.0	0.0	0.0	0.0	0.0	0.0	0.0	0.0
0.0	0.0	0.0	0.0	0.0	0.0	0.0	0.0
0.0	0.0	0.0	0.0	0.0	0.0	0.0	0.0
0.0	0.0	0.0	0.0	0.0	0.0	0.0	0.0
0.0	0.0	0.0	0.0	0.0	0.0	0.0	0.0
0.0	0.0	0.0	0.0	0.0	0.0	0.0	0.0
0.0	0.0	0.0	0.0	0.0	0.0	0.0	0.0
0.0	0.0	0.0	0.0	0.0	0.0	0.0	0.0
0.0	0.0	0.0	0.0	0.0	0.0	0.0	0.0
0.0	0.0	0.0	0.0	0.0	0.0	0.0	0.0
0.0	0.0	0.0	0.0	0.0	0.0	0.0	0.0
0.0	0.0	0.0	0.0	0.0	0.0	0.0	0.0
0.0	0.0	0.0	0.0	0.0	0.0	0.0	0.0
0.0	0.0	0.0	0.0	0.0	0.0	0.0	0.0
0.0	0.0	0.0	0.0	0.0	0.0	0.0	0.0
0.0	0.0	0.0	0.0	0.0	0.0	0.0	0.0
0.0	0.0	0.0	0.0	0.0	0.0	0.0	0.0
0.0	0.0	0.0	0.0	0.0	0.0	0.0	0.0
0.0	0.0	0.0	0.0	0.0	0.0	0.0	0.0
0.0	0.0	0.0	0.0	0.0	0.0	0.0	0.0
0.0	0.0	0.0	0.0	0.0	0.0	0.0	0.0
0.0	0.0	0.0	0.0	0.0	0.0	0.0	0.0
0.0	0.0	0.0	0.0	0.0	0.0	0.0	0.0
0.0	0.0	0.0	0.0	0.0	0.0	0.0	0.0
0.0	0.0	0.0	0.0	0.0	0.0	0.0	0.0
0.0	0.0	0.0	0.0	0.0	0.0	0.0	0.0
0.0	0.0	0.0	0.0	0.0	0.0	0.0	0.0

0.0	0.0	0.0	0.0	0.0	0.0	0.0	0.0
0.0	0.0	0.0	0.0	0.0	0.0	0.0	0.0
0.0	0.0	0.0	0.0	0.0	0.0	0.0	0.0
0.0	0.0	0.0	0.0	0.0	0.0	0.0	0.0
0.0	0.0	0.0	0.0	0.0	0.0	0.0	0.0
0.0	0.0	0.0	0.0	0.0	0.0	0.0	0.0
0.0	0.0	0.0	0.0	0.0	0.0	0.0	0.0
0.0	0.0	0.0	0.0	0.0	0.0	0.0	0.0
0.0	0.0	0.0	0.0	0.0	0.0	0.0	0.0
0.0	0.0	0.0	0.0	0.0	0.0	0.0	0.0
0.0	0.0	0.0	0.0	0.0	0.0	0.0	0.0
0.0	0.0	0.0	0.0	0.0	0.0	0.0	0.0
0.0	0.0	0.0	0.0	0.0	0.0	0.0	0.0
0.0	0.0	0.0	0.0	0.0	0.0	0.0	0.0
0.0	0.0	0.0	0.0	0.0	0.0	0.0	0.0
0.0	0.0	0.0	0.0	0.0	0.0	0.0	0.0
0.0	0.0	0.0	0.0	0.0	0.0	0.0	0.0
0.0	0.0	0.0	0.0	0.0	0.0	0.0	0.0
0.0	0.0	0.0	0.0	0.0	0.0	0.0	0.0
0.0	0.0	0.0	0.0	0.0	0.0	0.0	0.0
0.0	0.0	0.0	0.0	0.0	0.0	0.0	0.0
0.0	0.0	0.0	0.0	0.0	0.0	0.0	0.0
0.0	0.0	0.0	0.0	0.0	0.0	0.0	0.0
0.0	0.0	0.0	0.0	0.0	0.0	0.0	0.0
0.0	0.0	0.0	0.0	0.0	0.0	0.0	0.0
0.0	0.0	0.0	0.0	0.0	0.0	0.0	0.0
0.0	0.0	0.0	0.0	0.0	0.0	0.0	0.0
0.0	0.0	0.0	0.0	0.0	0.0	0.0	0.0
0.0	0.0	0.0	0.0	0.0	0.0	0.0	0.0
0.0	0.0	0.0	0.0	0.0	0.0	0.0	0.0
0.0	0.0	0.0	0.0	0.0	0.0	0.0	0.0
0.0	0.0	0.0	0.0	0.0	0.0	0.0	0.0
0.0	0.0	0.0	0.0	0.0	0.0	0.0	0.0
0.0	0.0	0.0	0.0	0.0	0.0	0.0	0.0
0.0	0.0	0.0	0.0	0.0	0.0	0.0	0.0
0.0	0.0	0.0	0.0	0.0	0.0	0.0	0.0
0.0	0.0	0.0	0.0	0.0	0.0	0.0	0.0
0.0	0.0	0.0	0.0	0.0	0.0	0.0	0.0
0.0	0.0	0.0	0.0	0.0	0.0	0.0	0.0
0.0	0.0	0.0	0.0	0.0	0.0	0.0	0.0
0.0	0.0	0.0	0.0	0.0	0.0	0.0	0.0
0.0	0.0	0.0	0.0	0.0	0.0	0.0	0.0
0.0	0.0	0.0	0.0	0.0	0.0	0.0	0.0
0.0	0.0	0.0	0.0	0.0	0.0	0.0	0.0
0.0	0.0	0.0	0.0	0.0	0.0	0.0	0.0
0.0	0.0	0.0	0.0	0.0	0.0	0.0	0.0
0.0	0.0	0.0	0.0	0.0	0.0	0.0	0.0
0.0	0.0	0.0	0.0	0.0	0.0	0.0	0.0

0.0	0.0	0.0	0.0	0.0	0.0	0.0	0.0
0.0	0.0	0.0	0.0	0.0	0.0	0.0	0.0
0.0	0.0	0.0	0.0	0.0	0.0	0.0	0.0
0.0	0.0	0.0	0.0	0.0	0.0	0.0	0.0
0.0	0.0	0.0	0.0	0.0	0.0	0.0	0.0
0.0	0.0	0.0	0.0	0.0	0.0	0.0	0.0
0.0	0.0	0.0	0.0	0.0	0.0	0.0	0.0
0.0	0.0	0.0	0.0	0.0	0.0	0.0	0.0
0.0	0.0	0.0	0.0	0.0	0.0	0.0	0.0
0.0	0.0	0.0	0.0	0.0	0.0	0.0	0.0
0.0	0.0	0.0	0.0	0.0	0.0	0.0	0.0
0.0	0.0	0.0	0.0	0.0	0.0	0.0	0.0
0.0	0.0	0.0	0.0	0.0	0.0	0.0	0.0
0.0	0.0	0.0	0.0	0.0	0.0	0.0	0.0
0.0	0.0	0.0	0.0	0.0	0.0	0.0	0.0
0.0	0.0	0.0	0.0	0.0	0.0	0.0	0.0
0.0	0.0	0.0	0.0	0.0	0.0	0.0	0.0
0.0	0.0	0.0	0.0	0.0	0.0	0.0	0.0
0.0	0.0	0.0	0.0	0.0	0.0	0.0	0.0
0.0	0.0	0.0	0.0	0.0	0.0	0.0	0.0
0.0	0.0	0.0	0.0	0.0	0.0	0.0	0.0
0.0	0.0	0.0	0.0	0.0	0.0	0.0	0.0
0.0	0.0	0.0	0.0	0.0	0.0	0.0	0.0
0.0	0.0	0.0	0.0	0.0	0.0	0.0	0.0
0.0	0.0	0.0	0.0	0.0	0.0	0.0	0.0
0.0	0.0	0.0	0.0	0.0	0.0	0.0	0.0
0.0	0.0	0.0	0.0	0.0	0.0	0.0	0.0
0.0	0.0	0.0	0.0	0.0	0.0	0.0	0.0
0.0	0.0	0.0	0.0	0.0	0.0	0.0	0.0
0.0	0.0	0.0	0.0	0.0	0.0	0.0	0.0
0.0	0.0	0.0	0.0	0.0	0.0	0.0	0.0
0.0	0.0	0.0	0.0	0.0	0.0	0.0	0.0
0.0	0.0	0.0	0.0	0.0	0.0	0.0	0.0
0.0	0.0	0.0	0.0	0.0	0.0	0.0	0.0
0.0	0.0	0.0	0.0	0.0	0.0	0.0	0.0
0.0	0.0	0.0	0.0	0.0	0.0	0.0	0.0
0.0	0.0	0.0	0.0	0.0	0.0	0.0	0.0
0.0	0.0	0.0	0.0	0.0	0.0	0.0	0.0
0.0	0.0	0.0	0.0	0.0	0.0	0.0	0.0
0.0	0.0	0.0	0.0	0.0	0.0	0.0	0.0
0.0	0.0	0.0	0.0	0.0	0.0	0.0	0.0
0.0	0.0	0.0	0.0	0.0	0.0	0.0	0.0
0.0	0.0	0.0	0.0	0.0	0.0	0.0	0.0
0.0	0.0	0.0	0.0	0.0	0.0	0.0	0.0
0.0	0.0	0.0	0.0	0.0	0.0	0.0	0.0
0.0	0.0	0.0	0.0	0.0	0.0	0.0	0.0
0.0	0.0	0.0	0.0	0.0	0.0	0.0	0.0

0.0	0.0	0.0	0.0	0.0	0.0	0.0	0.0
0.0	0.0	0.0	0.0	0.0	0.0	0.0	0.0
0.0	0.0	0.0	0.0	0.0	0.0	0.0	0.0
0.0	0.0	0.0	0.0	0.0	0.0	0.0	0.0
0.0	0.0	0.0	0.0	0.0	0.0	0.0	0.0
0.0	0.0	0.0	0.0	0.0	0.0	0.0	0.0
0.0	0.0	0.0	0.0	0.0	0.0	0.0	0.0
0.0	0.0	0.0	0.0	0.0	0.0	0.0	0.0
0.0	0.0	0.0	0.0	0.0	0.0	0.0	0.0
0.0	0.0	0.0	0.0	0.0	0.0	0.0	0.0
0.0	0.0	0.0	0.0	0.0	0.0	0.0	0.0
0.0	0.0	0.0	0.0	0.0	0.0	0.0	0.0
0.0	0.0	0.0	0.0	0.0	0.0	0.0	0.0
0.0	0.0	0.0	0.0	0.0	0.0	0.0	0.0
0.0	0.0	0.0	0.0	0.0	0.0	0.0	0.0
0.0	0.0	0.0	0.0	0.0	0.0	0.0	0.0
0.0	0.0	0.0	0.0	0.0	0.0	0.0	0.0
0.0	0.0	0.0	0.0	0.0	0.0	0.0	0.0
0.0	0.0	0.0	0.0	0.0	0.0	0.0	0.0
0.0	0.0	0.0	0.0	0.0	0.0	0.0	0.0
0.0	0.0	0.0	0.0	0.0	0.0	0.0	0.0
0.0	0.0	0.0	0.0	0.0	0.0	0.0	0.0
0.0	0.0	0.0	0.0	0.0	0.0	0.0	0.0
0.0	0.0	0.0	0.0	0.0	0.0	0.0	0.0
0.0	0.0	0.0	0.0	0.0	0.0	0.0	0.0
0.0	0.0	0.0	0.0	0.0	0.0	0.0	0.0
0.0	0.0	0.0	0.0	0.0	0.0	0.0	0.0
0.0	0.0	0.0	0.0	0.0	0.0	0.0	0.0
0.0	0.0	0.0	0.0	0.0	0.0	0.0	0.0
0.0	0.0	0.0	0.0	0.0	0.0	0.0	0.0
0.0	0.0	0.0	0.0	0.0	0.0	0.0	0.0
0.0	0.0	0.0	0.0	0.0	0.0	0.0	0.0
0.0	0.0	0.0	0.0	0.0	0.0	0.0	0.0
0.0	0.0	0.0	0.0	0.0	0.0	0.0	0.0
0.0	0.0	0.0	0.0	0.0	0.0	0.0	0.0
0.0	0.0	0.0	0.0	0.0	0.0	0.0	0.0
0.0	0.0	0.0	0.0	0.0	0.0	0.0	0.0
0.0	0.0	0.0	0.0	0.0	0.0	0.0	0.0
0.0	0.0	0.0	0.0	0.0	0.0	0.0	0.0
0.0	0.0	0.0	0.0	0.0	0.0	0.0	0.0
0.0	0.0	0.0	0.0	0.0	0.0	0.0	0.0
0.0	0.0	0.0	0.0	0.0	0.0	0.0	0.0
0.0	0.0	0.0	0.0	0.0	0.0	0.0	0.0
0.0	0.0	0.0	0.0	0.0	0.0	0.0	0.0
0.0	0.0	0.0	0.0	0.0	0.0	0.0	0.0
0.0	0.0	0.0	0.0	0.0	0.0	0.0	0.0
0.0	0.0	0.0	0.0	0.0	0.0	0.0	0.0

0.0	0.0	0.0	0.0	0.0	0.0	0.0	0.0
0.0	0.0	0.0	0.0	0.0	0.0	0.0	0.0
0.0	0.0	0.0	0.0	0.0	0.0	0.0	0.0
0.0	0.0	0.0	0.0	0.0	0.0	0.0	0.0
0.0	0.0	0.0	0.0	0.0	0.0	0.0	0.0
0.0	0.0	0.0	0.0	0.0	0.0	0.0	0.0
0.0	0.0	0.0	0.0	0.0	0.0	0.0	0.0
0.0	0.0	0.0	0.0	0.0	0.0	0.0	0.0
0.0	0.0	0.0	0.0	0.0	0.0	0.0	0.0
0.0	0.0	0.0	0.0	0.0	0.0	0.0	0.0
0.0	0.0	0.0	0.0	0.0	0.0	0.0	0.0
0.0	0.0	0.0	0.0	0.0	0.0	0.0	0.0
0.0	0.0	0.0	0.0	0.0	0.0	0.0	0.0
0.0	0.0	0.0	0.0	0.0	0.0	0.0	0.0
0.0	0.0	0.0	0.0	0.0	0.0	0.0	0.0
0.0	0.0	0.0	0.0	0.0	0.0	0.0	0.0
0.0	0.0	0.0	0.0	0.0	0.0	0.0	0.0
0.0	0.0	0.0	0.0	0.0	0.0	0.0	0.0
0.0	0.0	0.0	0.0	0.0	0.0	0.0	0.0
0.0	0.0	0.0	0.0	0.0	0.0	0.0	0.0
0.0	0.0	0.0	0.0	0.0	0.0	0.0	0.0
0.0	0.0	0.0	0.0	0.0	0.0	0.0	0.0
0.0	0.0	0.0	0.0	0.0	0.0	0.0	0.0
0.0	0.0	0.0	0.0	0.0	0.0	0.0	0.0
0.0	0.0	0.0	0.0	0.0	0.0	0.0	0.0
0.0	0.0	0.0	0.0	0.0	0.0	0.0	0.0
0.0	0.0	0.0	0.0	0.0	0.0	0.0	0.0
0.0	0.0	0.0	0.0	0.0	0.0	0.0	0.0
0.0	0.0	0.0	0.0	0.0	0.0	0.0	0.0
0.0	0.0	0.0	0.0	0.0	0.0	0.0	0.0
0.0	0.0	0.0	0.0	0.0	0.0	0.0	0.0
0.0	0.0	0.0	0.0	0.0	0.0	0.0	0.0
0.0	0.0	0.0	0.0	0.0	0.0	0.0	0.0
0.0	0.0	0.0	0.0	0.0	0.0	0.0	0.0
0.0	0.0	0.0	0.0	0.0	0.0	0.0	0.0
0.0	0.0	0.0	0.0	0.0	0.0	0.0	0.0
0.0	0.0	0.0	0.0	0.0	0.0	0.0	0.0
0.0	0.0	0.0	0.0	0.0	0.0	0.0	0.0
0.0	0.0	0.0	0.0	0.0	0.0	0.0	0.0
0.0	0.0	0.0	0.0	0.0	0.0	0.0	0.0
0.0	0.0	0.0	0.0	0.0	0.0	0.0	0.0
0.0	0.0	0.0	0.0	0.0	0.0	0.0	0.0
0.0	0.0	0.0	0.0	0.0	0.0	0.0	0.0
0.0	0.0	0.0	0.0	0.0	0.0	0.0	0.0
0.0	0.0	0.0	0.0	0.0	0.0	0.0	0.0
0.0	0.0	0.0	0.0	0.0	0.0	0.0	0.0
0.0	0.0	0.0	0.0	0.0	0.0	0.0	0.0
0.0	0.0	0.0	0.0	0.0	0.0	0.0	0.0

0.0	0.0	0.0	0.0	0.0	0.0	0.0	0.0
0.0	0.0	0.0	0.0	0.0	0.0	0.0	0.0
0.0	0.0	0.0	0.0	0.0	0.0	0.0	0.0
0.0	0.0	0.0	0.0	0.0	0.0	0.0	0.0
0.0	0.0	0.0	0.0	0.0	0.0	0.0	0.0
0.0	0.0	0.0	0.0	0.0	0.0	0.0	0.0
0.0	0.0	0.0	0.0	0.0	0.0	0.0	0.0
0.0	0.0	0.0	0.0	0.0	0.0	0.0	0.0
0.0	0.0	0.0	0.0	0.0	0.0	0.0	0.0
0.0	0.0	0.0	0.0	0.0	0.0	0.0	0.0
0.0	0.0	0.0	0.0	0.0	0.0	0.0	0.0
0.0	0.0	0.0	0.0	0.0	0.0	0.0	0.0
0.0	0.0	0.0	0.0	0.0	0.0	0.0	0.0
0.0	0.0	0.0	0.0	0.0	0.0	0.0	0.0
0.0	0.0	0.0	0.0	0.0	0.0	0.0	0.0
0.0	0.0	0.0	0.0	0.0	0.0	0.0	0.0
0.0	0.0	0.0	0.0	0.0	0.0	0.0	0.0
0.0	0.0	0.0	0.0	0.0	0.0	0.0	0.0
0.0	0.0	0.0	0.0	0.0	0.0	0.0	0.0
0.0	0.0	0.0	0.0	0.0	0.0	0.0	0.0
0.0	0.0	0.0	0.0	0.0	0.0	0.0	0.0
0.0	0.0	0.0	0.0	0.0	0.0	0.0	0.0
0.0	0.0	0.0	0.0	0.0	0.0	0.0	0.0
0.0	0.0	0.0	0.0	0.0	0.0	0.0	0.0
0.0	0.0	0.0	0.0	0.0	0.0	0.0	0.0
0.0	0.0	0.0	0.0	0.0	0.0	0.0	0.0
0.0	0.0	0.0	0.0	0.0	0.0	0.0	0.0
0.0	0.0	0.0	0.0	0.0	0.0	0.0	0.0
0.0	0.0	0.0	0.0	0.0	0.0	0.0	0.0
0.0	0.0	0.0	0.0	0.0	0.0	0.0	0.0
0.0	0.0	0.0	0.0	0.0	0.0	0.0	0.0
0.0	0.0	0.0	0.0	0.0	0.0	0.0	0.0
0.0	0.0	0.0	0.0	0.0	0.0	0.0	0.0
0.0	0.0	0.0	0.0	0.0	0.0	0.0	0.0
0.0	0.0	0.0	0.0	0.0	0.0	0.0	0.0
0.0	0.0	0.0	0.0	0.0	0.0	0.0	0.0
0.0	0.0	0.0	0.0	0.0	0.0	0.0	0.0
0.0	0.0	0.0	0.0	0.0	0.0	0.0	0.0
0.0	0.0	0.0	0.0	0.0	0.0	0.0	0.0
0.0	0.0	0.0	0.0	0.0	0.0	0.0	0.0
0.0	0.0	0.0	0.0	0.0	0.0	0.0	0.0
0.0	0.0	0.0	0.0	0.0	0.0	0.0	0.0
0.0	0.0	0.0	0.0	0.0	0.0	0.0	0.0
0.0	0.0	0.0	0.0	0.0	0.0	0.0	0.0
0.0	0.0	0.0	0.0	0.0	0.0	0.0	0.0
0.0	0.0	0.0	0.0	0.0	0.0	0.0	0.0
0.0	0.0	0.0	0.0	0.0	0.0	0.0	0.0

0.0	0.0	0.0	0.0	0.0	0.0	0.0	0.0
0.0	0.0	0.0	0.0	0.0	0.0	0.0	0.0
0.0	0.0	0.0	0.0	0.0	0.0	0.0	0.0
0.0	0.0	0.0	0.0	0.0	0.0	0.0	0.0
0.0	0.0	0.0	0.0	0.0	0.0	0.0	0.0
0.0	0.0	0.0	0.0	0.0	0.0	0.0	0.0
0.0	0.0	0.0	0.0	0.0	0.0	0.0	0.0
0.0	0.0	0.0	0.0	0.0	0.0	0.0	0.0
0.0	0.0	0.0	0.0	0.0	0.0	0.0	0.0
0.0	0.0	0.0	0.0	0.0	0.0	0.0	0.0
0.0	0.0	0.0	0.0	0.0	0.0	0.0	0.0
0.0	0.0	0.0	0.0	0.0	0.0	0.0	0.0
0.0	0.0	0.0	0.0	0.0	0.0	0.0	0.0
0.0	0.0	0.0	0.0	0.0	0.0	0.0	0.0
0.0	0.0	0.0	0.0	0.0	0.0	0.0	0.0
0.0	0.0	0.0	0.0	0.0	0.0	0.0	0.0
0.0	0.0	0.0	0.0	0.0	0.0	0.0	0.0
0.0	0.0	0.0	0.0	0.0	0.0	0.0	0.0
0.0	0.0	0.0	0.0	0.0	0.0	0.0	0.0
0.0	0.0	0.0	0.0	0.0	0.0	0.0	0.0
0.0	0.0	0.0	0.0	0.0	0.0	0.0	0.0
0.0	0.0	0.0	0.0	0.0	0.0	0.0	0.0
0.0	0.0	0.0	0.0	0.0	0.0	0.0	0.0
0.0	0.0	0.0	0.0	0.0	0.0	0.0	0.0
0.0	0.0	0.0	0.0	0.0	0.0	0.0	0.0
0.0	0.0	0.0	0.0	0.0	0.0	0.0	0.0
0.0	0.0	0.0	0.0	0.0	0.0	0.0	0.0
0.0	0.0	0.0	0.0	0.0	0.0	0.0	0.0
0.0	0.0	0.0	0.0	0.0	0.0	0.0	0.0
0.0	0.0	0.0	0.0	0.0	0.0	0.0	0.0
0.0	0.0	0.0	0.0	0.0	0.0	0.0	0.0
0.0	0.0	0.0	0.0	0.0	0.0	0.0	0.0
0.0	0.0	0.0	0.0	0.0	0.0	0.0	0.0
0.0	0.0	0.0	0.0	0.0	0.0	0.0	0.0
0.0	0.0	0.0	0.0	0.0	0.0	0.0	0.0
0.0	0.0	0.0	0.0	0.0	0.0	0.0	0.0
0.0	0.0	0.0	0.0	0.0	0.0	0.0	0.0
0.0	0.0	0.0	0.0	0.0	0.0	0.0	0.0
0.0	0.0	0.0	0.0	0.0	0.0	0.0	0.0
0.0	0.0	0.0	0.0	0.0	0.0	0.0	0.0
0.0	0.0	0.0	0.0	0.0	0.0	0.0	0.0
0.0	0.0	0.0	0.0	0.0	0.0	0.0	0.0
0.0	0.0	0.0	0.0	0.0	0.0	0.0	0.0
0.0	0.0	0.0	0.0	0.0	0.0	0.0	0.0
0.0	0.0	0.0	0.0	0.0	0.0	0.0	0.0
0.0	0.0	0.0	0.0	0.0	0.0	0.0	0.0
0.0	0.0	0.0	0.0	0.0	0.0	0.0	0.0

0.0	0.0	0.0	0.0	0.0	0.0	0.0	0.0
0.0	0.0	0.0	0.0	0.0	0.0	0.0	0.0
0.0	0.0	0.0	0.0	0.0	0.0	0.0	0.0
0.0	0.0	0.0	0.0	0.0	0.0	0.0	0.0
0.0	0.0	0.0	0.0	0.0	0.0	0.0	0.0
0.0	0.0	0.0	0.0	0.0	0.0	0.0	0.0
0.0	0.0	0.0	0.0	0.0	0.0	0.0	0.0
0.0	0.0	0.0	0.0	0.0	0.0	0.0	0.0
0.0	0.0	0.0	0.0	0.0	0.0	0.0	0.0
0.0	0.0	0.0	0.0	0.0	0.0	0.0	0.0
0.0	0.0	0.0	0.0	0.0	0.0	0.0	0.0
0.0	0.0	0.0	0.0	0.0	0.0	0.0	0.0
0.0	0.0	0.0	0.0	0.0	0.0	0.0	0.0
0.0	0.0	0.0	0.0	0.0	0.0	0.0	0.0
0.0	0.0	0.0	0.0	0.0	0.0	0.0	0.0
0.0	0.0	0.0	0.0	0.0	0.0	0.0	0.0
0.0	0.0	0.0	0.0	0.0	0.0	0.0	0.0
0.0	0.0	0.0	0.0	0.0	0.0	0.0	0.0
0.0	0.0	0.0	0.0	0.0	0.0	0.0	0.0
0.0	0.0	0.0	0.0	0.0	0.0	0.0	0.0
0.0	0.0	0.0	0.0	0.0	0.0	0.0	0.0
0.0	0.0	0.0	0.0	0.0	0.0	0.0	0.0
0.0	0.0	0.0	0.0	0.0	0.0	0.0	0.0
0.0	0.0	0.0	0.0	0.0	0.0	0.0	0.0
0.0	0.0	0.0	0.0	0.0	0.0	0.0	0.0
0.0	0.0	0.0	0.0	0.0	0.0	0.0	0.0
0.0	0.0	0.0	0.0	0.0	0.0	0.0	0.0
0.0	0.0	0.0	0.0	0.0	0.0	0.0	0.0
0.0	0.0	0.0	0.0	0.0	0.0	0.0	0.0
0.0	0.0	0.0	0.0	0.0	0.0	0.0	0.0
0.0	0.0	0.0	0.0	0.0	0.0	0.0	0.0
0.0	0.0	0.0	0.0	0.0	0.0	0.0	0.0
0.0	0.0	0.0	0.0	0.0	0.0	0.0	0.0
0.0	0.0	0.0	0.0	0.0	0.0	0.0	0.0
0.0	0.0	0.0	0.0	0.0	0.0	0.0	0.0
0.0	0.0	0.0	0.0	0.0	0.0	0.0	0.0
0.0	0.0	0.0	0.0	0.0	0.0	0.0	0.0
0.0	0.0	0.0	0.0	0.0	0.0	0.0	0.0
0.0	0.0	0.0	0.0	0.0	0.0	0.0	0.0
0.0	0.0	0.0	0.0	0.0	0.0	0.0	0.0
0.0	0.0	0.0	0.0	0.0	0.0	0.0	0.0
0.0	0.0	0.0	0.0	0.0	0.0	0.0	0.0
0.0	0.0	0.0	0.0	0.0	0.0	0.0	0.0
0.0	0.0	0.0	0.0	0.0	0.0	0.0	0.0
0.0	0.0	0.0	0.0	0.0	0.0	0.0	0.0
0.0	0.0	0.0	0.0	0.0	0.0	0.0	0.0
0.0	0.0	0.0	0.0	0.0	0.0	0.0	0.0

0.0	0.0	0.0	0.0	0.0	0.0	0.0	0.0
0.0	0.0	0.0	0.0	0.0	0.0	0.0	0.0
0.0	0.0	0.0	0.0	0.0	0.0	0.0	0.0
0.0	0.0	0.0	0.0	0.0	0.0	0.0	0.0
0.0	0.0	0.0	0.0	0.0	0.0	0.0	0.0
0.0	0.0	0.0	0.0	0.0	0.0	0.0	0.0
0.0	0.0	0.0	0.0	0.0	0.0	0.0	0.0
0.0	0.0	0.0	0.0	0.0	0.0	0.0	0.0
0.0	0.0	0.0	0.0	0.0	0.0	0.0	0.0
0.0	0.0	0.0	0.0	0.0	0.0	0.0	0.0
0.0	0.0	0.0	0.0	0.0	0.0	0.0	0.0
0.0	0.0	0.0	0.0	0.0	0.0	0.0	0.0
0.0	0.0	0.0	0.0	0.0	0.0	0.0	0.0
0.0	0.0	0.0	0.0	0.0	0.0	0.0	0.0
0.0	0.0	0.0	0.0	0.0	0.0	0.0	0.0
0.0	0.0	0.0	0.0	0.0	0.0	0.0	0.0
0.0	0.0	0.0	0.0	0.0	0.0	0.0	0.0
0.0	0.0	0.0	0.0	0.0	0.0	0.0	0.0
0.0	0.0	0.0	0.0	0.0	0.0	0.0	0.0
0.0	0.0	0.0	0.0	0.0	0.0	0.0	0.0
0.0	0.0	0.0	0.0	0.0	0.0	0.0	0.0
0.0	0.0	0.0	0.0	0.0	0.0	0.0	0.0
0.0	0.0	0.0	0.0	0.0	0.0	0.0	0.0
0.0	0.0	0.0	0.0	0.0	0.0	0.0	0.0
0.0	0.0	0.0	0.0	0.0	0.0	0.0	0.0
0.0	0.0	0.0	0.0	0.0	0.0	0.0	0.0
0.0	0.0	0.0	0.0	0.0	0.0	0.0	0.0
0.0	0.0	0.0	0.0	0.0	0.0	0.0	0.0
0.0	0.0	0.0	0.0	0.0	0.0	0.0	0.0
0.0	0.0	0.0	0.0	0.0	0.0	0.0	0.0
0.0	0.0	0.0	0.0	0.0	0.0	0.0	0.0
0.0	0.0	0.0	0.0	0.0	0.0	0.0	0.0
0.0	0.0	0.0	0.0	0.0	0.0	0.0	0.0
0.0	0.0	0.0	0.0	0.0	0.0	0.0	0.0
0.0	0.0	0.0	0.0	0.0	0.0	0.0	0.0
0.0	0.0	0.0	0.0	0.0	0.0	0.0	0.0
0.0	0.0	0.0	0.0	0.0	0.0	0.0	0.0
0.0	0.0	0.0	0.0	0.0	0.0	0.0	0.0
0.0	0.0	0.0	0.0	0.0	0.0	0.0	0.0
0.0	0.0	0.0	0.0	0.0	0.0	0.0	0.0
0.0	0.0	0.0	0.0	0.0	0.0	0.0	0.0
0.0	0.0	0.0	0.0	0.0	0.0	0.0	0.0
0.0	0.0	0.0	0.0	0.0	0.0	0.0	0.0
0.0	0.0	0.0	0.0	0.0	0.0	0.0	0.0
0.0	0.0	0.0	0.0	0.0	0.0	0.0	0.0
0.0	0.0	0.0	0.0	0.0	0.0	0.0	0.0
0.0	0.0	0.0	0.0	0.0	0.0	0.0	0.0

0.0	0.0	0.0	0.0	0.0	0.0	0.0	0.0
0.0	0.0	0.0	0.0	0.0	0.0	0.0	0.0
0.0	0.0	0.0	0.0	0.0	0.0	0.0	0.0
0.0	0.0	0.0	0.0	0.0	0.0	0.0	0.0
0.0	0.0	0.0	0.0	0.0	0.0	0.0	0.0
0.0	0.0	0.0	0.0	0.0	0.0	0.0	0.0
0.0	0.0	0.0	0.0	0.0	0.0	0.0	0.0
0.0	0.0	0.0	0.0	0.0	0.0	0.0	0.0
0.0	0.0	0.0	0.0	0.0	0.0	0.0	0.0
0.0	0.0	0.0	0.0	0.0	0.0	0.0	0.0
0.0	0.0	0.0	0.0	0.0	0.0	0.0	0.0
0.0	0.0	0.0	0.0	0.0	0.0	0.0	0.0
0.0	0.0	0.0	0.0	0.0	0.0	0.0	0.0
0.0	0.0	0.0	0.0	0.0	0.0	0.0	0.0
0.0	0.0	0.0	0.0	0.0	0.0	0.0	0.0
0.0	0.0	0.0	0.0	0.0	0.0	0.0	0.0
0.0	0.0	0.0	0.0	0.0	0.0	0.0	0.0
0.0	0.0	0.0	0.0	0.0	0.0	0.0	0.0
0.0	0.0	0.0	0.0	0.0	0.0	0.0	0.0
0.0	0.0	0.0	0.0	0.0	0.0	0.0	0.0
0.0	0.0	0.0	0.0	0.0	0.0	0.0	0.0
0.0	0.0	0.0	0.0	0.0	0.0	0.0	0.0
0.0	0.0	0.0	0.0	0.0	0.0	0.0	0.0
0.0	0.0	0.0	0.0	0.0	0.0	0.0	0.0
0.0	0.0	0.0	0.0	0.0	0.0	0.0	0.0
0.0	0.0	0.0	0.0	0.0	0.0	0.0	0.0
0.0	0.0	0.0	0.0	0.0	0.0	0.0	0.0
0.0	0.0	0.0	0.0	0.0	0.0	0.0	0.0
0.0	0.0	0.0	0.0	0.0	0.0	0.0	0.0
0.0	0.0	0.0	0.0	0.0	0.0	0.0	0.0
0.0	0.0	0.0	0.0	0.0	0.0	0.0	0.0
0.0	0.0	0.0	0.0	0.0	0.0	0.0	0.0
0.0	0.0	0.0	0.0	0.0	0.0	0.0	0.0
0.0	0.0	0.0	0.0	0.0	0.0	0.0	0.0
0.0	0.0	0.0	0.0	0.0	0.0	0.0	0.0
0.0	0.0	0.0	0.0	0.0	0.0	0.0	0.0
0.0	0.0	0.0	0.0	0.0	0.0	0.0	0.0
0.0	0.0	0.0	0.0	0.0	0.0	0.0	0.0
0.0	0.0	0.0	0.0	0.0	0.0	0.0	0.0
0.0	0.0	0.0	0.0	0.0	0.0	0.0	0.0
0.0	0.0	0.0	0.0	0.0	0.0	0.0	0.0
0.0	0.0	0.0	0.0	0.0	0.0	0.0	0.0
0.0	0.0	0.0	0.0	0.0	0.0	0.0	0.0
0.0	0.0	0.0	0.0	0.0	0.0	0.0	0.0
0.0	0.0	0.0	0.0	0.0	0.0	0.0	0.0
0.0	0.0	0.0	0.0	0.0	0.0	0.0	0.0
0.0	0.0	0.0	0.0	0.0	0.0	0.0	0.0

0.0	0.0	0.0	0.0	0.0	0.0	0.0	0.0
0.0	0.0	0.0	0.0	0.0	0.0	0.0	0.0
0.0	0.0	0.0	0.0	0.0	0.0	0.0	0.0
0.0	0.0	0.0	0.0	0.0	0.0	0.0	0.0
0.0	0.0	0.0	0.0	0.0	0.0	0.0	0.0
0.0	0.0	0.0	0.0	0.0	0.0	0.0	0.0
0.0	0.0	0.0	0.0	0.0	0.0	0.0	0.0
0.0	0.0	0.0	0.0	0.0	0.0	0.0	0.0
0.0	0.0	0.0	0.0	0.0	0.0	0.0	0.0
0.0	0.0	0.0	0.0	0.0	0.0	0.0	0.0
0.0	0.0	0.0	0.0	0.0	0.0	0.0	0.0
0.0	0.0	0.0	0.0	0.0	0.0	0.0	0.0
0.0	0.0	0.0	0.0	0.0	0.0	0.0	0.0
0.0	0.0	0.0	0.0	0.0	0.0	0.0	0.0
0.0	0.0	0.0	0.0	0.0	0.0	0.0	0.0
0.0	0.0	0.0	0.0	0.0	0.0	0.0	0.0
0.0	0.0	0.0	0.0	0.0	0.0	0.0	0.0
0.0	0.0	0.0	0.0	0.0	0.0	0.0	0.0
0.0	0.0	0.0	0.0	0.0	0.0	0.0	0.0
0.0	0.0	0.0	0.0	0.0	0.0	0.0	0.0
0.0	0.0	0.0	0.0	0.0	0.0	0.0	0.0
0.0	0.0	0.0	0.0	0.0	0.0	0.0	0.0
0.0	0.0	0.0	0.0	0.0	0.0	0.0	0.0
0.0	0.0	0.0	0.0	0.0	0.0	0.0	0.0
0.0	0.0	0.0	0.0	0.0	0.0	0.0	0.0
0.0	0.0	0.0	0.0	0.0	0.0	0.0	0.0
0.0	0.0	0.0	0.0	0.0	0.0	0.0	0.0
0.0	0.0	0.0	0.0	0.0	0.0	0.0	0.0
0.0	0.0	0.0	0.0	0.0	0.0	0.0	0.0
0.0	0.0	0.0	0.0	0.0	0.0	0.0	0.0
0.0	0.0	0.0	0.0	0.0	0.0	0.0	0.0
0.0	0.0	0.0	0.0	0.0	0.0	0.0	0.0
0.0	0.0	0.0	0.0	0.0	0.0	0.0	0.0
0.0	0.0	0.0	0.0	0.0	0.0	0.0	0.0
0.0	0.0	0.0	0.0	0.0	0.0	0.0	0.0
0.0	0.0	0.0	0.0	0.0	0.0	0.0	0.0
0.0	0.0	0.0	0.0	0.0	0.0	0.0	0.0
0.0	0.0	0.0	0.0	0.0	0.0	0.0	0.0
0.0	0.0	0.0	0.0	0.0	0.0	0.0	0.0
0.0	0.0	0.0	0.0	0.0	0.0	0.0	0.0
0.0	0.0	0.0	0.0	0.0	0.0	0.0	0.0
0.0	0.0	0.0	0.0	0.0	0.0	0.0	0.0
0.0	0.0	0.0	0.0	0.0	0.0	0.0	0.0
0.0	0.0	0.0	0.0	0.0	0.0	0.0	0.0
0.0	0.0	0.0	0.0	0.0	0.0	0.0	0.0
0.0	0.0	0.0	0.0	0.0	0.0	0.0	0.0
0.0	0.0	0.0	0.0	0.0	0.0	0.0	0.0
0.0	0.0	0.0	0.0	0.0	0.0	0.0	0.0

0.0	0.0	0.0	0.0	0.0	0.0	0.0	0.0
0.0	0.0	0.0	0.0	0.0	0.0	0.0	0.0
0.0	0.0	0.0	0.0	0.0	0.0	0.0	0.0
0.0	0.0	0.0	0.0	0.0	0.0	0.0	0.0
0.0	0.0	0.0	0.0	0.0	0.0	0.0	0.0
0.0	0.0	0.0	0.0	0.0	0.0	0.0	0.0
0.0	0.0	0.0	0.0	0.0	0.0	0.0	0.0
0.0	0.0	0.0	0.0	0.0	0.0	0.0	0.0
0.0	0.0	0.0	0.0	0.0	0.0	0.0	0.0
0.0	0.0	0.0	0.0	0.0	0.0	0.0	0.0
0.0	0.0	0.0	0.0	0.0	0.0	0.0	0.0
0.0	0.0	0.0	0.0	0.0	0.0	0.0	0.0
0.0	0.0	0.0	0.0	0.0	0.0	0.0	0.0
0.0	0.0	0.0	0.0	0.0	0.0	0.0	0.0
0.0	0.0	0.0	0.0	0.0	0.0	0.0	0.0
0.0	0.0	0.0	0.0	0.0	0.0	0.0	0.0
0.0	0.0	0.0	0.0	0.0	0.0	0.0	0.0
0.0	0.0	0.0	0.0	0.0	0.0	0.0	0.0
0.0	0.0	0.0	0.0	0.0	0.0	0.0	0.0
0.0	0.0	0.0	0.0	0.0	0.0	0.0	0.0
0.0	0.0	0.0	0.0	0.0	0.0	0.0	0.0
0.0	0.0	0.0	0.0	0.0	0.0	0.0	0.0
0.0	0.0	0.0	0.0	0.0	0.0	0.0	0.0
22	60	49	47	18	58	49	64
1	1	1	1	1	1	1	1
19.4	30.25453437	26	42.6	20.5	26.07924077	38.4	21.9

11635.TW.DU	11635.TW.CE.	11635.TW.AU	11635.CIR52.(	11635.CIR19.(	11635.TW.CT.	11635.CIR23.(	11635.TW.BN
4.0	8677.0	7.0	219.0	2.0	723.0	4696.0	13.0
95.0	0.0	0.0	147.0	8.0	0.0	0.0	71.0
0.0	0.0	0.0	1.0	1.0	0.0	0.0	4.0
0.0	0.0	256.0	0.0	2.0	0.0	0.0	150.0
194.0	2.0	520.0	0.0	820.0	1.0	0.0	0.0
547.0	0.0	826.0	0.0	121.0	0.0	0.0	1954.0
0.0	0.0	0.0	0.0	0.0	0.0	0.0	0.0
0.0	0.0	0.0	0.0	0.0	0.0	0.0	0.0
0.0	0.0	0.0	0.0	0.0	0.0	0.0	0.0
0.0	1.0	46.0	0.0	4.0	0.0	0.0	0.0
0.0	0.0	252.0	1180.0	44.0	0.0	261.0	276.0
0.0	0.0	0.0	0.0	0.0	0.0	0.0	0.0
0.0	17.0	11.0	0.0	546.0	0.0	0.0	0.0
0.0	0.0	0.0	0.0	93.0	15.0	0.0	0.0
0.0	0.0	113.0	0.0	40.0	3.0	0.0	0.0
695.0	3.0	709.0	0.0	243.0	4.0	0.0	858.0
4.0	0.0	32.0	0.0	0.0	0.0	0.0	0.0
0.0	0.0	0.0	4.0	0.0	47.0	3.0	0.0
36.0	28.0	192.0	671.0	2.0	1.0	2.0	51.0
181.0	1.0	863.0	554.0	2.0	0.0	0.0	0.0
75.0	0.0	151.0	0.0	0.0	0.0	0.0	157.0
0.0	0.0	0.0	0.0	0.0	0.0	0.0	0.0
23.0	0.0	14.0	0.0	0.0	0.0	0.0	0.0
17.0	3.0	0.0	0.0	8.0	0.0	0.0	0.0
0.0	0.0	0.0	0.0	0.0	0.0	0.0	0.0
40.0	0.0	41.0	0.0	123.0	0.0	0.0	2.0
0.0	0.0	0.0	0.0	0.0	0.0	0.0	0.0
58.0	0.0	24.0	0.0	145.0	0.0	48.0	125.0
0.0	0.0	0.0	0.0	0.0	1.0	0.0	0.0
0.0	0.0	60.0	0.0	23.0	0.0	0.0	0.0
0.0	8.0	31.0	77.0	1.0	25.0	2.0	33.0
0.0	1.0	16.0	0.0	0.0	1.0	0.0	46.0
0.0	0.0	92.0	0.0	0.0	0.0	0.0	0.0
0.0	0.0	0.0	0.0	39.0	0.0	0.0	0.0
47.0	7.0	18.0	0.0	24.0	13.0	0.0	12.0
0.0	0.0	0.0	0.0	0.0	0.0	0.0	0.0
0.0	0.0	1.0	0.0	0.0	0.0	0.0	0.0
44.0	16.0	50.0	0.0	0.0	2.0	0.0	52.0
0.0	0.0	0.0	0.0	4.0	0.0	0.0	0.0
0.0	2.0	28.0	0.0	0.0	2.0	0.0	27.0
53.0	0.0	5.0	0.0	304.0	4.0	464.0	0.0
0.0	0.0	28.0	0.0	0.0	0.0	0.0	0.0
5.0	5.0	17.0	0.0	0.0	3.0	0.0	6.0
257.0	1.0	476.0	412.0	0.0	1.0	0.0	176.0
10.0	0.0	0.0	0.0	26.0	0.0	0.0	1.0
0.0	4.0	63.0	77.0	0.0	3.0	0.0	56.0

1.0	0.0	2.0	90.0	5.0	0.0	27.0	1.0
0.0	0.0	137.0	0.0	621.0	189.0	0.0	0.0
0.0	0.0	0.0	0.0	0.0	0.0	0.0	0.0
0.0	0.0	0.0	0.0	0.0	0.0	0.0	0.0
0.0	0.0	0.0	0.0	0.0	0.0	0.0	0.0
73.0	0.0	21.0	0.0	0.0	0.0	0.0	26.0
5.0	0.0	0.0	0.0	0.0	0.0	0.0	6.0
0.0	0.0	0.0	0.0	0.0	0.0	0.0	0.0
0.0	3.0	0.0	0.0	576.0	9.0	0.0	0.0
0.0	0.0	0.0	0.0	0.0	0.0	0.0	0.0
0.0	2.0	0.0	0.0	0.0	9.0	0.0	0.0
3.0	0.0	1.0	0.0	3.0	6.0	0.0	0.0
0.0	0.0	0.0	0.0	0.0	0.0	0.0	0.0
0.0	0.0	0.0	0.0	0.0	0.0	0.0	0.0
8.0	0.0	0.0	0.0	0.0	0.0	0.0	0.0
0.0	0.0	0.0	0.0	0.0	0.0	0.0	0.0
4.0	0.0	3.0	0.0	2.0	0.0	0.0	0.0
0.0	0.0	0.0	0.0	0.0	0.0	0.0	0.0
0.0	3.0	0.0	136.0	1.0	3.0	0.0	124.0
0.0	0.0	23.0	0.0	0.0	0.0	0.0	0.0
0.0	0.0	0.0	0.0	0.0	0.0	0.0	0.0
0.0	0.0	10.0	0.0	0.0	0.0	0.0	0.0
0.0	0.0	0.0	0.0	0.0	0.0	0.0	0.0
0.0	0.0	77.0	0.0	0.0	0.0	0.0	77.0
0.0	1.0	0.0	0.0	0.0	0.0	0.0	0.0
115.0	0.0	0.0	0.0	1.0	0.0	0.0	0.0
0.0	0.0	0.0	0.0	0.0	0.0	0.0	0.0
0.0	0.0	0.0	0.0	0.0	0.0	0.0	0.0
0.0	0.0	0.0	0.0	0.0	0.0	0.0	0.0
0.0	0.0	0.0	0.0	0.0	0.0	0.0	0.0
0.0	0.0	0.0	0.0	0.0	0.0	0.0	0.0
0.0	0.0	0.0	0.0	0.0	0.0	0.0	0.0
0.0	0.0	9.0	0.0	0.0	0.0	0.0	0.0
0.0	0.0	0.0	0.0	0.0	0.0	0.0	0.0
7.0	9.0	129.0	0.0	0.0	0.0	0.0	0.0
125.0	0.0	18.0	0.0	0.0	5.0	0.0	0.0
660.0	0.0	231.0	0.0	10.0	0.0	0.0	0.0
0.0	0.0	0.0	0.0	0.0	0.0	0.0	0.0
0.0	0.0	0.0	1534.0	946.0	6.0	0.0	448.0
47.0	0.0	9.0	0.0	0.0	0.0	0.0	0.0
0.0	0.0	0.0	0.0	0.0	0.0	0.0	0.0
0.0	0.0	0.0	0.0	0.0	0.0	0.0	0.0
95.0	0.0	36.0	0.0	102.0	2.0	45.0	0.0
0.0	0.0	39.0	12.0	7.0	0.0	0.0	35.0
0.0	0.0	14.0	12.0	4.0	0.0	0.0	0.0
0.0	0.0	0.0	0.0	0.0	0.0	0.0	0.0
23.0	1.0	3.0	0.0	1.0	0.0	0.0	0.0

153.0	4.0	20.0	0.0	13.0	0.0	0.0	15.0
5.0	0.0	3.0	0.0	0.0	0.0	0.0	0.0
0.0	1.0	365.0	0.0	728.0	0.0	17.0	28.0
0.0	0.0	0.0	0.0	0.0	0.0	0.0	0.0
112.0	0.0	2.0	0.0	5.0	0.0	17.0	0.0
0.0	0.0	0.0	0.0	0.0	0.0	0.0	0.0
30.0	0.0	43.0	136.0	43.0	0.0	0.0	251.0
0.0	0.0	0.0	0.0	0.0	0.0	0.0	0.0
1.0	0.0	0.0	0.0	0.0	0.0	0.0	4.0
86.0	0.0	0.0	0.0	0.0	0.0	0.0	0.0
63.0	0.0	24.0	0.0	44.0	0.0	0.0	0.0
0.0	0.0	0.0	0.0	0.0	0.0	0.0	0.0
0.0	0.0	11.0	0.0	0.0	0.0	0.0	0.0
0.0	0.0	0.0	0.0	0.0	0.0	0.0	0.0
2.0	5.0	25.0	0.0	50.0	4.0	0.0	49.0
13.0	0.0	0.0	0.0	0.0	0.0	0.0	0.0
0.0	0.0	0.0	0.0	0.0	0.0	0.0	0.0
0.0	0.0	0.0	0.0	0.0	0.0	0.0	0.0
0.0	0.0	0.0	0.0	0.0	0.0	0.0	0.0
0.0	0.0	0.0	0.0	0.0	0.0	0.0	0.0
21.0	1.0	37.0	0.0	1.0	1.0	0.0	8.0
26.0	1.0	0.0	0.0	0.0	0.0	0.0	53.0
0.0	0.0	7.0	0.0	54.0	0.0	0.0	0.0
0.0	0.0	0.0	0.0	0.0	0.0	0.0	0.0
0.0	0.0	0.0	0.0	0.0	0.0	0.0	0.0
0.0	0.0	0.0	0.0	0.0	0.0	0.0	0.0
0.0	0.0	6.0	0.0	0.0	0.0	0.0	0.0
0.0	0.0	0.0	0.0	0.0	0.0	0.0	0.0
219.0	0.0	0.0	1.0	0.0	0.0	0.0	1.0
0.0	0.0	0.0	0.0	0.0	0.0	0.0	314.0
0.0	0.0	0.0	0.0	0.0	0.0	0.0	0.0
153.0	0.0	0.0	0.0	0.0	0.0	0.0	0.0
0.0	0.0	27.0	0.0	0.0	0.0	0.0	0.0
0.0	0.0	0.0	0.0	0.0	0.0	0.0	0.0
0.0	0.0	0.0	0.0	0.0	0.0	0.0	0.0
76.0	0.0	31.0	0.0	691.0	0.0	0.0	0.0
0.0	0.0	0.0	0.0	0.0	0.0	0.0	0.0
0.0	0.0	22.0	0.0	0.0	0.0	0.0	13.0
237.0	0.0	61.0	0.0	16.0	0.0	0.0	0.0
0.0	0.0	0.0	0.0	0.0	0.0	0.0	0.0
0.0	0.0	0.0	0.0	0.0	0.0	0.0	0.0
461.0	0.0	43.0	0.0	106.0	0.0	0.0	168.0
892.0	0.0	1020.0	0.0	0.0	0.0	0.0	0.0
77.0	0.0	0.0	0.0	9.0	0.0	0.0	0.0
5.0	0.0	0.0	229.0	0.0	0.0	0.0	0.0
0.0	0.0	0.0	0.0	0.0	0.0	0.0	0.0
11.0	0.0	4.0	0.0	36.0	0.0	0.0	0.0

0.0	0.0	0.0	0.0	0.0	0.0	0.0	0.0
0.0	0.0	0.0	0.0	0.0	0.0	0.0	0.0
0.0	0.0	0.0	0.0	0.0	0.0	0.0	0.0
0.0	0.0	0.0	0.0	0.0	0.0	0.0	0.0
0.0	2.0	8.0	0.0	9.0	0.0	9.0	0.0
0.0	0.0	0.0	0.0	0.0	0.0	0.0	0.0
0.0	0.0	0.0	0.0	0.0	0.0	0.0	0.0
0.0	0.0	0.0	24.0	0.0	0.0	0.0	0.0
1.0	0.0	1.0	0.0	10.0	0.0	0.0	6.0
0.0	0.0	0.0	0.0	0.0	0.0	0.0	0.0
21.0	0.0	27.0	0.0	0.0	0.0	0.0	39.0
0.0	0.0	0.0	0.0	0.0	0.0	0.0	1.0
0.0	0.0	6.0	12.0	0.0	0.0	0.0	3.0
3.0	0.0	35.0	0.0	0.0	0.0	0.0	0.0
13.0	0.0	0.0	0.0	50.0	3.0	0.0	0.0
0.0	0.0	0.0	0.0	0.0	0.0	0.0	0.0
0.0	0.0	0.0	0.0	0.0	0.0	0.0	0.0
1.0	0.0	1.0	0.0	0.0	0.0	34.0	17.0
0.0	3.0	17.0	84.0	0.0	7.0	0.0	13.0
0.0	0.0	0.0	0.0	0.0	0.0	0.0	0.0
0.0	0.0	0.0	0.0	0.0	0.0	0.0	0.0
24.0	0.0	0.0	0.0	0.0	0.0	0.0	0.0
0.0	0.0	3.0	0.0	2.0	0.0	4.0	0.0
0.0	0.0	2.0	0.0	0.0	0.0	0.0	0.0
7.0	0.0	0.0	0.0	0.0	0.0	0.0	0.0
1.0	0.0	0.0	0.0	0.0	0.0	0.0	35.0
0.0	0.0	0.0	0.0	0.0	0.0	0.0	0.0
0.0	0.0	14.0	0.0	0.0	0.0	0.0	0.0
0.0	0.0	0.0	0.0	0.0	0.0	0.0	0.0
0.0	0.0	0.0	0.0	0.0	0.0	0.0	0.0
0.0	2.0	0.0	0.0	7.0	2.0	0.0	0.0
0.0	0.0	3.0	0.0	0.0	0.0	0.0	0.0
0.0	0.0	0.0	21.0	0.0	0.0	0.0	0.0
36.0	0.0	0.0	0.0	0.0	0.0	0.0	0.0
0.0	0.0	14.0	0.0	0.0	0.0	0.0	11.0
0.0	0.0	0.0	0.0	0.0	0.0	0.0	0.0
13.0	0.0	0.0	0.0	0.0	0.0	16.0	0.0
0.0	0.0	1.0	0.0	3.0	0.0	0.0	0.0
0.0	0.0	9.0	0.0	15.0	0.0	0.0	21.0
7.0	0.0	0.0	0.0	71.0	0.0	0.0	13.0
8.0	0.0	5.0	11.0	9.0	0.0	0.0	6.0
15.0	0.0	13.0	0.0	0.0	0.0	0.0	0.0
0.0	0.0	17.0	0.0	0.0	0.0	0.0	30.0
0.0	0.0	0.0	0.0	0.0	0.0	0.0	0.0
10.0	0.0	91.0	0.0	1.0	0.0	140.0	14.0
0.0	0.0	0.0	0.0	0.0	0.0	0.0	0.0
0.0	0.0	0.0	0.0	0.0	0.0	0.0	0.0

0.0	0.0	0.0	0.0	0.0	0.0	0.0	0.0
0.0	0.0	3.0	0.0	1.0	1.0	0.0	0.0
0.0	0.0	8.0	0.0	15.0	0.0	0.0	0.0
30.0	0.0	9.0	0.0	0.0	0.0	0.0	0.0
0.0	0.0	11.0	0.0	4.0	0.0	0.0	0.0
0.0	0.0	0.0	0.0	0.0	0.0	0.0	0.0
0.0	0.0	0.0	0.0	0.0	0.0	0.0	0.0
0.0	0.0	8.0	0.0	0.0	0.0	1.0	15.0
4.0	0.0	3.0	384.0	2.0	0.0	31.0	19.0
0.0	0.0	0.0	0.0	0.0	0.0	0.0	0.0
11.0	0.0	2.0	0.0	0.0	0.0	0.0	0.0
0.0	0.0	3.0	0.0	3.0	0.0	0.0	10.0
0.0	0.0	4.0	0.0	0.0	0.0	0.0	0.0
3.0	0.0	0.0	65.0	2.0	0.0	28.0	9.0
1.0	0.0	0.0	0.0	0.0	0.0	0.0	0.0
0.0	1.0	5.0	0.0	0.0	1.0	0.0	0.0
0.0	0.0	0.0	0.0	0.0	0.0	0.0	0.0
0.0	0.0	0.0	0.0	0.0	0.0	0.0	0.0
0.0	0.0	0.0	0.0	0.0	0.0	0.0	0.0
0.0	0.0	0.0	0.0	0.0	0.0	0.0	5.0
9.0	5.0	0.0	0.0	0.0	0.0	0.0	0.0
0.0	0.0	0.0	0.0	0.0	0.0	0.0	3.0
0.0	0.0	1.0	0.0	0.0	0.0	0.0	15.0
0.0	0.0	0.0	0.0	0.0	0.0	0.0	0.0
0.0	0.0	0.0	0.0	0.0	0.0	0.0	0.0
0.0	0.0	0.0	0.0	0.0	0.0	0.0	0.0
0.0	1.0	0.0	0.0	0.0	0.0	0.0	0.0
0.0	0.0	0.0	0.0	0.0	0.0	0.0	0.0
2.0	0.0	11.0	0.0	54.0	0.0	0.0	0.0
0.0	0.0	0.0	0.0	0.0	0.0	0.0	0.0
0.0	0.0	0.0	0.0	0.0	0.0	0.0	0.0
2.0	1.0	1.0	0.0	36.0	0.0	0.0	0.0
0.0	0.0	0.0	0.0	0.0	0.0	0.0	0.0
0.0	0.0	0.0	0.0	0.0	0.0	0.0	0.0
0.0	0.0	0.0	0.0	0.0	0.0	0.0	0.0
0.0	0.0	0.0	0.0	0.0	0.0	0.0	0.0
0.0	0.0	0.0	0.0	0.0	0.0	0.0	0.0
0.0	1.0	0.0	0.0	0.0	0.0	0.0	0.0
0.0	0.0	0.0	0.0	0.0	0.0	0.0	0.0
0.0	3.0	0.0	0.0	0.0	0.0	0.0	0.0
5.0	0.0	2.0	0.0	4.0	0.0	0.0	5.0
0.0	0.0	2.0	0.0	0.0	0.0	0.0	0.0
0.0	0.0	0.0	0.0	0.0	0.0	0.0	0.0
0.0	0.0	0.0	0.0	0.0	0.0	0.0	0.0
0.0	0.0	0.0	0.0	0.0	0.0	0.0	0.0
0.0	0.0	0.0	11.0	0.0	0.0	0.0	0.0
0.0	0.0	5.0	0.0	0.0	0.0	0.0	0.0

0.0	0.0	1.0	0.0	3.0	0.0	0.0	0.0
0.0	0.0	2.0	0.0	0.0	0.0	0.0	0.0
0.0	0.0	0.0	0.0	1.0	0.0	0.0	0.0
23.0	0.0	0.0	0.0	0.0	0.0	0.0	4.0
0.0	0.0	0.0	0.0	0.0	0.0	0.0	0.0
0.0	0.0	0.0	0.0	0.0	0.0	0.0	0.0
7.0	0.0	2.0	0.0	0.0	0.0	0.0	0.0
0.0	0.0	3.0	0.0	54.0	0.0	0.0	0.0
0.0	0.0	0.0	0.0	0.0	0.0	0.0	0.0
0.0	0.0	0.0	0.0	0.0	0.0	0.0	0.0
0.0	0.0	0.0	0.0	0.0	0.0	0.0	0.0
0.0	0.0	0.0	0.0	0.0	0.0	0.0	0.0
0.0	0.0	0.0	0.0	0.0	0.0	0.0	0.0
0.0	0.0	8.0	0.0	0.0	1.0	0.0	0.0
0.0	0.0	0.0	0.0	0.0	0.0	0.0	0.0
0.0	0.0	0.0	0.0	0.0	0.0	0.0	0.0
8.0	0.0	0.0	0.0	4.0	7.0	0.0	0.0
0.0	0.0	0.0	0.0	0.0	0.0	0.0	0.0
3.0	0.0	2.0	0.0	0.0	0.0	0.0	0.0
0.0	0.0	0.0	0.0	0.0	0.0	0.0	0.0
0.0	0.0	0.0	0.0	0.0	0.0	0.0	0.0
18.0	0.0	20.0	0.0	0.0	0.0	0.0	0.0
2.0	0.0	3.0	0.0	0.0	0.0	0.0	1.0
0.0	0.0	0.0	0.0	0.0	0.0	0.0	0.0
0.0	0.0	0.0	0.0	0.0	0.0	7.0	0.0
0.0	0.0	0.0	0.0	0.0	0.0	0.0	0.0
7.0	0.0	0.0	0.0	4.0	0.0	0.0	1.0
0.0	0.0	0.0	0.0	0.0	0.0	0.0	0.0
2.0	0.0	1.0	0.0	0.0	0.0	0.0	0.0
0.0	0.0	0.0	0.0	0.0	0.0	0.0	0.0
0.0	0.0	0.0	0.0	0.0	0.0	0.0	3.0
3.0	0.0	0.0	0.0	0.0	0.0	0.0	0.0
0.0	0.0	0.0	0.0	0.0	0.0	0.0	0.0
0.0	0.0	0.0	0.0	0.0	0.0	0.0	0.0
0.0	0.0	0.0	0.0	0.0	0.0	0.0	0.0
0.0	0.0	13.0	0.0	0.0	0.0	0.0	0.0
3.0	0.0	1.0	0.0	3.0	0.0	0.0	0.0
20.0	0.0	5.0	0.0	29.0	13.0	0.0	0.0
0.0	0.0	0.0	0.0	0.0	0.0	0.0	0.0
0.0	0.0	0.0	0.0	0.0	0.0	0.0	0.0
0.0	0.0	0.0	0.0	0.0	0.0	0.0	0.0
0.0	0.0	0.0	6.0	0.0	0.0	1.0	0.0
6.0	0.0	3.0	0.0	1.0	0.0	0.0	6.0
7.0	0.0	2.0	148.0	2.0	0.0	382.0	1.0
0.0	0.0	0.0	0.0	0.0	0.0	0.0	0.0
4.0	0.0	0.0	0.0	0.0	0.0	0.0	0.0
0.0	0.0	0.0	0.0	0.0	0.0	0.0	0.0

2.0	0.0	0.0	0.0	1.0	1.0	0.0	0.0
0.0	0.0	0.0	0.0	0.0	0.0	0.0	0.0
0.0	0.0	0.0	0.0	0.0	0.0	0.0	0.0
0.0	0.0	0.0	0.0	0.0	0.0	0.0	0.0
1.0	0.0	1.0	0.0	0.0	0.0	0.0	0.0
0.0	0.0	0.0	0.0	0.0	0.0	0.0	0.0
2.0	0.0	8.0	0.0	0.0	0.0	0.0	0.0
0.0	0.0	0.0	0.0	0.0	0.0	0.0	0.0
0.0	0.0	0.0	0.0	3.0	0.0	0.0	0.0
0.0	0.0	0.0	0.0	0.0	0.0	0.0	0.0
0.0	0.0	2.0	0.0	3.0	0.0	0.0	0.0
5.0	1.0	0.0	0.0	0.0	0.0	0.0	0.0
0.0	0.0	0.0	2.0	0.0	0.0	0.0	0.0
0.0	0.0	0.0	0.0	0.0	0.0	0.0	0.0
0.0	0.0	0.0	0.0	0.0	0.0	0.0	0.0
2.0	1.0	1.0	73.0	7.0	5.0	10.0	0.0
0.0	0.0	0.0	0.0	0.0	0.0	0.0	0.0
0.0	0.0	50.0	0.0	0.0	0.0	0.0	0.0
0.0	0.0	0.0	0.0	6.0	0.0	0.0	0.0
0.0	0.0	0.0	14.0	1.0	0.0	11.0	2.0
1.0	0.0	0.0	0.0	0.0	0.0	0.0	0.0
0.0	0.0	0.0	0.0	0.0	0.0	0.0	0.0
0.0	0.0	0.0	0.0	0.0	0.0	0.0	0.0
0.0	0.0	0.0	0.0	0.0	0.0	0.0	0.0
0.0	0.0	0.0	0.0	1.0	0.0	0.0	0.0
0.0	0.0	0.0	0.0	0.0	0.0	0.0	0.0
0.0	0.0	0.0	29.0	0.0	0.0	5.0	0.0
15.0	0.0	0.0	0.0	0.0	0.0	0.0	0.0
0.0	0.0	0.0	0.0	1.0	2.0	0.0	0.0
0.0	0.0	0.0	0.0	0.0	0.0	0.0	0.0
0.0	0.0	3.0	0.0	0.0	0.0	0.0	0.0
0.0	0.0	0.0	0.0	0.0	0.0	0.0	0.0
0.0	0.0	0.0	0.0	0.0	0.0	0.0	0.0
4.0	0.0	2.0	0.0	2.0	0.0	0.0	1.0
0.0	1.0	0.0	0.0	0.0	0.0	0.0	0.0
0.0	0.0	0.0	0.0	0.0	0.0	0.0	0.0
2.0	0.0	2.0	0.0	7.0	0.0	0.0	0.0
0.0	0.0	0.0	0.0	0.0	0.0	0.0	0.0
0.0	0.0	2.0	0.0	0.0	0.0	0.0	0.0
0.0	0.0	0.0	0.0	0.0	0.0	0.0	0.0
0.0	0.0	4.0	0.0	0.0	0.0	0.0	0.0
0.0	0.0	0.0	0.0	0.0	0.0	0.0	0.0
0.0	0.0	0.0	0.0	3.0	0.0	0.0	0.0
6.0	0.0	0.0	0.0	1.0	0.0	0.0	0.0
0.0	0.0	0.0	0.0	0.0	0.0	0.0	0.0
0.0	0.0	0.0	0.0	0.0	0.0	0.0	0.0
0.0	0.0	0.0	0.0	14.0	0.0	0.0	0.0

0.0	0.0	0.0	0.0	0.0	0.0	0.0	0.0
0.0	0.0	0.0	0.0	0.0	0.0	0.0	0.0
0.0	0.0	0.0	0.0	0.0	0.0	0.0	0.0
0.0	0.0	0.0	0.0	0.0	0.0	0.0	0.0
0.0	0.0	0.0	0.0	1.0	0.0	0.0	0.0
0.0	0.0	0.0	0.0	2.0	0.0	0.0	0.0
0.0	0.0	0.0	0.0	0.0	0.0	0.0	0.0
0.0	0.0	0.0	0.0	0.0	0.0	0.0	0.0
2.0	0.0	0.0	19.0	31.0	0.0	0.0	46.0
0.0	0.0	0.0	0.0	0.0	0.0	0.0	0.0
2.0	0.0	0.0	0.0	0.0	0.0	0.0	0.0
0.0	0.0	0.0	0.0	0.0	0.0	0.0	0.0
0.0	0.0	0.0	0.0	0.0	0.0	0.0	0.0
0.0	0.0	0.0	0.0	0.0	0.0	0.0	0.0
0.0	0.0	0.0	0.0	3.0	0.0	0.0	0.0
0.0	0.0	0.0	0.0	0.0	0.0	0.0	0.0
0.0	0.0	0.0	17.0	0.0	0.0	0.0	2.0
0.0	0.0	0.0	0.0	0.0	0.0	0.0	0.0
0.0	0.0	0.0	0.0	0.0	0.0	0.0	0.0
0.0	0.0	1.0	0.0	8.0	0.0	0.0	0.0
0.0	0.0	2.0	0.0	0.0	0.0	0.0	5.0
0.0	0.0	0.0	0.0	0.0	0.0	0.0	0.0
0.0	0.0	0.0	0.0	3.0	0.0	0.0	0.0
0.0	0.0	0.0	0.0	0.0	0.0	0.0	0.0
0.0	0.0	0.0	0.0	0.0	0.0	0.0	0.0
0.0	0.0	0.0	0.0	0.0	0.0	0.0	0.0
0.0	0.0	0.0	0.0	0.0	0.0	0.0	0.0
0.0	0.0	0.0	0.0	0.0	0.0	0.0	42.0
0.0	0.0	0.0	0.0	0.0	0.0	0.0	0.0
0.0	0.0	0.0	0.0	38.0	0.0	0.0	0.0
0.0	0.0	0.0	0.0	0.0	0.0	0.0	0.0
0.0	0.0	0.0	0.0	0.0	0.0	0.0	0.0
0.0	0.0	0.0	0.0	0.0	0.0	0.0	0.0
0.0	0.0	0.0	0.0	8.0	0.0	0.0	0.0
7.0	0.0	0.0	0.0	2.0	0.0	0.0	0.0
0.0	0.0	0.0	0.0	0.0	0.0	0.0	0.0
0.0	0.0	0.0	0.0	0.0	0.0	0.0	0.0
46.0	0.0	0.0	0.0	0.0	0.0	0.0	0.0
0.0	0.0	0.0	0.0	0.0	0.0	0.0	0.0
0.0	0.0	0.0	0.0	0.0	0.0	0.0	0.0
0.0	0.0	0.0	0.0	0.0	0.0	0.0	0.0
0.0	0.0	2.0	0.0	0.0	0.0	0.0	0.0
0.0	0.0	0.0	0.0	0.0	0.0	0.0	0.0
0.0	0.0	0.0	0.0	0.0	0.0	0.0	0.0
0.0	0.0	0.0	0.0	0.0	0.0	0.0	0.0
1.0	0.0	0.0	0.0	0.0	0.0	0.0	0.0
0.0	0.0	0.0	0.0	0.0	0.0	0.0	0.0

0.0	0.0	0.0	0.0	0.0	0.0	0.0	2.0
0.0	0.0	0.0	0.0	0.0	0.0	0.0	0.0
0.0	0.0	0.0	0.0	0.0	0.0	0.0	0.0
0.0	0.0	0.0	0.0	0.0	0.0	0.0	0.0
0.0	0.0	0.0	0.0	0.0	0.0	0.0	0.0
94.0	0.0	0.0	1271.0	113.0	0.0	1980.0	81.0
0.0	0.0	0.0	0.0	0.0	0.0	0.0	0.0
0.0	0.0	0.0	0.0	2.0	0.0	0.0	0.0
5.0	0.0	3.0	0.0	0.0	0.0	0.0	0.0
3.0	0.0	6.0	0.0	2.0	1.0	0.0	0.0
2.0	0.0	1.0	0.0	5.0	0.0	0.0	16.0
0.0	0.0	0.0	0.0	0.0	0.0	0.0	0.0
0.0	0.0	0.0	0.0	0.0	0.0	0.0	0.0
0.0	0.0	0.0	0.0	0.0	0.0	0.0	0.0
0.0	0.0	3.0	0.0	0.0	0.0	0.0	0.0
0.0	0.0	0.0	0.0	0.0	0.0	0.0	0.0
0.0	0.0	8.0	0.0	0.0	0.0	0.0	0.0
0.0	0.0	0.0	0.0	0.0	0.0	0.0	8.0
0.0	0.0	0.0	0.0	0.0	0.0	0.0	0.0
0.0	0.0	0.0	0.0	0.0	0.0	0.0	0.0
0.0	0.0	0.0	0.0	0.0	0.0	0.0	0.0
0.0	0.0	0.0	0.0	0.0	0.0	0.0	0.0
0.0	0.0	0.0	0.0	0.0	0.0	0.0	0.0
0.0	0.0	0.0	0.0	0.0	0.0	0.0	0.0
0.0	0.0	0.0	0.0	0.0	0.0	0.0	2.0
0.0	0.0	0.0	0.0	0.0	0.0	0.0	0.0
0.0	0.0	0.0	0.0	0.0	0.0	0.0	0.0
0.0	0.0	0.0	0.0	0.0	0.0	0.0	0.0
0.0	0.0	0.0	0.0	0.0	0.0	0.0	0.0
0.0	0.0	4.0	0.0	0.0	0.0	0.0	0.0
0.0	0.0	0.0	0.0	0.0	0.0	0.0	0.0
0.0	0.0	0.0	0.0	0.0	0.0	0.0	0.0
0.0	0.0	0.0	3.0	0.0	0.0	0.0	0.0
0.0	0.0	0.0	0.0	0.0	0.0	0.0	0.0
0.0	0.0	0.0	0.0	0.0	0.0	0.0	0.0
0.0	0.0	1.0	0.0	0.0	0.0	0.0	0.0
38.0	1.0	34.0	0.0	0.0	0.0	0.0	0.0
0.0	0.0	0.0	0.0	0.0	0.0	0.0	0.0
0.0	2.0	0.0	0.0	0.0	0.0	0.0	0.0
0.0	0.0	0.0	0.0	0.0	0.0	0.0	0.0
0.0	0.0	0.0	2.0	0.0	224.0	0.0	0.0
0.0	0.0	314.0	0.0	0.0	0.0	0.0	0.0
0.0	0.0	0.0	0.0	0.0	0.0	0.0	0.0
0.0	0.0	0.0	0.0	0.0	0.0	0.0	0.0
0.0	0.0	0.0	0.0	0.0	0.0	0.0	0.0
0.0	0.0	0.0	0.0	0.0	0.0	0.0	0.0
1.0	2.0	0.0	0.0	0.0	1.0	0.0	0.0

0.0	0.0	0.0	0.0	0.0	0.0	19.0	2.0
0.0	0.0	0.0	0.0	0.0	0.0	0.0	2.0
0.0	0.0	0.0	4.0	0.0	0.0	0.0	0.0
1.0	0.0	0.0	10.0	0.0	0.0	18.0	0.0
0.0	0.0	0.0	0.0	0.0	0.0	0.0	0.0
0.0	0.0	0.0	0.0	0.0	0.0	0.0	0.0
0.0	0.0	2.0	0.0	0.0	0.0	0.0	0.0
0.0	0.0	0.0	0.0	0.0	0.0	0.0	0.0
0.0	0.0	0.0	0.0	0.0	0.0	0.0	0.0
0.0	0.0	0.0	0.0	0.0	0.0	0.0	0.0
0.0	0.0	0.0	0.0	0.0	0.0	0.0	0.0
0.0	0.0	0.0	0.0	0.0	0.0	0.0	0.0
0.0	0.0	10.0	2.0	0.0	0.0	0.0	0.0
0.0	0.0	0.0	0.0	0.0	0.0	0.0	0.0
0.0	0.0	0.0	0.0	0.0	0.0	0.0	0.0
0.0	0.0	0.0	0.0	0.0	0.0	0.0	0.0
0.0	0.0	0.0	0.0	0.0	0.0	0.0	0.0
0.0	0.0	0.0	0.0	0.0	0.0	0.0	0.0
97.0	0.0	3.0	109.0	0.0	0.0	0.0	0.0
0.0	0.0	0.0	0.0	0.0	0.0	0.0	0.0
55.0	0.0	3.0	0.0	0.0	0.0	0.0	0.0
0.0	0.0	4.0	0.0	0.0	0.0	0.0	0.0
0.0	0.0	0.0	0.0	0.0	0.0	0.0	0.0
0.0	0.0	0.0	0.0	0.0	3.0	0.0	0.0
0.0	0.0	0.0	0.0	0.0	0.0	0.0	0.0
0.0	0.0	0.0	0.0	0.0	0.0	0.0	0.0
0.0	0.0	0.0	0.0	0.0	0.0	0.0	0.0
0.0	0.0	0.0	0.0	0.0	0.0	0.0	0.0
0.0	0.0	0.0	0.0	0.0	0.0	0.0	33.0
0.0	0.0	0.0	0.0	0.0	0.0	0.0	0.0
18.0	0.0	0.0	0.0	0.0	0.0	0.0	0.0
9.0	0.0	0.0	0.0	0.0	0.0	0.0	0.0
0.0	0.0	0.0	0.0	0.0	0.0	0.0	0.0
0.0	0.0	0.0	0.0	0.0	0.0	0.0	0.0
0.0	0.0	3.0	0.0	50.0	0.0	0.0	0.0
0.0	0.0	0.0	0.0	0.0	0.0	0.0	0.0
0.0	0.0	6.0	0.0	0.0	0.0	0.0	0.0
0.0	0.0	0.0	0.0	0.0	0.0	0.0	14.0
0.0	0.0	0.0	0.0	0.0	1.0	0.0	0.0
1.0	0.0	2.0	0.0	0.0	0.0	0.0	0.0
0.0	0.0	0.0	0.0	0.0	0.0	0.0	0.0
0.0	0.0	0.0	0.0	0.0	0.0	0.0	0.0
0.0	0.0	0.0	0.0	0.0	0.0	0.0	0.0
0.0	0.0	0.0	1.0	0.0	0.0	0.0	0.0
43.0	1.0	0.0	95.0	1.0	0.0	0.0	0.0
3.0	0.0	0.0	0.0	0.0	0.0	0.0	0.0
0.0	0.0	0.0	0.0	0.0	0.0	0.0	1.0

0.0	0.0	0.0	0.0	0.0	0.0	0.0	0.0
0.0	0.0	2.0	0.0	6.0	0.0	0.0	2.0
0.0	0.0	0.0	0.0	1.0	0.0	0.0	0.0
0.0	1.0	2.0	0.0	1.0	0.0	0.0	0.0
0.0	0.0	1.0	0.0	14.0	0.0	0.0	0.0
0.0	0.0	2.0	0.0	0.0	0.0	0.0	0.0
0.0	0.0	0.0	0.0	0.0	0.0	0.0	0.0
0.0	0.0	0.0	0.0	0.0	0.0	0.0	0.0
0.0	0.0	0.0	0.0	0.0	0.0	0.0	0.0
0.0	0.0	2.0	0.0	0.0	0.0	0.0	0.0
0.0	0.0	0.0	0.0	0.0	0.0	0.0	0.0
0.0	0.0	0.0	0.0	0.0	0.0	0.0	0.0
0.0	0.0	0.0	0.0	0.0	0.0	0.0	0.0
0.0	0.0	0.0	0.0	32.0	0.0	0.0	0.0
0.0	0.0	1.0	2.0	0.0	0.0	0.0	2.0
0.0	0.0	0.0	0.0	0.0	0.0	0.0	0.0
0.0	0.0	0.0	0.0	0.0	0.0	0.0	0.0
2.0	0.0	0.0	0.0	0.0	0.0	0.0	2.0
0.0	0.0	0.0	0.0	0.0	0.0	0.0	0.0
0.0	0.0	0.0	0.0	0.0	0.0	0.0	0.0
0.0	0.0	0.0	0.0	0.0	0.0	0.0	0.0
0.0	1.0	0.0	0.0	0.0	0.0	0.0	0.0
0.0	0.0	0.0	0.0	0.0	0.0	0.0	0.0
0.0	0.0	0.0	0.0	0.0	0.0	0.0	0.0
5.0	0.0	4.0	0.0	0.0	0.0	0.0	5.0
0.0	0.0	0.0	0.0	0.0	0.0	0.0	0.0
0.0	0.0	0.0	0.0	0.0	0.0	0.0	1.0
0.0	0.0	4.0	70.0	2.0	0.0	0.0	0.0
0.0	0.0	0.0	0.0	0.0	0.0	0.0	0.0
0.0	0.0	2.0	0.0	0.0	0.0	0.0	0.0
0.0	0.0	0.0	0.0	1.0	0.0	0.0	0.0
0.0	0.0	0.0	0.0	0.0	0.0	0.0	0.0
0.0	0.0	0.0	0.0	0.0	0.0	0.0	0.0
0.0	0.0	0.0	0.0	0.0	0.0	0.0	0.0
0.0	0.0	0.0	0.0	0.0	0.0	0.0	0.0
0.0	0.0	0.0	0.0	0.0	0.0	0.0	0.0
0.0	0.0	0.0	0.0	0.0	0.0	0.0	0.0
0.0	0.0	0.0	0.0	0.0	0.0	0.0	0.0
0.0	0.0	1.0	0.0	1.0	0.0	0.0	0.0
0.0	0.0	0.0	0.0	0.0	0.0	0.0	0.0
0.0	0.0	0.0	0.0	0.0	0.0	0.0	0.0
3.0	0.0	0.0	0.0	0.0	0.0	0.0	0.0
0.0	0.0	2.0	50.0	33.0	0.0	441.0	0.0
0.0	0.0	0.0	0.0	0.0	0.0	0.0	0.0
0.0	0.0	0.0	0.0	4.0	0.0	0.0	0.0
0.0	0.0	0.0	0.0	0.0	0.0	0.0	0.0
0.0	0.0	0.0	0.0	0.0	0.0	0.0	0.0

0.0	0.0	0.0	0.0	2.0	0.0	0.0	1.0
0.0	0.0	0.0	0.0	0.0	0.0	0.0	0.0
0.0	0.0	0.0	0.0	0.0	0.0	0.0	0.0
0.0	0.0	0.0	0.0	0.0	0.0	0.0	0.0
4.0	0.0	0.0	0.0	6.0	0.0	0.0	0.0
0.0	0.0	0.0	0.0	0.0	1.0	0.0	0.0
3.0	0.0	0.0	0.0	3.0	0.0	0.0	0.0
0.0	0.0	0.0	0.0	5.0	0.0	0.0	0.0
0.0	0.0	0.0	0.0	0.0	0.0	0.0	0.0
0.0	0.0	0.0	0.0	0.0	0.0	0.0	0.0
0.0	0.0	1.0	0.0	0.0	0.0	0.0	0.0
0.0	0.0	0.0	0.0	0.0	0.0	0.0	0.0
0.0	0.0	0.0	0.0	0.0	0.0	0.0	0.0
0.0	0.0	0.0	0.0	0.0	0.0	0.0	0.0
0.0	0.0	0.0	0.0	0.0	0.0	0.0	0.0
0.0	0.0	0.0	0.0	0.0	0.0	0.0	0.0
0.0	0.0	0.0	0.0	0.0	0.0	0.0	0.0
0.0	0.0	0.0	0.0	0.0	0.0	0.0	0.0
0.0	0.0	0.0	0.0	0.0	0.0	0.0	0.0
0.0	0.0	0.0	0.0	5.0	0.0	0.0	0.0
0.0	0.0	0.0	0.0	0.0	0.0	0.0	0.0
0.0	0.0	0.0	0.0	0.0	0.0	0.0	0.0
0.0	0.0	0.0	0.0	0.0	0.0	0.0	0.0
0.0	0.0	0.0	0.0	0.0	0.0	0.0	0.0
0.0	0.0	0.0	0.0	0.0	0.0	0.0	0.0
0.0	1.0	0.0	0.0	0.0	0.0	0.0	0.0
0.0	0.0	0.0	0.0	0.0	0.0	0.0	0.0
0.0	0.0	0.0	0.0	0.0	0.0	0.0	0.0
0.0	0.0	0.0	0.0	0.0	47.0	0.0	0.0
0.0	0.0	0.0	0.0	0.0	0.0	0.0	0.0
0.0	1.0	0.0	0.0	0.0	0.0	0.0	0.0
0.0	0.0	0.0	0.0	0.0	6.0	0.0	0.0
0.0	0.0	0.0	0.0	0.0	0.0	0.0	0.0
0.0	0.0	0.0	0.0	0.0	0.0	0.0	0.0
0.0	0.0	5.0	0.0	0.0	0.0	0.0	0.0
0.0	0.0	0.0	0.0	0.0	1.0	0.0	0.0
0.0	0.0	0.0	0.0	0.0	0.0	0.0	0.0
0.0	0.0	0.0	0.0	0.0	0.0	0.0	0.0
0.0	0.0	0.0	0.0	0.0	0.0	0.0	0.0
0.0	0.0	0.0	0.0	0.0	0.0	0.0	0.0
0.0	0.0	1.0	0.0	0.0	0.0	0.0	0.0
0.0	0.0	0.0	0.0	0.0	0.0	0.0	0.0
0.0	0.0	0.0	0.0	0.0	0.0	0.0	0.0
0.0	0.0	0.0	0.0	0.0	0.0	0.0	0.0
0.0	0.0	0.0	0.0	0.0	0.0	0.0	0.0
0.0	0.0	0.0	0.0	0.0	0.0	0.0	0.0
0.0	0.0	0.0	0.0	0.0	0.0	0.0	0.0

0.0	0.0	0.0	0.0	0.0	0.0	0.0	0.0
0.0	0.0	0.0	0.0	0.0	0.0	0.0	0.0
0.0	0.0	0.0	0.0	0.0	0.0	0.0	0.0
0.0	0.0	0.0	0.0	0.0	0.0	0.0	0.0
0.0	0.0	0.0	0.0	0.0	0.0	0.0	0.0
0.0	0.0	0.0	0.0	0.0	0.0	0.0	0.0
1.0	0.0	0.0	5.0	0.0	0.0	0.0	14.0
3.0	0.0	0.0	11.0	0.0	0.0	7.0	0.0
0.0	0.0	0.0	54.0	0.0	0.0	7.0	0.0
0.0	0.0	0.0	0.0	0.0	0.0	0.0	0.0
0.0	0.0	0.0	0.0	0.0	0.0	0.0	7.0
0.0	0.0	0.0	0.0	0.0	0.0	0.0	0.0
0.0	0.0	0.0	0.0	0.0	0.0	0.0	0.0
17.0	0.0	0.0	0.0	2.0	0.0	1.0	2.0
0.0	0.0	0.0	10.0	0.0	0.0	10.0	0.0
0.0	0.0	0.0	0.0	0.0	0.0	0.0	0.0
0.0	0.0	0.0	0.0	0.0	0.0	0.0	0.0
0.0	0.0	0.0	23.0	0.0	0.0	0.0	0.0
0.0	0.0	0.0	0.0	0.0	0.0	0.0	0.0
0.0	0.0	0.0	0.0	0.0	0.0	0.0	0.0
0.0	0.0	0.0	0.0	0.0	0.0	0.0	0.0
0.0	0.0	0.0	0.0	0.0	0.0	0.0	0.0
2.0	0.0	0.0	0.0	0.0	0.0	0.0	0.0
0.0	0.0	0.0	0.0	0.0	0.0	0.0	0.0
0.0	0.0	0.0	0.0	0.0	0.0	0.0	0.0
0.0	0.0	0.0	2.0	0.0	0.0	0.0	0.0
0.0	0.0	0.0	0.0	0.0	0.0	1.0	0.0
0.0	0.0	0.0	0.0	0.0	0.0	0.0	0.0
0.0	0.0	0.0	0.0	0.0	0.0	0.0	0.0
0.0	0.0	0.0	0.0	0.0	0.0	0.0	0.0
0.0	0.0	0.0	0.0	0.0	0.0	0.0	0.0
0.0	1.0	0.0	0.0	0.0	0.0	0.0	0.0
0.0	0.0	0.0	0.0	0.0	0.0	0.0	0.0
0.0	0.0	0.0	0.0	0.0	0.0	0.0	0.0
0.0	0.0	0.0	0.0	0.0	0.0	0.0	0.0
0.0	0.0	0.0	0.0	0.0	0.0	0.0	0.0
0.0	0.0	0.0	0.0	0.0	0.0	0.0	0.0
0.0	0.0	0.0	0.0	0.0	0.0	0.0	0.0
0.0	0.0	0.0	0.0	0.0	0.0	0.0	0.0
0.0	0.0	0.0	0.0	0.0	0.0	0.0	0.0
0.0	0.0	0.0	0.0	0.0	0.0	0.0	0.0
0.0	0.0	0.0	0.0	0.0	0.0	0.0	0.0
0.0	4.0	0.0	0.0	0.0	0.0	0.0	0.0
0.0	0.0	0.0	0.0	0.0	0.0	0.0	0.0
0.0	0.0	0.0	0.0	0.0	0.0	0.0	0.0
3.0	0.0	0.0	0.0	0.0	0.0	10.0	0.0
0.0	0.0	0.0	0.0	2.0	0.0	0.0	0.0

0.0	0.0	0.0	0.0	0.0	0.0	0.0	0.0
31.0	0.0	0.0	0.0	0.0	0.0	0.0	134.0
0.0	0.0	0.0	0.0	3.0	0.0	0.0	0.0
0.0	0.0	0.0	0.0	0.0	0.0	0.0	0.0
5.0	0.0	0.0	0.0	0.0	0.0	0.0	0.0
0.0	0.0	0.0	0.0	0.0	0.0	0.0	0.0
0.0	0.0	0.0	0.0	0.0	0.0	0.0	0.0
0.0	0.0	0.0	0.0	0.0	0.0	0.0	0.0
0.0	0.0	2.0	0.0	0.0	0.0	0.0	0.0
0.0	0.0	0.0	0.0	1.0	0.0	0.0	0.0
0.0	0.0	0.0	0.0	0.0	0.0	0.0	0.0
0.0	0.0	4.0	0.0	0.0	0.0	0.0	0.0
0.0	0.0	3.0	0.0	0.0	0.0	0.0	2.0
0.0	0.0	0.0	0.0	0.0	0.0	0.0	0.0
0.0	0.0	0.0	0.0	3.0	0.0	0.0	0.0
0.0	0.0	0.0	0.0	0.0	0.0	0.0	0.0
0.0	0.0	0.0	0.0	0.0	0.0	0.0	0.0
0.0	0.0	0.0	0.0	0.0	0.0	0.0	0.0
3.0	0.0	0.0	0.0	0.0	0.0	0.0	0.0
0.0	0.0	0.0	0.0	0.0	0.0	0.0	0.0
0.0	0.0	0.0	0.0	0.0	0.0	0.0	0.0
0.0	0.0	0.0	0.0	0.0	0.0	0.0	0.0
99.0	0.0	0.0	0.0	0.0	1.0	15.0	20.0
42.0	0.0	0.0	0.0	0.0	0.0	0.0	0.0
0.0	0.0	0.0	0.0	1.0	0.0	0.0	3.0
0.0	0.0	32.0	0.0	0.0	0.0	0.0	0.0
0.0	0.0	0.0	0.0	0.0	0.0	0.0	0.0
0.0	0.0	0.0	0.0	0.0	0.0	0.0	0.0
0.0	0.0	6.0	0.0	72.0	0.0	0.0	0.0
0.0	0.0	0.0	0.0	0.0	0.0	0.0	0.0
0.0	0.0	0.0	0.0	3.0	0.0	0.0	0.0
0.0	0.0	0.0	0.0	0.0	0.0	0.0	0.0
0.0	0.0	0.0	0.0	0.0	0.0	0.0	0.0
0.0	0.0	0.0	0.0	0.0	0.0	0.0	0.0
0.0	0.0	0.0	0.0	0.0	0.0	0.0	0.0
0.0	0.0	0.0	0.0	0.0	0.0	0.0	0.0
0.0	0.0	0.0	0.0	0.0	0.0	0.0	0.0
5.0	0.0	0.0	0.0	0.0	0.0	0.0	2.0
0.0	0.0	0.0	0.0	2.0	0.0	0.0	0.0
0.0	0.0	0.0	0.0	0.0	0.0	0.0	33.0
0.0	0.0	0.0	0.0	0.0	0.0	0.0	0.0
0.0	0.0	1.0	0.0	0.0	0.0	0.0	0.0
0.0	0.0	6.0	0.0	0.0	0.0	0.0	4.0
0.0	0.0	0.0	0.0	0.0	0.0	0.0	0.0
0.0	0.0	0.0	0.0	0.0	0.0	0.0	0.0
0.0	0.0	2.0	0.0	0.0	0.0	0.0	3.0
0.0	0.0	0.0	65.0	0.0	0.0	0.0	0.0

0.0	0.0	0.0	0.0	0.0	0.0	0.0	0.0
0.0	0.0	1.0	0.0	0.0	0.0	0.0	0.0
6.0	0.0	0.0	0.0	1.0	0.0	0.0	2.0
0.0	0.0	0.0	0.0	0.0	0.0	0.0	0.0
0.0	0.0	2.0	0.0	3.0	0.0	0.0	0.0
0.0	0.0	0.0	0.0	0.0	0.0	0.0	0.0
0.0	0.0	0.0	0.0	0.0	0.0	0.0	0.0
0.0	0.0	0.0	0.0	0.0	0.0	0.0	0.0
2.0	0.0	0.0	0.0	0.0	0.0	0.0	0.0
0.0	0.0	1.0	166.0	1.0	0.0	44.0	0.0
0.0	0.0	0.0	0.0	0.0	0.0	0.0	0.0
0.0	0.0	0.0	0.0	2.0	0.0	0.0	0.0
0.0	0.0	0.0	0.0	6.0	0.0	0.0	0.0
0.0	0.0	0.0	0.0	0.0	0.0	0.0	0.0
0.0	0.0	0.0	0.0	0.0	0.0	0.0	0.0
0.0	0.0	0.0	0.0	0.0	0.0	0.0	0.0
0.0	0.0	0.0	0.0	0.0	0.0	0.0	0.0
0.0	0.0	0.0	0.0	0.0	0.0	0.0	0.0
0.0	0.0	0.0	0.0	0.0	0.0	0.0	0.0
0.0	0.0	0.0	0.0	0.0	0.0	0.0	0.0
0.0	0.0	0.0	0.0	0.0	0.0	0.0	0.0
0.0	0.0	0.0	0.0	0.0	0.0	0.0	0.0
0.0	0.0	0.0	0.0	0.0	0.0	0.0	0.0
0.0	0.0	0.0	0.0	0.0	0.0	0.0	0.0
0.0	0.0	0.0	0.0	0.0	0.0	0.0	0.0
0.0	0.0	0.0	0.0	0.0	0.0	0.0	0.0
0.0	0.0	0.0	0.0	0.0	0.0	0.0	0.0
0.0	0.0	0.0	0.0	1.0	0.0	0.0	0.0
0.0	0.0	0.0	0.0	0.0	0.0	0.0	0.0
0.0	0.0	0.0	0.0	0.0	0.0	0.0	0.0
0.0	0.0	0.0	0.0	0.0	0.0	0.0	0.0
0.0	0.0	0.0	0.0	0.0	0.0	0.0	0.0
0.0	0.0	0.0	0.0	7.0	0.0	0.0	0.0
0.0	0.0	0.0	7.0	0.0	0.0	0.0	0.0
0.0	0.0	0.0	0.0	0.0	0.0	0.0	0.0
0.0	0.0	0.0	0.0	0.0	0.0	0.0	0.0
0.0	0.0	0.0	0.0	0.0	0.0	0.0	0.0
2.0	0.0	0.0	0.0	0.0	0.0	0.0	0.0
2.0	0.0	0.0	0.0	3.0	0.0	0.0	0.0
0.0	0.0	0.0	0.0	0.0	0.0	0.0	0.0
0.0	0.0	0.0	0.0	0.0	0.0	0.0	0.0
0.0	0.0	0.0	0.0	0.0	0.0	0.0	2.0
0.0	0.0	0.0	0.0	0.0	0.0	0.0	0.0
0.0	0.0	0.0	0.0	0.0	0.0	0.0	0.0
0.0	0.0	0.0	0.0	0.0	0.0	0.0	0.0
0.0	0.0	0.0	0.0	0.0	0.0	0.0	0.0
0.0	0.0	0.0	0.0	0.0	0.0	0.0	0.0

0.0	0.0	0.0	0.0	0.0	0.0	0.0	0.0
0.0	0.0	0.0	0.0	0.0	0.0	0.0	0.0
0.0	0.0	0.0	0.0	0.0	0.0	0.0	0.0
0.0	0.0	0.0	0.0	1.0	0.0	0.0	0.0
0.0	0.0	0.0	0.0	0.0	0.0	0.0	0.0
0.0	0.0	0.0	0.0	0.0	0.0	0.0	0.0
0.0	0.0	0.0	0.0	0.0	0.0	0.0	0.0
0.0	0.0	4.0	0.0	0.0	0.0	0.0	0.0
0.0	0.0	0.0	0.0	0.0	0.0	0.0	0.0
0.0	0.0	0.0	0.0	0.0	0.0	0.0	0.0
0.0	0.0	0.0	0.0	0.0	0.0	0.0	0.0
0.0	0.0	0.0	0.0	0.0	0.0	0.0	0.0
0.0	0.0	0.0	0.0	0.0	0.0	0.0	0.0
0.0	0.0	0.0	0.0	0.0	0.0	0.0	0.0
0.0	0.0	0.0	0.0	0.0	0.0	0.0	0.0
0.0	0.0	0.0	0.0	0.0	0.0	0.0	0.0
0.0	0.0	0.0	0.0	0.0	0.0	0.0	0.0
0.0	0.0	0.0	0.0	0.0	0.0	0.0	0.0
0.0	2.0	0.0	0.0	0.0	0.0	0.0	0.0
0.0	0.0	0.0	0.0	0.0	0.0	0.0	0.0
0.0	4.0	0.0	0.0	0.0	0.0	0.0	0.0
0.0	0.0	0.0	0.0	0.0	0.0	0.0	0.0
0.0	0.0	0.0	0.0	0.0	0.0	0.0	0.0
0.0	0.0	0.0	0.0	0.0	0.0	0.0	0.0
0.0	0.0	0.0	0.0	0.0	0.0	0.0	0.0
0.0	0.0	0.0	0.0	0.0	0.0	0.0	0.0
0.0	0.0	0.0	0.0	0.0	0.0	0.0	0.0
0.0	0.0	0.0	0.0	0.0	0.0	0.0	0.0
0.0	0.0	0.0	0.0	0.0	0.0	0.0	0.0
0.0	0.0	0.0	0.0	0.0	0.0	0.0	0.0
0.0	0.0	0.0	0.0	0.0	0.0	0.0	0.0
0.0	0.0	0.0	0.0	0.0	0.0	0.0	0.0
0.0	0.0	0.0	0.0	0.0	0.0	0.0	0.0
0.0	0.0	0.0	0.0	0.0	0.0	0.0	0.0
0.0	0.0	0.0	0.0	0.0	0.0	0.0	0.0
0.0	0.0	0.0	0.0	0.0	0.0	0.0	0.0
0.0	0.0	0.0	0.0	13.0	0.0	0.0	0.0
0.0	0.0	0.0	0.0	0.0	0.0	0.0	0.0
0.0	0.0	0.0	0.0	0.0	0.0	0.0	0.0
0.0	0.0	0.0	0.0	0.0	0.0	0.0	0.0
6.0	1.0	0.0	0.0	0.0	0.0	0.0	0.0
0.0	0.0	0.0	0.0	0.0	60.0	0.0	0.0
0.0	0.0	0.0	0.0	0.0	0.0	0.0	0.0
0.0	0.0	0.0	0.0	0.0	0.0	0.0	0.0
0.0	0.0	0.0	0.0	0.0	0.0	0.0	0.0
0.0	0.0	0.0	0.0	0.0	0.0	0.0	0.0
0.0	0.0	0.0	0.0	0.0	0.0	0.0	0.0

0.0	0.0	0.0	0.0	0.0	0.0	0.0	0.0
0.0	0.0	0.0	0.0	0.0	0.0	0.0	0.0
0.0	0.0	0.0	0.0	0.0	0.0	0.0	0.0
0.0	0.0	0.0	0.0	0.0	0.0	0.0	0.0
0.0	0.0	0.0	0.0	0.0	0.0	0.0	0.0
0.0	0.0	0.0	0.0	0.0	0.0	0.0	0.0
0.0	0.0	0.0	0.0	0.0	0.0	0.0	0.0
0.0	0.0	0.0	0.0	0.0	0.0	0.0	0.0
0.0	0.0	0.0	0.0	0.0	0.0	0.0	0.0
0.0	0.0	0.0	0.0	0.0	0.0	0.0	0.0
0.0	0.0	0.0	0.0	0.0	0.0	0.0	0.0
0.0	0.0	0.0	0.0	0.0	0.0	0.0	0.0
0.0	0.0	0.0	0.0	0.0	0.0	0.0	0.0
0.0	0.0	0.0	4.0	0.0	0.0	0.0	0.0
0.0	0.0	0.0	0.0	0.0	0.0	0.0	0.0
0.0	0.0	0.0	0.0	0.0	0.0	0.0	0.0
0.0	0.0	0.0	0.0	0.0	0.0	0.0	0.0
0.0	0.0	0.0	0.0	0.0	0.0	0.0	0.0
0.0	0.0	0.0	0.0	0.0	0.0	0.0	0.0
0.0	0.0	0.0	0.0	0.0	0.0	0.0	0.0
0.0	0.0	0.0	0.0	0.0	0.0	0.0	0.0
9.0	0.0	3.0	0.0	0.0	0.0	0.0	0.0
0.0	0.0	0.0	0.0	0.0	0.0	0.0	0.0
0.0	0.0	0.0	0.0	0.0	0.0	0.0	0.0
0.0	0.0	0.0	0.0	0.0	0.0	0.0	0.0
0.0	0.0	0.0	0.0	0.0	0.0	0.0	0.0
0.0	0.0	0.0	0.0	0.0	0.0	0.0	0.0
2.0	0.0	0.0	0.0	0.0	0.0	0.0	0.0
0.0	0.0	0.0	0.0	0.0	0.0	0.0	0.0
0.0	0.0	0.0	0.0	0.0	0.0	0.0	0.0
0.0	0.0	0.0	0.0	0.0	0.0	0.0	0.0
0.0	0.0	0.0	0.0	0.0	0.0	0.0	0.0
0.0	0.0	0.0	0.0	0.0	0.0	0.0	0.0
0.0	0.0	0.0	0.0	0.0	0.0	0.0	0.0
0.0	0.0	0.0	0.0	0.0	0.0	0.0	0.0
1.0	0.0	0.0	0.0	0.0	0.0	0.0	0.0
0.0	0.0	0.0	0.0	0.0	0.0	0.0	0.0
0.0	0.0	0.0	0.0	0.0	0.0	0.0	0.0
0.0	0.0	0.0	0.0	0.0	0.0	0.0	0.0
0.0	0.0	0.0	0.0	0.0	0.0	0.0	0.0
0.0	0.0	0.0	0.0	0.0	0.0	0.0	0.0
0.0	0.0	0.0	0.0	0.0	0.0	0.0	2.0
0.0	0.0	1.0	0.0	3.0	0.0	0.0	0.0
0.0	0.0	0.0	0.0	0.0	0.0	0.0	0.0
0.0	0.0	0.0	0.0	0.0	0.0	0.0	0.0
0.0	0.0	0.0	0.0	0.0	0.0	0.0	0.0
0.0	0.0	0.0	0.0	0.0	0.0	0.0	0.0

0.0	0.0	0.0	0.0	0.0	0.0	0.0	0.0
0.0	0.0	0.0	0.0	0.0	0.0	0.0	0.0
0.0	0.0	0.0	0.0	0.0	0.0	0.0	0.0
0.0	0.0	0.0	0.0	0.0	0.0	0.0	0.0
0.0	0.0	0.0	0.0	0.0	0.0	0.0	0.0
0.0	0.0	0.0	0.0	0.0	0.0	0.0	0.0
0.0	0.0	0.0	0.0	0.0	0.0	0.0	0.0
0.0	0.0	0.0	0.0	0.0	0.0	0.0	0.0
0.0	0.0	0.0	0.0	0.0	0.0	0.0	0.0
0.0	0.0	0.0	0.0	0.0	0.0	0.0	0.0
0.0	0.0	0.0	0.0	0.0	0.0	0.0	0.0
0.0	0.0	0.0	0.0	0.0	0.0	0.0	0.0
0.0	0.0	0.0	0.0	0.0	0.0	0.0	0.0
0.0	0.0	0.0	0.0	0.0	0.0	0.0	0.0
0.0	0.0	0.0	0.0	0.0	0.0	0.0	0.0
2.0	0.0	2.0	0.0	0.0	0.0	0.0	1.0
0.0	0.0	0.0	0.0	0.0	0.0	0.0	0.0
0.0	0.0	0.0	0.0	0.0	0.0	0.0	0.0
0.0	0.0	0.0	0.0	0.0	0.0	0.0	0.0
0.0	0.0	0.0	0.0	0.0	0.0	0.0	0.0
0.0	0.0	0.0	0.0	0.0	0.0	0.0	0.0
0.0	0.0	0.0	0.0	0.0	0.0	0.0	0.0
0.0	0.0	0.0	0.0	0.0	0.0	0.0	0.0
0.0	0.0	0.0	0.0	0.0	0.0	0.0	0.0
0.0	0.0	0.0	0.0	0.0	0.0	0.0	0.0
0.0	0.0	0.0	0.0	0.0	0.0	0.0	0.0
0.0	0.0	0.0	0.0	0.0	0.0	0.0	0.0
0.0	0.0	0.0	0.0	0.0	0.0	0.0	0.0
0.0	0.0	0.0	0.0	0.0	0.0	0.0	0.0
0.0	0.0	0.0	0.0	0.0	0.0	0.0	0.0
0.0	0.0	0.0	0.0	0.0	0.0	0.0	0.0
0.0	0.0	52.0	0.0	0.0	0.0	0.0	0.0
0.0	0.0	0.0	0.0	0.0	0.0	0.0	0.0
0.0	0.0	19.0	0.0	0.0	0.0	0.0	0.0
0.0	0.0	5.0	0.0	0.0	0.0	0.0	0.0
0.0	0.0	0.0	0.0	0.0	0.0	0.0	0.0
0.0	0.0	0.0	0.0	0.0	0.0	0.0	0.0
0.0	0.0	10.0	0.0	0.0	0.0	0.0	0.0
2.0	0.0	0.0	4.0	0.0	0.0	0.0	0.0
0.0	0.0	0.0	0.0	0.0	0.0	0.0	0.0
264.0	0.0	0.0	0.0	0.0	0.0	0.0	0.0
0.0	0.0	40.0	0.0	0.0	0.0	0.0	0.0
0.0	0.0	0.0	0.0	0.0	0.0	0.0	0.0
0.0	0.0	0.0	0.0	0.0	0.0	0.0	0.0
0.0	0.0	0.0	39.0	0.0	0.0	0.0	0.0
0.0	0.0	0.0	0.0	143.0	0.0	0.0	0.0
0.0	0.0	0.0	0.0	0.0	2.0	0.0	0.0

0.0	0.0	0.0	0.0	0.0	0.0	0.0	0.0
0.0	0.0	0.0	0.0	0.0	0.0	0.0	0.0
0.0	0.0	4.0	0.0	0.0	0.0	0.0	0.0
0.0	0.0	0.0	0.0	0.0	0.0	0.0	0.0
0.0	0.0	0.0	0.0	0.0	0.0	0.0	0.0
0.0	0.0	0.0	0.0	0.0	0.0	0.0	0.0
0.0	0.0	0.0	0.0	0.0	0.0	0.0	0.0
0.0	0.0	0.0	0.0	0.0	0.0	0.0	0.0
0.0	0.0	1.0	0.0	0.0	0.0	0.0	0.0
0.0	0.0	0.0	0.0	0.0	0.0	0.0	0.0
0.0	26.0	1.0	0.0	0.0	6.0	0.0	0.0
0.0	0.0	0.0	0.0	0.0	0.0	0.0	0.0
0.0	0.0	0.0	0.0	0.0	0.0	0.0	0.0
0.0	0.0	0.0	0.0	0.0	0.0	0.0	0.0
0.0	0.0	3.0	0.0	0.0	0.0	0.0	0.0
0.0	0.0	0.0	0.0	0.0	0.0	0.0	0.0
81.0	0.0	53.0	0.0	0.0	0.0	0.0	0.0
0.0	0.0	0.0	0.0	0.0	0.0	0.0	0.0
0.0	0.0	0.0	0.0	0.0	0.0	0.0	0.0
0.0	0.0	0.0	0.0	0.0	0.0	0.0	0.0
11.0	0.0	0.0	0.0	2.0	0.0	0.0	0.0
0.0	0.0	0.0	0.0	0.0	0.0	0.0	0.0
0.0	0.0	0.0	0.0	0.0	0.0	0.0	0.0
0.0	0.0	0.0	0.0	0.0	0.0	0.0	0.0
4.0	0.0	0.0	0.0	0.0	0.0	0.0	0.0
0.0	0.0	0.0	0.0	0.0	0.0	0.0	0.0
0.0	0.0	0.0	0.0	0.0	0.0	0.0	0.0
0.0	0.0	0.0	0.0	0.0	0.0	0.0	0.0
2.0	0.0	0.0	0.0	0.0	0.0	0.0	0.0
0.0	0.0	0.0	0.0	0.0	0.0	0.0	0.0
0.0	0.0	0.0	0.0	0.0	0.0	0.0	0.0
0.0	0.0	0.0	0.0	0.0	0.0	0.0	0.0
0.0	0.0	0.0	0.0	0.0	2.0	0.0	0.0
0.0	0.0	1.0	0.0	0.0	0.0	0.0	0.0
0.0	0.0	0.0	0.0	0.0	0.0	0.0	0.0
0.0	0.0	0.0	0.0	0.0	0.0	0.0	0.0
0.0	0.0	0.0	0.0	0.0	0.0	0.0	0.0
0.0	0.0	0.0	0.0	0.0	0.0	0.0	0.0
0.0	0.0	0.0	0.0	0.0	0.0	0.0	0.0
0.0	0.0	0.0	0.0	2.0	0.0	0.0	0.0
0.0	0.0	0.0	0.0	0.0	0.0	0.0	0.0
0.0	0.0	0.0	0.0	0.0	0.0	0.0	0.0
0.0	0.0	0.0	0.0	0.0	0.0	0.0	0.0
0.0	0.0	0.0	0.0	0.0	0.0	0.0	0.0
0.0	0.0	0.0	0.0	0.0	0.0	0.0	0.0
0.0	0.0	0.0	0.0	0.0	0.0	0.0	0.0
0.0	0.0	0.0	0.0	0.0	0.0	0.0	0.0

13.0	0.0	8.0	0.0	0.0	0.0	0.0	0.0
0.0	0.0	0.0	0.0	0.0	0.0	0.0	0.0
0.0	0.0	0.0	0.0	0.0	0.0	0.0	0.0
0.0	0.0	0.0	0.0	0.0	7486.0	0.0	0.0
0.0	0.0	0.0	0.0	0.0	0.0	0.0	0.0
0.0	0.0	0.0	0.0	0.0	0.0	0.0	0.0
0.0	0.0	0.0	0.0	0.0	0.0	0.0	0.0
0.0	0.0	0.0	0.0	0.0	0.0	0.0	0.0
0.0	0.0	0.0	0.0	0.0	0.0	0.0	0.0
0.0	0.0	0.0	0.0	0.0	0.0	0.0	0.0
0.0	0.0	0.0	0.0	0.0	0.0	0.0	0.0
0.0	0.0	0.0	0.0	0.0	0.0	0.0	0.0
0.0	0.0	0.0	0.0	0.0	0.0	0.0	0.0
0.0	0.0	0.0	0.0	0.0	0.0	0.0	0.0
0.0	0.0	0.0	0.0	0.0	0.0	0.0	0.0
0.0	0.0	0.0	0.0	0.0	0.0	0.0	0.0
0.0	0.0	0.0	0.0	0.0	0.0	0.0	0.0
0.0	0.0	2.0	0.0	0.0	0.0	0.0	0.0
0.0	0.0	0.0	0.0	0.0	0.0	0.0	0.0
0.0	0.0	0.0	0.0	0.0	0.0	0.0	0.0
0.0	0.0	0.0	0.0	0.0	0.0	0.0	0.0
0.0	0.0	0.0	0.0	0.0	0.0	0.0	0.0
0.0	0.0	0.0	0.0	0.0	0.0	0.0	0.0
0.0	0.0	0.0	0.0	0.0	0.0	0.0	0.0
0.0	0.0	0.0	0.0	0.0	0.0	0.0	0.0
0.0	0.0	0.0	0.0	0.0	0.0	0.0	0.0
0.0	0.0	0.0	0.0	0.0	0.0	0.0	2.0
0.0	0.0	0.0	0.0	0.0	0.0	0.0	0.0
0.0	0.0	0.0	0.0	0.0	0.0	0.0	0.0
0.0	0.0	0.0	0.0	0.0	0.0	0.0	0.0
0.0	0.0	0.0	0.0	0.0	0.0	0.0	0.0
0.0	0.0	0.0	0.0	0.0	0.0	0.0	0.0
0.0	0.0	0.0	0.0	0.0	0.0	0.0	0.0
0.0	0.0	0.0	0.0	0.0	0.0	0.0	0.0
0.0	0.0	0.0	0.0	0.0	0.0	0.0	0.0
0.0	0.0	0.0	0.0	0.0	0.0	0.0	0.0
0.0	0.0	0.0	0.0	0.0	0.0	0.0	0.0
0.0	0.0	0.0	0.0	0.0	0.0	0.0	0.0
0.0	0.0	0.0	0.0	0.0	0.0	0.0	0.0
0.0	0.0	0.0	0.0	0.0	0.0	0.0	0.0
0.0	0.0	0.0	0.0	0.0	0.0	0.0	0.0
0.0	0.0	0.0	0.0	0.0	0.0	0.0	0.0
0.0	48.0	0.0	0.0	0.0	0.0	0.0	0.0
0.0	0.0	0.0	0.0	0.0	0.0	0.0	0.0
0.0	0.0	0.0	0.0	0.0	0.0	0.0	0.0
0.0	0.0	0.0	0.0	0.0	0.0	0.0	0.0
0.0	0.0	0.0	0.0	0.0	0.0	0.0	0.0

0.0	0.0	0.0	0.0	0.0	0.0	0.0	0.0
293.0	45.0	0.0	0.0	0.0	0.0	0.0	0.0
0.0	0.0	0.0	17.0	0.0	0.0	0.0	0.0
0.0	0.0	0.0	0.0	0.0	0.0	0.0	0.0
0.0	0.0	0.0	0.0	0.0	0.0	0.0	0.0
0.0	0.0	0.0	0.0	0.0	0.0	0.0	0.0
0.0	0.0	0.0	0.0	0.0	0.0	0.0	0.0
0.0	0.0	0.0	0.0	0.0	0.0	0.0	0.0
0.0	0.0	0.0	0.0	0.0	0.0	0.0	0.0
0.0	0.0	0.0	0.0	0.0	0.0	0.0	0.0
0.0	0.0	0.0	0.0	5.0	0.0	0.0	0.0
0.0	0.0	36.0	0.0	0.0	0.0	0.0	0.0
0.0	0.0	27.0	0.0	572.0	0.0	0.0	0.0
0.0	0.0	0.0	39.0	0.0	0.0	0.0	0.0
0.0	0.0	0.0	0.0	0.0	0.0	0.0	0.0
0.0	0.0	0.0	0.0	0.0	0.0	0.0	0.0
0.0	0.0	0.0	4.0	0.0	0.0	0.0	0.0
0.0	0.0	0.0	0.0	0.0	0.0	0.0	0.0
0.0	0.0	0.0	0.0	0.0	0.0	0.0	0.0
0.0	0.0	0.0	0.0	0.0	0.0	0.0	0.0
0.0	0.0	0.0	0.0	0.0	0.0	0.0	0.0
0.0	0.0	0.0	0.0	0.0	0.0	0.0	0.0
0.0	0.0	0.0	0.0	0.0	0.0	0.0	0.0
0.0	0.0	0.0	0.0	0.0	0.0	0.0	0.0
0.0	0.0	0.0	0.0	0.0	0.0	0.0	0.0
0.0	0.0	0.0	0.0	0.0	0.0	0.0	0.0
0.0	0.0	0.0	0.0	0.0	0.0	0.0	0.0
0.0	0.0	0.0	0.0	0.0	0.0	0.0	0.0
0.0	0.0	0.0	0.0	0.0	0.0	0.0	0.0
0.0	0.0	0.0	0.0	0.0	0.0	0.0	0.0
0.0	0.0	0.0	0.0	0.0	0.0	0.0	16.0
0.0	0.0	0.0	0.0	0.0	0.0	0.0	0.0
0.0	0.0	0.0	0.0	0.0	0.0	0.0	0.0
0.0	0.0	0.0	0.0	0.0	0.0	0.0	0.0
0.0	0.0	0.0	0.0	0.0	0.0	0.0	4.0
0.0	0.0	0.0	0.0	0.0	0.0	0.0	0.0
0.0	0.0	0.0	0.0	0.0	0.0	0.0	0.0
0.0	0.0	0.0	0.0	0.0	0.0	0.0	0.0
0.0	0.0	0.0	0.0	0.0	0.0	0.0	0.0
0.0	0.0	0.0	0.0	0.0	0.0	0.0	0.0
0.0	0.0	0.0	0.0	0.0	0.0	0.0	0.0
0.0	0.0	0.0	0.0	0.0	0.0	0.0	0.0
0.0	0.0	0.0	0.0	0.0	0.0	0.0	0.0
0.0	0.0	0.0	0.0	0.0	0.0	0.0	0.0
0.0	0.0	0.0	0.0	0.0	0.0	0.0	0.0
0.0	0.0	0.0	0.0	0.0	0.0	0.0	0.0
0.0	0.0	0.0	1.0	0.0	0.0	0.0	0.0

0.0	0.0	0.0	0.0	0.0	0.0	0.0	0.0
0.0	0.0	0.0	0.0	0.0	0.0	0.0	0.0
0.0	0.0	0.0	0.0	0.0	0.0	0.0	0.0
0.0	0.0	0.0	0.0	0.0	0.0	0.0	0.0
0.0	0.0	0.0	0.0	0.0	0.0	0.0	0.0
0.0	0.0	0.0	0.0	0.0	0.0	0.0	0.0
0.0	0.0	0.0	0.0	0.0	0.0	1.0	0.0
0.0	0.0	0.0	0.0	0.0	0.0	0.0	0.0
0.0	0.0	0.0	0.0	0.0	0.0	0.0	0.0
0.0	0.0	0.0	0.0	0.0	0.0	0.0	0.0
0.0	0.0	0.0	0.0	0.0	0.0	0.0	0.0
0.0	0.0	0.0	0.0	0.0	0.0	0.0	0.0
0.0	0.0	0.0	0.0	0.0	0.0	0.0	0.0
0.0	0.0	0.0	0.0	0.0	0.0	0.0	0.0
0.0	0.0	0.0	0.0	0.0	0.0	0.0	0.0
0.0	0.0	0.0	0.0	0.0	0.0	4.0	0.0
0.0	0.0	0.0	0.0	0.0	0.0	0.0	0.0
0.0	0.0	0.0	0.0	0.0	0.0	0.0	0.0
0.0	0.0	0.0	0.0	0.0	0.0	0.0	0.0
0.0	0.0	0.0	0.0	0.0	0.0	0.0	0.0
0.0	0.0	0.0	0.0	0.0	0.0	0.0	0.0
0.0	0.0	0.0	0.0	0.0	0.0	0.0	0.0
0.0	0.0	0.0	0.0	0.0	0.0	0.0	0.0
0.0	0.0	0.0	0.0	0.0	4.0	0.0	0.0
0.0	0.0	67.0	0.0	0.0	0.0	0.0	0.0
0.0	0.0	0.0	0.0	0.0	0.0	0.0	0.0
0.0	0.0	0.0	0.0	0.0	0.0	0.0	0.0
0.0	0.0	0.0	0.0	0.0	0.0	0.0	0.0
0.0	0.0	0.0	0.0	0.0	0.0	0.0	0.0
0.0	0.0	0.0	0.0	0.0	0.0	0.0	0.0
0.0	0.0	0.0	0.0	0.0	0.0	0.0	0.0
0.0	0.0	0.0	7.0	0.0	0.0	0.0	0.0
0.0	0.0	0.0	0.0	1.0	0.0	0.0	0.0
0.0	0.0	0.0	0.0	0.0	0.0	0.0	0.0
0.0	0.0	0.0	0.0	0.0	0.0	0.0	0.0
0.0	0.0	0.0	0.0	0.0	0.0	0.0	0.0
0.0	0.0	0.0	0.0	0.0	0.0	0.0	0.0
0.0	0.0	0.0	0.0	0.0	0.0	0.0	0.0
0.0	0.0	0.0	0.0	0.0	0.0	0.0	0.0
0.0	0.0	0.0	0.0	0.0	0.0	0.0	0.0
0.0	0.0	0.0	0.0	0.0	0.0	0.0	0.0
0.0	0.0	0.0	0.0	0.0	0.0	0.0	0.0
0.0	0.0	0.0	0.0	0.0	0.0	0.0	0.0
0.0	0.0	0.0	0.0	0.0	0.0	0.0	0.0
0.0	0.0	0.0	0.0	0.0	0.0	0.0	0.0
0.0	1.0	0.0	0.0	2.0	0.0	0.0	0.0
0.0	0.0	0.0	0.0	0.0	0.0	0.0	0.0

0.0	0.0	0.0	0.0	0.0	0.0	0.0	0.0
1.0	0.0	2.0	0.0	5.0	0.0	0.0	0.0
0.0	0.0	0.0	0.0	0.0	0.0	0.0	0.0
0.0	0.0	0.0	1.0	0.0	0.0	0.0	0.0
0.0	0.0	0.0	0.0	0.0	0.0	0.0	0.0
0.0	0.0	0.0	0.0	0.0	0.0	0.0	0.0
0.0	0.0	0.0	0.0	0.0	0.0	0.0	0.0
0.0	0.0	0.0	0.0	0.0	0.0	0.0	0.0
0.0	0.0	0.0	0.0	0.0	0.0	0.0	0.0
0.0	0.0	0.0	0.0	0.0	0.0	0.0	0.0
0.0	0.0	0.0	0.0	0.0	0.0	0.0	0.0
0.0	0.0	0.0	0.0	0.0	0.0	0.0	0.0
0.0	0.0	0.0	0.0	0.0	0.0	0.0	0.0
0.0	0.0	0.0	0.0	0.0	0.0	0.0	0.0
0.0	0.0	0.0	0.0	0.0	0.0	0.0	0.0
0.0	0.0	0.0	0.0	0.0	0.0	0.0	0.0
0.0	0.0	0.0	0.0	0.0	0.0	0.0	0.0
0.0	0.0	0.0	0.0	0.0	0.0	0.0	0.0
2.0	0.0	0.0	0.0	0.0	0.0	0.0	0.0
0.0	0.0	0.0	0.0	0.0	0.0	0.0	0.0
0.0	0.0	0.0	0.0	0.0	0.0	0.0	0.0
0.0	0.0	0.0	0.0	0.0	0.0	0.0	0.0
0.0	0.0	0.0	0.0	0.0	0.0	0.0	0.0
0.0	0.0	0.0	0.0	0.0	0.0	0.0	0.0
0.0	0.0	0.0	0.0	0.0	0.0	0.0	0.0
0.0	0.0	0.0	0.0	0.0	0.0	0.0	0.0
0.0	0.0	0.0	0.0	6.0	0.0	0.0	0.0
0.0	0.0	0.0	0.0	0.0	0.0	0.0	0.0
0.0	0.0	0.0	0.0	0.0	0.0	0.0	0.0
0.0	0.0	0.0	0.0	0.0	0.0	0.0	0.0
0.0	0.0	0.0	0.0	0.0	0.0	0.0	0.0
0.0	0.0	0.0	0.0	0.0	0.0	0.0	0.0
0.0	0.0	0.0	0.0	0.0	0.0	0.0	0.0
0.0	0.0	0.0	0.0	1.0	0.0	0.0	0.0
0.0	0.0	0.0	0.0	0.0	0.0	0.0	0.0
0.0	0.0	0.0	3.0	0.0	0.0	0.0	0.0
0.0	0.0	0.0	0.0	0.0	0.0	0.0	0.0
0.0	0.0	0.0	0.0	0.0	0.0	0.0	0.0
0.0	0.0	0.0	0.0	0.0	0.0	0.0	0.0
0.0	0.0	0.0	0.0	0.0	0.0	0.0	0.0
0.0	0.0	0.0	0.0	1.0	0.0	0.0	0.0
0.0	0.0	0.0	0.0	0.0	0.0	0.0	0.0
0.0	0.0	0.0	0.0	0.0	0.0	0.0	0.0
0.0	0.0	0.0	0.0	0.0	0.0	0.0	0.0
0.0	0.0	0.0	0.0	0.0	0.0	0.0	0.0
0.0	0.0	0.0	0.0	0.0	0.0	0.0	0.0
0.0	0.0	0.0	0.0	0.0	1.0	0.0	0.0

0.0	0.0	0.0	0.0	0.0	0.0	0.0	0.0
0.0	0.0	0.0	0.0	0.0	0.0	0.0	0.0
0.0	0.0	0.0	0.0	0.0	0.0	0.0	0.0
0.0	0.0	0.0	0.0	0.0	0.0	0.0	0.0
0.0	0.0	0.0	0.0	0.0	0.0	0.0	0.0
0.0	0.0	0.0	0.0	0.0	0.0	0.0	0.0
0.0	0.0	0.0	0.0	0.0	0.0	0.0	0.0
0.0	0.0	0.0	0.0	0.0	0.0	0.0	0.0
0.0	0.0	0.0	0.0	0.0	0.0	0.0	0.0
0.0	0.0	0.0	0.0	0.0	0.0	0.0	0.0
0.0	0.0	0.0	0.0	0.0	0.0	0.0	0.0
0.0	0.0	0.0	0.0	0.0	0.0	0.0	0.0
0.0	0.0	0.0	0.0	0.0	0.0	0.0	0.0
0.0	0.0	0.0	0.0	0.0	0.0	0.0	0.0
0.0	0.0	0.0	0.0	0.0	0.0	0.0	0.0
0.0	0.0	0.0	0.0	0.0	0.0	0.0	0.0
0.0	0.0	0.0	0.0	0.0	0.0	0.0	0.0
0.0	0.0	0.0	0.0	0.0	0.0	0.0	0.0
0.0	0.0	0.0	0.0	0.0	0.0	0.0	0.0
0.0	0.0	0.0	0.0	0.0	0.0	0.0	0.0
0.0	0.0	0.0	0.0	0.0	0.0	0.0	0.0
0.0	0.0	0.0	0.0	0.0	0.0	0.0	0.0
415.0	0.0	0.0	0.0	0.0	0.0	0.0	0.0
163.0	0.0	0.0	0.0	0.0	0.0	0.0	0.0
86.0	0.0	0.0	0.0	0.0	0.0	0.0	0.0
51.0	0.0	0.0	0.0	0.0	0.0	0.0	0.0
40.0	0.0	0.0	0.0	0.0	0.0	0.0	0.0
30.0	0.0	0.0	0.0	0.0	0.0	0.0	0.0
25.0	0.0	0.0	0.0	0.0	0.0	0.0	0.0
26.0	0.0	0.0	0.0	0.0	0.0	0.0	0.0
17.0	0.0	0.0	0.0	0.0	0.0	0.0	0.0
13.0	0.0	0.0	0.0	1.0	0.0	0.0	0.0
10.0	0.0	0.0	0.0	0.0	0.0	0.0	0.0
12.0	0.0	0.0	0.0	0.0	0.0	0.0	0.0
6.0	0.0	0.0	0.0	0.0	0.0	0.0	0.0
8.0	0.0	0.0	0.0	0.0	0.0	0.0	0.0
9.0	0.0	0.0	0.0	0.0	0.0	0.0	0.0
3.0	0.0	0.0	0.0	0.0	0.0	0.0	0.0
6.0	0.0	0.0	0.0	0.0	0.0	0.0	0.0
6.0	0.0	0.0	0.0	0.0	0.0	0.0	0.0
6.0	0.0	0.0	0.0	0.0	0.0	0.0	0.0
5.0	0.0	0.0	0.0	0.0	0.0	0.0	0.0
4.0	0.0	0.0	0.0	0.0	0.0	0.0	0.0
4.0	0.0	0.0	0.0	0.0	0.0	0.0	0.0
5.0	0.0	0.0	0.0	0.0	0.0	0.0	1.0
3.0	0.0	0.0	0.0	0.0	0.0	0.0	0.0
3.0	0.0	0.0	0.0	0.0	0.0	0.0	0.0

4.0	0.0	0.0	0.0	0.0	0.0	0.0	0.0
4.0	0.0	0.0	0.0	0.0	0.0	0.0	0.0
5.0	0.0	0.0	0.0	0.0	0.0	0.0	0.0
4.0	0.0	0.0	0.0	0.0	0.0	0.0	0.0
1.0	0.0	0.0	0.0	0.0	0.0	0.0	0.0
2.0	0.0	0.0	0.0	0.0	0.0	0.0	0.0
3.0	0.0	0.0	0.0	0.0	0.0	0.0	0.0
5.0	0.0	0.0	0.0	0.0	0.0	0.0	0.0
1.0	0.0	0.0	0.0	0.0	0.0	0.0	0.0
1.0	0.0	0.0	0.0	0.0	0.0	0.0	0.0
2.0	0.0	0.0	0.0	0.0	0.0	0.0	0.0
1.0	0.0	0.0	0.0	0.0	0.0	0.0	0.0
3.0	0.0	0.0	0.0	0.0	0.0	0.0	0.0
1.0	0.0	0.0	0.0	1.0	0.0	0.0	1.0
1.0	0.0	0.0	0.0	0.0	0.0	0.0	0.0
2.0	0.0	0.0	0.0	0.0	0.0	0.0	0.0
2.0	0.0	0.0	0.0	0.0	0.0	0.0	0.0
2.0	0.0	0.0	0.0	0.0	0.0	0.0	0.0
2.0	0.0	0.0	0.0	0.0	0.0	0.0	0.0
3.0	0.0	0.0	0.0	0.0	0.0	0.0	0.0
2.0	0.0	0.0	0.0	0.0	0.0	0.0	0.0
3.0	0.0	0.0	0.0	0.0	0.0	0.0	0.0
2.0	0.0	0.0	0.0	0.0	0.0	0.0	0.0
1.0	0.0	0.0	0.0	0.0	0.0	0.0	0.0
2.0	0.0	0.0	0.0	0.0	0.0	0.0	0.0
2.0	0.0	0.0	0.0	0.0	0.0	0.0	0.0
0.0	0.0	0.0	0.0	0.0	0.0	0.0	0.0
1.0	0.0	0.0	0.0	0.0	0.0	0.0	0.0
0.0	0.0	0.0	0.0	0.0	0.0	0.0	0.0
2.0	0.0	0.0	0.0	0.0	0.0	0.0	0.0
1.0	0.0	0.0	0.0	0.0	0.0	0.0	0.0
1.0	0.0	0.0	0.0	0.0	0.0	0.0	0.0
1.0	0.0	0.0	0.0	0.0	0.0	0.0	0.0
1.0	0.0	0.0	0.0	0.0	0.0	0.0	0.0
1.0	0.0	2.0	0.0	0.0	0.0	0.0	0.0
2.0	0.0	0.0	0.0	0.0	0.0	0.0	0.0
2.0	0.0	0.0	0.0	0.0	0.0	0.0	0.0
1.0	0.0	0.0	0.0	0.0	0.0	0.0	0.0
2.0	0.0	0.0	0.0	0.0	0.0	0.0	0.0
1.0	0.0	0.0	0.0	0.0	0.0	0.0	0.0
2.0	0.0	0.0	0.0	0.0	0.0	0.0	0.0
1.0	0.0	0.0	0.0	0.0	0.0	0.0	0.0
1.0	0.0	0.0	0.0	0.0	0.0	0.0	0.0
2.0	0.0	0.0	0.0	0.0	0.0	0.0	0.0
1.0	0.0	0.0	0.0	0.0	0.0	0.0	0.0
1.0	0.0	0.0	0.0	0.0	0.0	0.0	0.0
2.0	0.0	0.0	0.0	0.0	0.0	0.0	0.0

1.0	0.0	0.0	0.0	0.0	0.0	0.0	0.0
1.0	0.0	0.0	0.0	0.0	0.0	0.0	0.0
2.0	0.0	0.0	0.0	0.0	0.0	0.0	0.0
1.0	0.0	0.0	0.0	0.0	0.0	0.0	0.0
1.0	0.0	0.0	0.0	0.0	0.0	0.0	0.0
1.0	0.0	0.0	0.0	0.0	0.0	0.0	0.0
1.0	0.0	0.0	0.0	0.0	0.0	0.0	0.0
1.0	0.0	0.0	0.0	0.0	0.0	0.0	0.0
1.0	0.0	0.0	0.0	0.0	0.0	0.0	0.0
1.0	0.0	0.0	0.0	0.0	0.0	0.0	0.0
1.0	0.0	0.0	0.0	0.0	0.0	0.0	0.0
1.0	0.0	0.0	0.0	0.0	0.0	0.0	0.0
1.0	0.0	0.0	0.0	0.0	0.0	0.0	0.0
1.0	0.0	0.0	0.0	0.0	0.0	0.0	0.0
1.0	0.0	0.0	0.0	0.0	0.0	0.0	0.0
1.0	0.0	0.0	0.0	0.0	0.0	0.0	0.0
1.0	0.0	0.0	0.0	0.0	0.0	0.0	0.0
1.0	0.0	0.0	0.0	0.0	0.0	0.0	0.0
1.0	0.0	0.0	0.0	0.0	0.0	0.0	0.0
1.0	0.0	0.0	0.0	0.0	0.0	0.0	0.0
1.0	0.0	0.0	0.0	0.0	0.0	0.0	0.0
0.0	6.0	0.0	0.0	0.0	0.0	0.0	0.0
0.0	3.0	0.0	0.0	0.0	0.0	0.0	0.0
0.0	2.0	0.0	0.0	0.0	0.0	0.0	0.0
0.0	3.0	0.0	0.0	0.0	0.0	0.0	0.0
0.0	5.0	0.0	0.0	0.0	0.0	0.0	0.0
0.0	0.0	0.0	0.0	0.0	0.0	0.0	0.0
0.0	1.0	0.0	0.0	0.0	0.0	0.0	0.0
0.0	0.0	0.0	0.0	0.0	0.0	0.0	0.0
0.0	0.0	0.0	0.0	0.0	4.0	0.0	0.0
0.0	2.0	0.0	0.0	0.0	0.0	0.0	0.0
0.0	0.0	0.0	0.0	0.0	0.0	0.0	0.0
0.0	1.0	0.0	0.0	0.0	0.0	0.0	0.0
0.0	1.0	0.0	0.0	0.0	0.0	0.0	0.0
0.0	1.0	0.0	0.0	0.0	0.0	0.0	0.0
0.0	1.0	0.0	0.0	0.0	0.0	0.0	0.0
0.0	1.0	0.0	0.0	0.0	0.0	0.0	0.0
0.0	1.0	0.0	0.0	0.0	0.0	0.0	0.0
0.0	1.0	0.0	0.0	0.0	0.0	0.0	0.0
0.0	0.0	58.0	0.0	0.0	0.0	0.0	0.0
0.0	0.0	12.0	0.0	0.0	0.0	0.0	0.0
0.0	0.0	9.0	0.0	0.0	0.0	0.0	0.0
0.0	0.0	10.0	0.0	0.0	0.0	0.0	0.0
0.0	0.0	8.0	0.0	0.0	0.0	0.0	0.0
0.0	0.0	6.0	0.0	0.0	0.0	0.0	0.0
0.0	0.0	6.0	0.0	0.0	0.0	0.0	0.0
0.0	0.0	4.0	0.0	0.0	0.0	0.0	0.0

0.0	0.0	4.0	0.0	0.0	0.0	0.0	0.0
0.0	0.0	3.0	0.0	0.0	0.0	0.0	0.0
0.0	0.0	3.0	0.0	0.0	0.0	0.0	0.0
0.0	0.0	4.0	0.0	0.0	0.0	0.0	0.0
0.0	0.0	2.0	0.0	0.0	0.0	0.0	0.0
0.0	0.0	3.0	0.0	0.0	0.0	0.0	0.0
0.0	0.0	4.0	0.0	0.0	0.0	0.0	0.0
0.0	0.0	1.0	0.0	0.0	0.0	0.0	0.0
0.0	0.0	2.0	0.0	0.0	0.0	0.0	0.0
0.0	0.0	3.0	0.0	0.0	0.0	0.0	0.0
0.0	0.0	2.0	0.0	0.0	0.0	0.0	0.0
0.0	0.0	3.0	0.0	0.0	0.0	0.0	0.0
0.0	0.0	1.0	0.0	0.0	0.0	0.0	0.0
0.0	0.0	3.0	0.0	0.0	0.0	0.0	0.0
0.0	0.0	2.0	0.0	0.0	0.0	0.0	0.0
0.0	0.0	3.0	0.0	0.0	0.0	0.0	0.0
0.0	0.0	3.0	0.0	0.0	0.0	0.0	0.0
0.0	0.0	1.0	0.0	0.0	0.0	0.0	0.0
0.0	0.0	1.0	0.0	0.0	0.0	0.0	0.0
0.0	0.0	1.0	0.0	0.0	0.0	0.0	0.0
0.0	0.0	1.0	0.0	3.0	0.0	0.0	0.0
0.0	0.0	1.0	0.0	0.0	0.0	0.0	0.0
0.0	0.0	1.0	0.0	0.0	0.0	0.0	0.0
0.0	0.0	1.0	0.0	0.0	0.0	0.0	0.0
0.0	0.0	1.0	0.0	0.0	0.0	0.0	0.0
0.0	0.0	2.0	0.0	3.0	0.0	0.0	1.0
0.0	0.0	0.0	0.0	0.0	0.0	0.0	0.0
0.0	0.0	2.0	0.0	0.0	0.0	0.0	0.0
0.0	0.0	2.0	0.0	0.0	0.0	0.0	0.0
0.0	0.0	2.0	0.0	0.0	0.0	0.0	0.0
0.0	0.0	1.0	0.0	0.0	0.0	0.0	0.0
0.0	0.0	1.0	0.0	0.0	0.0	0.0	0.0
0.0	0.0	2.0	0.0	0.0	0.0	0.0	0.0
0.0	0.0	2.0	0.0	0.0	0.0	0.0	0.0
0.0	0.0	2.0	0.0	0.0	0.0	0.0	0.0
0.0	0.0	1.0	0.0	0.0	0.0	0.0	0.0
0.0	0.0	2.0	0.0	0.0	0.0	0.0	0.0
0.0	0.0	1.0	0.0	0.0	0.0	0.0	0.0
0.0	0.0	1.0	0.0	0.0	0.0	0.0	0.0
0.0	0.0	1.0	0.0	0.0	0.0	0.0	0.0
0.0	0.0	1.0	0.0	0.0	0.0	0.0	0.0
0.0	0.0	1.0	0.0	0.0	0.0	0.0	0.0
0.0	0.0	1.0	0.0	0.0	0.0	0.0	0.0
0.0	0.0	1.0	0.0	0.0	0.0	0.0	0.0
0.0	0.0	0.0	0.0	0.0	0.0	0.0	0.0
0.0	0.0	1.0	0.0	0.0	0.0	0.0	0.0
0.0	0.0	1.0	0.0	0.0	0.0	0.0	0.0

0.0	0.0	1.0	0.0	0.0	0.0	0.0	0.0
0.0	0.0	0.0	0.0	50.0	0.0	0.0	0.0
0.0	0.0	2.0	0.0	0.0	0.0	0.0	0.0
0.0	0.0	2.0	0.0	0.0	0.0	0.0	0.0
0.0	0.0	1.0	0.0	0.0	0.0	0.0	0.0
0.0	0.0	1.0	0.0	0.0	0.0	0.0	0.0
0.0	0.0	1.0	0.0	0.0	0.0	0.0	0.0
0.0	0.0	1.0	0.0	0.0	0.0	0.0	0.0
0.0	0.0	1.0	0.0	0.0	0.0	0.0	0.0
0.0	0.0	1.0	0.0	0.0	0.0	0.0	0.0
0.0	0.0	1.0	0.0	0.0	0.0	0.0	0.0
0.0	0.0	1.0	0.0	0.0	0.0	0.0	0.0
0.0	0.0	1.0	0.0	0.0	0.0	0.0	0.0
0.0	0.0	1.0	0.0	0.0	0.0	0.0	0.0
0.0	0.0	1.0	0.0	0.0	0.0	0.0	0.0
0.0	0.0	1.0	0.0	0.0	0.0	0.0	0.0
0.0	0.0	1.0	0.0	0.0	0.0	0.0	0.0
0.0	0.0	1.0	0.0	0.0	0.0	0.0	0.0
0.0	0.0	0.0	95.0	0.0	0.0	0.0	0.0
0.0	0.0	0.0	79.0	0.0	0.0	0.0	0.0
0.0	0.0	0.0	59.0	0.0	0.0	0.0	0.0
0.0	0.0	0.0	48.0	0.0	0.0	0.0	0.0
0.0	0.0	0.0	49.0	4.0	0.0	0.0	0.0
0.0	0.0	0.0	41.0	0.0	0.0	0.0	0.0
0.0	0.0	0.0	32.0	0.0	0.0	0.0	0.0
0.0	0.0	0.0	24.0	0.0	0.0	0.0	1.0
0.0	0.0	0.0	13.0	0.0	0.0	0.0	0.0
0.0	0.0	0.0	6.0	0.0	0.0	0.0	0.0
0.0	0.0	0.0	5.0	0.0	0.0	57.0	0.0
0.0	0.0	0.0	5.0	0.0	0.0	0.0	0.0
0.0	0.0	0.0	3.0	0.0	0.0	0.0	0.0
0.0	0.0	0.0	5.0	0.0	0.0	0.0	0.0
0.0	0.0	0.0	5.0	0.0	0.0	5.0	2.0
0.0	0.0	0.0	4.0	1.0	0.0	0.0	0.0
0.0	0.0	0.0	5.0	0.0	0.0	0.0	0.0
0.0	0.0	0.0	2.0	0.0	0.0	3.0	0.0
0.0	0.0	0.0	1.0	0.0	0.0	0.0	0.0
0.0	0.0	0.0	1.0	0.0	0.0	0.0	0.0
0.0	0.0	0.0	2.0	0.0	0.0	0.0	0.0
0.0	0.0	0.0	2.0	0.0	0.0	0.0	0.0
0.0	0.0	0.0	1.0	0.0	0.0	0.0	0.0
0.0	0.0	0.0	2.0	0.0	0.0	0.0	0.0
0.0	0.0	0.0	1.0	0.0	0.0	0.0	0.0
0.0	0.0	0.0	2.0	0.0	0.0	0.0	0.0
0.0	0.0	0.0	2.0	0.0	0.0	0.0	0.0
0.0	0.0	0.0	2.0	0.0	0.0	0.0	0.0
0.0	0.0	0.0	1.0	0.0	0.0	10.0	0.0

0.0	0.0	0.0	2.0	0.0	0.0	0.0	0.0
0.0	0.0	0.0	2.0	0.0	0.0	0.0	0.0
0.0	0.0	0.0	2.0	0.0	0.0	0.0	0.0
0.0	0.0	0.0	0.0	0.0	0.0	0.0	2.0
0.0	0.0	0.0	1.0	0.0	0.0	0.0	0.0
0.0	0.0	0.0	1.0	0.0	0.0	0.0	0.0
0.0	0.0	0.0	1.0	0.0	0.0	0.0	0.0
0.0	0.0	0.0	1.0	0.0	0.0	0.0	0.0
0.0	0.0	0.0	0.0	128.0	0.0	0.0	0.0
0.0	0.0	0.0	0.0	108.0	0.0	0.0	0.0
0.0	0.0	0.0	0.0	18.0	0.0	0.0	0.0
0.0	0.0	0.0	0.0	22.0	0.0	0.0	0.0
0.0	0.0	0.0	0.0	17.0	0.0	0.0	0.0
0.0	0.0	0.0	0.0	17.0	0.0	0.0	0.0
0.0	0.0	0.0	0.0	15.0	0.0	0.0	0.0
0.0	0.0	0.0	0.0	14.0	0.0	0.0	0.0
0.0	0.0	0.0	0.0	12.0	0.0	0.0	0.0
0.0	0.0	0.0	0.0	13.0	0.0	0.0	0.0
0.0	0.0	0.0	0.0	16.0	0.0	0.0	843.0
0.0	0.0	0.0	0.0	7.0	0.0	0.0	0.0
0.0	0.0	0.0	0.0	9.0	0.0	0.0	0.0
0.0	0.0	0.0	0.0	11.0	0.0	0.0	0.0
0.0	0.0	0.0	0.0	6.0	0.0	0.0	0.0
0.0	0.0	0.0	0.0	9.0	0.0	0.0	0.0
0.0	0.0	0.0	0.0	10.0	0.0	0.0	0.0
0.0	0.0	0.0	0.0	4.0	0.0	0.0	0.0
0.0	0.0	0.0	0.0	9.0	0.0	0.0	0.0
0.0	0.0	0.0	0.0	6.0	0.0	0.0	0.0
0.0	0.0	0.0	0.0	7.0	0.0	0.0	0.0
0.0	0.0	0.0	0.0	5.0	0.0	0.0	0.0
0.0	0.0	0.0	0.0	5.0	0.0	0.0	0.0
0.0	0.0	0.0	0.0	3.0	0.0	0.0	0.0
0.0	0.0	0.0	0.0	4.0	0.0	0.0	0.0
0.0	0.0	0.0	0.0	3.0	0.0	0.0	0.0
0.0	0.0	0.0	0.0	3.0	0.0	0.0	0.0
0.0	0.0	0.0	0.0	4.0	0.0	0.0	0.0
0.0	0.0	0.0	0.0	4.0	0.0	0.0	0.0
0.0	0.0	0.0	0.0	2.0	0.0	0.0	0.0
0.0	0.0	0.0	0.0	3.0	0.0	0.0	0.0
0.0	0.0	0.0	0.0	4.0	0.0	0.0	0.0
0.0	0.0	0.0	0.0	1.0	0.0	0.0	0.0
0.0	0.0	0.0	0.0	1.0	0.0	0.0	0.0
0.0	0.0	0.0	0.0	2.0	0.0	0.0	0.0
0.0	0.0	0.0	0.0	1.0	0.0	0.0	0.0
0.0	0.0	0.0	0.0	1.0	0.0	0.0	0.0
0.0	0.0	0.0	0.0	2.0	0.0	0.0	0.0
0.0	0.0	0.0	0.0	1.0	0.0	0.0	0.0

0.0	0.0	0.0	0.0	2.0	0.0	0.0	0.0
0.0	0.0	0.0	0.0	0.0	0.0	0.0	0.0
0.0	0.0	0.0	0.0	1.0	0.0	0.0	0.0
0.0	0.0	0.0	0.0	1.0	0.0	0.0	0.0
0.0	0.0	0.0	0.0	1.0	0.0	0.0	0.0
0.0	0.0	0.0	0.0	0.0	0.0	0.0	0.0
0.0	0.0	0.0	0.0	1.0	0.0	0.0	2.0
0.0	0.0	0.0	0.0	2.0	0.0	0.0	0.0
0.0	0.0	0.0	0.0	0.0	0.0	0.0	0.0
0.0	0.0	0.0	0.0	2.0	0.0	0.0	0.0
0.0	0.0	0.0	0.0	0.0	0.0	0.0	0.0
0.0	0.0	0.0	0.0	2.0	0.0	0.0	0.0
0.0	0.0	0.0	0.0	1.0	0.0	0.0	0.0
0.0	0.0	0.0	0.0	0.0	0.0	0.0	0.0
0.0	0.0	0.0	0.0	1.0	0.0	0.0	0.0
0.0	0.0	0.0	0.0	1.0	0.0	0.0	0.0
0.0	0.0	0.0	0.0	1.0	0.0	0.0	1.0
0.0	0.0	0.0	0.0	0.0	0.0	0.0	0.0
0.0	0.0	0.0	0.0	2.0	0.0	0.0	0.0
0.0	0.0	0.0	0.0	1.0	0.0	0.0	0.0
0.0	0.0	0.0	0.0	1.0	0.0	0.0	0.0
0.0	0.0	0.0	0.0	0.0	0.0	0.0	0.0
0.0	0.0	0.0	0.0	1.0	0.0	0.0	0.0
0.0	0.0	0.0	0.0	1.0	0.0	0.0	0.0
0.0	0.0	0.0	0.0	1.0	0.0	0.0	0.0
0.0	0.0	0.0	0.0	0.0	0.0	0.0	0.0
0.0	0.0	0.0	0.0	2.0	0.0	1.0	0.0
0.0	0.0	0.0	0.0	1.0	0.0	0.0	0.0
0.0	0.0	0.0	0.0	0.0	0.0	0.0	0.0
0.0	0.0	0.0	0.0	0.0	5.0	0.0	0.0
0.0	0.0	0.0	0.0	0.0	1.0	0.0	0.0
0.0	0.0	0.0	0.0	0.0	2.0	0.0	0.0
0.0	0.0	0.0	0.0	0.0	2.0	0.0	0.0
0.0	0.0	0.0	0.0	0.0	0.0	0.0	0.0
0.0	0.0	0.0	0.0	0.0	1.0	0.0	0.0
0.0	0.0	0.0	0.0	0.0	2.0	0.0	0.0
0.0	0.0	0.0	0.0	0.0	1.0	0.0	0.0
0.0	0.0	0.0	0.0	0.0	1.0	0.0	0.0
0.0	0.0	0.0	0.0	0.0	1.0	0.0	0.0
0.0	0.0	0.0	0.0	0.0	0.0	0.0	0.0
0.0	0.0	0.0	0.0	0.0	1.0	0.0	0.0
0.0	0.0	0.0	0.0	0.0	1.0	0.0	0.0
0.0	0.0	0.0	0.0	0.0	2.0	0.0	0.0
0.0	0.0	0.0	0.0	0.0	1.0	0.0	0.0
0.0	0.0	0.0	0.0	0.0	0.0	0.0	0.0
0.0	0.0	0.0	0.0	0.0	1.0	0.0	0.0
0.0	0.0	0.0	0.0	0.0	1.0	0.0	0.0

0.0	0.0	0.0	0.0	0.0	0.0	47.0	0.0
0.0	0.0	0.0	0.0	0.0	0.0	12.0	0.0
0.0	0.0	0.0	0.0	0.0	0.0	8.0	0.0
0.0	0.0	0.0	0.0	0.0	0.0	7.0	0.0
0.0	0.0	0.0	0.0	0.0	0.0	3.0	0.0
0.0	0.0	0.0	0.0	0.0	0.0	4.0	0.0
0.0	0.0	0.0	0.0	0.0	0.0	2.0	0.0
0.0	0.0	0.0	0.0	0.0	0.0	2.0	0.0
0.0	0.0	0.0	0.0	0.0	0.0	3.0	0.0
0.0	0.0	0.0	0.0	0.0	0.0	4.0	0.0
0.0	0.0	0.0	0.0	0.0	0.0	3.0	0.0
0.0	0.0	0.0	0.0	0.0	0.0	2.0	0.0
0.0	0.0	0.0	0.0	0.0	0.0	2.0	0.0
0.0	0.0	0.0	0.0	0.0	0.0	2.0	0.0
0.0	0.0	0.0	0.0	0.0	0.0	2.0	0.0
0.0	0.0	0.0	0.0	0.0	0.0	1.0	0.0
0.0	0.0	0.0	0.0	0.0	0.0	0.0	0.0
0.0	0.0	0.0	0.0	0.0	0.0	1.0	0.0
0.0	0.0	0.0	0.0	0.0	0.0	0.0	0.0
0.0	0.0	0.0	0.0	0.0	0.0	0.0	1567.0
0.0	0.0	0.0	0.0	0.0	0.0	0.0	3.0
0.0	0.0	0.0	0.0	0.0	0.0	0.0	2.0
0.0	0.0	0.0	0.0	0.0	0.0	0.0	1.0
0.0	0.0	0.0	0.0	0.0	0.0	0.0	3.0
0.0	0.0	0.0	0.0	0.0	0.0	0.0	1.0
0.0	0.0	0.0	0.0	0.0	0.0	0.0	3.0
0.0	0.0	0.0	0.0	0.0	0.0	0.0	2.0
0.0	0.0	0.0	0.0	0.0	0.0	0.0	3.0
0.0	0.0	0.0	0.0	0.0	0.0	0.0	2.0
0.0	0.0	0.0	0.0	0.0	0.0	0.0	3.0
0.0	0.0	0.0	0.0	0.0	0.0	0.0	1.0
0.0	0.0	0.0	0.0	0.0	0.0	0.0	2.0
0.0	0.0	0.0	0.0	0.0	0.0	0.0	2.0
0.0	0.0	0.0	0.0	0.0	0.0	0.0	1.0
0.0	0.0	0.0	0.0	0.0	0.0	0.0	2.0
0.0	0.0	0.0	0.0	0.0	0.0	0.0	1.0
0.0	0.0	0.0	0.0	0.0	0.0	0.0	1.0
0.0	0.0	0.0	0.0	0.0	0.0	0.0	1.0
0.0	0.0	0.0	0.0	0.0	0.0	0.0	1.0
0.0	0.0	0.0	0.0	0.0	0.0	0.0	1.0
0.0	0.0	0.0	0.0	0.0	0.0	0.0	2.0
0.0	0.0	0.0	0.0	0.0	0.0	0.0	1.0
0.0	0.0	0.0	0.0	0.0	0.0	0.0	2.0
0.0	0.0	0.0	0.0	0.0	0.0	0.0	1.0
0.0	0.0	0.0	0.0	0.0	0.0	0.0	1.0
0.0	0.0	0.0	0.0	0.0	0.0	0.0	2.0
0.0	0.0	0.0	0.0	0.0	0.0	0.0	1.0

0.0	0.0	0.0	0.0	0.0	0.0	0.0	1.0
0.0	0.0	0.0	0.0	0.0	0.0	0.0	1.0
0.0	0.0	0.0	0.0	0.0	0.0	0.0	1.0
0.0	0.0	0.0	0.0	0.0	0.0	0.0	0.0
0.0	0.0	0.0	0.0	0.0	0.0	0.0	1.0
0.0	0.0	0.0	0.0	0.0	0.0	0.0	1.0
0.0	0.0	0.0	0.0	0.0	0.0	0.0	0.0
0.0	0.0	0.0	0.0	0.0	0.0	0.0	0.0
0.0	0.0	0.0	0.0	0.0	0.0	0.0	1.0
0.0	0.0	0.0	0.0	0.0	0.0	0.0	1.0
0.0	0.0	0.0	0.0	0.0	0.0	0.0	1.0
0.0	0.0	0.0	0.0	0.0	0.0	0.0	1.0
0.0	0.0	0.0	0.0	0.0	0.0	0.0	1.0
0.0	0.0	0.0	0.0	0.0	0.0	0.0	1.0
0.0	0.0	0.0	0.0	0.0	0.0	0.0	0.0
0.0	0.0	0.0	0.0	0.0	0.0	0.0	0.0
0.0	0.0	0.0	0.0	0.0	0.0	0.0	0.0
0.0	0.0	0.0	0.0	0.0	0.0	0.0	0.0
0.0	0.0	0.0	0.0	0.0	0.0	0.0	0.0
0.0	0.0	0.0	0.0	0.0	0.0	0.0	0.0
0.0	0.0	0.0	0.0	0.0	0.0	0.0	0.0
0.0	0.0	0.0	0.0	0.0	0.0	0.0	0.0
0.0	0.0	0.0	0.0	0.0	0.0	0.0	0.0
0.0	0.0	0.0	0.0	0.0	0.0	0.0	0.0
0.0	0.0	0.0	0.0	0.0	0.0	0.0	0.0
0.0	0.0	0.0	0.0	0.0	0.0	0.0	0.0
0.0	0.0	0.0	0.0	0.0	0.0	0.0	0.0
0.0	0.0	0.0	0.0	0.0	0.0	0.0	0.0
0.0	0.0	0.0	0.0	0.0	0.0	0.0	0.0
0.0	0.0	0.0	0.0	0.0	0.0	0.0	0.0
0.0	0.0	0.0	0.0	0.0	0.0	0.0	0.0
0.0	0.0	0.0	0.0	0.0	0.0	0.0	0.0
0.0	0.0	0.0	0.0	0.0	0.0	0.0	0.0
0.0	0.0	0.0	0.0	0.0	0.0	0.0	0.0
0.0	0.0	0.0	0.0	0.0	0.0	0.0	0.0
0.0	0.0	0.0	0.0	0.0	0.0	0.0	0.0
0.0	0.0	0.0	0.0	0.0	0.0	0.0	0.0
0.0	0.0	0.0	0.0	0.0	0.0	0.0	0.0
0.0	0.0	0.0	0.0	0.0	0.0	0.0	0.0
0.0	0.0	0.0	0.0	0.0	0.0	0.0	0.0
0.0	0.0	0.0	0.0	0.0	0.0	0.0	0.0
0.0	0.0	0.0	0.0	0.0	0.0	0.0	0.0
0.0	0.0	0.0	0.0	0.0	0.0	0.0	0.0
0.0	0.0	0.0	0.0	0.0	0.0	0.0	0.0
0.0	0.0	0.0	0.0	0.0	0.0	0.0	0.0
0.0	0.0	0.0	0.0	0.0	0.0	0.0	0.0
0.0	0.0	0.0	0.0	0.0	0.0	0.0	0.0

0.0	0.0	0.0	0.0	0.0	0.0	0.0	0.0
0.0	0.0	0.0	0.0	0.0	0.0	0.0	0.0
0.0	0.0	0.0	0.0	0.0	0.0	0.0	0.0
0.0	0.0	0.0	0.0	0.0	0.0	0.0	0.0
0.0	0.0	0.0	0.0	0.0	0.0	0.0	0.0
0.0	0.0	0.0	0.0	0.0	0.0	0.0	0.0
0.0	0.0	0.0	0.0	0.0	0.0	0.0	0.0
0.0	0.0	0.0	0.0	0.0	0.0	0.0	0.0
0.0	0.0	0.0	0.0	0.0	0.0	0.0	0.0
0.0	0.0	0.0	0.0	0.0	0.0	0.0	0.0
0.0	0.0	0.0	0.0	0.0	0.0	0.0	0.0
0.0	0.0	0.0	0.0	0.0	0.0	0.0	0.0
0.0	0.0	0.0	0.0	0.0	0.0	0.0	0.0
0.0	0.0	0.0	0.0	0.0	0.0	0.0	0.0
0.0	0.0	0.0	0.0	0.0	0.0	0.0	0.0
0.0	0.0	0.0	0.0	0.0	0.0	0.0	0.0
0.0	0.0	0.0	0.0	0.0	0.0	0.0	0.0
0.0	0.0	0.0	0.0	0.0	0.0	0.0	0.0
0.0	0.0	0.0	0.0	0.0	0.0	0.0	0.0
0.0	0.0	0.0	0.0	0.0	0.0	0.0	0.0
0.0	0.0	0.0	0.0	0.0	0.0	0.0	0.0
0.0	0.0	0.0	0.0	0.0	0.0	0.0	0.0
0.0	0.0	0.0	0.0	0.0	0.0	0.0	0.0
0.0	0.0	0.0	0.0	0.0	0.0	0.0	0.0
0.0	0.0	0.0	0.0	0.0	0.0	0.0	0.0
0.0	0.0	0.0	0.0	0.0	0.0	0.0	0.0
0.0	0.0	0.0	0.0	0.0	0.0	0.0	0.0
0.0	0.0	0.0	0.0	0.0	0.0	0.0	0.0
0.0	0.0	0.0	0.0	0.0	0.0	0.0	0.0
0.0	0.0	0.0	0.0	0.0	0.0	0.0	0.0
0.0	0.0	0.0	0.0	0.0	0.0	0.0	0.0
0.0	0.0	0.0	0.0	0.0	0.0	0.0	0.0
0.0	0.0	0.0	0.0	0.0	0.0	0.0	0.0
0.0	0.0	0.0	0.0	0.0	0.0	0.0	0.0
0.0	0.0	0.0	0.0	0.0	0.0	0.0	0.0
0.0	0.0	0.0	0.0	0.0	0.0	0.0	0.0
0.0	0.0	0.0	0.0	0.0	0.0	0.0	0.0
0.0	0.0	0.0	0.0	0.0	0.0	0.0	0.0
0.0	0.0	0.0	0.0	0.0	0.0	0.0	0.0
0.0	0.0	0.0	0.0	0.0	0.0	0.0	0.0
0.0	0.0	0.0	0.0	0.0	0.0	0.0	0.0
0.0	0.0	0.0	0.0	0.0	0.0	0.0	0.0
0.0	0.0	0.0	0.0	0.0	0.0	0.0	0.0
0.0	0.0	0.0	0.0	0.0	0.0	0.0	0.0
0.0	0.0	0.0	0.0	0.0	0.0	0.0	0.0
0.0	0.0	0.0	0.0	0.0	0.0	0.0	0.0
0.0	0.0	0.0	0.0	0.0	0.0	0.0	0.0
0.0	0.0	0.0	0.0	0.0	0.0	0.0	0.0

0.0	0.0	0.0	0.0	0.0	0.0	0.0	0.0
0.0	0.0	0.0	0.0	0.0	0.0	0.0	0.0
0.0	0.0	0.0	0.0	0.0	0.0	0.0	0.0
0.0	0.0	0.0	0.0	0.0	0.0	0.0	0.0
0.0	0.0	0.0	0.0	0.0	0.0	0.0	0.0
0.0	0.0	0.0	0.0	0.0	0.0	0.0	0.0
0.0	0.0	0.0	0.0	0.0	0.0	0.0	0.0
0.0	0.0	0.0	0.0	0.0	0.0	0.0	0.0
0.0	0.0	0.0	0.0	0.0	0.0	0.0	0.0
0.0	0.0	0.0	0.0	0.0	0.0	0.0	0.0
0.0	0.0	0.0	0.0	0.0	0.0	0.0	0.0
0.0	0.0	0.0	0.0	0.0	0.0	0.0	0.0
0.0	0.0	0.0	0.0	0.0	0.0	0.0	0.0
0.0	0.0	0.0	0.0	0.0	0.0	0.0	0.0
0.0	0.0	0.0	0.0	0.0	0.0	0.0	0.0
0.0	0.0	0.0	0.0	0.0	0.0	0.0	0.0
0.0	0.0	0.0	0.0	0.0	0.0	0.0	0.0
0.0	0.0	0.0	0.0	0.0	0.0	0.0	0.0
0.0	0.0	0.0	0.0	0.0	0.0	0.0	0.0
0.0	0.0	0.0	0.0	0.0	0.0	0.0	0.0
0.0	0.0	0.0	0.0	0.0	0.0	0.0	0.0
0.0	0.0	0.0	0.0	0.0	0.0	0.0	0.0
0.0	0.0	0.0	0.0	0.0	0.0	0.0	0.0
0.0	0.0	0.0	0.0	0.0	0.0	0.0	0.0
0.0	0.0	0.0	0.0	0.0	0.0	0.0	0.0
0.0	0.0	0.0	0.0	0.0	0.0	0.0	0.0
0.0	0.0	0.0	0.0	0.0	0.0	0.0	0.0
0.0	0.0	0.0	0.0	0.0	0.0	0.0	0.0
0.0	0.0	0.0	0.0	0.0	0.0	0.0	0.0
0.0	0.0	0.0	0.0	0.0	0.0	0.0	0.0
0.0	0.0	0.0	0.0	0.0	0.0	0.0	0.0
0.0	0.0	0.0	0.0	0.0	0.0	0.0	0.0
0.0	0.0	0.0	0.0	0.0	0.0	0.0	0.0
0.0	0.0	0.0	0.0	0.0	0.0	0.0	0.0
0.0	0.0	0.0	0.0	0.0	0.0	0.0	0.0
0.0	0.0	0.0	0.0	0.0	0.0	0.0	0.0
0.0	0.0	0.0	0.0	0.0	0.0	0.0	0.0
0.0	0.0	0.0	0.0	0.0	0.0	0.0	0.0
0.0	0.0	0.0	0.0	0.0	0.0	0.0	0.0
0.0	0.0	0.0	0.0	0.0	0.0	0.0	0.0
0.0	0.0	0.0	0.0	0.0	0.0	0.0	0.0
0.0	0.0	0.0	0.0	0.0	0.0	0.0	0.0
0.0	0.0	0.0	0.0	0.0	0.0	0.0	0.0
0.0	0.0	0.0	0.0	0.0	0.0	0.0	0.0
0.0	0.0	0.0	0.0	0.0	0.0	0.0	0.0
0.0	0.0	0.0	0.0	0.0	0.0	0.0	0.0
0.0	0.0	0.0	0.0	0.0	0.0	0.0	0.0

0.0	0.0	0.0	0.0	0.0	0.0	0.0	0.0
0.0	0.0	0.0	0.0	0.0	0.0	0.0	0.0
0.0	0.0	0.0	0.0	0.0	0.0	0.0	0.0
0.0	0.0	0.0	0.0	0.0	0.0	0.0	0.0
0.0	0.0	0.0	0.0	0.0	0.0	0.0	0.0
0.0	0.0	0.0	0.0	0.0	0.0	0.0	0.0
0.0	0.0	0.0	0.0	0.0	0.0	0.0	0.0
0.0	0.0	0.0	0.0	0.0	0.0	0.0	0.0
0.0	0.0	0.0	0.0	0.0	0.0	0.0	0.0
0.0	0.0	0.0	0.0	0.0	0.0	0.0	0.0
0.0	0.0	0.0	0.0	0.0	0.0	0.0	0.0
0.0	0.0	0.0	0.0	0.0	0.0	0.0	0.0
0.0	0.0	0.0	0.0	0.0	0.0	0.0	0.0
0.0	0.0	0.0	0.0	0.0	0.0	0.0	0.0
0.0	0.0	0.0	0.0	0.0	0.0	0.0	0.0
0.0	0.0	0.0	0.0	0.0	0.0	0.0	0.0
0.0	0.0	0.0	0.0	0.0	0.0	0.0	0.0
0.0	0.0	0.0	0.0	0.0	0.0	0.0	0.0
0.0	0.0	0.0	0.0	0.0	0.0	0.0	0.0
0.0	0.0	0.0	0.0	0.0	0.0	0.0	0.0
0.0	0.0	0.0	0.0	0.0	0.0	0.0	0.0
0.0	0.0	0.0	0.0	0.0	0.0	0.0	0.0
0.0	0.0	0.0	0.0	0.0	0.0	0.0	0.0
0.0	0.0	0.0	0.0	0.0	0.0	0.0	0.0
0.0	0.0	0.0	0.0	0.0	0.0	0.0	0.0
0.0	0.0	0.0	0.0	0.0	0.0	0.0	0.0
0.0	0.0	0.0	0.0	0.0	0.0	0.0	0.0
0.0	0.0	0.0	0.0	0.0	0.0	0.0	0.0
0.0	0.0	0.0	0.0	0.0	0.0	0.0	0.0
0.0	0.0	0.0	0.0	0.0	0.0	0.0	0.0
0.0	0.0	0.0	0.0	0.0	0.0	0.0	0.0
0.0	0.0	0.0	0.0	0.0	0.0	0.0	0.0
0.0	0.0	0.0	0.0	0.0	0.0	0.0	0.0
0.0	0.0	0.0	0.0	0.0	0.0	0.0	0.0
0.0	0.0	0.0	0.0	0.0	0.0	0.0	0.0
0.0	0.0	0.0	0.0	0.0	0.0	0.0	0.0
0.0	0.0	0.0	0.0	0.0	0.0	0.0	0.0
0.0	0.0	0.0	0.0	0.0	0.0	0.0	0.0
0.0	0.0	0.0	0.0	0.0	0.0	0.0	0.0
0.0	0.0	0.0	0.0	0.0	0.0	0.0	0.0
0.0	0.0	0.0	0.0	0.0	0.0	0.0	0.0
0.0	0.0	0.0	0.0	0.0	0.0	0.0	0.0
0.0	0.0	0.0	0.0	0.0	0.0	0.0	0.0
0.0	0.0	0.0	0.0	0.0	0.0	0.0	0.0
0.0	0.0	0.0	0.0	0.0	0.0	0.0	0.0
0.0	0.0	0.0	0.0	0.0	0.0	0.0	0.0
0.0	0.0	0.0	0.0	0.0	0.0	0.0	0.0

0.0	0.0	0.0	0.0	0.0	0.0	0.0	0.0
0.0	0.0	0.0	0.0	0.0	0.0	0.0	0.0
0.0	0.0	0.0	0.0	0.0	0.0	0.0	0.0
0.0	0.0	0.0	0.0	0.0	0.0	0.0	0.0
0.0	0.0	0.0	0.0	0.0	0.0	0.0	0.0
0.0	0.0	0.0	0.0	0.0	0.0	0.0	0.0
0.0	0.0	0.0	0.0	0.0	0.0	0.0	0.0
0.0	0.0	0.0	0.0	0.0	0.0	0.0	0.0
0.0	0.0	0.0	0.0	0.0	0.0	0.0	0.0
0.0	0.0	0.0	0.0	0.0	0.0	0.0	0.0
0.0	0.0	0.0	0.0	0.0	0.0	0.0	0.0
0.0	0.0	0.0	0.0	0.0	0.0	0.0	0.0
0.0	0.0	0.0	0.0	0.0	0.0	0.0	0.0
0.0	0.0	0.0	0.0	0.0	0.0	0.0	0.0
0.0	0.0	0.0	0.0	0.0	0.0	0.0	0.0
0.0	0.0	0.0	0.0	0.0	0.0	0.0	0.0
0.0	0.0	0.0	0.0	0.0	0.0	0.0	0.0
0.0	0.0	0.0	0.0	0.0	0.0	0.0	0.0
0.0	0.0	0.0	0.0	0.0	0.0	0.0	0.0
0.0	0.0	0.0	0.0	0.0	0.0	0.0	0.0
0.0	0.0	0.0	0.0	0.0	0.0	0.0	0.0
0.0	0.0	0.0	0.0	0.0	0.0	0.0	0.0
0.0	0.0	0.0	0.0	0.0	0.0	0.0	0.0
0.0	0.0	0.0	0.0	0.0	0.0	0.0	0.0
0.0	0.0	0.0	0.0	0.0	0.0	0.0	0.0
0.0	0.0	0.0	0.0	0.0	0.0	0.0	0.0
0.0	0.0	0.0	0.0	0.0	0.0	0.0	0.0
0.0	0.0	0.0	0.0	0.0	0.0	0.0	0.0
0.0	0.0	0.0	0.0	0.0	0.0	0.0	0.0
0.0	0.0	0.0	0.0	0.0	0.0	0.0	0.0
0.0	0.0	0.0	0.0	0.0	0.0	0.0	0.0
0.0	0.0	0.0	0.0	0.0	0.0	0.0	0.0
0.0	0.0	0.0	0.0	0.0	0.0	0.0	0.0
0.0	0.0	0.0	0.0	0.0	0.0	0.0	0.0
0.0	0.0	0.0	0.0	0.0	0.0	0.0	0.0
0.0	0.0	0.0	0.0	0.0	0.0	0.0	0.0
0.0	0.0	0.0	0.0	0.0	0.0	0.0	0.0
0.0	0.0	0.0	0.0	0.0	0.0	0.0	0.0
0.0	0.0	0.0	0.0	0.0	0.0	0.0	0.0
0.0	0.0	0.0	0.0	0.0	0.0	0.0	0.0
0.0	0.0	0.0	0.0	0.0	0.0	0.0	0.0
0.0	0.0	0.0	0.0	0.0	0.0	0.0	0.0
0.0	0.0	0.0	0.0	0.0	0.0	0.0	0.0
0.0	0.0	0.0	0.0	0.0	0.0	0.0	0.0
0.0	0.0	0.0	0.0	0.0	0.0	0.0	0.0
0.0	0.0	0.0	0.0	0.0	0.0	0.0	0.0
0.0	0.0	0.0	0.0	0.0	0.0	0.0	0.0

0.0	0.0	0.0	0.0	0.0	0.0	0.0	0.0
0.0	0.0	0.0	0.0	0.0	0.0	0.0	0.0
0.0	0.0	0.0	0.0	0.0	0.0	0.0	0.0
0.0	0.0	0.0	0.0	0.0	0.0	0.0	0.0
0.0	0.0	0.0	0.0	0.0	0.0	0.0	0.0
0.0	0.0	0.0	0.0	0.0	0.0	0.0	0.0
0.0	0.0	0.0	0.0	0.0	0.0	0.0	0.0
0.0	0.0	0.0	0.0	0.0	0.0	0.0	0.0
0.0	0.0	0.0	0.0	0.0	0.0	0.0	0.0
0.0	0.0	0.0	0.0	0.0	0.0	0.0	0.0
0.0	0.0	0.0	0.0	0.0	0.0	0.0	0.0
0.0	0.0	0.0	0.0	0.0	0.0	0.0	0.0
0.0	0.0	0.0	0.0	0.0	0.0	0.0	0.0
0.0	0.0	0.0	0.0	0.0	0.0	0.0	0.0
0.0	0.0	0.0	0.0	0.0	0.0	0.0	0.0
0.0	0.0	0.0	0.0	0.0	0.0	0.0	0.0
0.0	0.0	0.0	0.0	0.0	0.0	0.0	0.0
0.0	0.0	0.0	0.0	0.0	0.0	0.0	0.0
0.0	0.0	0.0	0.0	0.0	0.0	0.0	0.0
0.0	0.0	0.0	0.0	0.0	0.0	0.0	0.0
0.0	0.0	0.0	0.0	0.0	0.0	0.0	0.0
0.0	0.0	0.0	0.0	0.0	0.0	0.0	0.0
0.0	0.0	0.0	0.0	0.0	0.0	0.0	0.0
0.0	0.0	0.0	0.0	0.0	0.0	0.0	0.0
0.0	0.0	0.0	0.0	0.0	0.0	0.0	0.0
0.0	0.0	0.0	0.0	0.0	0.0	0.0	0.0
0.0	0.0	0.0	0.0	0.0	0.0	0.0	0.0
0.0	0.0	0.0	0.0	0.0	0.0	0.0	0.0
0.0	0.0	0.0	0.0	0.0	0.0	0.0	0.0
0.0	0.0	0.0	0.0	0.0	0.0	0.0	0.0
0.0	0.0	0.0	0.0	0.0	0.0	0.0	0.0
0.0	0.0	0.0	0.0	0.0	0.0	0.0	0.0
0.0	0.0	0.0	0.0	0.0	0.0	0.0	0.0
0.0	0.0	0.0	0.0	0.0	0.0	0.0	0.0
0.0	0.0	0.0	0.0	0.0	0.0	0.0	0.0
0.0	0.0	0.0	0.0	0.0	0.0	0.0	0.0
0.0	0.0	0.0	0.0	0.0	0.0	0.0	0.0
0.0	0.0	0.0	0.0	0.0	0.0	0.0	0.0
0.0	0.0	0.0	0.0	0.0	0.0	0.0	0.0
0.0	0.0	0.0	0.0	0.0	0.0	0.0	0.0
0.0	0.0	0.0	0.0	0.0	0.0	0.0	0.0
0.0	0.0	0.0	0.0	0.0	0.0	0.0	0.0
0.0	0.0	0.0	0.0	0.0	0.0	0.0	0.0
0.0	0.0	0.0	0.0	0.0	0.0	0.0	0.0
0.0	0.0	0.0	0.0	0.0	0.0	0.0	0.0
0.0	0.0	0.0	0.0	0.0	0.0	0.0	0.0
0.0	0.0	0.0	0.0	0.0	0.0	0.0	0.0
0.0	0.0	0.0	0.0	0.0	0.0	0.0	0.0

0.0	0.0	0.0	0.0	0.0	0.0	0.0	0.0
0.0	0.0	0.0	0.0	0.0	0.0	0.0	0.0
0.0	0.0	0.0	0.0	0.0	0.0	0.0	0.0
0.0	0.0	0.0	0.0	0.0	0.0	0.0	0.0
0.0	0.0	0.0	0.0	0.0	0.0	0.0	0.0
0.0	0.0	0.0	0.0	0.0	0.0	0.0	0.0
0.0	0.0	0.0	0.0	0.0	0.0	0.0	0.0
0.0	0.0	0.0	0.0	0.0	0.0	0.0	0.0
0.0	0.0	0.0	0.0	0.0	0.0	0.0	0.0
0.0	0.0	0.0	0.0	0.0	0.0	0.0	0.0
0.0	0.0	0.0	0.0	0.0	0.0	0.0	0.0
0.0	0.0	0.0	0.0	0.0	0.0	0.0	0.0
0.0	0.0	0.0	0.0	0.0	0.0	0.0	0.0
0.0	0.0	0.0	0.0	0.0	0.0	0.0	0.0
0.0	0.0	0.0	0.0	0.0	0.0	0.0	0.0
0.0	0.0	0.0	0.0	0.0	0.0	0.0	0.0
0.0	0.0	0.0	0.0	0.0	0.0	0.0	0.0
0.0	0.0	0.0	0.0	0.0	0.0	0.0	0.0
0.0	0.0	0.0	0.0	0.0	0.0	0.0	0.0
0.0	0.0	0.0	0.0	0.0	0.0	0.0	0.0
0.0	0.0	0.0	0.0	0.0	0.0	0.0	0.0
0.0	0.0	0.0	0.0	0.0	0.0	0.0	0.0
0.0	0.0	0.0	0.0	0.0	0.0	0.0	0.0
0.0	0.0	0.0	0.0	0.0	0.0	0.0	0.0
0.0	0.0	0.0	0.0	0.0	0.0	0.0	0.0
0.0	0.0	0.0	0.0	0.0	0.0	0.0	0.0
0.0	0.0	0.0	0.0	0.0	0.0	0.0	0.0
0.0	0.0	0.0	0.0	0.0	0.0	0.0	0.0
0.0	0.0	0.0	0.0	0.0	0.0	0.0	0.0
0.0	0.0	0.0	0.0	0.0	0.0	0.0	0.0
0.0	0.0	0.0	0.0	0.0	0.0	0.0	0.0
0.0	0.0	0.0	0.0	0.0	0.0	0.0	0.0
0.0	0.0	0.0	0.0	0.0	0.0	0.0	0.0
0.0	0.0	0.0	0.0	0.0	0.0	0.0	0.0
0.0	0.0	0.0	0.0	0.0	0.0	0.0	0.0
0.0	0.0	0.0	0.0	0.0	0.0	0.0	0.0
0.0	0.0	0.0	0.0	0.0	0.0	0.0	0.0
0.0	0.0	0.0	0.0	0.0	0.0	0.0	0.0
0.0	0.0	0.0	0.0	0.0	0.0	0.0	0.0
0.0	0.0	0.0	0.0	0.0	0.0	0.0	0.0
0.0	0.0	0.0	0.0	0.0	0.0	0.0	0.0
0.0	0.0	0.0	0.0	0.0	0.0	0.0	0.0
0.0	0.0	0.0	0.0	0.0	0.0	0.0	0.0
0.0	0.0	0.0	0.0	0.0	0.0	0.0	0.0
0.0	0.0	0.0	0.0	0.0	0.0	0.0	0.0
0.0	0.0	0.0	0.0	0.0	0.0	0.0	0.0
0.0	0.0	0.0	0.0	0.0	0.0	0.0	0.0
0.0	0.0	0.0	0.0	0.0	0.0	0.0	0.0

0.0	0.0	0.0	0.0	0.0	0.0	0.0	0.0
0.0	0.0	0.0	0.0	0.0	0.0	0.0	0.0
0.0	0.0	0.0	0.0	0.0	0.0	0.0	0.0
0.0	0.0	0.0	0.0	0.0	0.0	0.0	0.0
0.0	0.0	0.0	0.0	0.0	0.0	0.0	0.0
0.0	0.0	0.0	0.0	0.0	0.0	0.0	0.0
0.0	0.0	0.0	0.0	0.0	0.0	0.0	0.0
0.0	0.0	0.0	0.0	0.0	0.0	0.0	0.0
0.0	0.0	0.0	0.0	0.0	0.0	0.0	0.0
0.0	0.0	0.0	0.0	0.0	0.0	0.0	0.0
0.0	0.0	0.0	0.0	0.0	0.0	0.0	0.0
0.0	0.0	0.0	0.0	0.0	0.0	0.0	0.0
0.0	0.0	0.0	0.0	0.0	0.0	0.0	0.0
0.0	0.0	0.0	0.0	0.0	0.0	0.0	0.0
0.0	0.0	0.0	0.0	0.0	0.0	0.0	0.0
0.0	0.0	0.0	0.0	0.0	0.0	0.0	0.0
0.0	0.0	0.0	0.0	0.0	0.0	0.0	0.0
0.0	0.0	0.0	0.0	0.0	0.0	0.0	0.0
0.0	0.0	0.0	0.0	0.0	0.0	0.0	0.0
0.0	0.0	0.0	0.0	0.0	0.0	0.0	0.0
0.0	0.0	0.0	0.0	0.0	0.0	0.0	0.0
0.0	0.0	0.0	0.0	0.0	0.0	0.0	0.0
0.0	0.0	0.0	0.0	0.0	0.0	0.0	0.0
0.0	0.0	0.0	0.0	0.0	0.0	0.0	0.0
0.0	0.0	0.0	0.0	0.0	0.0	0.0	0.0
0.0	0.0	0.0	0.0	0.0	0.0	0.0	0.0
0.0	0.0	0.0	0.0	0.0	0.0	0.0	0.0
0.0	0.0	0.0	0.0	0.0	0.0	0.0	0.0
0.0	0.0	0.0	0.0	0.0	0.0	0.0	0.0
0.0	0.0	0.0	0.0	0.0	0.0	0.0	0.0
0.0	0.0	0.0	0.0	0.0	0.0	0.0	0.0
0.0	0.0	0.0	0.0	0.0	0.0	0.0	0.0
0.0	0.0	0.0	0.0	0.0	0.0	0.0	0.0
0.0	0.0	0.0	0.0	0.0	0.0	0.0	0.0
0.0	0.0	0.0	0.0	0.0	0.0	0.0	0.0
0.0	0.0	0.0	0.0	0.0	0.0	0.0	0.0
0.0	0.0	0.0	0.0	0.0	0.0	0.0	0.0
0.0	0.0	0.0	0.0	0.0	0.0	0.0	0.0
0.0	0.0	0.0	0.0	0.0	0.0	0.0	0.0
0.0	0.0	0.0	0.0	0.0	0.0	0.0	0.0
0.0	0.0	0.0	0.0	0.0	0.0	0.0	0.0
0.0	0.0	0.0	0.0	0.0	0.0	0.0	0.0
0.0	0.0	0.0	0.0	0.0	0.0	0.0	0.0
0.0	0.0	0.0	0.0	0.0	0.0	0.0	0.0
0.0	0.0	0.0	0.0	0.0	0.0	0.0	0.0
0.0	0.0	0.0	0.0	0.0	0.0	0.0	0.0
0.0	0.0	0.0	0.0	0.0	0.0	0.0	0.0
0.0	0.0	0.0	0.0	0.0	0.0	0.0	0.0

0.0	0.0	0.0	0.0	0.0	0.0	0.0	0.0
0.0	0.0	0.0	0.0	0.0	0.0	0.0	0.0
0.0	0.0	0.0	0.0	0.0	0.0	0.0	0.0
0.0	0.0	0.0	0.0	0.0	0.0	0.0	0.0
0.0	0.0	0.0	0.0	0.0	0.0	0.0	0.0
0.0	0.0	0.0	0.0	0.0	0.0	0.0	0.0
0.0	0.0	0.0	0.0	0.0	0.0	0.0	0.0
0.0	0.0	0.0	0.0	0.0	0.0	0.0	0.0
0.0	0.0	0.0	0.0	0.0	0.0	0.0	0.0
0.0	0.0	0.0	0.0	0.0	0.0	0.0	0.0
0.0	0.0	0.0	0.0	0.0	0.0	0.0	0.0
0.0	0.0	0.0	0.0	0.0	0.0	0.0	0.0
0.0	0.0	0.0	0.0	0.0	0.0	0.0	0.0
0.0	0.0	0.0	0.0	0.0	0.0	0.0	0.0
0.0	0.0	0.0	0.0	0.0	0.0	0.0	0.0
0.0	0.0	0.0	0.0	0.0	0.0	0.0	0.0
0.0	0.0	0.0	0.0	0.0	0.0	0.0	0.0
0.0	0.0	0.0	0.0	0.0	0.0	0.0	0.0
0.0	0.0	0.0	0.0	0.0	0.0	0.0	0.0
0.0	0.0	0.0	0.0	0.0	0.0	0.0	0.0
0.0	0.0	0.0	0.0	0.0	0.0	0.0	0.0
0.0	0.0	0.0	0.0	0.0	0.0	0.0	0.0
0.0	0.0	0.0	0.0	0.0	0.0	0.0	0.0
0.0	0.0	0.0	0.0	0.0	0.0	0.0	0.0
0.0	0.0	0.0	0.0	0.0	0.0	0.0	0.0
0.0	0.0	0.0	0.0	0.0	0.0	0.0	0.0
0.0	0.0	0.0	0.0	0.0	0.0	0.0	0.0
0.0	0.0	0.0	0.0	0.0	0.0	0.0	0.0
0.0	0.0	0.0	0.0	0.0	0.0	0.0	0.0
0.0	0.0	0.0	0.0	0.0	0.0	0.0	0.0
0.0	0.0	0.0	0.0	0.0	0.0	0.0	0.0
0.0	0.0	0.0	0.0	0.0	0.0	0.0	0.0
0.0	0.0	0.0	0.0	0.0	0.0	0.0	0.0
0.0	0.0	0.0	0.0	0.0	0.0	0.0	0.0
0.0	0.0	0.0	0.0	0.0	0.0	0.0	0.0
0.0	0.0	0.0	0.0	0.0	0.0	0.0	0.0
0.0	0.0	0.0	0.0	0.0	0.0	0.0	0.0
0.0	0.0	0.0	0.0	0.0	0.0	0.0	0.0
0.0	0.0	0.0	0.0	0.0	0.0	0.0	0.0
0.0	0.0	0.0	0.0	0.0	0.0	0.0	0.0
0.0	0.0	0.0	0.0	0.0	0.0	0.0	0.0
0.0	0.0	0.0	0.0	0.0	0.0	0.0	0.0
0.0	0.0	0.0	0.0	0.0	0.0	0.0	0.0
0.0	0.0	0.0	0.0	0.0	0.0	0.0	0.0
0.0	0.0	0.0	0.0	0.0	0.0	0.0	0.0
0.0	0.0	0.0	0.0	0.0	0.0	0.0	0.0
0.0	0.0	0.0	0.0	0.0	0.0	0.0	0.0

0.0	0.0	0.0	0.0	0.0	0.0	0.0	0.0
0.0	0.0	0.0	0.0	0.0	0.0	0.0	0.0
0.0	0.0	0.0	0.0	0.0	0.0	0.0	0.0
0.0	0.0	0.0	0.0	0.0	0.0	0.0	0.0
0.0	0.0	0.0	0.0	0.0	0.0	0.0	0.0
0.0	0.0	0.0	0.0	0.0	0.0	0.0	0.0
0.0	0.0	0.0	0.0	0.0	0.0	0.0	0.0
0.0	0.0	0.0	0.0	0.0	0.0	0.0	0.0
0.0	0.0	0.0	0.0	0.0	0.0	0.0	0.0
0.0	0.0	0.0	0.0	0.0	0.0	0.0	0.0
0.0	0.0	0.0	0.0	0.0	0.0	0.0	0.0
0.0	0.0	0.0	0.0	0.0	0.0	0.0	0.0
0.0	0.0	0.0	0.0	0.0	0.0	0.0	0.0
0.0	0.0	0.0	0.0	0.0	0.0	0.0	0.0
0.0	0.0	0.0	0.0	0.0	0.0	0.0	0.0
0.0	0.0	0.0	0.0	0.0	0.0	0.0	0.0
0.0	0.0	0.0	0.0	0.0	0.0	0.0	0.0
0.0	0.0	0.0	0.0	0.0	0.0	0.0	0.0
0.0	0.0	0.0	0.0	0.0	0.0	0.0	0.0
0.0	0.0	0.0	0.0	0.0	0.0	0.0	0.0
0.0	0.0	0.0	0.0	0.0	0.0	0.0	0.0
0.0	0.0	0.0	0.0	0.0	0.0	0.0	0.0
0.0	0.0	0.0	0.0	0.0	0.0	0.0	0.0
0.0	0.0	0.0	0.0	0.0	0.0	0.0	0.0
0.0	0.0	0.0	0.0	0.0	0.0	0.0	0.0
0.0	0.0	0.0	0.0	0.0	0.0	0.0	0.0
0.0	0.0	0.0	0.0	0.0	0.0	0.0	0.0
0.0	0.0	0.0	0.0	0.0	0.0	0.0	0.0
0.0	0.0	0.0	0.0	0.0	0.0	0.0	0.0
0.0	0.0	0.0	0.0	0.0	0.0	0.0	0.0
0.0	0.0	0.0	0.0	0.0	0.0	0.0	0.0
0.0	0.0	0.0	0.0	0.0	0.0	0.0	0.0
0.0	0.0	0.0	0.0	0.0	0.0	0.0	0.0
0.0	0.0	0.0	0.0	0.0	0.0	0.0	0.0
0.0	0.0	0.0	0.0	0.0	0.0	0.0	0.0
0.0	0.0	0.0	0.0	0.0	0.0	0.0	0.0
0.0	0.0	0.0	0.0	0.0	0.0	0.0	0.0
0.0	0.0	0.0	0.0	0.0	0.0	0.0	0.0
0.0	0.0	0.0	0.0	0.0	0.0	0.0	0.0
0.0	0.0	0.0	0.0	0.0	0.0	0.0	0.0
0.0	0.0	0.0	0.0	0.0	0.0	0.0	0.0
0.0	0.0	0.0	0.0	0.0	0.0	0.0	0.0
0.0	0.0	0.0	0.0	0.0	0.0	0.0	0.0
0.0	0.0	0.0	0.0	0.0	0.0	0.0	0.0
0.0	0.0	0.0	0.0	0.0	0.0	0.0	0.0
0.0	0.0	0.0	0.0	0.0	0.0	0.0	0.0
0.0	0.0	0.0	0.0	0.0	0.0	0.0	0.0

0.0	0.0	0.0	0.0	0.0	0.0	0.0	0.0
0.0	0.0	0.0	0.0	0.0	0.0	0.0	0.0
0.0	0.0	0.0	0.0	0.0	0.0	0.0	0.0
0.0	0.0	0.0	0.0	0.0	0.0	0.0	0.0
0.0	0.0	0.0	0.0	0.0	0.0	0.0	0.0
0.0	0.0	0.0	0.0	0.0	0.0	0.0	0.0
0.0	0.0	0.0	0.0	0.0	0.0	0.0	0.0
0.0	0.0	0.0	0.0	0.0	0.0	0.0	0.0
0.0	0.0	0.0	0.0	0.0	0.0	0.0	0.0
0.0	0.0	0.0	0.0	0.0	0.0	0.0	0.0
0.0	0.0	0.0	0.0	0.0	0.0	0.0	0.0
0.0	0.0	0.0	0.0	0.0	0.0	0.0	0.0
0.0	0.0	0.0	0.0	0.0	0.0	0.0	0.0
0.0	0.0	0.0	0.0	0.0	0.0	0.0	0.0
0.0	0.0	0.0	0.0	0.0	0.0	0.0	0.0
0.0	0.0	0.0	0.0	0.0	0.0	0.0	0.0
0.0	0.0	0.0	0.0	0.0	0.0	0.0	0.0
0.0	0.0	0.0	0.0	0.0	0.0	0.0	0.0
0.0	0.0	0.0	0.0	0.0	0.0	0.0	0.0
0.0	0.0	0.0	0.0	0.0	0.0	0.0	0.0
0.0	0.0	0.0	0.0	0.0	0.0	0.0	0.0
0.0	0.0	0.0	0.0	0.0	0.0	0.0	0.0
0.0	0.0	0.0	0.0	0.0	0.0	0.0	0.0
0.0	0.0	0.0	0.0	0.0	0.0	0.0	0.0
0.0	0.0	0.0	0.0	0.0	0.0	0.0	0.0
0.0	0.0	0.0	0.0	0.0	0.0	0.0	0.0
0.0	0.0	0.0	0.0	0.0	0.0	0.0	0.0
0.0	0.0	0.0	0.0	0.0	0.0	0.0	0.0
0.0	0.0	0.0	0.0	0.0	0.0	0.0	0.0
0.0	0.0	0.0	0.0	0.0	0.0	0.0	0.0
0.0	0.0	0.0	0.0	0.0	0.0	0.0	0.0
0.0	0.0	0.0	0.0	0.0	0.0	0.0	0.0
0.0	0.0	0.0	0.0	0.0	0.0	0.0	0.0
0.0	0.0	0.0	0.0	0.0	0.0	0.0	0.0
0.0	0.0	0.0	0.0	0.0	0.0	0.0	0.0
0.0	0.0	0.0	0.0	0.0	0.0	0.0	0.0
0.0	0.0	0.0	0.0	0.0	0.0	0.0	0.0
0.0	0.0	0.0	0.0	0.0	0.0	0.0	0.0
0.0	0.0	0.0	0.0	0.0	0.0	0.0	0.0
0.0	0.0	0.0	0.0	0.0	0.0	0.0	0.0
0.0	0.0	0.0	0.0	0.0	0.0	0.0	0.0
0.0	0.0	0.0	0.0	0.0	0.0	0.0	0.0
0.0	0.0	0.0	0.0	0.0	0.0	0.0	0.0
0.0	0.0	0.0	0.0	0.0	0.0	0.0	0.0
0.0	0.0	0.0	0.0	0.0	0.0	0.0	0.0
0.0	0.0	0.0	0.0	0.0	0.0	0.0	0.0
0.0	0.0	0.0	0.0	0.0	0.0	0.0	0.0
0.0	0.0	0.0	0.0	0.0	0.0	0.0	0.0

0.0	0.0	0.0	0.0	0.0	0.0	0.0	0.0
0.0	0.0	0.0	0.0	0.0	0.0	0.0	0.0
0.0	0.0	0.0	0.0	0.0	0.0	0.0	0.0
0.0	0.0	0.0	0.0	0.0	0.0	0.0	0.0
0.0	0.0	0.0	0.0	0.0	0.0	0.0	0.0
0.0	0.0	0.0	0.0	0.0	0.0	0.0	0.0
0.0	0.0	0.0	0.0	0.0	0.0	0.0	0.0
0.0	0.0	0.0	0.0	0.0	0.0	0.0	0.0
0.0	0.0	0.0	0.0	0.0	0.0	0.0	0.0
0.0	0.0	0.0	0.0	0.0	0.0	0.0	0.0
0.0	0.0	0.0	0.0	0.0	0.0	0.0	0.0
0.0	0.0	0.0	0.0	0.0	0.0	0.0	0.0
0.0	0.0	0.0	0.0	0.0	0.0	0.0	0.0
0.0	0.0	0.0	0.0	0.0	0.0	0.0	0.0
0.0	0.0	0.0	0.0	0.0	0.0	0.0	0.0
0.0	0.0	0.0	0.0	0.0	0.0	0.0	0.0
0.0	0.0	0.0	0.0	0.0	0.0	0.0	0.0
0.0	0.0	0.0	0.0	0.0	0.0	0.0	0.0
0.0	0.0	0.0	0.0	0.0	0.0	0.0	0.0
0.0	0.0	0.0	0.0	0.0	0.0	0.0	0.0
0.0	0.0	0.0	0.0	0.0	0.0	0.0	0.0
0.0	0.0	0.0	0.0	0.0	0.0	0.0	0.0
0.0	0.0	0.0	0.0	0.0	0.0	0.0	0.0
0.0	0.0	0.0	0.0	0.0	0.0	0.0	0.0
0.0	0.0	0.0	0.0	0.0	0.0	0.0	0.0
0.0	0.0	0.0	0.0	0.0	0.0	0.0	0.0
0.0	0.0	0.0	0.0	0.0	0.0	0.0	0.0
0.0	0.0	0.0	0.0	0.0	0.0	0.0	0.0
0.0	0.0	0.0	0.0	0.0	0.0	0.0	0.0
0.0	0.0	0.0	0.0	0.0	0.0	0.0	0.0
0.0	0.0	0.0	0.0	0.0	0.0	0.0	0.0
0.0	0.0	0.0	0.0	0.0	0.0	0.0	0.0
0.0	0.0	0.0	0.0	0.0	0.0	0.0	0.0
0.0	0.0	0.0	0.0	0.0	0.0	0.0	0.0
0.0	0.0	0.0	0.0	0.0	0.0	0.0	0.0
0.0	0.0	0.0	0.0	0.0	0.0	0.0	0.0
0.0	0.0	0.0	0.0	0.0	0.0	0.0	0.0
0.0	0.0	0.0	0.0	0.0	0.0	0.0	0.0
0.0	0.0	0.0	0.0	0.0	0.0	0.0	0.0
0.0	0.0	0.0	0.0	0.0	0.0	0.0	0.0
0.0	0.0	0.0	0.0	0.0	0.0	0.0	0.0
0.0	0.0	0.0	0.0	0.0	0.0	0.0	0.0
0.0	0.0	0.0	0.0	0.0	0.0	0.0	0.0
0.0	0.0	0.0	0.0	0.0	0.0	0.0	0.0
0.0	0.0	0.0	0.0	0.0	0.0	0.0	0.0
0.0	0.0	0.0	0.0	0.0	0.0	0.0	0.0
0.0	0.0	0.0	0.0	0.0	0.0	0.0	0.0

0.0	0.0	0.0	0.0	0.0	0.0	0.0	0.0
0.0	0.0	0.0	0.0	0.0	0.0	0.0	0.0
0.0	0.0	0.0	0.0	0.0	0.0	0.0	0.0
0.0	0.0	0.0	0.0	0.0	0.0	0.0	0.0
0.0	0.0	0.0	0.0	0.0	0.0	0.0	0.0
0.0	0.0	0.0	0.0	0.0	0.0	0.0	0.0
0.0	0.0	0.0	0.0	0.0	0.0	0.0	0.0
0.0	0.0	0.0	0.0	0.0	0.0	0.0	0.0
0.0	0.0	0.0	0.0	0.0	0.0	0.0	0.0
0.0	0.0	0.0	0.0	0.0	0.0	0.0	0.0
0.0	0.0	0.0	0.0	0.0	0.0	0.0	0.0
0.0	0.0	0.0	0.0	0.0	0.0	0.0	0.0
0.0	0.0	0.0	0.0	0.0	0.0	0.0	0.0
0.0	0.0	0.0	0.0	0.0	0.0	0.0	0.0
0.0	0.0	0.0	0.0	0.0	0.0	0.0	0.0
0.0	0.0	0.0	0.0	0.0	0.0	0.0	0.0
0.0	0.0	0.0	0.0	0.0	0.0	0.0	0.0
0.0	0.0	0.0	0.0	0.0	0.0	0.0	0.0
0.0	0.0	0.0	0.0	0.0	0.0	0.0	0.0
0.0	0.0	0.0	0.0	0.0	0.0	0.0	0.0
0.0	0.0	0.0	0.0	0.0	0.0	0.0	0.0
0.0	0.0	0.0	0.0	0.0	0.0	0.0	0.0
0.0	0.0	0.0	0.0	0.0	0.0	0.0	0.0
0.0	0.0	0.0	0.0	0.0	0.0	0.0	0.0
0.0	0.0	0.0	0.0	0.0	0.0	0.0	0.0
0.0	0.0	0.0	0.0	0.0	0.0	0.0	0.0
0.0	0.0	0.0	0.0	0.0	0.0	0.0	0.0
0.0	0.0	0.0	0.0	0.0	0.0	0.0	0.0
0.0	0.0	0.0	0.0	0.0	0.0	0.0	0.0
0.0	0.0	0.0	0.0	0.0	0.0	0.0	0.0
0.0	0.0	0.0	0.0	0.0	0.0	0.0	0.0
0.0	0.0	0.0	0.0	0.0	0.0	0.0	0.0
0.0	0.0	0.0	0.0	0.0	0.0	0.0	0.0
0.0	0.0	0.0	0.0	0.0	0.0	0.0	0.0
0.0	0.0	0.0	0.0	0.0	0.0	0.0	0.0
0.0	0.0	0.0	0.0	0.0	0.0	0.0	0.0
0.0	0.0	0.0	0.0	0.0	0.0	0.0	0.0
0.0	0.0	0.0	0.0	0.0	0.0	0.0	0.0
0.0	0.0	0.0	0.0	0.0	0.0	0.0	0.0
0.0	0.0	0.0	0.0	0.0	0.0	0.0	0.0
0.0	0.0	0.0	0.0	0.0	0.0	0.0	0.0
0.0	0.0	0.0	0.0	0.0	0.0	0.0	0.0
0.0	0.0	0.0	0.0	0.0	0.0	0.0	0.0
0.0	0.0	0.0	0.0	0.0	0.0	0.0	0.0
0.0	0.0	0.0	0.0	0.0	0.0	0.0	0.0
0.0	0.0	0.0	0.0	0.0	0.0	0.0	0.0
0.0	0.0	0.0	0.0	0.0	0.0	0.0	0.0

0.0	0.0	0.0	0.0	0.0	0.0	0.0	0.0
0.0	0.0	0.0	0.0	0.0	0.0	0.0	0.0
0.0	0.0	0.0	0.0	0.0	0.0	0.0	0.0
0.0	0.0	0.0	0.0	0.0	0.0	0.0	0.0
0.0	0.0	0.0	0.0	0.0	0.0	0.0	0.0
0.0	0.0	0.0	0.0	0.0	0.0	0.0	0.0
0.0	0.0	0.0	0.0	0.0	0.0	0.0	0.0
0.0	0.0	0.0	0.0	0.0	0.0	0.0	0.0
0.0	0.0	0.0	0.0	0.0	0.0	0.0	0.0
0.0	0.0	0.0	0.0	0.0	0.0	0.0	0.0
0.0	0.0	0.0	0.0	0.0	0.0	0.0	0.0
0.0	0.0	0.0	0.0	0.0	0.0	0.0	0.0
0.0	0.0	0.0	0.0	0.0	0.0	0.0	0.0
0.0	0.0	0.0	0.0	0.0	0.0	0.0	0.0
0.0	0.0	0.0	0.0	0.0	0.0	0.0	0.0
0.0	0.0	0.0	0.0	0.0	0.0	0.0	0.0
0.0	0.0	0.0	0.0	0.0	0.0	0.0	0.0
0.0	0.0	0.0	0.0	0.0	0.0	0.0	0.0
0.0	0.0	0.0	0.0	0.0	0.0	0.0	0.0
0.0	0.0	0.0	0.0	0.0	0.0	0.0	0.0
0.0	0.0	0.0	0.0	0.0	0.0	0.0	0.0
0.0	0.0	0.0	0.0	0.0	0.0	0.0	0.0
0.0	0.0	0.0	0.0	0.0	0.0	0.0	0.0
0.0	0.0	0.0	0.0	0.0	0.0	0.0	0.0
0.0	0.0	0.0	0.0	0.0	0.0	0.0	0.0
0.0	0.0	0.0	0.0	0.0	0.0	0.0	0.0
0.0	0.0	0.0	0.0	0.0	0.0	0.0	0.0
0.0	0.0	0.0	0.0	0.0	0.0	0.0	0.0
0.0	0.0	0.0	0.0	0.0	0.0	0.0	0.0
0.0	0.0	0.0	0.0	0.0	0.0	0.0	0.0
0.0	0.0	0.0	0.0	0.0	0.0	0.0	0.0
0.0	0.0	0.0	0.0	0.0	0.0	0.0	0.0
0.0	0.0	0.0	0.0	0.0	0.0	0.0	0.0
0.0	0.0	0.0	0.0	0.0	0.0	0.0	0.0
0.0	0.0	0.0	0.0	0.0	0.0	0.0	0.0
0.0	0.0	0.0	0.0	0.0	0.0	0.0	0.0
0.0	0.0	0.0	0.0	0.0	0.0	0.0	0.0
0.0	0.0	0.0	0.0	0.0	0.0	0.0	0.0
0.0	0.0	0.0	0.0	0.0	0.0	0.0	0.0
0.0	0.0	0.0	0.0	0.0	0.0	0.0	0.0
0.0	0.0	0.0	0.0	0.0	0.0	0.0	0.0
0.0	0.0	0.0	0.0	0.0	0.0	0.0	0.0
0.0	0.0	0.0	0.0	0.0	0.0	0.0	0.0
0.0	0.0	0.0	0.0	0.0	0.0	0.0	0.0
0.0	0.0	0.0	0.0	0.0	0.0	0.0	0.0
0.0	0.0	0.0	0.0	0.0	0.0	0.0	0.0
0.0	0.0	0.0	0.0	0.0	0.0	0.0	0.0

0.0	0.0	0.0	0.0	0.0	0.0	0.0	0.0
0.0	0.0	0.0	0.0	0.0	0.0	0.0	0.0
0.0	0.0	0.0	0.0	0.0	0.0	0.0	0.0
0.0	0.0	0.0	0.0	0.0	0.0	0.0	0.0
0.0	0.0	0.0	0.0	0.0	0.0	0.0	0.0
0.0	0.0	0.0	0.0	0.0	0.0	0.0	0.0
0.0	0.0	0.0	0.0	0.0	0.0	0.0	0.0
0.0	0.0	0.0	0.0	0.0	0.0	0.0	0.0
0.0	0.0	0.0	0.0	0.0	0.0	0.0	0.0
0.0	0.0	0.0	0.0	0.0	0.0	0.0	0.0
0.0	0.0	0.0	0.0	0.0	0.0	0.0	0.0
0.0	0.0	0.0	0.0	0.0	0.0	0.0	0.0
0.0	0.0	0.0	0.0	0.0	0.0	0.0	0.0
0.0	0.0	0.0	0.0	0.0	0.0	0.0	0.0
0.0	0.0	0.0	0.0	0.0	0.0	0.0	0.0
0.0	0.0	0.0	0.0	0.0	0.0	0.0	0.0
0.0	0.0	0.0	0.0	0.0	0.0	0.0	0.0
0.0	0.0	0.0	0.0	0.0	0.0	0.0	0.0
0.0	0.0	0.0	0.0	0.0	0.0	0.0	0.0
0.0	0.0	0.0	0.0	0.0	0.0	0.0	0.0
0.0	0.0	0.0	0.0	0.0	0.0	0.0	0.0
0.0	0.0	0.0	0.0	0.0	0.0	0.0	0.0
0.0	0.0	0.0	0.0	0.0	0.0	0.0	0.0
0.0	0.0	0.0	0.0	0.0	0.0	0.0	0.0
0.0	0.0	0.0	0.0	0.0	0.0	0.0	0.0
0.0	0.0	0.0	0.0	0.0	0.0	0.0	0.0
0.0	0.0	0.0	0.0	0.0	0.0	0.0	0.0
0.0	0.0	0.0	0.0	0.0	0.0	0.0	0.0
0.0	0.0	0.0	0.0	0.0	0.0	0.0	0.0
0.0	0.0	0.0	0.0	0.0	0.0	0.0	0.0
0.0	0.0	0.0	0.0	0.0	0.0	0.0	0.0
0.0	0.0	0.0	0.0	0.0	0.0	0.0	0.0
0.0	0.0	0.0	0.0	0.0	0.0	0.0	0.0
0.0	0.0	0.0	0.0	0.0	0.0	0.0	0.0
0.0	0.0	0.0	0.0	0.0	0.0	0.0	0.0
0.0	0.0	0.0	0.0	0.0	0.0	0.0	0.0
0.0	0.0	0.0	0.0	0.0	0.0	0.0	0.0
0.0	0.0	0.0	0.0	0.0	0.0	0.0	0.0
0.0	0.0	0.0	0.0	0.0	0.0	0.0	0.0
0.0	0.0	0.0	0.0	0.0	0.0	0.0	0.0
0.0	0.0	0.0	0.0	0.0	0.0	0.0	0.0
0.0	0.0	0.0	0.0	0.0	0.0	0.0	0.0
0.0	0.0	0.0	0.0	0.0	0.0	0.0	0.0
0.0	0.0	0.0	0.0	0.0	0.0	0.0	0.0
0.0	0.0	0.0	0.0	0.0	0.0	0.0	0.0
0.0	0.0	0.0	0.0	0.0	0.0	0.0	0.0
0.0	0.0	0.0	0.0	0.0	0.0	0.0	0.0
0.0	0.0	0.0	0.0	0.0	0.0	0.0	0.0

0.0	0.0	0.0	0.0	0.0	0.0	0.0	0.0
0.0	0.0	0.0	0.0	0.0	0.0	0.0	0.0
0.0	0.0	0.0	0.0	0.0	0.0	0.0	0.0
0.0	0.0	0.0	0.0	0.0	0.0	0.0	0.0
0.0	0.0	0.0	0.0	0.0	0.0	0.0	0.0
0.0	0.0	0.0	0.0	0.0	0.0	0.0	0.0
0.0	0.0	0.0	0.0	0.0	0.0	0.0	0.0
0.0	0.0	0.0	0.0	0.0	0.0	0.0	0.0
0.0	0.0	0.0	0.0	0.0	0.0	0.0	0.0
0.0	0.0	0.0	0.0	0.0	0.0	0.0	0.0
0.0	0.0	0.0	0.0	0.0	0.0	0.0	0.0
0.0	0.0	0.0	0.0	0.0	0.0	0.0	0.0
0.0	0.0	0.0	0.0	0.0	0.0	0.0	0.0
0.0	0.0	0.0	0.0	0.0	0.0	0.0	0.0
0.0	0.0	0.0	0.0	0.0	0.0	0.0	0.0
0.0	0.0	0.0	0.0	0.0	0.0	0.0	0.0
0.0	0.0	0.0	0.0	0.0	0.0	0.0	0.0
0.0	0.0	0.0	0.0	0.0	0.0	0.0	0.0
0.0	0.0	0.0	0.0	0.0	0.0	0.0	0.0
0.0	0.0	0.0	0.0	0.0	0.0	0.0	0.0
0.0	0.0	0.0	0.0	0.0	0.0	0.0	0.0
0.0	0.0	0.0	0.0	0.0	0.0	0.0	0.0
0.0	0.0	0.0	0.0	0.0	0.0	0.0	0.0
0.0	0.0	0.0	0.0	0.0	0.0	0.0	0.0
0.0	0.0	0.0	0.0	0.0	0.0	0.0	0.0
0.0	0.0	0.0	0.0	0.0	0.0	0.0	0.0
0.0	0.0	0.0	0.0	0.0	0.0	0.0	0.0
0.0	0.0	0.0	0.0	0.0	0.0	0.0	0.0
0.0	0.0	0.0	0.0	0.0	0.0	0.0	0.0
0.0	0.0	0.0	0.0	0.0	0.0	0.0	0.0
0.0	0.0	0.0	0.0	0.0	0.0	0.0	0.0
0.0	0.0	0.0	0.0	0.0	0.0	0.0	0.0
0.0	0.0	0.0	0.0	0.0	0.0	0.0	0.0
0.0	0.0	0.0	0.0	0.0	0.0	0.0	0.0
0.0	0.0	0.0	0.0	0.0	0.0	0.0	0.0
0.0	0.0	0.0	0.0	0.0	0.0	0.0	0.0
0.0	0.0	0.0	0.0	0.0	0.0	0.0	0.0
0.0	0.0	0.0	0.0	0.0	0.0	0.0	0.0
0.0	0.0	0.0	0.0	0.0	0.0	0.0	0.0
0.0	0.0	0.0	0.0	0.0	0.0	0.0	0.0
0.0	0.0	0.0	0.0	0.0	0.0	0.0	0.0
0.0	0.0	0.0	0.0	0.0	0.0	0.0	0.0
0.0	0.0	0.0	0.0	0.0	0.0	0.0	0.0
0.0	0.0	0.0	0.0	0.0	0.0	0.0	0.0
0.0	0.0	0.0	0.0	0.0	0.0	0.0	0.0
0.0	0.0	0.0	0.0	0.0	0.0	0.0	0.0
0.0	0.0	0.0	0.0	0.0	0.0	0.0	0.0
0.0	0.0	0.0	0.0	0.0	0.0	0.0	0.0

0.0	0.0	0.0	0.0	0.0	0.0	0.0	0.0
0.0	0.0	0.0	0.0	0.0	0.0	0.0	0.0
0.0	0.0	0.0	0.0	0.0	0.0	0.0	0.0
0.0	0.0	0.0	0.0	0.0	0.0	0.0	0.0
0.0	0.0	0.0	0.0	0.0	0.0	0.0	0.0
0.0	0.0	0.0	0.0	0.0	0.0	0.0	0.0
0.0	0.0	0.0	0.0	0.0	0.0	0.0	0.0
0.0	0.0	0.0	0.0	0.0	0.0	0.0	0.0
0.0	0.0	0.0	0.0	0.0	0.0	0.0	0.0
0.0	0.0	0.0	0.0	0.0	0.0	0.0	0.0
0.0	0.0	0.0	0.0	0.0	0.0	0.0	0.0
0.0	0.0	0.0	0.0	0.0	0.0	0.0	0.0
0.0	0.0	0.0	0.0	0.0	0.0	0.0	0.0
0.0	0.0	0.0	0.0	0.0	0.0	0.0	0.0
0.0	0.0	0.0	0.0	0.0	0.0	0.0	0.0
0.0	0.0	0.0	0.0	0.0	0.0	0.0	0.0
0.0	0.0	0.0	0.0	0.0	0.0	0.0	0.0
0.0	0.0	0.0	0.0	0.0	0.0	0.0	0.0
0.0	0.0	0.0	0.0	0.0	0.0	0.0	0.0
0.0	0.0	0.0	0.0	0.0	0.0	0.0	0.0
0.0	0.0	0.0	0.0	0.0	0.0	0.0	0.0
0.0	0.0	0.0	0.0	0.0	0.0	0.0	0.0
0.0	0.0	0.0	0.0	0.0	0.0	0.0	0.0
0.0	0.0	0.0	0.0	0.0	0.0	0.0	0.0
0.0	0.0	0.0	0.0	0.0	0.0	0.0	0.0
0.0	0.0	0.0	0.0	0.0	0.0	0.0	0.0
0.0	0.0	0.0	0.0	0.0	0.0	0.0	0.0
0.0	0.0	0.0	0.0	0.0	0.0	0.0	0.0
0.0	0.0	0.0	0.0	0.0	0.0	0.0	0.0
0.0	0.0	0.0	0.0	0.0	0.0	0.0	0.0
0.0	0.0	0.0	0.0	0.0	0.0	0.0	0.0
0.0	0.0	0.0	0.0	0.0	0.0	0.0	0.0
0.0	0.0	0.0	0.0	0.0	0.0	0.0	0.0
0.0	0.0	0.0	0.0	0.0	0.0	0.0	0.0
0.0	0.0	0.0	0.0	0.0	0.0	0.0	0.0
0.0	0.0	0.0	0.0	0.0	0.0	0.0	0.0
0.0	0.0	0.0	0.0	0.0	0.0	0.0	0.0
0.0	0.0	0.0	0.0	0.0	0.0	0.0	0.0
0.0	0.0	0.0	0.0	0.0	0.0	0.0	0.0
0.0	0.0	0.0	0.0	0.0	0.0	0.0	0.0
0.0	0.0	0.0	0.0	0.0	0.0	0.0	0.0
0.0	0.0	0.0	0.0	0.0	0.0	0.0	0.0
0.0	0.0	0.0	0.0	0.0	0.0	0.0	0.0
0.0	0.0	0.0	0.0	0.0	0.0	0.0	0.0
0.0	0.0	0.0	0.0	0.0	0.0	0.0	0.0
0.0	0.0	0.0	0.0	0.0	0.0	0.0	0.0
0.0	0.0	0.0	0.0	0.0	0.0	0.0	0.0
0.0	0.0	0.0	0.0	0.0	0.0	0.0	0.0

0.0	0.0	0.0	0.0	0.0	0.0	0.0	0.0
0.0	0.0	0.0	0.0	0.0	0.0	0.0	0.0
0.0	0.0	0.0	0.0	0.0	0.0	0.0	0.0
0.0	0.0	0.0	0.0	0.0	0.0	0.0	0.0
0.0	0.0	0.0	0.0	0.0	0.0	0.0	0.0
0.0	0.0	0.0	0.0	0.0	0.0	0.0	0.0
0.0	0.0	0.0	0.0	0.0	0.0	0.0	0.0
0.0	0.0	0.0	0.0	0.0	0.0	0.0	0.0
0.0	0.0	0.0	0.0	0.0	0.0	0.0	0.0
0.0	0.0	0.0	0.0	0.0	0.0	0.0	0.0
0.0	0.0	0.0	0.0	0.0	0.0	0.0	0.0
0.0	0.0	0.0	0.0	0.0	0.0	0.0	0.0
0.0	0.0	0.0	0.0	0.0	0.0	0.0	0.0
0.0	0.0	0.0	0.0	0.0	0.0	0.0	0.0
0.0	0.0	0.0	0.0	0.0	0.0	0.0	0.0
0.0	0.0	0.0	0.0	0.0	0.0	0.0	0.0
0.0	0.0	0.0	0.0	0.0	0.0	0.0	0.0
0.0	0.0	0.0	0.0	0.0	0.0	0.0	0.0
0.0	0.0	0.0	0.0	0.0	0.0	0.0	0.0
0.0	0.0	0.0	0.0	0.0	0.0	0.0	0.0
0.0	0.0	0.0	0.0	0.0	0.0	0.0	0.0
0.0	0.0	0.0	0.0	0.0	0.0	0.0	0.0
0.0	0.0	0.0	0.0	0.0	0.0	0.0	0.0
0.0	0.0	0.0	0.0	0.0	0.0	0.0	0.0
0.0	0.0	0.0	0.0	0.0	0.0	0.0	0.0
0.0	0.0	0.0	0.0	0.0	0.0	0.0	0.0
0.0	0.0	0.0	0.0	0.0	0.0	0.0	0.0
0.0	0.0	0.0	0.0	0.0	0.0	0.0	0.0
0.0	0.0	0.0	0.0	0.0	0.0	0.0	0.0
0.0	0.0	0.0	0.0	0.0	0.0	0.0	0.0
0.0	0.0	0.0	0.0	0.0	0.0	0.0	0.0
0.0	0.0	0.0	0.0	0.0	0.0	0.0	0.0
0.0	0.0	0.0	0.0	0.0	0.0	0.0	0.0
0.0	0.0	0.0	0.0	0.0	0.0	0.0	0.0
0.0	0.0	0.0	0.0	0.0	0.0	0.0	0.0
0.0	0.0	0.0	0.0	0.0	0.0	0.0	0.0
0.0	0.0	0.0	0.0	0.0	0.0	0.0	0.0
0.0	0.0	0.0	0.0	0.0	0.0	0.0	0.0
0.0	0.0	0.0	0.0	0.0	0.0	0.0	0.0
0.0	0.0	0.0	0.0	0.0	0.0	0.0	0.0
0.0	0.0	0.0	0.0	0.0	0.0	0.0	0.0
0.0	0.0	0.0	0.0	0.0	0.0	0.0	0.0
0.0	0.0	0.0	0.0	0.0	0.0	0.0	0.0
0.0	0.0	0.0	0.0	0.0	0.0	0.0	0.0
0.0	0.0	0.0	0.0	0.0	0.0	0.0	0.0
0.0	0.0	0.0	0.0	0.0	0.0	0.0	0.0
0.0	0.0	0.0	0.0	0.0	0.0	0.0	0.0

0.0	0.0	0.0	0.0	0.0	0.0	0.0	0.0
0.0	0.0	0.0	0.0	0.0	0.0	0.0	0.0
0.0	0.0	0.0	0.0	0.0	0.0	0.0	0.0
0.0	0.0	0.0	0.0	0.0	0.0	0.0	0.0
0.0	0.0	0.0	0.0	0.0	0.0	0.0	0.0
0.0	0.0	0.0	0.0	0.0	0.0	0.0	0.0
0.0	0.0	0.0	0.0	0.0	0.0	0.0	0.0
0.0	0.0	0.0	0.0	0.0	0.0	0.0	0.0
0.0	0.0	0.0	0.0	0.0	0.0	0.0	0.0
0.0	0.0	0.0	0.0	0.0	0.0	0.0	0.0
0.0	0.0	0.0	0.0	0.0	0.0	0.0	0.0
0.0	0.0	0.0	0.0	0.0	0.0	0.0	0.0
0.0	0.0	0.0	0.0	0.0	0.0	0.0	0.0
0.0	0.0	0.0	0.0	0.0	0.0	0.0	0.0
0.0	0.0	0.0	0.0	0.0	0.0	0.0	0.0
0.0	0.0	0.0	0.0	0.0	0.0	0.0	0.0
0.0	0.0	0.0	0.0	0.0	0.0	0.0	0.0
0.0	0.0	0.0	0.0	0.0	0.0	0.0	0.0
0.0	0.0	0.0	0.0	0.0	0.0	0.0	0.0
0.0	0.0	0.0	0.0	0.0	0.0	0.0	0.0
0.0	0.0	0.0	0.0	0.0	0.0	0.0	0.0
0.0	0.0	0.0	0.0	0.0	0.0	0.0	0.0
0.0	0.0	0.0	0.0	0.0	0.0	0.0	0.0
0.0	0.0	0.0	0.0	0.0	0.0	0.0	0.0
0.0	0.0	0.0	0.0	0.0	0.0	0.0	0.0
0.0	0.0	0.0	0.0	0.0	0.0	0.0	0.0
0.0	0.0	0.0	0.0	0.0	0.0	0.0	0.0
0.0	0.0	0.0	0.0	0.0	0.0	0.0	0.0
0.0	0.0	0.0	0.0	0.0	0.0	0.0	0.0
0.0	0.0	0.0	0.0	0.0	0.0	0.0	0.0
0.0	0.0	0.0	0.0	0.0	0.0	0.0	0.0
0.0	0.0	0.0	0.0	0.0	0.0	0.0	0.0
0.0	0.0	0.0	0.0	0.0	0.0	0.0	0.0
0.0	0.0	0.0	0.0	0.0	0.0	0.0	0.0
0.0	0.0	0.0	0.0	0.0	0.0	0.0	0.0
0.0	0.0	0.0	0.0	0.0	0.0	0.0	0.0
0.0	0.0	0.0	0.0	0.0	0.0	0.0	0.0
0.0	0.0	0.0	0.0	0.0	0.0	0.0	0.0
0.0	0.0	0.0	0.0	0.0	0.0	0.0	0.0
0.0	0.0	0.0	0.0	0.0	0.0	0.0	0.0
0.0	0.0	0.0	0.0	0.0	0.0	0.0	0.0
0.0	0.0	0.0	0.0	0.0	0.0	0.0	0.0
0.0	0.0	0.0	0.0	0.0	0.0	0.0	0.0
0.0	0.0	0.0	0.0	0.0	0.0	0.0	0.0
0.0	0.0	0.0	0.0	0.0	0.0	0.0	0.0
0.0	0.0	0.0	0.0	0.0	0.0	0.0	0.0
0.0	0.0	0.0	0.0	0.0	0.0	0.0	0.0

0.0	0.0	0.0	0.0	0.0	0.0	0.0	0.0
0.0	0.0	0.0	0.0	0.0	0.0	0.0	0.0
0.0	0.0	0.0	0.0	0.0	0.0	0.0	0.0
0.0	0.0	0.0	0.0	0.0	0.0	0.0	0.0
0.0	0.0	0.0	0.0	0.0	0.0	0.0	0.0
0.0	0.0	0.0	0.0	0.0	0.0	0.0	0.0
0.0	0.0	0.0	0.0	0.0	0.0	0.0	0.0
0.0	0.0	0.0	0.0	0.0	0.0	0.0	0.0
0.0	0.0	0.0	0.0	0.0	0.0	0.0	0.0
0.0	0.0	0.0	0.0	0.0	0.0	0.0	0.0
0.0	0.0	0.0	0.0	0.0	0.0	0.0	0.0
0.0	0.0	0.0	0.0	0.0	0.0	0.0	0.0
0.0	0.0	0.0	0.0	0.0	0.0	0.0	0.0
0.0	0.0	0.0	0.0	0.0	0.0	0.0	0.0
0.0	0.0	0.0	0.0	0.0	0.0	0.0	0.0
0.0	0.0	0.0	0.0	0.0	0.0	0.0	0.0
0.0	0.0	0.0	0.0	0.0	0.0	0.0	0.0
0.0	0.0	0.0	0.0	0.0	0.0	0.0	0.0
0.0	0.0	0.0	0.0	0.0	0.0	0.0	0.0
0.0	0.0	0.0	0.0	0.0	0.0	0.0	0.0
0.0	0.0	0.0	0.0	0.0	0.0	0.0	0.0
0.0	0.0	0.0	0.0	0.0	0.0	0.0	0.0
0.0	0.0	0.0	0.0	0.0	0.0	0.0	0.0
0.0	0.0	0.0	0.0	0.0	0.0	0.0	0.0
0.0	0.0	0.0	0.0	0.0	0.0	0.0	0.0
0.0	0.0	0.0	0.0	0.0	0.0	0.0	0.0
0.0	0.0	0.0	0.0	0.0	0.0	0.0	0.0
0.0	0.0	0.0	0.0	0.0	0.0	0.0	0.0
0.0	0.0	0.0	0.0	0.0	0.0	0.0	0.0
0.0	0.0	0.0	0.0	0.0	0.0	0.0	0.0
0.0	0.0	0.0	0.0	0.0	0.0	0.0	0.0
0.0	0.0	0.0	0.0	0.0	0.0	0.0	0.0
0.0	0.0	0.0	0.0	0.0	0.0	0.0	0.0
0.0	0.0	0.0	0.0	0.0	0.0	0.0	0.0
0.0	0.0	0.0	0.0	0.0	0.0	0.0	0.0
0.0	0.0	0.0	0.0	0.0	0.0	0.0	0.0
0.0	0.0	0.0	0.0	0.0	0.0	0.0	0.0
0.0	0.0	0.0	0.0	0.0	0.0	0.0	0.0
0.0	0.0	0.0	0.0	0.0	0.0	0.0	0.0
0.0	0.0	0.0	0.0	0.0	0.0	0.0	0.0
0.0	0.0	0.0	0.0	0.0	0.0	0.0	0.0
0.0	0.0	0.0	0.0	0.0	0.0	0.0	0.0
0.0	0.0	0.0	0.0	0.0	0.0	0.0	0.0
0.0	0.0	0.0	0.0	0.0	0.0	0.0	0.0
0.0	0.0	0.0	0.0	0.0	0.0	0.0	0.0
0.0	0.0	0.0	0.0	0.0	0.0	0.0	0.0
0.0	0.0	0.0	0.0	0.0	0.0	0.0	0.0
0.0	0.0	0.0	0.0	0.0	0.0	0.0	0.0

0.0	0.0	0.0	0.0	0.0	0.0	0.0	0.0
0.0	0.0	0.0	0.0	0.0	0.0	0.0	0.0
0.0	0.0	0.0	0.0	0.0	0.0	0.0	0.0
0.0	0.0	0.0	0.0	0.0	0.0	0.0	0.0
0.0	0.0	0.0	0.0	0.0	0.0	0.0	0.0
0.0	0.0	0.0	0.0	0.0	0.0	0.0	0.0
0.0	0.0	0.0	0.0	0.0	0.0	0.0	0.0
0.0	0.0	0.0	0.0	0.0	0.0	0.0	0.0
0.0	0.0	0.0	0.0	0.0	0.0	0.0	0.0
0.0	0.0	0.0	0.0	0.0	0.0	0.0	0.0
0.0	0.0	0.0	0.0	0.0	0.0	0.0	0.0
0.0	0.0	0.0	0.0	0.0	0.0	0.0	0.0
0.0	0.0	0.0	0.0	0.0	0.0	0.0	0.0
0.0	0.0	0.0	0.0	0.0	0.0	0.0	0.0
0.0	0.0	0.0	0.0	0.0	0.0	0.0	0.0
0.0	0.0	0.0	0.0	0.0	0.0	0.0	0.0
0.0	0.0	0.0	0.0	0.0	0.0	0.0	0.0
0.0	0.0	0.0	0.0	0.0	0.0	0.0	0.0
0.0	0.0	0.0	0.0	0.0	0.0	0.0	0.0
0.0	0.0	0.0	0.0	0.0	0.0	0.0	0.0
0.0	0.0	0.0	0.0	0.0	0.0	0.0	0.0
0.0	0.0	0.0	0.0	0.0	0.0	0.0	0.0
0.0	0.0	0.0	0.0	0.0	0.0	0.0	0.0
0.0	0.0	0.0	0.0	0.0	0.0	0.0	0.0
0.0	0.0	0.0	0.0	0.0	0.0	0.0	0.0
0.0	0.0	0.0	0.0	0.0	0.0	0.0	0.0
0.0	0.0	0.0	0.0	0.0	0.0	0.0	0.0
0.0	0.0	0.0	0.0	0.0	0.0	0.0	0.0
0.0	0.0	0.0	0.0	0.0	0.0	0.0	0.0
0.0	0.0	0.0	0.0	0.0	0.0	0.0	0.0
0.0	0.0	0.0	0.0	0.0	0.0	0.0	0.0
0.0	0.0	0.0	0.0	0.0	0.0	0.0	0.0
0.0	0.0	0.0	0.0	0.0	0.0	0.0	0.0
0.0	0.0	0.0	0.0	0.0	0.0	0.0	0.0
0.0	0.0	0.0	0.0	0.0	0.0	0.0	0.0
0.0	0.0	0.0	0.0	0.0	0.0	0.0	0.0
0.0	0.0	0.0	0.0	0.0	0.0	0.0	0.0
0.0	0.0	0.0	0.0	0.0	0.0	0.0	0.0
0.0	0.0	0.0	0.0	0.0	0.0	0.0	0.0
0.0	0.0	0.0	0.0	0.0	0.0	0.0	0.0
0.0	0.0	0.0	0.0	0.0	0.0	0.0	0.0
0.0	0.0	0.0	0.0	0.0	0.0	0.0	0.0
0.0	0.0	0.0	0.0	0.0	0.0	0.0	0.0
0.0	0.0	0.0	0.0	0.0	0.0	0.0	0.0
0.0	0.0	0.0	0.0	0.0	0.0	0.0	0.0
0.0	0.0	0.0	0.0	0.0	0.0	0.0	0.0
0.0	0.0	0.0	0.0	0.0	0.0	0.0	0.0

0.0	0.0	0.0	0.0	0.0	0.0	0.0	0.0
0.0	0.0	0.0	0.0	0.0	0.0	0.0	0.0
0.0	0.0	0.0	0.0	0.0	0.0	0.0	0.0
0.0	0.0	0.0	0.0	0.0	0.0	0.0	0.0
0.0	0.0	0.0	0.0	0.0	0.0	0.0	0.0
0.0	0.0	0.0	0.0	0.0	0.0	0.0	0.0
0.0	0.0	0.0	0.0	0.0	0.0	0.0	0.0
0.0	0.0	0.0	0.0	0.0	0.0	0.0	0.0
0.0	0.0	0.0	0.0	0.0	0.0	0.0	0.0
0.0	0.0	0.0	0.0	0.0	0.0	0.0	0.0
0.0	0.0	0.0	0.0	0.0	0.0	0.0	0.0
0.0	0.0	0.0	0.0	0.0	0.0	0.0	0.0
0.0	0.0	0.0	0.0	0.0	0.0	0.0	0.0
0.0	0.0	0.0	0.0	0.0	0.0	0.0	0.0
0.0	0.0	0.0	0.0	0.0	0.0	0.0	0.0
0.0	0.0	0.0	0.0	0.0	0.0	0.0	0.0
0.0	0.0	0.0	0.0	0.0	0.0	0.0	0.0
0.0	0.0	0.0	0.0	0.0	0.0	0.0	0.0
0.0	0.0	0.0	0.0	0.0	0.0	0.0	0.0
0.0	0.0	0.0	0.0	0.0	0.0	0.0	0.0
0.0	0.0	0.0	0.0	0.0	0.0	0.0	0.0
0.0	0.0	0.0	0.0	0.0	0.0	0.0	0.0
0.0	0.0	0.0	0.0	0.0	0.0	0.0	0.0
0.0	0.0	0.0	0.0	0.0	0.0	0.0	0.0
0.0	0.0	0.0	0.0	0.0	0.0	0.0	0.0
0.0	0.0	0.0	0.0	0.0	0.0	0.0	0.0
0.0	0.0	0.0	0.0	0.0	0.0	0.0	0.0
0.0	0.0	0.0	0.0	0.0	0.0	0.0	0.0
0.0	0.0	0.0	0.0	0.0	0.0	0.0	0.0
0.0	0.0	0.0	0.0	0.0	0.0	0.0	0.0
0.0	0.0	0.0	0.0	0.0	0.0	0.0	0.0
0.0	0.0	0.0	0.0	0.0	0.0	0.0	0.0
0.0	0.0	0.0	0.0	0.0	0.0	0.0	0.0
0.0	0.0	0.0	0.0	0.0	0.0	0.0	0.0
0.0	0.0	0.0	0.0	0.0	0.0	0.0	0.0
0.0	0.0	0.0	0.0	0.0	0.0	0.0	0.0
0.0	0.0	0.0	0.0	0.0	0.0	0.0	0.0
0.0	0.0	0.0	0.0	0.0	0.0	0.0	0.0
0.0	0.0	0.0	0.0	0.0	0.0	0.0	0.0
0.0	0.0	0.0	0.0	0.0	0.0	0.0	0.0
0.0	0.0	0.0	0.0	0.0	0.0	0.0	0.0
0.0	0.0	0.0	0.0	0.0	0.0	0.0	0.0
0.0	0.0	0.0	0.0	0.0	0.0	0.0	0.0
0.0	0.0	0.0	0.0	0.0	0.0	0.0	0.0
0.0	0.0	0.0	0.0	0.0	0.0	0.0	0.0
0.0	0.0	0.0	0.0	0.0	0.0	0.0	0.0
0.0	0.0	0.0	0.0	0.0	0.0	0.0	0.0

0.0	0.0	0.0	0.0	0.0	0.0	0.0	0.0
0.0	0.0	0.0	0.0	0.0	0.0	0.0	0.0
0.0	0.0	0.0	0.0	0.0	0.0	0.0	0.0
0.0	0.0	0.0	0.0	0.0	0.0	0.0	0.0
0.0	0.0	0.0	0.0	0.0	0.0	0.0	0.0
0.0	0.0	0.0	0.0	0.0	0.0	0.0	0.0
0.0	0.0	0.0	0.0	0.0	0.0	0.0	0.0
0.0	0.0	0.0	0.0	0.0	0.0	0.0	0.0
0.0	0.0	0.0	0.0	0.0	0.0	0.0	0.0
0.0	0.0	0.0	0.0	0.0	0.0	0.0	0.0
0.0	0.0	0.0	0.0	0.0	0.0	0.0	0.0
0.0	0.0	0.0	0.0	0.0	0.0	0.0	0.0
0.0	0.0	0.0	0.0	0.0	0.0	0.0	0.0
0.0	0.0	0.0	0.0	0.0	0.0	0.0	0.0
0.0	0.0	0.0	0.0	0.0	0.0	0.0	0.0
0.0	0.0	0.0	0.0	0.0	0.0	0.0	0.0
0.0	0.0	0.0	0.0	0.0	0.0	0.0	0.0
0.0	0.0	0.0	0.0	0.0	0.0	0.0	0.0
0.0	0.0	0.0	0.0	0.0	0.0	0.0	0.0
0.0	0.0	0.0	0.0	0.0	0.0	0.0	0.0
0.0	0.0	0.0	0.0	0.0	0.0	0.0	0.0
0.0	0.0	0.0	0.0	0.0	0.0	0.0	0.0
0.0	0.0	0.0	0.0	0.0	0.0	0.0	0.0
0.0	0.0	0.0	0.0	0.0	0.0	0.0	0.0
0.0	0.0	0.0	0.0	0.0	0.0	0.0	0.0
0.0	0.0	0.0	0.0	0.0	0.0	0.0	0.0
0.0	0.0	0.0	0.0	0.0	0.0	0.0	0.0
0.0	0.0	0.0	0.0	0.0	0.0	0.0	0.0
0.0	0.0	0.0	0.0	0.0	0.0	0.0	0.0
0.0	0.0	0.0	0.0	0.0	0.0	0.0	0.0
0.0	0.0	0.0	0.0	0.0	0.0	0.0	0.0
0.0	0.0	0.0	0.0	0.0	0.0	0.0	0.0
0.0	0.0	0.0	0.0	0.0	0.0	0.0	0.0
0.0	0.0	0.0	0.0	0.0	0.0	0.0	0.0
0.0	0.0	0.0	0.0	0.0	0.0	0.0	0.0
0.0	0.0	0.0	0.0	0.0	0.0	0.0	0.0
0.0	0.0	0.0	0.0	0.0	0.0	0.0	0.0
0.0	0.0	0.0	0.0	0.0	0.0	0.0	0.0
0.0	0.0	0.0	0.0	0.0	0.0	0.0	0.0
0.0	0.0	0.0	0.0	0.0	0.0	0.0	0.0
0.0	0.0	0.0	0.0	0.0	0.0	0.0	0.0
0.0	0.0	0.0	0.0	0.0	0.0	0.0	0.0
0.0	0.0	0.0	0.0	0.0	0.0	0.0	0.0
0.0	0.0	0.0	0.0	0.0	0.0	0.0	0.0
0.0	0.0	0.0	0.0	0.0	0.0	0.0	0.0
0.0	0.0	0.0	0.0	0.0	0.0	0.0	0.0
0.0	0.0	0.0	0.0	0.0	0.0	0.0	0.0
0.0	0.0	0.0	0.0	0.0	0.0	0.0	0.0

0.0	0.0	0.0	0.0	0.0	0.0	0.0	0.0
0.0	0.0	0.0	0.0	0.0	0.0	0.0	0.0
0.0	0.0	0.0	0.0	0.0	0.0	0.0	0.0
0.0	0.0	0.0	0.0	0.0	0.0	0.0	0.0
0.0	0.0	0.0	0.0	0.0	0.0	0.0	0.0
0.0	0.0	0.0	0.0	0.0	0.0	0.0	0.0
0.0	0.0	0.0	0.0	0.0	0.0	0.0	0.0
0.0	0.0	0.0	0.0	0.0	0.0	0.0	0.0
0.0	0.0	0.0	0.0	0.0	0.0	0.0	0.0
0.0	0.0	0.0	0.0	0.0	0.0	0.0	0.0
0.0	0.0	0.0	0.0	0.0	0.0	0.0	0.0
0.0	0.0	0.0	0.0	0.0	0.0	0.0	0.0
0.0	0.0	0.0	0.0	0.0	0.0	0.0	0.0
0.0	0.0	0.0	0.0	0.0	0.0	0.0	0.0
0.0	0.0	0.0	0.0	0.0	0.0	0.0	0.0
0.0	0.0	0.0	0.0	0.0	0.0	0.0	0.0
0.0	0.0	0.0	0.0	0.0	0.0	0.0	0.0
0.0	0.0	0.0	0.0	0.0	0.0	0.0	0.0
0.0	0.0	0.0	0.0	0.0	0.0	0.0	0.0
0.0	0.0	0.0	0.0	0.0	0.0	0.0	0.0
0.0	0.0	0.0	0.0	0.0	0.0	0.0	0.0
0.0	0.0	0.0	0.0	0.0	0.0	0.0	0.0
0.0	0.0	0.0	0.0	0.0	0.0	0.0	0.0
0.0	0.0	0.0	0.0	0.0	0.0	0.0	0.0
0.0	0.0	0.0	0.0	0.0	0.0	0.0	0.0
0.0	0.0	0.0	0.0	0.0	0.0	0.0	0.0
0.0	0.0	0.0	0.0	0.0	0.0	0.0	0.0
0.0	0.0	0.0	0.0	0.0	0.0	0.0	0.0
0.0	0.0	0.0	0.0	0.0	0.0	0.0	0.0
0.0	0.0	0.0	0.0	0.0	0.0	0.0	0.0
0.0	0.0	0.0	0.0	0.0	0.0	0.0	0.0
0.0	0.0	0.0	0.0	0.0	0.0	0.0	0.0
0.0	0.0	0.0	0.0	0.0	0.0	0.0	0.0
0.0	0.0	0.0	0.0	0.0	0.0	0.0	0.0
0.0	0.0	0.0	0.0	0.0	0.0	0.0	0.0
0.0	0.0	0.0	0.0	0.0	0.0	0.0	0.0
0.0	0.0	0.0	0.0	0.0	0.0	0.0	0.0
0.0	0.0	0.0	0.0	0.0	0.0	0.0	0.0
0.0	0.0	0.0	0.0	0.0	0.0	0.0	0.0
0.0	0.0	0.0	0.0	0.0	0.0	0.0	0.0
0.0	0.0	0.0	0.0	0.0	0.0	0.0	0.0
0.0	0.0	0.0	0.0	0.0	0.0	0.0	0.0
0.0	0.0	0.0	0.0	0.0	0.0	0.0	0.0
0.0	0.0	0.0	0.0	0.0	0.0	0.0	0.0
0.0	0.0	0.0	0.0	0.0	0.0	0.0	0.0
0.0	0.0	0.0	0.0	0.0	0.0	0.0	0.0
0.0	0.0	0.0	0.0	0.0	0.0	0.0	0.0
0.0	0.0	0.0	0.0	0.0	0.0	0.0	0.0

0.0	0.0	0.0	0.0	0.0	0.0	0.0	0.0
0.0	0.0	0.0	0.0	0.0	0.0	0.0	0.0
0.0	0.0	0.0	0.0	0.0	0.0	0.0	0.0
0.0	0.0	0.0	0.0	0.0	0.0	0.0	0.0
0.0	0.0	0.0	0.0	0.0	0.0	0.0	0.0
0.0	0.0	0.0	0.0	0.0	0.0	0.0	0.0
0.0	0.0	0.0	0.0	0.0	0.0	0.0	0.0
0.0	0.0	0.0	0.0	0.0	0.0	0.0	0.0
0.0	0.0	0.0	0.0	0.0	0.0	0.0	0.0
0.0	0.0	0.0	0.0	0.0	0.0	0.0	0.0
0.0	0.0	0.0	0.0	0.0	0.0	0.0	0.0
0.0	0.0	0.0	0.0	0.0	0.0	0.0	0.0
0.0	0.0	0.0	0.0	0.0	0.0	0.0	0.0
0.0	0.0	0.0	0.0	0.0	0.0	0.0	0.0
0.0	0.0	0.0	0.0	0.0	0.0	0.0	0.0
0.0	0.0	0.0	0.0	0.0	0.0	0.0	0.0
0.0	0.0	0.0	0.0	0.0	0.0	0.0	0.0
0.0	0.0	0.0	0.0	0.0	0.0	0.0	0.0
0.0	0.0	0.0	0.0	0.0	0.0	0.0	0.0
0.0	0.0	0.0	0.0	0.0	0.0	0.0	0.0
0.0	0.0	0.0	0.0	0.0	0.0	0.0	0.0
0.0	0.0	0.0	0.0	0.0	0.0	0.0	0.0
0.0	0.0	0.0	0.0	0.0	0.0	0.0	0.0
0.0	0.0	0.0	0.0	0.0	0.0	0.0	0.0
0.0	0.0	0.0	0.0	0.0	0.0	0.0	0.0
0.0	0.0	0.0	0.0	0.0	0.0	0.0	0.0
0.0	0.0	0.0	0.0	0.0	0.0	0.0	0.0
0.0	0.0	0.0	0.0	0.0	0.0	0.0	0.0
0.0	0.0	0.0	0.0	0.0	0.0	0.0	0.0
0.0	0.0	0.0	0.0	0.0	0.0	0.0	0.0
0.0	0.0	0.0	0.0	0.0	0.0	0.0	0.0
0.0	0.0	0.0	0.0	0.0	0.0	0.0	0.0
0.0	0.0	0.0	0.0	0.0	0.0	0.0	0.0
0.0	0.0	0.0	0.0	0.0	0.0	0.0	0.0
0.0	0.0	0.0	0.0	0.0	0.0	0.0	0.0
0.0	0.0	0.0	0.0	0.0	0.0	0.0	0.0
0.0	0.0	0.0	0.0	0.0	0.0	0.0	0.0
0.0	0.0	0.0	0.0	0.0	0.0	0.0	0.0
0.0	0.0	0.0	0.0	0.0	0.0	0.0	0.0
0.0	0.0	0.0	0.0	0.0	0.0	0.0	0.0
0.0	0.0	0.0	0.0	0.0	0.0	0.0	0.0
0.0	0.0	0.0	0.0	0.0	0.0	0.0	0.0
0.0	0.0	0.0	0.0	0.0	0.0	0.0	0.0
0.0	0.0	0.0	0.0	0.0	0.0	0.0	0.0
0.0	0.0	0.0	0.0	0.0	0.0	0.0	0.0
0.0	0.0	0.0	0.0	0.0	0.0	0.0	0.0
0.0	0.0	0.0	0.0	0.0	0.0	0.0	0.0

0.0	0.0	0.0	0.0	0.0	0.0	0.0	0.0
0.0	0.0	0.0	0.0	0.0	0.0	0.0	0.0
0.0	0.0	0.0	0.0	0.0	0.0	0.0	0.0
0.0	0.0	0.0	0.0	0.0	0.0	0.0	0.0
0.0	0.0	0.0	0.0	0.0	0.0	0.0	0.0
0.0	0.0	0.0	0.0	0.0	0.0	0.0	0.0
0.0	0.0	0.0	0.0	0.0	0.0	0.0	0.0
0.0	0.0	0.0	0.0	0.0	0.0	0.0	0.0
0.0	0.0	0.0	0.0	0.0	0.0	0.0	0.0
0.0	0.0	0.0	0.0	0.0	0.0	0.0	0.0
0.0	0.0	0.0	0.0	0.0	0.0	0.0	0.0
0.0	0.0	0.0	0.0	0.0	0.0	0.0	0.0
0.0	0.0	0.0	0.0	0.0	0.0	0.0	0.0
0.0	0.0	0.0	0.0	0.0	0.0	0.0	0.0
0.0	0.0	0.0	0.0	0.0	0.0	0.0	0.0
0.0	0.0	0.0	0.0	0.0	0.0	0.0	0.0
0.0	0.0	0.0	0.0	0.0	0.0	0.0	0.0
0.0	0.0	0.0	0.0	0.0	0.0	0.0	0.0
0.0	0.0	0.0	0.0	0.0	0.0	0.0	0.0
0.0	0.0	0.0	0.0	0.0	0.0	0.0	0.0
0.0	0.0	0.0	0.0	0.0	0.0	0.0	0.0
0.0	0.0	0.0	0.0	0.0	0.0	0.0	0.0
0.0	0.0	0.0	0.0	0.0	0.0	0.0	0.0
0.0	0.0	0.0	0.0	0.0	0.0	0.0	0.0
0.0	0.0	0.0	0.0	0.0	0.0	0.0	0.0
0.0	0.0	0.0	0.0	0.0	0.0	0.0	0.0
0.0	0.0	0.0	0.0	0.0	0.0	0.0	0.0
0.0	0.0	0.0	0.0	0.0	0.0	0.0	0.0
0.0	0.0	0.0	0.0	0.0	0.0	0.0	0.0
0.0	0.0	0.0	0.0	0.0	0.0	0.0	0.0
0.0	0.0	0.0	0.0	0.0	0.0	0.0	0.0
0.0	0.0	0.0	0.0	0.0	0.0	0.0	0.0
0.0	0.0	0.0	0.0	0.0	0.0	0.0	0.0
0.0	0.0	0.0	0.0	0.0	0.0	0.0	0.0
0.0	0.0	0.0	0.0	0.0	0.0	0.0	0.0
0.0	0.0	0.0	0.0	0.0	0.0	0.0	0.0
0.0	0.0	0.0	0.0	0.0	0.0	0.0	0.0
0.0	0.0	0.0	0.0	0.0	0.0	0.0	0.0
0.0	0.0	0.0	0.0	0.0	0.0	0.0	0.0
0.0	0.0	0.0	0.0	0.0	0.0	0.0	0.0
0.0	0.0	0.0	0.0	0.0	0.0	0.0	0.0
0.0	0.0	0.0	0.0	0.0	0.0	0.0	0.0
0.0	0.0	0.0	0.0	0.0	0.0	0.0	0.0
0.0	0.0	0.0	0.0	0.0	0.0	0.0	0.0
0.0	0.0	0.0	0.0	0.0	0.0	0.0	0.0
0.0	0.0	0.0	0.0	0.0	0.0	0.0	0.0
0.0	0.0	0.0	0.0	0.0	0.0	0.0	0.0
0.0	0.0	0.0	0.0	0.0	0.0	0.0	0.0

0.0	0.0	0.0	0.0	0.0	0.0	0.0	0.0
0.0	0.0	0.0	0.0	0.0	0.0	0.0	0.0
0.0	0.0	0.0	0.0	0.0	0.0	0.0	0.0
0.0	0.0	0.0	0.0	0.0	0.0	0.0	0.0
0.0	0.0	0.0	0.0	0.0	0.0	0.0	0.0
0.0	0.0	0.0	0.0	0.0	0.0	0.0	0.0
0.0	0.0	0.0	0.0	0.0	0.0	0.0	0.0
0.0	0.0	0.0	0.0	0.0	0.0	0.0	0.0
0.0	0.0	0.0	0.0	0.0	0.0	0.0	0.0
0.0	0.0	0.0	0.0	0.0	0.0	0.0	0.0
0.0	0.0	0.0	0.0	0.0	0.0	0.0	0.0
0.0	0.0	0.0	0.0	0.0	0.0	0.0	0.0
0.0	0.0	0.0	0.0	0.0	0.0	0.0	0.0
0.0	0.0	0.0	0.0	0.0	0.0	0.0	0.0
0.0	0.0	0.0	0.0	0.0	0.0	0.0	0.0
0.0	0.0	0.0	0.0	0.0	0.0	0.0	0.0
0.0	0.0	0.0	0.0	0.0	0.0	0.0	0.0
0.0	0.0	0.0	0.0	0.0	0.0	0.0	0.0
0.0	0.0	0.0	0.0	0.0	0.0	0.0	0.0
0.0	0.0	0.0	0.0	0.0	0.0	0.0	0.0
0.0	0.0	0.0	0.0	0.0	0.0	0.0	0.0
0.0	0.0	0.0	0.0	0.0	0.0	0.0	0.0
0.0	0.0	0.0	0.0	0.0	0.0	0.0	0.0
0.0	0.0	0.0	0.0	0.0	0.0	0.0	0.0
0.0	0.0	0.0	0.0	0.0	0.0	0.0	0.0
0.0	0.0	0.0	0.0	0.0	0.0	0.0	0.0
0.0	0.0	0.0	0.0	0.0	0.0	0.0	0.0
0.0	0.0	0.0	0.0	0.0	0.0	0.0	0.0
0.0	0.0	0.0	0.0	0.0	0.0	0.0	0.0
0.0	0.0	0.0	0.0	0.0	0.0	0.0	0.0
0.0	0.0	0.0	0.0	0.0	0.0	0.0	0.0
0.0	0.0	0.0	0.0	0.0	0.0	0.0	0.0
0.0	0.0	0.0	0.0	0.0	0.0	0.0	0.0
0.0	0.0	0.0	0.0	0.0	0.0	0.0	0.0
0.0	0.0	0.0	0.0	0.0	0.0	0.0	0.0
0.0	0.0	0.0	0.0	0.0	0.0	0.0	0.0
0.0	0.0	0.0	0.0	0.0	0.0	0.0	0.0
0.0	0.0	0.0	0.0	0.0	0.0	0.0	0.0
0.0	0.0	0.0	0.0	0.0	0.0	0.0	0.0
0.0	0.0	0.0	0.0	0.0	0.0	0.0	0.0
0.0	0.0	0.0	0.0	0.0	0.0	0.0	0.0
0.0	0.0	0.0	0.0	0.0	0.0	0.0	0.0
0.0	0.0	0.0	0.0	0.0	0.0	0.0	0.0
0.0	0.0	0.0	0.0	0.0	0.0	0.0	0.0
0.0	0.0	0.0	0.0	0.0	0.0	0.0	0.0
0.0	0.0	0.0	0.0	0.0	0.0	0.0	0.0
0.0	0.0	0.0	0.0	0.0	0.0	0.0	0.0
0.0	0.0	0.0	0.0	0.0	0.0	0.0	0.0

0.0	0.0	0.0	0.0	0.0	0.0	0.0	0.0
0.0	0.0	0.0	0.0	0.0	0.0	0.0	0.0
0.0	0.0	0.0	0.0	0.0	0.0	0.0	0.0
0.0	0.0	0.0	0.0	0.0	0.0	0.0	0.0
0.0	0.0	0.0	0.0	0.0	0.0	0.0	0.0
0.0	0.0	0.0	0.0	0.0	0.0	0.0	0.0
0.0	0.0	0.0	0.0	0.0	0.0	0.0	0.0
0.0	0.0	0.0	0.0	0.0	0.0	0.0	0.0
0.0	0.0	0.0	0.0	0.0	0.0	0.0	0.0
0.0	0.0	0.0	0.0	0.0	0.0	0.0	0.0
0.0	0.0	0.0	0.0	0.0	0.0	0.0	0.0
0.0	0.0	0.0	0.0	0.0	0.0	0.0	0.0
0.0	0.0	0.0	0.0	0.0	0.0	0.0	0.0
0.0	0.0	0.0	0.0	0.0	0.0	0.0	0.0
0.0	0.0	0.0	0.0	0.0	0.0	0.0	0.0
0.0	0.0	0.0	0.0	0.0	0.0	0.0	0.0
0.0	0.0	0.0	0.0	0.0	0.0	0.0	0.0
0.0	0.0	0.0	0.0	0.0	0.0	0.0	0.0
0.0	0.0	0.0	0.0	0.0	0.0	0.0	0.0
0.0	0.0	0.0	0.0	0.0	0.0	0.0	0.0
0.0	0.0	0.0	0.0	0.0	0.0	0.0	0.0
0.0	0.0	0.0	0.0	0.0	0.0	0.0	0.0
0.0	0.0	0.0	0.0	0.0	0.0	0.0	0.0
0.0	0.0	0.0	0.0	0.0	0.0	0.0	0.0
0.0	0.0	0.0	0.0	0.0	0.0	0.0	0.0
0.0	0.0	0.0	0.0	0.0	0.0	0.0	0.0
0.0	0.0	0.0	0.0	0.0	0.0	0.0	0.0
0.0	0.0	0.0	0.0	0.0	0.0	0.0	0.0
0.0	0.0	0.0	0.0	0.0	0.0	0.0	0.0
0.0	0.0	0.0	0.0	0.0	0.0	0.0	0.0
0.0	0.0	0.0	0.0	0.0	0.0	0.0	0.0
0.0	0.0	0.0	0.0	0.0	0.0	0.0	0.0
0.0	0.0	0.0	0.0	0.0	0.0	0.0	0.0
0.0	0.0	0.0	0.0	0.0	0.0	0.0	0.0
0.0	0.0	0.0	0.0	0.0	0.0	0.0	0.0
0.0	0.0	0.0	0.0	0.0	0.0	0.0	0.0
0.0	0.0	0.0	0.0	0.0	0.0	0.0	0.0
0.0	0.0	0.0	0.0	0.0	0.0	0.0	0.0
0.0	0.0	0.0	0.0	0.0	0.0	0.0	0.0
0.0	0.0	0.0	0.0	0.0	0.0	0.0	0.0
0.0	0.0	0.0	0.0	0.0	0.0	0.0	0.0
0.0	0.0	0.0	0.0	0.0	0.0	0.0	0.0
0.0	0.0	0.0	0.0	0.0	0.0	0.0	0.0
0.0	0.0	0.0	0.0	0.0	0.0	0.0	0.0
0.0	0.0	0.0	0.0	0.0	0.0	0.0	0.0
0.0	0.0	0.0	0.0	0.0	0.0	0.0	0.0
0.0	0.0	0.0	0.0	0.0	0.0	0.0	0.0
0.0	0.0	0.0	0.0	0.0	0.0	0.0	0.0

0.0	0.0	0.0	0.0	0.0	0.0	0.0	0.0
0.0	0.0	0.0	0.0	0.0	0.0	0.0	0.0
0.0	0.0	0.0	0.0	0.0	0.0	0.0	0.0
0.0	0.0	0.0	0.0	0.0	0.0	0.0	0.0
0.0	0.0	0.0	0.0	0.0	0.0	0.0	0.0
0.0	0.0	0.0	0.0	0.0	0.0	0.0	0.0
0.0	0.0	0.0	0.0	0.0	0.0	0.0	0.0
0.0	0.0	0.0	0.0	0.0	0.0	0.0	0.0
0.0	0.0	0.0	0.0	0.0	0.0	0.0	0.0
0.0	0.0	0.0	0.0	0.0	0.0	0.0	0.0
0.0	0.0	0.0	0.0	0.0	0.0	0.0	0.0
0.0	0.0	0.0	0.0	0.0	0.0	0.0	0.0
0.0	0.0	0.0	0.0	0.0	0.0	0.0	0.0
0.0	0.0	0.0	0.0	0.0	0.0	0.0	0.0
0.0	0.0	0.0	0.0	0.0	0.0	0.0	0.0
0.0	0.0	0.0	0.0	0.0	0.0	0.0	0.0
0.0	0.0	0.0	0.0	0.0	0.0	0.0	0.0
0.0	0.0	0.0	0.0	0.0	0.0	0.0	0.0
0.0	0.0	0.0	0.0	0.0	0.0	0.0	0.0
0.0	0.0	0.0	0.0	0.0	0.0	0.0	0.0
0.0	0.0	0.0	0.0	0.0	0.0	0.0	0.0
0.0	0.0	0.0	0.0	0.0	0.0	0.0	0.0
0.0	0.0	0.0	0.0	0.0	0.0	0.0	0.0
0.0	0.0	0.0	0.0	0.0	0.0	0.0	0.0
0.0	0.0	0.0	0.0	0.0	0.0	0.0	0.0
0.0	0.0	0.0	0.0	0.0	0.0	0.0	0.0
0.0	0.0	0.0	0.0	0.0	0.0	0.0	0.0
0.0	0.0	0.0	0.0	0.0	0.0	0.0	0.0
0.0	0.0	0.0	0.0	0.0	0.0	0.0	0.0
0.0	0.0	0.0	0.0	0.0	0.0	0.0	0.0
0.0	0.0	0.0	0.0	0.0	0.0	0.0	0.0
0.0	0.0	0.0	0.0	0.0	0.0	0.0	0.0
0.0	0.0	0.0	0.0	0.0	0.0	0.0	0.0
0.0	0.0	0.0	0.0	0.0	0.0	0.0	0.0
0.0	0.0	0.0	0.0	0.0	0.0	0.0	0.0
0.0	0.0	0.0	0.0	0.0	0.0	0.0	0.0
0.0	0.0	0.0	0.0	0.0	0.0	0.0	0.0
0.0	0.0	0.0	0.0	0.0	0.0	0.0	0.0
0.0	0.0	0.0	0.0	0.0	0.0	0.0	0.0
0.0	0.0	0.0	0.0	0.0	0.0	0.0	0.0
0.0	0.0	0.0	0.0	0.0	0.0	0.0	0.0
0.0	0.0	0.0	0.0	0.0	0.0	0.0	0.0
0.0	0.0	0.0	0.0	0.0	0.0	0.0	0.0
0.0	0.0	0.0	0.0	0.0	0.0	0.0	0.0
0.0	0.0	0.0	0.0	0.0	0.0	0.0	0.0
0.0	0.0	0.0	0.0	0.0	0.0	0.0	0.0
0.0	0.0	0.0	0.0	0.0	0.0	0.0	0.0

0.0	0.0	0.0	0.0	0.0	0.0	0.0	0.0
0.0	0.0	0.0	0.0	0.0	0.0	0.0	0.0
0.0	0.0	0.0	0.0	0.0	0.0	0.0	0.0
0.0	0.0	0.0	0.0	0.0	0.0	0.0	0.0
0.0	0.0	0.0	0.0	0.0	0.0	0.0	0.0
0.0	0.0	0.0	0.0	0.0	0.0	0.0	0.0
0.0	0.0	0.0	0.0	0.0	0.0	0.0	0.0
0.0	0.0	0.0	0.0	0.0	0.0	0.0	0.0
0.0	0.0	0.0	0.0	0.0	0.0	0.0	0.0
0.0	0.0	0.0	0.0	0.0	0.0	0.0	0.0
0.0	0.0	0.0	0.0	0.0	0.0	0.0	0.0
0.0	0.0	0.0	0.0	0.0	0.0	0.0	0.0
0.0	0.0	0.0	0.0	0.0	0.0	0.0	0.0
0.0	0.0	0.0	0.0	0.0	0.0	0.0	0.0
0.0	0.0	0.0	0.0	0.0	0.0	0.0	0.0
0.0	0.0	0.0	0.0	0.0	0.0	0.0	0.0
0.0	0.0	0.0	0.0	0.0	0.0	0.0	0.0
0.0	0.0	0.0	0.0	0.0	0.0	0.0	0.0
0.0	0.0	0.0	0.0	0.0	0.0	0.0	0.0
0.0	0.0	0.0	0.0	0.0	0.0	0.0	0.0
0.0	0.0	0.0	0.0	0.0	0.0	0.0	0.0
0.0	0.0	0.0	0.0	0.0	0.0	0.0	0.0
0.0	0.0	0.0	0.0	0.0	0.0	0.0	0.0
0.0	0.0	0.0	0.0	0.0	0.0	0.0	0.0
0.0	0.0	0.0	0.0	0.0	0.0	0.0	0.0
0.0	0.0	0.0	0.0	0.0	0.0	0.0	0.0
0.0	0.0	0.0	0.0	0.0	0.0	0.0	0.0
0.0	0.0	0.0	0.0	0.0	0.0	0.0	0.0
0.0	0.0	0.0	0.0	0.0	0.0	0.0	0.0
0.0	0.0	0.0	0.0	0.0	0.0	0.0	0.0
0.0	0.0	0.0	0.0	0.0	0.0	0.0	0.0
0.0	0.0	0.0	0.0	0.0	0.0	0.0	0.0
0.0	0.0	0.0	0.0	0.0	0.0	0.0	0.0
0.0	0.0	0.0	0.0	0.0	0.0	0.0	0.0
0.0	0.0	0.0	0.0	0.0	0.0	0.0	0.0
0.0	0.0	0.0	0.0	0.0	0.0	0.0	0.0
0.0	0.0	0.0	0.0	0.0	0.0	0.0	0.0
0.0	0.0	0.0	0.0	0.0	0.0	0.0	0.0
0.0	0.0	0.0	0.0	0.0	0.0	0.0	0.0
0.0	0.0	0.0	0.0	0.0	0.0	0.0	0.0
0.0	0.0	0.0	0.0	0.0	0.0	0.0	0.0
0.0	0.0	0.0	0.0	0.0	0.0	0.0	0.0
0.0	0.0	0.0	0.0	0.0	0.0	0.0	0.0
0.0	0.0	0.0	0.0	0.0	0.0	0.0	0.0
0.0	0.0	0.0	0.0	0.0	0.0	0.0	0.0
0.0	0.0	0.0	0.0	0.0	0.0	0.0	0.0
0.0	0.0	0.0	0.0	0.0	0.0	0.0	0.0

0.0	0.0	0.0	0.0	0.0	0.0	0.0	0.0
0.0	0.0	0.0	0.0	0.0	0.0	0.0	0.0
0.0	0.0	0.0	0.0	0.0	0.0	0.0	0.0
0.0	0.0	0.0	0.0	0.0	0.0	0.0	0.0
0.0	0.0	0.0	0.0	0.0	0.0	0.0	0.0
0.0	0.0	0.0	0.0	0.0	0.0	0.0	0.0
0.0	0.0	0.0	0.0	0.0	0.0	0.0	0.0
0.0	0.0	0.0	0.0	0.0	0.0	0.0	0.0
0.0	0.0	0.0	0.0	0.0	0.0	0.0	0.0
0.0	0.0	0.0	0.0	0.0	0.0	0.0	0.0
0.0	0.0	0.0	0.0	0.0	0.0	0.0	0.0
0.0	0.0	0.0	0.0	0.0	0.0	0.0	0.0
0.0	0.0	0.0	0.0	0.0	0.0	0.0	0.0
0.0	0.0	0.0	0.0	0.0	0.0	0.0	0.0
0.0	0.0	0.0	0.0	0.0	0.0	0.0	0.0
0.0	0.0	0.0	0.0	0.0	0.0	0.0	0.0
0.0	0.0	0.0	0.0	0.0	0.0	0.0	0.0
0.0	0.0	0.0	0.0	0.0	0.0	0.0	0.0
0.0	0.0	0.0	0.0	0.0	0.0	0.0	0.0
0.0	0.0	0.0	0.0	0.0	0.0	0.0	0.0
0.0	0.0	0.0	0.0	0.0	0.0	0.0	0.0
0.0	0.0	0.0	0.0	0.0	0.0	0.0	0.0
0.0	0.0	0.0	0.0	0.0	0.0	0.0	0.0
0.0	0.0	0.0	0.0	0.0	0.0	0.0	0.0
0.0	0.0	0.0	0.0	0.0	0.0	0.0	0.0
0.0	0.0	0.0	0.0	0.0	0.0	0.0	0.0
0.0	0.0	0.0	0.0	0.0	0.0	0.0	0.0
0.0	0.0	0.0	0.0	0.0	0.0	0.0	0.0
0.0	0.0	0.0	0.0	0.0	0.0	0.0	0.0
0.0	0.0	0.0	0.0	0.0	0.0	0.0	0.0
0.0	0.0	0.0	0.0	0.0	0.0	0.0	0.0
0.0	0.0	0.0	0.0	0.0	0.0	0.0	0.0
0.0	0.0	0.0	0.0	0.0	0.0	0.0	0.0
0.0	0.0	0.0	0.0	0.0	0.0	0.0	0.0
0.0	0.0	0.0	0.0	0.0	0.0	0.0	0.0
0.0	0.0	0.0	0.0	0.0	0.0	0.0	0.0
0.0	0.0	0.0	0.0	0.0	0.0	0.0	0.0
0.0	0.0	0.0	0.0	0.0	0.0	0.0	0.0
0.0	0.0	0.0	0.0	0.0	0.0	0.0	0.0
0.0	0.0	0.0	0.0	0.0	0.0	0.0	0.0
0.0	0.0	0.0	0.0	0.0	0.0	0.0	0.0
0.0	0.0	0.0	0.0	0.0	0.0	0.0	0.0
0.0	0.0	0.0	0.0	0.0	0.0	0.0	0.0
0.0	0.0	0.0	0.0	0.0	0.0	0.0	0.0
0.0	0.0	0.0	0.0	0.0	0.0	0.0	0.0
0.0	0.0	0.0	0.0	0.0	0.0	0.0	0.0
0.0	0.0	0.0	0.0	0.0	0.0	0.0	0.0

0.0	0.0	0.0	0.0	0.0	0.0	0.0	0.0
0.0	0.0	0.0	0.0	0.0	0.0	0.0	0.0
0.0	0.0	0.0	0.0	0.0	0.0	0.0	0.0
0.0	0.0	0.0	0.0	0.0	0.0	0.0	0.0
0.0	0.0	0.0	0.0	0.0	0.0	0.0	0.0
0.0	0.0	0.0	0.0	0.0	0.0	0.0	0.0
0.0	0.0	0.0	0.0	0.0	0.0	0.0	0.0
0.0	0.0	0.0	0.0	0.0	0.0	0.0	0.0
0.0	0.0	0.0	0.0	0.0	0.0	0.0	0.0
0.0	0.0	0.0	0.0	0.0	0.0	0.0	0.0
0.0	0.0	0.0	0.0	0.0	0.0	0.0	0.0
0.0	0.0	0.0	0.0	0.0	0.0	0.0	0.0
0.0	0.0	0.0	0.0	0.0	0.0	0.0	0.0
0.0	0.0	0.0	0.0	0.0	0.0	0.0	0.0
0.0	0.0	0.0	0.0	0.0	0.0	0.0	0.0
0.0	0.0	0.0	0.0	0.0	0.0	0.0	0.0
0.0	0.0	0.0	0.0	0.0	0.0	0.0	0.0
0.0	0.0	0.0	0.0	0.0	0.0	0.0	0.0
0.0	0.0	0.0	0.0	0.0	0.0	0.0	0.0
0.0	0.0	0.0	0.0	0.0	0.0	0.0	0.0
0.0	0.0	0.0	0.0	0.0	0.0	0.0	0.0
0.0	0.0	0.0	0.0	0.0	0.0	0.0	0.0
0.0	0.0	0.0	0.0	0.0	0.0	0.0	0.0
0.0	0.0	0.0	0.0	0.0	0.0	0.0	0.0
0.0	0.0	0.0	0.0	0.0	0.0	0.0	0.0
0.0	0.0	0.0	0.0	0.0	0.0	0.0	0.0
0.0	0.0	0.0	0.0	0.0	0.0	0.0	0.0
0.0	0.0	0.0	0.0	0.0	0.0	0.0	0.0
0.0	0.0	0.0	0.0	0.0	0.0	0.0	0.0
0.0	0.0	0.0	0.0	0.0	0.0	0.0	0.0
0.0	0.0	0.0	0.0	0.0	0.0	0.0	0.0
0.0	0.0	0.0	0.0	0.0	0.0	0.0	0.0
0.0	0.0	0.0	0.0	0.0	0.0	0.0	0.0
0.0	0.0	0.0	0.0	0.0	0.0	0.0	0.0
0.0	0.0	0.0	0.0	0.0	0.0	0.0	0.0
0.0	0.0	0.0	0.0	0.0	0.0	0.0	0.0
0.0	0.0	0.0	0.0	0.0	0.0	0.0	0.0
0.0	0.0	0.0	0.0	0.0	0.0	0.0	0.0
0.0	0.0	0.0	0.0	0.0	0.0	0.0	0.0
0.0	0.0	0.0	0.0	0.0	0.0	0.0	0.0
0.0	0.0	0.0	0.0	0.0	0.0	0.0	0.0
0.0	0.0	0.0	0.0	0.0	0.0	0.0	0.0
0.0	0.0	0.0	0.0	0.0	0.0	0.0	0.0
0.0	0.0	0.0	0.0	0.0	0.0	0.0	0.0
0.0	0.0	0.0	0.0	0.0	0.0	0.0	0.0
0.0	0.0	0.0	0.0	0.0	0.0	0.0	0.0
0.0	0.0	0.0	0.0	0.0	0.0	0.0	0.0

0.0	0.0	0.0	0.0	0.0	0.0	0.0	0.0
0.0	0.0	0.0	0.0	0.0	0.0	0.0	0.0
0.0	0.0	0.0	0.0	0.0	0.0	0.0	0.0
0.0	0.0	0.0	0.0	0.0	0.0	0.0	0.0
0.0	0.0	0.0	0.0	0.0	0.0	0.0	0.0
0.0	0.0	0.0	0.0	0.0	0.0	0.0	0.0
0.0	0.0	0.0	0.0	0.0	0.0	0.0	0.0
0.0	0.0	0.0	0.0	0.0	0.0	0.0	0.0
0.0	0.0	0.0	0.0	0.0	0.0	0.0	0.0
0.0	0.0	0.0	0.0	0.0	0.0	0.0	0.0
0.0	0.0	0.0	0.0	0.0	0.0	0.0	0.0
0.0	0.0	0.0	0.0	0.0	0.0	0.0	0.0
0.0	0.0	0.0	0.0	0.0	0.0	0.0	0.0
0.0	0.0	0.0	0.0	0.0	0.0	0.0	0.0
0.0	0.0	0.0	0.0	0.0	0.0	0.0	0.0
0.0	0.0	0.0	0.0	0.0	0.0	0.0	0.0
0.0	0.0	0.0	0.0	0.0	0.0	0.0	0.0
0.0	0.0	0.0	0.0	0.0	0.0	0.0	0.0
0.0	0.0	0.0	0.0	0.0	0.0	0.0	0.0
0.0	0.0	0.0	0.0	0.0	0.0	0.0	0.0
0.0	0.0	0.0	0.0	0.0	0.0	0.0	0.0
0.0	0.0	0.0	0.0	0.0	0.0	0.0	0.0
0.0	0.0	0.0	0.0	0.0	0.0	0.0	0.0
0.0	0.0	0.0	0.0	0.0	0.0	0.0	0.0
0.0	0.0	0.0	0.0	0.0	0.0	0.0	0.0
0.0	0.0	0.0	0.0	0.0	0.0	0.0	0.0
0.0	0.0	0.0	0.0	0.0	0.0	0.0	0.0
0.0	0.0	0.0	0.0	0.0	0.0	0.0	0.0
0.0	0.0	0.0	0.0	0.0	0.0	0.0	0.0
0.0	0.0	0.0	0.0	0.0	0.0	0.0	0.0
0.0	0.0	0.0	0.0	0.0	0.0	0.0	0.0
0.0	0.0	0.0	0.0	0.0	0.0	0.0	0.0
0.0	0.0	0.0	0.0	0.0	0.0	0.0	0.0
0.0	0.0	0.0	0.0	0.0	0.0	0.0	0.0
0.0	0.0	0.0	0.0	0.0	0.0	0.0	0.0
0.0	0.0	0.0	0.0	0.0	0.0	0.0	0.0
0.0	0.0	0.0	0.0	0.0	0.0	0.0	0.0
0.0	0.0	0.0	0.0	0.0	0.0	0.0	0.0
0.0	0.0	0.0	0.0	0.0	0.0	0.0	0.0
0.0	0.0	0.0	0.0	0.0	0.0	0.0	0.0
0.0	0.0	0.0	0.0	0.0	0.0	0.0	0.0
0.0	0.0	0.0	0.0	0.0	0.0	0.0	0.0
0.0	0.0	0.0	0.0	0.0	0.0	0.0	0.0
0.0	0.0	0.0	0.0	0.0	0.0	0.0	0.0
0.0	0.0	0.0	0.0	0.0	0.0	0.0	0.0
0.0	0.0	0.0	0.0	0.0	0.0	0.0	0.0
0.0	0.0	0.0	0.0	0.0	0.0	0.0	0.0

0.0	0.0	0.0	0.0	0.0	0.0	0.0	0.0
0.0	0.0	0.0	0.0	0.0	0.0	0.0	0.0
0.0	0.0	0.0	0.0	0.0	0.0	0.0	0.0
0.0	0.0	0.0	0.0	0.0	0.0	0.0	0.0
0.0	0.0	0.0	0.0	0.0	0.0	0.0	0.0
0.0	0.0	0.0	0.0	0.0	0.0	0.0	0.0
0.0	0.0	0.0	0.0	0.0	0.0	0.0	0.0
0.0	0.0	0.0	0.0	0.0	0.0	0.0	0.0
0.0	0.0	0.0	0.0	0.0	0.0	0.0	0.0
0.0	0.0	0.0	0.0	0.0	0.0	0.0	0.0
0.0	0.0	0.0	0.0	0.0	0.0	0.0	0.0
0.0	0.0	0.0	0.0	0.0	0.0	0.0	0.0
0.0	0.0	0.0	0.0	0.0	0.0	0.0	0.0
0.0	0.0	0.0	0.0	0.0	0.0	0.0	0.0
0.0	0.0	0.0	0.0	0.0	0.0	0.0	0.0
0.0	0.0	0.0	0.0	0.0	0.0	0.0	0.0
0.0	0.0	0.0	0.0	0.0	0.0	0.0	0.0
0.0	0.0	0.0	0.0	0.0	0.0	0.0	0.0
0.0	0.0	0.0	0.0	0.0	0.0	0.0	0.0
0.0	0.0	0.0	0.0	0.0	0.0	0.0	0.0
0.0	0.0	0.0	0.0	0.0	0.0	0.0	0.0
0.0	0.0	0.0	0.0	0.0	0.0	0.0	0.0
0.0	0.0	0.0	0.0	0.0	0.0	0.0	0.0
0.0	0.0	0.0	0.0	0.0	0.0	0.0	0.0
0.0	0.0	0.0	0.0	0.0	0.0	0.0	0.0
0.0	0.0	0.0	0.0	0.0	0.0	0.0	0.0
0.0	0.0	0.0	0.0	0.0	0.0	0.0	0.0
0.0	0.0	0.0	0.0	0.0	0.0	0.0	0.0
0.0	0.0	0.0	0.0	0.0	0.0	0.0	0.0
0.0	0.0	0.0	0.0	0.0	0.0	0.0	0.0
0.0	0.0	0.0	0.0	0.0	0.0	0.0	0.0
0.0	0.0	0.0	0.0	0.0	0.0	0.0	0.0
0.0	0.0	0.0	0.0	0.0	0.0	0.0	0.0
0.0	0.0	0.0	0.0	0.0	0.0	0.0	0.0
0.0	0.0	0.0	0.0	0.0	0.0	0.0	0.0
0.0	0.0	0.0	0.0	0.0	0.0	0.0	0.0
0.0	0.0	0.0	0.0	0.0	0.0	0.0	0.0
0.0	0.0	0.0	0.0	0.0	0.0	0.0	0.0
0.0	0.0	0.0	0.0	0.0	0.0	0.0	0.0
0.0	0.0	0.0	0.0	0.0	0.0	0.0	0.0
0.0	0.0	0.0	0.0	0.0	0.0	0.0	0.0
0.0	0.0	0.0	0.0	0.0	0.0	0.0	0.0
0.0	0.0	0.0	0.0	0.0	0.0	0.0	0.0
0.0	0.0	0.0	0.0	0.0	0.0	0.0	0.0
0.0	0.0	0.0	0.0	0.0	0.0	0.0	0.0
0.0	0.0	0.0	0.0	0.0	0.0	0.0	0.0
0.0	0.0	0.0	0.0	0.0	0.0	0.0	0.0

0.0	0.0	0.0	0.0	0.0	0.0	0.0	0.0
0.0	0.0	0.0	0.0	0.0	0.0	0.0	0.0
0.0	0.0	0.0	0.0	0.0	0.0	0.0	0.0
0.0	0.0	0.0	0.0	0.0	0.0	0.0	0.0
0.0	0.0	0.0	0.0	0.0	0.0	0.0	0.0
0.0	0.0	0.0	0.0	0.0	0.0	0.0	0.0
0.0	0.0	0.0	0.0	0.0	0.0	0.0	0.0
0.0	0.0	0.0	0.0	0.0	0.0	0.0	0.0
0.0	0.0	0.0	0.0	0.0	0.0	0.0	0.0
0.0	0.0	0.0	0.0	0.0	0.0	0.0	0.0
0.0	0.0	0.0	0.0	0.0	0.0	0.0	0.0
0.0	0.0	0.0	0.0	0.0	0.0	0.0	0.0
0.0	0.0	0.0	0.0	0.0	0.0	0.0	0.0
0.0	0.0	0.0	0.0	0.0	0.0	0.0	0.0
0.0	0.0	0.0	0.0	0.0	0.0	0.0	0.0
0.0	0.0	0.0	0.0	0.0	0.0	0.0	0.0
0.0	0.0	0.0	0.0	0.0	0.0	0.0	0.0
0.0	0.0	0.0	0.0	0.0	0.0	0.0	0.0
0.0	0.0	0.0	0.0	0.0	0.0	0.0	0.0
0.0	0.0	0.0	0.0	0.0	0.0	0.0	0.0
0.0	0.0	0.0	0.0	0.0	0.0	0.0	0.0
0.0	0.0	0.0	0.0	0.0	0.0	0.0	0.0
0.0	0.0	0.0	0.0	0.0	0.0	0.0	0.0
0.0	0.0	0.0	0.0	0.0	0.0	0.0	0.0
0.0	0.0	0.0	0.0	0.0	0.0	0.0	0.0
0.0	0.0	0.0	0.0	0.0	0.0	0.0	0.0
0.0	0.0	0.0	0.0	0.0	0.0	0.0	0.0
0.0	0.0	0.0	0.0	0.0	0.0	0.0	0.0
0.0	0.0	0.0	0.0	0.0	0.0	0.0	0.0
0.0	0.0	0.0	0.0	0.0	0.0	0.0	0.0
0.0	0.0	0.0	0.0	0.0	0.0	0.0	0.0
0.0	0.0	0.0	0.0	0.0	0.0	0.0	0.0
0.0	0.0	0.0	0.0	0.0	0.0	0.0	0.0
0.0	0.0	0.0	0.0	0.0	0.0	0.0	0.0
0.0	0.0	0.0	0.0	0.0	0.0	0.0	0.0
0.0	0.0	0.0	0.0	0.0	0.0	0.0	0.0
0.0	0.0	0.0	0.0	0.0	0.0	0.0	0.0
0.0	0.0	0.0	0.0	0.0	0.0	0.0	0.0
0.0	0.0	0.0	0.0	0.0	0.0	0.0	0.0
0.0	0.0	0.0	0.0	0.0	0.0	0.0	0.0
0.0	0.0	0.0	0.0	0.0	0.0	0.0	0.0
0.0	0.0	0.0	0.0	0.0	0.0	0.0	0.0
0.0	0.0	0.0	0.0	0.0	0.0	0.0	0.0
0.0	0.0	0.0	0.0	0.0	0.0	0.0	0.0
0.0	0.0	0.0	0.0	0.0	0.0	0.0	0.0
0.0	0.0	0.0	0.0	0.0	0.0	0.0	0.0
0.0	0.0	0.0	0.0	0.0	0.0	0.0	0.0
0.0	0.0	0.0	0.0	0.0	0.0	0.0	0.0

0.0	0.0	0.0	0.0	0.0	0.0	0.0	0.0
0.0	0.0	0.0	0.0	0.0	0.0	0.0	0.0
0.0	0.0	0.0	0.0	0.0	0.0	0.0	0.0
0.0	0.0	0.0	0.0	0.0	0.0	0.0	0.0
0.0	0.0	0.0	0.0	0.0	0.0	0.0	0.0
0.0	0.0	0.0	0.0	0.0	0.0	0.0	0.0
0.0	0.0	0.0	0.0	0.0	0.0	0.0	0.0
0.0	0.0	0.0	0.0	0.0	0.0	0.0	0.0
0.0	0.0	0.0	0.0	0.0	0.0	0.0	0.0
0.0	0.0	0.0	0.0	0.0	0.0	0.0	0.0
0.0	0.0	0.0	0.0	0.0	0.0	0.0	0.0
0.0	0.0	0.0	0.0	0.0	0.0	0.0	0.0
0.0	0.0	0.0	0.0	0.0	0.0	0.0	0.0
0.0	0.0	0.0	0.0	0.0	0.0	0.0	0.0
0.0	0.0	0.0	0.0	0.0	0.0	0.0	0.0
0.0	0.0	0.0	0.0	0.0	0.0	0.0	0.0
0.0	0.0	0.0	0.0	0.0	0.0	0.0	0.0
0.0	0.0	0.0	0.0	0.0	0.0	0.0	0.0
0.0	0.0	0.0	0.0	0.0	0.0	0.0	0.0
0.0	0.0	0.0	0.0	0.0	0.0	0.0	0.0
0.0	0.0	0.0	0.0	0.0	0.0	0.0	0.0
0.0	0.0	0.0	0.0	0.0	0.0	0.0	0.0
0.0	0.0	0.0	0.0	0.0	0.0	0.0	0.0
0.0	0.0	0.0	0.0	0.0	0.0	0.0	0.0
0.0	0.0	0.0	0.0	0.0	0.0	0.0	0.0
0.0	0.0	0.0	0.0	0.0	0.0	0.0	0.0
0.0	0.0	0.0	0.0	0.0	0.0	0.0	0.0
0.0	0.0	0.0	0.0	0.0	0.0	0.0	0.0
0.0	0.0	0.0	0.0	0.0	0.0	0.0	0.0
0.0	0.0	0.0	0.0	0.0	0.0	0.0	0.0
0.0	0.0	0.0	0.0	0.0	0.0	0.0	0.0
0.0	0.0	0.0	0.0	0.0	0.0	0.0	0.0
0.0	0.0	0.0	0.0	0.0	0.0	0.0	0.0
0.0	0.0	0.0	0.0	0.0	0.0	0.0	0.0
0.0	0.0	0.0	0.0	0.0	0.0	0.0	0.0
0.0	0.0	0.0	0.0	0.0	0.0	0.0	0.0
0.0	0.0	0.0	0.0	0.0	0.0	0.0	0.0
0.0	0.0	0.0	0.0	0.0	0.0	0.0	0.0
0.0	0.0	0.0	0.0	0.0	0.0	0.0	0.0
0.0	0.0	0.0	0.0	0.0	0.0	0.0	0.0
0.0	0.0	0.0	0.0	0.0	0.0	0.0	0.0
0.0	0.0	0.0	0.0	0.0	0.0	0.0	0.0
0.0	0.0	0.0	0.0	0.0	0.0	0.0	0.0
0.0	0.0	0.0	0.0	0.0	0.0	0.0	0.0
0.0	0.0	0.0	0.0	0.0	0.0	0.0	0.0
0.0	0.0	0.0	0.0	0.0	0.0	0.0	0.0
0.0	0.0	0.0	0.0	0.0	0.0	0.0	0.0

0.0	0.0	0.0	0.0	0.0	0.0	0.0	0.0
0.0	0.0	0.0	0.0	0.0	0.0	0.0	0.0
0.0	0.0	0.0	0.0	0.0	0.0	0.0	0.0
0.0	0.0	0.0	0.0	0.0	0.0	0.0	0.0
0.0	0.0	0.0	0.0	0.0	0.0	0.0	0.0
0.0	0.0	0.0	0.0	0.0	0.0	0.0	0.0
0.0	0.0	0.0	0.0	0.0	0.0	0.0	0.0
0.0	0.0	0.0	0.0	0.0	0.0	0.0	0.0
0.0	0.0	0.0	0.0	0.0	0.0	0.0	0.0
0.0	0.0	0.0	0.0	0.0	0.0	0.0	0.0
0.0	0.0	0.0	0.0	0.0	0.0	0.0	0.0
0.0	0.0	0.0	0.0	0.0	0.0	0.0	0.0
0.0	0.0	0.0	0.0	0.0	0.0	0.0	0.0
0.0	0.0	0.0	0.0	0.0	0.0	0.0	0.0
0.0	0.0	0.0	0.0	0.0	0.0	0.0	0.0
0.0	0.0	0.0	0.0	0.0	0.0	0.0	0.0
0.0	0.0	0.0	0.0	0.0	0.0	0.0	0.0
0.0	0.0	0.0	0.0	0.0	0.0	0.0	0.0
0.0	0.0	0.0	0.0	0.0	0.0	0.0	0.0
0.0	0.0	0.0	0.0	0.0	0.0	0.0	0.0
0.0	0.0	0.0	0.0	0.0	0.0	0.0	0.0
0.0	0.0	0.0	0.0	0.0	0.0	0.0	0.0
0.0	0.0	0.0	0.0	0.0	0.0	0.0	0.0
0.0	0.0	0.0	0.0	0.0	0.0	0.0	0.0
0.0	0.0	0.0	0.0	0.0	0.0	0.0	0.0
0.0	0.0	0.0	0.0	0.0	0.0	0.0	0.0
0.0	0.0	0.0	0.0	0.0	0.0	0.0	0.0
0.0	0.0	0.0	0.0	0.0	0.0	0.0	0.0
0.0	0.0	0.0	0.0	0.0	0.0	0.0	0.0
0.0	0.0	0.0	0.0	0.0	0.0	0.0	0.0
0.0	0.0	0.0	0.0	0.0	0.0	0.0	0.0
0.0	0.0	0.0	0.0	0.0	0.0	0.0	0.0
0.0	0.0	0.0	0.0	0.0	0.0	0.0	0.0
0.0	0.0	0.0	0.0	0.0	0.0	0.0	0.0
0.0	0.0	0.0	0.0	0.0	0.0	0.0	0.0
0.0	0.0	0.0	0.0	0.0	0.0	0.0	0.0
0.0	0.0	0.0	0.0	0.0	0.0	0.0	0.0
0.0	0.0	0.0	0.0	0.0	0.0	0.0	0.0
0.0	0.0	0.0	0.0	0.0	0.0	0.0	0.0
0.0	0.0	0.0	0.0	0.0	0.0	0.0	0.0
0.0	0.0	0.0	0.0	0.0	0.0	0.0	0.0
0.0	0.0	0.0	0.0	0.0	0.0	0.0	0.0
0.0	0.0	0.0	0.0	0.0	0.0	0.0	0.0
0.0	0.0	0.0	0.0	0.0	0.0	0.0	0.0
0.0	0.0	0.0	0.0	0.0	0.0	0.0	0.0
0.0	0.0	0.0	0.0	0.0	0.0	0.0	0.0
0.0	0.0	0.0	0.0	0.0	0.0	0.0	0.0
0.0	0.0	0.0	0.0	0.0	0.0	0.0	0.0

0.0	0.0	0.0	0.0	0.0	0.0	0.0	0.0
0.0	0.0	0.0	0.0	0.0	0.0	0.0	0.0
0.0	0.0	0.0	0.0	0.0	0.0	0.0	0.0
0.0	0.0	0.0	0.0	0.0	0.0	0.0	0.0
0.0	0.0	0.0	0.0	0.0	0.0	0.0	0.0
0.0	0.0	0.0	0.0	0.0	0.0	0.0	0.0
0.0	0.0	0.0	0.0	0.0	0.0	0.0	0.0
0.0	0.0	0.0	0.0	0.0	0.0	0.0	0.0
0.0	0.0	0.0	0.0	0.0	0.0	0.0	0.0
0.0	0.0	0.0	0.0	0.0	0.0	0.0	0.0
0.0	0.0	0.0	0.0	0.0	0.0	0.0	0.0
0.0	0.0	0.0	0.0	0.0	0.0	0.0	0.0
0.0	0.0	0.0	0.0	0.0	0.0	0.0	0.0
0.0	0.0	0.0	0.0	0.0	0.0	0.0	0.0
0.0	0.0	0.0	0.0	0.0	0.0	0.0	0.0
0.0	0.0	0.0	0.0	0.0	0.0	0.0	0.0
0.0	0.0	0.0	0.0	0.0	0.0	0.0	0.0
0.0	0.0	0.0	0.0	0.0	0.0	0.0	0.0
0.0	0.0	0.0	0.0	0.0	0.0	0.0	0.0
0.0	0.0	0.0	0.0	0.0	0.0	0.0	0.0
0.0	0.0	0.0	0.0	0.0	0.0	0.0	0.0
0.0	0.0	0.0	0.0	0.0	0.0	0.0	0.0
0.0	0.0	0.0	0.0	0.0	0.0	0.0	0.0
0.0	0.0	0.0	0.0	0.0	0.0	0.0	0.0
0.0	0.0	0.0	0.0	0.0	0.0	0.0	0.0
0.0	0.0	0.0	0.0	0.0	0.0	0.0	0.0
0.0	0.0	0.0	0.0	0.0	0.0	0.0	0.0
0.0	0.0	0.0	0.0	0.0	0.0	0.0	0.0
0.0	0.0	0.0	0.0	0.0	0.0	0.0	0.0
0.0	0.0	0.0	0.0	0.0	0.0	0.0	0.0
0.0	0.0	0.0	0.0	0.0	0.0	0.0	0.0
0.0	0.0	0.0	0.0	0.0	0.0	0.0	0.0
0.0	0.0	0.0	0.0	0.0	0.0	0.0	0.0
0.0	0.0	0.0	0.0	0.0	0.0	0.0	0.0
0.0	0.0	0.0	0.0	0.0	0.0	0.0	0.0
0.0	0.0	0.0	0.0	0.0	0.0	0.0	0.0
0.0	0.0	0.0	0.0	0.0	0.0	0.0	0.0
0.0	0.0	0.0	0.0	0.0	0.0	0.0	0.0
0.0	0.0	0.0	0.0	0.0	0.0	0.0	0.0
0.0	0.0	0.0	0.0	0.0	0.0	0.0	0.0
0.0	0.0	0.0	0.0	0.0	0.0	0.0	0.0
0.0	0.0	0.0	0.0	0.0	0.0	0.0	0.0
0.0	0.0	0.0	0.0	0.0	0.0	0.0	0.0
0.0	0.0	0.0	0.0	0.0	0.0	0.0	0.0
0.0	0.0	0.0	0.0	0.0	0.0	0.0	0.0
0.0	0.0	0.0	0.0	0.0	0.0	0.0	0.0
0.0	0.0	0.0	0.0	0.0	0.0	0.0	0.0

0.0	0.0	0.0	0.0	0.0	0.0	0.0	0.0
0.0	0.0	0.0	0.0	0.0	0.0	0.0	0.0
0.0	0.0	0.0	0.0	0.0	0.0	0.0	0.0
0.0	0.0	0.0	0.0	0.0	0.0	0.0	0.0
0.0	0.0	0.0	0.0	0.0	0.0	0.0	0.0
0.0	0.0	0.0	0.0	0.0	0.0	0.0	0.0
0.0	0.0	0.0	0.0	0.0	0.0	0.0	0.0
0.0	0.0	0.0	0.0	0.0	0.0	0.0	0.0
0.0	0.0	0.0	0.0	0.0	0.0	0.0	0.0
0.0	0.0	0.0	0.0	0.0	0.0	0.0	0.0
0.0	0.0	0.0	0.0	0.0	0.0	0.0	0.0
0.0	0.0	0.0	0.0	0.0	0.0	0.0	0.0
0.0	0.0	0.0	0.0	0.0	0.0	0.0	0.0
0.0	0.0	0.0	0.0	0.0	0.0	0.0	0.0
0.0	0.0	0.0	0.0	0.0	0.0	0.0	0.0
0.0	0.0	0.0	0.0	0.0	0.0	0.0	0.0
0.0	0.0	0.0	0.0	0.0	0.0	0.0	0.0
0.0	0.0	0.0	0.0	0.0	0.0	0.0	0.0
0.0	0.0	0.0	0.0	0.0	0.0	0.0	0.0
0.0	0.0	0.0	0.0	0.0	0.0	0.0	0.0
0.0	0.0	0.0	0.0	0.0	0.0	0.0	0.0
0.0	0.0	0.0	0.0	0.0	0.0	0.0	0.0
0.0	0.0	0.0	0.0	0.0	0.0	0.0	0.0
0.0	0.0	0.0	0.0	0.0	0.0	0.0	0.0
0.0	0.0	0.0	0.0	0.0	0.0	0.0	0.0
0.0	0.0	0.0	0.0	0.0	0.0	0.0	0.0
0.0	0.0	0.0	0.0	0.0	0.0	0.0	0.0
0.0	0.0	0.0	0.0	0.0	0.0	0.0	0.0
0.0	0.0	0.0	0.0	0.0	0.0	0.0	0.0
0.0	0.0	0.0	0.0	0.0	0.0	0.0	0.0
0.0	0.0	0.0	0.0	0.0	0.0	0.0	0.0
0.0	0.0	0.0	0.0	0.0	0.0	0.0	0.0
0.0	0.0	0.0	0.0	0.0	0.0	0.0	0.0
0.0	0.0	0.0	0.0	0.0	0.0	0.0	0.0
0.0	0.0	0.0	0.0	0.0	0.0	0.0	0.0
0.0	0.0	0.0	0.0	0.0	0.0	0.0	0.0
0.0	0.0	0.0	0.0	0.0	0.0	0.0	0.0
0.0	0.0	0.0	0.0	0.0	0.0	0.0	0.0
0.0	0.0	0.0	0.0	0.0	0.0	0.0	0.0
0.0	0.0	0.0	0.0	0.0	0.0	0.0	0.0
0.0	0.0	0.0	0.0	0.0	0.0	0.0	0.0
0.0	0.0	0.0	0.0	0.0	0.0	0.0	0.0
0.0	0.0	0.0	0.0	0.0	0.0	0.0	0.0
0.0	0.0	0.0	0.0	0.0	0.0	0.0	0.0
0.0	0.0	0.0	0.0	0.0	0.0	0.0	0.0
0.0	0.0	0.0	0.0	0.0	0.0	0.0	0.0
0.0	0.0	0.0	0.0	0.0	0.0	0.0	0.0

0.0	0.0	0.0	0.0	0.0	0.0	0.0	0.0
0.0	0.0	0.0	0.0	0.0	0.0	0.0	0.0
0.0	0.0	0.0	0.0	0.0	0.0	0.0	0.0
0.0	0.0	0.0	0.0	0.0	0.0	0.0	0.0
0.0	0.0	0.0	0.0	0.0	0.0	0.0	0.0
0.0	0.0	0.0	0.0	0.0	0.0	0.0	0.0
0.0	0.0	0.0	0.0	0.0	0.0	0.0	0.0
0.0	0.0	0.0	0.0	0.0	0.0	0.0	0.0
0.0	0.0	0.0	0.0	0.0	0.0	0.0	0.0
0.0	0.0	0.0	0.0	0.0	0.0	0.0	0.0
0.0	0.0	0.0	0.0	0.0	0.0	0.0	0.0
0.0	0.0	0.0	0.0	0.0	0.0	0.0	0.0
0.0	0.0	0.0	0.0	0.0	0.0	0.0	0.0
0.0	0.0	0.0	0.0	0.0	0.0	0.0	0.0
0.0	0.0	0.0	0.0	0.0	0.0	0.0	0.0
0.0	0.0	0.0	0.0	0.0	0.0	0.0	0.0
0.0	0.0	0.0	0.0	0.0	0.0	0.0	0.0
0.0	0.0	0.0	0.0	0.0	0.0	0.0	0.0
0.0	0.0	0.0	0.0	0.0	0.0	0.0	0.0
0.0	0.0	0.0	0.0	0.0	0.0	0.0	0.0
0.0	0.0	0.0	0.0	0.0	0.0	0.0	0.0
0.0	0.0	0.0	0.0	0.0	0.0	0.0	0.0
0.0	0.0	0.0	0.0	0.0	0.0	0.0	0.0
0.0	0.0	0.0	0.0	0.0	0.0	0.0	0.0
0.0	0.0	0.0	0.0	0.0	0.0	0.0	0.0
0.0	0.0	0.0	0.0	0.0	0.0	0.0	0.0
0.0	0.0	0.0	0.0	0.0	0.0	0.0	0.0
0.0	0.0	0.0	0.0	0.0	0.0	0.0	0.0
0.0	0.0	0.0	0.0	0.0	0.0	0.0	0.0
0.0	0.0	0.0	0.0	0.0	0.0	0.0	0.0
0.0	0.0	0.0	0.0	0.0	0.0	0.0	0.0
0.0	0.0	0.0	0.0	0.0	0.0	0.0	0.0
0.0	0.0	0.0	0.0	0.0	0.0	0.0	0.0
0.0	0.0	0.0	0.0	0.0	0.0	0.0	0.0
0.0	0.0	0.0	0.0	0.0	0.0	0.0	0.0
0.0	0.0	0.0	0.0	0.0	0.0	0.0	0.0
0.0	0.0	0.0	0.0	0.0	0.0	0.0	0.0
0.0	0.0	0.0	0.0	0.0	0.0	0.0	0.0
0.0	0.0	0.0	0.0	0.0	0.0	0.0	0.0
0.0	0.0	0.0	0.0	0.0	0.0	0.0	0.0
0.0	0.0	0.0	0.0	0.0	0.0	0.0	0.0
0.0	0.0	0.0	0.0	0.0	0.0	0.0	0.0
0.0	0.0	0.0	0.0	0.0	0.0	0.0	0.0
0.0	0.0	0.0	0.0	0.0	0.0	0.0	0.0
0.0	0.0	0.0	0.0	0.0	0.0	0.0	0.0
0.0	0.0	0.0	0.0	0.0	0.0	0.0	0.0
0.0	0.0	0.0	0.0	0.0	0.0	0.0	0.0
0.0	0.0	0.0	0.0	0.0	0.0	0.0	0.0

0.0	0.0	0.0	0.0	0.0	0.0	0.0	0.0
0.0	0.0	0.0	0.0	0.0	0.0	0.0	0.0
0.0	0.0	0.0	0.0	0.0	0.0	0.0	0.0
0.0	0.0	0.0	0.0	0.0	0.0	0.0	0.0
0.0	0.0	0.0	0.0	0.0	0.0	0.0	0.0
0.0	0.0	0.0	0.0	0.0	0.0	0.0	0.0
0.0	0.0	0.0	0.0	0.0	0.0	0.0	0.0
0.0	0.0	0.0	0.0	0.0	0.0	0.0	0.0
0.0	0.0	0.0	0.0	0.0	0.0	0.0	0.0
0.0	0.0	0.0	0.0	0.0	0.0	0.0	0.0
0.0	0.0	0.0	0.0	0.0	0.0	0.0	0.0
0.0	0.0	0.0	0.0	0.0	0.0	0.0	0.0
0.0	0.0	0.0	0.0	0.0	0.0	0.0	0.0
0.0	0.0	0.0	0.0	0.0	0.0	0.0	0.0
0.0	0.0	0.0	0.0	0.0	0.0	0.0	0.0
0.0	0.0	0.0	0.0	0.0	0.0	0.0	0.0
0.0	0.0	0.0	0.0	0.0	0.0	0.0	0.0
0.0	0.0	0.0	0.0	0.0	0.0	0.0	0.0
0.0	0.0	0.0	0.0	0.0	0.0	0.0	0.0
0.0	0.0	0.0	0.0	0.0	0.0	0.0	0.0
0.0	0.0	0.0	0.0	0.0	0.0	0.0	0.0
0.0	0.0	0.0	0.0	0.0	0.0	0.0	0.0
0.0	0.0	0.0	0.0	0.0	0.0	0.0	0.0
0.0	0.0	0.0	0.0	0.0	0.0	0.0	0.0
0.0	0.0	0.0	0.0	0.0	0.0	0.0	0.0
0.0	0.0	0.0	0.0	0.0	0.0	0.0	0.0
0.0	0.0	0.0	0.0	0.0	0.0	0.0	0.0
0.0	0.0	0.0	0.0	0.0	0.0	0.0	0.0
0.0	0.0	0.0	0.0	0.0	0.0	0.0	0.0
0.0	0.0	0.0	0.0	0.0	0.0	0.0	0.0
0.0	0.0	0.0	0.0	0.0	0.0	0.0	0.0
0.0	0.0	0.0	0.0	0.0	0.0	0.0	0.0
0.0	0.0	0.0	0.0	0.0	0.0	0.0	0.0
0.0	0.0	0.0	0.0	0.0	0.0	0.0	0.0
0.0	0.0	0.0	0.0	0.0	0.0	0.0	0.0
0.0	0.0	0.0	0.0	0.0	0.0	0.0	0.0
0.0	0.0	0.0	0.0	0.0	0.0	0.0	0.0
0.0	0.0	0.0	0.0	0.0	0.0	0.0	0.0
0.0	0.0	0.0	0.0	0.0	0.0	0.0	0.0
0.0	0.0	0.0	0.0	0.0	0.0	0.0	0.0
0.0	0.0	0.0	0.0	0.0	0.0	0.0	0.0
0.0	0.0	0.0	0.0	0.0	0.0	0.0	0.0
0.0	0.0	0.0	0.0	0.0	0.0	0.0	0.0
0.0	0.0	0.0	0.0	0.0	0.0	0.0	0.0
0.0	0.0	0.0	0.0	0.0	0.0	0.0	0.0
0.0	0.0	0.0	0.0	0.0	0.0	0.0	0.0
0.0	0.0	0.0	0.0	0.0	0.0	0.0	0.0

0.0	0.0	0.0	0.0	0.0	0.0	0.0	0.0
0.0	0.0	0.0	0.0	0.0	0.0	0.0	0.0
0.0	0.0	0.0	0.0	0.0	0.0	0.0	0.0
0.0	0.0	0.0	0.0	0.0	0.0	0.0	0.0
0.0	0.0	0.0	0.0	0.0	0.0	0.0	0.0
0.0	0.0	0.0	0.0	0.0	0.0	0.0	0.0
0.0	0.0	0.0	0.0	0.0	0.0	0.0	0.0
0.0	0.0	0.0	0.0	0.0	0.0	0.0	0.0
0.0	0.0	0.0	0.0	0.0	0.0	0.0	0.0
0.0	0.0	0.0	0.0	0.0	0.0	0.0	0.0
0.0	0.0	0.0	0.0	0.0	0.0	0.0	0.0
0.0	0.0	0.0	0.0	0.0	0.0	0.0	0.0
0.0	0.0	0.0	0.0	0.0	0.0	0.0	0.0
0.0	0.0	0.0	0.0	0.0	0.0	0.0	0.0
0.0	0.0	0.0	0.0	0.0	0.0	0.0	0.0
0.0	0.0	0.0	0.0	0.0	0.0	0.0	0.0
0.0	0.0	0.0	0.0	0.0	0.0	0.0	0.0
0.0	0.0	0.0	0.0	0.0	0.0	0.0	0.0
0.0	0.0	0.0	0.0	0.0	0.0	0.0	0.0
0.0	0.0	0.0	0.0	0.0	0.0	0.0	0.0
0.0	0.0	0.0	0.0	0.0	0.0	0.0	0.0
0.0	0.0	0.0	0.0	0.0	0.0	0.0	0.0
0.0	0.0	0.0	0.0	0.0	0.0	0.0	0.0
0.0	0.0	0.0	0.0	0.0	0.0	0.0	0.0
0.0	0.0	0.0	0.0	0.0	0.0	0.0	0.0
0.0	0.0	0.0	0.0	0.0	0.0	0.0	0.0
0.0	0.0	0.0	0.0	0.0	0.0	0.0	0.0
0.0	0.0	0.0	0.0	0.0	0.0	0.0	0.0
0.0	0.0	0.0	0.0	0.0	0.0	0.0	0.0
0.0	0.0	0.0	0.0	0.0	0.0	0.0	0.0
0.0	0.0	0.0	0.0	0.0	0.0	0.0	0.0
0.0	0.0	0.0	0.0	0.0	0.0	0.0	0.0
0.0	0.0	0.0	0.0	0.0	0.0	0.0	0.0
0.0	0.0	0.0	0.0	0.0	0.0	0.0	0.0
0.0	0.0	0.0	0.0	0.0	0.0	0.0	0.0
0.0	0.0	0.0	0.0	0.0	0.0	0.0	0.0
0.0	0.0	0.0	0.0	0.0	0.0	0.0	0.0
0.0	0.0	0.0	0.0	0.0	0.0	0.0	0.0
0.0	0.0	0.0	0.0	0.0	0.0	0.0	0.0
0.0	0.0	0.0	0.0	0.0	0.0	0.0	0.0
0.0	0.0	0.0	0.0	0.0	0.0	0.0	0.0
0.0	0.0	0.0	0.0	0.0	0.0	0.0	0.0
0.0	0.0	0.0	0.0	0.0	0.0	0.0	0.0
0.0	0.0	0.0	0.0	0.0	0.0	0.0	0.0
0.0	0.0	0.0	0.0	0.0	0.0	0.0	0.0
0.0	0.0	0.0	0.0	0.0	0.0	0.0	0.0
0.0	0.0	0.0	0.0	0.0	0.0	0.0	0.0

0.0	0.0	0.0	0.0	0.0	0.0	0.0	0.0
0.0	0.0	0.0	0.0	0.0	0.0	0.0	0.0
0.0	0.0	0.0	0.0	0.0	0.0	0.0	0.0
0.0	0.0	0.0	0.0	0.0	0.0	0.0	0.0
0.0	0.0	0.0	0.0	0.0	0.0	0.0	0.0
0.0	0.0	0.0	0.0	0.0	0.0	0.0	0.0
0.0	0.0	0.0	0.0	0.0	0.0	0.0	0.0
0.0	0.0	0.0	0.0	0.0	0.0	0.0	0.0
0.0	0.0	0.0	0.0	0.0	0.0	0.0	0.0
0.0	0.0	0.0	0.0	0.0	0.0	0.0	0.0
0.0	0.0	0.0	0.0	0.0	0.0	0.0	0.0
0.0	0.0	0.0	0.0	0.0	0.0	0.0	0.0
0.0	0.0	0.0	0.0	0.0	0.0	0.0	0.0
0.0	0.0	0.0	0.0	0.0	0.0	0.0	0.0
0.0	0.0	0.0	0.0	0.0	0.0	0.0	0.0
0.0	0.0	0.0	0.0	0.0	0.0	0.0	0.0
0.0	0.0	0.0	0.0	0.0	0.0	0.0	0.0
0.0	0.0	0.0	0.0	0.0	0.0	0.0	0.0
0.0	0.0	0.0	0.0	0.0	0.0	0.0	0.0
0.0	0.0	0.0	0.0	0.0	0.0	0.0	0.0
0.0	0.0	0.0	0.0	0.0	0.0	0.0	0.0
0.0	0.0	0.0	0.0	0.0	0.0	0.0	0.0
0.0	0.0	0.0	0.0	0.0	0.0	0.0	0.0
0.0	0.0	0.0	0.0	0.0	0.0	0.0	0.0
0.0	0.0	0.0	0.0	0.0	0.0	0.0	0.0
0.0	0.0	0.0	0.0	0.0	0.0	0.0	0.0
0.0	0.0	0.0	0.0	0.0	0.0	0.0	0.0
0.0	0.0	0.0	0.0	0.0	0.0	0.0	0.0
0.0	0.0	0.0	0.0	0.0	0.0	0.0	0.0
0.0	0.0	0.0	0.0	0.0	0.0	0.0	0.0
0.0	0.0	0.0	0.0	0.0	0.0	0.0	0.0
0.0	0.0	0.0	0.0	0.0	0.0	0.0	0.0
0.0	0.0	0.0	0.0	0.0	0.0	0.0	0.0
0.0	0.0	0.0	0.0	0.0	0.0	0.0	0.0
0.0	0.0	0.0	0.0	0.0	0.0	0.0	0.0
0.0	0.0	0.0	0.0	0.0	0.0	0.0	0.0
0.0	0.0	0.0	0.0	0.0	0.0	0.0	0.0
0.0	0.0	0.0	0.0	0.0	0.0	0.0	0.0
0.0	0.0	0.0	0.0	0.0	0.0	0.0	0.0
0.0	0.0	0.0	0.0	0.0	0.0	0.0	0.0
0.0	0.0	0.0	0.0	0.0	0.0	0.0	0.0
0.0	0.0	0.0	0.0	0.0	0.0	0.0	0.0
0.0	0.0	0.0	0.0	0.0	0.0	0.0	0.0
0.0	0.0	0.0	0.0	0.0	0.0	0.0	0.0
0.0	0.0	0.0	0.0	0.0	0.0	0.0	0.0
0.0	0.0	0.0	0.0	0.0	0.0	0.0	0.0
0.0	0.0	0.0	0.0	0.0	0.0	0.0	0.0
0.0	0.0	0.0	0.0	0.0	0.0	0.0	0.0

0.0	0.0	0.0	0.0	0.0	0.0	0.0	0.0
0.0	0.0	0.0	0.0	0.0	0.0	0.0	0.0
0.0	0.0	0.0	0.0	0.0	0.0	0.0	0.0
0.0	0.0	0.0	0.0	0.0	0.0	0.0	0.0
0.0	0.0	0.0	0.0	0.0	0.0	0.0	0.0
0.0	0.0	0.0	0.0	0.0	0.0	0.0	0.0
0.0	0.0	0.0	0.0	0.0	0.0	0.0	0.0
0.0	0.0	0.0	0.0	0.0	0.0	0.0	0.0
0.0	0.0	0.0	0.0	0.0	0.0	0.0	0.0
0.0	0.0	0.0	0.0	0.0	0.0	0.0	0.0
0.0	0.0	0.0	0.0	0.0	0.0	0.0	0.0
0.0	0.0	0.0	0.0	0.0	0.0	0.0	0.0
0.0	0.0	0.0	0.0	0.0	0.0	0.0	0.0
0.0	0.0	0.0	0.0	0.0	0.0	0.0	0.0
0.0	0.0	0.0	0.0	0.0	0.0	0.0	0.0
0.0	0.0	0.0	0.0	0.0	0.0	0.0	0.0
0.0	0.0	0.0	0.0	0.0	0.0	0.0	0.0
0.0	0.0	0.0	0.0	0.0	0.0	0.0	0.0
0.0	0.0	0.0	0.0	0.0	0.0	0.0	0.0
0.0	0.0	0.0	0.0	0.0	0.0	0.0	0.0
0.0	0.0	0.0	0.0	0.0	0.0	0.0	0.0
0.0	0.0	0.0	0.0	0.0	0.0	0.0	0.0
0.0	0.0	0.0	0.0	0.0	0.0	0.0	0.0
0.0	0.0	0.0	0.0	0.0	0.0	0.0	0.0
0.0	0.0	0.0	0.0	0.0	0.0	0.0	0.0
0.0	0.0	0.0	0.0	0.0	0.0	0.0	0.0
0.0	0.0	0.0	0.0	0.0	0.0	0.0	0.0
0.0	0.0	0.0	0.0	0.0	0.0	0.0	0.0
0.0	0.0	0.0	0.0	0.0	0.0	0.0	0.0
0.0	0.0	0.0	0.0	0.0	0.0	0.0	0.0
0.0	0.0	0.0	0.0	0.0	0.0	0.0	0.0
0.0	0.0	0.0	0.0	0.0	0.0	0.0	0.0
0.0	0.0	0.0	0.0	0.0	0.0	0.0	0.0
0.0	0.0	0.0	0.0	0.0	0.0	0.0	0.0
0.0	0.0	0.0	0.0	0.0	0.0	0.0	0.0
0.0	0.0	0.0	0.0	0.0	0.0	0.0	0.0
0.0	0.0	0.0	0.0	0.0	0.0	0.0	0.0
0.0	0.0	0.0	0.0	0.0	0.0	0.0	0.0
0.0	0.0	0.0	0.0	0.0	0.0	0.0	0.0
0.0	0.0	0.0	0.0	0.0	0.0	0.0	0.0
0.0	0.0	0.0	0.0	0.0	0.0	0.0	0.0
0.0	0.0	0.0	0.0	0.0	0.0	0.0	0.0
0.0	0.0	0.0	0.0	0.0	0.0	0.0	0.0
0.0	0.0	0.0	0.0	0.0	0.0	0.0	0.0
0.0	0.0	0.0	0.0	0.0	0.0	0.0	0.0
0.0	0.0	0.0	0.0	0.0	0.0	0.0	0.0
0.0	0.0	0.0	0.0	0.0	0.0	0.0	0.0

0.0	0.0	0.0	0.0	0.0	0.0	0.0	0.0
0.0	0.0	0.0	0.0	0.0	0.0	0.0	0.0
0.0	0.0	0.0	0.0	0.0	0.0	0.0	0.0
0.0	0.0	0.0	0.0	0.0	0.0	0.0	0.0
0.0	0.0	0.0	0.0	0.0	0.0	0.0	0.0
0.0	0.0	0.0	0.0	0.0	0.0	0.0	0.0
0.0	0.0	0.0	0.0	0.0	0.0	0.0	0.0
0.0	0.0	0.0	0.0	0.0	0.0	0.0	0.0
0.0	0.0	0.0	0.0	0.0	0.0	0.0	0.0
0.0	0.0	0.0	0.0	0.0	0.0	0.0	0.0
0.0	0.0	0.0	0.0	0.0	0.0	0.0	0.0
0.0	0.0	0.0	0.0	0.0	0.0	0.0	0.0
0.0	0.0	0.0	0.0	0.0	0.0	0.0	0.0
0.0	0.0	0.0	0.0	0.0	0.0	0.0	0.0
0.0	0.0	0.0	0.0	0.0	0.0	0.0	0.0
0.0	0.0	0.0	0.0	0.0	0.0	0.0	0.0
0.0	0.0	0.0	0.0	0.0	0.0	0.0	0.0
0.0	0.0	0.0	0.0	0.0	0.0	0.0	0.0
0.0	0.0	0.0	0.0	0.0	0.0	0.0	0.0
0.0	0.0	0.0	0.0	0.0	0.0	0.0	0.0
0.0	0.0	0.0	0.0	0.0	0.0	0.0	0.0
0.0	0.0	0.0	0.0	0.0	0.0	0.0	0.0
0.0	0.0	0.0	0.0	0.0	0.0	0.0	0.0
0.0	0.0	0.0	0.0	0.0	0.0	0.0	0.0
0.0	0.0	0.0	0.0	0.0	0.0	0.0	0.0
0.0	0.0	0.0	0.0	0.0	0.0	0.0	0.0
0.0	0.0	0.0	0.0	0.0	0.0	0.0	0.0
0.0	0.0	0.0	0.0	0.0	0.0	0.0	0.0
0.0	0.0	0.0	0.0	0.0	0.0	0.0	0.0
0.0	0.0	0.0	0.0	0.0	0.0	0.0	0.0
0.0	0.0	0.0	0.0	0.0	0.0	0.0	0.0
0.0	0.0	0.0	0.0	0.0	0.0	0.0	0.0
0.0	0.0	0.0	0.0	0.0	0.0	0.0	0.0
0.0	0.0	0.0	0.0	0.0	0.0	0.0	0.0
0.0	0.0	0.0	0.0	0.0	0.0	0.0	0.0
0.0	0.0	0.0	0.0	0.0	0.0	0.0	0.0
0.0	0.0	0.0	0.0	0.0	0.0	0.0	0.0
0.0	0.0	0.0	0.0	0.0	0.0	0.0	0.0
0.0	0.0	0.0	0.0	0.0	0.0	0.0	0.0
0.0	0.0	0.0	0.0	0.0	0.0	0.0	0.0
0.0	0.0	0.0	0.0	0.0	0.0	0.0	0.0
0.0	0.0	0.0	0.0	0.0	0.0	0.0	0.0
0.0	0.0	0.0	0.0	0.0	0.0	0.0	0.0
0.0	0.0	0.0	0.0	0.0	0.0	0.0	0.0
0.0	0.0	0.0	0.0	0.0	0.0	0.0	0.0
0.0	0.0	0.0	0.0	0.0	0.0	0.0	0.0
0.0	0.0	0.0	0.0	0.0	0.0	0.0	0.0

0.0	0.0	0.0	0.0	0.0	0.0	0.0	0.0
0.0	0.0	0.0	0.0	0.0	0.0	0.0	0.0
0.0	0.0	0.0	0.0	0.0	0.0	0.0	0.0
0.0	0.0	0.0	0.0	0.0	0.0	0.0	0.0
0.0	0.0	0.0	0.0	0.0	0.0	0.0	0.0
0.0	0.0	0.0	0.0	0.0	0.0	0.0	0.0
0.0	0.0	0.0	0.0	0.0	0.0	0.0	0.0
0.0	0.0	0.0	0.0	0.0	0.0	0.0	0.0
0.0	0.0	0.0	0.0	0.0	0.0	0.0	0.0
0.0	0.0	0.0	0.0	0.0	0.0	0.0	0.0
0.0	0.0	0.0	0.0	0.0	0.0	0.0	0.0
0.0	0.0	0.0	0.0	0.0	0.0	0.0	0.0
0.0	0.0	0.0	0.0	0.0	0.0	0.0	0.0
0.0	0.0	0.0	0.0	0.0	0.0	0.0	0.0
0.0	0.0	0.0	0.0	0.0	0.0	0.0	0.0
0.0	0.0	0.0	0.0	0.0	0.0	0.0	0.0
0.0	0.0	0.0	0.0	0.0	0.0	0.0	0.0
0.0	0.0	0.0	0.0	0.0	0.0	0.0	0.0
0.0	0.0	0.0	0.0	0.0	0.0	0.0	0.0
0.0	0.0	0.0	0.0	0.0	0.0	0.0	0.0
0.0	0.0	0.0	0.0	0.0	0.0	0.0	0.0
0.0	0.0	0.0	0.0	0.0	0.0	0.0	0.0
0.0	0.0	0.0	0.0	0.0	0.0	0.0	0.0
0.0	0.0	0.0	0.0	0.0	0.0	0.0	0.0
0.0	0.0	0.0	0.0	0.0	0.0	0.0	0.0
0.0	0.0	0.0	0.0	0.0	0.0	0.0	0.0
0.0	0.0	0.0	0.0	0.0	0.0	0.0	0.0
0.0	0.0	0.0	0.0	0.0	0.0	0.0	0.0
0.0	0.0	0.0	0.0	0.0	0.0	0.0	0.0
0.0	0.0	0.0	0.0	0.0	0.0	0.0	0.0
0.0	0.0	0.0	0.0	0.0	0.0	0.0	0.0
0.0	0.0	0.0	0.0	0.0	0.0	0.0	0.0
0.0	0.0	0.0	0.0	0.0	0.0	0.0	0.0
0.0	0.0	0.0	0.0	0.0	0.0	0.0	0.0
0.0	0.0	0.0	0.0	0.0	0.0	0.0	0.0
0.0	0.0	0.0	0.0	0.0	0.0	0.0	0.0
0.0	0.0	0.0	0.0	0.0	0.0	0.0	0.0
0.0	0.0	0.0	0.0	0.0	0.0	0.0	0.0
0.0	0.0	0.0	0.0	0.0	0.0	0.0	0.0
0.0	0.0	0.0	0.0	0.0	0.0	0.0	0.0
0.0	0.0	0.0	0.0	0.0	0.0	0.0	0.0
0.0	0.0	0.0	0.0	0.0	0.0	0.0	0.0
0.0	0.0	0.0	0.0	0.0	0.0	0.0	0.0
0.0	0.0	0.0	0.0	0.0	0.0	0.0	0.0
0.0	0.0	0.0	0.0	0.0	0.0	0.0	0.0
0.0	0.0	0.0	0.0	0.0	0.0	0.0	0.0
0.0	0.0	0.0	0.0	0.0	0.0	0.0	0.0
0.0	0.0	0.0	0.0	0.0	0.0	0.0	0.0

0.0	0.0	0.0	0.0	0.0	0.0	0.0	0.0
0.0	0.0	0.0	0.0	0.0	0.0	0.0	0.0
0.0	0.0	0.0	0.0	0.0	0.0	0.0	0.0
0.0	0.0	0.0	0.0	0.0	0.0	0.0	0.0
0.0	0.0	0.0	0.0	0.0	0.0	0.0	0.0
0.0	0.0	0.0	0.0	0.0	0.0	0.0	0.0
0.0	0.0	0.0	0.0	0.0	0.0	0.0	0.0
0.0	0.0	0.0	0.0	0.0	0.0	0.0	0.0
0.0	0.0	0.0	0.0	0.0	0.0	0.0	0.0
0.0	0.0	0.0	0.0	0.0	0.0	0.0	0.0
0.0	0.0	0.0	0.0	0.0	0.0	0.0	0.0
0.0	0.0	0.0	0.0	0.0	0.0	0.0	0.0
0.0	0.0	0.0	0.0	0.0	0.0	0.0	0.0
0.0	0.0	0.0	0.0	0.0	0.0	0.0	0.0
0.0	0.0	0.0	0.0	0.0	0.0	0.0	0.0
0.0	0.0	0.0	0.0	0.0	0.0	0.0	0.0
0.0	0.0	0.0	0.0	0.0	0.0	0.0	0.0
0.0	0.0	0.0	0.0	0.0	0.0	0.0	0.0
0.0	0.0	0.0	0.0	0.0	0.0	0.0	0.0
0.0	0.0	0.0	0.0	0.0	0.0	0.0	0.0
0.0	0.0	0.0	0.0	0.0	0.0	0.0	0.0
0.0	0.0	0.0	0.0	0.0	0.0	0.0	0.0
0.0	0.0	0.0	0.0	0.0	0.0	0.0	0.0
0.0	0.0	0.0	0.0	0.0	0.0	0.0	0.0
0.0	0.0	0.0	0.0	0.0	0.0	0.0	0.0
0.0	0.0	0.0	0.0	0.0	0.0	0.0	0.0
0.0	0.0	0.0	0.0	0.0	0.0	0.0	0.0
0.0	0.0	0.0	0.0	0.0	0.0	0.0	0.0
0.0	0.0	0.0	0.0	0.0	0.0	0.0	0.0
0.0	0.0	0.0	0.0	0.0	0.0	0.0	0.0
0.0	0.0	0.0	0.0	0.0	0.0	0.0	0.0
0.0	0.0	0.0	0.0	0.0	0.0	0.0	0.0
0.0	0.0	0.0	0.0	0.0	0.0	0.0	0.0
0.0	0.0	0.0	0.0	0.0	0.0	0.0	0.0
0.0	0.0	0.0	0.0	0.0	0.0	0.0	0.0
0.0	0.0	0.0	0.0	0.0	0.0	0.0	0.0
0.0	0.0	0.0	0.0	0.0	0.0	0.0	0.0
0.0	0.0	0.0	0.0	0.0	0.0	0.0	0.0
0.0	0.0	0.0	0.0	0.0	0.0	0.0	0.0
0.0	0.0	0.0	0.0	0.0	0.0	0.0	0.0
0.0	0.0	0.0	0.0	0.0	0.0	0.0	0.0
0.0	0.0	0.0	0.0	0.0	0.0	0.0	0.0
0.0	0.0	0.0	0.0	0.0	0.0	0.0	0.0
0.0	0.0	0.0	0.0	0.0	0.0	0.0	0.0
0.0	0.0	0.0	0.0	0.0	0.0	0.0	0.0
0.0	0.0	0.0	0.0	0.0	0.0	0.0	0.0
0.0	0.0	0.0	0.0	0.0	0.0	0.0	0.0

0.0	0.0	0.0	0.0	0.0	0.0	0.0	0.0
0.0	0.0	0.0	0.0	0.0	0.0	0.0	0.0
0.0	0.0	0.0	0.0	0.0	0.0	0.0	0.0
0.0	0.0	0.0	0.0	0.0	0.0	0.0	0.0
0.0	0.0	0.0	0.0	0.0	0.0	0.0	0.0
0.0	0.0	0.0	0.0	0.0	0.0	0.0	0.0
0.0	0.0	0.0	0.0	0.0	0.0	0.0	0.0
0.0	0.0	0.0	0.0	0.0	0.0	0.0	0.0
0.0	0.0	0.0	0.0	0.0	0.0	0.0	0.0
0.0	0.0	0.0	0.0	0.0	0.0	0.0	0.0
0.0	0.0	0.0	0.0	0.0	0.0	0.0	0.0
0.0	0.0	0.0	0.0	0.0	0.0	0.0	0.0
0.0	0.0	0.0	0.0	0.0	0.0	0.0	0.0
0.0	0.0	0.0	0.0	0.0	0.0	0.0	0.0
0.0	0.0	0.0	0.0	0.0	0.0	0.0	0.0
0.0	0.0	0.0	0.0	0.0	0.0	0.0	0.0
0.0	0.0	0.0	0.0	0.0	0.0	0.0	0.0
0.0	0.0	0.0	0.0	0.0	0.0	0.0	0.0
0.0	0.0	0.0	0.0	0.0	0.0	0.0	0.0
0.0	0.0	0.0	0.0	0.0	0.0	0.0	0.0
0.0	0.0	0.0	0.0	0.0	0.0	0.0	0.0
0.0	0.0	0.0	0.0	0.0	0.0	0.0	0.0
0.0	0.0	0.0	0.0	0.0	0.0	0.0	0.0
0.0	0.0	0.0	0.0	0.0	0.0	0.0	0.0
0.0	0.0	0.0	0.0	0.0	0.0	0.0	0.0
0.0	0.0	0.0	0.0	0.0	0.0	0.0	0.0
0.0	0.0	0.0	0.0	0.0	0.0	0.0	0.0
0.0	0.0	0.0	0.0	0.0	0.0	0.0	0.0
0.0	0.0	0.0	0.0	0.0	0.0	0.0	0.0
0.0	0.0	0.0	0.0	0.0	0.0	0.0	0.0
0.0	0.0	0.0	0.0	0.0	0.0	0.0	0.0
0.0	0.0	0.0	0.0	0.0	0.0	0.0	0.0
0.0	0.0	0.0	0.0	0.0	0.0	0.0	0.0
0.0	0.0	0.0	0.0	0.0	0.0	0.0	0.0
0.0	0.0	0.0	0.0	0.0	0.0	0.0	0.0
0.0	0.0	0.0	0.0	0.0	0.0	0.0	0.0
0.0	0.0	0.0	0.0	0.0	0.0	0.0	0.0
0.0	0.0	0.0	0.0	0.0	0.0	0.0	0.0
0.0	0.0	0.0	0.0	0.0	0.0	0.0	0.0
0.0	0.0	0.0	0.0	0.0	0.0	0.0	0.0
0.0	0.0	0.0	0.0	0.0	0.0	0.0	0.0
0.0	0.0	0.0	0.0	0.0	0.0	0.0	0.0
0.0	0.0	0.0	0.0	0.0	0.0	0.0	0.0
0.0	0.0	0.0	0.0	0.0	0.0	0.0	0.0
0.0	0.0	0.0	0.0	0.0	0.0	0.0	0.0
0.0	0.0	0.0	0.0	0.0	0.0	0.0	0.0
0.0	0.0	0.0	0.0	0.0	0.0	0.0	0.0

0.0	0.0	0.0	0.0	0.0	0.0	0.0	0.0
0.0	0.0	0.0	0.0	0.0	0.0	0.0	0.0
0.0	0.0	0.0	0.0	0.0	0.0	0.0	0.0
0.0	0.0	0.0	0.0	0.0	0.0	0.0	0.0
0.0	0.0	0.0	0.0	0.0	0.0	0.0	0.0
0.0	0.0	0.0	0.0	0.0	0.0	0.0	0.0
0.0	0.0	0.0	0.0	0.0	0.0	0.0	0.0
0.0	0.0	0.0	0.0	0.0	0.0	0.0	0.0
0.0	0.0	0.0	0.0	0.0	0.0	0.0	0.0
0.0	0.0	0.0	0.0	0.0	0.0	0.0	0.0
0.0	0.0	0.0	0.0	0.0	0.0	0.0	0.0
0.0	0.0	0.0	0.0	0.0	0.0	0.0	0.0
0.0	0.0	0.0	0.0	0.0	0.0	0.0	0.0
0.0	0.0	0.0	0.0	0.0	0.0	0.0	0.0
0.0	0.0	0.0	0.0	0.0	0.0	0.0	0.0
0.0	0.0	0.0	0.0	0.0	0.0	0.0	0.0
0.0	0.0	0.0	0.0	0.0	0.0	0.0	0.0
0.0	0.0	0.0	0.0	0.0	0.0	0.0	0.0
0.0	0.0	0.0	0.0	0.0	0.0	0.0	0.0
0.0	0.0	0.0	0.0	0.0	0.0	0.0	0.0
0.0	0.0	0.0	0.0	0.0	0.0	0.0	0.0
0.0	0.0	0.0	0.0	0.0	0.0	0.0	0.0
0.0	0.0	0.0	0.0	0.0	0.0	0.0	0.0
0.0	0.0	0.0	0.0	0.0	0.0	0.0	0.0
0.0	0.0	0.0	0.0	0.0	0.0	0.0	0.0
0.0	0.0	0.0	0.0	0.0	0.0	0.0	0.0
0.0	0.0	0.0	0.0	0.0	0.0	0.0	0.0
0.0	0.0	0.0	0.0	0.0	0.0	0.0	0.0
0.0	0.0	0.0	0.0	0.0	0.0	0.0	0.0
0.0	0.0	0.0	0.0	0.0	0.0	0.0	0.0
0.0	0.0	0.0	0.0	0.0	0.0	0.0	0.0
0.0	0.0	0.0	0.0	0.0	0.0	0.0	0.0
0.0	0.0	0.0	0.0	0.0	0.0	0.0	0.0
0.0	0.0	0.0	0.0	0.0	0.0	0.0	0.0
0.0	0.0	0.0	0.0	0.0	0.0	0.0	0.0
0.0	0.0	0.0	0.0	0.0	0.0	0.0	0.0
0.0	0.0	0.0	0.0	0.0	0.0	0.0	0.0
0.0	0.0	0.0	0.0	0.0	0.0	0.0	0.0
0.0	0.0	0.0	0.0	0.0	0.0	0.0	0.0
0.0	0.0	0.0	0.0	0.0	0.0	0.0	0.0
0.0	0.0	0.0	0.0	0.0	0.0	0.0	0.0
0.0	0.0	0.0	0.0	0.0	0.0	0.0	0.0
0.0	0.0	0.0	0.0	0.0	0.0	0.0	0.0
0.0	0.0	0.0	0.0	0.0	0.0	0.0	0.0
0.0	0.0	0.0	0.0	0.0	0.0	0.0	0.0
0.0	0.0	0.0	0.0	0.0	0.0	0.0	0.0
0.0	0.0	0.0	0.0	0.0	0.0	0.0	0.0
0.0	0.0	0.0	0.0	0.0	0.0	0.0	0.0

0.0	0.0	0.0	0.0	0.0	0.0	0.0	0.0
0.0	0.0	0.0	0.0	0.0	0.0	0.0	0.0
0.0	0.0	0.0	0.0	0.0	0.0	0.0	0.0
0.0	0.0	0.0	0.0	0.0	0.0	0.0	0.0
0.0	0.0	0.0	0.0	0.0	0.0	0.0	0.0
0.0	0.0	0.0	0.0	0.0	0.0	0.0	0.0
0.0	0.0	0.0	0.0	0.0	0.0	0.0	0.0
0.0	0.0	0.0	0.0	0.0	0.0	0.0	0.0
0.0	0.0	0.0	0.0	0.0	0.0	0.0	0.0
0.0	0.0	0.0	0.0	0.0	0.0	0.0	0.0
0.0	0.0	0.0	0.0	0.0	0.0	0.0	0.0
0.0	0.0	0.0	0.0	0.0	0.0	0.0	0.0
0.0	0.0	0.0	0.0	0.0	0.0	0.0	0.0
0.0	0.0	0.0	0.0	0.0	0.0	0.0	0.0
0.0	0.0	0.0	0.0	0.0	0.0	0.0	0.0
0.0	0.0	0.0	0.0	0.0	0.0	0.0	0.0
0.0	0.0	0.0	0.0	0.0	0.0	0.0	0.0
0.0	0.0	0.0	0.0	0.0	0.0	0.0	0.0
0.0	0.0	0.0	0.0	0.0	0.0	0.0	0.0
0.0	0.0	0.0	0.0	0.0	0.0	0.0	0.0
0.0	0.0	0.0	0.0	0.0	0.0	0.0	0.0
0.0	0.0	0.0	0.0	0.0	0.0	0.0	0.0
0.0	0.0	0.0	0.0	0.0	0.0	0.0	0.0
0.0	0.0	0.0	0.0	0.0	0.0	0.0	0.0
0.0	0.0	0.0	0.0	0.0	0.0	0.0	0.0
0.0	0.0	0.0	0.0	0.0	0.0	0.0	0.0
0.0	0.0	0.0	0.0	0.0	0.0	0.0	0.0
0.0	0.0	0.0	0.0	0.0	0.0	0.0	0.0
0.0	0.0	0.0	0.0	0.0	0.0	0.0	0.0
0.0	0.0	0.0	0.0	0.0	0.0	0.0	0.0
0.0	0.0	0.0	0.0	0.0	0.0	0.0	0.0
0.0	0.0	0.0	0.0	0.0	0.0	0.0	0.0
0.0	0.0	0.0	0.0	0.0	0.0	0.0	0.0
0.0	0.0	0.0	0.0	0.0	0.0	0.0	0.0
0.0	0.0	0.0	0.0	0.0	0.0	0.0	0.0
0.0	0.0	0.0	0.0	0.0	0.0	0.0	0.0
0.0	0.0	0.0	0.0	0.0	0.0	0.0	0.0
0.0	0.0	0.0	0.0	0.0	0.0	0.0	0.0
0.0	0.0	0.0	0.0	0.0	0.0	0.0	0.0
0.0	0.0	0.0	0.0	0.0	0.0	0.0	0.0
0.0	0.0	0.0	0.0	0.0	0.0	0.0	0.0
0.0	0.0	0.0	0.0	0.0	0.0	0.0	0.0
0.0	0.0	0.0	0.0	0.0	0.0	0.0	0.0
0.0	0.0	0.0	0.0	0.0	0.0	0.0	0.0
0.0	0.0	0.0	0.0	0.0	0.0	0.0	0.0
0.0	0.0	0.0	0.0	0.0	0.0	0.0	0.0
0.0	0.0	0.0	0.0	0.0	0.0	0.0	0.0
0.0	0.0	0.0	0.0	0.0	0.0	0.0	0.0

0.0	0.0	0.0	0.0	0.0	0.0	0.0	0.0
0.0	0.0	0.0	0.0	0.0	0.0	0.0	0.0
0.0	0.0	0.0	0.0	0.0	0.0	0.0	0.0
0.0	0.0	0.0	0.0	0.0	0.0	0.0	0.0
0.0	0.0	0.0	0.0	0.0	0.0	0.0	0.0
0.0	0.0	0.0	0.0	0.0	0.0	0.0	0.0
0.0	0.0	0.0	0.0	0.0	0.0	0.0	0.0
0.0	0.0	0.0	0.0	0.0	0.0	0.0	0.0
0.0	0.0	0.0	0.0	0.0	0.0	0.0	0.0
0.0	0.0	0.0	0.0	0.0	0.0	0.0	0.0
0.0	0.0	0.0	0.0	0.0	0.0	0.0	0.0
0.0	0.0	0.0	0.0	0.0	0.0	0.0	0.0
0.0	0.0	0.0	0.0	0.0	0.0	0.0	0.0
0.0	0.0	0.0	0.0	0.0	0.0	0.0	0.0
0.0	0.0	0.0	0.0	0.0	0.0	0.0	0.0
0.0	0.0	0.0	0.0	0.0	0.0	0.0	0.0
0.0	0.0	0.0	0.0	0.0	0.0	0.0	0.0
0.0	0.0	0.0	0.0	0.0	0.0	0.0	0.0
0.0	0.0	0.0	0.0	0.0	0.0	0.0	0.0
0.0	0.0	0.0	0.0	0.0	0.0	0.0	0.0
0.0	0.0	0.0	0.0	0.0	0.0	0.0	0.0
0.0	0.0	0.0	0.0	0.0	0.0	0.0	0.0
0.0	0.0	0.0	0.0	0.0	0.0	0.0	0.0
0.0	0.0	0.0	0.0	0.0	0.0	0.0	0.0
0.0	0.0	0.0	0.0	0.0	0.0	0.0	0.0
0.0	0.0	0.0	0.0	0.0	0.0	0.0	0.0
0.0	0.0	0.0	0.0	0.0	0.0	0.0	0.0
0.0	0.0	0.0	0.0	0.0	0.0	0.0	0.0
0.0	0.0	0.0	0.0	0.0	0.0	0.0	0.0
0.0	0.0	0.0	0.0	0.0	0.0	0.0	0.0
0.0	0.0	0.0	0.0	0.0	0.0	0.0	0.0
0.0	0.0	0.0	0.0	0.0	0.0	0.0	0.0
0.0	0.0	0.0	0.0	0.0	0.0	0.0	0.0
0.0	0.0	0.0	0.0	0.0	0.0	0.0	0.0
0.0	0.0	0.0	0.0	0.0	0.0	0.0	0.0
0.0	0.0	0.0	0.0	0.0	0.0	0.0	0.0
0.0	0.0	0.0	0.0	0.0	0.0	0.0	0.0
0.0	0.0	0.0	0.0	0.0	0.0	0.0	0.0
0.0	0.0	0.0	0.0	0.0	0.0	0.0	0.0
0.0	0.0	0.0	0.0	0.0	0.0	0.0	0.0
0.0	0.0	0.0	0.0	0.0	0.0	0.0	0.0
0.0	0.0	0.0	0.0	0.0	0.0	0.0	0.0
0.0	0.0	0.0	0.0	0.0	0.0	0.0	0.0
0.0	0.0	0.0	0.0	0.0	0.0	0.0	0.0
0.0	0.0	0.0	0.0	0.0	0.0	0.0	0.0
0.0	0.0	0.0	0.0	0.0	0.0	0.0	0.0
0.0	0.0	0.0	0.0	0.0	0.0	0.0	0.0

0.0	0.0	0.0	0.0	0.0	0.0	0.0	0.0
0.0	0.0	0.0	0.0	0.0	0.0	0.0	0.0
0.0	0.0	0.0	0.0	0.0	0.0	0.0	0.0
0.0	0.0	0.0	0.0	0.0	0.0	0.0	0.0
0.0	0.0	0.0	0.0	0.0	0.0	0.0	0.0
0.0	0.0	0.0	0.0	0.0	0.0	0.0	0.0
0.0	0.0	0.0	0.0	0.0	0.0	0.0	0.0
0.0	0.0	0.0	0.0	0.0	0.0	0.0	0.0
0.0	0.0	0.0	0.0	0.0	0.0	0.0	0.0
0.0	0.0	0.0	0.0	0.0	0.0	0.0	0.0
0.0	0.0	0.0	0.0	0.0	0.0	0.0	0.0
0.0	0.0	0.0	0.0	0.0	0.0	0.0	0.0
0.0	0.0	0.0	0.0	0.0	0.0	0.0	0.0
0.0	0.0	0.0	0.0	0.0	0.0	0.0	0.0
0.0	0.0	0.0	0.0	0.0	0.0	0.0	0.0
0.0	0.0	0.0	0.0	0.0	0.0	0.0	0.0
0.0	0.0	0.0	0.0	0.0	0.0	0.0	0.0
0.0	0.0	0.0	0.0	0.0	0.0	0.0	0.0
0.0	0.0	0.0	0.0	0.0	0.0	0.0	0.0
0.0	0.0	0.0	0.0	0.0	0.0	0.0	0.0
0.0	0.0	0.0	0.0	0.0	0.0	0.0	0.0
0.0	0.0	0.0	0.0	0.0	0.0	0.0	0.0
0.0	0.0	0.0	0.0	0.0	0.0	0.0	0.0
0.0	0.0	0.0	0.0	0.0	0.0	0.0	0.0
0.0	0.0	0.0	0.0	0.0	0.0	0.0	0.0
0.0	0.0	0.0	0.0	0.0	0.0	0.0	0.0
0.0	0.0	0.0	0.0	0.0	0.0	0.0	0.0
0.0	0.0	0.0	0.0	0.0	0.0	0.0	0.0
0.0	0.0	0.0	0.0	0.0	0.0	0.0	0.0
0.0	0.0	0.0	0.0	0.0	0.0	0.0	0.0
0.0	0.0	0.0	0.0	0.0	0.0	0.0	0.0
0.0	0.0	0.0	0.0	0.0	0.0	0.0	0.0
0.0	0.0	0.0	0.0	0.0	0.0	0.0	0.0
0.0	0.0	0.0	0.0	0.0	0.0	0.0	0.0
0.0	0.0	0.0	0.0	0.0	0.0	0.0	0.0
0.0	0.0	0.0	0.0	0.0	0.0	0.0	0.0
0.0	0.0	0.0	0.0	0.0	0.0	0.0	0.0
0.0	0.0	0.0	0.0	0.0	0.0	0.0	0.0
0.0	0.0	0.0	0.0	0.0	0.0	0.0	0.0
0.0	0.0	0.0	0.0	0.0	0.0	0.0	0.0
0.0	0.0	0.0	0.0	0.0	0.0	0.0	0.0
0.0	0.0	0.0	0.0	0.0	0.0	0.0	0.0
0.0	0.0	0.0	0.0	0.0	0.0	0.0	0.0
0.0	0.0	0.0	0.0	0.0	0.0	0.0	0.0
0.0	0.0	0.0	0.0	0.0	0.0	0.0	0.0
0.0	0.0	0.0	0.0	0.0	0.0	0.0	0.0
0.0	0.0	0.0	0.0	0.0	0.0	0.0	0.0
0.0	0.0	0.0	0.0	0.0	0.0	0.0	0.0

0.0	0.0	0.0	0.0	0.0	0.0	0.0	0.0
0.0	0.0	0.0	0.0	0.0	0.0	0.0	0.0
0.0	0.0	0.0	0.0	0.0	0.0	0.0	0.0
0.0	0.0	0.0	0.0	0.0	0.0	0.0	0.0
0.0	0.0	0.0	0.0	0.0	0.0	0.0	0.0
0.0	0.0	0.0	0.0	0.0	0.0	0.0	0.0
0.0	0.0	0.0	0.0	0.0	0.0	0.0	0.0
0.0	0.0	0.0	0.0	0.0	0.0	0.0	0.0
0.0	0.0	0.0	0.0	0.0	0.0	0.0	0.0
0.0	0.0	0.0	0.0	0.0	0.0	0.0	0.0
0.0	0.0	0.0	0.0	0.0	0.0	0.0	0.0
0.0	0.0	0.0	0.0	0.0	0.0	0.0	0.0
0.0	0.0	0.0	0.0	0.0	0.0	0.0	0.0
0.0	0.0	0.0	0.0	0.0	0.0	0.0	0.0
0.0	0.0	0.0	0.0	0.0	0.0	0.0	0.0
0.0	0.0	0.0	0.0	0.0	0.0	0.0	0.0
0.0	0.0	0.0	0.0	0.0	0.0	0.0	0.0
0.0	0.0	0.0	0.0	0.0	0.0	0.0	0.0
0.0	0.0	0.0	0.0	0.0	0.0	0.0	0.0
0.0	0.0	0.0	0.0	0.0	0.0	0.0	0.0
0.0	0.0	0.0	0.0	0.0	0.0	0.0	0.0
0.0	0.0	0.0	0.0	0.0	0.0	0.0	0.0
0.0	0.0	0.0	0.0	0.0	0.0	0.0	0.0
0.0	0.0	0.0	0.0	0.0	0.0	0.0	0.0
0.0	0.0	0.0	0.0	0.0	0.0	0.0	0.0
0.0	0.0	0.0	0.0	0.0	0.0	0.0	0.0
0.0	0.0	0.0	0.0	0.0	0.0	0.0	0.0
0.0	0.0	0.0	0.0	0.0	0.0	0.0	0.0
0.0	0.0	0.0	0.0	0.0	0.0	0.0	0.0
0.0	0.0	0.0	0.0	0.0	0.0	0.0	0.0
0.0	0.0	0.0	0.0	0.0	0.0	0.0	0.0
0.0	0.0	0.0	0.0	0.0	0.0	0.0	0.0
0.0	0.0	0.0	0.0	0.0	0.0	0.0	0.0
0.0	0.0	0.0	0.0	0.0	0.0	0.0	0.0
0.0	0.0	0.0	0.0	0.0	0.0	0.0	0.0
0.0	0.0	0.0	0.0	0.0	0.0	0.0	0.0
0.0	0.0	0.0	0.0	0.0	0.0	0.0	0.0
0.0	0.0	0.0	0.0	0.0	0.0	0.0	0.0
0.0	0.0	0.0	0.0	0.0	0.0	0.0	0.0
0.0	0.0	0.0	0.0	0.0	0.0	0.0	0.0
0.0	0.0	0.0	0.0	0.0	0.0	0.0	0.0
0.0	0.0	0.0	0.0	0.0	0.0	0.0	0.0
0.0	0.0	0.0	0.0	0.0	0.0	0.0	0.0
0.0	0.0	0.0	0.0	0.0	0.0	0.0	0.0
0.0	0.0	0.0	0.0	0.0	0.0	0.0	0.0
0.0	0.0	0.0	0.0	0.0	0.0	0.0	0.0
0.0	0.0	0.0	0.0	0.0	0.0	0.0	0.0

0.0	0.0	0.0	0.0	0.0	0.0	0.0	0.0
0.0	0.0	0.0	0.0	0.0	0.0	0.0	0.0
0.0	0.0	0.0	0.0	0.0	0.0	0.0	0.0
0.0	0.0	0.0	0.0	0.0	0.0	0.0	0.0
0.0	0.0	0.0	0.0	0.0	0.0	0.0	0.0
0.0	0.0	0.0	0.0	0.0	0.0	0.0	0.0
0.0	0.0	0.0	0.0	0.0	0.0	0.0	0.0
0.0	0.0	0.0	0.0	0.0	0.0	0.0	0.0
0.0	0.0	0.0	0.0	0.0	0.0	0.0	0.0
0.0	0.0	0.0	0.0	0.0	0.0	0.0	0.0
0.0	0.0	0.0	0.0	0.0	0.0	0.0	0.0
0.0	0.0	0.0	0.0	0.0	0.0	0.0	0.0
0.0	0.0	0.0	0.0	0.0	0.0	0.0	0.0
0.0	0.0	0.0	0.0	0.0	0.0	0.0	0.0
0.0	0.0	0.0	0.0	0.0	0.0	0.0	0.0
0.0	0.0	0.0	0.0	0.0	0.0	0.0	0.0
0.0	0.0	0.0	0.0	0.0	0.0	0.0	0.0
0.0	0.0	0.0	0.0	0.0	0.0	0.0	0.0
0.0	0.0	0.0	0.0	0.0	0.0	0.0	0.0
0.0	0.0	0.0	0.0	0.0	0.0	0.0	0.0
0.0	0.0	0.0	0.0	0.0	0.0	0.0	0.0
0.0	0.0	0.0	0.0	0.0	0.0	0.0	0.0
0.0	0.0	0.0	0.0	0.0	0.0	0.0	0.0
0.0	0.0	0.0	0.0	0.0	0.0	0.0	0.0
0.0	0.0	0.0	0.0	0.0	0.0	0.0	0.0
0.0	0.0	0.0	0.0	0.0	0.0	0.0	0.0
0.0	0.0	0.0	0.0	0.0	0.0	0.0	0.0
0.0	0.0	0.0	0.0	0.0	0.0	0.0	0.0
0.0	0.0	0.0	0.0	0.0	0.0	0.0	0.0
0.0	0.0	0.0	0.0	0.0	0.0	0.0	0.0
0.0	0.0	0.0	0.0	0.0	0.0	0.0	0.0
0.0	0.0	0.0	0.0	0.0	0.0	0.0	0.0
0.0	0.0	0.0	0.0	0.0	0.0	0.0	0.0
0.0	0.0	0.0	0.0	0.0	0.0	0.0	0.0
0.0	0.0	0.0	0.0	0.0	0.0	0.0	0.0
0.0	0.0	0.0	0.0	0.0	0.0	0.0	0.0
0.0	0.0	0.0	0.0	0.0	0.0	0.0	0.0
0.0	0.0	0.0	0.0	0.0	0.0	0.0	0.0
0.0	0.0	0.0	0.0	0.0	0.0	0.0	0.0
0.0	0.0	0.0	0.0	0.0	0.0	0.0	0.0
0.0	0.0	0.0	0.0	0.0	0.0	0.0	0.0
0.0	0.0	0.0	0.0	0.0	0.0	0.0	0.0
0.0	0.0	0.0	0.0	0.0	0.0	0.0	0.0
0.0	0.0	0.0	0.0	0.0	0.0	0.0	0.0
0.0	0.0	0.0	0.0	0.0	0.0	0.0	0.0
0.0	0.0	0.0	0.0	0.0	0.0	0.0	0.0
0.0	0.0	0.0	0.0	0.0	0.0	0.0	0.0
0.0	0.0	0.0	0.0	0.0	0.0	0.0	0.0

0.0	0.0	0.0	0.0	0.0	0.0	0.0	0.0
0.0	0.0	0.0	0.0	0.0	0.0	0.0	0.0
0.0	0.0	0.0	0.0	0.0	0.0	0.0	0.0
0.0	0.0	0.0	0.0	0.0	0.0	0.0	0.0
0.0	0.0	0.0	0.0	0.0	0.0	0.0	0.0
0.0	0.0	0.0	0.0	0.0	0.0	0.0	0.0
0.0	0.0	0.0	0.0	0.0	0.0	0.0	0.0
0.0	0.0	0.0	0.0	0.0	0.0	0.0	0.0
0.0	0.0	0.0	0.0	0.0	0.0	0.0	0.0
0.0	0.0	0.0	0.0	0.0	0.0	0.0	0.0
0.0	0.0	0.0	0.0	0.0	0.0	0.0	0.0
0.0	0.0	0.0	0.0	0.0	0.0	0.0	0.0
0.0	0.0	0.0	0.0	0.0	0.0	0.0	0.0
0.0	0.0	0.0	0.0	0.0	0.0	0.0	0.0
0.0	0.0	0.0	0.0	0.0	0.0	0.0	0.0
0.0	0.0	0.0	0.0	0.0	0.0	0.0	0.0
0.0	0.0	0.0	0.0	0.0	0.0	0.0	0.0
0.0	0.0	0.0	0.0	0.0	0.0	0.0	0.0
0.0	0.0	0.0	0.0	0.0	0.0	0.0	0.0
0.0	0.0	0.0	0.0	0.0	0.0	0.0	0.0
0.0	0.0	0.0	0.0	0.0	0.0	0.0	0.0
0.0	0.0	0.0	0.0	0.0	0.0	0.0	0.0
0.0	0.0	0.0	0.0	0.0	0.0	0.0	0.0
0.0	0.0	0.0	0.0	0.0	0.0	0.0	0.0
0.0	0.0	0.0	0.0	0.0	0.0	0.0	0.0
0.0	0.0	0.0	0.0	0.0	0.0	0.0	0.0
0.0	0.0	0.0	0.0	0.0	0.0	0.0	0.0
0.0	0.0	0.0	0.0	0.0	0.0	0.0	0.0
0.0	0.0	0.0	0.0	0.0	0.0	0.0	0.0
0.0	0.0	0.0	0.0	0.0	0.0	0.0	0.0
0.0	0.0	0.0	0.0	0.0	0.0	0.0	0.0
0.0	0.0	0.0	0.0	0.0	0.0	0.0	0.0
0.0	0.0	0.0	0.0	0.0	0.0	0.0	0.0
0.0	0.0	0.0	0.0	0.0	0.0	0.0	0.0
0.0	0.0	0.0	0.0	0.0	0.0	0.0	0.0
0.0	0.0	0.0	0.0	0.0	0.0	0.0	0.0
0.0	0.0	0.0	0.0	0.0	0.0	0.0	0.0
0.0	0.0	0.0	0.0	0.0	0.0	0.0	0.0
0.0	0.0	0.0	0.0	0.0	0.0	0.0	0.0
0.0	0.0	0.0	0.0	0.0	0.0	0.0	0.0
0.0	0.0	0.0	0.0	0.0	0.0	0.0	0.0
0.0	0.0	0.0	0.0	0.0	0.0	0.0	0.0
0.0	0.0	0.0	0.0	0.0	0.0	0.0	0.0
0.0	0.0	0.0	0.0	0.0	0.0	0.0	0.0
0.0	0.0	0.0	0.0	0.0	0.0	0.0	0.0
0.0	0.0	0.0	0.0	0.0	0.0	0.0	0.0
0.0	0.0	0.0	0.0	0.0	0.0	0.0	0.0
0.0	0.0	0.0	0.0	0.0	0.0	0.0	0.0

0.0	0.0	0.0	0.0	0.0	0.0	0.0	0.0
0.0	0.0	0.0	0.0	0.0	0.0	0.0	0.0
0.0	0.0	0.0	0.0	0.0	0.0	0.0	0.0
0.0	0.0	0.0	0.0	0.0	0.0	0.0	0.0
0.0	0.0	0.0	0.0	0.0	0.0	0.0	0.0
0.0	0.0	0.0	0.0	0.0	0.0	0.0	0.0
0.0	0.0	0.0	0.0	0.0	0.0	0.0	0.0
0.0	0.0	0.0	0.0	0.0	0.0	0.0	0.0
0.0	0.0	0.0	0.0	0.0	0.0	0.0	0.0
0.0	0.0	0.0	0.0	0.0	0.0	0.0	0.0
0.0	0.0	0.0	0.0	0.0	0.0	0.0	0.0
0.0	0.0	0.0	0.0	0.0	0.0	0.0	0.0
0.0	0.0	0.0	0.0	0.0	0.0	0.0	0.0
0.0	0.0	0.0	0.0	0.0	0.0	0.0	0.0
0.0	0.0	0.0	0.0	0.0	0.0	0.0	0.0
0.0	0.0	0.0	0.0	0.0	0.0	0.0	0.0
0.0	0.0	0.0	0.0	0.0	0.0	0.0	0.0
0.0	0.0	0.0	0.0	0.0	0.0	0.0	0.0
0.0	0.0	0.0	0.0	0.0	0.0	0.0	0.0
0.0	0.0	0.0	0.0	0.0	0.0	0.0	0.0
0.0	0.0	0.0	0.0	0.0	0.0	0.0	0.0
0.0	0.0	0.0	0.0	0.0	0.0	0.0	0.0
0.0	0.0	0.0	0.0	0.0	0.0	0.0	0.0
0.0	0.0	0.0	0.0	0.0	0.0	0.0	0.0
0.0	0.0	0.0	0.0	0.0	0.0	0.0	0.0
0.0	0.0	0.0	0.0	0.0	0.0	0.0	0.0
0.0	0.0	0.0	0.0	0.0	0.0	0.0	0.0
0.0	0.0	0.0	0.0	0.0	0.0	0.0	0.0
0.0	0.0	0.0	0.0	0.0	0.0	0.0	0.0
0.0	0.0	0.0	0.0	0.0	0.0	0.0	0.0
0.0	0.0	0.0	0.0	0.0	0.0	0.0	0.0
0.0	0.0	0.0	0.0	0.0	0.0	0.0	0.0
0.0	0.0	0.0	0.0	0.0	0.0	0.0	0.0
0.0	0.0	0.0	0.0	0.0	0.0	0.0	0.0
0.0	0.0	0.0	0.0	0.0	0.0	0.0	0.0
0.0	0.0	0.0	0.0	0.0	0.0	0.0	0.0
0.0	0.0	0.0	0.0	0.0	0.0	0.0	0.0
0.0	0.0	0.0	0.0	0.0	0.0	0.0	0.0
0.0	0.0	0.0	0.0	0.0	0.0	0.0	0.0
0.0	0.0	0.0	0.0	0.0	0.0	0.0	0.0
0.0	0.0	0.0	0.0	0.0	0.0	0.0	0.0
0.0	0.0	0.0	0.0	0.0	0.0	0.0	0.0
0.0	0.0	0.0	0.0	0.0	0.0	0.0	0.0
0.0	0.0	0.0	0.0	0.0	0.0	0.0	0.0
0.0	0.0	0.0	0.0	0.0	0.0	0.0	0.0
0.0	0.0	0.0	0.0	0.0	0.0	0.0	0.0
0.0	0.0	0.0	0.0	0.0	0.0	0.0	0.0

0.0	0.0	0.0	0.0	0.0	0.0	0.0	0.0
0.0	0.0	0.0	0.0	0.0	0.0	0.0	0.0
0.0	0.0	0.0	0.0	0.0	0.0	0.0	0.0
0.0	0.0	0.0	0.0	0.0	0.0	0.0	0.0
0.0	0.0	0.0	0.0	0.0	0.0	0.0	0.0
0.0	0.0	0.0	0.0	0.0	0.0	0.0	0.0
0.0	0.0	0.0	0.0	0.0	0.0	0.0	0.0
0.0	0.0	0.0	0.0	0.0	0.0	0.0	0.0
0.0	0.0	0.0	0.0	0.0	0.0	0.0	0.0
0.0	0.0	0.0	0.0	0.0	0.0	0.0	0.0
0.0	0.0	0.0	0.0	0.0	0.0	0.0	0.0
0.0	0.0	0.0	0.0	0.0	0.0	0.0	0.0
0.0	0.0	0.0	0.0	0.0	0.0	0.0	0.0
0.0	0.0	0.0	0.0	0.0	0.0	0.0	0.0
0.0	0.0	0.0	0.0	0.0	0.0	0.0	0.0
0.0	0.0	0.0	0.0	0.0	0.0	0.0	0.0
0.0	0.0	0.0	0.0	0.0	0.0	0.0	0.0
0.0	0.0	0.0	0.0	0.0	0.0	0.0	0.0
0.0	0.0	0.0	0.0	0.0	0.0	0.0	0.0
0.0	0.0	0.0	0.0	0.0	0.0	0.0	0.0
0.0	0.0	0.0	0.0	0.0	0.0	0.0	0.0
0.0	0.0	0.0	0.0	0.0	0.0	0.0	0.0
0.0	0.0	0.0	0.0	0.0	0.0	0.0	0.0
0.0	0.0	0.0	0.0	0.0	0.0	0.0	0.0
0.0	0.0	0.0	0.0	0.0	0.0	0.0	0.0
0.0	0.0	0.0	0.0	0.0	0.0	0.0	0.0
0.0	0.0	0.0	0.0	0.0	0.0	0.0	0.0
0.0	0.0	0.0	0.0	0.0	0.0	0.0	0.0
0.0	0.0	0.0	0.0	0.0	0.0	0.0	0.0
0.0	0.0	0.0	0.0	0.0	0.0	0.0	0.0
0.0	0.0	0.0	0.0	0.0	0.0	0.0	0.0
0.0	0.0	0.0	0.0	0.0	0.0	0.0	0.0
0.0	0.0	0.0	0.0	0.0	0.0	0.0	0.0
0.0	0.0	0.0	0.0	0.0	0.0	0.0	0.0
0.0	0.0	0.0	0.0	0.0	0.0	0.0	0.0
0.0	0.0	0.0	0.0	0.0	0.0	0.0	0.0
0.0	0.0	0.0	0.0	0.0	0.0	0.0	0.0
0.0	0.0	0.0	0.0	0.0	0.0	0.0	0.0
0.0	0.0	0.0	0.0	0.0	0.0	0.0	0.0
0.0	0.0	0.0	0.0	0.0	0.0	0.0	0.0
0.0	0.0	0.0	0.0	0.0	0.0	0.0	0.0
0.0	0.0	0.0	0.0	0.0	0.0	0.0	0.0
0.0	0.0	0.0	0.0	0.0	0.0	0.0	0.0
0.0	0.0	0.0	0.0	0.0	0.0	0.0	0.0
0.0	0.0	0.0	0.0	0.0	0.0	0.0	0.0
0.0	0.0	0.0	0.0	0.0	0.0	0.0	0.0
0.0	0.0	0.0	0.0	0.0	0.0	0.0	0.0

0.0	0.0	0.0	0.0	0.0	0.0	0.0	0.0
0.0	0.0	0.0	0.0	0.0	0.0	0.0	0.0
0.0	0.0	0.0	0.0	0.0	0.0	0.0	0.0
0.0	0.0	0.0	0.0	0.0	0.0	0.0	0.0
0.0	0.0	0.0	0.0	0.0	0.0	0.0	0.0
0.0	0.0	0.0	0.0	0.0	0.0	0.0	0.0
0.0	0.0	0.0	0.0	0.0	0.0	0.0	0.0
0.0	0.0	0.0	0.0	0.0	0.0	0.0	0.0
0.0	0.0	0.0	0.0	0.0	0.0	0.0	0.0
0.0	0.0	0.0	0.0	0.0	0.0	0.0	0.0
0.0	0.0	0.0	0.0	0.0	0.0	0.0	0.0
0.0	0.0	0.0	0.0	0.0	0.0	0.0	0.0
0.0	0.0	0.0	0.0	0.0	0.0	0.0	0.0
0.0	0.0	0.0	0.0	0.0	0.0	0.0	0.0
0.0	0.0	0.0	0.0	0.0	0.0	0.0	0.0
0.0	0.0	0.0	0.0	0.0	0.0	0.0	0.0
0.0	0.0	0.0	0.0	0.0	0.0	0.0	0.0
0.0	0.0	0.0	0.0	0.0	0.0	0.0	0.0
0.0	0.0	0.0	0.0	0.0	0.0	0.0	0.0
0.0	0.0	0.0	0.0	0.0	0.0	0.0	0.0
0.0	0.0	0.0	0.0	0.0	0.0	0.0	0.0
0.0	0.0	0.0	0.0	0.0	0.0	0.0	0.0
0.0	0.0	0.0	0.0	0.0	0.0	0.0	0.0
0.0	0.0	0.0	0.0	0.0	0.0	0.0	0.0
0.0	0.0	0.0	0.0	0.0	0.0	0.0	0.0
0.0	0.0	0.0	0.0	0.0	0.0	0.0	0.0
0.0	0.0	0.0	0.0	0.0	0.0	0.0	0.0
0.0	0.0	0.0	0.0	0.0	0.0	0.0	0.0
0.0	0.0	0.0	0.0	0.0	0.0	0.0	0.0
0.0	0.0	0.0	0.0	0.0	0.0	0.0	0.0
0.0	0.0	0.0	0.0	0.0	0.0	0.0	0.0
0.0	0.0	0.0	0.0	0.0	0.0	0.0	0.0
0.0	0.0	0.0	0.0	0.0	0.0	0.0	0.0
0.0	0.0	0.0	0.0	0.0	0.0	0.0	0.0
0.0	0.0	0.0	0.0	0.0	0.0	0.0	0.0
0.0	0.0	0.0	0.0	0.0	0.0	0.0	0.0
0.0	0.0	0.0	0.0	0.0	0.0	0.0	0.0
0.0	0.0	0.0	0.0	0.0	0.0	0.0	0.0
0.0	0.0	0.0	0.0	0.0	0.0	0.0	0.0
0.0	0.0	0.0	0.0	0.0	0.0	0.0	0.0
0.0	0.0	0.0	0.0	0.0	0.0	0.0	0.0
0.0	0.0	0.0	0.0	0.0	0.0	0.0	0.0
0.0	0.0	0.0	0.0	0.0	0.0	0.0	0.0
0.0	0.0	0.0	0.0	0.0	0.0	0.0	0.0
0.0	0.0	0.0	0.0	0.0	0.0	0.0	0.0
0.0	0.0	0.0	0.0	0.0	0.0	0.0	0.0
0.0	0.0	0.0	0.0	0.0	0.0	0.0	0.0
0.0	0.0	0.0	0.0	0.0	0.0	0.0	0.0

0.0	0.0	0.0	0.0	0.0	0.0	0.0	0.0
0.0	0.0	0.0	0.0	0.0	0.0	0.0	0.0
0.0	0.0	0.0	0.0	0.0	0.0	0.0	0.0
0.0	0.0	0.0	0.0	0.0	0.0	0.0	0.0
0.0	0.0	0.0	0.0	0.0	0.0	0.0	0.0
0.0	0.0	0.0	0.0	0.0	0.0	0.0	0.0
0.0	0.0	0.0	0.0	0.0	0.0	0.0	0.0
0.0	0.0	0.0	0.0	0.0	0.0	0.0	0.0
0.0	0.0	0.0	0.0	0.0	0.0	0.0	0.0
0.0	0.0	0.0	0.0	0.0	0.0	0.0	0.0
0.0	0.0	0.0	0.0	0.0	0.0	0.0	0.0
0.0	0.0	0.0	0.0	0.0	0.0	0.0	0.0
0.0	0.0	0.0	0.0	0.0	0.0	0.0	0.0
0.0	0.0	0.0	0.0	0.0	0.0	0.0	0.0
0.0	0.0	0.0	0.0	0.0	0.0	0.0	0.0
0.0	0.0	0.0	0.0	0.0	0.0	0.0	0.0
0.0	0.0	0.0	0.0	0.0	0.0	0.0	0.0
0.0	0.0	0.0	0.0	0.0	0.0	0.0	0.0
0.0	0.0	0.0	0.0	0.0	0.0	0.0	0.0
0.0	0.0	0.0	0.0	0.0	0.0	0.0	0.0
0.0	0.0	0.0	0.0	0.0	0.0	0.0	0.0
0.0	0.0	0.0	0.0	0.0	0.0	0.0	0.0
0.0	0.0	0.0	0.0	0.0	0.0	0.0	0.0
0.0	0.0	0.0	0.0	0.0	0.0	0.0	0.0
0.0	0.0	0.0	0.0	0.0	0.0	0.0	0.0
0.0	0.0	0.0	0.0	0.0	0.0	0.0	0.0
0.0	0.0	0.0	0.0	0.0	0.0	0.0	0.0
0.0	0.0	0.0	0.0	0.0	0.0	0.0	0.0
0.0	0.0	0.0	0.0	0.0	0.0	0.0	0.0
0.0	0.0	0.0	0.0	0.0	0.0	0.0	0.0
0.0	0.0	0.0	0.0	0.0	0.0	0.0	0.0
0.0	0.0	0.0	0.0	0.0	0.0	0.0	0.0
0.0	0.0	0.0	0.0	0.0	0.0	0.0	0.0
0.0	0.0	0.0	0.0	0.0	0.0	0.0	0.0
0.0	0.0	0.0	0.0	0.0	0.0	0.0	0.0
0.0	0.0	0.0	0.0	0.0	0.0	0.0	0.0
0.0	0.0	0.0	0.0	0.0	0.0	0.0	0.0
0.0	0.0	0.0	0.0	0.0	0.0	0.0	0.0
0.0	0.0	0.0	0.0	0.0	0.0	0.0	0.0
0.0	0.0	0.0	0.0	0.0	0.0	0.0	0.0
0.0	0.0	0.0	0.0	0.0	0.0	0.0	0.0
0.0	0.0	0.0	0.0	0.0	0.0	0.0	0.0
0.0	0.0	0.0	0.0	0.0	0.0	0.0	0.0
0.0	0.0	0.0	0.0	0.0	0.0	0.0	0.0
0.0	0.0	0.0	0.0	0.0	0.0	0.0	0.0
0.0	0.0	0.0	0.0	0.0	0.0	0.0	0.0
0.0	0.0	0.0	0.0	0.0	0.0	0.0	0.0
0.0	0.0	0.0	0.0	0.0	0.0	0.0	0.0

0.0	0.0	0.0	0.0	0.0	0.0	0.0	0.0
0.0	0.0	0.0	0.0	0.0	0.0	0.0	0.0
0.0	0.0	0.0	0.0	0.0	0.0	0.0	0.0
0.0	0.0	0.0	0.0	0.0	0.0	0.0	0.0
0.0	0.0	0.0	0.0	0.0	0.0	0.0	0.0
0.0	0.0	0.0	0.0	0.0	0.0	0.0	0.0
0.0	0.0	0.0	0.0	0.0	0.0	0.0	0.0
0.0	0.0	0.0	0.0	0.0	0.0	0.0	0.0
0.0	0.0	0.0	0.0	0.0	0.0	0.0	0.0
0.0	0.0	0.0	0.0	0.0	0.0	0.0	0.0
0.0	0.0	0.0	0.0	0.0	0.0	0.0	0.0
0.0	0.0	0.0	0.0	0.0	0.0	0.0	0.0
0.0	0.0	0.0	0.0	0.0	0.0	0.0	0.0
0.0	0.0	0.0	0.0	0.0	0.0	0.0	0.0
0.0	0.0	0.0	0.0	0.0	0.0	0.0	0.0
0.0	0.0	0.0	0.0	0.0	0.0	0.0	0.0
0.0	0.0	0.0	0.0	0.0	0.0	0.0	0.0
0.0	0.0	0.0	0.0	0.0	0.0	0.0	0.0
0.0	0.0	0.0	0.0	0.0	0.0	0.0	0.0
0.0	0.0	0.0	0.0	0.0	0.0	0.0	0.0
0.0	0.0	0.0	0.0	0.0	0.0	0.0	0.0
0.0	0.0	0.0	0.0	0.0	0.0	0.0	0.0
0.0	0.0	0.0	0.0	0.0	0.0	0.0	0.0
0.0	0.0	0.0	0.0	0.0	0.0	0.0	0.0
0.0	0.0	0.0	0.0	0.0	0.0	0.0	0.0
0.0	0.0	0.0	0.0	0.0	0.0	0.0	0.0
0.0	0.0	0.0	0.0	0.0	0.0	0.0	0.0
0.0	0.0	0.0	0.0	0.0	0.0	0.0	0.0
0.0	0.0	0.0	0.0	0.0	0.0	0.0	0.0
0.0	0.0	0.0	0.0	0.0	0.0	0.0	0.0
0.0	0.0	0.0	0.0	0.0	0.0	0.0	0.0
0.0	0.0	0.0	0.0	0.0	0.0	0.0	0.0
0.0	0.0	0.0	0.0	0.0	0.0	0.0	0.0
0.0	0.0	0.0	0.0	0.0	0.0	0.0	0.0
0.0	0.0	0.0	0.0	0.0	0.0	0.0	0.0
0.0	0.0	0.0	0.0	0.0	0.0	0.0	0.0
0.0	0.0	0.0	0.0	0.0	0.0	0.0	0.0
0.0	0.0	0.0	0.0	0.0	0.0	0.0	0.0
0.0	0.0	0.0	0.0	0.0	0.0	0.0	0.0
0.0	0.0	0.0	0.0	0.0	0.0	0.0	0.0
0.0	0.0	0.0	0.0	0.0	0.0	0.0	0.0
0.0	0.0	0.0	0.0	0.0	0.0	0.0	0.0
0.0	0.0	0.0	0.0	0.0	0.0	0.0	0.0
0.0	0.0	0.0	0.0	0.0	0.0	0.0	0.0
0.0	0.0	0.0	0.0	0.0	0.0	0.0	0.0
0.0	0.0	0.0	0.0	0.0	0.0	0.0	0.0
0.0	0.0	0.0	0.0	0.0	0.0	0.0	0.0

0.0	0.0	0.0	0.0	0.0	0.0	0.0	0.0
0.0	0.0	0.0	0.0	0.0	0.0	0.0	0.0
0.0	0.0	0.0	0.0	0.0	0.0	0.0	0.0
0.0	0.0	0.0	0.0	0.0	0.0	0.0	0.0
0.0	0.0	0.0	0.0	0.0	0.0	0.0	0.0
0.0	0.0	0.0	0.0	0.0	0.0	0.0	0.0
0.0	0.0	0.0	0.0	0.0	0.0	0.0	0.0
0.0	0.0	0.0	0.0	0.0	0.0	0.0	0.0
0.0	0.0	0.0	0.0	0.0	0.0	0.0	0.0
0.0	0.0	0.0	0.0	0.0	0.0	0.0	0.0
0.0	0.0	0.0	0.0	0.0	0.0	0.0	0.0
0.0	0.0	0.0	0.0	0.0	0.0	0.0	0.0
0.0	0.0	0.0	0.0	0.0	0.0	0.0	0.0
0.0	0.0	0.0	0.0	0.0	0.0	0.0	0.0
0.0	0.0	0.0	0.0	0.0	0.0	0.0	0.0
0.0	0.0	0.0	0.0	0.0	0.0	0.0	0.0
0.0	0.0	0.0	0.0	0.0	0.0	0.0	0.0
0.0	0.0	0.0	0.0	0.0	0.0	0.0	0.0
0.0	0.0	0.0	0.0	0.0	0.0	0.0	0.0
0.0	0.0	0.0	0.0	0.0	0.0	0.0	0.0
0.0	0.0	0.0	0.0	0.0	0.0	0.0	0.0
0.0	0.0	0.0	0.0	0.0	0.0	0.0	0.0
0.0	0.0	0.0	0.0	0.0	0.0	0.0	0.0
0.0	0.0	0.0	0.0	0.0	0.0	0.0	0.0
0.0	0.0	0.0	0.0	0.0	0.0	0.0	0.0
0.0	0.0	0.0	0.0	0.0	0.0	0.0	0.0
0.0	0.0	0.0	0.0	0.0	0.0	0.0	0.0
0.0	0.0	0.0	0.0	0.0	0.0	0.0	0.0
0.0	0.0	0.0	0.0	0.0	0.0	0.0	0.0
0.0	0.0	0.0	0.0	0.0	0.0	0.0	0.0
0.0	0.0	0.0	0.0	0.0	0.0	0.0	0.0
0.0	0.0	0.0	0.0	0.0	0.0	0.0	0.0
0.0	0.0	0.0	0.0	0.0	0.0	0.0	0.0
0.0	0.0	0.0	0.0	0.0	0.0	0.0	0.0
0.0	0.0	0.0	0.0	0.0	0.0	0.0	0.0
0.0	0.0	0.0	0.0	0.0	0.0	0.0	0.0
0.0	0.0	0.0	0.0	0.0	0.0	0.0	0.0
0.0	0.0	0.0	0.0	0.0	0.0	0.0	0.0
0.0	0.0	0.0	0.0	0.0	0.0	0.0	0.0
0.0	0.0	0.0	0.0	0.0	0.0	0.0	0.0
0.0	0.0	0.0	0.0	0.0	0.0	0.0	0.0
0.0	0.0	0.0	0.0	0.0	0.0	0.0	0.0
0.0	0.0	0.0	0.0	0.0	0.0	0.0	0.0
0.0	0.0	0.0	0.0	0.0	0.0	0.0	0.0
0.0	0.0	0.0	0.0	0.0	0.0	0.0	0.0
0.0	0.0	0.0	0.0	0.0	0.0	0.0	0.0
0.0	0.0	0.0	0.0	0.0	0.0	0.0	0.0
0.0	0.0	0.0	0.0	0.0	0.0	0.0	0.0

0.0	0.0	0.0	0.0	0.0	0.0	0.0	0.0
0.0	0.0	0.0	0.0	0.0	0.0	0.0	0.0
0.0	0.0	0.0	0.0	0.0	0.0	0.0	0.0
0.0	0.0	0.0	0.0	0.0	0.0	0.0	0.0
0.0	0.0	0.0	0.0	0.0	0.0	0.0	0.0
0.0	0.0	0.0	0.0	0.0	0.0	0.0	0.0
0.0	0.0	0.0	0.0	0.0	0.0	0.0	0.0
0.0	0.0	0.0	0.0	0.0	0.0	0.0	0.0
0.0	0.0	0.0	0.0	0.0	0.0	0.0	0.0
0.0	0.0	0.0	0.0	0.0	0.0	0.0	0.0
0.0	0.0	0.0	0.0	0.0	0.0	0.0	0.0
0.0	0.0	0.0	0.0	0.0	0.0	0.0	0.0
0.0	0.0	0.0	0.0	0.0	0.0	0.0	0.0
0.0	0.0	0.0	0.0	0.0	0.0	0.0	0.0
0.0	0.0	0.0	0.0	0.0	0.0	0.0	0.0
0.0	0.0	0.0	0.0	0.0	0.0	0.0	0.0
0.0	0.0	0.0	0.0	0.0	0.0	0.0	0.0
0.0	0.0	0.0	0.0	0.0	0.0	0.0	0.0
0.0	0.0	0.0	0.0	0.0	0.0	0.0	0.0
0.0	0.0	0.0	0.0	0.0	0.0	0.0	0.0
0.0	0.0	0.0	0.0	0.0	0.0	0.0	0.0
0.0	0.0	0.0	0.0	0.0	0.0	0.0	0.0
0.0	0.0	0.0	0.0	0.0	0.0	0.0	0.0
0.0	0.0	0.0	0.0	0.0	0.0	0.0	0.0
0.0	0.0	0.0	0.0	0.0	0.0	0.0	0.0
0.0	0.0	0.0	0.0	0.0	0.0	0.0	0.0
0.0	0.0	0.0	0.0	0.0	0.0	0.0	0.0
0.0	0.0	0.0	0.0	0.0	0.0	0.0	0.0
0.0	0.0	0.0	0.0	0.0	0.0	0.0	0.0
0.0	0.0	0.0	0.0	0.0	0.0	0.0	0.0
0.0	0.0	0.0	0.0	0.0	0.0	0.0	0.0
0.0	0.0	0.0	0.0	0.0	0.0	0.0	0.0
0.0	0.0	0.0	0.0	0.0	0.0	0.0	0.0
0.0	0.0	0.0	0.0	0.0	0.0	0.0	0.0
0.0	0.0	0.0	0.0	0.0	0.0	0.0	0.0
0.0	0.0	0.0	0.0	0.0	0.0	0.0	0.0
0.0	0.0	0.0	0.0	0.0	0.0	0.0	0.0
0.0	0.0	0.0	0.0	0.0	0.0	0.0	0.0
0.0	0.0	0.0	0.0	0.0	0.0	0.0	0.0
0.0	0.0	0.0	0.0	0.0	0.0	0.0	0.0
0.0	0.0	0.0	0.0	0.0	0.0	0.0	0.0
0.0	0.0	0.0	0.0	0.0	0.0	0.0	0.0
0.0	0.0	0.0	0.0	0.0	0.0	0.0	0.0
0.0	0.0	0.0	0.0	0.0	0.0	0.0	0.0
0.0	0.0	0.0	0.0	0.0	0.0	0.0	0.0
0.0	0.0	0.0	0.0	0.0	0.0	0.0	0.0
0.0	0.0	0.0	0.0	0.0	0.0	0.0	0.0

0.0	0.0	0.0	0.0	0.0	0.0	0.0	0.0
0.0	0.0	0.0	0.0	0.0	0.0	0.0	0.0
0.0	0.0	0.0	0.0	0.0	0.0	0.0	0.0
0.0	0.0	0.0	0.0	0.0	0.0	0.0	0.0
0.0	0.0	0.0	0.0	0.0	0.0	0.0	0.0
0.0	0.0	0.0	0.0	0.0	0.0	0.0	0.0
0.0	0.0	0.0	0.0	0.0	0.0	0.0	0.0
0.0	0.0	0.0	0.0	0.0	0.0	0.0	0.0
0.0	0.0	0.0	0.0	0.0	0.0	0.0	0.0
0.0	0.0	0.0	0.0	0.0	0.0	0.0	0.0
0.0	0.0	0.0	0.0	0.0	0.0	0.0	0.0
0.0	0.0	0.0	0.0	0.0	0.0	0.0	0.0
0.0	0.0	0.0	0.0	0.0	0.0	0.0	0.0
0.0	0.0	0.0	0.0	0.0	0.0	0.0	0.0
0.0	0.0	0.0	0.0	0.0	0.0	0.0	0.0
0.0	0.0	0.0	0.0	0.0	0.0	0.0	0.0
0.0	0.0	0.0	0.0	0.0	0.0	0.0	0.0
0.0	0.0	0.0	0.0	0.0	0.0	0.0	0.0
0.0	0.0	0.0	0.0	0.0	0.0	0.0	0.0
0.0	0.0	0.0	0.0	0.0	0.0	0.0	0.0
0.0	0.0	0.0	0.0	0.0	0.0	0.0	0.0
0.0	0.0	0.0	0.0	0.0	0.0	0.0	0.0
0.0	0.0	0.0	0.0	0.0	0.0	0.0	0.0
0.0	0.0	0.0	0.0	0.0	0.0	0.0	0.0
0.0	0.0	0.0	0.0	0.0	0.0	0.0	0.0
0.0	0.0	0.0	0.0	0.0	0.0	0.0	0.0
0.0	0.0	0.0	0.0	0.0	0.0	0.0	0.0
0.0	0.0	0.0	0.0	0.0	0.0	0.0	0.0
0.0	0.0	0.0	0.0	0.0	0.0	0.0	0.0
0.0	0.0	0.0	0.0	0.0	0.0	0.0	0.0
0.0	0.0	0.0	0.0	0.0	0.0	0.0	0.0
0.0	0.0	0.0	0.0	0.0	0.0	0.0	0.0
0.0	0.0	0.0	0.0	0.0	0.0	0.0	0.0
0.0	0.0	0.0	0.0	0.0	0.0	0.0	0.0
0.0	0.0	0.0	0.0	0.0	0.0	0.0	0.0
0.0	0.0	0.0	0.0	0.0	0.0	0.0	0.0
0.0	0.0	0.0	0.0	0.0	0.0	0.0	0.0
0.0	0.0	0.0	0.0	0.0	0.0	0.0	0.0
0.0	0.0	0.0	0.0	0.0	0.0	0.0	0.0
0.0	0.0	0.0	0.0	0.0	0.0	0.0	0.0
0.0	0.0	0.0	0.0	0.0	0.0	0.0	0.0
0.0	0.0	0.0	0.0	0.0	0.0	0.0	0.0
0.0	0.0	0.0	0.0	0.0	0.0	0.0	0.0
0.0	0.0	0.0	0.0	0.0	0.0	0.0	0.0
0.0	0.0	0.0	0.0	0.0	0.0	0.0	0.0
0.0	0.0	0.0	0.0	0.0	0.0	0.0	0.0
0.0	0.0	0.0	0.0	0.0	0.0	0.0	0.0

0.0	0.0	0.0	0.0	0.0	0.0	0.0	0.0
0.0	0.0	0.0	0.0	0.0	0.0	0.0	0.0
0.0	0.0	0.0	0.0	0.0	0.0	0.0	0.0
0.0	0.0	0.0	0.0	0.0	0.0	0.0	0.0
0.0	0.0	0.0	0.0	0.0	0.0	0.0	0.0
0.0	0.0	0.0	0.0	0.0	0.0	0.0	0.0
0.0	0.0	0.0	0.0	0.0	0.0	0.0	0.0
0.0	0.0	0.0	0.0	0.0	0.0	0.0	0.0
0.0	0.0	0.0	0.0	0.0	0.0	0.0	0.0
0.0	0.0	0.0	0.0	0.0	0.0	0.0	0.0
0.0	0.0	0.0	0.0	0.0	0.0	0.0	0.0
0.0	0.0	0.0	0.0	0.0	0.0	0.0	0.0
0.0	0.0	0.0	0.0	0.0	0.0	0.0	0.0
0.0	0.0	0.0	0.0	0.0	0.0	0.0	0.0
0.0	0.0	0.0	0.0	0.0	0.0	0.0	0.0
0.0	0.0	0.0	0.0	0.0	0.0	0.0	0.0
0.0	0.0	0.0	0.0	0.0	0.0	0.0	0.0
0.0	0.0	0.0	0.0	0.0	0.0	0.0	0.0
0.0	0.0	0.0	0.0	0.0	0.0	0.0	0.0
0.0	0.0	0.0	0.0	0.0	0.0	0.0	0.0
0.0	0.0	0.0	0.0	0.0	0.0	0.0	0.0
0.0	0.0	0.0	0.0	0.0	0.0	0.0	0.0
0.0	0.0	0.0	0.0	0.0	0.0	0.0	0.0
0.0	0.0	0.0	0.0	0.0	0.0	0.0	0.0
0.0	0.0	0.0	0.0	0.0	0.0	0.0	0.0
0.0	0.0	0.0	0.0	0.0	0.0	0.0	0.0
0.0	0.0	0.0	0.0	0.0	0.0	0.0	0.0
0.0	0.0	0.0	0.0	0.0	0.0	0.0	0.0
0.0	0.0	0.0	0.0	0.0	0.0	0.0	0.0
0.0	0.0	0.0	0.0	0.0	0.0	0.0	0.0
0.0	0.0	0.0	0.0	0.0	0.0	0.0	0.0
0.0	0.0	0.0	0.0	0.0	0.0	0.0	0.0
0.0	0.0	0.0	0.0	0.0	0.0	0.0	0.0
0.0	0.0	0.0	0.0	0.0	0.0	0.0	0.0
0.0	0.0	0.0	0.0	0.0	0.0	0.0	0.0
0.0	0.0	0.0	0.0	0.0	0.0	0.0	0.0
0.0	0.0	0.0	0.0	0.0	0.0	0.0	0.0
0.0	0.0	0.0	0.0	0.0	0.0	0.0	0.0
0.0	0.0	0.0	0.0	0.0	0.0	0.0	0.0
0.0	0.0	0.0	0.0	0.0	0.0	0.0	0.0
0.0	0.0	0.0	0.0	0.0	0.0	0.0	0.0
0.0	0.0	0.0	0.0	0.0	0.0	0.0	0.0
0.0	0.0	0.0	0.0	0.0	0.0	0.0	0.0
0.0	0.0	0.0	0.0	0.0	0.0	0.0	0.0
0.0	0.0	0.0	0.0	0.0	0.0	0.0	0.0
0.0	0.0	0.0	0.0	0.0	0.0	0.0	0.0
0.0	0.0	0.0	0.0	0.0	0.0	0.0	0.0

0.0	0.0	0.0	0.0	0.0	0.0	0.0	0.0
0.0	0.0	0.0	0.0	0.0	0.0	0.0	0.0
0.0	0.0	0.0	0.0	0.0	0.0	0.0	0.0
0.0	0.0	0.0	0.0	0.0	0.0	0.0	0.0
0.0	0.0	0.0	0.0	0.0	0.0	0.0	0.0
0.0	0.0	0.0	0.0	0.0	0.0	0.0	0.0
0.0	0.0	0.0	0.0	0.0	0.0	0.0	0.0
0.0	0.0	0.0	0.0	0.0	0.0	0.0	0.0
0.0	0.0	0.0	0.0	0.0	0.0	0.0	0.0
0.0	0.0	0.0	0.0	0.0	0.0	0.0	0.0
0.0	0.0	0.0	0.0	0.0	0.0	0.0	0.0
0.0	0.0	0.0	0.0	0.0	0.0	0.0	0.0
0.0	0.0	0.0	0.0	0.0	0.0	0.0	0.0
0.0	0.0	0.0	0.0	0.0	0.0	0.0	0.0
0.0	0.0	0.0	0.0	0.0	0.0	0.0	0.0
0.0	0.0	0.0	0.0	0.0	0.0	0.0	0.0
0.0	0.0	0.0	0.0	0.0	0.0	0.0	0.0
0.0	0.0	0.0	0.0	0.0	0.0	0.0	0.0
0.0	0.0	0.0	0.0	0.0	0.0	0.0	0.0
0.0	0.0	0.0	0.0	0.0	0.0	0.0	0.0
0.0	0.0	0.0	0.0	0.0	0.0	0.0	0.0
0.0	0.0	0.0	0.0	0.0	0.0	0.0	0.0
0.0	0.0	0.0	0.0	0.0	0.0	0.0	0.0
0.0	0.0	0.0	0.0	0.0	0.0	0.0	0.0
0.0	0.0	0.0	0.0	0.0	0.0	0.0	0.0
0.0	0.0	0.0	0.0	0.0	0.0	0.0	0.0
0.0	0.0	0.0	0.0	0.0	0.0	0.0	0.0
0.0	0.0	0.0	0.0	0.0	0.0	0.0	0.0
0.0	0.0	0.0	0.0	0.0	0.0	0.0	0.0
0.0	0.0	0.0	0.0	0.0	0.0	0.0	0.0
0.0	0.0	0.0	0.0	0.0	0.0	0.0	0.0
0.0	0.0	0.0	0.0	0.0	0.0	0.0	0.0
0.0	0.0	0.0	0.0	0.0	0.0	0.0	0.0
0.0	0.0	0.0	0.0	0.0	0.0	0.0	0.0
0.0	0.0	0.0	0.0	0.0	0.0	0.0	0.0
0.0	0.0	0.0	0.0	0.0	0.0	0.0	0.0
0.0	0.0	0.0	0.0	0.0	0.0	0.0	0.0
0.0	0.0	0.0	0.0	0.0	0.0	0.0	0.0
0.0	0.0	0.0	0.0	0.0	0.0	0.0	0.0
0.0	0.0	0.0	0.0	0.0	0.0	0.0	0.0
0.0	0.0	0.0	0.0	0.0	0.0	0.0	0.0
0.0	0.0	0.0	0.0	0.0	0.0	0.0	0.0
0.0	0.0	0.0	0.0	0.0	0.0	0.0	0.0
0.0	0.0	0.0	0.0	0.0	0.0	0.0	0.0
0.0	0.0	0.0	0.0	0.0	0.0	0.0	0.0
0.0	0.0	0.0	0.0	0.0	0.0	0.0	0.0
0.0	0.0	0.0	0.0	0.0	0.0	0.0	0.0

0.0	0.0	0.0	0.0	0.0	0.0	0.0	0.0
0.0	0.0	0.0	0.0	0.0	0.0	0.0	0.0
0.0	0.0	0.0	0.0	0.0	0.0	0.0	0.0
0.0	0.0	0.0	0.0	0.0	0.0	0.0	0.0
0.0	0.0	0.0	0.0	0.0	0.0	0.0	0.0
0.0	0.0	0.0	0.0	0.0	0.0	0.0	0.0
0.0	0.0	0.0	0.0	0.0	0.0	0.0	0.0
0.0	0.0	0.0	0.0	0.0	0.0	0.0	0.0
0.0	0.0	0.0	0.0	0.0	0.0	0.0	0.0
0.0	0.0	0.0	0.0	0.0	0.0	0.0	0.0
0.0	0.0	0.0	0.0	0.0	0.0	0.0	0.0
0.0	0.0	0.0	0.0	0.0	0.0	0.0	0.0
0.0	0.0	0.0	0.0	0.0	0.0	0.0	0.0
0.0	0.0	0.0	0.0	0.0	0.0	0.0	0.0
0.0	0.0	0.0	0.0	0.0	0.0	0.0	0.0
0.0	0.0	0.0	0.0	0.0	0.0	0.0	0.0
0.0	0.0	0.0	0.0	0.0	0.0	0.0	0.0
0.0	0.0	0.0	0.0	0.0	0.0	0.0	0.0
0.0	0.0	0.0	0.0	0.0	0.0	0.0	0.0
0.0	0.0	0.0	0.0	0.0	0.0	0.0	0.0
0.0	0.0	0.0	0.0	0.0	0.0	0.0	0.0
0.0	0.0	0.0	0.0	0.0	0.0	0.0	0.0
0.0	0.0	0.0	0.0	0.0	0.0	0.0	0.0
0.0	0.0	0.0	0.0	0.0	0.0	0.0	0.0
0.0	0.0	0.0	0.0	0.0	0.0	0.0	0.0
0.0	0.0	0.0	0.0	0.0	0.0	0.0	0.0
0.0	0.0	0.0	0.0	0.0	0.0	0.0	0.0
0.0	0.0	0.0	0.0	0.0	0.0	0.0	0.0
0.0	0.0	0.0	0.0	0.0	0.0	0.0	0.0
0.0	0.0	0.0	0.0	0.0	0.0	0.0	0.0
0.0	0.0	0.0	0.0	0.0	0.0	0.0	0.0
0.0	0.0	0.0	0.0	0.0	0.0	0.0	0.0
0.0	0.0	0.0	0.0	0.0	0.0	0.0	0.0
0.0	0.0	0.0	0.0	0.0	0.0	0.0	0.0
0.0	0.0	0.0	0.0	0.0	0.0	0.0	0.0
0.0	0.0	0.0	0.0	0.0	0.0	0.0	0.0
0.0	0.0	0.0	0.0	0.0	0.0	0.0	0.0
0.0	0.0	0.0	0.0	0.0	0.0	0.0	0.0
0.0	0.0	0.0	0.0	0.0	0.0	0.0	0.0
0.0	0.0	0.0	0.0	0.0	0.0	0.0	0.0
0.0	0.0	0.0	0.0	0.0	0.0	0.0	0.0
0.0	0.0	0.0	0.0	0.0	0.0	0.0	0.0
0.0	0.0	0.0	0.0	0.0	0.0	0.0	0.0
0.0	0.0	0.0	0.0	0.0	0.0	0.0	0.0
0.0	0.0	0.0	0.0	0.0	0.0	0.0	0.0
0.0	0.0	0.0	0.0	0.0	0.0	0.0	0.0
0.0	0.0	0.0	0.0	0.0	0.0	0.0	0.0
0.0	0.0	0.0	0.0	0.0	0.0	0.0	0.0

0.0	0.0	0.0	0.0	0.0	0.0	0.0	0.0
0.0	0.0	0.0	0.0	0.0	0.0	0.0	0.0
0.0	0.0	0.0	0.0	0.0	0.0	0.0	0.0
0.0	0.0	0.0	0.0	0.0	0.0	0.0	0.0
0.0	0.0	0.0	0.0	0.0	0.0	0.0	0.0
0.0	0.0	0.0	0.0	0.0	0.0	0.0	0.0
0.0	0.0	0.0	0.0	0.0	0.0	0.0	0.0
0.0	0.0	0.0	0.0	0.0	0.0	0.0	0.0
0.0	0.0	0.0	0.0	0.0	0.0	0.0	0.0
0.0	0.0	0.0	0.0	0.0	0.0	0.0	0.0
0.0	0.0	0.0	0.0	0.0	0.0	0.0	0.0
0.0	0.0	0.0	0.0	0.0	0.0	0.0	0.0
0.0	0.0	0.0	0.0	0.0	0.0	0.0	0.0
0.0	0.0	0.0	0.0	0.0	0.0	0.0	0.0
0.0	0.0	0.0	0.0	0.0	0.0	0.0	0.0
0.0	0.0	0.0	0.0	0.0	0.0	0.0	0.0
0.0	0.0	0.0	0.0	0.0	0.0	0.0	0.0
0.0	0.0	0.0	0.0	0.0	0.0	0.0	0.0
0.0	0.0	0.0	0.0	0.0	0.0	0.0	0.0
0.0	0.0	0.0	0.0	0.0	0.0	0.0	0.0
0.0	0.0	0.0	0.0	0.0	0.0	0.0	0.0
0.0	0.0	0.0	0.0	0.0	0.0	0.0	0.0
0.0	0.0	0.0	0.0	0.0	0.0	0.0	0.0
0.0	0.0	0.0	0.0	0.0	0.0	0.0	0.0
0.0	0.0	0.0	0.0	0.0	0.0	0.0	0.0
0.0	0.0	0.0	0.0	0.0	0.0	0.0	0.0
0.0	0.0	0.0	0.0	0.0	0.0	0.0	0.0
0.0	0.0	0.0	0.0	0.0	0.0	0.0	0.0
0.0	0.0	0.0	0.0	0.0	0.0	0.0	0.0
0.0	0.0	0.0	0.0	0.0	0.0	0.0	0.0
0.0	0.0	0.0	0.0	0.0	0.0	0.0	0.0
0.0	0.0	0.0	0.0	0.0	0.0	0.0	0.0
0.0	0.0	0.0	0.0	0.0	0.0	0.0	0.0
0.0	0.0	0.0	0.0	0.0	0.0	0.0	0.0
0.0	0.0	0.0	0.0	0.0	0.0	0.0	0.0
0.0	0.0	0.0	0.0	0.0	0.0	0.0	0.0
0.0	0.0	0.0	0.0	0.0	0.0	0.0	0.0
0.0	0.0	0.0	0.0	0.0	0.0	0.0	0.0
0.0	0.0	0.0	0.0	0.0	0.0	0.0	0.0
0.0	0.0	0.0	0.0	0.0	0.0	0.0	0.0
0.0	0.0	0.0	0.0	0.0	0.0	0.0	0.0
0.0	0.0	0.0	0.0	0.0	0.0	0.0	0.0
0.0	0.0	0.0	0.0	0.0	0.0	0.0	0.0
0.0	0.0	0.0	0.0	0.0	0.0	0.0	0.0
0.0	0.0	0.0	0.0	0.0	0.0	0.0	0.0
0.0	0.0	0.0	0.0	0.0	0.0	0.0	0.0
0.0	0.0	0.0	0.0	0.0	0.0	0.0	0.0

0.0	0.0	0.0	0.0	0.0	0.0	0.0	0.0
0.0	0.0	0.0	0.0	0.0	0.0	0.0	0.0
0.0	0.0	0.0	0.0	0.0	0.0	0.0	0.0
0.0	0.0	0.0	0.0	0.0	0.0	0.0	0.0
0.0	0.0	0.0	0.0	0.0	0.0	0.0	0.0
0.0	0.0	0.0	0.0	0.0	0.0	0.0	0.0
0.0	0.0	0.0	0.0	0.0	0.0	0.0	0.0
0.0	0.0	0.0	0.0	0.0	0.0	0.0	0.0
0.0	0.0	0.0	0.0	0.0	0.0	0.0	0.0
0.0	0.0	0.0	0.0	0.0	0.0	0.0	0.0
0.0	0.0	0.0	0.0	0.0	0.0	0.0	0.0
0.0	0.0	0.0	0.0	0.0	0.0	0.0	0.0
0.0	0.0	0.0	0.0	0.0	0.0	0.0	0.0
0.0	0.0	0.0	0.0	0.0	0.0	0.0	0.0
0.0	0.0	0.0	0.0	0.0	0.0	0.0	0.0
0.0	0.0	0.0	0.0	0.0	0.0	0.0	0.0
0.0	0.0	0.0	0.0	0.0	0.0	0.0	0.0
0.0	0.0	0.0	0.0	0.0	0.0	0.0	0.0
0.0	0.0	0.0	0.0	0.0	0.0	0.0	0.0
0.0	0.0	0.0	0.0	0.0	0.0	0.0	0.0
0.0	0.0	0.0	0.0	0.0	0.0	0.0	0.0
0.0	0.0	0.0	0.0	0.0	0.0	0.0	0.0
0.0	0.0	0.0	0.0	0.0	0.0	0.0	0.0
0.0	0.0	0.0	0.0	0.0	0.0	0.0	0.0
0.0	0.0	0.0	0.0	0.0	0.0	0.0	0.0
0.0	0.0	0.0	0.0	0.0	0.0	0.0	0.0
0.0	0.0	0.0	0.0	0.0	0.0	0.0	0.0
0.0	0.0	0.0	0.0	0.0	0.0	0.0	0.0
0.0	0.0	0.0	0.0	0.0	0.0	0.0	0.0
0.0	0.0	0.0	0.0	0.0	0.0	0.0	0.0
0.0	0.0	0.0	0.0	0.0	0.0	0.0	0.0
0.0	0.0	0.0	0.0	0.0	0.0	0.0	0.0
0.0	0.0	0.0	0.0	0.0	0.0	0.0	0.0
0.0	0.0	0.0	0.0	0.0	0.0	0.0	0.0
0.0	0.0	0.0	0.0	0.0	0.0	0.0	0.0
0.0	0.0	0.0	0.0	0.0	0.0	0.0	0.0
0.0	0.0	0.0	0.0	0.0	0.0	0.0	0.0
0.0	0.0	0.0	0.0	0.0	0.0	0.0	0.0
0.0	0.0	0.0	0.0	0.0	0.0	0.0	0.0
0.0	0.0	0.0	0.0	0.0	0.0	0.0	0.0
0.0	0.0	0.0	0.0	0.0	0.0	0.0	0.0
0.0	0.0	0.0	0.0	0.0	0.0	0.0	0.0
0.0	0.0	0.0	0.0	0.0	0.0	0.0	0.0
0.0	0.0	0.0	0.0	0.0	0.0	0.0	0.0
0.0	0.0	0.0	0.0	0.0	0.0	0.0	0.0
0.0	0.0	0.0	0.0	0.0	0.0	0.0	0.0
0.0	0.0	0.0	0.0	0.0	0.0	0.0	0.0

0.0	0.0	0.0	0.0	0.0	0.0	0.0	0.0
0.0	0.0	0.0	0.0	0.0	0.0	0.0	0.0
0.0	0.0	0.0	0.0	0.0	0.0	0.0	0.0
0.0	0.0	0.0	0.0	0.0	0.0	0.0	0.0
0.0	0.0	0.0	0.0	0.0	0.0	0.0	0.0
0.0	0.0	0.0	0.0	0.0	0.0	0.0	0.0
0.0	0.0	0.0	0.0	0.0	0.0	0.0	0.0
0.0	0.0	0.0	0.0	0.0	0.0	0.0	0.0
0.0	0.0	0.0	0.0	0.0	0.0	0.0	0.0
0.0	0.0	0.0	0.0	0.0	0.0	0.0	0.0
0.0	0.0	0.0	0.0	0.0	0.0	0.0	0.0
0.0	0.0	0.0	0.0	0.0	0.0	0.0	0.0
0.0	0.0	0.0	0.0	0.0	0.0	0.0	0.0
0.0	0.0	0.0	0.0	0.0	0.0	0.0	0.0
0.0	0.0	0.0	0.0	0.0	0.0	0.0	0.0
0.0	0.0	0.0	0.0	0.0	0.0	0.0	0.0
0.0	0.0	0.0	0.0	0.0	0.0	0.0	0.0
0.0	0.0	0.0	0.0	0.0	0.0	0.0	0.0
0.0	0.0	0.0	0.0	0.0	0.0	0.0	0.0
0.0	0.0	0.0	0.0	0.0	0.0	0.0	0.0
0.0	0.0	0.0	0.0	0.0	0.0	0.0	0.0
0.0	0.0	0.0	0.0	0.0	0.0	0.0	0.0
0.0	0.0	0.0	0.0	0.0	0.0	0.0	0.0
0.0	0.0	0.0	0.0	0.0	0.0	0.0	0.0
0.0	0.0	0.0	0.0	0.0	0.0	0.0	0.0
0.0	0.0	0.0	0.0	0.0	0.0	0.0	0.0
0.0	0.0	0.0	0.0	0.0	0.0	0.0	0.0
0.0	0.0	0.0	0.0	0.0	0.0	0.0	0.0
0.0	0.0	0.0	0.0	0.0	0.0	0.0	0.0
0.0	0.0	0.0	0.0	0.0	0.0	0.0	0.0
0.0	0.0	0.0	0.0	0.0	0.0	0.0	0.0
0.0	0.0	0.0	0.0	0.0	0.0	0.0	0.0
0.0	0.0	0.0	0.0	0.0	0.0	0.0	0.0
0.0	0.0	0.0	0.0	0.0	0.0	0.0	0.0
0.0	0.0	0.0	0.0	0.0	0.0	0.0	0.0
0.0	0.0	0.0	0.0	0.0	0.0	0.0	0.0
0.0	0.0	0.0	0.0	0.0	0.0	0.0	0.0
0.0	0.0	0.0	0.0	0.0	0.0	0.0	0.0
0.0	0.0	0.0	0.0	0.0	0.0	0.0	0.0
0.0	0.0	0.0	0.0	0.0	0.0	0.0	0.0
0.0	0.0	0.0	0.0	0.0	0.0	0.0	0.0
0.0	0.0	0.0	0.0	0.0	0.0	0.0	0.0
0.0	0.0	0.0	0.0	0.0	0.0	0.0	0.0
0.0	0.0	0.0	0.0	0.0	0.0	0.0	0.0
0.0	0.0	0.0	0.0	0.0	0.0	0.0	0.0
0.0	0.0	0.0	0.0	0.0	0.0	0.0	0.0
0.0	0.0	0.0	0.0	0.0	0.0	0.0	0.0

0.0	0.0	0.0	0.0	0.0	0.0	0.0	0.0
0.0	0.0	0.0	0.0	0.0	0.0	0.0	0.0
0.0	0.0	0.0	0.0	0.0	0.0	0.0	0.0
0.0	0.0	0.0	0.0	0.0	0.0	0.0	0.0
0.0	0.0	0.0	0.0	0.0	0.0	0.0	0.0
0.0	0.0	0.0	0.0	0.0	0.0	0.0	0.0
0.0	0.0	0.0	0.0	0.0	0.0	0.0	0.0
0.0	0.0	0.0	0.0	0.0	0.0	0.0	0.0
0.0	0.0	0.0	0.0	0.0	0.0	0.0	0.0
0.0	0.0	0.0	0.0	0.0	0.0	0.0	0.0
0.0	0.0	0.0	0.0	0.0	0.0	0.0	0.0
0.0	0.0	0.0	0.0	0.0	0.0	0.0	0.0
0.0	0.0	0.0	0.0	0.0	0.0	0.0	0.0
0.0	0.0	0.0	0.0	0.0	0.0	0.0	0.0
0.0	0.0	0.0	0.0	0.0	0.0	0.0	0.0
0.0	0.0	0.0	0.0	0.0	0.0	0.0	0.0
0.0	0.0	0.0	0.0	0.0	0.0	0.0	0.0
0.0	0.0	0.0	0.0	0.0	0.0	0.0	0.0
0.0	0.0	0.0	0.0	0.0	0.0	0.0	0.0
0.0	0.0	0.0	0.0	0.0	0.0	0.0	0.0
0.0	0.0	0.0	0.0	0.0	0.0	0.0	0.0
0.0	0.0	0.0	0.0	0.0	0.0	0.0	0.0
0.0	0.0	0.0	0.0	0.0	0.0	0.0	0.0
0.0	0.0	0.0	0.0	0.0	0.0	0.0	0.0
0.0	0.0	0.0	0.0	0.0	0.0	0.0	0.0
0.0	0.0	0.0	0.0	0.0	0.0	0.0	0.0
0.0	0.0	0.0	0.0	0.0	0.0	0.0	0.0
0.0	0.0	0.0	0.0	0.0	0.0	0.0	0.0
0.0	0.0	0.0	0.0	0.0	0.0	0.0	0.0
0.0	0.0	0.0	0.0	0.0	0.0	0.0	0.0
0.0	0.0	0.0	0.0	0.0	0.0	0.0	0.0
0.0	0.0	0.0	0.0	0.0	0.0	0.0	0.0
0.0	0.0	0.0	0.0	0.0	0.0	0.0	0.0
0.0	0.0	0.0	0.0	0.0	0.0	0.0	0.0
0.0	0.0	0.0	0.0	0.0	0.0	0.0	0.0
0.0	0.0	0.0	0.0	0.0	0.0	0.0	0.0
0.0	0.0	0.0	0.0	0.0	0.0	0.0	0.0
0.0	0.0	0.0	0.0	0.0	0.0	0.0	0.0
0.0	0.0	0.0	0.0	0.0	0.0	0.0	0.0
0.0	0.0	0.0	0.0	0.0	0.0	0.0	0.0
0.0	0.0	0.0	0.0	0.0	0.0	0.0	0.0
0.0	0.0	0.0	0.0	0.0	0.0	0.0	0.0
0.0	0.0	0.0	0.0	0.0	0.0	0.0	0.0
0.0	0.0	0.0	0.0	0.0	0.0	0.0	0.0
0.0	0.0	0.0	0.0	0.0	0.0	0.0	0.0
0.0	0.0	0.0	0.0	0.0	0.0	0.0	0.0
0.0	0.0	0.0	0.0	0.0	0.0	0.0	0.0

0.0	0.0	0.0	0.0	0.0	0.0	0.0	0.0
0.0	0.0	0.0	0.0	0.0	0.0	0.0	0.0
0.0	0.0	0.0	0.0	0.0	0.0	0.0	0.0
0.0	0.0	0.0	0.0	0.0	0.0	0.0	0.0
0.0	0.0	0.0	0.0	0.0	0.0	0.0	0.0
0.0	0.0	0.0	0.0	0.0	0.0	0.0	0.0
0.0	0.0	0.0	0.0	0.0	0.0	0.0	0.0
0.0	0.0	0.0	0.0	0.0	0.0	0.0	0.0
0.0	0.0	0.0	0.0	0.0	0.0	0.0	0.0
0.0	0.0	0.0	0.0	0.0	0.0	0.0	0.0
0.0	0.0	0.0	0.0	0.0	0.0	0.0	0.0
0.0	0.0	0.0	0.0	0.0	0.0	0.0	0.0
0.0	0.0	0.0	0.0	0.0	0.0	0.0	0.0
0.0	0.0	0.0	0.0	0.0	0.0	0.0	0.0
0.0	0.0	0.0	0.0	0.0	0.0	0.0	0.0
0.0	0.0	0.0	0.0	0.0	0.0	0.0	0.0
0.0	0.0	0.0	0.0	0.0	0.0	0.0	0.0
0.0	0.0	0.0	0.0	0.0	0.0	0.0	0.0
0.0	0.0	0.0	0.0	0.0	0.0	0.0	0.0
0.0	0.0	0.0	0.0	0.0	0.0	0.0	0.0
0.0	0.0	0.0	0.0	0.0	0.0	0.0	0.0
0.0	0.0	0.0	0.0	0.0	0.0	0.0	0.0
0.0	0.0	0.0	0.0	0.0	0.0	0.0	0.0
0.0	0.0	0.0	0.0	0.0	0.0	0.0	0.0
0.0	0.0	0.0	0.0	0.0	0.0	0.0	0.0
0.0	0.0	0.0	0.0	0.0	0.0	0.0	0.0
0.0	0.0	0.0	0.0	0.0	0.0	0.0	0.0
0.0	0.0	0.0	0.0	0.0	0.0	0.0	0.0
0.0	0.0	0.0	0.0	0.0	0.0	0.0	0.0
0.0	0.0	0.0	0.0	0.0	0.0	0.0	0.0
0.0	0.0	0.0	0.0	0.0	0.0	0.0	0.0
0.0	0.0	0.0	0.0	0.0	0.0	0.0	0.0
0.0	0.0	0.0	0.0	0.0	0.0	0.0	0.0
0.0	0.0	0.0	0.0	0.0	0.0	0.0	0.0
0.0	0.0	0.0	0.0	0.0	0.0	0.0	0.0
0.0	0.0	0.0	0.0	0.0	0.0	0.0	0.0
0.0	0.0	0.0	0.0	0.0	0.0	0.0	0.0
0.0	0.0	0.0	0.0	0.0	0.0	0.0	0.0
0.0	0.0	0.0	0.0	0.0	0.0	0.0	0.0
0.0	0.0	0.0	0.0	0.0	0.0	0.0	0.0
0.0	0.0	0.0	0.0	0.0	0.0	0.0	0.0
0.0	0.0	0.0	0.0	0.0	0.0	0.0	0.0
0.0	0.0	0.0	0.0	0.0	0.0	0.0	0.0
0.0	0.0	0.0	0.0	0.0	0.0	0.0	0.0
0.0	0.0	0.0	0.0	0.0	0.0	0.0	0.0
0.0	0.0	0.0	0.0	0.0	0.0	0.0	0.0
0.0	0.0	0.0	0.0	0.0	0.0	0.0	0.0
0.0	0.0	0.0	0.0	0.0	0.0	0.0	0.0

0.0	0.0	0.0	0.0	0.0	0.0	0.0	0.0
0.0	0.0	0.0	0.0	0.0	0.0	0.0	0.0
0.0	0.0	0.0	0.0	0.0	0.0	0.0	0.0
0.0	0.0	0.0	0.0	0.0	0.0	0.0	0.0
0.0	0.0	0.0	0.0	0.0	0.0	0.0	0.0
0.0	0.0	0.0	0.0	0.0	0.0	0.0	0.0
0.0	0.0	0.0	0.0	0.0	0.0	0.0	0.0
0.0	0.0	0.0	0.0	0.0	0.0	0.0	0.0
0.0	0.0	0.0	0.0	0.0	0.0	0.0	0.0
0.0	0.0	0.0	0.0	0.0	0.0	0.0	0.0
0.0	0.0	0.0	0.0	0.0	0.0	0.0	0.0
0.0	0.0	0.0	0.0	0.0	0.0	0.0	0.0
0.0	0.0	0.0	0.0	0.0	0.0	0.0	0.0
0.0	0.0	0.0	0.0	0.0	0.0	0.0	0.0
0.0	0.0	0.0	0.0	0.0	0.0	0.0	0.0
0.0	0.0	0.0	0.0	0.0	0.0	0.0	0.0
0.0	0.0	0.0	0.0	0.0	0.0	0.0	0.0
0.0	0.0	0.0	0.0	0.0	0.0	0.0	0.0
0.0	0.0	0.0	0.0	0.0	0.0	0.0	0.0
0.0	0.0	0.0	0.0	0.0	0.0	0.0	0.0
0.0	0.0	0.0	0.0	0.0	0.0	0.0	0.0
0.0	0.0	0.0	0.0	0.0	0.0	0.0	0.0
0.0	0.0	0.0	0.0	0.0	0.0	0.0	0.0
0.0	0.0	0.0	0.0	0.0	0.0	0.0	0.0
0.0	0.0	0.0	0.0	0.0	0.0	0.0	0.0
0.0	0.0	0.0	0.0	0.0	0.0	0.0	0.0
0.0	0.0	0.0	0.0	0.0	0.0	0.0	0.0
0.0	0.0	0.0	0.0	0.0	0.0	0.0	0.0
0.0	0.0	0.0	0.0	0.0	0.0	0.0	0.0
0.0	0.0	0.0	0.0	0.0	0.0	0.0	0.0
0.0	0.0	0.0	0.0	0.0	0.0	0.0	0.0
0.0	0.0	0.0	0.0	0.0	0.0	0.0	0.0
0.0	0.0	0.0	0.0	0.0	0.0	0.0	0.0
0.0	0.0	0.0	0.0	0.0	0.0	0.0	0.0
0.0	0.0	0.0	0.0	0.0	0.0	0.0	0.0
0.0	0.0	0.0	0.0	0.0	0.0	0.0	0.0
0.0	0.0	0.0	0.0	0.0	0.0	0.0	0.0
0.0	0.0	0.0	0.0	0.0	0.0	0.0	0.0
0.0	0.0	0.0	0.0	0.0	0.0	0.0	0.0
0.0	0.0	0.0	0.0	0.0	0.0	0.0	0.0
0.0	0.0	0.0	0.0	0.0	0.0	0.0	0.0
0.0	0.0	0.0	0.0	0.0	0.0	0.0	0.0
0.0	0.0	0.0	0.0	0.0	0.0	0.0	0.0
0.0	0.0	0.0	0.0	0.0	0.0	0.0	0.0
0.0	0.0	0.0	0.0	0.0	0.0	0.0	0.0
0.0	0.0	0.0	0.0	0.0	0.0	0.0	0.0
0.0	0.0	0.0	0.0	0.0	0.0	0.0	0.0

0.0	0.0	0.0	0.0	0.0	0.0	0.0	0.0
0.0	0.0	0.0	0.0	0.0	0.0	0.0	0.0
0.0	0.0	0.0	0.0	0.0	0.0	0.0	0.0
0.0	0.0	0.0	0.0	0.0	0.0	0.0	0.0
0.0	0.0	0.0	0.0	0.0	0.0	0.0	0.0
0.0	0.0	0.0	0.0	0.0	0.0	0.0	0.0
0.0	0.0	0.0	0.0	0.0	0.0	0.0	0.0
0.0	0.0	0.0	0.0	0.0	0.0	0.0	0.0
0.0	0.0	0.0	0.0	0.0	0.0	0.0	0.0
0.0	0.0	0.0	0.0	0.0	0.0	0.0	0.0
0.0	0.0	0.0	0.0	0.0	0.0	0.0	0.0
0.0	0.0	0.0	0.0	0.0	0.0	0.0	0.0
0.0	0.0	0.0	0.0	0.0	0.0	0.0	0.0
0.0	0.0	0.0	0.0	0.0	0.0	0.0	0.0
0.0	0.0	0.0	0.0	0.0	0.0	0.0	0.0
0.0	0.0	0.0	0.0	0.0	0.0	0.0	0.0
0.0	0.0	0.0	0.0	0.0	0.0	0.0	0.0
0.0	0.0	0.0	0.0	0.0	0.0	0.0	0.0
0.0	0.0	0.0	0.0	0.0	0.0	0.0	0.0
0.0	0.0	0.0	0.0	0.0	0.0	0.0	0.0
0.0	0.0	0.0	0.0	0.0	0.0	0.0	0.0
0.0	0.0	0.0	0.0	0.0	0.0	0.0	0.0
0.0	0.0	0.0	0.0	0.0	0.0	0.0	0.0
0.0	0.0	0.0	0.0	0.0	0.0	0.0	0.0
0.0	0.0	0.0	0.0	0.0	0.0	0.0	0.0
0.0	0.0	0.0	0.0	0.0	0.0	0.0	0.0
0.0	0.0	0.0	0.0	0.0	0.0	0.0	0.0
0.0	0.0	0.0	0.0	0.0	0.0	0.0	0.0
0.0	0.0	0.0	0.0	0.0	0.0	0.0	0.0
0.0	0.0	0.0	0.0	0.0	0.0	0.0	0.0
0.0	0.0	0.0	0.0	0.0	0.0	0.0	0.0
0.0	0.0	0.0	0.0	0.0	0.0	0.0	0.0
0.0	0.0	0.0	0.0	0.0	0.0	0.0	0.0
0.0	0.0	0.0	0.0	0.0	0.0	0.0	0.0
0.0	0.0	0.0	0.0	0.0	0.0	0.0	0.0
0.0	0.0	0.0	0.0	0.0	0.0	0.0	0.0
0.0	0.0	0.0	0.0	0.0	0.0	0.0	0.0
0.0	0.0	0.0	0.0	0.0	0.0	0.0	0.0
0.0	0.0	0.0	0.0	0.0	0.0	0.0	0.0
0.0	0.0	0.0	0.0	0.0	0.0	0.0	0.0
0.0	0.0	0.0	0.0	0.0	0.0	0.0	0.0
0.0	0.0	0.0	0.0	0.0	0.0	0.0	0.0
0.0	0.0	0.0	0.0	0.0	0.0	0.0	0.0
0.0	0.0	0.0	0.0	0.0	0.0	0.0	0.0
0.0	0.0	0.0	0.0	0.0	0.0	0.0	0.0
0.0	0.0	0.0	0.0	0.0	0.0	0.0	0.0
0.0	0.0	0.0	0.0	0.0	0.0	0.0	0.0
0.0	0.0	0.0	0.0	0.0	0.0	0.0	0.0

0.0	0.0	0.0	0.0	0.0	0.0	0.0	0.0
0.0	0.0	0.0	0.0	0.0	0.0	0.0	0.0
0.0	0.0	0.0	0.0	0.0	0.0	0.0	0.0
0.0	0.0	0.0	0.0	0.0	0.0	0.0	0.0
0.0	0.0	0.0	0.0	0.0	0.0	0.0	0.0
0.0	0.0	0.0	0.0	0.0	0.0	0.0	0.0
0.0	0.0	0.0	0.0	0.0	0.0	0.0	0.0
0.0	0.0	0.0	0.0	0.0	0.0	0.0	0.0
0.0	0.0	0.0	0.0	0.0	0.0	0.0	0.0
0.0	0.0	0.0	0.0	0.0	0.0	0.0	0.0
0.0	0.0	0.0	0.0	0.0	0.0	0.0	0.0
0.0	0.0	0.0	0.0	0.0	0.0	0.0	0.0
0.0	0.0	0.0	0.0	0.0	0.0	0.0	0.0
0.0	0.0	0.0	0.0	0.0	0.0	0.0	0.0
0.0	0.0	0.0	0.0	0.0	0.0	0.0	0.0
0.0	0.0	0.0	0.0	0.0	0.0	0.0	0.0
0.0	0.0	0.0	0.0	0.0	0.0	0.0	0.0
0.0	0.0	0.0	0.0	0.0	0.0	0.0	0.0
0.0	0.0	0.0	0.0	0.0	0.0	0.0	0.0
0.0	0.0	0.0	0.0	0.0	0.0	0.0	0.0
0.0	0.0	0.0	0.0	0.0	0.0	0.0	0.0
0.0	0.0	0.0	0.0	0.0	0.0	0.0	0.0
0.0	0.0	0.0	0.0	0.0	0.0	0.0	0.0
0.0	0.0	0.0	0.0	0.0	0.0	0.0	0.0
0.0	0.0	0.0	0.0	0.0	0.0	0.0	0.0
0.0	0.0	0.0	0.0	0.0	0.0	0.0	0.0
0.0	0.0	0.0	0.0	0.0	0.0	0.0	0.0
0.0	0.0	0.0	0.0	0.0	0.0	0.0	0.0
0.0	0.0	0.0	0.0	0.0	0.0	0.0	0.0
0.0	0.0	0.0	0.0	0.0	0.0	0.0	0.0
0.0	0.0	0.0	0.0	0.0	0.0	0.0	0.0
0.0	0.0	0.0	0.0	0.0	0.0	0.0	0.0
0.0	0.0	0.0	0.0	0.0	0.0	0.0	0.0
0.0	0.0	0.0	0.0	0.0	0.0	0.0	0.0
0.0	0.0	0.0	0.0	0.0	0.0	0.0	0.0
0.0	0.0	0.0	0.0	0.0	0.0	0.0	0.0
0.0	0.0	0.0	0.0	0.0	0.0	0.0	0.0
0.0	0.0	0.0	0.0	0.0	0.0	0.0	0.0
0.0	0.0	0.0	0.0	0.0	0.0	0.0	0.0
0.0	0.0	0.0	0.0	0.0	0.0	0.0	0.0
0.0	0.0	0.0	0.0	0.0	0.0	0.0	0.0
0.0	0.0	0.0	0.0	0.0	0.0	0.0	0.0
0.0	0.0	0.0	0.0	0.0	0.0	0.0	0.0
0.0	0.0	0.0	0.0	0.0	0.0	0.0	0.0
0.0	0.0	0.0	0.0	0.0	0.0	0.0	0.0
0.0	0.0	0.0	0.0	0.0	0.0	0.0	0.0
0.0	0.0	0.0	0.0	0.0	0.0	0.0	0.0
0.0	0.0	0.0	0.0	0.0	0.0	0.0	0.0

0.0	0.0	0.0	0.0	0.0	0.0	0.0	0.0
0.0	0.0	0.0	0.0	0.0	0.0	0.0	0.0
0.0	0.0	0.0	0.0	0.0	0.0	0.0	0.0
0.0	0.0	0.0	0.0	0.0	0.0	0.0	0.0
0.0	0.0	0.0	0.0	0.0	0.0	0.0	0.0
0.0	0.0	0.0	0.0	0.0	0.0	0.0	0.0
0.0	0.0	0.0	0.0	0.0	0.0	0.0	0.0
0.0	0.0	0.0	0.0	0.0	0.0	0.0	0.0
0.0	0.0	0.0	0.0	0.0	0.0	0.0	0.0
0.0	0.0	0.0	0.0	0.0	0.0	0.0	0.0
0.0	0.0	0.0	0.0	0.0	0.0	0.0	0.0
0.0	0.0	0.0	0.0	0.0	0.0	0.0	0.0
0.0	0.0	0.0	0.0	0.0	0.0	0.0	0.0
0.0	0.0	0.0	0.0	0.0	0.0	0.0	0.0
0.0	0.0	0.0	0.0	0.0	0.0	0.0	0.0
0.0	0.0	0.0	0.0	0.0	0.0	0.0	0.0
0.0	0.0	0.0	0.0	0.0	0.0	0.0	0.0
0.0	0.0	0.0	0.0	0.0	0.0	0.0	0.0
0.0	0.0	0.0	0.0	0.0	0.0	0.0	0.0
0.0	0.0	0.0	0.0	0.0	0.0	0.0	0.0
0.0	0.0	0.0	0.0	0.0	0.0	0.0	0.0
0.0	0.0	0.0	0.0	0.0	0.0	0.0	0.0
0.0	0.0	0.0	0.0	0.0	0.0	0.0	0.0
0.0	0.0	0.0	0.0	0.0	0.0	0.0	0.0
0.0	0.0	0.0	0.0	0.0	0.0	0.0	0.0
0.0	0.0	0.0	0.0	0.0	0.0	0.0	0.0
0.0	0.0	0.0	0.0	0.0	0.0	0.0	0.0
0.0	0.0	0.0	0.0	0.0	0.0	0.0	0.0
0.0	0.0	0.0	0.0	0.0	0.0	0.0	0.0
0.0	0.0	0.0	0.0	0.0	0.0	0.0	0.0
0.0	0.0	0.0	0.0	0.0	0.0	0.0	0.0
0.0	0.0	0.0	0.0	0.0	0.0	0.0	0.0
0.0	0.0	0.0	0.0	0.0	0.0	0.0	0.0
0.0	0.0	0.0	0.0	0.0	0.0	0.0	0.0
0.0	0.0	0.0	0.0	0.0	0.0	0.0	0.0
0.0	0.0	0.0	0.0	0.0	0.0	0.0	0.0
0.0	0.0	0.0	0.0	0.0	0.0	0.0	0.0
0.0	0.0	0.0	0.0	0.0	0.0	0.0	0.0
0.0	0.0	0.0	0.0	0.0	0.0	0.0	0.0
0.0	0.0	0.0	0.0	0.0	0.0	0.0	0.0
0.0	0.0	0.0	0.0	0.0	0.0	0.0	0.0
0.0	0.0	0.0	0.0	0.0	0.0	0.0	0.0
0.0	0.0	0.0	0.0	0.0	0.0	0.0	0.0
0.0	0.0	0.0	0.0	0.0	0.0	0.0	0.0
0.0	0.0	0.0	0.0	0.0	0.0	0.0	0.0
0.0	0.0	0.0	0.0	0.0	0.0	0.0	0.0
0.0	0.0	0.0	0.0	0.0	0.0	0.0	0.0

0.0	0.0	0.0	0.0	0.0	0.0	0.0	0.0
0.0	0.0	0.0	0.0	0.0	0.0	0.0	0.0
0.0	0.0	0.0	0.0	0.0	0.0	0.0	0.0
0.0	0.0	0.0	0.0	0.0	0.0	0.0	0.0
0.0	0.0	0.0	0.0	0.0	0.0	0.0	0.0
0.0	0.0	0.0	0.0	0.0	0.0	0.0	0.0
0.0	0.0	0.0	0.0	0.0	0.0	0.0	0.0
0.0	0.0	0.0	0.0	0.0	0.0	0.0	0.0
0.0	0.0	0.0	0.0	0.0	0.0	0.0	0.0
0.0	0.0	0.0	0.0	0.0	0.0	0.0	0.0
0.0	0.0	0.0	0.0	0.0	0.0	0.0	0.0
0.0	0.0	0.0	0.0	0.0	0.0	0.0	0.0
0.0	0.0	0.0	0.0	0.0	0.0	0.0	0.0
0.0	0.0	0.0	0.0	0.0	0.0	0.0	0.0
0.0	0.0	0.0	0.0	0.0	0.0	0.0	0.0
0.0	0.0	0.0	0.0	0.0	0.0	0.0	0.0
0.0	0.0	0.0	0.0	0.0	0.0	0.0	0.0
0.0	0.0	0.0	0.0	0.0	0.0	0.0	0.0
0.0	0.0	0.0	0.0	0.0	0.0	0.0	0.0
0.0	0.0	0.0	0.0	0.0	0.0	0.0	0.0
0.0	0.0	0.0	0.0	0.0	0.0	0.0	0.0
0.0	0.0	0.0	0.0	0.0	0.0	0.0	0.0
0.0	0.0	0.0	0.0	0.0	0.0	0.0	0.0
0.0	0.0	0.0	0.0	0.0	0.0	0.0	0.0
0.0	0.0	0.0	0.0	0.0	0.0	0.0	0.0
0.0	0.0	0.0	0.0	0.0	0.0	0.0	0.0
0.0	0.0	0.0	0.0	0.0	0.0	0.0	0.0
0.0	0.0	0.0	0.0	0.0	0.0	0.0	0.0
0.0	0.0	0.0	0.0	0.0	0.0	0.0	0.0
0.0	0.0	0.0	0.0	0.0	0.0	0.0	0.0
0.0	0.0	0.0	0.0	0.0	0.0	0.0	0.0
0.0	0.0	0.0	0.0	0.0	0.0	0.0	0.0
0.0	0.0	0.0	0.0	0.0	0.0	0.0	0.0
0.0	0.0	0.0	0.0	0.0	0.0	0.0	0.0
0.0	0.0	0.0	0.0	0.0	0.0	0.0	0.0
0.0	0.0	0.0	0.0	0.0	0.0	0.0	0.0
0.0	0.0	0.0	0.0	0.0	0.0	0.0	0.0
0.0	0.0	0.0	0.0	0.0	0.0	0.0	0.0
0.0	0.0	0.0	0.0	0.0	0.0	0.0	0.0
0.0	0.0	0.0	0.0	0.0	0.0	0.0	0.0
0.0	0.0	0.0	0.0	0.0	0.0	0.0	0.0
0.0	0.0	0.0	0.0	0.0	0.0	0.0	0.0
0.0	0.0	0.0	0.0	0.0	0.0	0.0	0.0
0.0	0.0	0.0	0.0	0.0	0.0	0.0	0.0
0.0	0.0	0.0	0.0	0.0	0.0	0.0	0.0
0.0	0.0	0.0	0.0	0.0	0.0	0.0	0.0
0.0	0.0	0.0	0.0	0.0	0.0	0.0	0.0
0.0	0.0	0.0	0.0	0.0	0.0	0.0	0.0

0.0	0.0	0.0	0.0	0.0	0.0	0.0	0.0
0.0	0.0	0.0	0.0	0.0	0.0	0.0	0.0
0.0	0.0	0.0	0.0	0.0	0.0	0.0	0.0
0.0	0.0	0.0	0.0	0.0	0.0	0.0	0.0
0.0	0.0	0.0	0.0	0.0	0.0	0.0	0.0
0.0	0.0	0.0	0.0	0.0	0.0	0.0	0.0
0.0	0.0	0.0	0.0	0.0	0.0	0.0	0.0
0.0	0.0	0.0	0.0	0.0	0.0	0.0	0.0
0.0	0.0	0.0	0.0	0.0	0.0	0.0	0.0
0.0	0.0	0.0	0.0	0.0	0.0	0.0	0.0
0.0	0.0	0.0	0.0	0.0	0.0	0.0	0.0
0.0	0.0	0.0	0.0	0.0	0.0	0.0	0.0
0.0	0.0	0.0	0.0	0.0	0.0	0.0	0.0
0.0	0.0	0.0	0.0	0.0	0.0	0.0	0.0
0.0	0.0	0.0	0.0	0.0	0.0	0.0	0.0
0.0	0.0	0.0	0.0	0.0	0.0	0.0	0.0
0.0	0.0	0.0	0.0	0.0	0.0	0.0	0.0
0.0	0.0	0.0	0.0	0.0	0.0	0.0	0.0
0.0	0.0	0.0	0.0	0.0	0.0	0.0	0.0
0.0	0.0	0.0	0.0	0.0	0.0	0.0	0.0
0.0	0.0	0.0	0.0	0.0	0.0	0.0	0.0
0.0	0.0	0.0	0.0	0.0	0.0	0.0	0.0
0.0	0.0	0.0	0.0	0.0	0.0	0.0	0.0
0.0	0.0	0.0	0.0	0.0	0.0	0.0	0.0
0.0	0.0	0.0	0.0	0.0	0.0	0.0	0.0
0.0	0.0	0.0	0.0	0.0	0.0	0.0	0.0
0.0	0.0	0.0	0.0	0.0	0.0	0.0	0.0
0.0	0.0	0.0	0.0	0.0	0.0	0.0	0.0
0.0	0.0	0.0	0.0	0.0	0.0	0.0	0.0
0.0	0.0	0.0	0.0	0.0	0.0	0.0	0.0
0.0	0.0	0.0	0.0	0.0	0.0	0.0	0.0
0.0	0.0	0.0	0.0	0.0	0.0	0.0	0.0
0.0	0.0	0.0	0.0	0.0	0.0	0.0	0.0
0.0	0.0	0.0	0.0	0.0	0.0	0.0	0.0
0.0	0.0	0.0	0.0	0.0	0.0	0.0	0.0
0.0	0.0	0.0	0.0	0.0	0.0	0.0	0.0
0.0	0.0	0.0	0.0	0.0	0.0	0.0	0.0
0.0	0.0	0.0	0.0	0.0	0.0	0.0	0.0
0.0	0.0	0.0	0.0	0.0	0.0	0.0	0.0
0.0	0.0	0.0	0.0	0.0	0.0	0.0	0.0
0.0	0.0	0.0	0.0	0.0	0.0	0.0	0.0
0.0	0.0	0.0	0.0	0.0	0.0	0.0	0.0
0.0	0.0	0.0	0.0	0.0	0.0	0.0	0.0
0.0	0.0	0.0	0.0	0.0	0.0	0.0	0.0
0.0	0.0	0.0	0.0	0.0	0.0	0.0	0.0
0.0	0.0	0.0	0.0	0.0	0.0	0.0	0.0
0.0	0.0	0.0	0.0	0.0	0.0	0.0	0.0
0.0	0.0	0.0	0.0	0.0	0.0	0.0	0.0

0.0	0.0	0.0	0.0	0.0	0.0	0.0	0.0
0.0	0.0	0.0	0.0	0.0	0.0	0.0	0.0
0.0	0.0	0.0	0.0	0.0	0.0	0.0	0.0
0.0	0.0	0.0	0.0	0.0	0.0	0.0	0.0
0.0	0.0	0.0	0.0	0.0	0.0	0.0	0.0
0.0	0.0	0.0	0.0	0.0	0.0	0.0	0.0
0.0	0.0	0.0	0.0	0.0	0.0	0.0	0.0
0.0	0.0	0.0	0.0	0.0	0.0	0.0	0.0
0.0	0.0	0.0	0.0	0.0	0.0	0.0	0.0
0.0	0.0	0.0	0.0	0.0	0.0	0.0	0.0
0.0	0.0	0.0	0.0	0.0	0.0	0.0	0.0
0.0	0.0	0.0	0.0	0.0	0.0	0.0	0.0
0.0	0.0	0.0	0.0	0.0	0.0	0.0	0.0
0.0	0.0	0.0	0.0	0.0	0.0	0.0	0.0
0.0	0.0	0.0	0.0	0.0	0.0	0.0	0.0
0.0	0.0	0.0	0.0	0.0	0.0	0.0	0.0
0.0	0.0	0.0	0.0	0.0	0.0	0.0	0.0
0.0	0.0	0.0	0.0	0.0	0.0	0.0	0.0
0.0	0.0	0.0	0.0	0.0	0.0	0.0	0.0
0.0	0.0	0.0	0.0	0.0	0.0	0.0	0.0
0.0	0.0	0.0	0.0	0.0	0.0	0.0	0.0
0.0	0.0	0.0	0.0	0.0	0.0	0.0	0.0
0.0	0.0	0.0	0.0	0.0	0.0	0.0	0.0
0.0	0.0	0.0	0.0	0.0	0.0	0.0	0.0
0.0	0.0	0.0	0.0	0.0	0.0	0.0	0.0
0.0	0.0	0.0	0.0	0.0	0.0	0.0	0.0
0.0	0.0	0.0	0.0	0.0	0.0	0.0	0.0
0.0	0.0	0.0	0.0	0.0	0.0	0.0	0.0
0.0	0.0	0.0	0.0	0.0	0.0	0.0	0.0
0.0	0.0	0.0	0.0	0.0	0.0	0.0	0.0
0.0	0.0	0.0	0.0	0.0	0.0	0.0	0.0
0.0	0.0	0.0	0.0	0.0	0.0	0.0	0.0
0.0	0.0	0.0	0.0	0.0	0.0	0.0	0.0
0.0	0.0	0.0	0.0	0.0	0.0	0.0	0.0
0.0	0.0	0.0	0.0	0.0	0.0	0.0	0.0
0.0	0.0	0.0	0.0	0.0	0.0	0.0	0.0
0.0	0.0	0.0	0.0	0.0	0.0	0.0	0.0
0.0	0.0	0.0	0.0	0.0	0.0	0.0	0.0
0.0	0.0	0.0	0.0	0.0	0.0	0.0	0.0
0.0	0.0	0.0	0.0	0.0	0.0	0.0	0.0
0.0	0.0	0.0	0.0	0.0	0.0	0.0	0.0
0.0	0.0	0.0	0.0	0.0	0.0	0.0	0.0
0.0	0.0	0.0	0.0	0.0	0.0	0.0	0.0
0.0	0.0	0.0	0.0	0.0	0.0	0.0	0.0
0.0	0.0	0.0	0.0	0.0	0.0	0.0	0.0
0.0	0.0	0.0	0.0	0.0	0.0	0.0	0.0
0.0	0.0	0.0	0.0	0.0	0.0	0.0	0.0
0.0	0.0	0.0	0.0	0.0	0.0	0.0	0.0

0.0	0.0	0.0	0.0	0.0	0.0	0.0	0.0
0.0	0.0	0.0	0.0	0.0	0.0	0.0	0.0
0.0	0.0	0.0	0.0	0.0	0.0	0.0	0.0
0.0	0.0	0.0	0.0	0.0	0.0	0.0	0.0
0.0	0.0	0.0	0.0	0.0	0.0	0.0	0.0
0.0	0.0	0.0	0.0	0.0	0.0	0.0	0.0
0.0	0.0	0.0	0.0	0.0	0.0	0.0	0.0
0.0	0.0	0.0	0.0	0.0	0.0	0.0	0.0
0.0	0.0	0.0	0.0	0.0	0.0	0.0	0.0
0.0	0.0	0.0	0.0	0.0	0.0	0.0	0.0
0.0	0.0	0.0	0.0	0.0	0.0	0.0	0.0
0.0	0.0	0.0	0.0	0.0	0.0	0.0	0.0
0.0	0.0	0.0	0.0	0.0	0.0	0.0	0.0
0.0	0.0	0.0	0.0	0.0	0.0	0.0	0.0
0.0	0.0	0.0	0.0	0.0	0.0	0.0	0.0
0.0	0.0	0.0	0.0	0.0	0.0	0.0	0.0
0.0	0.0	0.0	0.0	0.0	0.0	0.0	0.0
0.0	0.0	0.0	0.0	0.0	0.0	0.0	0.0
0.0	0.0	0.0	0.0	0.0	0.0	0.0	0.0
0.0	0.0	0.0	0.0	0.0	0.0	0.0	0.0
0.0	0.0	0.0	0.0	0.0	0.0	0.0	0.0
0.0	0.0	0.0	0.0	0.0	0.0	0.0	0.0
0.0	0.0	0.0	0.0	0.0	0.0	0.0	0.0
0.0	0.0	0.0	0.0	0.0	0.0	0.0	0.0
0.0	0.0	0.0	0.0	0.0	0.0	0.0	0.0
0.0	0.0	0.0	0.0	0.0	0.0	0.0	0.0
0.0	0.0	0.0	0.0	0.0	0.0	0.0	0.0
0.0	0.0	0.0	0.0	0.0	0.0	0.0	0.0
0.0	0.0	0.0	0.0	0.0	0.0	0.0	0.0
0.0	0.0	0.0	0.0	0.0	0.0	0.0	0.0
0.0	0.0	0.0	0.0	0.0	0.0	0.0	0.0
0.0	0.0	0.0	0.0	0.0	0.0	0.0	0.0
0.0	0.0	0.0	0.0	0.0	0.0	0.0	0.0
0.0	0.0	0.0	0.0	0.0	0.0	0.0	0.0
0.0	0.0	0.0	0.0	0.0	0.0	0.0	0.0
0.0	0.0	0.0	0.0	0.0	0.0	0.0	0.0
0.0	0.0	0.0	0.0	0.0	0.0	0.0	0.0
0.0	0.0	0.0	0.0	0.0	0.0	0.0	0.0
0.0	0.0	0.0	0.0	0.0	0.0	0.0	0.0
0.0	0.0	0.0	0.0	0.0	0.0	0.0	0.0
0.0	0.0	0.0	0.0	0.0	0.0	0.0	0.0
0.0	0.0	0.0	0.0	0.0	0.0	0.0	0.0
0.0	0.0	0.0	0.0	0.0	0.0	0.0	0.0
0.0	0.0	0.0	0.0	0.0	0.0	0.0	0.0
0.0	0.0	0.0	0.0	0.0	0.0	0.0	0.0
0.0	0.0	0.0	0.0	0.0	0.0	0.0	0.0
0.0	0.0	0.0	0.0	0.0	0.0	0.0	0.0

0.0	0.0	0.0	0.0	0.0	0.0	0.0	0.0
0.0	0.0	0.0	0.0	0.0	0.0	0.0	0.0
0.0	0.0	0.0	0.0	0.0	0.0	0.0	0.0
0.0	0.0	0.0	0.0	0.0	0.0	0.0	0.0
0.0	0.0	0.0	0.0	0.0	0.0	0.0	0.0
0.0	0.0	0.0	0.0	0.0	0.0	0.0	0.0
0.0	0.0	0.0	0.0	0.0	0.0	0.0	0.0
0.0	0.0	0.0	0.0	0.0	0.0	0.0	0.0
0.0	0.0	0.0	0.0	0.0	0.0	0.0	0.0
0.0	0.0	0.0	0.0	0.0	0.0	0.0	0.0
0.0	0.0	0.0	0.0	0.0	0.0	0.0	0.0
0.0	0.0	0.0	0.0	0.0	0.0	0.0	0.0
0.0	0.0	0.0	0.0	0.0	0.0	0.0	0.0
0.0	0.0	0.0	0.0	0.0	0.0	0.0	0.0
0.0	0.0	0.0	0.0	0.0	0.0	0.0	0.0
0.0	0.0	0.0	0.0	0.0	0.0	0.0	0.0
0.0	0.0	0.0	0.0	0.0	0.0	0.0	0.0
0.0	0.0	0.0	0.0	0.0	0.0	0.0	0.0
0.0	0.0	0.0	0.0	0.0	0.0	0.0	0.0
0.0	0.0	0.0	0.0	0.0	0.0	0.0	0.0
0.0	0.0	0.0	0.0	0.0	0.0	0.0	0.0
0.0	0.0	0.0	0.0	0.0	0.0	0.0	0.0
0.0	0.0	0.0	0.0	0.0	0.0	0.0	0.0
0.0	0.0	0.0	0.0	0.0	0.0	0.0	0.0
0.0	0.0	0.0	0.0	0.0	0.0	0.0	0.0
0.0	0.0	0.0	0.0	0.0	0.0	0.0	0.0
0.0	0.0	0.0	0.0	0.0	0.0	0.0	0.0
0.0	0.0	0.0	0.0	0.0	0.0	0.0	0.0
0.0	0.0	0.0	0.0	0.0	0.0	0.0	0.0
0.0	0.0	0.0	0.0	0.0	0.0	0.0	0.0
0.0	0.0	0.0	0.0	0.0	0.0	0.0	0.0
0.0	0.0	0.0	0.0	0.0	0.0	0.0	0.0
0.0	0.0	0.0	0.0	0.0	0.0	0.0	0.0
0.0	0.0	0.0	0.0	0.0	0.0	0.0	0.0
0.0	0.0	0.0	0.0	0.0	0.0	0.0	0.0
0.0	0.0	0.0	0.0	0.0	0.0	0.0	0.0
0.0	0.0	0.0	0.0	0.0	0.0	0.0	0.0
0.0	0.0	0.0	0.0	0.0	0.0	0.0	0.0
0.0	0.0	0.0	0.0	0.0	0.0	0.0	0.0
0.0	0.0	0.0	0.0	0.0	0.0	0.0	0.0
0.0	0.0	0.0	0.0	0.0	0.0	0.0	0.0
0.0	0.0	0.0	0.0	0.0	0.0	0.0	0.0
0.0	0.0	0.0	0.0	0.0	0.0	0.0	0.0
0.0	0.0	0.0	0.0	0.0	0.0	0.0	0.0
0.0	0.0	0.0	0.0	0.0	0.0	0.0	0.0
0.0	0.0	0.0	0.0	0.0	0.0	0.0	0.0
0.0	0.0	0.0	0.0	0.0	0.0	0.0	0.0

0.0	0.0	0.0	0.0	0.0	0.0	0.0	0.0
0.0	0.0	0.0	0.0	0.0	0.0	0.0	0.0
0.0	0.0	0.0	0.0	0.0	0.0	0.0	0.0
0.0	0.0	0.0	0.0	0.0	0.0	0.0	0.0
0.0	0.0	0.0	0.0	0.0	0.0	0.0	0.0
0.0	0.0	0.0	0.0	0.0	0.0	0.0	0.0
0.0	0.0	0.0	0.0	0.0	0.0	0.0	0.0
0.0	0.0	0.0	0.0	0.0	0.0	0.0	0.0
0.0	0.0	0.0	0.0	0.0	0.0	0.0	0.0
0.0	0.0	0.0	0.0	0.0	0.0	0.0	0.0
0.0	0.0	0.0	0.0	0.0	0.0	0.0	0.0
0.0	0.0	0.0	0.0	0.0	0.0	0.0	0.0
0.0	0.0	0.0	0.0	0.0	0.0	0.0	0.0
0.0	0.0	0.0	0.0	0.0	0.0	0.0	0.0
0.0	0.0	0.0	0.0	0.0	0.0	0.0	0.0
0.0	0.0	0.0	0.0	0.0	0.0	0.0	0.0
0.0	0.0	0.0	0.0	0.0	0.0	0.0	0.0
0.0	0.0	0.0	0.0	0.0	0.0	0.0	0.0
0.0	0.0	0.0	0.0	0.0	0.0	0.0	0.0
0.0	0.0	0.0	0.0	0.0	0.0	0.0	0.0
0.0	0.0	0.0	0.0	0.0	0.0	0.0	0.0
0.0	0.0	0.0	0.0	0.0	0.0	0.0	0.0
0.0	0.0	0.0	0.0	0.0	0.0	0.0	0.0
0.0	0.0	0.0	0.0	0.0	0.0	0.0	0.0
0.0	0.0	0.0	0.0	0.0	0.0	0.0	0.0
0.0	0.0	0.0	0.0	0.0	0.0	0.0	0.0
0.0	0.0	0.0	0.0	0.0	0.0	0.0	0.0
0.0	0.0	0.0	0.0	0.0	0.0	0.0	0.0
0.0	0.0	0.0	0.0	0.0	0.0	0.0	0.0
0.0	0.0	0.0	0.0	0.0	0.0	0.0	0.0
0.0	0.0	0.0	0.0	0.0	0.0	0.0	0.0
0.0	0.0	0.0	0.0	0.0	0.0	0.0	0.0
0.0	0.0	0.0	0.0	0.0	0.0	0.0	0.0
0.0	0.0	0.0	0.0	0.0	0.0	0.0	0.0
0.0	0.0	0.0	0.0	0.0	0.0	0.0	0.0
0.0	0.0	0.0	0.0	0.0	0.0	0.0	0.0
0.0	0.0	0.0	0.0	0.0	0.0	0.0	0.0
0.0	0.0	0.0	0.0	0.0	0.0	0.0	0.0
0.0	0.0	0.0	0.0	0.0	0.0	0.0	0.0
0.0	0.0	0.0	0.0	0.0	0.0	0.0	0.0
0.0	0.0	0.0	0.0	0.0	0.0	0.0	0.0
0.0	0.0	0.0	0.0	0.0	0.0	0.0	0.0
0.0	0.0	0.0	0.0	0.0	0.0	0.0	0.0
0.0	0.0	0.0	0.0	0.0	0.0	0.0	0.0
0.0	0.0	0.0	0.0	0.0	0.0	0.0	0.0
0.0	0.0	0.0	0.0	0.0	0.0	0.0	0.0
0.0	0.0	0.0	0.0	0.0	0.0	0.0	0.0
0.0	0.0	0.0	0.0	0.0	0.0	0.0	0.0

0.0	0.0	0.0	0.0	0.0	0.0	0.0	0.0
0.0	0.0	0.0	0.0	0.0	0.0	0.0	0.0
0.0	0.0	0.0	0.0	0.0	0.0	0.0	0.0
0.0	0.0	0.0	0.0	0.0	0.0	0.0	0.0
0.0	0.0	0.0	0.0	0.0	0.0	0.0	0.0
0.0	0.0	0.0	0.0	0.0	0.0	0.0	0.0
0.0	0.0	0.0	0.0	0.0	0.0	0.0	0.0
0.0	0.0	0.0	0.0	0.0	0.0	0.0	0.0
0.0	0.0	0.0	0.0	0.0	0.0	0.0	0.0
0.0	0.0	0.0	0.0	0.0	0.0	0.0	0.0
0.0	0.0	0.0	0.0	0.0	0.0	0.0	0.0
0.0	0.0	0.0	0.0	0.0	0.0	0.0	0.0
0.0	0.0	0.0	0.0	0.0	0.0	0.0	0.0
0.0	0.0	0.0	0.0	0.0	0.0	0.0	0.0
0.0	0.0	0.0	0.0	0.0	0.0	0.0	0.0
0.0	0.0	0.0	0.0	0.0	0.0	0.0	0.0
0.0	0.0	0.0	0.0	0.0	0.0	0.0	0.0
0.0	0.0	0.0	0.0	0.0	0.0	0.0	0.0
0.0	0.0	0.0	0.0	0.0	0.0	0.0	0.0
0.0	0.0	0.0	0.0	0.0	0.0	0.0	0.0
0.0	0.0	0.0	0.0	0.0	0.0	0.0	0.0
0.0	0.0	0.0	0.0	0.0	0.0	0.0	0.0
0.0	0.0	0.0	0.0	0.0	0.0	0.0	0.0
0.0	0.0	0.0	0.0	0.0	0.0	0.0	0.0
0.0	0.0	0.0	0.0	0.0	0.0	0.0	0.0
0.0	0.0	0.0	0.0	0.0	0.0	0.0	0.0
0.0	0.0	0.0	0.0	0.0	0.0	0.0	0.0
0.0	0.0	0.0	0.0	0.0	0.0	0.0	0.0
0.0	0.0	0.0	0.0	0.0	0.0	0.0	0.0
0.0	0.0	0.0	0.0	0.0	0.0	0.0	0.0
0.0	0.0	0.0	0.0	0.0	0.0	0.0	0.0
0.0	0.0	0.0	0.0	0.0	0.0	0.0	0.0
0.0	0.0	0.0	0.0	0.0	0.0	0.0	0.0
0.0	0.0	0.0	0.0	0.0	0.0	0.0	0.0
0.0	0.0	0.0	0.0	0.0	0.0	0.0	0.0
0.0	0.0	0.0	0.0	0.0	0.0	0.0	0.0
0.0	0.0	0.0	0.0	0.0	0.0	0.0	0.0
0.0	0.0	0.0	0.0	0.0	0.0	0.0	0.0
0.0	0.0	0.0	0.0	0.0	0.0	0.0	0.0
0.0	0.0	0.0	0.0	0.0	0.0	0.0	0.0
0.0	0.0	0.0	0.0	0.0	0.0	0.0	0.0
0.0	0.0	0.0	0.0	0.0	0.0	0.0	0.0
0.0	0.0	0.0	0.0	0.0	0.0	0.0	0.0
0.0	0.0	0.0	0.0	0.0	0.0	0.0	0.0
0.0	0.0	0.0	0.0	0.0	0.0	0.0	0.0
0.0	0.0	0.0	0.0	0.0	0.0	0.0	0.0
0.0	0.0	0.0	0.0	0.0	0.0	0.0	0.0
0.0	0.0	0.0	0.0	0.0	0.0	0.0	0.0

0.0	0.0	0.0	0.0	0.0	0.0	0.0	0.0
0.0	0.0	0.0	0.0	0.0	0.0	0.0	0.0
0.0	0.0	0.0	0.0	0.0	0.0	0.0	0.0
0.0	0.0	0.0	0.0	0.0	0.0	0.0	0.0
0.0	0.0	0.0	0.0	0.0	0.0	0.0	0.0
0.0	0.0	0.0	0.0	0.0	0.0	0.0	0.0
0.0	0.0	0.0	0.0	0.0	0.0	0.0	0.0
0.0	0.0	0.0	0.0	0.0	0.0	0.0	0.0
0.0	0.0	0.0	0.0	0.0	0.0	0.0	0.0
0.0	0.0	0.0	0.0	0.0	0.0	0.0	0.0
0.0	0.0	0.0	0.0	0.0	0.0	0.0	0.0
0.0	0.0	0.0	0.0	0.0	0.0	0.0	0.0
0.0	0.0	0.0	0.0	0.0	0.0	0.0	0.0
0.0	0.0	0.0	0.0	0.0	0.0	0.0	0.0
0.0	0.0	0.0	0.0	0.0	0.0	0.0	0.0
0.0	0.0	0.0	0.0	0.0	0.0	0.0	0.0
0.0	0.0	0.0	0.0	0.0	0.0	0.0	0.0
0.0	0.0	0.0	0.0	0.0	0.0	0.0	0.0
0.0	0.0	0.0	0.0	0.0	0.0	0.0	0.0
0.0	0.0	0.0	0.0	0.0	0.0	0.0	0.0
0.0	0.0	0.0	0.0	0.0	0.0	0.0	0.0
0.0	0.0	0.0	0.0	0.0	0.0	0.0	0.0
0.0	0.0	0.0	0.0	0.0	0.0	0.0	0.0
0.0	0.0	0.0	0.0	0.0	0.0	0.0	0.0
0.0	0.0	0.0	0.0	0.0	0.0	0.0	0.0
0.0	0.0	0.0	0.0	0.0	0.0	0.0	0.0
0.0	0.0	0.0	0.0	0.0	0.0	0.0	0.0
0.0	0.0	0.0	0.0	0.0	0.0	0.0	0.0
0.0	0.0	0.0	0.0	0.0	0.0	0.0	0.0
0.0	0.0	0.0	0.0	0.0	0.0	0.0	0.0
0.0	0.0	0.0	0.0	0.0	0.0	0.0	0.0
0.0	0.0	0.0	0.0	0.0	0.0	0.0	0.0
0.0	0.0	0.0	0.0	0.0	0.0	0.0	0.0
0.0	0.0	0.0	0.0	0.0	0.0	0.0	0.0
0.0	0.0	0.0	0.0	0.0	0.0	0.0	0.0
0.0	0.0	0.0	0.0	0.0	0.0	0.0	0.0
0.0	0.0	0.0	0.0	0.0	0.0	0.0	0.0
0.0	0.0	0.0	0.0	0.0	0.0	0.0	0.0
0.0	0.0	0.0	0.0	0.0	0.0	0.0	0.0
0.0	0.0	0.0	0.0	0.0	0.0	0.0	0.0
0.0	0.0	0.0	0.0	0.0	0.0	0.0	0.0
0.0	0.0	0.0	0.0	0.0	0.0	0.0	0.0
0.0	0.0	0.0	0.0	0.0	0.0	0.0	0.0
0.0	0.0	0.0	0.0	0.0	0.0	0.0	0.0
0.0	0.0	0.0	0.0	0.0	0.0	0.0	0.0
0.0	0.0	0.0	0.0	0.0	0.0	0.0	0.0
0.0	0.0	0.0	0.0	0.0	0.0	0.0	0.0
0.0	0.0	0.0	0.0	0.0	0.0	0.0	0.0

0.0	0.0	0.0	0.0	0.0	0.0	0.0	0.0
0.0	0.0	0.0	0.0	0.0	0.0	0.0	0.0
0.0	0.0	0.0	0.0	0.0	0.0	0.0	0.0
0.0	0.0	0.0	0.0	0.0	0.0	0.0	0.0
0.0	0.0	0.0	0.0	0.0	0.0	0.0	0.0
0.0	0.0	0.0	0.0	0.0	0.0	0.0	0.0
0.0	0.0	0.0	0.0	0.0	0.0	0.0	0.0
0.0	0.0	0.0	0.0	0.0	0.0	0.0	0.0
0.0	0.0	0.0	0.0	0.0	0.0	0.0	0.0
0.0	0.0	0.0	0.0	0.0	0.0	0.0	0.0
0.0	0.0	0.0	0.0	0.0	0.0	0.0	0.0
0.0	0.0	0.0	0.0	0.0	0.0	0.0	0.0
0.0	0.0	0.0	0.0	0.0	0.0	0.0	0.0
0.0	0.0	0.0	0.0	0.0	0.0	0.0	0.0
0.0	0.0	0.0	0.0	0.0	0.0	0.0	0.0
0.0	0.0	0.0	0.0	0.0	0.0	0.0	0.0
0.0	0.0	0.0	0.0	0.0	0.0	0.0	0.0
0.0	0.0	0.0	0.0	0.0	0.0	0.0	0.0
0.0	0.0	0.0	0.0	0.0	0.0	0.0	0.0
0.0	0.0	0.0	0.0	0.0	0.0	0.0	0.0
0.0	0.0	0.0	0.0	0.0	0.0	0.0	0.0
0.0	0.0	0.0	0.0	0.0	0.0	0.0	0.0
0.0	0.0	0.0	0.0	0.0	0.0	0.0	0.0
0.0	0.0	0.0	0.0	0.0	0.0	0.0	0.0
0.0	0.0	0.0	0.0	0.0	0.0	0.0	0.0
0.0	0.0	0.0	0.0	0.0	0.0	0.0	0.0
0.0	0.0	0.0	0.0	0.0	0.0	0.0	0.0
0.0	0.0	0.0	0.0	0.0	0.0	0.0	0.0
0.0	0.0	0.0	0.0	0.0	0.0	0.0	0.0
0.0	0.0	0.0	0.0	0.0	0.0	0.0	0.0
0.0	0.0	0.0	0.0	0.0	0.0	0.0	0.0
0.0	0.0	0.0	0.0	0.0	0.0	0.0	0.0
0.0	0.0	0.0	0.0	0.0	0.0	0.0	0.0
0.0	0.0	0.0	0.0	0.0	0.0	0.0	0.0
0.0	0.0	0.0	0.0	0.0	0.0	0.0	0.0
0.0	0.0	0.0	0.0	0.0	0.0	0.0	0.0
0.0	0.0	0.0	0.0	0.0	0.0	0.0	0.0
0.0	0.0	0.0	0.0	0.0	0.0	0.0	0.0
0.0	0.0	0.0	0.0	0.0	0.0	0.0	0.0
0.0	0.0	0.0	0.0	0.0	0.0	0.0	0.0
0.0	0.0	0.0	0.0	0.0	0.0	0.0	0.0
0.0	0.0	0.0	0.0	0.0	0.0	0.0	0.0
0.0	0.0	0.0	0.0	0.0	0.0	0.0	0.0
0.0	0.0	0.0	0.0	0.0	0.0	0.0	0.0
0.0	0.0	0.0	0.0	0.0	0.0	0.0	0.0
0.0	0.0	0.0	0.0	0.0	0.0	0.0	0.0
0.0	0.0	0.0	0.0	0.0	0.0	0.0	0.0
0.0	0.0	0.0	0.0	0.0	0.0	0.0	0.0

0.0	0.0	0.0	0.0	0.0	0.0	0.0	0.0
0.0	0.0	0.0	0.0	0.0	0.0	0.0	0.0
0.0	0.0	0.0	0.0	0.0	0.0	0.0	0.0
0.0	0.0	0.0	0.0	0.0	0.0	0.0	0.0
0.0	0.0	0.0	0.0	0.0	0.0	0.0	0.0
0.0	0.0	0.0	0.0	0.0	0.0	0.0	0.0
0.0	0.0	0.0	0.0	0.0	0.0	0.0	0.0
0.0	0.0	0.0	0.0	0.0	0.0	0.0	0.0
0.0	0.0	0.0	0.0	0.0	0.0	0.0	0.0
0.0	0.0	0.0	0.0	0.0	0.0	0.0	0.0
0.0	0.0	0.0	0.0	0.0	0.0	0.0	0.0
0.0	0.0	0.0	0.0	0.0	0.0	0.0	0.0
0.0	0.0	0.0	0.0	0.0	0.0	0.0	0.0
0.0	0.0	0.0	0.0	0.0	0.0	0.0	0.0
0.0	0.0	0.0	0.0	0.0	0.0	0.0	0.0
0.0	0.0	0.0	0.0	0.0	0.0	0.0	0.0
0.0	0.0	0.0	0.0	0.0	0.0	0.0	0.0
0.0	0.0	0.0	0.0	0.0	0.0	0.0	0.0
0.0	0.0	0.0	0.0	0.0	0.0	0.0	0.0
0.0	0.0	0.0	0.0	0.0	0.0	0.0	0.0
0.0	0.0	0.0	0.0	0.0	0.0	0.0	0.0
0.0	0.0	0.0	0.0	0.0	0.0	0.0	0.0
0.0	0.0	0.0	0.0	0.0	0.0	0.0	0.0
0.0	0.0	0.0	0.0	0.0	0.0	0.0	0.0
0.0	0.0	0.0	0.0	0.0	0.0	0.0	0.0
0.0	0.0	0.0	0.0	0.0	0.0	0.0	0.0
0.0	0.0	0.0	0.0	0.0	0.0	0.0	0.0
0.0	0.0	0.0	0.0	0.0	0.0	0.0	0.0
0.0	0.0	0.0	0.0	0.0	0.0	0.0	0.0
0.0	0.0	0.0	0.0	0.0	0.0	0.0	0.0
0.0	0.0	0.0	0.0	0.0	0.0	0.0	0.0
0.0	0.0	0.0	0.0	0.0	0.0	0.0	0.0
0.0	0.0	0.0	0.0	0.0	0.0	0.0	0.0
0.0	0.0	0.0	0.0	0.0	0.0	0.0	0.0
0.0	0.0	0.0	0.0	0.0	0.0	0.0	0.0
0.0	0.0	0.0	0.0	0.0	0.0	0.0	0.0
0.0	0.0	0.0	0.0	0.0	0.0	0.0	0.0
0.0	0.0	0.0	0.0	0.0	0.0	0.0	0.0
0.0	0.0	0.0	0.0	0.0	0.0	0.0	0.0
0.0	0.0	0.0	0.0	0.0	0.0	0.0	0.0
0.0	0.0	0.0	0.0	0.0	0.0	0.0	0.0
0.0	0.0	0.0	0.0	0.0	0.0	0.0	0.0
0.0	0.0	0.0	0.0	0.0	0.0	0.0	0.0
0.0	0.0	0.0	0.0	0.0	0.0	0.0	0.0
0.0	0.0	0.0	0.0	0.0	0.0	0.0	0.0
0.0	0.0	0.0	0.0	0.0	0.0	0.0	0.0
0.0	0.0	0.0	0.0	0.0	0.0	0.0	0.0

0.0	0.0	0.0	0.0	0.0	0.0	0.0	0.0
0.0	0.0	0.0	0.0	0.0	0.0	0.0	0.0
0.0	0.0	0.0	0.0	0.0	0.0	0.0	0.0
0.0	0.0	0.0	0.0	0.0	0.0	0.0	0.0
0.0	0.0	0.0	0.0	0.0	0.0	0.0	0.0
0.0	0.0	0.0	0.0	0.0	0.0	0.0	0.0
0.0	0.0	0.0	0.0	0.0	0.0	0.0	0.0
0.0	0.0	0.0	0.0	0.0	0.0	0.0	0.0
0.0	0.0	0.0	0.0	0.0	0.0	0.0	0.0
0.0	0.0	0.0	0.0	0.0	0.0	0.0	0.0
0.0	0.0	0.0	0.0	0.0	0.0	0.0	0.0
0.0	0.0	0.0	0.0	0.0	0.0	0.0	0.0
0.0	0.0	0.0	0.0	0.0	0.0	0.0	0.0
0.0	0.0	0.0	0.0	0.0	0.0	0.0	0.0
0.0	0.0	0.0	0.0	0.0	0.0	0.0	0.0
0.0	0.0	0.0	0.0	0.0	0.0	0.0	0.0
0.0	0.0	0.0	0.0	0.0	0.0	0.0	0.0
0.0	0.0	0.0	0.0	0.0	0.0	0.0	0.0
0.0	0.0	0.0	0.0	0.0	0.0	0.0	0.0
0.0	0.0	0.0	0.0	0.0	0.0	0.0	0.0
0.0	0.0	0.0	0.0	0.0	0.0	0.0	0.0
0.0	0.0	0.0	0.0	0.0	0.0	0.0	0.0
0.0	0.0	0.0	0.0	0.0	0.0	0.0	0.0
0.0	0.0	0.0	0.0	0.0	0.0	0.0	0.0
0.0	0.0	0.0	0.0	0.0	0.0	0.0	0.0
0.0	0.0	0.0	0.0	0.0	0.0	0.0	0.0
0.0	0.0	0.0	0.0	0.0	0.0	0.0	0.0
0.0	0.0	0.0	0.0	0.0	0.0	0.0	0.0
0.0	0.0	0.0	0.0	0.0	0.0	0.0	0.0
0.0	0.0	0.0	0.0	0.0	0.0	0.0	0.0
0.0	0.0	0.0	0.0	0.0	0.0	0.0	0.0
0.0	0.0	0.0	0.0	0.0	0.0	0.0	0.0
0.0	0.0	0.0	0.0	0.0	0.0	0.0	0.0
0.0	0.0	0.0	0.0	0.0	0.0	0.0	0.0
0.0	0.0	0.0	0.0	0.0	0.0	0.0	0.0
0.0	0.0	0.0	0.0	0.0	0.0	0.0	0.0
0.0	0.0	0.0	0.0	0.0	0.0	0.0	0.0
0.0	0.0	0.0	0.0	0.0	0.0	0.0	0.0
0.0	0.0	0.0	0.0	0.0	0.0	0.0	0.0
0.0	0.0	0.0	0.0	0.0	0.0	0.0	0.0
0.0	0.0	0.0	0.0	0.0	0.0	0.0	0.0
0.0	0.0	0.0	0.0	0.0	0.0	0.0	0.0
0.0	0.0	0.0	0.0	0.0	0.0	0.0	0.0
0.0	0.0	0.0	0.0	0.0	0.0	0.0	0.0
0.0	0.0	0.0	0.0	0.0	0.0	0.0	0.0
0.0	0.0	0.0	0.0	0.0	0.0	0.0	0.0
0.0	0.0	0.0	0.0	0.0	0.0	0.0	0.0

0.0	0.0	0.0	0.0	0.0	0.0	0.0	0.0
0.0	0.0	0.0	0.0	0.0	0.0	0.0	0.0
0.0	0.0	0.0	0.0	0.0	0.0	0.0	0.0
0.0	0.0	0.0	0.0	0.0	0.0	0.0	0.0
0.0	0.0	0.0	0.0	0.0	0.0	0.0	0.0
0.0	0.0	0.0	0.0	0.0	0.0	0.0	0.0
0.0	0.0	0.0	0.0	0.0	0.0	0.0	0.0
0.0	0.0	0.0	0.0	0.0	0.0	0.0	0.0
0.0	0.0	0.0	0.0	0.0	0.0	0.0	0.0
0.0	0.0	0.0	0.0	0.0	0.0	0.0	0.0
0.0	0.0	0.0	0.0	0.0	0.0	0.0	0.0
0.0	0.0	0.0	0.0	0.0	0.0	0.0	0.0
0.0	0.0	0.0	0.0	0.0	0.0	0.0	0.0
0.0	0.0	0.0	0.0	0.0	0.0	0.0	0.0
0.0	0.0	0.0	0.0	0.0	0.0	0.0	0.0
0.0	0.0	0.0	0.0	0.0	0.0	0.0	0.0
0.0	0.0	0.0	0.0	0.0	0.0	0.0	0.0
0.0	0.0	0.0	0.0	0.0	0.0	0.0	0.0
0.0	0.0	0.0	0.0	0.0	0.0	0.0	0.0
0.0	0.0	0.0	0.0	0.0	0.0	0.0	0.0
0.0	0.0	0.0	0.0	0.0	0.0	0.0	0.0
0.0	0.0	0.0	0.0	0.0	0.0	0.0	0.0
0.0	0.0	0.0	0.0	0.0	0.0	0.0	0.0
0.0	0.0	0.0	0.0	0.0	0.0	0.0	0.0
0.0	0.0	0.0	0.0	0.0	0.0	0.0	0.0
0.0	0.0	0.0	0.0	0.0	0.0	0.0	0.0
0.0	0.0	0.0	0.0	0.0	0.0	0.0	0.0
0.0	0.0	0.0	0.0	0.0	0.0	0.0	0.0
0.0	0.0	0.0	0.0	0.0	0.0	0.0	0.0
0.0	0.0	0.0	0.0	0.0	0.0	0.0	0.0
0.0	0.0	0.0	0.0	0.0	0.0	0.0	0.0
0.0	0.0	0.0	0.0	0.0	0.0	0.0	0.0
0.0	0.0	0.0	0.0	0.0	0.0	0.0	0.0
0.0	0.0	0.0	0.0	0.0	0.0	0.0	0.0
0.0	0.0	0.0	0.0	0.0	0.0	0.0	0.0
0.0	0.0	0.0	0.0	0.0	0.0	0.0	0.0
0.0	0.0	0.0	0.0	0.0	0.0	0.0	0.0
0.0	0.0	0.0	0.0	0.0	0.0	0.0	0.0
0.0	0.0	0.0	0.0	0.0	0.0	0.0	0.0
0.0	0.0	0.0	0.0	0.0	0.0	0.0	0.0
0.0	0.0	0.0	0.0	0.0	0.0	0.0	0.0
0.0	0.0	0.0	0.0	0.0	0.0	0.0	0.0
0.0	0.0	0.0	0.0	0.0	0.0	0.0	0.0
0.0	0.0	0.0	0.0	0.0	0.0	0.0	0.0
0.0	0.0	0.0	0.0	0.0	0.0	0.0	0.0
0.0	0.0	0.0	0.0	0.0	0.0	0.0	0.0
0.0	0.0	0.0	0.0	0.0	0.0	0.0	0.0

0.0	0.0	0.0	0.0	0.0	0.0	0.0	0.0
0.0	0.0	0.0	0.0	0.0	0.0	0.0	0.0
0.0	0.0	0.0	0.0	0.0	0.0	0.0	0.0
0.0	0.0	0.0	0.0	0.0	0.0	0.0	0.0
0.0	0.0	0.0	0.0	0.0	0.0	0.0	0.0
0.0	0.0	0.0	0.0	0.0	0.0	0.0	0.0
0.0	0.0	0.0	0.0	0.0	0.0	0.0	0.0
0.0	0.0	0.0	0.0	0.0	0.0	0.0	0.0
0.0	0.0	0.0	0.0	0.0	0.0	0.0	0.0
0.0	0.0	0.0	0.0	0.0	0.0	0.0	0.0
0.0	0.0	0.0	0.0	0.0	0.0	0.0	0.0
0.0	0.0	0.0	0.0	0.0	0.0	0.0	0.0
0.0	0.0	0.0	0.0	0.0	0.0	0.0	0.0
0.0	0.0	0.0	0.0	0.0	0.0	0.0	0.0
0.0	0.0	0.0	0.0	0.0	0.0	0.0	0.0
0.0	0.0	0.0	0.0	0.0	0.0	0.0	0.0
0.0	0.0	0.0	0.0	0.0	0.0	0.0	0.0
0.0	0.0	0.0	0.0	0.0	0.0	0.0	0.0
0.0	0.0	0.0	0.0	0.0	0.0	0.0	0.0
0.0	0.0	0.0	0.0	0.0	0.0	0.0	0.0
0.0	0.0	0.0	0.0	0.0	0.0	0.0	0.0
0.0	0.0	0.0	0.0	0.0	0.0	0.0	0.0
0.0	0.0	0.0	0.0	0.0	0.0	0.0	0.0
0.0	0.0	0.0	0.0	0.0	0.0	0.0	0.0
0.0	0.0	0.0	0.0	0.0	0.0	0.0	0.0
0.0	0.0	0.0	0.0	0.0	0.0	0.0	0.0
0.0	0.0	0.0	0.0	0.0	0.0	0.0	0.0
0.0	0.0	0.0	0.0	0.0	0.0	0.0	0.0
0.0	0.0	0.0	0.0	0.0	0.0	0.0	0.0
0.0	0.0	0.0	0.0	0.0	0.0	0.0	0.0
0.0	0.0	0.0	0.0	0.0	0.0	0.0	0.0
0.0	0.0	0.0	0.0	0.0	0.0	0.0	0.0
0.0	0.0	0.0	0.0	0.0	0.0	0.0	0.0
0.0	0.0	0.0	0.0	0.0	0.0	0.0	0.0
0.0	0.0	0.0	0.0	0.0	0.0	0.0	0.0
0.0	0.0	0.0	0.0	0.0	0.0	0.0	0.0
0.0	0.0	0.0	0.0	0.0	0.0	0.0	0.0
0.0	0.0	0.0	0.0	0.0	0.0	0.0	0.0
0.0	0.0	0.0	0.0	0.0	0.0	0.0	0.0
0.0	0.0	0.0	0.0	0.0	0.0	0.0	0.0
0.0	0.0	0.0	0.0	0.0	0.0	0.0	0.0
0.0	0.0	0.0	0.0	0.0	0.0	0.0	0.0
0.0	0.0	0.0	0.0	0.0	0.0	0.0	0.0
0.0	0.0	0.0	0.0	0.0	0.0	0.0	0.0
0.0	0.0	0.0	0.0	0.0	0.0	0.0	0.0
0.0	0.0	0.0	0.0	0.0	0.0	0.0	0.0
0.0	0.0	0.0	0.0	0.0	0.0	0.0	0.0
0.0	0.0	0.0	0.0	0.0	0.0	0.0	0.0

0.0	0.0	0.0	0.0	0.0	0.0	0.0	0.0
0.0	0.0	0.0	0.0	0.0	0.0	0.0	0.0
0.0	0.0	0.0	0.0	0.0	0.0	0.0	0.0
0.0	0.0	0.0	0.0	0.0	0.0	0.0	0.0
0.0	0.0	0.0	0.0	0.0	0.0	0.0	0.0
0.0	0.0	0.0	0.0	0.0	0.0	0.0	0.0
0.0	0.0	0.0	0.0	0.0	0.0	0.0	0.0
0.0	0.0	0.0	0.0	0.0	0.0	0.0	0.0
0.0	0.0	0.0	0.0	0.0	0.0	0.0	0.0
0.0	0.0	0.0	0.0	0.0	0.0	0.0	0.0
0.0	0.0	0.0	0.0	0.0	0.0	0.0	0.0
0.0	0.0	0.0	0.0	0.0	0.0	0.0	0.0
0.0	0.0	0.0	0.0	0.0	0.0	0.0	0.0
0.0	0.0	0.0	0.0	0.0	0.0	0.0	0.0
0.0	0.0	0.0	0.0	0.0	0.0	0.0	0.0
0.0	0.0	0.0	0.0	0.0	0.0	0.0	0.0
0.0	0.0	0.0	0.0	0.0	0.0	0.0	0.0
0.0	0.0	0.0	0.0	0.0	0.0	0.0	0.0
0.0	0.0	0.0	0.0	0.0	0.0	0.0	0.0
0.0	0.0	0.0	0.0	0.0	0.0	0.0	0.0
0.0	0.0	0.0	0.0	0.0	0.0	0.0	0.0
0.0	0.0	0.0	0.0	0.0	0.0	0.0	0.0
0.0	0.0	0.0	0.0	0.0	0.0	0.0	0.0
0.0	0.0	0.0	0.0	0.0	0.0	0.0	0.0
0.0	0.0	0.0	0.0	0.0	0.0	0.0	0.0
0.0	0.0	0.0	0.0	0.0	0.0	0.0	0.0
0.0	0.0	0.0	0.0	0.0	0.0	0.0	0.0
0.0	0.0	0.0	0.0	0.0	0.0	0.0	0.0
0.0	0.0	0.0	0.0	0.0	0.0	0.0	0.0
0.0	0.0	0.0	0.0	0.0	0.0	0.0	0.0
0.0	0.0	0.0	0.0	0.0	0.0	0.0	0.0
0.0	0.0	0.0	0.0	0.0	0.0	0.0	0.0
0.0	0.0	0.0	0.0	0.0	0.0	0.0	0.0
0.0	0.0	0.0	0.0	0.0	0.0	0.0	0.0
0.0	0.0	0.0	0.0	0.0	0.0	0.0	0.0
0.0	0.0	0.0	0.0	0.0	0.0	0.0	0.0
0.0	0.0	0.0	0.0	0.0	0.0	0.0	0.0
0.0	0.0	0.0	0.0	0.0	0.0	0.0	0.0
0.0	0.0	0.0	0.0	0.0	0.0	0.0	0.0
0.0	0.0	0.0	0.0	0.0	0.0	0.0	0.0
0.0	0.0	0.0	0.0	0.0	0.0	0.0	0.0
0.0	0.0	0.0	0.0	0.0	0.0	0.0	0.0
0.0	0.0	0.0	0.0	0.0	0.0	0.0	0.0
0.0	0.0	0.0	0.0	0.0	0.0	0.0	0.0
0.0	0.0	0.0	0.0	0.0	0.0	0.0	0.0
0.0	0.0	0.0	0.0	0.0	0.0	0.0	0.0
0.0	0.0	0.0	0.0	0.0	0.0	0.0	0.0

0.0	0.0	0.0	0.0	0.0	0.0	0.0	0.0
0.0	0.0	0.0	0.0	0.0	0.0	0.0	0.0
0.0	0.0	0.0	0.0	0.0	0.0	0.0	0.0
0.0	0.0	0.0	0.0	0.0	0.0	0.0	0.0
0.0	0.0	0.0	0.0	0.0	0.0	0.0	0.0
0.0	0.0	0.0	0.0	0.0	0.0	0.0	0.0
0.0	0.0	0.0	0.0	0.0	0.0	0.0	0.0
0.0	0.0	0.0	0.0	0.0	0.0	0.0	0.0
0.0	0.0	0.0	0.0	0.0	0.0	0.0	0.0
0.0	0.0	0.0	0.0	0.0	0.0	0.0	0.0
0.0	0.0	0.0	0.0	0.0	0.0	0.0	0.0
0.0	0.0	0.0	0.0	0.0	0.0	0.0	0.0
0.0	0.0	0.0	0.0	0.0	0.0	0.0	0.0
0.0	0.0	0.0	0.0	0.0	0.0	0.0	0.0
0.0	0.0	0.0	0.0	0.0	0.0	0.0	0.0
0.0	0.0	0.0	0.0	0.0	0.0	0.0	0.0
0.0	0.0	0.0	0.0	0.0	0.0	0.0	0.0
0.0	0.0	0.0	0.0	0.0	0.0	0.0	0.0
0.0	0.0	0.0	0.0	0.0	0.0	0.0	0.0
0.0	0.0	0.0	0.0	0.0	0.0	0.0	0.0
0.0	0.0	0.0	0.0	0.0	0.0	0.0	0.0
0.0	0.0	0.0	0.0	0.0	0.0	0.0	0.0
0.0	0.0	0.0	0.0	0.0	0.0	0.0	0.0
0.0	0.0	0.0	0.0	0.0	0.0	0.0	0.0
0.0	0.0	0.0	0.0	0.0	0.0	0.0	0.0
0.0	0.0	0.0	0.0	0.0	0.0	0.0	0.0
0.0	0.0	0.0	0.0	0.0	0.0	0.0	0.0
0.0	0.0	0.0	0.0	0.0	0.0	0.0	0.0
0.0	0.0	0.0	0.0	0.0	0.0	0.0	0.0
0.0	0.0	0.0	0.0	0.0	0.0	0.0	0.0
0.0	0.0	0.0	0.0	0.0	0.0	0.0	0.0
0.0	0.0	0.0	0.0	0.0	0.0	0.0	0.0
0.0	0.0	0.0	0.0	0.0	0.0	0.0	0.0
0.0	0.0	0.0	0.0	0.0	0.0	0.0	0.0
0.0	0.0	0.0	0.0	0.0	0.0	0.0	0.0
0.0	0.0	0.0	0.0	0.0	0.0	0.0	0.0
0.0	0.0	0.0	0.0	0.0	0.0	0.0	0.0
0.0	0.0	0.0	0.0	0.0	0.0	0.0	0.0
0.0	0.0	0.0	0.0	0.0	0.0	0.0	0.0
0.0	0.0	0.0	0.0	0.0	0.0	0.0	0.0
0.0	0.0	0.0	0.0	0.0	0.0	0.0	0.0
0.0	0.0	0.0	0.0	0.0	0.0	0.0	0.0
0.0	0.0	0.0	0.0	0.0	0.0	0.0	0.0
0.0	0.0	0.0	0.0	0.0	0.0	0.0	0.0
0.0	0.0	0.0	0.0	0.0	0.0	0.0	0.0
0.0	0.0	0.0	0.0	0.0	0.0	0.0	0.0
0.0	0.0	0.0	0.0	0.0	0.0	0.0	0.0

0.0	0.0	0.0	0.0	0.0	0.0	0.0	0.0
0.0	0.0	0.0	0.0	0.0	0.0	0.0	0.0
0.0	0.0	0.0	0.0	0.0	0.0	0.0	0.0
0.0	0.0	0.0	0.0	0.0	0.0	0.0	0.0
0.0	0.0	0.0	0.0	0.0	0.0	0.0	0.0
0.0	0.0	0.0	0.0	0.0	0.0	0.0	0.0
0.0	0.0	0.0	0.0	0.0	0.0	0.0	0.0
0.0	0.0	0.0	0.0	0.0	0.0	0.0	0.0
0.0	0.0	0.0	0.0	0.0	0.0	0.0	0.0
0.0	0.0	0.0	0.0	0.0	0.0	0.0	0.0
0.0	0.0	0.0	0.0	0.0	0.0	0.0	0.0
0.0	0.0	0.0	0.0	0.0	0.0	0.0	0.0
0.0	0.0	0.0	0.0	0.0	0.0	0.0	0.0
0.0	0.0	0.0	0.0	0.0	0.0	0.0	0.0
0.0	0.0	0.0	0.0	0.0	0.0	0.0	0.0
0.0	0.0	0.0	0.0	0.0	0.0	0.0	0.0
0.0	0.0	0.0	0.0	0.0	0.0	0.0	0.0
0.0	0.0	0.0	0.0	0.0	0.0	0.0	0.0
0.0	0.0	0.0	0.0	0.0	0.0	0.0	0.0
0.0	0.0	0.0	0.0	0.0	0.0	0.0	0.0
0.0	0.0	0.0	0.0	0.0	0.0	0.0	0.0
0.0	0.0	0.0	0.0	0.0	0.0	0.0	0.0
0.0	0.0	0.0	0.0	0.0	0.0	0.0	0.0
62	74	51	66	40	71	69	56
0	1	1	1	1	1	1	1
33.7	23.5	25.8	22.16311078	29.12766688	25.2	30.67119499	25.4

11635.TW.CG	11635.TW.AW	11635.TW.AP.	11635.CIR57.(	11635.TW.BZ.	11635.TW.DP	11635.TW.BI.(	11635.TW.CS.
16.0	1.0	1678.0	2321.0	16.0	5465.0	2.0	4.0
0.0	0.0	0.0	0.0	0.0	2.0	0.0	0.0
10.0	0.0	0.0	0.0	1.0	0.0	0.0	1.0
9.0	4.0	59.0	0.0	0.0	35.0	183.0	0.0
3.0	0.0	561.0	0.0	290.0	25.0	26.0	2.0
10.0	6.0	444.0	0.0	355.0	0.0	71.0	22.0
0.0	0.0	0.0	0.0	0.0	0.0	0.0	0.0
0.0	307.0	0.0	0.0	0.0	0.0	9.0	0.0
0.0	326.0	0.0	0.0	0.0	0.0	0.0	0.0
3.0	14.0	174.0	1.0	0.0	48.0	208.0	100.0
1.0	0.0	85.0	0.0	181.0	1.0	0.0	0.0
0.0	0.0	0.0	0.0	0.0	0.0	0.0	0.0
0.0	0.0	0.0	0.0	276.0	0.0	120.0	0.0
47.0	1148.0	0.0	0.0	0.0	166.0	10.0	155.0
2.0	0.0	0.0	0.0	0.0	0.0	1.0	0.0
26.0	0.0	356.0	2.0	570.0	47.0	264.0	4.0
0.0	0.0	2.0	0.0	41.0	4.0	7.0	0.0
4056.0	41.0	3.0	0.0	0.0	444.0	0.0	479.0
244.0	68.0	34.0	0.0	161.0	71.0	34.0	9.0
0.0	0.0	474.0	0.0	566.0	388.0	177.0	0.0
98.0	49.0	85.0	0.0	65.0	0.0	31.0	13.0
0.0	33.0	0.0	0.0	0.0	0.0	111.0	0.0
0.0	0.0	5.0	0.0	27.0	0.0	1.0	0.0
0.0	0.0	30.0	0.0	0.0	0.0	0.0	0.0
0.0	0.0	0.0	0.0	94.0	0.0	53.0	0.0
289.0	355.0	48.0	0.0	25.0	158.0	3.0	1.0
0.0	0.0	0.0	0.0	0.0	0.0	0.0	0.0
9.0	0.0	55.0	0.0	97.0	5.0	17.0	1.0
0.0	34.0	0.0	0.0	0.0	0.0	0.0	0.0
1.0	4.0	71.0	0.0	22.0	0.0	2.0	0.0
343.0	3.0	8.0	2.0	11.0	306.0	35.0	0.0
71.0	265.0	12.0	0.0	4.0	0.0	24.0	0.0
0.0	0.0	0.0	0.0	0.0	60.0	0.0	0.0
6.0	0.0	0.0	0.0	65.0	0.0	45.0	0.0
40.0	95.0	12.0	0.0	11.0	0.0	19.0	26.0
0.0	0.0	0.0	0.0	0.0	0.0	0.0	0.0
0.0	0.0	0.0	0.0	0.0	0.0	17.0	0.0
190.0	87.0	15.0	0.0	47.0	0.0	19.0	21.0
0.0	29.0	0.0	0.0	0.0	0.0	37.0	0.0
12.0	2.0	3.0	0.0	64.0	16.0	44.0	10.0
0.0	0.0	27.0	0.0	31.0	1.0	2.0	0.0
0.0	2.0	0.0	0.0	166.0	0.0	47.0	0.0
113.0	40.0	0.0	0.0	6.0	157.0	1.0	7.0
6.0	0.0	76.0	1.0	184.0	5.0	114.0	0.0
0.0	0.0	4.0	0.0	9.0	1.0	4.0	0.0
24.0	242.0	22.0	0.0	123.0	0.0	41.0	4.0

58.0	38.0	1.0	19.0	2.0	11.0	0.0	4.0
0.0	0.0	0.0	0.0	167.0	0.0	0.0	0.0
0.0	1.0	5.0	0.0	0.0	1.0	3.0	0.0
0.0	0.0	0.0	0.0	0.0	0.0	0.0	0.0
0.0	0.0	0.0	0.0	0.0	0.0	0.0	0.0
1.0	19.0	5.0	0.0	9.0	0.0	0.0	0.0
6.0	1.0	77.0	0.0	135.0	4.0	17.0	0.0
0.0	0.0	0.0	0.0	0.0	0.0	0.0	0.0
30.0	0.0	153.0	0.0	0.0	0.0	419.0	0.0
0.0	0.0	0.0	0.0	0.0	0.0	0.0	0.0
62.0	0.0	0.0	0.0	6.0	0.0	0.0	0.0
22.0	27.0	0.0	0.0	1.0	0.0	3.0	3.0
0.0	0.0	0.0	0.0	0.0	0.0	0.0	0.0
0.0	0.0	0.0	0.0	0.0	0.0	0.0	0.0
0.0	0.0	42.0	0.0	0.0	0.0	120.0	0.0
0.0	0.0	0.0	0.0	0.0	0.0	0.0	0.0
0.0	0.0	0.0	0.0	0.0	0.0	0.0	0.0
0.0	0.0	0.0	0.0	0.0	0.0	0.0	0.0
25.0	3.0	0.0	0.0	104.0	0.0	5.0	0.0
0.0	1.0	0.0	0.0	0.0	0.0	0.0	0.0
0.0	0.0	0.0	0.0	0.0	0.0	0.0	0.0
0.0	0.0	0.0	0.0	0.0	0.0	0.0	0.0
0.0	0.0	19.0	0.0	0.0	0.0	0.0	0.0
2.0	30.0	31.0	0.0	71.0	0.0	41.0	0.0
0.0	0.0	0.0	0.0	0.0	0.0	303.0	0.0
1.0	1.0	115.0	0.0	0.0	0.0	47.0	0.0
0.0	0.0	0.0	0.0	0.0	0.0	0.0	0.0
0.0	0.0	0.0	0.0	0.0	0.0	0.0	0.0
0.0	0.0	0.0	0.0	0.0	0.0	1.0	0.0
0.0	0.0	0.0	0.0	0.0	0.0	0.0	0.0
0.0	0.0	0.0	0.0	0.0	0.0	0.0	0.0
0.0	0.0	0.0	0.0	0.0	0.0	0.0	0.0
0.0	0.0	0.0	0.0	231.0	0.0	0.0	0.0
0.0	0.0	0.0	0.0	0.0	0.0	0.0	0.0
357.0	0.0	9.0	0.0	549.0	0.0	31.0	5.0
0.0	1.0	0.0	0.0	212.0	0.0	0.0	0.0
13.0	0.0	60.0	0.0	373.0	0.0	0.0	0.0
0.0	0.0	0.0	0.0	0.0	0.0	0.0	0.0
6.0	0.0	115.0	0.0	100.0	85.0	19.0	0.0
1.0	0.0	38.0	0.0	204.0	0.0	5.0	0.0
0.0	0.0	25.0	0.0	231.0	0.0	102.0	0.0
0.0	0.0	0.0	0.0	0.0	0.0	0.0	0.0
1.0	0.0	92.0	0.0	89.0	0.0	71.0	0.0
0.0	2.0	0.0	0.0	0.0	14.0	0.0	0.0
0.0	1.0	40.0	0.0	82.0	0.0	25.0	0.0
0.0	0.0	0.0	0.0	0.0	0.0	0.0	0.0
2.0	4.0	28.0	0.0	65.0	1.0	17.0	0.0

2.0	2.0	69.0	0.0	79.0	12.0	36.0	2.0
0.0	2.0	16.0	0.0	27.0	3.0	0.0	8.0
9.0	0.0	19.0	0.0	0.0	7.0	113.0	567.0
0.0	0.0	0.0	0.0	100.0	0.0	0.0	0.0
0.0	0.0	48.0	0.0	99.0	0.0	7.0	0.0
0.0	0.0	0.0	0.0	66.0	0.0	0.0	0.0
3.0	1.0	89.0	0.0	78.0	9.0	63.0	0.0
0.0	0.0	0.0	0.0	70.0	0.0	0.0	0.0
0.0	3.0	4.0	0.0	44.0	0.0	56.0	0.0
0.0	0.0	0.0	0.0	81.0	0.0	0.0	0.0
1.0	0.0	22.0	0.0	104.0	0.0	97.0	0.0
0.0	0.0	0.0	0.0	59.0	0.0	0.0	0.0
4.0	0.0	47.0	0.0	66.0	0.0	322.0	0.0
0.0	0.0	0.0	0.0	105.0	0.0	4.0	0.0
2.0	0.0	63.0	0.0	70.0	0.0	25.0	2.0
0.0	0.0	0.0	0.0	69.0	0.0	0.0	0.0
0.0	0.0	0.0	0.0	64.0	0.0	0.0	0.0
0.0	0.0	14.0	0.0	58.0	0.0	0.0	0.0
0.0	0.0	36.0	0.0	94.0	0.0	17.0	0.0
41.0	0.0	20.0	0.0	67.0	0.0	24.0	0.0
1.0	5.0	15.0	2.0	34.0	1.0	10.0	1.0
0.0	0.0	4.0	0.0	0.0	1.0	30.0	0.0
0.0	0.0	0.0	0.0	40.0	0.0	33.0	0.0
37.0	0.0	0.0	0.0	64.0	0.0	0.0	0.0
0.0	2.0	0.0	0.0	39.0	0.0	57.0	0.0
0.0	0.0	0.0	0.0	41.0	0.0	130.0	0.0
4.0	1.0	0.0	0.0	0.0	0.0	16.0	0.0
0.0	0.0	0.0	0.0	0.0	14.0	0.0	0.0
0.0	0.0	2.0	0.0	25.0	8.0	10.0	0.0
0.0	1.0	0.0	0.0	8.0	0.0	11.0	0.0
0.0	0.0	1.0	0.0	29.0	0.0	2.0	0.0
0.0	0.0	122.0	0.0	36.0	8.0	0.0	0.0
3.0	0.0	39.0	0.0	32.0	0.0	27.0	0.0
0.0	0.0	0.0	0.0	0.0	0.0	0.0	0.0
0.0	0.0	0.0	0.0	33.0	0.0	0.0	0.0
9.0	0.0	190.0	0.0	26.0	0.0	157.0	0.0
0.0	0.0	0.0	0.0	0.0	0.0	0.0	0.0
0.0	0.0	27.0	0.0	27.0	5.0	5.0	0.0
11.0	3.0	222.0	0.0	0.0	10.0	0.0	0.0
0.0	0.0	0.0	0.0	36.0	0.0	0.0	0.0
0.0	0.0	0.0	0.0	25.0	0.0	0.0	0.0
0.0	1.0	121.0	0.0	0.0	14.0	36.0	0.0
0.0	0.0	0.0	0.0	0.0	0.0	15.0	0.0
6.0	0.0	25.0	0.0	18.0	0.0	10.0	0.0
0.0	0.0	10.0	0.0	2.0	0.0	0.0	2.0
0.0	0.0	0.0	0.0	39.0	0.0	0.0	0.0
0.0	0.0	48.0	0.0	19.0	0.0	0.0	0.0

0.0	0.0	0.0	3.0	0.0	0.0	0.0	0.0
1.0	0.0	5.0	0.0	22.0	0.0	10.0	0.0
2.0	0.0	0.0	0.0	16.0	2.0	0.0	0.0
0.0	0.0	0.0	0.0	21.0	0.0	0.0	0.0
0.0	0.0	46.0	0.0	10.0	7.0	12.0	0.0
0.0	3.0	0.0	0.0	0.0	0.0	0.0	0.0
0.0	0.0	0.0	0.0	25.0	0.0	0.0	0.0
2.0	2.0	15.0	0.0	20.0	0.0	0.0	0.0
0.0	0.0	11.0	0.0	1.0	0.0	0.0	0.0
9.0	0.0	0.0	0.0	26.0	0.0	0.0	0.0
0.0	0.0	0.0	0.0	21.0	2.0	3.0	0.0
0.0	0.0	13.0	0.0	17.0	0.0	0.0	0.0
0.0	2.0	12.0	0.0	11.0	6.0	6.0	2.0
0.0	0.0	0.0	0.0	12.0	0.0	0.0	0.0
1.0	0.0	30.0	0.0	0.0	0.0	0.0	0.0
0.0	3.0	0.0	0.0	0.0	0.0	0.0	0.0
0.0	0.0	0.0	0.0	22.0	0.0	1.0	0.0
0.0	0.0	0.0	0.0	13.0	0.0	0.0	0.0
90.0	2.0	0.0	0.0	6.0	124.0	0.0	1.0
0.0	0.0	0.0	0.0	0.0	0.0	0.0	0.0
0.0	0.0	0.0	0.0	0.0	10.0	0.0	0.0
0.0	0.0	2.0	0.0	9.0	0.0	3.0	0.0
0.0	0.0	0.0	0.0	2.0	2.0	2.0	0.0
0.0	0.0	5.0	0.0	11.0	0.0	8.0	0.0
1.0	0.0	0.0	0.0	8.0	0.0	13.0	0.0
0.0	0.0	0.0	0.0	6.0	0.0	3.0	0.0
0.0	0.0	0.0	0.0	12.0	0.0	0.0	0.0
0.0	0.0	3.0	0.0	10.0	0.0	5.0	0.0
0.0	0.0	2.0	0.0	9.0	0.0	0.0	0.0
0.0	0.0	0.0	0.0	0.0	0.0	0.0	0.0
2.0	1.0	7.0	0.0	23.0	20.0	2.0	0.0
0.0	0.0	0.0	0.0	9.0	0.0	0.0	0.0
6.0	10.0	3.0	0.0	0.0	0.0	0.0	0.0
0.0	0.0	0.0	0.0	0.0	0.0	2.0	0.0
0.0	0.0	8.0	0.0	9.0	0.0	0.0	0.0
0.0	0.0	0.0	0.0	5.0	0.0	3.0	0.0
2.0	0.0	0.0	0.0	8.0	21.0	0.0	0.0
0.0	0.0	0.0	0.0	0.0	6.0	8.0	0.0
0.0	0.0	8.0	0.0	8.0	4.0	0.0	0.0
0.0	0.0	27.0	0.0	11.0	10.0	5.0	0.0
10.0	0.0	0.0	0.0	16.0	11.0	2.0	0.0
0.0	1.0	13.0	0.0	12.0	0.0	3.0	0.0
0.0	0.0	8.0	0.0	7.0	0.0	8.0	0.0
0.0	0.0	0.0	0.0	12.0	0.0	0.0	0.0
3.0	0.0	11.0	0.0	13.0	5.0	20.0	2.0
0.0	0.0	1.0	0.0	3.0	0.0	0.0	0.0
0.0	0.0	0.0	0.0	5.0	0.0	0.0	0.0

0.0	0.0	0.0	0.0	11.0	0.0	0.0	0.0
0.0	0.0	0.0	0.0	4.0	2.0	0.0	0.0
0.0	0.0	2.0	0.0	4.0	0.0	0.0	0.0
0.0	0.0	16.0	0.0	1.0	0.0	77.0	0.0
0.0	0.0	12.0	0.0	5.0	0.0	23.0	0.0
0.0	1.0	0.0	0.0	7.0	0.0	0.0	0.0
21.0	60.0	8.0	0.0	5.0	0.0	10.0	2.0
0.0	0.0	0.0	0.0	5.0	0.0	0.0	0.0
0.0	0.0	1.0	92.0	1.0	0.0	0.0	0.0
0.0	0.0	0.0	0.0	3.0	0.0	0.0	0.0
0.0	0.0	0.0	0.0	7.0	0.0	7.0	0.0
0.0	0.0	5.0	0.0	4.0	0.0	7.0	0.0
0.0	0.0	5.0	0.0	1.0	0.0	0.0	0.0
0.0	0.0	0.0	0.0	0.0	5.0	0.0	0.0
0.0	0.0	0.0	0.0	6.0	0.0	0.0	0.0
5.0	5.0	0.0	0.0	2.0	1.0	4.0	13.0
0.0	0.0	0.0	0.0	0.0	0.0	0.0	0.0
0.0	0.0	0.0	0.0	8.0	0.0	0.0	0.0
0.0	0.0	0.0	0.0	0.0	0.0	0.0	0.0
0.0	0.0	0.0	0.0	9.0	0.0	0.0	0.0
13.0	26.0	0.0	0.0	3.0	150.0	0.0	54.0
0.0	0.0	0.0	0.0	0.0	0.0	0.0	0.0
0.0	0.0	0.0	0.0	0.0	0.0	0.0	0.0
0.0	0.0	3.0	0.0	5.0	0.0	5.0	0.0
0.0	0.0	0.0	0.0	0.0	0.0	0.0	0.0
0.0	0.0	0.0	0.0	3.0	0.0	0.0	0.0
0.0	0.0	0.0	0.0	0.0	0.0	0.0	0.0
0.0	0.0	0.0	0.0	2.0	0.0	0.0	0.0
1.0	0.0	0.0	0.0	6.0	0.0	0.0	0.0
0.0	0.0	0.0	0.0	1.0	0.0	0.0	0.0
0.0	0.0	0.0	0.0	0.0	0.0	0.0	0.0
0.0	0.0	4.0	0.0	1.0	0.0	4.0	0.0
0.0	0.0	0.0	0.0	0.0	0.0	0.0	0.0
0.0	0.0	0.0	0.0	2.0	0.0	1.0	0.0
0.0	0.0	0.0	0.0	1.0	0.0	0.0	0.0
1.0	0.0	8.0	0.0	4.0	0.0	0.0	0.0
0.0	0.0	0.0	0.0	1.0	0.0	1.0	0.0
0.0	0.0	0.0	0.0	3.0	0.0	0.0	0.0
0.0	0.0	6.0	0.0	0.0	0.0	0.0	0.0
0.0	0.0	2.0	0.0	8.0	0.0	4.0	0.0
0.0	0.0	0.0	0.0	4.0	0.0	0.0	0.0
0.0	0.0	0.0	0.0	0.0	0.0	4.0	0.0
0.0	0.0	0.0	0.0	0.0	0.0	0.0	0.0
0.0	0.0	3.0	0.0	5.0	0.0	0.0	0.0
0.0	0.0	0.0	0.0	2.0	0.0	0.0	0.0
0.0	0.0	0.0	0.0	0.0	0.0	0.0	0.0
0.0	0.0	0.0	0.0	3.0	0.0	2.0	0.0

0.0	0.0	11.0	0.0	0.0	0.0	0.0	0.0
0.0	0.0	4.0	0.0	5.0	0.0	0.0	0.0
0.0	0.0	2.0	0.0	0.0	2.0	0.0	0.0
0.0	0.0	4.0	0.0	0.0	0.0	0.0	0.0
46.0	0.0	0.0	0.0	1.0	0.0	0.0	0.0
0.0	0.0	0.0	0.0	0.0	0.0	0.0	0.0
0.0	0.0	0.0	0.0	1.0	0.0	1.0	0.0
0.0	0.0	8.0	0.0	4.0	0.0	0.0	0.0
0.0	0.0	0.0	0.0	1.0	0.0	0.0	0.0
0.0	0.0	0.0	0.0	0.0	0.0	0.0	0.0
0.0	0.0	0.0	0.0	3.0	0.0	0.0	0.0
0.0	0.0	0.0	0.0	0.0	0.0	0.0	0.0
0.0	0.0	0.0	0.0	0.0	0.0	0.0	0.0
0.0	0.0	31.0	0.0	0.0	0.0	36.0	0.0
0.0	0.0	0.0	0.0	0.0	0.0	3.0	0.0
0.0	0.0	71.0	0.0	0.0	0.0	0.0	0.0
0.0	0.0	0.0	0.0	2.0	0.0	0.0	0.0
0.0	0.0	0.0	0.0	0.0	0.0	0.0	0.0
0.0	0.0	0.0	0.0	3.0	0.0	1.0	0.0
0.0	0.0	0.0	0.0	0.0	0.0	5.0	0.0
0.0	0.0	0.0	0.0	2.0	0.0	7.0	0.0
0.0	0.0	1.0	0.0	1.0	0.0	2.0	0.0
0.0	0.0	0.0	0.0	0.0	0.0	0.0	0.0
0.0	0.0	1.0	0.0	0.0	0.0	0.0	0.0
0.0	0.0	0.0	0.0	1.0	0.0	0.0	0.0
0.0	0.0	19.0	0.0	4.0	0.0	3.0	0.0
9.0	0.0	0.0	0.0	5.0	0.0	0.0	0.0
0.0	0.0	0.0	0.0	0.0	0.0	0.0	0.0
0.0	0.0	0.0	0.0	2.0	0.0	0.0	0.0
0.0	0.0	0.0	0.0	0.0	0.0	0.0	0.0
0.0	0.0	0.0	0.0	1.0	0.0	0.0	0.0
0.0	0.0	4.0	0.0	0.0	0.0	0.0	0.0
0.0	0.0	0.0	0.0	0.0	0.0	0.0	0.0
0.0	0.0	0.0	0.0	0.0	0.0	0.0	0.0
1.0	0.0	1.0	0.0	1.0	0.0	14.0	0.0
0.0	0.0	0.0	0.0	4.0	0.0	4.0	0.0
0.0	0.0	0.0	0.0	4.0	0.0	0.0	0.0
17.0	0.0	3.0	0.0	3.0	15.0	0.0	0.0
0.0	0.0	1.0	0.0	0.0	0.0	0.0	0.0
0.0	0.0	0.0	0.0	1.0	0.0	0.0	0.0
0.0	0.0	0.0	0.0	0.0	0.0	0.0	0.0
0.0	0.0	0.0	0.0	0.0	0.0	0.0	0.0
0.0	0.0	2.0	0.0	1.0	0.0	0.0	0.0
0.0	0.0	0.0	0.0	0.0	0.0	0.0	0.0
0.0	0.0	0.0	0.0	0.0	1.0	0.0	0.0
0.0	0.0	0.0	0.0	0.0	0.0	0.0	0.0
0.0	0.0	0.0	0.0	1.0	0.0	1.0	0.0

0.0	0.0	0.0	0.0	0.0	1.0	0.0	0.0
0.0	0.0	0.0	0.0	0.0	0.0	0.0	0.0
0.0	0.0	0.0	0.0	0.0	0.0	0.0	0.0
0.0	0.0	0.0	0.0	0.0	0.0	1.0	0.0
0.0	0.0	0.0	39.0	1.0	0.0	0.0	0.0
0.0	0.0	0.0	0.0	0.0	0.0	0.0	0.0
0.0	0.0	0.0	0.0	1.0	1.0	0.0	0.0
0.0	0.0	0.0	0.0	0.0	0.0	0.0	0.0
0.0	0.0	2.0	0.0	1.0	0.0	0.0	0.0
0.0	0.0	0.0	0.0	3.0	0.0	0.0	0.0
0.0	0.0	0.0	0.0	2.0	1.0	0.0	0.0
7.0	0.0	1.0	0.0	0.0	0.0	9.0	3.0
0.0	1.0	0.0	0.0	0.0	0.0	0.0	0.0
0.0	0.0	0.0	0.0	0.0	0.0	0.0	0.0
0.0	0.0	0.0	0.0	0.0	0.0	6.0	0.0
3.0	22.0	2.0	0.0	0.0	185.0	6.0	9.0
0.0	0.0	0.0	0.0	3.0	0.0	0.0	0.0
0.0	0.0	0.0	0.0	0.0	0.0	0.0	0.0
2.0	0.0	10.0	0.0	3.0	0.0	0.0	0.0
0.0	0.0	0.0	0.0	0.0	0.0	0.0	0.0
0.0	0.0	0.0	0.0	0.0	0.0	0.0	0.0
0.0	0.0	0.0	0.0	7.0	0.0	0.0	0.0
0.0	0.0	0.0	0.0	0.0	0.0	0.0	0.0
0.0	0.0	0.0	0.0	0.0	0.0	0.0	0.0
0.0	0.0	3.0	0.0	2.0	0.0	4.0	2.0
0.0	0.0	0.0	0.0	3.0	0.0	0.0	0.0
5.0	2.0	0.0	1.0	0.0	1.0	0.0	4.0
0.0	0.0	0.0	0.0	0.0	1.0	0.0	0.0
0.0	0.0	3.0	0.0	0.0	0.0	0.0	0.0
0.0	0.0	0.0	0.0	0.0	0.0	0.0	0.0
0.0	0.0	0.0	0.0	0.0	0.0	0.0	0.0
0.0	0.0	0.0	0.0	0.0	0.0	0.0	0.0
0.0	0.0	0.0	0.0	0.0	0.0	0.0	0.0
0.0	0.0	0.0	0.0	4.0	0.0	0.0	0.0
0.0	0.0	0.0	0.0	0.0	0.0	0.0	0.0
0.0	0.0	0.0	0.0	0.0	0.0	0.0	0.0
0.0	0.0	2.0	0.0	0.0	0.0	0.0	0.0
0.0	0.0	0.0	0.0	1.0	0.0	2.0	0.0
0.0	0.0	0.0	0.0	0.0	0.0	0.0	0.0
0.0	0.0	0.0	0.0	0.0	0.0	0.0	0.0
0.0	0.0	0.0	0.0	4.0	0.0	0.0	0.0
0.0	0.0	0.0	0.0	3.0	0.0	0.0	0.0
0.0	0.0	0.0	0.0	0.0	0.0	3.0	0.0
0.0	0.0	2.0	0.0	1.0	0.0	0.0	1.0
0.0	0.0	0.0	0.0	2.0	0.0	0.0	0.0
0.0	0.0	0.0	0.0	0.0	0.0	0.0	0.0
0.0	0.0	4.0	0.0	0.0	0.0	3.0	0.0

0.0	0.0	0.0	0.0	0.0	0.0	0.0	0.0
0.0	0.0	0.0	0.0	0.0	0.0	0.0	0.0
0.0	0.0	0.0	0.0	0.0	0.0	0.0	0.0
0.0	0.0	0.0	0.0	0.0	0.0	0.0	0.0
0.0	13.0	0.0	0.0	0.0	0.0	0.0	0.0
0.0	0.0	0.0	0.0	0.0	0.0	0.0	0.0
0.0	0.0	0.0	0.0	2.0	0.0	0.0	0.0
0.0	0.0	0.0	0.0	0.0	0.0	0.0	0.0
0.0	0.0	0.0	0.0	0.0	5.0	0.0	0.0
0.0	0.0	0.0	0.0	0.0	0.0	0.0	0.0
0.0	0.0	0.0	0.0	3.0	0.0	0.0	0.0
0.0	0.0	0.0	0.0	0.0	0.0	0.0	0.0
0.0	0.0	0.0	0.0	0.0	0.0	0.0	0.0
0.0	0.0	0.0	0.0	0.0	0.0	0.0	0.0
0.0	0.0	0.0	0.0	0.0	0.0	0.0	0.0
0.0	0.0	0.0	0.0	0.0	0.0	0.0	0.0
0.0	0.0	9.0	0.0	0.0	0.0	0.0	0.0
0.0	0.0	0.0	0.0	0.0	0.0	1.0	0.0
0.0	0.0	0.0	0.0	0.0	1.0	4.0	0.0
0.0	0.0	0.0	0.0	3.0	0.0	0.0	0.0
0.0	0.0	0.0	0.0	0.0	2.0	0.0	0.0
0.0	0.0	0.0	0.0	0.0	0.0	0.0	0.0
0.0	0.0	2.0	0.0	2.0	0.0	0.0	0.0
0.0	0.0	0.0	0.0	0.0	0.0	0.0	0.0
0.0	0.0	0.0	0.0	0.0	0.0	0.0	0.0
0.0	0.0	0.0	0.0	0.0	0.0	0.0	0.0
0.0	0.0	0.0	0.0	0.0	1.0	0.0	0.0
0.0	0.0	0.0	0.0	3.0	0.0	0.0	0.0
0.0	0.0	0.0	0.0	0.0	0.0	0.0	0.0
0.0	0.0	0.0	0.0	0.0	0.0	0.0	0.0
0.0	0.0	0.0	0.0	0.0	0.0	0.0	0.0
0.0	0.0	0.0	0.0	0.0	0.0	0.0	0.0
0.0	0.0	0.0	0.0	0.0	0.0	0.0	0.0
0.0	0.0	2.0	0.0	0.0	1.0	0.0	0.0
0.0	0.0	0.0	0.0	0.0	0.0	0.0	0.0
0.0	0.0	0.0	0.0	0.0	0.0	0.0	0.0
0.0	1.0	0.0	0.0	3.0	0.0	0.0	0.0
0.0	0.0	0.0	0.0	0.0	0.0	0.0	0.0
0.0	0.0	0.0	0.0	2.0	0.0	0.0	0.0
0.0	0.0	2.0	0.0	0.0	0.0	0.0	0.0
0.0	0.0	0.0	0.0	0.0	0.0	0.0	2.0
0.0	0.0	0.0	0.0	0.0	0.0	4.0	0.0
0.0	0.0	0.0	0.0	0.0	0.0	0.0	0.0
0.0	0.0	0.0	0.0	0.0	0.0	0.0	0.0
0.0	0.0	0.0	0.0	0.0	0.0	0.0	0.0
0.0	0.0	0.0	0.0	0.0	0.0	0.0	0.0
0.0	0.0	0.0	0.0	0.0	0.0	0.0	0.0

0.0	0.0	0.0	0.0	0.0	0.0	0.0	0.0
0.0	0.0	0.0	0.0	4.0	0.0	2.0	0.0
0.0	0.0	0.0	0.0	0.0	0.0	0.0	0.0
0.0	0.0	0.0	0.0	0.0	0.0	0.0	0.0
0.0	0.0	1.0	0.0	0.0	0.0	0.0	0.0
273.0	0.0	26.0	0.0	0.0	0.0	0.0	0.0
0.0	0.0	0.0	0.0	0.0	0.0	0.0	0.0
0.0	0.0	0.0	0.0	0.0	0.0	0.0	0.0
0.0	0.0	0.0	0.0	4.0	0.0	0.0	0.0
4.0	0.0	0.0	0.0	2.0	6.0	0.0	0.0
0.0	0.0	0.0	0.0	6.0	0.0	0.0	0.0
0.0	0.0	0.0	0.0	0.0	0.0	0.0	0.0
0.0	0.0	0.0	0.0	0.0	0.0	0.0	0.0
0.0	0.0	0.0	0.0	0.0	0.0	0.0	0.0
0.0	0.0	0.0	0.0	1.0	0.0	0.0	0.0
0.0	0.0	0.0	0.0	3.0	0.0	0.0	0.0
1.0	0.0	1.0	0.0	0.0	0.0	11.0	0.0
0.0	0.0	0.0	0.0	0.0	0.0	0.0	0.0
0.0	0.0	0.0	0.0	2.0	0.0	0.0	0.0
0.0	0.0	0.0	0.0	0.0	0.0	0.0	0.0
0.0	0.0	0.0	0.0	0.0	0.0	0.0	0.0
0.0	0.0	0.0	0.0	0.0	0.0	0.0	0.0
0.0	0.0	0.0	0.0	0.0	0.0	0.0	0.0
555.0	0.0	0.0	2.0	0.0	156.0	0.0	0.0
0.0	0.0	0.0	2451.0	0.0	0.0	0.0	0.0
0.0	0.0	0.0	0.0	0.0	0.0	0.0	0.0
0.0	0.0	0.0	0.0	1.0	0.0	0.0	0.0
0.0	0.0	0.0	0.0	0.0	0.0	0.0	0.0
0.0	0.0	0.0	0.0	0.0	0.0	0.0	0.0
2.0	0.0	5.0	0.0	3.0	0.0	0.0	0.0
0.0	0.0	0.0	0.0	0.0	0.0	0.0	0.0
0.0	0.0	0.0	0.0	0.0	0.0	0.0	0.0
0.0	0.0	0.0	0.0	0.0	0.0	0.0	0.0
0.0	0.0	0.0	0.0	0.0	0.0	0.0	0.0
0.0	0.0	0.0	0.0	0.0	0.0	0.0	0.0
0.0	0.0	0.0	0.0	1.0	0.0	0.0	0.0
0.0	0.0	0.0	0.0	0.0	0.0	0.0	2.0
0.0	0.0	0.0	0.0	0.0	0.0	0.0	0.0
2.0	0.0	0.0	0.0	3.0	0.0	0.0	0.0
0.0	0.0	0.0	0.0	0.0	0.0	0.0	0.0
0.0	0.0	0.0	45.0	0.0	0.0	0.0	0.0
0.0	0.0	120.0	0.0	0.0	18.0	0.0	0.0
0.0	0.0	0.0	0.0	0.0	0.0	0.0	0.0
0.0	0.0	0.0	0.0	0.0	0.0	0.0	0.0
0.0	0.0	0.0	0.0	0.0	0.0	0.0	0.0
0.0	0.0	0.0	0.0	0.0	0.0	0.0	0.0
0.0	1.0	0.0	1.0	1.0	253.0	1.0	0.0

0.0	0.0	0.0	0.0	0.0	0.0	0.0	0.0
0.0	0.0	0.0	2.0	0.0	1.0	0.0	0.0
0.0	0.0	0.0	0.0	3.0	0.0	0.0	0.0
0.0	0.0	0.0	76.0	0.0	2.0	0.0	0.0
0.0	0.0	0.0	0.0	0.0	0.0	0.0	0.0
0.0	0.0	0.0	0.0	0.0	0.0	0.0	0.0
0.0	0.0	0.0	0.0	2.0	0.0	0.0	0.0
0.0	0.0	0.0	0.0	0.0	0.0	0.0	0.0
0.0	0.0	0.0	0.0	0.0	0.0	0.0	0.0
0.0	0.0	0.0	0.0	0.0	0.0	0.0	0.0
0.0	0.0	0.0	0.0	0.0	0.0	0.0	0.0
0.0	0.0	0.0	0.0	0.0	0.0	0.0	0.0
0.0	0.0	11.0	0.0	0.0	0.0	2.0	0.0
0.0	0.0	0.0	0.0	0.0	0.0	0.0	0.0
0.0	0.0	0.0	0.0	0.0	0.0	0.0	0.0
0.0	2.0	0.0	0.0	0.0	0.0	0.0	0.0
0.0	0.0	0.0	0.0	0.0	0.0	0.0	0.0
0.0	0.0	0.0	0.0	0.0	0.0	0.0	0.0
0.0	0.0	5.0	0.0	0.0	20.0	0.0	0.0
0.0	0.0	0.0	0.0	0.0	0.0	0.0	0.0
0.0	0.0	24.0	0.0	0.0	2.0	61.0	0.0
0.0	0.0	0.0	0.0	0.0	0.0	0.0	0.0
0.0	0.0	0.0	0.0	0.0	0.0	0.0	0.0
0.0	0.0	0.0	0.0	0.0	0.0	0.0	0.0
0.0	0.0	0.0	0.0	0.0	0.0	0.0	0.0
0.0	0.0	0.0	0.0	0.0	0.0	0.0	0.0
0.0	0.0	0.0	0.0	0.0	0.0	0.0	0.0
0.0	0.0	0.0	0.0	0.0	0.0	0.0	0.0
26.0	1.0	0.0	0.0	0.0	0.0	0.0	0.0
0.0	0.0	0.0	0.0	0.0	0.0	0.0	0.0
4.0	0.0	0.0	0.0	0.0	0.0	22.0	0.0
0.0	0.0	6.0	0.0	0.0	0.0	2.0	0.0
0.0	0.0	0.0	0.0	0.0	0.0	0.0	0.0
0.0	0.0	0.0	0.0	0.0	0.0	0.0	0.0
0.0	0.0	0.0	0.0	0.0	0.0	26.0	0.0
0.0	0.0	0.0	0.0	0.0	0.0	0.0	0.0
0.0	0.0	3.0	0.0	1.0	0.0	16.0	0.0
0.0	0.0	0.0	0.0	0.0	0.0	0.0	0.0
0.0	27.0	0.0	0.0	0.0	10.0	3.0	0.0
0.0	0.0	4.0	0.0	4.0	0.0	2.0	0.0
0.0	0.0	0.0	0.0	0.0	0.0	0.0	0.0
0.0	0.0	0.0	0.0	0.0	0.0	0.0	0.0
0.0	0.0	6.0	0.0	0.0	0.0	0.0	0.0
0.0	0.0	0.0	0.0	0.0	0.0	0.0	0.0
2.0	0.0	87.0	0.0	0.0	0.0	20.0	0.0
0.0	0.0	0.0	0.0	0.0	0.0	0.0	0.0
0.0	0.0	0.0	0.0	0.0	0.0	0.0	0.0

0.0	0.0	0.0	0.0	0.0	0.0	0.0	0.0
0.0	0.0	4.0	0.0	0.0	0.0	0.0	0.0
0.0	0.0	0.0	0.0	0.0	1.0	0.0	0.0
1.0	4019.0	0.0	2.0	0.0	2.0	0.0	0.0
0.0	0.0	0.0	0.0	0.0	1.0	0.0	0.0
0.0	0.0	0.0	0.0	0.0	0.0	0.0	0.0
0.0	0.0	0.0	0.0	0.0	0.0	0.0	0.0
0.0	0.0	0.0	0.0	0.0	0.0	0.0	0.0
0.0	0.0	0.0	0.0	0.0	0.0	0.0	0.0
0.0	0.0	0.0	0.0	0.0	0.0	0.0	0.0
0.0	0.0	0.0	0.0	0.0	0.0	0.0	0.0
0.0	0.0	0.0	0.0	0.0	0.0	0.0	0.0
0.0	0.0	0.0	0.0	1.0	0.0	0.0	0.0
0.0	0.0	0.0	0.0	0.0	8.0	2.0	0.0
2.0	1.0	0.0	0.0	2.0	0.0	0.0	4627.0
0.0	0.0	0.0	0.0	0.0	0.0	0.0	0.0
0.0	0.0	0.0	0.0	0.0	0.0	0.0	0.0
0.0	0.0	0.0	0.0	0.0	0.0	0.0	0.0
0.0	0.0	0.0	0.0	0.0	0.0	0.0	0.0
0.0	0.0	0.0	0.0	0.0	0.0	0.0	0.0
0.0	0.0	0.0	0.0	0.0	0.0	0.0	0.0
0.0	0.0	1.0	0.0	0.0	0.0	0.0	0.0
0.0	0.0	0.0	0.0	0.0	0.0	0.0	0.0
0.0	0.0	0.0	0.0	0.0	0.0	0.0	0.0
0.0	0.0	2.0	0.0	0.0	0.0	0.0	0.0
0.0	0.0	0.0	0.0	0.0	0.0	0.0	0.0
0.0	0.0	0.0	0.0	0.0	0.0	0.0	0.0
0.0	0.0	0.0	0.0	0.0	0.0	0.0	0.0
0.0	0.0	0.0	0.0	0.0	0.0	0.0	0.0
0.0	0.0	0.0	0.0	0.0	0.0	0.0	0.0
0.0	0.0	0.0	0.0	0.0	0.0	0.0	0.0
0.0	0.0	0.0	0.0	0.0	0.0	0.0	0.0
0.0	0.0	0.0	0.0	0.0	0.0	0.0	0.0
0.0	0.0	0.0	0.0	0.0	0.0	0.0	0.0
0.0	0.0	0.0	0.0	0.0	0.0	0.0	0.0
0.0	0.0	0.0	0.0	0.0	0.0	0.0	0.0
0.0	0.0	0.0	0.0	0.0	0.0	0.0	0.0
0.0	0.0	0.0	0.0	0.0	0.0	0.0	0.0
0.0	0.0	0.0	0.0	0.0	0.0	0.0	0.0
0.0	0.0	0.0	0.0	0.0	0.0	0.0	0.0
0.0	0.0	0.0	0.0	0.0	0.0	0.0	0.0
0.0	0.0	0.0	0.0	0.0	0.0	1.0	0.0
0.0	0.0	0.0	0.0	0.0	0.0	0.0	0.0
0.0	0.0	0.0	0.0	0.0	0.0	0.0	0.0
0.0	0.0	0.0	0.0	0.0	0.0	0.0	0.0
0.0	0.0	0.0	0.0	0.0	0.0	0.0	0.0

0.0	0.0	0.0	0.0	0.0	6.0	1.0	1.0
0.0	0.0	0.0	0.0	0.0	0.0	0.0	0.0
0.0	0.0	0.0	0.0	0.0	0.0	0.0	0.0
7.0	66.0	0.0	0.0	0.0	1.0	0.0	0.0
0.0	0.0	0.0	0.0	0.0	0.0	1.0	0.0
0.0	0.0	0.0	0.0	0.0	0.0	0.0	0.0
1.0	0.0	2.0	0.0	0.0	0.0	0.0	0.0
0.0	0.0	2.0	0.0	0.0	0.0	2.0	0.0
0.0	0.0	0.0	0.0	0.0	0.0	0.0	0.0
0.0	0.0	0.0	0.0	0.0	0.0	0.0	0.0
0.0	0.0	0.0	0.0	0.0	0.0	0.0	0.0
0.0	0.0	0.0	0.0	0.0	0.0	0.0	0.0
0.0	0.0	0.0	0.0	0.0	0.0	0.0	0.0
0.0	0.0	0.0	0.0	0.0	0.0	0.0	0.0
0.0	0.0	0.0	0.0	0.0	0.0	0.0	0.0
0.0	0.0	0.0	0.0	0.0	0.0	0.0	0.0
0.0	0.0	0.0	0.0	0.0	0.0	0.0	0.0
0.0	0.0	0.0	0.0	0.0	0.0	0.0	0.0
0.0	0.0	0.0	0.0	0.0	0.0	0.0	0.0
0.0	0.0	0.0	0.0	0.0	0.0	0.0	0.0
0.0	0.0	0.0	0.0	0.0	0.0	0.0	0.0
0.0	0.0	0.0	0.0	0.0	0.0	0.0	0.0
0.0	0.0	0.0	0.0	0.0	0.0	0.0	0.0
0.0	0.0	0.0	0.0	0.0	0.0	0.0	0.0
0.0	0.0	0.0	0.0	0.0	0.0	0.0	0.0
1.0	0.0	0.0	0.0	0.0	0.0	0.0	0.0
0.0	0.0	0.0	0.0	0.0	0.0	0.0	0.0
0.0	0.0	0.0	0.0	0.0	0.0	0.0	0.0
0.0	0.0	0.0	0.0	0.0	0.0	0.0	0.0
0.0	0.0	0.0	0.0	0.0	0.0	0.0	0.0
0.0	0.0	0.0	0.0	0.0	0.0	0.0	0.0
0.0	0.0	0.0	0.0	0.0	0.0	0.0	0.0
0.0	0.0	0.0	0.0	0.0	0.0	0.0	0.0
0.0	0.0	0.0	0.0	0.0	0.0	0.0	0.0
0.0	0.0	9.0	0.0	0.0	0.0	0.0	3.0
0.0	0.0	0.0	0.0	0.0	0.0	0.0	0.0
0.0	0.0	0.0	0.0	0.0	0.0	0.0	0.0
0.0	0.0	0.0	0.0	0.0	0.0	0.0	0.0
0.0	0.0	0.0	0.0	0.0	0.0	0.0	0.0
0.0	0.0	0.0	0.0	0.0	0.0	0.0	0.0
0.0	0.0	0.0	0.0	0.0	0.0	0.0	0.0
0.0	0.0	0.0	0.0	0.0	0.0	0.0	0.0
0.0	0.0	0.0	0.0	0.0	3.0	0.0	0.0
0.0	0.0	0.0	0.0	0.0	0.0	0.0	0.0
0.0	0.0	0.0	0.0	0.0	0.0	0.0	0.0
0.0	0.0	0.0	0.0	0.0	0.0	0.0	0.0
0.0	0.0	0.0	0.0	0.0	0.0	0.0	0.0

0.0	0.0	0.0	0.0	0.0	0.0	0.0	0.0
0.0	0.0	0.0	0.0	0.0	0.0	0.0	0.0
0.0	0.0	0.0	0.0	0.0	0.0	0.0	0.0
0.0	0.0	0.0	0.0	0.0	0.0	0.0	0.0
0.0	0.0	0.0	0.0	0.0	0.0	0.0	0.0
0.0	0.0	0.0	0.0	0.0	0.0	0.0	0.0
0.0	0.0	0.0	0.0	0.0	12.0	0.0	0.0
6.0	0.0	0.0	0.0	6.0	16.0	0.0	1.0
0.0	0.0	0.0	0.0	0.0	1.0	0.0	0.0
0.0	0.0	0.0	0.0	0.0	0.0	0.0	0.0
2.0	0.0	0.0	0.0	0.0	8.0	0.0	0.0
0.0	0.0	0.0	0.0	0.0	0.0	0.0	0.0
0.0	0.0	0.0	0.0	0.0	0.0	0.0	0.0
4.0	0.0	0.0	0.0	0.0	17.0	0.0	0.0
0.0	0.0	0.0	0.0	0.0	0.0	0.0	0.0
3.0	0.0	0.0	0.0	0.0	0.0	0.0	3.0
0.0	3.0	0.0	0.0	0.0	0.0	0.0	2.0
0.0	0.0	0.0	0.0	0.0	0.0	0.0	0.0
0.0	0.0	0.0	0.0	0.0	2.0	0.0	0.0
0.0	0.0	0.0	0.0	0.0	0.0	0.0	0.0
0.0	0.0	0.0	0.0	0.0	0.0	0.0	0.0
0.0	0.0	0.0	0.0	0.0	1.0	0.0	0.0
3.0	0.0	0.0	0.0	0.0	0.0	0.0	0.0
0.0	3.0	0.0	0.0	0.0	0.0	0.0	0.0
0.0	2.0	0.0	0.0	0.0	0.0	0.0	0.0
109.0	2.0	0.0	1.0	0.0	0.0	0.0	0.0
0.0	0.0	0.0	0.0	0.0	1.0	0.0	1.0
0.0	0.0	0.0	0.0	0.0	0.0	0.0	0.0
0.0	0.0	0.0	0.0	0.0	0.0	0.0	0.0
0.0	0.0	0.0	0.0	0.0	0.0	0.0	0.0
0.0	0.0	0.0	0.0	0.0	0.0	0.0	0.0
0.0	0.0	0.0	0.0	0.0	0.0	0.0	0.0
0.0	0.0	0.0	0.0	0.0	0.0	0.0	0.0
0.0	0.0	0.0	0.0	0.0	0.0	0.0	0.0
0.0	0.0	0.0	0.0	0.0	0.0	0.0	0.0
0.0	0.0	0.0	0.0	0.0	0.0	0.0	0.0
0.0	0.0	0.0	0.0	2.0	0.0	0.0	0.0
0.0	0.0	0.0	0.0	0.0	0.0	0.0	0.0
0.0	0.0	0.0	0.0	0.0	0.0	0.0	0.0
4.0	8.0	0.0	0.0	0.0	0.0	0.0	0.0
0.0	0.0	0.0	0.0	0.0	0.0	0.0	0.0
0.0	0.0	0.0	0.0	0.0	0.0	0.0	0.0
0.0	0.0	0.0	0.0	0.0	0.0	0.0	0.0
0.0	0.0	0.0	0.0	26.0	0.0	0.0	0.0
0.0	0.0	0.0	0.0	0.0	0.0	0.0	0.0
0.0	0.0	1.0	0.0	0.0	0.0	0.0	0.0
0.0	0.0	0.0	0.0	0.0	0.0	0.0	0.0

0.0	0.0	0.0	0.0	0.0	0.0	0.0	0.0
0.0	0.0	28.0	0.0	0.0	5.0	6.0	0.0
0.0	0.0	0.0	0.0	0.0	0.0	0.0	0.0
0.0	0.0	0.0	0.0	0.0	0.0	3.0	0.0
0.0	0.0	1.0	0.0	0.0	0.0	0.0	0.0
0.0	0.0	0.0	0.0	0.0	0.0	0.0	0.0
0.0	0.0	0.0	0.0	0.0	0.0	0.0	0.0
0.0	0.0	0.0	0.0	0.0	0.0	0.0	0.0
0.0	0.0	0.0	0.0	0.0	0.0	0.0	0.0
0.0	0.0	0.0	0.0	0.0	0.0	0.0	0.0
0.0	0.0	0.0	0.0	0.0	0.0	0.0	0.0
3.0	0.0	7.0	0.0	0.0	0.0	0.0	0.0
0.0	0.0	1.0	0.0	1.0	0.0	2.0	0.0
0.0	0.0	0.0	0.0	0.0	0.0	0.0	0.0
0.0	0.0	0.0	0.0	0.0	0.0	0.0	0.0
0.0	0.0	0.0	0.0	0.0	0.0	0.0	0.0
0.0	0.0	4.0	0.0	0.0	0.0	4.0	0.0
0.0	0.0	0.0	0.0	0.0	0.0	0.0	0.0
0.0	0.0	0.0	0.0	0.0	0.0	0.0	0.0
0.0	0.0	0.0	0.0	0.0	0.0	0.0	0.0
0.0	0.0	0.0	0.0	0.0	0.0	0.0	0.0
1.0	0.0	0.0	0.0	0.0	0.0	0.0	0.0
10.0	1.0	0.0	0.0	0.0	0.0	0.0	15.0
0.0	0.0	5.0	0.0	0.0	0.0	0.0	0.0
0.0	0.0	0.0	0.0	0.0	0.0	16.0	0.0
0.0	0.0	0.0	0.0	0.0	0.0	0.0	0.0
0.0	0.0	0.0	0.0	0.0	0.0	5.0	2.0
0.0	0.0	0.0	0.0	0.0	0.0	0.0	0.0
0.0	0.0	0.0	0.0	0.0	0.0	75.0	0.0
0.0	0.0	18.0	0.0	0.0	0.0	67.0	0.0
0.0	0.0	16.0	0.0	0.0	0.0	4.0	0.0
0.0	0.0	0.0	0.0	0.0	0.0	0.0	0.0
0.0	0.0	0.0	0.0	0.0	0.0	0.0	0.0
0.0	0.0	0.0	0.0	0.0	0.0	3.0	0.0
0.0	0.0	0.0	0.0	0.0	0.0	0.0	0.0
0.0	0.0	0.0	0.0	0.0	0.0	0.0	0.0
0.0	0.0	0.0	0.0	0.0	0.0	0.0	0.0
0.0	0.0	1.0	0.0	0.0	0.0	0.0	0.0
0.0	0.0	0.0	0.0	0.0	0.0	0.0	0.0
0.0	0.0	0.0	0.0	0.0	0.0	0.0	0.0
0.0	0.0	0.0	0.0	0.0	0.0	0.0	0.0
0.0	0.0	12.0	0.0	0.0	0.0	91.0	0.0
0.0	0.0	0.0	0.0	0.0	0.0	3.0	0.0
0.0	0.0	0.0	0.0	0.0	0.0	0.0	0.0
0.0	0.0	0.0	0.0	0.0	0.0	0.0	0.0
0.0	0.0	0.0	0.0	0.0	0.0	0.0	0.0
0.0	0.0	0.0	0.0	0.0	0.0	0.0	0.0

0.0	0.0	0.0	0.0	0.0	0.0	0.0	0.0
0.0	0.0	0.0	0.0	0.0	0.0	0.0	0.0
0.0	0.0	0.0	0.0	0.0	0.0	0.0	0.0
0.0	0.0	0.0	0.0	2.0	0.0	0.0	0.0
0.0	0.0	0.0	0.0	2.0	0.0	0.0	0.0
0.0	0.0	0.0	0.0	0.0	0.0	1.0	0.0
0.0	0.0	0.0	0.0	0.0	0.0	0.0	0.0
0.0	0.0	0.0	0.0	0.0	0.0	0.0	0.0
4.0	0.0	20.0	0.0	0.0	0.0	14.0	0.0
0.0	0.0	0.0	0.0	0.0	0.0	0.0	0.0
0.0	0.0	0.0	0.0	0.0	0.0	0.0	0.0
0.0	0.0	7.0	0.0	0.0	0.0	3.0	0.0
0.0	0.0	0.0	0.0	0.0	0.0	0.0	0.0
0.0	0.0	0.0	0.0	0.0	0.0	0.0	0.0
0.0	0.0	0.0	0.0	0.0	0.0	0.0	0.0
0.0	5.0	0.0	0.0	0.0	0.0	0.0	0.0
0.0	0.0	0.0	0.0	0.0	0.0	0.0	0.0
0.0	1.0	1.0	0.0	0.0	0.0	0.0	0.0
0.0	0.0	0.0	0.0	0.0	0.0	0.0	0.0
0.0	0.0	0.0	0.0	0.0	0.0	0.0	0.0
0.0	0.0	0.0	0.0	0.0	0.0	0.0	0.0
0.0	0.0	0.0	0.0	0.0	0.0	0.0	0.0
0.0	0.0	0.0	0.0	0.0	0.0	0.0	0.0
0.0	1.0	0.0	0.0	0.0	0.0	0.0	0.0
0.0	0.0	0.0	0.0	0.0	0.0	0.0	0.0
0.0	0.0	0.0	0.0	0.0	0.0	4.0	0.0
0.0	0.0	0.0	0.0	0.0	0.0	0.0	0.0
0.0	0.0	0.0	0.0	0.0	0.0	0.0	0.0
0.0	0.0	0.0	0.0	0.0	0.0	0.0	0.0
0.0	0.0	0.0	0.0	0.0	0.0	0.0	0.0
0.0	0.0	0.0	0.0	0.0	0.0	0.0	0.0
0.0	0.0	0.0	0.0	0.0	0.0	0.0	0.0
0.0	0.0	0.0	0.0	0.0	0.0	0.0	0.0
0.0	0.0	6.0	0.0	0.0	0.0	0.0	0.0
0.0	3.0	0.0	0.0	0.0	0.0	0.0	0.0
0.0	0.0	0.0	0.0	0.0	0.0	0.0	0.0
0.0	0.0	0.0	0.0	0.0	0.0	0.0	0.0
0.0	0.0	0.0	0.0	0.0	0.0	0.0	0.0
0.0	0.0	0.0	0.0	2.0	0.0	0.0	0.0
0.0	0.0	0.0	0.0	0.0	0.0	0.0	0.0
0.0	1.0	0.0	0.0	0.0	0.0	0.0	0.0
0.0	0.0	0.0	0.0	0.0	0.0	0.0	0.0
0.0	0.0	0.0	0.0	0.0	0.0	0.0	0.0
0.0	0.0	0.0	0.0	0.0	0.0	0.0	0.0
0.0	0.0	0.0	0.0	0.0	0.0	0.0	0.0
0.0	0.0	0.0	0.0	0.0	0.0	0.0	0.0
0.0	0.0	0.0	0.0	0.0	0.0	1.0	0.0

0.0	0.0	0.0	0.0	0.0	0.0	0.0	0.0
0.0	0.0	0.0	0.0	0.0	0.0	0.0	0.0
0.0	0.0	0.0	0.0	0.0	0.0	0.0	0.0
0.0	0.0	0.0	0.0	0.0	0.0	0.0	0.0
0.0	0.0	0.0	0.0	0.0	0.0	0.0	0.0
0.0	0.0	0.0	0.0	0.0	0.0	0.0	0.0
0.0	0.0	0.0	0.0	0.0	0.0	0.0	0.0
0.0	0.0	0.0	0.0	0.0	0.0	0.0	0.0
0.0	0.0	0.0	0.0	0.0	0.0	0.0	0.0
0.0	0.0	0.0	0.0	0.0	0.0	0.0	0.0
0.0	0.0	0.0	0.0	0.0	0.0	0.0	0.0
0.0	0.0	0.0	0.0	0.0	0.0	0.0	0.0
0.0	0.0	0.0	0.0	0.0	0.0	0.0	0.0
0.0	0.0	0.0	0.0	0.0	0.0	0.0	0.0
0.0	0.0	0.0	0.0	0.0	0.0	0.0	0.0
0.0	0.0	0.0	0.0	0.0	0.0	0.0	0.0
0.0	0.0	0.0	0.0	0.0	0.0	0.0	0.0
2.0	0.0	0.0	0.0	0.0	0.0	0.0	0.0
0.0	0.0	0.0	0.0	0.0	0.0	179.0	0.0
0.0	0.0	0.0	0.0	0.0	0.0	0.0	0.0
0.0	0.0	0.0	0.0	0.0	0.0	3.0	0.0
0.0	0.0	0.0	0.0	0.0	0.0	0.0	0.0
0.0	0.0	0.0	0.0	0.0	0.0	0.0	0.0
0.0	0.0	0.0	0.0	0.0	0.0	0.0	0.0
0.0	0.0	150.0	0.0	0.0	4.0	0.0	0.0
0.0	746.0	0.0	0.0	0.0	0.0	0.0	0.0
0.0	0.0	0.0	0.0	0.0	0.0	0.0	0.0
0.0	0.0	0.0	0.0	0.0	0.0	0.0	0.0
0.0	0.0	0.0	0.0	0.0	45.0	0.0	0.0
0.0	0.0	0.0	0.0	0.0	0.0	0.0	0.0
0.0	0.0	0.0	0.0	0.0	0.0	0.0	0.0
0.0	0.0	0.0	0.0	0.0	0.0	0.0	0.0
0.0	0.0	0.0	0.0	0.0	0.0	37.0	0.0
0.0	12.0	0.0	0.0	0.0	0.0	38.0	0.0
0.0	0.0	0.0	0.0	0.0	0.0	0.0	0.0
13.0	0.0	0.0	0.0	0.0	0.0	0.0	0.0
0.0	0.0	0.0	0.0	0.0	0.0	0.0	0.0
0.0	0.0	0.0	0.0	0.0	0.0	0.0	0.0
0.0	0.0	0.0	0.0	0.0	0.0	0.0	0.0
0.0	0.0	0.0	0.0	0.0	0.0	76.0	0.0
24.0	0.0	0.0	0.0	1.0	102.0	0.0	18.0
0.0	0.0	0.0	0.0	0.0	0.0	0.0	0.0
0.0	0.0	12.0	0.0	0.0	0.0	0.0	0.0
0.0	0.0	0.0	0.0	0.0	0.0	0.0	0.0
0.0	0.0	0.0	0.0	0.0	0.0	0.0	0.0
0.0	0.0	0.0	0.0	0.0	0.0	0.0	0.0
0.0	158.0	11.0	0.0	0.0	0.0	1.0	0.0

0.0	0.0	0.0	0.0	0.0	0.0	0.0	0.0
0.0	0.0	0.0	0.0	0.0	0.0	0.0	0.0
1.0	0.0	72.0	0.0	0.0	0.0	43.0	0.0
0.0	0.0	0.0	0.0	0.0	0.0	530.0	0.0
0.0	0.0	0.0	0.0	0.0	0.0	0.0	0.0
0.0	0.0	0.0	0.0	0.0	0.0	0.0	0.0
0.0	0.0	0.0	0.0	0.0	0.0	0.0	0.0
0.0	0.0	0.0	0.0	0.0	0.0	757.0	0.0
0.0	0.0	0.0	0.0	0.0	0.0	0.0	0.0
0.0	0.0	0.0	0.0	0.0	0.0	0.0	0.0
0.0	0.0	0.0	0.0	0.0	0.0	0.0	0.0
0.0	0.0	0.0	0.0	0.0	0.0	22.0	0.0
0.0	0.0	0.0	0.0	0.0	0.0	0.0	0.0
0.0	0.0	0.0	0.0	6.0	0.0	0.0	0.0
0.0	0.0	0.0	0.0	0.0	0.0	0.0	0.0
0.0	0.0	0.0	0.0	0.0	0.0	0.0	0.0
0.0	0.0	1.0	0.0	0.0	0.0	0.0	0.0
0.0	0.0	0.0	0.0	0.0	0.0	0.0	0.0
0.0	0.0	0.0	0.0	0.0	0.0	0.0	0.0
0.0	0.0	0.0	0.0	0.0	0.0	0.0	0.0
0.0	0.0	0.0	0.0	0.0	0.0	0.0	0.0
0.0	0.0	0.0	0.0	1.0	0.0	0.0	0.0
1.0	0.0	0.0	0.0	0.0	0.0	7.0	0.0
0.0	0.0	0.0	0.0	0.0	0.0	0.0	0.0
0.0	0.0	0.0	0.0	2.0	0.0	0.0	0.0
0.0	0.0	0.0	0.0	0.0	0.0	0.0	0.0
0.0	0.0	0.0	0.0	2.0	0.0	0.0	0.0
0.0	0.0	1.0	0.0	0.0	0.0	0.0	0.0
0.0	0.0	0.0	0.0	0.0	0.0	0.0	0.0
0.0	0.0	0.0	0.0	0.0	0.0	0.0	0.0
0.0	0.0	0.0	0.0	0.0	0.0	0.0	0.0
0.0	0.0	2.0	0.0	0.0	0.0	0.0	0.0
0.0	0.0	0.0	0.0	0.0	0.0	0.0	0.0
2.0	0.0	0.0	0.0	3.0	0.0	18.0	0.0
0.0	0.0	0.0	0.0	0.0	0.0	0.0	0.0
0.0	0.0	2.0	0.0	0.0	0.0	3.0	0.0
0.0	0.0	0.0	0.0	0.0	0.0	0.0	0.0
0.0	0.0	0.0	0.0	0.0	0.0	0.0	0.0
0.0	0.0	0.0	0.0	0.0	0.0	2.0	0.0
0.0	0.0	0.0	0.0	0.0	0.0	0.0	0.0
0.0	0.0	0.0	0.0	0.0	0.0	0.0	0.0
0.0	0.0	0.0	0.0	0.0	0.0	0.0	0.0
0.0	0.0	1.0	0.0	0.0	0.0	0.0	0.0
0.0	0.0	0.0	0.0	0.0	0.0	0.0	0.0
0.0	0.0	0.0	0.0	0.0	0.0	0.0	0.0
0.0	0.0	0.0	0.0	0.0	0.0	0.0	0.0
0.0	0.0	0.0	0.0	0.0	0.0	0.0	0.0

0.0	0.0	0.0	0.0	0.0	0.0	0.0	0.0
0.0	0.0	0.0	0.0	0.0	0.0	0.0	0.0
0.0	0.0	0.0	0.0	0.0	0.0	0.0	0.0
0.0	0.0	0.0	0.0	0.0	0.0	0.0	0.0
0.0	0.0	0.0	0.0	0.0	0.0	0.0	0.0
0.0	0.0	0.0	0.0	0.0	0.0	0.0	0.0
0.0	0.0	0.0	0.0	0.0	0.0	0.0	0.0
0.0	0.0	0.0	0.0	0.0	0.0	0.0	0.0
0.0	0.0	0.0	0.0	0.0	0.0	0.0	0.0
0.0	0.0	0.0	0.0	0.0	0.0	0.0	0.0
0.0	87.0	0.0	0.0	0.0	0.0	0.0	0.0
0.0	0.0	0.0	0.0	0.0	0.0	0.0	0.0
0.0	0.0	0.0	0.0	0.0	0.0	0.0	0.0
1.0	0.0	0.0	0.0	0.0	0.0	0.0	0.0
0.0	0.0	0.0	0.0	0.0	0.0	0.0	0.0
2.0	0.0	0.0	0.0	0.0	5.0	0.0	0.0
0.0	0.0	0.0	0.0	0.0	0.0	0.0	0.0
0.0	0.0	0.0	0.0	0.0	0.0	0.0	0.0
0.0	0.0	0.0	0.0	0.0	0.0	0.0	0.0
0.0	0.0	0.0	0.0	0.0	0.0	0.0	0.0
0.0	0.0	0.0	0.0	0.0	0.0	7.0	0.0
0.0	0.0	0.0	0.0	0.0	0.0	0.0	0.0
0.0	0.0	0.0	0.0	0.0	0.0	0.0	0.0
0.0	0.0	0.0	0.0	0.0	0.0	0.0	0.0
0.0	0.0	0.0	0.0	0.0	0.0	0.0	0.0
0.0	0.0	0.0	0.0	0.0	0.0	0.0	0.0
0.0	0.0	41.0	0.0	0.0	0.0	14.0	0.0
0.0	0.0	0.0	0.0	0.0	0.0	0.0	0.0
0.0	0.0	0.0	0.0	0.0	0.0	0.0	0.0
0.0	0.0	0.0	0.0	0.0	0.0	13.0	0.0
0.0	0.0	0.0	0.0	0.0	0.0	0.0	0.0
0.0	0.0	0.0	0.0	0.0	0.0	0.0	0.0
0.0	0.0	0.0	0.0	0.0	0.0	0.0	0.0
1.0	0.0	0.0	0.0	0.0	0.0	0.0	0.0
0.0	0.0	0.0	0.0	0.0	0.0	0.0	0.0
0.0	0.0	0.0	0.0	0.0	0.0	9.0	0.0
0.0	0.0	8.0	0.0	0.0	0.0	0.0	0.0
0.0	0.0	0.0	0.0	0.0	0.0	2.0	0.0
0.0	0.0	0.0	0.0	0.0	0.0	0.0	0.0
0.0	0.0	0.0	0.0	0.0	0.0	54.0	0.0
1.0	0.0	96.0	0.0	0.0	0.0	6.0	2.0
0.0	0.0	0.0	0.0	0.0	0.0	17.0	0.0
0.0	0.0	0.0	0.0	0.0	0.0	0.0	0.0
0.0	0.0	0.0	0.0	0.0	0.0	10.0	0.0
0.0	0.0	0.0	0.0	0.0	0.0	0.0	0.0
0.0	0.0	0.0	0.0	0.0	1.0	0.0	0.0
0.0	0.0	0.0	0.0	0.0	0.0	4.0	0.0

0.0	0.0	0.0	0.0	0.0	0.0	0.0	0.0
0.0	0.0	0.0	0.0	0.0	0.0	0.0	0.0
0.0	0.0	0.0	0.0	0.0	0.0	4.0	0.0
0.0	0.0	4.0	0.0	0.0	0.0	0.0	0.0
0.0	0.0	0.0	0.0	0.0	0.0	0.0	0.0
0.0	0.0	0.0	0.0	0.0	0.0	0.0	0.0
0.0	0.0	0.0	0.0	0.0	0.0	0.0	0.0
0.0	0.0	0.0	0.0	0.0	0.0	0.0	0.0
0.0	0.0	0.0	0.0	0.0	0.0	0.0	0.0
0.0	0.0	0.0	0.0	0.0	0.0	44.0	0.0
0.0	0.0	0.0	0.0	0.0	0.0	0.0	0.0
0.0	0.0	0.0	0.0	0.0	0.0	0.0	0.0
0.0	0.0	0.0	0.0	0.0	0.0	0.0	0.0
0.0	0.0	0.0	0.0	0.0	0.0	0.0	0.0
0.0	0.0	0.0	0.0	0.0	0.0	4.0	0.0
0.0	0.0	0.0	0.0	0.0	0.0	2.0	0.0
0.0	0.0	0.0	0.0	0.0	1.0	0.0	0.0
0.0	0.0	0.0	0.0	3.0	0.0	0.0	0.0
0.0	0.0	0.0	0.0	0.0	0.0	0.0	0.0
0.0	0.0	0.0	0.0	0.0	0.0	0.0	0.0
0.0	0.0	0.0	0.0	0.0	0.0	0.0	0.0
0.0	0.0	0.0	0.0	0.0	0.0	0.0	0.0
0.0	0.0	0.0	0.0	0.0	0.0	0.0	0.0
0.0	0.0	3.0	0.0	0.0	0.0	2.0	0.0
0.0	0.0	0.0	0.0	0.0	0.0	10.0	0.0
1.0	0.0	0.0	0.0	0.0	0.0	10.0	0.0
0.0	0.0	0.0	0.0	0.0	0.0	0.0	0.0
2.0	0.0	0.0	0.0	0.0	0.0	0.0	0.0
0.0	0.0	0.0	0.0	0.0	0.0	0.0	0.0
0.0	0.0	0.0	0.0	3.0	0.0	0.0	0.0
0.0	0.0	0.0	0.0	0.0	0.0	0.0	0.0
0.0	0.0	0.0	0.0	0.0	0.0	0.0	0.0
0.0	0.0	0.0	0.0	0.0	0.0	0.0	0.0
0.0	0.0	0.0	0.0	0.0	0.0	0.0	0.0
0.0	0.0	0.0	0.0	0.0	0.0	3.0	0.0
0.0	0.0	2.0	0.0	0.0	0.0	1.0	0.0
0.0	0.0	0.0	0.0	0.0	0.0	1.0	0.0
0.0	0.0	0.0	0.0	0.0	0.0	0.0	0.0
0.0	0.0	0.0	0.0	0.0	0.0	0.0	0.0
0.0	0.0	0.0	0.0	0.0	0.0	0.0	0.0
0.0	0.0	0.0	0.0	3.0	0.0	0.0	0.0
0.0	0.0	0.0	0.0	0.0	0.0	4.0	0.0
0.0	0.0	0.0	0.0	0.0	0.0	0.0	0.0
0.0	0.0	0.0	0.0	2.0	1.0	0.0	0.0
0.0	0.0	0.0	0.0	0.0	0.0	0.0	2.0
0.0	0.0	0.0	0.0	0.0	0.0	0.0	0.0
0.0	0.0	0.0	0.0	0.0	0.0	0.0	0.0

0.0	0.0	0.0	0.0	0.0	0.0	31.0	0.0
0.0	0.0	0.0	0.0	0.0	0.0	0.0	0.0
0.0	0.0	0.0	0.0	0.0	0.0	0.0	0.0
0.0	0.0	0.0	0.0	1.0	0.0	0.0	0.0
0.0	0.0	0.0	0.0	0.0	0.0	0.0	0.0
0.0	0.0	0.0	0.0	0.0	0.0	0.0	0.0
0.0	0.0	0.0	0.0	0.0	0.0	0.0	0.0
0.0	0.0	0.0	0.0	0.0	0.0	0.0	0.0
0.0	0.0	0.0	0.0	0.0	0.0	0.0	1203.0
0.0	0.0	0.0	0.0	0.0	0.0	0.0	0.0
0.0	0.0	0.0	0.0	0.0	0.0	0.0	0.0
0.0	0.0	0.0	0.0	0.0	0.0	0.0	0.0
0.0	0.0	0.0	0.0	0.0	0.0	0.0	0.0
0.0	0.0	0.0	0.0	0.0	0.0	0.0	0.0
0.0	0.0	0.0	0.0	0.0	0.0	1.0	0.0
0.0	0.0	0.0	0.0	0.0	0.0	0.0	0.0
0.0	0.0	2.0	0.0	0.0	0.0	0.0	0.0
0.0	0.0	0.0	0.0	0.0	0.0	0.0	0.0
0.0	0.0	0.0	0.0	0.0	0.0	0.0	0.0
0.0	0.0	0.0	0.0	0.0	0.0	0.0	0.0
0.0	0.0	0.0	0.0	0.0	0.0	0.0	0.0
0.0	0.0	0.0	0.0	0.0	0.0	0.0	0.0
0.0	0.0	0.0	0.0	0.0	0.0	0.0	0.0
0.0	0.0	0.0	0.0	0.0	0.0	0.0	0.0
0.0	0.0	0.0	0.0	0.0	0.0	0.0	0.0
0.0	0.0	0.0	0.0	0.0	0.0	0.0	0.0
0.0	0.0	0.0	0.0	0.0	0.0	0.0	0.0
0.0	0.0	0.0	0.0	0.0	0.0	0.0	0.0
0.0	0.0	0.0	0.0	0.0	0.0	0.0	0.0
0.0	0.0	0.0	0.0	0.0	0.0	0.0	0.0
0.0	0.0	0.0	0.0	0.0	0.0	0.0	0.0
0.0	0.0	0.0	0.0	0.0	0.0	0.0	0.0
0.0	0.0	0.0	0.0	0.0	0.0	0.0	0.0
0.0	0.0	0.0	0.0	0.0	0.0	0.0	0.0
0.0	0.0	0.0	0.0	0.0	0.0	0.0	0.0
0.0	0.0	0.0	0.0	0.0	0.0	0.0	0.0
0.0	0.0	0.0	0.0	0.0	0.0	0.0	0.0
0.0	0.0	0.0	0.0	0.0	0.0	0.0	0.0
725.0	0.0	0.0	0.0	0.0	0.0	0.0	0.0
0.0	0.0	0.0	0.0	0.0	0.0	0.0	0.0
0.0	0.0	0.0	0.0	0.0	0.0	0.0	0.0
0.0	0.0	0.0	0.0	0.0	0.0	0.0	0.0
0.0	0.0	0.0	0.0	0.0	0.0	0.0	0.0
0.0	0.0	0.0	0.0	0.0	0.0	0.0	0.0
0.0	0.0	0.0	0.0	0.0	0.0	0.0	0.0
0.0	0.0	0.0	0.0	0.0	0.0	0.0	0.0

0.0	0.0	0.0	0.0	0.0	0.0	1.0	0.0
0.0	0.0	17.0	0.0	0.0	0.0	253.0	0.0
0.0	0.0	0.0	0.0	0.0	0.0	0.0	0.0
0.0	0.0	0.0	0.0	0.0	0.0	0.0	0.0
0.0	0.0	0.0	0.0	0.0	0.0	0.0	0.0
0.0	0.0	0.0	0.0	0.0	0.0	0.0	0.0
0.0	0.0	0.0	0.0	0.0	0.0	0.0	0.0
0.0	0.0	0.0	0.0	0.0	0.0	0.0	0.0
0.0	0.0	0.0	0.0	0.0	0.0	0.0	0.0
0.0	0.0	0.0	0.0	0.0	0.0	0.0	0.0
0.0	0.0	0.0	0.0	0.0	0.0	0.0	0.0
0.0	0.0	0.0	0.0	0.0	0.0	0.0	0.0
3.0	0.0	0.0	0.0	0.0	0.0	0.0	0.0
0.0	0.0	0.0	0.0	0.0	0.0	0.0	0.0
0.0	0.0	0.0	0.0	0.0	0.0	0.0	0.0
0.0	0.0	0.0	0.0	0.0	0.0	0.0	0.0
0.0	0.0	0.0	0.0	0.0	0.0	0.0	0.0
0.0	0.0	0.0	0.0	0.0	0.0	0.0	0.0
0.0	0.0	0.0	0.0	0.0	0.0	0.0	0.0
0.0	0.0	0.0	0.0	0.0	0.0	0.0	0.0
0.0	0.0	0.0	0.0	0.0	0.0	0.0	0.0
0.0	0.0	0.0	0.0	0.0	0.0	0.0	0.0
0.0	0.0	0.0	0.0	0.0	0.0	0.0	0.0
0.0	0.0	0.0	0.0	0.0	0.0	0.0	0.0
0.0	0.0	0.0	0.0	0.0	0.0	0.0	0.0
0.0	0.0	0.0	0.0	0.0	0.0	0.0	0.0
0.0	0.0	0.0	0.0	0.0	0.0	0.0	0.0
2.0	3.0	0.0	3.0	0.0	0.0	0.0	0.0
0.0	0.0	0.0	0.0	0.0	0.0	0.0	0.0
0.0	0.0	0.0	0.0	0.0	0.0	0.0	0.0
0.0	0.0	0.0	0.0	0.0	0.0	0.0	0.0
0.0	0.0	0.0	0.0	0.0	0.0	0.0	0.0
0.0	0.0	0.0	0.0	0.0	0.0	0.0	0.0
0.0	0.0	0.0	0.0	0.0	0.0	0.0	0.0
0.0	0.0	0.0	0.0	0.0	0.0	0.0	0.0
1.0	0.0	0.0	0.0	0.0	0.0	0.0	0.0
0.0	0.0	0.0	0.0	0.0	0.0	0.0	0.0
0.0	0.0	0.0	0.0	0.0	0.0	0.0	0.0
0.0	0.0	0.0	0.0	0.0	0.0	0.0	0.0
0.0	0.0	0.0	0.0	0.0	0.0	0.0	0.0
0.0	0.0	0.0	0.0	0.0	0.0	0.0	0.0
0.0	0.0	0.0	0.0	0.0	0.0	0.0	0.0
0.0	0.0	0.0	0.0	0.0	0.0	0.0	0.0
0.0	0.0	0.0	0.0	0.0	0.0	0.0	0.0
1.0	0.0	0.0	0.0	1.0	0.0	0.0	0.0
0.0	7.0	0.0	0.0	0.0	0.0	0.0	0.0
0.0	0.0	0.0	0.0	0.0	0.0	0.0	0.0

0.0	0.0	0.0	0.0	0.0	0.0	0.0	0.0
0.0	0.0	0.0	0.0	0.0	0.0	0.0	0.0
0.0	0.0	0.0	0.0	0.0	0.0	0.0	0.0
0.0	0.0	0.0	0.0	0.0	0.0	0.0	0.0
0.0	0.0	0.0	0.0	0.0	0.0	0.0	0.0
0.0	0.0	0.0	0.0	0.0	0.0	0.0	0.0
0.0	0.0	0.0	0.0	0.0	0.0	0.0	0.0
0.0	0.0	0.0	0.0	0.0	0.0	0.0	0.0
0.0	0.0	0.0	0.0	0.0	0.0	0.0	0.0
0.0	0.0	0.0	0.0	0.0	0.0	2.0	0.0
0.0	0.0	0.0	0.0	0.0	0.0	0.0	0.0
0.0	0.0	0.0	0.0	0.0	0.0	0.0	0.0
0.0	0.0	0.0	0.0	0.0	0.0	0.0	0.0
0.0	0.0	0.0	0.0	0.0	0.0	0.0	0.0
0.0	0.0	0.0	0.0	0.0	0.0	0.0	0.0
0.0	0.0	0.0	0.0	0.0	0.0	0.0	0.0
0.0	0.0	0.0	0.0	0.0	0.0	0.0	0.0
0.0	0.0	2.0	0.0	0.0	0.0	0.0	0.0
0.0	0.0	0.0	0.0	0.0	0.0	0.0	0.0
0.0	0.0	0.0	0.0	0.0	0.0	0.0	0.0
0.0	5.0	0.0	0.0	1.0	0.0	0.0	2.0
0.0	0.0	1.0	0.0	0.0	0.0	0.0	0.0
0.0	0.0	0.0	0.0	0.0	0.0	0.0	0.0
0.0	0.0	0.0	0.0	0.0	0.0	106.0	0.0
0.0	0.0	0.0	0.0	0.0	0.0	0.0	0.0
0.0	0.0	0.0	0.0	0.0	0.0	0.0	0.0
0.0	0.0	0.0	0.0	0.0	0.0	0.0	0.0
0.0	0.0	0.0	0.0	0.0	0.0	0.0	0.0
0.0	0.0	0.0	0.0	0.0	0.0	0.0	0.0
0.0	0.0	2.0	0.0	0.0	0.0	0.0	0.0
0.0	0.0	0.0	0.0	0.0	0.0	0.0	0.0
0.0	0.0	0.0	12.0	0.0	0.0	0.0	0.0
2.0	0.0	0.0	0.0	2.0	0.0	0.0	0.0
0.0	0.0	0.0	0.0	0.0	0.0	0.0	0.0
0.0	0.0	0.0	0.0	0.0	1.0	0.0	0.0
0.0	0.0	0.0	0.0	0.0	0.0	0.0	0.0
0.0	0.0	0.0	0.0	0.0	0.0	0.0	0.0
0.0	0.0	0.0	0.0	0.0	0.0	0.0	0.0
0.0	0.0	0.0	0.0	0.0	0.0	0.0	0.0
0.0	0.0	0.0	0.0	0.0	0.0	0.0	0.0
0.0	0.0	0.0	0.0	0.0	0.0	0.0	0.0
0.0	0.0	0.0	0.0	0.0	0.0	0.0	0.0
0.0	0.0	0.0	0.0	0.0	0.0	0.0	0.0
9.0	0.0	0.0	0.0	0.0	0.0	0.0	0.0
0.0	0.0	0.0	0.0	0.0	0.0	0.0	0.0
0.0	0.0	0.0	0.0	0.0	0.0	0.0	0.0
0.0	0.0	0.0	0.0	0.0	0.0	0.0	0.0

0.0	0.0	0.0	0.0	0.0	0.0	0.0	0.0
0.0	0.0	0.0	0.0	0.0	0.0	0.0	0.0
0.0	0.0	0.0	0.0	0.0	0.0	0.0	0.0
0.0	0.0	0.0	0.0	0.0	3.0	0.0	0.0
0.0	0.0	0.0	0.0	0.0	0.0	0.0	0.0
0.0	0.0	0.0	0.0	0.0	0.0	0.0	0.0
0.0	0.0	0.0	0.0	0.0	0.0	0.0	0.0
0.0	0.0	0.0	0.0	0.0	0.0	0.0	0.0
0.0	0.0	0.0	0.0	0.0	0.0	0.0	0.0
0.0	0.0	0.0	0.0	0.0	0.0	0.0	0.0
0.0	0.0	0.0	0.0	0.0	0.0	0.0	0.0
0.0	0.0	0.0	0.0	0.0	0.0	0.0	0.0
0.0	0.0	0.0	0.0	0.0	0.0	0.0	0.0
0.0	0.0	0.0	0.0	0.0	0.0	0.0	0.0
0.0	0.0	0.0	0.0	0.0	0.0	0.0	0.0
0.0	0.0	0.0	0.0	0.0	0.0	0.0	0.0
0.0	0.0	0.0	0.0	0.0	0.0	0.0	0.0
18.0	0.0	0.0	0.0	0.0	0.0	10.0	0.0
0.0	0.0	0.0	0.0	0.0	0.0	0.0	0.0
0.0	0.0	0.0	0.0	0.0	0.0	0.0	0.0
0.0	0.0	0.0	0.0	0.0	0.0	0.0	0.0
0.0	0.0	0.0	0.0	0.0	0.0	0.0	0.0
0.0	0.0	0.0	0.0	0.0	0.0	0.0	0.0
0.0	0.0	0.0	0.0	0.0	0.0	0.0	0.0
0.0	0.0	0.0	0.0	0.0	0.0	0.0	0.0
0.0	0.0	0.0	0.0	0.0	0.0	0.0	0.0
0.0	0.0	0.0	0.0	0.0	0.0	0.0	0.0
0.0	0.0	0.0	0.0	0.0	0.0	0.0	0.0
0.0	0.0	0.0	0.0	0.0	0.0	0.0	0.0
0.0	0.0	0.0	0.0	0.0	0.0	0.0	0.0
0.0	0.0	0.0	0.0	0.0	0.0	0.0	0.0
0.0	0.0	0.0	0.0	0.0	0.0	0.0	0.0
0.0	0.0	0.0	0.0	0.0	0.0	0.0	0.0
0.0	0.0	2.0	0.0	2.0	0.0	0.0	0.0
0.0	0.0	0.0	0.0	0.0	0.0	0.0	0.0
0.0	0.0	0.0	0.0	0.0	0.0	0.0	0.0
0.0	0.0	0.0	0.0	0.0	0.0	0.0	0.0
1.0	1.0	0.0	52.0	0.0	0.0	0.0	0.0
2.0	0.0	0.0	0.0	0.0	0.0	0.0	0.0
0.0	0.0	0.0	0.0	0.0	0.0	0.0	0.0
0.0	0.0	0.0	0.0	0.0	0.0	0.0	0.0
0.0	0.0	0.0	0.0	0.0	0.0	0.0	0.0
0.0	0.0	0.0	0.0	0.0	0.0	0.0	0.0
0.0	0.0	0.0	0.0	0.0	0.0	0.0	0.0
0.0	0.0	0.0	0.0	0.0	0.0	0.0	0.0
0.0	0.0	0.0	0.0	0.0	0.0	0.0	0.0
0.0	0.0	0.0	0.0	0.0	0.0	0.0	0.0

0.0	0.0	0.0	0.0	0.0	0.0	0.0	0.0
0.0	0.0	0.0	0.0	0.0	0.0	0.0	0.0
0.0	0.0	0.0	0.0	0.0	0.0	0.0	0.0
0.0	0.0	0.0	0.0	0.0	2.0	0.0	0.0
0.0	0.0	0.0	0.0	0.0	0.0	0.0	0.0
0.0	0.0	0.0	0.0	0.0	0.0	0.0	0.0
0.0	0.0	0.0	0.0	0.0	0.0	0.0	0.0
0.0	0.0	0.0	0.0	0.0	0.0	0.0	0.0
0.0	0.0	0.0	0.0	0.0	0.0	0.0	0.0
0.0	0.0	0.0	0.0	0.0	0.0	0.0	0.0
0.0	0.0	0.0	0.0	0.0	0.0	0.0	0.0
0.0	0.0	0.0	0.0	0.0	0.0	0.0	0.0
0.0	0.0	0.0	0.0	0.0	0.0	0.0	0.0
0.0	0.0	0.0	0.0	0.0	0.0	0.0	0.0
0.0	0.0	0.0	0.0	0.0	0.0	0.0	0.0
0.0	0.0	0.0	0.0	0.0	0.0	2.0	0.0
0.0	0.0	0.0	0.0	1.0	0.0	0.0	0.0
0.0	0.0	0.0	0.0	0.0	0.0	0.0	0.0
0.0	0.0	0.0	0.0	0.0	0.0	0.0	0.0
0.0	0.0	0.0	0.0	0.0	0.0	0.0	0.0
0.0	0.0	0.0	0.0	0.0	0.0	2.0	0.0
0.0	0.0	0.0	0.0	0.0	0.0	0.0	0.0
0.0	0.0	0.0	0.0	0.0	0.0	0.0	0.0
0.0	0.0	0.0	0.0	0.0	0.0	0.0	0.0
0.0	0.0	0.0	0.0	0.0	0.0	0.0	0.0
0.0	0.0	0.0	0.0	0.0	0.0	0.0	0.0
0.0	0.0	0.0	0.0	0.0	0.0	0.0	0.0
0.0	0.0	0.0	0.0	0.0	0.0	0.0	0.0
0.0	0.0	0.0	0.0	0.0	0.0	2.0	0.0
0.0	0.0	0.0	0.0	0.0	0.0	0.0	0.0
0.0	0.0	0.0	0.0	0.0	0.0	4.0	0.0
0.0	0.0	0.0	0.0	0.0	0.0	0.0	0.0
0.0	0.0	0.0	0.0	0.0	0.0	0.0	0.0
0.0	0.0	0.0	0.0	0.0	0.0	0.0	0.0
0.0	0.0	0.0	0.0	0.0	0.0	0.0	0.0
0.0	0.0	0.0	0.0	0.0	0.0	0.0	0.0
0.0	0.0	0.0	0.0	0.0	0.0	0.0	0.0
0.0	0.0	0.0	0.0	0.0	0.0	0.0	0.0
0.0	0.0	21.0	1.0	0.0	1.0	0.0	0.0
0.0	0.0	0.0	0.0	0.0	0.0	0.0	0.0
0.0	0.0	0.0	0.0	0.0	0.0	0.0	0.0
0.0	0.0	0.0	0.0	0.0	0.0	0.0	0.0
0.0	0.0	0.0	0.0	0.0	0.0	0.0	0.0
0.0	0.0	0.0	0.0	0.0	0.0	0.0	0.0
0.0	0.0	0.0	0.0	0.0	0.0	0.0	0.0
0.0	0.0	0.0	0.0	0.0	0.0	0.0	0.0
0.0	0.0	0.0	0.0	0.0	0.0	0.0	0.0

0.0	0.0	0.0	0.0	0.0	0.0	0.0	0.0
0.0	0.0	0.0	0.0	0.0	0.0	0.0	0.0
0.0	0.0	0.0	0.0	0.0	0.0	0.0	0.0
0.0	0.0	0.0	0.0	0.0	0.0	0.0	0.0
0.0	0.0	0.0	0.0	0.0	0.0	0.0	0.0
0.0	0.0	0.0	0.0	0.0	0.0	0.0	0.0
0.0	0.0	0.0	0.0	0.0	0.0	0.0	0.0
0.0	0.0	0.0	0.0	0.0	0.0	0.0	0.0
0.0	0.0	0.0	0.0	0.0	0.0	7.0	0.0
0.0	0.0	0.0	0.0	1.0	0.0	0.0	0.0
0.0	0.0	0.0	0.0	0.0	0.0	0.0	0.0
0.0	0.0	0.0	0.0	0.0	0.0	0.0	0.0
0.0	0.0	0.0	0.0	0.0	0.0	0.0	0.0
0.0	0.0	0.0	0.0	0.0	0.0	0.0	0.0
0.0	0.0	0.0	0.0	0.0	0.0	0.0	0.0
0.0	0.0	0.0	0.0	0.0	0.0	0.0	0.0
0.0	0.0	0.0	0.0	0.0	0.0	11.0	0.0
0.0	0.0	0.0	0.0	0.0	0.0	0.0	0.0
0.0	0.0	0.0	0.0	1.0	0.0	0.0	0.0
0.0	0.0	0.0	0.0	0.0	0.0	0.0	0.0
0.0	0.0	2.0	0.0	0.0	0.0	0.0	0.0
0.0	0.0	0.0	0.0	0.0	0.0	0.0	0.0
0.0	0.0	0.0	0.0	0.0	0.0	0.0	0.0
0.0	0.0	0.0	0.0	0.0	0.0	0.0	0.0
0.0	0.0	3.0	0.0	0.0	0.0	0.0	0.0
0.0	0.0	0.0	0.0	0.0	0.0	0.0	0.0
0.0	0.0	0.0	0.0	0.0	0.0	0.0	0.0
0.0	0.0	0.0	0.0	0.0	0.0	0.0	0.0
0.0	0.0	0.0	0.0	0.0	0.0	0.0	0.0
0.0	0.0	0.0	0.0	0.0	0.0	0.0	0.0
0.0	0.0	0.0	0.0	0.0	0.0	0.0	0.0
0.0	0.0	0.0	0.0	0.0	0.0	0.0	0.0
0.0	0.0	0.0	0.0	0.0	0.0	0.0	0.0
0.0	0.0	0.0	0.0	0.0	0.0	0.0	0.0
0.0	0.0	0.0	0.0	0.0	0.0	0.0	0.0
0.0	0.0	0.0	0.0	0.0	0.0	0.0	0.0
0.0	0.0	0.0	0.0	0.0	0.0	0.0	0.0
0.0	0.0	0.0	0.0	0.0	0.0	0.0	0.0
0.0	0.0	0.0	0.0	0.0	0.0	0.0	0.0
0.0	0.0	0.0	0.0	0.0	1.0	0.0	0.0
0.0	0.0	0.0	0.0	0.0	0.0	0.0	0.0
0.0	0.0	5.0	0.0	0.0	0.0	0.0	0.0
0.0	0.0	0.0	0.0	0.0	0.0	0.0	0.0
0.0	0.0	0.0	0.0	0.0	0.0	0.0	0.0
0.0	0.0	0.0	0.0	0.0	0.0	0.0	0.0
0.0	0.0	0.0	0.0	0.0	0.0	0.0	0.0
0.0	0.0	0.0	0.0	0.0	0.0	0.0	0.0

0.0	0.0	0.0	0.0	0.0	0.0	0.0	0.0
0.0	0.0	0.0	0.0	0.0	0.0	0.0	0.0
0.0	0.0	0.0	0.0	0.0	0.0	0.0	0.0
0.0	0.0	0.0	0.0	0.0	0.0	0.0	0.0
0.0	0.0	0.0	0.0	0.0	0.0	0.0	0.0
0.0	0.0	0.0	0.0	0.0	0.0	0.0	0.0
0.0	0.0	0.0	0.0	0.0	0.0	0.0	0.0
0.0	0.0	0.0	0.0	0.0	0.0	0.0	0.0
0.0	0.0	0.0	0.0	0.0	0.0	0.0	0.0
0.0	0.0	0.0	0.0	0.0	0.0	0.0	0.0
0.0	0.0	0.0	0.0	0.0	0.0	0.0	0.0
0.0	0.0	0.0	0.0	0.0	0.0	0.0	0.0
0.0	0.0	0.0	0.0	0.0	0.0	0.0	0.0
0.0	0.0	0.0	0.0	0.0	0.0	0.0	0.0
0.0	0.0	0.0	0.0	0.0	0.0	0.0	0.0
0.0	0.0	0.0	0.0	0.0	0.0	0.0	0.0
0.0	0.0	0.0	0.0	0.0	0.0	0.0	0.0
0.0	0.0	0.0	0.0	0.0	0.0	0.0	0.0
0.0	0.0	0.0	0.0	0.0	0.0	0.0	0.0
0.0	0.0	0.0	0.0	0.0	0.0	0.0	0.0
0.0	0.0	0.0	0.0	0.0	0.0	0.0	0.0
0.0	0.0	0.0	0.0	0.0	0.0	0.0	0.0
0.0	0.0	0.0	0.0	0.0	0.0	0.0	0.0
0.0	0.0	0.0	0.0	0.0	0.0	0.0	0.0
0.0	0.0	0.0	0.0	0.0	0.0	0.0	0.0
0.0	0.0	0.0	0.0	0.0	0.0	0.0	0.0
0.0	0.0	0.0	0.0	0.0	0.0	0.0	0.0
0.0	0.0	0.0	0.0	0.0	0.0	0.0	0.0
0.0	2.0	0.0	0.0	0.0	0.0	0.0	0.0
0.0	0.0	0.0	0.0	0.0	0.0	0.0	0.0
0.0	0.0	0.0	0.0	0.0	0.0	0.0	0.0
0.0	0.0	0.0	0.0	0.0	2.0	0.0	0.0
1.0	0.0	6.0	0.0	0.0	0.0	0.0	0.0
0.0	0.0	0.0	0.0	0.0	0.0	0.0	0.0
0.0	0.0	0.0	0.0	0.0	0.0	0.0	0.0
0.0	0.0	0.0	0.0	0.0	0.0	0.0	0.0
0.0	0.0	0.0	0.0	0.0	0.0	0.0	0.0
0.0	0.0	0.0	0.0	0.0	0.0	0.0	0.0
0.0	0.0	0.0	0.0	0.0	0.0	0.0	0.0
0.0	0.0	0.0	0.0	0.0	0.0	0.0	0.0
0.0	0.0	0.0	0.0	0.0	0.0	0.0	0.0
0.0	0.0	0.0	0.0	0.0	4.0	0.0	0.0
0.0	0.0	0.0	0.0	0.0	0.0	0.0	0.0
0.0	1.0	0.0	0.0	0.0	0.0	60.0	0.0
0.0	0.0	0.0	0.0	0.0	0.0	0.0	0.0
0.0	0.0	0.0	0.0	0.0	0.0	0.0	0.0
0.0	0.0	0.0	0.0	0.0	0.0	0.0	0.0

0.0	0.0	0.0	0.0	0.0	0.0	0.0	0.0
0.0	0.0	0.0	0.0	0.0	0.0	0.0	0.0
0.0	0.0	0.0	0.0	0.0	0.0	0.0	0.0
0.0	0.0	0.0	0.0	0.0	0.0	17.0	0.0
0.0	0.0	0.0	0.0	0.0	2.0	0.0	0.0
0.0	0.0	0.0	0.0	0.0	0.0	0.0	0.0
0.0	0.0	0.0	0.0	2.0	0.0	0.0	0.0
0.0	0.0	0.0	0.0	0.0	0.0	0.0	0.0
0.0	0.0	0.0	0.0	0.0	0.0	0.0	0.0
0.0	0.0	0.0	0.0	0.0	0.0	0.0	0.0
0.0	0.0	0.0	0.0	0.0	0.0	0.0	0.0
0.0	0.0	0.0	0.0	0.0	0.0	3.0	0.0
0.0	0.0	0.0	0.0	0.0	0.0	0.0	0.0
0.0	0.0	0.0	0.0	0.0	0.0	0.0	0.0
0.0	0.0	0.0	0.0	0.0	0.0	0.0	0.0
0.0	0.0	0.0	0.0	0.0	0.0	0.0	0.0
0.0	0.0	0.0	0.0	0.0	0.0	0.0	0.0
0.0	0.0	0.0	0.0	0.0	0.0	0.0	0.0
0.0	0.0	0.0	0.0	0.0	0.0	0.0	0.0
0.0	0.0	0.0	0.0	0.0	0.0	0.0	0.0
0.0	0.0	0.0	0.0	0.0	0.0	0.0	0.0
0.0	0.0	0.0	0.0	0.0	0.0	0.0	0.0
0.0	0.0	0.0	0.0	0.0	0.0	0.0	0.0
0.0	0.0	0.0	0.0	0.0	0.0	0.0	0.0
0.0	0.0	0.0	0.0	0.0	0.0	0.0	0.0
0.0	0.0	0.0	0.0	0.0	0.0	0.0	0.0
0.0	0.0	0.0	0.0	0.0	0.0	0.0	0.0
0.0	0.0	0.0	0.0	0.0	0.0	0.0	0.0
0.0	0.0	0.0	0.0	0.0	0.0	0.0	0.0
0.0	0.0	0.0	0.0	0.0	0.0	0.0	0.0
0.0	0.0	0.0	0.0	0.0	0.0	0.0	0.0
0.0	0.0	0.0	0.0	0.0	0.0	0.0	0.0
0.0	0.0	0.0	0.0	0.0	0.0	0.0	0.0
0.0	0.0	0.0	0.0	0.0	0.0	0.0	0.0
0.0	0.0	0.0	0.0	0.0	0.0	0.0	0.0
0.0	0.0	0.0	0.0	0.0	0.0	0.0	0.0
0.0	0.0	0.0	0.0	0.0	0.0	0.0	0.0
0.0	0.0	0.0	0.0	0.0	0.0	0.0	0.0
0.0	0.0	0.0	0.0	0.0	0.0	0.0	0.0
0.0	0.0	0.0	0.0	0.0	0.0	0.0	0.0
0.0	0.0	0.0	0.0	0.0	0.0	0.0	0.0
0.0	0.0	0.0	0.0	0.0	0.0	0.0	0.0
0.0	0.0	0.0	0.0	0.0	0.0	0.0	0.0
0.0	0.0	0.0	0.0	0.0	0.0	0.0	0.0
0.0	0.0	0.0	0.0	0.0	0.0	0.0	0.0
0.0	0.0	0.0	0.0	0.0	0.0	2.0	0.0
0.0	0.0	0.0	0.0	0.0	0.0	0.0	0.0

0.0	0.0	0.0	0.0	0.0	0.0	0.0	0.0
0.0	0.0	14.0	0.0	0.0	0.0	0.0	0.0
0.0	0.0	0.0	0.0	0.0	0.0	0.0	0.0
0.0	0.0	0.0	0.0	0.0	0.0	0.0	0.0
0.0	0.0	0.0	0.0	0.0	0.0	0.0	0.0
0.0	0.0	0.0	0.0	0.0	0.0	0.0	0.0
0.0	0.0	0.0	0.0	0.0	0.0	0.0	0.0
0.0	0.0	0.0	0.0	0.0	0.0	0.0	0.0
0.0	0.0	0.0	0.0	0.0	0.0	0.0	0.0
0.0	0.0	0.0	0.0	0.0	0.0	0.0	0.0
0.0	0.0	0.0	0.0	0.0	0.0	0.0	0.0
0.0	0.0	0.0	0.0	0.0	0.0	0.0	0.0
0.0	0.0	0.0	0.0	0.0	0.0	0.0	0.0
0.0	0.0	0.0	0.0	0.0	0.0	0.0	0.0
0.0	0.0	0.0	0.0	0.0	0.0	0.0	0.0
0.0	0.0	0.0	0.0	0.0	0.0	0.0	0.0
0.0	0.0	0.0	0.0	0.0	0.0	0.0	0.0
0.0	0.0	0.0	0.0	0.0	0.0	0.0	0.0
0.0	0.0	0.0	0.0	0.0	0.0	0.0	0.0
0.0	0.0	0.0	0.0	0.0	0.0	0.0	0.0
0.0	0.0	0.0	0.0	0.0	0.0	0.0	0.0
0.0	0.0	0.0	0.0	0.0	0.0	0.0	0.0
0.0	0.0	0.0	0.0	0.0	6.0	0.0	0.0
0.0	0.0	0.0	0.0	0.0	0.0	0.0	0.0
0.0	0.0	0.0	0.0	0.0	0.0	0.0	0.0
0.0	0.0	0.0	0.0	0.0	0.0	0.0	0.0
0.0	0.0	0.0	0.0	0.0	0.0	0.0	0.0
0.0	0.0	0.0	0.0	0.0	0.0	0.0	0.0
0.0	0.0	0.0	0.0	0.0	0.0	0.0	0.0
0.0	0.0	0.0	0.0	0.0	0.0	0.0	0.0
0.0	0.0	0.0	0.0	0.0	0.0	0.0	0.0
0.0	0.0	0.0	0.0	0.0	0.0	0.0	0.0
0.0	0.0	0.0	0.0	0.0	0.0	0.0	0.0
0.0	0.0	0.0	0.0	0.0	0.0	0.0	0.0
0.0	0.0	0.0	0.0	0.0	0.0	0.0	0.0
0.0	0.0	0.0	0.0	0.0	0.0	0.0	0.0
0.0	0.0	0.0	0.0	0.0	0.0	0.0	0.0
0.0	0.0	0.0	0.0	0.0	0.0	0.0	0.0
0.0	0.0	0.0	0.0	0.0	0.0	0.0	0.0
0.0	0.0	0.0	0.0	0.0	0.0	0.0	0.0
0.0	0.0	0.0	0.0	0.0	0.0	0.0	0.0
0.0	0.0	0.0	0.0	0.0	0.0	0.0	0.0
0.0	0.0	0.0	0.0	0.0	0.0	0.0	0.0
0.0	0.0	0.0	0.0	0.0	0.0	0.0	0.0
0.0	0.0	0.0	0.0	0.0	0.0	0.0	0.0
0.0	0.0	0.0	0.0	0.0	0.0	0.0	0.0
0.0	3.0	0.0	0.0	0.0	0.0	0.0	0.0

0.0	0.0	0.0	0.0	0.0	0.0	0.0	0.0
0.0	0.0	0.0	0.0	0.0	0.0	0.0	0.0
0.0	0.0	0.0	0.0	0.0	0.0	0.0	0.0
0.0	0.0	0.0	0.0	0.0	0.0	0.0	0.0
0.0	0.0	0.0	0.0	0.0	0.0	0.0	0.0
0.0	0.0	0.0	0.0	0.0	0.0	0.0	0.0
0.0	0.0	0.0	0.0	0.0	0.0	0.0	0.0
0.0	0.0	0.0	0.0	0.0	0.0	0.0	0.0
0.0	0.0	0.0	0.0	0.0	0.0	0.0	0.0
0.0	0.0	0.0	0.0	0.0	0.0	0.0	0.0
0.0	0.0	0.0	0.0	0.0	0.0	0.0	0.0
0.0	0.0	0.0	0.0	0.0	0.0	0.0	0.0
0.0	0.0	0.0	0.0	0.0	0.0	0.0	0.0
0.0	0.0	0.0	0.0	0.0	0.0	0.0	0.0
0.0	0.0	2.0	0.0	0.0	0.0	0.0	0.0
0.0	0.0	0.0	0.0	0.0	0.0	0.0	0.0
0.0	0.0	0.0	0.0	0.0	0.0	0.0	0.0
0.0	0.0	0.0	0.0	0.0	0.0	0.0	0.0
0.0	0.0	0.0	0.0	0.0	0.0	0.0	0.0
0.0	0.0	0.0	0.0	0.0	0.0	0.0	0.0
0.0	0.0	0.0	0.0	0.0	0.0	0.0	0.0
0.0	0.0	0.0	0.0	0.0	0.0	0.0	0.0
0.0	0.0	0.0	0.0	0.0	0.0	0.0	0.0
0.0	0.0	0.0	0.0	0.0	0.0	0.0	0.0
0.0	0.0	0.0	0.0	0.0	0.0	0.0	0.0
0.0	0.0	0.0	0.0	0.0	0.0	1.0	0.0
0.0	0.0	0.0	0.0	0.0	0.0	0.0	0.0
0.0	0.0	0.0	0.0	0.0	0.0	0.0	0.0
0.0	0.0	0.0	0.0	0.0	0.0	0.0	0.0
0.0	0.0	0.0	0.0	0.0	0.0	0.0	0.0
0.0	0.0	0.0	0.0	0.0	0.0	0.0	1.0
0.0	0.0	0.0	0.0	0.0	0.0	0.0	0.0
0.0	3.0	0.0	0.0	0.0	0.0	0.0	0.0
0.0	0.0	0.0	0.0	0.0	0.0	0.0	0.0
0.0	0.0	0.0	0.0	0.0	0.0	0.0	0.0
0.0	0.0	0.0	0.0	0.0	0.0	0.0	0.0
0.0	0.0	0.0	0.0	0.0	0.0	0.0	0.0
0.0	0.0	0.0	0.0	0.0	0.0	0.0	0.0
0.0	0.0	0.0	0.0	0.0	0.0	0.0	0.0
0.0	0.0	0.0	0.0	0.0	0.0	0.0	0.0
0.0	0.0	0.0	0.0	0.0	0.0	0.0	0.0
0.0	0.0	1.0	0.0	0.0	0.0	0.0	0.0
0.0	0.0	0.0	0.0	0.0	0.0	0.0	0.0
0.0	0.0	0.0	0.0	0.0	0.0	0.0	0.0
0.0	0.0	0.0	0.0	0.0	0.0	0.0	0.0
0.0	0.0	0.0	0.0	0.0	0.0	0.0	0.0
0.0	0.0	0.0	0.0	0.0	0.0	0.0	0.0

0.0	0.0	0.0	0.0	0.0	0.0	0.0	0.0
0.0	0.0	0.0	0.0	0.0	1.0	0.0	0.0
0.0	0.0	2.0	0.0	0.0	0.0	0.0	0.0
0.0	0.0	0.0	0.0	0.0	0.0	0.0	0.0
0.0	0.0	0.0	0.0	0.0	0.0	0.0	0.0
0.0	0.0	0.0	0.0	0.0	0.0	0.0	0.0
0.0	0.0	0.0	0.0	0.0	0.0	0.0	0.0
0.0	0.0	0.0	0.0	0.0	0.0	0.0	0.0
0.0	0.0	0.0	0.0	0.0	0.0	0.0	0.0
0.0	0.0	0.0	0.0	0.0	0.0	0.0	0.0
0.0	0.0	0.0	0.0	0.0	0.0	1.0	0.0
0.0	0.0	0.0	0.0	0.0	0.0	0.0	0.0
0.0	0.0	0.0	0.0	0.0	0.0	0.0	0.0
0.0	0.0	0.0	0.0	0.0	0.0	0.0	0.0
0.0	0.0	0.0	0.0	0.0	0.0	0.0	0.0
0.0	0.0	0.0	0.0	0.0	0.0	0.0	0.0
0.0	0.0	0.0	0.0	0.0	0.0	0.0	0.0
0.0	0.0	0.0	0.0	0.0	0.0	0.0	0.0
0.0	0.0	0.0	0.0	0.0	0.0	0.0	0.0
0.0	0.0	0.0	0.0	0.0	0.0	0.0	0.0
0.0	0.0	0.0	0.0	0.0	0.0	0.0	0.0
0.0	0.0	0.0	0.0	0.0	0.0	0.0	0.0
0.0	0.0	0.0	0.0	0.0	0.0	0.0	0.0
0.0	0.0	0.0	0.0	0.0	0.0	0.0	0.0
0.0	0.0	0.0	0.0	0.0	0.0	0.0	0.0
0.0	1.0	0.0	0.0	0.0	0.0	0.0	0.0
0.0	0.0	0.0	0.0	0.0	0.0	0.0	0.0
0.0	0.0	0.0	0.0	0.0	0.0	0.0	0.0
0.0	0.0	0.0	0.0	0.0	0.0	0.0	0.0
0.0	0.0	0.0	0.0	0.0	0.0	0.0	0.0
0.0	0.0	0.0	0.0	0.0	0.0	0.0	8.0
0.0	0.0	0.0	0.0	0.0	0.0	0.0	0.0
0.0	0.0	0.0	0.0	0.0	0.0	0.0	0.0
0.0	0.0	0.0	0.0	0.0	0.0	0.0	0.0
0.0	0.0	0.0	0.0	0.0	0.0	0.0	0.0
0.0	0.0	0.0	0.0	0.0	0.0	5.0	0.0
0.0	0.0	0.0	0.0	0.0	0.0	0.0	0.0
0.0	0.0	0.0	0.0	0.0	0.0	0.0	0.0
0.0	0.0	0.0	0.0	0.0	0.0	0.0	0.0
0.0	0.0	0.0	0.0	0.0	0.0	1.0	0.0
1.0	0.0	0.0	0.0	0.0	0.0	0.0	0.0
0.0	0.0	0.0	0.0	0.0	0.0	0.0	0.0
0.0	0.0	0.0	0.0	0.0	0.0	0.0	0.0
0.0	0.0	0.0	0.0	0.0	0.0	0.0	0.0
0.0	0.0	0.0	0.0	0.0	0.0	0.0	0.0
0.0	0.0	0.0	0.0	0.0	0.0	0.0	0.0
0.0	0.0	0.0	0.0	0.0	0.0	0.0	0.0

0.0	0.0	0.0	0.0	0.0	0.0	0.0	0.0
0.0	0.0	0.0	0.0	0.0	0.0	0.0	0.0
0.0	0.0	0.0	0.0	0.0	2.0	0.0	0.0
0.0	0.0	0.0	0.0	0.0	0.0	0.0	0.0
0.0	0.0	0.0	0.0	0.0	0.0	0.0	0.0
0.0	0.0	0.0	0.0	0.0	0.0	0.0	0.0
0.0	0.0	0.0	0.0	0.0	0.0	0.0	0.0
0.0	0.0	0.0	0.0	0.0	0.0	0.0	0.0
0.0	0.0	0.0	0.0	0.0	0.0	0.0	0.0
0.0	0.0	0.0	0.0	0.0	0.0	0.0	0.0
0.0	0.0	0.0	0.0	0.0	0.0	0.0	0.0
0.0	0.0	0.0	0.0	0.0	0.0	0.0	0.0
0.0	0.0	0.0	0.0	0.0	0.0	0.0	0.0
0.0	0.0	0.0	0.0	0.0	0.0	0.0	0.0
0.0	0.0	0.0	0.0	0.0	0.0	0.0	0.0
0.0	0.0	0.0	0.0	0.0	0.0	0.0	0.0
0.0	0.0	0.0	0.0	0.0	0.0	0.0	0.0
0.0	0.0	0.0	0.0	0.0	0.0	0.0	0.0
0.0	0.0	0.0	0.0	0.0	0.0	0.0	0.0
0.0	0.0	0.0	0.0	0.0	0.0	0.0	0.0
0.0	0.0	0.0	0.0	0.0	0.0	0.0	0.0
0.0	0.0	0.0	0.0	0.0	0.0	0.0	0.0
0.0	0.0	0.0	4.0	0.0	0.0	0.0	0.0
0.0	0.0	0.0	0.0	0.0	0.0	0.0	0.0
0.0	0.0	0.0	0.0	0.0	0.0	0.0	0.0
0.0	0.0	0.0	0.0	0.0	0.0	0.0	0.0
0.0	0.0	0.0	0.0	0.0	0.0	0.0	0.0
0.0	0.0	0.0	0.0	0.0	0.0	0.0	0.0
0.0	0.0	0.0	0.0	0.0	0.0	0.0	0.0
0.0	0.0	0.0	0.0	0.0	0.0	0.0	0.0
0.0	0.0	0.0	0.0	0.0	0.0	0.0	0.0
0.0	0.0	0.0	0.0	0.0	0.0	0.0	1.0
0.0	0.0	0.0	0.0	0.0	0.0	0.0	0.0
0.0	0.0	0.0	0.0	0.0	0.0	0.0	0.0
0.0	0.0	0.0	0.0	0.0	0.0	0.0	0.0
0.0	0.0	0.0	0.0	0.0	0.0	0.0	0.0
0.0	0.0	0.0	0.0	0.0	0.0	0.0	0.0
0.0	0.0	0.0	0.0	0.0	0.0	0.0	0.0
0.0	0.0	0.0	0.0	0.0	0.0	0.0	0.0
0.0	0.0	0.0	0.0	0.0	0.0	0.0	0.0
0.0	0.0	0.0	0.0	0.0	0.0	0.0	0.0
0.0	0.0	0.0	0.0	0.0	0.0	0.0	0.0
0.0	0.0	0.0	0.0	0.0	0.0	0.0	0.0
0.0	0.0	0.0	0.0	0.0	0.0	0.0	0.0
0.0	0.0	0.0	0.0	0.0	0.0	0.0	0.0
0.0	0.0	0.0	0.0	0.0	0.0	0.0	0.0
0.0	0.0	0.0	0.0	0.0	0.0	0.0	0.0

0.0	0.0	0.0	0.0	0.0	0.0	0.0	0.0
0.0	0.0	0.0	0.0	0.0	0.0	0.0	0.0
0.0	0.0	0.0	0.0	0.0	0.0	0.0	0.0
0.0	0.0	0.0	0.0	0.0	0.0	0.0	0.0
0.0	0.0	0.0	0.0	0.0	0.0	0.0	0.0
0.0	0.0	0.0	0.0	0.0	0.0	0.0	0.0
0.0	0.0	0.0	0.0	0.0	0.0	0.0	0.0
0.0	0.0	0.0	0.0	0.0	0.0	0.0	0.0
0.0	0.0	0.0	0.0	0.0	0.0	0.0	0.0
0.0	0.0	0.0	0.0	0.0	0.0	0.0	0.0
0.0	0.0	0.0	0.0	0.0	0.0	0.0	0.0
0.0	0.0	0.0	0.0	0.0	0.0	0.0	0.0
0.0	0.0	0.0	0.0	0.0	0.0	0.0	0.0
0.0	0.0	0.0	0.0	0.0	0.0	0.0	0.0
269.0	0.0	0.0	0.0	0.0	0.0	0.0	0.0
60.0	0.0	0.0	0.0	0.0	0.0	0.0	0.0
44.0	0.0	0.0	0.0	0.0	0.0	0.0	0.0
14.0	0.0	0.0	0.0	0.0	0.0	0.0	0.0
18.0	0.0	0.0	2769.0	0.0	0.0	0.0	0.0
17.0	0.0	0.0	0.0	0.0	0.0	0.0	0.0
12.0	0.0	0.0	0.0	0.0	0.0	0.0	0.0
7.0	0.0	0.0	0.0	0.0	0.0	0.0	0.0
13.0	0.0	0.0	0.0	0.0	0.0	0.0	0.0
6.0	0.0	0.0	0.0	0.0	0.0	0.0	0.0
8.0	0.0	0.0	0.0	0.0	0.0	0.0	0.0
3.0	0.0	0.0	0.0	0.0	0.0	0.0	0.0
5.0	0.0	0.0	0.0	0.0	6.0	0.0	0.0
3.0	0.0	0.0	0.0	0.0	0.0	0.0	0.0
3.0	0.0	0.0	0.0	0.0	0.0	0.0	0.0
2.0	2.0	0.0	0.0	0.0	0.0	0.0	0.0
4.0	0.0	0.0	0.0	0.0	0.0	0.0	0.0
3.0	0.0	0.0	0.0	0.0	0.0	0.0	0.0
3.0	0.0	0.0	0.0	0.0	0.0	0.0	0.0
3.0	0.0	0.0	0.0	0.0	0.0	0.0	0.0
1.0	0.0	0.0	0.0	0.0	0.0	0.0	0.0
2.0	0.0	0.0	0.0	0.0	0.0	0.0	0.0
1.0	0.0	0.0	0.0	0.0	3.0	0.0	0.0
2.0	0.0	0.0	0.0	0.0	0.0	0.0	0.0
1.0	0.0	0.0	0.0	0.0	0.0	0.0	0.0
1.0	0.0	0.0	0.0	0.0	0.0	0.0	0.0
0.0	0.0	0.0	0.0	0.0	0.0	0.0	0.0
1.0	0.0	0.0	0.0	0.0	0.0	0.0	0.0
0.0	0.0	0.0	0.0	0.0	0.0	0.0	0.0
1.0	0.0	0.0	0.0	0.0	0.0	6.0	0.0
0.0	0.0	0.0	0.0	0.0	0.0	0.0	0.0
0.0	0.0	0.0	0.0	0.0	0.0	0.0	0.0
1.0	0.0	0.0	0.0	0.0	0.0	0.0	0.0

1.0	0.0	0.0	0.0	0.0	0.0	0.0	0.0
0.0	0.0	0.0	0.0	0.0	0.0	0.0	0.0
2.0	0.0	0.0	0.0	0.0	0.0	0.0	0.0
1.0	0.0	0.0	0.0	0.0	0.0	0.0	0.0
1.0	0.0	0.0	0.0	0.0	0.0	19.0	0.0
0.0	0.0	0.0	0.0	0.0	0.0	0.0	0.0
2.0	0.0	0.0	0.0	0.0	0.0	0.0	0.0
0.0	0.0	0.0	0.0	0.0	0.0	0.0	0.0
0.0	6.0	0.0	0.0	0.0	0.0	0.0	0.0
1.0	0.0	0.0	0.0	0.0	0.0	0.0	0.0
0.0	1.0	0.0	0.0	0.0	0.0	0.0	0.0
2.0	0.0	0.0	0.0	0.0	0.0	0.0	0.0
1.0	0.0	0.0	0.0	0.0	0.0	0.0	0.0
1.0	0.0	0.0	0.0	0.0	0.0	0.0	0.0
0.0	1.0	0.0	0.0	0.0	0.0	0.0	0.0
1.0	0.0	0.0	0.0	0.0	0.0	0.0	0.0
1.0	0.0	0.0	0.0	0.0	0.0	0.0	0.0
1.0	0.0	0.0	0.0	0.0	0.0	0.0	0.0
0.0	0.0	0.0	0.0	0.0	0.0	0.0	0.0
2.0	0.0	0.0	0.0	0.0	0.0	0.0	0.0
2.0	0.0	0.0	0.0	0.0	0.0	0.0	0.0
1.0	0.0	5.0	0.0	0.0	0.0	0.0	0.0
0.0	0.0	11.0	0.0	0.0	0.0	6.0	0.0
2.0	0.0	0.0	0.0	0.0	0.0	0.0	0.0
1.0	0.0	0.0	0.0	0.0	0.0	0.0	0.0
1.0	0.0	0.0	0.0	0.0	0.0	0.0	0.0
2.0	0.0	0.0	0.0	0.0	0.0	0.0	0.0
2.0	0.0	0.0	0.0	0.0	0.0	2.0	0.0
1.0	0.0	0.0	0.0	0.0	0.0	0.0	0.0
1.0	0.0	0.0	0.0	0.0	0.0	0.0	0.0
1.0	0.0	0.0	0.0	0.0	0.0	0.0	0.0
1.0	0.0	0.0	0.0	0.0	0.0	0.0	0.0
1.0	0.0	0.0	0.0	0.0	0.0	0.0	0.0
1.0	0.0	0.0	0.0	0.0	0.0	0.0	0.0
1.0	0.0	0.0	0.0	0.0	0.0	0.0	0.0
1.0	0.0	0.0	0.0	0.0	0.0	0.0	0.0
0.0	92.0	0.0	0.0	0.0	0.0	0.0	0.0
0.0	56.0	0.0	0.0	0.0	0.0	0.0	0.0
0.0	39.0	0.0	0.0	0.0	0.0	0.0	0.0
0.0	44.0	0.0	0.0	0.0	0.0	15.0	0.0
0.0	12.0	0.0	0.0	0.0	0.0	0.0	0.0
0.0	8.0	0.0	0.0	0.0	0.0	0.0	0.0
0.0	7.0	0.0	0.0	0.0	0.0	0.0	0.0
0.0	6.0	0.0	0.0	0.0	0.0	0.0	0.0
0.0	4.0	0.0	0.0	0.0	0.0	2.0	0.0
0.0	6.0	0.0	0.0	0.0	0.0	0.0	0.0
0.0	5.0	0.0	0.0	0.0	0.0	0.0	0.0

0.0	8.0	0.0	0.0	0.0	0.0	0.0	0.0
0.0	6.0	0.0	0.0	0.0	0.0	0.0	0.0
0.0	3.0	0.0	0.0	0.0	0.0	0.0	0.0
0.0	4.0	0.0	0.0	0.0	0.0	0.0	0.0
0.0	2.0	0.0	0.0	0.0	0.0	0.0	0.0
0.0	2.0	0.0	0.0	0.0	0.0	0.0	0.0
0.0	1.0	0.0	0.0	0.0	0.0	0.0	0.0
0.0	2.0	0.0	0.0	0.0	0.0	0.0	0.0
0.0	2.0	0.0	0.0	0.0	0.0	0.0	0.0
0.0	3.0	0.0	0.0	0.0	0.0	0.0	0.0
0.0	2.0	0.0	0.0	0.0	0.0	0.0	0.0
0.0	1.0	0.0	0.0	0.0	0.0	0.0	0.0
0.0	0.0	0.0	0.0	0.0	0.0	0.0	0.0
0.0	3.0	0.0	0.0	0.0	0.0	0.0	0.0
0.0	0.0	0.0	0.0	0.0	0.0	0.0	0.0
0.0	0.0	0.0	0.0	0.0	0.0	0.0	0.0
0.0	1.0	0.0	0.0	0.0	0.0	3.0	0.0
0.0	0.0	0.0	0.0	0.0	0.0	0.0	0.0
0.0	3.0	0.0	0.0	0.0	0.0	0.0	0.0
0.0	1.0	0.0	0.0	0.0	0.0	0.0	0.0
0.0	1.0	0.0	0.0	0.0	0.0	0.0	0.0
0.0	2.0	0.0	0.0	0.0	0.0	0.0	0.0
0.0	2.0	0.0	0.0	0.0	0.0	0.0	0.0
0.0	2.0	0.0	0.0	0.0	0.0	0.0	0.0
0.0	1.0	0.0	0.0	0.0	0.0	0.0	0.0
0.0	1.0	0.0	0.0	0.0	0.0	0.0	0.0
0.0	0.0	0.0	0.0	0.0	0.0	0.0	0.0
0.0	2.0	0.0	0.0	0.0	0.0	0.0	0.0
0.0	1.0	0.0	0.0	0.0	1.0	0.0	0.0
0.0	0.0	0.0	0.0	0.0	0.0	0.0	0.0
0.0	1.0	0.0	0.0	0.0	0.0	0.0	0.0
0.0	1.0	0.0	0.0	0.0	0.0	0.0	0.0
0.0	0.0	0.0	0.0	0.0	0.0	0.0	0.0
0.0	1.0	0.0	0.0	0.0	0.0	0.0	0.0
0.0	1.0	0.0	0.0	0.0	0.0	0.0	0.0
0.0	2.0	0.0	0.0	0.0	0.0	0.0	0.0
0.0	0.0	0.0	0.0	0.0	0.0	0.0	0.0
0.0	1.0	0.0	0.0	0.0	0.0	0.0	0.0
0.0	1.0	0.0	0.0	0.0	0.0	0.0	0.0
0.0	2.0	0.0	0.0	0.0	0.0	0.0	0.0
0.0	1.0	0.0	0.0	0.0	0.0	0.0	0.0
0.0	0.0	0.0	0.0	0.0	0.0	0.0	0.0
0.0	0.0	0.0	0.0	0.0	0.0	0.0	0.0
0.0	0.0	0.0	0.0	0.0	0.0	0.0	0.0
0.0	1.0	0.0	0.0	0.0	0.0	0.0	0.0
0.0	1.0	0.0	0.0	0.0	0.0	0.0	0.0
0.0	1.0	0.0	0.0	0.0	0.0	0.0	0.0

0.0	1.0	0.0	0.0	0.0	0.0	0.0	0.0
0.0	0.0	0.0	0.0	0.0	0.0	0.0	0.0
0.0	0.0	0.0	0.0	0.0	0.0	0.0	0.0
0.0	1.0	0.0	0.0	0.0	0.0	0.0	0.0
0.0	1.0	0.0	0.0	0.0	0.0	0.0	0.0
0.0	0.0	0.0	0.0	0.0	0.0	0.0	0.0
0.0	1.0	0.0	0.0	0.0	0.0	0.0	0.0
0.0	1.0	0.0	0.0	0.0	0.0	0.0	0.0
0.0	0.0	189.0	0.0	0.0	0.0	0.0	0.0
0.0	0.0	158.0	0.0	0.0	0.0	0.0	0.0
0.0	0.0	127.0	0.0	0.0	0.0	0.0	0.0
0.0	0.0	104.0	0.0	0.0	0.0	0.0	0.0
0.0	0.0	86.0	0.0	0.0	0.0	0.0	0.0
0.0	0.0	84.0	0.0	0.0	0.0	0.0	0.0
0.0	0.0	78.0	0.0	0.0	0.0	0.0	0.0
0.0	0.0	54.0	0.0	0.0	0.0	0.0	0.0
0.0	0.0	24.0	0.0	0.0	0.0	116.0	0.0
0.0	0.0	20.0	0.0	0.0	0.0	0.0	0.0
0.0	0.0	11.0	0.0	0.0	0.0	0.0	0.0
0.0	0.0	16.0	0.0	0.0	0.0	0.0	0.0
0.0	0.0	13.0	0.0	0.0	0.0	0.0	0.0
0.0	0.0	7.0	0.0	0.0	0.0	0.0	0.0
0.0	0.0	5.0	0.0	0.0	0.0	0.0	0.0
0.0	0.0	5.0	0.0	0.0	0.0	0.0	0.0
0.0	0.0	4.0	0.0	0.0	0.0	0.0	0.0
0.0	0.0	2.0	0.0	0.0	0.0	0.0	0.0
0.0	0.0	3.0	0.0	0.0	0.0	0.0	0.0
0.0	0.0	4.0	0.0	0.0	0.0	0.0	0.0
0.0	0.0	1.0	0.0	0.0	0.0	0.0	0.0
0.0	0.0	1.0	0.0	0.0	0.0	0.0	0.0
0.0	0.0	1.0	0.0	0.0	0.0	0.0	0.0
0.0	0.0	1.0	0.0	0.0	0.0	0.0	0.0
0.0	0.0	3.0	0.0	0.0	0.0	0.0	0.0
0.0	0.0	1.0	0.0	0.0	0.0	0.0	0.0
0.0	0.0	0.0	0.0	0.0	0.0	0.0	0.0
0.0	0.0	2.0	0.0	0.0	0.0	0.0	0.0
0.0	0.0	1.0	0.0	0.0	0.0	0.0	0.0
0.0	0.0	2.0	0.0	0.0	0.0	0.0	0.0
0.0	0.0	2.0	0.0	0.0	0.0	0.0	0.0
0.0	0.0	1.0	0.0	0.0	0.0	0.0	0.0
0.0	0.0	1.0	0.0	0.0	0.0	0.0	0.0
0.0	0.0	3.0	0.0	0.0	0.0	0.0	0.0
0.0	0.0	2.0	0.0	0.0	0.0	0.0	0.0
0.0	0.0	1.0	0.0	0.0	0.0	0.0	0.0
0.0	0.0	1.0	0.0	0.0	0.0	0.0	0.0
0.0	0.0	1.0	0.0	0.0	0.0	0.0	0.0
0.0	0.0	1.0	0.0	0.0	0.0	0.0	0.0

0.0	0.0	2.0	0.0	0.0	0.0	3.0	0.0
0.0	0.0	1.0	0.0	0.0	0.0	0.0	0.0
0.0	0.0	1.0	0.0	0.0	0.0	0.0	0.0
0.0	0.0	2.0	0.0	0.0	0.0	0.0	0.0
0.0	0.0	2.0	0.0	0.0	0.0	0.0	0.0
0.0	0.0	1.0	0.0	0.0	0.0	0.0	0.0
0.0	0.0	1.0	0.0	0.0	0.0	0.0	0.0
0.0	0.0	1.0	0.0	0.0	0.0	0.0	0.0
0.0	0.0	1.0	0.0	0.0	0.0	0.0	0.0
0.0	0.0	0.0	0.0	0.0	0.0	0.0	0.0
0.0	0.0	1.0	0.0	0.0	0.0	0.0	0.0
0.0	0.0	2.0	0.0	1.0	0.0	0.0	0.0
0.0	0.0	1.0	0.0	0.0	0.0	0.0	0.0
0.0	0.0	1.0	0.0	0.0	0.0	0.0	0.0
0.0	0.0	1.0	0.0	0.0	0.0	0.0	0.0
0.0	0.0	1.0	0.0	0.0	0.0	0.0	0.0
0.0	0.0	1.0	0.0	0.0	0.0	0.0	0.0
0.0	0.0	1.0	0.0	0.0	0.0	0.0	0.0
0.0	0.0	1.0	0.0	0.0	0.0	0.0	0.0
0.0	0.0	1.0	0.0	0.0	0.0	0.0	0.0
0.0	0.0	2.0	0.0	0.0	0.0	0.0	0.0
0.0	0.0	1.0	0.0	0.0	0.0	0.0	0.0
0.0	0.0	1.0	0.0	0.0	0.0	0.0	0.0
0.0	0.0	1.0	0.0	0.0	0.0	0.0	0.0
0.0	0.0	1.0	0.0	0.0	0.0	0.0	0.0
0.0	0.0	1.0	0.0	0.0	0.0	0.0	0.0
0.0	0.0	1.0	0.0	0.0	0.0	0.0	0.0
0.0	0.0	1.0	0.0	0.0	0.0	0.0	0.0
0.0	0.0	1.0	0.0	0.0	0.0	0.0	0.0
0.0	0.0	1.0	0.0	0.0	0.0	0.0	0.0
0.0	0.0	1.0	0.0	0.0	0.0	0.0	0.0
0.0	0.0	1.0	0.0	0.0	0.0	0.0	0.0
0.0	0.0	1.0	0.0	0.0	0.0	0.0	0.0
0.0	0.0	1.0	0.0	0.0	0.0	0.0	0.0
0.0	0.0	1.0	0.0	0.0	0.0	0.0	0.0
0.0	0.0	1.0	0.0	0.0	0.0	0.0	0.0
0.0	0.0	1.0	0.0	0.0	0.0	0.0	0.0
0.0	0.0	0.0	0.0	0.0	0.0	0.0	0.0
0.0	0.0	1.0	0.0	0.0	0.0	0.0	0.0
0.0	0.0	1.0	0.0	0.0	0.0	0.0	0.0
0.0	0.0	1.0	0.0	0.0	0.0	0.0	0.0
0.0	0.0	1.0	0.0	0.0	0.0	5.0	0.0
0.0	0.0	1.0	0.0	0.0	0.0	0.0	0.0
0.0	0.0	0.0	764.0	0.0	0.0	0.0	0.0
0.0	0.0	0.0	264.0	0.0	0.0	0.0	0.0
0.0	0.0	0.0	58.0	0.0	0.0	0.0	0.0
0.0	0.0	0.0	2.0	0.0	0.0	0.0	0.0

0.0	0.0	0.0	4.0	0.0	0.0	0.0	0.0
0.0	0.0	0.0	1.0	0.0	0.0	0.0	0.0
0.0	0.0	0.0	2.0	0.0	0.0	0.0	0.0
0.0	0.0	0.0	1.0	0.0	0.0	0.0	0.0
0.0	0.0	0.0	0.0	4.0	0.0	0.0	0.0
0.0	0.0	0.0	0.0	3.0	0.0	0.0	0.0
0.0	0.0	0.0	0.0	4.0	0.0	0.0	0.0
0.0	0.0	0.0	0.0	2.0	0.0	0.0	0.0
0.0	0.0	0.0	0.0	1.0	0.0	0.0	0.0
0.0	0.0	0.0	0.0	2.0	0.0	0.0	0.0
0.0	0.0	0.0	0.0	3.0	0.0	0.0	0.0
0.0	0.0	0.0	0.0	3.0	0.0	0.0	0.0
0.0	0.0	0.0	0.0	1.0	0.0	0.0	0.0
0.0	0.0	0.0	0.0	2.0	0.0	0.0	0.0
0.0	0.0	0.0	0.0	1.0	0.0	0.0	0.0
0.0	0.0	0.0	0.0	2.0	0.0	0.0	0.0
0.0	0.0	0.0	0.0	2.0	0.0	0.0	0.0
0.0	0.0	0.0	0.0	1.0	0.0	0.0	0.0
0.0	0.0	0.0	0.0	1.0	0.0	0.0	0.0
0.0	0.0	0.0	0.0	0.0	0.0	0.0	0.0
0.0	0.0	0.0	0.0	2.0	0.0	0.0	0.0
0.0	0.0	0.0	0.0	1.0	0.0	0.0	0.0
0.0	0.0	0.0	0.0	1.0	0.0	0.0	0.0
0.0	0.0	0.0	0.0	1.0	0.0	0.0	0.0
0.0	0.0	0.0	0.0	0.0	0.0	0.0	0.0
0.0	0.0	0.0	0.0	1.0	0.0	0.0	0.0
0.0	0.0	0.0	0.0	1.0	0.0	0.0	0.0
0.0	0.0	0.0	0.0	1.0	0.0	0.0	0.0
0.0	0.0	0.0	0.0	2.0	0.0	0.0	0.0
0.0	0.0	0.0	0.0	1.0	0.0	0.0	0.0
0.0	0.0	0.0	0.0	1.0	0.0	0.0	0.0
0.0	0.0	0.0	0.0	0.0	0.0	0.0	0.0
0.0	0.0	0.0	0.0	1.0	0.0	0.0	0.0
0.0	0.0	0.0	0.0	1.0	0.0	0.0	0.0
0.0	0.0	0.0	0.0	0.0	0.0	0.0	0.0
0.0	0.0	0.0	0.0	2.0	0.0	0.0	0.0
0.0	0.0	0.0	0.0	2.0	0.0	0.0	0.0
0.0	0.0	0.0	0.0	0.0	0.0	1.0	0.0
0.0	0.0	0.0	0.0	1.0	0.0	0.0	0.0
0.0	0.0	0.0	0.0	1.0	0.0	0.0	0.0
0.0	0.0	0.0	0.0	2.0	0.0	0.0	0.0
0.0	0.0	0.0	0.0	1.0	0.0	0.0	0.0
0.0	0.0	0.0	0.0	1.0	0.0	0.0	0.0
0.0	0.0	0.0	0.0	1.0	0.0	0.0	0.0
0.0	0.0	0.0	0.0	0.0	0.0	0.0	0.0
0.0	0.0	0.0	0.0	1.0	0.0	0.0	0.0
0.0	0.0	0.0	0.0	1.0	0.0	0.0	0.0

0.0	0.0	0.0	0.0	1.0	0.0	0.0	0.0
0.0	0.0	0.0	0.0	1.0	0.0	0.0	0.0
0.0	0.0	0.0	0.0	1.0	0.0	0.0	0.0
0.0	0.0	0.0	0.0	1.0	0.0	0.0	0.0
0.0	0.0	0.0	0.0	1.0	0.0	0.0	0.0
0.0	0.0	0.0	0.0	1.0	0.0	0.0	0.0
0.0	0.0	0.0	0.0	0.0	0.0	0.0	0.0
0.0	0.0	0.0	0.0	1.0	0.0	0.0	0.0
0.0	0.0	0.0	0.0	0.0	9.0	0.0	0.0
0.0	0.0	0.0	0.0	0.0	8.0	0.0	0.0
0.0	0.0	0.0	0.0	0.0	3.0	0.0	0.0
0.0	0.0	0.0	0.0	0.0	1.0	0.0	0.0
0.0	0.0	0.0	0.0	0.0	1.0	0.0	0.0
0.0	0.0	0.0	0.0	0.0	1.0	0.0	0.0
0.0	0.0	0.0	0.0	0.0	1.0	0.0	0.0
0.0	0.0	0.0	0.0	0.0	0.0	0.0	0.0
0.0	0.0	0.0	0.0	0.0	1.0	0.0	0.0
0.0	0.0	0.0	0.0	0.0	0.0	0.0	0.0
0.0	0.0	0.0	0.0	0.0	1.0	0.0	0.0
0.0	0.0	0.0	0.0	0.0	0.0	0.0	0.0
0.0	0.0	0.0	0.0	0.0	2.0	0.0	0.0
0.0	0.0	0.0	0.0	0.0	1.0	0.0	0.0
0.0	0.0	0.0	0.0	0.0	0.0	0.0	0.0
0.0	0.0	0.0	0.0	0.0	1.0	0.0	0.0
0.0	0.0	0.0	0.0	0.0	1.0	0.0	0.0
0.0	0.0	0.0	0.0	0.0	1.0	0.0	0.0
0.0	0.0	0.0	0.0	0.0	0.0	0.0	0.0
0.0	0.0	0.0	0.0	0.0	1.0	0.0	0.0
0.0	0.0	0.0	0.0	0.0	0.0	0.0	0.0
0.0	0.0	0.0	0.0	0.0	1.0	0.0	0.0
0.0	0.0	0.0	0.0	0.0	0.0	251.0	0.0
0.0	0.0	0.0	0.0	0.0	0.0	155.0	0.0
0.0	0.0	0.0	0.0	0.0	0.0	128.0	0.0
0.0	0.0	0.0	0.0	0.0	0.0	73.0	0.0
0.0	0.0	0.0	0.0	0.0	0.0	62.0	0.0
0.0	0.0	0.0	0.0	0.0	0.0	60.0	0.0
0.0	0.0	0.0	0.0	0.0	0.0	43.0	0.0
0.0	0.0	0.0	0.0	0.0	0.0	41.0	0.0
0.0	0.0	0.0	0.0	0.0	0.0	31.0	0.0
0.0	0.0	0.0	0.0	0.0	0.0	22.0	0.0
0.0	0.0	0.0	0.0	0.0	0.0	29.0	0.0
0.0	0.0	0.0	0.0	0.0	0.0	16.0	0.0
0.0	0.0	0.0	0.0	0.0	0.0	23.0	0.0
0.0	0.0	0.0	0.0	0.0	0.0	18.0	0.0
0.0	0.0	0.0	0.0	0.0	0.0	13.0	0.0
0.0	0.0	0.0	0.0	0.0	0.0	17.0	0.0
0.0	0.0	0.0	0.0	0.0	0.0	14.0	0.0

0.0	0.0	0.0	0.0	0.0	0.0	18.0	0.0
0.0	0.0	0.0	0.0	0.0	0.0	14.0	0.0
0.0	0.0	0.0	0.0	0.0	0.0	7.0	0.0
0.0	0.0	0.0	0.0	0.0	0.0	7.0	0.0
0.0	0.0	0.0	0.0	0.0	0.0	4.0	0.0
0.0	0.0	0.0	0.0	0.0	0.0	4.0	0.0
0.0	0.0	0.0	0.0	0.0	0.0	6.0	0.0
0.0	0.0	0.0	0.0	0.0	0.0	8.0	0.0
0.0	0.0	0.0	0.0	0.0	0.0	3.0	0.0
0.0	0.0	0.0	0.0	0.0	0.0	4.0	0.0
0.0	0.0	0.0	0.0	0.0	0.0	2.0	0.0
0.0	0.0	0.0	0.0	0.0	0.0	5.0	0.0
0.0	0.0	0.0	0.0	0.0	0.0	6.0	0.0
0.0	0.0	0.0	0.0	0.0	0.0	3.0	0.0
0.0	0.0	0.0	0.0	0.0	0.0	4.0	0.0
0.0	0.0	0.0	0.0	0.0	0.0	3.0	0.0
0.0	0.0	0.0	0.0	0.0	0.0	1.0	0.0
0.0	0.0	0.0	0.0	0.0	0.0	1.0	0.0
0.0	0.0	0.0	0.0	0.0	0.0	1.0	0.0
0.0	0.0	0.0	0.0	0.0	0.0	2.0	0.0
0.0	0.0	0.0	0.0	0.0	0.0	2.0	0.0
0.0	0.0	0.0	0.0	0.0	0.0	2.0	0.0
0.0	0.0	0.0	0.0	0.0	0.0	2.0	0.0
0.0	0.0	0.0	0.0	0.0	0.0	3.0	0.0
0.0	0.0	0.0	0.0	0.0	0.0	2.0	0.0
0.0	0.0	0.0	0.0	0.0	0.0	2.0	0.0
0.0	0.0	0.0	0.0	0.0	0.0	1.0	0.0
0.0	0.0	0.0	0.0	0.0	0.0	2.0	0.0
0.0	0.0	0.0	0.0	0.0	0.0	1.0	0.0
0.0	0.0	0.0	0.0	0.0	0.0	1.0	0.0
0.0	0.0	0.0	0.0	0.0	0.0	0.0	0.0
0.0	0.0	0.0	0.0	0.0	0.0	2.0	0.0
0.0	0.0	0.0	0.0	0.0	0.0	3.0	0.0
0.0	0.0	0.0	0.0	0.0	0.0	1.0	0.0
0.0	0.0	0.0	0.0	0.0	0.0	1.0	0.0
0.0	0.0	0.0	0.0	0.0	0.0	1.0	0.0
0.0	0.0	0.0	0.0	0.0	0.0	2.0	0.0
0.0	0.0	0.0	0.0	0.0	0.0	1.0	0.0
0.0	0.0	0.0	0.0	0.0	0.0	1.0	0.0
0.0	0.0	0.0	0.0	0.0	0.0	2.0	0.0
0.0	0.0	0.0	0.0	0.0	0.0	1.0	0.0
0.0	0.0	0.0	0.0	0.0	0.0	0.0	0.0
0.0	0.0	0.0	0.0	0.0	0.0	1.0	0.0
0.0	0.0	0.0	0.0	0.0	0.0	1.0	0.0
0.0	0.0	0.0	0.0	0.0	0.0	1.0	0.0
0.0	0.0	0.0	0.0	0.0	0.0	1.0	0.0
0.0	0.0	0.0	0.0	0.0	0.0	1.0	0.0

0.0	0.0	0.0	0.0	0.0	0.0	0.0	0.0
0.0	0.0	0.0	0.0	0.0	0.0	2.0	0.0
0.0	0.0	0.0	0.0	0.0	0.0	1.0	0.0
0.0	0.0	0.0	0.0	0.0	0.0	1.0	0.0
0.0	0.0	0.0	0.0	0.0	0.0	2.0	0.0
0.0	0.0	0.0	0.0	0.0	0.0	1.0	0.0
0.0	0.0	0.0	0.0	0.0	0.0	1.0	0.0
0.0	0.0	0.0	0.0	0.0	0.0	2.0	0.0
0.0	0.0	0.0	0.0	0.0	0.0	2.0	0.0
0.0	0.0	0.0	0.0	0.0	0.0	1.0	0.0
0.0	0.0	0.0	0.0	0.0	0.0	2.0	0.0
0.0	0.0	0.0	0.0	0.0	0.0	2.0	0.0
0.0	0.0	0.0	0.0	0.0	0.0	1.0	0.0
0.0	0.0	0.0	0.0	0.0	0.0	1.0	0.0
0.0	0.0	0.0	0.0	0.0	0.0	1.0	0.0
0.0	0.0	0.0	0.0	0.0	0.0	2.0	0.0
0.0	0.0	0.0	0.0	0.0	0.0	1.0	0.0
0.0	0.0	0.0	0.0	0.0	0.0	1.0	0.0
0.0	0.0	0.0	0.0	0.0	0.0	1.0	0.0
0.0	0.0	0.0	0.0	0.0	0.0	0.0	0.0
0.0	0.0	0.0	0.0	0.0	0.0	2.0	0.0
0.0	0.0	0.0	0.0	0.0	0.0	1.0	0.0
0.0	0.0	0.0	0.0	0.0	0.0	0.0	0.0
0.0	0.0	0.0	0.0	0.0	0.0	1.0	0.0
0.0	0.0	0.0	0.0	0.0	0.0	1.0	0.0
0.0	0.0	0.0	0.0	0.0	0.0	1.0	0.0
0.0	0.0	0.0	0.0	0.0	0.0	1.0	0.0
0.0	0.0	0.0	0.0	0.0	0.0	1.0	0.0
0.0	0.0	0.0	0.0	0.0	0.0	1.0	0.0
0.0	0.0	0.0	0.0	0.0	0.0	0.0	0.0
0.0	0.0	0.0	0.0	0.0	0.0	1.0	0.0
0.0	0.0	0.0	0.0	0.0	0.0	1.0	0.0
0.0	0.0	0.0	0.0	0.0	0.0	1.0	0.0
0.0	0.0	0.0	0.0	0.0	0.0	1.0	0.0
0.0	0.0	0.0	0.0	0.0	0.0	0.0	0.0
0.0	0.0	0.0	0.0	0.0	0.0	1.0	0.0
0.0	0.0	0.0	0.0	0.0	0.0	1.0	0.0
0.0	0.0	0.0	0.0	0.0	0.0	0.0	0.0
0.0	0.0	0.0	0.0	0.0	0.0	0.0	1368.0
0.0	0.0	0.0	0.0	0.0	0.0	0.0	108.0
0.0	0.0	0.0	0.0	0.0	0.0	0.0	38.0
0.0	0.0	0.0	0.0	0.0	0.0	0.0	24.0
0.0	0.0	0.0	0.0	0.0	0.0	0.0	6.0
0.0	0.0	0.0	0.0	0.0	0.0	0.0	3.0
0.0	0.0	0.0	0.0	0.0	0.0	0.0	3.0
0.0	0.0	0.0	0.0	0.0	0.0	0.0	3.0
0.0	0.0	0.0	0.0	0.0	0.0	0.0	2.0

0.0	0.0	0.0	0.0	0.0	0.0	0.0	2.0
0.0	0.0	0.0	0.0	0.0	0.0	0.0	1.0
0.0	0.0	0.0	0.0	0.0	0.0	0.0	0.0
0.0	0.0	0.0	0.0	0.0	0.0	0.0	0.0
0.0	0.0	0.0	0.0	0.0	0.0	0.0	0.0
0.0	0.0	0.0	0.0	0.0	0.0	0.0	0.0
0.0	0.0	0.0	0.0	0.0	0.0	0.0	0.0
0.0	0.0	0.0	0.0	0.0	0.0	0.0	0.0
0.0	0.0	0.0	0.0	0.0	0.0	0.0	0.0
0.0	0.0	0.0	0.0	0.0	0.0	0.0	0.0
0.0	0.0	0.0	0.0	0.0	0.0	0.0	0.0
0.0	0.0	0.0	0.0	0.0	0.0	0.0	0.0
0.0	0.0	0.0	0.0	0.0	0.0	0.0	0.0
0.0	0.0	0.0	0.0	0.0	0.0	0.0	0.0
0.0	0.0	0.0	0.0	0.0	0.0	0.0	0.0
0.0	0.0	0.0	0.0	0.0	0.0	0.0	0.0
0.0	0.0	0.0	0.0	0.0	0.0	0.0	0.0
0.0	0.0	0.0	0.0	0.0	0.0	0.0	0.0
0.0	0.0	0.0	0.0	0.0	0.0	0.0	0.0
0.0	0.0	0.0	0.0	0.0	0.0	0.0	0.0
0.0	0.0	0.0	0.0	0.0	0.0	0.0	0.0
0.0	0.0	0.0	0.0	0.0	0.0	0.0	0.0
0.0	0.0	0.0	0.0	0.0	0.0	0.0	0.0
0.0	0.0	0.0	0.0	0.0	0.0	0.0	0.0
0.0	0.0	0.0	0.0	0.0	0.0	0.0	0.0
0.0	0.0	0.0	0.0	0.0	0.0	0.0	0.0
0.0	0.0	0.0	0.0	0.0	0.0	0.0	0.0
0.0	0.0	0.0	0.0	0.0	0.0	0.0	0.0
0.0	0.0	0.0	0.0	0.0	0.0	0.0	0.0
0.0	0.0	0.0	0.0	0.0	0.0	0.0	0.0
0.0	0.0	0.0	0.0	0.0	0.0	0.0	0.0
0.0	0.0	0.0	0.0	0.0	0.0	0.0	0.0
0.0	0.0	0.0	0.0	0.0	0.0	0.0	0.0
0.0	0.0	0.0	0.0	0.0	0.0	0.0	0.0
0.0	0.0	0.0	0.0	0.0	0.0	0.0	0.0
0.0	0.0	0.0	0.0	0.0	0.0	0.0	0.0
0.0	0.0	0.0	0.0	0.0	0.0	0.0	0.0
0.0	0.0	0.0	0.0	0.0	0.0	0.0	0.0
0.0	0.0	0.0	0.0	0.0	0.0	0.0	0.0
0.0	0.0	0.0	0.0	0.0	0.0	0.0	0.0
0.0	0.0	0.0	0.0	0.0	0.0	0.0	0.0
0.0	0.0	0.0	0.0	0.0	0.0	0.0	0.0
0.0	0.0	0.0	0.0	0.0	0.0	0.0	0.0
0.0	0.0	0.0	0.0	0.0	0.0	0.0	0.0
0.0	0.0	0.0	0.0	0.0	0.0	0.0	0.0
0.0	0.0	0.0	0.0	0.0	0.0	0.0	0.0
0.0	0.0	0.0	0.0	0.0	0.0	0.0	0.0

0.0	0.0	0.0	0.0	0.0	0.0	0.0	0.0
0.0	0.0	0.0	0.0	0.0	0.0	0.0	0.0
0.0	0.0	0.0	0.0	0.0	0.0	0.0	0.0
0.0	0.0	0.0	0.0	0.0	0.0	0.0	0.0
0.0	0.0	0.0	0.0	0.0	0.0	0.0	0.0
0.0	0.0	0.0	0.0	0.0	0.0	0.0	0.0
0.0	0.0	0.0	0.0	0.0	0.0	0.0	0.0
0.0	0.0	0.0	0.0	0.0	0.0	0.0	0.0
0.0	0.0	0.0	0.0	0.0	0.0	0.0	0.0
0.0	0.0	0.0	0.0	0.0	0.0	0.0	0.0
0.0	0.0	0.0	0.0	0.0	0.0	0.0	0.0
0.0	0.0	0.0	0.0	0.0	0.0	0.0	0.0
0.0	0.0	0.0	0.0	0.0	0.0	0.0	0.0
0.0	0.0	0.0	0.0	0.0	0.0	0.0	0.0
0.0	0.0	0.0	0.0	0.0	0.0	0.0	0.0
0.0	0.0	0.0	0.0	0.0	0.0	0.0	0.0
0.0	0.0	0.0	0.0	0.0	0.0	0.0	0.0
0.0	0.0	0.0	0.0	0.0	0.0	0.0	0.0
0.0	0.0	0.0	0.0	0.0	0.0	0.0	0.0
0.0	0.0	0.0	0.0	0.0	0.0	0.0	0.0
0.0	0.0	0.0	0.0	0.0	0.0	0.0	0.0
0.0	0.0	0.0	0.0	0.0	0.0	0.0	0.0
0.0	0.0	0.0	0.0	0.0	0.0	0.0	0.0
0.0	0.0	0.0	0.0	0.0	0.0	0.0	0.0
0.0	0.0	0.0	0.0	0.0	0.0	0.0	0.0
0.0	0.0	0.0	0.0	0.0	0.0	0.0	0.0
0.0	0.0	0.0	0.0	0.0	0.0	0.0	0.0
0.0	0.0	0.0	0.0	0.0	0.0	0.0	0.0
0.0	0.0	0.0	0.0	0.0	0.0	0.0	0.0
0.0	0.0	0.0	0.0	0.0	0.0	0.0	0.0
0.0	0.0	0.0	0.0	0.0	0.0	0.0	0.0
0.0	0.0	0.0	0.0	0.0	0.0	0.0	0.0
0.0	0.0	0.0	0.0	0.0	0.0	0.0	0.0
0.0	0.0	0.0	0.0	0.0	0.0	0.0	0.0
0.0	0.0	0.0	0.0	0.0	0.0	0.0	0.0
0.0	0.0	0.0	0.0	0.0	0.0	0.0	0.0
0.0	0.0	0.0	0.0	0.0	0.0	0.0	0.0
0.0	0.0	0.0	0.0	0.0	0.0	0.0	0.0
0.0	0.0	0.0	0.0	0.0	0.0	0.0	0.0
0.0	0.0	0.0	0.0	0.0	0.0	0.0	0.0
0.0	0.0	0.0	0.0	0.0	0.0	0.0	0.0
0.0	0.0	0.0	0.0	0.0	0.0	0.0	0.0
0.0	0.0	0.0	0.0	0.0	0.0	0.0	0.0
0.0	0.0	0.0	0.0	0.0	0.0	0.0	0.0
0.0	0.0	0.0	0.0	0.0	0.0	0.0	0.0
0.0	0.0	0.0	0.0	0.0	0.0	0.0	0.0
0.0	0.0	0.0	0.0	0.0	0.0	0.0	0.0
0.0	0.0	0.0	0.0	0.0	0.0	0.0	0.0

0.0	0.0	0.0	0.0	0.0	0.0	0.0	0.0
0.0	0.0	0.0	0.0	0.0	0.0	0.0	0.0
0.0	0.0	0.0	0.0	0.0	0.0	0.0	0.0
0.0	0.0	0.0	0.0	0.0	0.0	0.0	0.0
0.0	0.0	0.0	0.0	0.0	0.0	0.0	0.0
0.0	0.0	0.0	0.0	0.0	0.0	0.0	0.0
0.0	0.0	0.0	0.0	0.0	0.0	0.0	0.0
0.0	0.0	0.0	0.0	0.0	0.0	0.0	0.0
0.0	0.0	0.0	0.0	0.0	0.0	0.0	0.0
0.0	0.0	0.0	0.0	0.0	0.0	0.0	0.0
0.0	0.0	0.0	0.0	0.0	0.0	0.0	0.0
0.0	0.0	0.0	0.0	0.0	0.0	0.0	0.0
0.0	0.0	0.0	0.0	0.0	0.0	0.0	0.0
0.0	0.0	0.0	0.0	0.0	0.0	0.0	0.0
0.0	0.0	0.0	0.0	0.0	0.0	0.0	0.0
0.0	0.0	0.0	0.0	0.0	0.0	0.0	0.0
0.0	0.0	0.0	0.0	0.0	0.0	0.0	0.0
0.0	0.0	0.0	0.0	0.0	0.0	0.0	0.0
0.0	0.0	0.0	0.0	0.0	0.0	0.0	0.0
0.0	0.0	0.0	0.0	0.0	0.0	0.0	0.0
0.0	0.0	0.0	0.0	0.0	0.0	0.0	0.0
0.0	0.0	0.0	0.0	0.0	0.0	0.0	0.0
0.0	0.0	0.0	0.0	0.0	0.0	0.0	0.0
0.0	0.0	0.0	0.0	0.0	0.0	0.0	0.0
0.0	0.0	0.0	0.0	0.0	0.0	0.0	0.0
0.0	0.0	0.0	0.0	0.0	0.0	0.0	0.0
0.0	0.0	0.0	0.0	0.0	0.0	0.0	0.0
0.0	0.0	0.0	0.0	0.0	0.0	0.0	0.0
0.0	0.0	0.0	0.0	0.0	0.0	0.0	0.0
0.0	0.0	0.0	0.0	0.0	0.0	0.0	0.0
0.0	0.0	0.0	0.0	0.0	0.0	0.0	0.0
0.0	0.0	0.0	0.0	0.0	0.0	0.0	0.0
0.0	0.0	0.0	0.0	0.0	0.0	0.0	0.0
0.0	0.0	0.0	0.0	0.0	0.0	0.0	0.0
0.0	0.0	0.0	0.0	0.0	0.0	0.0	0.0
0.0	0.0	0.0	0.0	0.0	0.0	0.0	0.0
0.0	0.0	0.0	0.0	0.0	0.0	0.0	0.0
0.0	0.0	0.0	0.0	0.0	0.0	0.0	0.0
0.0	0.0	0.0	0.0	0.0	0.0	0.0	0.0
0.0	0.0	0.0	0.0	0.0	0.0	0.0	0.0
0.0	0.0	0.0	0.0	0.0	0.0	0.0	0.0
0.0	0.0	0.0	0.0	0.0	0.0	0.0	0.0
0.0	0.0	0.0	0.0	0.0	0.0	0.0	0.0
0.0	0.0	0.0	0.0	0.0	0.0	0.0	0.0
0.0	0.0	0.0	0.0	0.0	0.0	0.0	0.0
0.0	0.0	0.0	0.0	0.0	0.0	0.0	0.0
0.0	0.0	0.0	0.0	0.0	0.0	0.0	0.0

0.0	0.0	0.0	0.0	0.0	0.0	0.0	0.0
0.0	0.0	0.0	0.0	0.0	0.0	0.0	0.0
0.0	0.0	0.0	0.0	0.0	0.0	0.0	0.0
0.0	0.0	0.0	0.0	0.0	0.0	0.0	0.0
0.0	0.0	0.0	0.0	0.0	0.0	0.0	0.0
0.0	0.0	0.0	0.0	0.0	0.0	0.0	0.0
0.0	0.0	0.0	0.0	0.0	0.0	0.0	0.0
0.0	0.0	0.0	0.0	0.0	0.0	0.0	0.0
0.0	0.0	0.0	0.0	0.0	0.0	0.0	0.0
0.0	0.0	0.0	0.0	0.0	0.0	0.0	0.0
0.0	0.0	0.0	0.0	0.0	0.0	0.0	0.0
0.0	0.0	0.0	0.0	0.0	0.0	0.0	0.0
0.0	0.0	0.0	0.0	0.0	0.0	0.0	0.0
0.0	0.0	0.0	0.0	0.0	0.0	0.0	0.0
0.0	0.0	0.0	0.0	0.0	0.0	0.0	0.0
0.0	0.0	0.0	0.0	0.0	0.0	0.0	0.0
0.0	0.0	0.0	0.0	0.0	0.0	0.0	0.0
0.0	0.0	0.0	0.0	0.0	0.0	0.0	0.0
0.0	0.0	0.0	0.0	0.0	0.0	0.0	0.0
0.0	0.0	0.0	0.0	0.0	0.0	0.0	0.0
0.0	0.0	0.0	0.0	0.0	0.0	0.0	0.0
0.0	0.0	0.0	0.0	0.0	0.0	0.0	0.0
0.0	0.0	0.0	0.0	0.0	0.0	0.0	0.0
0.0	0.0	0.0	0.0	0.0	0.0	0.0	0.0
0.0	0.0	0.0	0.0	0.0	0.0	0.0	0.0
0.0	0.0	0.0	0.0	0.0	0.0	0.0	0.0
0.0	0.0	0.0	0.0	0.0	0.0	0.0	0.0
0.0	0.0	0.0	0.0	0.0	0.0	0.0	0.0
0.0	0.0	0.0	0.0	0.0	0.0	0.0	0.0
0.0	0.0	0.0	0.0	0.0	0.0	0.0	0.0
0.0	0.0	0.0	0.0	0.0	0.0	0.0	0.0
0.0	0.0	0.0	0.0	0.0	0.0	0.0	0.0
0.0	0.0	0.0	0.0	0.0	0.0	0.0	0.0
0.0	0.0	0.0	0.0	0.0	0.0	0.0	0.0
0.0	0.0	0.0	0.0	0.0	0.0	0.0	0.0
0.0	0.0	0.0	0.0	0.0	0.0	0.0	0.0
0.0	0.0	0.0	0.0	0.0	0.0	0.0	0.0
0.0	0.0	0.0	0.0	0.0	0.0	0.0	0.0
0.0	0.0	0.0	0.0	0.0	0.0	0.0	0.0
0.0	0.0	0.0	0.0	0.0	0.0	0.0	0.0
0.0	0.0	0.0	0.0	0.0	0.0	0.0	0.0
0.0	0.0	0.0	0.0	0.0	0.0	0.0	0.0
0.0	0.0	0.0	0.0	0.0	0.0	0.0	0.0
0.0	0.0	0.0	0.0	0.0	0.0	0.0	0.0
0.0	0.0	0.0	0.0	0.0	0.0	0.0	0.0
0.0	0.0	0.0	0.0	0.0	0.0	0.0	0.0
0.0	0.0	0.0	0.0	0.0	0.0	0.0	0.0

0.0	0.0	0.0	0.0	0.0	0.0	0.0	0.0
0.0	0.0	0.0	0.0	0.0	0.0	0.0	0.0
0.0	0.0	0.0	0.0	0.0	0.0	0.0	0.0
0.0	0.0	0.0	0.0	0.0	0.0	0.0	0.0
0.0	0.0	0.0	0.0	0.0	0.0	0.0	0.0
0.0	0.0	0.0	0.0	0.0	0.0	0.0	0.0
0.0	0.0	0.0	0.0	0.0	0.0	0.0	0.0
0.0	0.0	0.0	0.0	0.0	0.0	0.0	0.0
0.0	0.0	0.0	0.0	0.0	0.0	0.0	0.0
0.0	0.0	0.0	0.0	0.0	0.0	0.0	0.0
0.0	0.0	0.0	0.0	0.0	0.0	0.0	0.0
0.0	0.0	0.0	0.0	0.0	0.0	0.0	0.0
0.0	0.0	0.0	0.0	0.0	0.0	0.0	0.0
0.0	0.0	0.0	0.0	0.0	0.0	0.0	0.0
0.0	0.0	0.0	0.0	0.0	0.0	0.0	0.0
0.0	0.0	0.0	0.0	0.0	0.0	0.0	0.0
0.0	0.0	0.0	0.0	0.0	0.0	0.0	0.0
0.0	0.0	0.0	0.0	0.0	0.0	0.0	0.0
0.0	0.0	0.0	0.0	0.0	0.0	0.0	0.0
0.0	0.0	0.0	0.0	0.0	0.0	0.0	0.0
0.0	0.0	0.0	0.0	0.0	0.0	0.0	0.0
0.0	0.0	0.0	0.0	0.0	0.0	0.0	0.0
0.0	0.0	0.0	0.0	0.0	0.0	0.0	0.0
0.0	0.0	0.0	0.0	0.0	0.0	0.0	0.0
0.0	0.0	0.0	0.0	0.0	0.0	0.0	0.0
0.0	0.0	0.0	0.0	0.0	0.0	0.0	0.0
0.0	0.0	0.0	0.0	0.0	0.0	0.0	0.0
0.0	0.0	0.0	0.0	0.0	0.0	0.0	0.0
0.0	0.0	0.0	0.0	0.0	0.0	0.0	0.0
0.0	0.0	0.0	0.0	0.0	0.0	0.0	0.0
0.0	0.0	0.0	0.0	0.0	0.0	0.0	0.0
0.0	0.0	0.0	0.0	0.0	0.0	0.0	0.0
0.0	0.0	0.0	0.0	0.0	0.0	0.0	0.0
0.0	0.0	0.0	0.0	0.0	0.0	0.0	0.0
0.0	0.0	0.0	0.0	0.0	0.0	0.0	0.0
0.0	0.0	0.0	0.0	0.0	0.0	0.0	0.0
0.0	0.0	0.0	0.0	0.0	0.0	0.0	0.0
0.0	0.0	0.0	0.0	0.0	0.0	0.0	0.0
0.0	0.0	0.0	0.0	0.0	0.0	0.0	0.0
0.0	0.0	0.0	0.0	0.0	0.0	0.0	0.0
0.0	0.0	0.0	0.0	0.0	0.0	0.0	0.0
0.0	0.0	0.0	0.0	0.0	0.0	0.0	0.0
0.0	0.0	0.0	0.0	0.0	0.0	0.0	0.0
0.0	0.0	0.0	0.0	0.0	0.0	0.0	0.0
0.0	0.0	0.0	0.0	0.0	0.0	0.0	0.0
0.0	0.0	0.0	0.0	0.0	0.0	0.0	0.0
0.0	0.0	0.0	0.0	0.0	0.0	0.0	0.0
0.0	0.0	0.0	0.0	0.0	0.0	0.0	0.0

0.0	0.0	0.0	0.0	0.0	0.0	0.0	0.0
0.0	0.0	0.0	0.0	0.0	0.0	0.0	0.0
0.0	0.0	0.0	0.0	0.0	0.0	0.0	0.0
0.0	0.0	0.0	0.0	0.0	0.0	0.0	0.0
0.0	0.0	0.0	0.0	0.0	0.0	0.0	0.0
0.0	0.0	0.0	0.0	0.0	0.0	0.0	0.0
0.0	0.0	0.0	0.0	0.0	0.0	0.0	0.0
0.0	0.0	0.0	0.0	0.0	0.0	0.0	0.0
0.0	0.0	0.0	0.0	0.0	0.0	0.0	0.0
0.0	0.0	0.0	0.0	0.0	0.0	0.0	0.0
0.0	0.0	0.0	0.0	0.0	0.0	0.0	0.0
0.0	0.0	0.0	0.0	0.0	0.0	0.0	0.0
0.0	0.0	0.0	0.0	0.0	0.0	0.0	0.0
0.0	0.0	0.0	0.0	0.0	0.0	0.0	0.0
0.0	0.0	0.0	0.0	0.0	0.0	0.0	0.0
0.0	0.0	0.0	0.0	0.0	0.0	0.0	0.0
0.0	0.0	0.0	0.0	0.0	0.0	0.0	0.0
0.0	0.0	0.0	0.0	0.0	0.0	0.0	0.0
0.0	0.0	0.0	0.0	0.0	0.0	0.0	0.0
0.0	0.0	0.0	0.0	0.0	0.0	0.0	0.0
0.0	0.0	0.0	0.0	0.0	0.0	0.0	0.0
0.0	0.0	0.0	0.0	0.0	0.0	0.0	0.0
0.0	0.0	0.0	0.0	0.0	0.0	0.0	0.0
0.0	0.0	0.0	0.0	0.0	0.0	0.0	0.0
0.0	0.0	0.0	0.0	0.0	0.0	0.0	0.0
0.0	0.0	0.0	0.0	0.0	0.0	0.0	0.0
0.0	0.0	0.0	0.0	0.0	0.0	0.0	0.0
0.0	0.0	0.0	0.0	0.0	0.0	0.0	0.0
0.0	0.0	0.0	0.0	0.0	0.0	0.0	0.0
0.0	0.0	0.0	0.0	0.0	0.0	0.0	0.0
0.0	0.0	0.0	0.0	0.0	0.0	0.0	0.0
0.0	0.0	0.0	0.0	0.0	0.0	0.0	0.0
0.0	0.0	0.0	0.0	0.0	0.0	0.0	0.0
0.0	0.0	0.0	0.0	0.0	0.0	0.0	0.0
0.0	0.0	0.0	0.0	0.0	0.0	0.0	0.0
0.0	0.0	0.0	0.0	0.0	0.0	0.0	0.0
0.0	0.0	0.0	0.0	0.0	0.0	0.0	0.0
0.0	0.0	0.0	0.0	0.0	0.0	0.0	0.0
0.0	0.0	0.0	0.0	0.0	0.0	0.0	0.0
0.0	0.0	0.0	0.0	0.0	0.0	0.0	0.0
0.0	0.0	0.0	0.0	0.0	0.0	0.0	0.0
0.0	0.0	0.0	0.0	0.0	0.0	0.0	0.0
0.0	0.0	0.0	0.0	0.0	0.0	0.0	0.0
0.0	0.0	0.0	0.0	0.0	0.0	0.0	0.0
0.0	0.0	0.0	0.0	0.0	0.0	0.0	0.0
0.0	0.0	0.0	0.0	0.0	0.0	0.0	0.0
0.0	0.0	0.0	0.0	0.0	0.0	0.0	0.0
0.0	0.0	0.0	0.0	0.0	0.0	0.0	0.0

0.0	0.0	0.0	0.0	0.0	0.0	0.0	0.0
0.0	0.0	0.0	0.0	0.0	0.0	0.0	0.0
0.0	0.0	0.0	0.0	0.0	0.0	0.0	0.0
0.0	0.0	0.0	0.0	0.0	0.0	0.0	0.0
0.0	0.0	0.0	0.0	0.0	0.0	0.0	0.0
0.0	0.0	0.0	0.0	0.0	0.0	0.0	0.0
0.0	0.0	0.0	0.0	0.0	0.0	0.0	0.0
0.0	0.0	0.0	0.0	0.0	0.0	0.0	0.0
0.0	0.0	0.0	0.0	0.0	0.0	0.0	0.0
0.0	0.0	0.0	0.0	0.0	0.0	0.0	0.0
0.0	0.0	0.0	0.0	0.0	0.0	0.0	0.0
0.0	0.0	0.0	0.0	0.0	0.0	0.0	0.0
0.0	0.0	0.0	0.0	0.0	0.0	0.0	0.0
0.0	0.0	0.0	0.0	0.0	0.0	0.0	0.0
0.0	0.0	0.0	0.0	0.0	0.0	0.0	0.0
0.0	0.0	0.0	0.0	0.0	0.0	0.0	0.0
0.0	0.0	0.0	0.0	0.0	0.0	0.0	0.0
0.0	0.0	0.0	0.0	0.0	0.0	0.0	0.0
0.0	0.0	0.0	0.0	0.0	0.0	0.0	0.0
0.0	0.0	0.0	0.0	0.0	0.0	0.0	0.0
0.0	0.0	0.0	0.0	0.0	0.0	0.0	0.0
0.0	0.0	0.0	0.0	0.0	0.0	0.0	0.0
0.0	0.0	0.0	0.0	0.0	0.0	0.0	0.0
0.0	0.0	0.0	0.0	0.0	0.0	0.0	0.0
0.0	0.0	0.0	0.0	0.0	0.0	0.0	0.0
0.0	0.0	0.0	0.0	0.0	0.0	0.0	0.0
0.0	0.0	0.0	0.0	0.0	0.0	0.0	0.0
0.0	0.0	0.0	0.0	0.0	0.0	0.0	0.0
0.0	0.0	0.0	0.0	0.0	0.0	0.0	0.0
0.0	0.0	0.0	0.0	0.0	0.0	0.0	0.0
0.0	0.0	0.0	0.0	0.0	0.0	0.0	0.0
0.0	0.0	0.0	0.0	0.0	0.0	0.0	0.0
0.0	0.0	0.0	0.0	0.0	0.0	0.0	0.0
0.0	0.0	0.0	0.0	0.0	0.0	0.0	0.0
0.0	0.0	0.0	0.0	0.0	0.0	0.0	0.0
0.0	0.0	0.0	0.0	0.0	0.0	0.0	0.0
0.0	0.0	0.0	0.0	0.0	0.0	0.0	0.0
0.0	0.0	0.0	0.0	0.0	0.0	0.0	0.0
0.0	0.0	0.0	0.0	0.0	0.0	0.0	0.0
0.0	0.0	0.0	0.0	0.0	0.0	0.0	0.0
0.0	0.0	0.0	0.0	0.0	0.0	0.0	0.0
0.0	0.0	0.0	0.0	0.0	0.0	0.0	0.0
0.0	0.0	0.0	0.0	0.0	0.0	0.0	0.0
0.0	0.0	0.0	0.0	0.0	0.0	0.0	0.0
0.0	0.0	0.0	0.0	0.0	0.0	0.0	0.0
0.0	0.0	0.0	0.0	0.0	0.0	0.0	0.0
0.0	0.0	0.0	0.0	0.0	0.0	0.0	0.0

0.0	0.0	0.0	0.0	0.0	0.0	0.0	0.0
0.0	0.0	0.0	0.0	0.0	0.0	0.0	0.0
0.0	0.0	0.0	0.0	0.0	0.0	0.0	0.0
0.0	0.0	0.0	0.0	0.0	0.0	0.0	0.0
0.0	0.0	0.0	0.0	0.0	0.0	0.0	0.0
0.0	0.0	0.0	0.0	0.0	0.0	0.0	0.0
0.0	0.0	0.0	0.0	0.0	0.0	0.0	0.0
0.0	0.0	0.0	0.0	0.0	0.0	0.0	0.0
0.0	0.0	0.0	0.0	0.0	0.0	0.0	0.0
0.0	0.0	0.0	0.0	0.0	0.0	0.0	0.0
0.0	0.0	0.0	0.0	0.0	0.0	0.0	0.0
0.0	0.0	0.0	0.0	0.0	0.0	0.0	0.0
0.0	0.0	0.0	0.0	0.0	0.0	0.0	0.0
0.0	0.0	0.0	0.0	0.0	0.0	0.0	0.0
0.0	0.0	0.0	0.0	0.0	0.0	0.0	0.0
0.0	0.0	0.0	0.0	0.0	0.0	0.0	0.0
0.0	0.0	0.0	0.0	0.0	0.0	0.0	0.0
0.0	0.0	0.0	0.0	0.0	0.0	0.0	0.0
0.0	0.0	0.0	0.0	0.0	0.0	0.0	0.0
0.0	0.0	0.0	0.0	0.0	0.0	0.0	0.0
0.0	0.0	0.0	0.0	0.0	0.0	0.0	0.0
0.0	0.0	0.0	0.0	0.0	0.0	0.0	0.0
0.0	0.0	0.0	0.0	0.0	0.0	0.0	0.0
0.0	0.0	0.0	0.0	0.0	0.0	0.0	0.0
0.0	0.0	0.0	0.0	0.0	0.0	0.0	0.0
0.0	0.0	0.0	0.0	0.0	0.0	0.0	0.0
0.0	0.0	0.0	0.0	0.0	0.0	0.0	0.0
0.0	0.0	0.0	0.0	0.0	0.0	0.0	0.0
0.0	0.0	0.0	0.0	0.0	0.0	0.0	0.0
0.0	0.0	0.0	0.0	0.0	0.0	0.0	0.0
0.0	0.0	0.0	0.0	0.0	0.0	0.0	0.0
0.0	0.0	0.0	0.0	0.0	0.0	0.0	0.0
0.0	0.0	0.0	0.0	0.0	0.0	0.0	0.0
0.0	0.0	0.0	0.0	0.0	0.0	0.0	0.0
0.0	0.0	0.0	0.0	0.0	0.0	0.0	0.0
0.0	0.0	0.0	0.0	0.0	0.0	0.0	0.0
0.0	0.0	0.0	0.0	0.0	0.0	0.0	0.0
0.0	0.0	0.0	0.0	0.0	0.0	0.0	0.0
0.0	0.0	0.0	0.0	0.0	0.0	0.0	0.0
0.0	0.0	0.0	0.0	0.0	0.0	0.0	0.0
0.0	0.0	0.0	0.0	0.0	0.0	0.0	0.0
0.0	0.0	0.0	0.0	0.0	0.0	0.0	0.0
0.0	0.0	0.0	0.0	0.0	0.0	0.0	0.0
0.0	0.0	0.0	0.0	0.0	0.0	0.0	0.0
0.0	0.0	0.0	0.0	0.0	0.0	0.0	0.0
0.0	0.0	0.0	0.0	0.0	0.0	0.0	0.0
0.0	0.0	0.0	0.0	0.0	0.0	0.0	0.0
0.0	0.0	0.0	0.0	0.0	0.0	0.0	0.0

0.0	0.0	0.0	0.0	0.0	0.0	0.0	0.0
0.0	0.0	0.0	0.0	0.0	0.0	0.0	0.0
0.0	0.0	0.0	0.0	0.0	0.0	0.0	0.0
0.0	0.0	0.0	0.0	0.0	0.0	0.0	0.0
0.0	0.0	0.0	0.0	0.0	0.0	0.0	0.0
0.0	0.0	0.0	0.0	0.0	0.0	0.0	0.0
0.0	0.0	0.0	0.0	0.0	0.0	0.0	0.0
0.0	0.0	0.0	0.0	0.0	0.0	0.0	0.0
0.0	0.0	0.0	0.0	0.0	0.0	0.0	0.0
0.0	0.0	0.0	0.0	0.0	0.0	0.0	0.0
0.0	0.0	0.0	0.0	0.0	0.0	0.0	0.0
0.0	0.0	0.0	0.0	0.0	0.0	0.0	0.0
0.0	0.0	0.0	0.0	0.0	0.0	0.0	0.0
0.0	0.0	0.0	0.0	0.0	0.0	0.0	0.0
0.0	0.0	0.0	0.0	0.0	0.0	0.0	0.0
0.0	0.0	0.0	0.0	0.0	0.0	0.0	0.0
0.0	0.0	0.0	0.0	0.0	0.0	0.0	0.0
0.0	0.0	0.0	0.0	0.0	0.0	0.0	0.0
0.0	0.0	0.0	0.0	0.0	0.0	0.0	0.0
0.0	0.0	0.0	0.0	0.0	0.0	0.0	0.0
0.0	0.0	0.0	0.0	0.0	0.0	0.0	0.0
0.0	0.0	0.0	0.0	0.0	0.0	0.0	0.0
0.0	0.0	0.0	0.0	0.0	0.0	0.0	0.0
0.0	0.0	0.0	0.0	0.0	0.0	0.0	0.0
0.0	0.0	0.0	0.0	0.0	0.0	0.0	0.0
0.0	0.0	0.0	0.0	0.0	0.0	0.0	0.0
0.0	0.0	0.0	0.0	0.0	0.0	0.0	0.0
0.0	0.0	0.0	0.0	0.0	0.0	0.0	0.0
0.0	0.0	0.0	0.0	0.0	0.0	0.0	0.0
0.0	0.0	0.0	0.0	0.0	0.0	0.0	0.0
0.0	0.0	0.0	0.0	0.0	0.0	0.0	0.0
0.0	0.0	0.0	0.0	0.0	0.0	0.0	0.0
0.0	0.0	0.0	0.0	0.0	0.0	0.0	0.0
0.0	0.0	0.0	0.0	0.0	0.0	0.0	0.0
0.0	0.0	0.0	0.0	0.0	0.0	0.0	0.0
0.0	0.0	0.0	0.0	0.0	0.0	0.0	0.0
0.0	0.0	0.0	0.0	0.0	0.0	0.0	0.0
0.0	0.0	0.0	0.0	0.0	0.0	0.0	0.0
0.0	0.0	0.0	0.0	0.0	0.0	0.0	0.0
0.0	0.0	0.0	0.0	0.0	0.0	0.0	0.0
0.0	0.0	0.0	0.0	0.0	0.0	0.0	0.0
0.0	0.0	0.0	0.0	0.0	0.0	0.0	0.0
0.0	0.0	0.0	0.0	0.0	0.0	0.0	0.0
0.0	0.0	0.0	0.0	0.0	0.0	0.0	0.0
0.0	0.0	0.0	0.0	0.0	0.0	0.0	0.0
0.0	0.0	0.0	0.0	0.0	0.0	0.0	0.0
0.0	0.0	0.0	0.0	0.0	0.0	0.0	0.0
0.0	0.0	0.0	0.0	0.0	0.0	0.0	0.0

0.0	0.0	0.0	0.0	0.0	0.0	0.0	0.0
0.0	0.0	0.0	0.0	0.0	0.0	0.0	0.0
0.0	0.0	0.0	0.0	0.0	0.0	0.0	0.0
0.0	0.0	0.0	0.0	0.0	0.0	0.0	0.0
0.0	0.0	0.0	0.0	0.0	0.0	0.0	0.0
0.0	0.0	0.0	0.0	0.0	0.0	0.0	0.0
0.0	0.0	0.0	0.0	0.0	0.0	0.0	0.0
0.0	0.0	0.0	0.0	0.0	0.0	0.0	0.0
0.0	0.0	0.0	0.0	0.0	0.0	0.0	0.0
0.0	0.0	0.0	0.0	0.0	0.0	0.0	0.0
0.0	0.0	0.0	0.0	0.0	0.0	0.0	0.0
0.0	0.0	0.0	0.0	0.0	0.0	0.0	0.0
0.0	0.0	0.0	0.0	0.0	0.0	0.0	0.0
0.0	0.0	0.0	0.0	0.0	0.0	0.0	0.0
0.0	0.0	0.0	0.0	0.0	0.0	0.0	0.0
0.0	0.0	0.0	0.0	0.0	0.0	0.0	0.0
0.0	0.0	0.0	0.0	0.0	0.0	0.0	0.0
0.0	0.0	0.0	0.0	0.0	0.0	0.0	0.0
0.0	0.0	0.0	0.0	0.0	0.0	0.0	0.0
0.0	0.0	0.0	0.0	0.0	0.0	0.0	0.0
0.0	0.0	0.0	0.0	0.0	0.0	0.0	0.0
0.0	0.0	0.0	0.0	0.0	0.0	0.0	0.0
0.0	0.0	0.0	0.0	0.0	0.0	0.0	0.0
0.0	0.0	0.0	0.0	0.0	0.0	0.0	0.0
0.0	0.0	0.0	0.0	0.0	0.0	0.0	0.0
0.0	0.0	0.0	0.0	0.0	0.0	0.0	0.0
0.0	0.0	0.0	0.0	0.0	0.0	0.0	0.0
0.0	0.0	0.0	0.0	0.0	0.0	0.0	0.0
0.0	0.0	0.0	0.0	0.0	0.0	0.0	0.0
0.0	0.0	0.0	0.0	0.0	0.0	0.0	0.0
0.0	0.0	0.0	0.0	0.0	0.0	0.0	0.0
0.0	0.0	0.0	0.0	0.0	0.0	0.0	0.0
0.0	0.0	0.0	0.0	0.0	0.0	0.0	0.0
0.0	0.0	0.0	0.0	0.0	0.0	0.0	0.0
0.0	0.0	0.0	0.0	0.0	0.0	0.0	0.0
0.0	0.0	0.0	0.0	0.0	0.0	0.0	0.0
0.0	0.0	0.0	0.0	0.0	0.0	0.0	0.0
0.0	0.0	0.0	0.0	0.0	0.0	0.0	0.0
0.0	0.0	0.0	0.0	0.0	0.0	0.0	0.0
0.0	0.0	0.0	0.0	0.0	0.0	0.0	0.0
0.0	0.0	0.0	0.0	0.0	0.0	0.0	0.0
0.0	0.0	0.0	0.0	0.0	0.0	0.0	0.0
0.0	0.0	0.0	0.0	0.0	0.0	0.0	0.0
0.0	0.0	0.0	0.0	0.0	0.0	0.0	0.0
0.0	0.0	0.0	0.0	0.0	0.0	0.0	0.0
0.0	0.0	0.0	0.0	0.0	0.0	0.0	0.0
0.0	0.0	0.0	0.0	0.0	0.0	0.0	0.0

0.0	0.0	0.0	0.0	0.0	0.0	0.0	0.0
0.0	0.0	0.0	0.0	0.0	0.0	0.0	0.0
0.0	0.0	0.0	0.0	0.0	0.0	0.0	0.0
0.0	0.0	0.0	0.0	0.0	0.0	0.0	0.0
0.0	0.0	0.0	0.0	0.0	0.0	0.0	0.0
0.0	0.0	0.0	0.0	0.0	0.0	0.0	0.0
0.0	0.0	0.0	0.0	0.0	0.0	0.0	0.0
0.0	0.0	0.0	0.0	0.0	0.0	0.0	0.0
0.0	0.0	0.0	0.0	0.0	0.0	0.0	0.0
0.0	0.0	0.0	0.0	0.0	0.0	0.0	0.0
0.0	0.0	0.0	0.0	0.0	0.0	0.0	0.0
0.0	0.0	0.0	0.0	0.0	0.0	0.0	0.0
0.0	0.0	0.0	0.0	0.0	0.0	0.0	0.0
0.0	0.0	0.0	0.0	0.0	0.0	0.0	0.0
0.0	0.0	0.0	0.0	0.0	0.0	0.0	0.0
0.0	0.0	0.0	0.0	0.0	0.0	0.0	0.0
0.0	0.0	0.0	0.0	0.0	0.0	0.0	0.0
0.0	0.0	0.0	0.0	0.0	0.0	0.0	0.0
0.0	0.0	0.0	0.0	0.0	0.0	0.0	0.0
0.0	0.0	0.0	0.0	0.0	0.0	0.0	0.0
0.0	0.0	0.0	0.0	0.0	0.0	0.0	0.0
0.0	0.0	0.0	0.0	0.0	0.0	0.0	0.0
0.0	0.0	0.0	0.0	0.0	0.0	0.0	0.0
0.0	0.0	0.0	0.0	0.0	0.0	0.0	0.0
0.0	0.0	0.0	0.0	0.0	0.0	0.0	0.0
0.0	0.0	0.0	0.0	0.0	0.0	0.0	0.0
0.0	0.0	0.0	0.0	0.0	0.0	0.0	0.0
0.0	0.0	0.0	0.0	0.0	0.0	0.0	0.0
0.0	0.0	0.0	0.0	0.0	0.0	0.0	0.0
0.0	0.0	0.0	0.0	0.0	0.0	0.0	0.0
0.0	0.0	0.0	0.0	0.0	0.0	0.0	0.0
0.0	0.0	0.0	0.0	0.0	0.0	0.0	0.0
0.0	0.0	0.0	0.0	0.0	0.0	0.0	0.0
0.0	0.0	0.0	0.0	0.0	0.0	0.0	0.0
0.0	0.0	0.0	0.0	0.0	0.0	0.0	0.0
0.0	0.0	0.0	0.0	0.0	0.0	0.0	0.0
0.0	0.0	0.0	0.0	0.0	0.0	0.0	0.0
0.0	0.0	0.0	0.0	0.0	0.0	0.0	0.0
0.0	0.0	0.0	0.0	0.0	0.0	0.0	0.0
0.0	0.0	0.0	0.0	0.0	0.0	0.0	0.0
0.0	0.0	0.0	0.0	0.0	0.0	0.0	0.0
0.0	0.0	0.0	0.0	0.0	0.0	0.0	0.0
0.0	0.0	0.0	0.0	0.0	0.0	0.0	0.0
0.0	0.0	0.0	0.0	0.0	0.0	0.0	0.0
0.0	0.0	0.0	0.0	0.0	0.0	0.0	0.0
0.0	0.0	0.0	0.0	0.0	0.0	0.0	0.0
0.0	0.0	0.0	0.0	0.0	0.0	0.0	0.0

0.0	0.0	0.0	0.0	0.0	0.0	0.0	0.0
0.0	0.0	0.0	0.0	0.0	0.0	0.0	0.0
0.0	0.0	0.0	0.0	0.0	0.0	0.0	0.0
0.0	0.0	0.0	0.0	0.0	0.0	0.0	0.0
0.0	0.0	0.0	0.0	0.0	0.0	0.0	0.0
0.0	0.0	0.0	0.0	0.0	0.0	0.0	0.0
0.0	0.0	0.0	0.0	0.0	0.0	0.0	0.0
0.0	0.0	0.0	0.0	0.0	0.0	0.0	0.0
0.0	0.0	0.0	0.0	0.0	0.0	0.0	0.0
0.0	0.0	0.0	0.0	0.0	0.0	0.0	0.0
0.0	0.0	0.0	0.0	0.0	0.0	0.0	0.0
0.0	0.0	0.0	0.0	0.0	0.0	0.0	0.0
0.0	0.0	0.0	0.0	0.0	0.0	0.0	0.0
0.0	0.0	0.0	0.0	0.0	0.0	0.0	0.0
0.0	0.0	0.0	0.0	0.0	0.0	0.0	0.0
0.0	0.0	0.0	0.0	0.0	0.0	0.0	0.0
0.0	0.0	0.0	0.0	0.0	0.0	0.0	0.0
0.0	0.0	0.0	0.0	0.0	0.0	0.0	0.0
0.0	0.0	0.0	0.0	0.0	0.0	0.0	0.0
0.0	0.0	0.0	0.0	0.0	0.0	0.0	0.0
0.0	0.0	0.0	0.0	0.0	0.0	0.0	0.0
0.0	0.0	0.0	0.0	0.0	0.0	0.0	0.0
0.0	0.0	0.0	0.0	0.0	0.0	0.0	0.0
0.0	0.0	0.0	0.0	0.0	0.0	0.0	0.0
0.0	0.0	0.0	0.0	0.0	0.0	0.0	0.0
0.0	0.0	0.0	0.0	0.0	0.0	0.0	0.0
0.0	0.0	0.0	0.0	0.0	0.0	0.0	0.0
0.0	0.0	0.0	0.0	0.0	0.0	0.0	0.0
0.0	0.0	0.0	0.0	0.0	0.0	0.0	0.0
0.0	0.0	0.0	0.0	0.0	0.0	0.0	0.0
0.0	0.0	0.0	0.0	0.0	0.0	0.0	0.0
0.0	0.0	0.0	0.0	0.0	0.0	0.0	0.0
0.0	0.0	0.0	0.0	0.0	0.0	0.0	0.0
0.0	0.0	0.0	0.0	0.0	0.0	0.0	0.0
0.0	0.0	0.0	0.0	0.0	0.0	0.0	0.0
0.0	0.0	0.0	0.0	0.0	0.0	0.0	0.0
0.0	0.0	0.0	0.0	0.0	0.0	0.0	0.0
0.0	0.0	0.0	0.0	0.0	0.0	0.0	0.0
0.0	0.0	0.0	0.0	0.0	0.0	0.0	0.0
0.0	0.0	0.0	0.0	0.0	0.0	0.0	0.0
0.0	0.0	0.0	0.0	0.0	0.0	0.0	0.0
0.0	0.0	0.0	0.0	0.0	0.0	0.0	0.0
0.0	0.0	0.0	0.0	0.0	0.0	0.0	0.0
0.0	0.0	0.0	0.0	0.0	0.0	0.0	0.0
0.0	0.0	0.0	0.0	0.0	0.0	0.0	0.0
0.0	0.0	0.0	0.0	0.0	0.0	0.0	0.0
0.0	0.0	0.0	0.0	0.0	0.0	0.0	0.0
0.0	0.0	0.0	0.0	0.0	0.0	0.0	0.0

0.0	0.0	0.0	0.0	0.0	0.0	0.0	0.0
0.0	0.0	0.0	0.0	0.0	0.0	0.0	0.0
0.0	0.0	0.0	0.0	0.0	0.0	0.0	0.0
0.0	0.0	0.0	0.0	0.0	0.0	0.0	0.0
0.0	0.0	0.0	0.0	0.0	0.0	0.0	0.0
0.0	0.0	0.0	0.0	0.0	0.0	0.0	0.0
0.0	0.0	0.0	0.0	0.0	0.0	0.0	0.0
0.0	0.0	0.0	0.0	0.0	0.0	0.0	0.0
0.0	0.0	0.0	0.0	0.0	0.0	0.0	0.0
0.0	0.0	0.0	0.0	0.0	0.0	0.0	0.0
0.0	0.0	0.0	0.0	0.0	0.0	0.0	0.0
0.0	0.0	0.0	0.0	0.0	0.0	0.0	0.0
0.0	0.0	0.0	0.0	0.0	0.0	0.0	0.0
0.0	0.0	0.0	0.0	0.0	0.0	0.0	0.0
0.0	0.0	0.0	0.0	0.0	0.0	0.0	0.0
0.0	0.0	0.0	0.0	0.0	0.0	0.0	0.0
0.0	0.0	0.0	0.0	0.0	0.0	0.0	0.0
0.0	0.0	0.0	0.0	0.0	0.0	0.0	0.0
0.0	0.0	0.0	0.0	0.0	0.0	0.0	0.0
0.0	0.0	0.0	0.0	0.0	0.0	0.0	0.0
0.0	0.0	0.0	0.0	0.0	0.0	0.0	0.0
0.0	0.0	0.0	0.0	0.0	0.0	0.0	0.0
0.0	0.0	0.0	0.0	0.0	0.0	0.0	0.0
0.0	0.0	0.0	0.0	0.0	0.0	0.0	0.0
0.0	0.0	0.0	0.0	0.0	0.0	0.0	0.0
0.0	0.0	0.0	0.0	0.0	0.0	0.0	0.0
0.0	0.0	0.0	0.0	0.0	0.0	0.0	0.0
0.0	0.0	0.0	0.0	0.0	0.0	0.0	0.0
0.0	0.0	0.0	0.0	0.0	0.0	0.0	0.0
0.0	0.0	0.0	0.0	0.0	0.0	0.0	0.0
0.0	0.0	0.0	0.0	0.0	0.0	0.0	0.0
0.0	0.0	0.0	0.0	0.0	0.0	0.0	0.0
0.0	0.0	0.0	0.0	0.0	0.0	0.0	0.0
0.0	0.0	0.0	0.0	0.0	0.0	0.0	0.0
0.0	0.0	0.0	0.0	0.0	0.0	0.0	0.0
0.0	0.0	0.0	0.0	0.0	0.0	0.0	0.0
0.0	0.0	0.0	0.0	0.0	0.0	0.0	0.0
0.0	0.0	0.0	0.0	0.0	0.0	0.0	0.0
0.0	0.0	0.0	0.0	0.0	0.0	0.0	0.0
0.0	0.0	0.0	0.0	0.0	0.0	0.0	0.0
0.0	0.0	0.0	0.0	0.0	0.0	0.0	0.0
0.0	0.0	0.0	0.0	0.0	0.0	0.0	0.0
0.0	0.0	0.0	0.0	0.0	0.0	0.0	0.0
0.0	0.0	0.0	0.0	0.0	0.0	0.0	0.0
0.0	0.0	0.0	0.0	0.0	0.0	0.0	0.0
0.0	0.0	0.0	0.0	0.0	0.0	0.0	0.0
0.0	0.0	0.0	0.0	0.0	0.0	0.0	0.0

0.0	0.0	0.0	0.0	0.0	0.0	0.0	0.0
0.0	0.0	0.0	0.0	0.0	0.0	0.0	0.0
0.0	0.0	0.0	0.0	0.0	0.0	0.0	0.0
0.0	0.0	0.0	0.0	0.0	0.0	0.0	0.0
0.0	0.0	0.0	0.0	0.0	0.0	0.0	0.0
0.0	0.0	0.0	0.0	0.0	0.0	0.0	0.0
0.0	0.0	0.0	0.0	0.0	0.0	0.0	0.0
0.0	0.0	0.0	0.0	0.0	0.0	0.0	0.0
0.0	0.0	0.0	0.0	0.0	0.0	0.0	0.0
0.0	0.0	0.0	0.0	0.0	0.0	0.0	0.0
0.0	0.0	0.0	0.0	0.0	0.0	0.0	0.0
0.0	0.0	0.0	0.0	0.0	0.0	0.0	0.0
0.0	0.0	0.0	0.0	0.0	0.0	0.0	0.0
0.0	0.0	0.0	0.0	0.0	0.0	0.0	0.0
0.0	0.0	0.0	0.0	0.0	0.0	0.0	0.0
0.0	0.0	0.0	0.0	0.0	0.0	0.0	0.0
0.0	0.0	0.0	0.0	0.0	0.0	0.0	0.0
0.0	0.0	0.0	0.0	0.0	0.0	0.0	0.0
0.0	0.0	0.0	0.0	0.0	0.0	0.0	0.0
0.0	0.0	0.0	0.0	0.0	0.0	0.0	0.0
0.0	0.0	0.0	0.0	0.0	0.0	0.0	0.0
0.0	0.0	0.0	0.0	0.0	0.0	0.0	0.0
0.0	0.0	0.0	0.0	0.0	0.0	0.0	0.0
0.0	0.0	0.0	0.0	0.0	0.0	0.0	0.0
0.0	0.0	0.0	0.0	0.0	0.0	0.0	0.0
0.0	0.0	0.0	0.0	0.0	0.0	0.0	0.0
0.0	0.0	0.0	0.0	0.0	0.0	0.0	0.0
0.0	0.0	0.0	0.0	0.0	0.0	0.0	0.0
0.0	0.0	0.0	0.0	0.0	0.0	0.0	0.0
0.0	0.0	0.0	0.0	0.0	0.0	0.0	0.0
0.0	0.0	0.0	0.0	0.0	0.0	0.0	0.0
0.0	0.0	0.0	0.0	0.0	0.0	0.0	0.0
0.0	0.0	0.0	0.0	0.0	0.0	0.0	0.0
0.0	0.0	0.0	0.0	0.0	0.0	0.0	0.0
0.0	0.0	0.0	0.0	0.0	0.0	0.0	0.0
0.0	0.0	0.0	0.0	0.0	0.0	0.0	0.0
0.0	0.0	0.0	0.0	0.0	0.0	0.0	0.0
0.0	0.0	0.0	0.0	0.0	0.0	0.0	0.0
0.0	0.0	0.0	0.0	0.0	0.0	0.0	0.0
0.0	0.0	0.0	0.0	0.0	0.0	0.0	0.0
0.0	0.0	0.0	0.0	0.0	0.0	0.0	0.0
0.0	0.0	0.0	0.0	0.0	0.0	0.0	0.0
0.0	0.0	0.0	0.0	0.0	0.0	0.0	0.0
0.0	0.0	0.0	0.0	0.0	0.0	0.0	0.0
0.0	0.0	0.0	0.0	0.0	0.0	0.0	0.0
0.0	0.0	0.0	0.0	0.0	0.0	0.0	0.0
0.0	0.0	0.0	0.0	0.0	0.0	0.0	0.0

0.0	0.0	0.0	0.0	0.0	0.0	0.0	0.0
0.0	0.0	0.0	0.0	0.0	0.0	0.0	0.0
0.0	0.0	0.0	0.0	0.0	0.0	0.0	0.0
0.0	0.0	0.0	0.0	0.0	0.0	0.0	0.0
0.0	0.0	0.0	0.0	0.0	0.0	0.0	0.0
0.0	0.0	0.0	0.0	0.0	0.0	0.0	0.0
0.0	0.0	0.0	0.0	0.0	0.0	0.0	0.0
0.0	0.0	0.0	0.0	0.0	0.0	0.0	0.0
0.0	0.0	0.0	0.0	0.0	0.0	0.0	0.0
0.0	0.0	0.0	0.0	0.0	0.0	0.0	0.0
0.0	0.0	0.0	0.0	0.0	0.0	0.0	0.0
0.0	0.0	0.0	0.0	0.0	0.0	0.0	0.0
0.0	0.0	0.0	0.0	0.0	0.0	0.0	0.0
0.0	0.0	0.0	0.0	0.0	0.0	0.0	0.0
0.0	0.0	0.0	0.0	0.0	0.0	0.0	0.0
0.0	0.0	0.0	0.0	0.0	0.0	0.0	0.0
0.0	0.0	0.0	0.0	0.0	0.0	0.0	0.0
0.0	0.0	0.0	0.0	0.0	0.0	0.0	0.0
0.0	0.0	0.0	0.0	0.0	0.0	0.0	0.0
0.0	0.0	0.0	0.0	0.0	0.0	0.0	0.0
0.0	0.0	0.0	0.0	0.0	0.0	0.0	0.0
0.0	0.0	0.0	0.0	0.0	0.0	0.0	0.0
0.0	0.0	0.0	0.0	0.0	0.0	0.0	0.0
0.0	0.0	0.0	0.0	0.0	0.0	0.0	0.0
0.0	0.0	0.0	0.0	0.0	0.0	0.0	0.0
0.0	0.0	0.0	0.0	0.0	0.0	0.0	0.0
0.0	0.0	0.0	0.0	0.0	0.0	0.0	0.0
0.0	0.0	0.0	0.0	0.0	0.0	0.0	0.0
0.0	0.0	0.0	0.0	0.0	0.0	0.0	0.0
0.0	0.0	0.0	0.0	0.0	0.0	0.0	0.0
0.0	0.0	0.0	0.0	0.0	0.0	0.0	0.0
0.0	0.0	0.0	0.0	0.0	0.0	0.0	0.0
0.0	0.0	0.0	0.0	0.0	0.0	0.0	0.0
0.0	0.0	0.0	0.0	0.0	0.0	0.0	0.0
0.0	0.0	0.0	0.0	0.0	0.0	0.0	0.0
0.0	0.0	0.0	0.0	0.0	0.0	0.0	0.0
0.0	0.0	0.0	0.0	0.0	0.0	0.0	0.0
0.0	0.0	0.0	0.0	0.0	0.0	0.0	0.0
0.0	0.0	0.0	0.0	0.0	0.0	0.0	0.0
0.0	0.0	0.0	0.0	0.0	0.0	0.0	0.0
0.0	0.0	0.0	0.0	0.0	0.0	0.0	0.0
0.0	0.0	0.0	0.0	0.0	0.0	0.0	0.0
0.0	0.0	0.0	0.0	0.0	0.0	0.0	0.0
0.0	0.0	0.0	0.0	0.0	0.0	0.0	0.0
0.0	0.0	0.0	0.0	0.0	0.0	0.0	0.0
0.0	0.0	0.0	0.0	0.0	0.0	0.0	0.0
0.0	0.0	0.0	0.0	0.0	0.0	0.0	0.0

0.0	0.0	0.0	0.0	0.0	0.0	0.0	0.0
0.0	0.0	0.0	0.0	0.0	0.0	0.0	0.0
0.0	0.0	0.0	0.0	0.0	0.0	0.0	0.0
0.0	0.0	0.0	0.0	0.0	0.0	0.0	0.0
0.0	0.0	0.0	0.0	0.0	0.0	0.0	0.0
0.0	0.0	0.0	0.0	0.0	0.0	0.0	0.0
0.0	0.0	0.0	0.0	0.0	0.0	0.0	0.0
0.0	0.0	0.0	0.0	0.0	0.0	0.0	0.0
0.0	0.0	0.0	0.0	0.0	0.0	0.0	0.0
0.0	0.0	0.0	0.0	0.0	0.0	0.0	0.0
0.0	0.0	0.0	0.0	0.0	0.0	0.0	0.0
0.0	0.0	0.0	0.0	0.0	0.0	0.0	0.0
0.0	0.0	0.0	0.0	0.0	0.0	0.0	0.0
0.0	0.0	0.0	0.0	0.0	0.0	0.0	0.0
0.0	0.0	0.0	0.0	0.0	0.0	0.0	0.0
0.0	0.0	0.0	0.0	0.0	0.0	0.0	0.0
0.0	0.0	0.0	0.0	0.0	0.0	0.0	0.0
0.0	0.0	0.0	0.0	0.0	0.0	0.0	0.0
0.0	0.0	0.0	0.0	0.0	0.0	0.0	0.0
0.0	0.0	0.0	0.0	0.0	0.0	0.0	0.0
0.0	0.0	0.0	0.0	0.0	0.0	0.0	0.0
0.0	0.0	0.0	0.0	0.0	0.0	0.0	0.0
0.0	0.0	0.0	0.0	0.0	0.0	0.0	0.0
0.0	0.0	0.0	0.0	0.0	0.0	0.0	0.0
0.0	0.0	0.0	0.0	0.0	0.0	0.0	0.0
0.0	0.0	0.0	0.0	0.0	0.0	0.0	0.0
0.0	0.0	0.0	0.0	0.0	0.0	0.0	0.0
0.0	0.0	0.0	0.0	0.0	0.0	0.0	0.0
0.0	0.0	0.0	0.0	0.0	0.0	0.0	0.0
0.0	0.0	0.0	0.0	0.0	0.0	0.0	0.0
0.0	0.0	0.0	0.0	0.0	0.0	0.0	0.0
0.0	0.0	0.0	0.0	0.0	0.0	0.0	0.0
0.0	0.0	0.0	0.0	0.0	0.0	0.0	0.0
0.0	0.0	0.0	0.0	0.0	0.0	0.0	0.0
0.0	0.0	0.0	0.0	0.0	0.0	0.0	0.0
0.0	0.0	0.0	0.0	0.0	0.0	0.0	0.0
0.0	0.0	0.0	0.0	0.0	0.0	0.0	0.0
0.0	0.0	0.0	0.0	0.0	0.0	0.0	0.0
0.0	0.0	0.0	0.0	0.0	0.0	0.0	0.0
0.0	0.0	0.0	0.0	0.0	0.0	0.0	0.0
0.0	0.0	0.0	0.0	0.0	0.0	0.0	0.0
0.0	0.0	0.0	0.0	0.0	0.0	0.0	0.0
0.0	0.0	0.0	0.0	0.0	0.0	0.0	0.0
0.0	0.0	0.0	0.0	0.0	0.0	0.0	0.0
0.0	0.0	0.0	0.0	0.0	0.0	0.0	0.0
0.0	0.0	0.0	0.0	0.0	0.0	0.0	0.0
0.0	0.0	0.0	0.0	0.0	0.0	0.0	0.0
0.0	0.0	0.0	0.0	0.0	0.0	0.0	0.0

0.0	0.0	0.0	0.0	0.0	0.0	0.0	0.0
0.0	0.0	0.0	0.0	0.0	0.0	0.0	0.0
0.0	0.0	0.0	0.0	0.0	0.0	0.0	0.0
0.0	0.0	0.0	0.0	0.0	0.0	0.0	0.0
0.0	0.0	0.0	0.0	0.0	0.0	0.0	0.0
0.0	0.0	0.0	0.0	0.0	0.0	0.0	0.0
0.0	0.0	0.0	0.0	0.0	0.0	0.0	0.0
0.0	0.0	0.0	0.0	0.0	0.0	0.0	0.0
0.0	0.0	0.0	0.0	0.0	0.0	0.0	0.0
0.0	0.0	0.0	0.0	0.0	0.0	0.0	0.0
0.0	0.0	0.0	0.0	0.0	0.0	0.0	0.0
0.0	0.0	0.0	0.0	0.0	0.0	0.0	0.0
0.0	0.0	0.0	0.0	0.0	0.0	0.0	0.0
0.0	0.0	0.0	0.0	0.0	0.0	0.0	0.0
0.0	0.0	0.0	0.0	0.0	0.0	0.0	0.0
0.0	0.0	0.0	0.0	0.0	0.0	0.0	0.0
0.0	0.0	0.0	0.0	0.0	0.0	0.0	0.0
0.0	0.0	0.0	0.0	0.0	0.0	0.0	0.0
0.0	0.0	0.0	0.0	0.0	0.0	0.0	0.0
0.0	0.0	0.0	0.0	0.0	0.0	0.0	0.0
0.0	0.0	0.0	0.0	0.0	0.0	0.0	0.0
0.0	0.0	0.0	0.0	0.0	0.0	0.0	0.0
0.0	0.0	0.0	0.0	0.0	0.0	0.0	0.0
0.0	0.0	0.0	0.0	0.0	0.0	0.0	0.0
0.0	0.0	0.0	0.0	0.0	0.0	0.0	0.0
0.0	0.0	0.0	0.0	0.0	0.0	0.0	0.0
0.0	0.0	0.0	0.0	0.0	0.0	0.0	0.0
0.0	0.0	0.0	0.0	0.0	0.0	0.0	0.0
0.0	0.0	0.0	0.0	0.0	0.0	0.0	0.0
0.0	0.0	0.0	0.0	0.0	0.0	0.0	0.0
0.0	0.0	0.0	0.0	0.0	0.0	0.0	0.0
0.0	0.0	0.0	0.0	0.0	0.0	0.0	0.0
0.0	0.0	0.0	0.0	0.0	0.0	0.0	0.0
0.0	0.0	0.0	0.0	0.0	0.0	0.0	0.0
0.0	0.0	0.0	0.0	0.0	0.0	0.0	0.0
0.0	0.0	0.0	0.0	0.0	0.0	0.0	0.0
0.0	0.0	0.0	0.0	0.0	0.0	0.0	0.0
0.0	0.0	0.0	0.0	0.0	0.0	0.0	0.0
0.0	0.0	0.0	0.0	0.0	0.0	0.0	0.0
0.0	0.0	0.0	0.0	0.0	0.0	0.0	0.0
0.0	0.0	0.0	0.0	0.0	0.0	0.0	0.0
0.0	0.0	0.0	0.0	0.0	0.0	0.0	0.0
0.0	0.0	0.0	0.0	0.0	0.0	0.0	0.0
0.0	0.0	0.0	0.0	0.0	0.0	0.0	0.0
0.0	0.0	0.0	0.0	0.0	0.0	0.0	0.0
0.0	0.0	0.0	0.0	0.0	0.0	0.0	0.0
0.0	0.0	0.0	0.0	0.0	0.0	0.0	0.0

0.0	0.0	0.0	0.0	0.0	0.0	0.0	0.0
0.0	0.0	0.0	0.0	0.0	0.0	0.0	0.0
0.0	0.0	0.0	0.0	0.0	0.0	0.0	0.0
0.0	0.0	0.0	0.0	0.0	0.0	0.0	0.0
0.0	0.0	0.0	0.0	0.0	0.0	0.0	0.0
0.0	0.0	0.0	0.0	0.0	0.0	0.0	0.0
0.0	0.0	0.0	0.0	0.0	0.0	0.0	0.0
0.0	0.0	0.0	0.0	0.0	0.0	0.0	0.0
0.0	0.0	0.0	0.0	0.0	0.0	0.0	0.0
0.0	0.0	0.0	0.0	0.0	0.0	0.0	0.0
0.0	0.0	0.0	0.0	0.0	0.0	0.0	0.0
0.0	0.0	0.0	0.0	0.0	0.0	0.0	0.0
0.0	0.0	0.0	0.0	0.0	0.0	0.0	0.0
0.0	0.0	0.0	0.0	0.0	0.0	0.0	0.0
0.0	0.0	0.0	0.0	0.0	0.0	0.0	0.0
0.0	0.0	0.0	0.0	0.0	0.0	0.0	0.0
0.0	0.0	0.0	0.0	0.0	0.0	0.0	0.0
0.0	0.0	0.0	0.0	0.0	0.0	0.0	0.0
0.0	0.0	0.0	0.0	0.0	0.0	0.0	0.0
0.0	0.0	0.0	0.0	0.0	0.0	0.0	0.0
0.0	0.0	0.0	0.0	0.0	0.0	0.0	0.0
0.0	0.0	0.0	0.0	0.0	0.0	0.0	0.0
0.0	0.0	0.0	0.0	0.0	0.0	0.0	0.0
0.0	0.0	0.0	0.0	0.0	0.0	0.0	0.0
0.0	0.0	0.0	0.0	0.0	0.0	0.0	0.0
0.0	0.0	0.0	0.0	0.0	0.0	0.0	0.0
0.0	0.0	0.0	0.0	0.0	0.0	0.0	0.0
0.0	0.0	0.0	0.0	0.0	0.0	0.0	0.0
0.0	0.0	0.0	0.0	0.0	0.0	0.0	0.0
0.0	0.0	0.0	0.0	0.0	0.0	0.0	0.0
0.0	0.0	0.0	0.0	0.0	0.0	0.0	0.0
0.0	0.0	0.0	0.0	0.0	0.0	0.0	0.0
0.0	0.0	0.0	0.0	0.0	0.0	0.0	0.0
0.0	0.0	0.0	0.0	0.0	0.0	0.0	0.0
0.0	0.0	0.0	0.0	0.0	0.0	0.0	0.0
0.0	0.0	0.0	0.0	0.0	0.0	0.0	0.0
0.0	0.0	0.0	0.0	0.0	0.0	0.0	0.0
0.0	0.0	0.0	0.0	0.0	0.0	0.0	0.0
0.0	0.0	0.0	0.0	0.0	0.0	0.0	0.0
0.0	0.0	0.0	0.0	0.0	0.0	0.0	0.0
0.0	0.0	0.0	0.0	0.0	0.0	0.0	0.0
0.0	0.0	0.0	0.0	0.0	0.0	0.0	0.0
0.0	0.0	0.0	0.0	0.0	0.0	0.0	0.0
0.0	0.0	0.0	0.0	0.0	0.0	0.0	0.0
0.0	0.0	0.0	0.0	0.0	0.0	0.0	0.0
0.0	0.0	0.0	0.0	0.0	0.0	0.0	0.0
0.0	0.0	0.0	0.0	0.0	0.0	0.0	0.0

0.0	0.0	0.0	0.0	0.0	0.0	0.0	0.0
0.0	0.0	0.0	0.0	0.0	0.0	0.0	0.0
0.0	0.0	0.0	0.0	0.0	0.0	0.0	0.0
0.0	0.0	0.0	0.0	0.0	0.0	0.0	0.0
0.0	0.0	0.0	0.0	0.0	0.0	0.0	0.0
0.0	0.0	0.0	0.0	0.0	0.0	0.0	0.0
0.0	0.0	0.0	0.0	0.0	0.0	0.0	0.0
0.0	0.0	0.0	0.0	0.0	0.0	0.0	0.0
0.0	0.0	0.0	0.0	0.0	0.0	0.0	0.0
0.0	0.0	0.0	0.0	0.0	0.0	0.0	0.0
0.0	0.0	0.0	0.0	0.0	0.0	0.0	0.0
0.0	0.0	0.0	0.0	0.0	0.0	0.0	0.0
0.0	0.0	0.0	0.0	0.0	0.0	0.0	0.0
0.0	0.0	0.0	0.0	0.0	0.0	0.0	0.0
0.0	0.0	0.0	0.0	0.0	0.0	0.0	0.0
0.0	0.0	0.0	0.0	0.0	0.0	0.0	0.0
0.0	0.0	0.0	0.0	0.0	0.0	0.0	0.0
0.0	0.0	0.0	0.0	0.0	0.0	0.0	0.0
0.0	0.0	0.0	0.0	0.0	0.0	0.0	0.0
0.0	0.0	0.0	0.0	0.0	0.0	0.0	0.0
0.0	0.0	0.0	0.0	0.0	0.0	0.0	0.0
0.0	0.0	0.0	0.0	0.0	0.0	0.0	0.0
0.0	0.0	0.0	0.0	0.0	0.0	0.0	0.0
0.0	0.0	0.0	0.0	0.0	0.0	0.0	0.0
0.0	0.0	0.0	0.0	0.0	0.0	0.0	0.0
0.0	0.0	0.0	0.0	0.0	0.0	0.0	0.0
0.0	0.0	0.0	0.0	0.0	0.0	0.0	0.0
0.0	0.0	0.0	0.0	0.0	0.0	0.0	0.0
0.0	0.0	0.0	0.0	0.0	0.0	0.0	0.0
0.0	0.0	0.0	0.0	0.0	0.0	0.0	0.0
0.0	0.0	0.0	0.0	0.0	0.0	0.0	0.0
0.0	0.0	0.0	0.0	0.0	0.0	0.0	0.0
0.0	0.0	0.0	0.0	0.0	0.0	0.0	0.0
0.0	0.0	0.0	0.0	0.0	0.0	0.0	0.0
0.0	0.0	0.0	0.0	0.0	0.0	0.0	0.0
0.0	0.0	0.0	0.0	0.0	0.0	0.0	0.0
0.0	0.0	0.0	0.0	0.0	0.0	0.0	0.0
0.0	0.0	0.0	0.0	0.0	0.0	0.0	0.0
0.0	0.0	0.0	0.0	0.0	0.0	0.0	0.0
0.0	0.0	0.0	0.0	0.0	0.0	0.0	0.0
0.0	0.0	0.0	0.0	0.0	0.0	0.0	0.0
0.0	0.0	0.0	0.0	0.0	0.0	0.0	0.0
0.0	0.0	0.0	0.0	0.0	0.0	0.0	0.0
0.0	0.0	0.0	0.0	0.0	0.0	0.0	0.0
0.0	0.0	0.0	0.0	0.0	0.0	0.0	0.0
0.0	0.0	0.0	0.0	0.0	0.0	0.0	0.0
0.0	0.0	0.0	0.0	0.0	0.0	0.0	0.0
0.0	0.0	0.0	0.0	0.0	0.0	0.0	0.0

0.0	0.0	0.0	0.0	0.0	0.0	0.0	0.0
0.0	0.0	0.0	0.0	0.0	0.0	0.0	0.0
0.0	0.0	0.0	0.0	0.0	0.0	0.0	0.0
0.0	0.0	0.0	0.0	0.0	0.0	0.0	0.0
0.0	0.0	0.0	0.0	0.0	0.0	0.0	0.0
0.0	0.0	0.0	0.0	0.0	0.0	0.0	0.0
0.0	0.0	0.0	0.0	0.0	0.0	0.0	0.0
0.0	0.0	0.0	0.0	0.0	0.0	0.0	0.0
0.0	0.0	0.0	0.0	0.0	0.0	0.0	0.0
0.0	0.0	0.0	0.0	0.0	0.0	0.0	0.0
0.0	0.0	0.0	0.0	0.0	0.0	0.0	0.0
0.0	0.0	0.0	0.0	0.0	0.0	0.0	0.0
0.0	0.0	0.0	0.0	0.0	0.0	0.0	0.0
0.0	0.0	0.0	0.0	0.0	0.0	0.0	0.0
0.0	0.0	0.0	0.0	0.0	0.0	0.0	0.0
0.0	0.0	0.0	0.0	0.0	0.0	0.0	0.0
0.0	0.0	0.0	0.0	0.0	0.0	0.0	0.0
0.0	0.0	0.0	0.0	0.0	0.0	0.0	0.0
0.0	0.0	0.0	0.0	0.0	0.0	0.0	0.0
0.0	0.0	0.0	0.0	0.0	0.0	0.0	0.0
0.0	0.0	0.0	0.0	0.0	0.0	0.0	0.0
0.0	0.0	0.0	0.0	0.0	0.0	0.0	0.0
0.0	0.0	0.0	0.0	0.0	0.0	0.0	0.0
0.0	0.0	0.0	0.0	0.0	0.0	0.0	0.0
0.0	0.0	0.0	0.0	0.0	0.0	0.0	0.0
0.0	0.0	0.0	0.0	0.0	0.0	0.0	0.0
0.0	0.0	0.0	0.0	0.0	0.0	0.0	0.0
0.0	0.0	0.0	0.0	0.0	0.0	0.0	0.0
0.0	0.0	0.0	0.0	0.0	0.0	0.0	0.0
0.0	0.0	0.0	0.0	0.0	0.0	0.0	0.0
0.0	0.0	0.0	0.0	0.0	0.0	0.0	0.0
0.0	0.0	0.0	0.0	0.0	0.0	0.0	0.0
0.0	0.0	0.0	0.0	0.0	0.0	0.0	0.0
0.0	0.0	0.0	0.0	0.0	0.0	0.0	0.0
0.0	0.0	0.0	0.0	0.0	0.0	0.0	0.0
0.0	0.0	0.0	0.0	0.0	0.0	0.0	0.0
0.0	0.0	0.0	0.0	0.0	0.0	0.0	0.0
0.0	0.0	0.0	0.0	0.0	0.0	0.0	0.0
0.0	0.0	0.0	0.0	0.0	0.0	0.0	0.0
0.0	0.0	0.0	0.0	0.0	0.0	0.0	0.0
0.0	0.0	0.0	0.0	0.0	0.0	0.0	0.0
0.0	0.0	0.0	0.0	0.0	0.0	0.0	0.0
0.0	0.0	0.0	0.0	0.0	0.0	0.0	0.0
0.0	0.0	0.0	0.0	0.0	0.0	0.0	0.0
0.0	0.0	0.0	0.0	0.0	0.0	0.0	0.0
0.0	0.0	0.0	0.0	0.0	0.0	0.0	0.0
0.0	0.0	0.0	0.0	0.0	0.0	0.0	0.0
0.0	0.0	0.0	0.0	0.0	0.0	0.0	0.0

0.0	0.0	0.0	0.0	0.0	0.0	0.0	0.0
0.0	0.0	0.0	0.0	0.0	0.0	0.0	0.0
0.0	0.0	0.0	0.0	0.0	0.0	0.0	0.0
0.0	0.0	0.0	0.0	0.0	0.0	0.0	0.0
0.0	0.0	0.0	0.0	0.0	0.0	0.0	0.0
0.0	0.0	0.0	0.0	0.0	0.0	0.0	0.0
0.0	0.0	0.0	0.0	0.0	0.0	0.0	0.0
0.0	0.0	0.0	0.0	0.0	0.0	0.0	0.0
0.0	0.0	0.0	0.0	0.0	0.0	0.0	0.0
0.0	0.0	0.0	0.0	0.0	0.0	0.0	0.0
0.0	0.0	0.0	0.0	0.0	0.0	0.0	0.0
0.0	0.0	0.0	0.0	0.0	0.0	0.0	0.0
0.0	0.0	0.0	0.0	0.0	0.0	0.0	0.0
0.0	0.0	0.0	0.0	0.0	0.0	0.0	0.0
0.0	0.0	0.0	0.0	0.0	0.0	0.0	0.0
0.0	0.0	0.0	0.0	0.0	0.0	0.0	0.0
0.0	0.0	0.0	0.0	0.0	0.0	0.0	0.0
0.0	0.0	0.0	0.0	0.0	0.0	0.0	0.0
0.0	0.0	0.0	0.0	0.0	0.0	0.0	0.0
0.0	0.0	0.0	0.0	0.0	0.0	0.0	0.0
0.0	0.0	0.0	0.0	0.0	0.0	0.0	0.0
0.0	0.0	0.0	0.0	0.0	0.0	0.0	0.0
0.0	0.0	0.0	0.0	0.0	0.0	0.0	0.0
0.0	0.0	0.0	0.0	0.0	0.0	0.0	0.0
0.0	0.0	0.0	0.0	0.0	0.0	0.0	0.0
0.0	0.0	0.0	0.0	0.0	0.0	0.0	0.0
0.0	0.0	0.0	0.0	0.0	0.0	0.0	0.0
0.0	0.0	0.0	0.0	0.0	0.0	0.0	0.0
0.0	0.0	0.0	0.0	0.0	0.0	0.0	0.0
0.0	0.0	0.0	0.0	0.0	0.0	0.0	0.0
0.0	0.0	0.0	0.0	0.0	0.0	0.0	0.0
0.0	0.0	0.0	0.0	0.0	0.0	0.0	0.0
0.0	0.0	0.0	0.0	0.0	0.0	0.0	0.0
0.0	0.0	0.0	0.0	0.0	0.0	0.0	0.0
0.0	0.0	0.0	0.0	0.0	0.0	0.0	0.0
0.0	0.0	0.0	0.0	0.0	0.0	0.0	0.0
0.0	0.0	0.0	0.0	0.0	0.0	0.0	0.0
0.0	0.0	0.0	0.0	0.0	0.0	0.0	0.0
0.0	0.0	0.0	0.0	0.0	0.0	0.0	0.0
0.0	0.0	0.0	0.0	0.0	0.0	0.0	0.0
0.0	0.0	0.0	0.0	0.0	0.0	0.0	0.0
0.0	0.0	0.0	0.0	0.0	0.0	0.0	0.0
0.0	0.0	0.0	0.0	0.0	0.0	0.0	0.0
0.0	0.0	0.0	0.0	0.0	0.0	0.0	0.0
0.0	0.0	0.0	0.0	0.0	0.0	0.0	0.0
0.0	0.0	0.0	0.0	0.0	0.0	0.0	0.0
0.0	0.0	0.0	0.0	0.0	0.0	0.0	0.0

0.0	0.0	0.0	0.0	0.0	0.0	0.0	0.0
0.0	0.0	0.0	0.0	0.0	0.0	0.0	0.0
0.0	0.0	0.0	0.0	0.0	0.0	0.0	0.0
0.0	0.0	0.0	0.0	0.0	0.0	0.0	0.0
0.0	0.0	0.0	0.0	0.0	0.0	0.0	0.0
0.0	0.0	0.0	0.0	0.0	0.0	0.0	0.0
0.0	0.0	0.0	0.0	0.0	0.0	0.0	0.0
0.0	0.0	0.0	0.0	0.0	0.0	0.0	0.0
0.0	0.0	0.0	0.0	0.0	0.0	0.0	0.0
0.0	0.0	0.0	0.0	0.0	0.0	0.0	0.0
0.0	0.0	0.0	0.0	0.0	0.0	0.0	0.0
0.0	0.0	0.0	0.0	0.0	0.0	0.0	0.0
0.0	0.0	0.0	0.0	0.0	0.0	0.0	0.0
0.0	0.0	0.0	0.0	0.0	0.0	0.0	0.0
0.0	0.0	0.0	0.0	0.0	0.0	0.0	0.0
0.0	0.0	0.0	0.0	0.0	0.0	0.0	0.0
0.0	0.0	0.0	0.0	0.0	0.0	0.0	0.0
0.0	0.0	0.0	0.0	0.0	0.0	0.0	0.0
0.0	0.0	0.0	0.0	0.0	0.0	0.0	0.0
0.0	0.0	0.0	0.0	0.0	0.0	0.0	0.0
0.0	0.0	0.0	0.0	0.0	0.0	0.0	0.0
0.0	0.0	0.0	0.0	0.0	0.0	0.0	0.0
0.0	0.0	0.0	0.0	0.0	0.0	0.0	0.0
0.0	0.0	0.0	0.0	0.0	0.0	0.0	0.0
0.0	0.0	0.0	0.0	0.0	0.0	0.0	0.0
0.0	0.0	0.0	0.0	0.0	0.0	0.0	0.0
0.0	0.0	0.0	0.0	0.0	0.0	0.0	0.0
0.0	0.0	0.0	0.0	0.0	0.0	0.0	0.0
0.0	0.0	0.0	0.0	0.0	0.0	0.0	0.0
0.0	0.0	0.0	0.0	0.0	0.0	0.0	0.0
0.0	0.0	0.0	0.0	0.0	0.0	0.0	0.0
0.0	0.0	0.0	0.0	0.0	0.0	0.0	0.0
0.0	0.0	0.0	0.0	0.0	0.0	0.0	0.0
0.0	0.0	0.0	0.0	0.0	0.0	0.0	0.0
0.0	0.0	0.0	0.0	0.0	0.0	0.0	0.0
0.0	0.0	0.0	0.0	0.0	0.0	0.0	0.0
0.0	0.0	0.0	0.0	0.0	0.0	0.0	0.0
0.0	0.0	0.0	0.0	0.0	0.0	0.0	0.0
0.0	0.0	0.0	0.0	0.0	0.0	0.0	0.0
0.0	0.0	0.0	0.0	0.0	0.0	0.0	0.0
0.0	0.0	0.0	0.0	0.0	0.0	0.0	0.0
0.0	0.0	0.0	0.0	0.0	0.0	0.0	0.0
0.0	0.0	0.0	0.0	0.0	0.0	0.0	0.0
0.0	0.0	0.0	0.0	0.0	0.0	0.0	0.0
0.0	0.0	0.0	0.0	0.0	0.0	0.0	0.0
0.0	0.0	0.0	0.0	0.0	0.0	0.0	0.0
0.0	0.0	0.0	0.0	0.0	0.0	0.0	0.0
0.0	0.0	0.0	0.0	0.0	0.0	0.0	0.0

0.0	0.0	0.0	0.0	0.0	0.0	0.0	0.0
0.0	0.0	0.0	0.0	0.0	0.0	0.0	0.0
0.0	0.0	0.0	0.0	0.0	0.0	0.0	0.0
0.0	0.0	0.0	0.0	0.0	0.0	0.0	0.0
0.0	0.0	0.0	0.0	0.0	0.0	0.0	0.0
0.0	0.0	0.0	0.0	0.0	0.0	0.0	0.0
0.0	0.0	0.0	0.0	0.0	0.0	0.0	0.0
0.0	0.0	0.0	0.0	0.0	0.0	0.0	0.0
0.0	0.0	0.0	0.0	0.0	0.0	0.0	0.0
0.0	0.0	0.0	0.0	0.0	0.0	0.0	0.0
0.0	0.0	0.0	0.0	0.0	0.0	0.0	0.0
0.0	0.0	0.0	0.0	0.0	0.0	0.0	0.0
0.0	0.0	0.0	0.0	0.0	0.0	0.0	0.0
0.0	0.0	0.0	0.0	0.0	0.0	0.0	0.0
0.0	0.0	0.0	0.0	0.0	0.0	0.0	0.0
0.0	0.0	0.0	0.0	0.0	0.0	0.0	0.0
0.0	0.0	0.0	0.0	0.0	0.0	0.0	0.0
0.0	0.0	0.0	0.0	0.0	0.0	0.0	0.0
0.0	0.0	0.0	0.0	0.0	0.0	0.0	0.0
0.0	0.0	0.0	0.0	0.0	0.0	0.0	0.0
0.0	0.0	0.0	0.0	0.0	0.0	0.0	0.0
0.0	0.0	0.0	0.0	0.0	0.0	0.0	0.0
0.0	0.0	0.0	0.0	0.0	0.0	0.0	0.0
0.0	0.0	0.0	0.0	0.0	0.0	0.0	0.0
0.0	0.0	0.0	0.0	0.0	0.0	0.0	0.0
0.0	0.0	0.0	0.0	0.0	0.0	0.0	0.0
0.0	0.0	0.0	0.0	0.0	0.0	0.0	0.0
0.0	0.0	0.0	0.0	0.0	0.0	0.0	0.0
0.0	0.0	0.0	0.0	0.0	0.0	0.0	0.0
0.0	0.0	0.0	0.0	0.0	0.0	0.0	0.0
0.0	0.0	0.0	0.0	0.0	0.0	0.0	0.0
0.0	0.0	0.0	0.0	0.0	0.0	0.0	0.0
0.0	0.0	0.0	0.0	0.0	0.0	0.0	0.0
0.0	0.0	0.0	0.0	0.0	0.0	0.0	0.0
0.0	0.0	0.0	0.0	0.0	0.0	0.0	0.0
0.0	0.0	0.0	0.0	0.0	0.0	0.0	0.0
0.0	0.0	0.0	0.0	0.0	0.0	0.0	0.0
0.0	0.0	0.0	0.0	0.0	0.0	0.0	0.0
0.0	0.0	0.0	0.0	0.0	0.0	0.0	0.0
0.0	0.0	0.0	0.0	0.0	0.0	0.0	0.0
0.0	0.0	0.0	0.0	0.0	0.0	0.0	0.0
0.0	0.0	0.0	0.0	0.0	0.0	0.0	0.0
0.0	0.0	0.0	0.0	0.0	0.0	0.0	0.0
0.0	0.0	0.0	0.0	0.0	0.0	0.0	0.0
0.0	0.0	0.0	0.0	0.0	0.0	0.0	0.0
0.0	0.0	0.0	0.0	0.0	0.0	0.0	0.0
0.0	0.0	0.0	0.0	0.0	0.0	0.0	0.0
0.0	0.0	0.0	0.0	0.0	0.0	0.0	0.0

0.0	0.0	0.0	0.0	0.0	0.0	0.0	0.0
0.0	0.0	0.0	0.0	0.0	0.0	0.0	0.0
0.0	0.0	0.0	0.0	0.0	0.0	0.0	0.0
0.0	0.0	0.0	0.0	0.0	0.0	0.0	0.0
0.0	0.0	0.0	0.0	0.0	0.0	0.0	0.0
0.0	0.0	0.0	0.0	0.0	0.0	0.0	0.0
0.0	0.0	0.0	0.0	0.0	0.0	0.0	0.0
0.0	0.0	0.0	0.0	0.0	0.0	0.0	0.0
0.0	0.0	0.0	0.0	0.0	0.0	0.0	0.0
0.0	0.0	0.0	0.0	0.0	0.0	0.0	0.0
0.0	0.0	0.0	0.0	0.0	0.0	0.0	0.0
0.0	0.0	0.0	0.0	0.0	0.0	0.0	0.0
0.0	0.0	0.0	0.0	0.0	0.0	0.0	0.0
0.0	0.0	0.0	0.0	0.0	0.0	0.0	0.0
0.0	0.0	0.0	0.0	0.0	0.0	0.0	0.0
0.0	0.0	0.0	0.0	0.0	0.0	0.0	0.0
0.0	0.0	0.0	0.0	0.0	0.0	0.0	0.0
0.0	0.0	0.0	0.0	0.0	0.0	0.0	0.0
0.0	0.0	0.0	0.0	0.0	0.0	0.0	0.0
0.0	0.0	0.0	0.0	0.0	0.0	0.0	0.0
0.0	0.0	0.0	0.0	0.0	0.0	0.0	0.0
0.0	0.0	0.0	0.0	0.0	0.0	0.0	0.0
0.0	0.0	0.0	0.0	0.0	0.0	0.0	0.0
0.0	0.0	0.0	0.0	0.0	0.0	0.0	0.0
0.0	0.0	0.0	0.0	0.0	0.0	0.0	0.0
0.0	0.0	0.0	0.0	0.0	0.0	0.0	0.0
0.0	0.0	0.0	0.0	0.0	0.0	0.0	0.0
0.0	0.0	0.0	0.0	0.0	0.0	0.0	0.0
0.0	0.0	0.0	0.0	0.0	0.0	0.0	0.0
0.0	0.0	0.0	0.0	0.0	0.0	0.0	0.0
0.0	0.0	0.0	0.0	0.0	0.0	0.0	0.0
0.0	0.0	0.0	0.0	0.0	0.0	0.0	0.0
0.0	0.0	0.0	0.0	0.0	0.0	0.0	0.0
0.0	0.0	0.0	0.0	0.0	0.0	0.0	0.0
0.0	0.0	0.0	0.0	0.0	0.0	0.0	0.0
0.0	0.0	0.0	0.0	0.0	0.0	0.0	0.0
0.0	0.0	0.0	0.0	0.0	0.0	0.0	0.0
0.0	0.0	0.0	0.0	0.0	0.0	0.0	0.0
0.0	0.0	0.0	0.0	0.0	0.0	0.0	0.0
0.0	0.0	0.0	0.0	0.0	0.0	0.0	0.0
0.0	0.0	0.0	0.0	0.0	0.0	0.0	0.0
0.0	0.0	0.0	0.0	0.0	0.0	0.0	0.0
0.0	0.0	0.0	0.0	0.0	0.0	0.0	0.0
0.0	0.0	0.0	0.0	0.0	0.0	0.0	0.0
0.0	0.0	0.0	0.0	0.0	0.0	0.0	0.0
0.0	0.0	0.0	0.0	0.0	0.0	0.0	0.0
0.0	0.0	0.0	0.0	0.0	0.0	0.0	0.0
0.0	0.0	0.0	0.0	0.0	0.0	0.0	0.0

0.0	0.0	0.0	0.0	0.0	0.0	0.0	0.0
0.0	0.0	0.0	0.0	0.0	0.0	0.0	0.0
0.0	0.0	0.0	0.0	0.0	0.0	0.0	0.0
0.0	0.0	0.0	0.0	0.0	0.0	0.0	0.0
0.0	0.0	0.0	0.0	0.0	0.0	0.0	0.0
0.0	0.0	0.0	0.0	0.0	0.0	0.0	0.0
0.0	0.0	0.0	0.0	0.0	0.0	0.0	0.0
0.0	0.0	0.0	0.0	0.0	0.0	0.0	0.0
0.0	0.0	0.0	0.0	0.0	0.0	0.0	0.0
0.0	0.0	0.0	0.0	0.0	0.0	0.0	0.0
0.0	0.0	0.0	0.0	0.0	0.0	0.0	0.0
0.0	0.0	0.0	0.0	0.0	0.0	0.0	0.0
0.0	0.0	0.0	0.0	0.0	0.0	0.0	0.0
0.0	0.0	0.0	0.0	0.0	0.0	0.0	0.0
0.0	0.0	0.0	0.0	0.0	0.0	0.0	0.0
0.0	0.0	0.0	0.0	0.0	0.0	0.0	0.0
0.0	0.0	0.0	0.0	0.0	0.0	0.0	0.0
0.0	0.0	0.0	0.0	0.0	0.0	0.0	0.0
0.0	0.0	0.0	0.0	0.0	0.0	0.0	0.0
0.0	0.0	0.0	0.0	0.0	0.0	0.0	0.0
0.0	0.0	0.0	0.0	0.0	0.0	0.0	0.0
0.0	0.0	0.0	0.0	0.0	0.0	0.0	0.0
0.0	0.0	0.0	0.0	0.0	0.0	0.0	0.0
0.0	0.0	0.0	0.0	0.0	0.0	0.0	0.0
0.0	0.0	0.0	0.0	0.0	0.0	0.0	0.0
0.0	0.0	0.0	0.0	0.0	0.0	0.0	0.0
0.0	0.0	0.0	0.0	0.0	0.0	0.0	0.0
0.0	0.0	0.0	0.0	0.0	0.0	0.0	0.0
0.0	0.0	0.0	0.0	0.0	0.0	0.0	0.0
0.0	0.0	0.0	0.0	0.0	0.0	0.0	0.0
0.0	0.0	0.0	0.0	0.0	0.0	0.0	0.0
0.0	0.0	0.0	0.0	0.0	0.0	0.0	0.0
0.0	0.0	0.0	0.0	0.0	0.0	0.0	0.0
0.0	0.0	0.0	0.0	0.0	0.0	0.0	0.0
0.0	0.0	0.0	0.0	0.0	0.0	0.0	0.0
0.0	0.0	0.0	0.0	0.0	0.0	0.0	0.0
0.0	0.0	0.0	0.0	0.0	0.0	0.0	0.0
0.0	0.0	0.0	0.0	0.0	0.0	0.0	0.0
0.0	0.0	0.0	0.0	0.0	0.0	0.0	0.0
0.0	0.0	0.0	0.0	0.0	0.0	0.0	0.0
0.0	0.0	0.0	0.0	0.0	0.0	0.0	0.0
0.0	0.0	0.0	0.0	0.0	0.0	0.0	0.0
0.0	0.0	0.0	0.0	0.0	0.0	0.0	0.0
0.0	0.0	0.0	0.0	0.0	0.0	0.0	0.0
0.0	0.0	0.0	0.0	0.0	0.0	0.0	0.0
0.0	0.0	0.0	0.0	0.0	0.0	0.0	0.0
0.0	0.0	0.0	0.0	0.0	0.0	0.0	0.0

0.0	0.0	0.0	0.0	0.0	0.0	0.0	0.0
0.0	0.0	0.0	0.0	0.0	0.0	0.0	0.0
0.0	0.0	0.0	0.0	0.0	0.0	0.0	0.0
0.0	0.0	0.0	0.0	0.0	0.0	0.0	0.0
0.0	0.0	0.0	0.0	0.0	0.0	0.0	0.0
0.0	0.0	0.0	0.0	0.0	0.0	0.0	0.0
0.0	0.0	0.0	0.0	0.0	0.0	0.0	0.0
0.0	0.0	0.0	0.0	0.0	0.0	0.0	0.0
0.0	0.0	0.0	0.0	0.0	0.0	0.0	0.0
0.0	0.0	0.0	0.0	0.0	0.0	0.0	0.0
0.0	0.0	0.0	0.0	0.0	0.0	0.0	0.0
0.0	0.0	0.0	0.0	0.0	0.0	0.0	0.0
0.0	0.0	0.0	0.0	0.0	0.0	0.0	0.0
0.0	0.0	0.0	0.0	0.0	0.0	0.0	0.0
0.0	0.0	0.0	0.0	0.0	0.0	0.0	0.0
0.0	0.0	0.0	0.0	0.0	0.0	0.0	0.0
0.0	0.0	0.0	0.0	0.0	0.0	0.0	0.0
0.0	0.0	0.0	0.0	0.0	0.0	0.0	0.0
0.0	0.0	0.0	0.0	0.0	0.0	0.0	0.0
0.0	0.0	0.0	0.0	0.0	0.0	0.0	0.0
0.0	0.0	0.0	0.0	0.0	0.0	0.0	0.0
0.0	0.0	0.0	0.0	0.0	0.0	0.0	0.0
0.0	0.0	0.0	0.0	0.0	0.0	0.0	0.0
0.0	0.0	0.0	0.0	0.0	0.0	0.0	0.0
0.0	0.0	0.0	0.0	0.0	0.0	0.0	0.0
0.0	0.0	0.0	0.0	0.0	0.0	0.0	0.0
0.0	0.0	0.0	0.0	0.0	0.0	0.0	0.0
0.0	0.0	0.0	0.0	0.0	0.0	0.0	0.0
0.0	0.0	0.0	0.0	0.0	0.0	0.0	0.0
0.0	0.0	0.0	0.0	0.0	0.0	0.0	0.0
0.0	0.0	0.0	0.0	0.0	0.0	0.0	0.0
0.0	0.0	0.0	0.0	0.0	0.0	0.0	0.0
0.0	0.0	0.0	0.0	0.0	0.0	0.0	0.0
0.0	0.0	0.0	0.0	0.0	0.0	0.0	0.0
0.0	0.0	0.0	0.0	0.0	0.0	0.0	0.0
0.0	0.0	0.0	0.0	0.0	0.0	0.0	0.0
0.0	0.0	0.0	0.0	0.0	0.0	0.0	0.0
0.0	0.0	0.0	0.0	0.0	0.0	0.0	0.0
0.0	0.0	0.0	0.0	0.0	0.0	0.0	0.0
0.0	0.0	0.0	0.0	0.0	0.0	0.0	0.0
0.0	0.0	0.0	0.0	0.0	0.0	0.0	0.0
0.0	0.0	0.0	0.0	0.0	0.0	0.0	0.0
0.0	0.0	0.0	0.0	0.0	0.0	0.0	0.0
0.0	0.0	0.0	0.0	0.0	0.0	0.0	0.0
0.0	0.0	0.0	0.0	0.0	0.0	0.0	0.0
0.0	0.0	0.0	0.0	0.0	0.0	0.0	0.0
0.0	0.0	0.0	0.0	0.0	0.0	0.0	0.0

0.0	0.0	0.0	0.0	0.0	0.0	0.0	0.0
0.0	0.0	0.0	0.0	0.0	0.0	0.0	0.0
0.0	0.0	0.0	0.0	0.0	0.0	0.0	0.0
0.0	0.0	0.0	0.0	0.0	0.0	0.0	0.0
0.0	0.0	0.0	0.0	0.0	0.0	0.0	0.0
0.0	0.0	0.0	0.0	0.0	0.0	0.0	0.0
0.0	0.0	0.0	0.0	0.0	0.0	0.0	0.0
0.0	0.0	0.0	0.0	0.0	0.0	0.0	0.0
0.0	0.0	0.0	0.0	0.0	0.0	0.0	0.0
0.0	0.0	0.0	0.0	0.0	0.0	0.0	0.0
0.0	0.0	0.0	0.0	0.0	0.0	0.0	0.0
0.0	0.0	0.0	0.0	0.0	0.0	0.0	0.0
0.0	0.0	0.0	0.0	0.0	0.0	0.0	0.0
0.0	0.0	0.0	0.0	0.0	0.0	0.0	0.0
0.0	0.0	0.0	0.0	0.0	0.0	0.0	0.0
0.0	0.0	0.0	0.0	0.0	0.0	0.0	0.0
0.0	0.0	0.0	0.0	0.0	0.0	0.0	0.0
0.0	0.0	0.0	0.0	0.0	0.0	0.0	0.0
0.0	0.0	0.0	0.0	0.0	0.0	0.0	0.0
0.0	0.0	0.0	0.0	0.0	0.0	0.0	0.0
0.0	0.0	0.0	0.0	0.0	0.0	0.0	0.0
0.0	0.0	0.0	0.0	0.0	0.0	0.0	0.0
0.0	0.0	0.0	0.0	0.0	0.0	0.0	0.0
0.0	0.0	0.0	0.0	0.0	0.0	0.0	0.0
0.0	0.0	0.0	0.0	0.0	0.0	0.0	0.0
0.0	0.0	0.0	0.0	0.0	0.0	0.0	0.0
0.0	0.0	0.0	0.0	0.0	0.0	0.0	0.0
0.0	0.0	0.0	0.0	0.0	0.0	0.0	0.0
0.0	0.0	0.0	0.0	0.0	0.0	0.0	0.0
0.0	0.0	0.0	0.0	0.0	0.0	0.0	0.0
0.0	0.0	0.0	0.0	0.0	0.0	0.0	0.0
0.0	0.0	0.0	0.0	0.0	0.0	0.0	0.0
0.0	0.0	0.0	0.0	0.0	0.0	0.0	0.0
0.0	0.0	0.0	0.0	0.0	0.0	0.0	0.0
0.0	0.0	0.0	0.0	0.0	0.0	0.0	0.0
0.0	0.0	0.0	0.0	0.0	0.0	0.0	0.0
0.0	0.0	0.0	0.0	0.0	0.0	0.0	0.0
0.0	0.0	0.0	0.0	0.0	0.0	0.0	0.0
0.0	0.0	0.0	0.0	0.0	0.0	0.0	0.0
0.0	0.0	0.0	0.0	0.0	0.0	0.0	0.0
0.0	0.0	0.0	0.0	0.0	0.0	0.0	0.0
0.0	0.0	0.0	0.0	0.0	0.0	0.0	0.0
0.0	0.0	0.0	0.0	0.0	0.0	0.0	0.0
0.0	0.0	0.0	0.0	0.0	0.0	0.0	0.0
0.0	0.0	0.0	0.0	0.0	0.0	0.0	0.0
0.0	0.0	0.0	0.0	0.0	0.0	0.0	0.0
0.0	0.0	0.0	0.0	0.0	0.0	0.0	0.0

0.0	0.0	0.0	0.0	0.0	0.0	0.0	0.0
0.0	0.0	0.0	0.0	0.0	0.0	0.0	0.0
0.0	0.0	0.0	0.0	0.0	0.0	0.0	0.0
0.0	0.0	0.0	0.0	0.0	0.0	0.0	0.0
0.0	0.0	0.0	0.0	0.0	0.0	0.0	0.0
0.0	0.0	0.0	0.0	0.0	0.0	0.0	0.0
0.0	0.0	0.0	0.0	0.0	0.0	0.0	0.0
0.0	0.0	0.0	0.0	0.0	0.0	0.0	0.0
0.0	0.0	0.0	0.0	0.0	0.0	0.0	0.0
0.0	0.0	0.0	0.0	0.0	0.0	0.0	0.0
0.0	0.0	0.0	0.0	0.0	0.0	0.0	0.0
0.0	0.0	0.0	0.0	0.0	0.0	0.0	0.0
0.0	0.0	0.0	0.0	0.0	0.0	0.0	0.0
0.0	0.0	0.0	0.0	0.0	0.0	0.0	0.0
0.0	0.0	0.0	0.0	0.0	0.0	0.0	0.0
0.0	0.0	0.0	0.0	0.0	0.0	0.0	0.0
0.0	0.0	0.0	0.0	0.0	0.0	0.0	0.0
0.0	0.0	0.0	0.0	0.0	0.0	0.0	0.0
0.0	0.0	0.0	0.0	0.0	0.0	0.0	0.0
0.0	0.0	0.0	0.0	0.0	0.0	0.0	0.0
0.0	0.0	0.0	0.0	0.0	0.0	0.0	0.0
0.0	0.0	0.0	0.0	0.0	0.0	0.0	0.0
0.0	0.0	0.0	0.0	0.0	0.0	0.0	0.0
0.0	0.0	0.0	0.0	0.0	0.0	0.0	0.0
0.0	0.0	0.0	0.0	0.0	0.0	0.0	0.0
0.0	0.0	0.0	0.0	0.0	0.0	0.0	0.0
0.0	0.0	0.0	0.0	0.0	0.0	0.0	0.0
0.0	0.0	0.0	0.0	0.0	0.0	0.0	0.0
0.0	0.0	0.0	0.0	0.0	0.0	0.0	0.0
0.0	0.0	0.0	0.0	0.0	0.0	0.0	0.0
0.0	0.0	0.0	0.0	0.0	0.0	0.0	0.0
0.0	0.0	0.0	0.0	0.0	0.0	0.0	0.0
0.0	0.0	0.0	0.0	0.0	0.0	0.0	0.0
0.0	0.0	0.0	0.0	0.0	0.0	0.0	0.0
0.0	0.0	0.0	0.0	0.0	0.0	0.0	0.0
0.0	0.0	0.0	0.0	0.0	0.0	0.0	0.0
0.0	0.0	0.0	0.0	0.0	0.0	0.0	0.0
0.0	0.0	0.0	0.0	0.0	0.0	0.0	0.0
0.0	0.0	0.0	0.0	0.0	0.0	0.0	0.0
0.0	0.0	0.0	0.0	0.0	0.0	0.0	0.0
0.0	0.0	0.0	0.0	0.0	0.0	0.0	0.0
0.0	0.0	0.0	0.0	0.0	0.0	0.0	0.0
0.0	0.0	0.0	0.0	0.0	0.0	0.0	0.0
0.0	0.0	0.0	0.0	0.0	0.0	0.0	0.0
0.0	0.0	0.0	0.0	0.0	0.0	0.0	0.0
0.0	0.0	0.0	0.0	0.0	0.0	0.0	0.0
0.0	0.0	0.0	0.0	0.0	0.0	0.0	0.0
0.0	0.0	0.0	0.0	0.0	0.0	0.0	0.0

0.0	0.0	0.0	0.0	0.0	0.0	0.0	0.0
0.0	0.0	0.0	0.0	0.0	0.0	0.0	0.0
0.0	0.0	0.0	0.0	0.0	0.0	0.0	0.0
0.0	0.0	0.0	0.0	0.0	0.0	0.0	0.0
0.0	0.0	0.0	0.0	0.0	0.0	0.0	0.0
0.0	0.0	0.0	0.0	0.0	0.0	0.0	0.0
0.0	0.0	0.0	0.0	0.0	0.0	0.0	0.0
0.0	0.0	0.0	0.0	0.0	0.0	0.0	0.0
0.0	0.0	0.0	0.0	0.0	0.0	0.0	0.0
0.0	0.0	0.0	0.0	0.0	0.0	0.0	0.0
0.0	0.0	0.0	0.0	0.0	0.0	0.0	0.0
0.0	0.0	0.0	0.0	0.0	0.0	0.0	0.0
0.0	0.0	0.0	0.0	0.0	0.0	0.0	0.0
0.0	0.0	0.0	0.0	0.0	0.0	0.0	0.0
0.0	0.0	0.0	0.0	0.0	0.0	0.0	0.0
0.0	0.0	0.0	0.0	0.0	0.0	0.0	0.0
0.0	0.0	0.0	0.0	0.0	0.0	0.0	0.0
0.0	0.0	0.0	0.0	0.0	0.0	0.0	0.0
0.0	0.0	0.0	0.0	0.0	0.0	0.0	0.0
0.0	0.0	0.0	0.0	0.0	0.0	0.0	0.0
0.0	0.0	0.0	0.0	0.0	0.0	0.0	0.0
0.0	0.0	0.0	0.0	0.0	0.0	0.0	0.0
0.0	0.0	0.0	0.0	0.0	0.0	0.0	0.0
0.0	0.0	0.0	0.0	0.0	0.0	0.0	0.0
0.0	0.0	0.0	0.0	0.0	0.0	0.0	0.0
0.0	0.0	0.0	0.0	0.0	0.0	0.0	0.0
0.0	0.0	0.0	0.0	0.0	0.0	0.0	0.0
0.0	0.0	0.0	0.0	0.0	0.0	0.0	0.0
0.0	0.0	0.0	0.0	0.0	0.0	0.0	0.0
0.0	0.0	0.0	0.0	0.0	0.0	0.0	0.0
0.0	0.0	0.0	0.0	0.0	0.0	0.0	0.0
0.0	0.0	0.0	0.0	0.0	0.0	0.0	0.0
0.0	0.0	0.0	0.0	0.0	0.0	0.0	0.0
0.0	0.0	0.0	0.0	0.0	0.0	0.0	0.0
0.0	0.0	0.0	0.0	0.0	0.0	0.0	0.0
0.0	0.0	0.0	0.0	0.0	0.0	0.0	0.0
0.0	0.0	0.0	0.0	0.0	0.0	0.0	0.0
0.0	0.0	0.0	0.0	0.0	0.0	0.0	0.0
0.0	0.0	0.0	0.0	0.0	0.0	0.0	0.0
0.0	0.0	0.0	0.0	0.0	0.0	0.0	0.0
0.0	0.0	0.0	0.0	0.0	0.0	0.0	0.0
0.0	0.0	0.0	0.0	0.0	0.0	0.0	0.0
0.0	0.0	0.0	0.0	0.0	0.0	0.0	0.0
0.0	0.0	0.0	0.0	0.0	0.0	0.0	0.0
0.0	0.0	0.0	0.0	0.0	0.0	0.0	0.0
0.0	0.0	0.0	0.0	0.0	0.0	0.0	0.0
0.0	0.0	0.0	0.0	0.0	0.0	0.0	0.0

0.0	0.0	0.0	0.0	0.0	0.0	0.0	0.0
0.0	0.0	0.0	0.0	0.0	0.0	0.0	0.0
0.0	0.0	0.0	0.0	0.0	0.0	0.0	0.0
0.0	0.0	0.0	0.0	0.0	0.0	0.0	0.0
0.0	0.0	0.0	0.0	0.0	0.0	0.0	0.0
0.0	0.0	0.0	0.0	0.0	0.0	0.0	0.0
0.0	0.0	0.0	0.0	0.0	0.0	0.0	0.0
0.0	0.0	0.0	0.0	0.0	0.0	0.0	0.0
0.0	0.0	0.0	0.0	0.0	0.0	0.0	0.0
0.0	0.0	0.0	0.0	0.0	0.0	0.0	0.0
0.0	0.0	0.0	0.0	0.0	0.0	0.0	0.0
0.0	0.0	0.0	0.0	0.0	0.0	0.0	0.0
0.0	0.0	0.0	0.0	0.0	0.0	0.0	0.0
0.0	0.0	0.0	0.0	0.0	0.0	0.0	0.0
0.0	0.0	0.0	0.0	0.0	0.0	0.0	0.0
0.0	0.0	0.0	0.0	0.0	0.0	0.0	0.0
0.0	0.0	0.0	0.0	0.0	0.0	0.0	0.0
0.0	0.0	0.0	0.0	0.0	0.0	0.0	0.0
0.0	0.0	0.0	0.0	0.0	0.0	0.0	0.0
0.0	0.0	0.0	0.0	0.0	0.0	0.0	0.0
0.0	0.0	0.0	0.0	0.0	0.0	0.0	0.0
0.0	0.0	0.0	0.0	0.0	0.0	0.0	0.0
0.0	0.0	0.0	0.0	0.0	0.0	0.0	0.0
0.0	0.0	0.0	0.0	0.0	0.0	0.0	0.0
0.0	0.0	0.0	0.0	0.0	0.0	0.0	0.0
0.0	0.0	0.0	0.0	0.0	0.0	0.0	0.0
0.0	0.0	0.0	0.0	0.0	0.0	0.0	0.0
0.0	0.0	0.0	0.0	0.0	0.0	0.0	0.0
0.0	0.0	0.0	0.0	0.0	0.0	0.0	0.0
0.0	0.0	0.0	0.0	0.0	0.0	0.0	0.0
0.0	0.0	0.0	0.0	0.0	0.0	0.0	0.0
0.0	0.0	0.0	0.0	0.0	0.0	0.0	0.0
0.0	0.0	0.0	0.0	0.0	0.0	0.0	0.0
0.0	0.0	0.0	0.0	0.0	0.0	0.0	0.0
0.0	0.0	0.0	0.0	0.0	0.0	0.0	0.0
0.0	0.0	0.0	0.0	0.0	0.0	0.0	0.0
0.0	0.0	0.0	0.0	0.0	0.0	0.0	0.0
0.0	0.0	0.0	0.0	0.0	0.0	0.0	0.0
0.0	0.0	0.0	0.0	0.0	0.0	0.0	0.0
0.0	0.0	0.0	0.0	0.0	0.0	0.0	0.0
0.0	0.0	0.0	0.0	0.0	0.0	0.0	0.0
0.0	0.0	0.0	0.0	0.0	0.0	0.0	0.0
0.0	0.0	0.0	0.0	0.0	0.0	0.0	0.0
0.0	0.0	0.0	0.0	0.0	0.0	0.0	0.0
0.0	0.0	0.0	0.0	0.0	0.0	0.0	0.0
0.0	0.0	0.0	0.0	0.0	0.0	0.0	0.0
0.0	0.0	0.0	0.0	0.0	0.0	0.0	0.0

0.0	0.0	0.0	0.0	0.0	0.0	0.0	0.0
0.0	0.0	0.0	0.0	0.0	0.0	0.0	0.0
0.0	0.0	0.0	0.0	0.0	0.0	0.0	0.0
0.0	0.0	0.0	0.0	0.0	0.0	0.0	0.0
0.0	0.0	0.0	0.0	0.0	0.0	0.0	0.0
0.0	0.0	0.0	0.0	0.0	0.0	0.0	0.0
0.0	0.0	0.0	0.0	0.0	0.0	0.0	0.0
0.0	0.0	0.0	0.0	0.0	0.0	0.0	0.0
0.0	0.0	0.0	0.0	0.0	0.0	0.0	0.0
0.0	0.0	0.0	0.0	0.0	0.0	0.0	0.0
0.0	0.0	0.0	0.0	0.0	0.0	0.0	0.0
0.0	0.0	0.0	0.0	0.0	0.0	0.0	0.0
0.0	0.0	0.0	0.0	0.0	0.0	0.0	0.0
0.0	0.0	0.0	0.0	0.0	0.0	0.0	0.0
0.0	0.0	0.0	0.0	0.0	0.0	0.0	0.0
0.0	0.0	0.0	0.0	0.0	0.0	0.0	0.0
0.0	0.0	0.0	0.0	0.0	0.0	0.0	0.0
0.0	0.0	0.0	0.0	0.0	0.0	0.0	0.0
0.0	0.0	0.0	0.0	0.0	0.0	0.0	0.0
0.0	0.0	0.0	0.0	0.0	0.0	0.0	0.0
0.0	0.0	0.0	0.0	0.0	0.0	0.0	0.0
0.0	0.0	0.0	0.0	0.0	0.0	0.0	0.0
0.0	0.0	0.0	0.0	0.0	0.0	0.0	0.0
0.0	0.0	0.0	0.0	0.0	0.0	0.0	0.0
0.0	0.0	0.0	0.0	0.0	0.0	0.0	0.0
0.0	0.0	0.0	0.0	0.0	0.0	0.0	0.0
0.0	0.0	0.0	0.0	0.0	0.0	0.0	0.0
0.0	0.0	0.0	0.0	0.0	0.0	0.0	0.0
0.0	0.0	0.0	0.0	0.0	0.0	0.0	0.0
0.0	0.0	0.0	0.0	0.0	0.0	0.0	0.0
0.0	0.0	0.0	0.0	0.0	0.0	0.0	0.0
0.0	0.0	0.0	0.0	0.0	0.0	0.0	0.0
0.0	0.0	0.0	0.0	0.0	0.0	0.0	0.0
0.0	0.0	0.0	0.0	0.0	0.0	0.0	0.0
0.0	0.0	0.0	0.0	0.0	0.0	0.0	0.0
0.0	0.0	0.0	0.0	0.0	0.0	0.0	0.0
0.0	0.0	0.0	0.0	0.0	0.0	0.0	0.0
0.0	0.0	0.0	0.0	0.0	0.0	0.0	0.0
0.0	0.0	0.0	0.0	0.0	0.0	0.0	0.0
0.0	0.0	0.0	0.0	0.0	0.0	0.0	0.0
0.0	0.0	0.0	0.0	0.0	0.0	0.0	0.0
0.0	0.0	0.0	0.0	0.0	0.0	0.0	0.0
0.0	0.0	0.0	0.0	0.0	0.0	0.0	0.0
0.0	0.0	0.0	0.0	0.0	0.0	0.0	0.0
0.0	0.0	0.0	0.0	0.0	0.0	0.0	0.0
0.0	0.0	0.0	0.0	0.0	0.0	0.0	0.0
0.0	0.0	0.0	0.0	0.0	0.0	0.0	0.0
0.0	0.0	0.0	0.0	0.0	0.0	0.0	0.0

0.0	0.0	0.0	0.0	0.0	0.0	0.0	0.0
0.0	0.0	0.0	0.0	0.0	0.0	0.0	0.0
0.0	0.0	0.0	0.0	0.0	0.0	0.0	0.0
0.0	0.0	0.0	0.0	0.0	0.0	0.0	0.0
0.0	0.0	0.0	0.0	0.0	0.0	0.0	0.0
0.0	0.0	0.0	0.0	0.0	0.0	0.0	0.0
0.0	0.0	0.0	0.0	0.0	0.0	0.0	0.0
0.0	0.0	0.0	0.0	0.0	0.0	0.0	0.0
0.0	0.0	0.0	0.0	0.0	0.0	0.0	0.0
0.0	0.0	0.0	0.0	0.0	0.0	0.0	0.0
0.0	0.0	0.0	0.0	0.0	0.0	0.0	0.0
0.0	0.0	0.0	0.0	0.0	0.0	0.0	0.0
0.0	0.0	0.0	0.0	0.0	0.0	0.0	0.0
0.0	0.0	0.0	0.0	0.0	0.0	0.0	0.0
0.0	0.0	0.0	0.0	0.0	0.0	0.0	0.0
0.0	0.0	0.0	0.0	0.0	0.0	0.0	0.0
0.0	0.0	0.0	0.0	0.0	0.0	0.0	0.0
0.0	0.0	0.0	0.0	0.0	0.0	0.0	0.0
0.0	0.0	0.0	0.0	0.0	0.0	0.0	0.0
0.0	0.0	0.0	0.0	0.0	0.0	0.0	0.0
0.0	0.0	0.0	0.0	0.0	0.0	0.0	0.0
0.0	0.0	0.0	0.0	0.0	0.0	0.0	0.0
0.0	0.0	0.0	0.0	0.0	0.0	0.0	0.0
0.0	0.0	0.0	0.0	0.0	0.0	0.0	0.0
0.0	0.0	0.0	0.0	0.0	0.0	0.0	0.0
0.0	0.0	0.0	0.0	0.0	0.0	0.0	0.0
0.0	0.0	0.0	0.0	0.0	0.0	0.0	0.0
0.0	0.0	0.0	0.0	0.0	0.0	0.0	0.0
0.0	0.0	0.0	0.0	0.0	0.0	0.0	0.0
0.0	0.0	0.0	0.0	0.0	0.0	0.0	0.0
0.0	0.0	0.0	0.0	0.0	0.0	0.0	0.0
0.0	0.0	0.0	0.0	0.0	0.0	0.0	0.0
0.0	0.0	0.0	0.0	0.0	0.0	0.0	0.0
0.0	0.0	0.0	0.0	0.0	0.0	0.0	0.0
0.0	0.0	0.0	0.0	0.0	0.0	0.0	0.0
0.0	0.0	0.0	0.0	0.0	0.0	0.0	0.0
0.0	0.0	0.0	0.0	0.0	0.0	0.0	0.0
0.0	0.0	0.0	0.0	0.0	0.0	0.0	0.0
0.0	0.0	0.0	0.0	0.0	0.0	0.0	0.0
0.0	0.0	0.0	0.0	0.0	0.0	0.0	0.0
0.0	0.0	0.0	0.0	0.0	0.0	0.0	0.0
0.0	0.0	0.0	0.0	0.0	0.0	0.0	0.0
0.0	0.0	0.0	0.0	0.0	0.0	0.0	0.0
0.0	0.0	0.0	0.0	0.0	0.0	0.0	0.0
0.0	0.0	0.0	0.0	0.0	0.0	0.0	0.0
0.0	0.0	0.0	0.0	0.0	0.0	0.0	0.0
0.0	0.0	0.0	0.0	0.0	0.0	0.0	0.0

0.0	0.0	0.0	0.0	0.0	0.0	0.0	0.0
0.0	0.0	0.0	0.0	0.0	0.0	0.0	0.0
0.0	0.0	0.0	0.0	0.0	0.0	0.0	0.0
0.0	0.0	0.0	0.0	0.0	0.0	0.0	0.0
0.0	0.0	0.0	0.0	0.0	0.0	0.0	0.0
0.0	0.0	0.0	0.0	0.0	0.0	0.0	0.0
0.0	0.0	0.0	0.0	0.0	0.0	0.0	0.0
0.0	0.0	0.0	0.0	0.0	0.0	0.0	0.0
0.0	0.0	0.0	0.0	0.0	0.0	0.0	0.0
0.0	0.0	0.0	0.0	0.0	0.0	0.0	0.0
0.0	0.0	0.0	0.0	0.0	0.0	0.0	0.0
0.0	0.0	0.0	0.0	0.0	0.0	0.0	0.0
0.0	0.0	0.0	0.0	0.0	0.0	0.0	0.0
0.0	0.0	0.0	0.0	0.0	0.0	0.0	0.0
0.0	0.0	0.0	0.0	0.0	0.0	0.0	0.0
0.0	0.0	0.0	0.0	0.0	0.0	0.0	0.0
0.0	0.0	0.0	0.0	0.0	0.0	0.0	0.0
0.0	0.0	0.0	0.0	0.0	0.0	0.0	0.0
0.0	0.0	0.0	0.0	0.0	0.0	0.0	0.0
0.0	0.0	0.0	0.0	0.0	0.0	0.0	0.0
0.0	0.0	0.0	0.0	0.0	0.0	0.0	0.0
0.0	0.0	0.0	0.0	0.0	0.0	0.0	0.0
0.0	0.0	0.0	0.0	0.0	0.0	0.0	0.0
0.0	0.0	0.0	0.0	0.0	0.0	0.0	0.0
0.0	0.0	0.0	0.0	0.0	0.0	0.0	0.0
0.0	0.0	0.0	0.0	0.0	0.0	0.0	0.0
0.0	0.0	0.0	0.0	0.0	0.0	0.0	0.0
0.0	0.0	0.0	0.0	0.0	0.0	0.0	0.0
0.0	0.0	0.0	0.0	0.0	0.0	0.0	0.0
0.0	0.0	0.0	0.0	0.0	0.0	0.0	0.0
0.0	0.0	0.0	0.0	0.0	0.0	0.0	0.0
0.0	0.0	0.0	0.0	0.0	0.0	0.0	0.0
0.0	0.0	0.0	0.0	0.0	0.0	0.0	0.0
0.0	0.0	0.0	0.0	0.0	0.0	0.0	0.0
0.0	0.0	0.0	0.0	0.0	0.0	0.0	0.0
0.0	0.0	0.0	0.0	0.0	0.0	0.0	0.0
0.0	0.0	0.0	0.0	0.0	0.0	0.0	0.0
0.0	0.0	0.0	0.0	0.0	0.0	0.0	0.0
0.0	0.0	0.0	0.0	0.0	0.0	0.0	0.0
0.0	0.0	0.0	0.0	0.0	0.0	0.0	0.0
0.0	0.0	0.0	0.0	0.0	0.0	0.0	0.0
0.0	0.0	0.0	0.0	0.0	0.0	0.0	0.0
0.0	0.0	0.0	0.0	0.0	0.0	0.0	0.0
0.0	0.0	0.0	0.0	0.0	0.0	0.0	0.0
0.0	0.0	0.0	0.0	0.0	0.0	0.0	0.0
0.0	0.0	0.0	0.0	0.0	0.0	0.0	0.0
0.0	0.0	0.0	0.0	0.0	0.0	0.0	0.0
0.0	0.0	0.0	0.0	0.0	0.0	0.0	0.0

0.0	0.0	0.0	0.0	0.0	0.0	0.0	0.0
0.0	0.0	0.0	0.0	0.0	0.0	0.0	0.0
0.0	0.0	0.0	0.0	0.0	0.0	0.0	0.0
0.0	0.0	0.0	0.0	0.0	0.0	0.0	0.0
0.0	0.0	0.0	0.0	0.0	0.0	0.0	0.0
0.0	0.0	0.0	0.0	0.0	0.0	0.0	0.0
0.0	0.0	0.0	0.0	0.0	0.0	0.0	0.0
0.0	0.0	0.0	0.0	0.0	0.0	0.0	0.0
0.0	0.0	0.0	0.0	0.0	0.0	0.0	0.0
0.0	0.0	0.0	0.0	0.0	0.0	0.0	0.0
0.0	0.0	0.0	0.0	0.0	0.0	0.0	0.0
0.0	0.0	0.0	0.0	0.0	0.0	0.0	0.0
0.0	0.0	0.0	0.0	0.0	0.0	0.0	0.0
0.0	0.0	0.0	0.0	0.0	0.0	0.0	0.0
0.0	0.0	0.0	0.0	0.0	0.0	0.0	0.0
0.0	0.0	0.0	0.0	0.0	0.0	0.0	0.0
0.0	0.0	0.0	0.0	0.0	0.0	0.0	0.0
0.0	0.0	0.0	0.0	0.0	0.0	0.0	0.0
0.0	0.0	0.0	0.0	0.0	0.0	0.0	0.0
0.0	0.0	0.0	0.0	0.0	0.0	0.0	0.0
0.0	0.0	0.0	0.0	0.0	0.0	0.0	0.0
0.0	0.0	0.0	0.0	0.0	0.0	0.0	0.0
0.0	0.0	0.0	0.0	0.0	0.0	0.0	0.0
0.0	0.0	0.0	0.0	0.0	0.0	0.0	0.0
0.0	0.0	0.0	0.0	0.0	0.0	0.0	0.0
0.0	0.0	0.0	0.0	0.0	0.0	0.0	0.0
0.0	0.0	0.0	0.0	0.0	0.0	0.0	0.0
0.0	0.0	0.0	0.0	0.0	0.0	0.0	0.0
0.0	0.0	0.0	0.0	0.0	0.0	0.0	0.0
0.0	0.0	0.0	0.0	0.0	0.0	0.0	0.0
0.0	0.0	0.0	0.0	0.0	0.0	0.0	0.0
0.0	0.0	0.0	0.0	0.0	0.0	0.0	0.0
0.0	0.0	0.0	0.0	0.0	0.0	0.0	0.0
0.0	0.0	0.0	0.0	0.0	0.0	0.0	0.0
0.0	0.0	0.0	0.0	0.0	0.0	0.0	0.0
0.0	0.0	0.0	0.0	0.0	0.0	0.0	0.0
0.0	0.0	0.0	0.0	0.0	0.0	0.0	0.0
0.0	0.0	0.0	0.0	0.0	0.0	0.0	0.0
0.0	0.0	0.0	0.0	0.0	0.0	0.0	0.0
0.0	0.0	0.0	0.0	0.0	0.0	0.0	0.0
0.0	0.0	0.0	0.0	0.0	0.0	0.0	0.0
0.0	0.0	0.0	0.0	0.0	0.0	0.0	0.0
0.0	0.0	0.0	0.0	0.0	0.0	0.0	0.0
0.0	0.0	0.0	0.0	0.0	0.0	0.0	0.0
0.0	0.0	0.0	0.0	0.0	0.0	0.0	0.0
0.0	0.0	0.0	0.0	0.0	0.0	0.0	0.0
0.0	0.0	0.0	0.0	0.0	0.0	0.0	0.0

0.0	0.0	0.0	0.0	0.0	0.0	0.0	0.0
0.0	0.0	0.0	0.0	0.0	0.0	0.0	0.0
0.0	0.0	0.0	0.0	0.0	0.0	0.0	0.0
0.0	0.0	0.0	0.0	0.0	0.0	0.0	0.0
0.0	0.0	0.0	0.0	0.0	0.0	0.0	0.0
0.0	0.0	0.0	0.0	0.0	0.0	0.0	0.0
0.0	0.0	0.0	0.0	0.0	0.0	0.0	0.0
0.0	0.0	0.0	0.0	0.0	0.0	0.0	0.0
0.0	0.0	0.0	0.0	0.0	0.0	0.0	0.0
0.0	0.0	0.0	0.0	0.0	0.0	0.0	0.0
0.0	0.0	0.0	0.0	0.0	0.0	0.0	0.0
0.0	0.0	0.0	0.0	0.0	0.0	0.0	0.0
0.0	0.0	0.0	0.0	0.0	0.0	0.0	0.0
0.0	0.0	0.0	0.0	0.0	0.0	0.0	0.0
0.0	0.0	0.0	0.0	0.0	0.0	0.0	0.0
0.0	0.0	0.0	0.0	0.0	0.0	0.0	0.0
0.0	0.0	0.0	0.0	0.0	0.0	0.0	0.0
0.0	0.0	0.0	0.0	0.0	0.0	0.0	0.0
0.0	0.0	0.0	0.0	0.0	0.0	0.0	0.0
0.0	0.0	0.0	0.0	0.0	0.0	0.0	0.0
0.0	0.0	0.0	0.0	0.0	0.0	0.0	0.0
0.0	0.0	0.0	0.0	0.0	0.0	0.0	0.0
0.0	0.0	0.0	0.0	0.0	0.0	0.0	0.0
0.0	0.0	0.0	0.0	0.0	0.0	0.0	0.0
0.0	0.0	0.0	0.0	0.0	0.0	0.0	0.0
0.0	0.0	0.0	0.0	0.0	0.0	0.0	0.0
0.0	0.0	0.0	0.0	0.0	0.0	0.0	0.0
0.0	0.0	0.0	0.0	0.0	0.0	0.0	0.0
0.0	0.0	0.0	0.0	0.0	0.0	0.0	0.0
0.0	0.0	0.0	0.0	0.0	0.0	0.0	0.0
0.0	0.0	0.0	0.0	0.0	0.0	0.0	0.0
0.0	0.0	0.0	0.0	0.0	0.0	0.0	0.0
0.0	0.0	0.0	0.0	0.0	0.0	0.0	0.0
0.0	0.0	0.0	0.0	0.0	0.0	0.0	0.0
0.0	0.0	0.0	0.0	0.0	0.0	0.0	0.0
0.0	0.0	0.0	0.0	0.0	0.0	0.0	0.0
0.0	0.0	0.0	0.0	0.0	0.0	0.0	0.0
0.0	0.0	0.0	0.0	0.0	0.0	0.0	0.0
0.0	0.0	0.0	0.0	0.0	0.0	0.0	0.0
0.0	0.0	0.0	0.0	0.0	0.0	0.0	0.0
0.0	0.0	0.0	0.0	0.0	0.0	0.0	0.0
0.0	0.0	0.0	0.0	0.0	0.0	0.0	0.0
0.0	0.0	0.0	0.0	0.0	0.0	0.0	0.0
0.0	0.0	0.0	0.0	0.0	0.0	0.0	0.0
0.0	0.0	0.0	0.0	0.0	0.0	0.0	0.0
0.0	0.0	0.0	0.0	0.0	0.0	0.0	0.0
0.0	0.0	0.0	0.0	0.0	0.0	0.0	0.0

0.0	0.0	0.0	0.0	0.0	0.0	0.0	0.0
0.0	0.0	0.0	0.0	0.0	0.0	0.0	0.0
0.0	0.0	0.0	0.0	0.0	0.0	0.0	0.0
0.0	0.0	0.0	0.0	0.0	0.0	0.0	0.0
0.0	0.0	0.0	0.0	0.0	0.0	0.0	0.0
0.0	0.0	0.0	0.0	0.0	0.0	0.0	0.0
0.0	0.0	0.0	0.0	0.0	0.0	0.0	0.0
0.0	0.0	0.0	0.0	0.0	0.0	0.0	0.0
0.0	0.0	0.0	0.0	0.0	0.0	0.0	0.0
0.0	0.0	0.0	0.0	0.0	0.0	0.0	0.0
0.0	0.0	0.0	0.0	0.0	0.0	0.0	0.0
0.0	0.0	0.0	0.0	0.0	0.0	0.0	0.0
0.0	0.0	0.0	0.0	0.0	0.0	0.0	0.0
0.0	0.0	0.0	0.0	0.0	0.0	0.0	0.0
0.0	0.0	0.0	0.0	0.0	0.0	0.0	0.0
0.0	0.0	0.0	0.0	0.0	0.0	0.0	0.0
0.0	0.0	0.0	0.0	0.0	0.0	0.0	0.0
0.0	0.0	0.0	0.0	0.0	0.0	0.0	0.0
0.0	0.0	0.0	0.0	0.0	0.0	0.0	0.0
0.0	0.0	0.0	0.0	0.0	0.0	0.0	0.0
0.0	0.0	0.0	0.0	0.0	0.0	0.0	0.0
0.0	0.0	0.0	0.0	0.0	0.0	0.0	0.0
0.0	0.0	0.0	0.0	0.0	0.0	0.0	0.0
0.0	0.0	0.0	0.0	0.0	0.0	0.0	0.0
0.0	0.0	0.0	0.0	0.0	0.0	0.0	0.0
0.0	0.0	0.0	0.0	0.0	0.0	0.0	0.0
0.0	0.0	0.0	0.0	0.0	0.0	0.0	0.0
0.0	0.0	0.0	0.0	0.0	0.0	0.0	0.0
0.0	0.0	0.0	0.0	0.0	0.0	0.0	0.0
0.0	0.0	0.0	0.0	0.0	0.0	0.0	0.0
0.0	0.0	0.0	0.0	0.0	0.0	0.0	0.0
0.0	0.0	0.0	0.0	0.0	0.0	0.0	0.0
0.0	0.0	0.0	0.0	0.0	0.0	0.0	0.0
0.0	0.0	0.0	0.0	0.0	0.0	0.0	0.0
0.0	0.0	0.0	0.0	0.0	0.0	0.0	0.0
0.0	0.0	0.0	0.0	0.0	0.0	0.0	0.0
0.0	0.0	0.0	0.0	0.0	0.0	0.0	0.0
0.0	0.0	0.0	0.0	0.0	0.0	0.0	0.0
0.0	0.0	0.0	0.0	0.0	0.0	0.0	0.0
0.0	0.0	0.0	0.0	0.0	0.0	0.0	0.0
0.0	0.0	0.0	0.0	0.0	0.0	0.0	0.0
0.0	0.0	0.0	0.0	0.0	0.0	0.0	0.0
0.0	0.0	0.0	0.0	0.0	0.0	0.0	0.0
0.0	0.0	0.0	0.0	0.0	0.0	0.0	0.0
0.0	0.0	0.0	0.0	0.0	0.0	0.0	0.0
0.0	0.0	0.0	0.0	0.0	0.0	0.0	0.0
0.0	0.0	0.0	0.0	0.0	0.0	0.0	0.0

0.0	0.0	0.0	0.0	0.0	0.0	0.0	0.0
0.0	0.0	0.0	0.0	0.0	0.0	0.0	0.0
0.0	0.0	0.0	0.0	0.0	0.0	0.0	0.0
0.0	0.0	0.0	0.0	0.0	0.0	0.0	0.0
0.0	0.0	0.0	0.0	0.0	0.0	0.0	0.0
0.0	0.0	0.0	0.0	0.0	0.0	0.0	0.0
0.0	0.0	0.0	0.0	0.0	0.0	0.0	0.0
0.0	0.0	0.0	0.0	0.0	0.0	0.0	0.0
0.0	0.0	0.0	0.0	0.0	0.0	0.0	0.0
0.0	0.0	0.0	0.0	0.0	0.0	0.0	0.0
0.0	0.0	0.0	0.0	0.0	0.0	0.0	0.0
0.0	0.0	0.0	0.0	0.0	0.0	0.0	0.0
0.0	0.0	0.0	0.0	0.0	0.0	0.0	0.0
0.0	0.0	0.0	0.0	0.0	0.0	0.0	0.0
0.0	0.0	0.0	0.0	0.0	0.0	0.0	0.0
0.0	0.0	0.0	0.0	0.0	0.0	0.0	0.0
0.0	0.0	0.0	0.0	0.0	0.0	0.0	0.0
0.0	0.0	0.0	0.0	0.0	0.0	0.0	0.0
0.0	0.0	0.0	0.0	0.0	0.0	0.0	0.0
0.0	0.0	0.0	0.0	0.0	0.0	0.0	0.0
0.0	0.0	0.0	0.0	0.0	0.0	0.0	0.0
0.0	0.0	0.0	0.0	0.0	0.0	0.0	0.0
0.0	0.0	0.0	0.0	0.0	0.0	0.0	0.0
0.0	0.0	0.0	0.0	0.0	0.0	0.0	0.0
0.0	0.0	0.0	0.0	0.0	0.0	0.0	0.0
0.0	0.0	0.0	0.0	0.0	0.0	0.0	0.0
0.0	0.0	0.0	0.0	0.0	0.0	0.0	0.0
0.0	0.0	0.0	0.0	0.0	0.0	0.0	0.0
0.0	0.0	0.0	0.0	0.0	0.0	0.0	0.0
0.0	0.0	0.0	0.0	0.0	0.0	0.0	0.0
0.0	0.0	0.0	0.0	0.0	0.0	0.0	0.0
0.0	0.0	0.0	0.0	0.0	0.0	0.0	0.0
0.0	0.0	0.0	0.0	0.0	0.0	0.0	0.0
0.0	0.0	0.0	0.0	0.0	0.0	0.0	0.0
0.0	0.0	0.0	0.0	0.0	0.0	0.0	0.0
0.0	0.0	0.0	0.0	0.0	0.0	0.0	0.0
0.0	0.0	0.0	0.0	0.0	0.0	0.0	0.0
0.0	0.0	0.0	0.0	0.0	0.0	0.0	0.0
0.0	0.0	0.0	0.0	0.0	0.0	0.0	0.0
0.0	0.0	0.0	0.0	0.0	0.0	0.0	0.0
0.0	0.0	0.0	0.0	0.0	0.0	0.0	0.0
0.0	0.0	0.0	0.0	0.0	0.0	0.0	0.0
0.0	0.0	0.0	0.0	0.0	0.0	0.0	0.0
0.0	0.0	0.0	0.0	0.0	0.0	0.0	0.0
0.0	0.0	0.0	0.0	0.0	0.0	0.0	0.0
0.0	0.0	0.0	0.0	0.0	0.0	0.0	0.0
0.0	0.0	0.0	0.0	0.0	0.0	0.0	0.0

0.0	0.0	0.0	0.0	0.0	0.0	0.0	0.0
0.0	0.0	0.0	0.0	0.0	0.0	0.0	0.0
0.0	0.0	0.0	0.0	0.0	0.0	0.0	0.0
0.0	0.0	0.0	0.0	0.0	0.0	0.0	0.0
0.0	0.0	0.0	0.0	0.0	0.0	0.0	0.0
0.0	0.0	0.0	0.0	0.0	0.0	0.0	0.0
0.0	0.0	0.0	0.0	0.0	0.0	0.0	0.0
0.0	0.0	0.0	0.0	0.0	0.0	0.0	0.0
0.0	0.0	0.0	0.0	0.0	0.0	0.0	0.0
0.0	0.0	0.0	0.0	0.0	0.0	0.0	0.0
0.0	0.0	0.0	0.0	0.0	0.0	0.0	0.0
0.0	0.0	0.0	0.0	0.0	0.0	0.0	0.0
0.0	0.0	0.0	0.0	0.0	0.0	0.0	0.0
0.0	0.0	0.0	0.0	0.0	0.0	0.0	0.0
0.0	0.0	0.0	0.0	0.0	0.0	0.0	0.0
0.0	0.0	0.0	0.0	0.0	0.0	0.0	0.0
0.0	0.0	0.0	0.0	0.0	0.0	0.0	0.0
0.0	0.0	0.0	0.0	0.0	0.0	0.0	0.0
0.0	0.0	0.0	0.0	0.0	0.0	0.0	0.0
0.0	0.0	0.0	0.0	0.0	0.0	0.0	0.0
0.0	0.0	0.0	0.0	0.0	0.0	0.0	0.0
0.0	0.0	0.0	0.0	0.0	0.0	0.0	0.0
0.0	0.0	0.0	0.0	0.0	0.0	0.0	0.0
0.0	0.0	0.0	0.0	0.0	0.0	0.0	0.0
0.0	0.0	0.0	0.0	0.0	0.0	0.0	0.0
0.0	0.0	0.0	0.0	0.0	0.0	0.0	0.0
0.0	0.0	0.0	0.0	0.0	0.0	0.0	0.0
0.0	0.0	0.0	0.0	0.0	0.0	0.0	0.0
0.0	0.0	0.0	0.0	0.0	0.0	0.0	0.0
0.0	0.0	0.0	0.0	0.0	0.0	0.0	0.0
0.0	0.0	0.0	0.0	0.0	0.0	0.0	0.0
0.0	0.0	0.0	0.0	0.0	0.0	0.0	0.0
0.0	0.0	0.0	0.0	0.0	0.0	0.0	0.0
0.0	0.0	0.0	0.0	0.0	0.0	0.0	0.0
0.0	0.0	0.0	0.0	0.0	0.0	0.0	0.0
0.0	0.0	0.0	0.0	0.0	0.0	0.0	0.0
0.0	0.0	0.0	0.0	0.0	0.0	0.0	0.0
0.0	0.0	0.0	0.0	0.0	0.0	0.0	0.0
0.0	0.0	0.0	0.0	0.0	0.0	0.0	0.0
0.0	0.0	0.0	0.0	0.0	0.0	0.0	0.0
0.0	0.0	0.0	0.0	0.0	0.0	0.0	0.0
0.0	0.0	0.0	0.0	0.0	0.0	0.0	0.0
0.0	0.0	0.0	0.0	0.0	0.0	0.0	0.0
0.0	0.0	0.0	0.0	0.0	0.0	0.0	0.0
0.0	0.0	0.0	0.0	0.0	0.0	0.0	0.0
0.0	0.0	0.0	0.0	0.0	0.0	0.0	0.0
0.0	0.0	0.0	0.0	0.0	0.0	0.0	0.0

0.0	0.0	0.0	0.0	0.0	0.0	0.0	0.0
0.0	0.0	0.0	0.0	0.0	0.0	0.0	0.0
0.0	0.0	0.0	0.0	0.0	0.0	0.0	0.0
0.0	0.0	0.0	0.0	0.0	0.0	0.0	0.0
0.0	0.0	0.0	0.0	0.0	0.0	0.0	0.0
0.0	0.0	0.0	0.0	0.0	0.0	0.0	0.0
0.0	0.0	0.0	0.0	0.0	0.0	0.0	0.0
0.0	0.0	0.0	0.0	0.0	0.0	0.0	0.0
0.0	0.0	0.0	0.0	0.0	0.0	0.0	0.0
0.0	0.0	0.0	0.0	0.0	0.0	0.0	0.0
0.0	0.0	0.0	0.0	0.0	0.0	0.0	0.0
0.0	0.0	0.0	0.0	0.0	0.0	0.0	0.0
0.0	0.0	0.0	0.0	0.0	0.0	0.0	0.0
0.0	0.0	0.0	0.0	0.0	0.0	0.0	0.0
0.0	0.0	0.0	0.0	0.0	0.0	0.0	0.0
0.0	0.0	0.0	0.0	0.0	0.0	0.0	0.0
0.0	0.0	0.0	0.0	0.0	0.0	0.0	0.0
0.0	0.0	0.0	0.0	0.0	0.0	0.0	0.0
0.0	0.0	0.0	0.0	0.0	0.0	0.0	0.0
0.0	0.0	0.0	0.0	0.0	0.0	0.0	0.0
0.0	0.0	0.0	0.0	0.0	0.0	0.0	0.0
0.0	0.0	0.0	0.0	0.0	0.0	0.0	0.0
0.0	0.0	0.0	0.0	0.0	0.0	0.0	0.0
0.0	0.0	0.0	0.0	0.0	0.0	0.0	0.0
0.0	0.0	0.0	0.0	0.0	0.0	0.0	0.0
0.0	0.0	0.0	0.0	0.0	0.0	0.0	0.0
0.0	0.0	0.0	0.0	0.0	0.0	0.0	0.0
0.0	0.0	0.0	0.0	0.0	0.0	0.0	0.0
0.0	0.0	0.0	0.0	0.0	0.0	0.0	0.0
0.0	0.0	0.0	0.0	0.0	0.0	0.0	0.0
0.0	0.0	0.0	0.0	0.0	0.0	0.0	0.0
0.0	0.0	0.0	0.0	0.0	0.0	0.0	0.0
0.0	0.0	0.0	0.0	0.0	0.0	0.0	0.0
0.0	0.0	0.0	0.0	0.0	0.0	0.0	0.0
0.0	0.0	0.0	0.0	0.0	0.0	0.0	0.0
0.0	0.0	0.0	0.0	0.0	0.0	0.0	0.0
0.0	0.0	0.0	0.0	0.0	0.0	0.0	0.0
0.0	0.0	0.0	0.0	0.0	0.0	0.0	0.0
0.0	0.0	0.0	0.0	0.0	0.0	0.0	0.0
0.0	0.0	0.0	0.0	0.0	0.0	0.0	0.0
0.0	0.0	0.0	0.0	0.0	0.0	0.0	0.0
0.0	0.0	0.0	0.0	0.0	0.0	0.0	0.0
0.0	0.0	0.0	0.0	0.0	0.0	0.0	0.0
0.0	0.0	0.0	0.0	0.0	0.0	0.0	0.0
0.0	0.0	0.0	0.0	0.0	0.0	0.0	0.0
0.0	0.0	0.0	0.0	0.0	0.0	0.0	0.0
0.0	0.0	0.0	0.0	0.0	0.0	0.0	0.0

0.0	0.0	0.0	0.0	0.0	0.0	0.0	0.0
0.0	0.0	0.0	0.0	0.0	0.0	0.0	0.0
0.0	0.0	0.0	0.0	0.0	0.0	0.0	0.0
0.0	0.0	0.0	0.0	0.0	0.0	0.0	0.0
0.0	0.0	0.0	0.0	0.0	0.0	0.0	0.0
0.0	0.0	0.0	0.0	0.0	0.0	0.0	0.0
0.0	0.0	0.0	0.0	0.0	0.0	0.0	0.0
0.0	0.0	0.0	0.0	0.0	0.0	0.0	0.0
0.0	0.0	0.0	0.0	0.0	0.0	0.0	0.0
0.0	0.0	0.0	0.0	0.0	0.0	0.0	0.0
0.0	0.0	0.0	0.0	0.0	0.0	0.0	0.0
0.0	0.0	0.0	0.0	0.0	0.0	0.0	0.0
0.0	0.0	0.0	0.0	0.0	0.0	0.0	0.0
0.0	0.0	0.0	0.0	0.0	0.0	0.0	0.0
0.0	0.0	0.0	0.0	0.0	0.0	0.0	0.0
0.0	0.0	0.0	0.0	0.0	0.0	0.0	0.0
0.0	0.0	0.0	0.0	0.0	0.0	0.0	0.0
0.0	0.0	0.0	0.0	0.0	0.0	0.0	0.0
0.0	0.0	0.0	0.0	0.0	0.0	0.0	0.0
0.0	0.0	0.0	0.0	0.0	0.0	0.0	0.0
0.0	0.0	0.0	0.0	0.0	0.0	0.0	0.0
0.0	0.0	0.0	0.0	0.0	0.0	0.0	0.0
0.0	0.0	0.0	0.0	0.0	0.0	0.0	0.0
0.0	0.0	0.0	0.0	0.0	0.0	0.0	0.0
0.0	0.0	0.0	0.0	0.0	0.0	0.0	0.0
0.0	0.0	0.0	0.0	0.0	0.0	0.0	0.0
0.0	0.0	0.0	0.0	0.0	0.0	0.0	0.0
0.0	0.0	0.0	0.0	0.0	0.0	0.0	0.0
0.0	0.0	0.0	0.0	0.0	0.0	0.0	0.0
0.0	0.0	0.0	0.0	0.0	0.0	0.0	0.0
0.0	0.0	0.0	0.0	0.0	0.0	0.0	0.0
0.0	0.0	0.0	0.0	0.0	0.0	0.0	0.0
0.0	0.0	0.0	0.0	0.0	0.0	0.0	0.0
0.0	0.0	0.0	0.0	0.0	0.0	0.0	0.0
0.0	0.0	0.0	0.0	0.0	0.0	0.0	0.0
0.0	0.0	0.0	0.0	0.0	0.0	0.0	0.0
0.0	0.0	0.0	0.0	0.0	0.0	0.0	0.0
0.0	0.0	0.0	0.0	0.0	0.0	0.0	0.0
0.0	0.0	0.0	0.0	0.0	0.0	0.0	0.0
0.0	0.0	0.0	0.0	0.0	0.0	0.0	0.0
0.0	0.0	0.0	0.0	0.0	0.0	0.0	0.0
0.0	0.0	0.0	0.0	0.0	0.0	0.0	0.0
0.0	0.0	0.0	0.0	0.0	0.0	0.0	0.0
0.0	0.0	0.0	0.0	0.0	0.0	0.0	0.0
0.0	0.0	0.0	0.0	0.0	0.0	0.0	0.0
0.0	0.0	0.0	0.0	0.0	0.0	0.0	0.0
0.0	0.0	0.0	0.0	0.0	0.0	0.0	0.0

0.0	0.0	0.0	0.0	0.0	0.0	0.0	0.0
0.0	0.0	0.0	0.0	0.0	0.0	0.0	0.0
0.0	0.0	0.0	0.0	0.0	0.0	0.0	0.0
0.0	0.0	0.0	0.0	0.0	0.0	0.0	0.0
0.0	0.0	0.0	0.0	0.0	0.0	0.0	0.0
0.0	0.0	0.0	0.0	0.0	0.0	0.0	0.0
0.0	0.0	0.0	0.0	0.0	0.0	0.0	0.0
0.0	0.0	0.0	0.0	0.0	0.0	0.0	0.0
0.0	0.0	0.0	0.0	0.0	0.0	0.0	0.0
0.0	0.0	0.0	0.0	0.0	0.0	0.0	0.0
0.0	0.0	0.0	0.0	0.0	0.0	0.0	0.0
0.0	0.0	0.0	0.0	0.0	0.0	0.0	0.0
0.0	0.0	0.0	0.0	0.0	0.0	0.0	0.0
0.0	0.0	0.0	0.0	0.0	0.0	0.0	0.0
0.0	0.0	0.0	0.0	0.0	0.0	0.0	0.0
0.0	0.0	0.0	0.0	0.0	0.0	0.0	0.0
0.0	0.0	0.0	0.0	0.0	0.0	0.0	0.0
0.0	0.0	0.0	0.0	0.0	0.0	0.0	0.0
0.0	0.0	0.0	0.0	0.0	0.0	0.0	0.0
0.0	0.0	0.0	0.0	0.0	0.0	0.0	0.0
0.0	0.0	0.0	0.0	0.0	0.0	0.0	0.0
0.0	0.0	0.0	0.0	0.0	0.0	0.0	0.0
0.0	0.0	0.0	0.0	0.0	0.0	0.0	0.0
0.0	0.0	0.0	0.0	0.0	0.0	0.0	0.0
0.0	0.0	0.0	0.0	0.0	0.0	0.0	0.0
0.0	0.0	0.0	0.0	0.0	0.0	0.0	0.0
0.0	0.0	0.0	0.0	0.0	0.0	0.0	0.0
0.0	0.0	0.0	0.0	0.0	0.0	0.0	0.0
0.0	0.0	0.0	0.0	0.0	0.0	0.0	0.0
0.0	0.0	0.0	0.0	0.0	0.0	0.0	0.0
0.0	0.0	0.0	0.0	0.0	0.0	0.0	0.0
0.0	0.0	0.0	0.0	0.0	0.0	0.0	0.0
0.0	0.0	0.0	0.0	0.0	0.0	0.0	0.0
0.0	0.0	0.0	0.0	0.0	0.0	0.0	0.0
0.0	0.0	0.0	0.0	0.0	0.0	0.0	0.0
0.0	0.0	0.0	0.0	0.0	0.0	0.0	0.0
0.0	0.0	0.0	0.0	0.0	0.0	0.0	0.0
0.0	0.0	0.0	0.0	0.0	0.0	0.0	0.0
0.0	0.0	0.0	0.0	0.0	0.0	0.0	0.0
0.0	0.0	0.0	0.0	0.0	0.0	0.0	0.0
0.0	0.0	0.0	0.0	0.0	0.0	0.0	0.0
0.0	0.0	0.0	0.0	0.0	0.0	0.0	0.0
0.0	0.0	0.0	0.0	0.0	0.0	0.0	0.0
0.0	0.0	0.0	0.0	0.0	0.0	0.0	0.0
0.0	0.0	0.0	0.0	0.0	0.0	0.0	0.0
0.0	0.0	0.0	0.0	0.0	0.0	0.0	0.0
0.0	0.0	0.0	0.0	0.0	0.0	0.0	0.0

0.0	0.0	0.0	0.0	0.0	0.0	0.0	0.0
0.0	0.0	0.0	0.0	0.0	0.0	0.0	0.0
0.0	0.0	0.0	0.0	0.0	0.0	0.0	0.0
0.0	0.0	0.0	0.0	0.0	0.0	0.0	0.0
0.0	0.0	0.0	0.0	0.0	0.0	0.0	0.0
0.0	0.0	0.0	0.0	0.0	0.0	0.0	0.0
0.0	0.0	0.0	0.0	0.0	0.0	0.0	0.0
0.0	0.0	0.0	0.0	0.0	0.0	0.0	0.0
0.0	0.0	0.0	0.0	0.0	0.0	0.0	0.0
0.0	0.0	0.0	0.0	0.0	0.0	0.0	0.0
0.0	0.0	0.0	0.0	0.0	0.0	0.0	0.0
0.0	0.0	0.0	0.0	0.0	0.0	0.0	0.0
0.0	0.0	0.0	0.0	0.0	0.0	0.0	0.0
0.0	0.0	0.0	0.0	0.0	0.0	0.0	0.0
0.0	0.0	0.0	0.0	0.0	0.0	0.0	0.0
0.0	0.0	0.0	0.0	0.0	0.0	0.0	0.0
0.0	0.0	0.0	0.0	0.0	0.0	0.0	0.0
0.0	0.0	0.0	0.0	0.0	0.0	0.0	0.0
0.0	0.0	0.0	0.0	0.0	0.0	0.0	0.0
0.0	0.0	0.0	0.0	0.0	0.0	0.0	0.0
0.0	0.0	0.0	0.0	0.0	0.0	0.0	0.0
0.0	0.0	0.0	0.0	0.0	0.0	0.0	0.0
0.0	0.0	0.0	0.0	0.0	0.0	0.0	0.0
0.0	0.0	0.0	0.0	0.0	0.0	0.0	0.0
0.0	0.0	0.0	0.0	0.0	0.0	0.0	0.0
0.0	0.0	0.0	0.0	0.0	0.0	0.0	0.0
0.0	0.0	0.0	0.0	0.0	0.0	0.0	0.0
0.0	0.0	0.0	0.0	0.0	0.0	0.0	0.0
0.0	0.0	0.0	0.0	0.0	0.0	0.0	0.0
0.0	0.0	0.0	0.0	0.0	0.0	0.0	0.0
0.0	0.0	0.0	0.0	0.0	0.0	0.0	0.0
0.0	0.0	0.0	0.0	0.0	0.0	0.0	0.0
0.0	0.0	0.0	0.0	0.0	0.0	0.0	0.0
0.0	0.0	0.0	0.0	0.0	0.0	0.0	0.0
0.0	0.0	0.0	0.0	0.0	0.0	0.0	0.0
0.0	0.0	0.0	0.0	0.0	0.0	0.0	0.0
0.0	0.0	0.0	0.0	0.0	0.0	0.0	0.0
0.0	0.0	0.0	0.0	0.0	0.0	0.0	0.0
0.0	0.0	0.0	0.0	0.0	0.0	0.0	0.0
0.0	0.0	0.0	0.0	0.0	0.0	0.0	0.0
0.0	0.0	0.0	0.0	0.0	0.0	0.0	0.0
0.0	0.0	0.0	0.0	0.0	0.0	0.0	0.0
0.0	0.0	0.0	0.0	0.0	0.0	0.0	0.0
0.0	0.0	0.0	0.0	0.0	0.0	0.0	0.0
0.0	0.0	0.0	0.0	0.0	0.0	0.0	0.0
0.0	0.0	0.0	0.0	0.0	0.0	0.0	0.0
0.0	0.0	0.0	0.0	0.0	0.0	0.0	0.0

0.0	0.0	0.0	0.0	0.0	0.0	0.0	0.0
0.0	0.0	0.0	0.0	0.0	0.0	0.0	0.0
0.0	0.0	0.0	0.0	0.0	0.0	0.0	0.0
0.0	0.0	0.0	0.0	0.0	0.0	0.0	0.0
0.0	0.0	0.0	0.0	0.0	0.0	0.0	0.0
0.0	0.0	0.0	0.0	0.0	0.0	0.0	0.0
0.0	0.0	0.0	0.0	0.0	0.0	0.0	0.0
0.0	0.0	0.0	0.0	0.0	0.0	0.0	0.0
0.0	0.0	0.0	0.0	0.0	0.0	0.0	0.0
0.0	0.0	0.0	0.0	0.0	0.0	0.0	0.0
0.0	0.0	0.0	0.0	0.0	0.0	0.0	0.0
0.0	0.0	0.0	0.0	0.0	0.0	0.0	0.0
0.0	0.0	0.0	0.0	0.0	0.0	0.0	0.0
0.0	0.0	0.0	0.0	0.0	0.0	0.0	0.0
0.0	0.0	0.0	0.0	0.0	0.0	0.0	0.0
0.0	0.0	0.0	0.0	0.0	0.0	0.0	0.0
0.0	0.0	0.0	0.0	0.0	0.0	0.0	0.0
0.0	0.0	0.0	0.0	0.0	0.0	0.0	0.0
0.0	0.0	0.0	0.0	0.0	0.0	0.0	0.0
0.0	0.0	0.0	0.0	0.0	0.0	0.0	0.0
0.0	0.0	0.0	0.0	0.0	0.0	0.0	0.0
0.0	0.0	0.0	0.0	0.0	0.0	0.0	0.0
0.0	0.0	0.0	0.0	0.0	0.0	0.0	0.0
0.0	0.0	0.0	0.0	0.0	0.0	0.0	0.0
0.0	0.0	0.0	0.0	0.0	0.0	0.0	0.0
0.0	0.0	0.0	0.0	0.0	0.0	0.0	0.0
0.0	0.0	0.0	0.0	0.0	0.0	0.0	0.0
0.0	0.0	0.0	0.0	0.0	0.0	0.0	0.0
0.0	0.0	0.0	0.0	0.0	0.0	0.0	0.0
0.0	0.0	0.0	0.0	0.0	0.0	0.0	0.0
0.0	0.0	0.0	0.0	0.0	0.0	0.0	0.0
0.0	0.0	0.0	0.0	0.0	0.0	0.0	0.0
0.0	0.0	0.0	0.0	0.0	0.0	0.0	0.0
0.0	0.0	0.0	0.0	0.0	0.0	0.0	0.0
0.0	0.0	0.0	0.0	0.0	0.0	0.0	0.0
0.0	0.0	0.0	0.0	0.0	0.0	0.0	0.0
0.0	0.0	0.0	0.0	0.0	0.0	0.0	0.0
0.0	0.0	0.0	0.0	0.0	0.0	0.0	0.0
0.0	0.0	0.0	0.0	0.0	0.0	0.0	0.0
0.0	0.0	0.0	0.0	0.0	0.0	0.0	0.0
0.0	0.0	0.0	0.0	0.0	0.0	0.0	0.0
0.0	0.0	0.0	0.0	0.0	0.0	0.0	0.0
0.0	0.0	0.0	0.0	0.0	0.0	0.0	0.0
0.0	0.0	0.0	0.0	0.0	0.0	0.0	0.0
0.0	0.0	0.0	0.0	0.0	0.0	0.0	0.0
0.0	0.0	0.0	0.0	0.0	0.0	0.0	0.0
0.0	0.0	0.0	0.0	0.0	0.0	0.0	0.0

0.0	0.0	0.0	0.0	0.0	0.0	0.0	0.0
0.0	0.0	0.0	0.0	0.0	0.0	0.0	0.0
0.0	0.0	0.0	0.0	0.0	0.0	0.0	0.0
0.0	0.0	0.0	0.0	0.0	0.0	0.0	0.0
0.0	0.0	0.0	0.0	0.0	0.0	0.0	0.0
0.0	0.0	0.0	0.0	0.0	0.0	0.0	0.0
0.0	0.0	0.0	0.0	0.0	0.0	0.0	0.0
0.0	0.0	0.0	0.0	0.0	0.0	0.0	0.0
0.0	0.0	0.0	0.0	0.0	0.0	0.0	0.0
0.0	0.0	0.0	0.0	0.0	0.0	0.0	0.0
0.0	0.0	0.0	0.0	0.0	0.0	0.0	0.0
0.0	0.0	0.0	0.0	0.0	0.0	0.0	0.0
0.0	0.0	0.0	0.0	0.0	0.0	0.0	0.0
0.0	0.0	0.0	0.0	0.0	0.0	0.0	0.0
0.0	0.0	0.0	0.0	0.0	0.0	0.0	0.0
0.0	0.0	0.0	0.0	0.0	0.0	0.0	0.0
0.0	0.0	0.0	0.0	0.0	0.0	0.0	0.0
0.0	0.0	0.0	0.0	0.0	0.0	0.0	0.0
0.0	0.0	0.0	0.0	0.0	0.0	0.0	0.0
0.0	0.0	0.0	0.0	0.0	0.0	0.0	0.0
0.0	0.0	0.0	0.0	0.0	0.0	0.0	0.0
0.0	0.0	0.0	0.0	0.0	0.0	0.0	0.0
0.0	0.0	0.0	0.0	0.0	0.0	0.0	0.0
0.0	0.0	0.0	0.0	0.0	0.0	0.0	0.0
0.0	0.0	0.0	0.0	0.0	0.0	0.0	0.0
0.0	0.0	0.0	0.0	0.0	0.0	0.0	0.0
0.0	0.0	0.0	0.0	0.0	0.0	0.0	0.0
0.0	0.0	0.0	0.0	0.0	0.0	0.0	0.0
0.0	0.0	0.0	0.0	0.0	0.0	0.0	0.0
0.0	0.0	0.0	0.0	0.0	0.0	0.0	0.0
0.0	0.0	0.0	0.0	0.0	0.0	0.0	0.0
0.0	0.0	0.0	0.0	0.0	0.0	0.0	0.0
0.0	0.0	0.0	0.0	0.0	0.0	0.0	0.0
0.0	0.0	0.0	0.0	0.0	0.0	0.0	0.0
0.0	0.0	0.0	0.0	0.0	0.0	0.0	0.0
0.0	0.0	0.0	0.0	0.0	0.0	0.0	0.0
0.0	0.0	0.0	0.0	0.0	0.0	0.0	0.0
0.0	0.0	0.0	0.0	0.0	0.0	0.0	0.0
0.0	0.0	0.0	0.0	0.0	0.0	0.0	0.0
0.0	0.0	0.0	0.0	0.0	0.0	0.0	0.0
0.0	0.0	0.0	0.0	0.0	0.0	0.0	0.0
0.0	0.0	0.0	0.0	0.0	0.0	0.0	0.0
0.0	0.0	0.0	0.0	0.0	0.0	0.0	0.0
0.0	0.0	0.0	0.0	0.0	0.0	0.0	0.0
0.0	0.0	0.0	0.0	0.0	0.0	0.0	0.0
0.0	0.0	0.0	0.0	0.0	0.0	0.0	0.0
0.0	0.0	0.0	0.0	0.0	0.0	0.0	0.0

0.0	0.0	0.0	0.0	0.0	0.0	0.0	0.0
0.0	0.0	0.0	0.0	0.0	0.0	0.0	0.0
0.0	0.0	0.0	0.0	0.0	0.0	0.0	0.0
0.0	0.0	0.0	0.0	0.0	0.0	0.0	0.0
0.0	0.0	0.0	0.0	0.0	0.0	0.0	0.0
0.0	0.0	0.0	0.0	0.0	0.0	0.0	0.0
0.0	0.0	0.0	0.0	0.0	0.0	0.0	0.0
0.0	0.0	0.0	0.0	0.0	0.0	0.0	0.0
0.0	0.0	0.0	0.0	0.0	0.0	0.0	0.0
0.0	0.0	0.0	0.0	0.0	0.0	0.0	0.0
0.0	0.0	0.0	0.0	0.0	0.0	0.0	0.0
0.0	0.0	0.0	0.0	0.0	0.0	0.0	0.0
0.0	0.0	0.0	0.0	0.0	0.0	0.0	0.0
0.0	0.0	0.0	0.0	0.0	0.0	0.0	0.0
0.0	0.0	0.0	0.0	0.0	0.0	0.0	0.0
0.0	0.0	0.0	0.0	0.0	0.0	0.0	0.0
0.0	0.0	0.0	0.0	0.0	0.0	0.0	0.0
0.0	0.0	0.0	0.0	0.0	0.0	0.0	0.0
0.0	0.0	0.0	0.0	0.0	0.0	0.0	0.0
0.0	0.0	0.0	0.0	0.0	0.0	0.0	0.0
0.0	0.0	0.0	0.0	0.0	0.0	0.0	0.0
0.0	0.0	0.0	0.0	0.0	0.0	0.0	0.0
0.0	0.0	0.0	0.0	0.0	0.0	0.0	0.0
0.0	0.0	0.0	0.0	0.0	0.0	0.0	0.0
0.0	0.0	0.0	0.0	0.0	0.0	0.0	0.0
0.0	0.0	0.0	0.0	0.0	0.0	0.0	0.0
0.0	0.0	0.0	0.0	0.0	0.0	0.0	0.0
0.0	0.0	0.0	0.0	0.0	0.0	0.0	0.0
0.0	0.0	0.0	0.0	0.0	0.0	0.0	0.0
0.0	0.0	0.0	0.0	0.0	0.0	0.0	0.0
0.0	0.0	0.0	0.0	0.0	0.0	0.0	0.0
0.0	0.0	0.0	0.0	0.0	0.0	0.0	0.0
0.0	0.0	0.0	0.0	0.0	0.0	0.0	0.0
0.0	0.0	0.0	0.0	0.0	0.0	0.0	0.0
0.0	0.0	0.0	0.0	0.0	0.0	0.0	0.0
0.0	0.0	0.0	0.0	0.0	0.0	0.0	0.0
0.0	0.0	0.0	0.0	0.0	0.0	0.0	0.0
0.0	0.0	0.0	0.0	0.0	0.0	0.0	0.0
0.0	0.0	0.0	0.0	0.0	0.0	0.0	0.0
0.0	0.0	0.0	0.0	0.0	0.0	0.0	0.0
0.0	0.0	0.0	0.0	0.0	0.0	0.0	0.0
0.0	0.0	0.0	0.0	0.0	0.0	0.0	0.0
0.0	0.0	0.0	0.0	0.0	0.0	0.0	0.0
0.0	0.0	0.0	0.0	0.0	0.0	0.0	0.0
0.0	0.0	0.0	0.0	0.0	0.0	0.0	0.0
0.0	0.0	0.0	0.0	0.0	0.0	0.0	0.0
0.0	0.0	0.0	0.0	0.0	0.0	0.0	0.0

0.0	0.0	0.0	0.0	0.0	0.0	0.0	0.0
0.0	0.0	0.0	0.0	0.0	0.0	0.0	0.0
0.0	0.0	0.0	0.0	0.0	0.0	0.0	0.0
0.0	0.0	0.0	0.0	0.0	0.0	0.0	0.0
0.0	0.0	0.0	0.0	0.0	0.0	0.0	0.0
0.0	0.0	0.0	0.0	0.0	0.0	0.0	0.0
0.0	0.0	0.0	0.0	0.0	0.0	0.0	0.0
0.0	0.0	0.0	0.0	0.0	0.0	0.0	0.0
0.0	0.0	0.0	0.0	0.0	0.0	0.0	0.0
0.0	0.0	0.0	0.0	0.0	0.0	0.0	0.0
0.0	0.0	0.0	0.0	0.0	0.0	0.0	0.0
0.0	0.0	0.0	0.0	0.0	0.0	0.0	0.0
0.0	0.0	0.0	0.0	0.0	0.0	0.0	0.0
0.0	0.0	0.0	0.0	0.0	0.0	0.0	0.0
0.0	0.0	0.0	0.0	0.0	0.0	0.0	0.0
0.0	0.0	0.0	0.0	0.0	0.0	0.0	0.0
0.0	0.0	0.0	0.0	0.0	0.0	0.0	0.0
0.0	0.0	0.0	0.0	0.0	0.0	0.0	0.0
0.0	0.0	0.0	0.0	0.0	0.0	0.0	0.0
0.0	0.0	0.0	0.0	0.0	0.0	0.0	0.0
0.0	0.0	0.0	0.0	0.0	0.0	0.0	0.0
0.0	0.0	0.0	0.0	0.0	0.0	0.0	0.0
0.0	0.0	0.0	0.0	0.0	0.0	0.0	0.0
0.0	0.0	0.0	0.0	0.0	0.0	0.0	0.0
0.0	0.0	0.0	0.0	0.0	0.0	0.0	0.0
0.0	0.0	0.0	0.0	0.0	0.0	0.0	0.0
0.0	0.0	0.0	0.0	0.0	0.0	0.0	0.0
0.0	0.0	0.0	0.0	0.0	0.0	0.0	0.0
0.0	0.0	0.0	0.0	0.0	0.0	0.0	0.0
0.0	0.0	0.0	0.0	0.0	0.0	0.0	0.0
0.0	0.0	0.0	0.0	0.0	0.0	0.0	0.0
0.0	0.0	0.0	0.0	0.0	0.0	0.0	0.0
0.0	0.0	0.0	0.0	0.0	0.0	0.0	0.0
0.0	0.0	0.0	0.0	0.0	0.0	0.0	0.0
0.0	0.0	0.0	0.0	0.0	0.0	0.0	0.0
0.0	0.0	0.0	0.0	0.0	0.0	0.0	0.0
0.0	0.0	0.0	0.0	0.0	0.0	0.0	0.0
0.0	0.0	0.0	0.0	0.0	0.0	0.0	0.0
0.0	0.0	0.0	0.0	0.0	0.0	0.0	0.0
0.0	0.0	0.0	0.0	0.0	0.0	0.0	0.0
0.0	0.0	0.0	0.0	0.0	0.0	0.0	0.0
0.0	0.0	0.0	0.0	0.0	0.0	0.0	0.0
0.0	0.0	0.0	0.0	0.0	0.0	0.0	0.0
0.0	0.0	0.0	0.0	0.0	0.0	0.0	0.0
0.0	0.0	0.0	0.0	0.0	0.0	0.0	0.0
0.0	0.0	0.0	0.0	0.0	0.0	0.0	0.0
0.0	0.0	0.0	0.0	0.0	0.0	0.0	0.0

0.0	0.0	0.0	0.0	0.0	0.0	0.0	0.0
0.0	0.0	0.0	0.0	0.0	0.0	0.0	0.0
0.0	0.0	0.0	0.0	0.0	0.0	0.0	0.0
0.0	0.0	0.0	0.0	0.0	0.0	0.0	0.0
0.0	0.0	0.0	0.0	0.0	0.0	0.0	0.0
0.0	0.0	0.0	0.0	0.0	0.0	0.0	0.0
0.0	0.0	0.0	0.0	0.0	0.0	0.0	0.0
0.0	0.0	0.0	0.0	0.0	0.0	0.0	0.0
0.0	0.0	0.0	0.0	0.0	0.0	0.0	0.0
0.0	0.0	0.0	0.0	0.0	0.0	0.0	0.0
0.0	0.0	0.0	0.0	0.0	0.0	0.0	0.0
0.0	0.0	0.0	0.0	0.0	0.0	0.0	0.0
0.0	0.0	0.0	0.0	0.0	0.0	0.0	0.0
0.0	0.0	0.0	0.0	0.0	0.0	0.0	0.0
0.0	0.0	0.0	0.0	0.0	0.0	0.0	0.0
0.0	0.0	0.0	0.0	0.0	0.0	0.0	0.0
0.0	0.0	0.0	0.0	0.0	0.0	0.0	0.0
0.0	0.0	0.0	0.0	0.0	0.0	0.0	0.0
0.0	0.0	0.0	0.0	0.0	0.0	0.0	0.0
0.0	0.0	0.0	0.0	0.0	0.0	0.0	0.0
0.0	0.0	0.0	0.0	0.0	0.0	0.0	0.0
0.0	0.0	0.0	0.0	0.0	0.0	0.0	0.0
0.0	0.0	0.0	0.0	0.0	0.0	0.0	0.0
0.0	0.0	0.0	0.0	0.0	0.0	0.0	0.0
0.0	0.0	0.0	0.0	0.0	0.0	0.0	0.0
0.0	0.0	0.0	0.0	0.0	0.0	0.0	0.0
0.0	0.0	0.0	0.0	0.0	0.0	0.0	0.0
0.0	0.0	0.0	0.0	0.0	0.0	0.0	0.0
0.0	0.0	0.0	0.0	0.0	0.0	0.0	0.0
0.0	0.0	0.0	0.0	0.0	0.0	0.0	0.0
0.0	0.0	0.0	0.0	0.0	0.0	0.0	0.0
0.0	0.0	0.0	0.0	0.0	0.0	0.0	0.0
0.0	0.0	0.0	0.0	0.0	0.0	0.0	0.0
0.0	0.0	0.0	0.0	0.0	0.0	0.0	0.0
0.0	0.0	0.0	0.0	0.0	0.0	0.0	0.0
0.0	0.0	0.0	0.0	0.0	0.0	0.0	0.0
0.0	0.0	0.0	0.0	0.0	0.0	0.0	0.0
0.0	0.0	0.0	0.0	0.0	0.0	0.0	0.0
0.0	0.0	0.0	0.0	0.0	0.0	0.0	0.0
0.0	0.0	0.0	0.0	0.0	0.0	0.0	0.0
0.0	0.0	0.0	0.0	0.0	0.0	0.0	0.0
0.0	0.0	0.0	0.0	0.0	0.0	0.0	0.0
0.0	0.0	0.0	0.0	0.0	0.0	0.0	0.0
0.0	0.0	0.0	0.0	0.0	0.0	0.0	0.0
0.0	0.0	0.0	0.0	0.0	0.0	0.0	0.0
0.0	0.0	0.0	0.0	0.0	0.0	0.0	0.0
0.0	0.0	0.0	0.0	0.0	0.0	0.0	0.0

0.0	0.0	0.0	0.0	0.0	0.0	0.0	0.0
0.0	0.0	0.0	0.0	0.0	0.0	0.0	0.0
0.0	0.0	0.0	0.0	0.0	0.0	0.0	0.0
0.0	0.0	0.0	0.0	0.0	0.0	0.0	0.0
0.0	0.0	0.0	0.0	0.0	0.0	0.0	0.0
0.0	0.0	0.0	0.0	0.0	0.0	0.0	0.0
0.0	0.0	0.0	0.0	0.0	0.0	0.0	0.0
0.0	0.0	0.0	0.0	0.0	0.0	0.0	0.0
0.0	0.0	0.0	0.0	0.0	0.0	0.0	0.0
0.0	0.0	0.0	0.0	0.0	0.0	0.0	0.0
0.0	0.0	0.0	0.0	0.0	0.0	0.0	0.0
0.0	0.0	0.0	0.0	0.0	0.0	0.0	0.0
0.0	0.0	0.0	0.0	0.0	0.0	0.0	0.0
0.0	0.0	0.0	0.0	0.0	0.0	0.0	0.0
0.0	0.0	0.0	0.0	0.0	0.0	0.0	0.0
0.0	0.0	0.0	0.0	0.0	0.0	0.0	0.0
0.0	0.0	0.0	0.0	0.0	0.0	0.0	0.0
0.0	0.0	0.0	0.0	0.0	0.0	0.0	0.0
0.0	0.0	0.0	0.0	0.0	0.0	0.0	0.0
0.0	0.0	0.0	0.0	0.0	0.0	0.0	0.0
0.0	0.0	0.0	0.0	0.0	0.0	0.0	0.0
0.0	0.0	0.0	0.0	0.0	0.0	0.0	0.0
0.0	0.0	0.0	0.0	0.0	0.0	0.0	0.0
0.0	0.0	0.0	0.0	0.0	0.0	0.0	0.0
0.0	0.0	0.0	0.0	0.0	0.0	0.0	0.0
0.0	0.0	0.0	0.0	0.0	0.0	0.0	0.0
0.0	0.0	0.0	0.0	0.0	0.0	0.0	0.0
0.0	0.0	0.0	0.0	0.0	0.0	0.0	0.0
0.0	0.0	0.0	0.0	0.0	0.0	0.0	0.0
0.0	0.0	0.0	0.0	0.0	0.0	0.0	0.0
0.0	0.0	0.0	0.0	0.0	0.0	0.0	0.0
0.0	0.0	0.0	0.0	0.0	0.0	0.0	0.0
0.0	0.0	0.0	0.0	0.0	0.0	0.0	0.0
0.0	0.0	0.0	0.0	0.0	0.0	0.0	0.0
0.0	0.0	0.0	0.0	0.0	0.0	0.0	0.0
0.0	0.0	0.0	0.0	0.0	0.0	0.0	0.0
0.0	0.0	0.0	0.0	0.0	0.0	0.0	0.0
0.0	0.0	0.0	0.0	0.0	0.0	0.0	0.0
0.0	0.0	0.0	0.0	0.0	0.0	0.0	0.0
0.0	0.0	0.0	0.0	0.0	0.0	0.0	0.0
0.0	0.0	0.0	0.0	0.0	0.0	0.0	0.0
0.0	0.0	0.0	0.0	0.0	0.0	0.0	0.0
0.0	0.0	0.0	0.0	0.0	0.0	0.0	0.0
0.0	0.0	0.0	0.0	0.0	0.0	0.0	0.0
0.0	0.0	0.0	0.0	0.0	0.0	0.0	0.0
0.0	0.0	0.0	0.0	0.0	0.0	0.0	0.0
0.0	0.0	0.0	0.0	0.0	0.0	0.0	0.0

0.0	0.0	0.0	0.0	0.0	0.0	0.0	0.0
0.0	0.0	0.0	0.0	0.0	0.0	0.0	0.0
0.0	0.0	0.0	0.0	0.0	0.0	0.0	0.0
0.0	0.0	0.0	0.0	0.0	0.0	0.0	0.0
0.0	0.0	0.0	0.0	0.0	0.0	0.0	0.0
0.0	0.0	0.0	0.0	0.0	0.0	0.0	0.0
0.0	0.0	0.0	0.0	0.0	0.0	0.0	0.0
0.0	0.0	0.0	0.0	0.0	0.0	0.0	0.0
0.0	0.0	0.0	0.0	0.0	0.0	0.0	0.0
0.0	0.0	0.0	0.0	0.0	0.0	0.0	0.0
0.0	0.0	0.0	0.0	0.0	0.0	0.0	0.0
0.0	0.0	0.0	0.0	0.0	0.0	0.0	0.0
0.0	0.0	0.0	0.0	0.0	0.0	0.0	0.0
0.0	0.0	0.0	0.0	0.0	0.0	0.0	0.0
0.0	0.0	0.0	0.0	0.0	0.0	0.0	0.0
0.0	0.0	0.0	0.0	0.0	0.0	0.0	0.0
0.0	0.0	0.0	0.0	0.0	0.0	0.0	0.0
0.0	0.0	0.0	0.0	0.0	0.0	0.0	0.0
0.0	0.0	0.0	0.0	0.0	0.0	0.0	0.0
0.0	0.0	0.0	0.0	0.0	0.0	0.0	0.0
0.0	0.0	0.0	0.0	0.0	0.0	0.0	0.0
0.0	0.0	0.0	0.0	0.0	0.0	0.0	0.0
0.0	0.0	0.0	0.0	0.0	0.0	0.0	0.0
0.0	0.0	0.0	0.0	0.0	0.0	0.0	0.0
0.0	0.0	0.0	0.0	0.0	0.0	0.0	0.0
0.0	0.0	0.0	0.0	0.0	0.0	0.0	0.0
0.0	0.0	0.0	0.0	0.0	0.0	0.0	0.0
0.0	0.0	0.0	0.0	0.0	0.0	0.0	0.0
0.0	0.0	0.0	0.0	0.0	0.0	0.0	0.0
0.0	0.0	0.0	0.0	0.0	0.0	0.0	0.0
0.0	0.0	0.0	0.0	0.0	0.0	0.0	0.0
0.0	0.0	0.0	0.0	0.0	0.0	0.0	0.0
0.0	0.0	0.0	0.0	0.0	0.0	0.0	0.0
0.0	0.0	0.0	0.0	0.0	0.0	0.0	0.0
0.0	0.0	0.0	0.0	0.0	0.0	0.0	0.0
0.0	0.0	0.0	0.0	0.0	0.0	0.0	0.0
0.0	0.0	0.0	0.0	0.0	0.0	0.0	0.0
0.0	0.0	0.0	0.0	0.0	0.0	0.0	0.0
0.0	0.0	0.0	0.0	0.0	0.0	0.0	0.0
0.0	0.0	0.0	0.0	0.0	0.0	0.0	0.0
0.0	0.0	0.0	0.0	0.0	0.0	0.0	0.0
0.0	0.0	0.0	0.0	0.0	0.0	0.0	0.0
0.0	0.0	0.0	0.0	0.0	0.0	0.0	0.0
0.0	0.0	0.0	0.0	0.0	0.0	0.0	0.0
0.0	0.0	0.0	0.0	0.0	0.0	0.0	0.0
0.0	0.0	0.0	0.0	0.0	0.0	0.0	0.0
0.0	0.0	0.0	0.0	0.0	0.0	0.0	0.0

0.0	0.0	0.0	0.0	0.0	0.0	0.0	0.0
0.0	0.0	0.0	0.0	0.0	0.0	0.0	0.0
0.0	0.0	0.0	0.0	0.0	0.0	0.0	0.0
0.0	0.0	0.0	0.0	0.0	0.0	0.0	0.0
0.0	0.0	0.0	0.0	0.0	0.0	0.0	0.0
0.0	0.0	0.0	0.0	0.0	0.0	0.0	0.0
0.0	0.0	0.0	0.0	0.0	0.0	0.0	0.0
0.0	0.0	0.0	0.0	0.0	0.0	0.0	0.0
0.0	0.0	0.0	0.0	0.0	0.0	0.0	0.0
0.0	0.0	0.0	0.0	0.0	0.0	0.0	0.0
0.0	0.0	0.0	0.0	0.0	0.0	0.0	0.0
0.0	0.0	0.0	0.0	0.0	0.0	0.0	0.0
0.0	0.0	0.0	0.0	0.0	0.0	0.0	0.0
0.0	0.0	0.0	0.0	0.0	0.0	0.0	0.0
0.0	0.0	0.0	0.0	0.0	0.0	0.0	0.0
0.0	0.0	0.0	0.0	0.0	0.0	0.0	0.0
0.0	0.0	0.0	0.0	0.0	0.0	0.0	0.0
0.0	0.0	0.0	0.0	0.0	0.0	0.0	0.0
0.0	0.0	0.0	0.0	0.0	0.0	0.0	0.0
0.0	0.0	0.0	0.0	0.0	0.0	0.0	0.0
0.0	0.0	0.0	0.0	0.0	0.0	0.0	0.0
0.0	0.0	0.0	0.0	0.0	0.0	0.0	0.0
0.0	0.0	0.0	0.0	0.0	0.0	0.0	0.0
0.0	0.0	0.0	0.0	0.0	0.0	0.0	0.0
0.0	0.0	0.0	0.0	0.0	0.0	0.0	0.0
0.0	0.0	0.0	0.0	0.0	0.0	0.0	0.0
0.0	0.0	0.0	0.0	0.0	0.0	0.0	0.0
0.0	0.0	0.0	0.0	0.0	0.0	0.0	0.0
0.0	0.0	0.0	0.0	0.0	0.0	0.0	0.0
0.0	0.0	0.0	0.0	0.0	0.0	0.0	0.0
0.0	0.0	0.0	0.0	0.0	0.0	0.0	0.0
0.0	0.0	0.0	0.0	0.0	0.0	0.0	0.0
0.0	0.0	0.0	0.0	0.0	0.0	0.0	0.0
0.0	0.0	0.0	0.0	0.0	0.0	0.0	0.0
0.0	0.0	0.0	0.0	0.0	0.0	0.0	0.0
0.0	0.0	0.0	0.0	0.0	0.0	0.0	0.0
0.0	0.0	0.0	0.0	0.0	0.0	0.0	0.0
0.0	0.0	0.0	0.0	0.0	0.0	0.0	0.0
0.0	0.0	0.0	0.0	0.0	0.0	0.0	0.0
0.0	0.0	0.0	0.0	0.0	0.0	0.0	0.0
0.0	0.0	0.0	0.0	0.0	0.0	0.0	0.0
0.0	0.0	0.0	0.0	0.0	0.0	0.0	0.0
0.0	0.0	0.0	0.0	0.0	0.0	0.0	0.0
0.0	0.0	0.0	0.0	0.0	0.0	0.0	0.0
0.0	0.0	0.0	0.0	0.0	0.0	0.0	0.0
0.0	0.0	0.0	0.0	0.0	0.0	0.0	0.0
0.0	0.0	0.0	0.0	0.0	0.0	0.0	0.0

0.0	0.0	0.0	0.0	0.0	0.0	0.0	0.0
0.0	0.0	0.0	0.0	0.0	0.0	0.0	0.0
0.0	0.0	0.0	0.0	0.0	0.0	0.0	0.0
0.0	0.0	0.0	0.0	0.0	0.0	0.0	0.0
0.0	0.0	0.0	0.0	0.0	0.0	0.0	0.0
0.0	0.0	0.0	0.0	0.0	0.0	0.0	0.0
0.0	0.0	0.0	0.0	0.0	0.0	0.0	0.0
0.0	0.0	0.0	0.0	0.0	0.0	0.0	0.0
0.0	0.0	0.0	0.0	0.0	0.0	0.0	0.0
0.0	0.0	0.0	0.0	0.0	0.0	0.0	0.0
0.0	0.0	0.0	0.0	0.0	0.0	0.0	0.0
0.0	0.0	0.0	0.0	0.0	0.0	0.0	0.0
0.0	0.0	0.0	0.0	0.0	0.0	0.0	0.0
0.0	0.0	0.0	0.0	0.0	0.0	0.0	0.0
0.0	0.0	0.0	0.0	0.0	0.0	0.0	0.0
0.0	0.0	0.0	0.0	0.0	0.0	0.0	0.0
0.0	0.0	0.0	0.0	0.0	0.0	0.0	0.0
0.0	0.0	0.0	0.0	0.0	0.0	0.0	0.0
0.0	0.0	0.0	0.0	0.0	0.0	0.0	0.0
0.0	0.0	0.0	0.0	0.0	0.0	0.0	0.0
0.0	0.0	0.0	0.0	0.0	0.0	0.0	0.0
0.0	0.0	0.0	0.0	0.0	0.0	0.0	0.0
0.0	0.0	0.0	0.0	0.0	0.0	0.0	0.0
0.0	0.0	0.0	0.0	0.0	0.0	0.0	0.0
0.0	0.0	0.0	0.0	0.0	0.0	0.0	0.0
0.0	0.0	0.0	0.0	0.0	0.0	0.0	0.0
0.0	0.0	0.0	0.0	0.0	0.0	0.0	0.0
0.0	0.0	0.0	0.0	0.0	0.0	0.0	0.0
0.0	0.0	0.0	0.0	0.0	0.0	0.0	0.0
0.0	0.0	0.0	0.0	0.0	0.0	0.0	0.0
0.0	0.0	0.0	0.0	0.0	0.0	0.0	0.0
0.0	0.0	0.0	0.0	0.0	0.0	0.0	0.0
0.0	0.0	0.0	0.0	0.0	0.0	0.0	0.0
0.0	0.0	0.0	0.0	0.0	0.0	0.0	0.0
0.0	0.0	0.0	0.0	0.0	0.0	0.0	0.0
0.0	0.0	0.0	0.0	0.0	0.0	0.0	0.0
0.0	0.0	0.0	0.0	0.0	0.0	0.0	0.0
0.0	0.0	0.0	0.0	0.0	0.0	0.0	0.0
0.0	0.0	0.0	0.0	0.0	0.0	0.0	0.0
0.0	0.0	0.0	0.0	0.0	0.0	0.0	0.0
0.0	0.0	0.0	0.0	0.0	0.0	0.0	0.0
0.0	0.0	0.0	0.0	0.0	0.0	0.0	0.0
0.0	0.0	0.0	0.0	0.0	0.0	0.0	0.0
0.0	0.0	0.0	0.0	0.0	0.0	0.0	0.0
0.0	0.0	0.0	0.0	0.0	0.0	0.0	0.0
0.0	0.0	0.0	0.0	0.0	0.0	0.0	0.0
0.0	0.0	0.0	0.0	0.0	0.0	0.0	0.0

0.0	0.0	0.0	0.0	0.0	0.0	0.0	0.0
0.0	0.0	0.0	0.0	0.0	0.0	0.0	0.0
0.0	0.0	0.0	0.0	0.0	0.0	0.0	0.0
0.0	0.0	0.0	0.0	0.0	0.0	0.0	0.0
0.0	0.0	0.0	0.0	0.0	0.0	0.0	0.0
0.0	0.0	0.0	0.0	0.0	0.0	0.0	0.0
0.0	0.0	0.0	0.0	0.0	0.0	0.0	0.0
0.0	0.0	0.0	0.0	0.0	0.0	0.0	0.0
0.0	0.0	0.0	0.0	0.0	0.0	0.0	0.0
0.0	0.0	0.0	0.0	0.0	0.0	0.0	0.0
0.0	0.0	0.0	0.0	0.0	0.0	0.0	0.0
0.0	0.0	0.0	0.0	0.0	0.0	0.0	0.0
0.0	0.0	0.0	0.0	0.0	0.0	0.0	0.0
0.0	0.0	0.0	0.0	0.0	0.0	0.0	0.0
0.0	0.0	0.0	0.0	0.0	0.0	0.0	0.0
0.0	0.0	0.0	0.0	0.0	0.0	0.0	0.0
0.0	0.0	0.0	0.0	0.0	0.0	0.0	0.0
0.0	0.0	0.0	0.0	0.0	0.0	0.0	0.0
0.0	0.0	0.0	0.0	0.0	0.0	0.0	0.0
0.0	0.0	0.0	0.0	0.0	0.0	0.0	0.0
0.0	0.0	0.0	0.0	0.0	0.0	0.0	0.0
0.0	0.0	0.0	0.0	0.0	0.0	0.0	0.0
0.0	0.0	0.0	0.0	0.0	0.0	0.0	0.0
0.0	0.0	0.0	0.0	0.0	0.0	0.0	0.0
0.0	0.0	0.0	0.0	0.0	0.0	0.0	0.0
0.0	0.0	0.0	0.0	0.0	0.0	0.0	0.0
0.0	0.0	0.0	0.0	0.0	0.0	0.0	0.0
0.0	0.0	0.0	0.0	0.0	0.0	0.0	0.0
0.0	0.0	0.0	0.0	0.0	0.0	0.0	0.0
0.0	0.0	0.0	0.0	0.0	0.0	0.0	0.0
0.0	0.0	0.0	0.0	0.0	0.0	0.0	0.0
0.0	0.0	0.0	0.0	0.0	0.0	0.0	0.0
0.0	0.0	0.0	0.0	0.0	0.0	0.0	0.0
0.0	0.0	0.0	0.0	0.0	0.0	0.0	0.0
0.0	0.0	0.0	0.0	0.0	0.0	0.0	0.0
0.0	0.0	0.0	0.0	0.0	0.0	0.0	0.0
0.0	0.0	0.0	0.0	0.0	0.0	0.0	0.0
0.0	0.0	0.0	0.0	0.0	0.0	0.0	0.0
0.0	0.0	0.0	0.0	0.0	0.0	0.0	0.0
0.0	0.0	0.0	0.0	0.0	0.0	0.0	0.0
0.0	0.0	0.0	0.0	0.0	0.0	0.0	0.0
0.0	0.0	0.0	0.0	0.0	0.0	0.0	0.0
0.0	0.0	0.0	0.0	0.0	0.0	0.0	0.0
0.0	0.0	0.0	0.0	0.0	0.0	0.0	0.0
0.0	0.0	0.0	0.0	0.0	0.0	0.0	0.0
0.0	0.0	0.0	0.0	0.0	0.0	0.0	0.0
0.0	0.0	0.0	0.0	0.0	0.0	0.0	0.0

0.0	0.0	0.0	0.0	0.0	0.0	0.0	0.0
0.0	0.0	0.0	0.0	0.0	0.0	0.0	0.0
0.0	0.0	0.0	0.0	0.0	0.0	0.0	0.0
0.0	0.0	0.0	0.0	0.0	0.0	0.0	0.0
0.0	0.0	0.0	0.0	0.0	0.0	0.0	0.0
0.0	0.0	0.0	0.0	0.0	0.0	0.0	0.0
0.0	0.0	0.0	0.0	0.0	0.0	0.0	0.0
0.0	0.0	0.0	0.0	0.0	0.0	0.0	0.0
0.0	0.0	0.0	0.0	0.0	0.0	0.0	0.0
0.0	0.0	0.0	0.0	0.0	0.0	0.0	0.0
0.0	0.0	0.0	0.0	0.0	0.0	0.0	0.0
0.0	0.0	0.0	0.0	0.0	0.0	0.0	0.0
0.0	0.0	0.0	0.0	0.0	0.0	0.0	0.0
0.0	0.0	0.0	0.0	0.0	0.0	0.0	0.0
0.0	0.0	0.0	0.0	0.0	0.0	0.0	0.0
0.0	0.0	0.0	0.0	0.0	0.0	0.0	0.0
0.0	0.0	0.0	0.0	0.0	0.0	0.0	0.0
0.0	0.0	0.0	0.0	0.0	0.0	0.0	0.0
0.0	0.0	0.0	0.0	0.0	0.0	0.0	0.0
0.0	0.0	0.0	0.0	0.0	0.0	0.0	0.0
0.0	0.0	0.0	0.0	0.0	0.0	0.0	0.0
0.0	0.0	0.0	0.0	0.0	0.0	0.0	0.0
0.0	0.0	0.0	0.0	0.0	0.0	0.0	0.0
0.0	0.0	0.0	0.0	0.0	0.0	0.0	0.0
0.0	0.0	0.0	0.0	0.0	0.0	0.0	0.0
0.0	0.0	0.0	0.0	0.0	0.0	0.0	0.0
0.0	0.0	0.0	0.0	0.0	0.0	0.0	0.0
0.0	0.0	0.0	0.0	0.0	0.0	0.0	0.0
0.0	0.0	0.0	0.0	0.0	0.0	0.0	0.0
0.0	0.0	0.0	0.0	0.0	0.0	0.0	0.0
0.0	0.0	0.0	0.0	0.0	0.0	0.0	0.0
0.0	0.0	0.0	0.0	0.0	0.0	0.0	0.0
0.0	0.0	0.0	0.0	0.0	0.0	0.0	0.0
0.0	0.0	0.0	0.0	0.0	0.0	0.0	0.0
0.0	0.0	0.0	0.0	0.0	0.0	0.0	0.0
0.0	0.0	0.0	0.0	0.0	0.0	0.0	0.0
0.0	0.0	0.0	0.0	0.0	0.0	0.0	0.0
0.0	0.0	0.0	0.0	0.0	0.0	0.0	0.0
0.0	0.0	0.0	0.0	0.0	0.0	0.0	0.0
0.0	0.0	0.0	0.0	0.0	0.0	0.0	0.0
0.0	0.0	0.0	0.0	0.0	0.0	0.0	0.0
0.0	0.0	0.0	0.0	0.0	0.0	0.0	0.0
0.0	0.0	0.0	0.0	0.0	0.0	0.0	0.0
0.0	0.0	0.0	0.0	0.0	0.0	0.0	0.0
0.0	0.0	0.0	0.0	0.0	0.0	0.0	0.0
0.0	0.0	0.0	0.0	0.0	0.0	0.0	0.0
0.0	0.0	0.0	0.0	0.0	0.0	0.0	0.0

0.0	0.0	0.0	0.0	0.0	0.0	0.0	0.0
0.0	0.0	0.0	0.0	0.0	0.0	0.0	0.0
0.0	0.0	0.0	0.0	0.0	0.0	0.0	0.0
0.0	0.0	0.0	0.0	0.0	0.0	0.0	0.0
0.0	0.0	0.0	0.0	0.0	0.0	0.0	0.0
0.0	0.0	0.0	0.0	0.0	0.0	0.0	0.0
0.0	0.0	0.0	0.0	0.0	0.0	0.0	0.0
0.0	0.0	0.0	0.0	0.0	0.0	0.0	0.0
0.0	0.0	0.0	0.0	0.0	0.0	0.0	0.0
0.0	0.0	0.0	0.0	0.0	0.0	0.0	0.0
0.0	0.0	0.0	0.0	0.0	0.0	0.0	0.0
0.0	0.0	0.0	0.0	0.0	0.0	0.0	0.0
0.0	0.0	0.0	0.0	0.0	0.0	0.0	0.0
0.0	0.0	0.0	0.0	0.0	0.0	0.0	0.0
0.0	0.0	0.0	0.0	0.0	0.0	0.0	0.0
0.0	0.0	0.0	0.0	0.0	0.0	0.0	0.0
0.0	0.0	0.0	0.0	0.0	0.0	0.0	0.0
0.0	0.0	0.0	0.0	0.0	0.0	0.0	0.0
0.0	0.0	0.0	0.0	0.0	0.0	0.0	0.0
0.0	0.0	0.0	0.0	0.0	0.0	0.0	0.0
0.0	0.0	0.0	0.0	0.0	0.0	0.0	0.0
0.0	0.0	0.0	0.0	0.0	0.0	0.0	0.0
0.0	0.0	0.0	0.0	0.0	0.0	0.0	0.0
0.0	0.0	0.0	0.0	0.0	0.0	0.0	0.0
0.0	0.0	0.0	0.0	0.0	0.0	0.0	0.0
0.0	0.0	0.0	0.0	0.0	0.0	0.0	0.0
0.0	0.0	0.0	0.0	0.0	0.0	0.0	0.0
0.0	0.0	0.0	0.0	0.0	0.0	0.0	0.0
0.0	0.0	0.0	0.0	0.0	0.0	0.0	0.0
0.0	0.0	0.0	0.0	0.0	0.0	0.0	0.0
0.0	0.0	0.0	0.0	0.0	0.0	0.0	0.0
0.0	0.0	0.0	0.0	0.0	0.0	0.0	0.0
0.0	0.0	0.0	0.0	0.0	0.0	0.0	0.0
0.0	0.0	0.0	0.0	0.0	0.0	0.0	0.0
0.0	0.0	0.0	0.0	0.0	0.0	0.0	0.0
0.0	0.0	0.0	0.0	0.0	0.0	0.0	0.0
0.0	0.0	0.0	0.0	0.0	0.0	0.0	0.0
0.0	0.0	0.0	0.0	0.0	0.0	0.0	0.0
0.0	0.0	0.0	0.0	0.0	0.0	0.0	0.0
0.0	0.0	0.0	0.0	0.0	0.0	0.0	0.0
0.0	0.0	0.0	0.0	0.0	0.0	0.0	0.0
0.0	0.0	0.0	0.0	0.0	0.0	0.0	0.0
0.0	0.0	0.0	0.0	0.0	0.0	0.0	0.0
0.0	0.0	0.0	0.0	0.0	0.0	0.0	0.0
0.0	0.0	0.0	0.0	0.0	0.0	0.0	0.0
0.0	0.0	0.0	0.0	0.0	0.0	0.0	0.0
0.0	0.0	0.0	0.0	0.0	0.0	0.0	0.0
0.0	0.0	0.0	0.0	0.0	0.0	0.0	0.0

0.0	0.0	0.0	0.0	0.0	0.0	0.0	0.0
0.0	0.0	0.0	0.0	0.0	0.0	0.0	0.0
0.0	0.0	0.0	0.0	0.0	0.0	0.0	0.0
0.0	0.0	0.0	0.0	0.0	0.0	0.0	0.0
0.0	0.0	0.0	0.0	0.0	0.0	0.0	0.0
0.0	0.0	0.0	0.0	0.0	0.0	0.0	0.0
0.0	0.0	0.0	0.0	0.0	0.0	0.0	0.0
0.0	0.0	0.0	0.0	0.0	0.0	0.0	0.0
0.0	0.0	0.0	0.0	0.0	0.0	0.0	0.0
0.0	0.0	0.0	0.0	0.0	0.0	0.0	0.0
0.0	0.0	0.0	0.0	0.0	0.0	0.0	0.0
0.0	0.0	0.0	0.0	0.0	0.0	0.0	0.0
0.0	0.0	0.0	0.0	0.0	0.0	0.0	0.0
0.0	0.0	0.0	0.0	0.0	0.0	0.0	0.0
0.0	0.0	0.0	0.0	0.0	0.0	0.0	0.0
0.0	0.0	0.0	0.0	0.0	0.0	0.0	0.0
0.0	0.0	0.0	0.0	0.0	0.0	0.0	0.0
0.0	0.0	0.0	0.0	0.0	0.0	0.0	0.0
0.0	0.0	0.0	0.0	0.0	0.0	0.0	0.0
0.0	0.0	0.0	0.0	0.0	0.0	0.0	0.0
0.0	0.0	0.0	0.0	0.0	0.0	0.0	0.0
0.0	0.0	0.0	0.0	0.0	0.0	0.0	0.0
0.0	0.0	0.0	0.0	0.0	0.0	0.0	0.0
0.0	0.0	0.0	0.0	0.0	0.0	0.0	0.0
0.0	0.0	0.0	0.0	0.0	0.0	0.0	0.0
0.0	0.0	0.0	0.0	0.0	0.0	0.0	0.0
0.0	0.0	0.0	0.0	0.0	0.0	0.0	0.0
0.0	0.0	0.0	0.0	0.0	0.0	0.0	0.0
0.0	0.0	0.0	0.0	0.0	0.0	0.0	0.0
0.0	0.0	0.0	0.0	0.0	0.0	0.0	0.0
0.0	0.0	0.0	0.0	0.0	0.0	0.0	0.0
0.0	0.0	0.0	0.0	0.0	0.0	0.0	0.0
0.0	0.0	0.0	0.0	0.0	0.0	0.0	0.0
0.0	0.0	0.0	0.0	0.0	0.0	0.0	0.0
0.0	0.0	0.0	0.0	0.0	0.0	0.0	0.0
0.0	0.0	0.0	0.0	0.0	0.0	0.0	0.0
0.0	0.0	0.0	0.0	0.0	0.0	0.0	0.0
0.0	0.0	0.0	0.0	0.0	0.0	0.0	0.0
0.0	0.0	0.0	0.0	0.0	0.0	0.0	0.0
0.0	0.0	0.0	0.0	0.0	0.0	0.0	0.0
0.0	0.0	0.0	0.0	0.0	0.0	0.0	0.0
0.0	0.0	0.0	0.0	0.0	0.0	0.0	0.0
0.0	0.0	0.0	0.0	0.0	0.0	0.0	0.0
0.0	0.0	0.0	0.0	0.0	0.0	0.0	0.0
0.0	0.0	0.0	0.0	0.0	0.0	0.0	0.0
0.0	0.0	0.0	0.0	0.0	0.0	0.0	0.0
0.0	0.0	0.0	0.0	0.0	0.0	0.0	0.0
0.0	0.0	0.0	0.0	0.0	0.0	0.0	0.0

0.0	0.0	0.0	0.0	0.0	0.0	0.0	0.0
0.0	0.0	0.0	0.0	0.0	0.0	0.0	0.0
0.0	0.0	0.0	0.0	0.0	0.0	0.0	0.0
0.0	0.0	0.0	0.0	0.0	0.0	0.0	0.0
0.0	0.0	0.0	0.0	0.0	0.0	0.0	0.0
0.0	0.0	0.0	0.0	0.0	0.0	0.0	0.0
0.0	0.0	0.0	0.0	0.0	0.0	0.0	0.0
0.0	0.0	0.0	0.0	0.0	0.0	0.0	0.0
0.0	0.0	0.0	0.0	0.0	0.0	0.0	0.0
0.0	0.0	0.0	0.0	0.0	0.0	0.0	0.0
0.0	0.0	0.0	0.0	0.0	0.0	0.0	0.0
0.0	0.0	0.0	0.0	0.0	0.0	0.0	0.0
0.0	0.0	0.0	0.0	0.0	0.0	0.0	0.0
0.0	0.0	0.0	0.0	0.0	0.0	0.0	0.0
0.0	0.0	0.0	0.0	0.0	0.0	0.0	0.0
0.0	0.0	0.0	0.0	0.0	0.0	0.0	0.0
0.0	0.0	0.0	0.0	0.0	0.0	0.0	0.0
0.0	0.0	0.0	0.0	0.0	0.0	0.0	0.0
0.0	0.0	0.0	0.0	0.0	0.0	0.0	0.0
0.0	0.0	0.0	0.0	0.0	0.0	0.0	0.0
0.0	0.0	0.0	0.0	0.0	0.0	0.0	0.0
0.0	0.0	0.0	0.0	0.0	0.0	0.0	0.0
0.0	0.0	0.0	0.0	0.0	0.0	0.0	0.0
0.0	0.0	0.0	0.0	0.0	0.0	0.0	0.0
0.0	0.0	0.0	0.0	0.0	0.0	0.0	0.0
0.0	0.0	0.0	0.0	0.0	0.0	0.0	0.0
0.0	0.0	0.0	0.0	0.0	0.0	0.0	0.0
0.0	0.0	0.0	0.0	0.0	0.0	0.0	0.0
0.0	0.0	0.0	0.0	0.0	0.0	0.0	0.0
0.0	0.0	0.0	0.0	0.0	0.0	0.0	0.0
0.0	0.0	0.0	0.0	0.0	0.0	0.0	0.0
0.0	0.0	0.0	0.0	0.0	0.0	0.0	0.0
0.0	0.0	0.0	0.0	0.0	0.0	0.0	0.0
0.0	0.0	0.0	0.0	0.0	0.0	0.0	0.0
0.0	0.0	0.0	0.0	0.0	0.0	0.0	0.0
0.0	0.0	0.0	0.0	0.0	0.0	0.0	0.0
0.0	0.0	0.0	0.0	0.0	0.0	0.0	0.0
0.0	0.0	0.0	0.0	0.0	0.0	0.0	0.0
0.0	0.0	0.0	0.0	0.0	0.0	0.0	0.0
0.0	0.0	0.0	0.0	0.0	0.0	0.0	0.0
0.0	0.0	0.0	0.0	0.0	0.0	0.0	0.0
0.0	0.0	0.0	0.0	0.0	0.0	0.0	0.0
0.0	0.0	0.0	0.0	0.0	0.0	0.0	0.0
0.0	0.0	0.0	0.0	0.0	0.0	0.0	0.0
0.0	0.0	0.0	0.0	0.0	0.0	0.0	0.0
0.0	0.0	0.0	0.0	0.0	0.0	0.0	0.0
0.0	0.0	0.0	0.0	0.0	0.0	0.0	0.0

0.0	0.0	0.0	0.0	0.0	0.0	0.0	0.0
0.0	0.0	0.0	0.0	0.0	0.0	0.0	0.0
0.0	0.0	0.0	0.0	0.0	0.0	0.0	0.0
0.0	0.0	0.0	0.0	0.0	0.0	0.0	0.0
0.0	0.0	0.0	0.0	0.0	0.0	0.0	0.0
0.0	0.0	0.0	0.0	0.0	0.0	0.0	0.0
0.0	0.0	0.0	0.0	0.0	0.0	0.0	0.0
0.0	0.0	0.0	0.0	0.0	0.0	0.0	0.0
0.0	0.0	0.0	0.0	0.0	0.0	0.0	0.0
0.0	0.0	0.0	0.0	0.0	0.0	0.0	0.0
0.0	0.0	0.0	0.0	0.0	0.0	0.0	0.0
0.0	0.0	0.0	0.0	0.0	0.0	0.0	0.0
0.0	0.0	0.0	0.0	0.0	0.0	0.0	0.0
0.0	0.0	0.0	0.0	0.0	0.0	0.0	0.0
0.0	0.0	0.0	0.0	0.0	0.0	0.0	0.0
0.0	0.0	0.0	0.0	0.0	0.0	0.0	0.0
0.0	0.0	0.0	0.0	0.0	0.0	0.0	0.0
0.0	0.0	0.0	0.0	0.0	0.0	0.0	0.0
0.0	0.0	0.0	0.0	0.0	0.0	0.0	0.0
0.0	0.0	0.0	0.0	0.0	0.0	0.0	0.0
0.0	0.0	0.0	0.0	0.0	0.0	0.0	0.0
0.0	0.0	0.0	0.0	0.0	0.0	0.0	0.0
0.0	0.0	0.0	0.0	0.0	0.0	0.0	0.0
0.0	0.0	0.0	0.0	0.0	0.0	0.0	0.0
0.0	0.0	0.0	0.0	0.0	0.0	0.0	0.0
0.0	0.0	0.0	0.0	0.0	0.0	0.0	0.0
0.0	0.0	0.0	0.0	0.0	0.0	0.0	0.0
0.0	0.0	0.0	0.0	0.0	0.0	0.0	0.0
0.0	0.0	0.0	0.0	0.0	0.0	0.0	0.0
0.0	0.0	0.0	0.0	0.0	0.0	0.0	0.0
0.0	0.0	0.0	0.0	0.0	0.0	0.0	0.0
0.0	0.0	0.0	0.0	0.0	0.0	0.0	0.0
0.0	0.0	0.0	0.0	0.0	0.0	0.0	0.0
0.0	0.0	0.0	0.0	0.0	0.0	0.0	0.0
0.0	0.0	0.0	0.0	0.0	0.0	0.0	0.0
0.0	0.0	0.0	0.0	0.0	0.0	0.0	0.0
0.0	0.0	0.0	0.0	0.0	0.0	0.0	0.0
0.0	0.0	0.0	0.0	0.0	0.0	0.0	0.0
0.0	0.0	0.0	0.0	0.0	0.0	0.0	0.0
0.0	0.0	0.0	0.0	0.0	0.0	0.0	0.0
0.0	0.0	0.0	0.0	0.0	0.0	0.0	0.0
0.0	0.0	0.0	0.0	0.0	0.0	0.0	0.0
0.0	0.0	0.0	0.0	0.0	0.0	0.0	0.0
0.0	0.0	0.0	0.0	0.0	0.0	0.0	0.0
0.0	0.0	0.0	0.0	0.0	0.0	0.0	0.0
0.0	0.0	0.0	0.0	0.0	0.0	0.0	0.0
0.0	0.0	0.0	0.0	0.0	0.0	0.0	0.0
0.0	0.0	0.0	0.0	0.0	0.0	0.0	0.0

0.0	0.0	0.0	0.0	0.0	0.0	0.0	0.0
0.0	0.0	0.0	0.0	0.0	0.0	0.0	0.0
0.0	0.0	0.0	0.0	0.0	0.0	0.0	0.0
0.0	0.0	0.0	0.0	0.0	0.0	0.0	0.0
0.0	0.0	0.0	0.0	0.0	0.0	0.0	0.0
0.0	0.0	0.0	0.0	0.0	0.0	0.0	0.0
0.0	0.0	0.0	0.0	0.0	0.0	0.0	0.0
0.0	0.0	0.0	0.0	0.0	0.0	0.0	0.0
0.0	0.0	0.0	0.0	0.0	0.0	0.0	0.0
0.0	0.0	0.0	0.0	0.0	0.0	0.0	0.0
0.0	0.0	0.0	0.0	0.0	0.0	0.0	0.0
0.0	0.0	0.0	0.0	0.0	0.0	0.0	0.0
0.0	0.0	0.0	0.0	0.0	0.0	0.0	0.0
0.0	0.0	0.0	0.0	0.0	0.0	0.0	0.0
0.0	0.0	0.0	0.0	0.0	0.0	0.0	0.0
0.0	0.0	0.0	0.0	0.0	0.0	0.0	0.0
0.0	0.0	0.0	0.0	0.0	0.0	0.0	0.0
0.0	0.0	0.0	0.0	0.0	0.0	0.0	0.0
0.0	0.0	0.0	0.0	0.0	0.0	0.0	0.0
0.0	0.0	0.0	0.0	0.0	0.0	0.0	0.0
0.0	0.0	0.0	0.0	0.0	0.0	0.0	0.0
0.0	0.0	0.0	0.0	0.0	0.0	0.0	0.0
0.0	0.0	0.0	0.0	0.0	0.0	0.0	0.0
0.0	0.0	0.0	0.0	0.0	0.0	0.0	0.0
0.0	0.0	0.0	0.0	0.0	0.0	0.0	0.0
0.0	0.0	0.0	0.0	0.0	0.0	0.0	0.0
0.0	0.0	0.0	0.0	0.0	0.0	0.0	0.0
0.0	0.0	0.0	0.0	0.0	0.0	0.0	0.0
0.0	0.0	0.0	0.0	0.0	0.0	0.0	0.0
0.0	0.0	0.0	0.0	0.0	0.0	0.0	0.0
0.0	0.0	0.0	0.0	0.0	0.0	0.0	0.0
0.0	0.0	0.0	0.0	0.0	0.0	0.0	0.0
0.0	0.0	0.0	0.0	0.0	0.0	0.0	0.0
0.0	0.0	0.0	0.0	0.0	0.0	0.0	0.0
0.0	0.0	0.0	0.0	0.0	0.0	0.0	0.0
0.0	0.0	0.0	0.0	0.0	0.0	0.0	0.0
0.0	0.0	0.0	0.0	0.0	0.0	0.0	0.0
0.0	0.0	0.0	0.0	0.0	0.0	0.0	0.0
0.0	0.0	0.0	0.0	0.0	0.0	0.0	0.0
0.0	0.0	0.0	0.0	0.0	0.0	0.0	0.0
0.0	0.0	0.0	0.0	0.0	0.0	0.0	0.0
0.0	0.0	0.0	0.0	0.0	0.0	0.0	0.0
0.0	0.0	0.0	0.0	0.0	0.0	0.0	0.0
0.0	0.0	0.0	0.0	0.0	0.0	0.0	0.0
0.0	0.0	0.0	0.0	0.0	0.0	0.0	0.0
0.0	0.0	0.0	0.0	0.0	0.0	0.0	0.0
0.0	0.0	0.0	0.0	0.0	0.0	0.0	0.0

0.0	0.0	0.0	0.0	0.0	0.0	0.0	0.0
0.0	0.0	0.0	0.0	0.0	0.0	0.0	0.0
0.0	0.0	0.0	0.0	0.0	0.0	0.0	0.0
0.0	0.0	0.0	0.0	0.0	0.0	0.0	0.0
0.0	0.0	0.0	0.0	0.0	0.0	0.0	0.0
0.0	0.0	0.0	0.0	0.0	0.0	0.0	0.0
0.0	0.0	0.0	0.0	0.0	0.0	0.0	0.0
0.0	0.0	0.0	0.0	0.0	0.0	0.0	0.0
0.0	0.0	0.0	0.0	0.0	0.0	0.0	0.0
0.0	0.0	0.0	0.0	0.0	0.0	0.0	0.0
0.0	0.0	0.0	0.0	0.0	0.0	0.0	0.0
0.0	0.0	0.0	0.0	0.0	0.0	0.0	0.0
0.0	0.0	0.0	0.0	0.0	0.0	0.0	0.0
0.0	0.0	0.0	0.0	0.0	0.0	0.0	0.0
0.0	0.0	0.0	0.0	0.0	0.0	0.0	0.0
0.0	0.0	0.0	0.0	0.0	0.0	0.0	0.0
0.0	0.0	0.0	0.0	0.0	0.0	0.0	0.0
0.0	0.0	0.0	0.0	0.0	0.0	0.0	0.0
0.0	0.0	0.0	0.0	0.0	0.0	0.0	0.0
0.0	0.0	0.0	0.0	0.0	0.0	0.0	0.0
0.0	0.0	0.0	0.0	0.0	0.0	0.0	0.0
0.0	0.0	0.0	0.0	0.0	0.0	0.0	0.0
0.0	0.0	0.0	0.0	0.0	0.0	0.0	0.0
0.0	0.0	0.0	0.0	0.0	0.0	0.0	0.0
0.0	0.0	0.0	0.0	0.0	0.0	0.0	0.0
0.0	0.0	0.0	0.0	0.0	0.0	0.0	0.0
0.0	0.0	0.0	0.0	0.0	0.0	0.0	0.0
0.0	0.0	0.0	0.0	0.0	0.0	0.0	0.0
0.0	0.0	0.0	0.0	0.0	0.0	0.0	0.0
0.0	0.0	0.0	0.0	0.0	0.0	0.0	0.0
0.0	0.0	0.0	0.0	0.0	0.0	0.0	0.0
0.0	0.0	0.0	0.0	0.0	0.0	0.0	0.0
0.0	0.0	0.0	0.0	0.0	0.0	0.0	0.0
0.0	0.0	0.0	0.0	0.0	0.0	0.0	0.0
0.0	0.0	0.0	0.0	0.0	0.0	0.0	0.0
0.0	0.0	0.0	0.0	0.0	0.0	0.0	0.0
0.0	0.0	0.0	0.0	0.0	0.0	0.0	0.0
0.0	0.0	0.0	0.0	0.0	0.0	0.0	0.0
0.0	0.0	0.0	0.0	0.0	0.0	0.0	0.0
0.0	0.0	0.0	0.0	0.0	0.0	0.0	0.0
0.0	0.0	0.0	0.0	0.0	0.0	0.0	0.0
0.0	0.0	0.0	0.0	0.0	0.0	0.0	0.0
0.0	0.0	0.0	0.0	0.0	0.0	0.0	0.0
0.0	0.0	0.0	0.0	0.0	0.0	0.0	0.0
0.0	0.0	0.0	0.0	0.0	0.0	0.0	0.0
0.0	0.0	0.0	0.0	0.0	0.0	0.0	0.0
0.0	0.0	0.0	0.0	0.0	0.0	0.0	0.0

0.0	0.0	0.0	0.0	0.0	0.0	0.0	0.0
0.0	0.0	0.0	0.0	0.0	0.0	0.0	0.0
0.0	0.0	0.0	0.0	0.0	0.0	0.0	0.0
0.0	0.0	0.0	0.0	0.0	0.0	0.0	0.0
0.0	0.0	0.0	0.0	0.0	0.0	0.0	0.0
0.0	0.0	0.0	0.0	0.0	0.0	0.0	0.0
0.0	0.0	0.0	0.0	0.0	0.0	0.0	0.0
0.0	0.0	0.0	0.0	0.0	0.0	0.0	0.0
0.0	0.0	0.0	0.0	0.0	0.0	0.0	0.0
0.0	0.0	0.0	0.0	0.0	0.0	0.0	0.0
0.0	0.0	0.0	0.0	0.0	0.0	0.0	0.0
0.0	0.0	0.0	0.0	0.0	0.0	0.0	0.0
0.0	0.0	0.0	0.0	0.0	0.0	0.0	0.0
0.0	0.0	0.0	0.0	0.0	0.0	0.0	0.0
0.0	0.0	0.0	0.0	0.0	0.0	0.0	0.0
0.0	0.0	0.0	0.0	0.0	0.0	0.0	0.0
0.0	0.0	0.0	0.0	0.0	0.0	0.0	0.0
0.0	0.0	0.0	0.0	0.0	0.0	0.0	0.0
0.0	0.0	0.0	0.0	0.0	0.0	0.0	0.0
0.0	0.0	0.0	0.0	0.0	0.0	0.0	0.0
0.0	0.0	0.0	0.0	0.0	0.0	0.0	0.0
0.0	0.0	0.0	0.0	0.0	0.0	0.0	0.0
0.0	0.0	0.0	0.0	0.0	0.0	0.0	0.0
0.0	0.0	0.0	0.0	0.0	0.0	0.0	0.0
0.0	0.0	0.0	0.0	0.0	0.0	0.0	0.0
0.0	0.0	0.0	0.0	0.0	0.0	0.0	0.0
0.0	0.0	0.0	0.0	0.0	0.0	0.0	0.0
0.0	0.0	0.0	0.0	0.0	0.0	0.0	0.0
0.0	0.0	0.0	0.0	0.0	0.0	0.0	0.0
0.0	0.0	0.0	0.0	0.0	0.0	0.0	0.0
0.0	0.0	0.0	0.0	0.0	0.0	0.0	0.0
0.0	0.0	0.0	0.0	0.0	0.0	0.0	0.0
0.0	0.0	0.0	0.0	0.0	0.0	0.0	0.0
0.0	0.0	0.0	0.0	0.0	0.0	0.0	0.0
0.0	0.0	0.0	0.0	0.0	0.0	0.0	0.0
0.0	0.0	0.0	0.0	0.0	0.0	0.0	0.0
0.0	0.0	0.0	0.0	0.0	0.0	0.0	0.0
0.0	0.0	0.0	0.0	0.0	0.0	0.0	0.0
0.0	0.0	0.0	0.0	0.0	0.0	0.0	0.0
0.0	0.0	0.0	0.0	0.0	0.0	0.0	0.0
0.0	0.0	0.0	0.0	0.0	0.0	0.0	0.0
0.0	0.0	0.0	0.0	0.0	0.0	0.0	0.0
0.0	0.0	0.0	0.0	0.0	0.0	0.0	0.0
0.0	0.0	0.0	0.0	0.0	0.0	0.0	0.0
0.0	0.0	0.0	0.0	0.0	0.0	0.0	0.0
0.0	0.0	0.0	0.0	0.0	0.0	0.0	0.0
0.0	0.0	0.0	0.0	0.0	0.0	0.0	0.0
0.0	0.0	0.0	0.0	0.0	0.0	0.0	0.0

0.0	0.0	0.0	0.0	0.0	0.0	0.0	0.0
0.0	0.0	0.0	0.0	0.0	0.0	0.0	0.0
0.0	0.0	0.0	0.0	0.0	0.0	0.0	0.0
0.0	0.0	0.0	0.0	0.0	0.0	0.0	0.0
0.0	0.0	0.0	0.0	0.0	0.0	0.0	0.0
0.0	0.0	0.0	0.0	0.0	0.0	0.0	0.0
0.0	0.0	0.0	0.0	0.0	0.0	0.0	0.0
0.0	0.0	0.0	0.0	0.0	0.0	0.0	0.0
0.0	0.0	0.0	0.0	0.0	0.0	0.0	0.0
0.0	0.0	0.0	0.0	0.0	0.0	0.0	0.0
0.0	0.0	0.0	0.0	0.0	0.0	0.0	0.0
0.0	0.0	0.0	0.0	0.0	0.0	0.0	0.0
0.0	0.0	0.0	0.0	0.0	0.0	0.0	0.0
0.0	0.0	0.0	0.0	0.0	0.0	0.0	0.0
0.0	0.0	0.0	0.0	0.0	0.0	0.0	0.0
0.0	0.0	0.0	0.0	0.0	0.0	0.0	0.0
0.0	0.0	0.0	0.0	0.0	0.0	0.0	0.0
0.0	0.0	0.0	0.0	0.0	0.0	0.0	0.0
0.0	0.0	0.0	0.0	0.0	0.0	0.0	0.0
0.0	0.0	0.0	0.0	0.0	0.0	0.0	0.0
0.0	0.0	0.0	0.0	0.0	0.0	0.0	0.0
0.0	0.0	0.0	0.0	0.0	0.0	0.0	0.0
0.0	0.0	0.0	0.0	0.0	0.0	0.0	0.0
0.0	0.0	0.0	0.0	0.0	0.0	0.0	0.0
0.0	0.0	0.0	0.0	0.0	0.0	0.0	0.0
0.0	0.0	0.0	0.0	0.0	0.0	0.0	0.0
0.0	0.0	0.0	0.0	0.0	0.0	0.0	0.0
0.0	0.0	0.0	0.0	0.0	0.0	0.0	0.0
0.0	0.0	0.0	0.0	0.0	0.0	0.0	0.0
0.0	0.0	0.0	0.0	0.0	0.0	0.0	0.0
0.0	0.0	0.0	0.0	0.0	0.0	0.0	0.0
0.0	0.0	0.0	0.0	0.0	0.0	0.0	0.0
0.0	0.0	0.0	0.0	0.0	0.0	0.0	0.0
0.0	0.0	0.0	0.0	0.0	0.0	0.0	0.0
0.0	0.0	0.0	0.0	0.0	0.0	0.0	0.0
0.0	0.0	0.0	0.0	0.0	0.0	0.0	0.0
0.0	0.0	0.0	0.0	0.0	0.0	0.0	0.0
0.0	0.0	0.0	0.0	0.0	0.0	0.0	0.0
0.0	0.0	0.0	0.0	0.0	0.0	0.0	0.0
0.0	0.0	0.0	0.0	0.0	0.0	0.0	0.0
0.0	0.0	0.0	0.0	0.0	0.0	0.0	0.0
0.0	0.0	0.0	0.0	0.0	0.0	0.0	0.0
0.0	0.0	0.0	0.0	0.0	0.0	0.0	0.0
0.0	0.0	0.0	0.0	0.0	0.0	0.0	0.0
0.0	0.0	0.0	0.0	0.0	0.0	0.0	0.0
0.0	0.0	0.0	0.0	0.0	0.0	0.0	0.0
0.0	0.0	0.0	0.0	0.0	0.0	0.0	0.0
0.0	0.0	0.0	0.0	0.0	0.0	0.0	0.0

0.0	0.0	0.0	0.0	0.0	0.0	0.0	0.0
0.0	0.0	0.0	0.0	0.0	0.0	0.0	0.0
0.0	0.0	0.0	0.0	0.0	0.0	0.0	0.0
0.0	0.0	0.0	0.0	0.0	0.0	0.0	0.0
0.0	0.0	0.0	0.0	0.0	0.0	0.0	0.0
0.0	0.0	0.0	0.0	0.0	0.0	0.0	0.0
0.0	0.0	0.0	0.0	0.0	0.0	0.0	0.0
0.0	0.0	0.0	0.0	0.0	0.0	0.0	0.0
0.0	0.0	0.0	0.0	0.0	0.0	0.0	0.0
0.0	0.0	0.0	0.0	0.0	0.0	0.0	0.0
0.0	0.0	0.0	0.0	0.0	0.0	0.0	0.0
0.0	0.0	0.0	0.0	0.0	0.0	0.0	0.0
0.0	0.0	0.0	0.0	0.0	0.0	0.0	0.0
0.0	0.0	0.0	0.0	0.0	0.0	0.0	0.0
0.0	0.0	0.0	0.0	0.0	0.0	0.0	0.0
0.0	0.0	0.0	0.0	0.0	0.0	0.0	0.0
0.0	0.0	0.0	0.0	0.0	0.0	0.0	0.0
0.0	0.0	0.0	0.0	0.0	0.0	0.0	0.0
0.0	0.0	0.0	0.0	0.0	0.0	0.0	0.0
0.0	0.0	0.0	0.0	0.0	0.0	0.0	0.0
0.0	0.0	0.0	0.0	0.0	0.0	0.0	0.0
0.0	0.0	0.0	0.0	0.0	0.0	0.0	0.0
0.0	0.0	0.0	0.0	0.0	0.0	0.0	0.0
0.0	0.0	0.0	0.0	0.0	0.0	0.0	0.0
0.0	0.0	0.0	0.0	0.0	0.0	0.0	0.0
0.0	0.0	0.0	0.0	0.0	0.0	0.0	0.0
0.0	0.0	0.0	0.0	0.0	0.0	0.0	0.0
0.0	0.0	0.0	0.0	0.0	0.0	0.0	0.0
0.0	0.0	0.0	0.0	0.0	0.0	0.0	0.0
0.0	0.0	0.0	0.0	0.0	0.0	0.0	0.0
0.0	0.0	0.0	0.0	0.0	0.0	0.0	0.0
0.0	0.0	0.0	0.0	0.0	0.0	0.0	0.0
0.0	0.0	0.0	0.0	0.0	0.0	0.0	0.0
0.0	0.0	0.0	0.0	0.0	0.0	0.0	0.0
0.0	0.0	0.0	0.0	0.0	0.0	0.0	0.0
0.0	0.0	0.0	0.0	0.0	0.0	0.0	0.0
0.0	0.0	0.0	0.0	0.0	0.0	0.0	0.0
0.0	0.0	0.0	0.0	0.0	0.0	0.0	0.0
0.0	0.0	0.0	0.0	0.0	0.0	0.0	0.0
0.0	0.0	0.0	0.0	0.0	0.0	0.0	0.0
0.0	0.0	0.0	0.0	0.0	0.0	0.0	0.0
0.0	0.0	0.0	0.0	0.0	0.0	0.0	0.0
0.0	0.0	0.0	0.0	0.0	0.0	0.0	0.0
0.0	0.0	0.0	0.0	0.0	0.0	0.0	0.0
0.0	0.0	0.0	0.0	0.0	0.0	0.0	0.0
0.0	0.0	0.0	0.0	0.0	0.0	0.0	0.0
0.0	0.0	0.0	0.0	0.0	0.0	0.0	0.0

0.0	0.0	0.0	0.0	0.0	0.0	0.0	0.0
0.0	0.0	0.0	0.0	0.0	0.0	0.0	0.0
0.0	0.0	0.0	0.0	0.0	0.0	0.0	0.0
0.0	0.0	0.0	0.0	0.0	0.0	0.0	0.0
0.0	0.0	0.0	0.0	0.0	0.0	0.0	0.0
0.0	0.0	0.0	0.0	0.0	0.0	0.0	0.0
0.0	0.0	0.0	0.0	0.0	0.0	0.0	0.0
0.0	0.0	0.0	0.0	0.0	0.0	0.0	0.0
0.0	0.0	0.0	0.0	0.0	0.0	0.0	0.0
0.0	0.0	0.0	0.0	0.0	0.0	0.0	0.0
0.0	0.0	0.0	0.0	0.0	0.0	0.0	0.0
0.0	0.0	0.0	0.0	0.0	0.0	0.0	0.0
0.0	0.0	0.0	0.0	0.0	0.0	0.0	0.0
0.0	0.0	0.0	0.0	0.0	0.0	0.0	0.0
0.0	0.0	0.0	0.0	0.0	0.0	0.0	0.0
0.0	0.0	0.0	0.0	0.0	0.0	0.0	0.0
0.0	0.0	0.0	0.0	0.0	0.0	0.0	0.0
0.0	0.0	0.0	0.0	0.0	0.0	0.0	0.0
0.0	0.0	0.0	0.0	0.0	0.0	0.0	0.0
0.0	0.0	0.0	0.0	0.0	0.0	0.0	0.0
0.0	0.0	0.0	0.0	0.0	0.0	0.0	0.0
0.0	0.0	0.0	0.0	0.0	0.0	0.0	0.0
0.0	0.0	0.0	0.0	0.0	0.0	0.0	0.0
0.0	0.0	0.0	0.0	0.0	0.0	0.0	0.0
0.0	0.0	0.0	0.0	0.0	0.0	0.0	0.0
0.0	0.0	0.0	0.0	0.0	0.0	0.0	0.0
0.0	0.0	0.0	0.0	0.0	0.0	0.0	0.0
0.0	0.0	0.0	0.0	0.0	0.0	0.0	0.0
0.0	0.0	0.0	0.0	0.0	0.0	0.0	0.0
0.0	0.0	0.0	0.0	0.0	0.0	0.0	0.0
0.0	0.0	0.0	0.0	0.0	0.0	0.0	0.0
0.0	0.0	0.0	0.0	0.0	0.0	0.0	0.0
0.0	0.0	0.0	0.0	0.0	0.0	0.0	0.0
0.0	0.0	0.0	0.0	0.0	0.0	0.0	0.0
0.0	0.0	0.0	0.0	0.0	0.0	0.0	0.0
0.0	0.0	0.0	0.0	0.0	0.0	0.0	0.0
0.0	0.0	0.0	0.0	0.0	0.0	0.0	0.0
0.0	0.0	0.0	0.0	0.0	0.0	0.0	0.0
0.0	0.0	0.0	0.0	0.0	0.0	0.0	0.0
0.0	0.0	0.0	0.0	0.0	0.0	0.0	0.0
0.0	0.0	0.0	0.0	0.0	0.0	0.0	0.0
0.0	0.0	0.0	0.0	0.0	0.0	0.0	0.0
0.0	0.0	0.0	0.0	0.0	0.0	0.0	0.0
0.0	0.0	0.0	0.0	0.0	0.0	0.0	0.0
0.0	0.0	0.0	0.0	0.0	0.0	0.0	0.0
0.0	0.0	0.0	0.0	0.0	0.0	0.0	0.0
0.0	0.0	0.0	0.0	0.0	0.0	0.0	0.0
0.0	0.0	0.0	0.0	0.0	0.0	0.0	0.0

0.0	0.0	0.0	0.0	0.0	0.0	0.0	0.0
0.0	0.0	0.0	0.0	0.0	0.0	0.0	0.0
0.0	0.0	0.0	0.0	0.0	0.0	0.0	0.0
0.0	0.0	0.0	0.0	0.0	0.0	0.0	0.0
0.0	0.0	0.0	0.0	0.0	0.0	0.0	0.0
0.0	0.0	0.0	0.0	0.0	0.0	0.0	0.0
0.0	0.0	0.0	0.0	0.0	0.0	0.0	0.0
0.0	0.0	0.0	0.0	0.0	0.0	0.0	0.0
0.0	0.0	0.0	0.0	0.0	0.0	0.0	0.0
0.0	0.0	0.0	0.0	0.0	0.0	0.0	0.0
0.0	0.0	0.0	0.0	0.0	0.0	0.0	0.0
0.0	0.0	0.0	0.0	0.0	0.0	0.0	0.0
0.0	0.0	0.0	0.0	0.0	0.0	0.0	0.0
0.0	0.0	0.0	0.0	0.0	0.0	0.0	0.0
0.0	0.0	0.0	0.0	0.0	0.0	0.0	0.0
0.0	0.0	0.0	0.0	0.0	0.0	0.0	0.0
0.0	0.0	0.0	0.0	0.0	0.0	0.0	0.0
0.0	0.0	0.0	0.0	0.0	0.0	0.0	0.0
0.0	0.0	0.0	0.0	0.0	0.0	0.0	0.0
0.0	0.0	0.0	0.0	0.0	0.0	0.0	0.0
0.0	0.0	0.0	0.0	0.0	0.0	0.0	0.0
0.0	0.0	0.0	0.0	0.0	0.0	0.0	0.0
0.0	0.0	0.0	0.0	0.0	0.0	0.0	0.0
0.0	0.0	0.0	0.0	0.0	0.0	0.0	0.0
0.0	0.0	0.0	0.0	0.0	0.0	0.0	0.0
0.0	0.0	0.0	0.0	0.0	0.0	0.0	0.0
0.0	0.0	0.0	0.0	0.0	0.0	0.0	0.0
0.0	0.0	0.0	0.0	0.0	0.0	0.0	0.0
0.0	0.0	0.0	0.0	0.0	0.0	0.0	0.0
0.0	0.0	0.0	0.0	0.0	0.0	0.0	0.0
0.0	0.0	0.0	0.0	0.0	0.0	0.0	0.0
0.0	0.0	0.0	0.0	0.0	0.0	0.0	0.0
0.0	0.0	0.0	0.0	0.0	0.0	0.0	0.0
0.0	0.0	0.0	0.0	0.0	0.0	0.0	0.0
0.0	0.0	0.0	0.0	0.0	0.0	0.0	0.0
0.0	0.0	0.0	0.0	0.0	0.0	0.0	0.0
0.0	0.0	0.0	0.0	0.0	0.0	0.0	0.0
0.0	0.0	0.0	0.0	0.0	0.0	0.0	0.0
0.0	0.0	0.0	0.0	0.0	0.0	0.0	0.0
0.0	0.0	0.0	0.0	0.0	0.0	0.0	0.0
0.0	0.0	0.0	0.0	0.0	0.0	0.0	0.0
0.0	0.0	0.0	0.0	0.0	0.0	0.0	0.0
0.0	0.0	0.0	0.0	0.0	0.0	0.0	0.0
0.0	0.0	0.0	0.0	0.0	0.0	0.0	0.0
0.0	0.0	0.0	0.0	0.0	0.0	0.0	0.0
0.0	0.0	0.0	0.0	0.0	0.0	0.0	0.0
0.0	0.0	0.0	0.0	0.0	0.0	0.0	0.0

0.0	0.0	0.0	0.0	0.0	0.0	0.0	0.0
0.0	0.0	0.0	0.0	0.0	0.0	0.0	0.0
0.0	0.0	0.0	0.0	0.0	0.0	0.0	0.0
0.0	0.0	0.0	0.0	0.0	0.0	0.0	0.0
0.0	0.0	0.0	0.0	0.0	0.0	0.0	0.0
0.0	0.0	0.0	0.0	0.0	0.0	0.0	0.0
0.0	0.0	0.0	0.0	0.0	0.0	0.0	0.0
0.0	0.0	0.0	0.0	0.0	0.0	0.0	0.0
0.0	0.0	0.0	0.0	0.0	0.0	0.0	0.0
0.0	0.0	0.0	0.0	0.0	0.0	0.0	0.0
0.0	0.0	0.0	0.0	0.0	0.0	0.0	0.0
0.0	0.0	0.0	0.0	0.0	0.0	0.0	0.0
0.0	0.0	0.0	0.0	0.0	0.0	0.0	0.0
0.0	0.0	0.0	0.0	0.0	0.0	0.0	0.0
0.0	0.0	0.0	0.0	0.0	0.0	0.0	0.0
0.0	0.0	0.0	0.0	0.0	0.0	0.0	0.0
0.0	0.0	0.0	0.0	0.0	0.0	0.0	0.0
0.0	0.0	0.0	0.0	0.0	0.0	0.0	0.0
0.0	0.0	0.0	0.0	0.0	0.0	0.0	0.0
0.0	0.0	0.0	0.0	0.0	0.0	0.0	0.0
0.0	0.0	0.0	0.0	0.0	0.0	0.0	0.0
0.0	0.0	0.0	0.0	0.0	0.0	0.0	0.0
0.0	0.0	0.0	0.0	0.0	0.0	0.0	0.0
0.0	0.0	0.0	0.0	0.0	0.0	0.0	0.0
0.0	0.0	0.0	0.0	0.0	0.0	0.0	0.0
0.0	0.0	0.0	0.0	0.0	0.0	0.0	0.0
0.0	0.0	0.0	0.0	0.0	0.0	0.0	0.0
0.0	0.0	0.0	0.0	0.0	0.0	0.0	0.0
0.0	0.0	0.0	0.0	0.0	0.0	0.0	0.0
0.0	0.0	0.0	0.0	0.0	0.0	0.0	0.0
0.0	0.0	0.0	0.0	0.0	0.0	0.0	0.0
0.0	0.0	0.0	0.0	0.0	0.0	0.0	0.0
0.0	0.0	0.0	0.0	0.0	0.0	0.0	0.0
0.0	0.0	0.0	0.0	0.0	0.0	0.0	0.0
0.0	0.0	0.0	0.0	0.0	0.0	0.0	0.0
0.0	0.0	0.0	0.0	0.0	0.0	0.0	0.0
0.0	0.0	0.0	0.0	0.0	0.0	0.0	0.0
0.0	0.0	0.0	0.0	0.0	0.0	0.0	0.0
0.0	0.0	0.0	0.0	0.0	0.0	0.0	0.0
0.0	0.0	0.0	0.0	0.0	0.0	0.0	0.0
0.0	0.0	0.0	0.0	0.0	0.0	0.0	0.0
0.0	0.0	0.0	0.0	0.0	0.0	0.0	0.0
0.0	0.0	0.0	0.0	0.0	0.0	0.0	0.0
0.0	0.0	0.0	0.0	0.0	0.0	0.0	0.0
0.0	0.0	0.0	0.0	0.0	0.0	0.0	0.0
0.0	0.0	0.0	0.0	0.0	0.0	0.0	0.0
0.0	0.0	0.0	0.0	0.0	0.0	0.0	0.0

0.0	0.0	0.0	0.0	0.0	0.0	0.0	0.0
0.0	0.0	0.0	0.0	0.0	0.0	0.0	0.0
0.0	0.0	0.0	0.0	0.0	0.0	0.0	0.0
0.0	0.0	0.0	0.0	0.0	0.0	0.0	0.0
0.0	0.0	0.0	0.0	0.0	0.0	0.0	0.0
0.0	0.0	0.0	0.0	0.0	0.0	0.0	0.0
0.0	0.0	0.0	0.0	0.0	0.0	0.0	0.0
0.0	0.0	0.0	0.0	0.0	0.0	0.0	0.0
0.0	0.0	0.0	0.0	0.0	0.0	0.0	0.0
0.0	0.0	0.0	0.0	0.0	0.0	0.0	0.0
0.0	0.0	0.0	0.0	0.0	0.0	0.0	0.0
0.0	0.0	0.0	0.0	0.0	0.0	0.0	0.0
0.0	0.0	0.0	0.0	0.0	0.0	0.0	0.0
0.0	0.0	0.0	0.0	0.0	0.0	0.0	0.0
0.0	0.0	0.0	0.0	0.0	0.0	0.0	0.0
0.0	0.0	0.0	0.0	0.0	0.0	0.0	0.0
0.0	0.0	0.0	0.0	0.0	0.0	0.0	0.0
0.0	0.0	0.0	0.0	0.0	0.0	0.0	0.0
0.0	0.0	0.0	0.0	0.0	0.0	0.0	0.0
0.0	0.0	0.0	0.0	0.0	0.0	0.0	0.0
0.0	0.0	0.0	0.0	0.0	0.0	0.0	0.0
0.0	0.0	0.0	0.0	0.0	0.0	0.0	0.0
0.0	0.0	0.0	0.0	0.0	0.0	0.0	0.0
0.0	0.0	0.0	0.0	0.0	0.0	0.0	0.0
0.0	0.0	0.0	0.0	0.0	0.0	0.0	0.0
0.0	0.0	0.0	0.0	0.0	0.0	0.0	0.0
0.0	0.0	0.0	0.0	0.0	0.0	0.0	0.0
0.0	0.0	0.0	0.0	0.0	0.0	0.0	0.0
0.0	0.0	0.0	0.0	0.0	0.0	0.0	0.0
0.0	0.0	0.0	0.0	0.0	0.0	0.0	0.0
0.0	0.0	0.0	0.0	0.0	0.0	0.0	0.0
0.0	0.0	0.0	0.0	0.0	0.0	0.0	0.0
0.0	0.0	0.0	0.0	0.0	0.0	0.0	0.0
0.0	0.0	0.0	0.0	0.0	0.0	0.0	0.0
0.0	0.0	0.0	0.0	0.0	0.0	0.0	0.0
0.0	0.0	0.0	0.0	0.0	0.0	0.0	0.0
0.0	0.0	0.0	0.0	0.0	0.0	0.0	0.0
0.0	0.0	0.0	0.0	0.0	0.0	0.0	0.0
0.0	0.0	0.0	0.0	0.0	0.0	0.0	0.0
0.0	0.0	0.0	0.0	0.0	0.0	0.0	0.0
0.0	0.0	0.0	0.0	0.0	0.0	0.0	0.0
0.0	0.0	0.0	0.0	0.0	0.0	0.0	0.0
0.0	0.0	0.0	0.0	0.0	0.0	0.0	0.0
0.0	0.0	0.0	0.0	0.0	0.0	0.0	0.0
0.0	0.0	0.0	0.0	0.0	0.0	0.0	0.0
0.0	0.0	0.0	0.0	0.0	0.0	0.0	0.0
0.0	0.0	0.0	0.0	0.0	0.0	0.0	0.0
0.0	0.0	0.0	0.0	0.0	0.0	0.0	0.0

0.0	0.0	0.0	0.0	0.0	0.0	0.0	0.0
0.0	0.0	0.0	0.0	0.0	0.0	0.0	0.0
0.0	0.0	0.0	0.0	0.0	0.0	0.0	0.0
0.0	0.0	0.0	0.0	0.0	0.0	0.0	0.0
0.0	0.0	0.0	0.0	0.0	0.0	0.0	0.0
0.0	0.0	0.0	0.0	0.0	0.0	0.0	0.0
0.0	0.0	0.0	0.0	0.0	0.0	0.0	0.0
0.0	0.0	0.0	0.0	0.0	0.0	0.0	0.0
0.0	0.0	0.0	0.0	0.0	0.0	0.0	0.0
0.0	0.0	0.0	0.0	0.0	0.0	0.0	0.0
0.0	0.0	0.0	0.0	0.0	0.0	0.0	0.0
0.0	0.0	0.0	0.0	0.0	0.0	0.0	0.0
0.0	0.0	0.0	0.0	0.0	0.0	0.0	0.0
0.0	0.0	0.0	0.0	0.0	0.0	0.0	0.0
0.0	0.0	0.0	0.0	0.0	0.0	0.0	0.0
0.0	0.0	0.0	0.0	0.0	0.0	0.0	0.0
0.0	0.0	0.0	0.0	0.0	0.0	0.0	0.0
0.0	0.0	0.0	0.0	0.0	0.0	0.0	0.0
0.0	0.0	0.0	0.0	0.0	0.0	0.0	0.0
0.0	0.0	0.0	0.0	0.0	0.0	0.0	0.0
0.0	0.0	0.0	0.0	0.0	0.0	0.0	0.0
0.0	0.0	0.0	0.0	0.0	0.0	0.0	0.0
0.0	0.0	0.0	0.0	0.0	0.0	0.0	0.0
0.0	0.0	0.0	0.0	0.0	0.0	0.0	0.0
0.0	0.0	0.0	0.0	0.0	0.0	0.0	0.0
0.0	0.0	0.0	0.0	0.0	0.0	0.0	0.0
0.0	0.0	0.0	0.0	0.0	0.0	0.0	0.0
0.0	0.0	0.0	0.0	0.0	0.0	0.0	0.0
0.0	0.0	0.0	0.0	0.0	0.0	0.0	0.0
0.0	0.0	0.0	0.0	0.0	0.0	0.0	0.0
0.0	0.0	0.0	0.0	0.0	0.0	0.0	0.0
0.0	0.0	0.0	0.0	0.0	0.0	0.0	0.0
0.0	0.0	0.0	0.0	0.0	0.0	0.0	0.0
0.0	0.0	0.0	0.0	0.0	0.0	0.0	0.0
0.0	0.0	0.0	0.0	0.0	0.0	0.0	0.0
0.0	0.0	0.0	0.0	0.0	0.0	0.0	0.0
0.0	0.0	0.0	0.0	0.0	0.0	0.0	0.0
0.0	0.0	0.0	0.0	0.0	0.0	0.0	0.0
0.0	0.0	0.0	0.0	0.0	0.0	0.0	0.0
0.0	0.0	0.0	0.0	0.0	0.0	0.0	0.0
0.0	0.0	0.0	0.0	0.0	0.0	0.0	0.0
0.0	0.0	0.0	0.0	0.0	0.0	0.0	0.0
0.0	0.0	0.0	0.0	0.0	0.0	0.0	0.0
0.0	0.0	0.0	0.0	0.0	0.0	0.0	0.0
0.0	0.0	0.0	0.0	0.0	0.0	0.0	0.0
0.0	0.0	0.0	0.0	0.0	0.0	0.0	0.0
0.0	0.0	0.0	0.0	0.0	0.0	0.0	0.0

0.0	0.0	0.0	0.0	0.0	0.0	0.0	0.0
0.0	0.0	0.0	0.0	0.0	0.0	0.0	0.0
0.0	0.0	0.0	0.0	0.0	0.0	0.0	0.0
0.0	0.0	0.0	0.0	0.0	0.0	0.0	0.0
0.0	0.0	0.0	0.0	0.0	0.0	0.0	0.0
0.0	0.0	0.0	0.0	0.0	0.0	0.0	0.0
0.0	0.0	0.0	0.0	0.0	0.0	0.0	0.0
0.0	0.0	0.0	0.0	0.0	0.0	0.0	0.0
0.0	0.0	0.0	0.0	0.0	0.0	0.0	0.0
0.0	0.0	0.0	0.0	0.0	0.0	0.0	0.0
0.0	0.0	0.0	0.0	0.0	0.0	0.0	0.0
0.0	0.0	0.0	0.0	0.0	0.0	0.0	0.0
0.0	0.0	0.0	0.0	0.0	0.0	0.0	0.0
0.0	0.0	0.0	0.0	0.0	0.0	0.0	0.0
0.0	0.0	0.0	0.0	0.0	0.0	0.0	0.0
0.0	0.0	0.0	0.0	0.0	0.0	0.0	0.0
0.0	0.0	0.0	0.0	0.0	0.0	0.0	0.0
0.0	0.0	0.0	0.0	0.0	0.0	0.0	0.0
0.0	0.0	0.0	0.0	0.0	0.0	0.0	0.0
0.0	0.0	0.0	0.0	0.0	0.0	0.0	0.0
0.0	0.0	0.0	0.0	0.0	0.0	0.0	0.0
0.0	0.0	0.0	0.0	0.0	0.0	0.0	0.0
0.0	0.0	0.0	0.0	0.0	0.0	0.0	0.0
0.0	0.0	0.0	0.0	0.0	0.0	0.0	0.0
0.0	0.0	0.0	0.0	0.0	0.0	0.0	0.0
0.0	0.0	0.0	0.0	0.0	0.0	0.0	0.0
0.0	0.0	0.0	0.0	0.0	0.0	0.0	0.0
0.0	0.0	0.0	0.0	0.0	0.0	0.0	0.0
0.0	0.0	0.0	0.0	0.0	0.0	0.0	0.0
0.0	0.0	0.0	0.0	0.0	0.0	0.0	0.0
0.0	0.0	0.0	0.0	0.0	0.0	0.0	0.0
0.0	0.0	0.0	0.0	0.0	0.0	0.0	0.0
0.0	0.0	0.0	0.0	0.0	0.0	0.0	0.0
0.0	0.0	0.0	0.0	0.0	0.0	0.0	0.0
0.0	0.0	0.0	0.0	0.0	0.0	0.0	0.0
0.0	0.0	0.0	0.0	0.0	0.0	0.0	0.0
0.0	0.0	0.0	0.0	0.0	0.0	0.0	0.0
0.0	0.0	0.0	0.0	0.0	0.0	0.0	0.0
0.0	0.0	0.0	0.0	0.0	0.0	0.0	0.0
0.0	0.0	0.0	0.0	0.0	0.0	0.0	0.0
0.0	0.0	0.0	0.0	0.0	0.0	0.0	0.0
0.0	0.0	0.0	0.0	0.0	0.0	0.0	0.0
0.0	0.0	0.0	0.0	0.0	0.0	0.0	0.0
0.0	0.0	0.0	0.0	0.0	0.0	0.0	0.0
0.0	0.0	0.0	0.0	0.0	0.0	0.0	0.0
0.0	0.0	0.0	0.0	0.0	0.0	0.0	0.0
0.0	0.0	0.0	0.0	0.0	0.0	0.0	0.0

0.0	0.0	0.0	0.0	0.0	0.0	0.0	0.0
0.0	0.0	0.0	0.0	0.0	0.0	0.0	0.0
0.0	0.0	0.0	0.0	0.0	0.0	0.0	0.0
0.0	0.0	0.0	0.0	0.0	0.0	0.0	0.0
0.0	0.0	0.0	0.0	0.0	0.0	0.0	0.0
0.0	0.0	0.0	0.0	0.0	0.0	0.0	0.0
0.0	0.0	0.0	0.0	0.0	0.0	0.0	0.0
0.0	0.0	0.0	0.0	0.0	0.0	0.0	0.0
0.0	0.0	0.0	0.0	0.0	0.0	0.0	0.0
0.0	0.0	0.0	0.0	0.0	0.0	0.0	0.0
0.0	0.0	0.0	0.0	0.0	0.0	0.0	0.0
0.0	0.0	0.0	0.0	0.0	0.0	0.0	0.0
0.0	0.0	0.0	0.0	0.0	0.0	0.0	0.0
0.0	0.0	0.0	0.0	0.0	0.0	0.0	0.0
0.0	0.0	0.0	0.0	0.0	0.0	0.0	0.0
0.0	0.0	0.0	0.0	0.0	0.0	0.0	0.0
0.0	0.0	0.0	0.0	0.0	0.0	0.0	0.0
0.0	0.0	0.0	0.0	0.0	0.0	0.0	0.0
0.0	0.0	0.0	0.0	0.0	0.0	0.0	0.0
0.0	0.0	0.0	0.0	0.0	0.0	0.0	0.0
0.0	0.0	0.0	0.0	0.0	0.0	0.0	0.0
0.0	0.0	0.0	0.0	0.0	0.0	0.0	0.0
0.0	0.0	0.0	0.0	0.0	0.0	0.0	0.0
0.0	0.0	0.0	0.0	0.0	0.0	0.0	0.0
0.0	0.0	0.0	0.0	0.0	0.0	0.0	0.0
0.0	0.0	0.0	0.0	0.0	0.0	0.0	0.0
0.0	0.0	0.0	0.0	0.0	0.0	0.0	0.0
0.0	0.0	0.0	0.0	0.0	0.0	0.0	0.0
0.0	0.0	0.0	0.0	0.0	0.0	0.0	0.0
0.0	0.0	0.0	0.0	0.0	0.0	0.0	0.0
0.0	0.0	0.0	0.0	0.0	0.0	0.0	0.0
0.0	0.0	0.0	0.0	0.0	0.0	0.0	0.0
0.0	0.0	0.0	0.0	0.0	0.0	0.0	0.0
0.0	0.0	0.0	0.0	0.0	0.0	0.0	0.0
0.0	0.0	0.0	0.0	0.0	0.0	0.0	0.0
0.0	0.0	0.0	0.0	0.0	0.0	0.0	0.0
0.0	0.0	0.0	0.0	0.0	0.0	0.0	0.0
0.0	0.0	0.0	0.0	0.0	0.0	0.0	0.0
0.0	0.0	0.0	0.0	0.0	0.0	0.0	0.0
0.0	0.0	0.0	0.0	0.0	0.0	0.0	0.0
0.0	0.0	0.0	0.0	0.0	0.0	0.0	0.0
0.0	0.0	0.0	0.0	0.0	0.0	0.0	0.0
0.0	0.0	0.0	0.0	0.0	0.0	0.0	0.0
0.0	0.0	0.0	0.0	0.0	0.0	0.0	0.0
0.0	0.0	0.0	0.0	0.0	0.0	0.0	0.0
0.0	0.0	0.0	0.0	0.0	0.0	0.0	0.0
0.0	0.0	0.0	0.0	0.0	0.0	0.0	0.0
0.0	0.0	0.0	0.0	0.0	0.0	0.0	0.0

0.0	0.0	0.0	0.0	0.0	0.0	0.0	0.0
0.0	0.0	0.0	0.0	0.0	0.0	0.0	0.0
0.0	0.0	0.0	0.0	0.0	0.0	0.0	0.0
0.0	0.0	0.0	0.0	0.0	0.0	0.0	0.0
0.0	0.0	0.0	0.0	0.0	0.0	0.0	0.0
0.0	0.0	0.0	0.0	0.0	0.0	0.0	0.0
0.0	0.0	0.0	0.0	0.0	0.0	0.0	0.0
0.0	0.0	0.0	0.0	0.0	0.0	0.0	0.0
0.0	0.0	0.0	0.0	0.0	0.0	0.0	0.0
0.0	0.0	0.0	0.0	0.0	0.0	0.0	0.0
0.0	0.0	0.0	0.0	0.0	0.0	0.0	0.0
0.0	0.0	0.0	0.0	0.0	0.0	0.0	0.0
0.0	0.0	0.0	0.0	0.0	0.0	0.0	0.0
0.0	0.0	0.0	0.0	0.0	0.0	0.0	0.0
0.0	0.0	0.0	0.0	0.0	0.0	0.0	0.0
0.0	0.0	0.0	0.0	0.0	0.0	0.0	0.0
0.0	0.0	0.0	0.0	0.0	0.0	0.0	0.0
0.0	0.0	0.0	0.0	0.0	0.0	0.0	0.0
0.0	0.0	0.0	0.0	0.0	0.0	0.0	0.0
0.0	0.0	0.0	0.0	0.0	0.0	0.0	0.0
0.0	0.0	0.0	0.0	0.0	0.0	0.0	0.0
0.0	0.0	0.0	0.0	0.0	0.0	0.0	0.0
0.0	0.0	0.0	0.0	0.0	0.0	0.0	0.0
0.0	0.0	0.0	0.0	0.0	0.0	0.0	0.0
0.0	0.0	0.0	0.0	0.0	0.0	0.0	0.0
0.0	0.0	0.0	0.0	0.0	0.0	0.0	0.0
0.0	0.0	0.0	0.0	0.0	0.0	0.0	0.0
0.0	0.0	0.0	0.0	0.0	0.0	0.0	0.0
0.0	0.0	0.0	0.0	0.0	0.0	0.0	0.0
0.0	0.0	0.0	0.0	0.0	0.0	0.0	0.0
0.0	0.0	0.0	0.0	0.0	0.0	0.0	0.0
0.0	0.0	0.0	0.0	0.0	0.0	0.0	0.0
0.0	0.0	0.0	0.0	0.0	0.0	0.0	0.0
0.0	0.0	0.0	0.0	0.0	0.0	0.0	0.0
0.0	0.0	0.0	0.0	0.0	0.0	0.0	0.0
0.0	0.0	0.0	0.0	0.0	0.0	0.0	0.0
0.0	0.0	0.0	0.0	0.0	0.0	0.0	0.0
0.0	0.0	0.0	0.0	0.0	0.0	0.0	0.0
0.0	0.0	0.0	0.0	0.0	0.0	0.0	0.0
0.0	0.0	0.0	0.0	0.0	0.0	0.0	0.0
0.0	0.0	0.0	0.0	0.0	0.0	0.0	0.0
0.0	0.0	0.0	0.0	0.0	0.0	0.0	0.0
0.0	0.0	0.0	0.0	0.0	0.0	0.0	0.0
0.0	0.0	0.0	0.0	0.0	0.0	0.0	0.0
0.0	0.0	0.0	0.0	0.0	0.0	0.0	0.0
0.0	0.0	0.0	0.0	0.0	0.0	0.0	0.0
0.0	0.0	0.0	0.0	0.0	0.0	0.0	0.0
0.0	0.0	0.0	0.0	0.0	0.0	0.0	0.0

0.0	0.0	0.0	0.0	0.0	0.0	0.0	0.0
0.0	0.0	0.0	0.0	0.0	0.0	0.0	0.0
0.0	0.0	0.0	0.0	0.0	0.0	0.0	0.0
0.0	0.0	0.0	0.0	0.0	0.0	0.0	0.0
0.0	0.0	0.0	0.0	0.0	0.0	0.0	0.0
0.0	0.0	0.0	0.0	0.0	0.0	0.0	0.0
0.0	0.0	0.0	0.0	0.0	0.0	0.0	0.0
0.0	0.0	0.0	0.0	0.0	0.0	0.0	0.0
0.0	0.0	0.0	0.0	0.0	0.0	0.0	0.0
0.0	0.0	0.0	0.0	0.0	0.0	0.0	0.0
0.0	0.0	0.0	0.0	0.0	0.0	0.0	0.0
0.0	0.0	0.0	0.0	0.0	0.0	0.0	0.0
0.0	0.0	0.0	0.0	0.0	0.0	0.0	0.0
0.0	0.0	0.0	0.0	0.0	0.0	0.0	0.0
0.0	0.0	0.0	0.0	0.0	0.0	0.0	0.0
0.0	0.0	0.0	0.0	0.0	0.0	0.0	0.0
0.0	0.0	0.0	0.0	0.0	0.0	0.0	0.0
0.0	0.0	0.0	0.0	0.0	0.0	0.0	0.0
0.0	0.0	0.0	0.0	0.0	0.0	0.0	0.0
0.0	0.0	0.0	0.0	0.0	0.0	0.0	0.0
0.0	0.0	0.0	0.0	0.0	0.0	0.0	0.0
0.0	0.0	0.0	0.0	0.0	0.0	0.0	0.0
0.0	0.0	0.0	0.0	0.0	0.0	0.0	0.0
0.0	0.0	0.0	0.0	0.0	0.0	0.0	0.0
0.0	0.0	0.0	0.0	0.0	0.0	0.0	0.0
0.0	0.0	0.0	0.0	0.0	0.0	0.0	0.0
0.0	0.0	0.0	0.0	0.0	0.0	0.0	0.0
0.0	0.0	0.0	0.0	0.0	0.0	0.0	0.0
0.0	0.0	0.0	0.0	0.0	0.0	0.0	0.0
0.0	0.0	0.0	0.0	0.0	0.0	0.0	0.0
0.0	0.0	0.0	0.0	0.0	0.0	0.0	0.0
0.0	0.0	0.0	0.0	0.0	0.0	0.0	0.0
0.0	0.0	0.0	0.0	0.0	0.0	0.0	0.0
0.0	0.0	0.0	0.0	0.0	0.0	0.0	0.0
0.0	0.0	0.0	0.0	0.0	0.0	0.0	0.0
0.0	0.0	0.0	0.0	0.0	0.0	0.0	0.0
0.0	0.0	0.0	0.0	0.0	0.0	0.0	0.0
0.0	0.0	0.0	0.0	0.0	0.0	0.0	0.0
0.0	0.0	0.0	0.0	0.0	0.0	0.0	0.0
0.0	0.0	0.0	0.0	0.0	0.0	0.0	0.0
0.0	0.0	0.0	0.0	0.0	0.0	0.0	0.0
0.0	0.0	0.0	0.0	0.0	0.0	0.0	0.0
0.0	0.0	0.0	0.0	0.0	0.0	0.0	0.0
0.0	0.0	0.0	0.0	0.0	0.0	0.0	0.0
0.0	0.0	0.0	0.0	0.0	0.0	0.0	0.0
0.0	0.0	0.0	0.0	0.0	0.0	0.0	0.0
0.0	0.0	0.0	0.0	0.0	0.0	0.0	0.0

0.0	0.0	0.0	0.0	0.0	0.0	0.0	0.0
0.0	0.0	0.0	0.0	0.0	0.0	0.0	0.0
0.0	0.0	0.0	0.0	0.0	0.0	0.0	0.0
0.0	0.0	0.0	0.0	0.0	0.0	0.0	0.0
0.0	0.0	0.0	0.0	0.0	0.0	0.0	0.0
0.0	0.0	0.0	0.0	0.0	0.0	0.0	0.0
0.0	0.0	0.0	0.0	0.0	0.0	0.0	0.0
0.0	0.0	0.0	0.0	0.0	0.0	0.0	0.0
0.0	0.0	0.0	0.0	0.0	0.0	0.0	0.0
0.0	0.0	0.0	0.0	0.0	0.0	0.0	0.0
0.0	0.0	0.0	0.0	0.0	0.0	0.0	0.0
0.0	0.0	0.0	0.0	0.0	0.0	0.0	0.0
0.0	0.0	0.0	0.0	0.0	0.0	0.0	0.0
0.0	0.0	0.0	0.0	0.0	0.0	0.0	0.0
0.0	0.0	0.0	0.0	0.0	0.0	0.0	0.0
0.0	0.0	0.0	0.0	0.0	0.0	0.0	0.0
0.0	0.0	0.0	0.0	0.0	0.0	0.0	0.0
0.0	0.0	0.0	0.0	0.0	0.0	0.0	0.0
0.0	0.0	0.0	0.0	0.0	0.0	0.0	0.0
0.0	0.0	0.0	0.0	0.0	0.0	0.0	0.0
0.0	0.0	0.0	0.0	0.0	0.0	0.0	0.0
0.0	0.0	0.0	0.0	0.0	0.0	0.0	0.0
0.0	0.0	0.0	0.0	0.0	0.0	0.0	0.0
0.0	0.0	0.0	0.0	0.0	0.0	0.0	0.0
0.0	0.0	0.0	0.0	0.0	0.0	0.0	0.0
0.0	0.0	0.0	0.0	0.0	0.0	0.0	0.0
0.0	0.0	0.0	0.0	0.0	0.0	0.0	0.0
0.0	0.0	0.0	0.0	0.0	0.0	0.0	0.0
0.0	0.0	0.0	0.0	0.0	0.0	0.0	0.0
0.0	0.0	0.0	0.0	0.0	0.0	0.0	0.0
0.0	0.0	0.0	0.0	0.0	0.0	0.0	0.0
0.0	0.0	0.0	0.0	0.0	0.0	0.0	0.0
0.0	0.0	0.0	0.0	0.0	0.0	0.0	0.0
0.0	0.0	0.0	0.0	0.0	0.0	0.0	0.0
0.0	0.0	0.0	0.0	0.0	0.0	0.0	0.0
0.0	0.0	0.0	0.0	0.0	0.0	0.0	0.0
0.0	0.0	0.0	0.0	0.0	0.0	0.0	0.0
0.0	0.0	0.0	0.0	0.0	0.0	0.0	0.0
0.0	0.0	0.0	0.0	0.0	0.0	0.0	0.0
0.0	0.0	0.0	0.0	0.0	0.0	0.0	0.0
0.0	0.0	0.0	0.0	0.0	0.0	0.0	0.0
0.0	0.0	0.0	0.0	0.0	0.0	0.0	0.0
0.0	0.0	0.0	0.0	0.0	0.0	0.0	0.0
0.0	0.0	0.0	0.0	0.0	0.0	0.0	0.0
0.0	0.0	0.0	0.0	0.0	0.0	0.0	0.0
0.0	0.0	0.0	0.0	0.0	0.0	0.0	0.0
0.0	0.0	0.0	0.0	0.0	0.0	0.0	0.0

0.0	0.0	0.0	0.0	0.0	0.0	0.0	0.0
0.0	0.0	0.0	0.0	0.0	0.0	0.0	0.0
0.0	0.0	0.0	0.0	0.0	0.0	0.0	0.0
0.0	0.0	0.0	0.0	0.0	0.0	0.0	0.0
0.0	0.0	0.0	0.0	0.0	0.0	0.0	0.0
0.0	0.0	0.0	0.0	0.0	0.0	0.0	0.0
0.0	0.0	0.0	0.0	0.0	0.0	0.0	0.0
0.0	0.0	0.0	0.0	0.0	0.0	0.0	0.0
0.0	0.0	0.0	0.0	0.0	0.0	0.0	0.0
0.0	0.0	0.0	0.0	0.0	0.0	0.0	0.0
0.0	0.0	0.0	0.0	0.0	0.0	0.0	0.0
0.0	0.0	0.0	0.0	0.0	0.0	0.0	0.0
0.0	0.0	0.0	0.0	0.0	0.0	0.0	0.0
0.0	0.0	0.0	0.0	0.0	0.0	0.0	0.0
0.0	0.0	0.0	0.0	0.0	0.0	0.0	0.0
0.0	0.0	0.0	0.0	0.0	0.0	0.0	0.0
0.0	0.0	0.0	0.0	0.0	0.0	0.0	0.0
0.0	0.0	0.0	0.0	0.0	0.0	0.0	0.0
0.0	0.0	0.0	0.0	0.0	0.0	0.0	0.0
0.0	0.0	0.0	0.0	0.0	0.0	0.0	0.0
0.0	0.0	0.0	0.0	0.0	0.0	0.0	0.0
0.0	0.0	0.0	0.0	0.0	0.0	0.0	0.0
0.0	0.0	0.0	0.0	0.0	0.0	0.0	0.0
0.0	0.0	0.0	0.0	0.0	0.0	0.0	0.0
0.0	0.0	0.0	0.0	0.0	0.0	0.0	0.0
0.0	0.0	0.0	0.0	0.0	0.0	0.0	0.0
0.0	0.0	0.0	0.0	0.0	0.0	0.0	0.0
0.0	0.0	0.0	0.0	0.0	0.0	0.0	0.0
0.0	0.0	0.0	0.0	0.0	0.0	0.0	0.0
0.0	0.0	0.0	0.0	0.0	0.0	0.0	0.0
0.0	0.0	0.0	0.0	0.0	0.0	0.0	0.0
0.0	0.0	0.0	0.0	0.0	0.0	0.0	0.0
0.0	0.0	0.0	0.0	0.0	0.0	0.0	0.0
0.0	0.0	0.0	0.0	0.0	0.0	0.0	0.0
0.0	0.0	0.0	0.0	0.0	0.0	0.0	0.0
0.0	0.0	0.0	0.0	0.0	0.0	0.0	0.0
0.0	0.0	0.0	0.0	0.0	0.0	0.0	0.0
0.0	0.0	0.0	0.0	0.0	0.0	0.0	0.0
0.0	0.0	0.0	0.0	0.0	0.0	0.0	0.0
0.0	0.0	0.0	0.0	0.0	0.0	0.0	0.0
0.0	0.0	0.0	0.0	0.0	0.0	0.0	0.0
0.0	0.0	0.0	0.0	0.0	0.0	0.0	0.0
0.0	0.0	0.0	0.0	0.0	0.0	0.0	0.0
0.0	0.0	0.0	0.0	0.0	0.0	0.0	0.0
0.0	0.0	0.0	0.0	0.0	0.0	0.0	0.0
0.0	0.0	0.0	0.0	0.0	0.0	0.0	0.0
0.0	0.0	0.0	0.0	0.0	0.0	0.0	0.0

0.0	0.0	0.0	0.0	0.0	0.0	0.0	0.0
0.0	0.0	0.0	0.0	0.0	0.0	0.0	0.0
0.0	0.0	0.0	0.0	0.0	0.0	0.0	0.0
0.0	0.0	0.0	0.0	0.0	0.0	0.0	0.0
0.0	0.0	0.0	0.0	0.0	0.0	0.0	0.0
0.0	0.0	0.0	0.0	0.0	0.0	0.0	0.0
0.0	0.0	0.0	0.0	0.0	0.0	0.0	0.0
0.0	0.0	0.0	0.0	0.0	0.0	0.0	0.0
0.0	0.0	0.0	0.0	0.0	0.0	0.0	0.0
0.0	0.0	0.0	0.0	0.0	0.0	0.0	0.0
0.0	0.0	0.0	0.0	0.0	0.0	0.0	0.0
0.0	0.0	0.0	0.0	0.0	0.0	0.0	0.0
0.0	0.0	0.0	0.0	0.0	0.0	0.0	0.0
0.0	0.0	0.0	0.0	0.0	0.0	0.0	0.0
0.0	0.0	0.0	0.0	0.0	0.0	0.0	0.0
0.0	0.0	0.0	0.0	0.0	0.0	0.0	0.0
0.0	0.0	0.0	0.0	0.0	0.0	0.0	0.0
0.0	0.0	0.0	0.0	0.0	0.0	0.0	0.0
0.0	0.0	0.0	0.0	0.0	0.0	0.0	0.0
0.0	0.0	0.0	0.0	0.0	0.0	0.0	0.0
0.0	0.0	0.0	0.0	0.0	0.0	0.0	0.0
0.0	0.0	0.0	0.0	0.0	0.0	0.0	0.0
0.0	0.0	0.0	0.0	0.0	0.0	0.0	0.0
0.0	0.0	0.0	0.0	0.0	0.0	0.0	0.0
0.0	0.0	0.0	0.0	0.0	0.0	0.0	0.0
0.0	0.0	0.0	0.0	0.0	0.0	0.0	0.0
0.0	0.0	0.0	0.0	0.0	0.0	0.0	0.0
0.0	0.0	0.0	0.0	0.0	0.0	0.0	0.0
0.0	0.0	0.0	0.0	0.0	0.0	0.0	0.0
0.0	0.0	0.0	0.0	0.0	0.0	0.0	0.0
0.0	0.0	0.0	0.0	0.0	0.0	0.0	0.0
0.0	0.0	0.0	0.0	0.0	0.0	0.0	0.0
0.0	0.0	0.0	0.0	0.0	0.0	0.0	0.0
0.0	0.0	0.0	0.0	0.0	0.0	0.0	0.0
0.0	0.0	0.0	0.0	0.0	0.0	0.0	0.0
0.0	0.0	0.0	0.0	0.0	0.0	0.0	0.0
0.0	0.0	0.0	0.0	0.0	0.0	0.0	0.0
0.0	0.0	0.0	0.0	0.0	0.0	0.0	0.0
0.0	0.0	0.0	0.0	0.0	0.0	0.0	0.0
0.0	0.0	0.0	0.0	0.0	0.0	0.0	0.0
0.0	0.0	0.0	0.0	0.0	0.0	0.0	0.0
0.0	0.0	0.0	0.0	0.0	0.0	0.0	0.0
0.0	0.0	0.0	0.0	0.0	0.0	0.0	0.0
0.0	0.0	0.0	0.0	0.0	0.0	0.0	0.0
0.0	0.0	0.0	0.0	0.0	0.0	0.0	0.0
0.0	0.0	0.0	0.0	0.0	0.0	0.0	0.0
0.0	0.0	0.0	0.0	0.0	0.0	0.0	0.0

0.0	0.0	0.0	0.0	0.0	0.0	0.0	0.0
0.0	0.0	0.0	0.0	0.0	0.0	0.0	0.0
0.0	0.0	0.0	0.0	0.0	0.0	0.0	0.0
0.0	0.0	0.0	0.0	0.0	0.0	0.0	0.0
0.0	0.0	0.0	0.0	0.0	0.0	0.0	0.0
0.0	0.0	0.0	0.0	0.0	0.0	0.0	0.0
0.0	0.0	0.0	0.0	0.0	0.0	0.0	0.0
0.0	0.0	0.0	0.0	0.0	0.0	0.0	0.0
0.0	0.0	0.0	0.0	0.0	0.0	0.0	0.0
0.0	0.0	0.0	0.0	0.0	0.0	0.0	0.0
0.0	0.0	0.0	0.0	0.0	0.0	0.0	0.0
0.0	0.0	0.0	0.0	0.0	0.0	0.0	0.0
0.0	0.0	0.0	0.0	0.0	0.0	0.0	0.0
0.0	0.0	0.0	0.0	0.0	0.0	0.0	0.0
0.0	0.0	0.0	0.0	0.0	0.0	0.0	0.0
0.0	0.0	0.0	0.0	0.0	0.0	0.0	0.0
0.0	0.0	0.0	0.0	0.0	0.0	0.0	0.0
0.0	0.0	0.0	0.0	0.0	0.0	0.0	0.0
0.0	0.0	0.0	0.0	0.0	0.0	0.0	0.0
0.0	0.0	0.0	0.0	0.0	0.0	0.0	0.0
0.0	0.0	0.0	0.0	0.0	0.0	0.0	0.0
0.0	0.0	0.0	0.0	0.0	0.0	0.0	0.0
0.0	0.0	0.0	0.0	0.0	0.0	0.0	0.0
0.0	0.0	0.0	0.0	0.0	0.0	0.0	0.0
0.0	0.0	0.0	0.0	0.0	0.0	0.0	0.0
0.0	0.0	0.0	0.0	0.0	0.0	0.0	0.0
0.0	0.0	0.0	0.0	0.0	0.0	0.0	0.0
0.0	0.0	0.0	0.0	0.0	0.0	0.0	0.0
0.0	0.0	0.0	0.0	0.0	0.0	0.0	0.0
0.0	0.0	0.0	0.0	0.0	0.0	0.0	0.0
0.0	0.0	0.0	0.0	0.0	0.0	0.0	0.0
0.0	0.0	0.0	0.0	0.0	0.0	0.0	0.0
0.0	0.0	0.0	0.0	0.0	0.0	0.0	0.0
0.0	0.0	0.0	0.0	0.0	0.0	0.0	0.0
0.0	0.0	0.0	0.0	0.0	0.0	0.0	0.0
0.0	0.0	0.0	0.0	0.0	0.0	0.0	0.0
0.0	0.0	0.0	0.0	0.0	0.0	0.0	0.0
0.0	0.0	0.0	0.0	0.0	0.0	0.0	0.0
0.0	0.0	0.0	0.0	0.0	0.0	0.0	0.0
0.0	0.0	0.0	0.0	0.0	0.0	0.0	0.0
0.0	0.0	0.0	0.0	0.0	0.0	0.0	0.0
0.0	0.0	0.0	0.0	0.0	0.0	0.0	0.0
0.0	0.0	0.0	0.0	0.0	0.0	0.0	0.0
0.0	0.0	0.0	0.0	0.0	0.0	0.0	0.0
0.0	0.0	0.0	0.0	0.0	0.0	0.0	0.0
0.0	0.0	0.0	0.0	0.0	0.0	0.0	0.0
0.0	0.0	0.0	0.0	0.0	0.0	0.0	0.0

0.0	0.0	0.0	0.0	0.0	0.0	0.0	0.0
0.0	0.0	0.0	0.0	0.0	0.0	0.0	0.0
0.0	0.0	0.0	0.0	0.0	0.0	0.0	0.0
0.0	0.0	0.0	0.0	0.0	0.0	0.0	0.0
0.0	0.0	0.0	0.0	0.0	0.0	0.0	0.0
0.0	0.0	0.0	0.0	0.0	0.0	0.0	0.0
0.0	0.0	0.0	0.0	0.0	0.0	0.0	0.0
0.0	0.0	0.0	0.0	0.0	0.0	0.0	0.0
0.0	0.0	0.0	0.0	0.0	0.0	0.0	0.0
0.0	0.0	0.0	0.0	0.0	0.0	0.0	0.0
0.0	0.0	0.0	0.0	0.0	0.0	0.0	0.0
0.0	0.0	0.0	0.0	0.0	0.0	0.0	0.0
0.0	0.0	0.0	0.0	0.0	0.0	0.0	0.0
0.0	0.0	0.0	0.0	0.0	0.0	0.0	0.0
0.0	0.0	0.0	0.0	0.0	0.0	0.0	0.0
0.0	0.0	0.0	0.0	0.0	0.0	0.0	0.0
0.0	0.0	0.0	0.0	0.0	0.0	0.0	0.0
0.0	0.0	0.0	0.0	0.0	0.0	0.0	0.0
0.0	0.0	0.0	0.0	0.0	0.0	0.0	0.0
0.0	0.0	0.0	0.0	0.0	0.0	0.0	0.0
0.0	0.0	0.0	0.0	0.0	0.0	0.0	0.0
0.0	0.0	0.0	0.0	0.0	0.0	0.0	0.0
0.0	0.0	0.0	0.0	0.0	0.0	0.0	0.0
0.0	0.0	0.0	0.0	0.0	0.0	0.0	0.0
0.0	0.0	0.0	0.0	0.0	0.0	0.0	0.0
0.0	0.0	0.0	0.0	0.0	0.0	0.0	0.0
0.0	0.0	0.0	0.0	0.0	0.0	0.0	0.0
0.0	0.0	0.0	0.0	0.0	0.0	0.0	0.0
0.0	0.0	0.0	0.0	0.0	0.0	0.0	0.0
0.0	0.0	0.0	0.0	0.0	0.0	0.0	0.0
0.0	0.0	0.0	0.0	0.0	0.0	0.0	0.0
0.0	0.0	0.0	0.0	0.0	0.0	0.0	0.0
0.0	0.0	0.0	0.0	0.0	0.0	0.0	0.0
0.0	0.0	0.0	0.0	0.0	0.0	0.0	0.0
0.0	0.0	0.0	0.0	0.0	0.0	0.0	0.0
0.0	0.0	0.0	0.0	0.0	0.0	0.0	0.0
0.0	0.0	0.0	0.0	0.0	0.0	0.0	0.0
0.0	0.0	0.0	0.0	0.0	0.0	0.0	0.0
0.0	0.0	0.0	0.0	0.0	0.0	0.0	0.0
0.0	0.0	0.0	0.0	0.0	0.0	0.0	0.0
0.0	0.0	0.0	0.0	0.0	0.0	0.0	0.0
0.0	0.0	0.0	0.0	0.0	0.0	0.0	0.0
0.0	0.0	0.0	0.0	0.0	0.0	0.0	0.0
0.0	0.0	0.0	0.0	0.0	0.0	0.0	0.0
0.0	0.0	0.0	0.0	0.0	0.0	0.0	0.0
0.0	0.0	0.0	0.0	0.0	0.0	0.0	0.0
0.0	0.0	0.0	0.0	0.0	0.0	0.0	0.0

0.0	0.0	0.0	0.0	0.0	0.0	0.0	0.0
0.0	0.0	0.0	0.0	0.0	0.0	0.0	0.0
0.0	0.0	0.0	0.0	0.0	0.0	0.0	0.0
0.0	0.0	0.0	0.0	0.0	0.0	0.0	0.0
0.0	0.0	0.0	0.0	0.0	0.0	0.0	0.0
0.0	0.0	0.0	0.0	0.0	0.0	0.0	0.0
0.0	0.0	0.0	0.0	0.0	0.0	0.0	0.0
0.0	0.0	0.0	0.0	0.0	0.0	0.0	0.0
0.0	0.0	0.0	0.0	0.0	0.0	0.0	0.0
0.0	0.0	0.0	0.0	0.0	0.0	0.0	0.0
0.0	0.0	0.0	0.0	0.0	0.0	0.0	0.0
0.0	0.0	0.0	0.0	0.0	0.0	0.0	0.0
0.0	0.0	0.0	0.0	0.0	0.0	0.0	0.0
0.0	0.0	0.0	0.0	0.0	0.0	0.0	0.0
0.0	0.0	0.0	0.0	0.0	0.0	0.0	0.0
0.0	0.0	0.0	0.0	0.0	0.0	0.0	0.0
0.0	0.0	0.0	0.0	0.0	0.0	0.0	0.0
0.0	0.0	0.0	0.0	0.0	0.0	0.0	0.0
0.0	0.0	0.0	0.0	0.0	0.0	0.0	0.0
0.0	0.0	0.0	0.0	0.0	0.0	0.0	0.0
0.0	0.0	0.0	0.0	0.0	0.0	0.0	0.0
0.0	0.0	0.0	0.0	0.0	0.0	0.0	0.0
0.0	0.0	0.0	0.0	0.0	0.0	0.0	0.0
0.0	0.0	0.0	0.0	0.0	0.0	0.0	0.0
0.0	0.0	0.0	0.0	0.0	0.0	0.0	0.0
0.0	0.0	0.0	0.0	0.0	0.0	0.0	0.0
0.0	0.0	0.0	0.0	0.0	0.0	0.0	0.0
0.0	0.0	0.0	0.0	0.0	0.0	0.0	0.0
0.0	0.0	0.0	0.0	0.0	0.0	0.0	0.0
0.0	0.0	0.0	0.0	0.0	0.0	0.0	0.0
0.0	0.0	0.0	0.0	0.0	0.0	0.0	0.0
0.0	0.0	0.0	0.0	0.0	0.0	0.0	0.0
0.0	0.0	0.0	0.0	0.0	0.0	0.0	0.0
0.0	0.0	0.0	0.0	0.0	0.0	0.0	0.0
0.0	0.0	0.0	0.0	0.0	0.0	0.0	0.0
0.0	0.0	0.0	0.0	0.0	0.0	0.0	0.0
0.0	0.0	0.0	0.0	0.0	0.0	0.0	0.0
0.0	0.0	0.0	0.0	0.0	0.0	0.0	0.0
0.0	0.0	0.0	0.0	0.0	0.0	0.0	0.0
0.0	0.0	0.0	0.0	0.0	0.0	0.0	0.0
0.0	0.0	0.0	0.0	0.0	0.0	0.0	0.0
0.0	0.0	0.0	0.0	0.0	0.0	0.0	0.0
0.0	0.0	0.0	0.0	0.0	0.0	0.0	0.0
0.0	0.0	0.0	0.0	0.0	0.0	0.0	0.0
0.0	0.0	0.0	0.0	0.0	0.0	0.0	0.0
0.0	0.0	0.0	0.0	0.0	0.0	0.0	0.0
0.0	0.0	0.0	0.0	0.0	0.0	0.0	0.0

0.0	0.0	0.0	0.0	0.0	0.0	0.0	0.0
0.0	0.0	0.0	0.0	0.0	0.0	0.0	0.0
0.0	0.0	0.0	0.0	0.0	0.0	0.0	0.0
0.0	0.0	0.0	0.0	0.0	0.0	0.0	0.0
0.0	0.0	0.0	0.0	0.0	0.0	0.0	0.0
0.0	0.0	0.0	0.0	0.0	0.0	0.0	0.0
0.0	0.0	0.0	0.0	0.0	0.0	0.0	0.0
0.0	0.0	0.0	0.0	0.0	0.0	0.0	0.0
0.0	0.0	0.0	0.0	0.0	0.0	0.0	0.0
0.0	0.0	0.0	0.0	0.0	0.0	0.0	0.0
0.0	0.0	0.0	0.0	0.0	0.0	0.0	0.0
0.0	0.0	0.0	0.0	0.0	0.0	0.0	0.0
0.0	0.0	0.0	0.0	0.0	0.0	0.0	0.0
0.0	0.0	0.0	0.0	0.0	0.0	0.0	0.0
0.0	0.0	0.0	0.0	0.0	0.0	0.0	0.0
0.0	0.0	0.0	0.0	0.0	0.0	0.0	0.0
0.0	0.0	0.0	0.0	0.0	0.0	0.0	0.0
0.0	0.0	0.0	0.0	0.0	0.0	0.0	0.0
0.0	0.0	0.0	0.0	0.0	0.0	0.0	0.0
0.0	0.0	0.0	0.0	0.0	0.0	0.0	0.0
0.0	0.0	0.0	0.0	0.0	0.0	0.0	0.0
0.0	0.0	0.0	0.0	0.0	0.0	0.0	0.0
0.0	0.0	0.0	0.0	0.0	0.0	0.0	0.0
0.0	0.0	0.0	0.0	0.0	0.0	0.0	0.0
0.0	0.0	0.0	0.0	0.0	0.0	0.0	0.0
0.0	0.0	0.0	0.0	0.0	0.0	0.0	0.0
0.0	0.0	0.0	0.0	0.0	0.0	0.0	0.0
0.0	0.0	0.0	0.0	0.0	0.0	0.0	0.0
0.0	0.0	0.0	0.0	0.0	0.0	0.0	0.0
0.0	0.0	0.0	0.0	0.0	0.0	0.0	0.0
0.0	0.0	0.0	0.0	0.0	0.0	0.0	0.0
0.0	0.0	0.0	0.0	0.0	0.0	0.0	0.0
0.0	0.0	0.0	0.0	0.0	0.0	0.0	0.0
0.0	0.0	0.0	0.0	0.0	0.0	0.0	0.0
0.0	0.0	0.0	0.0	0.0	0.0	0.0	0.0
0.0	0.0	0.0	0.0	0.0	0.0	0.0	0.0
0.0	0.0	0.0	0.0	0.0	0.0	0.0	0.0
0.0	0.0	0.0	0.0	0.0	0.0	0.0	0.0
0.0	0.0	0.0	0.0	0.0	0.0	0.0	0.0
0.0	0.0	0.0	0.0	0.0	0.0	0.0	0.0
0.0	0.0	0.0	0.0	0.0	0.0	0.0	0.0
0.0	0.0	0.0	0.0	0.0	0.0	0.0	0.0
0.0	0.0	0.0	0.0	0.0	0.0	0.0	0.0
0.0	0.0	0.0	0.0	0.0	0.0	0.0	0.0
0.0	0.0	0.0	0.0	0.0	0.0	0.0	0.0
0.0	0.0	0.0	0.0	0.0	0.0	0.0	0.0
0.0	0.0	0.0	0.0	0.0	0.0	0.0	0.0

0.0	0.0	0.0	0.0	0.0	0.0	0.0	0.0
0.0	0.0	0.0	0.0	0.0	0.0	0.0	0.0
0.0	0.0	0.0	0.0	0.0	0.0	0.0	0.0
0.0	0.0	0.0	0.0	0.0	0.0	0.0	0.0
0.0	0.0	0.0	0.0	0.0	0.0	0.0	0.0
0.0	0.0	0.0	0.0	0.0	0.0	0.0	0.0
0.0	0.0	0.0	0.0	0.0	0.0	0.0	0.0
0.0	0.0	0.0	0.0	0.0	0.0	0.0	0.0
0.0	0.0	0.0	0.0	0.0	0.0	0.0	0.0
0.0	0.0	0.0	0.0	0.0	0.0	0.0	0.0
0.0	0.0	0.0	0.0	0.0	0.0	0.0	0.0
0.0	0.0	0.0	0.0	0.0	0.0	0.0	0.0
0.0	0.0	0.0	0.0	0.0	0.0	0.0	0.0
0.0	0.0	0.0	0.0	0.0	0.0	0.0	0.0
0.0	0.0	0.0	0.0	0.0	0.0	0.0	0.0
0.0	0.0	0.0	0.0	0.0	0.0	0.0	0.0
0.0	0.0	0.0	0.0	0.0	0.0	0.0	0.0
0.0	0.0	0.0	0.0	0.0	0.0	0.0	0.0
0.0	0.0	0.0	0.0	0.0	0.0	0.0	0.0
0.0	0.0	0.0	0.0	0.0	0.0	0.0	0.0
0.0	0.0	0.0	0.0	0.0	0.0	0.0	0.0
0.0	0.0	0.0	0.0	0.0	0.0	0.0	0.0
0.0	0.0	0.0	0.0	0.0	0.0	0.0	0.0
0.0	0.0	0.0	0.0	0.0	0.0	0.0	0.0
0.0	0.0	0.0	0.0	0.0	0.0	0.0	0.0
0.0	0.0	0.0	0.0	0.0	0.0	0.0	0.0
0.0	0.0	0.0	0.0	0.0	0.0	0.0	0.0
0.0	0.0	0.0	0.0	0.0	0.0	0.0	0.0
0.0	0.0	0.0	0.0	0.0	0.0	0.0	0.0
0.0	0.0	0.0	0.0	0.0	0.0	0.0	0.0
0.0	0.0	0.0	0.0	0.0	0.0	0.0	0.0
0.0	0.0	0.0	0.0	0.0	0.0	0.0	0.0
0.0	0.0	0.0	0.0	0.0	0.0	0.0	0.0
0.0	0.0	0.0	0.0	0.0	0.0	0.0	0.0
0.0	0.0	0.0	0.0	0.0	0.0	0.0	0.0
0.0	0.0	0.0	0.0	0.0	0.0	0.0	0.0
0.0	0.0	0.0	0.0	0.0	0.0	0.0	0.0
0.0	0.0	0.0	0.0	0.0	0.0	0.0	0.0
0.0	0.0	0.0	0.0	0.0	0.0	0.0	0.0
0.0	0.0	0.0	0.0	0.0	0.0	0.0	0.0
0.0	0.0	0.0	0.0	0.0	0.0	0.0	0.0
0.0	0.0	0.0	0.0	0.0	0.0	0.0	0.0
0.0	0.0	0.0	0.0	0.0	0.0	0.0	0.0
0.0	0.0	0.0	0.0	0.0	0.0	0.0	0.0
0.0	0.0	0.0	0.0	0.0	0.0	0.0	0.0
0.0	0.0	0.0	0.0	0.0	0.0	0.0	0.0
0.0	0.0	0.0	0.0	0.0	0.0	0.0	0.0

0.0	0.0	0.0	0.0	0.0	0.0	0.0	0.0
0.0	0.0	0.0	0.0	0.0	0.0	0.0	0.0
0.0	0.0	0.0	0.0	0.0	0.0	0.0	0.0
0.0	0.0	0.0	0.0	0.0	0.0	0.0	0.0
0.0	0.0	0.0	0.0	0.0	0.0	0.0	0.0
0.0	0.0	0.0	0.0	0.0	0.0	0.0	0.0
0.0	0.0	0.0	0.0	0.0	0.0	0.0	0.0
0.0	0.0	0.0	0.0	0.0	0.0	0.0	0.0
0.0	0.0	0.0	0.0	0.0	0.0	0.0	0.0
0.0	0.0	0.0	0.0	0.0	0.0	0.0	0.0
0.0	0.0	0.0	0.0	0.0	0.0	0.0	0.0
0.0	0.0	0.0	0.0	0.0	0.0	0.0	0.0
0.0	0.0	0.0	0.0	0.0	0.0	0.0	0.0
0.0	0.0	0.0	0.0	0.0	0.0	0.0	0.0
0.0	0.0	0.0	0.0	0.0	0.0	0.0	0.0
0.0	0.0	0.0	0.0	0.0	0.0	0.0	0.0
0.0	0.0	0.0	0.0	0.0	0.0	0.0	0.0
0.0	0.0	0.0	0.0	0.0	0.0	0.0	0.0
0.0	0.0	0.0	0.0	0.0	0.0	0.0	0.0
0.0	0.0	0.0	0.0	0.0	0.0	0.0	0.0
0.0	0.0	0.0	0.0	0.0	0.0	0.0	0.0
0.0	0.0	0.0	0.0	0.0	0.0	0.0	0.0
0.0	0.0	0.0	0.0	0.0	0.0	0.0	0.0
0.0	0.0	0.0	0.0	0.0	0.0	0.0	0.0
0.0	0.0	0.0	0.0	0.0	0.0	0.0	0.0
0.0	0.0	0.0	0.0	0.0	0.0	0.0	0.0
0.0	0.0	0.0	0.0	0.0	0.0	0.0	0.0
0.0	0.0	0.0	0.0	0.0	0.0	0.0	0.0
0.0	0.0	0.0	0.0	0.0	0.0	0.0	0.0
0.0	0.0	0.0	0.0	0.0	0.0	0.0	0.0
0.0	0.0	0.0	0.0	0.0	0.0	0.0	0.0
0.0	0.0	0.0	0.0	0.0	0.0	0.0	0.0
0.0	0.0	0.0	0.0	0.0	0.0	0.0	0.0
0.0	0.0	0.0	0.0	0.0	0.0	0.0	0.0
0.0	0.0	0.0	0.0	0.0	0.0	0.0	0.0
0.0	0.0	0.0	0.0	0.0	0.0	0.0	0.0
0.0	0.0	0.0	0.0	0.0	0.0	0.0	0.0
0.0	0.0	0.0	0.0	0.0	0.0	0.0	0.0
0.0	0.0	0.0	0.0	0.0	0.0	0.0	0.0
0.0	0.0	0.0	0.0	0.0	0.0	0.0	0.0
0.0	0.0	0.0	0.0	0.0	0.0	0.0	0.0
0.0	0.0	0.0	0.0	0.0	0.0	0.0	0.0
0.0	0.0	0.0	0.0	0.0	0.0	0.0	0.0
0.0	0.0	0.0	0.0	0.0	0.0	0.0	0.0
0.0	0.0	0.0	0.0	0.0	0.0	0.0	0.0
0.0	0.0	0.0	0.0	0.0	0.0	0.0	0.0
0.0	0.0	0.0	0.0	0.0	0.0	0.0	0.0

0.0	0.0	0.0	0.0	0.0	0.0	0.0	0.0
0.0	0.0	0.0	0.0	0.0	0.0	0.0	0.0
0.0	0.0	0.0	0.0	0.0	0.0	0.0	0.0
0.0	0.0	0.0	0.0	0.0	0.0	0.0	0.0
0.0	0.0	0.0	0.0	0.0	0.0	0.0	0.0
0.0	0.0	0.0	0.0	0.0	0.0	0.0	0.0
0.0	0.0	0.0	0.0	0.0	0.0	0.0	0.0
0.0	0.0	0.0	0.0	0.0	0.0	0.0	0.0
0.0	0.0	0.0	0.0	0.0	0.0	0.0	0.0
0.0	0.0	0.0	0.0	0.0	0.0	0.0	0.0
0.0	0.0	0.0	0.0	0.0	0.0	0.0	0.0
0.0	0.0	0.0	0.0	0.0	0.0	0.0	0.0
0.0	0.0	0.0	0.0	0.0	0.0	0.0	0.0
0.0	0.0	0.0	0.0	0.0	0.0	0.0	0.0
0.0	0.0	0.0	0.0	0.0	0.0	0.0	0.0
0.0	0.0	0.0	0.0	0.0	0.0	0.0	0.0
0.0	0.0	0.0	0.0	0.0	0.0	0.0	0.0
0.0	0.0	0.0	0.0	0.0	0.0	0.0	0.0
0.0	0.0	0.0	0.0	0.0	0.0	0.0	0.0
0.0	0.0	0.0	0.0	0.0	0.0	0.0	0.0
0.0	0.0	0.0	0.0	0.0	0.0	0.0	0.0
0.0	0.0	0.0	0.0	0.0	0.0	0.0	0.0
0.0	0.0	0.0	0.0	0.0	0.0	0.0	0.0
0.0	0.0	0.0	0.0	0.0	0.0	0.0	0.0
0.0	0.0	0.0	0.0	0.0	0.0	0.0	0.0
0.0	0.0	0.0	0.0	0.0	0.0	0.0	0.0
0.0	0.0	0.0	0.0	0.0	0.0	0.0	0.0
0.0	0.0	0.0	0.0	0.0	0.0	0.0	0.0
0.0	0.0	0.0	0.0	0.0	0.0	0.0	0.0
0.0	0.0	0.0	0.0	0.0	0.0	0.0	0.0
0.0	0.0	0.0	0.0	0.0	0.0	0.0	0.0
0.0	0.0	0.0	0.0	0.0	0.0	0.0	0.0
0.0	0.0	0.0	0.0	0.0	0.0	0.0	0.0
0.0	0.0	0.0	0.0	0.0	0.0	0.0	0.0
0.0	0.0	0.0	0.0	0.0	0.0	0.0	0.0
0.0	0.0	0.0	0.0	0.0	0.0	0.0	0.0
0.0	0.0	0.0	0.0	0.0	0.0	0.0	0.0
0.0	0.0	0.0	0.0	0.0	0.0	0.0	0.0
0.0	0.0	0.0	0.0	0.0	0.0	0.0	0.0
0.0	0.0	0.0	0.0	0.0	0.0	0.0	0.0
0.0	0.0	0.0	0.0	0.0	0.0	0.0	0.0
0.0	0.0	0.0	0.0	0.0	0.0	0.0	0.0
0.0	0.0	0.0	0.0	0.0	0.0	0.0	0.0
0.0	0.0	0.0	0.0	0.0	0.0	0.0	0.0
0.0	0.0	0.0	0.0	0.0	0.0	0.0	0.0
0.0	0.0	0.0	0.0	0.0	0.0	0.0	0.0
0.0	0.0	0.0	0.0	0.0	0.0	0.0	0.0

0.0	0.0	0.0	0.0	0.0	0.0	0.0	0.0
0.0	0.0	0.0	0.0	0.0	0.0	0.0	0.0
0.0	0.0	0.0	0.0	0.0	0.0	0.0	0.0
0.0	0.0	0.0	0.0	0.0	0.0	0.0	0.0
0.0	0.0	0.0	0.0	0.0	0.0	0.0	0.0
0.0	0.0	0.0	0.0	0.0	0.0	0.0	0.0
0.0	0.0	0.0	0.0	0.0	0.0	0.0	0.0
0.0	0.0	0.0	0.0	0.0	0.0	0.0	0.0
0.0	0.0	0.0	0.0	0.0	0.0	0.0	0.0
0.0	0.0	0.0	0.0	0.0	0.0	0.0	0.0
0.0	0.0	0.0	0.0	0.0	0.0	0.0	0.0
0.0	0.0	0.0	0.0	0.0	0.0	0.0	0.0
0.0	0.0	0.0	0.0	0.0	0.0	0.0	0.0
0.0	0.0	0.0	0.0	0.0	0.0	0.0	0.0
0.0	0.0	0.0	0.0	0.0	0.0	0.0	0.0
0.0	0.0	0.0	0.0	0.0	0.0	0.0	0.0
0.0	0.0	0.0	0.0	0.0	0.0	0.0	0.0
0.0	0.0	0.0	0.0	0.0	0.0	0.0	0.0
0.0	0.0	0.0	0.0	0.0	0.0	0.0	0.0
0.0	0.0	0.0	0.0	0.0	0.0	0.0	0.0
0.0	0.0	0.0	0.0	0.0	0.0	0.0	0.0
0.0	0.0	0.0	0.0	0.0	0.0	0.0	0.0
0.0	0.0	0.0	0.0	0.0	0.0	0.0	0.0
64	21	71	79	52	29	18	25
0	0	1	1	1	1	1	1
25	29	24.6	22.97668038	24.1	19.3	23.3	20.2

11635.TW.CB.	11635.TW.EH	11635.CIR55.(	11635.CIR26.(	11635.TW.BK.	11635.TW.DA	11635.TW.AA	11635.TW.CQ
2.0	541.0	24.0	3794.0	368.0	1.0	1.0	5.0
88.0	0.0	0.0	0.0	0.0	0.0	0.0	154.0
1.0	4222.0	0.0	0.0	2.0	1.0	1.0	0.0
0.0	0.0	234.0	0.0	88.0	0.0	2.0	0.0
408.0	0.0	106.0	0.0	1218.0	95.0	0.0	311.0
2210.0	0.0	11.0	10.0	111.0	146.0	3.0	85.0
0.0	0.0	0.0	0.0	0.0	0.0	0.0	0.0
0.0	0.0	0.0	0.0	0.0	0.0	0.0	0.0
0.0	0.0	123.0	0.0	0.0	0.0	0.0	0.0
0.0	0.0	202.0	13.0	227.0	0.0	0.0	0.0
58.0	0.0	595.0	2.0	74.0	9.0	0.0	58.0
0.0	0.0	0.0	0.0	0.0	0.0	0.0	0.0
156.0	0.0	36.0	1.0	0.0	0.0	0.0	0.0
21.0	5.0	1.0	0.0	2.0	1743.0	406.0	11.0
74.0	0.0	562.0	0.0	178.0	3.0	1.0	0.0
904.0	2.0	2298.0	3.0	508.0	208.0	0.0	2932.0
0.0	0.0	0.0	22.0	5.0	0.0	0.0	0.0
1.0	7.0	0.0	0.0	30.0	3944.0	61.0	0.0
126.0	14.0	175.0	1559.0	233.0	172.0	2.0	37.0
1808.0	0.0	342.0	0.0	30.0	0.0	2.0	1301.0
8.0	0.0	46.0	261.0	570.0	0.0	0.0	0.0
0.0	0.0	0.0	0.0	0.0	0.0	0.0	0.0
0.0	0.0	8.0	4.0	2.0	0.0	0.0	0.0
0.0	0.0	2.0	0.0	0.0	0.0	0.0	0.0
0.0	0.0	0.0	0.0	0.0	0.0	0.0	0.0
17.0	0.0	10.0	144.0	1.0	0.0	5.0	0.0
0.0	0.0	0.0	0.0	0.0	0.0	0.0	0.0
97.0	0.0	113.0	0.0	20.0	0.0	0.0	49.0
0.0	0.0	0.0	0.0	0.0	0.0	0.0	0.0
0.0	0.0	0.0	0.0	24.0	0.0	0.0	4.0
35.0	173.0	23.0	144.0	9.0	11.0	4.0	83.0
7.0	5.0	86.0	161.0	16.0	0.0	0.0	31.0
654.0	0.0	3.0	0.0	13.0	0.0	0.0	0.0
0.0	0.0	0.0	0.0	0.0	0.0	0.0	0.0
0.0	0.0	63.0	0.0	134.0	0.0	16.0	40.0
1.0	0.0	6.0	0.0	0.0	0.0	0.0	0.0
0.0	0.0	0.0	0.0	0.0	0.0	0.0	0.0
14.0	126.0	62.0	759.0	17.0	1.0	6.0	84.0
52.0	0.0	0.0	0.0	0.0	0.0	0.0	0.0
5.0	0.0	19.0	96.0	38.0	1.0	0.0	0.0
120.0	0.0	39.0	1.0	0.0	72.0	1.0	223.0
0.0	0.0	0.0	0.0	0.0	0.0	0.0	0.0
14.0	8.0	30.0	78.0	21.0	15.0	5.0	6.0
228.0	2.0	23.0	638.0	68.0	3.0	2.0	570.0
3.0	0.0	17.0	1.0	2.0	2.0	0.0	14.0
0.0	0.0	41.0	0.0	9.0	0.0	5.0	0.0

4.0	4.0	6.0	2.0	6.0	1.0	2.0	7.0
0.0	0.0	0.0	0.0	0.0	0.0	0.0	389.0
0.0	0.0	0.0	0.0	0.0	0.0	0.0	0.0
0.0	0.0	0.0	0.0	0.0	0.0	0.0	0.0
0.0	0.0	0.0	0.0	0.0	0.0	0.0	0.0
24.0	0.0	8.0	57.0	0.0	2.0	0.0	6.0
0.0	0.0	7.0	6.0	35.0	0.0	0.0	0.0
0.0	0.0	0.0	0.0	0.0	0.0	0.0	0.0
0.0	0.0	0.0	0.0	0.0	0.0	0.0	0.0
0.0	0.0	6.0	0.0	0.0	0.0	0.0	0.0
0.0	0.0	0.0	0.0	0.0	0.0	0.0	2.0
1.0	0.0	0.0	0.0	2.0	0.0	6.0	0.0
0.0	0.0	0.0	0.0	0.0	0.0	0.0	0.0
0.0	0.0	0.0	0.0	0.0	0.0	0.0	0.0
0.0	0.0	0.0	0.0	20.0	0.0	0.0	0.0
0.0	0.0	0.0	0.0	0.0	0.0	0.0	0.0
0.0	0.0	0.0	0.0	1.0	2.0	0.0	0.0
0.0	0.0	0.0	0.0	0.0	0.0	0.0	0.0
0.0	0.0	9.0	0.0	212.0	0.0	4.0	0.0
38.0	0.0	0.0	0.0	0.0	0.0	0.0	0.0
0.0	0.0	0.0	0.0	0.0	0.0	0.0	0.0
0.0	0.0	0.0	0.0	1.0	0.0	1.0	0.0
0.0	0.0	0.0	0.0	0.0	0.0	0.0	0.0
0.0	0.0	12.0	198.0	58.0	0.0	0.0	0.0
0.0	0.0	0.0	0.0	0.0	0.0	0.0	0.0
0.0	0.0	0.0	0.0	0.0	0.0	0.0	24.0
0.0	0.0	0.0	0.0	0.0	0.0	0.0	0.0
0.0	0.0	0.0	0.0	0.0	0.0	0.0	0.0
0.0	0.0	0.0	0.0	0.0	6.0	0.0	20.0
0.0	0.0	0.0	0.0	0.0	0.0	0.0	0.0
0.0	0.0	0.0	0.0	0.0	0.0	0.0	0.0
0.0	0.0	0.0	0.0	0.0	0.0	0.0	0.0
0.0	0.0	0.0	0.0	0.0	0.0	0.0	0.0
0.0	0.0	0.0	0.0	0.0	0.0	0.0	0.0
75.0	0.0	0.0	0.0	58.0	0.0	14.0	30.0
0.0	0.0	0.0	0.0	0.0	0.0	0.0	0.0
0.0	0.0	2.0	0.0	4.0	24.0	0.0	0.0
0.0	0.0	0.0	0.0	0.0	0.0	0.0	0.0
58.0	0.0	0.0	0.0	83.0	46.0	4.0	0.0
1.0	0.0	0.0	0.0	153.0	0.0	0.0	0.0
0.0	0.0	0.0	0.0	74.0	0.0	0.0	0.0
0.0	0.0	0.0	0.0	0.0	0.0	0.0	0.0
33.0	0.0	161.0	0.0	66.0	0.0	0.0	0.0
111.0	0.0	0.0	0.0	0.0	0.0	0.0	0.0
0.0	0.0	6.0	0.0	12.0	56.0	3.0	49.0
0.0	0.0	0.0	0.0	0.0	0.0	0.0	0.0
0.0	0.0	26.0	0.0	10.0	1.0	6.0	0.0

8.0	0.0	14.0	209.0	35.0	0.0	0.0	0.0
56.0	0.0	12.0	0.0	10.0	0.0	0.0	0.0
15.0	0.0	680.0	0.0	177.0	187.0	1.0	0.0
0.0	0.0	0.0	0.0	0.0	0.0	0.0	0.0
10.0	0.0	0.0	0.0	76.0	23.0	0.0	0.0
0.0	0.0	0.0	0.0	0.0	0.0	0.0	0.0
0.0	0.0	10.0	0.0	75.0	49.0	0.0	0.0
0.0	0.0	0.0	0.0	0.0	0.0	0.0	0.0
45.0	0.0	5.0	0.0	0.0	0.0	3.0	0.0
0.0	0.0	0.0	0.0	2.0	16.0	0.0	0.0
0.0	0.0	9.0	0.0	94.0	0.0	0.0	0.0
0.0	0.0	0.0	0.0	0.0	0.0	0.0	0.0
0.0	0.0	0.0	0.0	135.0	0.0	0.0	0.0
0.0	0.0	0.0	0.0	37.0	0.0	0.0	0.0
76.0	0.0	14.0	0.0	77.0	0.0	0.0	0.0
0.0	0.0	0.0	0.0	0.0	0.0	0.0	0.0
0.0	0.0	0.0	0.0	0.0	0.0	0.0	0.0
0.0	0.0	0.0	0.0	0.0	0.0	0.0	0.0
0.0	0.0	0.0	0.0	0.0	0.0	0.0	0.0
0.0	0.0	0.0	0.0	0.0	0.0	0.0	0.0
0.0	1.0	9.0	75.0	11.0	0.0	0.0	32.0
0.0	0.0	0.0	0.0	0.0	0.0	0.0	0.0
41.0	0.0	25.0	0.0	20.0	0.0	0.0	0.0
0.0	0.0	0.0	0.0	0.0	0.0	0.0	0.0
0.0	0.0	0.0	0.0	0.0	0.0	0.0	0.0
0.0	0.0	0.0	0.0	0.0	0.0	0.0	0.0
0.0	0.0	126.0	0.0	0.0	0.0	0.0	0.0
0.0	0.0	0.0	0.0	264.0	0.0	0.0	0.0
0.0	0.0	1.0	0.0	2.0	21.0	0.0	14.0
112.0	0.0	0.0	0.0	0.0	0.0	5.0	0.0
0.0	0.0	0.0	0.0	0.0	0.0	0.0	0.0
0.0	0.0	0.0	0.0	0.0	0.0	0.0	0.0
0.0	0.0	0.0	0.0	0.0	0.0	0.0	0.0
0.0	0.0	0.0	0.0	0.0	0.0	0.0	0.0
0.0	0.0	0.0	0.0	0.0	10.0	0.0	0.0
0.0	0.0	0.0	0.0	8.0	0.0	0.0	0.0
0.0	0.0	0.0	0.0	0.0	0.0	0.0	0.0
0.0	0.0	0.0	0.0	2.0	0.0	0.0	0.0
0.0	2.0	31.0	0.0	0.0	0.0	0.0	0.0
0.0	0.0	0.0	0.0	0.0	0.0	0.0	0.0
0.0	0.0	0.0	0.0	0.0	0.0	0.0	0.0
162.0	2.0	0.0	0.0	875.0	413.0	0.0	0.0
0.0	0.0	0.0	0.0	0.0	0.0	0.0	0.0
0.0	0.0	0.0	0.0	0.0	0.0	0.0	0.0
39.0	0.0	9.0	0.0	0.0	19.0	0.0	0.0
0.0	0.0	0.0	0.0	0.0	0.0	0.0	0.0
5.0	0.0	62.0	0.0	29.0	2.0	0.0	69.0

0.0	0.0	0.0	0.0	0.0	0.0	0.0	0.0
0.0	0.0	0.0	0.0	0.0	0.0	0.0	0.0
0.0	0.0	0.0	0.0	0.0	0.0	0.0	0.0
0.0	0.0	0.0	0.0	0.0	0.0	0.0	0.0
0.0	0.0	0.0	0.0	48.0	0.0	1.0	0.0
0.0	0.0	0.0	0.0	0.0	0.0	0.0	0.0
0.0	0.0	0.0	0.0	0.0	0.0	0.0	0.0
19.0	0.0	0.0	0.0	0.0	2.0	0.0	0.0
4.0	0.0	17.0	0.0	23.0	18.0	0.0	11.0
0.0	0.0	0.0	0.0	0.0	0.0	0.0	0.0
69.0	0.0	52.0	0.0	25.0	11.0	0.0	0.0
0.0	0.0	25.0	0.0	17.0	0.0	0.0	0.0
0.0	0.0	10.0	0.0	9.0	0.0	0.0	0.0
0.0	0.0	61.0	0.0	0.0	2.0	0.0	80.0
51.0	0.0	18.0	0.0	0.0	0.0	0.0	0.0
0.0	0.0	0.0	0.0	0.0	0.0	8.0	0.0
0.0	0.0	0.0	0.0	0.0	0.0	0.0	0.0
0.0	0.0	0.0	0.0	4.0	0.0	0.0	12.0
1.0	5.0	3.0	0.0	4.0	3.0	1.0	54.0
0.0	0.0	0.0	0.0	1.0	0.0	0.0	7.0
4.0	0.0	1024.0	2.0	2.0	0.0	0.0	0.0
9.0	0.0	0.0	0.0	0.0	17.0	0.0	0.0
1.0	0.0	1.0	0.0	14.0	2.0	0.0	3.0
0.0	0.0	6.0	0.0	3.0	0.0	0.0	0.0
0.0	0.0	0.0	0.0	2.0	0.0	0.0	0.0
0.0	0.0	0.0	0.0	12.0	66.0	0.0	0.0
0.0	0.0	0.0	0.0	5.0	0.0	0.0	0.0
0.0	0.0	4.0	0.0	0.0	0.0	0.0	0.0
0.0	0.0	0.0	0.0	1.0	0.0	0.0	0.0
0.0	0.0	0.0	0.0	0.0	0.0	0.0	0.0
0.0	0.0	0.0	0.0	0.0	11.0	0.0	0.0
0.0	0.0	0.0	0.0	3.0	0.0	0.0	0.0
0.0	0.0	0.0	0.0	0.0	0.0	0.0	0.0
0.0	0.0	0.0	0.0	0.0	0.0	0.0	0.0
4.0	0.0	0.0	0.0	5.0	0.0	0.0	12.0
0.0	0.0	0.0	0.0	3.0	0.0	0.0	0.0
27.0	0.0	0.0	27.0	34.0	3.0	0.0	30.0
0.0	0.0	21.0	0.0	8.0	0.0	0.0	0.0
16.0	0.0	42.0	0.0	64.0	7.0	0.0	0.0
0.0	0.0	3.0	0.0	6.0	0.0	0.0	0.0
4.0	0.0	0.0	0.0	10.0	7.0	2.0	1.0
29.0	0.0	8.0	0.0	32.0	0.0	0.0	5.0
10.0	0.0	5.0	0.0	1.0	3.0	0.0	0.0
0.0	0.0	0.0	0.0	0.0	0.0	0.0	0.0
0.0	1.0	28.0	104.0	292.0	0.0	0.0	0.0
0.0	0.0	0.0	0.0	0.0	0.0	0.0	0.0
0.0	0.0	0.0	0.0	0.0	0.0	0.0	0.0

0.0	0.0	0.0	0.0	0.0	0.0	0.0	0.0
0.0	0.0	0.0	0.0	37.0	0.0	0.0	0.0
0.0	0.0	0.0	0.0	35.0	16.0	0.0	0.0
0.0	0.0	72.0	0.0	0.0	0.0	0.0	0.0
0.0	0.0	0.0	0.0	23.0	0.0	0.0	0.0
0.0	0.0	0.0	0.0	0.0	0.0	0.0	0.0
0.0	0.0	1.0	0.0	0.0	0.0	0.0	0.0
23.0	0.0	41.0	0.0	17.0	7.0	0.0	23.0
4.0	0.0	24.0	0.0	9.0	1.0	0.0	95.0
0.0	0.0	0.0	0.0	1.0	0.0	0.0	0.0
9.0	0.0	0.0	0.0	0.0	0.0	0.0	0.0
3.0	0.0	28.0	0.0	0.0	0.0	0.0	14.0
2.0	0.0	0.0	0.0	0.0	1.0	0.0	0.0
4.0	10.0	0.0	204.0	1.0	17.0	0.0	178.0
4.0	0.0	13.0	0.0	2.0	0.0	0.0	10.0
0.0	0.0	5.0	11.0	2.0	0.0	0.0	17.0
0.0	0.0	0.0	0.0	0.0	0.0	0.0	0.0
0.0	0.0	0.0	0.0	0.0	0.0	0.0	0.0
0.0	0.0	0.0	0.0	0.0	0.0	0.0	0.0
0.0	0.0	9.0	20.0	3.0	3.0	0.0	42.0
0.0	0.0	0.0	44.0	1.0	6.0	4.0	0.0
1.0	0.0	0.0	0.0	0.0	0.0	0.0	0.0
0.0	0.0	0.0	0.0	0.0	0.0	0.0	0.0
0.0	0.0	4.0	44.0	0.0	0.0	0.0	0.0
0.0	0.0	0.0	0.0	0.0	0.0	0.0	0.0
0.0	0.0	0.0	0.0	0.0	0.0	0.0	0.0
0.0	0.0	0.0	0.0	0.0	0.0	0.0	0.0
0.0	0.0	0.0	0.0	0.0	0.0	0.0	0.0
0.0	0.0	0.0	0.0	0.0	0.0	0.0	0.0
0.0	0.0	0.0	0.0	0.0	0.0	0.0	0.0
0.0	0.0	0.0	0.0	0.0	0.0	0.0	0.0
0.0	0.0	0.0	0.0	0.0	0.0	0.0	0.0
0.0	0.0	0.0	0.0	0.0	0.0	0.0	0.0
0.0	0.0	0.0	0.0	0.0	0.0	0.0	0.0
0.0	0.0	0.0	0.0	0.0	0.0	0.0	0.0
0.0	0.0	0.0	0.0	0.0	0.0	0.0	0.0
0.0	0.0	0.0	0.0	0.0	0.0	0.0	0.0
0.0	0.0	0.0	0.0	0.0	0.0	0.0	0.0
0.0	0.0	0.0	0.0	1.0	0.0	0.0	0.0
2.0	0.0	2.0	0.0	6.0	2.0	0.0	4.0
0.0	0.0	5.0	0.0	4.0	0.0	0.0	0.0
0.0	0.0	0.0	0.0	0.0	0.0	0.0	0.0
0.0	0.0	0.0	0.0	0.0	0.0	0.0	0.0
0.0	0.0	0.0	0.0	0.0	0.0	0.0	0.0
0.0	0.0	0.0	0.0	0.0	0.0	0.0	0.0
0.0	0.0	19.0	0.0	0.0	0.0	0.0	0.0

0.0	0.0	1.0	0.0	13.0	6.0	0.0	0.0
0.0	0.0	0.0	0.0	6.0	0.0	0.0	0.0
0.0	0.0	0.0	0.0	0.0	48.0	0.0	0.0
16.0	0.0	0.0	0.0	0.0	0.0	0.0	0.0
0.0	0.0	0.0	0.0	0.0	0.0	0.0	0.0
0.0	0.0	7.0	0.0	0.0	0.0	0.0	0.0
0.0	0.0	1.0	0.0	2.0	0.0	0.0	0.0
0.0	0.0	0.0	0.0	2.0	0.0	0.0	0.0
0.0	0.0	0.0	0.0	0.0	0.0	0.0	0.0
0.0	0.0	0.0	0.0	0.0	0.0	0.0	0.0
0.0	0.0	0.0	0.0	0.0	0.0	0.0	0.0
0.0	0.0	0.0	0.0	0.0	0.0	0.0	0.0
0.0	0.0	0.0	0.0	0.0	0.0	0.0	0.0
0.0	0.0	0.0	0.0	0.0	0.0	0.0	0.0
0.0	0.0	0.0	0.0	0.0	0.0	0.0	0.0
0.0	0.0	0.0	0.0	0.0	0.0	0.0	0.0
0.0	0.0	0.0	0.0	1.0	0.0	0.0	0.0
0.0	0.0	0.0	0.0	0.0	0.0	0.0	0.0
0.0	0.0	5.0	0.0	0.0	2.0	0.0	1.0
0.0	0.0	0.0	0.0	0.0	0.0	0.0	0.0
0.0	0.0	0.0	0.0	3.0	0.0	0.0	0.0
0.0	0.0	0.0	0.0	0.0	0.0	0.0	0.0
2.0	0.0	0.0	0.0	0.0	1.0	0.0	0.0
0.0	0.0	0.0	0.0	0.0	0.0	0.0	0.0
0.0	0.0	0.0	0.0	0.0	0.0	0.0	0.0
0.0	0.0	0.0	0.0	0.0	0.0	0.0	0.0
0.0	1.0	1.0	0.0	1.0	0.0	1.0	3.0
0.0	0.0	0.0	0.0	0.0	0.0	0.0	0.0
0.0	0.0	1.0	0.0	0.0	0.0	0.0	2.0
0.0	0.0	0.0	0.0	0.0	0.0	0.0	0.0
0.0	0.0	0.0	0.0	0.0	0.0	0.0	0.0
0.0	0.0	0.0	1.0	0.0	0.0	0.0	0.0
0.0	0.0	0.0	0.0	0.0	0.0	0.0	0.0
0.0	0.0	0.0	0.0	0.0	0.0	0.0	0.0
0.0	0.0	0.0	0.0	0.0	0.0	0.0	0.0
0.0	0.0	1.0	0.0	0.0	0.0	0.0	0.0
0.0	0.0	0.0	0.0	15.0	0.0	0.0	0.0
0.0	0.0	0.0	0.0	20.0	0.0	0.0	0.0
0.0	0.0	0.0	0.0	0.0	0.0	0.0	0.0
0.0	0.0	0.0	0.0	0.0	0.0	0.0	0.0
0.0	0.0	0.0	0.0	0.0	0.0	0.0	0.0
0.0	0.0	0.0	0.0	0.0	0.0	0.0	0.0
7.0	0.0	8.0	0.0	7.0	0.0	0.0	23.0
3.0	0.0	11.0	0.0	0.0	4.0	0.0	34.0
0.0	0.0	0.0	0.0	0.0	0.0	0.0	0.0
0.0	0.0	0.0	0.0	0.0	3.0	0.0	0.0
0.0	0.0	0.0	0.0	0.0	0.0	0.0	0.0

0.0	0.0	0.0	0.0	0.0	0.0	0.0	0.0
0.0	0.0	0.0	0.0	0.0	0.0	0.0	0.0
0.0	0.0	0.0	0.0	0.0	0.0	0.0	0.0
0.0	0.0	0.0	0.0	0.0	0.0	0.0	0.0
0.0	0.0	0.0	0.0	1.0	0.0	0.0	0.0
0.0	0.0	0.0	0.0	0.0	0.0	0.0	0.0
2.0	0.0	0.0	0.0	6.0	0.0	0.0	0.0
0.0	0.0	0.0	0.0	0.0	0.0	0.0	0.0
0.0	0.0	0.0	0.0	0.0	0.0	0.0	0.0
0.0	0.0	0.0	0.0	0.0	0.0	0.0	0.0
0.0	0.0	0.0	0.0	0.0	0.0	0.0	0.0
0.0	0.0	0.0	0.0	8.0	0.0	0.0	0.0
0.0	0.0	0.0	0.0	0.0	0.0	0.0	0.0
0.0	0.0	0.0	0.0	0.0	0.0	0.0	0.0
0.0	0.0	0.0	0.0	0.0	0.0	0.0	0.0
7.0	0.0	3.0	0.0	4.0	0.0	87.0	0.0
0.0	0.0	0.0	0.0	0.0	0.0	0.0	0.0
0.0	0.0	0.0	0.0	0.0	12.0	0.0	0.0
0.0	0.0	0.0	0.0	0.0	0.0	0.0	0.0
0.0	0.0	0.0	1.0	0.0	0.0	0.0	7.0
0.0	0.0	0.0	0.0	76.0	0.0	0.0	0.0
0.0	0.0	0.0	0.0	0.0	0.0	0.0	0.0
0.0	0.0	0.0	0.0	0.0	0.0	0.0	0.0
0.0	0.0	0.0	0.0	0.0	0.0	0.0	0.0
0.0	0.0	0.0	10.0	0.0	2.0	0.0	0.0
0.0	0.0	0.0	0.0	0.0	0.0	0.0	0.0
0.0	1.0	1.0	3.0	0.0	0.0	0.0	0.0
3.0	0.0	0.0	27.0	0.0	0.0	0.0	5.0
0.0	0.0	0.0	0.0	0.0	0.0	0.0	0.0
0.0	0.0	0.0	0.0	0.0	0.0	0.0	0.0
0.0	0.0	0.0	0.0	0.0	0.0	0.0	0.0
0.0	0.0	0.0	0.0	0.0	0.0	0.0	0.0
0.0	0.0	0.0	0.0	0.0	0.0	0.0	0.0
0.0	0.0	0.0	0.0	8.0	1.0	0.0	4.0
0.0	0.0	0.0	0.0	0.0	0.0	0.0	0.0
0.0	0.0	0.0	0.0	0.0	0.0	0.0	0.0
0.0	0.0	0.0	0.0	0.0	0.0	0.0	0.0
0.0	0.0	0.0	0.0	0.0	0.0	0.0	0.0
0.0	0.0	0.0	0.0	0.0	0.0	0.0	0.0
0.0	0.0	0.0	0.0	2.0	0.0	0.0	0.0
4.0	0.0	2.0	0.0	6.0	0.0	0.0	0.0
0.0	0.0	0.0	0.0	0.0	0.0	0.0	0.0
0.0	0.0	0.0	0.0	0.0	0.0	0.0	0.0
2.0	0.0	1.0	2.0	5.0	1.0	0.0	0.0
0.0	0.0	0.0	0.0	0.0	0.0	0.0	0.0
0.0	0.0	0.0	0.0	0.0	0.0	0.0	0.0
0.0	0.0	0.0	0.0	17.0	0.0	0.0	0.0

0.0	0.0	0.0	0.0	0.0	0.0	0.0	0.0
0.0	0.0	0.0	0.0	0.0	0.0	0.0	0.0
0.0	0.0	0.0	0.0	0.0	0.0	0.0	0.0
0.0	0.0	0.0	0.0	0.0	0.0	0.0	0.0
0.0	0.0	0.0	0.0	0.0	0.0	0.0	0.0
0.0	0.0	0.0	0.0	0.0	0.0	0.0	0.0
0.0	0.0	2.0	0.0	1.0	0.0	0.0	0.0
0.0	0.0	0.0	0.0	0.0	0.0	0.0	0.0
0.0	22.0	0.0	14.0	0.0	22.0	1.0	1.0
0.0	0.0	0.0	0.0	0.0	0.0	0.0	0.0
0.0	0.0	0.0	0.0	0.0	0.0	0.0	0.0
0.0	0.0	0.0	0.0	0.0	0.0	0.0	0.0
0.0	0.0	0.0	0.0	0.0	0.0	0.0	0.0
0.0	0.0	0.0	0.0	0.0	0.0	0.0	0.0
0.0	0.0	0.0	0.0	1.0	0.0	0.0	0.0
0.0	0.0	0.0	0.0	0.0	0.0	0.0	0.0
2.0	0.0	0.0	0.0	3.0	2.0	0.0	0.0
0.0	0.0	0.0	0.0	0.0	0.0	0.0	0.0
0.0	0.0	0.0	0.0	0.0	0.0	0.0	0.0
0.0	0.0	0.0	0.0	0.0	0.0	0.0	0.0
0.0	0.0	0.0	0.0	0.0	0.0	0.0	0.0
0.0	0.0	0.0	0.0	0.0	0.0	0.0	0.0
0.0	0.0	0.0	0.0	0.0	0.0	0.0	0.0
0.0	0.0	0.0	0.0	0.0	0.0	0.0	0.0
0.0	0.0	0.0	0.0	0.0	0.0	0.0	0.0
0.0	0.0	0.0	0.0	0.0	0.0	0.0	0.0
0.0	0.0	0.0	0.0	0.0	0.0	0.0	0.0
4.0	0.0	30.0	0.0	6.0	2.0	0.0	56.0
0.0	0.0	0.0	0.0	0.0	0.0	0.0	0.0
0.0	0.0	0.0	0.0	0.0	0.0	0.0	0.0
0.0	0.0	0.0	0.0	0.0	0.0	0.0	0.0
0.0	0.0	0.0	0.0	0.0	0.0	0.0	0.0
0.0	0.0	0.0	0.0	0.0	0.0	0.0	0.0
0.0	0.0	0.0	0.0	1.0	0.0	0.0	0.0
1.0	0.0	0.0	0.0	0.0	0.0	0.0	0.0
0.0	0.0	0.0	0.0	0.0	0.0	0.0	0.0
0.0	0.0	0.0	0.0	0.0	0.0	0.0	0.0
0.0	0.0	0.0	0.0	0.0	0.0	0.0	0.0
0.0	0.0	0.0	0.0	0.0	0.0	0.0	1.0
0.0	0.0	0.0	0.0	0.0	0.0	0.0	0.0
0.0	0.0	0.0	0.0	0.0	0.0	0.0	0.0
0.0	0.0	0.0	0.0	0.0	0.0	0.0	0.0
0.0	0.0	0.0	0.0	0.0	0.0	0.0	0.0
0.0	0.0	0.0	0.0	0.0	0.0	0.0	0.0
0.0	0.0	0.0	0.0	0.0	0.0	0.0	0.0
2.0	0.0	0.0	0.0	1.0	0.0	0.0	0.0
0.0	0.0	0.0	0.0	0.0	0.0	0.0	0.0

0.0	0.0	0.0	0.0	1.0	0.0	0.0	0.0
0.0	0.0	0.0	0.0	0.0	0.0	0.0	0.0
0.0	0.0	0.0	0.0	0.0	0.0	0.0	0.0
0.0	0.0	0.0	0.0	0.0	2.0	0.0	0.0
0.0	0.0	0.0	0.0	0.0	0.0	0.0	0.0
0.0	0.0	0.0	0.0	0.0	0.0	0.0	0.0
0.0	0.0	0.0	0.0	0.0	0.0	0.0	0.0
0.0	0.0	0.0	0.0	0.0	0.0	0.0	1.0
0.0	0.0	0.0	0.0	0.0	0.0	0.0	0.0
0.0	0.0	0.0	0.0	2.0	0.0	0.0	0.0
0.0	0.0	0.0	0.0	5.0	0.0	0.0	0.0
0.0	0.0	0.0	0.0	0.0	0.0	0.0	0.0
0.0	0.0	0.0	0.0	0.0	0.0	0.0	0.0
0.0	0.0	0.0	0.0	0.0	0.0	0.0	0.0
0.0	0.0	0.0	0.0	67.0	0.0	0.0	0.0
0.0	0.0	1.0	0.0	0.0	0.0	0.0	0.0
0.0	0.0	0.0	0.0	0.0	0.0	0.0	0.0
0.0	0.0	0.0	0.0	0.0	0.0	0.0	0.0
0.0	0.0	0.0	0.0	0.0	0.0	0.0	0.0
0.0	0.0	0.0	0.0	0.0	0.0	0.0	0.0
0.0	0.0	0.0	0.0	0.0	0.0	0.0	0.0
0.0	0.0	0.0	0.0	0.0	0.0	0.0	0.0
0.0	0.0	0.0	0.0	0.0	0.0	0.0	0.0
0.0	22.0	0.0	0.0	178.0	0.0	0.0	0.0
0.0	0.0	0.0	0.0	0.0	2.0	0.0	0.0
0.0	0.0	0.0	0.0	0.0	0.0	0.0	0.0
0.0	0.0	0.0	0.0	0.0	0.0	0.0	0.0
0.0	0.0	0.0	0.0	0.0	0.0	0.0	0.0
0.0	0.0	0.0	0.0	0.0	0.0	0.0	0.0
0.0	0.0	3.0	0.0	2.0	0.0	0.0	0.0
0.0	0.0	0.0	0.0	0.0	0.0	0.0	0.0
0.0	0.0	0.0	0.0	0.0	0.0	0.0	0.0
0.0	0.0	0.0	0.0	0.0	649.0	0.0	0.0
0.0	0.0	0.0	0.0	0.0	0.0	0.0	0.0
0.0	0.0	0.0	0.0	0.0	0.0	0.0	0.0
0.0	0.0	0.0	0.0	0.0	0.0	0.0	0.0
0.0	0.0	0.0	0.0	0.0	0.0	1.0	0.0
0.0	0.0	0.0	0.0	0.0	0.0	0.0	0.0
0.0	0.0	0.0	9.0	0.0	0.0	0.0	0.0
0.0	0.0	0.0	0.0	0.0	0.0	0.0	0.0
0.0	0.0	0.0	0.0	0.0	0.0	0.0	0.0
0.0	0.0	0.0	0.0	227.0	227.0	0.0	0.0
0.0	0.0	0.0	0.0	0.0	13.0	0.0	0.0
0.0	0.0	0.0	0.0	0.0	0.0	0.0	0.0
0.0	0.0	0.0	0.0	0.0	0.0	0.0	0.0
0.0	47.0	0.0	0.0	0.0	0.0	0.0	0.0
3.0	0.0	1.0	2.0	3.0	0.0	0.0	0.0

1.0	0.0	1.0	0.0	0.0	0.0	0.0	0.0
0.0	0.0	0.0	0.0	0.0	0.0	0.0	4.0
3.0	0.0	0.0	0.0	0.0	2.0	0.0	0.0
3.0	1.0	2.0	0.0	0.0	0.0	0.0	2.0
0.0	0.0	0.0	0.0	0.0	0.0	0.0	0.0
0.0	0.0	0.0	0.0	0.0	0.0	0.0	0.0
0.0	0.0	0.0	0.0	0.0	0.0	0.0	0.0
0.0	0.0	0.0	0.0	0.0	0.0	0.0	0.0
0.0	0.0	0.0	0.0	0.0	0.0	0.0	0.0
0.0	0.0	0.0	0.0	0.0	0.0	0.0	0.0
0.0	0.0	0.0	0.0	0.0	0.0	0.0	0.0
0.0	0.0	0.0	0.0	0.0	0.0	0.0	0.0
6.0	0.0	0.0	0.0	2.0	0.0	0.0	0.0
0.0	0.0	0.0	0.0	0.0	0.0	0.0	0.0
0.0	0.0	0.0	0.0	0.0	0.0	0.0	0.0
0.0	0.0	0.0	0.0	0.0	0.0	0.0	0.0
0.0	0.0	0.0	0.0	0.0	0.0	0.0	0.0
0.0	0.0	0.0	0.0	0.0	0.0	0.0	0.0
29.0	0.0	0.0	0.0	0.0	0.0	0.0	0.0
0.0	0.0	0.0	0.0	0.0	0.0	0.0	63.0
8.0	0.0	12.0	0.0	0.0	0.0	0.0	0.0
0.0	0.0	0.0	0.0	0.0	0.0	0.0	0.0
0.0	0.0	0.0	0.0	0.0	0.0	0.0	0.0
0.0	0.0	0.0	0.0	0.0	0.0	0.0	0.0
0.0	0.0	0.0	0.0	0.0	0.0	0.0	0.0
0.0	0.0	0.0	0.0	0.0	0.0	0.0	0.0
0.0	0.0	0.0	0.0	0.0	0.0	0.0	0.0
0.0	0.0	0.0	0.0	0.0	0.0	0.0	0.0
0.0	0.0	0.0	0.0	2.0	0.0	0.0	0.0
0.0	0.0	0.0	0.0	0.0	0.0	0.0	0.0
0.0	0.0	0.0	0.0	0.0	11.0	0.0	0.0
0.0	0.0	0.0	0.0	4.0	0.0	0.0	0.0
0.0	0.0	0.0	0.0	0.0	0.0	0.0	0.0
0.0	0.0	0.0	0.0	0.0	0.0	0.0	0.0
0.0	0.0	0.0	0.0	4.0	0.0	0.0	0.0
0.0	0.0	0.0	0.0	0.0	0.0	0.0	0.0
0.0	0.0	0.0	9.0	5.0	0.0	0.0	0.0
0.0	0.0	0.0	4.0	1.0	0.0	0.0	0.0
0.0	0.0	0.0	0.0	2.0	0.0	254.0	0.0
0.0	0.0	0.0	0.0	0.0	0.0	0.0	0.0
0.0	0.0	0.0	0.0	0.0	0.0	0.0	0.0
0.0	0.0	0.0	0.0	0.0	0.0	0.0	0.0
0.0	0.0	0.0	0.0	0.0	0.0	0.0	0.0
0.0	0.0	0.0	0.0	0.0	0.0	0.0	0.0
46.0	0.0	0.0	0.0	6.0	22.0	0.0	0.0
2.0	0.0	6.0	0.0	0.0	5.0	0.0	0.0
0.0	0.0	0.0	0.0	0.0	0.0	0.0	0.0

0.0	0.0	0.0	0.0	0.0	0.0	0.0	0.0
0.0	0.0	0.0	0.0	4.0	0.0	0.0	0.0
0.0	0.0	0.0	0.0	1.0	0.0	0.0	0.0
1.0	0.0	1.0	0.0	5.0	0.0	0.0	0.0
0.0	0.0	0.0	0.0	0.0	2.0	0.0	34.0
0.0	0.0	0.0	0.0	0.0	0.0	0.0	0.0
0.0	0.0	0.0	0.0	1.0	0.0	0.0	0.0
0.0	0.0	0.0	0.0	0.0	0.0	0.0	0.0
0.0	0.0	0.0	0.0	0.0	0.0	0.0	0.0
0.0	0.0	0.0	0.0	0.0	0.0	0.0	0.0
0.0	0.0	0.0	0.0	0.0	0.0	0.0	0.0
0.0	0.0	0.0	0.0	0.0	0.0	4.0	0.0
1.0	0.0	0.0	0.0	1.0	0.0	0.0	0.0
0.0	0.0	1.0	0.0	0.0	117.0	0.0	0.0
0.0	0.0	0.0	1.0	1.0	2.0	639.0	0.0
0.0	0.0	0.0	0.0	0.0	0.0	0.0	0.0
0.0	0.0	0.0	0.0	0.0	0.0	0.0	0.0
0.0	0.0	0.0	0.0	2.0	0.0	0.0	0.0
0.0	0.0	0.0	0.0	1.0	0.0	0.0	0.0
1.0	0.0	1.0	0.0	0.0	0.0	0.0	1.0
0.0	0.0	0.0	0.0	0.0	0.0	0.0	0.0
0.0	0.0	0.0	0.0	0.0	0.0	4.0	0.0
0.0	0.0	0.0	0.0	0.0	0.0	0.0	0.0
0.0	0.0	0.0	0.0	0.0	0.0	0.0	0.0
3.0	0.0	9.0	0.0	3.0	0.0	0.0	5.0
0.0	0.0	0.0	0.0	0.0	0.0	0.0	0.0
0.0	0.0	0.0	0.0	0.0	0.0	0.0	0.0
2.0	0.0	0.0	0.0	1.0	1.0	0.0	0.0
0.0	0.0	0.0	0.0	0.0	0.0	0.0	0.0
0.0	0.0	0.0	0.0	0.0	0.0	0.0	0.0
0.0	0.0	0.0	0.0	0.0	0.0	0.0	0.0
1.0	0.0	0.0	0.0	0.0	18.0	0.0	0.0
0.0	0.0	0.0	0.0	0.0	0.0	0.0	0.0
0.0	0.0	0.0	0.0	0.0	0.0	0.0	0.0
0.0	0.0	0.0	0.0	0.0	0.0	0.0	0.0
0.0	0.0	0.0	0.0	0.0	0.0	0.0	0.0
0.0	0.0	0.0	0.0	0.0	0.0	0.0	0.0
0.0	0.0	0.0	0.0	0.0	0.0	0.0	0.0
0.0	0.0	0.0	0.0	0.0	0.0	0.0	0.0
0.0	0.0	0.0	0.0	0.0	0.0	0.0	0.0
0.0	0.0	0.0	0.0	0.0	0.0	0.0	0.0
0.0	0.0	0.0	0.0	1.0	0.0	1.0	0.0
0.0	0.0	3.0	0.0	0.0	6.0	0.0	1.0
0.0	0.0	0.0	0.0	0.0	0.0	0.0	0.0
0.0	0.0	4.0	0.0	0.0	0.0	0.0	0.0
0.0	0.0	0.0	0.0	0.0	0.0	0.0	0.0
0.0	0.0	0.0	0.0	0.0	0.0	0.0	0.0

5.0	0.0	0.0	0.0	0.0	1.0	0.0	0.0
0.0	0.0	0.0	0.0	0.0	0.0	0.0	0.0
0.0	0.0	0.0	0.0	0.0	0.0	0.0	0.0
0.0	3.0	0.0	3.0	0.0	7.0	0.0	0.0
1.0	0.0	0.0	3.0	1.0	2.0	0.0	0.0
0.0	0.0	0.0	0.0	0.0	0.0	0.0	0.0
0.0	3.0	0.0	0.0	0.0	0.0	0.0	0.0
0.0	0.0	0.0	0.0	0.0	0.0	0.0	0.0
0.0	0.0	0.0	0.0	0.0	0.0	0.0	0.0
0.0	0.0	0.0	0.0	0.0	0.0	0.0	0.0
0.0	0.0	0.0	0.0	0.0	0.0	0.0	0.0
0.0	0.0	0.0	0.0	0.0	0.0	0.0	0.0
0.0	0.0	0.0	0.0	0.0	0.0	0.0	0.0
0.0	0.0	0.0	0.0	0.0	0.0	0.0	0.0
0.0	0.0	0.0	0.0	0.0	0.0	0.0	0.0
0.0	0.0	0.0	0.0	0.0	0.0	0.0	0.0
0.0	0.0	0.0	0.0	0.0	0.0	0.0	0.0
0.0	0.0	0.0	0.0	0.0	0.0	0.0	0.0
0.0	0.0	0.0	0.0	0.0	0.0	0.0	0.0
0.0	0.0	0.0	0.0	1.0	0.0	0.0	0.0
0.0	0.0	0.0	0.0	0.0	0.0	0.0	0.0
0.0	0.0	0.0	0.0	0.0	0.0	0.0	0.0
0.0	0.0	0.0	0.0	0.0	0.0	0.0	0.0
0.0	0.0	0.0	0.0	0.0	0.0	0.0	0.0
0.0	0.0	0.0	0.0	0.0	0.0	0.0	0.0
0.0	0.0	0.0	0.0	0.0	0.0	0.0	0.0
0.0	0.0	0.0	0.0	0.0	0.0	0.0	0.0
0.0	0.0	0.0	0.0	0.0	0.0	803.0	0.0
0.0	0.0	0.0	0.0	0.0	0.0	0.0	0.0
0.0	0.0	0.0	0.0	0.0	0.0	0.0	0.0
0.0	0.0	0.0	0.0	0.0	0.0	0.0	0.0
0.0	0.0	0.0	0.0	0.0	0.0	0.0	0.0
0.0	0.0	0.0	0.0	0.0	0.0	0.0	0.0
0.0	0.0	451.0	0.0	0.0	0.0	0.0	0.0
0.0	15.0	0.0	0.0	0.0	0.0	0.0	0.0
0.0	0.0	0.0	0.0	0.0	0.0	0.0	0.0
0.0	0.0	0.0	0.0	0.0	0.0	0.0	0.0
0.0	0.0	0.0	0.0	0.0	0.0	0.0	0.0
0.0	0.0	0.0	0.0	0.0	0.0	0.0	0.0
1.0	0.0	0.0	0.0	0.0	0.0	0.0	0.0
0.0	0.0	0.0	0.0	0.0	0.0	0.0	0.0
0.0	0.0	0.0	0.0	0.0	0.0	0.0	0.0
0.0	0.0	0.0	0.0	2.0	0.0	0.0	0.0
0.0	0.0	0.0	0.0	0.0	0.0	0.0	0.0
0.0	0.0	0.0	0.0	0.0	0.0	0.0	0.0
0.0	2.0	0.0	0.0	0.0	0.0	0.0	0.0
0.0	0.0	0.0	0.0	0.0	0.0	0.0	0.0

0.0	0.0	0.0	0.0	0.0	0.0	0.0	0.0
0.0	0.0	0.0	0.0	0.0	0.0	0.0	0.0
0.0	0.0	0.0	0.0	0.0	0.0	0.0	0.0
0.0	0.0	0.0	0.0	0.0	0.0	0.0	0.0
0.0	0.0	0.0	0.0	0.0	0.0	0.0	0.0
0.0	0.0	0.0	0.0	0.0	0.0	0.0	0.0
1.0	12.0	1.0	9.0	4.0	8.0	0.0	77.0
0.0	9.0	0.0	20.0	1.0	0.0	0.0	4.0
0.0	36.0	0.0	0.0	0.0	5.0	0.0	10.0
0.0	0.0	0.0	0.0	0.0	0.0	0.0	0.0
0.0	0.0	0.0	0.0	0.0	0.0	2.0	0.0
0.0	0.0	0.0	0.0	0.0	0.0	0.0	0.0
0.0	0.0	0.0	0.0	0.0	0.0	0.0	0.0
2.0	3.0	0.0	0.0	0.0	0.0	0.0	0.0
0.0	0.0	0.0	0.0	0.0	0.0	0.0	0.0
1.0	0.0	0.0	0.0	0.0	0.0	0.0	0.0
0.0	0.0	1.0	0.0	0.0	0.0	0.0	0.0
0.0	0.0	0.0	0.0	0.0	0.0	0.0	0.0
0.0	0.0	0.0	0.0	0.0	8.0	0.0	0.0
0.0	0.0	0.0	0.0	0.0	0.0	0.0	0.0
0.0	0.0	0.0	0.0	0.0	0.0	0.0	0.0
0.0	0.0	0.0	0.0	0.0	0.0	0.0	0.0
0.0	0.0	0.0	1.0	0.0	0.0	0.0	1.0
0.0	0.0	2.0	0.0	0.0	0.0	0.0	0.0
0.0	0.0	0.0	0.0	0.0	0.0	0.0	0.0
0.0	0.0	0.0	0.0	0.0	0.0	0.0	0.0
0.0	0.0	0.0	1.0	0.0	0.0	0.0	0.0
0.0	0.0	0.0	0.0	0.0	0.0	0.0	0.0
0.0	0.0	0.0	0.0	0.0	0.0	0.0	0.0
0.0	0.0	0.0	0.0	0.0	0.0	0.0	0.0
0.0	0.0	0.0	0.0	0.0	0.0	0.0	0.0
0.0	0.0	0.0	0.0	0.0	0.0	0.0	0.0
0.0	0.0	0.0	0.0	0.0	0.0	0.0	0.0
0.0	0.0	0.0	0.0	0.0	0.0	0.0	0.0
0.0	0.0	0.0	0.0	0.0	0.0	0.0	0.0
0.0	0.0	0.0	0.0	0.0	0.0	0.0	0.0
3.0	0.0	0.0	0.0	0.0	0.0	0.0	0.0
0.0	0.0	0.0	0.0	0.0	0.0	0.0	0.0
0.0	0.0	0.0	0.0	0.0	0.0	0.0	0.0
0.0	0.0	0.0	0.0	0.0	0.0	0.0	0.0
0.0	0.0	0.0	0.0	0.0	0.0	0.0	0.0
0.0	0.0	0.0	0.0	0.0	0.0	0.0	0.0
0.0	0.0	0.0	0.0	0.0	0.0	0.0	0.0
0.0	0.0	0.0	0.0	0.0	0.0	0.0	0.0
0.0	0.0	0.0	0.0	0.0	0.0	0.0	0.0
1.0	0.0	0.0	0.0	2.0	0.0	0.0	29.0
29.0	0.0	0.0	0.0	0.0	0.0	0.0	0.0

0.0	0.0	0.0	0.0	0.0	0.0	0.0	0.0
0.0	0.0	0.0	0.0	112.0	0.0	0.0	0.0
0.0	0.0	0.0	0.0	0.0	0.0	0.0	0.0
0.0	0.0	0.0	0.0	0.0	0.0	0.0	0.0
0.0	0.0	0.0	2.0	4.0	0.0	0.0	0.0
0.0	0.0	0.0	0.0	0.0	0.0	0.0	0.0
0.0	0.0	3.0	0.0	0.0	0.0	0.0	0.0
0.0	0.0	0.0	0.0	0.0	0.0	0.0	0.0
0.0	0.0	0.0	0.0	4.0	0.0	0.0	0.0
0.0	0.0	0.0	0.0	0.0	0.0	0.0	0.0
0.0	0.0	0.0	0.0	0.0	0.0	0.0	0.0
2.0	0.0	0.0	0.0	0.0	0.0	0.0	0.0
0.0	0.0	0.0	0.0	0.0	0.0	0.0	0.0
0.0	0.0	0.0	0.0	0.0	0.0	0.0	0.0
0.0	0.0	2.0	0.0	0.0	0.0	0.0	0.0
0.0	0.0	0.0	0.0	0.0	0.0	0.0	0.0
0.0	0.0	0.0	0.0	0.0	0.0	0.0	0.0
0.0	0.0	0.0	0.0	0.0	0.0	0.0	0.0
0.0	0.0	0.0	0.0	0.0	0.0	0.0	0.0
0.0	0.0	0.0	0.0	0.0	0.0	0.0	0.0
0.0	0.0	0.0	0.0	0.0	0.0	0.0	0.0
0.0	0.0	0.0	0.0	0.0	0.0	0.0	0.0
0.0	0.0	0.0	0.0	0.0	41.0	0.0	0.0
0.0	0.0	0.0	0.0	103.0	0.0	0.0	0.0
1.0	0.0	0.0	44.0	0.0	0.0	0.0	456.0
0.0	0.0	0.0	0.0	0.0	0.0	0.0	0.0
13.0	0.0	1.0	0.0	0.0	0.0	0.0	0.0
0.0	0.0	0.0	0.0	0.0	0.0	0.0	0.0
10.0	0.0	1.0	0.0	3.0	0.0	0.0	0.0
0.0	0.0	0.0	0.0	30.0	0.0	0.0	0.0
3.0	0.0	0.0	0.0	0.0	0.0	0.0	0.0
0.0	0.0	0.0	0.0	0.0	0.0	0.0	0.0
0.0	0.0	0.0	0.0	0.0	0.0	0.0	0.0
0.0	0.0	0.0	0.0	0.0	0.0	0.0	0.0
0.0	0.0	0.0	0.0	0.0	0.0	0.0	0.0
0.0	0.0	0.0	0.0	0.0	0.0	0.0	0.0
0.0	0.0	0.0	0.0	0.0	0.0	0.0	0.0
0.0	0.0	0.0	0.0	0.0	0.0	0.0	0.0
0.0	0.0	0.0	0.0	0.0	0.0	0.0	0.0
0.0	0.0	0.0	0.0	6.0	0.0	0.0	0.0
0.0	0.0	0.0	0.0	0.0	0.0	0.0	0.0
0.0	0.0	0.0	0.0	0.0	0.0	0.0	0.0
1.0	0.0	0.0	0.0	3.0	0.0	0.0	0.0
0.0	0.0	0.0	0.0	0.0	0.0	0.0	0.0
0.0	0.0	0.0	0.0	0.0	0.0	0.0	0.0
0.0	0.0	0.0	0.0	0.0	0.0	0.0	0.0
0.0	0.0	0.0	0.0	0.0	0.0	0.0	0.0

0.0	0.0	0.0	0.0	0.0	0.0	0.0	0.0
0.0	0.0	0.0	0.0	0.0	0.0	0.0	0.0
5.0	0.0	0.0	0.0	0.0	0.0	0.0	0.0
0.0	0.0	0.0	0.0	0.0	0.0	0.0	0.0
1.0	0.0	0.0	0.0	0.0	0.0	0.0	0.0
0.0	0.0	0.0	0.0	0.0	0.0	0.0	0.0
0.0	0.0	0.0	0.0	0.0	0.0	0.0	0.0
0.0	0.0	0.0	0.0	0.0	0.0	0.0	0.0
0.0	0.0	0.0	0.0	0.0	0.0	0.0	0.0
0.0	0.0	5.0	0.0	0.0	2.0	0.0	0.0
0.0	0.0	0.0	0.0	0.0	0.0	0.0	0.0
0.0	0.0	0.0	0.0	0.0	2.0	0.0	0.0
0.0	0.0	0.0	0.0	2.0	2.0	0.0	0.0
0.0	3.0	0.0	6.0	0.0	0.0	0.0	6.0
0.0	0.0	0.0	0.0	0.0	0.0	0.0	0.0
0.0	0.0	0.0	0.0	0.0	0.0	0.0	0.0
0.0	0.0	0.0	0.0	0.0	0.0	0.0	0.0
0.0	0.0	1.0	0.0	0.0	0.0	0.0	0.0
0.0	0.0	0.0	0.0	0.0	0.0	0.0	0.0
0.0	0.0	0.0	0.0	0.0	0.0	0.0	0.0
0.0	0.0	0.0	0.0	0.0	0.0	0.0	0.0
0.0	0.0	0.0	0.0	0.0	0.0	0.0	0.0
0.0	0.0	0.0	0.0	0.0	0.0	0.0	0.0
0.0	0.0	0.0	0.0	0.0	0.0	0.0	0.0
0.0	0.0	0.0	0.0	0.0	0.0	0.0	0.0
0.0	0.0	0.0	0.0	0.0	0.0	0.0	0.0
0.0	0.0	0.0	0.0	0.0	0.0	0.0	0.0
0.0	0.0	0.0	0.0	0.0	0.0	0.0	0.0
0.0	0.0	0.0	0.0	0.0	0.0	0.0	0.0
0.0	0.0	2.0	0.0	0.0	0.0	0.0	0.0
0.0	0.0	0.0	0.0	0.0	0.0	0.0	0.0
0.0	0.0	0.0	0.0	0.0	0.0	0.0	0.0
0.0	0.0	0.0	0.0	1.0	0.0	0.0	0.0
0.0	0.0	0.0	0.0	0.0	0.0	0.0	0.0
0.0	0.0	20.0	0.0	0.0	0.0	0.0	0.0
0.0	0.0	0.0	0.0	0.0	0.0	0.0	0.0
0.0	0.0	1.0	0.0	0.0	0.0	0.0	2.0
0.0	0.0	0.0	0.0	0.0	0.0	0.0	0.0
0.0	0.0	2.0	0.0	0.0	0.0	0.0	0.0
0.0	0.0	0.0	0.0	0.0	0.0	0.0	0.0
0.0	0.0	0.0	0.0	0.0	0.0	0.0	0.0
0.0	0.0	0.0	0.0	1.0	0.0	0.0	0.0
0.0	0.0	0.0	0.0	0.0	0.0	0.0	0.0
0.0	0.0	0.0	0.0	0.0	0.0	0.0	0.0
0.0	0.0	0.0	0.0	0.0	0.0	0.0	0.0
0.0	0.0	0.0	0.0	0.0	0.0	0.0	0.0
0.0	0.0	0.0	0.0	0.0	0.0	0.0	0.0

0.0	0.0	0.0	0.0	0.0	0.0	0.0	0.0
0.0	0.0	0.0	0.0	0.0	0.0	0.0	0.0
0.0	0.0	0.0	0.0	0.0	0.0	0.0	0.0
0.0	0.0	0.0	0.0	0.0	0.0	0.0	0.0
0.0	0.0	0.0	0.0	0.0	0.0	0.0	0.0
0.0	0.0	0.0	0.0	0.0	0.0	0.0	0.0
0.0	0.0	0.0	0.0	0.0	0.0	0.0	0.0
0.0	0.0	0.0	0.0	0.0	0.0	0.0	0.0
0.0	0.0	0.0	0.0	0.0	0.0	0.0	0.0
0.0	0.0	0.0	0.0	0.0	0.0	0.0	0.0
0.0	0.0	0.0	0.0	0.0	0.0	0.0	0.0
0.0	0.0	0.0	0.0	0.0	0.0	0.0	0.0
0.0	0.0	0.0	0.0	0.0	0.0	0.0	0.0
0.0	0.0	0.0	0.0	0.0	0.0	0.0	0.0
0.0	0.0	0.0	0.0	0.0	0.0	0.0	0.0
0.0	0.0	0.0	0.0	0.0	0.0	0.0	0.0
0.0	0.0	0.0	0.0	0.0	0.0	0.0	0.0
0.0	0.0	0.0	0.0	4.0	0.0	0.0	0.0
0.0	0.0	0.0	0.0	0.0	0.0	0.0	0.0
0.0	0.0	0.0	0.0	0.0	0.0	0.0	0.0
0.0	0.0	0.0	0.0	0.0	0.0	0.0	0.0
0.0	0.0	0.0	0.0	0.0	0.0	0.0	0.0
0.0	0.0	0.0	0.0	0.0	0.0	0.0	0.0
0.0	0.0	0.0	0.0	0.0	0.0	0.0	0.0
0.0	0.0	0.0	0.0	58.0	0.0	0.0	0.0
0.0	0.0	0.0	0.0	0.0	0.0	7.0	0.0
0.0	0.0	0.0	0.0	0.0	0.0	0.0	0.0
0.0	0.0	0.0	0.0	0.0	0.0	0.0	0.0
0.0	0.0	0.0	0.0	0.0	0.0	0.0	0.0
0.0	0.0	0.0	0.0	0.0	0.0	0.0	0.0
0.0	0.0	0.0	0.0	0.0	0.0	0.0	0.0
0.0	0.0	0.0	0.0	0.0	0.0	0.0	0.0
0.0	0.0	0.0	0.0	0.0	0.0	0.0	0.0
0.0	0.0	0.0	0.0	0.0	0.0	0.0	0.0
0.0	0.0	4.0	0.0	0.0	0.0	0.0	0.0
0.0	0.0	0.0	0.0	0.0	0.0	0.0	0.0
0.0	0.0	0.0	0.0	0.0	0.0	0.0	0.0
0.0	0.0	0.0	0.0	0.0	0.0	0.0	0.0
0.0	0.0	0.0	0.0	0.0	0.0	0.0	0.0
0.0	0.0	0.0	0.0	13.0	0.0	0.0	0.0
0.0	0.0	0.0	0.0	0.0	0.0	2.0	0.0
0.0	0.0	0.0	0.0	0.0	0.0	0.0	0.0
0.0	0.0	0.0	0.0	0.0	0.0	0.0	0.0
0.0	0.0	0.0	0.0	0.0	0.0	0.0	0.0
0.0	0.0	0.0	0.0	0.0	0.0	0.0	0.0
0.0	0.0	0.0	0.0	0.0	0.0	0.0	0.0
0.0	0.0	0.0	0.0	0.0	0.0	0.0	0.0

0.0	0.0	0.0	0.0	0.0	0.0	0.0	0.0
0.0	0.0	0.0	0.0	0.0	0.0	0.0	0.0
0.0	0.0	0.0	0.0	0.0	0.0	0.0	0.0
0.0	0.0	0.0	0.0	0.0	0.0	0.0	0.0
0.0	0.0	0.0	0.0	0.0	0.0	0.0	0.0
0.0	0.0	0.0	0.0	0.0	0.0	0.0	0.0
0.0	0.0	0.0	0.0	0.0	0.0	0.0	0.0
0.0	0.0	0.0	0.0	0.0	0.0	0.0	0.0
0.0	0.0	0.0	0.0	0.0	0.0	0.0	0.0
0.0	0.0	0.0	0.0	0.0	0.0	0.0	0.0
0.0	0.0	0.0	0.0	0.0	0.0	0.0	0.0
0.0	0.0	0.0	0.0	0.0	0.0	0.0	0.0
0.0	0.0	0.0	0.0	0.0	0.0	0.0	0.0
0.0	0.0	0.0	0.0	0.0	0.0	0.0	0.0
0.0	0.0	0.0	0.0	0.0	0.0	0.0	0.0
0.0	0.0	0.0	0.0	0.0	0.0	0.0	0.0
0.0	0.0	0.0	0.0	0.0	0.0	0.0	0.0
0.0	0.0	0.0	0.0	0.0	0.0	0.0	0.0
0.0	0.0	0.0	0.0	0.0	0.0	0.0	0.0
0.0	0.0	0.0	0.0	2.0	0.0	0.0	0.0
0.0	0.0	0.0	0.0	0.0	0.0	0.0	0.0
0.0	0.0	0.0	0.0	2.0	0.0	0.0	0.0
0.0	0.0	0.0	0.0	0.0	0.0	0.0	0.0
0.0	0.0	0.0	0.0	7.0	0.0	0.0	0.0
0.0	0.0	0.0	0.0	0.0	0.0	0.0	0.0
0.0	0.0	0.0	0.0	0.0	0.0	0.0	0.0
0.0	0.0	0.0	0.0	0.0	0.0	0.0	0.0
0.0	0.0	1.0	0.0	1.0	0.0	0.0	0.0
0.0	0.0	0.0	0.0	0.0	0.0	0.0	0.0
0.0	0.0	0.0	0.0	0.0	0.0	0.0	0.0
0.0	0.0	0.0	0.0	0.0	0.0	0.0	0.0
0.0	0.0	0.0	0.0	0.0	0.0	0.0	0.0
0.0	0.0	0.0	0.0	0.0	0.0	0.0	0.0
0.0	0.0	0.0	0.0	0.0	0.0	0.0	0.0
0.0	0.0	0.0	0.0	0.0	0.0	0.0	0.0
0.0	0.0	0.0	0.0	0.0	0.0	0.0	0.0
0.0	0.0	0.0	0.0	0.0	0.0	0.0	0.0
0.0	0.0	0.0	0.0	0.0	0.0	0.0	0.0
0.0	0.0	1.0	0.0	0.0	0.0	0.0	0.0
0.0	0.0	0.0	0.0	0.0	0.0	0.0	0.0
0.0	0.0	0.0	0.0	0.0	0.0	0.0	0.0
0.0	0.0	0.0	0.0	0.0	0.0	0.0	0.0
0.0	0.0	0.0	0.0	1.0	0.0	0.0	0.0
0.0	0.0	0.0	0.0	0.0	0.0	0.0	0.0
0.0	0.0	0.0	0.0	0.0	0.0	0.0	0.0
0.0	0.0	0.0	0.0	0.0	0.0	0.0	0.0
0.0	0.0	0.0	0.0	0.0	0.0	0.0	0.0

0.0	0.0	0.0	0.0	0.0	0.0	0.0	0.0
0.0	0.0	0.0	0.0	0.0	0.0	0.0	0.0
0.0	0.0	0.0	0.0	0.0	0.0	0.0	0.0
0.0	0.0	0.0	0.0	0.0	0.0	0.0	0.0
0.0	0.0	0.0	0.0	0.0	0.0	0.0	0.0
0.0	0.0	0.0	0.0	0.0	0.0	0.0	0.0
0.0	0.0	0.0	0.0	0.0	0.0	0.0	0.0
0.0	0.0	0.0	0.0	0.0	0.0	0.0	0.0
0.0	0.0	0.0	0.0	0.0	0.0	0.0	0.0
0.0	0.0	0.0	0.0	0.0	0.0	0.0	0.0
0.0	0.0	0.0	0.0	0.0	0.0	23.0	0.0
0.0	0.0	0.0	0.0	0.0	0.0	0.0	0.0
0.0	0.0	0.0	0.0	0.0	0.0	0.0	0.0
0.0	0.0	0.0	0.0	0.0	0.0	0.0	0.0
0.0	0.0	0.0	0.0	0.0	0.0	0.0	0.0
0.0	0.0	0.0	0.0	4.0	0.0	0.0	0.0
0.0	0.0	0.0	0.0	0.0	0.0	0.0	0.0
0.0	0.0	0.0	0.0	0.0	0.0	0.0	0.0
0.0	0.0	0.0	0.0	0.0	0.0	0.0	0.0
0.0	0.0	0.0	0.0	0.0	0.0	0.0	0.0
0.0	0.0	0.0	0.0	0.0	0.0	0.0	0.0
0.0	0.0	0.0	0.0	0.0	0.0	0.0	0.0
0.0	0.0	0.0	0.0	0.0	0.0	0.0	0.0
0.0	0.0	0.0	0.0	0.0	0.0	0.0	0.0
0.0	0.0	0.0	0.0	0.0	0.0	0.0	0.0
0.0	0.0	0.0	0.0	0.0	0.0	0.0	0.0
0.0	0.0	0.0	0.0	0.0	0.0	0.0	0.0
0.0	0.0	0.0	0.0	0.0	0.0	0.0	0.0
0.0	0.0	0.0	0.0	0.0	0.0	0.0	0.0
0.0	0.0	0.0	0.0	0.0	0.0	0.0	0.0
0.0	0.0	0.0	0.0	0.0	0.0	0.0	0.0
27.0	0.0	0.0	0.0	0.0	0.0	0.0	0.0
0.0	0.0	0.0	0.0	0.0	0.0	0.0	0.0
0.0	0.0	0.0	0.0	0.0	0.0	0.0	0.0
0.0	0.0	0.0	0.0	0.0	0.0	0.0	0.0
0.0	0.0	0.0	0.0	0.0	1.0	0.0	0.0
0.0	0.0	0.0	0.0	0.0	0.0	0.0	0.0
0.0	0.0	0.0	0.0	0.0	0.0	0.0	0.0
0.0	0.0	0.0	0.0	0.0	0.0	0.0	0.0
0.0	0.0	137.0	0.0	37.0	0.0	0.0	0.0
0.0	0.0	0.0	0.0	14.0	14.0	3.0	0.0
0.0	0.0	0.0	0.0	0.0	0.0	0.0	0.0
0.0	0.0	0.0	0.0	0.0	0.0	0.0	0.0
0.0	0.0	0.0	0.0	0.0	0.0	0.0	0.0
0.0	0.0	0.0	0.0	22.0	0.0	0.0	0.0
0.0	0.0	0.0	0.0	3.0	0.0	0.0	0.0
0.0	0.0	0.0	0.0	0.0	0.0	0.0	0.0

0.0	0.0	0.0	0.0	0.0	0.0	0.0	0.0
0.0	0.0	0.0	0.0	0.0	0.0	0.0	0.0
0.0	0.0	0.0	0.0	0.0	0.0	0.0	0.0
0.0	0.0	0.0	0.0	0.0	0.0	0.0	0.0
0.0	0.0	0.0	0.0	0.0	0.0	0.0	0.0
0.0	0.0	0.0	0.0	0.0	0.0	0.0	0.0
0.0	0.0	0.0	0.0	0.0	0.0	0.0	0.0
0.0	0.0	0.0	0.0	0.0	0.0	0.0	0.0
0.0	0.0	0.0	0.0	1.0	0.0	0.0	0.0
0.0	0.0	0.0	0.0	0.0	0.0	0.0	0.0
0.0	0.0	0.0	0.0	0.0	0.0	0.0	0.0
0.0	0.0	0.0	0.0	0.0	0.0	0.0	0.0
0.0	0.0	0.0	0.0	0.0	0.0	0.0	0.0
0.0	0.0	0.0	0.0	0.0	0.0	0.0	0.0
0.0	0.0	0.0	0.0	0.0	0.0	0.0	0.0
0.0	0.0	0.0	0.0	0.0	0.0	0.0	0.0
0.0	0.0	0.0	0.0	0.0	0.0	0.0	0.0
0.0	0.0	0.0	0.0	0.0	0.0	0.0	0.0
0.0	0.0	0.0	0.0	0.0	0.0	0.0	0.0
0.0	0.0	0.0	0.0	0.0	0.0	0.0	0.0
0.0	0.0	0.0	0.0	1.0	0.0	0.0	0.0
0.0	0.0	0.0	0.0	0.0	0.0	0.0	0.0
0.0	0.0	0.0	0.0	0.0	0.0	0.0	0.0
2.0	0.0	0.0	0.0	1.0	0.0	0.0	0.0
0.0	0.0	0.0	0.0	0.0	0.0	0.0	0.0
0.0	0.0	0.0	0.0	0.0	0.0	0.0	0.0
0.0	0.0	0.0	0.0	0.0	0.0	0.0	0.0
0.0	0.0	0.0	0.0	0.0	0.0	0.0	0.0
0.0	0.0	0.0	0.0	0.0	0.0	0.0	0.0
0.0	0.0	0.0	0.0	0.0	0.0	0.0	0.0
0.0	0.0	0.0	0.0	0.0	0.0	0.0	0.0
0.0	0.0	0.0	0.0	0.0	0.0	0.0	0.0
0.0	0.0	0.0	0.0	0.0	0.0	0.0	0.0
0.0	0.0	0.0	0.0	0.0	0.0	0.0	0.0
0.0	0.0	0.0	0.0	0.0	0.0	0.0	0.0
0.0	0.0	0.0	0.0	0.0	0.0	0.0	0.0
0.0	0.0	0.0	0.0	0.0	0.0	0.0	0.0
0.0	0.0	0.0	0.0	0.0	0.0	0.0	0.0
0.0	0.0	0.0	0.0	0.0	0.0	0.0	0.0
0.0	0.0	0.0	0.0	1.0	0.0	0.0	0.0
0.0	0.0	0.0	0.0	0.0	0.0	0.0	0.0
0.0	0.0	0.0	0.0	0.0	0.0	0.0	0.0
0.0	0.0	0.0	0.0	0.0	0.0	0.0	0.0
0.0	14.0	0.0	0.0	0.0	9.0	0.0	0.0
0.0	0.0	0.0	0.0	0.0	0.0	0.0	0.0
0.0	0.0	0.0	0.0	0.0	0.0	0.0	0.0
0.0	0.0	0.0	0.0	0.0	0.0	0.0	0.0

0.0	0.0	0.0	0.0	0.0	0.0	0.0	0.0
0.0	0.0	0.0	0.0	0.0	0.0	0.0	0.0
0.0	0.0	0.0	0.0	0.0	0.0	0.0	0.0
0.0	0.0	0.0	0.0	0.0	0.0	0.0	0.0
0.0	0.0	0.0	0.0	0.0	0.0	0.0	0.0
0.0	0.0	0.0	0.0	0.0	0.0	0.0	0.0
0.0	0.0	0.0	0.0	0.0	0.0	0.0	0.0
0.0	0.0	0.0	0.0	0.0	0.0	0.0	0.0
0.0	0.0	0.0	0.0	0.0	0.0	0.0	0.0
0.0	0.0	0.0	0.0	0.0	0.0	0.0	0.0
0.0	0.0	0.0	0.0	0.0	0.0	0.0	0.0
0.0	0.0	0.0	0.0	0.0	0.0	0.0	0.0
0.0	0.0	0.0	0.0	0.0	0.0	121.0	0.0
0.0	0.0	0.0	0.0	0.0	0.0	0.0	0.0
0.0	0.0	0.0	0.0	0.0	0.0	0.0	0.0
0.0	0.0	0.0	0.0	0.0	0.0	0.0	0.0
0.0	0.0	0.0	0.0	0.0	0.0	0.0	0.0
0.0	0.0	0.0	0.0	0.0	0.0	0.0	0.0
0.0	0.0	0.0	0.0	0.0	0.0	0.0	0.0
0.0	0.0	0.0	0.0	0.0	0.0	0.0	0.0
0.0	0.0	0.0	0.0	0.0	2.0	0.0	0.0
0.0	0.0	0.0	0.0	4.0	0.0	0.0	0.0
0.0	0.0	0.0	0.0	0.0	0.0	0.0	0.0
0.0	0.0	0.0	0.0	0.0	0.0	0.0	0.0
0.0	0.0	0.0	0.0	0.0	0.0	0.0	0.0
0.0	0.0	0.0	0.0	0.0	0.0	0.0	0.0
0.0	0.0	0.0	0.0	0.0	0.0	0.0	0.0
0.0	0.0	0.0	0.0	0.0	0.0	0.0	0.0
0.0	0.0	0.0	0.0	0.0	0.0	0.0	0.0
0.0	0.0	0.0	0.0	0.0	0.0	0.0	0.0
0.0	0.0	0.0	0.0	0.0	0.0	0.0	0.0
0.0	0.0	0.0	0.0	0.0	0.0	0.0	0.0
0.0	0.0	0.0	0.0	0.0	0.0	0.0	0.0
0.0	0.0	0.0	0.0	0.0	0.0	0.0	0.0
0.0	0.0	0.0	0.0	0.0	0.0	0.0	0.0
0.0	0.0	0.0	0.0	0.0	0.0	0.0	0.0
0.0	0.0	0.0	0.0	0.0	0.0	0.0	0.0
0.0	0.0	0.0	0.0	0.0	0.0	0.0	0.0
0.0	10.0	0.0	0.0	1.0	0.0	0.0	0.0
0.0	0.0	0.0	0.0	0.0	0.0	12.0	0.0
0.0	0.0	0.0	0.0	0.0	0.0	0.0	0.0
0.0	0.0	0.0	0.0	0.0	0.0	0.0	0.0
0.0	0.0	0.0	0.0	0.0	0.0	0.0	0.0
0.0	0.0	0.0	0.0	0.0	0.0	0.0	0.0
0.0	0.0	0.0	0.0	0.0	0.0	0.0	0.0
0.0	0.0	0.0	0.0	0.0	0.0	0.0	0.0
0.0	0.0	0.0	0.0	0.0	0.0	0.0	0.0

0.0	0.0	0.0	0.0	1.0	0.0	0.0	0.0
0.0	0.0	0.0	0.0	0.0	21.0	0.0	0.0
0.0	0.0	0.0	0.0	0.0	0.0	0.0	0.0
0.0	0.0	0.0	0.0	0.0	0.0	0.0	0.0
0.0	0.0	0.0	0.0	0.0	0.0	0.0	0.0
0.0	0.0	0.0	0.0	0.0	0.0	0.0	0.0
0.0	0.0	0.0	0.0	0.0	0.0	0.0	0.0
0.0	0.0	0.0	0.0	0.0	0.0	0.0	0.0
0.0	0.0	0.0	0.0	0.0	0.0	0.0	0.0
0.0	0.0	0.0	0.0	0.0	0.0	0.0	0.0
0.0	0.0	0.0	0.0	0.0	0.0	0.0	0.0
0.0	0.0	0.0	0.0	0.0	0.0	0.0	372.0
0.0	0.0	4.0	0.0	6.0	0.0	0.0	0.0
0.0	10.0	0.0	0.0	0.0	3.0	0.0	108.0
0.0	0.0	0.0	0.0	0.0	0.0	0.0	0.0
0.0	0.0	0.0	0.0	0.0	0.0	0.0	0.0
0.0	0.0	0.0	0.0	0.0	0.0	0.0	1.0
0.0	0.0	0.0	0.0	0.0	0.0	0.0	0.0
0.0	0.0	0.0	0.0	0.0	0.0	0.0	0.0
0.0	0.0	0.0	0.0	0.0	0.0	0.0	0.0
0.0	0.0	0.0	0.0	0.0	0.0	0.0	0.0
0.0	0.0	0.0	0.0	0.0	0.0	0.0	0.0
0.0	0.0	0.0	0.0	0.0	0.0	0.0	0.0
0.0	0.0	0.0	0.0	0.0	0.0	0.0	0.0
0.0	0.0	0.0	0.0	0.0	0.0	0.0	0.0
0.0	0.0	0.0	0.0	0.0	0.0	0.0	0.0
0.0	0.0	0.0	0.0	0.0	0.0	0.0	0.0
0.0	0.0	0.0	0.0	0.0	0.0	0.0	0.0
0.0	0.0	0.0	0.0	0.0	0.0	0.0	0.0
0.0	0.0	0.0	0.0	0.0	0.0	0.0	0.0
0.0	0.0	0.0	0.0	2.0	0.0	0.0	0.0
0.0	0.0	0.0	0.0	0.0	0.0	0.0	0.0
0.0	0.0	0.0	0.0	0.0	0.0	0.0	0.0
0.0	0.0	0.0	0.0	0.0	0.0	0.0	0.0
0.0	0.0	0.0	0.0	0.0	0.0	0.0	0.0
0.0	0.0	0.0	0.0	0.0	0.0	0.0	0.0
0.0	0.0	0.0	0.0	0.0	0.0	0.0	0.0
0.0	0.0	0.0	0.0	0.0	0.0	0.0	0.0
0.0	0.0	0.0	0.0	1.0	0.0	0.0	0.0
0.0	0.0	0.0	0.0	0.0	0.0	0.0	0.0
0.0	0.0	0.0	0.0	0.0	0.0	0.0	0.0
0.0	0.0	0.0	0.0	0.0	0.0	0.0	0.0
0.0	0.0	0.0	0.0	0.0	0.0	0.0	0.0
0.0	0.0	0.0	0.0	0.0	0.0	0.0	0.0
0.0	0.0	0.0	0.0	0.0	0.0	0.0	0.0
0.0	0.0	0.0	0.0	0.0	0.0	0.0	0.0
0.0	0.0	0.0	0.0	0.0	0.0	0.0	0.0

0.0	0.0	0.0	0.0	0.0	0.0	0.0	0.0
0.0	0.0	0.0	0.0	0.0	0.0	0.0	0.0
0.0	0.0	0.0	0.0	0.0	0.0	0.0	0.0
0.0	0.0	0.0	0.0	0.0	0.0	0.0	0.0
0.0	0.0	0.0	0.0	0.0	0.0	0.0	0.0
0.0	0.0	0.0	0.0	0.0	0.0	0.0	0.0
1.0	0.0	0.0	0.0	0.0	0.0	0.0	0.0
0.0	0.0	0.0	0.0	0.0	0.0	0.0	0.0
0.0	0.0	0.0	0.0	0.0	0.0	0.0	0.0
0.0	0.0	6.0	0.0	0.0	0.0	0.0	0.0
0.0	0.0	0.0	0.0	0.0	0.0	11.0	0.0
1.0	0.0	0.0	0.0	0.0	0.0	0.0	0.0
0.0	0.0	0.0	0.0	0.0	0.0	0.0	0.0
0.0	0.0	0.0	0.0	0.0	0.0	0.0	0.0
0.0	0.0	0.0	0.0	0.0	0.0	0.0	0.0
0.0	0.0	0.0	0.0	0.0	0.0	0.0	0.0
0.0	0.0	0.0	0.0	0.0	0.0	0.0	0.0
0.0	0.0	0.0	0.0	0.0	0.0	0.0	0.0
0.0	3.0	0.0	0.0	0.0	0.0	0.0	0.0
0.0	0.0	3.0	9.0	2.0	0.0	0.0	0.0
0.0	0.0	0.0	0.0	0.0	0.0	0.0	0.0
0.0	0.0	0.0	0.0	2.0	0.0	0.0	0.0
0.0	0.0	0.0	0.0	0.0	0.0	0.0	0.0
0.0	0.0	0.0	0.0	0.0	0.0	0.0	0.0
0.0	0.0	0.0	0.0	0.0	39.0	0.0	0.0
0.0	0.0	0.0	0.0	0.0	0.0	0.0	0.0
0.0	0.0	0.0	0.0	0.0	0.0	0.0	0.0
0.0	0.0	0.0	0.0	0.0	0.0	0.0	0.0
0.0	0.0	0.0	0.0	0.0	0.0	0.0	0.0
0.0	0.0	0.0	0.0	0.0	0.0	0.0	0.0
0.0	0.0	0.0	0.0	0.0	0.0	0.0	0.0
0.0	0.0	0.0	0.0	0.0	0.0	0.0	0.0
0.0	0.0	0.0	0.0	0.0	0.0	0.0	0.0
0.0	0.0	0.0	0.0	0.0	0.0	0.0	0.0
0.0	0.0	0.0	0.0	0.0	0.0	0.0	0.0
0.0	0.0	0.0	0.0	0.0	0.0	0.0	0.0
0.0	0.0	0.0	0.0	0.0	0.0	0.0	0.0
0.0	0.0	0.0	0.0	0.0	0.0	0.0	0.0
0.0	0.0	0.0	0.0	1.0	0.0	0.0	0.0
0.0	0.0	0.0	0.0	0.0	0.0	0.0	0.0
0.0	0.0	0.0	0.0	0.0	0.0	0.0	0.0
0.0	0.0	0.0	0.0	0.0	0.0	0.0	0.0
0.0	0.0	0.0	0.0	0.0	0.0	0.0	0.0
0.0	0.0	0.0	0.0	0.0	0.0	0.0	0.0
0.0	0.0	0.0	0.0	0.0	0.0	0.0	0.0
0.0	0.0	0.0	0.0	0.0	0.0	0.0	0.0
0.0	0.0	0.0	0.0	0.0	0.0	0.0	0.0

0.0	0.0	0.0	0.0	0.0	0.0	0.0	0.0
0.0	0.0	1.0	0.0	1.0	0.0	0.0	0.0
0.0	0.0	0.0	0.0	0.0	0.0	0.0	0.0
0.0	0.0	0.0	0.0	2.0	0.0	0.0	0.0
0.0	0.0	0.0	0.0	0.0	0.0	0.0	0.0
0.0	0.0	0.0	0.0	0.0	0.0	0.0	0.0
0.0	0.0	0.0	0.0	0.0	0.0	0.0	0.0
0.0	0.0	0.0	0.0	0.0	0.0	0.0	0.0
0.0	0.0	0.0	0.0	0.0	0.0	0.0	0.0
0.0	0.0	0.0	0.0	0.0	0.0	0.0	0.0
0.0	0.0	0.0	0.0	0.0	0.0	0.0	0.0
0.0	0.0	0.0	0.0	0.0	0.0	0.0	0.0
0.0	0.0	0.0	0.0	0.0	0.0	0.0	0.0
13.0	0.0	0.0	0.0	0.0	0.0	0.0	0.0
0.0	0.0	0.0	0.0	0.0	0.0	0.0	0.0
0.0	0.0	0.0	0.0	0.0	0.0	0.0	0.0
0.0	0.0	0.0	0.0	0.0	0.0	0.0	0.0
0.0	0.0	0.0	0.0	0.0	0.0	0.0	0.0
0.0	0.0	0.0	0.0	0.0	0.0	0.0	2.0
0.0	0.0	0.0	0.0	0.0	0.0	0.0	0.0
0.0	0.0	0.0	0.0	0.0	0.0	0.0	0.0
0.0	0.0	0.0	0.0	0.0	0.0	0.0	0.0
0.0	0.0	0.0	0.0	0.0	0.0	0.0	0.0
0.0	0.0	0.0	0.0	0.0	2.0	0.0	0.0
0.0	0.0	0.0	0.0	0.0	0.0	0.0	0.0
0.0	0.0	0.0	0.0	0.0	0.0	0.0	0.0
0.0	0.0	0.0	0.0	0.0	0.0	0.0	0.0
0.0	0.0	0.0	0.0	0.0	0.0	0.0	0.0
0.0	0.0	0.0	0.0	0.0	0.0	0.0	0.0
0.0	0.0	0.0	0.0	0.0	0.0	0.0	0.0
0.0	0.0	0.0	0.0	0.0	0.0	0.0	0.0
0.0	0.0	0.0	0.0	0.0	0.0	0.0	0.0
0.0	0.0	0.0	0.0	0.0	0.0	0.0	0.0
0.0	0.0	0.0	0.0	0.0	0.0	0.0	0.0
0.0	0.0	0.0	0.0	0.0	0.0	0.0	0.0
0.0	1.0	0.0	3.0	0.0	0.0	0.0	0.0
0.0	0.0	0.0	0.0	0.0	0.0	0.0	0.0
0.0	0.0	0.0	0.0	0.0	0.0	0.0	0.0
0.0	0.0	0.0	0.0	0.0	0.0	0.0	0.0
0.0	0.0	0.0	0.0	0.0	0.0	0.0	0.0
0.0	0.0	0.0	0.0	0.0	0.0	0.0	0.0
0.0	0.0	0.0	0.0	0.0	0.0	0.0	0.0
0.0	0.0	0.0	0.0	0.0	0.0	0.0	0.0
3.0	0.0	0.0	0.0	0.0	0.0	0.0	0.0
0.0	0.0	0.0	0.0	0.0	0.0	0.0	0.0
0.0	0.0	0.0	0.0	0.0	0.0	0.0	0.0
0.0	0.0	0.0	0.0	0.0	0.0	0.0	0.0

0.0	0.0	0.0	0.0	0.0	0.0	0.0	0.0
0.0	0.0	0.0	0.0	0.0	0.0	0.0	0.0
0.0	0.0	0.0	0.0	0.0	0.0	3.0	0.0
0.0	0.0	0.0	0.0	0.0	0.0	0.0	0.0
0.0	0.0	0.0	0.0	0.0	0.0	0.0	0.0
1.0	0.0	0.0	0.0	0.0	0.0	0.0	0.0
0.0	0.0	0.0	0.0	0.0	0.0	0.0	0.0
0.0	0.0	0.0	0.0	0.0	0.0	0.0	0.0
0.0	0.0	0.0	0.0	0.0	0.0	0.0	0.0
0.0	0.0	0.0	0.0	0.0	0.0	0.0	0.0
0.0	0.0	0.0	0.0	0.0	0.0	0.0	0.0
0.0	0.0	0.0	0.0	0.0	0.0	0.0	0.0
0.0	0.0	0.0	0.0	0.0	0.0	0.0	0.0
0.0	0.0	0.0	0.0	0.0	0.0	0.0	0.0
0.0	0.0	0.0	0.0	0.0	0.0	0.0	0.0
0.0	0.0	0.0	0.0	0.0	0.0	0.0	0.0
0.0	0.0	0.0	0.0	0.0	0.0	0.0	0.0
0.0	0.0	0.0	0.0	0.0	0.0	0.0	0.0
0.0	0.0	0.0	0.0	0.0	0.0	0.0	0.0
0.0	0.0	0.0	0.0	0.0	0.0	0.0	0.0
0.0	0.0	0.0	0.0	0.0	0.0	0.0	0.0
0.0	0.0	0.0	0.0	0.0	0.0	0.0	0.0
0.0	0.0	0.0	0.0	0.0	0.0	0.0	0.0
0.0	0.0	0.0	0.0	0.0	0.0	0.0	0.0
0.0	0.0	0.0	0.0	0.0	0.0	0.0	0.0
0.0	0.0	0.0	0.0	0.0	0.0	0.0	0.0
0.0	0.0	0.0	0.0	0.0	0.0	0.0	0.0
0.0	0.0	0.0	0.0	0.0	0.0	0.0	0.0
0.0	0.0	0.0	0.0	0.0	0.0	0.0	0.0
0.0	0.0	0.0	0.0	0.0	0.0	0.0	0.0
0.0	0.0	0.0	0.0	0.0	0.0	0.0	0.0
6.0	0.0	0.0	0.0	0.0	0.0	0.0	0.0
0.0	0.0	0.0	0.0	0.0	0.0	0.0	0.0
0.0	0.0	0.0	0.0	0.0	0.0	0.0	0.0
0.0	0.0	0.0	0.0	0.0	0.0	0.0	0.0
0.0	0.0	0.0	0.0	0.0	0.0	0.0	0.0
0.0	0.0	0.0	0.0	0.0	0.0	0.0	0.0
0.0	0.0	0.0	0.0	0.0	0.0	0.0	0.0
0.0	0.0	1.0	0.0	13.0	0.0	0.0	0.0
0.0	0.0	0.0	0.0	0.0	0.0	0.0	0.0
0.0	0.0	0.0	0.0	0.0	0.0	0.0	0.0
0.0	0.0	0.0	0.0	0.0	0.0	0.0	0.0
5.0	0.0	2.0	0.0	0.0	0.0	0.0	0.0
0.0	0.0	0.0	0.0	0.0	0.0	0.0	0.0
0.0	0.0	1.0	0.0	0.0	0.0	0.0	0.0
0.0	0.0	0.0	0.0	0.0	0.0	0.0	0.0
0.0	0.0	0.0	0.0	0.0	0.0	0.0	0.0

0.0	0.0	0.0	0.0	0.0	0.0	0.0	0.0
0.0	0.0	0.0	0.0	0.0	0.0	0.0	0.0
0.0	0.0	0.0	0.0	0.0	0.0	0.0	0.0
0.0	0.0	0.0	0.0	0.0	0.0	0.0	0.0
0.0	0.0	0.0	0.0	0.0	0.0	0.0	0.0
0.0	0.0	0.0	0.0	0.0	0.0	0.0	0.0
0.0	0.0	0.0	0.0	0.0	0.0	0.0	0.0
0.0	0.0	0.0	0.0	0.0	0.0	0.0	0.0
0.0	0.0	0.0	0.0	0.0	0.0	0.0	0.0
0.0	0.0	0.0	0.0	0.0	0.0	0.0	0.0
0.0	0.0	0.0	0.0	0.0	0.0	0.0	0.0
0.0	0.0	0.0	0.0	0.0	0.0	0.0	0.0
0.0	0.0	0.0	0.0	0.0	0.0	0.0	0.0
0.0	0.0	0.0	0.0	0.0	0.0	0.0	0.0
0.0	0.0	0.0	0.0	0.0	0.0	0.0	0.0
0.0	0.0	0.0	0.0	0.0	0.0	0.0	0.0
0.0	0.0	0.0	0.0	0.0	0.0	0.0	0.0
0.0	0.0	0.0	0.0	1.0	0.0	0.0	0.0
0.0	0.0	0.0	0.0	0.0	0.0	0.0	0.0
0.0	0.0	0.0	0.0	0.0	0.0	0.0	0.0
0.0	0.0	0.0	0.0	3.0	0.0	0.0	0.0
0.0	0.0	0.0	0.0	0.0	0.0	0.0	0.0
0.0	0.0	0.0	0.0	0.0	0.0	0.0	0.0
0.0	0.0	0.0	0.0	0.0	0.0	0.0	0.0
0.0	0.0	0.0	0.0	0.0	0.0	0.0	0.0
0.0	0.0	0.0	0.0	0.0	0.0	0.0	0.0
0.0	0.0	0.0	0.0	0.0	0.0	0.0	0.0
0.0	0.0	0.0	0.0	0.0	0.0	0.0	0.0
1.0	0.0	0.0	0.0	0.0	0.0	0.0	0.0
0.0	0.0	0.0	0.0	0.0	0.0	0.0	0.0
0.0	0.0	0.0	0.0	0.0	0.0	0.0	0.0
0.0	0.0	0.0	0.0	0.0	0.0	0.0	0.0
0.0	0.0	0.0	0.0	0.0	0.0	0.0	0.0
0.0	0.0	0.0	0.0	0.0	0.0	0.0	0.0
0.0	0.0	0.0	0.0	0.0	0.0	0.0	0.0
0.0	0.0	0.0	0.0	0.0	0.0	0.0	0.0
0.0	0.0	0.0	0.0	0.0	0.0	0.0	0.0
0.0	0.0	0.0	0.0	0.0	0.0	0.0	0.0
0.0	0.0	0.0	0.0	0.0	0.0	0.0	0.0
0.0	0.0	0.0	0.0	0.0	0.0	0.0	0.0
0.0	0.0	0.0	0.0	0.0	0.0	0.0	0.0
0.0	0.0	0.0	0.0	0.0	0.0	0.0	0.0
0.0	0.0	0.0	0.0	0.0	0.0	0.0	0.0
0.0	0.0	0.0	0.0	0.0	0.0	0.0	0.0
0.0	0.0	0.0	0.0	0.0	0.0	0.0	0.0
0.0	0.0	0.0	0.0	0.0	0.0	0.0	0.0
0.0	0.0	0.0	0.0	0.0	0.0	0.0	0.0

0.0	0.0	0.0	0.0	0.0	0.0	0.0	0.0
0.0	0.0	0.0	0.0	0.0	0.0	0.0	0.0
0.0	0.0	0.0	0.0	0.0	0.0	0.0	0.0
0.0	0.0	0.0	0.0	0.0	0.0	0.0	0.0
0.0	0.0	0.0	0.0	0.0	0.0	0.0	0.0
0.0	0.0	0.0	0.0	0.0	0.0	0.0	0.0
0.0	0.0	0.0	0.0	0.0	0.0	0.0	0.0
0.0	0.0	0.0	0.0	0.0	0.0	0.0	0.0
0.0	0.0	0.0	0.0	0.0	0.0	0.0	0.0
0.0	0.0	0.0	0.0	0.0	0.0	0.0	0.0
0.0	0.0	0.0	0.0	0.0	0.0	0.0	0.0
0.0	0.0	1.0	0.0	0.0	0.0	0.0	0.0
0.0	0.0	0.0	0.0	0.0	0.0	0.0	0.0
0.0	0.0	0.0	0.0	0.0	0.0	0.0	0.0
0.0	0.0	0.0	0.0	0.0	0.0	0.0	0.0
0.0	0.0	0.0	0.0	0.0	0.0	0.0	0.0
0.0	0.0	0.0	0.0	0.0	0.0	0.0	0.0
0.0	0.0	0.0	0.0	0.0	0.0	0.0	0.0
0.0	0.0	0.0	0.0	0.0	0.0	0.0	0.0
0.0	0.0	0.0	0.0	0.0	0.0	0.0	0.0
0.0	0.0	0.0	0.0	0.0	0.0	0.0	0.0
0.0	0.0	0.0	0.0	0.0	0.0	9.0	0.0
0.0	0.0	0.0	0.0	0.0	0.0	0.0	0.0
0.0	0.0	0.0	0.0	0.0	0.0	0.0	0.0
0.0	0.0	19.0	0.0	0.0	0.0	0.0	0.0
0.0	0.0	0.0	0.0	0.0	0.0	0.0	0.0
0.0	0.0	0.0	0.0	0.0	0.0	0.0	0.0
0.0	0.0	0.0	0.0	0.0	0.0	0.0	0.0
0.0	0.0	0.0	0.0	0.0	0.0	0.0	0.0
0.0	0.0	0.0	0.0	0.0	0.0	0.0	0.0
0.0	0.0	0.0	0.0	0.0	0.0	0.0	0.0
0.0	0.0	0.0	0.0	0.0	0.0	0.0	0.0
0.0	0.0	0.0	0.0	0.0	0.0	0.0	0.0
0.0	0.0	0.0	0.0	0.0	0.0	0.0	0.0
0.0	0.0	0.0	0.0	0.0	0.0	0.0	0.0
0.0	0.0	0.0	0.0	0.0	0.0	0.0	0.0
0.0	0.0	0.0	0.0	0.0	0.0	0.0	0.0
0.0	0.0	0.0	0.0	0.0	0.0	0.0	0.0
0.0	0.0	0.0	0.0	0.0	0.0	0.0	0.0
0.0	0.0	0.0	0.0	0.0	0.0	0.0	0.0
0.0	0.0	0.0	0.0	0.0	0.0	0.0	0.0
0.0	0.0	0.0	0.0	0.0	0.0	0.0	0.0
0.0	0.0	0.0	0.0	0.0	0.0	0.0	0.0
0.0	0.0	0.0	0.0	0.0	0.0	0.0	0.0
0.0	0.0	0.0	0.0	0.0	0.0	0.0	0.0
0.0	0.0	0.0	0.0	0.0	0.0	0.0	0.0
0.0	0.0	0.0	0.0	0.0	0.0	0.0	0.0

0.0	0.0	0.0	0.0	0.0	0.0	0.0	0.0
0.0	0.0	0.0	0.0	0.0	0.0	0.0	0.0
0.0	0.0	0.0	0.0	0.0	0.0	0.0	0.0
0.0	0.0	0.0	0.0	0.0	0.0	0.0	0.0
0.0	0.0	0.0	0.0	0.0	0.0	0.0	0.0
0.0	0.0	0.0	0.0	0.0	0.0	0.0	0.0
0.0	0.0	0.0	0.0	0.0	0.0	0.0	0.0
0.0	0.0	0.0	0.0	0.0	0.0	0.0	0.0
0.0	0.0	0.0	0.0	0.0	0.0	0.0	0.0
0.0	0.0	0.0	0.0	0.0	0.0	0.0	0.0
0.0	0.0	0.0	0.0	0.0	0.0	0.0	0.0
0.0	0.0	0.0	0.0	0.0	0.0	0.0	0.0
0.0	0.0	0.0	0.0	0.0	0.0	0.0	0.0
0.0	0.0	0.0	0.0	0.0	0.0	0.0	0.0
0.0	0.0	0.0	0.0	0.0	0.0	0.0	0.0
0.0	0.0	0.0	0.0	0.0	0.0	0.0	0.0
0.0	0.0	0.0	0.0	0.0	0.0	0.0	0.0
0.0	0.0	0.0	0.0	0.0	0.0	0.0	0.0
0.0	0.0	0.0	0.0	0.0	0.0	0.0	0.0
0.0	0.0	0.0	0.0	0.0	0.0	0.0	0.0
0.0	0.0	0.0	0.0	0.0	0.0	0.0	0.0
0.0	0.0	0.0	0.0	0.0	0.0	0.0	0.0
0.0	0.0	0.0	0.0	0.0	0.0	0.0	0.0
0.0	0.0	0.0	0.0	0.0	0.0	0.0	0.0
0.0	0.0	0.0	0.0	0.0	0.0	0.0	0.0
0.0	0.0	0.0	0.0	0.0	0.0	0.0	0.0
0.0	0.0	0.0	0.0	0.0	0.0	0.0	0.0
0.0	0.0	0.0	0.0	0.0	0.0	0.0	0.0
0.0	0.0	0.0	0.0	0.0	0.0	0.0	0.0
0.0	0.0	0.0	0.0	0.0	0.0	0.0	0.0
0.0	0.0	0.0	0.0	0.0	0.0	0.0	0.0
0.0	0.0	0.0	0.0	0.0	0.0	0.0	0.0
0.0	0.0	0.0	0.0	0.0	0.0	0.0	0.0
0.0	0.0	0.0	0.0	0.0	0.0	0.0	0.0
0.0	0.0	0.0	0.0	0.0	0.0	0.0	0.0
0.0	0.0	0.0	0.0	0.0	0.0	0.0	0.0
0.0	0.0	0.0	0.0	0.0	0.0	0.0	0.0
0.0	0.0	0.0	0.0	0.0	0.0	0.0	0.0
0.0	0.0	0.0	0.0	0.0	0.0	0.0	0.0
0.0	0.0	0.0	0.0	0.0	0.0	0.0	0.0
0.0	0.0	0.0	0.0	0.0	0.0	0.0	0.0
0.0	0.0	0.0	0.0	0.0	0.0	0.0	0.0
0.0	0.0	0.0	0.0	0.0	0.0	0.0	0.0
0.0	0.0	0.0	0.0	0.0	0.0	0.0	0.0
0.0	0.0	0.0	0.0	0.0	0.0	0.0	0.0
0.0	0.0	0.0	0.0	0.0	0.0	0.0	0.0
0.0	0.0	0.0	0.0	0.0	0.0	0.0	0.0

0.0	0.0	0.0	0.0	0.0	0.0	0.0	0.0
0.0	0.0	0.0	0.0	0.0	0.0	0.0	0.0
0.0	0.0	0.0	0.0	0.0	0.0	0.0	0.0
0.0	0.0	0.0	0.0	0.0	0.0	0.0	0.0
0.0	0.0	0.0	0.0	0.0	0.0	0.0	0.0
0.0	0.0	0.0	0.0	0.0	0.0	0.0	0.0
0.0	0.0	0.0	0.0	0.0	0.0	0.0	0.0
0.0	0.0	0.0	0.0	0.0	0.0	0.0	0.0
0.0	0.0	0.0	0.0	0.0	0.0	0.0	0.0
0.0	0.0	0.0	0.0	0.0	0.0	0.0	0.0
0.0	0.0	0.0	0.0	0.0	0.0	0.0	0.0
0.0	0.0	0.0	0.0	0.0	0.0	0.0	0.0
0.0	0.0	0.0	0.0	0.0	0.0	0.0	0.0
0.0	0.0	0.0	0.0	0.0	0.0	0.0	0.0
0.0	0.0	0.0	0.0	0.0	0.0	0.0	0.0
0.0	0.0	0.0	0.0	0.0	0.0	0.0	0.0
0.0	0.0	0.0	0.0	0.0	0.0	0.0	0.0
0.0	0.0	0.0	0.0	0.0	0.0	0.0	0.0
0.0	0.0	0.0	0.0	0.0	0.0	0.0	0.0
0.0	0.0	0.0	0.0	0.0	0.0	0.0	0.0
0.0	0.0	0.0	0.0	0.0	0.0	0.0	0.0
0.0	0.0	0.0	0.0	0.0	0.0	0.0	0.0
0.0	0.0	0.0	0.0	0.0	1.0	0.0	0.0
0.0	0.0	0.0	0.0	0.0	0.0	0.0	0.0
0.0	0.0	0.0	0.0	0.0	0.0	0.0	0.0
0.0	0.0	0.0	0.0	0.0	0.0	0.0	0.0
0.0	0.0	0.0	0.0	0.0	0.0	0.0	0.0
0.0	0.0	0.0	0.0	0.0	0.0	0.0	0.0
0.0	0.0	0.0	0.0	0.0	0.0	0.0	0.0
0.0	0.0	0.0	0.0	0.0	0.0	0.0	0.0
0.0	0.0	0.0	0.0	0.0	0.0	0.0	0.0
0.0	0.0	0.0	0.0	0.0	0.0	0.0	0.0
0.0	0.0	0.0	0.0	0.0	0.0	0.0	0.0
0.0	0.0	0.0	0.0	0.0	0.0	0.0	0.0
0.0	0.0	0.0	0.0	0.0	0.0	1.0	1.0
0.0	0.0	0.0	0.0	0.0	0.0	0.0	0.0
0.0	0.0	0.0	0.0	0.0	0.0	0.0	0.0
0.0	0.0	0.0	0.0	0.0	0.0	0.0	0.0
0.0	0.0	0.0	0.0	0.0	0.0	0.0	0.0
0.0	0.0	0.0	0.0	0.0	0.0	0.0	0.0
0.0	0.0	0.0	0.0	0.0	0.0	0.0	0.0
0.0	0.0	0.0	0.0	0.0	0.0	0.0	0.0
0.0	0.0	0.0	0.0	0.0	0.0	0.0	0.0
0.0	0.0	0.0	0.0	0.0	0.0	0.0	0.0
0.0	0.0	0.0	0.0	0.0	0.0	0.0	0.0
0.0	0.0	0.0	0.0	0.0	0.0	0.0	0.0
0.0	0.0	0.0	0.0	0.0	0.0	0.0	0.0

0.0	0.0	0.0	0.0	0.0	0.0	0.0	0.0
0.0	0.0	0.0	0.0	0.0	0.0	0.0	0.0
0.0	0.0	0.0	0.0	0.0	0.0	0.0	0.0
0.0	0.0	0.0	0.0	0.0	0.0	0.0	0.0
0.0	0.0	0.0	0.0	0.0	0.0	0.0	0.0
0.0	0.0	0.0	0.0	0.0	0.0	0.0	0.0
0.0	0.0	0.0	0.0	0.0	0.0	0.0	0.0
0.0	0.0	0.0	0.0	0.0	0.0	0.0	0.0
0.0	0.0	0.0	0.0	0.0	0.0	0.0	0.0
0.0	0.0	0.0	0.0	0.0	0.0	0.0	0.0
0.0	0.0	0.0	0.0	0.0	0.0	0.0	0.0
0.0	0.0	0.0	0.0	0.0	0.0	0.0	0.0
0.0	0.0	0.0	0.0	0.0	1.0	0.0	0.0
0.0	0.0	0.0	0.0	0.0	0.0	0.0	0.0
0.0	0.0	0.0	0.0	0.0	0.0	0.0	0.0
0.0	0.0	0.0	0.0	0.0	0.0	0.0	0.0
0.0	0.0	0.0	0.0	0.0	0.0	0.0	0.0
0.0	0.0	0.0	0.0	0.0	0.0	0.0	0.0
0.0	0.0	0.0	2.0	0.0	0.0	0.0	0.0
0.0	0.0	0.0	0.0	0.0	0.0	0.0	0.0
0.0	0.0	0.0	0.0	0.0	0.0	0.0	0.0
0.0	0.0	0.0	0.0	0.0	0.0	0.0	0.0
0.0	0.0	0.0	0.0	0.0	0.0	0.0	0.0
0.0	0.0	0.0	0.0	0.0	0.0	0.0	0.0
0.0	0.0	0.0	0.0	0.0	0.0	0.0	0.0
0.0	0.0	0.0	0.0	1.0	0.0	0.0	0.0
0.0	0.0	0.0	0.0	0.0	0.0	0.0	0.0
0.0	0.0	0.0	0.0	0.0	0.0	0.0	0.0
0.0	0.0	2.0	0.0	0.0	0.0	0.0	0.0
0.0	0.0	0.0	0.0	0.0	0.0	0.0	0.0
0.0	0.0	0.0	0.0	0.0	0.0	0.0	0.0
0.0	0.0	0.0	0.0	0.0	0.0	0.0	0.0
0.0	0.0	0.0	0.0	0.0	0.0	0.0	0.0
0.0	0.0	0.0	0.0	0.0	0.0	0.0	0.0
0.0	0.0	0.0	0.0	0.0	0.0	0.0	0.0
0.0	0.0	0.0	0.0	0.0	0.0	0.0	0.0
0.0	0.0	0.0	0.0	0.0	0.0	0.0	0.0
0.0	0.0	0.0	0.0	0.0	0.0	0.0	0.0
0.0	0.0	0.0	0.0	0.0	0.0	0.0	0.0
0.0	0.0	0.0	0.0	0.0	0.0	0.0	0.0
0.0	0.0	0.0	0.0	0.0	0.0	0.0	0.0
0.0	0.0	0.0	0.0	0.0	0.0	0.0	0.0
0.0	0.0	0.0	0.0	0.0	0.0	0.0	0.0
0.0	0.0	0.0	0.0	0.0	0.0	0.0	0.0
0.0	0.0	0.0	0.0	0.0	0.0	0.0	0.0
0.0	0.0	0.0	0.0	0.0	0.0	0.0	0.0
0.0	0.0	0.0	0.0	0.0	0.0	0.0	0.0

0.0	0.0	0.0	0.0	0.0	0.0	0.0	0.0
0.0	0.0	0.0	0.0	9.0	0.0	0.0	0.0
0.0	0.0	0.0	0.0	0.0	0.0	0.0	0.0
0.0	0.0	0.0	0.0	0.0	0.0	0.0	0.0
0.0	0.0	0.0	0.0	0.0	0.0	0.0	0.0
0.0	0.0	0.0	0.0	0.0	0.0	0.0	0.0
2.0	0.0	0.0	0.0	0.0	0.0	0.0	0.0
0.0	0.0	0.0	0.0	0.0	0.0	0.0	0.0
0.0	0.0	0.0	0.0	0.0	0.0	0.0	0.0
0.0	0.0	0.0	0.0	0.0	0.0	0.0	0.0
0.0	0.0	0.0	0.0	0.0	0.0	0.0	0.0
0.0	0.0	0.0	0.0	0.0	0.0	0.0	0.0
0.0	0.0	0.0	0.0	0.0	0.0	0.0	0.0
0.0	0.0	0.0	0.0	0.0	0.0	0.0	0.0
0.0	0.0	0.0	0.0	0.0	0.0	0.0	0.0
0.0	0.0	0.0	0.0	0.0	0.0	0.0	0.0
0.0	0.0	0.0	0.0	0.0	0.0	0.0	0.0
0.0	0.0	0.0	0.0	0.0	0.0	0.0	0.0
0.0	0.0	0.0	0.0	0.0	0.0	0.0	0.0
0.0	0.0	0.0	0.0	0.0	0.0	0.0	0.0
0.0	0.0	0.0	0.0	0.0	0.0	0.0	0.0
0.0	0.0	0.0	0.0	0.0	0.0	0.0	0.0
0.0	0.0	1.0	0.0	0.0	0.0	0.0	0.0
0.0	0.0	0.0	0.0	0.0	0.0	0.0	0.0
0.0	0.0	0.0	0.0	0.0	0.0	0.0	0.0
0.0	0.0	0.0	0.0	0.0	0.0	0.0	8.0
0.0	0.0	0.0	0.0	0.0	0.0	0.0	0.0
0.0	0.0	0.0	0.0	0.0	0.0	0.0	0.0
0.0	0.0	0.0	0.0	0.0	0.0	0.0	0.0
0.0	0.0	0.0	0.0	0.0	0.0	0.0	0.0
0.0	0.0	0.0	0.0	0.0	0.0	0.0	0.0
0.0	0.0	0.0	0.0	0.0	0.0	0.0	0.0
0.0	0.0	0.0	0.0	0.0	0.0	0.0	0.0
0.0	0.0	0.0	0.0	0.0	0.0	0.0	0.0
0.0	0.0	0.0	0.0	0.0	0.0	0.0	0.0
0.0	0.0	0.0	0.0	0.0	0.0	0.0	0.0
0.0	0.0	0.0	0.0	0.0	0.0	0.0	0.0
0.0	0.0	0.0	0.0	0.0	0.0	0.0	0.0
0.0	0.0	0.0	0.0	0.0	0.0	0.0	0.0
0.0	0.0	0.0	0.0	0.0	0.0	0.0	0.0
0.0	0.0	0.0	0.0	0.0	0.0	0.0	0.0
0.0	0.0	0.0	0.0	0.0	0.0	0.0	0.0
0.0	0.0	0.0	0.0	0.0	0.0	0.0	0.0
0.0	0.0	0.0	0.0	0.0	0.0	0.0	0.0
0.0	0.0	0.0	0.0	0.0	0.0	0.0	0.0
0.0	0.0	0.0	0.0	0.0	0.0	0.0	0.0
0.0	0.0	0.0	0.0	0.0	0.0	0.0	0.0

0.0	0.0	0.0	0.0	0.0	0.0	0.0	0.0
0.0	0.0	0.0	0.0	0.0	0.0	0.0	0.0
0.0	0.0	0.0	0.0	0.0	0.0	0.0	150.0
0.0	0.0	0.0	0.0	0.0	0.0	0.0	0.0
0.0	0.0	0.0	0.0	0.0	0.0	0.0	0.0
0.0	0.0	0.0	0.0	0.0	0.0	0.0	0.0
0.0	0.0	0.0	0.0	0.0	0.0	0.0	0.0
0.0	0.0	0.0	0.0	0.0	0.0	0.0	0.0
0.0	0.0	0.0	0.0	0.0	0.0	0.0	0.0
0.0	0.0	0.0	0.0	0.0	0.0	0.0	0.0
0.0	0.0	0.0	0.0	0.0	0.0	0.0	0.0
0.0	0.0	0.0	0.0	0.0	0.0	0.0	0.0
0.0	0.0	0.0	0.0	0.0	0.0	0.0	0.0
0.0	0.0	0.0	0.0	0.0	0.0	0.0	0.0
0.0	0.0	0.0	0.0	0.0	0.0	0.0	0.0
0.0	0.0	0.0	0.0	0.0	0.0	0.0	0.0
0.0	0.0	0.0	0.0	1.0	0.0	0.0	0.0
0.0	0.0	0.0	0.0	0.0	0.0	0.0	0.0
0.0	0.0	0.0	0.0	0.0	0.0	0.0	0.0
0.0	0.0	0.0	0.0	0.0	0.0	0.0	0.0
0.0	0.0	4.0	0.0	0.0	0.0	0.0	0.0
0.0	0.0	0.0	0.0	0.0	0.0	0.0	0.0
0.0	0.0	0.0	0.0	0.0	0.0	0.0	0.0
0.0	0.0	0.0	0.0	0.0	0.0	0.0	0.0
0.0	0.0	0.0	0.0	0.0	0.0	0.0	0.0
0.0	0.0	0.0	0.0	0.0	0.0	0.0	0.0
0.0	0.0	0.0	0.0	0.0	0.0	0.0	0.0
0.0	0.0	0.0	0.0	0.0	0.0	0.0	0.0
0.0	0.0	0.0	0.0	0.0	0.0	0.0	0.0
1.0	0.0	0.0	0.0	0.0	0.0	0.0	0.0
0.0	0.0	0.0	0.0	0.0	0.0	0.0	0.0
0.0	0.0	0.0	0.0	0.0	0.0	0.0	51.0
0.0	0.0	0.0	0.0	0.0	0.0	0.0	0.0
0.0	0.0	0.0	0.0	0.0	0.0	0.0	0.0
0.0	0.0	0.0	0.0	0.0	0.0	0.0	0.0
0.0	0.0	0.0	0.0	0.0	0.0	0.0	0.0
0.0	0.0	0.0	0.0	0.0	0.0	0.0	0.0
0.0	0.0	0.0	0.0	0.0	0.0	0.0	0.0
0.0	0.0	0.0	0.0	0.0	0.0	0.0	0.0
0.0	0.0	0.0	0.0	0.0	0.0	0.0	0.0
0.0	0.0	0.0	0.0	0.0	0.0	0.0	0.0
0.0	0.0	0.0	0.0	0.0	0.0	0.0	0.0
0.0	0.0	0.0	0.0	0.0	0.0	0.0	0.0
0.0	0.0	0.0	0.0	0.0	0.0	0.0	0.0
0.0	0.0	0.0	0.0	0.0	0.0	0.0	0.0
0.0	0.0	0.0	0.0	0.0	0.0	0.0	0.0
0.0	0.0	0.0	0.0	0.0	0.0	0.0	0.0

0.0	0.0	0.0	0.0	0.0	0.0	0.0	0.0
0.0	0.0	0.0	0.0	0.0	0.0	0.0	0.0
0.0	0.0	0.0	0.0	0.0	0.0	0.0	0.0
0.0	0.0	0.0	0.0	1.0	0.0	0.0	0.0
0.0	0.0	0.0	0.0	0.0	0.0	0.0	0.0
0.0	0.0	0.0	0.0	0.0	0.0	0.0	0.0
0.0	0.0	0.0	0.0	0.0	0.0	0.0	0.0
0.0	0.0	0.0	0.0	0.0	0.0	0.0	0.0
0.0	0.0	0.0	0.0	0.0	0.0	0.0	0.0
0.0	0.0	0.0	0.0	0.0	0.0	0.0	0.0
0.0	0.0	0.0	0.0	0.0	0.0	0.0	0.0
0.0	0.0	0.0	0.0	0.0	0.0	0.0	0.0
0.0	0.0	0.0	0.0	0.0	0.0	0.0	0.0
0.0	0.0	0.0	0.0	0.0	0.0	0.0	0.0
0.0	0.0	0.0	0.0	0.0	0.0	0.0	0.0
0.0	0.0	0.0	0.0	0.0	0.0	0.0	0.0
0.0	0.0	0.0	0.0	0.0	0.0	0.0	0.0
0.0	0.0	0.0	0.0	1.0	0.0	0.0	0.0
0.0	0.0	0.0	0.0	0.0	0.0	1.0	0.0
0.0	0.0	0.0	0.0	0.0	0.0	0.0	0.0
0.0	0.0	0.0	0.0	0.0	0.0	0.0	0.0
0.0	0.0	0.0	11.0	0.0	0.0	0.0	13.0
0.0	0.0	0.0	0.0	0.0	0.0	0.0	0.0
0.0	0.0	0.0	0.0	1.0	0.0	0.0	0.0
0.0	0.0	0.0	0.0	0.0	0.0	0.0	0.0
0.0	0.0	0.0	0.0	0.0	0.0	0.0	0.0
0.0	0.0	0.0	0.0	0.0	0.0	0.0	0.0
0.0	0.0	0.0	0.0	0.0	0.0	0.0	0.0
0.0	0.0	0.0	0.0	0.0	0.0	0.0	0.0
0.0	0.0	0.0	0.0	0.0	0.0	7.0	0.0
0.0	0.0	0.0	0.0	0.0	0.0	0.0	0.0
0.0	0.0	0.0	0.0	0.0	0.0	0.0	0.0
2.0	0.0	0.0	0.0	0.0	0.0	0.0	0.0
0.0	0.0	0.0	0.0	0.0	0.0	0.0	0.0
0.0	0.0	0.0	0.0	0.0	0.0	0.0	0.0
0.0	0.0	0.0	0.0	0.0	0.0	0.0	0.0
0.0	0.0	0.0	0.0	0.0	0.0	1.0	0.0
0.0	0.0	0.0	0.0	0.0	0.0	0.0	0.0
0.0	0.0	0.0	0.0	0.0	0.0	0.0	0.0
0.0	0.0	0.0	0.0	0.0	0.0	0.0	0.0
0.0	0.0	0.0	0.0	0.0	0.0	0.0	0.0
0.0	0.0	0.0	0.0	0.0	0.0	0.0	0.0
0.0	0.0	0.0	0.0	0.0	0.0	0.0	0.0
0.0	0.0	0.0	0.0	0.0	0.0	0.0	0.0
0.0	0.0	0.0	0.0	0.0	0.0	0.0	0.0
0.0	0.0	0.0	0.0	0.0	0.0	0.0	0.0
0.0	0.0	0.0	0.0	0.0	0.0	0.0	0.0

0.0	0.0	0.0	0.0	0.0	0.0	0.0	0.0
0.0	0.0	0.0	0.0	0.0	0.0	0.0	0.0
0.0	0.0	0.0	0.0	0.0	0.0	0.0	0.0
0.0	0.0	0.0	0.0	0.0	0.0	0.0	0.0
0.0	0.0	0.0	0.0	0.0	0.0	0.0	0.0
0.0	0.0	0.0	0.0	0.0	0.0	0.0	0.0
0.0	0.0	0.0	0.0	0.0	0.0	0.0	0.0
0.0	0.0	0.0	0.0	0.0	0.0	0.0	0.0
0.0	0.0	0.0	0.0	0.0	0.0	0.0	0.0
0.0	0.0	0.0	0.0	0.0	0.0	0.0	0.0
0.0	0.0	0.0	0.0	0.0	0.0	0.0	0.0
0.0	0.0	0.0	0.0	0.0	0.0	0.0	0.0
0.0	0.0	0.0	0.0	0.0	0.0	0.0	0.0
0.0	0.0	0.0	0.0	0.0	0.0	0.0	0.0
0.0	0.0	0.0	0.0	0.0	0.0	0.0	0.0
0.0	0.0	0.0	0.0	0.0	0.0	0.0	0.0
0.0	0.0	0.0	0.0	0.0	0.0	0.0	0.0
0.0	0.0	0.0	0.0	0.0	0.0	0.0	0.0
0.0	0.0	0.0	0.0	0.0	0.0	0.0	0.0
0.0	0.0	0.0	0.0	0.0	0.0	0.0	0.0
0.0	0.0	0.0	0.0	0.0	0.0	0.0	0.0
0.0	0.0	0.0	0.0	0.0	0.0	0.0	0.0
0.0	0.0	0.0	0.0	0.0	0.0	0.0	0.0
0.0	0.0	0.0	0.0	0.0	0.0	0.0	0.0
0.0	0.0	0.0	0.0	0.0	0.0	0.0	0.0
0.0	0.0	0.0	0.0	0.0	0.0	0.0	0.0
0.0	0.0	0.0	0.0	0.0	0.0	0.0	0.0
0.0	0.0	0.0	0.0	0.0	0.0	0.0	0.0
0.0	0.0	0.0	0.0	0.0	0.0	0.0	0.0
0.0	0.0	0.0	0.0	0.0	0.0	0.0	0.0
0.0	0.0	0.0	0.0	0.0	0.0	0.0	0.0
0.0	0.0	0.0	0.0	0.0	0.0	0.0	0.0
0.0	0.0	0.0	0.0	0.0	0.0	0.0	0.0
0.0	0.0	0.0	0.0	0.0	0.0	0.0	0.0
0.0	0.0	0.0	0.0	0.0	0.0	0.0	0.0
0.0	0.0	0.0	0.0	0.0	0.0	0.0	0.0
0.0	0.0	0.0	0.0	0.0	0.0	0.0	0.0
0.0	0.0	0.0	0.0	0.0	0.0	0.0	0.0
0.0	0.0	0.0	0.0	0.0	0.0	0.0	0.0
25.0	0.0	0.0	0.0	0.0	0.0	0.0	0.0
0.0	0.0	0.0	0.0	0.0	0.0	0.0	0.0
0.0	0.0	0.0	0.0	0.0	0.0	0.0	0.0
0.0	0.0	0.0	0.0	0.0	0.0	0.0	0.0
0.0	0.0	0.0	0.0	0.0	0.0	0.0	0.0
0.0	0.0	0.0	0.0	0.0	0.0	0.0	0.0
0.0	0.0	0.0	0.0	0.0	0.0	0.0	0.0
0.0	0.0	0.0	0.0	0.0	0.0	0.0	0.0

0.0	0.0	0.0	0.0	0.0	0.0	0.0	0.0
0.0	0.0	0.0	0.0	0.0	0.0	0.0	0.0
0.0	0.0	0.0	0.0	0.0	0.0	0.0	0.0
0.0	0.0	0.0	0.0	0.0	0.0	0.0	0.0
0.0	0.0	0.0	0.0	0.0	0.0	0.0	0.0
0.0	0.0	0.0	0.0	0.0	0.0	0.0	0.0
0.0	0.0	0.0	0.0	0.0	0.0	0.0	0.0
0.0	0.0	0.0	0.0	0.0	0.0	0.0	0.0
0.0	0.0	0.0	0.0	0.0	0.0	0.0	0.0
0.0	0.0	0.0	0.0	0.0	0.0	0.0	0.0
0.0	0.0	0.0	0.0	0.0	0.0	0.0	0.0
0.0	0.0	0.0	0.0	0.0	0.0	0.0	0.0
0.0	0.0	0.0	0.0	0.0	0.0	0.0	0.0
0.0	0.0	0.0	0.0	0.0	0.0	0.0	0.0
2.0	0.0	0.0	0.0	0.0	0.0	0.0	0.0
0.0	0.0	0.0	0.0	0.0	0.0	0.0	0.0
0.0	0.0	0.0	0.0	0.0	0.0	0.0	0.0
0.0	0.0	0.0	0.0	0.0	0.0	0.0	0.0
0.0	0.0	0.0	0.0	0.0	0.0	0.0	0.0
0.0	0.0	0.0	0.0	0.0	0.0	0.0	0.0
0.0	0.0	0.0	0.0	0.0	0.0	0.0	0.0
0.0	0.0	0.0	0.0	0.0	0.0	0.0	0.0
0.0	0.0	0.0	0.0	0.0	0.0	0.0	0.0
0.0	0.0	0.0	0.0	0.0	0.0	0.0	0.0
0.0	0.0	0.0	0.0	0.0	0.0	0.0	0.0
0.0	0.0	0.0	0.0	0.0	0.0	0.0	0.0
0.0	0.0	0.0	0.0	0.0	0.0	0.0	0.0
0.0	0.0	0.0	0.0	0.0	0.0	0.0	0.0
0.0	0.0	0.0	0.0	0.0	0.0	0.0	0.0
0.0	0.0	0.0	0.0	0.0	0.0	0.0	0.0
0.0	0.0	0.0	0.0	0.0	0.0	0.0	0.0
0.0	0.0	0.0	0.0	0.0	0.0	0.0	0.0
0.0	0.0	0.0	0.0	0.0	0.0	0.0	0.0
0.0	0.0	0.0	0.0	0.0	0.0	0.0	0.0
0.0	0.0	0.0	0.0	0.0	0.0	0.0	0.0
0.0	0.0	0.0	0.0	0.0	0.0	0.0	0.0
0.0	0.0	0.0	0.0	0.0	0.0	0.0	0.0
0.0	0.0	0.0	0.0	0.0	0.0	0.0	0.0
0.0	0.0	0.0	0.0	0.0	0.0	0.0	0.0
0.0	0.0	0.0	0.0	0.0	0.0	0.0	0.0
0.0	0.0	0.0	0.0	0.0	0.0	0.0	0.0
0.0	0.0	0.0	0.0	0.0	0.0	0.0	0.0
0.0	0.0	0.0	0.0	0.0	0.0	0.0	0.0
0.0	0.0	0.0	0.0	0.0	0.0	0.0	0.0
0.0	0.0	0.0	0.0	0.0	0.0	0.0	0.0
0.0	0.0	0.0	0.0	0.0	0.0	0.0	0.0
0.0	0.0	0.0	0.0	0.0	0.0	0.0	0.0

0.0	0.0	0.0	0.0	0.0	0.0	0.0	0.0
0.0	0.0	0.0	0.0	0.0	0.0	0.0	0.0
0.0	0.0	0.0	0.0	0.0	0.0	0.0	0.0
0.0	0.0	0.0	0.0	0.0	0.0	0.0	0.0
0.0	0.0	0.0	0.0	0.0	0.0	0.0	0.0
0.0	0.0	0.0	0.0	0.0	0.0	0.0	0.0
0.0	0.0	0.0	0.0	0.0	0.0	0.0	0.0
0.0	0.0	0.0	0.0	0.0	0.0	0.0	0.0
0.0	0.0	0.0	0.0	0.0	0.0	0.0	0.0
0.0	0.0	0.0	0.0	0.0	0.0	0.0	0.0
0.0	0.0	0.0	0.0	0.0	0.0	0.0	0.0
0.0	0.0	0.0	0.0	0.0	0.0	0.0	0.0
0.0	0.0	0.0	0.0	0.0	0.0	0.0	0.0
0.0	0.0	0.0	0.0	0.0	0.0	0.0	0.0
0.0	0.0	0.0	0.0	0.0	0.0	0.0	0.0
0.0	0.0	0.0	0.0	0.0	0.0	0.0	0.0
0.0	0.0	0.0	0.0	0.0	0.0	0.0	0.0
0.0	0.0	0.0	0.0	0.0	0.0	0.0	0.0
0.0	0.0	0.0	0.0	0.0	0.0	0.0	0.0
0.0	0.0	0.0	0.0	0.0	0.0	0.0	0.0
0.0	0.0	0.0	0.0	0.0	0.0	0.0	0.0
0.0	0.0	0.0	0.0	0.0	0.0	0.0	0.0
0.0	0.0	0.0	0.0	5.0	0.0	0.0	2.0
0.0	0.0	0.0	0.0	0.0	0.0	0.0	0.0
0.0	0.0	0.0	0.0	0.0	0.0	0.0	0.0
0.0	0.0	0.0	0.0	0.0	0.0	0.0	0.0
0.0	0.0	0.0	0.0	0.0	0.0	0.0	0.0
0.0	0.0	0.0	0.0	0.0	0.0	0.0	0.0
0.0	0.0	0.0	0.0	0.0	0.0	0.0	0.0
0.0	0.0	0.0	0.0	0.0	0.0	0.0	0.0
0.0	0.0	0.0	0.0	0.0	0.0	0.0	0.0
0.0	0.0	0.0	0.0	0.0	0.0	0.0	0.0
0.0	0.0	0.0	0.0	0.0	0.0	0.0	0.0
0.0	0.0	0.0	0.0	0.0	0.0	0.0	0.0
0.0	0.0	0.0	0.0	0.0	0.0	0.0	0.0
0.0	0.0	0.0	0.0	0.0	1.0	0.0	1.0
0.0	0.0	0.0	0.0	0.0	0.0	0.0	0.0
0.0	0.0	0.0	0.0	0.0	0.0	0.0	0.0
0.0	0.0	0.0	0.0	0.0	0.0	0.0	0.0
0.0	0.0	0.0	0.0	0.0	0.0	0.0	0.0
0.0	0.0	0.0	0.0	0.0	0.0	0.0	0.0
0.0	0.0	0.0	0.0	0.0	0.0	0.0	0.0
0.0	0.0	0.0	0.0	0.0	0.0	0.0	0.0
0.0	0.0	0.0	0.0	0.0	0.0	0.0	0.0
0.0	0.0	0.0	0.0	0.0	0.0	0.0	0.0
0.0	0.0	0.0	0.0	0.0	0.0	0.0	0.0
0.0	0.0	0.0	0.0	0.0	0.0	0.0	0.0

0.0	0.0	0.0	0.0	0.0	0.0	0.0	0.0
0.0	0.0	0.0	0.0	0.0	0.0	0.0	0.0
0.0	0.0	0.0	0.0	0.0	0.0	0.0	0.0
0.0	0.0	0.0	0.0	0.0	0.0	0.0	0.0
0.0	0.0	0.0	0.0	0.0	0.0	0.0	0.0
1.0	0.0	0.0	0.0	0.0	0.0	0.0	0.0
0.0	0.0	0.0	0.0	0.0	0.0	0.0	0.0
0.0	0.0	0.0	0.0	0.0	0.0	0.0	0.0
0.0	0.0	0.0	0.0	0.0	0.0	0.0	0.0
0.0	0.0	0.0	0.0	0.0	0.0	0.0	0.0
0.0	0.0	0.0	0.0	0.0	0.0	0.0	0.0
0.0	0.0	0.0	0.0	0.0	0.0	0.0	0.0
0.0	0.0	0.0	0.0	0.0	0.0	0.0	0.0
0.0	0.0	0.0	0.0	0.0	0.0	0.0	0.0
0.0	0.0	0.0	0.0	0.0	0.0	0.0	0.0
0.0	0.0	0.0	0.0	0.0	0.0	0.0	0.0
0.0	0.0	0.0	0.0	0.0	0.0	0.0	0.0
0.0	0.0	0.0	0.0	0.0	0.0	0.0	0.0
0.0	0.0	0.0	0.0	0.0	0.0	0.0	0.0
0.0	0.0	0.0	0.0	0.0	0.0	0.0	0.0
0.0	0.0	0.0	0.0	0.0	0.0	0.0	0.0
0.0	0.0	0.0	0.0	0.0	0.0	0.0	0.0
0.0	0.0	0.0	0.0	0.0	0.0	0.0	0.0
0.0	0.0	0.0	0.0	0.0	0.0	0.0	0.0
0.0	0.0	0.0	0.0	0.0	0.0	0.0	0.0
0.0	0.0	0.0	0.0	0.0	0.0	0.0	0.0
0.0	0.0	0.0	0.0	0.0	0.0	0.0	0.0
0.0	0.0	0.0	0.0	0.0	0.0	0.0	0.0
0.0	0.0	0.0	0.0	0.0	0.0	0.0	0.0
0.0	0.0	0.0	0.0	0.0	0.0	0.0	0.0
0.0	0.0	0.0	0.0	0.0	0.0	0.0	0.0
0.0	0.0	0.0	0.0	0.0	0.0	0.0	0.0
0.0	0.0	0.0	0.0	0.0	0.0	0.0	0.0
0.0	0.0	0.0	0.0	0.0	0.0	0.0	0.0
0.0	0.0	0.0	0.0	0.0	0.0	0.0	0.0
0.0	0.0	0.0	0.0	0.0	0.0	0.0	0.0
0.0	0.0	0.0	0.0	0.0	0.0	0.0	0.0
0.0	0.0	0.0	0.0	0.0	0.0	0.0	0.0
0.0	0.0	0.0	0.0	0.0	0.0	0.0	0.0
0.0	0.0	0.0	0.0	0.0	0.0	0.0	0.0
0.0	0.0	0.0	0.0	0.0	0.0	0.0	0.0
0.0	0.0	0.0	0.0	0.0	0.0	0.0	0.0
0.0	0.0	0.0	0.0	0.0	0.0	0.0	0.0
0.0	0.0	0.0	0.0	0.0	0.0	0.0	0.0
0.0	0.0	0.0	0.0	0.0	0.0	0.0	0.0
0.0	0.0	0.0	0.0	0.0	0.0	0.0	0.0
0.0	0.0	0.0	0.0	0.0	0.0	0.0	0.0

0.0	0.0	0.0	0.0	0.0	0.0	0.0	0.0
0.0	0.0	0.0	0.0	0.0	0.0	0.0	0.0
0.0	0.0	0.0	0.0	0.0	0.0	0.0	0.0
0.0	0.0	0.0	0.0	0.0	0.0	0.0	0.0
0.0	0.0	0.0	0.0	0.0	0.0	0.0	0.0
0.0	0.0	0.0	0.0	0.0	0.0	0.0	0.0
0.0	0.0	0.0	0.0	0.0	0.0	0.0	0.0
0.0	0.0	0.0	0.0	0.0	0.0	0.0	0.0
0.0	0.0	0.0	0.0	0.0	0.0	0.0	0.0
0.0	0.0	0.0	0.0	0.0	0.0	0.0	0.0
0.0	0.0	0.0	0.0	0.0	0.0	0.0	0.0
0.0	0.0	0.0	0.0	0.0	0.0	0.0	0.0
0.0	0.0	0.0	0.0	0.0	0.0	0.0	0.0
0.0	0.0	0.0	0.0	0.0	0.0	0.0	0.0
0.0	0.0	0.0	0.0	0.0	0.0	0.0	0.0
0.0	0.0	0.0	0.0	1.0	0.0	0.0	0.0
0.0	0.0	0.0	0.0	0.0	0.0	0.0	0.0
0.0	0.0	0.0	0.0	1.0	0.0	0.0	0.0
0.0	0.0	0.0	0.0	0.0	0.0	0.0	0.0
0.0	0.0	0.0	0.0	0.0	0.0	0.0	0.0
0.0	0.0	0.0	0.0	0.0	0.0	0.0	0.0
0.0	0.0	0.0	0.0	0.0	0.0	0.0	0.0
0.0	0.0	0.0	0.0	0.0	0.0	0.0	0.0
0.0	0.0	0.0	0.0	0.0	0.0	0.0	0.0
0.0	0.0	0.0	0.0	0.0	0.0	0.0	0.0
0.0	0.0	0.0	0.0	1.0	0.0	0.0	0.0
0.0	0.0	0.0	0.0	0.0	0.0	0.0	0.0
0.0	0.0	0.0	0.0	0.0	0.0	0.0	0.0
0.0	0.0	0.0	0.0	0.0	0.0	0.0	0.0
0.0	0.0	0.0	0.0	0.0	0.0	0.0	0.0
0.0	0.0	0.0	0.0	0.0	0.0	0.0	0.0
0.0	0.0	0.0	0.0	0.0	0.0	0.0	0.0
0.0	0.0	0.0	0.0	0.0	0.0	0.0	0.0
0.0	0.0	0.0	0.0	0.0	0.0	0.0	0.0
0.0	0.0	0.0	0.0	0.0	0.0	0.0	0.0
0.0	0.0	0.0	0.0	0.0	0.0	0.0	0.0
0.0	0.0	0.0	0.0	0.0	0.0	0.0	0.0
0.0	0.0	4.0	0.0	0.0	0.0	0.0	0.0
0.0	0.0	0.0	0.0	0.0	0.0	0.0	0.0
0.0	0.0	0.0	0.0	0.0	0.0	0.0	0.0
0.0	0.0	0.0	0.0	0.0	0.0	0.0	0.0
0.0	0.0	0.0	0.0	0.0	0.0	0.0	0.0
0.0	0.0	0.0	0.0	0.0	0.0	0.0	0.0
0.0	0.0	0.0	0.0	0.0	0.0	0.0	0.0
0.0	0.0	0.0	0.0	0.0	0.0	0.0	0.0
0.0	0.0	0.0	0.0	0.0	0.0	0.0	0.0
0.0	0.0	0.0	0.0	0.0	0.0	0.0	0.0

0.0	0.0	0.0	0.0	0.0	0.0	0.0	0.0
0.0	0.0	0.0	0.0	0.0	0.0	0.0	0.0
0.0	0.0	0.0	0.0	0.0	0.0	0.0	0.0
0.0	0.0	0.0	0.0	0.0	0.0	0.0	0.0
0.0	0.0	0.0	0.0	0.0	0.0	0.0	0.0
0.0	0.0	0.0	0.0	0.0	0.0	0.0	0.0
0.0	0.0	0.0	0.0	0.0	0.0	0.0	0.0
0.0	0.0	0.0	0.0	0.0	0.0	0.0	0.0
0.0	0.0	0.0	0.0	0.0	0.0	0.0	0.0
0.0	0.0	0.0	0.0	0.0	0.0	0.0	0.0
0.0	0.0	0.0	0.0	0.0	0.0	0.0	0.0
0.0	0.0	0.0	0.0	0.0	0.0	0.0	0.0
0.0	0.0	0.0	0.0	0.0	0.0	0.0	0.0
0.0	0.0	0.0	0.0	0.0	0.0	0.0	0.0
0.0	0.0	0.0	0.0	0.0	0.0	0.0	0.0
0.0	0.0	0.0	0.0	0.0	0.0	0.0	0.0
0.0	0.0	0.0	0.0	0.0	0.0	0.0	0.0
0.0	0.0	0.0	0.0	0.0	0.0	0.0	0.0
0.0	0.0	0.0	0.0	0.0	0.0	0.0	0.0
0.0	0.0	0.0	0.0	0.0	0.0	0.0	0.0
0.0	0.0	0.0	0.0	0.0	0.0	0.0	0.0
0.0	0.0	0.0	0.0	0.0	0.0	0.0	0.0
0.0	0.0	0.0	0.0	0.0	0.0	0.0	0.0
0.0	0.0	0.0	0.0	0.0	0.0	0.0	0.0
0.0	0.0	0.0	0.0	0.0	0.0	0.0	0.0
0.0	0.0	0.0	0.0	0.0	0.0	0.0	0.0
0.0	0.0	0.0	0.0	0.0	0.0	0.0	0.0
0.0	0.0	0.0	0.0	0.0	0.0	0.0	0.0
0.0	0.0	0.0	0.0	0.0	0.0	0.0	0.0
0.0	0.0	0.0	0.0	0.0	0.0	0.0	0.0
0.0	0.0	0.0	0.0	0.0	0.0	0.0	0.0
0.0	0.0	0.0	0.0	0.0	0.0	0.0	0.0
0.0	0.0	0.0	0.0	0.0	0.0	0.0	0.0
0.0	0.0	0.0	0.0	0.0	0.0	0.0	0.0
0.0	0.0	0.0	0.0	0.0	0.0	0.0	0.0
0.0	0.0	0.0	0.0	0.0	0.0	0.0	0.0
0.0	0.0	0.0	0.0	0.0	0.0	0.0	0.0
0.0	0.0	0.0	0.0	0.0	0.0	0.0	0.0
0.0	0.0	0.0	0.0	0.0	0.0	0.0	0.0
0.0	0.0	0.0	0.0	0.0	0.0	0.0	0.0
0.0	0.0	0.0	0.0	0.0	0.0	0.0	0.0
0.0	0.0	0.0	0.0	0.0	0.0	0.0	0.0
0.0	0.0	0.0	0.0	0.0	0.0	0.0	0.0
0.0	0.0	0.0	0.0	0.0	0.0	0.0	0.0
0.0	0.0	0.0	0.0	0.0	0.0	0.0	0.0
0.0	0.0	0.0	0.0	0.0	0.0	0.0	0.0
0.0	0.0	0.0	0.0	0.0	0.0	0.0	0.0

0.0	0.0	0.0	0.0	0.0	0.0	0.0	0.0
0.0	0.0	0.0	0.0	0.0	0.0	0.0	0.0
0.0	0.0	0.0	0.0	0.0	0.0	0.0	0.0
0.0	0.0	0.0	0.0	1.0	0.0	0.0	0.0
0.0	0.0	0.0	0.0	0.0	0.0	0.0	0.0
0.0	0.0	0.0	0.0	0.0	0.0	0.0	0.0
0.0	0.0	0.0	0.0	0.0	0.0	0.0	0.0
0.0	0.0	0.0	0.0	0.0	0.0	0.0	0.0
0.0	0.0	0.0	0.0	0.0	0.0	0.0	0.0
0.0	0.0	0.0	0.0	0.0	0.0	0.0	0.0
0.0	0.0	0.0	0.0	0.0	0.0	0.0	0.0
0.0	0.0	0.0	0.0	0.0	0.0	0.0	0.0
0.0	0.0	0.0	0.0	0.0	0.0	0.0	0.0
0.0	0.0	0.0	0.0	0.0	0.0	0.0	0.0
0.0	0.0	0.0	0.0	0.0	0.0	0.0	0.0
0.0	0.0	0.0	0.0	0.0	0.0	0.0	0.0
0.0	0.0	0.0	0.0	0.0	0.0	0.0	0.0
0.0	0.0	0.0	0.0	0.0	0.0	0.0	0.0
0.0	0.0	0.0	0.0	0.0	0.0	0.0	0.0
0.0	0.0	0.0	0.0	0.0	0.0	0.0	0.0
0.0	0.0	0.0	0.0	0.0	0.0	0.0	0.0
0.0	0.0	0.0	0.0	0.0	0.0	0.0	0.0
0.0	0.0	0.0	0.0	0.0	0.0	0.0	0.0
0.0	0.0	0.0	0.0	0.0	0.0	0.0	0.0
0.0	0.0	0.0	0.0	0.0	0.0	0.0	0.0
0.0	0.0	0.0	0.0	0.0	0.0	0.0	0.0
0.0	0.0	0.0	0.0	0.0	0.0	0.0	0.0
0.0	0.0	0.0	0.0	0.0	0.0	0.0	0.0
0.0	0.0	0.0	0.0	0.0	0.0	0.0	0.0
0.0	0.0	0.0	0.0	0.0	0.0	0.0	0.0
0.0	0.0	0.0	0.0	0.0	0.0	0.0	0.0
0.0	0.0	0.0	0.0	0.0	0.0	0.0	0.0
0.0	0.0	0.0	0.0	0.0	0.0	0.0	0.0
0.0	0.0	0.0	0.0	0.0	0.0	0.0	0.0
0.0	0.0	0.0	0.0	0.0	0.0	0.0	0.0
0.0	0.0	0.0	0.0	0.0	0.0	0.0	0.0
0.0	0.0	0.0	0.0	0.0	0.0	0.0	0.0
0.0	0.0	0.0	0.0	0.0	0.0	0.0	0.0
0.0	0.0	0.0	0.0	1.0	0.0	0.0	0.0
0.0	0.0	0.0	0.0	6.0	0.0	0.0	0.0
0.0	0.0	0.0	0.0	0.0	0.0	0.0	0.0
0.0	0.0	0.0	0.0	0.0	0.0	0.0	0.0
0.0	0.0	0.0	0.0	0.0	0.0	0.0	0.0
0.0	0.0	0.0	0.0	0.0	0.0	0.0	0.0
0.0	0.0	0.0	0.0	0.0	0.0	0.0	0.0
0.0	0.0	0.0	0.0	0.0	0.0	0.0	0.0
0.0	0.0	0.0	0.0	0.0	0.0	0.0	0.0

0.0	0.0	0.0	0.0	0.0	0.0	0.0	0.0
0.0	0.0	0.0	0.0	0.0	0.0	0.0	0.0
0.0	0.0	0.0	0.0	0.0	0.0	0.0	0.0
0.0	0.0	0.0	0.0	0.0	0.0	0.0	0.0
0.0	0.0	0.0	0.0	0.0	0.0	0.0	0.0
0.0	0.0	0.0	0.0	0.0	0.0	0.0	0.0
0.0	0.0	0.0	0.0	0.0	0.0	0.0	0.0
0.0	0.0	0.0	0.0	0.0	0.0	0.0	0.0
0.0	0.0	0.0	0.0	0.0	0.0	0.0	0.0
0.0	0.0	0.0	0.0	0.0	0.0	0.0	0.0
0.0	0.0	0.0	0.0	0.0	0.0	0.0	0.0
0.0	0.0	0.0	0.0	0.0	0.0	0.0	0.0
0.0	0.0	0.0	0.0	0.0	0.0	0.0	0.0
0.0	0.0	0.0	0.0	0.0	0.0	0.0	0.0
0.0	0.0	0.0	0.0	0.0	0.0	0.0	0.0
0.0	0.0	0.0	0.0	0.0	0.0	0.0	0.0
0.0	0.0	0.0	0.0	0.0	0.0	0.0	0.0
0.0	0.0	0.0	0.0	0.0	0.0	0.0	0.0
0.0	0.0	0.0	0.0	0.0	0.0	0.0	0.0
0.0	0.0	0.0	0.0	1.0	0.0	0.0	0.0
0.0	0.0	0.0	0.0	0.0	0.0	0.0	0.0
0.0	0.0	0.0	0.0	0.0	0.0	0.0	0.0
0.0	0.0	0.0	0.0	0.0	0.0	0.0	0.0
0.0	0.0	0.0	0.0	0.0	0.0	0.0	0.0
0.0	0.0	0.0	0.0	0.0	0.0	0.0	0.0
0.0	0.0	0.0	0.0	0.0	0.0	0.0	0.0
0.0	0.0	0.0	0.0	0.0	0.0	0.0	0.0
0.0	0.0	0.0	0.0	0.0	0.0	0.0	0.0
0.0	0.0	0.0	0.0	0.0	0.0	0.0	0.0
0.0	0.0	0.0	0.0	0.0	0.0	0.0	0.0
0.0	0.0	0.0	0.0	0.0	0.0	0.0	0.0
0.0	0.0	0.0	0.0	0.0	0.0	0.0	0.0
0.0	0.0	0.0	0.0	0.0	0.0	0.0	0.0
0.0	0.0	0.0	0.0	0.0	0.0	0.0	0.0
0.0	0.0	0.0	0.0	2.0	0.0	0.0	0.0
0.0	0.0	0.0	0.0	0.0	0.0	0.0	0.0
0.0	0.0	0.0	0.0	0.0	0.0	0.0	0.0
4.0	0.0	0.0	0.0	0.0	0.0	0.0	0.0
0.0	0.0	0.0	0.0	0.0	0.0	0.0	0.0
0.0	0.0	0.0	0.0	0.0	0.0	0.0	0.0
0.0	0.0	0.0	0.0	0.0	0.0	0.0	0.0
0.0	0.0	0.0	0.0	0.0	0.0	0.0	0.0
0.0	0.0	0.0	0.0	0.0	0.0	0.0	0.0
0.0	0.0	0.0	0.0	0.0	0.0	0.0	0.0
0.0	0.0	0.0	0.0	0.0	0.0	0.0	0.0
0.0	0.0	0.0	0.0	0.0	0.0	0.0	0.0
0.0	0.0	0.0	0.0	0.0	0.0	0.0	0.0

0.0	0.0	0.0	0.0	0.0	0.0	0.0	0.0
0.0	0.0	0.0	0.0	0.0	0.0	0.0	0.0
163.0	0.0	0.0	0.0	0.0	0.0	0.0	0.0
104.0	0.0	0.0	0.0	0.0	0.0	0.0	0.0
6.0	0.0	0.0	0.0	0.0	0.0	0.0	0.0
5.0	0.0	0.0	0.0	0.0	0.0	0.0	0.0
9.0	0.0	0.0	0.0	0.0	0.0	2.0	0.0
2.0	0.0	0.0	0.0	0.0	0.0	0.0	0.0
4.0	0.0	0.0	0.0	0.0	0.0	0.0	0.0
2.0	0.0	0.0	0.0	0.0	2.0	0.0	0.0
6.0	0.0	0.0	0.0	0.0	0.0	0.0	0.0
2.0	0.0	0.0	0.0	0.0	0.0	0.0	0.0
1.0	0.0	0.0	0.0	0.0	0.0	0.0	0.0
1.0	0.0	0.0	0.0	0.0	0.0	0.0	0.0
1.0	0.0	0.0	0.0	0.0	0.0	0.0	0.0
1.0	0.0	0.0	0.0	0.0	0.0	0.0	0.0
1.0	0.0	0.0	0.0	0.0	0.0	0.0	0.0
0.0	0.0	0.0	0.0	0.0	0.0	0.0	0.0
0.0	0.0	0.0	0.0	0.0	0.0	0.0	0.0
2.0	0.0	0.0	0.0	0.0	0.0	0.0	0.0
1.0	0.0	0.0	0.0	0.0	0.0	0.0	0.0
3.0	0.0	0.0	0.0	0.0	0.0	0.0	0.0
1.0	0.0	0.0	0.0	0.0	0.0	0.0	0.0
1.0	0.0	0.0	0.0	0.0	0.0	0.0	0.0
1.0	0.0	0.0	0.0	0.0	0.0	0.0	0.0
0.0	0.0	0.0	0.0	0.0	0.0	0.0	0.0
1.0	0.0	0.0	0.0	0.0	0.0	0.0	0.0
0.0	0.0	0.0	0.0	0.0	0.0	0.0	0.0
0.0	0.0	0.0	0.0	0.0	0.0	0.0	0.0
1.0	0.0	0.0	0.0	0.0	0.0	0.0	0.0
0.0	0.0	0.0	0.0	0.0	0.0	0.0	0.0
1.0	0.0	0.0	0.0	0.0	0.0	0.0	0.0
0.0	0.0	0.0	0.0	0.0	0.0	0.0	0.0
1.0	0.0	0.0	0.0	0.0	0.0	0.0	0.0
1.0	0.0	0.0	0.0	0.0	0.0	0.0	0.0
2.0	0.0	0.0	0.0	0.0	0.0	0.0	0.0
1.0	0.0	0.0	0.0	0.0	0.0	0.0	0.0
1.0	0.0	0.0	0.0	0.0	0.0	0.0	0.0
1.0	0.0	0.0	0.0	0.0	0.0	0.0	0.0
1.0	0.0	0.0	0.0	0.0	0.0	0.0	0.0
1.0	0.0	0.0	0.0	0.0	0.0	0.0	0.0
1.0	0.0	0.0	0.0	0.0	0.0	0.0	0.0
1.0	0.0	0.0	0.0	0.0	0.0	0.0	0.0
0.0	0.0	0.0	0.0	0.0	0.0	0.0	0.0
1.0	0.0	0.0	0.0	0.0	0.0	0.0	0.0
0.0	3616.0	0.0	0.0	0.0	0.0	0.0	0.0
0.0	16.0	0.0	0.0	0.0	0.0	0.0	0.0

0.0	8.0	0.0	0.0	0.0	0.0	0.0	0.0
0.0	3.0	0.0	0.0	0.0	0.0	0.0	0.0
0.0	2.0	0.0	0.0	0.0	0.0	0.0	0.0
0.0	1.0	0.0	0.0	0.0	0.0	0.0	0.0
0.0	1.0	0.0	0.0	0.0	0.0	0.0	0.0
0.0	1.0	0.0	0.0	0.0	0.0	0.0	0.0
0.0	1.0	0.0	0.0	0.0	0.0	0.0	0.0
0.0	2.0	0.0	0.0	0.0	0.0	0.0	0.0
0.0	1.0	0.0	0.0	0.0	0.0	0.0	0.0
0.0	0.0	30.0	0.0	0.0	0.0	0.0	0.0
0.0	0.0	20.0	0.0	0.0	0.0	0.0	0.0
0.0	0.0	10.0	0.0	0.0	0.0	0.0	0.0
0.0	0.0	7.0	0.0	0.0	0.0	0.0	0.0
0.0	0.0	3.0	0.0	0.0	0.0	0.0	0.0
0.0	0.0	4.0	0.0	0.0	0.0	0.0	0.0
0.0	0.0	1.0	0.0	0.0	0.0	0.0	0.0
0.0	0.0	1.0	0.0	0.0	0.0	0.0	0.0
0.0	0.0	2.0	0.0	0.0	0.0	0.0	3.0
0.0	0.0	1.0	0.0	0.0	0.0	0.0	0.0
0.0	0.0	3.0	0.0	0.0	0.0	0.0	0.0
0.0	0.0	0.0	0.0	0.0	0.0	0.0	0.0
0.0	0.0	0.0	0.0	0.0	0.0	0.0	4.0
0.0	0.0	1.0	0.0	0.0	0.0	0.0	0.0
0.0	0.0	1.0	0.0	0.0	0.0	0.0	0.0
0.0	0.0	2.0	0.0	0.0	0.0	0.0	0.0
0.0	0.0	0.0	0.0	2.0	0.0	0.0	6.0
0.0	0.0	1.0	0.0	0.0	0.0	0.0	0.0
0.0	0.0	0.0	0.0	0.0	0.0	0.0	0.0
0.0	0.0	0.0	0.0	0.0	0.0	0.0	0.0
0.0	0.0	1.0	0.0	0.0	0.0	0.0	0.0
0.0	0.0	1.0	0.0	0.0	0.0	0.0	0.0
0.0	0.0	1.0	0.0	0.0	0.0	0.0	0.0
0.0	0.0	1.0	0.0	0.0	0.0	0.0	0.0
0.0	0.0	1.0	0.0	0.0	0.0	0.0	0.0
0.0	0.0	0.0	0.0	0.0	1.0	0.0	0.0
0.0	0.0	1.0	0.0	0.0	0.0	0.0	0.0
0.0	0.0	1.0	0.0	0.0	0.0	0.0	0.0
0.0	0.0	0.0	36.0	0.0	0.0	0.0	0.0
0.0	0.0	0.0	29.0	0.0	0.0	0.0	0.0
0.0	0.0	0.0	31.0	0.0	0.0	0.0	0.0
0.0	0.0	0.0	7.0	0.0	0.0	0.0	0.0
0.0	0.0	0.0	1.0	0.0	0.0	0.0	0.0
0.0	0.0	0.0	1.0	0.0	0.0	0.0	0.0
0.0	0.0	0.0	1.0	0.0	0.0	0.0	0.0
0.0	0.0	0.0	1.0	0.0	0.0	0.0	0.0
0.0	0.0	0.0	1.0	0.0	0.0	0.0	0.0
0.0	0.0	0.0	1.0	0.0	0.0	0.0	0.0

0.0	0.0	0.0	0.0	0.0	0.0	0.0	0.0
0.0	0.0	0.0	0.0	56.0	0.0	0.0	0.0
0.0	0.0	0.0	0.0	35.0	0.0	0.0	0.0
0.0	0.0	0.0	0.0	18.0	0.0	0.0	0.0
0.0	0.0	0.0	0.0	17.0	0.0	0.0	0.0
0.0	0.0	0.0	0.0	15.0	0.0	0.0	0.0
0.0	0.0	0.0	0.0	5.0	0.0	0.0	0.0
0.0	0.0	0.0	0.0	5.0	0.0	0.0	0.0
0.0	0.0	0.0	0.0	4.0	0.0	0.0	0.0
0.0	0.0	0.0	0.0	2.0	0.0	0.0	0.0
0.0	0.0	0.0	0.0	2.0	0.0	0.0	0.0
0.0	0.0	0.0	0.0	1.0	0.0	0.0	0.0
0.0	0.0	0.0	0.0	1.0	0.0	0.0	0.0
0.0	0.0	0.0	0.0	2.0	0.0	0.0	0.0
0.0	0.0	0.0	0.0	3.0	0.0	0.0	0.0
0.0	0.0	0.0	0.0	0.0	0.0	0.0	0.0
0.0	0.0	0.0	0.0	1.0	0.0	0.0	0.0
0.0	0.0	0.0	0.0	1.0	0.0	0.0	0.0
0.0	0.0	0.0	0.0	0.0	0.0	0.0	0.0
0.0	0.0	0.0	0.0	1.0	0.0	0.0	0.0
0.0	0.0	0.0	0.0	1.0	0.0	0.0	0.0
0.0	0.0	0.0	0.0	0.0	0.0	0.0	0.0
0.0	0.0	0.0	0.0	0.0	0.0	0.0	0.0
0.0	0.0	0.0	0.0	1.0	0.0	0.0	0.0
0.0	0.0	0.0	0.0	0.0	0.0	0.0	0.0
0.0	0.0	0.0	0.0	0.0	0.0	0.0	0.0
0.0	0.0	0.0	0.0	1.0	0.0	0.0	0.0
0.0	0.0	0.0	0.0	1.0	0.0	0.0	0.0
0.0	0.0	0.0	0.0	2.0	0.0	0.0	0.0
0.0	0.0	0.0	0.0	1.0	0.0	0.0	0.0
0.0	0.0	0.0	0.0	1.0	0.0	0.0	0.0
0.0	0.0	0.0	0.0	0.0	0.0	0.0	0.0
0.0	0.0	0.0	0.0	0.0	0.0	0.0	0.0
0.0	0.0	0.0	0.0	2.0	0.0	0.0	0.0
0.0	0.0	0.0	0.0	1.0	0.0	0.0	0.0
0.0	0.0	0.0	0.0	1.0	0.0	0.0	0.0
0.0	0.0	0.0	0.0	1.0	0.0	0.0	0.0
0.0	0.0	0.0	0.0	2.0	0.0	0.0	0.0
0.0	0.0	0.0	0.0	1.0	0.0	0.0	0.0
0.0	0.0	0.0	0.0	1.0	0.0	0.0	0.0
0.0	0.0	0.0	0.0	1.0	0.0	0.0	0.0
0.0	0.0	0.0	0.0	1.0	0.0	0.0	0.0
0.0	0.0	0.0	0.0	1.0	0.0	0.0	0.0
0.0	0.0	0.0	0.0	1.0	0.0	0.0	0.0
0.0	0.0	0.0	0.0	1.0	0.0	0.0	0.0
0.0	0.0	0.0	0.0	2.0	0.0	0.0	0.0
0.0	0.0	0.0	0.0	1.0	0.0	0.0	0.0

0.0	0.0	0.0	0.0	0.0	0.0	0.0	0.0
0.0	0.0	0.0	0.0	1.0	0.0	0.0	0.0
0.0	0.0	0.0	0.0	1.0	0.0	0.0	0.0
0.0	0.0	0.0	0.0	1.0	0.0	0.0	0.0
0.0	0.0	0.0	0.0	0.0	0.0	0.0	0.0
0.0	0.0	0.0	0.0	0.0	0.0	0.0	0.0
0.0	0.0	0.0	0.0	0.0	0.0	0.0	0.0
0.0	0.0	0.0	0.0	2.0	0.0	0.0	0.0
0.0	0.0	0.0	0.0	1.0	0.0	0.0	0.0
0.0	0.0	0.0	0.0	1.0	0.0	0.0	0.0
0.0	0.0	0.0	0.0	1.0	0.0	0.0	0.0
0.0	0.0	0.0	0.0	1.0	0.0	0.0	0.0
0.0	0.0	0.0	0.0	1.0	0.0	0.0	0.0
0.0	0.0	0.0	0.0	1.0	0.0	0.0	0.0
0.0	0.0	0.0	0.0	1.0	0.0	0.0	0.0
0.0	0.0	0.0	0.0	1.0	0.0	0.0	0.0
0.0	0.0	0.0	0.0	1.0	0.0	0.0	0.0
0.0	0.0	0.0	0.0	0.0	0.0	0.0	0.0
0.0	0.0	0.0	0.0	1.0	0.0	0.0	0.0
0.0	0.0	0.0	0.0	0.0	0.0	0.0	3.0
0.0	0.0	0.0	0.0	0.0	0.0	0.0	0.0
0.0	0.0	0.0	0.0	1.0	0.0	0.0	0.0
0.0	0.0	0.0	0.0	1.0	0.0	0.0	0.0
0.0	0.0	0.0	0.0	1.0	0.0	0.0	0.0
0.0	0.0	0.0	0.0	0.0	0.0	0.0	0.0
0.0	0.0	0.0	0.0	1.0	0.0	0.0	0.0
0.0	0.0	0.0	0.0	0.0	48.0	0.0	0.0
0.0	0.0	0.0	0.0	0.0	16.0	0.0	0.0
0.0	0.0	0.0	0.0	0.0	15.0	0.0	0.0
0.0	0.0	0.0	0.0	0.0	14.0	0.0	0.0
0.0	0.0	0.0	0.0	0.0	7.0	0.0	0.0
0.0	0.0	0.0	0.0	0.0	3.0	0.0	0.0
0.0	0.0	0.0	0.0	0.0	1.0	0.0	0.0
0.0	0.0	0.0	0.0	0.0	2.0	0.0	30.0
0.0	0.0	0.0	0.0	0.0	2.0	0.0	0.0
0.0	0.0	0.0	0.0	0.0	3.0	0.0	0.0
0.0	0.0	0.0	0.0	0.0	1.0	0.0	0.0
0.0	0.0	0.0	0.0	0.0	1.0	0.0	0.0
0.0	0.0	0.0	0.0	0.0	2.0	0.0	0.0
0.0	0.0	0.0	0.0	0.0	1.0	0.0	0.0
0.0	0.0	0.0	0.0	0.0	1.0	0.0	0.0
0.0	0.0	0.0	0.0	0.0	2.0	0.0	0.0
0.0	0.0	0.0	0.0	0.0	1.0	0.0	0.0
0.0	0.0	0.0	0.0	0.0	1.0	0.0	0.0
0.0	0.0	0.0	0.0	0.0	1.0	0.0	0.0
0.0	0.0	0.0	0.0	0.0	2.0	0.0	0.0
0.0	0.0	0.0	0.0	0.0	0.0	0.0	0.0

0.0	0.0	0.0	0.0	0.0	1.0	0.0	0.0
0.0	0.0	0.0	0.0	0.0	2.0	0.0	0.0
0.0	0.0	0.0	0.0	0.0	0.0	0.0	0.0
0.0	0.0	0.0	0.0	0.0	1.0	0.0	0.0
0.0	0.0	0.0	0.0	0.0	1.0	0.0	0.0
0.0	0.0	0.0	0.0	0.0	0.0	0.0	0.0
0.0	0.0	0.0	0.0	0.0	1.0	0.0	0.0
0.0	0.0	0.0	0.0	0.0	0.0	0.0	0.0
0.0	0.0	0.0	0.0	0.0	0.0	0.0	0.0
0.0	0.0	0.0	0.0	0.0	0.0	0.0	0.0
0.0	0.0	0.0	0.0	0.0	0.0	0.0	0.0
0.0	0.0	0.0	0.0	0.0	0.0	0.0	0.0
0.0	0.0	0.0	0.0	0.0	0.0	0.0	0.0
0.0	0.0	0.0	0.0	0.0	0.0	0.0	0.0
0.0	0.0	0.0	0.0	0.0	0.0	0.0	0.0
0.0	0.0	0.0	0.0	0.0	0.0	0.0	0.0
0.0	0.0	0.0	0.0	0.0	0.0	5229.0	0.0
0.0	0.0	0.0	0.0	0.0	0.0	730.0	0.0
0.0	0.0	0.0	0.0	0.0	0.0	304.0	0.0
0.0	0.0	0.0	0.0	0.0	0.0	50.0	0.0
0.0	0.0	0.0	0.0	0.0	0.0	26.0	0.0
0.0	0.0	0.0	0.0	0.0	0.0	33.0	0.0
0.0	0.0	0.0	0.0	0.0	0.0	15.0	0.0
0.0	0.0	0.0	0.0	0.0	0.0	9.0	0.0
0.0	0.0	0.0	0.0	0.0	0.0	7.0	0.0
0.0	0.0	0.0	0.0	0.0	0.0	2.0	0.0
0.0	0.0	0.0	0.0	0.0	0.0	2.0	0.0
0.0	0.0	0.0	0.0	0.0	0.0	1.0	0.0
0.0	0.0	0.0	0.0	0.0	0.0	1.0	0.0
0.0	0.0	0.0	0.0	0.0	0.0	1.0	0.0
0.0	0.0	0.0	0.0	0.0	0.0	1.0	0.0
0.0	0.0	0.0	0.0	0.0	0.0	1.0	0.0
0.0	0.0	0.0	0.0	0.0	0.0	0.0	33.0
0.0	0.0	0.0	0.0	0.0	0.0	0.0	21.0
0.0	0.0	0.0	0.0	0.0	0.0	0.0	20.0
0.0	0.0	0.0	0.0	0.0	0.0	0.0	11.0
0.0	0.0	0.0	0.0	0.0	0.0	0.0	8.0
0.0	0.0	0.0	0.0	0.0	0.0	0.0	7.0
0.0	0.0	0.0	0.0	0.0	0.0	0.0	10.0
0.0	0.0	0.0	0.0	0.0	0.0	0.0	5.0
0.0	0.0	0.0	0.0	0.0	0.0	0.0	6.0
0.0	0.0	0.0	0.0	0.0	0.0	0.0	4.0
0.0	0.0	0.0	0.0	0.0	0.0	0.0	5.0
0.0	0.0	0.0	0.0	0.0	0.0	0.0	5.0
0.0	0.0	0.0	0.0	0.0	0.0	0.0	3.0
0.0	0.0	0.0	0.0	0.0	0.0	0.0	4.0
0.0	0.0	0.0	0.0	0.0	0.0	0.0	2.0

0.0	0.0	0.0	0.0	0.0	0.0	0.0	4.0
0.0	0.0	0.0	0.0	0.0	0.0	0.0	3.0
0.0	0.0	0.0	0.0	0.0	0.0	0.0	3.0
0.0	0.0	0.0	0.0	0.0	0.0	0.0	1.0
0.0	0.0	0.0	0.0	0.0	0.0	0.0	2.0
0.0	0.0	0.0	0.0	0.0	0.0	0.0	1.0
0.0	0.0	0.0	0.0	0.0	0.0	0.0	1.0
0.0	0.0	0.0	0.0	0.0	0.0	0.0	0.0
0.0	0.0	0.0	0.0	0.0	0.0	0.0	1.0
0.0	0.0	0.0	0.0	0.0	0.0	0.0	1.0
0.0	0.0	0.0	0.0	0.0	0.0	0.0	1.0
0.0	0.0	0.0	0.0	0.0	0.0	0.0	1.0
0.0	0.0	0.0	0.0	0.0	0.0	0.0	1.0
0.0	0.0	0.0	0.0	0.0	0.0	0.0	1.0
0.0	0.0	0.0	0.0	0.0	0.0	0.0	1.0
0.0	0.0	0.0	0.0	0.0	0.0	0.0	1.0
0.0	0.0	0.0	0.0	0.0	0.0	0.0	1.0
0.0	0.0	0.0	0.0	0.0	0.0	0.0	1.0
0.0	0.0	0.0	0.0	0.0	0.0	0.0	1.0
0.0	0.0	0.0	0.0	0.0	0.0	0.0	1.0
0.0	0.0	0.0	0.0	0.0	0.0	0.0	1.0
0.0	0.0	0.0	0.0	0.0	0.0	0.0	0.0
0.0	0.0	0.0	0.0	0.0	0.0	0.0	1.0
0.0	0.0	0.0	0.0	0.0	0.0	0.0	1.0
0.0	0.0	0.0	0.0	0.0	0.0	0.0	0.0
0.0	0.0	0.0	0.0	0.0	0.0	0.0	0.0
0.0	0.0	0.0	0.0	0.0	0.0	0.0	0.0
0.0	0.0	0.0	0.0	0.0	0.0	0.0	0.0
0.0	0.0	0.0	0.0	0.0	0.0	0.0	0.0
0.0	0.0	0.0	0.0	0.0	0.0	0.0	0.0
0.0	0.0	0.0	0.0	0.0	0.0	0.0	0.0
0.0	0.0	0.0	0.0	0.0	0.0	0.0	0.0
0.0	0.0	0.0	0.0	0.0	0.0	0.0	0.0
0.0	0.0	0.0	0.0	0.0	0.0	0.0	0.0
0.0	0.0	0.0	0.0	0.0	0.0	0.0	0.0
0.0	0.0	0.0	0.0	0.0	0.0	0.0	0.0
0.0	0.0	0.0	0.0	0.0	0.0	0.0	0.0
0.0	0.0	0.0	0.0	0.0	0.0	0.0	0.0
0.0	0.0	0.0	0.0	0.0	0.0	0.0	0.0
0.0	0.0	0.0	0.0	0.0	0.0	0.0	0.0
0.0	0.0	0.0	0.0	0.0	0.0	0.0	0.0
0.0	0.0	0.0	0.0	0.0	0.0	0.0	0.0
0.0	0.0	0.0	0.0	0.0	0.0	0.0	0.0
0.0	0.0	0.0	0.0	0.0	0.0	0.0	0.0
0.0	0.0	0.0	0.0	0.0	0.0	0.0	0.0
0.0	0.0	0.0	0.0	0.0	0.0	0.0	0.0
0.0	0.0	0.0	0.0	0.0	0.0	0.0	0.0

0.0	0.0	0.0	0.0	0.0	0.0	0.0	0.0
0.0	0.0	0.0	0.0	0.0	0.0	0.0	0.0
0.0	0.0	0.0	0.0	0.0	0.0	0.0	0.0
0.0	0.0	0.0	0.0	0.0	0.0	0.0	0.0
0.0	0.0	0.0	0.0	0.0	0.0	0.0	0.0
0.0	0.0	0.0	0.0	0.0	0.0	0.0	0.0
0.0	0.0	0.0	0.0	0.0	0.0	0.0	0.0
0.0	0.0	0.0	0.0	0.0	0.0	0.0	0.0
0.0	0.0	0.0	0.0	0.0	0.0	0.0	0.0
0.0	0.0	0.0	0.0	0.0	0.0	0.0	0.0
0.0	0.0	0.0	0.0	0.0	0.0	0.0	0.0
0.0	0.0	0.0	0.0	0.0	0.0	0.0	0.0
0.0	0.0	0.0	0.0	0.0	0.0	0.0	0.0
0.0	0.0	0.0	0.0	0.0	0.0	0.0	0.0
0.0	0.0	0.0	0.0	0.0	0.0	0.0	0.0
0.0	0.0	0.0	0.0	0.0	0.0	0.0	0.0
0.0	0.0	0.0	0.0	0.0	0.0	0.0	0.0
0.0	0.0	0.0	0.0	0.0	0.0	0.0	0.0
0.0	0.0	0.0	0.0	0.0	0.0	0.0	0.0
0.0	0.0	0.0	0.0	0.0	0.0	0.0	0.0
0.0	0.0	0.0	0.0	0.0	0.0	0.0	0.0
0.0	0.0	0.0	0.0	0.0	0.0	0.0	0.0
0.0	0.0	0.0	0.0	0.0	0.0	0.0	0.0
0.0	0.0	0.0	0.0	0.0	0.0	0.0	0.0
0.0	0.0	0.0	0.0	0.0	0.0	0.0	0.0
0.0	0.0	0.0	0.0	0.0	0.0	0.0	0.0
0.0	0.0	0.0	0.0	0.0	0.0	0.0	0.0
0.0	0.0	0.0	0.0	0.0	0.0	0.0	0.0
0.0	0.0	0.0	0.0	0.0	0.0	0.0	0.0
0.0	0.0	0.0	0.0	0.0	0.0	0.0	0.0
0.0	0.0	0.0	0.0	0.0	0.0	0.0	0.0
0.0	0.0	0.0	0.0	0.0	0.0	0.0	0.0
0.0	0.0	0.0	0.0	0.0	0.0	0.0	0.0
0.0	0.0	0.0	0.0	0.0	0.0	0.0	0.0
0.0	0.0	0.0	0.0	0.0	0.0	0.0	0.0
0.0	0.0	0.0	0.0	0.0	0.0	0.0	0.0
0.0	0.0	0.0	0.0	0.0	0.0	0.0	0.0
0.0	0.0	0.0	0.0	0.0	0.0	0.0	0.0
0.0	0.0	0.0	0.0	0.0	0.0	0.0	0.0
0.0	0.0	0.0	0.0	0.0	0.0	0.0	0.0
0.0	0.0	0.0	0.0	0.0	0.0	0.0	0.0
0.0	0.0	0.0	0.0	0.0	0.0	0.0	0.0
0.0	0.0	0.0	0.0	0.0	0.0	0.0	0.0
0.0	0.0	0.0	0.0	0.0	0.0	0.0	0.0
0.0	0.0	0.0	0.0	0.0	0.0	0.0	0.0
0.0	0.0	0.0	0.0	0.0	0.0	0.0	0.0
0.0	0.0	0.0	0.0	0.0	0.0	0.0	0.0
0.0	0.0	0.0	0.0	0.0	0.0	0.0	0.0

0.0	0.0	0.0	0.0	0.0	0.0	0.0	0.0
0.0	0.0	0.0	0.0	0.0	0.0	0.0	0.0
0.0	0.0	0.0	0.0	0.0	0.0	0.0	0.0
0.0	0.0	0.0	0.0	0.0	0.0	0.0	0.0
0.0	0.0	0.0	0.0	0.0	0.0	0.0	0.0
0.0	0.0	0.0	0.0	0.0	0.0	0.0	0.0
0.0	0.0	0.0	0.0	0.0	0.0	0.0	0.0
0.0	0.0	0.0	0.0	0.0	0.0	0.0	0.0
0.0	0.0	0.0	0.0	0.0	0.0	0.0	0.0
0.0	0.0	0.0	0.0	0.0	0.0	0.0	0.0
0.0	0.0	0.0	0.0	0.0	0.0	0.0	0.0
0.0	0.0	0.0	0.0	0.0	0.0	0.0	0.0
0.0	0.0	0.0	0.0	0.0	0.0	0.0	0.0
0.0	0.0	0.0	0.0	0.0	0.0	0.0	0.0
0.0	0.0	0.0	0.0	0.0	0.0	0.0	0.0
0.0	0.0	0.0	0.0	0.0	0.0	0.0	0.0
0.0	0.0	0.0	0.0	0.0	0.0	0.0	0.0
0.0	0.0	0.0	0.0	0.0	0.0	0.0	0.0
0.0	0.0	0.0	0.0	0.0	0.0	0.0	0.0
0.0	0.0	0.0	0.0	0.0	0.0	0.0	0.0
0.0	0.0	0.0	0.0	0.0	0.0	0.0	0.0
0.0	0.0	0.0	0.0	0.0	0.0	0.0	0.0
0.0	0.0	0.0	0.0	0.0	0.0	0.0	0.0
0.0	0.0	0.0	0.0	0.0	0.0	0.0	0.0
0.0	0.0	0.0	0.0	0.0	0.0	0.0	0.0
0.0	0.0	0.0	0.0	0.0	0.0	0.0	0.0
0.0	0.0	0.0	0.0	0.0	0.0	0.0	0.0
0.0	0.0	0.0	0.0	0.0	0.0	0.0	0.0
0.0	0.0	0.0	0.0	0.0	0.0	0.0	0.0
0.0	0.0	0.0	0.0	0.0	0.0	0.0	0.0
0.0	0.0	0.0	0.0	0.0	0.0	0.0	0.0
0.0	0.0	0.0	0.0	0.0	0.0	0.0	0.0
0.0	0.0	0.0	0.0	0.0	0.0	0.0	0.0
0.0	0.0	0.0	0.0	0.0	0.0	0.0	0.0
0.0	0.0	0.0	0.0	0.0	0.0	0.0	0.0
0.0	0.0	0.0	0.0	0.0	0.0	0.0	0.0
0.0	0.0	0.0	0.0	0.0	0.0	0.0	0.0
0.0	0.0	0.0	0.0	0.0	0.0	0.0	0.0
0.0	0.0	0.0	0.0	0.0	0.0	0.0	0.0
0.0	0.0	0.0	0.0	0.0	0.0	0.0	0.0
0.0	0.0	0.0	0.0	0.0	0.0	0.0	0.0
0.0	0.0	0.0	0.0	0.0	0.0	0.0	0.0
0.0	0.0	0.0	0.0	0.0	0.0	0.0	0.0
0.0	0.0	0.0	0.0	0.0	0.0	0.0	0.0
0.0	0.0	0.0	0.0	0.0	0.0	0.0	0.0
0.0	0.0	0.0	0.0	0.0	0.0	0.0	0.0
0.0	0.0	0.0	0.0	0.0	0.0	0.0	0.0

0.0	0.0	0.0	0.0	0.0	0.0	0.0	0.0
0.0	0.0	0.0	0.0	0.0	0.0	0.0	0.0
0.0	0.0	0.0	0.0	0.0	0.0	0.0	0.0
0.0	0.0	0.0	0.0	0.0	0.0	0.0	0.0
0.0	0.0	0.0	0.0	0.0	0.0	0.0	0.0
0.0	0.0	0.0	0.0	0.0	0.0	0.0	0.0
0.0	0.0	0.0	0.0	0.0	0.0	0.0	0.0
0.0	0.0	0.0	0.0	0.0	0.0	0.0	0.0
0.0	0.0	0.0	0.0	0.0	0.0	0.0	0.0
0.0	0.0	0.0	0.0	0.0	0.0	0.0	0.0
0.0	0.0	0.0	0.0	0.0	0.0	0.0	0.0
0.0	0.0	0.0	0.0	0.0	0.0	0.0	0.0
0.0	0.0	0.0	0.0	0.0	0.0	0.0	0.0
0.0	0.0	0.0	0.0	0.0	0.0	0.0	0.0
0.0	0.0	0.0	0.0	0.0	0.0	0.0	0.0
0.0	0.0	0.0	0.0	0.0	0.0	0.0	0.0
0.0	0.0	0.0	0.0	0.0	0.0	0.0	0.0
0.0	0.0	0.0	0.0	0.0	0.0	0.0	0.0
0.0	0.0	0.0	0.0	0.0	0.0	0.0	0.0
0.0	0.0	0.0	0.0	0.0	0.0	0.0	0.0
0.0	0.0	0.0	0.0	0.0	0.0	0.0	0.0
0.0	0.0	0.0	0.0	0.0	0.0	0.0	0.0
0.0	0.0	0.0	0.0	0.0	0.0	0.0	0.0
0.0	0.0	0.0	0.0	0.0	0.0	0.0	0.0
0.0	0.0	0.0	0.0	0.0	0.0	0.0	0.0
0.0	0.0	0.0	0.0	0.0	0.0	0.0	0.0
0.0	0.0	0.0	0.0	0.0	0.0	0.0	0.0
0.0	0.0	0.0	0.0	0.0	0.0	0.0	0.0
0.0	0.0	0.0	0.0	0.0	0.0	0.0	0.0
0.0	0.0	0.0	0.0	0.0	0.0	0.0	0.0
0.0	0.0	0.0	0.0	0.0	0.0	0.0	0.0
0.0	0.0	0.0	0.0	0.0	0.0	0.0	0.0
0.0	0.0	0.0	0.0	0.0	0.0	0.0	0.0
0.0	0.0	0.0	0.0	0.0	0.0	0.0	0.0
0.0	0.0	0.0	0.0	0.0	0.0	0.0	0.0
0.0	0.0	0.0	0.0	0.0	0.0	0.0	0.0
0.0	0.0	0.0	0.0	0.0	0.0	0.0	0.0
0.0	0.0	0.0	0.0	0.0	0.0	0.0	0.0
0.0	0.0	0.0	0.0	0.0	0.0	0.0	0.0
0.0	0.0	0.0	0.0	0.0	0.0	0.0	0.0
0.0	0.0	0.0	0.0	0.0	0.0	0.0	0.0
0.0	0.0	0.0	0.0	0.0	0.0	0.0	0.0
0.0	0.0	0.0	0.0	0.0	0.0	0.0	0.0
0.0	0.0	0.0	0.0	0.0	0.0	0.0	0.0
0.0	0.0	0.0	0.0	0.0	0.0	0.0	0.0
0.0	0.0	0.0	0.0	0.0	0.0	0.0	0.0
0.0	0.0	0.0	0.0	0.0	0.0	0.0	0.0
0.0	0.0	0.0	0.0	0.0	0.0	0.0	0.0

0.0	0.0	0.0	0.0	0.0	0.0	0.0	0.0
0.0	0.0	0.0	0.0	0.0	0.0	0.0	0.0
0.0	0.0	0.0	0.0	0.0	0.0	0.0	0.0
0.0	0.0	0.0	0.0	0.0	0.0	0.0	0.0
0.0	0.0	0.0	0.0	0.0	0.0	0.0	0.0
0.0	0.0	0.0	0.0	0.0	0.0	0.0	0.0
0.0	0.0	0.0	0.0	0.0	0.0	0.0	0.0
0.0	0.0	0.0	0.0	0.0	0.0	0.0	0.0
0.0	0.0	0.0	0.0	0.0	0.0	0.0	0.0
0.0	0.0	0.0	0.0	0.0	0.0	0.0	0.0
0.0	0.0	0.0	0.0	0.0	0.0	0.0	0.0
0.0	0.0	0.0	0.0	0.0	0.0	0.0	0.0
0.0	0.0	0.0	0.0	0.0	0.0	0.0	0.0
0.0	0.0	0.0	0.0	0.0	0.0	0.0	0.0
0.0	0.0	0.0	0.0	0.0	0.0	0.0	0.0
0.0	0.0	0.0	0.0	0.0	0.0	0.0	0.0
0.0	0.0	0.0	0.0	0.0	0.0	0.0	0.0
0.0	0.0	0.0	0.0	0.0	0.0	0.0	0.0
0.0	0.0	0.0	0.0	0.0	0.0	0.0	0.0
0.0	0.0	0.0	0.0	0.0	0.0	0.0	0.0
0.0	0.0	0.0	0.0	0.0	0.0	0.0	0.0
0.0	0.0	0.0	0.0	0.0	0.0	0.0	0.0
0.0	0.0	0.0	0.0	0.0	0.0	0.0	0.0
0.0	0.0	0.0	0.0	0.0	0.0	0.0	0.0
0.0	0.0	0.0	0.0	0.0	0.0	0.0	0.0
0.0	0.0	0.0	0.0	0.0	0.0	0.0	0.0
0.0	0.0	0.0	0.0	0.0	0.0	0.0	0.0
0.0	0.0	0.0	0.0	0.0	0.0	0.0	0.0
0.0	0.0	0.0	0.0	0.0	0.0	0.0	0.0
0.0	0.0	0.0	0.0	0.0	0.0	0.0	0.0
0.0	0.0	0.0	0.0	0.0	0.0	0.0	0.0
0.0	0.0	0.0	0.0	0.0	0.0	0.0	0.0
0.0	0.0	0.0	0.0	0.0	0.0	0.0	0.0
0.0	0.0	0.0	0.0	0.0	0.0	0.0	0.0
0.0	0.0	0.0	0.0	0.0	0.0	0.0	0.0
0.0	0.0	0.0	0.0	0.0	0.0	0.0	0.0
0.0	0.0	0.0	0.0	0.0	0.0	0.0	0.0
0.0	0.0	0.0	0.0	0.0	0.0	0.0	0.0
0.0	0.0	0.0	0.0	0.0	0.0	0.0	0.0
0.0	0.0	0.0	0.0	0.0	0.0	0.0	0.0
0.0	0.0	0.0	0.0	0.0	0.0	0.0	0.0
0.0	0.0	0.0	0.0	0.0	0.0	0.0	0.0
0.0	0.0	0.0	0.0	0.0	0.0	0.0	0.0
0.0	0.0	0.0	0.0	0.0	0.0	0.0	0.0
0.0	0.0	0.0	0.0	0.0	0.0	0.0	0.0
0.0	0.0	0.0	0.0	0.0	0.0	0.0	0.0
0.0	0.0	0.0	0.0	0.0	0.0	0.0	0.0

0.0	0.0	0.0	0.0	0.0	0.0	0.0	0.0
0.0	0.0	0.0	0.0	0.0	0.0	0.0	0.0
0.0	0.0	0.0	0.0	0.0	0.0	0.0	0.0
0.0	0.0	0.0	0.0	0.0	0.0	0.0	0.0
0.0	0.0	0.0	0.0	0.0	0.0	0.0	0.0
0.0	0.0	0.0	0.0	0.0	0.0	0.0	0.0
0.0	0.0	0.0	0.0	0.0	0.0	0.0	0.0
0.0	0.0	0.0	0.0	0.0	0.0	0.0	0.0
0.0	0.0	0.0	0.0	0.0	0.0	0.0	0.0
0.0	0.0	0.0	0.0	0.0	0.0	0.0	0.0
0.0	0.0	0.0	0.0	0.0	0.0	0.0	0.0
0.0	0.0	0.0	0.0	0.0	0.0	0.0	0.0
0.0	0.0	0.0	0.0	0.0	0.0	0.0	0.0
0.0	0.0	0.0	0.0	0.0	0.0	0.0	0.0
0.0	0.0	0.0	0.0	0.0	0.0	0.0	0.0
0.0	0.0	0.0	0.0	0.0	0.0	0.0	0.0
0.0	0.0	0.0	0.0	0.0	0.0	0.0	0.0
0.0	0.0	0.0	0.0	0.0	0.0	0.0	0.0
0.0	0.0	0.0	0.0	0.0	0.0	0.0	0.0
0.0	0.0	0.0	0.0	0.0	0.0	0.0	0.0
0.0	0.0	0.0	0.0	0.0	0.0	0.0	0.0
0.0	0.0	0.0	0.0	0.0	0.0	0.0	0.0
0.0	0.0	0.0	0.0	0.0	0.0	0.0	0.0
0.0	0.0	0.0	0.0	0.0	0.0	0.0	0.0
0.0	0.0	0.0	0.0	0.0	0.0	0.0	0.0
0.0	0.0	0.0	0.0	0.0	0.0	0.0	0.0
0.0	0.0	0.0	0.0	0.0	0.0	0.0	0.0
0.0	0.0	0.0	0.0	0.0	0.0	0.0	0.0
0.0	0.0	0.0	0.0	0.0	0.0	0.0	0.0
0.0	0.0	0.0	0.0	0.0	0.0	0.0	0.0
0.0	0.0	0.0	0.0	0.0	0.0	0.0	0.0
0.0	0.0	0.0	0.0	0.0	0.0	0.0	0.0
0.0	0.0	0.0	0.0	0.0	0.0	0.0	0.0
0.0	0.0	0.0	0.0	0.0	0.0	0.0	0.0
0.0	0.0	0.0	0.0	0.0	0.0	0.0	0.0
0.0	0.0	0.0	0.0	0.0	0.0	0.0	0.0
0.0	0.0	0.0	0.0	0.0	0.0	0.0	0.0
0.0	0.0	0.0	0.0	0.0	0.0	0.0	0.0
0.0	0.0	0.0	0.0	0.0	0.0	0.0	0.0
0.0	0.0	0.0	0.0	0.0	0.0	0.0	0.0
0.0	0.0	0.0	0.0	0.0	0.0	0.0	0.0
0.0	0.0	0.0	0.0	0.0	0.0	0.0	0.0
0.0	0.0	0.0	0.0	0.0	0.0	0.0	0.0
0.0	0.0	0.0	0.0	0.0	0.0	0.0	0.0
0.0	0.0	0.0	0.0	0.0	0.0	0.0	0.0
0.0	0.0	0.0	0.0	0.0	0.0	0.0	0.0
0.0	0.0	0.0	0.0	0.0	0.0	0.0	0.0

0.0	0.0	0.0	0.0	0.0	0.0	0.0	0.0
0.0	0.0	0.0	0.0	0.0	0.0	0.0	0.0
0.0	0.0	0.0	0.0	0.0	0.0	0.0	0.0
0.0	0.0	0.0	0.0	0.0	0.0	0.0	0.0
0.0	0.0	0.0	0.0	0.0	0.0	0.0	0.0
0.0	0.0	0.0	0.0	0.0	0.0	0.0	0.0
0.0	0.0	0.0	0.0	0.0	0.0	0.0	0.0
0.0	0.0	0.0	0.0	0.0	0.0	0.0	0.0
0.0	0.0	0.0	0.0	0.0	0.0	0.0	0.0
0.0	0.0	0.0	0.0	0.0	0.0	0.0	0.0
0.0	0.0	0.0	0.0	0.0	0.0	0.0	0.0
0.0	0.0	0.0	0.0	0.0	0.0	0.0	0.0
0.0	0.0	0.0	0.0	0.0	0.0	0.0	0.0
0.0	0.0	0.0	0.0	0.0	0.0	0.0	0.0
0.0	0.0	0.0	0.0	0.0	0.0	0.0	0.0
0.0	0.0	0.0	0.0	0.0	0.0	0.0	0.0
0.0	0.0	0.0	0.0	0.0	0.0	0.0	0.0
0.0	0.0	0.0	0.0	0.0	0.0	0.0	0.0
0.0	0.0	0.0	0.0	0.0	0.0	0.0	0.0
0.0	0.0	0.0	0.0	0.0	0.0	0.0	0.0
0.0	0.0	0.0	0.0	0.0	0.0	0.0	0.0
0.0	0.0	0.0	0.0	0.0	0.0	0.0	0.0
0.0	0.0	0.0	0.0	0.0	0.0	0.0	0.0
0.0	0.0	0.0	0.0	0.0	0.0	0.0	0.0
0.0	0.0	0.0	0.0	0.0	0.0	0.0	0.0
0.0	0.0	0.0	0.0	0.0	0.0	0.0	0.0
0.0	0.0	0.0	0.0	0.0	0.0	0.0	0.0
0.0	0.0	0.0	0.0	0.0	0.0	0.0	0.0
0.0	0.0	0.0	0.0	0.0	0.0	0.0	0.0
0.0	0.0	0.0	0.0	0.0	0.0	0.0	0.0
0.0	0.0	0.0	0.0	0.0	0.0	0.0	0.0
0.0	0.0	0.0	0.0	0.0	0.0	0.0	0.0
0.0	0.0	0.0	0.0	0.0	0.0	0.0	0.0
0.0	0.0	0.0	0.0	0.0	0.0	0.0	0.0
0.0	0.0	0.0	0.0	0.0	0.0	0.0	0.0
0.0	0.0	0.0	0.0	0.0	0.0	0.0	0.0
0.0	0.0	0.0	0.0	0.0	0.0	0.0	0.0
0.0	0.0	0.0	0.0	0.0	0.0	0.0	0.0
0.0	0.0	0.0	0.0	0.0	0.0	0.0	0.0
0.0	0.0	0.0	0.0	0.0	0.0	0.0	0.0
0.0	0.0	0.0	0.0	0.0	0.0	0.0	0.0
0.0	0.0	0.0	0.0	0.0	0.0	0.0	0.0
0.0	0.0	0.0	0.0	0.0	0.0	0.0	0.0
0.0	0.0	0.0	0.0	0.0	0.0	0.0	0.0
0.0	0.0	0.0	0.0	0.0	0.0	0.0	0.0
0.0	0.0	0.0	0.0	0.0	0.0	0.0	0.0
0.0	0.0	0.0	0.0	0.0	0.0	0.0	0.0
0.0	0.0	0.0	0.0	0.0	0.0	0.0	0.0

0.0	0.0	0.0	0.0	0.0	0.0	0.0	0.0
0.0	0.0	0.0	0.0	0.0	0.0	0.0	0.0
0.0	0.0	0.0	0.0	0.0	0.0	0.0	0.0
0.0	0.0	0.0	0.0	0.0	0.0	0.0	0.0
0.0	0.0	0.0	0.0	0.0	0.0	0.0	0.0
0.0	0.0	0.0	0.0	0.0	0.0	0.0	0.0
0.0	0.0	0.0	0.0	0.0	0.0	0.0	0.0
0.0	0.0	0.0	0.0	0.0	0.0	0.0	0.0
0.0	0.0	0.0	0.0	0.0	0.0	0.0	0.0
0.0	0.0	0.0	0.0	0.0	0.0	0.0	0.0
0.0	0.0	0.0	0.0	0.0	0.0	0.0	0.0
0.0	0.0	0.0	0.0	0.0	0.0	0.0	0.0
0.0	0.0	0.0	0.0	0.0	0.0	0.0	0.0
0.0	0.0	0.0	0.0	0.0	0.0	0.0	0.0
0.0	0.0	0.0	0.0	0.0	0.0	0.0	0.0
0.0	0.0	0.0	0.0	0.0	0.0	0.0	0.0
0.0	0.0	0.0	0.0	0.0	0.0	0.0	0.0
0.0	0.0	0.0	0.0	0.0	0.0	0.0	0.0
0.0	0.0	0.0	0.0	0.0	0.0	0.0	0.0
0.0	0.0	0.0	0.0	0.0	0.0	0.0	0.0
0.0	0.0	0.0	0.0	0.0	0.0	0.0	0.0
0.0	0.0	0.0	0.0	0.0	0.0	0.0	0.0
0.0	0.0	0.0	0.0	0.0	0.0	0.0	0.0
0.0	0.0	0.0	0.0	0.0	0.0	0.0	0.0
0.0	0.0	0.0	0.0	0.0	0.0	0.0	0.0
0.0	0.0	0.0	0.0	0.0	0.0	0.0	0.0
0.0	0.0	0.0	0.0	0.0	0.0	0.0	0.0
0.0	0.0	0.0	0.0	0.0	0.0	0.0	0.0
0.0	0.0	0.0	0.0	0.0	0.0	0.0	0.0
0.0	0.0	0.0	0.0	0.0	0.0	0.0	0.0
0.0	0.0	0.0	0.0	0.0	0.0	0.0	0.0
0.0	0.0	0.0	0.0	0.0	0.0	0.0	0.0
0.0	0.0	0.0	0.0	0.0	0.0	0.0	0.0
0.0	0.0	0.0	0.0	0.0	0.0	0.0	0.0
0.0	0.0	0.0	0.0	0.0	0.0	0.0	0.0
0.0	0.0	0.0	0.0	0.0	0.0	0.0	0.0
0.0	0.0	0.0	0.0	0.0	0.0	0.0	0.0
0.0	0.0	0.0	0.0	0.0	0.0	0.0	0.0
0.0	0.0	0.0	0.0	0.0	0.0	0.0	0.0
0.0	0.0	0.0	0.0	0.0	0.0	0.0	0.0
0.0	0.0	0.0	0.0	0.0	0.0	0.0	0.0
0.0	0.0	0.0	0.0	0.0	0.0	0.0	0.0
0.0	0.0	0.0	0.0	0.0	0.0	0.0	0.0
0.0	0.0	0.0	0.0	0.0	0.0	0.0	0.0
0.0	0.0	0.0	0.0	0.0	0.0	0.0	0.0
0.0	0.0	0.0	0.0	0.0	0.0	0.0	0.0
0.0	0.0	0.0	0.0	0.0	0.0	0.0	0.0

0.0	0.0	0.0	0.0	0.0	0.0	0.0	0.0
0.0	0.0	0.0	0.0	0.0	0.0	0.0	0.0
0.0	0.0	0.0	0.0	0.0	0.0	0.0	0.0
0.0	0.0	0.0	0.0	0.0	0.0	0.0	0.0
0.0	0.0	0.0	0.0	0.0	0.0	0.0	0.0
0.0	0.0	0.0	0.0	0.0	0.0	0.0	0.0
0.0	0.0	0.0	0.0	0.0	0.0	0.0	0.0
0.0	0.0	0.0	0.0	0.0	0.0	0.0	0.0
0.0	0.0	0.0	0.0	0.0	0.0	0.0	0.0
0.0	0.0	0.0	0.0	0.0	0.0	0.0	0.0
0.0	0.0	0.0	0.0	0.0	0.0	0.0	0.0
0.0	0.0	0.0	0.0	0.0	0.0	0.0	0.0
0.0	0.0	0.0	0.0	0.0	0.0	0.0	0.0
0.0	0.0	0.0	0.0	0.0	0.0	0.0	0.0
0.0	0.0	0.0	0.0	0.0	0.0	0.0	0.0
0.0	0.0	0.0	0.0	0.0	0.0	0.0	0.0
0.0	0.0	0.0	0.0	0.0	0.0	0.0	0.0
0.0	0.0	0.0	0.0	0.0	0.0	0.0	0.0
0.0	0.0	0.0	0.0	0.0	0.0	0.0	0.0
0.0	0.0	0.0	0.0	0.0	0.0	0.0	0.0
0.0	0.0	0.0	0.0	0.0	0.0	0.0	0.0
0.0	0.0	0.0	0.0	0.0	0.0	0.0	0.0
0.0	0.0	0.0	0.0	0.0	0.0	0.0	0.0
0.0	0.0	0.0	0.0	0.0	0.0	0.0	0.0
0.0	0.0	0.0	0.0	0.0	0.0	0.0	0.0
0.0	0.0	0.0	0.0	0.0	0.0	0.0	0.0
0.0	0.0	0.0	0.0	0.0	0.0	0.0	0.0
0.0	0.0	0.0	0.0	0.0	0.0	0.0	0.0
0.0	0.0	0.0	0.0	0.0	0.0	0.0	0.0
0.0	0.0	0.0	0.0	0.0	0.0	0.0	0.0
0.0	0.0	0.0	0.0	0.0	0.0	0.0	0.0
0.0	0.0	0.0	0.0	0.0	0.0	0.0	0.0
0.0	0.0	0.0	0.0	0.0	0.0	0.0	0.0
0.0	0.0	0.0	0.0	0.0	0.0	0.0	0.0
0.0	0.0	0.0	0.0	0.0	0.0	0.0	0.0
0.0	0.0	0.0	0.0	0.0	0.0	0.0	0.0
0.0	0.0	0.0	0.0	0.0	0.0	0.0	0.0
0.0	0.0	0.0	0.0	0.0	0.0	0.0	0.0
0.0	0.0	0.0	0.0	0.0	0.0	0.0	0.0
0.0	0.0	0.0	0.0	0.0	0.0	0.0	0.0
0.0	0.0	0.0	0.0	0.0	0.0	0.0	0.0
0.0	0.0	0.0	0.0	0.0	0.0	0.0	0.0
0.0	0.0	0.0	0.0	0.0	0.0	0.0	0.0
0.0	0.0	0.0	0.0	0.0	0.0	0.0	0.0
0.0	0.0	0.0	0.0	0.0	0.0	0.0	0.0
0.0	0.0	0.0	0.0	0.0	0.0	0.0	0.0
0.0	0.0	0.0	0.0	0.0	0.0	0.0	0.0

0.0	0.0	0.0	0.0	0.0	0.0	0.0	0.0
0.0	0.0	0.0	0.0	0.0	0.0	0.0	0.0
0.0	0.0	0.0	0.0	0.0	0.0	0.0	0.0
0.0	0.0	0.0	0.0	0.0	0.0	0.0	0.0
0.0	0.0	0.0	0.0	0.0	0.0	0.0	0.0
0.0	0.0	0.0	0.0	0.0	0.0	0.0	0.0
0.0	0.0	0.0	0.0	0.0	0.0	0.0	0.0
0.0	0.0	0.0	0.0	0.0	0.0	0.0	0.0
0.0	0.0	0.0	0.0	0.0	0.0	0.0	0.0
0.0	0.0	0.0	0.0	0.0	0.0	0.0	0.0
0.0	0.0	0.0	0.0	0.0	0.0	0.0	0.0
0.0	0.0	0.0	0.0	0.0	0.0	0.0	0.0
0.0	0.0	0.0	0.0	0.0	0.0	0.0	0.0
0.0	0.0	0.0	0.0	0.0	0.0	0.0	0.0
0.0	0.0	0.0	0.0	0.0	0.0	0.0	0.0
0.0	0.0	0.0	0.0	0.0	0.0	0.0	0.0
0.0	0.0	0.0	0.0	0.0	0.0	0.0	0.0
0.0	0.0	0.0	0.0	0.0	0.0	0.0	0.0
0.0	0.0	0.0	0.0	0.0	0.0	0.0	0.0
0.0	0.0	0.0	0.0	0.0	0.0	0.0	0.0
0.0	0.0	0.0	0.0	0.0	0.0	0.0	0.0
0.0	0.0	0.0	0.0	0.0	0.0	0.0	0.0
0.0	0.0	0.0	0.0	0.0	0.0	0.0	0.0
0.0	0.0	0.0	0.0	0.0	0.0	0.0	0.0
0.0	0.0	0.0	0.0	0.0	0.0	0.0	0.0
0.0	0.0	0.0	0.0	0.0	0.0	0.0	0.0
0.0	0.0	0.0	0.0	0.0	0.0	0.0	0.0
0.0	0.0	0.0	0.0	0.0	0.0	0.0	0.0
0.0	0.0	0.0	0.0	0.0	0.0	0.0	0.0
0.0	0.0	0.0	0.0	0.0	0.0	0.0	0.0
0.0	0.0	0.0	0.0	0.0	0.0	0.0	0.0
0.0	0.0	0.0	0.0	0.0	0.0	0.0	0.0
0.0	0.0	0.0	0.0	0.0	0.0	0.0	0.0
0.0	0.0	0.0	0.0	0.0	0.0	0.0	0.0
0.0	0.0	0.0	0.0	0.0	0.0	0.0	0.0
0.0	0.0	0.0	0.0	0.0	0.0	0.0	0.0
0.0	0.0	0.0	0.0	0.0	0.0	0.0	0.0
0.0	0.0	0.0	0.0	0.0	0.0	0.0	0.0
0.0	0.0	0.0	0.0	0.0	0.0	0.0	0.0
0.0	0.0	0.0	0.0	0.0	0.0	0.0	0.0
0.0	0.0	0.0	0.0	0.0	0.0	0.0	0.0
0.0	0.0	0.0	0.0	0.0	0.0	0.0	0.0
0.0	0.0	0.0	0.0	0.0	0.0	0.0	0.0
0.0	0.0	0.0	0.0	0.0	0.0	0.0	0.0
0.0	0.0	0.0	0.0	0.0	0.0	0.0	0.0
0.0	0.0	0.0	0.0	0.0	0.0	0.0	0.0
0.0	0.0	0.0	0.0	0.0	0.0	0.0	0.0
0.0	0.0	0.0	0.0	0.0	0.0	0.0	0.0

0.0	0.0	0.0	0.0	0.0	0.0	0.0	0.0
0.0	0.0	0.0	0.0	0.0	0.0	0.0	0.0
0.0	0.0	0.0	0.0	0.0	0.0	0.0	0.0
0.0	0.0	0.0	0.0	0.0	0.0	0.0	0.0
0.0	0.0	0.0	0.0	0.0	0.0	0.0	0.0
0.0	0.0	0.0	0.0	0.0	0.0	0.0	0.0
0.0	0.0	0.0	0.0	0.0	0.0	0.0	0.0
0.0	0.0	0.0	0.0	0.0	0.0	0.0	0.0
0.0	0.0	0.0	0.0	0.0	0.0	0.0	0.0
0.0	0.0	0.0	0.0	0.0	0.0	0.0	0.0
0.0	0.0	0.0	0.0	0.0	0.0	0.0	0.0
0.0	0.0	0.0	0.0	0.0	0.0	0.0	0.0
0.0	0.0	0.0	0.0	0.0	0.0	0.0	0.0
0.0	0.0	0.0	0.0	0.0	0.0	0.0	0.0
0.0	0.0	0.0	0.0	0.0	0.0	0.0	0.0
0.0	0.0	0.0	0.0	0.0	0.0	0.0	0.0
0.0	0.0	0.0	0.0	0.0	0.0	0.0	0.0
0.0	0.0	0.0	0.0	0.0	0.0	0.0	0.0
0.0	0.0	0.0	0.0	0.0	0.0	0.0	0.0
0.0	0.0	0.0	0.0	0.0	0.0	0.0	0.0
0.0	0.0	0.0	0.0	0.0	0.0	0.0	0.0
0.0	0.0	0.0	0.0	0.0	0.0	0.0	0.0
0.0	0.0	0.0	0.0	0.0	0.0	0.0	0.0
0.0	0.0	0.0	0.0	0.0	0.0	0.0	0.0
0.0	0.0	0.0	0.0	0.0	0.0	0.0	0.0
0.0	0.0	0.0	0.0	0.0	0.0	0.0	0.0
0.0	0.0	0.0	0.0	0.0	0.0	0.0	0.0
0.0	0.0	0.0	0.0	0.0	0.0	0.0	0.0
0.0	0.0	0.0	0.0	0.0	0.0	0.0	0.0
0.0	0.0	0.0	0.0	0.0	0.0	0.0	0.0
0.0	0.0	0.0	0.0	0.0	0.0	0.0	0.0
0.0	0.0	0.0	0.0	0.0	0.0	0.0	0.0
0.0	0.0	0.0	0.0	0.0	0.0	0.0	0.0
0.0	0.0	0.0	0.0	0.0	0.0	0.0	0.0
0.0	0.0	0.0	0.0	0.0	0.0	0.0	0.0
0.0	0.0	0.0	0.0	0.0	0.0	0.0	0.0
0.0	0.0	0.0	0.0	0.0	0.0	0.0	0.0
0.0	0.0	0.0	0.0	0.0	0.0	0.0	0.0
0.0	0.0	0.0	0.0	0.0	0.0	0.0	0.0
0.0	0.0	0.0	0.0	0.0	0.0	0.0	0.0
0.0	0.0	0.0	0.0	0.0	0.0	0.0	0.0
0.0	0.0	0.0	0.0	0.0	0.0	0.0	0.0
0.0	0.0	0.0	0.0	0.0	0.0	0.0	0.0
0.0	0.0	0.0	0.0	0.0	0.0	0.0	0.0
0.0	0.0	0.0	0.0	0.0	0.0	0.0	0.0
0.0	0.0	0.0	0.0	0.0	0.0	0.0	0.0
0.0	0.0	0.0	0.0	0.0	0.0	0.0	0.0

0.0	0.0	0.0	0.0	0.0	0.0	0.0	0.0
0.0	0.0	0.0	0.0	0.0	0.0	0.0	0.0
0.0	0.0	0.0	0.0	0.0	0.0	0.0	0.0
0.0	0.0	0.0	0.0	0.0	0.0	0.0	0.0
0.0	0.0	0.0	0.0	0.0	0.0	0.0	0.0
0.0	0.0	0.0	0.0	0.0	0.0	0.0	0.0
0.0	0.0	0.0	0.0	0.0	0.0	0.0	0.0
0.0	0.0	0.0	0.0	0.0	0.0	0.0	0.0
0.0	0.0	0.0	0.0	0.0	0.0	0.0	0.0
0.0	0.0	0.0	0.0	0.0	0.0	0.0	0.0
0.0	0.0	0.0	0.0	0.0	0.0	0.0	0.0
0.0	0.0	0.0	0.0	0.0	0.0	0.0	0.0
0.0	0.0	0.0	0.0	0.0	0.0	0.0	0.0
0.0	0.0	0.0	0.0	0.0	0.0	0.0	0.0
0.0	0.0	0.0	0.0	0.0	0.0	0.0	0.0
0.0	0.0	0.0	0.0	0.0	0.0	0.0	0.0
0.0	0.0	0.0	0.0	0.0	0.0	0.0	0.0
0.0	0.0	0.0	0.0	0.0	0.0	0.0	0.0
0.0	0.0	0.0	0.0	0.0	0.0	0.0	0.0
0.0	0.0	0.0	0.0	0.0	0.0	0.0	0.0
0.0	0.0	0.0	0.0	0.0	0.0	0.0	0.0
0.0	0.0	0.0	0.0	0.0	0.0	0.0	0.0
0.0	0.0	0.0	0.0	0.0	0.0	0.0	0.0
0.0	0.0	0.0	0.0	0.0	0.0	0.0	0.0
0.0	0.0	0.0	0.0	0.0	0.0	0.0	0.0
0.0	0.0	0.0	0.0	0.0	0.0	0.0	0.0
0.0	0.0	0.0	0.0	0.0	0.0	0.0	0.0
0.0	0.0	0.0	0.0	0.0	0.0	0.0	0.0
0.0	0.0	0.0	0.0	0.0	0.0	0.0	0.0
0.0	0.0	0.0	0.0	0.0	0.0	0.0	0.0
0.0	0.0	0.0	0.0	0.0	0.0	0.0	0.0
0.0	0.0	0.0	0.0	0.0	0.0	0.0	0.0
0.0	0.0	0.0	0.0	0.0	0.0	0.0	0.0
0.0	0.0	0.0	0.0	0.0	0.0	0.0	0.0
0.0	0.0	0.0	0.0	0.0	0.0	0.0	0.0
0.0	0.0	0.0	0.0	0.0	0.0	0.0	0.0
0.0	0.0	0.0	0.0	0.0	0.0	0.0	0.0
0.0	0.0	0.0	0.0	0.0	0.0	0.0	0.0
0.0	0.0	0.0	0.0	0.0	0.0	0.0	0.0
0.0	0.0	0.0	0.0	0.0	0.0	0.0	0.0
0.0	0.0	0.0	0.0	0.0	0.0	0.0	0.0
0.0	0.0	0.0	0.0	0.0	0.0	0.0	0.0
0.0	0.0	0.0	0.0	0.0	0.0	0.0	0.0
0.0	0.0	0.0	0.0	0.0	0.0	0.0	0.0
0.0	0.0	0.0	0.0	0.0	0.0	0.0	0.0
0.0	0.0	0.0	0.0	0.0	0.0	0.0	0.0
0.0	0.0	0.0	0.0	0.0	0.0	0.0	0.0
0.0	0.0	0.0	0.0	0.0	0.0	0.0	0.0

0.0	0.0	0.0	0.0	0.0	0.0	0.0	0.0
0.0	0.0	0.0	0.0	0.0	0.0	0.0	0.0
0.0	0.0	0.0	0.0	0.0	0.0	0.0	0.0
0.0	0.0	0.0	0.0	0.0	0.0	0.0	0.0
0.0	0.0	0.0	0.0	0.0	0.0	0.0	0.0
0.0	0.0	0.0	0.0	0.0	0.0	0.0	0.0
0.0	0.0	0.0	0.0	0.0	0.0	0.0	0.0
0.0	0.0	0.0	0.0	0.0	0.0	0.0	0.0
0.0	0.0	0.0	0.0	0.0	0.0	0.0	0.0
0.0	0.0	0.0	0.0	0.0	0.0	0.0	0.0
0.0	0.0	0.0	0.0	0.0	0.0	0.0	0.0
0.0	0.0	0.0	0.0	0.0	0.0	0.0	0.0
0.0	0.0	0.0	0.0	0.0	0.0	0.0	0.0
0.0	0.0	0.0	0.0	0.0	0.0	0.0	0.0
0.0	0.0	0.0	0.0	0.0	0.0	0.0	0.0
0.0	0.0	0.0	0.0	0.0	0.0	0.0	0.0
0.0	0.0	0.0	0.0	0.0	0.0	0.0	0.0
0.0	0.0	0.0	0.0	0.0	0.0	0.0	0.0
0.0	0.0	0.0	0.0	0.0	0.0	0.0	0.0
0.0	0.0	0.0	0.0	0.0	0.0	0.0	0.0
0.0	0.0	0.0	0.0	0.0	0.0	0.0	0.0
0.0	0.0	0.0	0.0	0.0	0.0	0.0	0.0
0.0	0.0	0.0	0.0	0.0	0.0	0.0	0.0
0.0	0.0	0.0	0.0	0.0	0.0	0.0	0.0
0.0	0.0	0.0	0.0	0.0	0.0	0.0	0.0
0.0	0.0	0.0	0.0	0.0	0.0	0.0	0.0
0.0	0.0	0.0	0.0	0.0	0.0	0.0	0.0
0.0	0.0	0.0	0.0	0.0	0.0	0.0	0.0
0.0	0.0	0.0	0.0	0.0	0.0	0.0	0.0
0.0	0.0	0.0	0.0	0.0	0.0	0.0	0.0
0.0	0.0	0.0	0.0	0.0	0.0	0.0	0.0
0.0	0.0	0.0	0.0	0.0	0.0	0.0	0.0
0.0	0.0	0.0	0.0	0.0	0.0	0.0	0.0
0.0	0.0	0.0	0.0	0.0	0.0	0.0	0.0
0.0	0.0	0.0	0.0	0.0	0.0	0.0	0.0
0.0	0.0	0.0	0.0	0.0	0.0	0.0	0.0
0.0	0.0	0.0	0.0	0.0	0.0	0.0	0.0
0.0	0.0	0.0	0.0	0.0	0.0	0.0	0.0
0.0	0.0	0.0	0.0	0.0	0.0	0.0	0.0
0.0	0.0	0.0	0.0	0.0	0.0	0.0	0.0
0.0	0.0	0.0	0.0	0.0	0.0	0.0	0.0
0.0	0.0	0.0	0.0	0.0	0.0	0.0	0.0
0.0	0.0	0.0	0.0	0.0	0.0	0.0	0.0
0.0	0.0	0.0	0.0	0.0	0.0	0.0	0.0
0.0	0.0	0.0	0.0	0.0	0.0	0.0	0.0
0.0	0.0	0.0	0.0	0.0	0.0	0.0	0.0
0.0	0.0	0.0	0.0	0.0	0.0	0.0	0.0
0.0	0.0	0.0	0.0	0.0	0.0	0.0	0.0

0.0	0.0	0.0	0.0	0.0	0.0	0.0	0.0
0.0	0.0	0.0	0.0	0.0	0.0	0.0	0.0
0.0	0.0	0.0	0.0	0.0	0.0	0.0	0.0
0.0	0.0	0.0	0.0	0.0	0.0	0.0	0.0
0.0	0.0	0.0	0.0	0.0	0.0	0.0	0.0
0.0	0.0	0.0	0.0	0.0	0.0	0.0	0.0
0.0	0.0	0.0	0.0	0.0	0.0	0.0	0.0
0.0	0.0	0.0	0.0	0.0	0.0	0.0	0.0
0.0	0.0	0.0	0.0	0.0	0.0	0.0	0.0
0.0	0.0	0.0	0.0	0.0	0.0	0.0	0.0
0.0	0.0	0.0	0.0	0.0	0.0	0.0	0.0
0.0	0.0	0.0	0.0	0.0	0.0	0.0	0.0
0.0	0.0	0.0	0.0	0.0	0.0	0.0	0.0
0.0	0.0	0.0	0.0	0.0	0.0	0.0	0.0
0.0	0.0	0.0	0.0	0.0	0.0	0.0	0.0
0.0	0.0	0.0	0.0	0.0	0.0	0.0	0.0
0.0	0.0	0.0	0.0	0.0	0.0	0.0	0.0
0.0	0.0	0.0	0.0	0.0	0.0	0.0	0.0
0.0	0.0	0.0	0.0	0.0	0.0	0.0	0.0
0.0	0.0	0.0	0.0	0.0	0.0	0.0	0.0
0.0	0.0	0.0	0.0	0.0	0.0	0.0	0.0
0.0	0.0	0.0	0.0	0.0	0.0	0.0	0.0
0.0	0.0	0.0	0.0	0.0	0.0	0.0	0.0
0.0	0.0	0.0	0.0	0.0	0.0	0.0	0.0
0.0	0.0	0.0	0.0	0.0	0.0	0.0	0.0
0.0	0.0	0.0	0.0	0.0	0.0	0.0	0.0
0.0	0.0	0.0	0.0	0.0	0.0	0.0	0.0
0.0	0.0	0.0	0.0	0.0	0.0	0.0	0.0
0.0	0.0	0.0	0.0	0.0	0.0	0.0	0.0
0.0	0.0	0.0	0.0	0.0	0.0	0.0	0.0
0.0	0.0	0.0	0.0	0.0	0.0	0.0	0.0
0.0	0.0	0.0	0.0	0.0	0.0	0.0	0.0
0.0	0.0	0.0	0.0	0.0	0.0	0.0	0.0
0.0	0.0	0.0	0.0	0.0	0.0	0.0	0.0
0.0	0.0	0.0	0.0	0.0	0.0	0.0	0.0
0.0	0.0	0.0	0.0	0.0	0.0	0.0	0.0
0.0	0.0	0.0	0.0	0.0	0.0	0.0	0.0
0.0	0.0	0.0	0.0	0.0	0.0	0.0	0.0
0.0	0.0	0.0	0.0	0.0	0.0	0.0	0.0
0.0	0.0	0.0	0.0	0.0	0.0	0.0	0.0
0.0	0.0	0.0	0.0	0.0	0.0	0.0	0.0
0.0	0.0	0.0	0.0	0.0	0.0	0.0	0.0
0.0	0.0	0.0	0.0	0.0	0.0	0.0	0.0
0.0	0.0	0.0	0.0	0.0	0.0	0.0	0.0
0.0	0.0	0.0	0.0	0.0	0.0	0.0	0.0
0.0	0.0	0.0	0.0	0.0	0.0	0.0	0.0
0.0	0.0	0.0	0.0	0.0	0.0	0.0	0.0
0.0	0.0	0.0	0.0	0.0	0.0	0.0	0.0

0.0	0.0	0.0	0.0	0.0	0.0	0.0	0.0
0.0	0.0	0.0	0.0	0.0	0.0	0.0	0.0
0.0	0.0	0.0	0.0	0.0	0.0	0.0	0.0
0.0	0.0	0.0	0.0	0.0	0.0	0.0	0.0
0.0	0.0	0.0	0.0	0.0	0.0	0.0	0.0
0.0	0.0	0.0	0.0	0.0	0.0	0.0	0.0
0.0	0.0	0.0	0.0	0.0	0.0	0.0	0.0
0.0	0.0	0.0	0.0	0.0	0.0	0.0	0.0
0.0	0.0	0.0	0.0	0.0	0.0	0.0	0.0
0.0	0.0	0.0	0.0	0.0	0.0	0.0	0.0
0.0	0.0	0.0	0.0	0.0	0.0	0.0	0.0
0.0	0.0	0.0	0.0	0.0	0.0	0.0	0.0
0.0	0.0	0.0	0.0	0.0	0.0	0.0	0.0
0.0	0.0	0.0	0.0	0.0	0.0	0.0	0.0
0.0	0.0	0.0	0.0	0.0	0.0	0.0	0.0
0.0	0.0	0.0	0.0	0.0	0.0	0.0	0.0
0.0	0.0	0.0	0.0	0.0	0.0	0.0	0.0
0.0	0.0	0.0	0.0	0.0	0.0	0.0	0.0
0.0	0.0	0.0	0.0	0.0	0.0	0.0	0.0
0.0	0.0	0.0	0.0	0.0	0.0	0.0	0.0
0.0	0.0	0.0	0.0	0.0	0.0	0.0	0.0
0.0	0.0	0.0	0.0	0.0	0.0	0.0	0.0
0.0	0.0	0.0	0.0	0.0	0.0	0.0	0.0
0.0	0.0	0.0	0.0	0.0	0.0	0.0	0.0
0.0	0.0	0.0	0.0	0.0	0.0	0.0	0.0
0.0	0.0	0.0	0.0	0.0	0.0	0.0	0.0
0.0	0.0	0.0	0.0	0.0	0.0	0.0	0.0
0.0	0.0	0.0	0.0	0.0	0.0	0.0	0.0
0.0	0.0	0.0	0.0	0.0	0.0	0.0	0.0
0.0	0.0	0.0	0.0	0.0	0.0	0.0	0.0
0.0	0.0	0.0	0.0	0.0	0.0	0.0	0.0
0.0	0.0	0.0	0.0	0.0	0.0	0.0	0.0
0.0	0.0	0.0	0.0	0.0	0.0	0.0	0.0
0.0	0.0	0.0	0.0	0.0	0.0	0.0	0.0
0.0	0.0	0.0	0.0	0.0	0.0	0.0	0.0
0.0	0.0	0.0	0.0	0.0	0.0	0.0	0.0
0.0	0.0	0.0	0.0	0.0	0.0	0.0	0.0
0.0	0.0	0.0	0.0	0.0	0.0	0.0	0.0
0.0	0.0	0.0	0.0	0.0	0.0	0.0	0.0
0.0	0.0	0.0	0.0	0.0	0.0	0.0	0.0
0.0	0.0	0.0	0.0	0.0	0.0	0.0	0.0
0.0	0.0	0.0	0.0	0.0	0.0	0.0	0.0
0.0	0.0	0.0	0.0	0.0	0.0	0.0	0.0
0.0	0.0	0.0	0.0	0.0	0.0	0.0	0.0
0.0	0.0	0.0	0.0	0.0	0.0	0.0	0.0
0.0	0.0	0.0	0.0	0.0	0.0	0.0	0.0
0.0	0.0	0.0	0.0	0.0	0.0	0.0	0.0

0.0	0.0	0.0	0.0	0.0	0.0	0.0	0.0
0.0	0.0	0.0	0.0	0.0	0.0	0.0	0.0
0.0	0.0	0.0	0.0	0.0	0.0	0.0	0.0
0.0	0.0	0.0	0.0	0.0	0.0	0.0	0.0
0.0	0.0	0.0	0.0	0.0	0.0	0.0	0.0
0.0	0.0	0.0	0.0	0.0	0.0	0.0	0.0
0.0	0.0	0.0	0.0	0.0	0.0	0.0	0.0
0.0	0.0	0.0	0.0	0.0	0.0	0.0	0.0
0.0	0.0	0.0	0.0	0.0	0.0	0.0	0.0
0.0	0.0	0.0	0.0	0.0	0.0	0.0	0.0
0.0	0.0	0.0	0.0	0.0	0.0	0.0	0.0
0.0	0.0	0.0	0.0	0.0	0.0	0.0	0.0
0.0	0.0	0.0	0.0	0.0	0.0	0.0	0.0
0.0	0.0	0.0	0.0	0.0	0.0	0.0	0.0
0.0	0.0	0.0	0.0	0.0	0.0	0.0	0.0
0.0	0.0	0.0	0.0	0.0	0.0	0.0	0.0
0.0	0.0	0.0	0.0	0.0	0.0	0.0	0.0
0.0	0.0	0.0	0.0	0.0	0.0	0.0	0.0
0.0	0.0	0.0	0.0	0.0	0.0	0.0	0.0
0.0	0.0	0.0	0.0	0.0	0.0	0.0	0.0
0.0	0.0	0.0	0.0	0.0	0.0	0.0	0.0
0.0	0.0	0.0	0.0	0.0	0.0	0.0	0.0
0.0	0.0	0.0	0.0	0.0	0.0	0.0	0.0
0.0	0.0	0.0	0.0	0.0	0.0	0.0	0.0
0.0	0.0	0.0	0.0	0.0	0.0	0.0	0.0
0.0	0.0	0.0	0.0	0.0	0.0	0.0	0.0
0.0	0.0	0.0	0.0	0.0	0.0	0.0	0.0
0.0	0.0	0.0	0.0	0.0	0.0	0.0	0.0
0.0	0.0	0.0	0.0	0.0	0.0	0.0	0.0
0.0	0.0	0.0	0.0	0.0	0.0	0.0	0.0
0.0	0.0	0.0	0.0	0.0	0.0	0.0	0.0
0.0	0.0	0.0	0.0	0.0	0.0	0.0	0.0
0.0	0.0	0.0	0.0	0.0	0.0	0.0	0.0
0.0	0.0	0.0	0.0	0.0	0.0	0.0	0.0
0.0	0.0	0.0	0.0	0.0	0.0	0.0	0.0
0.0	0.0	0.0	0.0	0.0	0.0	0.0	0.0
0.0	0.0	0.0	0.0	0.0	0.0	0.0	0.0
0.0	0.0	0.0	0.0	0.0	0.0	0.0	0.0
0.0	0.0	0.0	0.0	0.0	0.0	0.0	0.0
0.0	0.0	0.0	0.0	0.0	0.0	0.0	0.0
0.0	0.0	0.0	0.0	0.0	0.0	0.0	0.0
0.0	0.0	0.0	0.0	0.0	0.0	0.0	0.0
0.0	0.0	0.0	0.0	0.0	0.0	0.0	0.0
0.0	0.0	0.0	0.0	0.0	0.0	0.0	0.0
0.0	0.0	0.0	0.0	0.0	0.0	0.0	0.0
0.0	0.0	0.0	0.0	0.0	0.0	0.0	0.0
0.0	0.0	0.0	0.0	0.0	0.0	0.0	0.0
0.0	0.0	0.0	0.0	0.0	0.0	0.0	0.0

0.0	0.0	0.0	0.0	0.0	0.0	0.0	0.0
0.0	0.0	0.0	0.0	0.0	0.0	0.0	0.0
0.0	0.0	0.0	0.0	0.0	0.0	0.0	0.0
0.0	0.0	0.0	0.0	0.0	0.0	0.0	0.0
0.0	0.0	0.0	0.0	0.0	0.0	0.0	0.0
0.0	0.0	0.0	0.0	0.0	0.0	0.0	0.0
0.0	0.0	0.0	0.0	0.0	0.0	0.0	0.0
0.0	0.0	0.0	0.0	0.0	0.0	0.0	0.0
0.0	0.0	0.0	0.0	0.0	0.0	0.0	0.0
0.0	0.0	0.0	0.0	0.0	0.0	0.0	0.0
0.0	0.0	0.0	0.0	0.0	0.0	0.0	0.0
0.0	0.0	0.0	0.0	0.0	0.0	0.0	0.0
0.0	0.0	0.0	0.0	0.0	0.0	0.0	0.0
0.0	0.0	0.0	0.0	0.0	0.0	0.0	0.0
0.0	0.0	0.0	0.0	0.0	0.0	0.0	0.0
0.0	0.0	0.0	0.0	0.0	0.0	0.0	0.0
0.0	0.0	0.0	0.0	0.0	0.0	0.0	0.0
0.0	0.0	0.0	0.0	0.0	0.0	0.0	0.0
0.0	0.0	0.0	0.0	0.0	0.0	0.0	0.0
0.0	0.0	0.0	0.0	0.0	0.0	0.0	0.0
0.0	0.0	0.0	0.0	0.0	0.0	0.0	0.0
0.0	0.0	0.0	0.0	0.0	0.0	0.0	0.0
0.0	0.0	0.0	0.0	0.0	0.0	0.0	0.0
0.0	0.0	0.0	0.0	0.0	0.0	0.0	0.0
0.0	0.0	0.0	0.0	0.0	0.0	0.0	0.0
0.0	0.0	0.0	0.0	0.0	0.0	0.0	0.0
0.0	0.0	0.0	0.0	0.0	0.0	0.0	0.0
0.0	0.0	0.0	0.0	0.0	0.0	0.0	0.0
0.0	0.0	0.0	0.0	0.0	0.0	0.0	0.0
0.0	0.0	0.0	0.0	0.0	0.0	0.0	0.0
0.0	0.0	0.0	0.0	0.0	0.0	0.0	0.0
0.0	0.0	0.0	0.0	0.0	0.0	0.0	0.0
0.0	0.0	0.0	0.0	0.0	0.0	0.0	0.0
0.0	0.0	0.0	0.0	0.0	0.0	0.0	0.0
0.0	0.0	0.0	0.0	0.0	0.0	0.0	0.0
0.0	0.0	0.0	0.0	0.0	0.0	0.0	0.0
0.0	0.0	0.0	0.0	0.0	0.0	0.0	0.0
0.0	0.0	0.0	0.0	0.0	0.0	0.0	0.0
0.0	0.0	0.0	0.0	0.0	0.0	0.0	0.0
0.0	0.0	0.0	0.0	0.0	0.0	0.0	0.0
0.0	0.0	0.0	0.0	0.0	0.0	0.0	0.0
0.0	0.0	0.0	0.0	0.0	0.0	0.0	0.0
0.0	0.0	0.0	0.0	0.0	0.0	0.0	0.0
0.0	0.0	0.0	0.0	0.0	0.0	0.0	0.0
0.0	0.0	0.0	0.0	0.0	0.0	0.0	0.0
0.0	0.0	0.0	0.0	0.0	0.0	0.0	0.0
0.0	0.0	0.0	0.0	0.0	0.0	0.0	0.0
0.0	0.0	0.0	0.0	0.0	0.0	0.0	0.0

0.0	0.0	0.0	0.0	0.0	0.0	0.0	0.0
0.0	0.0	0.0	0.0	0.0	0.0	0.0	0.0
0.0	0.0	0.0	0.0	0.0	0.0	0.0	0.0
0.0	0.0	0.0	0.0	0.0	0.0	0.0	0.0
0.0	0.0	0.0	0.0	0.0	0.0	0.0	0.0
0.0	0.0	0.0	0.0	0.0	0.0	0.0	0.0
0.0	0.0	0.0	0.0	0.0	0.0	0.0	0.0
0.0	0.0	0.0	0.0	0.0	0.0	0.0	0.0
0.0	0.0	0.0	0.0	0.0	0.0	0.0	0.0
0.0	0.0	0.0	0.0	0.0	0.0	0.0	0.0
0.0	0.0	0.0	0.0	0.0	0.0	0.0	0.0
0.0	0.0	0.0	0.0	0.0	0.0	0.0	0.0
0.0	0.0	0.0	0.0	0.0	0.0	0.0	0.0
0.0	0.0	0.0	0.0	0.0	0.0	0.0	0.0
0.0	0.0	0.0	0.0	0.0	0.0	0.0	0.0
0.0	0.0	0.0	0.0	0.0	0.0	0.0	0.0
0.0	0.0	0.0	0.0	0.0	0.0	0.0	0.0
0.0	0.0	0.0	0.0	0.0	0.0	0.0	0.0
0.0	0.0	0.0	0.0	0.0	0.0	0.0	0.0
0.0	0.0	0.0	0.0	0.0	0.0	0.0	0.0
0.0	0.0	0.0	0.0	0.0	0.0	0.0	0.0
0.0	0.0	0.0	0.0	0.0	0.0	0.0	0.0
0.0	0.0	0.0	0.0	0.0	0.0	0.0	0.0
0.0	0.0	0.0	0.0	0.0	0.0	0.0	0.0
0.0	0.0	0.0	0.0	0.0	0.0	0.0	0.0
0.0	0.0	0.0	0.0	0.0	0.0	0.0	0.0
0.0	0.0	0.0	0.0	0.0	0.0	0.0	0.0
0.0	0.0	0.0	0.0	0.0	0.0	0.0	0.0
0.0	0.0	0.0	0.0	0.0	0.0	0.0	0.0
0.0	0.0	0.0	0.0	0.0	0.0	0.0	0.0
0.0	0.0	0.0	0.0	0.0	0.0	0.0	0.0
0.0	0.0	0.0	0.0	0.0	0.0	0.0	0.0
0.0	0.0	0.0	0.0	0.0	0.0	0.0	0.0
0.0	0.0	0.0	0.0	0.0	0.0	0.0	0.0
0.0	0.0	0.0	0.0	0.0	0.0	0.0	0.0
0.0	0.0	0.0	0.0	0.0	0.0	0.0	0.0
0.0	0.0	0.0	0.0	0.0	0.0	0.0	0.0
0.0	0.0	0.0	0.0	0.0	0.0	0.0	0.0
0.0	0.0	0.0	0.0	0.0	0.0	0.0	0.0
0.0	0.0	0.0	0.0	0.0	0.0	0.0	0.0
0.0	0.0	0.0	0.0	0.0	0.0	0.0	0.0
0.0	0.0	0.0	0.0	0.0	0.0	0.0	0.0
0.0	0.0	0.0	0.0	0.0	0.0	0.0	0.0
0.0	0.0	0.0	0.0	0.0	0.0	0.0	0.0
0.0	0.0	0.0	0.0	0.0	0.0	0.0	0.0
0.0	0.0	0.0	0.0	0.0	0.0	0.0	0.0
0.0	0.0	0.0	0.0	0.0	0.0	0.0	0.0

0.0	0.0	0.0	0.0	0.0	0.0	0.0	0.0
0.0	0.0	0.0	0.0	0.0	0.0	0.0	0.0
0.0	0.0	0.0	0.0	0.0	0.0	0.0	0.0
0.0	0.0	0.0	0.0	0.0	0.0	0.0	0.0
0.0	0.0	0.0	0.0	0.0	0.0	0.0	0.0
0.0	0.0	0.0	0.0	0.0	0.0	0.0	0.0
0.0	0.0	0.0	0.0	0.0	0.0	0.0	0.0
0.0	0.0	0.0	0.0	0.0	0.0	0.0	0.0
0.0	0.0	0.0	0.0	0.0	0.0	0.0	0.0
0.0	0.0	0.0	0.0	0.0	0.0	0.0	0.0
0.0	0.0	0.0	0.0	0.0	0.0	0.0	0.0
0.0	0.0	0.0	0.0	0.0	0.0	0.0	0.0
0.0	0.0	0.0	0.0	0.0	0.0	0.0	0.0
0.0	0.0	0.0	0.0	0.0	0.0	0.0	0.0
0.0	0.0	0.0	0.0	0.0	0.0	0.0	0.0
0.0	0.0	0.0	0.0	0.0	0.0	0.0	0.0
0.0	0.0	0.0	0.0	0.0	0.0	0.0	0.0
0.0	0.0	0.0	0.0	0.0	0.0	0.0	0.0
0.0	0.0	0.0	0.0	0.0	0.0	0.0	0.0
0.0	0.0	0.0	0.0	0.0	0.0	0.0	0.0
0.0	0.0	0.0	0.0	0.0	0.0	0.0	0.0
0.0	0.0	0.0	0.0	0.0	0.0	0.0	0.0
0.0	0.0	0.0	0.0	0.0	0.0	0.0	0.0
0.0	0.0	0.0	0.0	0.0	0.0	0.0	0.0
0.0	0.0	0.0	0.0	0.0	0.0	0.0	0.0
0.0	0.0	0.0	0.0	0.0	0.0	0.0	0.0
0.0	0.0	0.0	0.0	0.0	0.0	0.0	0.0
0.0	0.0	0.0	0.0	0.0	0.0	0.0	0.0
0.0	0.0	0.0	0.0	0.0	0.0	0.0	0.0
0.0	0.0	0.0	0.0	0.0	0.0	0.0	0.0
0.0	0.0	0.0	0.0	0.0	0.0	0.0	0.0
0.0	0.0	0.0	0.0	0.0	0.0	0.0	0.0
0.0	0.0	0.0	0.0	0.0	0.0	0.0	0.0
0.0	0.0	0.0	0.0	0.0	0.0	0.0	0.0
0.0	0.0	0.0	0.0	0.0	0.0	0.0	0.0
0.0	0.0	0.0	0.0	0.0	0.0	0.0	0.0
0.0	0.0	0.0	0.0	0.0	0.0	0.0	0.0
0.0	0.0	0.0	0.0	0.0	0.0	0.0	0.0
0.0	0.0	0.0	0.0	0.0	0.0	0.0	0.0
0.0	0.0	0.0	0.0	0.0	0.0	0.0	0.0
0.0	0.0	0.0	0.0	0.0	0.0	0.0	0.0
0.0	0.0	0.0	0.0	0.0	0.0	0.0	0.0
0.0	0.0	0.0	0.0	0.0	0.0	0.0	0.0
0.0	0.0	0.0	0.0	0.0	0.0	0.0	0.0
0.0	0.0	0.0	0.0	0.0	0.0	0.0	0.0
0.0	0.0	0.0	0.0	0.0	0.0	0.0	0.0
0.0	0.0	0.0	0.0	0.0	0.0	0.0	0.0

0.0	0.0	0.0	0.0	0.0	0.0	0.0	0.0
0.0	0.0	0.0	0.0	0.0	0.0	0.0	0.0
0.0	0.0	0.0	0.0	0.0	0.0	0.0	0.0
0.0	0.0	0.0	0.0	0.0	0.0	0.0	0.0
0.0	0.0	0.0	0.0	0.0	0.0	0.0	0.0
0.0	0.0	0.0	0.0	0.0	0.0	0.0	0.0
0.0	0.0	0.0	0.0	0.0	0.0	0.0	0.0
0.0	0.0	0.0	0.0	0.0	0.0	0.0	0.0
0.0	0.0	0.0	0.0	0.0	0.0	0.0	0.0
0.0	0.0	0.0	0.0	0.0	0.0	0.0	0.0
0.0	0.0	0.0	0.0	0.0	0.0	0.0	0.0
0.0	0.0	0.0	0.0	0.0	0.0	0.0	0.0
0.0	0.0	0.0	0.0	0.0	0.0	0.0	0.0
0.0	0.0	0.0	0.0	0.0	0.0	0.0	0.0
0.0	0.0	0.0	0.0	0.0	0.0	0.0	0.0
0.0	0.0	0.0	0.0	0.0	0.0	0.0	0.0
0.0	0.0	0.0	0.0	0.0	0.0	0.0	0.0
0.0	0.0	0.0	0.0	0.0	0.0	0.0	0.0
0.0	0.0	0.0	0.0	0.0	0.0	0.0	0.0
0.0	0.0	0.0	0.0	0.0	0.0	0.0	0.0
0.0	0.0	0.0	0.0	0.0	0.0	0.0	0.0
0.0	0.0	0.0	0.0	0.0	0.0	0.0	0.0
0.0	0.0	0.0	0.0	0.0	0.0	0.0	0.0
0.0	0.0	0.0	0.0	0.0	0.0	0.0	0.0
0.0	0.0	0.0	0.0	0.0	0.0	0.0	0.0
0.0	0.0	0.0	0.0	0.0	0.0	0.0	0.0
0.0	0.0	0.0	0.0	0.0	0.0	0.0	0.0
0.0	0.0	0.0	0.0	0.0	0.0	0.0	0.0
0.0	0.0	0.0	0.0	0.0	0.0	0.0	0.0
0.0	0.0	0.0	0.0	0.0	0.0	0.0	0.0
0.0	0.0	0.0	0.0	0.0	0.0	0.0	0.0
0.0	0.0	0.0	0.0	0.0	0.0	0.0	0.0
0.0	0.0	0.0	0.0	0.0	0.0	0.0	0.0
0.0	0.0	0.0	0.0	0.0	0.0	0.0	0.0
0.0	0.0	0.0	0.0	0.0	0.0	0.0	0.0
0.0	0.0	0.0	0.0	0.0	0.0	0.0	0.0
0.0	0.0	0.0	0.0	0.0	0.0	0.0	0.0
0.0	0.0	0.0	0.0	0.0	0.0	0.0	0.0
0.0	0.0	0.0	0.0	0.0	0.0	0.0	0.0
0.0	0.0	0.0	0.0	0.0	0.0	0.0	0.0
0.0	0.0	0.0	0.0	0.0	0.0	0.0	0.0
0.0	0.0	0.0	0.0	0.0	0.0	0.0	0.0
0.0	0.0	0.0	0.0	0.0	0.0	0.0	0.0
0.0	0.0	0.0	0.0	0.0	0.0	0.0	0.0
0.0	0.0	0.0	0.0	0.0	0.0	0.0	0.0
0.0	0.0	0.0	0.0	0.0	0.0	0.0	0.0
0.0	0.0	0.0	0.0	0.0	0.0	0.0	0.0
0.0	0.0	0.0	0.0	0.0	0.0	0.0	0.0

0.0	0.0	0.0	0.0	0.0	0.0	0.0	0.0
0.0	0.0	0.0	0.0	0.0	0.0	0.0	0.0
0.0	0.0	0.0	0.0	0.0	0.0	0.0	0.0
0.0	0.0	0.0	0.0	0.0	0.0	0.0	0.0
0.0	0.0	0.0	0.0	0.0	0.0	0.0	0.0
0.0	0.0	0.0	0.0	0.0	0.0	0.0	0.0
0.0	0.0	0.0	0.0	0.0	0.0	0.0	0.0
0.0	0.0	0.0	0.0	0.0	0.0	0.0	0.0
0.0	0.0	0.0	0.0	0.0	0.0	0.0	0.0
0.0	0.0	0.0	0.0	0.0	0.0	0.0	0.0
0.0	0.0	0.0	0.0	0.0	0.0	0.0	0.0
0.0	0.0	0.0	0.0	0.0	0.0	0.0	0.0
0.0	0.0	0.0	0.0	0.0	0.0	0.0	0.0
0.0	0.0	0.0	0.0	0.0	0.0	0.0	0.0
0.0	0.0	0.0	0.0	0.0	0.0	0.0	0.0
0.0	0.0	0.0	0.0	0.0	0.0	0.0	0.0
0.0	0.0	0.0	0.0	0.0	0.0	0.0	0.0
0.0	0.0	0.0	0.0	0.0	0.0	0.0	0.0
0.0	0.0	0.0	0.0	0.0	0.0	0.0	0.0
0.0	0.0	0.0	0.0	0.0	0.0	0.0	0.0
0.0	0.0	0.0	0.0	0.0	0.0	0.0	0.0
0.0	0.0	0.0	0.0	0.0	0.0	0.0	0.0
0.0	0.0	0.0	0.0	0.0	0.0	0.0	0.0
0.0	0.0	0.0	0.0	0.0	0.0	0.0	0.0
0.0	0.0	0.0	0.0	0.0	0.0	0.0	0.0
0.0	0.0	0.0	0.0	0.0	0.0	0.0	0.0
0.0	0.0	0.0	0.0	0.0	0.0	0.0	0.0
0.0	0.0	0.0	0.0	0.0	0.0	0.0	0.0
0.0	0.0	0.0	0.0	0.0	0.0	0.0	0.0
0.0	0.0	0.0	0.0	0.0	0.0	0.0	0.0
0.0	0.0	0.0	0.0	0.0	0.0	0.0	0.0
0.0	0.0	0.0	0.0	0.0	0.0	0.0	0.0
0.0	0.0	0.0	0.0	0.0	0.0	0.0	0.0
0.0	0.0	0.0	0.0	0.0	0.0	0.0	0.0
0.0	0.0	0.0	0.0	0.0	0.0	0.0	0.0
0.0	0.0	0.0	0.0	0.0	0.0	0.0	0.0
0.0	0.0	0.0	0.0	0.0	0.0	0.0	0.0
0.0	0.0	0.0	0.0	0.0	0.0	0.0	0.0
0.0	0.0	0.0	0.0	0.0	0.0	0.0	0.0
0.0	0.0	0.0	0.0	0.0	0.0	0.0	0.0
0.0	0.0	0.0	0.0	0.0	0.0	0.0	0.0
0.0	0.0	0.0	0.0	0.0	0.0	0.0	0.0
0.0	0.0	0.0	0.0	0.0	0.0	0.0	0.0
0.0	0.0	0.0	0.0	0.0	0.0	0.0	0.0
0.0	0.0	0.0	0.0	0.0	0.0	0.0	0.0
0.0	0.0	0.0	0.0	0.0	0.0	0.0	0.0
0.0	0.0	0.0	0.0	0.0	0.0	0.0	0.0

0.0	0.0	0.0	0.0	0.0	0.0	0.0	0.0
0.0	0.0	0.0	0.0	0.0	0.0	0.0	0.0
0.0	0.0	0.0	0.0	0.0	0.0	0.0	0.0
0.0	0.0	0.0	0.0	0.0	0.0	0.0	0.0
0.0	0.0	0.0	0.0	0.0	0.0	0.0	0.0
0.0	0.0	0.0	0.0	0.0	0.0	0.0	0.0
0.0	0.0	0.0	0.0	0.0	0.0	0.0	0.0
0.0	0.0	0.0	0.0	0.0	0.0	0.0	0.0
0.0	0.0	0.0	0.0	0.0	0.0	0.0	0.0
0.0	0.0	0.0	0.0	0.0	0.0	0.0	0.0
0.0	0.0	0.0	0.0	0.0	0.0	0.0	0.0
0.0	0.0	0.0	0.0	0.0	0.0	0.0	0.0
0.0	0.0	0.0	0.0	0.0	0.0	0.0	0.0
0.0	0.0	0.0	0.0	0.0	0.0	0.0	0.0
0.0	0.0	0.0	0.0	0.0	0.0	0.0	0.0
0.0	0.0	0.0	0.0	0.0	0.0	0.0	0.0
0.0	0.0	0.0	0.0	0.0	0.0	0.0	0.0
0.0	0.0	0.0	0.0	0.0	0.0	0.0	0.0
0.0	0.0	0.0	0.0	0.0	0.0	0.0	0.0
0.0	0.0	0.0	0.0	0.0	0.0	0.0	0.0
0.0	0.0	0.0	0.0	0.0	0.0	0.0	0.0
0.0	0.0	0.0	0.0	0.0	0.0	0.0	0.0
0.0	0.0	0.0	0.0	0.0	0.0	0.0	0.0
0.0	0.0	0.0	0.0	0.0	0.0	0.0	0.0
0.0	0.0	0.0	0.0	0.0	0.0	0.0	0.0
0.0	0.0	0.0	0.0	0.0	0.0	0.0	0.0
0.0	0.0	0.0	0.0	0.0	0.0	0.0	0.0
0.0	0.0	0.0	0.0	0.0	0.0	0.0	0.0
0.0	0.0	0.0	0.0	0.0	0.0	0.0	0.0
0.0	0.0	0.0	0.0	0.0	0.0	0.0	0.0
0.0	0.0	0.0	0.0	0.0	0.0	0.0	0.0
0.0	0.0	0.0	0.0	0.0	0.0	0.0	0.0
0.0	0.0	0.0	0.0	0.0	0.0	0.0	0.0
0.0	0.0	0.0	0.0	0.0	0.0	0.0	0.0
0.0	0.0	0.0	0.0	0.0	0.0	0.0	0.0
0.0	0.0	0.0	0.0	0.0	0.0	0.0	0.0
0.0	0.0	0.0	0.0	0.0	0.0	0.0	0.0
0.0	0.0	0.0	0.0	0.0	0.0	0.0	0.0
0.0	0.0	0.0	0.0	0.0	0.0	0.0	0.0
0.0	0.0	0.0	0.0	0.0	0.0	0.0	0.0
0.0	0.0	0.0	0.0	0.0	0.0	0.0	0.0
0.0	0.0	0.0	0.0	0.0	0.0	0.0	0.0
0.0	0.0	0.0	0.0	0.0	0.0	0.0	0.0
0.0	0.0	0.0	0.0	0.0	0.0	0.0	0.0
0.0	0.0	0.0	0.0	0.0	0.0	0.0	0.0
0.0	0.0	0.0	0.0	0.0	0.0	0.0	0.0
0.0	0.0	0.0	0.0	0.0	0.0	0.0	0.0

0.0	0.0	0.0	0.0	0.0	0.0	0.0	0.0
0.0	0.0	0.0	0.0	0.0	0.0	0.0	0.0
0.0	0.0	0.0	0.0	0.0	0.0	0.0	0.0
0.0	0.0	0.0	0.0	0.0	0.0	0.0	0.0
0.0	0.0	0.0	0.0	0.0	0.0	0.0	0.0
0.0	0.0	0.0	0.0	0.0	0.0	0.0	0.0
0.0	0.0	0.0	0.0	0.0	0.0	0.0	0.0
0.0	0.0	0.0	0.0	0.0	0.0	0.0	0.0
0.0	0.0	0.0	0.0	0.0	0.0	0.0	0.0
0.0	0.0	0.0	0.0	0.0	0.0	0.0	0.0
0.0	0.0	0.0	0.0	0.0	0.0	0.0	0.0
0.0	0.0	0.0	0.0	0.0	0.0	0.0	0.0
0.0	0.0	0.0	0.0	0.0	0.0	0.0	0.0
0.0	0.0	0.0	0.0	0.0	0.0	0.0	0.0
0.0	0.0	0.0	0.0	0.0	0.0	0.0	0.0
0.0	0.0	0.0	0.0	0.0	0.0	0.0	0.0
0.0	0.0	0.0	0.0	0.0	0.0	0.0	0.0
0.0	0.0	0.0	0.0	0.0	0.0	0.0	0.0
0.0	0.0	0.0	0.0	0.0	0.0	0.0	0.0
0.0	0.0	0.0	0.0	0.0	0.0	0.0	0.0
0.0	0.0	0.0	0.0	0.0	0.0	0.0	0.0
0.0	0.0	0.0	0.0	0.0	0.0	0.0	0.0
0.0	0.0	0.0	0.0	0.0	0.0	0.0	0.0
0.0	0.0	0.0	0.0	0.0	0.0	0.0	0.0
0.0	0.0	0.0	0.0	0.0	0.0	0.0	0.0
0.0	0.0	0.0	0.0	0.0	0.0	0.0	0.0
0.0	0.0	0.0	0.0	0.0	0.0	0.0	0.0
0.0	0.0	0.0	0.0	0.0	0.0	0.0	0.0
0.0	0.0	0.0	0.0	0.0	0.0	0.0	0.0
0.0	0.0	0.0	0.0	0.0	0.0	0.0	0.0
0.0	0.0	0.0	0.0	0.0	0.0	0.0	0.0
0.0	0.0	0.0	0.0	0.0	0.0	0.0	0.0
0.0	0.0	0.0	0.0	0.0	0.0	0.0	0.0
0.0	0.0	0.0	0.0	0.0	0.0	0.0	0.0
0.0	0.0	0.0	0.0	0.0	0.0	0.0	0.0
0.0	0.0	0.0	0.0	0.0	0.0	0.0	0.0
0.0	0.0	0.0	0.0	0.0	0.0	0.0	0.0
0.0	0.0	0.0	0.0	0.0	0.0	0.0	0.0
0.0	0.0	0.0	0.0	0.0	0.0	0.0	0.0
0.0	0.0	0.0	0.0	0.0	0.0	0.0	0.0
0.0	0.0	0.0	0.0	0.0	0.0	0.0	0.0
0.0	0.0	0.0	0.0	0.0	0.0	0.0	0.0
0.0	0.0	0.0	0.0	0.0	0.0	0.0	0.0
0.0	0.0	0.0	0.0	0.0	0.0	0.0	0.0
0.0	0.0	0.0	0.0	0.0	0.0	0.0	0.0
0.0	0.0	0.0	0.0	0.0	0.0	0.0	0.0
0.0	0.0	0.0	0.0	0.0	0.0	0.0	0.0

0.0	0.0	0.0	0.0	0.0	0.0	0.0	0.0
0.0	0.0	0.0	0.0	0.0	0.0	0.0	0.0
0.0	0.0	0.0	0.0	0.0	0.0	0.0	0.0
0.0	0.0	0.0	0.0	0.0	0.0	0.0	0.0
0.0	0.0	0.0	0.0	0.0	0.0	0.0	0.0
0.0	0.0	0.0	0.0	0.0	0.0	0.0	0.0
0.0	0.0	0.0	0.0	0.0	0.0	0.0	0.0
0.0	0.0	0.0	0.0	0.0	0.0	0.0	0.0
0.0	0.0	0.0	0.0	0.0	0.0	0.0	0.0
0.0	0.0	0.0	0.0	0.0	0.0	0.0	0.0
0.0	0.0	0.0	0.0	0.0	0.0	0.0	0.0
0.0	0.0	0.0	0.0	0.0	0.0	0.0	0.0
0.0	0.0	0.0	0.0	0.0	0.0	0.0	0.0
0.0	0.0	0.0	0.0	0.0	0.0	0.0	0.0
0.0	0.0	0.0	0.0	0.0	0.0	0.0	0.0
0.0	0.0	0.0	0.0	0.0	0.0	0.0	0.0
0.0	0.0	0.0	0.0	0.0	0.0	0.0	0.0
0.0	0.0	0.0	0.0	0.0	0.0	0.0	0.0
0.0	0.0	0.0	0.0	0.0	0.0	0.0	0.0
0.0	0.0	0.0	0.0	0.0	0.0	0.0	0.0
0.0	0.0	0.0	0.0	0.0	0.0	0.0	0.0
0.0	0.0	0.0	0.0	0.0	0.0	0.0	0.0
0.0	0.0	0.0	0.0	0.0	0.0	0.0	0.0
0.0	0.0	0.0	0.0	0.0	0.0	0.0	0.0
0.0	0.0	0.0	0.0	0.0	0.0	0.0	0.0
0.0	0.0	0.0	0.0	0.0	0.0	0.0	0.0
0.0	0.0	0.0	0.0	0.0	0.0	0.0	0.0
0.0	0.0	0.0	0.0	0.0	0.0	0.0	0.0
0.0	0.0	0.0	0.0	0.0	0.0	0.0	0.0
0.0	0.0	0.0	0.0	0.0	0.0	0.0	0.0
0.0	0.0	0.0	0.0	0.0	0.0	0.0	0.0
0.0	0.0	0.0	0.0	0.0	0.0	0.0	0.0
0.0	0.0	0.0	0.0	0.0	0.0	0.0	0.0
0.0	0.0	0.0	0.0	0.0	0.0	0.0	0.0
0.0	0.0	0.0	0.0	0.0	0.0	0.0	0.0
0.0	0.0	0.0	0.0	0.0	0.0	0.0	0.0
0.0	0.0	0.0	0.0	0.0	0.0	0.0	0.0
0.0	0.0	0.0	0.0	0.0	0.0	0.0	0.0
0.0	0.0	0.0	0.0	0.0	0.0	0.0	0.0
0.0	0.0	0.0	0.0	0.0	0.0	0.0	0.0
0.0	0.0	0.0	0.0	0.0	0.0	0.0	0.0
0.0	0.0	0.0	0.0	0.0	0.0	0.0	0.0
0.0	0.0	0.0	0.0	0.0	0.0	0.0	0.0
0.0	0.0	0.0	0.0	0.0	0.0	0.0	0.0
0.0	0.0	0.0	0.0	0.0	0.0	0.0	0.0
0.0	0.0	0.0	0.0	0.0	0.0	0.0	0.0
0.0	0.0	0.0	0.0	0.0	0.0	0.0	0.0

0.0	0.0	0.0	0.0	0.0	0.0	0.0	0.0
0.0	0.0	0.0	0.0	0.0	0.0	0.0	0.0
0.0	0.0	0.0	0.0	0.0	0.0	0.0	0.0
0.0	0.0	0.0	0.0	0.0	0.0	0.0	0.0
0.0	0.0	0.0	0.0	0.0	0.0	0.0	0.0
0.0	0.0	0.0	0.0	0.0	0.0	0.0	0.0
0.0	0.0	0.0	0.0	0.0	0.0	0.0	0.0
0.0	0.0	0.0	0.0	0.0	0.0	0.0	0.0
0.0	0.0	0.0	0.0	0.0	0.0	0.0	0.0
0.0	0.0	0.0	0.0	0.0	0.0	0.0	0.0
0.0	0.0	0.0	0.0	0.0	0.0	0.0	0.0
0.0	0.0	0.0	0.0	0.0	0.0	0.0	0.0
0.0	0.0	0.0	0.0	0.0	0.0	0.0	0.0
0.0	0.0	0.0	0.0	0.0	0.0	0.0	0.0
0.0	0.0	0.0	0.0	0.0	0.0	0.0	0.0
0.0	0.0	0.0	0.0	0.0	0.0	0.0	0.0
0.0	0.0	0.0	0.0	0.0	0.0	0.0	0.0
0.0	0.0	0.0	0.0	0.0	0.0	0.0	0.0
0.0	0.0	0.0	0.0	0.0	0.0	0.0	0.0
0.0	0.0	0.0	0.0	0.0	0.0	0.0	0.0
0.0	0.0	0.0	0.0	0.0	0.0	0.0	0.0
0.0	0.0	0.0	0.0	0.0	0.0	0.0	0.0
0.0	0.0	0.0	0.0	0.0	0.0	0.0	0.0
0.0	0.0	0.0	0.0	0.0	0.0	0.0	0.0
0.0	0.0	0.0	0.0	0.0	0.0	0.0	0.0
0.0	0.0	0.0	0.0	0.0	0.0	0.0	0.0
0.0	0.0	0.0	0.0	0.0	0.0	0.0	0.0
0.0	0.0	0.0	0.0	0.0	0.0	0.0	0.0
0.0	0.0	0.0	0.0	0.0	0.0	0.0	0.0
0.0	0.0	0.0	0.0	0.0	0.0	0.0	0.0
0.0	0.0	0.0	0.0	0.0	0.0	0.0	0.0
0.0	0.0	0.0	0.0	0.0	0.0	0.0	0.0
0.0	0.0	0.0	0.0	0.0	0.0	0.0	0.0
0.0	0.0	0.0	0.0	0.0	0.0	0.0	0.0
0.0	0.0	0.0	0.0	0.0	0.0	0.0	0.0
0.0	0.0	0.0	0.0	0.0	0.0	0.0	0.0
0.0	0.0	0.0	0.0	0.0	0.0	0.0	0.0
0.0	0.0	0.0	0.0	0.0	0.0	0.0	0.0
0.0	0.0	0.0	0.0	0.0	0.0	0.0	0.0
0.0	0.0	0.0	0.0	0.0	0.0	0.0	0.0
0.0	0.0	0.0	0.0	0.0	0.0	0.0	0.0
0.0	0.0	0.0	0.0	0.0	0.0	0.0	0.0
0.0	0.0	0.0	0.0	0.0	0.0	0.0	0.0
0.0	0.0	0.0	0.0	0.0	0.0	0.0	0.0
0.0	0.0	0.0	0.0	0.0	0.0	0.0	0.0
0.0	0.0	0.0	0.0	0.0	0.0	0.0	0.0
0.0	0.0	0.0	0.0	0.0	0.0	0.0	0.0
0.0	0.0	0.0	0.0	0.0	0.0	0.0	0.0

0.0	0.0	0.0	0.0	0.0	0.0	0.0	0.0
0.0	0.0	0.0	0.0	0.0	0.0	0.0	0.0
0.0	0.0	0.0	0.0	0.0	0.0	0.0	0.0
0.0	0.0	0.0	0.0	0.0	0.0	0.0	0.0
0.0	0.0	0.0	0.0	0.0	0.0	0.0	0.0
0.0	0.0	0.0	0.0	0.0	0.0	0.0	0.0
0.0	0.0	0.0	0.0	0.0	0.0	0.0	0.0
0.0	0.0	0.0	0.0	0.0	0.0	0.0	0.0
0.0	0.0	0.0	0.0	0.0	0.0	0.0	0.0
0.0	0.0	0.0	0.0	0.0	0.0	0.0	0.0
0.0	0.0	0.0	0.0	0.0	0.0	0.0	0.0
0.0	0.0	0.0	0.0	0.0	0.0	0.0	0.0
0.0	0.0	0.0	0.0	0.0	0.0	0.0	0.0
0.0	0.0	0.0	0.0	0.0	0.0	0.0	0.0
0.0	0.0	0.0	0.0	0.0	0.0	0.0	0.0
0.0	0.0	0.0	0.0	0.0	0.0	0.0	0.0
0.0	0.0	0.0	0.0	0.0	0.0	0.0	0.0
0.0	0.0	0.0	0.0	0.0	0.0	0.0	0.0
0.0	0.0	0.0	0.0	0.0	0.0	0.0	0.0
0.0	0.0	0.0	0.0	0.0	0.0	0.0	0.0
0.0	0.0	0.0	0.0	0.0	0.0	0.0	0.0
0.0	0.0	0.0	0.0	0.0	0.0	0.0	0.0
0.0	0.0	0.0	0.0	0.0	0.0	0.0	0.0
0.0	0.0	0.0	0.0	0.0	0.0	0.0	0.0
0.0	0.0	0.0	0.0	0.0	0.0	0.0	0.0
0.0	0.0	0.0	0.0	0.0	0.0	0.0	0.0
0.0	0.0	0.0	0.0	0.0	0.0	0.0	0.0
0.0	0.0	0.0	0.0	0.0	0.0	0.0	0.0
0.0	0.0	0.0	0.0	0.0	0.0	0.0	0.0
0.0	0.0	0.0	0.0	0.0	0.0	0.0	0.0
0.0	0.0	0.0	0.0	0.0	0.0	0.0	0.0
0.0	0.0	0.0	0.0	0.0	0.0	0.0	0.0
0.0	0.0	0.0	0.0	0.0	0.0	0.0	0.0
0.0	0.0	0.0	0.0	0.0	0.0	0.0	0.0
0.0	0.0	0.0	0.0	0.0	0.0	0.0	0.0
0.0	0.0	0.0	0.0	0.0	0.0	0.0	0.0
0.0	0.0	0.0	0.0	0.0	0.0	0.0	0.0
0.0	0.0	0.0	0.0	0.0	0.0	0.0	0.0
0.0	0.0	0.0	0.0	0.0	0.0	0.0	0.0
0.0	0.0	0.0	0.0	0.0	0.0	0.0	0.0
0.0	0.0	0.0	0.0	0.0	0.0	0.0	0.0
0.0	0.0	0.0	0.0	0.0	0.0	0.0	0.0
0.0	0.0	0.0	0.0	0.0	0.0	0.0	0.0
0.0	0.0	0.0	0.0	0.0	0.0	0.0	0.0
0.0	0.0	0.0	0.0	0.0	0.0	0.0	0.0
0.0	0.0	0.0	0.0	0.0	0.0	0.0	0.0
0.0	0.0	0.0	0.0	0.0	0.0	0.0	0.0

0.0	0.0	0.0	0.0	0.0	0.0	0.0	0.0
0.0	0.0	0.0	0.0	0.0	0.0	0.0	0.0
0.0	0.0	0.0	0.0	0.0	0.0	0.0	0.0
0.0	0.0	0.0	0.0	0.0	0.0	0.0	0.0
0.0	0.0	0.0	0.0	0.0	0.0	0.0	0.0
0.0	0.0	0.0	0.0	0.0	0.0	0.0	0.0
0.0	0.0	0.0	0.0	0.0	0.0	0.0	0.0
0.0	0.0	0.0	0.0	0.0	0.0	0.0	0.0
0.0	0.0	0.0	0.0	0.0	0.0	0.0	0.0
0.0	0.0	0.0	0.0	0.0	0.0	0.0	0.0
0.0	0.0	0.0	0.0	0.0	0.0	0.0	0.0
0.0	0.0	0.0	0.0	0.0	0.0	0.0	0.0
0.0	0.0	0.0	0.0	0.0	0.0	0.0	0.0
0.0	0.0	0.0	0.0	0.0	0.0	0.0	0.0
0.0	0.0	0.0	0.0	0.0	0.0	0.0	0.0
0.0	0.0	0.0	0.0	0.0	0.0	0.0	0.0
0.0	0.0	0.0	0.0	0.0	0.0	0.0	0.0
0.0	0.0	0.0	0.0	0.0	0.0	0.0	0.0
0.0	0.0	0.0	0.0	0.0	0.0	0.0	0.0
0.0	0.0	0.0	0.0	0.0	0.0	0.0	0.0
0.0	0.0	0.0	0.0	0.0	0.0	0.0	0.0
0.0	0.0	0.0	0.0	0.0	0.0	0.0	0.0
0.0	0.0	0.0	0.0	0.0	0.0	0.0	0.0
0.0	0.0	0.0	0.0	0.0	0.0	0.0	0.0
0.0	0.0	0.0	0.0	0.0	0.0	0.0	0.0
0.0	0.0	0.0	0.0	0.0	0.0	0.0	0.0
0.0	0.0	0.0	0.0	0.0	0.0	0.0	0.0
0.0	0.0	0.0	0.0	0.0	0.0	0.0	0.0
0.0	0.0	0.0	0.0	0.0	0.0	0.0	0.0
0.0	0.0	0.0	0.0	0.0	0.0	0.0	0.0
0.0	0.0	0.0	0.0	0.0	0.0	0.0	0.0
0.0	0.0	0.0	0.0	0.0	0.0	0.0	0.0
0.0	0.0	0.0	0.0	0.0	0.0	0.0	0.0
0.0	0.0	0.0	0.0	0.0	0.0	0.0	0.0
0.0	0.0	0.0	0.0	0.0	0.0	0.0	0.0
0.0	0.0	0.0	0.0	0.0	0.0	0.0	0.0
0.0	0.0	0.0	0.0	0.0	0.0	0.0	0.0
0.0	0.0	0.0	0.0	0.0	0.0	0.0	0.0
0.0	0.0	0.0	0.0	0.0	0.0	0.0	0.0
0.0	0.0	0.0	0.0	0.0	0.0	0.0	0.0
0.0	0.0	0.0	0.0	0.0	0.0	0.0	0.0
0.0	0.0	0.0	0.0	0.0	0.0	0.0	0.0
0.0	0.0	0.0	0.0	0.0	0.0	0.0	0.0
0.0	0.0	0.0	0.0	0.0	0.0	0.0	0.0
0.0	0.0	0.0	0.0	0.0	0.0	0.0	0.0
0.0	0.0	0.0	0.0	0.0	0.0	0.0	0.0
0.0	0.0	0.0	0.0	0.0	0.0	0.0	0.0
0.0	0.0	0.0	0.0	0.0	0.0	0.0	0.0

0.0	0.0	0.0	0.0	0.0	0.0	0.0	0.0
0.0	0.0	0.0	0.0	0.0	0.0	0.0	0.0
0.0	0.0	0.0	0.0	0.0	0.0	0.0	0.0
0.0	0.0	0.0	0.0	0.0	0.0	0.0	0.0
0.0	0.0	0.0	0.0	0.0	0.0	0.0	0.0
0.0	0.0	0.0	0.0	0.0	0.0	0.0	0.0
0.0	0.0	0.0	0.0	0.0	0.0	0.0	0.0
0.0	0.0	0.0	0.0	0.0	0.0	0.0	0.0
0.0	0.0	0.0	0.0	0.0	0.0	0.0	0.0
0.0	0.0	0.0	0.0	0.0	0.0	0.0	0.0
0.0	0.0	0.0	0.0	0.0	0.0	0.0	0.0
0.0	0.0	0.0	0.0	0.0	0.0	0.0	0.0
0.0	0.0	0.0	0.0	0.0	0.0	0.0	0.0
0.0	0.0	0.0	0.0	0.0	0.0	0.0	0.0
0.0	0.0	0.0	0.0	0.0	0.0	0.0	0.0
0.0	0.0	0.0	0.0	0.0	0.0	0.0	0.0
0.0	0.0	0.0	0.0	0.0	0.0	0.0	0.0
0.0	0.0	0.0	0.0	0.0	0.0	0.0	0.0
0.0	0.0	0.0	0.0	0.0	0.0	0.0	0.0
0.0	0.0	0.0	0.0	0.0	0.0	0.0	0.0
0.0	0.0	0.0	0.0	0.0	0.0	0.0	0.0
0.0	0.0	0.0	0.0	0.0	0.0	0.0	0.0
0.0	0.0	0.0	0.0	0.0	0.0	0.0	0.0
0.0	0.0	0.0	0.0	0.0	0.0	0.0	0.0
0.0	0.0	0.0	0.0	0.0	0.0	0.0	0.0
0.0	0.0	0.0	0.0	0.0	0.0	0.0	0.0
0.0	0.0	0.0	0.0	0.0	0.0	0.0	0.0
0.0	0.0	0.0	0.0	0.0	0.0	0.0	0.0
0.0	0.0	0.0	0.0	0.0	0.0	0.0	0.0
0.0	0.0	0.0	0.0	0.0	0.0	0.0	0.0
0.0	0.0	0.0	0.0	0.0	0.0	0.0	0.0
0.0	0.0	0.0	0.0	0.0	0.0	0.0	0.0
0.0	0.0	0.0	0.0	0.0	0.0	0.0	0.0
0.0	0.0	0.0	0.0	0.0	0.0	0.0	0.0
0.0	0.0	0.0	0.0	0.0	0.0	0.0	0.0
0.0	0.0	0.0	0.0	0.0	0.0	0.0	0.0
0.0	0.0	0.0	0.0	0.0	0.0	0.0	0.0
0.0	0.0	0.0	0.0	0.0	0.0	0.0	0.0
0.0	0.0	0.0	0.0	0.0	0.0	0.0	0.0
0.0	0.0	0.0	0.0	0.0	0.0	0.0	0.0
0.0	0.0	0.0	0.0	0.0	0.0	0.0	0.0
0.0	0.0	0.0	0.0	0.0	0.0	0.0	0.0
0.0	0.0	0.0	0.0	0.0	0.0	0.0	0.0
0.0	0.0	0.0	0.0	0.0	0.0	0.0	0.0
0.0	0.0	0.0	0.0	0.0	0.0	0.0	0.0
0.0	0.0	0.0	0.0	0.0	0.0	0.0	0.0
0.0	0.0	0.0	0.0	0.0	0.0	0.0	0.0

0.0	0.0	0.0	0.0	0.0	0.0	0.0	0.0
0.0	0.0	0.0	0.0	0.0	0.0	0.0	0.0
0.0	0.0	0.0	0.0	0.0	0.0	0.0	0.0
0.0	0.0	0.0	0.0	0.0	0.0	0.0	0.0
0.0	0.0	0.0	0.0	0.0	0.0	0.0	0.0
0.0	0.0	0.0	0.0	0.0	0.0	0.0	0.0
0.0	0.0	0.0	0.0	0.0	0.0	0.0	0.0
0.0	0.0	0.0	0.0	0.0	0.0	0.0	0.0
0.0	0.0	0.0	0.0	0.0	0.0	0.0	0.0
0.0	0.0	0.0	0.0	0.0	0.0	0.0	0.0
0.0	0.0	0.0	0.0	0.0	0.0	0.0	0.0
0.0	0.0	0.0	0.0	0.0	0.0	0.0	0.0
0.0	0.0	0.0	0.0	0.0	0.0	0.0	0.0
0.0	0.0	0.0	0.0	0.0	0.0	0.0	0.0
0.0	0.0	0.0	0.0	0.0	0.0	0.0	0.0
0.0	0.0	0.0	0.0	0.0	0.0	0.0	0.0
0.0	0.0	0.0	0.0	0.0	0.0	0.0	0.0
0.0	0.0	0.0	0.0	0.0	0.0	0.0	0.0
0.0	0.0	0.0	0.0	0.0	0.0	0.0	0.0
0.0	0.0	0.0	0.0	0.0	0.0	0.0	0.0
0.0	0.0	0.0	0.0	0.0	0.0	0.0	0.0
0.0	0.0	0.0	0.0	0.0	0.0	0.0	0.0
0.0	0.0	0.0	0.0	0.0	0.0	0.0	0.0
0.0	0.0	0.0	0.0	0.0	0.0	0.0	0.0
0.0	0.0	0.0	0.0	0.0	0.0	0.0	0.0
0.0	0.0	0.0	0.0	0.0	0.0	0.0	0.0
0.0	0.0	0.0	0.0	0.0	0.0	0.0	0.0
0.0	0.0	0.0	0.0	0.0	0.0	0.0	0.0
0.0	0.0	0.0	0.0	0.0	0.0	0.0	0.0
0.0	0.0	0.0	0.0	0.0	0.0	0.0	0.0
0.0	0.0	0.0	0.0	0.0	0.0	0.0	0.0
0.0	0.0	0.0	0.0	0.0	0.0	0.0	0.0
0.0	0.0	0.0	0.0	0.0	0.0	0.0	0.0
0.0	0.0	0.0	0.0	0.0	0.0	0.0	0.0
0.0	0.0	0.0	0.0	0.0	0.0	0.0	0.0
0.0	0.0	0.0	0.0	0.0	0.0	0.0	0.0
0.0	0.0	0.0	0.0	0.0	0.0	0.0	0.0
0.0	0.0	0.0	0.0	0.0	0.0	0.0	0.0
0.0	0.0	0.0	0.0	0.0	0.0	0.0	0.0
0.0	0.0	0.0	0.0	0.0	0.0	0.0	0.0
0.0	0.0	0.0	0.0	0.0	0.0	0.0	0.0
0.0	0.0	0.0	0.0	0.0	0.0	0.0	0.0
0.0	0.0	0.0	0.0	0.0	0.0	0.0	0.0
0.0	0.0	0.0	0.0	0.0	0.0	0.0	0.0
0.0	0.0	0.0	0.0	0.0	0.0	0.0	0.0
0.0	0.0	0.0	0.0	0.0	0.0	0.0	0.0
0.0	0.0	0.0	0.0	0.0	0.0	0.0	0.0

0.0	0.0	0.0	0.0	0.0	0.0	0.0	0.0
0.0	0.0	0.0	0.0	0.0	0.0	0.0	0.0
0.0	0.0	0.0	0.0	0.0	0.0	0.0	0.0
0.0	0.0	0.0	0.0	0.0	0.0	0.0	0.0
0.0	0.0	0.0	0.0	0.0	0.0	0.0	0.0
0.0	0.0	0.0	0.0	0.0	0.0	0.0	0.0
0.0	0.0	0.0	0.0	0.0	0.0	0.0	0.0
0.0	0.0	0.0	0.0	0.0	0.0	0.0	0.0
0.0	0.0	0.0	0.0	0.0	0.0	0.0	0.0
0.0	0.0	0.0	0.0	0.0	0.0	0.0	0.0
0.0	0.0	0.0	0.0	0.0	0.0	0.0	0.0
0.0	0.0	0.0	0.0	0.0	0.0	0.0	0.0
0.0	0.0	0.0	0.0	0.0	0.0	0.0	0.0
0.0	0.0	0.0	0.0	0.0	0.0	0.0	0.0
0.0	0.0	0.0	0.0	0.0	0.0	0.0	0.0
0.0	0.0	0.0	0.0	0.0	0.0	0.0	0.0
0.0	0.0	0.0	0.0	0.0	0.0	0.0	0.0
0.0	0.0	0.0	0.0	0.0	0.0	0.0	0.0
0.0	0.0	0.0	0.0	0.0	0.0	0.0	0.0
0.0	0.0	0.0	0.0	0.0	0.0	0.0	0.0
0.0	0.0	0.0	0.0	0.0	0.0	0.0	0.0
0.0	0.0	0.0	0.0	0.0	0.0	0.0	0.0
0.0	0.0	0.0	0.0	0.0	0.0	0.0	0.0
0.0	0.0	0.0	0.0	0.0	0.0	0.0	0.0
0.0	0.0	0.0	0.0	0.0	0.0	0.0	0.0
0.0	0.0	0.0	0.0	0.0	0.0	0.0	0.0
0.0	0.0	0.0	0.0	0.0	0.0	0.0	0.0
0.0	0.0	0.0	0.0	0.0	0.0	0.0	0.0
0.0	0.0	0.0	0.0	0.0	0.0	0.0	0.0
0.0	0.0	0.0	0.0	0.0	0.0	0.0	0.0
0.0	0.0	0.0	0.0	0.0	0.0	0.0	0.0
0.0	0.0	0.0	0.0	0.0	0.0	0.0	0.0
0.0	0.0	0.0	0.0	0.0	0.0	0.0	0.0
0.0	0.0	0.0	0.0	0.0	0.0	0.0	0.0
0.0	0.0	0.0	0.0	0.0	0.0	0.0	0.0
0.0	0.0	0.0	0.0	0.0	0.0	0.0	0.0
0.0	0.0	0.0	0.0	0.0	0.0	0.0	0.0
0.0	0.0	0.0	0.0	0.0	0.0	0.0	0.0
0.0	0.0	0.0	0.0	0.0	0.0	0.0	0.0
0.0	0.0	0.0	0.0	0.0	0.0	0.0	0.0
0.0	0.0	0.0	0.0	0.0	0.0	0.0	0.0
0.0	0.0	0.0	0.0	0.0	0.0	0.0	0.0
0.0	0.0	0.0	0.0	0.0	0.0	0.0	0.0
0.0	0.0	0.0	0.0	0.0	0.0	0.0	0.0
0.0	0.0	0.0	0.0	0.0	0.0	0.0	0.0
0.0	0.0	0.0	0.0	0.0	0.0	0.0	0.0
0.0	0.0	0.0	0.0	0.0	0.0	0.0	0.0

0.0	0.0	0.0	0.0	0.0	0.0	0.0	0.0
0.0	0.0	0.0	0.0	0.0	0.0	0.0	0.0
0.0	0.0	0.0	0.0	0.0	0.0	0.0	0.0
0.0	0.0	0.0	0.0	0.0	0.0	0.0	0.0
0.0	0.0	0.0	0.0	0.0	0.0	0.0	0.0
0.0	0.0	0.0	0.0	0.0	0.0	0.0	0.0
0.0	0.0	0.0	0.0	0.0	0.0	0.0	0.0
0.0	0.0	0.0	0.0	0.0	0.0	0.0	0.0
0.0	0.0	0.0	0.0	0.0	0.0	0.0	0.0
0.0	0.0	0.0	0.0	0.0	0.0	0.0	0.0
0.0	0.0	0.0	0.0	0.0	0.0	0.0	0.0
0.0	0.0	0.0	0.0	0.0	0.0	0.0	0.0
0.0	0.0	0.0	0.0	0.0	0.0	0.0	0.0
0.0	0.0	0.0	0.0	0.0	0.0	0.0	0.0
0.0	0.0	0.0	0.0	0.0	0.0	0.0	0.0
0.0	0.0	0.0	0.0	0.0	0.0	0.0	0.0
0.0	0.0	0.0	0.0	0.0	0.0	0.0	0.0
0.0	0.0	0.0	0.0	0.0	0.0	0.0	0.0
0.0	0.0	0.0	0.0	0.0	0.0	0.0	0.0
0.0	0.0	0.0	0.0	0.0	0.0	0.0	0.0
0.0	0.0	0.0	0.0	0.0	0.0	0.0	0.0
0.0	0.0	0.0	0.0	0.0	0.0	0.0	0.0
0.0	0.0	0.0	0.0	0.0	0.0	0.0	0.0
0.0	0.0	0.0	0.0	0.0	0.0	0.0	0.0
0.0	0.0	0.0	0.0	0.0	0.0	0.0	0.0
0.0	0.0	0.0	0.0	0.0	0.0	0.0	0.0
0.0	0.0	0.0	0.0	0.0	0.0	0.0	0.0
0.0	0.0	0.0	0.0	0.0	0.0	0.0	0.0
0.0	0.0	0.0	0.0	0.0	0.0	0.0	0.0
0.0	0.0	0.0	0.0	0.0	0.0	0.0	0.0
0.0	0.0	0.0	0.0	0.0	0.0	0.0	0.0
0.0	0.0	0.0	0.0	0.0	0.0	0.0	0.0
0.0	0.0	0.0	0.0	0.0	0.0	0.0	0.0
0.0	0.0	0.0	0.0	0.0	0.0	0.0	0.0
0.0	0.0	0.0	0.0	0.0	0.0	0.0	0.0
0.0	0.0	0.0	0.0	0.0	0.0	0.0	0.0
0.0	0.0	0.0	0.0	0.0	0.0	0.0	0.0
0.0	0.0	0.0	0.0	0.0	0.0	0.0	0.0
0.0	0.0	0.0	0.0	0.0	0.0	0.0	0.0
0.0	0.0	0.0	0.0	0.0	0.0	0.0	0.0
0.0	0.0	0.0	0.0	0.0	0.0	0.0	0.0
0.0	0.0	0.0	0.0	0.0	0.0	0.0	0.0
0.0	0.0	0.0	0.0	0.0	0.0	0.0	0.0
0.0	0.0	0.0	0.0	0.0	0.0	0.0	0.0
0.0	0.0	0.0	0.0	0.0	0.0	0.0	0.0
0.0	0.0	0.0	0.0	0.0	0.0	0.0	0.0
0.0	0.0	0.0	0.0	0.0	0.0	0.0	0.0

0.0	0.0	0.0	0.0	0.0	0.0	0.0	0.0
0.0	0.0	0.0	0.0	0.0	0.0	0.0	0.0
0.0	0.0	0.0	0.0	0.0	0.0	0.0	0.0
0.0	0.0	0.0	0.0	0.0	0.0	0.0	0.0
0.0	0.0	0.0	0.0	0.0	0.0	0.0	0.0
0.0	0.0	0.0	0.0	0.0	0.0	0.0	0.0
0.0	0.0	0.0	0.0	0.0	0.0	0.0	0.0
0.0	0.0	0.0	0.0	0.0	0.0	0.0	0.0
0.0	0.0	0.0	0.0	0.0	0.0	0.0	0.0
0.0	0.0	0.0	0.0	0.0	0.0	0.0	0.0
0.0	0.0	0.0	0.0	0.0	0.0	0.0	0.0
0.0	0.0	0.0	0.0	0.0	0.0	0.0	0.0
0.0	0.0	0.0	0.0	0.0	0.0	0.0	0.0
0.0	0.0	0.0	0.0	0.0	0.0	0.0	0.0
0.0	0.0	0.0	0.0	0.0	0.0	0.0	0.0
0.0	0.0	0.0	0.0	0.0	0.0	0.0	0.0
0.0	0.0	0.0	0.0	0.0	0.0	0.0	0.0
0.0	0.0	0.0	0.0	0.0	0.0	0.0	0.0
0.0	0.0	0.0	0.0	0.0	0.0	0.0	0.0
0.0	0.0	0.0	0.0	0.0	0.0	0.0	0.0
0.0	0.0	0.0	0.0	0.0	0.0	0.0	0.0
0.0	0.0	0.0	0.0	0.0	0.0	0.0	0.0
0.0	0.0	0.0	0.0	0.0	0.0	0.0	0.0
0.0	0.0	0.0	0.0	0.0	0.0	0.0	0.0
0.0	0.0	0.0	0.0	0.0	0.0	0.0	0.0
0.0	0.0	0.0	0.0	0.0	0.0	0.0	0.0
0.0	0.0	0.0	0.0	0.0	0.0	0.0	0.0
0.0	0.0	0.0	0.0	0.0	0.0	0.0	0.0
0.0	0.0	0.0	0.0	0.0	0.0	0.0	0.0
0.0	0.0	0.0	0.0	0.0	0.0	0.0	0.0
0.0	0.0	0.0	0.0	0.0	0.0	0.0	0.0
0.0	0.0	0.0	0.0	0.0	0.0	0.0	0.0
0.0	0.0	0.0	0.0	0.0	0.0	0.0	0.0
0.0	0.0	0.0	0.0	0.0	0.0	0.0	0.0
0.0	0.0	0.0	0.0	0.0	0.0	0.0	0.0
0.0	0.0	0.0	0.0	0.0	0.0	0.0	0.0
0.0	0.0	0.0	0.0	0.0	0.0	0.0	0.0
0.0	0.0	0.0	0.0	0.0	0.0	0.0	0.0
0.0	0.0	0.0	0.0	0.0	0.0	0.0	0.0
0.0	0.0	0.0	0.0	0.0	0.0	0.0	0.0
0.0	0.0	0.0	0.0	0.0	0.0	0.0	0.0
0.0	0.0	0.0	0.0	0.0	0.0	0.0	0.0
0.0	0.0	0.0	0.0	0.0	0.0	0.0	0.0
0.0	0.0	0.0	0.0	0.0	0.0	0.0	0.0
0.0	0.0	0.0	0.0	0.0	0.0	0.0	0.0
0.0	0.0	0.0	0.0	0.0	0.0	0.0	0.0
0.0	0.0	0.0	0.0	0.0	0.0	0.0	0.0

0.0	0.0	0.0	0.0	0.0	0.0	0.0	0.0
0.0	0.0	0.0	0.0	0.0	0.0	0.0	0.0
0.0	0.0	0.0	0.0	0.0	0.0	0.0	0.0
0.0	0.0	0.0	0.0	0.0	0.0	0.0	0.0
0.0	0.0	0.0	0.0	0.0	0.0	0.0	0.0
0.0	0.0	0.0	0.0	0.0	0.0	0.0	0.0
0.0	0.0	0.0	0.0	0.0	0.0	0.0	0.0
0.0	0.0	0.0	0.0	0.0	0.0	0.0	0.0
0.0	0.0	0.0	0.0	0.0	0.0	0.0	0.0
0.0	0.0	0.0	0.0	0.0	0.0	0.0	0.0
0.0	0.0	0.0	0.0	0.0	0.0	0.0	0.0
0.0	0.0	0.0	0.0	0.0	0.0	0.0	0.0
0.0	0.0	0.0	0.0	0.0	0.0	0.0	0.0
0.0	0.0	0.0	0.0	0.0	0.0	0.0	0.0
0.0	0.0	0.0	0.0	0.0	0.0	0.0	0.0
0.0	0.0	0.0	0.0	0.0	0.0	0.0	0.0
0.0	0.0	0.0	0.0	0.0	0.0	0.0	0.0
0.0	0.0	0.0	0.0	0.0	0.0	0.0	0.0
0.0	0.0	0.0	0.0	0.0	0.0	0.0	0.0
0.0	0.0	0.0	0.0	0.0	0.0	0.0	0.0
0.0	0.0	0.0	0.0	0.0	0.0	0.0	0.0
0.0	0.0	0.0	0.0	0.0	0.0	0.0	0.0
0.0	0.0	0.0	0.0	0.0	0.0	0.0	0.0
0.0	0.0	0.0	0.0	0.0	0.0	0.0	0.0
0.0	0.0	0.0	0.0	0.0	0.0	0.0	0.0
0.0	0.0	0.0	0.0	0.0	0.0	0.0	0.0
0.0	0.0	0.0	0.0	0.0	0.0	0.0	0.0
0.0	0.0	0.0	0.0	0.0	0.0	0.0	0.0
0.0	0.0	0.0	0.0	0.0	0.0	0.0	0.0
0.0	0.0	0.0	0.0	0.0	0.0	0.0	0.0
0.0	0.0	0.0	0.0	0.0	0.0	0.0	0.0
0.0	0.0	0.0	0.0	0.0	0.0	0.0	0.0
0.0	0.0	0.0	0.0	0.0	0.0	0.0	0.0
0.0	0.0	0.0	0.0	0.0	0.0	0.0	0.0
0.0	0.0	0.0	0.0	0.0	0.0	0.0	0.0
0.0	0.0	0.0	0.0	0.0	0.0	0.0	0.0
0.0	0.0	0.0	0.0	0.0	0.0	0.0	0.0
0.0	0.0	0.0	0.0	0.0	0.0	0.0	0.0
0.0	0.0	0.0	0.0	0.0	0.0	0.0	0.0
0.0	0.0	0.0	0.0	0.0	0.0	0.0	0.0
0.0	0.0	0.0	0.0	0.0	0.0	0.0	0.0
0.0	0.0	0.0	0.0	0.0	0.0	0.0	0.0
0.0	0.0	0.0	0.0	0.0	0.0	0.0	0.0
0.0	0.0	0.0	0.0	0.0	0.0	0.0	0.0
0.0	0.0	0.0	0.0	0.0	0.0	0.0	0.0
0.0	0.0	0.0	0.0	0.0	0.0	0.0	0.0
0.0	0.0	0.0	0.0	0.0	0.0	0.0	0.0
0.0	0.0	0.0	0.0	0.0	0.0	0.0	0.0

0.0	0.0	0.0	0.0	0.0	0.0	0.0	0.0
0.0	0.0	0.0	0.0	0.0	0.0	0.0	0.0
0.0	0.0	0.0	0.0	0.0	0.0	0.0	0.0
0.0	0.0	0.0	0.0	0.0	0.0	0.0	0.0
0.0	0.0	0.0	0.0	0.0	0.0	0.0	0.0
0.0	0.0	0.0	0.0	0.0	0.0	0.0	0.0
0.0	0.0	0.0	0.0	0.0	0.0	0.0	0.0
0.0	0.0	0.0	0.0	0.0	0.0	0.0	0.0
0.0	0.0	0.0	0.0	0.0	0.0	0.0	0.0
0.0	0.0	0.0	0.0	0.0	0.0	0.0	0.0
0.0	0.0	0.0	0.0	0.0	0.0	0.0	0.0
0.0	0.0	0.0	0.0	0.0	0.0	0.0	0.0
0.0	0.0	0.0	0.0	0.0	0.0	0.0	0.0
0.0	0.0	0.0	0.0	0.0	0.0	0.0	0.0
0.0	0.0	0.0	0.0	0.0	0.0	0.0	0.0
0.0	0.0	0.0	0.0	0.0	0.0	0.0	0.0
0.0	0.0	0.0	0.0	0.0	0.0	0.0	0.0
0.0	0.0	0.0	0.0	0.0	0.0	0.0	0.0
0.0	0.0	0.0	0.0	0.0	0.0	0.0	0.0
0.0	0.0	0.0	0.0	0.0	0.0	0.0	0.0
0.0	0.0	0.0	0.0	0.0	0.0	0.0	0.0
0.0	0.0	0.0	0.0	0.0	0.0	0.0	0.0
0.0	0.0	0.0	0.0	0.0	0.0	0.0	0.0
0.0	0.0	0.0	0.0	0.0	0.0	0.0	0.0
0.0	0.0	0.0	0.0	0.0	0.0	0.0	0.0
0.0	0.0	0.0	0.0	0.0	0.0	0.0	0.0
0.0	0.0	0.0	0.0	0.0	0.0	0.0	0.0
0.0	0.0	0.0	0.0	0.0	0.0	0.0	0.0
0.0	0.0	0.0	0.0	0.0	0.0	0.0	0.0
0.0	0.0	0.0	0.0	0.0	0.0	0.0	0.0
0.0	0.0	0.0	0.0	0.0	0.0	0.0	0.0
0.0	0.0	0.0	0.0	0.0	0.0	0.0	0.0
0.0	0.0	0.0	0.0	0.0	0.0	0.0	0.0
0.0	0.0	0.0	0.0	0.0	0.0	0.0	0.0
0.0	0.0	0.0	0.0	0.0	0.0	0.0	0.0
0.0	0.0	0.0	0.0	0.0	0.0	0.0	0.0
0.0	0.0	0.0	0.0	0.0	0.0	0.0	0.0
0.0	0.0	0.0	0.0	0.0	0.0	0.0	0.0
0.0	0.0	0.0	0.0	0.0	0.0	0.0	0.0
0.0	0.0	0.0	0.0	0.0	0.0	0.0	0.0
0.0	0.0	0.0	0.0	0.0	0.0	0.0	0.0
0.0	0.0	0.0	0.0	0.0	0.0	0.0	0.0
0.0	0.0	0.0	0.0	0.0	0.0	0.0	0.0
0.0	0.0	0.0	0.0	0.0	0.0	0.0	0.0
0.0	0.0	0.0	0.0	0.0	0.0	0.0	0.0
0.0	0.0	0.0	0.0	0.0	0.0	0.0	0.0
0.0	0.0	0.0	0.0	0.0	0.0	0.0	0.0

0.0	0.0	0.0	0.0	0.0	0.0	0.0	0.0
0.0	0.0	0.0	0.0	0.0	0.0	0.0	0.0
0.0	0.0	0.0	0.0	0.0	0.0	0.0	0.0
0.0	0.0	0.0	0.0	0.0	0.0	0.0	0.0
0.0	0.0	0.0	0.0	0.0	0.0	0.0	0.0
0.0	0.0	0.0	0.0	0.0	0.0	0.0	0.0
0.0	0.0	0.0	0.0	0.0	0.0	0.0	0.0
0.0	0.0	0.0	0.0	0.0	0.0	0.0	0.0
0.0	0.0	0.0	0.0	0.0	0.0	0.0	0.0
0.0	0.0	0.0	0.0	0.0	0.0	0.0	0.0
0.0	0.0	0.0	0.0	0.0	0.0	0.0	0.0
0.0	0.0	0.0	0.0	0.0	0.0	0.0	0.0
0.0	0.0	0.0	0.0	0.0	0.0	0.0	0.0
0.0	0.0	0.0	0.0	0.0	0.0	0.0	0.0
0.0	0.0	0.0	0.0	0.0	0.0	0.0	0.0
0.0	0.0	0.0	0.0	0.0	0.0	0.0	0.0
0.0	0.0	0.0	0.0	0.0	0.0	0.0	0.0
0.0	0.0	0.0	0.0	0.0	0.0	0.0	0.0
0.0	0.0	0.0	0.0	0.0	0.0	0.0	0.0
0.0	0.0	0.0	0.0	0.0	0.0	0.0	0.0
0.0	0.0	0.0	0.0	0.0	0.0	0.0	0.0
0.0	0.0	0.0	0.0	0.0	0.0	0.0	0.0
0.0	0.0	0.0	0.0	0.0	0.0	0.0	0.0
0.0	0.0	0.0	0.0	0.0	0.0	0.0	0.0
0.0	0.0	0.0	0.0	0.0	0.0	0.0	0.0
0.0	0.0	0.0	0.0	0.0	0.0	0.0	0.0
0.0	0.0	0.0	0.0	0.0	0.0	0.0	0.0
0.0	0.0	0.0	0.0	0.0	0.0	0.0	0.0
0.0	0.0	0.0	0.0	0.0	0.0	0.0	0.0
0.0	0.0	0.0	0.0	0.0	0.0	0.0	0.0
0.0	0.0	0.0	0.0	0.0	0.0	0.0	0.0
0.0	0.0	0.0	0.0	0.0	0.0	0.0	0.0
0.0	0.0	0.0	0.0	0.0	0.0	0.0	0.0
0.0	0.0	0.0	0.0	0.0	0.0	0.0	0.0
0.0	0.0	0.0	0.0	0.0	0.0	0.0	0.0
0.0	0.0	0.0	0.0	0.0	0.0	0.0	0.0
0.0	0.0	0.0	0.0	0.0	0.0	0.0	0.0
0.0	0.0	0.0	0.0	0.0	0.0	0.0	0.0
0.0	0.0	0.0	0.0	0.0	0.0	0.0	0.0
0.0	0.0	0.0	0.0	0.0	0.0	0.0	0.0
0.0	0.0	0.0	0.0	0.0	0.0	0.0	0.0
0.0	0.0	0.0	0.0	0.0	0.0	0.0	0.0
0.0	0.0	0.0	0.0	0.0	0.0	0.0	0.0
0.0	0.0	0.0	0.0	0.0	0.0	0.0	0.0
0.0	0.0	0.0	0.0	0.0	0.0	0.0	0.0
0.0	0.0	0.0	0.0	0.0	0.0	0.0	0.0
0.0	0.0	0.0	0.0	0.0	0.0	0.0	0.0

0.0	0.0	0.0	0.0	0.0	0.0	0.0	0.0
0.0	0.0	0.0	0.0	0.0	0.0	0.0	0.0
0.0	0.0	0.0	0.0	0.0	0.0	0.0	0.0
0.0	0.0	0.0	0.0	0.0	0.0	0.0	0.0
0.0	0.0	0.0	0.0	0.0	0.0	0.0	0.0
0.0	0.0	0.0	0.0	0.0	0.0	0.0	0.0
0.0	0.0	0.0	0.0	0.0	0.0	0.0	0.0
0.0	0.0	0.0	0.0	0.0	0.0	0.0	0.0
0.0	0.0	0.0	0.0	0.0	0.0	0.0	0.0
0.0	0.0	0.0	0.0	0.0	0.0	0.0	0.0
0.0	0.0	0.0	0.0	0.0	0.0	0.0	0.0
0.0	0.0	0.0	0.0	0.0	0.0	0.0	0.0
0.0	0.0	0.0	0.0	0.0	0.0	0.0	0.0
0.0	0.0	0.0	0.0	0.0	0.0	0.0	0.0
0.0	0.0	0.0	0.0	0.0	0.0	0.0	0.0
0.0	0.0	0.0	0.0	0.0	0.0	0.0	0.0
0.0	0.0	0.0	0.0	0.0	0.0	0.0	0.0
0.0	0.0	0.0	0.0	0.0	0.0	0.0	0.0
0.0	0.0	0.0	0.0	0.0	0.0	0.0	0.0
0.0	0.0	0.0	0.0	0.0	0.0	0.0	0.0
0.0	0.0	0.0	0.0	0.0	0.0	0.0	0.0
0.0	0.0	0.0	0.0	0.0	0.0	0.0	0.0
0.0	0.0	0.0	0.0	0.0	0.0	0.0	0.0
0.0	0.0	0.0	0.0	0.0	0.0	0.0	0.0
0.0	0.0	0.0	0.0	0.0	0.0	0.0	0.0
0.0	0.0	0.0	0.0	0.0	0.0	0.0	0.0
0.0	0.0	0.0	0.0	0.0	0.0	0.0	0.0
0.0	0.0	0.0	0.0	0.0	0.0	0.0	0.0
0.0	0.0	0.0	0.0	0.0	0.0	0.0	0.0
0.0	0.0	0.0	0.0	0.0	0.0	0.0	0.0
0.0	0.0	0.0	0.0	0.0	0.0	0.0	0.0
0.0	0.0	0.0	0.0	0.0	0.0	0.0	0.0
0.0	0.0	0.0	0.0	0.0	0.0	0.0	0.0
0.0	0.0	0.0	0.0	0.0	0.0	0.0	0.0
0.0	0.0	0.0	0.0	0.0	0.0	0.0	0.0
0.0	0.0	0.0	0.0	0.0	0.0	0.0	0.0
0.0	0.0	0.0	0.0	0.0	0.0	0.0	0.0
0.0	0.0	0.0	0.0	0.0	0.0	0.0	0.0
0.0	0.0	0.0	0.0	0.0	0.0	0.0	0.0
0.0	0.0	0.0	0.0	0.0	0.0	0.0	0.0
0.0	0.0	0.0	0.0	0.0	0.0	0.0	0.0
0.0	0.0	0.0	0.0	0.0	0.0	0.0	0.0
0.0	0.0	0.0	0.0	0.0	0.0	0.0	0.0
0.0	0.0	0.0	0.0	0.0	0.0	0.0	0.0
0.0	0.0	0.0	0.0	0.0	0.0	0.0	0.0
0.0	0.0	0.0	0.0	0.0	0.0	0.0	0.0
0.0	0.0	0.0	0.0	0.0	0.0	0.0	0.0

0.0	0.0	0.0	0.0	0.0	0.0	0.0	0.0
0.0	0.0	0.0	0.0	0.0	0.0	0.0	0.0
0.0	0.0	0.0	0.0	0.0	0.0	0.0	0.0
0.0	0.0	0.0	0.0	0.0	0.0	0.0	0.0
0.0	0.0	0.0	0.0	0.0	0.0	0.0	0.0
0.0	0.0	0.0	0.0	0.0	0.0	0.0	0.0
0.0	0.0	0.0	0.0	0.0	0.0	0.0	0.0
0.0	0.0	0.0	0.0	0.0	0.0	0.0	0.0
0.0	0.0	0.0	0.0	0.0	0.0	0.0	0.0
0.0	0.0	0.0	0.0	0.0	0.0	0.0	0.0
0.0	0.0	0.0	0.0	0.0	0.0	0.0	0.0
0.0	0.0	0.0	0.0	0.0	0.0	0.0	0.0
0.0	0.0	0.0	0.0	0.0	0.0	0.0	0.0
0.0	0.0	0.0	0.0	0.0	0.0	0.0	0.0
0.0	0.0	0.0	0.0	0.0	0.0	0.0	0.0
0.0	0.0	0.0	0.0	0.0	0.0	0.0	0.0
0.0	0.0	0.0	0.0	0.0	0.0	0.0	0.0
0.0	0.0	0.0	0.0	0.0	0.0	0.0	0.0
0.0	0.0	0.0	0.0	0.0	0.0	0.0	0.0
0.0	0.0	0.0	0.0	0.0	0.0	0.0	0.0
0.0	0.0	0.0	0.0	0.0	0.0	0.0	0.0
0.0	0.0	0.0	0.0	0.0	0.0	0.0	0.0
0.0	0.0	0.0	0.0	0.0	0.0	0.0	0.0
0.0	0.0	0.0	0.0	0.0	0.0	0.0	0.0
0.0	0.0	0.0	0.0	0.0	0.0	0.0	0.0
0.0	0.0	0.0	0.0	0.0	0.0	0.0	0.0
0.0	0.0	0.0	0.0	0.0	0.0	0.0	0.0
0.0	0.0	0.0	0.0	0.0	0.0	0.0	0.0
0.0	0.0	0.0	0.0	0.0	0.0	0.0	0.0
0.0	0.0	0.0	0.0	0.0	0.0	0.0	0.0
0.0	0.0	0.0	0.0	0.0	0.0	0.0	0.0
0.0	0.0	0.0	0.0	0.0	0.0	0.0	0.0
0.0	0.0	0.0	0.0	0.0	0.0	0.0	0.0
0.0	0.0	0.0	0.0	0.0	0.0	0.0	0.0
0.0	0.0	0.0	0.0	0.0	0.0	0.0	0.0
0.0	0.0	0.0	0.0	0.0	0.0	0.0	0.0
0.0	0.0	0.0	0.0	0.0	0.0	0.0	0.0
0.0	0.0	0.0	0.0	0.0	0.0	0.0	0.0
0.0	0.0	0.0	0.0	0.0	0.0	0.0	0.0
0.0	0.0	0.0	0.0	0.0	0.0	0.0	0.0
0.0	0.0	0.0	0.0	0.0	0.0	0.0	0.0
0.0	0.0	0.0	0.0	0.0	0.0	0.0	0.0
0.0	0.0	0.0	0.0	0.0	0.0	0.0	0.0
0.0	0.0	0.0	0.0	0.0	0.0	0.0	0.0
0.0	0.0	0.0	0.0	0.0	0.0	0.0	0.0
0.0	0.0	0.0	0.0	0.0	0.0	0.0	0.0
0.0	0.0	0.0	0.0	0.0	0.0	0.0	0.0

0.0	0.0	0.0	0.0	0.0	0.0	0.0	0.0
0.0	0.0	0.0	0.0	0.0	0.0	0.0	0.0
0.0	0.0	0.0	0.0	0.0	0.0	0.0	0.0
0.0	0.0	0.0	0.0	0.0	0.0	0.0	0.0
0.0	0.0	0.0	0.0	0.0	0.0	0.0	0.0
0.0	0.0	0.0	0.0	0.0	0.0	0.0	0.0
0.0	0.0	0.0	0.0	0.0	0.0	0.0	0.0
0.0	0.0	0.0	0.0	0.0	0.0	0.0	0.0
0.0	0.0	0.0	0.0	0.0	0.0	0.0	0.0
0.0	0.0	0.0	0.0	0.0	0.0	0.0	0.0
0.0	0.0	0.0	0.0	0.0	0.0	0.0	0.0
0.0	0.0	0.0	0.0	0.0	0.0	0.0	0.0
0.0	0.0	0.0	0.0	0.0	0.0	0.0	0.0
0.0	0.0	0.0	0.0	0.0	0.0	0.0	0.0
0.0	0.0	0.0	0.0	0.0	0.0	0.0	0.0
0.0	0.0	0.0	0.0	0.0	0.0	0.0	0.0
0.0	0.0	0.0	0.0	0.0	0.0	0.0	0.0
0.0	0.0	0.0	0.0	0.0	0.0	0.0	0.0
0.0	0.0	0.0	0.0	0.0	0.0	0.0	0.0
0.0	0.0	0.0	0.0	0.0	0.0	0.0	0.0
0.0	0.0	0.0	0.0	0.0	0.0	0.0	0.0
0.0	0.0	0.0	0.0	0.0	0.0	0.0	0.0
0.0	0.0	0.0	0.0	0.0	0.0	0.0	0.0
0.0	0.0	0.0	0.0	0.0	0.0	0.0	0.0
0.0	0.0	0.0	0.0	0.0	0.0	0.0	0.0
0.0	0.0	0.0	0.0	0.0	0.0	0.0	0.0
0.0	0.0	0.0	0.0	0.0	0.0	0.0	0.0
0.0	0.0	0.0	0.0	0.0	0.0	0.0	0.0
0.0	0.0	0.0	0.0	0.0	0.0	0.0	0.0
0.0	0.0	0.0	0.0	0.0	0.0	0.0	0.0
0.0	0.0	0.0	0.0	0.0	0.0	0.0	0.0
0.0	0.0	0.0	0.0	0.0	0.0	0.0	0.0
0.0	0.0	0.0	0.0	0.0	0.0	0.0	0.0
0.0	0.0	0.0	0.0	0.0	0.0	0.0	0.0
0.0	0.0	0.0	0.0	0.0	0.0	0.0	0.0
0.0	0.0	0.0	0.0	0.0	0.0	0.0	0.0
0.0	0.0	0.0	0.0	0.0	0.0	0.0	0.0
0.0	0.0	0.0	0.0	0.0	0.0	0.0	0.0
0.0	0.0	0.0	0.0	0.0	0.0	0.0	0.0
0.0	0.0	0.0	0.0	0.0	0.0	0.0	0.0
0.0	0.0	0.0	0.0	0.0	0.0	0.0	0.0
0.0	0.0	0.0	0.0	0.0	0.0	0.0	0.0
0.0	0.0	0.0	0.0	0.0	0.0	0.0	0.0
0.0	0.0	0.0	0.0	0.0	0.0	0.0	0.0
0.0	0.0	0.0	0.0	0.0	0.0	0.0	0.0
0.0	0.0	0.0	0.0	0.0	0.0	0.0	0.0
0.0	0.0	0.0	0.0	0.0	0.0	0.0	0.0

0.0	0.0	0.0	0.0	0.0	0.0	0.0	0.0
0.0	0.0	0.0	0.0	0.0	0.0	0.0	0.0
0.0	0.0	0.0	0.0	0.0	0.0	0.0	0.0
0.0	0.0	0.0	0.0	0.0	0.0	0.0	0.0
0.0	0.0	0.0	0.0	0.0	0.0	0.0	0.0
0.0	0.0	0.0	0.0	0.0	0.0	0.0	0.0
0.0	0.0	0.0	0.0	0.0	0.0	0.0	0.0
0.0	0.0	0.0	0.0	0.0	0.0	0.0	0.0
0.0	0.0	0.0	0.0	0.0	0.0	0.0	0.0
0.0	0.0	0.0	0.0	0.0	0.0	0.0	0.0
0.0	0.0	0.0	0.0	0.0	0.0	0.0	0.0
0.0	0.0	0.0	0.0	0.0	0.0	0.0	0.0
0.0	0.0	0.0	0.0	0.0	0.0	0.0	0.0
0.0	0.0	0.0	0.0	0.0	0.0	0.0	0.0
0.0	0.0	0.0	0.0	0.0	0.0	0.0	0.0
0.0	0.0	0.0	0.0	0.0	0.0	0.0	0.0
0.0	0.0	0.0	0.0	0.0	0.0	0.0	0.0
0.0	0.0	0.0	0.0	0.0	0.0	0.0	0.0
0.0	0.0	0.0	0.0	0.0	0.0	0.0	0.0
0.0	0.0	0.0	0.0	0.0	0.0	0.0	0.0
0.0	0.0	0.0	0.0	0.0	0.0	0.0	0.0
0.0	0.0	0.0	0.0	0.0	0.0	0.0	0.0
0.0	0.0	0.0	0.0	0.0	0.0	0.0	0.0
0.0	0.0	0.0	0.0	0.0	0.0	0.0	0.0
0.0	0.0	0.0	0.0	0.0	0.0	0.0	0.0
0.0	0.0	0.0	0.0	0.0	0.0	0.0	0.0
0.0	0.0	0.0	0.0	0.0	0.0	0.0	0.0
0.0	0.0	0.0	0.0	0.0	0.0	0.0	0.0
0.0	0.0	0.0	0.0	0.0	0.0	0.0	0.0
0.0	0.0	0.0	0.0	0.0	0.0	0.0	0.0
0.0	0.0	0.0	0.0	0.0	0.0	0.0	0.0
0.0	0.0	0.0	0.0	0.0	0.0	0.0	0.0
0.0	0.0	0.0	0.0	0.0	0.0	0.0	0.0
0.0	0.0	0.0	0.0	0.0	0.0	0.0	0.0
0.0	0.0	0.0	0.0	0.0	0.0	0.0	0.0
0.0	0.0	0.0	0.0	0.0	0.0	0.0	0.0
0.0	0.0	0.0	0.0	0.0	0.0	0.0	0.0
0.0	0.0	0.0	0.0	0.0	0.0	0.0	0.0
0.0	0.0	0.0	0.0	0.0	0.0	0.0	0.0
0.0	0.0	0.0	0.0	0.0	0.0	0.0	0.0
0.0	0.0	0.0	0.0	0.0	0.0	0.0	0.0
0.0	0.0	0.0	0.0	0.0	0.0	0.0	0.0
0.0	0.0	0.0	0.0	0.0	0.0	0.0	0.0
0.0	0.0	0.0	0.0	0.0	0.0	0.0	0.0
0.0	0.0	0.0	0.0	0.0	0.0	0.0	0.0
0.0	0.0	0.0	0.0	0.0	0.0	0.0	0.0
0.0	0.0	0.0	0.0	0.0	0.0	0.0	0.0

0.0	0.0	0.0	0.0	0.0	0.0	0.0	0.0
0.0	0.0	0.0	0.0	0.0	0.0	0.0	0.0
0.0	0.0	0.0	0.0	0.0	0.0	0.0	0.0
0.0	0.0	0.0	0.0	0.0	0.0	0.0	0.0
0.0	0.0	0.0	0.0	0.0	0.0	0.0	0.0
0.0	0.0	0.0	0.0	0.0	0.0	0.0	0.0
0.0	0.0	0.0	0.0	0.0	0.0	0.0	0.0
0.0	0.0	0.0	0.0	0.0	0.0	0.0	0.0
0.0	0.0	0.0	0.0	0.0	0.0	0.0	0.0
0.0	0.0	0.0	0.0	0.0	0.0	0.0	0.0
0.0	0.0	0.0	0.0	0.0	0.0	0.0	0.0
0.0	0.0	0.0	0.0	0.0	0.0	0.0	0.0
0.0	0.0	0.0	0.0	0.0	0.0	0.0	0.0
0.0	0.0	0.0	0.0	0.0	0.0	0.0	0.0
0.0	0.0	0.0	0.0	0.0	0.0	0.0	0.0
0.0	0.0	0.0	0.0	0.0	0.0	0.0	0.0
0.0	0.0	0.0	0.0	0.0	0.0	0.0	0.0
0.0	0.0	0.0	0.0	0.0	0.0	0.0	0.0
0.0	0.0	0.0	0.0	0.0	0.0	0.0	0.0
0.0	0.0	0.0	0.0	0.0	0.0	0.0	0.0
0.0	0.0	0.0	0.0	0.0	0.0	0.0	0.0
0.0	0.0	0.0	0.0	0.0	0.0	0.0	0.0
0.0	0.0	0.0	0.0	0.0	0.0	0.0	0.0
0.0	0.0	0.0	0.0	0.0	0.0	0.0	0.0
0.0	0.0	0.0	0.0	0.0	0.0	0.0	0.0
0.0	0.0	0.0	0.0	0.0	0.0	0.0	0.0
0.0	0.0	0.0	0.0	0.0	0.0	0.0	0.0
0.0	0.0	0.0	0.0	0.0	0.0	0.0	0.0
0.0	0.0	0.0	0.0	0.0	0.0	0.0	0.0
0.0	0.0	0.0	0.0	0.0	0.0	0.0	0.0
0.0	0.0	0.0	0.0	0.0	0.0	0.0	0.0
0.0	0.0	0.0	0.0	0.0	0.0	0.0	0.0
0.0	0.0	0.0	0.0	0.0	0.0	0.0	0.0
0.0	0.0	0.0	0.0	0.0	0.0	0.0	0.0
0.0	0.0	0.0	0.0	0.0	0.0	0.0	0.0
0.0	0.0	0.0	0.0	0.0	0.0	0.0	0.0
0.0	0.0	0.0	0.0	0.0	0.0	0.0	0.0
0.0	0.0	0.0	0.0	0.0	0.0	0.0	0.0
0.0	0.0	0.0	0.0	0.0	0.0	0.0	0.0
0.0	0.0	0.0	0.0	0.0	0.0	0.0	0.0
0.0	0.0	0.0	0.0	0.0	0.0	0.0	0.0
0.0	0.0	0.0	0.0	0.0	0.0	0.0	0.0
0.0	0.0	0.0	0.0	0.0	0.0	0.0	0.0
0.0	0.0	0.0	0.0	0.0	0.0	0.0	0.0
0.0	0.0	0.0	0.0	0.0	0.0	0.0	0.0
0.0	0.0	0.0	0.0	0.0	0.0	0.0	0.0
0.0	0.0	0.0	0.0	0.0	0.0	0.0	0.0

0.0	0.0	0.0	0.0	0.0	0.0	0.0	0.0
0.0	0.0	0.0	0.0	0.0	0.0	0.0	0.0
0.0	0.0	0.0	0.0	0.0	0.0	0.0	0.0
0.0	0.0	0.0	0.0	0.0	0.0	0.0	0.0
0.0	0.0	0.0	0.0	0.0	0.0	0.0	0.0
0.0	0.0	0.0	0.0	0.0	0.0	0.0	0.0
0.0	0.0	0.0	0.0	0.0	0.0	0.0	0.0
0.0	0.0	0.0	0.0	0.0	0.0	0.0	0.0
0.0	0.0	0.0	0.0	0.0	0.0	0.0	0.0
0.0	0.0	0.0	0.0	0.0	0.0	0.0	0.0
0.0	0.0	0.0	0.0	0.0	0.0	0.0	0.0
0.0	0.0	0.0	0.0	0.0	0.0	0.0	0.0
0.0	0.0	0.0	0.0	0.0	0.0	0.0	0.0
0.0	0.0	0.0	0.0	0.0	0.0	0.0	0.0
0.0	0.0	0.0	0.0	0.0	0.0	0.0	0.0
0.0	0.0	0.0	0.0	0.0	0.0	0.0	0.0
0.0	0.0	0.0	0.0	0.0	0.0	0.0	0.0
0.0	0.0	0.0	0.0	0.0	0.0	0.0	0.0
0.0	0.0	0.0	0.0	0.0	0.0	0.0	0.0
0.0	0.0	0.0	0.0	0.0	0.0	0.0	0.0
0.0	0.0	0.0	0.0	0.0	0.0	0.0	0.0
0.0	0.0	0.0	0.0	0.0	0.0	0.0	0.0
0.0	0.0	0.0	0.0	0.0	0.0	0.0	0.0
0.0	0.0	0.0	0.0	0.0	0.0	0.0	0.0
0.0	0.0	0.0	0.0	0.0	0.0	0.0	0.0
0.0	0.0	0.0	0.0	0.0	0.0	0.0	0.0
0.0	0.0	0.0	0.0	0.0	0.0	0.0	0.0
0.0	0.0	0.0	0.0	0.0	0.0	0.0	0.0
0.0	0.0	0.0	0.0	0.0	0.0	0.0	0.0
0.0	0.0	0.0	0.0	0.0	0.0	0.0	0.0
0.0	0.0	0.0	0.0	0.0	0.0	0.0	0.0
0.0	0.0	0.0	0.0	0.0	0.0	0.0	0.0
0.0	0.0	0.0	0.0	0.0	0.0	0.0	0.0
0.0	0.0	0.0	0.0	0.0	0.0	0.0	0.0
0.0	0.0	0.0	0.0	0.0	0.0	0.0	0.0
0.0	0.0	0.0	0.0	0.0	0.0	0.0	0.0
0.0	0.0	0.0	0.0	0.0	0.0	0.0	0.0
0.0	0.0	0.0	0.0	0.0	0.0	0.0	0.0
0.0	0.0	0.0	0.0	0.0	0.0	0.0	0.0
0.0	0.0	0.0	0.0	0.0	0.0	0.0	0.0
0.0	0.0	0.0	0.0	0.0	0.0	0.0	0.0
0.0	0.0	0.0	0.0	0.0	0.0	0.0	0.0
0.0	0.0	0.0	0.0	0.0	0.0	0.0	0.0
0.0	0.0	0.0	0.0	0.0	0.0	0.0	0.0
0.0	0.0	0.0	0.0	0.0	0.0	0.0	0.0
0.0	0.0	0.0	0.0	0.0	0.0	0.0	0.0
0.0	0.0	0.0	0.0	0.0	0.0	0.0	0.0

0.0	0.0	0.0	0.0	0.0	0.0	0.0	0.0
0.0	0.0	0.0	0.0	0.0	0.0	0.0	0.0
0.0	0.0	0.0	0.0	0.0	0.0	0.0	0.0
0.0	0.0	0.0	0.0	0.0	0.0	0.0	0.0
0.0	0.0	0.0	0.0	0.0	0.0	0.0	0.0
0.0	0.0	0.0	0.0	0.0	0.0	0.0	0.0
0.0	0.0	0.0	0.0	0.0	0.0	0.0	0.0
0.0	0.0	0.0	0.0	0.0	0.0	0.0	0.0
0.0	0.0	0.0	0.0	0.0	0.0	0.0	0.0
0.0	0.0	0.0	0.0	0.0	0.0	0.0	0.0
0.0	0.0	0.0	0.0	0.0	0.0	0.0	0.0
0.0	0.0	0.0	0.0	0.0	0.0	0.0	0.0
0.0	0.0	0.0	0.0	0.0	0.0	0.0	0.0
0.0	0.0	0.0	0.0	0.0	0.0	0.0	0.0
0.0	0.0	0.0	0.0	0.0	0.0	0.0	0.0
0.0	0.0	0.0	0.0	0.0	0.0	0.0	0.0
0.0	0.0	0.0	0.0	0.0	0.0	0.0	0.0
0.0	0.0	0.0	0.0	0.0	0.0	0.0	0.0
0.0	0.0	0.0	0.0	0.0	0.0	0.0	0.0
0.0	0.0	0.0	0.0	0.0	0.0	0.0	0.0
0.0	0.0	0.0	0.0	0.0	0.0	0.0	0.0
0.0	0.0	0.0	0.0	0.0	0.0	0.0	0.0
0.0	0.0	0.0	0.0	0.0	0.0	0.0	0.0
0.0	0.0	0.0	0.0	0.0	0.0	0.0	0.0
0.0	0.0	0.0	0.0	0.0	0.0	0.0	0.0
0.0	0.0	0.0	0.0	0.0	0.0	0.0	0.0
0.0	0.0	0.0	0.0	0.0	0.0	0.0	0.0
0.0	0.0	0.0	0.0	0.0	0.0	0.0	0.0
0.0	0.0	0.0	0.0	0.0	0.0	0.0	0.0
0.0	0.0	0.0	0.0	0.0	0.0	0.0	0.0
0.0	0.0	0.0	0.0	0.0	0.0	0.0	0.0
0.0	0.0	0.0	0.0	0.0	0.0	0.0	0.0
0.0	0.0	0.0	0.0	0.0	0.0	0.0	0.0
0.0	0.0	0.0	0.0	0.0	0.0	0.0	0.0
0.0	0.0	0.0	0.0	0.0	0.0	0.0	0.0
0.0	0.0	0.0	0.0	0.0	0.0	0.0	0.0
0.0	0.0	0.0	0.0	0.0	0.0	0.0	0.0
0.0	0.0	0.0	0.0	0.0	0.0	0.0	0.0
0.0	0.0	0.0	0.0	0.0	0.0	0.0	0.0
0.0	0.0	0.0	0.0	0.0	0.0	0.0	0.0
0.0	0.0	0.0	0.0	0.0	0.0	0.0	0.0
0.0	0.0	0.0	0.0	0.0	0.0	0.0	0.0
0.0	0.0	0.0	0.0	0.0	0.0	0.0	0.0
0.0	0.0	0.0	0.0	0.0	0.0	0.0	0.0
0.0	0.0	0.0	0.0	0.0	0.0	0.0	0.0
0.0	0.0	0.0	0.0	0.0	0.0	0.0	0.0
0.0	0.0	0.0	0.0	0.0	0.0	0.0	0.0

0.0	0.0	0.0	0.0	0.0	0.0	0.0	0.0
0.0	0.0	0.0	0.0	0.0	0.0	0.0	0.0
0.0	0.0	0.0	0.0	0.0	0.0	0.0	0.0
0.0	0.0	0.0	0.0	0.0	0.0	0.0	0.0
0.0	0.0	0.0	0.0	0.0	0.0	0.0	0.0
0.0	0.0	0.0	0.0	0.0	0.0	0.0	0.0
0.0	0.0	0.0	0.0	0.0	0.0	0.0	0.0
0.0	0.0	0.0	0.0	0.0	0.0	0.0	0.0
0.0	0.0	0.0	0.0	0.0	0.0	0.0	0.0
0.0	0.0	0.0	0.0	0.0	0.0	0.0	0.0
0.0	0.0	0.0	0.0	0.0	0.0	0.0	0.0
0.0	0.0	0.0	0.0	0.0	0.0	0.0	0.0
0.0	0.0	0.0	0.0	0.0	0.0	0.0	0.0
0.0	0.0	0.0	0.0	0.0	0.0	0.0	0.0
0.0	0.0	0.0	0.0	0.0	0.0	0.0	0.0
0.0	0.0	0.0	0.0	0.0	0.0	0.0	0.0
0.0	0.0	0.0	0.0	0.0	0.0	0.0	0.0
0.0	0.0	0.0	0.0	0.0	0.0	0.0	0.0
0.0	0.0	0.0	0.0	0.0	0.0	0.0	0.0
0.0	0.0	0.0	0.0	0.0	0.0	0.0	0.0
0.0	0.0	0.0	0.0	0.0	0.0	0.0	0.0
0.0	0.0	0.0	0.0	0.0	0.0	0.0	0.0
0.0	0.0	0.0	0.0	0.0	0.0	0.0	0.0
0.0	0.0	0.0	0.0	0.0	0.0	0.0	0.0
0.0	0.0	0.0	0.0	0.0	0.0	0.0	0.0
0.0	0.0	0.0	0.0	0.0	0.0	0.0	0.0
0.0	0.0	0.0	0.0	0.0	0.0	0.0	0.0
0.0	0.0	0.0	0.0	0.0	0.0	0.0	0.0
0.0	0.0	0.0	0.0	0.0	0.0	0.0	0.0
0.0	0.0	0.0	0.0	0.0	0.0	0.0	0.0
0.0	0.0	0.0	0.0	0.0	0.0	0.0	0.0
0.0	0.0	0.0	0.0	0.0	0.0	0.0	0.0
0.0	0.0	0.0	0.0	0.0	0.0	0.0	0.0
0.0	0.0	0.0	0.0	0.0	0.0	0.0	0.0
0.0	0.0	0.0	0.0	0.0	0.0	0.0	0.0
0.0	0.0	0.0	0.0	0.0	0.0	0.0	0.0
0.0	0.0	0.0	0.0	0.0	0.0	0.0	0.0
0.0	0.0	0.0	0.0	0.0	0.0	0.0	0.0
0.0	0.0	0.0	0.0	0.0	0.0	0.0	0.0
0.0	0.0	0.0	0.0	0.0	0.0	0.0	0.0
0.0	0.0	0.0	0.0	0.0	0.0	0.0	0.0
0.0	0.0	0.0	0.0	0.0	0.0	0.0	0.0
0.0	0.0	0.0	0.0	0.0	0.0	0.0	0.0
0.0	0.0	0.0	0.0	0.0	0.0	0.0	0.0
0.0	0.0	0.0	0.0	0.0	0.0	0.0	0.0
0.0	0.0	0.0	0.0	0.0	0.0	0.0	0.0
0.0	0.0	0.0	0.0	0.0	0.0	0.0	0.0

0.0	0.0	0.0	0.0	0.0	0.0	0.0	0.0
0.0	0.0	0.0	0.0	0.0	0.0	0.0	0.0
0.0	0.0	0.0	0.0	0.0	0.0	0.0	0.0
0.0	0.0	0.0	0.0	0.0	0.0	0.0	0.0
0.0	0.0	0.0	0.0	0.0	0.0	0.0	0.0
0.0	0.0	0.0	0.0	0.0	0.0	0.0	0.0
0.0	0.0	0.0	0.0	0.0	0.0	0.0	0.0
0.0	0.0	0.0	0.0	0.0	0.0	0.0	0.0
0.0	0.0	0.0	0.0	0.0	0.0	0.0	0.0
0.0	0.0	0.0	0.0	0.0	0.0	0.0	0.0
0.0	0.0	0.0	0.0	0.0	0.0	0.0	0.0
0.0	0.0	0.0	0.0	0.0	0.0	0.0	0.0
0.0	0.0	0.0	0.0	0.0	0.0	0.0	0.0
0.0	0.0	0.0	0.0	0.0	0.0	0.0	0.0
0.0	0.0	0.0	0.0	0.0	0.0	0.0	0.0
0.0	0.0	0.0	0.0	0.0	0.0	0.0	0.0
0.0	0.0	0.0	0.0	0.0	0.0	0.0	0.0
0.0	0.0	0.0	0.0	0.0	0.0	0.0	0.0
0.0	0.0	0.0	0.0	0.0	0.0	0.0	0.0
0.0	0.0	0.0	0.0	0.0	0.0	0.0	0.0
0.0	0.0	0.0	0.0	0.0	0.0	0.0	0.0
0.0	0.0	0.0	0.0	0.0	0.0	0.0	0.0
0.0	0.0	0.0	0.0	0.0	0.0	0.0	0.0
0.0	0.0	0.0	0.0	0.0	0.0	0.0	0.0
0.0	0.0	0.0	0.0	0.0	0.0	0.0	0.0
0.0	0.0	0.0	0.0	0.0	0.0	0.0	0.0
0.0	0.0	0.0	0.0	0.0	0.0	0.0	0.0
0.0	0.0	0.0	0.0	0.0	0.0	0.0	0.0
0.0	0.0	0.0	0.0	0.0	0.0	0.0	0.0
0.0	0.0	0.0	0.0	0.0	0.0	0.0	0.0
0.0	0.0	0.0	0.0	0.0	0.0	0.0	0.0
0.0	0.0	0.0	0.0	0.0	0.0	0.0	0.0
0.0	0.0	0.0	0.0	0.0	0.0	0.0	0.0
0.0	0.0	0.0	0.0	0.0	0.0	0.0	0.0
0.0	0.0	0.0	0.0	0.0	0.0	0.0	0.0
0.0	0.0	0.0	0.0	0.0	0.0	0.0	0.0
0.0	0.0	0.0	0.0	0.0	0.0	0.0	0.0
0.0	0.0	0.0	0.0	0.0	0.0	0.0	0.0
0.0	0.0	0.0	0.0	0.0	0.0	0.0	0.0
0.0	0.0	0.0	0.0	0.0	0.0	0.0	0.0
0.0	0.0	0.0	0.0	0.0	0.0	0.0	0.0
0.0	0.0	0.0	0.0	0.0	0.0	0.0	0.0
0.0	0.0	0.0	0.0	0.0	0.0	0.0	0.0
0.0	0.0	0.0	0.0	0.0	0.0	0.0	0.0
0.0	0.0	0.0	0.0	0.0	0.0	0.0	0.0
0.0	0.0	0.0	0.0	0.0	0.0	0.0	0.0
0.0	0.0	0.0	0.0	0.0	0.0	0.0	0.0
0.0	0.0	0.0	0.0	0.0	0.0	0.0	0.0

0.0	0.0	0.0	0.0	0.0	0.0	0.0	0.0
0.0	0.0	0.0	0.0	0.0	0.0	0.0	0.0
0.0	0.0	0.0	0.0	0.0	0.0	0.0	0.0
0.0	0.0	0.0	0.0	0.0	0.0	0.0	0.0
0.0	0.0	0.0	0.0	0.0	0.0	0.0	0.0
0.0	0.0	0.0	0.0	0.0	0.0	0.0	0.0
0.0	0.0	0.0	0.0	0.0	0.0	0.0	0.0
0.0	0.0	0.0	0.0	0.0	0.0	0.0	0.0
0.0	0.0	0.0	0.0	0.0	0.0	0.0	0.0
0.0	0.0	0.0	0.0	0.0	0.0	0.0	0.0
0.0	0.0	0.0	0.0	0.0	0.0	0.0	0.0
0.0	0.0	0.0	0.0	0.0	0.0	0.0	0.0
0.0	0.0	0.0	0.0	0.0	0.0	0.0	0.0
0.0	0.0	0.0	0.0	0.0	0.0	0.0	0.0
0.0	0.0	0.0	0.0	0.0	0.0	0.0	0.0
0.0	0.0	0.0	0.0	0.0	0.0	0.0	0.0
0.0	0.0	0.0	0.0	0.0	0.0	0.0	0.0
0.0	0.0	0.0	0.0	0.0	0.0	0.0	0.0
0.0	0.0	0.0	0.0	0.0	0.0	0.0	0.0
0.0	0.0	0.0	0.0	0.0	0.0	0.0	0.0
0.0	0.0	0.0	0.0	0.0	0.0	0.0	0.0
0.0	0.0	0.0	0.0	0.0	0.0	0.0	0.0
0.0	0.0	0.0	0.0	0.0	0.0	0.0	0.0
0.0	0.0	0.0	0.0	0.0	0.0	0.0	0.0
0.0	0.0	0.0	0.0	0.0	0.0	0.0	0.0
0.0	0.0	0.0	0.0	0.0	0.0	0.0	0.0
0.0	0.0	0.0	0.0	0.0	0.0	0.0	0.0
0.0	0.0	0.0	0.0	0.0	0.0	0.0	0.0
0.0	0.0	0.0	0.0	0.0	0.0	0.0	0.0
0.0	0.0	0.0	0.0	0.0	0.0	0.0	0.0
0.0	0.0	0.0	0.0	0.0	0.0	0.0	0.0
0.0	0.0	0.0	0.0	0.0	0.0	0.0	0.0
0.0	0.0	0.0	0.0	0.0	0.0	0.0	0.0
0.0	0.0	0.0	0.0	0.0	0.0	0.0	0.0
0.0	0.0	0.0	0.0	0.0	0.0	0.0	0.0
0.0	0.0	0.0	0.0	0.0	0.0	0.0	0.0
0.0	0.0	0.0	0.0	0.0	0.0	0.0	0.0
0.0	0.0	0.0	0.0	0.0	0.0	0.0	0.0
0.0	0.0	0.0	0.0	0.0	0.0	0.0	0.0
0.0	0.0	0.0	0.0	0.0	0.0	0.0	0.0
0.0	0.0	0.0	0.0	0.0	0.0	0.0	0.0
0.0	0.0	0.0	0.0	0.0	0.0	0.0	0.0
0.0	0.0	0.0	0.0	0.0	0.0	0.0	0.0
0.0	0.0	0.0	0.0	0.0	0.0	0.0	0.0
0.0	0.0	0.0	0.0	0.0	0.0	0.0	0.0
0.0	0.0	0.0	0.0	0.0	0.0	0.0	0.0
0.0	0.0	0.0	0.0	0.0	0.0	0.0	0.0
0.0	0.0	0.0	0.0	0.0	0.0	0.0	0.0

0.0	0.0	0.0	0.0	0.0	0.0	0.0	0.0
0.0	0.0	0.0	0.0	0.0	0.0	0.0	0.0
0.0	0.0	0.0	0.0	0.0	0.0	0.0	0.0
0.0	0.0	0.0	0.0	0.0	0.0	0.0	0.0
0.0	0.0	0.0	0.0	0.0	0.0	0.0	0.0
0.0	0.0	0.0	0.0	0.0	0.0	0.0	0.0
0.0	0.0	0.0	0.0	0.0	0.0	0.0	0.0
0.0	0.0	0.0	0.0	0.0	0.0	0.0	0.0
0.0	0.0	0.0	0.0	0.0	0.0	0.0	0.0
0.0	0.0	0.0	0.0	0.0	0.0	0.0	0.0
0.0	0.0	0.0	0.0	0.0	0.0	0.0	0.0
0.0	0.0	0.0	0.0	0.0	0.0	0.0	0.0
0.0	0.0	0.0	0.0	0.0	0.0	0.0	0.0
0.0	0.0	0.0	0.0	0.0	0.0	0.0	0.0
0.0	0.0	0.0	0.0	0.0	0.0	0.0	0.0
0.0	0.0	0.0	0.0	0.0	0.0	0.0	0.0
0.0	0.0	0.0	0.0	0.0	0.0	0.0	0.0
0.0	0.0	0.0	0.0	0.0	0.0	0.0	0.0
0.0	0.0	0.0	0.0	0.0	0.0	0.0	0.0
0.0	0.0	0.0	0.0	0.0	0.0	0.0	0.0
0.0	0.0	0.0	0.0	0.0	0.0	0.0	0.0
0.0	0.0	0.0	0.0	0.0	0.0	0.0	0.0
0.0	0.0	0.0	0.0	0.0	0.0	0.0	0.0
0.0	0.0	0.0	0.0	0.0	0.0	0.0	0.0
0.0	0.0	0.0	0.0	0.0	0.0	0.0	0.0
0.0	0.0	0.0	0.0	0.0	0.0	0.0	0.0
0.0	0.0	0.0	0.0	0.0	0.0	0.0	0.0
0.0	0.0	0.0	0.0	0.0	0.0	0.0	0.0
0.0	0.0	0.0	0.0	0.0	0.0	0.0	0.0
0.0	0.0	0.0	0.0	0.0	0.0	0.0	0.0
0.0	0.0	0.0	0.0	0.0	0.0	0.0	0.0
0.0	0.0	0.0	0.0	0.0	0.0	0.0	0.0
0.0	0.0	0.0	0.0	0.0	0.0	0.0	0.0
0.0	0.0	0.0	0.0	0.0	0.0	0.0	0.0
0.0	0.0	0.0	0.0	0.0	0.0	0.0	0.0
0.0	0.0	0.0	0.0	0.0	0.0	0.0	0.0
0.0	0.0	0.0	0.0	0.0	0.0	0.0	0.0
0.0	0.0	0.0	0.0	0.0	0.0	0.0	0.0
0.0	0.0	0.0	0.0	0.0	0.0	0.0	0.0
0.0	0.0	0.0	0.0	0.0	0.0	0.0	0.0
0.0	0.0	0.0	0.0	0.0	0.0	0.0	0.0
0.0	0.0	0.0	0.0	0.0	0.0	0.0	0.0
0.0	0.0	0.0	0.0	0.0	0.0	0.0	0.0
0.0	0.0	0.0	0.0	0.0	0.0	0.0	0.0
0.0	0.0	0.0	0.0	0.0	0.0	0.0	0.0
0.0	0.0	0.0	0.0	0.0	0.0	0.0	0.0
0.0	0.0	0.0	0.0	0.0	0.0	0.0	0.0

0.0	0.0	0.0	0.0	0.0	0.0	0.0	0.0
0.0	0.0	0.0	0.0	0.0	0.0	0.0	0.0
0.0	0.0	0.0	0.0	0.0	0.0	0.0	0.0
0.0	0.0	0.0	0.0	0.0	0.0	0.0	0.0
0.0	0.0	0.0	0.0	0.0	0.0	0.0	0.0
0.0	0.0	0.0	0.0	0.0	0.0	0.0	0.0
0.0	0.0	0.0	0.0	0.0	0.0	0.0	0.0
0.0	0.0	0.0	0.0	0.0	0.0	0.0	0.0
0.0	0.0	0.0	0.0	0.0	0.0	0.0	0.0
0.0	0.0	0.0	0.0	0.0	0.0	0.0	0.0
0.0	0.0	0.0	0.0	0.0	0.0	0.0	0.0
0.0	0.0	0.0	0.0	0.0	0.0	0.0	0.0
0.0	0.0	0.0	0.0	0.0	0.0	0.0	0.0
0.0	0.0	0.0	0.0	0.0	0.0	0.0	0.0
0.0	0.0	0.0	0.0	0.0	0.0	0.0	0.0
0.0	0.0	0.0	0.0	0.0	0.0	0.0	0.0
0.0	0.0	0.0	0.0	0.0	0.0	0.0	0.0
0.0	0.0	0.0	0.0	0.0	0.0	0.0	0.0
0.0	0.0	0.0	0.0	0.0	0.0	0.0	0.0
0.0	0.0	0.0	0.0	0.0	0.0	0.0	0.0
0.0	0.0	0.0	0.0	0.0	0.0	0.0	0.0
0.0	0.0	0.0	0.0	0.0	0.0	0.0	0.0
0.0	0.0	0.0	0.0	0.0	0.0	0.0	0.0
0.0	0.0	0.0	0.0	0.0	0.0	0.0	0.0
0.0	0.0	0.0	0.0	0.0	0.0	0.0	0.0
0.0	0.0	0.0	0.0	0.0	0.0	0.0	0.0
0.0	0.0	0.0	0.0	0.0	0.0	0.0	0.0
0.0	0.0	0.0	0.0	0.0	0.0	0.0	0.0
0.0	0.0	0.0	0.0	0.0	0.0	0.0	0.0
0.0	0.0	0.0	0.0	0.0	0.0	0.0	0.0
0.0	0.0	0.0	0.0	0.0	0.0	0.0	0.0
0.0	0.0	0.0	0.0	0.0	0.0	0.0	0.0
0.0	0.0	0.0	0.0	0.0	0.0	0.0	0.0
0.0	0.0	0.0	0.0	0.0	0.0	0.0	0.0
0.0	0.0	0.0	0.0	0.0	0.0	0.0	0.0
0.0	0.0	0.0	0.0	0.0	0.0	0.0	0.0
0.0	0.0	0.0	0.0	0.0	0.0	0.0	0.0
0.0	0.0	0.0	0.0	0.0	0.0	0.0	0.0
0.0	0.0	0.0	0.0	0.0	0.0	0.0	0.0
0.0	0.0	0.0	0.0	0.0	0.0	0.0	0.0
0.0	0.0	0.0	0.0	0.0	0.0	0.0	0.0
0.0	0.0	0.0	0.0	0.0	0.0	0.0	0.0
0.0	0.0	0.0	0.0	0.0	0.0	0.0	0.0
0.0	0.0	0.0	0.0	0.0	0.0	0.0	0.0
0.0	0.0	0.0	0.0	0.0	0.0	0.0	0.0
0.0	0.0	0.0	0.0	0.0	0.0	0.0	0.0
0.0	0.0	0.0	0.0	0.0	0.0	0.0	0.0
0.0	0.0	0.0	0.0	0.0	0.0	0.0	0.0

0.0	0.0	0.0	0.0	0.0	0.0	0.0	0.0
0.0	0.0	0.0	0.0	0.0	0.0	0.0	0.0
0.0	0.0	0.0	0.0	0.0	0.0	0.0	0.0
0.0	0.0	0.0	0.0	0.0	0.0	0.0	0.0
0.0	0.0	0.0	0.0	0.0	0.0	0.0	0.0
0.0	0.0	0.0	0.0	0.0	0.0	0.0	0.0
0.0	0.0	0.0	0.0	0.0	0.0	0.0	0.0
0.0	0.0	0.0	0.0	0.0	0.0	0.0	0.0
0.0	0.0	0.0	0.0	0.0	0.0	0.0	0.0
0.0	0.0	0.0	0.0	0.0	0.0	0.0	0.0
0.0	0.0	0.0	0.0	0.0	0.0	0.0	0.0
0.0	0.0	0.0	0.0	0.0	0.0	0.0	0.0
0.0	0.0	0.0	0.0	0.0	0.0	0.0	0.0
0.0	0.0	0.0	0.0	0.0	0.0	0.0	0.0
0.0	0.0	0.0	0.0	0.0	0.0	0.0	0.0
0.0	0.0	0.0	0.0	0.0	0.0	0.0	0.0
0.0	0.0	0.0	0.0	0.0	0.0	0.0	0.0
0.0	0.0	0.0	0.0	0.0	0.0	0.0	0.0
0.0	0.0	0.0	0.0	0.0	0.0	0.0	0.0
0.0	0.0	0.0	0.0	0.0	0.0	0.0	0.0
0.0	0.0	0.0	0.0	0.0	0.0	0.0	0.0
0.0	0.0	0.0	0.0	0.0	0.0	0.0	0.0
0.0	0.0	0.0	0.0	0.0	0.0	0.0	0.0
0.0	0.0	0.0	0.0	0.0	0.0	0.0	0.0
0.0	0.0	0.0	0.0	0.0	0.0	0.0	0.0
0.0	0.0	0.0	0.0	0.0	0.0	0.0	0.0
0.0	0.0	0.0	0.0	0.0	0.0	0.0	0.0
0.0	0.0	0.0	0.0	0.0	0.0	0.0	0.0
0.0	0.0	0.0	0.0	0.0	0.0	0.0	0.0
0.0	0.0	0.0	0.0	0.0	0.0	0.0	0.0
0.0	0.0	0.0	0.0	0.0	0.0	0.0	0.0
0.0	0.0	0.0	0.0	0.0	0.0	0.0	0.0
0.0	0.0	0.0	0.0	0.0	0.0	0.0	0.0
0.0	0.0	0.0	0.0	0.0	0.0	0.0	0.0
0.0	0.0	0.0	0.0	0.0	0.0	0.0	0.0
0.0	0.0	0.0	0.0	0.0	0.0	0.0	0.0
0.0	0.0	0.0	0.0	0.0	0.0	0.0	0.0
0.0	0.0	0.0	0.0	0.0	0.0	0.0	0.0
0.0	0.0	0.0	0.0	0.0	0.0	0.0	0.0
0.0	0.0	0.0	0.0	0.0	0.0	0.0	0.0
0.0	0.0	0.0	0.0	0.0	0.0	0.0	0.0
0.0	0.0	0.0	0.0	0.0	0.0	0.0	0.0
0.0	0.0	0.0	0.0	0.0	0.0	0.0	0.0
0.0	0.0	0.0	0.0	0.0	0.0	0.0	0.0
0.0	0.0	0.0	0.0	0.0	0.0	0.0	0.0
0.0	0.0	0.0	0.0	0.0	0.0	0.0	0.0
0.0	0.0	0.0	0.0	0.0	0.0	0.0	0.0
0.0	0.0	0.0	0.0	0.0	0.0	0.0	0.0

0.0	0.0	0.0	0.0	0.0	0.0	0.0	0.0
0.0	0.0	0.0	0.0	0.0	0.0	0.0	0.0
0.0	0.0	0.0	0.0	0.0	0.0	0.0	0.0
0.0	0.0	0.0	0.0	0.0	0.0	0.0	0.0
0.0	0.0	0.0	0.0	0.0	0.0	0.0	0.0
0.0	0.0	0.0	0.0	0.0	0.0	0.0	0.0
0.0	0.0	0.0	0.0	0.0	0.0	0.0	0.0
0.0	0.0	0.0	0.0	0.0	0.0	0.0	0.0
0.0	0.0	0.0	0.0	0.0	0.0	0.0	0.0
0.0	0.0	0.0	0.0	0.0	0.0	0.0	0.0
0.0	0.0	0.0	0.0	0.0	0.0	0.0	0.0
0.0	0.0	0.0	0.0	0.0	0.0	0.0	0.0
0.0	0.0	0.0	0.0	0.0	0.0	0.0	0.0
0.0	0.0	0.0	0.0	0.0	0.0	0.0	0.0
0.0	0.0	0.0	0.0	0.0	0.0	0.0	0.0
0.0	0.0	0.0	0.0	0.0	0.0	0.0	0.0
0.0	0.0	0.0	0.0	0.0	0.0	0.0	0.0
0.0	0.0	0.0	0.0	0.0	0.0	0.0	0.0
0.0	0.0	0.0	0.0	0.0	0.0	0.0	0.0
0.0	0.0	0.0	0.0	0.0	0.0	0.0	0.0
0.0	0.0	0.0	0.0	0.0	0.0	0.0	0.0
0.0	0.0	0.0	0.0	0.0	0.0	0.0	0.0
0.0	0.0	0.0	0.0	0.0	0.0	0.0	0.0
0.0	0.0	0.0	0.0	0.0	0.0	0.0	0.0
0.0	0.0	0.0	0.0	0.0	0.0	0.0	0.0
0.0	0.0	0.0	0.0	0.0	0.0	0.0	0.0
0.0	0.0	0.0	0.0	0.0	0.0	0.0	0.0
0.0	0.0	0.0	0.0	0.0	0.0	0.0	0.0
0.0	0.0	0.0	0.0	0.0	0.0	0.0	0.0
0.0	0.0	0.0	0.0	0.0	0.0	0.0	0.0
0.0	0.0	0.0	0.0	0.0	0.0	0.0	0.0
0.0	0.0	0.0	0.0	0.0	0.0	0.0	0.0
0.0	0.0	0.0	0.0	0.0	0.0	0.0	0.0
0.0	0.0	0.0	0.0	0.0	0.0	0.0	0.0
0.0	0.0	0.0	0.0	0.0	0.0	0.0	0.0
0.0	0.0	0.0	0.0	0.0	0.0	0.0	0.0
0.0	0.0	0.0	0.0	0.0	0.0	0.0	0.0
0.0	0.0	0.0	0.0	0.0	0.0	0.0	0.0
0.0	0.0	0.0	0.0	0.0	0.0	0.0	0.0
0.0	0.0	0.0	0.0	0.0	0.0	0.0	0.0
0.0	0.0	0.0	0.0	0.0	0.0	0.0	0.0
0.0	0.0	0.0	0.0	0.0	0.0	0.0	0.0
0.0	0.0	0.0	0.0	0.0	0.0	0.0	0.0
0.0	0.0	0.0	0.0	0.0	0.0	0.0	0.0
0.0	0.0	0.0	0.0	0.0	0.0	0.0	0.0
0.0	0.0	0.0	0.0	0.0	0.0	0.0	0.0
0.0	0.0	0.0	0.0	0.0	0.0	0.0	0.0

0.0	0.0	0.0	0.0	0.0	0.0	0.0	0.0
0.0	0.0	0.0	0.0	0.0	0.0	0.0	0.0
0.0	0.0	0.0	0.0	0.0	0.0	0.0	0.0
0.0	0.0	0.0	0.0	0.0	0.0	0.0	0.0
0.0	0.0	0.0	0.0	0.0	0.0	0.0	0.0
0.0	0.0	0.0	0.0	0.0	0.0	0.0	0.0
0.0	0.0	0.0	0.0	0.0	0.0	0.0	0.0
0.0	0.0	0.0	0.0	0.0	0.0	0.0	0.0
0.0	0.0	0.0	0.0	0.0	0.0	0.0	0.0
0.0	0.0	0.0	0.0	0.0	0.0	0.0	0.0
0.0	0.0	0.0	0.0	0.0	0.0	0.0	0.0
0.0	0.0	0.0	0.0	0.0	0.0	0.0	0.0
0.0	0.0	0.0	0.0	0.0	0.0	0.0	0.0
0.0	0.0	0.0	0.0	0.0	0.0	0.0	0.0
0.0	0.0	0.0	0.0	0.0	0.0	0.0	0.0
0.0	0.0	0.0	0.0	0.0	0.0	0.0	0.0
0.0	0.0	0.0	0.0	0.0	0.0	0.0	0.0
0.0	0.0	0.0	0.0	0.0	0.0	0.0	0.0
0.0	0.0	0.0	0.0	0.0	0.0	0.0	0.0
0.0	0.0	0.0	0.0	0.0	0.0	0.0	0.0
0.0	0.0	0.0	0.0	0.0	0.0	0.0	0.0
0.0	0.0	0.0	0.0	0.0	0.0	0.0	0.0
0.0	0.0	0.0	0.0	0.0	0.0	0.0	0.0
0.0	0.0	0.0	0.0	0.0	0.0	0.0	0.0
0.0	0.0	0.0	0.0	0.0	0.0	0.0	0.0
0.0	0.0	0.0	0.0	0.0	0.0	0.0	0.0
0.0	0.0	0.0	0.0	0.0	0.0	0.0	0.0
0.0	0.0	0.0	0.0	0.0	0.0	0.0	0.0
0.0	0.0	0.0	0.0	0.0	0.0	0.0	0.0
0.0	0.0	0.0	0.0	0.0	0.0	0.0	0.0
0.0	0.0	0.0	0.0	0.0	0.0	0.0	0.0
0.0	0.0	0.0	0.0	0.0	0.0	0.0	0.0
0.0	0.0	0.0	0.0	0.0	0.0	0.0	0.0
0.0	0.0	0.0	0.0	0.0	0.0	0.0	0.0
0.0	0.0	0.0	0.0	0.0	0.0	0.0	0.0
0.0	0.0	0.0	0.0	0.0	0.0	0.0	0.0
0.0	0.0	0.0	0.0	0.0	0.0	0.0	0.0
0.0	0.0	0.0	0.0	0.0	0.0	0.0	0.0
0.0	0.0	0.0	0.0	0.0	0.0	0.0	0.0
0.0	0.0	0.0	0.0	0.0	0.0	0.0	0.0
0.0	0.0	0.0	0.0	0.0	0.0	0.0	0.0
0.0	0.0	0.0	0.0	0.0	0.0	0.0	0.0
0.0	0.0	0.0	0.0	0.0	0.0	0.0	0.0
0.0	0.0	0.0	0.0	0.0	0.0	0.0	0.0
0.0	0.0	0.0	0.0	0.0	0.0	0.0	0.0
0.0	0.0	0.0	0.0	0.0	0.0	0.0	0.0
0.0	0.0	0.0	0.0	0.0	0.0	0.0	0.0

0.0	0.0	0.0	0.0	0.0	0.0	0.0	0.0
0.0	0.0	0.0	0.0	0.0	0.0	0.0	0.0
0.0	0.0	0.0	0.0	0.0	0.0	0.0	0.0
0.0	0.0	0.0	0.0	0.0	0.0	0.0	0.0
0.0	0.0	0.0	0.0	0.0	0.0	0.0	0.0
0.0	0.0	0.0	0.0	0.0	0.0	0.0	0.0
0.0	0.0	0.0	0.0	0.0	0.0	0.0	0.0
0.0	0.0	0.0	0.0	0.0	0.0	0.0	0.0
0.0	0.0	0.0	0.0	0.0	0.0	0.0	0.0
0.0	0.0	0.0	0.0	0.0	0.0	0.0	0.0
0.0	0.0	0.0	0.0	0.0	0.0	0.0	0.0
0.0	0.0	0.0	0.0	0.0	0.0	0.0	0.0
0.0	0.0	0.0	0.0	0.0	0.0	0.0	0.0
0.0	0.0	0.0	0.0	0.0	0.0	0.0	0.0
0.0	0.0	0.0	0.0	0.0	0.0	0.0	0.0
0.0	0.0	0.0	0.0	0.0	0.0	0.0	0.0
0.0	0.0	0.0	0.0	0.0	0.0	0.0	0.0
0.0	0.0	0.0	0.0	0.0	0.0	0.0	0.0
0.0	0.0	0.0	0.0	0.0	0.0	0.0	0.0
0.0	0.0	0.0	0.0	0.0	0.0	0.0	0.0
0.0	0.0	0.0	0.0	0.0	0.0	0.0	0.0
0.0	0.0	0.0	0.0	0.0	0.0	0.0	0.0
0.0	0.0	0.0	0.0	0.0	0.0	0.0	0.0
0.0	0.0	0.0	0.0	0.0	0.0	0.0	0.0
0.0	0.0	0.0	0.0	0.0	0.0	0.0	0.0
0.0	0.0	0.0	0.0	0.0	0.0	0.0	0.0
0.0	0.0	0.0	0.0	0.0	0.0	0.0	0.0
0.0	0.0	0.0	0.0	0.0	0.0	0.0	0.0
0.0	0.0	0.0	0.0	0.0	0.0	0.0	0.0
0.0	0.0	0.0	0.0	0.0	0.0	0.0	0.0
0.0	0.0	0.0	0.0	0.0	0.0	0.0	0.0
0.0	0.0	0.0	0.0	0.0	0.0	0.0	0.0
0.0	0.0	0.0	0.0	0.0	0.0	0.0	0.0
0.0	0.0	0.0	0.0	0.0	0.0	0.0	0.0
0.0	0.0	0.0	0.0	0.0	0.0	0.0	0.0
0.0	0.0	0.0	0.0	0.0	0.0	0.0	0.0
0.0	0.0	0.0	0.0	0.0	0.0	0.0	0.0
0.0	0.0	0.0	0.0	0.0	0.0	0.0	0.0
0.0	0.0	0.0	0.0	0.0	0.0	0.0	0.0
0.0	0.0	0.0	0.0	0.0	0.0	0.0	0.0
0.0	0.0	0.0	0.0	0.0	0.0	0.0	0.0
0.0	0.0	0.0	0.0	0.0	0.0	0.0	0.0
0.0	0.0	0.0	0.0	0.0	0.0	0.0	0.0
0.0	0.0	0.0	0.0	0.0	0.0	0.0	0.0
0.0	0.0	0.0	0.0	0.0	0.0	0.0	0.0
0.0	0.0	0.0	0.0	0.0	0.0	0.0	0.0
0.0	0.0	0.0	0.0	0.0	0.0	0.0	0.0

0.0	0.0	0.0	0.0	0.0	0.0	0.0	0.0
0.0	0.0	0.0	0.0	0.0	0.0	0.0	0.0
0.0	0.0	0.0	0.0	0.0	0.0	0.0	0.0
0.0	0.0	0.0	0.0	0.0	0.0	0.0	0.0
0.0	0.0	0.0	0.0	0.0	0.0	0.0	0.0
0.0	0.0	0.0	0.0	0.0	0.0	0.0	0.0
0.0	0.0	0.0	0.0	0.0	0.0	0.0	0.0
0.0	0.0	0.0	0.0	0.0	0.0	0.0	0.0
0.0	0.0	0.0	0.0	0.0	0.0	0.0	0.0
0.0	0.0	0.0	0.0	0.0	0.0	0.0	0.0
0.0	0.0	0.0	0.0	0.0	0.0	0.0	0.0
0.0	0.0	0.0	0.0	0.0	0.0	0.0	0.0
0.0	0.0	0.0	0.0	0.0	0.0	0.0	0.0
0.0	0.0	0.0	0.0	0.0	0.0	0.0	0.0
0.0	0.0	0.0	0.0	0.0	0.0	0.0	0.0
0.0	0.0	0.0	0.0	0.0	0.0	0.0	0.0
0.0	0.0	0.0	0.0	0.0	0.0	0.0	0.0
0.0	0.0	0.0	0.0	0.0	0.0	0.0	0.0
0.0	0.0	0.0	0.0	0.0	0.0	0.0	0.0
0.0	0.0	0.0	0.0	0.0	0.0	0.0	0.0
0.0	0.0	0.0	0.0	0.0	0.0	0.0	0.0
0.0	0.0	0.0	0.0	0.0	0.0	0.0	0.0
0.0	0.0	0.0	0.0	0.0	0.0	0.0	0.0
0.0	0.0	0.0	0.0	0.0	0.0	0.0	0.0
0.0	0.0	0.0	0.0	0.0	0.0	0.0	0.0
0.0	0.0	0.0	0.0	0.0	0.0	0.0	0.0
0.0	0.0	0.0	0.0	0.0	0.0	0.0	0.0
0.0	0.0	0.0	0.0	0.0	0.0	0.0	0.0
0.0	0.0	0.0	0.0	0.0	0.0	0.0	0.0
0.0	0.0	0.0	0.0	0.0	0.0	0.0	0.0
0.0	0.0	0.0	0.0	0.0	0.0	0.0	0.0
0.0	0.0	0.0	0.0	0.0	0.0	0.0	0.0
0.0	0.0	0.0	0.0	0.0	0.0	0.0	0.0
0.0	0.0	0.0	0.0	0.0	0.0	0.0	0.0
0.0	0.0	0.0	0.0	0.0	0.0	0.0	0.0
0.0	0.0	0.0	0.0	0.0	0.0	0.0	0.0
0.0	0.0	0.0	0.0	0.0	0.0	0.0	0.0
0.0	0.0	0.0	0.0	0.0	0.0	0.0	0.0
0.0	0.0	0.0	0.0	0.0	0.0	0.0	0.0
0.0	0.0	0.0	0.0	0.0	0.0	0.0	0.0
0.0	0.0	0.0	0.0	0.0	0.0	0.0	0.0
0.0	0.0	0.0	0.0	0.0	0.0	0.0	0.0
0.0	0.0	0.0	0.0	0.0	0.0	0.0	0.0
0.0	0.0	0.0	0.0	0.0	0.0	0.0	0.0
0.0	0.0	0.0	0.0	0.0	0.0	0.0	0.0
0.0	0.0	0.0	0.0	0.0	0.0	0.0	0.0
0.0	0.0	0.0	0.0	0.0	0.0	0.0	0.0
0.0	0.0	0.0	0.0	0.0	0.0	0.0	0.0

0.0	0.0	0.0	0.0	0.0	0.0	0.0	0.0
0.0	0.0	0.0	0.0	0.0	0.0	0.0	0.0
0.0	0.0	0.0	0.0	0.0	0.0	0.0	0.0
0.0	0.0	0.0	0.0	0.0	0.0	0.0	0.0
0.0	0.0	0.0	0.0	0.0	0.0	0.0	0.0
0.0	0.0	0.0	0.0	0.0	0.0	0.0	0.0
0.0	0.0	0.0	0.0	0.0	0.0	0.0	0.0
0.0	0.0	0.0	0.0	0.0	0.0	0.0	0.0
0.0	0.0	0.0	0.0	0.0	0.0	0.0	0.0
0.0	0.0	0.0	0.0	0.0	0.0	0.0	0.0
0.0	0.0	0.0	0.0	0.0	0.0	0.0	0.0
0.0	0.0	0.0	0.0	0.0	0.0	0.0	0.0
0.0	0.0	0.0	0.0	0.0	0.0	0.0	0.0
0.0	0.0	0.0	0.0	0.0	0.0	0.0	0.0
0.0	0.0	0.0	0.0	0.0	0.0	0.0	0.0
0.0	0.0	0.0	0.0	0.0	0.0	0.0	0.0
0.0	0.0	0.0	0.0	0.0	0.0	0.0	0.0
0.0	0.0	0.0	0.0	0.0	0.0	0.0	0.0
0.0	0.0	0.0	0.0	0.0	0.0	0.0	0.0
0.0	0.0	0.0	0.0	0.0	0.0	0.0	0.0
0.0	0.0	0.0	0.0	0.0	0.0	0.0	0.0
0.0	0.0	0.0	0.0	0.0	0.0	0.0	0.0
0.0	0.0	0.0	0.0	0.0	0.0	0.0	0.0
0.0	0.0	0.0	0.0	0.0	0.0	0.0	0.0
0.0	0.0	0.0	0.0	0.0	0.0	0.0	0.0
0.0	0.0	0.0	0.0	0.0	0.0	0.0	0.0
0.0	0.0	0.0	0.0	0.0	0.0	0.0	0.0
0.0	0.0	0.0	0.0	0.0	0.0	0.0	0.0
0.0	0.0	0.0	0.0	0.0	0.0	0.0	0.0
0.0	0.0	0.0	0.0	0.0	0.0	0.0	0.0
0.0	0.0	0.0	0.0	0.0	0.0	0.0	0.0
0.0	0.0	0.0	0.0	0.0	0.0	0.0	0.0
0.0	0.0	0.0	0.0	0.0	0.0	0.0	0.0
0.0	0.0	0.0	0.0	0.0	0.0	0.0	0.0
0.0	0.0	0.0	0.0	0.0	0.0	0.0	0.0
0.0	0.0	0.0	0.0	0.0	0.0	0.0	0.0
0.0	0.0	0.0	0.0	0.0	0.0	0.0	0.0
0.0	0.0	0.0	0.0	0.0	0.0	0.0	0.0
0.0	0.0	0.0	0.0	0.0	0.0	0.0	0.0
0.0	0.0	0.0	0.0	0.0	0.0	0.0	0.0
0.0	0.0	0.0	0.0	0.0	0.0	0.0	0.0
0.0	0.0	0.0	0.0	0.0	0.0	0.0	0.0
0.0	0.0	0.0	0.0	0.0	0.0	0.0	0.0
0.0	0.0	0.0	0.0	0.0	0.0	0.0	0.0
0.0	0.0	0.0	0.0	0.0	0.0	0.0	0.0
0.0	0.0	0.0	0.0	0.0	0.0	0.0	0.0
0.0	0.0	0.0	0.0	0.0	0.0	0.0	0.0
0.0	0.0	0.0	0.0	0.0	0.0	0.0	0.0

0.0	0.0	0.0	0.0	0.0	0.0	0.0	0.0
0.0	0.0	0.0	0.0	0.0	0.0	0.0	0.0
0.0	0.0	0.0	0.0	0.0	0.0	0.0	0.0
0.0	0.0	0.0	0.0	0.0	0.0	0.0	0.0
0.0	0.0	0.0	0.0	0.0	0.0	0.0	0.0
0.0	0.0	0.0	0.0	0.0	0.0	0.0	0.0
0.0	0.0	0.0	0.0	0.0	0.0	0.0	0.0
0.0	0.0	0.0	0.0	0.0	0.0	0.0	0.0
0.0	0.0	0.0	0.0	0.0	0.0	0.0	0.0
0.0	0.0	0.0	0.0	0.0	0.0	0.0	0.0
0.0	0.0	0.0	0.0	0.0	0.0	0.0	0.0
0.0	0.0	0.0	0.0	0.0	0.0	0.0	0.0
0.0	0.0	0.0	0.0	0.0	0.0	0.0	0.0
0.0	0.0	0.0	0.0	0.0	0.0	0.0	0.0
0.0	0.0	0.0	0.0	0.0	0.0	0.0	0.0
0.0	0.0	0.0	0.0	0.0	0.0	0.0	0.0
0.0	0.0	0.0	0.0	0.0	0.0	0.0	0.0
0.0	0.0	0.0	0.0	0.0	0.0	0.0	0.0
0.0	0.0	0.0	0.0	0.0	0.0	0.0	0.0
0.0	0.0	0.0	0.0	0.0	0.0	0.0	0.0
0.0	0.0	0.0	0.0	0.0	0.0	0.0	0.0
0.0	0.0	0.0	0.0	0.0	0.0	0.0	0.0
0.0	0.0	0.0	0.0	0.0	0.0	0.0	0.0
0.0	0.0	0.0	0.0	0.0	0.0	0.0	0.0
0.0	0.0	0.0	0.0	0.0	0.0	0.0	0.0
0.0	0.0	0.0	0.0	0.0	0.0	0.0	0.0
0.0	0.0	0.0	0.0	0.0	0.0	0.0	0.0
0.0	0.0	0.0	0.0	0.0	0.0	0.0	0.0
0.0	0.0	0.0	0.0	0.0	0.0	0.0	0.0
0.0	0.0	0.0	0.0	0.0	0.0	0.0	0.0
0.0	0.0	0.0	0.0	0.0	0.0	0.0	0.0
0.0	0.0	0.0	0.0	0.0	0.0	0.0	0.0
0.0	0.0	0.0	0.0	0.0	0.0	0.0	0.0
0.0	0.0	0.0	0.0	0.0	0.0	0.0	0.0
0.0	0.0	0.0	0.0	0.0	0.0	0.0	0.0
0.0	0.0	0.0	0.0	0.0	0.0	0.0	0.0
0.0	0.0	0.0	0.0	0.0	0.0	0.0	0.0
0.0	0.0	0.0	0.0	0.0	0.0	0.0	0.0
0.0	0.0	0.0	0.0	0.0	0.0	0.0	0.0
0.0	0.0	0.0	0.0	0.0	0.0	0.0	0.0
0.0	0.0	0.0	0.0	0.0	0.0	0.0	0.0
0.0	0.0	0.0	0.0	0.0	0.0	0.0	0.0
0.0	0.0	0.0	0.0	0.0	0.0	0.0	0.0
0.0	0.0	0.0	0.0	0.0	0.0	0.0	0.0
0.0	0.0	0.0	0.0	0.0	0.0	0.0	0.0
0.0	0.0	0.0	0.0	0.0	0.0	0.0	0.0
0.0	0.0	0.0	0.0	0.0	0.0	0.0	0.0
0.0	0.0	0.0	0.0	0.0	0.0	0.0	0.0

0.0	0.0	0.0	0.0	0.0	0.0	0.0	0.0
0.0	0.0	0.0	0.0	0.0	0.0	0.0	0.0
0.0	0.0	0.0	0.0	0.0	0.0	0.0	0.0
0.0	0.0	0.0	0.0	0.0	0.0	0.0	0.0
0.0	0.0	0.0	0.0	0.0	0.0	0.0	0.0
0.0	0.0	0.0	0.0	0.0	0.0	0.0	0.0
0.0	0.0	0.0	0.0	0.0	0.0	0.0	0.0
0.0	0.0	0.0	0.0	0.0	0.0	0.0	0.0
0.0	0.0	0.0	0.0	0.0	0.0	0.0	0.0
0.0	0.0	0.0	0.0	0.0	0.0	0.0	0.0
0.0	0.0	0.0	0.0	0.0	0.0	0.0	0.0
0.0	0.0	0.0	0.0	0.0	0.0	0.0	0.0
0.0	0.0	0.0	0.0	0.0	0.0	0.0	0.0
0.0	0.0	0.0	0.0	0.0	0.0	0.0	0.0
0.0	0.0	0.0	0.0	0.0	0.0	0.0	0.0
0.0	0.0	0.0	0.0	0.0	0.0	0.0	0.0
0.0	0.0	0.0	0.0	0.0	0.0	0.0	0.0
0.0	0.0	0.0	0.0	0.0	0.0	0.0	0.0
0.0	0.0	0.0	0.0	0.0	0.0	0.0	0.0
0.0	0.0	0.0	0.0	0.0	0.0	0.0	0.0
0.0	0.0	0.0	0.0	0.0	0.0	0.0	0.0
0.0	0.0	0.0	0.0	0.0	0.0	0.0	0.0
0.0	0.0	0.0	0.0	0.0	0.0	0.0	0.0
0.0	0.0	0.0	0.0	0.0	0.0	0.0	0.0
0.0	0.0	0.0	0.0	0.0	0.0	0.0	0.0
0.0	0.0	0.0	0.0	0.0	0.0	0.0	0.0
0.0	0.0	0.0	0.0	0.0	0.0	0.0	0.0
0.0	0.0	0.0	0.0	0.0	0.0	0.0	0.0
0.0	0.0	0.0	0.0	0.0	0.0	0.0	0.0
0.0	0.0	0.0	0.0	0.0	0.0	0.0	0.0
0.0	0.0	0.0	0.0	0.0	0.0	0.0	0.0
0.0	0.0	0.0	0.0	0.0	0.0	0.0	0.0
0.0	0.0	0.0	0.0	0.0	0.0	0.0	0.0
0.0	0.0	0.0	0.0	0.0	0.0	0.0	0.0
0.0	0.0	0.0	0.0	0.0	0.0	0.0	0.0
0.0	0.0	0.0	0.0	0.0	0.0	0.0	0.0
0.0	0.0	0.0	0.0	0.0	0.0	0.0	0.0
0.0	0.0	0.0	0.0	0.0	0.0	0.0	0.0
0.0	0.0	0.0	0.0	0.0	0.0	0.0	0.0
0.0	0.0	0.0	0.0	0.0	0.0	0.0	0.0
0.0	0.0	0.0	0.0	0.0	0.0	0.0	0.0
0.0	0.0	0.0	0.0	0.0	0.0	0.0	0.0
0.0	0.0	0.0	0.0	0.0	0.0	0.0	0.0
0.0	0.0	0.0	0.0	0.0	0.0	0.0	0.0
0.0	0.0	0.0	0.0	0.0	0.0	0.0	0.0
0.0	0.0	0.0	0.0	0.0	0.0	0.0	0.0
0.0	0.0	0.0	0.0	0.0	0.0	0.0	0.0
0.0	0.0	0.0	0.0	0.0	0.0	0.0	0.0

0.0	0.0	0.0	0.0	0.0	0.0	0.0	0.0
0.0	0.0	0.0	0.0	0.0	0.0	0.0	0.0
0.0	0.0	0.0	0.0	0.0	0.0	0.0	0.0
0.0	0.0	0.0	0.0	0.0	0.0	0.0	0.0
0.0	0.0	0.0	0.0	0.0	0.0	0.0	0.0
0.0	0.0	0.0	0.0	0.0	0.0	0.0	0.0
0.0	0.0	0.0	0.0	0.0	0.0	0.0	0.0
0.0	0.0	0.0	0.0	0.0	0.0	0.0	0.0
0.0	0.0	0.0	0.0	0.0	0.0	0.0	0.0
0.0	0.0	0.0	0.0	0.0	0.0	0.0	0.0
0.0	0.0	0.0	0.0	0.0	0.0	0.0	0.0
0.0	0.0	0.0	0.0	0.0	0.0	0.0	0.0
0.0	0.0	0.0	0.0	0.0	0.0	0.0	0.0
0.0	0.0	0.0	0.0	0.0	0.0	0.0	0.0
0.0	0.0	0.0	0.0	0.0	0.0	0.0	0.0
0.0	0.0	0.0	0.0	0.0	0.0	0.0	0.0
0.0	0.0	0.0	0.0	0.0	0.0	0.0	0.0
0.0	0.0	0.0	0.0	0.0	0.0	0.0	0.0
0.0	0.0	0.0	0.0	0.0	0.0	0.0	0.0
0.0	0.0	0.0	0.0	0.0	0.0	0.0	0.0
0.0	0.0	0.0	0.0	0.0	0.0	0.0	0.0
0.0	0.0	0.0	0.0	0.0	0.0	0.0	0.0
0.0	0.0	0.0	0.0	0.0	0.0	0.0	0.0
0.0	0.0	0.0	0.0	0.0	0.0	0.0	0.0
0.0	0.0	0.0	0.0	0.0	0.0	0.0	0.0
0.0	0.0	0.0	0.0	0.0	0.0	0.0	0.0
0.0	0.0	0.0	0.0	0.0	0.0	0.0	0.0
0.0	0.0	0.0	0.0	0.0	0.0	0.0	0.0
0.0	0.0	0.0	0.0	0.0	0.0	0.0	0.0
0.0	0.0	0.0	0.0	0.0	0.0	0.0	0.0
0.0	0.0	0.0	0.0	0.0	0.0	0.0	0.0
0.0	0.0	0.0	0.0	0.0	0.0	0.0	0.0
0.0	0.0	0.0	0.0	0.0	0.0	0.0	0.0
0.0	0.0	0.0	0.0	0.0	0.0	0.0	0.0
0.0	0.0	0.0	0.0	0.0	0.0	0.0	0.0
0.0	0.0	0.0	0.0	0.0	0.0	0.0	0.0
0.0	0.0	0.0	0.0	0.0	0.0	0.0	0.0
0.0	0.0	0.0	0.0	0.0	0.0	0.0	0.0
0.0	0.0	0.0	0.0	0.0	0.0	0.0	0.0
0.0	0.0	0.0	0.0	0.0	0.0	0.0	0.0
0.0	0.0	0.0	0.0	0.0	0.0	0.0	0.0
0.0	0.0	0.0	0.0	0.0	0.0	0.0	0.0
0.0	0.0	0.0	0.0	0.0	0.0	0.0	0.0
0.0	0.0	0.0	0.0	0.0	0.0	0.0	0.0
0.0	0.0	0.0	0.0	0.0	0.0	0.0	0.0
0.0	0.0	0.0	0.0	0.0	0.0	0.0	0.0
0.0	0.0	0.0	0.0	0.0	0.0	0.0	0.0

0.0	0.0	0.0	0.0	0.0	0.0	0.0	0.0
0.0	0.0	0.0	0.0	0.0	0.0	0.0	0.0
0.0	0.0	0.0	0.0	0.0	0.0	0.0	0.0
0.0	0.0	0.0	0.0	0.0	0.0	0.0	0.0
0.0	0.0	0.0	0.0	0.0	0.0	0.0	0.0
0.0	0.0	0.0	0.0	0.0	0.0	0.0	0.0
0.0	0.0	0.0	0.0	0.0	0.0	0.0	0.0
0.0	0.0	0.0	0.0	0.0	0.0	0.0	0.0
0.0	0.0	0.0	0.0	0.0	0.0	0.0	0.0
0.0	0.0	0.0	0.0	0.0	0.0	0.0	0.0
0.0	0.0	0.0	0.0	0.0	0.0	0.0	0.0
0.0	0.0	0.0	0.0	0.0	0.0	0.0	0.0
0.0	0.0	0.0	0.0	0.0	0.0	0.0	0.0
0.0	0.0	0.0	0.0	0.0	0.0	0.0	0.0
0.0	0.0	0.0	0.0	0.0	0.0	0.0	0.0
0.0	0.0	0.0	0.0	0.0	0.0	0.0	0.0
0.0	0.0	0.0	0.0	0.0	0.0	0.0	0.0
0.0	0.0	0.0	0.0	0.0	0.0	0.0	0.0
0.0	0.0	0.0	0.0	0.0	0.0	0.0	0.0
0.0	0.0	0.0	0.0	0.0	0.0	0.0	0.0
0.0	0.0	0.0	0.0	0.0	0.0	0.0	0.0
0.0	0.0	0.0	0.0	0.0	0.0	0.0	0.0
0.0	0.0	0.0	0.0	0.0	0.0	0.0	0.0
0.0	0.0	0.0	0.0	0.0	0.0	0.0	0.0
0.0	0.0	0.0	0.0	0.0	0.0	0.0	0.0
0.0	0.0	0.0	0.0	0.0	0.0	0.0	0.0
0.0	0.0	0.0	0.0	0.0	0.0	0.0	0.0
0.0	0.0	0.0	0.0	0.0	0.0	0.0	0.0
0.0	0.0	0.0	0.0	0.0	0.0	0.0	0.0
0.0	0.0	0.0	0.0	0.0	0.0	0.0	0.0
0.0	0.0	0.0	0.0	0.0	0.0	0.0	0.0
0.0	0.0	0.0	0.0	0.0	0.0	0.0	0.0
0.0	0.0	0.0	0.0	0.0	0.0	0.0	0.0
0.0	0.0	0.0	0.0	0.0	0.0	0.0	0.0
0.0	0.0	0.0	0.0	0.0	0.0	0.0	0.0
0.0	0.0	0.0	0.0	0.0	0.0	0.0	0.0
0.0	0.0	0.0	0.0	0.0	0.0	0.0	0.0
0.0	0.0	0.0	0.0	0.0	0.0	0.0	0.0
0.0	0.0	0.0	0.0	0.0	0.0	0.0	0.0
0.0	0.0	0.0	0.0	0.0	0.0	0.0	0.0
0.0	0.0	0.0	0.0	0.0	0.0	0.0	0.0
0.0	0.0	0.0	0.0	0.0	0.0	0.0	0.0
0.0	0.0	0.0	0.0	0.0	0.0	0.0	0.0
0.0	0.0	0.0	0.0	0.0	0.0	0.0	0.0
0.0	0.0	0.0	0.0	0.0	0.0	0.0	0.0
0.0	0.0	0.0	0.0	0.0	0.0	0.0	0.0
0.0	0.0	0.0	0.0	0.0	0.0	0.0	0.0

0.0	0.0	0.0	0.0	0.0	0.0	0.0	0.0
0.0	0.0	0.0	0.0	0.0	0.0	0.0	0.0
0.0	0.0	0.0	0.0	0.0	0.0	0.0	0.0
0.0	0.0	0.0	0.0	0.0	0.0	0.0	0.0
0.0	0.0	0.0	0.0	0.0	0.0	0.0	0.0
0.0	0.0	0.0	0.0	0.0	0.0	0.0	0.0
0.0	0.0	0.0	0.0	0.0	0.0	0.0	0.0
0.0	0.0	0.0	0.0	0.0	0.0	0.0	0.0
0.0	0.0	0.0	0.0	0.0	0.0	0.0	0.0
0.0	0.0	0.0	0.0	0.0	0.0	0.0	0.0
0.0	0.0	0.0	0.0	0.0	0.0	0.0	0.0
0.0	0.0	0.0	0.0	0.0	0.0	0.0	0.0
0.0	0.0	0.0	0.0	0.0	0.0	0.0	0.0
0.0	0.0	0.0	0.0	0.0	0.0	0.0	0.0
0.0	0.0	0.0	0.0	0.0	0.0	0.0	0.0
0.0	0.0	0.0	0.0	0.0	0.0	0.0	0.0
0.0	0.0	0.0	0.0	0.0	0.0	0.0	0.0
0.0	0.0	0.0	0.0	0.0	0.0	0.0	0.0
0.0	0.0	0.0	0.0	0.0	0.0	0.0	0.0
0.0	0.0	0.0	0.0	0.0	0.0	0.0	0.0
0.0	0.0	0.0	0.0	0.0	0.0	0.0	0.0
0.0	0.0	0.0	0.0	0.0	0.0	0.0	0.0
0.0	0.0	0.0	0.0	0.0	0.0	0.0	0.0
0.0	0.0	0.0	0.0	0.0	0.0	0.0	0.0
0.0	0.0	0.0	0.0	0.0	0.0	0.0	0.0
0.0	0.0	0.0	0.0	0.0	0.0	0.0	0.0
0.0	0.0	0.0	0.0	0.0	0.0	0.0	0.0
0.0	0.0	0.0	0.0	0.0	0.0	0.0	0.0
0.0	0.0	0.0	0.0	0.0	0.0	0.0	0.0
0.0	0.0	0.0	0.0	0.0	0.0	0.0	0.0
0.0	0.0	0.0	0.0	0.0	0.0	0.0	0.0
0.0	0.0	0.0	0.0	0.0	0.0	0.0	0.0
0.0	0.0	0.0	0.0	0.0	0.0	0.0	0.0
0.0	0.0	0.0	0.0	0.0	0.0	0.0	0.0
0.0	0.0	0.0	0.0	0.0	0.0	0.0	0.0
0.0	0.0	0.0	0.0	0.0	0.0	0.0	0.0
0.0	0.0	0.0	0.0	0.0	0.0	0.0	0.0
0.0	0.0	0.0	0.0	0.0	0.0	0.0	0.0
0.0	0.0	0.0	0.0	0.0	0.0	0.0	0.0
0.0	0.0	0.0	0.0	0.0	0.0	0.0	0.0
0.0	0.0	0.0	0.0	0.0	0.0	0.0	0.0
0.0	0.0	0.0	0.0	0.0	0.0	0.0	0.0
0.0	0.0	0.0	0.0	0.0	0.0	0.0	0.0
0.0	0.0	0.0	0.0	0.0	0.0	0.0	0.0
0.0	0.0	0.0	0.0	0.0	0.0	0.0	0.0
0.0	0.0	0.0	0.0	0.0	0.0	0.0	0.0
0.0	0.0	0.0	0.0	0.0	0.0	0.0	0.0

0.0	0.0	0.0	0.0	0.0	0.0	0.0	0.0
0.0	0.0	0.0	0.0	0.0	0.0	0.0	0.0
0.0	0.0	0.0	0.0	0.0	0.0	0.0	0.0
0.0	0.0	0.0	0.0	0.0	0.0	0.0	0.0
0.0	0.0	0.0	0.0	0.0	0.0	0.0	0.0
0.0	0.0	0.0	0.0	0.0	0.0	0.0	0.0
0.0	0.0	0.0	0.0	0.0	0.0	0.0	0.0
0.0	0.0	0.0	0.0	0.0	0.0	0.0	0.0
0.0	0.0	0.0	0.0	0.0	0.0	0.0	0.0
0.0	0.0	0.0	0.0	0.0	0.0	0.0	0.0
0.0	0.0	0.0	0.0	0.0	0.0	0.0	0.0
0.0	0.0	0.0	0.0	0.0	0.0	0.0	0.0
0.0	0.0	0.0	0.0	0.0	0.0	0.0	0.0
0.0	0.0	0.0	0.0	0.0	0.0	0.0	0.0
0.0	0.0	0.0	0.0	0.0	0.0	0.0	0.0
0.0	0.0	0.0	0.0	0.0	0.0	0.0	0.0
0.0	0.0	0.0	0.0	0.0	0.0	0.0	0.0
0.0	0.0	0.0	0.0	0.0	0.0	0.0	0.0
0.0	0.0	0.0	0.0	0.0	0.0	0.0	0.0
0.0	0.0	0.0	0.0	0.0	0.0	0.0	0.0
0.0	0.0	0.0	0.0	0.0	0.0	0.0	0.0
0.0	0.0	0.0	0.0	0.0	0.0	0.0	0.0
0.0	0.0	0.0	0.0	0.0	0.0	0.0	0.0
0.0	0.0	0.0	0.0	0.0	0.0	0.0	0.0
0.0	0.0	0.0	0.0	0.0	0.0	0.0	0.0
0.0	0.0	0.0	0.0	0.0	0.0	0.0	0.0
0.0	0.0	0.0	0.0	0.0	0.0	0.0	0.0
0.0	0.0	0.0	0.0	0.0	0.0	0.0	0.0
0.0	0.0	0.0	0.0	0.0	0.0	0.0	0.0
0.0	0.0	0.0	0.0	0.0	0.0	0.0	0.0
0.0	0.0	0.0	0.0	0.0	0.0	0.0	0.0
0.0	0.0	0.0	0.0	0.0	0.0	0.0	0.0
0.0	0.0	0.0	0.0	0.0	0.0	0.0	0.0
0.0	0.0	0.0	0.0	0.0	0.0	0.0	0.0
0.0	0.0	0.0	0.0	0.0	0.0	0.0	0.0
0.0	0.0	0.0	0.0	0.0	0.0	0.0	0.0
0.0	0.0	0.0	0.0	0.0	0.0	0.0	0.0
0.0	0.0	0.0	0.0	0.0	0.0	0.0	0.0
0.0	0.0	0.0	0.0	0.0	0.0	0.0	0.0
0.0	0.0	0.0	0.0	0.0	0.0	0.0	0.0
0.0	0.0	0.0	0.0	0.0	0.0	0.0	0.0
0.0	0.0	0.0	0.0	0.0	0.0	0.0	0.0
0.0	0.0	0.0	0.0	0.0	0.0	0.0	0.0
0.0	0.0	0.0	0.0	0.0	0.0	0.0	0.0
0.0	0.0	0.0	0.0	0.0	0.0	0.0	0.0
0.0	0.0	0.0	0.0	0.0	0.0	0.0	0.0
0.0	0.0	0.0	0.0	0.0	0.0	0.0	0.0
0.0	0.0	0.0	0.0	0.0	0.0	0.0	0.0

0.0	0.0	0.0	0.0	0.0	0.0	0.0	0.0
0.0	0.0	0.0	0.0	0.0	0.0	0.0	0.0
0.0	0.0	0.0	0.0	0.0	0.0	0.0	0.0
0.0	0.0	0.0	0.0	0.0	0.0	0.0	0.0
0.0	0.0	0.0	0.0	0.0	0.0	0.0	0.0
0.0	0.0	0.0	0.0	0.0	0.0	0.0	0.0
0.0	0.0	0.0	0.0	0.0	0.0	0.0	0.0
0.0	0.0	0.0	0.0	0.0	0.0	0.0	0.0
0.0	0.0	0.0	0.0	0.0	0.0	0.0	0.0
0.0	0.0	0.0	0.0	0.0	0.0	0.0	0.0
0.0	0.0	0.0	0.0	0.0	0.0	0.0	0.0
0.0	0.0	0.0	0.0	0.0	0.0	0.0	0.0
0.0	0.0	0.0	0.0	0.0	0.0	0.0	0.0
0.0	0.0	0.0	0.0	0.0	0.0	0.0	0.0
0.0	0.0	0.0	0.0	0.0	0.0	0.0	0.0
0.0	0.0	0.0	0.0	0.0	0.0	0.0	0.0
0.0	0.0	0.0	0.0	0.0	0.0	0.0	0.0
0.0	0.0	0.0	0.0	0.0	0.0	0.0	0.0
0.0	0.0	0.0	0.0	0.0	0.0	0.0	0.0
0.0	0.0	0.0	0.0	0.0	0.0	0.0	0.0
0.0	0.0	0.0	0.0	0.0	0.0	0.0	0.0
0.0	0.0	0.0	0.0	0.0	0.0	0.0	0.0
0.0	0.0	0.0	0.0	0.0	0.0	0.0	0.0
0.0	0.0	0.0	0.0	0.0	0.0	0.0	0.0
0.0	0.0	0.0	0.0	0.0	0.0	0.0	0.0
0.0	0.0	0.0	0.0	0.0	0.0	0.0	0.0
0.0	0.0	0.0	0.0	0.0	0.0	0.0	0.0
0.0	0.0	0.0	0.0	0.0	0.0	0.0	0.0
0.0	0.0	0.0	0.0	0.0	0.0	0.0	0.0
0.0	0.0	0.0	0.0	0.0	0.0	0.0	0.0
0.0	0.0	0.0	0.0	0.0	0.0	0.0	0.0
0.0	0.0	0.0	0.0	0.0	0.0	0.0	0.0
0.0	0.0	0.0	0.0	0.0	0.0	0.0	0.0
0.0	0.0	0.0	0.0	0.0	0.0	0.0	0.0
0.0	0.0	0.0	0.0	0.0	0.0	0.0	0.0
0.0	0.0	0.0	0.0	0.0	0.0	0.0	0.0
0.0	0.0	0.0	0.0	0.0	0.0	0.0	0.0
0.0	0.0	0.0	0.0	0.0	0.0	0.0	0.0
0.0	0.0	0.0	0.0	0.0	0.0	0.0	0.0
0.0	0.0	0.0	0.0	0.0	0.0	0.0	0.0
0.0	0.0	0.0	0.0	0.0	0.0	0.0	0.0
0.0	0.0	0.0	0.0	0.0	0.0	0.0	0.0
0.0	0.0	0.0	0.0	0.0	0.0	0.0	0.0
0.0	0.0	0.0	0.0	0.0	0.0	0.0	0.0
0.0	0.0	0.0	0.0	0.0	0.0	0.0	0.0
0.0	0.0	0.0	0.0	0.0	0.0	0.0	0.0
0.0	0.0	0.0	0.0	0.0	0.0	0.0	0.0

0.0	0.0	0.0	0.0	0.0	0.0	0.0	0.0
0.0	0.0	0.0	0.0	0.0	0.0	0.0	0.0
0.0	0.0	0.0	0.0	0.0	0.0	0.0	0.0
0.0	0.0	0.0	0.0	0.0	0.0	0.0	0.0
0.0	0.0	0.0	0.0	0.0	0.0	0.0	0.0
0.0	0.0	0.0	0.0	0.0	0.0	0.0	0.0
0.0	0.0	0.0	0.0	0.0	0.0	0.0	0.0
0.0	0.0	0.0	0.0	0.0	0.0	0.0	0.0
0.0	0.0	0.0	0.0	0.0	0.0	0.0	0.0
0.0	0.0	0.0	0.0	0.0	0.0	0.0	0.0
0.0	0.0	0.0	0.0	0.0	0.0	0.0	0.0
0.0	0.0	0.0	0.0	0.0	0.0	0.0	0.0
0.0	0.0	0.0	0.0	0.0	0.0	0.0	0.0
0.0	0.0	0.0	0.0	0.0	0.0	0.0	0.0
0.0	0.0	0.0	0.0	0.0	0.0	0.0	0.0
0.0	0.0	0.0	0.0	0.0	0.0	0.0	0.0
0.0	0.0	0.0	0.0	0.0	0.0	0.0	0.0
0.0	0.0	0.0	0.0	0.0	0.0	0.0	0.0
0.0	0.0	0.0	0.0	0.0	0.0	0.0	0.0
0.0	0.0	0.0	0.0	0.0	0.0	0.0	0.0
0.0	0.0	0.0	0.0	0.0	0.0	0.0	0.0
0.0	0.0	0.0	0.0	0.0	0.0	0.0	0.0
0.0	0.0	0.0	0.0	0.0	0.0	0.0	0.0
0.0	0.0	0.0	0.0	0.0	0.0	0.0	0.0
0.0	0.0	0.0	0.0	0.0	0.0	0.0	0.0
0.0	0.0	0.0	0.0	0.0	0.0	0.0	0.0
0.0	0.0	0.0	0.0	0.0	0.0	0.0	0.0
0.0	0.0	0.0	0.0	0.0	0.0	0.0	0.0
0.0	0.0	0.0	0.0	0.0	0.0	0.0	0.0
0.0	0.0	0.0	0.0	0.0	0.0	0.0	0.0
0.0	0.0	0.0	0.0	0.0	0.0	0.0	0.0
0.0	0.0	0.0	0.0	0.0	0.0	0.0	0.0
0.0	0.0	0.0	0.0	0.0	0.0	0.0	0.0
0.0	0.0	0.0	0.0	0.0	0.0	0.0	0.0
0.0	0.0	0.0	0.0	0.0	0.0	0.0	0.0
0.0	0.0	0.0	0.0	0.0	0.0	0.0	0.0
0.0	0.0	0.0	0.0	0.0	0.0	0.0	0.0
0.0	0.0	0.0	0.0	0.0	0.0	0.0	0.0
0.0	0.0	0.0	0.0	0.0	0.0	0.0	0.0
0.0	0.0	0.0	0.0	0.0	0.0	0.0	0.0
0.0	0.0	0.0	0.0	0.0	0.0	0.0	0.0
0.0	0.0	0.0	0.0	0.0	0.0	0.0	0.0
0.0	0.0	0.0	0.0	0.0	0.0	0.0	0.0
0.0	0.0	0.0	0.0	0.0	0.0	0.0	0.0
0.0	0.0	0.0	0.0	0.0	0.0	0.0	0.0
0.0	0.0	0.0	0.0	0.0	0.0	0.0	0.0
0.0	0.0	0.0	0.0	0.0	0.0	0.0	0.0
0.0	0.0	0.0	0.0	0.0	0.0	0.0	0.0

0.0	0.0	0.0	0.0	0.0	0.0	0.0	0.0
0.0	0.0	0.0	0.0	0.0	0.0	0.0	0.0
0.0	0.0	0.0	0.0	0.0	0.0	0.0	0.0
0.0	0.0	0.0	0.0	0.0	0.0	0.0	0.0
0.0	0.0	0.0	0.0	0.0	0.0	0.0	0.0
0.0	0.0	0.0	0.0	0.0	0.0	0.0	0.0
0.0	0.0	0.0	0.0	0.0	0.0	0.0	0.0
0.0	0.0	0.0	0.0	0.0	0.0	0.0	0.0
0.0	0.0	0.0	0.0	0.0	0.0	0.0	0.0
0.0	0.0	0.0	0.0	0.0	0.0	0.0	0.0
0.0	0.0	0.0	0.0	0.0	0.0	0.0	0.0
0.0	0.0	0.0	0.0	0.0	0.0	0.0	0.0
0.0	0.0	0.0	0.0	0.0	0.0	0.0	0.0
0.0	0.0	0.0	0.0	0.0	0.0	0.0	0.0
0.0	0.0	0.0	0.0	0.0	0.0	0.0	0.0
0.0	0.0	0.0	0.0	0.0	0.0	0.0	0.0
0.0	0.0	0.0	0.0	0.0	0.0	0.0	0.0
0.0	0.0	0.0	0.0	0.0	0.0	0.0	0.0
0.0	0.0	0.0	0.0	0.0	0.0	0.0	0.0
0.0	0.0	0.0	0.0	0.0	0.0	0.0	0.0
0.0	0.0	0.0	0.0	0.0	0.0	0.0	0.0
0.0	0.0	0.0	0.0	0.0	0.0	0.0	0.0
0.0	0.0	0.0	0.0	0.0	0.0	0.0	0.0
0.0	0.0	0.0	0.0	0.0	0.0	0.0	0.0
0.0	0.0	0.0	0.0	0.0	0.0	0.0	0.0
0.0	0.0	0.0	0.0	0.0	0.0	0.0	0.0
0.0	0.0	0.0	0.0	0.0	0.0	0.0	0.0
0.0	0.0	0.0	0.0	0.0	0.0	0.0	0.0
0.0	0.0	0.0	0.0	0.0	0.0	0.0	0.0
0.0	0.0	0.0	0.0	0.0	0.0	0.0	0.0
0.0	0.0	0.0	0.0	0.0	0.0	0.0	0.0
0.0	0.0	0.0	0.0	0.0	0.0	0.0	0.0
0.0	0.0	0.0	0.0	0.0	0.0	0.0	0.0
0.0	0.0	0.0	0.0	0.0	0.0	0.0	0.0
0.0	0.0	0.0	0.0	0.0	0.0	0.0	0.0
0.0	0.0	0.0	0.0	0.0	0.0	0.0	0.0
0.0	0.0	0.0	0.0	0.0	0.0	0.0	0.0
0.0	0.0	0.0	0.0	0.0	0.0	0.0	0.0
0.0	0.0	0.0	0.0	0.0	0.0	0.0	0.0
0.0	0.0	0.0	0.0	0.0	0.0	0.0	0.0
0.0	0.0	0.0	0.0	0.0	0.0	0.0	0.0
0.0	0.0	0.0	0.0	0.0	0.0	0.0	0.0
0.0	0.0	0.0	0.0	0.0	0.0	0.0	0.0
0.0	0.0	0.0	0.0	0.0	0.0	0.0	0.0
0.0	0.0	0.0	0.0	0.0	0.0	0.0	0.0
0.0	0.0	0.0	0.0	0.0	0.0	0.0	0.0
0.0	0.0	0.0	0.0	0.0	0.0	0.0	0.0

0.0	0.0	0.0	0.0	0.0	0.0	0.0	0.0
0.0	0.0	0.0	0.0	0.0	0.0	0.0	0.0
0.0	0.0	0.0	0.0	0.0	0.0	0.0	0.0
0.0	0.0	0.0	0.0	0.0	0.0	0.0	0.0
0.0	0.0	0.0	0.0	0.0	0.0	0.0	0.0
0.0	0.0	0.0	0.0	0.0	0.0	0.0	0.0
0.0	0.0	0.0	0.0	0.0	0.0	0.0	0.0
0.0	0.0	0.0	0.0	0.0	0.0	0.0	0.0
0.0	0.0	0.0	0.0	0.0	0.0	0.0	0.0
0.0	0.0	0.0	0.0	0.0	0.0	0.0	0.0
0.0	0.0	0.0	0.0	0.0	0.0	0.0	0.0
0.0	0.0	0.0	0.0	0.0	0.0	0.0	0.0
0.0	0.0	0.0	0.0	0.0	0.0	0.0	0.0
0.0	0.0	0.0	0.0	0.0	0.0	0.0	0.0
0.0	0.0	0.0	0.0	0.0	0.0	0.0	0.0
0.0	0.0	0.0	0.0	0.0	0.0	0.0	0.0
0.0	0.0	0.0	0.0	0.0	0.0	0.0	0.0
0.0	0.0	0.0	0.0	0.0	0.0	0.0	0.0
0.0	0.0	0.0	0.0	0.0	0.0	0.0	0.0
0.0	0.0	0.0	0.0	0.0	0.0	0.0	0.0
0.0	0.0	0.0	0.0	0.0	0.0	0.0	0.0
0.0	0.0	0.0	0.0	0.0	0.0	0.0	0.0
0.0	0.0	0.0	0.0	0.0	0.0	0.0	0.0
0.0	0.0	0.0	0.0	0.0	0.0	0.0	0.0
0.0	0.0	0.0	0.0	0.0	0.0	0.0	0.0
0.0	0.0	0.0	0.0	0.0	0.0	0.0	0.0
0.0	0.0	0.0	0.0	0.0	0.0	0.0	0.0
0.0	0.0	0.0	0.0	0.0	0.0	0.0	0.0
0.0	0.0	0.0	0.0	0.0	0.0	0.0	0.0
0.0	0.0	0.0	0.0	0.0	0.0	0.0	0.0
0.0	0.0	0.0	0.0	0.0	0.0	0.0	0.0
0.0	0.0	0.0	0.0	0.0	0.0	0.0	0.0
0.0	0.0	0.0	0.0	0.0	0.0	0.0	0.0
0.0	0.0	0.0	0.0	0.0	0.0	0.0	0.0
0.0	0.0	0.0	0.0	0.0	0.0	0.0	0.0
0.0	0.0	0.0	0.0	0.0	0.0	0.0	0.0
0.0	0.0	0.0	0.0	0.0	0.0	0.0	0.0
0.0	0.0	0.0	0.0	0.0	0.0	0.0	0.0
0.0	0.0	0.0	0.0	0.0	0.0	0.0	0.0
0.0	0.0	0.0	0.0	0.0	0.0	0.0	0.0
0.0	0.0	0.0	0.0	0.0	0.0	0.0	0.0
0.0	0.0	0.0	0.0	0.0	0.0	0.0	0.0
0.0	0.0	0.0	0.0	0.0	0.0	0.0	0.0
0.0	0.0	0.0	0.0	0.0	0.0	0.0	0.0
0.0	0.0	0.0	0.0	0.0	0.0	0.0	0.0
0.0	0.0	0.0	0.0	0.0	0.0	0.0	0.0
0.0	0.0	0.0	0.0	0.0	0.0	0.0	0.0

0.0	0.0	0.0	0.0	0.0	0.0	0.0	0.0
0.0	0.0	0.0	0.0	0.0	0.0	0.0	0.0
0.0	0.0	0.0	0.0	0.0	0.0	0.0	0.0
0.0	0.0	0.0	0.0	0.0	0.0	0.0	0.0
0.0	0.0	0.0	0.0	0.0	0.0	0.0	0.0
0.0	0.0	0.0	0.0	0.0	0.0	0.0	0.0
0.0	0.0	0.0	0.0	0.0	0.0	0.0	0.0
0.0	0.0	0.0	0.0	0.0	0.0	0.0	0.0
0.0	0.0	0.0	0.0	0.0	0.0	0.0	0.0
0.0	0.0	0.0	0.0	0.0	0.0	0.0	0.0
0.0	0.0	0.0	0.0	0.0	0.0	0.0	0.0
0.0	0.0	0.0	0.0	0.0	0.0	0.0	0.0
0.0	0.0	0.0	0.0	0.0	0.0	0.0	0.0
0.0	0.0	0.0	0.0	0.0	0.0	0.0	0.0
0.0	0.0	0.0	0.0	0.0	0.0	0.0	0.0
0.0	0.0	0.0	0.0	0.0	0.0	0.0	0.0
0.0	0.0	0.0	0.0	0.0	0.0	0.0	0.0
0.0	0.0	0.0	0.0	0.0	0.0	0.0	0.0
0.0	0.0	0.0	0.0	0.0	0.0	0.0	0.0
0.0	0.0	0.0	0.0	0.0	0.0	0.0	0.0
0.0	0.0	0.0	0.0	0.0	0.0	0.0	0.0
0.0	0.0	0.0	0.0	0.0	0.0	0.0	0.0
0.0	0.0	0.0	0.0	0.0	0.0	0.0	0.0
0.0	0.0	0.0	0.0	0.0	0.0	0.0	0.0
0.0	0.0	0.0	0.0	0.0	0.0	0.0	0.0
0.0	0.0	0.0	0.0	0.0	0.0	0.0	0.0
0.0	0.0	0.0	0.0	0.0	0.0	0.0	0.0
0.0	0.0	0.0	0.0	0.0	0.0	0.0	0.0
0.0	0.0	0.0	0.0	0.0	0.0	0.0	0.0
0.0	0.0	0.0	0.0	0.0	0.0	0.0	0.0
0.0	0.0	0.0	0.0	0.0	0.0	0.0	0.0
0.0	0.0	0.0	0.0	0.0	0.0	0.0	0.0
0.0	0.0	0.0	0.0	0.0	0.0	0.0	0.0
0.0	0.0	0.0	0.0	0.0	0.0	0.0	0.0
0.0	0.0	0.0	0.0	0.0	0.0	0.0	0.0
0.0	0.0	0.0	0.0	0.0	0.0	0.0	0.0
0.0	0.0	0.0	0.0	0.0	0.0	0.0	0.0
0.0	0.0	0.0	0.0	0.0	0.0	0.0	0.0
0.0	0.0	0.0	0.0	0.0	0.0	0.0	0.0
0.0	0.0	0.0	0.0	0.0	0.0	0.0	0.0
0.0	0.0	0.0	0.0	0.0	0.0	0.0	0.0
0.0	0.0	0.0	0.0	0.0	0.0	0.0	0.0
0.0	0.0	0.0	0.0	0.0	0.0	0.0	0.0
0.0	0.0	0.0	0.0	0.0	0.0	0.0	0.0
0.0	0.0	0.0	0.0	0.0	0.0	0.0	0.0
0.0	0.0	0.0	0.0	0.0	0.0	0.0	0.0
0.0	0.0	0.0	0.0	0.0	0.0	0.0	0.0

0.0	0.0	0.0	0.0	0.0	0.0	0.0	0.0
0.0	0.0	0.0	0.0	0.0	0.0	0.0	0.0
0.0	0.0	0.0	0.0	0.0	0.0	0.0	0.0
0.0	0.0	0.0	0.0	0.0	0.0	0.0	0.0
0.0	0.0	0.0	0.0	0.0	0.0	0.0	0.0
0.0	0.0	0.0	0.0	0.0	0.0	0.0	0.0
0.0	0.0	0.0	0.0	0.0	0.0	0.0	0.0
0.0	0.0	0.0	0.0	0.0	0.0	0.0	0.0
0.0	0.0	0.0	0.0	0.0	0.0	0.0	0.0
0.0	0.0	0.0	0.0	0.0	0.0	0.0	0.0
0.0	0.0	0.0	0.0	0.0	0.0	0.0	0.0
0.0	0.0	0.0	0.0	0.0	0.0	0.0	0.0
0.0	0.0	0.0	0.0	0.0	0.0	0.0	0.0
0.0	0.0	0.0	0.0	0.0	0.0	0.0	0.0
0.0	0.0	0.0	0.0	0.0	0.0	0.0	0.0
0.0	0.0	0.0	0.0	0.0	0.0	0.0	0.0
0.0	0.0	0.0	0.0	0.0	0.0	0.0	0.0
0.0	0.0	0.0	0.0	0.0	0.0	0.0	0.0
0.0	0.0	0.0	0.0	0.0	0.0	0.0	0.0
0.0	0.0	0.0	0.0	0.0	0.0	0.0	0.0
0.0	0.0	0.0	0.0	0.0	0.0	0.0	0.0
0.0	0.0	0.0	0.0	0.0	0.0	0.0	0.0
0.0	0.0	0.0	0.0	0.0	0.0	0.0	0.0
0.0	0.0	0.0	0.0	0.0	0.0	0.0	0.0
0.0	0.0	0.0	0.0	0.0	0.0	0.0	0.0
0.0	0.0	0.0	0.0	0.0	0.0	0.0	0.0
0.0	0.0	0.0	0.0	0.0	0.0	0.0	0.0
0.0	0.0	0.0	0.0	0.0	0.0	0.0	0.0
0.0	0.0	0.0	0.0	0.0	0.0	0.0	0.0
0.0	0.0	0.0	0.0	0.0	0.0	0.0	0.0
0.0	0.0	0.0	0.0	0.0	0.0	0.0	0.0
0.0	0.0	0.0	0.0	0.0	0.0	0.0	0.0
0.0	0.0	0.0	0.0	0.0	0.0	0.0	0.0
0.0	0.0	0.0	0.0	0.0	0.0	0.0	0.0
0.0	0.0	0.0	0.0	0.0	0.0	0.0	0.0
0.0	0.0	0.0	0.0	0.0	0.0	0.0	0.0
0.0	0.0	0.0	0.0	0.0	0.0	0.0	0.0
0.0	0.0	0.0	0.0	0.0	0.0	0.0	0.0
0.0	0.0	0.0	0.0	0.0	0.0	0.0	0.0
0.0	0.0	0.0	0.0	0.0	0.0	0.0	0.0
0.0	0.0	0.0	0.0	0.0	0.0	0.0	0.0
0.0	0.0	0.0	0.0	0.0	0.0	0.0	0.0
0.0	0.0	0.0	0.0	0.0	0.0	0.0	0.0
0.0	0.0	0.0	0.0	0.0	0.0	0.0	0.0
0.0	0.0	0.0	0.0	0.0	0.0	0.0	0.0
0.0	0.0	0.0	0.0	0.0	0.0	0.0	0.0
0.0	0.0	0.0	0.0	0.0	0.0	0.0	0.0

0.0	0.0	0.0	0.0	0.0	0.0	0.0	0.0
0.0	0.0	0.0	0.0	0.0	0.0	0.0	0.0
0.0	0.0	0.0	0.0	0.0	0.0	0.0	0.0
0.0	0.0	0.0	0.0	0.0	0.0	0.0	0.0
0.0	0.0	0.0	0.0	0.0	0.0	0.0	0.0
0.0	0.0	0.0	0.0	0.0	0.0	0.0	0.0
0.0	0.0	0.0	0.0	0.0	0.0	0.0	0.0
0.0	0.0	0.0	0.0	0.0	0.0	0.0	0.0
0.0	0.0	0.0	0.0	0.0	0.0	0.0	0.0
0.0	0.0	0.0	0.0	0.0	0.0	0.0	0.0
0.0	0.0	0.0	0.0	0.0	0.0	0.0	0.0
0.0	0.0	0.0	0.0	0.0	0.0	0.0	0.0
0.0	0.0	0.0	0.0	0.0	0.0	0.0	0.0
0.0	0.0	0.0	0.0	0.0	0.0	0.0	0.0
0.0	0.0	0.0	0.0	0.0	0.0	0.0	0.0
0.0	0.0	0.0	0.0	0.0	0.0	0.0	0.0
0.0	0.0	0.0	0.0	0.0	0.0	0.0	0.0
0.0	0.0	0.0	0.0	0.0	0.0	0.0	0.0
0.0	0.0	0.0	0.0	0.0	0.0	0.0	0.0
0.0	0.0	0.0	0.0	0.0	0.0	0.0	0.0
0.0	0.0	0.0	0.0	0.0	0.0	0.0	0.0
0.0	0.0	0.0	0.0	0.0	0.0	0.0	0.0
0.0	0.0	0.0	0.0	0.0	0.0	0.0	0.0
0.0	0.0	0.0	0.0	0.0	0.0	0.0	0.0
0.0	0.0	0.0	0.0	0.0	0.0	0.0	0.0
0.0	0.0	0.0	0.0	0.0	0.0	0.0	0.0
0.0	0.0	0.0	0.0	0.0	0.0	0.0	0.0
0.0	0.0	0.0	0.0	0.0	0.0	0.0	0.0
0.0	0.0	0.0	0.0	0.0	0.0	0.0	0.0
0.0	0.0	0.0	0.0	0.0	0.0	0.0	0.0
0.0	0.0	0.0	0.0	0.0	0.0	0.0	0.0
0.0	0.0	0.0	0.0	0.0	0.0	0.0	0.0
0.0	0.0	0.0	0.0	0.0	0.0	0.0	0.0
0.0	0.0	0.0	0.0	0.0	0.0	0.0	0.0
0.0	0.0	0.0	0.0	0.0	0.0	0.0	0.0
0.0	0.0	0.0	0.0	0.0	0.0	0.0	0.0
0.0	0.0	0.0	0.0	0.0	0.0	0.0	0.0
0.0	0.0	0.0	0.0	0.0	0.0	0.0	0.0
0.0	0.0	0.0	0.0	0.0	0.0	0.0	0.0
0.0	0.0	0.0	0.0	0.0	0.0	0.0	0.0
0.0	0.0	0.0	0.0	0.0	0.0	0.0	0.0
0.0	0.0	0.0	0.0	0.0	0.0	0.0	0.0
0.0	0.0	0.0	0.0	0.0	0.0	0.0	0.0
0.0	0.0	0.0	0.0	0.0	0.0	0.0	0.0
0.0	0.0	0.0	0.0	0.0	0.0	0.0	0.0
0.0	0.0	0.0	0.0	0.0	0.0	0.0	0.0
0.0	0.0	0.0	0.0	0.0	0.0	0.0	0.0
0.0	0.0	0.0	0.0	0.0	0.0	0.0	0.0

0.0	0.0	0.0	0.0	0.0	0.0	0.0	0.0
0.0	0.0	0.0	0.0	0.0	0.0	0.0	0.0
0.0	0.0	0.0	0.0	0.0	0.0	0.0	0.0
0.0	0.0	0.0	0.0	0.0	0.0	0.0	0.0
0.0	0.0	0.0	0.0	0.0	0.0	0.0	0.0
0.0	0.0	0.0	0.0	0.0	0.0	0.0	0.0
0.0	0.0	0.0	0.0	0.0	0.0	0.0	0.0
0.0	0.0	0.0	0.0	0.0	0.0	0.0	0.0
0.0	0.0	0.0	0.0	0.0	0.0	0.0	0.0
0.0	0.0	0.0	0.0	0.0	0.0	0.0	0.0
0.0	0.0	0.0	0.0	0.0	0.0	0.0	0.0
0.0	0.0	0.0	0.0	0.0	0.0	0.0	0.0
0.0	0.0	0.0	0.0	0.0	0.0	0.0	0.0
0.0	0.0	0.0	0.0	0.0	0.0	0.0	0.0
0.0	0.0	0.0	0.0	0.0	0.0	0.0	0.0
0.0	0.0	0.0	0.0	0.0	0.0	0.0	0.0
0.0	0.0	0.0	0.0	0.0	0.0	0.0	0.0
0.0	0.0	0.0	0.0	0.0	0.0	0.0	0.0
0.0	0.0	0.0	0.0	0.0	0.0	0.0	0.0
0.0	0.0	0.0	0.0	0.0	0.0	0.0	0.0
0.0	0.0	0.0	0.0	0.0	0.0	0.0	0.0
0.0	0.0	0.0	0.0	0.0	0.0	0.0	0.0
0.0	0.0	0.0	0.0	0.0	0.0	0.0	0.0
0.0	0.0	0.0	0.0	0.0	0.0	0.0	0.0
0.0	0.0	0.0	0.0	0.0	0.0	0.0	0.0
0.0	0.0	0.0	0.0	0.0	0.0	0.0	0.0
0.0	0.0	0.0	0.0	0.0	0.0	0.0	0.0
0.0	0.0	0.0	0.0	0.0	0.0	0.0	0.0
0.0	0.0	0.0	0.0	0.0	0.0	0.0	0.0
0.0	0.0	0.0	0.0	0.0	0.0	0.0	0.0
0.0	0.0	0.0	0.0	0.0	0.0	0.0	0.0
0.0	0.0	0.0	0.0	0.0	0.0	0.0	0.0
0.0	0.0	0.0	0.0	0.0	0.0	0.0	0.0
0.0	0.0	0.0	0.0	0.0	0.0	0.0	0.0
0.0	0.0	0.0	0.0	0.0	0.0	0.0	0.0
0.0	0.0	0.0	0.0	0.0	0.0	0.0	0.0
0.0	0.0	0.0	0.0	0.0	0.0	0.0	0.0
0.0	0.0	0.0	0.0	0.0	0.0	0.0	0.0
0.0	0.0	0.0	0.0	0.0	0.0	0.0	0.0
0.0	0.0	0.0	0.0	0.0	0.0	0.0	0.0
0.0	0.0	0.0	0.0	0.0	0.0	0.0	0.0
0.0	0.0	0.0	0.0	0.0	0.0	0.0	0.0
0.0	0.0	0.0	0.0	0.0	0.0	0.0	0.0
0.0	0.0	0.0	0.0	0.0	0.0	0.0	0.0
0.0	0.0	0.0	0.0	0.0	0.0	0.0	0.0
0.0	0.0	0.0	0.0	0.0	0.0	0.0	0.0
0.0	0.0	0.0	0.0	0.0	0.0	0.0	0.0

0.0	0.0	0.0	0.0	0.0	0.0	0.0	0.0
0.0	0.0	0.0	0.0	0.0	0.0	0.0	0.0
0.0	0.0	0.0	0.0	0.0	0.0	0.0	0.0
0.0	0.0	0.0	0.0	0.0	0.0	0.0	0.0
0.0	0.0	0.0	0.0	0.0	0.0	0.0	0.0
0.0	0.0	0.0	0.0	0.0	0.0	0.0	0.0
0.0	0.0	0.0	0.0	0.0	0.0	0.0	0.0
0.0	0.0	0.0	0.0	0.0	0.0	0.0	0.0
0.0	0.0	0.0	0.0	0.0	0.0	0.0	0.0
0.0	0.0	0.0	0.0	0.0	0.0	0.0	0.0
0.0	0.0	0.0	0.0	0.0	0.0	0.0	0.0
0.0	0.0	0.0	0.0	0.0	0.0	0.0	0.0
0.0	0.0	0.0	0.0	0.0	0.0	0.0	0.0
0.0	0.0	0.0	0.0	0.0	0.0	0.0	0.0
0.0	0.0	0.0	0.0	0.0	0.0	0.0	0.0
0.0	0.0	0.0	0.0	0.0	0.0	0.0	0.0
0.0	0.0	0.0	0.0	0.0	0.0	0.0	0.0
0.0	0.0	0.0	0.0	0.0	0.0	0.0	0.0
0.0	0.0	0.0	0.0	0.0	0.0	0.0	0.0
0.0	0.0	0.0	0.0	0.0	0.0	0.0	0.0
0.0	0.0	0.0	0.0	0.0	0.0	0.0	0.0
0.0	0.0	0.0	0.0	0.0	0.0	0.0	0.0
0.0	0.0	0.0	0.0	0.0	0.0	0.0	0.0
0.0	0.0	0.0	0.0	0.0	0.0	0.0	0.0
0.0	0.0	0.0	0.0	0.0	0.0	0.0	0.0
0.0	0.0	0.0	0.0	0.0	0.0	0.0	0.0
0.0	0.0	0.0	0.0	0.0	0.0	0.0	0.0
0.0	0.0	0.0	0.0	0.0	0.0	0.0	0.0
0.0	0.0	0.0	0.0	0.0	0.0	0.0	0.0
0.0	0.0	0.0	0.0	0.0	0.0	0.0	0.0
0.0	0.0	0.0	0.0	0.0	0.0	0.0	0.0
0.0	0.0	0.0	0.0	0.0	0.0	0.0	0.0
0.0	0.0	0.0	0.0	0.0	0.0	0.0	0.0
0.0	0.0	0.0	0.0	0.0	0.0	0.0	0.0
0.0	0.0	0.0	0.0	0.0	0.0	0.0	0.0
0.0	0.0	0.0	0.0	0.0	0.0	0.0	0.0
0.0	0.0	0.0	0.0	0.0	0.0	0.0	0.0
0.0	0.0	0.0	0.0	0.0	0.0	0.0	0.0
0.0	0.0	0.0	0.0	0.0	0.0	0.0	0.0
0.0	0.0	0.0	0.0	0.0	0.0	0.0	0.0
0.0	0.0	0.0	0.0	0.0	0.0	0.0	0.0
0.0	0.0	0.0	0.0	0.0	0.0	0.0	0.0
0.0	0.0	0.0	0.0	0.0	0.0	0.0	0.0
0.0	0.0	0.0	0.0	0.0	0.0	0.0	0.0
0.0	0.0	0.0	0.0	0.0	0.0	0.0	0.0
0.0	0.0	0.0	0.0	0.0	0.0	0.0	0.0
0.0	0.0	0.0	0.0	0.0	0.0	0.0	0.0

0.0	0.0	0.0	0.0	0.0	0.0	0.0	0.0
0.0	0.0	0.0	0.0	0.0	0.0	0.0	0.0
0.0	0.0	0.0	0.0	0.0	0.0	0.0	0.0
0.0	0.0	0.0	0.0	0.0	0.0	0.0	0.0
0.0	0.0	0.0	0.0	0.0	0.0	0.0	0.0
0.0	0.0	0.0	0.0	0.0	0.0	0.0	0.0
0.0	0.0	0.0	0.0	0.0	0.0	0.0	0.0
0.0	0.0	0.0	0.0	0.0	0.0	0.0	0.0
0.0	0.0	0.0	0.0	0.0	0.0	0.0	0.0
0.0	0.0	0.0	0.0	0.0	0.0	0.0	0.0
0.0	0.0	0.0	0.0	0.0	0.0	0.0	0.0
0.0	0.0	0.0	0.0	0.0	0.0	0.0	0.0
0.0	0.0	0.0	0.0	0.0	0.0	0.0	0.0
0.0	0.0	0.0	0.0	0.0	0.0	0.0	0.0
0.0	0.0	0.0	0.0	0.0	0.0	0.0	0.0
0.0	0.0	0.0	0.0	0.0	0.0	0.0	0.0
0.0	0.0	0.0	0.0	0.0	0.0	0.0	0.0
0.0	0.0	0.0	0.0	0.0	0.0	0.0	0.0
0.0	0.0	0.0	0.0	0.0	0.0	0.0	0.0
0.0	0.0	0.0	0.0	0.0	0.0	0.0	0.0
0.0	0.0	0.0	0.0	0.0	0.0	0.0	0.0
0.0	0.0	0.0	0.0	0.0	0.0	0.0	0.0
0.0	0.0	0.0	0.0	0.0	0.0	0.0	0.0
0.0	0.0	0.0	0.0	0.0	0.0	0.0	0.0
0.0	0.0	0.0	0.0	0.0	0.0	0.0	0.0
0.0	0.0	0.0	0.0	0.0	0.0	0.0	0.0
0.0	0.0	0.0	0.0	0.0	0.0	0.0	0.0
0.0	0.0	0.0	0.0	0.0	0.0	0.0	0.0
0.0	0.0	0.0	0.0	0.0	0.0	0.0	0.0
0.0	0.0	0.0	0.0	0.0	0.0	0.0	0.0
0.0	0.0	0.0	0.0	0.0	0.0	0.0	0.0
0.0	0.0	0.0	0.0	0.0	0.0	0.0	0.0
0.0	0.0	0.0	0.0	0.0	0.0	0.0	0.0
0.0	0.0	0.0	0.0	0.0	0.0	0.0	0.0
0.0	0.0	0.0	0.0	0.0	0.0	0.0	0.0
0.0	0.0	0.0	0.0	0.0	0.0	0.0	0.0
0.0	0.0	0.0	0.0	0.0	0.0	0.0	0.0
0.0	0.0	0.0	0.0	0.0	0.0	0.0	0.0
0.0	0.0	0.0	0.0	0.0	0.0	0.0	0.0
0.0	0.0	0.0	0.0	0.0	0.0	0.0	0.0
0.0	0.0	0.0	0.0	0.0	0.0	0.0	0.0
0.0	0.0	0.0	0.0	0.0	0.0	0.0	0.0
0.0	0.0	0.0	0.0	0.0	0.0	0.0	0.0
0.0	0.0	0.0	0.0	0.0	0.0	0.0	0.0
0.0	0.0	0.0	0.0	0.0	0.0	0.0	0.0
0.0	0.0	0.0	0.0	0.0	0.0	0.0	0.0
0.0	0.0	0.0	0.0	0.0	0.0	0.0	0.0

0.0	0.0	0.0	0.0	0.0	0.0	0.0	0.0
0.0	0.0	0.0	0.0	0.0	0.0	0.0	0.0
0.0	0.0	0.0	0.0	0.0	0.0	0.0	0.0
0.0	0.0	0.0	0.0	0.0	0.0	0.0	0.0
0.0	0.0	0.0	0.0	0.0	0.0	0.0	0.0
0.0	0.0	0.0	0.0	0.0	0.0	0.0	0.0
0.0	0.0	0.0	0.0	0.0	0.0	0.0	0.0
0.0	0.0	0.0	0.0	0.0	0.0	0.0	0.0
0.0	0.0	0.0	0.0	0.0	0.0	0.0	0.0
0.0	0.0	0.0	0.0	0.0	0.0	0.0	0.0
0.0	0.0	0.0	0.0	0.0	0.0	0.0	0.0
0.0	0.0	0.0	0.0	0.0	0.0	0.0	0.0
0.0	0.0	0.0	0.0	0.0	0.0	0.0	0.0
0.0	0.0	0.0	0.0	0.0	0.0	0.0	0.0
0.0	0.0	0.0	0.0	0.0	0.0	0.0	0.0
0.0	0.0	0.0	0.0	0.0	0.0	0.0	0.0
0.0	0.0	0.0	0.0	0.0	0.0	0.0	0.0
0.0	0.0	0.0	0.0	0.0	0.0	0.0	0.0
0.0	0.0	0.0	0.0	0.0	0.0	0.0	0.0
0.0	0.0	0.0	0.0	0.0	0.0	0.0	0.0
0.0	0.0	0.0	0.0	0.0	0.0	0.0	0.0
0.0	0.0	0.0	0.0	0.0	0.0	0.0	0.0
0.0	0.0	0.0	0.0	0.0	0.0	0.0	0.0
0.0	0.0	0.0	0.0	0.0	0.0	0.0	0.0
0.0	0.0	0.0	0.0	0.0	0.0	0.0	0.0
0.0	0.0	0.0	0.0	0.0	0.0	0.0	0.0
0.0	0.0	0.0	0.0	0.0	0.0	0.0	0.0
0.0	0.0	0.0	0.0	0.0	0.0	0.0	0.0
0.0	0.0	0.0	0.0	0.0	0.0	0.0	0.0
0.0	0.0	0.0	0.0	0.0	0.0	0.0	0.0
0.0	0.0	0.0	0.0	0.0	0.0	0.0	0.0
0.0	0.0	0.0	0.0	0.0	0.0	0.0	0.0
0.0	0.0	0.0	0.0	0.0	0.0	0.0	0.0
0.0	0.0	0.0	0.0	0.0	0.0	0.0	0.0
0.0	0.0	0.0	0.0	0.0	0.0	0.0	0.0
0.0	0.0	0.0	0.0	0.0	0.0	0.0	0.0
0.0	0.0	0.0	0.0	0.0	0.0	0.0	0.0
0.0	0.0	0.0	0.0	0.0	0.0	0.0	0.0
0.0	0.0	0.0	0.0	0.0	0.0	0.0	0.0
0.0	0.0	0.0	0.0	0.0	0.0	0.0	0.0
0.0	0.0	0.0	0.0	0.0	0.0	0.0	0.0
0.0	0.0	0.0	0.0	0.0	0.0	0.0	0.0
0.0	0.0	0.0	0.0	0.0	0.0	0.0	0.0
0.0	0.0	0.0	0.0	0.0	0.0	0.0	0.0
0.0	0.0	0.0	0.0	0.0	0.0	0.0	0.0
0.0	0.0	0.0	0.0	0.0	0.0	0.0	0.0
0.0	0.0	0.0	0.0	0.0	0.0	0.0	0.0

0.0	0.0	0.0	0.0	0.0	0.0	0.0	0.0
0.0	0.0	0.0	0.0	0.0	0.0	0.0	0.0
0.0	0.0	0.0	0.0	0.0	0.0	0.0	0.0
0.0	0.0	0.0	0.0	0.0	0.0	0.0	0.0
0.0	0.0	0.0	0.0	0.0	0.0	0.0	0.0
0.0	0.0	0.0	0.0	0.0	0.0	0.0	0.0
0.0	0.0	0.0	0.0	0.0	0.0	0.0	0.0
0.0	0.0	0.0	0.0	0.0	0.0	0.0	0.0
0.0	0.0	0.0	0.0	0.0	0.0	0.0	0.0
0.0	0.0	0.0	0.0	0.0	0.0	0.0	0.0
0.0	0.0	0.0	0.0	0.0	0.0	0.0	0.0
0.0	0.0	0.0	0.0	0.0	0.0	0.0	0.0
0.0	0.0	0.0	0.0	0.0	0.0	0.0	0.0
0.0	0.0	0.0	0.0	0.0	0.0	0.0	0.0
0.0	0.0	0.0	0.0	0.0	0.0	0.0	0.0
0.0	0.0	0.0	0.0	0.0	0.0	0.0	0.0
0.0	0.0	0.0	0.0	0.0	0.0	0.0	0.0
0.0	0.0	0.0	0.0	0.0	0.0	0.0	0.0
0.0	0.0	0.0	0.0	0.0	0.0	0.0	0.0
0.0	0.0	0.0	0.0	0.0	0.0	0.0	0.0
0.0	0.0	0.0	0.0	0.0	0.0	0.0	0.0
0.0	0.0	0.0	0.0	0.0	0.0	0.0	0.0
0.0	0.0	0.0	0.0	0.0	0.0	0.0	0.0
0.0	0.0	0.0	0.0	0.0	0.0	0.0	0.0
0.0	0.0	0.0	0.0	0.0	0.0	0.0	0.0
0.0	0.0	0.0	0.0	0.0	0.0	0.0	0.0
0.0	0.0	0.0	0.0	0.0	0.0	0.0	0.0
0.0	0.0	0.0	0.0	0.0	0.0	0.0	0.0
0.0	0.0	0.0	0.0	0.0	0.0	0.0	0.0
0.0	0.0	0.0	0.0	0.0	0.0	0.0	0.0
0.0	0.0	0.0	0.0	0.0	0.0	0.0	0.0
0.0	0.0	0.0	0.0	0.0	0.0	0.0	0.0
0.0	0.0	0.0	0.0	0.0	0.0	0.0	0.0
0.0	0.0	0.0	0.0	0.0	0.0	0.0	0.0
0.0	0.0	0.0	0.0	0.0	0.0	0.0	0.0
0.0	0.0	0.0	0.0	0.0	0.0	0.0	0.0
0.0	0.0	0.0	0.0	0.0	0.0	0.0	0.0
0.0	0.0	0.0	0.0	0.0	0.0	0.0	0.0
0.0	0.0	0.0	0.0	0.0	0.0	0.0	0.0
0.0	0.0	0.0	0.0	0.0	0.0	0.0	0.0
0.0	0.0	0.0	0.0	0.0	0.0	0.0	0.0
0.0	0.0	0.0	0.0	0.0	0.0	0.0	0.0
0.0	0.0	0.0	0.0	0.0	0.0	0.0	0.0
0.0	0.0	0.0	0.0	0.0	0.0	0.0	0.0
0.0	0.0	0.0	0.0	0.0	0.0	0.0	0.0
0.0	0.0	0.0	0.0	0.0	0.0	0.0	0.0
0.0	0.0	0.0	0.0	0.0	0.0	0.0	0.0

0.0	0.0	0.0	0.0	0.0	0.0	0.0	0.0
0.0	0.0	0.0	0.0	0.0	0.0	0.0	0.0
0.0	0.0	0.0	0.0	0.0	0.0	0.0	0.0
0.0	0.0	0.0	0.0	0.0	0.0	0.0	0.0
0.0	0.0	0.0	0.0	0.0	0.0	0.0	0.0
0.0	0.0	0.0	0.0	0.0	0.0	0.0	0.0
0.0	0.0	0.0	0.0	0.0	0.0	0.0	0.0
0.0	0.0	0.0	0.0	0.0	0.0	0.0	0.0
0.0	0.0	0.0	0.0	0.0	0.0	0.0	0.0
0.0	0.0	0.0	0.0	0.0	0.0	0.0	0.0
0.0	0.0	0.0	0.0	0.0	0.0	0.0	0.0
0.0	0.0	0.0	0.0	0.0	0.0	0.0	0.0
0.0	0.0	0.0	0.0	0.0	0.0	0.0	0.0
0.0	0.0	0.0	0.0	0.0	0.0	0.0	0.0
0.0	0.0	0.0	0.0	0.0	0.0	0.0	0.0
0.0	0.0	0.0	0.0	0.0	0.0	0.0	0.0
0.0	0.0	0.0	0.0	0.0	0.0	0.0	0.0
0.0	0.0	0.0	0.0	0.0	0.0	0.0	0.0
0.0	0.0	0.0	0.0	0.0	0.0	0.0	0.0
0.0	0.0	0.0	0.0	0.0	0.0	0.0	0.0
0.0	0.0	0.0	0.0	0.0	0.0	0.0	0.0
0.0	0.0	0.0	0.0	0.0	0.0	0.0	0.0
0.0	0.0	0.0	0.0	0.0	0.0	0.0	0.0
0.0	0.0	0.0	0.0	0.0	0.0	0.0	0.0
0.0	0.0	0.0	0.0	0.0	0.0	0.0	0.0
0.0	0.0	0.0	0.0	0.0	0.0	0.0	0.0
0.0	0.0	0.0	0.0	0.0	0.0	0.0	0.0
0.0	0.0	0.0	0.0	0.0	0.0	0.0	0.0
0.0	0.0	0.0	0.0	0.0	0.0	0.0	0.0
0.0	0.0	0.0	0.0	0.0	0.0	0.0	0.0
0.0	0.0	0.0	0.0	0.0	0.0	0.0	0.0
0.0	0.0	0.0	0.0	0.0	0.0	0.0	0.0
0.0	0.0	0.0	0.0	0.0	0.0	0.0	0.0
0.0	0.0	0.0	0.0	0.0	0.0	0.0	0.0
0.0	0.0	0.0	0.0	0.0	0.0	0.0	0.0
0.0	0.0	0.0	0.0	0.0	0.0	0.0	0.0
0.0	0.0	0.0	0.0	0.0	0.0	0.0	0.0
0.0	0.0	0.0	0.0	0.0	0.0	0.0	0.0
0.0	0.0	0.0	0.0	0.0	0.0	0.0	0.0
0.0	0.0	0.0	0.0	0.0	0.0	0.0	0.0
0.0	0.0	0.0	0.0	0.0	0.0	0.0	0.0
0.0	0.0	0.0	0.0	0.0	0.0	0.0	0.0
0.0	0.0	0.0	0.0	0.0	0.0	0.0	0.0
0.0	0.0	0.0	0.0	0.0	0.0	0.0	0.0
0.0	0.0	0.0	0.0	0.0	0.0	0.0	0.0
0.0	0.0	0.0	0.0	0.0	0.0	0.0	0.0
0.0	0.0	0.0	0.0	0.0	0.0	0.0	0.0

0.0	0.0	0.0	0.0	0.0	0.0	0.0	0.0
0.0	0.0	0.0	0.0	0.0	0.0	0.0	0.0
0.0	0.0	0.0	0.0	0.0	0.0	0.0	0.0
0.0	0.0	0.0	0.0	0.0	0.0	0.0	0.0
0.0	0.0	0.0	0.0	0.0	0.0	0.0	0.0
0.0	0.0	0.0	0.0	0.0	0.0	0.0	0.0
0.0	0.0	0.0	0.0	0.0	0.0	0.0	0.0
0.0	0.0	0.0	0.0	0.0	0.0	0.0	0.0
0.0	0.0	0.0	0.0	0.0	0.0	0.0	0.0
0.0	0.0	0.0	0.0	0.0	0.0	0.0	0.0
0.0	0.0	0.0	0.0	0.0	0.0	0.0	0.0
0.0	0.0	0.0	0.0	0.0	0.0	0.0	0.0
0.0	0.0	0.0	0.0	0.0	0.0	0.0	0.0
0.0	0.0	0.0	0.0	0.0	0.0	0.0	0.0
0.0	0.0	0.0	0.0	0.0	0.0	0.0	0.0
0.0	0.0	0.0	0.0	0.0	0.0	0.0	0.0
0.0	0.0	0.0	0.0	0.0	0.0	0.0	0.0
0.0	0.0	0.0	0.0	0.0	0.0	0.0	0.0
0.0	0.0	0.0	0.0	0.0	0.0	0.0	0.0
0.0	0.0	0.0	0.0	0.0	0.0	0.0	0.0
0.0	0.0	0.0	0.0	0.0	0.0	0.0	0.0
0.0	0.0	0.0	0.0	0.0	0.0	0.0	0.0
0.0	0.0	0.0	0.0	0.0	0.0	0.0	0.0
0.0	0.0	0.0	0.0	0.0	0.0	0.0	0.0
0.0	0.0	0.0	0.0	0.0	0.0	0.0	0.0
0.0	0.0	0.0	0.0	0.0	0.0	0.0	0.0
0.0	0.0	0.0	0.0	0.0	0.0	0.0	0.0
0.0	0.0	0.0	0.0	0.0	0.0	0.0	0.0
0.0	0.0	0.0	0.0	0.0	0.0	0.0	0.0
0.0	0.0	0.0	0.0	0.0	0.0	0.0	0.0
0.0	0.0	0.0	0.0	0.0	0.0	0.0	0.0
0.0	0.0	0.0	0.0	0.0	0.0	0.0	0.0
0.0	0.0	0.0	0.0	0.0	0.0	0.0	0.0
0.0	0.0	0.0	0.0	0.0	0.0	0.0	0.0
0.0	0.0	0.0	0.0	0.0	0.0	0.0	0.0
0.0	0.0	0.0	0.0	0.0	0.0	0.0	0.0
0.0	0.0	0.0	0.0	0.0	0.0	0.0	0.0
0.0	0.0	0.0	0.0	0.0	0.0	0.0	0.0
0.0	0.0	0.0	0.0	0.0	0.0	0.0	0.0
0.0	0.0	0.0	0.0	0.0	0.0	0.0	0.0
0.0	0.0	0.0	0.0	0.0	0.0	0.0	0.0
0.0	0.0	0.0	0.0	0.0	0.0	0.0	0.0
0.0	0.0	0.0	0.0	0.0	0.0	0.0	0.0
0.0	0.0	0.0	0.0	0.0	0.0	0.0	0.0
0.0	0.0	0.0	0.0	0.0	0.0	0.0	0.0
0.0	0.0	0.0	0.0	0.0	0.0	0.0	0.0
0.0	0.0	0.0	0.0	0.0	0.0	0.0	0.0

0.0	0.0	0.0	0.0	0.0	0.0	0.0	0.0
0.0	0.0	0.0	0.0	0.0	0.0	0.0	0.0
0.0	0.0	0.0	0.0	0.0	0.0	0.0	0.0
0.0	0.0	0.0	0.0	0.0	0.0	0.0	0.0
0.0	0.0	0.0	0.0	0.0	0.0	0.0	0.0
0.0	0.0	0.0	0.0	0.0	0.0	0.0	0.0
0.0	0.0	0.0	0.0	0.0	0.0	0.0	0.0
0.0	0.0	0.0	0.0	0.0	0.0	0.0	0.0
0.0	0.0	0.0	0.0	0.0	0.0	0.0	0.0
0.0	0.0	0.0	0.0	0.0	0.0	0.0	0.0
0.0	0.0	0.0	0.0	0.0	0.0	0.0	0.0
0.0	0.0	0.0	0.0	0.0	0.0	0.0	0.0
0.0	0.0	0.0	0.0	0.0	0.0	0.0	0.0
0.0	0.0	0.0	0.0	0.0	0.0	0.0	0.0
0.0	0.0	0.0	0.0	0.0	0.0	0.0	0.0
0.0	0.0	0.0	0.0	0.0	0.0	0.0	0.0
0.0	0.0	0.0	0.0	0.0	0.0	0.0	0.0
0.0	0.0	0.0	0.0	0.0	0.0	0.0	0.0
0.0	0.0	0.0	0.0	0.0	0.0	0.0	0.0
0.0	0.0	0.0	0.0	0.0	0.0	0.0	0.0
0.0	0.0	0.0	0.0	0.0	0.0	0.0	0.0
0.0	0.0	0.0	0.0	0.0	0.0	0.0	0.0
0.0	0.0	0.0	0.0	0.0	0.0	0.0	0.0
0.0	0.0	0.0	0.0	0.0	0.0	0.0	0.0
0.0	0.0	0.0	0.0	0.0	0.0	0.0	0.0
0.0	0.0	0.0	0.0	0.0	0.0	0.0	0.0
0.0	0.0	0.0	0.0	0.0	0.0	0.0	0.0
0.0	0.0	0.0	0.0	0.0	0.0	0.0	0.0
0.0	0.0	0.0	0.0	0.0	0.0	0.0	0.0
0.0	0.0	0.0	0.0	0.0	0.0	0.0	0.0
0.0	0.0	0.0	0.0	0.0	0.0	0.0	0.0
0.0	0.0	0.0	0.0	0.0	0.0	0.0	0.0
0.0	0.0	0.0	0.0	0.0	0.0	0.0	0.0
0.0	0.0	0.0	0.0	0.0	0.0	0.0	0.0
0.0	0.0	0.0	0.0	0.0	0.0	0.0	0.0
0.0	0.0	0.0	0.0	0.0	0.0	0.0	0.0
0.0	0.0	0.0	0.0	0.0	0.0	0.0	0.0
0.0	0.0	0.0	0.0	0.0	0.0	0.0	0.0
0.0	0.0	0.0	0.0	0.0	0.0	0.0	0.0
0.0	0.0	0.0	0.0	0.0	0.0	0.0	0.0
0.0	0.0	0.0	0.0	0.0	0.0	0.0	0.0
0.0	0.0	0.0	0.0	0.0	0.0	0.0	0.0
0.0	0.0	0.0	0.0	0.0	0.0	0.0	0.0
0.0	0.0	0.0	0.0	0.0	0.0	0.0	0.0
0.0	0.0	0.0	0.0	0.0	0.0	0.0	0.0
0.0	0.0	0.0	0.0	0.0	0.0	0.0	0.0
0.0	0.0	0.0	0.0	0.0	0.0	0.0	0.0

0.0	0.0	0.0	0.0	0.0	0.0	0.0	0.0
0.0	0.0	0.0	0.0	0.0	0.0	0.0	0.0
0.0	0.0	0.0	0.0	0.0	0.0	0.0	0.0
0.0	0.0	0.0	0.0	0.0	0.0	0.0	0.0
0.0	0.0	0.0	0.0	0.0	0.0	0.0	0.0
0.0	0.0	0.0	0.0	0.0	0.0	0.0	0.0
0.0	0.0	0.0	0.0	0.0	0.0	0.0	0.0
0.0	0.0	0.0	0.0	0.0	0.0	0.0	0.0
0.0	0.0	0.0	0.0	0.0	0.0	0.0	0.0
0.0	0.0	0.0	0.0	0.0	0.0	0.0	0.0
0.0	0.0	0.0	0.0	0.0	0.0	0.0	0.0
0.0	0.0	0.0	0.0	0.0	0.0	0.0	0.0
0.0	0.0	0.0	0.0	0.0	0.0	0.0	0.0
0.0	0.0	0.0	0.0	0.0	0.0	0.0	0.0
0.0	0.0	0.0	0.0	0.0	0.0	0.0	0.0
0.0	0.0	0.0	0.0	0.0	0.0	0.0	0.0
0.0	0.0	0.0	0.0	0.0	0.0	0.0	0.0
0.0	0.0	0.0	0.0	0.0	0.0	0.0	0.0
0.0	0.0	0.0	0.0	0.0	0.0	0.0	0.0
0.0	0.0	0.0	0.0	0.0	0.0	0.0	0.0
0.0	0.0	0.0	0.0	0.0	0.0	0.0	0.0
0.0	0.0	0.0	0.0	0.0	0.0	0.0	0.0
0.0	0.0	0.0	0.0	0.0	0.0	0.0	0.0
0.0	0.0	0.0	0.0	0.0	0.0	0.0	0.0
0.0	0.0	0.0	0.0	0.0	0.0	0.0	0.0
0.0	0.0	0.0	0.0	0.0	0.0	0.0	0.0
0.0	0.0	0.0	0.0	0.0	0.0	0.0	0.0
0.0	0.0	0.0	0.0	0.0	0.0	0.0	0.0
0.0	0.0	0.0	0.0	0.0	0.0	0.0	0.0
0.0	0.0	0.0	0.0	0.0	0.0	0.0	0.0
0.0	0.0	0.0	0.0	0.0	0.0	0.0	0.0
0.0	0.0	0.0	0.0	0.0	0.0	0.0	0.0
0.0	0.0	0.0	0.0	0.0	0.0	0.0	0.0
0.0	0.0	0.0	0.0	0.0	0.0	0.0	0.0
0.0	0.0	0.0	0.0	0.0	0.0	0.0	0.0
0.0	0.0	0.0	0.0	0.0	0.0	0.0	0.0
0.0	0.0	0.0	0.0	0.0	0.0	0.0	0.0
0.0	0.0	0.0	0.0	0.0	0.0	0.0	0.0
0.0	0.0	0.0	0.0	0.0	0.0	0.0	0.0
0.0	0.0	0.0	0.0	0.0	0.0	0.0	0.0
0.0	0.0	0.0	0.0	0.0	0.0	0.0	0.0
0.0	0.0	0.0	0.0	0.0	0.0	0.0	0.0
0.0	0.0	0.0	0.0	0.0	0.0	0.0	0.0
0.0	0.0	0.0	0.0	0.0	0.0	0.0	0.0
0.0	0.0	0.0	0.0	0.0	0.0	0.0	0.0
0.0	0.0	0.0	0.0	0.0	0.0	0.0	0.0
0.0	0.0	0.0	0.0	0.0	0.0	0.0	0.0

0.0	0.0	0.0	0.0	0.0	0.0	0.0	0.0
0.0	0.0	0.0	0.0	0.0	0.0	0.0	0.0
0.0	0.0	0.0	0.0	0.0	0.0	0.0	0.0
0.0	0.0	0.0	0.0	0.0	0.0	0.0	0.0
0.0	0.0	0.0	0.0	0.0	0.0	0.0	0.0
0.0	0.0	0.0	0.0	0.0	0.0	0.0	0.0
0.0	0.0	0.0	0.0	0.0	0.0	0.0	0.0
0.0	0.0	0.0	0.0	0.0	0.0	0.0	0.0
0.0	0.0	0.0	0.0	0.0	0.0	0.0	0.0
0.0	0.0	0.0	0.0	0.0	0.0	0.0	0.0
0.0	0.0	0.0	0.0	0.0	0.0	0.0	0.0
0.0	0.0	0.0	0.0	0.0	0.0	0.0	0.0
0.0	0.0	0.0	0.0	0.0	0.0	0.0	0.0
0.0	0.0	0.0	0.0	0.0	0.0	0.0	0.0
0.0	0.0	0.0	0.0	0.0	0.0	0.0	0.0
0.0	0.0	0.0	0.0	0.0	0.0	0.0	0.0
0.0	0.0	0.0	0.0	0.0	0.0	0.0	0.0
0.0	0.0	0.0	0.0	0.0	0.0	0.0	0.0
0.0	0.0	0.0	0.0	0.0	0.0	0.0	0.0
0.0	0.0	0.0	0.0	0.0	0.0	0.0	0.0
0.0	0.0	0.0	0.0	0.0	0.0	0.0	0.0
0.0	0.0	0.0	0.0	0.0	0.0	0.0	0.0
0.0	0.0	0.0	0.0	0.0	0.0	0.0	0.0
0.0	0.0	0.0	0.0	0.0	0.0	0.0	0.0
0.0	0.0	0.0	0.0	0.0	0.0	0.0	0.0
0.0	0.0	0.0	0.0	0.0	0.0	0.0	0.0
0.0	0.0	0.0	0.0	0.0	0.0	0.0	0.0
0.0	0.0	0.0	0.0	0.0	0.0	0.0	0.0
0.0	0.0	0.0	0.0	0.0	0.0	0.0	0.0
0.0	0.0	0.0	0.0	0.0	0.0	0.0	0.0
0.0	0.0	0.0	0.0	0.0	0.0	0.0	0.0
0.0	0.0	0.0	0.0	0.0	0.0	0.0	0.0
0.0	0.0	0.0	0.0	0.0	0.0	0.0	0.0
0.0	0.0	0.0	0.0	0.0	0.0	0.0	0.0
0.0	0.0	0.0	0.0	0.0	0.0	0.0	0.0
0.0	0.0	0.0	0.0	0.0	0.0	0.0	0.0
0.0	0.0	0.0	0.0	0.0	0.0	0.0	0.0
0.0	0.0	0.0	0.0	0.0	0.0	0.0	0.0
0.0	0.0	0.0	0.0	0.0	0.0	0.0	0.0
0.0	0.0	0.0	0.0	0.0	0.0	0.0	0.0
0.0	0.0	0.0	0.0	0.0	0.0	0.0	0.0
0.0	0.0	0.0	0.0	0.0	0.0	0.0	0.0
0.0	0.0	0.0	0.0	0.0	0.0	0.0	0.0
0.0	0.0	0.0	0.0	0.0	0.0	0.0	0.0
0.0	0.0	0.0	0.0	0.0	0.0	0.0	0.0
0.0	0.0	0.0	0.0	0.0	0.0	0.0	0.0
0.0	0.0	0.0	0.0	0.0	0.0	0.0	0.0
0.0	0.0	0.0	0.0	0.0	0.0	0.0	0.0

0.0	0.0	0.0	0.0	0.0	0.0	0.0	0.0
0.0	0.0	0.0	0.0	0.0	0.0	0.0	0.0
0.0	0.0	0.0	0.0	0.0	0.0	0.0	0.0
0.0	0.0	0.0	0.0	0.0	0.0	0.0	0.0
0.0	0.0	0.0	0.0	0.0	0.0	0.0	0.0
0.0	0.0	0.0	0.0	0.0	0.0	0.0	0.0
0.0	0.0	0.0	0.0	0.0	0.0	0.0	0.0
0.0	0.0	0.0	0.0	0.0	0.0	0.0	0.0
0.0	0.0	0.0	0.0	0.0	0.0	0.0	0.0
0.0	0.0	0.0	0.0	0.0	0.0	0.0	0.0
0.0	0.0	0.0	0.0	0.0	0.0	0.0	0.0
0.0	0.0	0.0	0.0	0.0	0.0	0.0	0.0
0.0	0.0	0.0	0.0	0.0	0.0	0.0	0.0
0.0	0.0	0.0	0.0	0.0	0.0	0.0	0.0
0.0	0.0	0.0	0.0	0.0	0.0	0.0	0.0
0.0	0.0	0.0	0.0	0.0	0.0	0.0	0.0
0.0	0.0	0.0	0.0	0.0	0.0	0.0	0.0
0.0	0.0	0.0	0.0	0.0	0.0	0.0	0.0
0.0	0.0	0.0	0.0	0.0	0.0	0.0	0.0
0.0	0.0	0.0	0.0	0.0	0.0	0.0	0.0
0.0	0.0	0.0	0.0	0.0	0.0	0.0	0.0
0.0	0.0	0.0	0.0	0.0	0.0	0.0	0.0
0.0	0.0	0.0	0.0	0.0	0.0	0.0	0.0
28	56	73	81	60	32	73	49
0	0	1	0	1	0	1	1
24.8	27.1	37.6635931	35.81376274	26.6	21.6	28.7	24

11635.TW.CI.(	11635.TW.AY.	11635.TW.BS.	11635.TW.DY	11635.CIR7.0(	11635.CIR5.0(	11635.CIR51.(	11635.TW.CZ.
1.0	3.0	187.0	2.0	2.0	76.0	0.0	538.0
40.0	0.0	0.0	0.0	1.0	0.0	314.0	215.0
0.0	0.0	1.0	0.0	0.0	0.0	1.0	0.0
125.0	487.0	0.0	89.0	3.0	693.0	369.0	0.0
9.0	297.0	140.0	28.0	0.0	0.0	56.0	0.0
259.0	301.0	639.0	282.0	1.0	4.0	1270.0	648.0
0.0	0.0	0.0	0.0	0.0	0.0	0.0	0.0
0.0	18.0	0.0	0.0	0.0	0.0	0.0	0.0
0.0	112.0	0.0	0.0	0.0	0.0	0.0	0.0
1271.0	15.0	0.0	267.0	12.0	0.0	276.0	0.0
15.0	695.0	320.0	2.0	1.0	175.0	1.0	247.0
0.0	0.0	0.0	0.0	0.0	0.0	0.0	0.0
3.0	92.0	0.0	0.0	0.0	92.0	43.0	0.0
2.0	9.0	0.0	346.0	32.0	14.0	9.0	9.0
0.0	2.0	186.0	0.0	0.0	787.0	6.0	0.0
29.0	924.0	1648.0	18.0	3.0	1140.0	190.0	1902.0
1.0	37.0	0.0	4.0	0.0	18.0	34.0	46.0
3.0	0.0	0.0	2.0	3390.0	1.0	0.0	0.0
216.0	225.0	566.0	688.0	64.0	462.0	154.0	165.0
986.0	408.0	85.0	112.0	12.0	244.0	470.0	0.0
0.0	26.0	0.0	72.0	11.0	0.0	115.0	0.0
0.0	0.0	0.0	0.0	0.0	0.0	747.0	0.0
0.0	15.0	0.0	14.0	0.0	1.0	43.0	109.0
0.0	0.0	0.0	0.0	0.0	0.0	0.0	0.0
0.0	0.0	0.0	0.0	0.0	0.0	0.0	0.0
57.0	44.0	34.0	769.0	16.0	11.0	37.0	0.0
0.0	0.0	0.0	0.0	0.0	0.0	0.0	0.0
0.0	90.0	1.0	0.0	0.0	83.0	58.0	0.0
0.0	4.0	0.0	32.0	0.0	0.0	0.0	0.0
6.0	69.0	0.0	0.0	0.0	0.0	14.0	0.0
14.0	14.0	68.0	394.0	81.0	72.0	59.0	146.0
0.0	13.0	8.0	0.0	528.0	73.0	50.0	0.0
187.0	0.0	0.0	0.0	0.0	85.0	0.0	0.0
0.0	0.0	0.0	0.0	0.0	0.0	17.0	0.0
0.0	24.0	0.0	50.0	1822.0	34.0	23.0	32.0
0.0	0.0	0.0	0.0	0.0	0.0	5.0	2.0
0.0	0.0	0.0	0.0	0.0	0.0	0.0	0.0
61.0	32.0	145.0	248.0	425.0	54.0	162.0	246.0
0.0	0.0	0.0	0.0	0.0	0.0	841.0	0.0
16.0	38.0	228.0	143.0	64.0	0.0	27.0	0.0
4.0	24.0	33.0	0.0	0.0	38.0	16.0	115.0
0.0	243.0	0.0	3.0	0.0	0.0	83.0	18.0
0.0	14.0	32.0	243.0	67.0	6.0	11.0	0.0
247.0	168.0	1185.0	259.0	3.0	78.0	47.0	219.0
79.0	5.0	1.0	31.0	0.0	9.0	17.0	23.0
0.0	18.0	502.0	136.0	595.0	0.0	38.0	112.0

325.0	7.0	1.0	13.0	15.0	6.0	6.0	36.0
0.0	0.0	0.0	0.0	0.0	0.0	127.0	0.0
0.0	0.0	0.0	0.0	1.0	0.0	0.0	0.0
0.0	0.0	0.0	0.0	0.0	0.0	0.0	0.0
0.0	0.0	0.0	1.0	0.0	0.0	0.0	0.0
0.0	38.0	11.0	15.0	0.0	3.0	33.0	34.0
3.0	60.0	0.0	4.0	0.0	8.0	26.0	55.0
0.0	0.0	0.0	0.0	0.0	0.0	0.0	0.0
0.0	0.0	0.0	0.0	0.0	0.0	0.0	0.0
0.0	0.0	0.0	4.0	1.0	38.0	0.0	0.0
0.0	0.0	0.0	0.0	51.0	0.0	0.0	0.0
0.0	0.0	3.0	0.0	0.0	0.0	7.0	0.0
0.0	0.0	0.0	0.0	0.0	0.0	0.0	0.0
0.0	0.0	0.0	0.0	0.0	0.0	0.0	0.0
0.0	81.0	1.0	5.0	0.0	0.0	106.0	0.0
0.0	0.0	0.0	0.0	0.0	0.0	0.0	0.0
0.0	0.0	0.0	0.0	0.0	0.0	0.0	0.0
0.0	0.0	0.0	0.0	0.0	0.0	0.0	0.0
0.0	7.0	0.0	212.0	14.0	0.0	39.0	0.0
0.0	0.0	0.0	0.0	0.0	0.0	0.0	0.0
0.0	0.0	0.0	0.0	0.0	0.0	0.0	0.0
0.0	0.0	0.0	0.0	0.0	0.0	0.0	0.0
0.0	0.0	0.0	6.0	0.0	0.0	0.0	0.0
0.0	81.0	0.0	92.0	177.0	12.0	25.0	0.0
0.0	0.0	0.0	0.0	0.0	0.0	66.0	0.0
0.0	85.0	0.0	3.0	0.0	16.0	1.0	0.0
0.0	0.0	0.0	0.0	0.0	0.0	0.0	0.0
0.0	0.0	0.0	0.0	0.0	0.0	0.0	0.0
0.0	0.0	0.0	0.0	0.0	0.0	0.0	0.0
0.0	0.0	0.0	0.0	0.0	0.0	0.0	0.0
0.0	0.0	0.0	0.0	0.0	0.0	0.0	0.0
0.0	0.0	0.0	0.0	0.0	0.0	0.0	0.0
0.0	0.0	0.0	0.0	0.0	0.0	0.0	0.0
0.0	0.0	0.0	0.0	0.0	0.0	0.0	0.0
639.0	0.0	777.0	666.0	260.0	1.0	27.0	54.0
0.0	1289.0	0.0	0.0	0.0	0.0	0.0	0.0
0.0	5.0	0.0	0.0	0.0	0.0	0.0	4.0
0.0	0.0	0.0	0.0	0.0	0.0	0.0	0.0
0.0	0.0	0.0	346.0	2.0	0.0	123.0	0.0
0.0	29.0	0.0	0.0	0.0	8.0	23.0	0.0
0.0	0.0	0.0	0.0	0.0	0.0	0.0	0.0
0.0	0.0	0.0	0.0	0.0	0.0	0.0	0.0
0.0	91.0	46.0	0.0	0.0	183.0	120.0	0.0
48.0	7.0	0.0	0.0	0.0	0.0	63.0	42.0
0.0	173.0	0.0	0.0	0.0	2.0	66.0	0.0
0.0	0.0	0.0	0.0	0.0	0.0	0.0	0.0
78.0	17.0	0.0	1.0	0.0	0.0	17.0	0.0

73.0	113.0	14.0	134.0	3.0	0.0	29.0	57.0
0.0	0.0	0.0	3.0	0.0	0.0	5.0	0.0
913.0	24.0	0.0	13.0	494.0	51.0	42.0	0.0
0.0	2.0	0.0	0.0	0.0	0.0	42.0	0.0
0.0	3.0	0.0	0.0	0.0	0.0	0.0	83.0
0.0	0.0	0.0	0.0	0.0	0.0	0.0	0.0
0.0	255.0	0.0	6.0	0.0	0.0	10.0	0.0
0.0	0.0	0.0	0.0	0.0	0.0	0.0	0.0
41.0	201.0	0.0	0.0	0.0	173.0	5.0	0.0
0.0	0.0	0.0	0.0	0.0	0.0	0.0	0.0
0.0	0.0	0.0	0.0	0.0	0.0	0.0	0.0
0.0	0.0	0.0	0.0	0.0	0.0	0.0	0.0
0.0	0.0	0.0	0.0	0.0	0.0	0.0	0.0
0.0	0.0	0.0	0.0	0.0	0.0	0.0	0.0
0.0	23.0	51.0	2.0	0.0	0.0	12.0	0.0
0.0	0.0	0.0	0.0	0.0	0.0	0.0	0.0
0.0	0.0	0.0	0.0	0.0	0.0	0.0	0.0
2.0	0.0	0.0	1.0	0.0	0.0	0.0	0.0
0.0	0.0	0.0	0.0	0.0	0.0	2.0	0.0
0.0	0.0	0.0	0.0	0.0	0.0	0.0	0.0
511.0	1.0	0.0	71.0	7.0	0.0	20.0	0.0
48.0	52.0	0.0	0.0	0.0	0.0	0.0	0.0
0.0	33.0	0.0	0.0	0.0	18.0	10.0	12.0
0.0	0.0	0.0	0.0	0.0	0.0	0.0	0.0
0.0	76.0	0.0	9.0	0.0	0.0	0.0	0.0
0.0	0.0	0.0	0.0	0.0	0.0	0.0	0.0
0.0	0.0	0.0	0.0	0.0	0.0	0.0	0.0
0.0	0.0	0.0	0.0	0.0	0.0	0.0	0.0
0.0	29.0	5.0	29.0	0.0	0.0	88.0	505.0
0.0	0.0	0.0	0.0	0.0	0.0	0.0	349.0
0.0	0.0	0.0	0.0	0.0	0.0	0.0	0.0
0.0	0.0	0.0	0.0	0.0	0.0	0.0	0.0
0.0	0.0	0.0	0.0	0.0	0.0	0.0	0.0
0.0	0.0	0.0	0.0	0.0	0.0	0.0	0.0
0.0	0.0	0.0	0.0	0.0	0.0	0.0	0.0
0.0	20.0	0.0	0.0	0.0	0.0	23.0	0.0
0.0	0.0	0.0	0.0	0.0	0.0	0.0	0.0
0.0	0.0	0.0	23.0	0.0	0.0	8.0	0.0
32.0	0.0	0.0	60.0	0.0	71.0	67.0	583.0
0.0	0.0	0.0	0.0	0.0	0.0	0.0	0.0
0.0	0.0	0.0	0.0	0.0	0.0	0.0	0.0
0.0	0.0	0.0	6.0	1.0	647.0	11.0	0.0
0.0	0.0	0.0	0.0	0.0	0.0	0.0	0.0
0.0	0.0	0.0	0.0	0.0	0.0	0.0	0.0
0.0	94.0	22.0	1.0	0.0	0.0	2.0	66.0
0.0	0.0	0.0	0.0	0.0	0.0	0.0	0.0
0.0	12.0	38.0	1.0	0.0	27.0	11.0	38.0

0.0	0.0	0.0	0.0	0.0	0.0	0.0	0.0
0.0	4.0	0.0	0.0	0.0	0.0	0.0	0.0
0.0	0.0	0.0	0.0	0.0	0.0	0.0	0.0
0.0	0.0	0.0	0.0	0.0	0.0	0.0	0.0
0.0	0.0	0.0	0.0	0.0	0.0	15.0	0.0
0.0	0.0	0.0	15.0	0.0	0.0	26.0	0.0
0.0	0.0	0.0	0.0	0.0	0.0	0.0	0.0
0.0	17.0	0.0	0.0	0.0	0.0	41.0	0.0
0.0	2.0	4.0	0.0	0.0	0.0	4.0	6.0
0.0	0.0	0.0	0.0	0.0	0.0	0.0	0.0
0.0	15.0	34.0	0.0	1.0	157.0	13.0	0.0
0.0	3.0	0.0	0.0	0.0	0.0	0.0	8.0
11.0	4.0	0.0	62.0	14.0	0.0	11.0	0.0
0.0	0.0	0.0	10.0	0.0	74.0	0.0	0.0
0.0	0.0	0.0	0.0	10.0	0.0	0.0	0.0
0.0	0.0	0.0	0.0	0.0	0.0	0.0	0.0
0.0	0.0	0.0	0.0	0.0	0.0	0.0	0.0
0.0	0.0	7.0	0.0	0.0	14.0	0.0	0.0
67.0	9.0	0.0	0.0	193.0	39.0	0.0	0.0
0.0	1.0	37.0	1.0	0.0	7.0	0.0	0.0
3.0	0.0	83.0	0.0	0.0	589.0	2.0	0.0
0.0	77.0	2.0	0.0	0.0	0.0	3.0	0.0
0.0	2.0	0.0	0.0	0.0	0.0	4.0	0.0
0.0	1.0	0.0	5.0	0.0	0.0	1.0	0.0
0.0	0.0	0.0	0.0	0.0	0.0	0.0	0.0
0.0	0.0	0.0	0.0	0.0	8.0	23.0	14.0
0.0	0.0	0.0	0.0	0.0	0.0	3.0	0.0
0.0	0.0	1.0	0.0	0.0	0.0	0.0	0.0
0.0	0.0	0.0	2.0	0.0	0.0	0.0	0.0
0.0	0.0	0.0	0.0	0.0	0.0	0.0	0.0
147.0	3.0	2.0	40.0	0.0	0.0	20.0	4.0
0.0	0.0	0.0	0.0	0.0	0.0	2.0	0.0
0.0	1.0	0.0	0.0	0.0	0.0	3.0	0.0
0.0	0.0	0.0	0.0	0.0	0.0	0.0	0.0
0.0	8.0	0.0	2.0	0.0	0.0	14.0	22.0
6.0	0.0	0.0	8.0	0.0	0.0	0.0	0.0
57.0	0.0	0.0	0.0	0.0	0.0	0.0	2.0
0.0	11.0	0.0	0.0	0.0	0.0	5.0	0.0
5.0	6.0	17.0	3.0	0.0	12.0	8.0	18.0
35.0	15.0	34.0	0.0	0.0	58.0	3.0	0.0
0.0	27.0	2.0	85.0	0.0	0.0	1.0	8.0
0.0	34.0	3.0	1.0	0.0	46.0	20.0	0.0
0.0	21.0	0.0	0.0	0.0	3.0	18.0	0.0
0.0	0.0	0.0	0.0	0.0	0.0	3.0	0.0
20.0	0.0	92.0	2.0	0.0	9.0	0.0	130.0
0.0	0.0	0.0	6.0	0.0	0.0	7.0	0.0
0.0	0.0	0.0	0.0	0.0	0.0	0.0	0.0

0.0	0.0	0.0	0.0	0.0	0.0	0.0	0.0
0.0	0.0	0.0	0.0	0.0	0.0	0.0	0.0
0.0	0.0	0.0	0.0	0.0	0.0	0.0	0.0
0.0	164.0	0.0	0.0	0.0	0.0	2.0	0.0
0.0	2.0	0.0	0.0	0.0	0.0	0.0	0.0
0.0	0.0	0.0	0.0	0.0	0.0	0.0	0.0
0.0	4.0	0.0	0.0	7.0	0.0	0.0	0.0
0.0	0.0	17.0	0.0	0.0	73.0	0.0	13.0
2.0	17.0	28.0	1.0	0.0	39.0	3.0	8.0
0.0	0.0	0.0	0.0	0.0	0.0	0.0	0.0
0.0	0.0	0.0	0.0	0.0	0.0	6.0	2.0
0.0	13.0	0.0	0.0	0.0	0.0	1.0	0.0
3.0	1.0	0.0	0.0	0.0	11.0	0.0	12.0
93.0	0.0	527.0	69.0	0.0	128.0	0.0	12.0
0.0	2.0	0.0	0.0	0.0	0.0	0.0	15.0
7.0	0.0	0.0	20.0	12.0	0.0	3.0	0.0
0.0	0.0	0.0	0.0	0.0	0.0	0.0	0.0
0.0	0.0	0.0	0.0	0.0	0.0	0.0	0.0
0.0	0.0	0.0	0.0	0.0	0.0	0.0	0.0
0.0	0.0	0.0	0.0	0.0	0.0	1.0	5.0
0.0	0.0	12.0	43.0	0.0	4.0	0.0	0.0
0.0	0.0	0.0	0.0	0.0	1.0	0.0	0.0
0.0	0.0	0.0	0.0	0.0	0.0	0.0	0.0
0.0	5.0	0.0	7.0	2.0	4.0	19.0	0.0
0.0	0.0	0.0	0.0	0.0	0.0	0.0	0.0
0.0	0.0	0.0	0.0	0.0	0.0	0.0	0.0
23.0	0.0	0.0	0.0	0.0	0.0	0.0	0.0
0.0	0.0	0.0	0.0	0.0	0.0	0.0	0.0
0.0	0.0	0.0	0.0	0.0	0.0	0.0	0.0
0.0	0.0	0.0	0.0	0.0	0.0	0.0	0.0
0.0	0.0	0.0	0.0	0.0	0.0	0.0	0.0
0.0	0.0	0.0	0.0	0.0	0.0	0.0	0.0
0.0	0.0	0.0	0.0	0.0	0.0	0.0	0.0
0.0	0.0	0.0	0.0	0.0	0.0	0.0	0.0
0.0	0.0	0.0	0.0	0.0	0.0	0.0	0.0
0.0	0.0	0.0	0.0	0.0	0.0	0.0	0.0
0.0	0.0	0.0	0.0	0.0	0.0	0.0	0.0
0.0	0.0	0.0	0.0	0.0	0.0	0.0	0.0
0.0	0.0	0.0	0.0	0.0	0.0	0.0	0.0
0.0	0.0	0.0	0.0	0.0	0.0	0.0	0.0
6.0	0.0	11.0	10.0	0.0	4.0	20.0	9.0
0.0	0.0	0.0	0.0	0.0	0.0	0.0	0.0
0.0	54.0	0.0	0.0	0.0	0.0	0.0	0.0
0.0	0.0	0.0	0.0	0.0	0.0	0.0	0.0
0.0	0.0	0.0	0.0	0.0	0.0	0.0	0.0
0.0	0.0	0.0	0.0	0.0	0.0	0.0	0.0
0.0	0.0	0.0	0.0	0.0	0.0	0.0	0.0

0.0	0.0	0.0	0.0	0.0	0.0	0.0	0.0
0.0	6.0	0.0	0.0	0.0	0.0	6.0	0.0
35.0	2.0	1.0	0.0	0.0	0.0	1.0	0.0
0.0	4.0	0.0	0.0	0.0	0.0	3.0	0.0
0.0	0.0	0.0	0.0	0.0	0.0	0.0	0.0
0.0	0.0	0.0	0.0	5.0	0.0	0.0	0.0
0.0	1.0	0.0	0.0	0.0	0.0	3.0	0.0
0.0	0.0	0.0	0.0	0.0	0.0	0.0	0.0
0.0	0.0	0.0	0.0	0.0	0.0	0.0	0.0
0.0	0.0	0.0	0.0	0.0	0.0	0.0	0.0
0.0	0.0	0.0	0.0	0.0	0.0	0.0	0.0
0.0	0.0	0.0	0.0	0.0	0.0	0.0	0.0
0.0	0.0	0.0	0.0	0.0	0.0	0.0	16.0
0.0	0.0	1.0	0.0	0.0	0.0	0.0	0.0
0.0	1.0	0.0	0.0	0.0	0.0	0.0	0.0
0.0	0.0	0.0	0.0	0.0	0.0	0.0	0.0
0.0	0.0	0.0	0.0	0.0	0.0	0.0	0.0
0.0	0.0	0.0	0.0	0.0	0.0	0.0	0.0
0.0	0.0	0.0	0.0	0.0	0.0	3.0	5.0
0.0	0.0	0.0	0.0	0.0	0.0	0.0	0.0
0.0	0.0	0.0	0.0	0.0	0.0	2.0	0.0
0.0	0.0	0.0	0.0	0.0	0.0	8.0	0.0
2.0	0.0	0.0	0.0	0.0	0.0	0.0	30.0
0.0	0.0	0.0	0.0	0.0	0.0	0.0	0.0
0.0	0.0	0.0	0.0	0.0	2.0	1.0	0.0
0.0	0.0	0.0	0.0	0.0	0.0	0.0	0.0
56.0	0.0	2.0	0.0	0.0	0.0	0.0	2.0
0.0	0.0	0.0	0.0	0.0	0.0	0.0	0.0
0.0	0.0	0.0	0.0	0.0	0.0	0.0	0.0
0.0	0.0	0.0	0.0	0.0	0.0	0.0	0.0
0.0	0.0	0.0	0.0	0.0	0.0	2.0	0.0
9.0	0.0	0.0	15.0	0.0	0.0	0.0	2.0
0.0	0.0	0.0	0.0	0.0	0.0	0.0	0.0
0.0	0.0	0.0	0.0	0.0	0.0	0.0	0.0
0.0	0.0	0.0	0.0	0.0	0.0	0.0	0.0
0.0	2.0	0.0	7.0	0.0	0.0	0.0	0.0
0.0	0.0	0.0	0.0	0.0	0.0	0.0	0.0
0.0	0.0	0.0	0.0	0.0	0.0	0.0	0.0
0.0	0.0	0.0	0.0	0.0	0.0	0.0	0.0
0.0	0.0	0.0	0.0	0.0	0.0	0.0	0.0
0.0	0.0	0.0	1.0	0.0	0.0	0.0	0.0
0.0	0.0	0.0	0.0	0.0	0.0	0.0	0.0
2.0	3.0	4.0	1.0	0.0	25.0	0.0	5.0
0.0	0.0	0.0	0.0	0.0	35.0	0.0	18.0
0.0	0.0	0.0	0.0	0.0	0.0	0.0	0.0
0.0	0.0	0.0	0.0	0.0	0.0	0.0	0.0
0.0	0.0	0.0	0.0	0.0	0.0	0.0	0.0

62.0	0.0	0.0	20.0	0.0	0.0	49.0	0.0
0.0	0.0	0.0	0.0	0.0	0.0	0.0	0.0
0.0	0.0	0.0	0.0	0.0	0.0	0.0	0.0
0.0	0.0	0.0	0.0	0.0	0.0	0.0	0.0
0.0	0.0	0.0	0.0	0.0	0.0	0.0	4.0
0.0	0.0	0.0	0.0	0.0	0.0	3.0	3.0
0.0	2.0	0.0	0.0	0.0	0.0	0.0	0.0
0.0	0.0	0.0	0.0	0.0	0.0	0.0	0.0
0.0	0.0	0.0	1.0	0.0	0.0	0.0	0.0
0.0	15.0	0.0	0.0	0.0	0.0	0.0	0.0
0.0	0.0	8.0	0.0	0.0	0.0	0.0	1.0
0.0	0.0	0.0	0.0	0.0	0.0	0.0	0.0
0.0	0.0	0.0	0.0	0.0	0.0	0.0	0.0
0.0	0.0	0.0	0.0	0.0	0.0	0.0	0.0
0.0	0.0	0.0	0.0	0.0	0.0	0.0	0.0
188.0	11.0	3.0	25.0	0.0	0.0	9.0	13.0
0.0	0.0	0.0	0.0	0.0	0.0	0.0	0.0
0.0	0.0	0.0	0.0	0.0	0.0	0.0	0.0
0.0	0.0	0.0	0.0	0.0	0.0	0.0	0.0
0.0	1.0	0.0	0.0	0.0	0.0	0.0	3.0
0.0	0.0	0.0	0.0	0.0	0.0	0.0	0.0
0.0	0.0	0.0	0.0	0.0	0.0	0.0	0.0
0.0	0.0	0.0	0.0	0.0	0.0	0.0	0.0
0.0	0.0	0.0	0.0	0.0	0.0	0.0	0.0
16.0	0.0	0.0	12.0	0.0	2.0	9.0	0.0
0.0	0.0	0.0	0.0	0.0	0.0	0.0	0.0
11.0	0.0	0.0	0.0	3.0	1.0	5.0	0.0
7.0	0.0	5.0	0.0	0.0	0.0	0.0	0.0
0.0	0.0	0.0	0.0	0.0	0.0	0.0	0.0
0.0	0.0	0.0	0.0	0.0	0.0	0.0	0.0
0.0	0.0	0.0	0.0	0.0	0.0	0.0	0.0
0.0	0.0	0.0	0.0	0.0	0.0	0.0	0.0
0.0	0.0	0.0	0.0	0.0	0.0	0.0	0.0
0.0	1.0	0.0	0.0	0.0	1.0	2.0	6.0
0.0	2.0	0.0	0.0	0.0	0.0	0.0	3.0
0.0	0.0	0.0	0.0	0.0	0.0	0.0	0.0
0.0	0.0	0.0	0.0	0.0	0.0	0.0	0.0
0.0	0.0	0.0	0.0	0.0	0.0	0.0	0.0
0.0	0.0	0.0	0.0	0.0	0.0	0.0	0.0
0.0	0.0	0.0	0.0	0.0	0.0	0.0	5.0
0.0	2.0	0.0	0.0	0.0	0.0	0.0	1.0
0.0	0.0	0.0	0.0	0.0	0.0	0.0	0.0
0.0	0.0	0.0	0.0	0.0	0.0	0.0	0.0
0.0	0.0	2.0	0.0	0.0	0.0	0.0	0.0
0.0	0.0	0.0	0.0	0.0	0.0	0.0	0.0
0.0	0.0	0.0	0.0	0.0	0.0	0.0	0.0
0.0	0.0	0.0	0.0	0.0	0.0	0.0	7.0

0.0	0.0	0.0	0.0	0.0	0.0	0.0	0.0
0.0	0.0	0.0	0.0	0.0	0.0	0.0	0.0
0.0	0.0	0.0	0.0	0.0	0.0	0.0	0.0
0.0	0.0	0.0	0.0	0.0	0.0	0.0	0.0
0.0	0.0	0.0	0.0	0.0	0.0	0.0	1.0
0.0	0.0	0.0	0.0	0.0	0.0	0.0	0.0
0.0	0.0	0.0	0.0	0.0	0.0	0.0	0.0
0.0	0.0	0.0	0.0	0.0	0.0	0.0	0.0
21.0	0.0	7.0	0.0	0.0	6.0	0.0	0.0
0.0	0.0	0.0	0.0	0.0	0.0	0.0	0.0
0.0	0.0	0.0	0.0	0.0	0.0	0.0	0.0
0.0	0.0	0.0	0.0	0.0	0.0	0.0	0.0
0.0	0.0	0.0	0.0	0.0	0.0	0.0	0.0
0.0	0.0	0.0	0.0	0.0	0.0	0.0	0.0
0.0	0.0	0.0	0.0	0.0	0.0	0.0	0.0
0.0	0.0	0.0	0.0	0.0	0.0	0.0	0.0
1.0	0.0	0.0	2.0	0.0	0.0	1.0	18.0
0.0	0.0	0.0	0.0	0.0	0.0	0.0	0.0
0.0	0.0	0.0	0.0	0.0	0.0	0.0	0.0
0.0	0.0	0.0	0.0	0.0	0.0	0.0	0.0
0.0	0.0	0.0	0.0	0.0	0.0	0.0	0.0
0.0	0.0	0.0	0.0	0.0	0.0	0.0	0.0
0.0	0.0	0.0	0.0	0.0	0.0	0.0	0.0
0.0	0.0	0.0	0.0	0.0	0.0	0.0	0.0
0.0	0.0	0.0	0.0	0.0	0.0	0.0	0.0
0.0	0.0	0.0	0.0	0.0	0.0	0.0	0.0
0.0	0.0	0.0	0.0	0.0	0.0	0.0	0.0
0.0	0.0	26.0	0.0	2.0	78.0	1.0	15.0
0.0	0.0	0.0	0.0	0.0	0.0	0.0	0.0
0.0	0.0	0.0	0.0	0.0	0.0	0.0	0.0
0.0	0.0	0.0	0.0	0.0	0.0	2.0	0.0
0.0	0.0	0.0	0.0	0.0	0.0	0.0	0.0
0.0	0.0	0.0	0.0	0.0	0.0	0.0	0.0
0.0	0.0	0.0	0.0	0.0	0.0	0.0	0.0
33.0	0.0	23.0	0.0	0.0	0.0	0.0	0.0
0.0	0.0	0.0	0.0	0.0	0.0	0.0	0.0
0.0	0.0	0.0	0.0	0.0	0.0	0.0	0.0
0.0	0.0	0.0	0.0	0.0	0.0	0.0	0.0
39.0	0.0	0.0	1.0	0.0	0.0	0.0	0.0
0.0	0.0	0.0	0.0	0.0	0.0	1.0	0.0
0.0	0.0	0.0	0.0	0.0	0.0	0.0	0.0
0.0	0.0	0.0	0.0	0.0	0.0	2.0	0.0
0.0	0.0	0.0	0.0	0.0	0.0	0.0	0.0
0.0	0.0	0.0	0.0	0.0	0.0	0.0	0.0
0.0	0.0	0.0	1.0	1.0	0.0	0.0	0.0
0.0	2.0	0.0	1.0	0.0	0.0	0.0	3.0
0.0	1.0	0.0	0.0	0.0	0.0	0.0	0.0

0.0	0.0	4.0	2.0	0.0	7.0	0.0	2.0
0.0	43.0	0.0	0.0	0.0	0.0	28.0	0.0
0.0	0.0	0.0	0.0	0.0	0.0	0.0	0.0
3.0	0.0	0.0	0.0	0.0	1.0	1.0	0.0
0.0	0.0	0.0	0.0	0.0	0.0	0.0	0.0
4.0	0.0	0.0	0.0	0.0	5.0	0.0	846.0
0.0	0.0	0.0	0.0	0.0	0.0	0.0	0.0
4.0	1.0	0.0	0.0	0.0	0.0	0.0	0.0
0.0	0.0	0.0	0.0	0.0	0.0	0.0	0.0
0.0	0.0	0.0	0.0	0.0	0.0	0.0	0.0
0.0	1.0	0.0	0.0	0.0	0.0	9.0	0.0
192.0	0.0	0.0	10.0	0.0	0.0	0.0	1.0
0.0	0.0	0.0	0.0	0.0	0.0	0.0	0.0
0.0	0.0	0.0	0.0	0.0	0.0	0.0	0.0
0.0	0.0	0.0	0.0	0.0	0.0	0.0	0.0
0.0	0.0	0.0	0.0	0.0	0.0	0.0	0.0
0.0	6.0	0.0	2.0	0.0	0.0	2.0	0.0
2.0	0.0	0.0	0.0	0.0	0.0	3.0	0.0
0.0	0.0	0.0	0.0	0.0	0.0	0.0	0.0
0.0	0.0	0.0	0.0	0.0	0.0	0.0	0.0
0.0	0.0	0.0	0.0	0.0	0.0	0.0	0.0
0.0	0.0	0.0	0.0	0.0	0.0	0.0	0.0
0.0	0.0	0.0	0.0	0.0	0.0	0.0	0.0
0.0	69.0	0.0	0.0	6.0	0.0	0.0	183.0
0.0	0.0	0.0	1.0	0.0	0.0	3.0	0.0
0.0	0.0	0.0	0.0	0.0	0.0	0.0	0.0
0.0	0.0	0.0	0.0	0.0	0.0	0.0	0.0
0.0	0.0	0.0	0.0	0.0	0.0	0.0	0.0
0.0	3.0	0.0	0.0	0.0	0.0	0.0	0.0
0.0	3.0	0.0	2.0	0.0	0.0	0.0	0.0
0.0	0.0	0.0	0.0	0.0	0.0	0.0	0.0
0.0	0.0	0.0	0.0	0.0	0.0	0.0	0.0
0.0	0.0	0.0	0.0	0.0	0.0	0.0	0.0
0.0	0.0	0.0	0.0	0.0	0.0	0.0	0.0
0.0	0.0	0.0	0.0	0.0	0.0	0.0	0.0
0.0	0.0	0.0	0.0	0.0	0.0	0.0	0.0
0.0	0.0	0.0	0.0	0.0	0.0	0.0	22.0
0.0	0.0	0.0	0.0	0.0	0.0	0.0	0.0
52.0	0.0	53.0	0.0	0.0	0.0	0.0	0.0
0.0	0.0	0.0	0.0	0.0	0.0	0.0	0.0
0.0	0.0	0.0	0.0	0.0	0.0	0.0	0.0
0.0	0.0	423.0	10.0	0.0	0.0	30.0	0.0
0.0	0.0	0.0	0.0	0.0	0.0	0.0	0.0
0.0	0.0	0.0	0.0	0.0	0.0	0.0	0.0
0.0	0.0	0.0	0.0	0.0	0.0	0.0	0.0
0.0	0.0	0.0	0.0	0.0	0.0	0.0	0.0
0.0	0.0	3.0	3.0	0.0	1.0	2.0	0.0

0.0	0.0	0.0	0.0	0.0	0.0	0.0	0.0
0.0	0.0	0.0	0.0	0.0	1.0	0.0	2.0
0.0	2.0	0.0	0.0	0.0	1.0	57.0	0.0
21.0	0.0	0.0	1.0	0.0	1.0	0.0	0.0
0.0	0.0	0.0	0.0	0.0	0.0	0.0	0.0
0.0	0.0	0.0	0.0	0.0	0.0	0.0	0.0
0.0	0.0	0.0	0.0	0.0	0.0	0.0	0.0
0.0	3.0	1.0	3.0	0.0	0.0	0.0	0.0
0.0	0.0	0.0	0.0	0.0	0.0	0.0	0.0
0.0	0.0	0.0	0.0	0.0	0.0	0.0	0.0
0.0	0.0	2.0	2.0	0.0	0.0	0.0	0.0
0.0	0.0	0.0	0.0	0.0	0.0	0.0	0.0
0.0	2.0	0.0	2.0	0.0	5.0	0.0	0.0
0.0	0.0	0.0	0.0	0.0	0.0	0.0	0.0
0.0	0.0	0.0	0.0	0.0	0.0	0.0	0.0
1.0	0.0	0.0	0.0	0.0	0.0	0.0	0.0
0.0	0.0	0.0	0.0	0.0	0.0	0.0	0.0
0.0	0.0	0.0	0.0	0.0	0.0	0.0	0.0
0.0	0.0	0.0	0.0	0.0	0.0	0.0	0.0
6.0	0.0	0.0	0.0	0.0	0.0	0.0	0.0
0.0	6.0	3.0	0.0	0.0	38.0	1.0	0.0
0.0	0.0	6.0	0.0	0.0	0.0	0.0	0.0
1.0	0.0	0.0	0.0	0.0	0.0	0.0	0.0
0.0	0.0	0.0	0.0	0.0	0.0	0.0	0.0
0.0	0.0	0.0	0.0	0.0	0.0	0.0	0.0
0.0	0.0	0.0	10.0	0.0	617.0	0.0	0.0
0.0	0.0	0.0	0.0	0.0	0.0	0.0	0.0
0.0	0.0	0.0	0.0	0.0	0.0	0.0	0.0
0.0	0.0	0.0	367.0	0.0	0.0	0.0	0.0
0.0	0.0	0.0	0.0	0.0	0.0	0.0	0.0
0.0	0.0	0.0	0.0	0.0	0.0	2.0	0.0
0.0	2.0	0.0	0.0	0.0	0.0	0.0	0.0
0.0	0.0	0.0	0.0	0.0	0.0	0.0	0.0
0.0	0.0	0.0	0.0	0.0	0.0	0.0	0.0
0.0	0.0	0.0	0.0	0.0	0.0	1.0	0.0
0.0	0.0	0.0	0.0	0.0	0.0	0.0	0.0
0.0	0.0	0.0	3.0	0.0	3.0	14.0	0.0
0.0	0.0	0.0	0.0	0.0	0.0	0.0	19.0
0.0	2.0	0.0	31.0	32.0	0.0	26.0	0.0
0.0	0.0	0.0	1.0	0.0	0.0	0.0	0.0
0.0	0.0	0.0	0.0	0.0	0.0	0.0	0.0
0.0	0.0	0.0	0.0	0.0	0.0	0.0	0.0
0.0	0.0	0.0	0.0	0.0	0.0	3.0	0.0
0.0	0.0	0.0	0.0	0.0	0.0	0.0	0.0
1.0	14.0	0.0	0.0	0.0	0.0	0.0	0.0
0.0	0.0	0.0	0.0	0.0	1.0	0.0	1.0
0.0	0.0	0.0	13.0	0.0	0.0	9.0	0.0

0.0	0.0	0.0	0.0	0.0	0.0	0.0	0.0
0.0	0.0	0.0	0.0	0.0	0.0	0.0	6.0
0.0	0.0	0.0	0.0	0.0	0.0	0.0	0.0
49.0	4.0	0.0	0.0	0.0	0.0	5.0	0.0
0.0	2.0	0.0	0.0	0.0	0.0	0.0	5.0
0.0	0.0	0.0	0.0	0.0	0.0	5.0	7.0
0.0	9.0	0.0	0.0	0.0	0.0	0.0	0.0
0.0	0.0	0.0	0.0	0.0	0.0	0.0	0.0
0.0	0.0	0.0	0.0	0.0	0.0	0.0	0.0
0.0	0.0	0.0	0.0	0.0	0.0	0.0	0.0
0.0	0.0	0.0	0.0	0.0	0.0	0.0	0.0
1.0	0.0	0.0	0.0	0.0	0.0	0.0	0.0
0.0	0.0	2.0	0.0	0.0	1.0	1.0	0.0
2.0	3.0	5.0	47.0	0.0	0.0	12.0	1.0
0.0	3.0	1.0	1.0	0.0	0.0	0.0	1.0
0.0	0.0	0.0	0.0	0.0	0.0	0.0	0.0
0.0	0.0	0.0	0.0	0.0	0.0	0.0	0.0
0.0	0.0	0.0	0.0	0.0	0.0	0.0	1.0
0.0	0.0	0.0	0.0	0.0	0.0	0.0	0.0
0.0	0.0	0.0	0.0	0.0	0.0	0.0	2.0
0.0	0.0	0.0	0.0	0.0	0.0	0.0	0.0
3.0	2.0	0.0	0.0	0.0	0.0	0.0	0.0
0.0	0.0	0.0	0.0	0.0	0.0	0.0	0.0
0.0	0.0	0.0	0.0	0.0	0.0	0.0	0.0
0.0	1.0	0.0	0.0	0.0	8.0	0.0	1.0
0.0	0.0	0.0	0.0	0.0	0.0	0.0	0.0
0.0	1.0	0.0	0.0	0.0	0.0	0.0	0.0
0.0	0.0	1.0	0.0	0.0	0.0	0.0	11.0
0.0	0.0	0.0	0.0	0.0	0.0	0.0	0.0
0.0	0.0	0.0	0.0	0.0	0.0	0.0	0.0
0.0	0.0	0.0	0.0	0.0	0.0	0.0	0.0
0.0	1.0	2.0	0.0	0.0	0.0	0.0	0.0
0.0	0.0	0.0	0.0	0.0	0.0	0.0	0.0
0.0	0.0	0.0	0.0	0.0	0.0	0.0	0.0
0.0	0.0	0.0	0.0	0.0	0.0	0.0	0.0
0.0	0.0	0.0	0.0	0.0	0.0	0.0	0.0
0.0	0.0	0.0	0.0	0.0	0.0	0.0	0.0
0.0	0.0	0.0	0.0	0.0	0.0	0.0	0.0
0.0	0.0	0.0	15.0	0.0	0.0	0.0	0.0
0.0	0.0	0.0	0.0	0.0	0.0	0.0	0.0
0.0	0.0	0.0	0.0	0.0	0.0	0.0	0.0
0.0	0.0	0.0	0.0	0.0	0.0	0.0	0.0
0.0	0.0	0.0	0.0	0.0	0.0	0.0	0.0
0.0	0.0	0.0	0.0	0.0	0.0	0.0	0.0
0.0	0.0	0.0	0.0	0.0	2.0	0.0	0.0
0.0	0.0	0.0	0.0	0.0	0.0	0.0	0.0
0.0	0.0	0.0	0.0	0.0	0.0	0.0	0.0

0.0	0.0	14.0	0.0	0.0	6.0	0.0	5.0
0.0	0.0	0.0	0.0	0.0	0.0	0.0	0.0
0.0	0.0	0.0	0.0	0.0	0.0	0.0	0.0
3.0	0.0	0.0	1.0	3.0	2.0	0.0	0.0
0.0	3.0	0.0	0.0	0.0	13.0	0.0	7.0
0.0	0.0	0.0	0.0	0.0	0.0	0.0	0.0
0.0	0.0	0.0	0.0	1.0	0.0	0.0	0.0
0.0	0.0	0.0	0.0	0.0	0.0	0.0	0.0
0.0	0.0	0.0	0.0	0.0	0.0	0.0	0.0
0.0	0.0	0.0	0.0	0.0	0.0	0.0	0.0
0.0	0.0	0.0	0.0	0.0	0.0	0.0	0.0
0.0	0.0	0.0	0.0	0.0	0.0	0.0	0.0
0.0	0.0	0.0	0.0	0.0	0.0	0.0	0.0
0.0	0.0	0.0	0.0	0.0	0.0	1.0	0.0
0.0	0.0	0.0	0.0	0.0	0.0	0.0	0.0
0.0	0.0	0.0	0.0	0.0	0.0	0.0	0.0
0.0	0.0	0.0	0.0	0.0	0.0	0.0	0.0
0.0	0.0	0.0	0.0	0.0	0.0	0.0	0.0
0.0	0.0	0.0	0.0	0.0	0.0	0.0	0.0
0.0	0.0	0.0	0.0	0.0	0.0	2.0	49.0
0.0	0.0	0.0	0.0	0.0	0.0	0.0	0.0
0.0	0.0	0.0	0.0	0.0	0.0	0.0	0.0
0.0	0.0	0.0	0.0	0.0	0.0	0.0	0.0
0.0	0.0	0.0	0.0	0.0	0.0	0.0	0.0
0.0	0.0	0.0	0.0	0.0	0.0	0.0	0.0
0.0	0.0	0.0	0.0	0.0	0.0	0.0	0.0
0.0	0.0	0.0	0.0	0.0	0.0	0.0	0.0
0.0	0.0	0.0	0.0	0.0	0.0	0.0	0.0
0.0	0.0	0.0	0.0	0.0	0.0	0.0	0.0
0.0	0.0	0.0	0.0	0.0	0.0	0.0	0.0
0.0	0.0	0.0	0.0	0.0	0.0	0.0	0.0
0.0	0.0	0.0	0.0	0.0	0.0	0.0	0.0
0.0	0.0	0.0	0.0	0.0	0.0	0.0	0.0
0.0	0.0	0.0	0.0	0.0	0.0	0.0	0.0
0.0	0.0	0.0	9.0	0.0	0.0	0.0	0.0
0.0	0.0	0.0	0.0	0.0	0.0	0.0	0.0
0.0	0.0	0.0	0.0	0.0	0.0	0.0	0.0
0.0	0.0	0.0	0.0	0.0	0.0	0.0	0.0
0.0	0.0	0.0	0.0	0.0	0.0	0.0	0.0
0.0	0.0	0.0	0.0	0.0	0.0	0.0	0.0
0.0	0.0	0.0	0.0	0.0	0.0	0.0	0.0
0.0	0.0	0.0	0.0	0.0	0.0	0.0	0.0
0.0	0.0	0.0	0.0	0.0	0.0	0.0	0.0
0.0	0.0	0.0	0.0	0.0	0.0	0.0	0.0
0.0	0.0	0.0	0.0	0.0	0.0	0.0	0.0
0.0	0.0	0.0	0.0	0.0	0.0	0.0	0.0
0.0	0.0	0.0	0.0	0.0	0.0	0.0	0.0

0.0	0.0	0.0	0.0	0.0	0.0	0.0	0.0
0.0	0.0	0.0	0.0	0.0	0.0	0.0	0.0
0.0	0.0	0.0	0.0	0.0	0.0	0.0	0.0
0.0	0.0	0.0	0.0	0.0	0.0	0.0	0.0
0.0	0.0	0.0	0.0	0.0	0.0	0.0	0.0
0.0	0.0	0.0	0.0	0.0	0.0	0.0	0.0
0.0	0.0	18.0	102.0	4.0	57.0	0.0	24.0
74.0	0.0	7.0	4.0	2.0	6.0	0.0	8.0
5.0	0.0	25.0	0.0	0.0	44.0	0.0	2.0
0.0	0.0	0.0	0.0	0.0	0.0	0.0	0.0
0.0	0.0	0.0	0.0	15.0	0.0	0.0	1.0
0.0	0.0	0.0	0.0	0.0	0.0	0.0	0.0
0.0	0.0	0.0	0.0	0.0	0.0	0.0	3.0
4.0	5.0	0.0	1.0	0.0	11.0	0.0	7.0
0.0	0.0	0.0	0.0	0.0	0.0	0.0	0.0
0.0	0.0	0.0	0.0	0.0	0.0	0.0	2.0
0.0	0.0	2.0	0.0	3.0	0.0	0.0	0.0
0.0	0.0	0.0	0.0	0.0	0.0	0.0	0.0
1.0	0.0	0.0	0.0	0.0	0.0	0.0	0.0
0.0	0.0	0.0	0.0	0.0	0.0	0.0	0.0
0.0	0.0	0.0	0.0	0.0	0.0	71.0	0.0
0.0	0.0	0.0	0.0	0.0	0.0	0.0	0.0
3.0	0.0	0.0	0.0	0.0	0.0	0.0	0.0
1.0	0.0	0.0	0.0	0.0	0.0	0.0	0.0
0.0	0.0	0.0	0.0	0.0	0.0	0.0	0.0
0.0	0.0	0.0	0.0	0.0	0.0	0.0	0.0
4.0	0.0	0.0	0.0	0.0	0.0	0.0	0.0
0.0	0.0	0.0	0.0	0.0	0.0	0.0	0.0
0.0	0.0	0.0	0.0	0.0	0.0	0.0	0.0
0.0	0.0	0.0	0.0	0.0	0.0	0.0	0.0
0.0	0.0	0.0	0.0	0.0	0.0	0.0	0.0
0.0	0.0	0.0	0.0	0.0	0.0	0.0	0.0
0.0	0.0	0.0	0.0	0.0	0.0	0.0	0.0
0.0	0.0	0.0	0.0	0.0	0.0	0.0	0.0
0.0	0.0	0.0	0.0	0.0	0.0	0.0	0.0
0.0	0.0	0.0	0.0	0.0	0.0	0.0	0.0
0.0	0.0	0.0	0.0	0.0	0.0	0.0	0.0
0.0	0.0	0.0	0.0	0.0	0.0	0.0	0.0
0.0	0.0	0.0	0.0	0.0	0.0	0.0	0.0
0.0	0.0	0.0	0.0	2.0	0.0	0.0	0.0
0.0	0.0	0.0	0.0	0.0	0.0	0.0	0.0
0.0	0.0	0.0	0.0	0.0	0.0	0.0	0.0
0.0	0.0	0.0	0.0	0.0	0.0	0.0	0.0
0.0	0.0	0.0	0.0	0.0	0.0	0.0	0.0
0.0	0.0	0.0	0.0	0.0	0.0	0.0	0.0
0.0	10.0	4.0	0.0	0.0	0.0	0.0	9.0
18.0	0.0	0.0	0.0	0.0	0.0	0.0	0.0

0.0	0.0	0.0	0.0	0.0	0.0	0.0	0.0
0.0	0.0	0.0	9.0	0.0	0.0	0.0	28.0
0.0	0.0	0.0	0.0	0.0	15.0	0.0	0.0
0.0	0.0	0.0	0.0	0.0	0.0	0.0	0.0
29.0	4.0	0.0	24.0	0.0	0.0	0.0	10.0
0.0	0.0	0.0	0.0	0.0	0.0	0.0	0.0
0.0	0.0	0.0	0.0	0.0	0.0	0.0	0.0
0.0	0.0	0.0	0.0	0.0	0.0	0.0	0.0
0.0	0.0	0.0	0.0	0.0	0.0	0.0	0.0
0.0	0.0	0.0	0.0	0.0	0.0	0.0	0.0
0.0	0.0	0.0	0.0	0.0	0.0	0.0	0.0
0.0	1.0	0.0	0.0	0.0	0.0	0.0	0.0
0.0	0.0	0.0	0.0	0.0	0.0	0.0	0.0
0.0	0.0	0.0	0.0	0.0	0.0	0.0	0.0
0.0	1.0	0.0	0.0	0.0	3.0	0.0	0.0
0.0	0.0	0.0	0.0	0.0	0.0	0.0	0.0
0.0	0.0	0.0	0.0	0.0	0.0	0.0	0.0
0.0	0.0	0.0	0.0	0.0	0.0	0.0	0.0
0.0	0.0	0.0	1.0	0.0	0.0	0.0	0.0
0.0	0.0	0.0	0.0	0.0	0.0	0.0	0.0
0.0	0.0	0.0	0.0	0.0	0.0	0.0	0.0
0.0	0.0	0.0	0.0	0.0	0.0	0.0	0.0
0.0	0.0	64.0	4.0	0.0	0.0	0.0	36.0
0.0	0.0	0.0	3.0	0.0	0.0	0.0	0.0
0.0	0.0	0.0	475.0	0.0	56.0	0.0	0.0
0.0	0.0	0.0	0.0	0.0	0.0	0.0	0.0
0.0	0.0	0.0	0.0	0.0	7.0	0.0	0.0
0.0	0.0	0.0	0.0	0.0	0.0	0.0	46.0
0.0	9.0	0.0	0.0	0.0	0.0	14.0	2.0
0.0	0.0	0.0	0.0	0.0	0.0	60.0	0.0
0.0	2.0	0.0	0.0	0.0	0.0	0.0	0.0
0.0	0.0	0.0	0.0	0.0	1.0	0.0	0.0
0.0	0.0	0.0	0.0	0.0	0.0	0.0	0.0
0.0	0.0	0.0	0.0	0.0	0.0	0.0	0.0
0.0	0.0	0.0	0.0	0.0	0.0	0.0	0.0
0.0	0.0	0.0	0.0	0.0	0.0	0.0	0.0
0.0	0.0	0.0	0.0	0.0	0.0	0.0	0.0
0.0	0.0	0.0	0.0	0.0	0.0	0.0	3.0
0.0	0.0	0.0	0.0	0.0	0.0	0.0	0.0
0.0	0.0	11.0	0.0	0.0	0.0	0.0	0.0
0.0	0.0	0.0	0.0	0.0	0.0	0.0	0.0
0.0	0.0	0.0	0.0	0.0	0.0	0.0	0.0
0.0	4.0	0.0	1.0	0.0	0.0	7.0	0.0
0.0	0.0	0.0	0.0	0.0	0.0	0.0	0.0
0.0	0.0	0.0	0.0	0.0	0.0	0.0	0.0
0.0	3.0	0.0	0.0	0.0	13.0	10.0	0.0
2.0	0.0	2.0	0.0	0.0	0.0	0.0	0.0

0.0	0.0	0.0	0.0	0.0	0.0	0.0	0.0
0.0	0.0	0.0	0.0	0.0	0.0	0.0	0.0
0.0	0.0	0.0	0.0	0.0	0.0	0.0	0.0
0.0	0.0	0.0	0.0	0.0	0.0	0.0	0.0
0.0	0.0	0.0	0.0	0.0	0.0	2.0	0.0
0.0	2.0	0.0	0.0	0.0	0.0	0.0	0.0
0.0	0.0	0.0	0.0	0.0	0.0	0.0	0.0
0.0	0.0	0.0	0.0	0.0	0.0	0.0	0.0
0.0	0.0	0.0	0.0	0.0	0.0	10.0	0.0
0.0	2.0	0.0	0.0	0.0	0.0	0.0	0.0
0.0	0.0	0.0	0.0	0.0	0.0	0.0	0.0
0.0	0.0	0.0	0.0	0.0	0.0	0.0	0.0
0.0	2.0	0.0	0.0	0.0	0.0	0.0	0.0
0.0	0.0	0.0	0.0	0.0	21.0	0.0	10.0
0.0	0.0	0.0	0.0	0.0	0.0	0.0	0.0
0.0	0.0	0.0	0.0	0.0	0.0	0.0	0.0
0.0	0.0	0.0	0.0	0.0	0.0	0.0	0.0
0.0	0.0	0.0	0.0	0.0	0.0	0.0	0.0
0.0	0.0	0.0	0.0	0.0	0.0	0.0	4.0
0.0	0.0	0.0	0.0	0.0	0.0	0.0	0.0
0.0	0.0	0.0	0.0	0.0	0.0	0.0	0.0
0.0	0.0	0.0	0.0	0.0	0.0	0.0	0.0
0.0	0.0	0.0	0.0	0.0	0.0	0.0	0.0
0.0	0.0	0.0	0.0	0.0	0.0	0.0	0.0
0.0	0.0	0.0	0.0	0.0	0.0	0.0	0.0
0.0	0.0	0.0	0.0	0.0	1.0	0.0	0.0
0.0	0.0	0.0	0.0	0.0	0.0	0.0	0.0
0.0	0.0	0.0	0.0	0.0	0.0	0.0	0.0
0.0	0.0	0.0	0.0	0.0	0.0	0.0	0.0
0.0	0.0	0.0	0.0	0.0	0.0	0.0	0.0
0.0	0.0	0.0	0.0	0.0	0.0	0.0	0.0
0.0	0.0	0.0	0.0	0.0	0.0	0.0	0.0
0.0	0.0	0.0	0.0	0.0	0.0	0.0	0.0
0.0	0.0	0.0	0.0	0.0	0.0	0.0	0.0
0.0	0.0	0.0	0.0	0.0	38.0	14.0	0.0
0.0	0.0	0.0	0.0	0.0	0.0	0.0	0.0
0.0	0.0	0.0	0.0	0.0	6.0	0.0	0.0
0.0	0.0	0.0	0.0	0.0	0.0	0.0	4.0
0.0	0.0	0.0	0.0	0.0	0.0	0.0	0.0
0.0	0.0	0.0	0.0	0.0	0.0	0.0	0.0
0.0	0.0	0.0	0.0	0.0	0.0	0.0	0.0
2.0	0.0	0.0	0.0	0.0	0.0	0.0	0.0
0.0	0.0	0.0	0.0	0.0	0.0	0.0	0.0
0.0	0.0	0.0	0.0	0.0	0.0	0.0	0.0
0.0	0.0	0.0	0.0	0.0	0.0	0.0	0.0
0.0	0.0	0.0	0.0	0.0	0.0	0.0	0.0
0.0	0.0	0.0	0.0	0.0	0.0	0.0	0.0

0.0	0.0	0.0	0.0	0.0	0.0	0.0	0.0
0.0	0.0	0.0	0.0	0.0	0.0	0.0	0.0
0.0	0.0	0.0	0.0	0.0	0.0	0.0	0.0
0.0	0.0	0.0	0.0	0.0	0.0	0.0	0.0
0.0	0.0	0.0	0.0	0.0	0.0	0.0	0.0
0.0	0.0	0.0	0.0	0.0	0.0	0.0	0.0
0.0	0.0	0.0	0.0	0.0	0.0	0.0	0.0
0.0	0.0	0.0	0.0	0.0	0.0	0.0	0.0
0.0	0.0	0.0	0.0	0.0	0.0	0.0	0.0
0.0	0.0	0.0	0.0	0.0	0.0	0.0	0.0
0.0	0.0	0.0	0.0	0.0	0.0	0.0	0.0
0.0	0.0	0.0	0.0	0.0	0.0	0.0	0.0
0.0	0.0	0.0	0.0	0.0	0.0	0.0	0.0
0.0	0.0	0.0	0.0	0.0	0.0	0.0	0.0
0.0	1.0	0.0	0.0	0.0	0.0	0.0	0.0
0.0	0.0	0.0	0.0	0.0	0.0	0.0	0.0
0.0	0.0	0.0	0.0	0.0	0.0	0.0	0.0
0.0	0.0	0.0	0.0	0.0	0.0	0.0	0.0
0.0	0.0	0.0	0.0	0.0	0.0	0.0	0.0
0.0	0.0	0.0	0.0	0.0	0.0	0.0	0.0
0.0	0.0	0.0	0.0	0.0	0.0	0.0	0.0
0.0	0.0	0.0	0.0	0.0	0.0	0.0	0.0
0.0	0.0	0.0	0.0	0.0	0.0	3.0	0.0
0.0	0.0	0.0	0.0	0.0	0.0	0.0	0.0
0.0	18.0	0.0	0.0	3.0	0.0	184.0	0.0
0.0	0.0	0.0	0.0	0.0	0.0	0.0	0.0
0.0	0.0	0.0	0.0	0.0	0.0	0.0	0.0
0.0	0.0	0.0	0.0	0.0	0.0	0.0	0.0
0.0	0.0	0.0	0.0	0.0	0.0	0.0	0.0
0.0	0.0	0.0	0.0	0.0	0.0	0.0	0.0
0.0	0.0	0.0	0.0	0.0	0.0	0.0	0.0
0.0	0.0	0.0	0.0	0.0	0.0	0.0	0.0
0.0	0.0	0.0	0.0	0.0	0.0	0.0	0.0
17.0	0.0	0.0	0.0	0.0	0.0	0.0	0.0
0.0	0.0	0.0	1.0	0.0	0.0	0.0	0.0
0.0	3.0	0.0	0.0	0.0	0.0	0.0	0.0
0.0	0.0	0.0	0.0	0.0	0.0	0.0	0.0
0.0	0.0	0.0	0.0	0.0	0.0	0.0	0.0
0.0	0.0	0.0	0.0	0.0	0.0	0.0	0.0
0.0	0.0	0.0	0.0	0.0	0.0	0.0	0.0
0.0	0.0	12.0	0.0	0.0	0.0	0.0	0.0
0.0	0.0	0.0	0.0	0.0	0.0	0.0	0.0
0.0	0.0	0.0	0.0	0.0	0.0	0.0	0.0
0.0	0.0	0.0	0.0	0.0	0.0	0.0	0.0
0.0	0.0	0.0	0.0	0.0	0.0	0.0	0.0
0.0	0.0	0.0	0.0	0.0	0.0	0.0	0.0
0.0	0.0	0.0	0.0	0.0	0.0	0.0	0.0

0.0	0.0	0.0	0.0	0.0	0.0	0.0	0.0
0.0	0.0	0.0	0.0	0.0	0.0	0.0	0.0
0.0	0.0	0.0	0.0	0.0	0.0	0.0	0.0
0.0	0.0	0.0	0.0	0.0	0.0	18.0	0.0
0.0	0.0	0.0	0.0	0.0	0.0	0.0	0.0
0.0	0.0	0.0	0.0	0.0	0.0	0.0	0.0
0.0	0.0	0.0	0.0	0.0	0.0	0.0	0.0
0.0	0.0	0.0	0.0	0.0	0.0	0.0	0.0
0.0	0.0	0.0	0.0	0.0	0.0	0.0	0.0
0.0	0.0	0.0	0.0	0.0	0.0	0.0	0.0
0.0	0.0	0.0	0.0	0.0	0.0	0.0	0.0
0.0	0.0	0.0	0.0	0.0	0.0	0.0	0.0
0.0	0.0	0.0	0.0	0.0	0.0	0.0	0.0
0.0	0.0	0.0	0.0	0.0	1.0	0.0	0.0
0.0	0.0	0.0	0.0	0.0	0.0	0.0	12.0
0.0	0.0	0.0	0.0	0.0	0.0	0.0	0.0
0.0	0.0	0.0	0.0	0.0	0.0	0.0	0.0
0.0	0.0	0.0	0.0	0.0	0.0	0.0	0.0
0.0	0.0	0.0	0.0	0.0	0.0	0.0	0.0
0.0	0.0	0.0	0.0	0.0	0.0	0.0	0.0
0.0	0.0	0.0	0.0	0.0	0.0	0.0	0.0
0.0	0.0	0.0	0.0	0.0	0.0	0.0	0.0
0.0	0.0	0.0	0.0	0.0	0.0	0.0	0.0
0.0	0.0	0.0	0.0	0.0	0.0	0.0	0.0
0.0	0.0	0.0	0.0	0.0	0.0	0.0	0.0
0.0	1.0	0.0	0.0	0.0	0.0	1.0	1.0
0.0	0.0	0.0	0.0	0.0	0.0	0.0	0.0
0.0	0.0	0.0	0.0	0.0	0.0	0.0	0.0
0.0	0.0	0.0	0.0	0.0	0.0	0.0	0.0
0.0	0.0	0.0	0.0	0.0	0.0	0.0	0.0
0.0	0.0	0.0	0.0	0.0	0.0	0.0	0.0
0.0	0.0	0.0	0.0	0.0	0.0	0.0	0.0
0.0	0.0	0.0	0.0	0.0	0.0	0.0	0.0
0.0	0.0	0.0	0.0	0.0	0.0	0.0	0.0
0.0	0.0	0.0	0.0	0.0	0.0	0.0	0.0
0.0	0.0	0.0	0.0	0.0	0.0	0.0	0.0
0.0	0.0	0.0	0.0	0.0	0.0	0.0	0.0
0.0	0.0	0.0	0.0	0.0	0.0	0.0	0.0
0.0	0.0	0.0	0.0	0.0	0.0	0.0	0.0
0.0	0.0	0.0	0.0	0.0	0.0	0.0	0.0
0.0	0.0	0.0	0.0	0.0	0.0	0.0	0.0
0.0	0.0	0.0	0.0	0.0	0.0	0.0	0.0
0.0	0.0	0.0	0.0	0.0	0.0	0.0	0.0
0.0	0.0	0.0	0.0	0.0	0.0	0.0	0.0
0.0	0.0	0.0	0.0	0.0	0.0	3.0	0.0
0.0	0.0	0.0	0.0	0.0	0.0	0.0	0.0
0.0	0.0	0.0	0.0	0.0	0.0	0.0	0.0

0.0	0.0	0.0	0.0	0.0	0.0	0.0	0.0
0.0	0.0	0.0	0.0	0.0	0.0	0.0	0.0
0.0	0.0	0.0	0.0	0.0	0.0	0.0	0.0
0.0	0.0	0.0	0.0	0.0	0.0	0.0	0.0
0.0	0.0	0.0	0.0	0.0	0.0	0.0	0.0
0.0	0.0	0.0	0.0	0.0	0.0	0.0	0.0
0.0	0.0	0.0	0.0	0.0	0.0	0.0	0.0
0.0	0.0	0.0	0.0	0.0	0.0	0.0	0.0
0.0	0.0	0.0	0.0	0.0	0.0	0.0	0.0
0.0	0.0	0.0	0.0	0.0	0.0	0.0	0.0
0.0	0.0	0.0	0.0	0.0	0.0	0.0	0.0
0.0	0.0	0.0	0.0	0.0	0.0	0.0	0.0
0.0	0.0	0.0	0.0	0.0	0.0	0.0	0.0
0.0	0.0	0.0	0.0	0.0	0.0	0.0	0.0
0.0	0.0	0.0	0.0	0.0	0.0	0.0	0.0
30.0	0.0	0.0	3.0	0.0	0.0	0.0	0.0
0.0	0.0	0.0	0.0	0.0	0.0	0.0	0.0
0.0	0.0	0.0	0.0	0.0	0.0	0.0	0.0
0.0	0.0	0.0	0.0	0.0	0.0	0.0	0.0
0.0	0.0	0.0	0.0	0.0	0.0	0.0	0.0
0.0	0.0	0.0	0.0	0.0	0.0	12.0	0.0
0.0	0.0	0.0	0.0	0.0	0.0	0.0	0.0
0.0	0.0	0.0	0.0	0.0	0.0	0.0	0.0
0.0	0.0	0.0	0.0	0.0	0.0	0.0	0.0
0.0	0.0	0.0	0.0	0.0	0.0	0.0	0.0
0.0	0.0	0.0	0.0	0.0	0.0	0.0	0.0
0.0	0.0	0.0	0.0	0.0	0.0	0.0	0.0
0.0	0.0	0.0	0.0	0.0	0.0	0.0	0.0
0.0	0.0	0.0	0.0	0.0	0.0	0.0	0.0
0.0	0.0	0.0	0.0	0.0	0.0	0.0	0.0
0.0	0.0	0.0	0.0	0.0	0.0	0.0	0.0
0.0	64.0	0.0	0.0	0.0	134.0	0.0	0.0
0.0	0.0	0.0	0.0	0.0	0.0	0.0	0.0
0.0	0.0	0.0	0.0	0.0	0.0	0.0	0.0
0.0	0.0	0.0	0.0	0.0	0.0	0.0	0.0
0.0	0.0	0.0	0.0	0.0	35.0	0.0	0.0
0.0	0.0	0.0	0.0	0.0	0.0	0.0	0.0
0.0	13.0	0.0	0.0	0.0	0.0	0.0	0.0
0.0	0.0	0.0	0.0	0.0	0.0	0.0	0.0
0.0	0.0	0.0	0.0	2.0	0.0	28.0	0.0
0.0	0.0	0.0	0.0	0.0	0.0	0.0	0.0
0.0	93.0	0.0	0.0	0.0	0.0	0.0	0.0
0.0	0.0	0.0	0.0	0.0	0.0	0.0	0.0
0.0	0.0	0.0	0.0	0.0	0.0	0.0	0.0
0.0	0.0	0.0	0.0	0.0	0.0	0.0	0.0
0.0	0.0	0.0	0.0	0.0	0.0	0.0	0.0
0.0	0.0	0.0	0.0	0.0	0.0	12.0	0.0

0.0	0.0	0.0	0.0	0.0	0.0	0.0	0.0
0.0	0.0	0.0	0.0	0.0	0.0	0.0	0.0
0.0	6.0	0.0	0.0	0.0	0.0	0.0	0.0
0.0	0.0	0.0	0.0	0.0	0.0	0.0	0.0
0.0	0.0	0.0	0.0	0.0	0.0	0.0	0.0
0.0	0.0	0.0	0.0	0.0	0.0	0.0	0.0
0.0	0.0	0.0	0.0	0.0	0.0	0.0	0.0
0.0	0.0	0.0	0.0	0.0	0.0	0.0	0.0
0.0	0.0	0.0	0.0	0.0	0.0	0.0	0.0
0.0	0.0	0.0	0.0	0.0	0.0	0.0	0.0
0.0	0.0	0.0	0.0	0.0	0.0	0.0	0.0
0.0	0.0	0.0	0.0	0.0	0.0	0.0	0.0
0.0	0.0	0.0	0.0	0.0	0.0	0.0	0.0
0.0	0.0	0.0	0.0	0.0	0.0	0.0	0.0
0.0	0.0	0.0	0.0	0.0	0.0	0.0	0.0
0.0	0.0	0.0	0.0	0.0	0.0	0.0	0.0
0.0	0.0	0.0	0.0	0.0	0.0	0.0	0.0
0.0	0.0	0.0	0.0	0.0	0.0	0.0	0.0
0.0	0.0	0.0	0.0	0.0	0.0	0.0	0.0
0.0	0.0	0.0	0.0	0.0	0.0	0.0	0.0
0.0	0.0	0.0	0.0	0.0	0.0	0.0	0.0
0.0	0.0	0.0	0.0	0.0	0.0	0.0	0.0
0.0	0.0	0.0	0.0	0.0	0.0	0.0	0.0
0.0	0.0	0.0	0.0	0.0	0.0	0.0	0.0
0.0	0.0	0.0	0.0	0.0	0.0	0.0	0.0
0.0	0.0	0.0	0.0	0.0	0.0	0.0	0.0
0.0	0.0	0.0	0.0	0.0	0.0	0.0	0.0
0.0	0.0	0.0	0.0	0.0	0.0	0.0	0.0
0.0	0.0	0.0	0.0	0.0	0.0	0.0	0.0
0.0	0.0	0.0	0.0	0.0	0.0	0.0	0.0
0.0	0.0	2.0	0.0	0.0	0.0	0.0	0.0
0.0	0.0	0.0	0.0	0.0	0.0	0.0	0.0
0.0	0.0	0.0	0.0	0.0	0.0	0.0	0.0
0.0	0.0	0.0	0.0	0.0	0.0	0.0	0.0
0.0	0.0	0.0	0.0	0.0	0.0	0.0	0.0
0.0	0.0	0.0	0.0	0.0	0.0	0.0	0.0
0.0	0.0	0.0	0.0	0.0	0.0	0.0	0.0
0.0	0.0	0.0	0.0	0.0	0.0	0.0	0.0
0.0	0.0	0.0	0.0	0.0	0.0	0.0	0.0
0.0	0.0	0.0	0.0	0.0	0.0	0.0	4.0
0.0	0.0	0.0	0.0	0.0	0.0	0.0	0.0
0.0	0.0	0.0	0.0	0.0	0.0	0.0	0.0
0.0	3.0	0.0	0.0	0.0	0.0	0.0	0.0
0.0	0.0	0.0	0.0	0.0	0.0	0.0	1.0
0.0	0.0	0.0	0.0	0.0	0.0	0.0	0.0
0.0	0.0	0.0	0.0	0.0	0.0	0.0	0.0
0.0	0.0	0.0	0.0	0.0	0.0	0.0	0.0

0.0	0.0	0.0	0.0	0.0	0.0	0.0	0.0
0.0	0.0	0.0	0.0	0.0	0.0	0.0	0.0
0.0	0.0	0.0	0.0	0.0	0.0	0.0	0.0
0.0	0.0	0.0	0.0	0.0	0.0	0.0	0.0
0.0	0.0	0.0	0.0	0.0	0.0	0.0	0.0
0.0	0.0	0.0	0.0	0.0	0.0	0.0	0.0
0.0	0.0	0.0	0.0	0.0	0.0	0.0	0.0
0.0	0.0	0.0	0.0	0.0	0.0	0.0	0.0
0.0	0.0	0.0	0.0	0.0	0.0	0.0	0.0
0.0	0.0	0.0	0.0	0.0	0.0	0.0	0.0
0.0	0.0	0.0	0.0	0.0	0.0	0.0	0.0
0.0	0.0	0.0	0.0	0.0	0.0	0.0	0.0
0.0	0.0	0.0	0.0	0.0	0.0	0.0	0.0
0.0	0.0	0.0	0.0	0.0	0.0	0.0	0.0
0.0	0.0	0.0	0.0	0.0	0.0	0.0	0.0
0.0	0.0	0.0	0.0	0.0	0.0	0.0	0.0
0.0	0.0	0.0	0.0	0.0	0.0	0.0	0.0
0.0	0.0	0.0	0.0	0.0	0.0	0.0	0.0
0.0	0.0	0.0	0.0	0.0	0.0	0.0	0.0
0.0	0.0	0.0	0.0	0.0	0.0	0.0	0.0
0.0	0.0	0.0	0.0	0.0	0.0	0.0	0.0
0.0	0.0	0.0	0.0	0.0	0.0	0.0	0.0
0.0	0.0	0.0	0.0	0.0	0.0	0.0	0.0
0.0	0.0	0.0	0.0	0.0	0.0	0.0	0.0
0.0	0.0	0.0	0.0	0.0	0.0	0.0	0.0
0.0	0.0	0.0	0.0	0.0	0.0	0.0	0.0
0.0	0.0	0.0	0.0	0.0	0.0	0.0	0.0
0.0	0.0	0.0	0.0	0.0	0.0	0.0	0.0
0.0	0.0	0.0	0.0	0.0	0.0	0.0	0.0
0.0	0.0	0.0	0.0	0.0	0.0	0.0	0.0
0.0	0.0	0.0	0.0	0.0	0.0	0.0	0.0
0.0	0.0	0.0	0.0	0.0	0.0	0.0	0.0
0.0	0.0	0.0	0.0	0.0	0.0	0.0	0.0
0.0	0.0	0.0	0.0	0.0	0.0	0.0	0.0
0.0	0.0	0.0	0.0	0.0	0.0	0.0	2.0
0.0	0.0	0.0	0.0	0.0	0.0	0.0	0.0
0.0	0.0	0.0	0.0	0.0	0.0	0.0	0.0
0.0	0.0	0.0	0.0	0.0	0.0	0.0	0.0
0.0	0.0	0.0	0.0	0.0	0.0	0.0	0.0
0.0	0.0	0.0	0.0	0.0	0.0	0.0	5.0
0.0	0.0	0.0	0.0	0.0	0.0	0.0	0.0
0.0	0.0	0.0	0.0	0.0	0.0	0.0	0.0
0.0	0.0	0.0	0.0	0.0	0.0	0.0	0.0
0.0	0.0	0.0	0.0	0.0	0.0	0.0	0.0
0.0	0.0	0.0	0.0	0.0	0.0	0.0	0.0
0.0	0.0	0.0	0.0	0.0	0.0	0.0	0.0
0.0	0.0	0.0	0.0	0.0	0.0	0.0	0.0

0.0	0.0	0.0	0.0	0.0	0.0	0.0	0.0
0.0	0.0	0.0	865.0	0.0	0.0	0.0	0.0
0.0	0.0	0.0	0.0	0.0	0.0	0.0	0.0
0.0	0.0	0.0	0.0	0.0	0.0	0.0	0.0
0.0	0.0	0.0	0.0	0.0	0.0	0.0	0.0
0.0	0.0	0.0	0.0	0.0	0.0	0.0	0.0
0.0	0.0	0.0	0.0	0.0	0.0	19.0	0.0
0.0	0.0	0.0	0.0	0.0	0.0	0.0	0.0
0.0	0.0	0.0	0.0	0.0	0.0	0.0	0.0
0.0	0.0	0.0	0.0	0.0	0.0	0.0	0.0
0.0	0.0	0.0	0.0	0.0	0.0	0.0	0.0
0.0	0.0	0.0	0.0	0.0	0.0	54.0	0.0
0.0	245.0	7.0	0.0	0.0	0.0	0.0	0.0
0.0	0.0	126.0	0.0	0.0	15.0	0.0	0.0
0.0	0.0	0.0	0.0	0.0	0.0	0.0	0.0
0.0	0.0	0.0	0.0	0.0	0.0	0.0	0.0
0.0	0.0	0.0	0.0	0.0	0.0	0.0	0.0
0.0	0.0	0.0	0.0	0.0	0.0	0.0	0.0
0.0	0.0	0.0	0.0	0.0	0.0	0.0	0.0
0.0	0.0	0.0	0.0	0.0	0.0	0.0	0.0
0.0	0.0	0.0	0.0	0.0	0.0	0.0	0.0
0.0	0.0	0.0	0.0	0.0	0.0	0.0	0.0
0.0	0.0	0.0	0.0	0.0	0.0	0.0	0.0
0.0	0.0	0.0	0.0	0.0	0.0	0.0	0.0
0.0	0.0	0.0	0.0	0.0	0.0	0.0	0.0
0.0	0.0	0.0	0.0	0.0	0.0	0.0	0.0
0.0	0.0	0.0	0.0	0.0	0.0	0.0	0.0
0.0	0.0	0.0	0.0	0.0	0.0	0.0	0.0
0.0	0.0	0.0	0.0	0.0	0.0	0.0	0.0
0.0	0.0	0.0	1.0	0.0	0.0	0.0	1.0
0.0	0.0	0.0	0.0	0.0	0.0	2.0	0.0
0.0	0.0	0.0	0.0	0.0	0.0	0.0	0.0
0.0	0.0	0.0	0.0	0.0	0.0	0.0	0.0
0.0	0.0	0.0	0.0	0.0	0.0	0.0	0.0
0.0	0.0	0.0	6.0	0.0	0.0	0.0	1.0
0.0	0.0	0.0	0.0	0.0	0.0	0.0	0.0
0.0	0.0	0.0	0.0	0.0	0.0	0.0	0.0
0.0	0.0	0.0	0.0	0.0	0.0	0.0	0.0
0.0	0.0	0.0	0.0	0.0	0.0	1.0	0.0
0.0	0.0	0.0	0.0	0.0	0.0	0.0	0.0
0.0	0.0	0.0	0.0	0.0	0.0	0.0	0.0
0.0	0.0	0.0	0.0	0.0	0.0	0.0	0.0
0.0	0.0	0.0	0.0	0.0	0.0	0.0	0.0
0.0	0.0	0.0	0.0	0.0	0.0	0.0	0.0
5.0	0.0	0.0	0.0	0.0	0.0	0.0	0.0
3.0	0.0	0.0	0.0	1.0	0.0	0.0	0.0
0.0	0.0	0.0	0.0	0.0	0.0	0.0	0.0

0.0	0.0	0.0	0.0	0.0	0.0	0.0	0.0
0.0	0.0	0.0	0.0	0.0	0.0	0.0	0.0
0.0	0.0	0.0	0.0	0.0	0.0	0.0	0.0
0.0	0.0	0.0	0.0	0.0	0.0	0.0	0.0
0.0	0.0	0.0	0.0	0.0	0.0	0.0	0.0
0.0	0.0	0.0	0.0	0.0	0.0	0.0	0.0
0.0	0.0	0.0	0.0	0.0	0.0	0.0	0.0
0.0	0.0	0.0	0.0	0.0	0.0	0.0	0.0
0.0	0.0	0.0	0.0	0.0	0.0	0.0	0.0
0.0	0.0	0.0	0.0	431.0	0.0	4.0	0.0
0.0	0.0	0.0	0.0	0.0	0.0	0.0	0.0
0.0	2.0	0.0	0.0	0.0	0.0	0.0	0.0
0.0	0.0	0.0	0.0	0.0	0.0	0.0	0.0
0.0	0.0	0.0	0.0	0.0	0.0	0.0	0.0
0.0	0.0	0.0	0.0	0.0	0.0	0.0	0.0
0.0	0.0	0.0	0.0	0.0	0.0	0.0	0.0
0.0	0.0	0.0	0.0	0.0	0.0	0.0	0.0
0.0	0.0	0.0	0.0	0.0	1.0	0.0	0.0
21.0	0.0	0.0	0.0	25.0	14.0	0.0	0.0
0.0	6.0	0.0	0.0	0.0	0.0	0.0	0.0
0.0	0.0	0.0	0.0	0.0	0.0	3.0	0.0
0.0	0.0	0.0	0.0	0.0	0.0	0.0	0.0
0.0	0.0	0.0	0.0	0.0	0.0	0.0	0.0
0.0	0.0	0.0	0.0	0.0	0.0	590.0	0.0
0.0	0.0	0.0	0.0	0.0	0.0	15.0	0.0
0.0	0.0	0.0	0.0	0.0	0.0	0.0	0.0
0.0	0.0	0.0	0.0	0.0	15.0	0.0	0.0
0.0	0.0	0.0	0.0	0.0	0.0	0.0	0.0
0.0	0.0	0.0	0.0	0.0	0.0	0.0	0.0
0.0	0.0	0.0	1.0	0.0	0.0	0.0	0.0
0.0	0.0	0.0	0.0	0.0	0.0	0.0	0.0
0.0	0.0	0.0	0.0	0.0	0.0	0.0	0.0
0.0	0.0	0.0	0.0	0.0	0.0	0.0	0.0
0.0	0.0	0.0	0.0	0.0	0.0	0.0	0.0
0.0	0.0	0.0	0.0	0.0	0.0	0.0	0.0
0.0	0.0	0.0	0.0	0.0	0.0	0.0	0.0
0.0	0.0	0.0	0.0	0.0	0.0	0.0	0.0
0.0	0.0	0.0	0.0	0.0	0.0	0.0	0.0
0.0	0.0	0.0	0.0	0.0	0.0	0.0	1.0
0.0	0.0	0.0	0.0	0.0	0.0	0.0	0.0
0.0	0.0	0.0	0.0	0.0	0.0	0.0	0.0
0.0	0.0	0.0	0.0	0.0	0.0	0.0	0.0
0.0	0.0	0.0	0.0	0.0	0.0	0.0	0.0
0.0	0.0	0.0	0.0	0.0	0.0	0.0	0.0
0.0	0.0	0.0	0.0	0.0	0.0	0.0	0.0
0.0	0.0	0.0	0.0	0.0	0.0	0.0	0.0
0.0	0.0	0.0	0.0	0.0	0.0	0.0	0.0

0.0	0.0	0.0	0.0	0.0	0.0	0.0	0.0
0.0	1.0	0.0	0.0	0.0	0.0	0.0	0.0
0.0	0.0	0.0	0.0	0.0	0.0	0.0	0.0
0.0	0.0	0.0	0.0	0.0	1.0	4.0	0.0
0.0	0.0	0.0	0.0	0.0	0.0	0.0	0.0
0.0	0.0	0.0	0.0	0.0	0.0	0.0	0.0
0.0	0.0	0.0	0.0	0.0	0.0	0.0	0.0
0.0	0.0	0.0	0.0	0.0	0.0	0.0	0.0
0.0	0.0	0.0	0.0	0.0	0.0	0.0	0.0
0.0	0.0	0.0	0.0	0.0	0.0	0.0	0.0
0.0	0.0	0.0	0.0	0.0	0.0	0.0	0.0
0.0	0.0	0.0	0.0	0.0	0.0	0.0	0.0
0.0	0.0	0.0	0.0	0.0	0.0	0.0	0.0
0.0	0.0	0.0	0.0	0.0	0.0	0.0	0.0
0.0	0.0	0.0	0.0	1.0	0.0	0.0	0.0
0.0	0.0	0.0	0.0	0.0	0.0	0.0	0.0
0.0	0.0	0.0	0.0	0.0	0.0	0.0	0.0
0.0	0.0	0.0	0.0	0.0	0.0	0.0	0.0
1.0	1.0	0.0	0.0	0.0	0.0	0.0	0.0
0.0	0.0	0.0	0.0	0.0	0.0	0.0	0.0
0.0	0.0	0.0	0.0	0.0	0.0	0.0	0.0
0.0	0.0	0.0	0.0	0.0	0.0	0.0	0.0
0.0	0.0	0.0	0.0	0.0	0.0	0.0	0.0
12.0	0.0	0.0	155.0	0.0	2.0	0.0	0.0
0.0	0.0	0.0	0.0	0.0	0.0	0.0	0.0
0.0	0.0	0.0	0.0	0.0	0.0	0.0	0.0
0.0	0.0	0.0	0.0	0.0	0.0	0.0	0.0
0.0	0.0	0.0	0.0	0.0	0.0	0.0	0.0
0.0	0.0	0.0	0.0	0.0	0.0	0.0	0.0
0.0	0.0	0.0	0.0	0.0	0.0	0.0	0.0
0.0	0.0	0.0	0.0	0.0	0.0	0.0	0.0
0.0	0.0	0.0	0.0	0.0	0.0	0.0	0.0
0.0	0.0	0.0	0.0	0.0	0.0	0.0	0.0
0.0	0.0	0.0	0.0	0.0	0.0	0.0	0.0
0.0	0.0	0.0	0.0	0.0	0.0	0.0	0.0
0.0	0.0	2.0	10.0	0.0	2.0	0.0	0.0
0.0	0.0	0.0	0.0	0.0	0.0	0.0	0.0
0.0	0.0	0.0	0.0	2.0	0.0	0.0	0.0
0.0	0.0	0.0	0.0	0.0	0.0	0.0	0.0
0.0	0.0	0.0	0.0	0.0	0.0	0.0	0.0
0.0	0.0	0.0	0.0	1.0	0.0	0.0	0.0
0.0	0.0	0.0	0.0	0.0	0.0	0.0	0.0
0.0	0.0	0.0	0.0	0.0	0.0	0.0	0.0
0.0	0.0	0.0	0.0	0.0	0.0	0.0	0.0
0.0	0.0	0.0	0.0	0.0	0.0	0.0	0.0
0.0	0.0	0.0	0.0	0.0	0.0	0.0	0.0
0.0	0.0	0.0	0.0	0.0	0.0	0.0	0.0

0.0	0.0	0.0	0.0	0.0	0.0	0.0	0.0
0.0	0.0	0.0	0.0	0.0	0.0	0.0	0.0
0.0	0.0	0.0	0.0	0.0	0.0	0.0	0.0
0.0	0.0	0.0	0.0	0.0	0.0	0.0	0.0
0.0	0.0	0.0	0.0	0.0	0.0	0.0	0.0
0.0	0.0	0.0	0.0	0.0	0.0	0.0	3.0
0.0	0.0	0.0	0.0	0.0	0.0	0.0	0.0
0.0	0.0	84.0	0.0	0.0	0.0	0.0	0.0
0.0	0.0	0.0	0.0	0.0	0.0	0.0	0.0
0.0	0.0	0.0	0.0	0.0	0.0	0.0	0.0
0.0	0.0	0.0	0.0	0.0	0.0	0.0	0.0
0.0	0.0	0.0	0.0	0.0	0.0	0.0	0.0
0.0	0.0	0.0	0.0	0.0	0.0	0.0	0.0
0.0	0.0	0.0	0.0	0.0	0.0	0.0	0.0
0.0	0.0	0.0	0.0	0.0	0.0	0.0	0.0
0.0	0.0	0.0	0.0	0.0	0.0	0.0	0.0
0.0	0.0	0.0	0.0	0.0	0.0	0.0	0.0
0.0	0.0	0.0	0.0	0.0	0.0	0.0	0.0
0.0	0.0	0.0	0.0	0.0	0.0	0.0	0.0
0.0	0.0	0.0	0.0	0.0	0.0	0.0	0.0
0.0	0.0	0.0	0.0	0.0	0.0	0.0	0.0
0.0	0.0	0.0	0.0	0.0	0.0	0.0	0.0
0.0	0.0	0.0	0.0	0.0	0.0	0.0	0.0
0.0	0.0	0.0	0.0	0.0	0.0	0.0	0.0
0.0	0.0	0.0	0.0	0.0	0.0	0.0	0.0
0.0	0.0	0.0	0.0	0.0	0.0	0.0	0.0
0.0	0.0	0.0	0.0	0.0	0.0	0.0	0.0
0.0	0.0	0.0	0.0	0.0	0.0	0.0	0.0
0.0	0.0	0.0	0.0	0.0	0.0	0.0	0.0
0.0	0.0	23.0	0.0	0.0	0.0	0.0	0.0
0.0	0.0	0.0	0.0	0.0	0.0	0.0	0.0
0.0	0.0	0.0	0.0	0.0	0.0	0.0	0.0
0.0	0.0	0.0	0.0	0.0	0.0	0.0	0.0
0.0	0.0	0.0	0.0	0.0	0.0	0.0	0.0
0.0	0.0	0.0	0.0	0.0	0.0	0.0	0.0
0.0	0.0	0.0	0.0	0.0	0.0	0.0	0.0
0.0	0.0	0.0	0.0	0.0	0.0	0.0	0.0
0.0	2.0	0.0	0.0	0.0	0.0	0.0	0.0
0.0	0.0	0.0	0.0	0.0	0.0	0.0	0.0
0.0	0.0	0.0	0.0	0.0	0.0	0.0	0.0
0.0	0.0	0.0	0.0	0.0	0.0	0.0	0.0
0.0	0.0	0.0	0.0	0.0	0.0	0.0	0.0
0.0	0.0	0.0	0.0	0.0	0.0	0.0	0.0
0.0	0.0	0.0	0.0	0.0	0.0	0.0	0.0
0.0	0.0	0.0	0.0	0.0	0.0	0.0	1.0
0.0	0.0	0.0	0.0	0.0	0.0	0.0	0.0
0.0	0.0	0.0	0.0	0.0	0.0	0.0	0.0

0.0	0.0	0.0	0.0	0.0	0.0	0.0	0.0
0.0	0.0	0.0	0.0	0.0	0.0	0.0	0.0
0.0	0.0	0.0	0.0	0.0	0.0	0.0	0.0
0.0	0.0	0.0	0.0	0.0	0.0	0.0	0.0
0.0	0.0	0.0	0.0	0.0	0.0	0.0	0.0
0.0	0.0	0.0	0.0	0.0	0.0	0.0	0.0
0.0	0.0	0.0	0.0	0.0	0.0	0.0	0.0
0.0	0.0	0.0	0.0	0.0	0.0	0.0	0.0
0.0	0.0	0.0	0.0	0.0	0.0	0.0	0.0
0.0	0.0	0.0	0.0	0.0	0.0	0.0	0.0
0.0	0.0	0.0	0.0	0.0	0.0	0.0	0.0
0.0	0.0	0.0	0.0	0.0	0.0	0.0	0.0
0.0	0.0	0.0	0.0	0.0	0.0	0.0	0.0
0.0	0.0	0.0	0.0	0.0	0.0	0.0	0.0
0.0	0.0	0.0	0.0	0.0	0.0	0.0	0.0
0.0	0.0	0.0	0.0	0.0	0.0	0.0	0.0
0.0	0.0	0.0	0.0	0.0	0.0	0.0	0.0
0.0	0.0	0.0	0.0	0.0	0.0	0.0	0.0
0.0	0.0	0.0	0.0	0.0	0.0	0.0	0.0
0.0	0.0	0.0	0.0	0.0	0.0	0.0	0.0
0.0	0.0	0.0	0.0	0.0	0.0	0.0	0.0
0.0	0.0	0.0	0.0	0.0	0.0	0.0	0.0
0.0	0.0	0.0	0.0	0.0	0.0	0.0	0.0
0.0	0.0	0.0	0.0	0.0	0.0	0.0	0.0
0.0	0.0	0.0	0.0	0.0	0.0	0.0	0.0
0.0	0.0	0.0	0.0	0.0	0.0	0.0	0.0
0.0	0.0	0.0	0.0	0.0	0.0	0.0	0.0
0.0	0.0	0.0	0.0	0.0	0.0	0.0	0.0
0.0	0.0	0.0	0.0	0.0	0.0	0.0	0.0
0.0	0.0	0.0	0.0	0.0	0.0	0.0	0.0
0.0	0.0	0.0	0.0	0.0	0.0	2.0	0.0
0.0	0.0	0.0	0.0	0.0	0.0	0.0	0.0
10.0	0.0	0.0	0.0	0.0	0.0	0.0	0.0
0.0	0.0	0.0	0.0	0.0	0.0	0.0	0.0
0.0	0.0	0.0	0.0	0.0	0.0	0.0	0.0
0.0	0.0	0.0	0.0	0.0	0.0	0.0	0.0
0.0	0.0	0.0	0.0	0.0	0.0	0.0	0.0
0.0	0.0	0.0	0.0	0.0	0.0	0.0	0.0
0.0	0.0	0.0	0.0	0.0	0.0	0.0	0.0
0.0	0.0	0.0	0.0	0.0	0.0	0.0	0.0
0.0	0.0	0.0	0.0	0.0	0.0	0.0	0.0
0.0	0.0	0.0	0.0	0.0	0.0	0.0	0.0
0.0	0.0	0.0	0.0	0.0	0.0	0.0	3.0
0.0	0.0	0.0	0.0	0.0	0.0	0.0	0.0
0.0	0.0	0.0	0.0	0.0	0.0	0.0	0.0
0.0	0.0	0.0	0.0	0.0	0.0	0.0	0.0
0.0	0.0	0.0	0.0	0.0	0.0	0.0	0.0

0.0	0.0	0.0	0.0	0.0	0.0	0.0	0.0
0.0	0.0	0.0	0.0	0.0	0.0	0.0	0.0
0.0	0.0	0.0	0.0	0.0	0.0	0.0	0.0
0.0	0.0	0.0	0.0	0.0	0.0	0.0	0.0
0.0	0.0	0.0	0.0	0.0	0.0	0.0	0.0
0.0	0.0	0.0	0.0	0.0	0.0	0.0	0.0
0.0	0.0	0.0	0.0	0.0	0.0	0.0	0.0
0.0	0.0	0.0	0.0	0.0	0.0	0.0	0.0
0.0	0.0	0.0	0.0	0.0	0.0	0.0	0.0
0.0	0.0	0.0	0.0	0.0	0.0	0.0	0.0
0.0	0.0	0.0	0.0	0.0	0.0	0.0	0.0
0.0	0.0	0.0	0.0	0.0	0.0	0.0	0.0
0.0	0.0	0.0	0.0	0.0	0.0	0.0	0.0
0.0	0.0	0.0	0.0	0.0	0.0	0.0	0.0
0.0	0.0	0.0	0.0	0.0	0.0	0.0	0.0
0.0	0.0	0.0	0.0	0.0	0.0	0.0	0.0
0.0	0.0	0.0	0.0	0.0	0.0	0.0	0.0
0.0	0.0	0.0	0.0	0.0	0.0	0.0	0.0
0.0	0.0	0.0	0.0	0.0	0.0	0.0	0.0
0.0	0.0	0.0	0.0	0.0	0.0	0.0	0.0
0.0	0.0	0.0	0.0	0.0	0.0	0.0	0.0
0.0	0.0	0.0	0.0	0.0	0.0	0.0	0.0
0.0	0.0	0.0	0.0	0.0	0.0	0.0	0.0
0.0	0.0	0.0	0.0	0.0	0.0	0.0	0.0
0.0	0.0	0.0	0.0	0.0	0.0	0.0	0.0
0.0	0.0	0.0	0.0	0.0	0.0	0.0	0.0
0.0	0.0	0.0	0.0	0.0	0.0	0.0	0.0
0.0	0.0	0.0	0.0	0.0	0.0	0.0	0.0
0.0	0.0	0.0	0.0	0.0	0.0	0.0	0.0
0.0	0.0	0.0	0.0	0.0	0.0	0.0	0.0
0.0	0.0	0.0	0.0	0.0	0.0	0.0	0.0
0.0	0.0	0.0	0.0	0.0	0.0	0.0	0.0
0.0	0.0	0.0	0.0	0.0	0.0	0.0	0.0
0.0	0.0	0.0	0.0	0.0	0.0	0.0	0.0
0.0	0.0	0.0	0.0	0.0	0.0	0.0	0.0
0.0	0.0	0.0	0.0	0.0	0.0	0.0	0.0
0.0	0.0	0.0	0.0	0.0	0.0	0.0	0.0
0.0	0.0	0.0	0.0	0.0	0.0	0.0	0.0
0.0	0.0	0.0	0.0	0.0	0.0	0.0	0.0
0.0	0.0	0.0	8.0	0.0	0.0	0.0	0.0
0.0	0.0	0.0	0.0	0.0	0.0	0.0	4.0
0.0	0.0	0.0	0.0	0.0	0.0	0.0	0.0
0.0	0.0	0.0	0.0	0.0	0.0	0.0	0.0
0.0	0.0	0.0	0.0	0.0	0.0	0.0	0.0
0.0	0.0	0.0	0.0	0.0	0.0	0.0	0.0
0.0	0.0	0.0	0.0	0.0	0.0	0.0	0.0
0.0	0.0	0.0	0.0	0.0	0.0	0.0	0.0

0.0	0.0	0.0	0.0	0.0	0.0	0.0	0.0
0.0	0.0	0.0	0.0	0.0	0.0	0.0	0.0
0.0	0.0	0.0	0.0	0.0	0.0	0.0	0.0
0.0	0.0	0.0	0.0	0.0	0.0	0.0	0.0
0.0	0.0	0.0	0.0	0.0	0.0	0.0	0.0
0.0	0.0	0.0	0.0	0.0	0.0	0.0	0.0
0.0	0.0	0.0	0.0	0.0	0.0	0.0	0.0
0.0	0.0	0.0	0.0	0.0	0.0	0.0	0.0
0.0	0.0	0.0	0.0	0.0	0.0	0.0	0.0
0.0	0.0	0.0	0.0	0.0	0.0	0.0	0.0
0.0	0.0	0.0	0.0	0.0	0.0	0.0	0.0
0.0	0.0	0.0	0.0	0.0	0.0	0.0	0.0
0.0	0.0	0.0	0.0	0.0	0.0	0.0	0.0
0.0	0.0	0.0	0.0	0.0	0.0	0.0	0.0
0.0	0.0	0.0	0.0	0.0	0.0	0.0	0.0
0.0	0.0	0.0	0.0	0.0	0.0	0.0	0.0
0.0	0.0	0.0	0.0	0.0	0.0	0.0	0.0
0.0	0.0	0.0	0.0	0.0	0.0	0.0	0.0
0.0	0.0	0.0	0.0	0.0	0.0	0.0	0.0
0.0	0.0	0.0	0.0	0.0	0.0	0.0	0.0
0.0	0.0	0.0	0.0	0.0	0.0	0.0	0.0
0.0	0.0	0.0	0.0	0.0	0.0	0.0	0.0
0.0	0.0	0.0	0.0	0.0	0.0	0.0	0.0
0.0	0.0	0.0	0.0	0.0	0.0	0.0	0.0
0.0	0.0	0.0	0.0	0.0	0.0	0.0	0.0
0.0	0.0	0.0	0.0	0.0	0.0	0.0	0.0
0.0	0.0	0.0	0.0	0.0	0.0	0.0	0.0
0.0	0.0	0.0	0.0	0.0	0.0	0.0	0.0
0.0	0.0	0.0	0.0	0.0	0.0	0.0	0.0
0.0	0.0	0.0	0.0	0.0	0.0	0.0	0.0
0.0	0.0	0.0	0.0	0.0	0.0	0.0	0.0
0.0	0.0	0.0	0.0	0.0	0.0	0.0	0.0
0.0	0.0	0.0	0.0	0.0	0.0	0.0	0.0
0.0	0.0	0.0	0.0	0.0	0.0	0.0	0.0
0.0	0.0	0.0	0.0	0.0	0.0	0.0	0.0
0.0	0.0	0.0	0.0	0.0	0.0	0.0	0.0
0.0	0.0	0.0	0.0	0.0	0.0	0.0	0.0
0.0	0.0	0.0	0.0	0.0	0.0	0.0	0.0
0.0	0.0	0.0	0.0	0.0	0.0	0.0	0.0
0.0	0.0	0.0	0.0	0.0	0.0	0.0	0.0
0.0	0.0	0.0	0.0	0.0	0.0	0.0	0.0
0.0	0.0	0.0	0.0	0.0	0.0	0.0	0.0
0.0	0.0	0.0	0.0	0.0	0.0	0.0	0.0
0.0	0.0	0.0	0.0	0.0	0.0	0.0	0.0
0.0	0.0	0.0	0.0	0.0	0.0	0.0	0.0
0.0	0.0	0.0	0.0	0.0	0.0	0.0	0.0
0.0	0.0	0.0	0.0	0.0	0.0	0.0	0.0
0.0	0.0	0.0	0.0	0.0	0.0	0.0	0.0

0.0	0.0	0.0	0.0	0.0	0.0	0.0	0.0
0.0	0.0	0.0	0.0	0.0	0.0	0.0	0.0
0.0	0.0	0.0	0.0	0.0	0.0	0.0	0.0
0.0	0.0	0.0	0.0	0.0	0.0	0.0	0.0
0.0	0.0	0.0	0.0	0.0	0.0	0.0	0.0
0.0	0.0	0.0	0.0	0.0	0.0	0.0	0.0
0.0	0.0	0.0	0.0	0.0	0.0	0.0	0.0
0.0	0.0	0.0	0.0	0.0	0.0	0.0	0.0
0.0	0.0	0.0	0.0	0.0	0.0	0.0	0.0
0.0	0.0	0.0	0.0	0.0	0.0	0.0	0.0
0.0	0.0	0.0	0.0	0.0	0.0	0.0	0.0
0.0	0.0	0.0	0.0	0.0	0.0	0.0	0.0
0.0	0.0	0.0	0.0	0.0	0.0	0.0	0.0
0.0	0.0	0.0	0.0	0.0	0.0	0.0	0.0
0.0	0.0	0.0	0.0	0.0	0.0	0.0	0.0
0.0	0.0	0.0	0.0	0.0	0.0	0.0	0.0
0.0	0.0	0.0	0.0	0.0	0.0	0.0	0.0
0.0	0.0	0.0	0.0	0.0	0.0	0.0	0.0
515.0	19.0	0.0	0.0	0.0	0.0	22.0	0.0
0.0	0.0	0.0	0.0	0.0	0.0	0.0	0.0
0.0	0.0	0.0	0.0	0.0	0.0	0.0	0.0
0.0	0.0	0.0	0.0	0.0	0.0	0.0	0.0
85.0	0.0	2.0	4.0	0.0	0.0	0.0	0.0
0.0	0.0	0.0	0.0	0.0	0.0	0.0	0.0
0.0	0.0	0.0	0.0	0.0	0.0	0.0	0.0
0.0	0.0	33.0	0.0	0.0	0.0	0.0	0.0
0.0	0.0	0.0	0.0	0.0	0.0	0.0	0.0
0.0	0.0	0.0	0.0	0.0	0.0	0.0	0.0
0.0	0.0	0.0	0.0	0.0	0.0	0.0	0.0
0.0	0.0	0.0	0.0	0.0	0.0	0.0	0.0
0.0	0.0	0.0	0.0	0.0	0.0	0.0	0.0
0.0	0.0	0.0	0.0	0.0	0.0	0.0	0.0
0.0	0.0	0.0	0.0	0.0	0.0	0.0	3.0
0.0	0.0	0.0	0.0	0.0	0.0	0.0	0.0
0.0	0.0	0.0	0.0	0.0	0.0	0.0	0.0
0.0	0.0	0.0	0.0	0.0	0.0	0.0	0.0
0.0	0.0	0.0	0.0	0.0	0.0	0.0	0.0
0.0	0.0	0.0	0.0	0.0	0.0	0.0	0.0
0.0	0.0	0.0	0.0	0.0	0.0	0.0	0.0
0.0	0.0	0.0	0.0	0.0	0.0	0.0	0.0
0.0	0.0	0.0	0.0	0.0	0.0	0.0	0.0
0.0	0.0	0.0	0.0	0.0	0.0	0.0	0.0
0.0	0.0	0.0	0.0	0.0	0.0	0.0	0.0
0.0	0.0	0.0	0.0	0.0	0.0	0.0	0.0
0.0	0.0	0.0	0.0	0.0	0.0	0.0	0.0
0.0	0.0	0.0	0.0	0.0	0.0	0.0	0.0
0.0	0.0	0.0	0.0	0.0	0.0	0.0	0.0

0.0	0.0	0.0	0.0	0.0	0.0	0.0	0.0
0.0	0.0	0.0	0.0	0.0	0.0	0.0	0.0
0.0	0.0	0.0	0.0	0.0	0.0	0.0	0.0
0.0	0.0	0.0	0.0	0.0	0.0	0.0	0.0
0.0	0.0	0.0	0.0	0.0	0.0	0.0	0.0
0.0	0.0	0.0	0.0	0.0	0.0	0.0	0.0
0.0	0.0	0.0	0.0	0.0	0.0	0.0	0.0
0.0	0.0	0.0	0.0	0.0	0.0	0.0	0.0
0.0	0.0	0.0	0.0	0.0	0.0	0.0	0.0
0.0	0.0	0.0	0.0	0.0	0.0	0.0	0.0
0.0	0.0	0.0	0.0	0.0	0.0	0.0	0.0
0.0	0.0	0.0	0.0	0.0	0.0	0.0	0.0
0.0	0.0	0.0	0.0	0.0	0.0	0.0	0.0
0.0	0.0	0.0	0.0	0.0	0.0	0.0	0.0
0.0	0.0	0.0	0.0	0.0	0.0	0.0	0.0
0.0	0.0	0.0	0.0	0.0	0.0	0.0	0.0
0.0	0.0	0.0	0.0	0.0	0.0	0.0	0.0
0.0	0.0	0.0	0.0	0.0	0.0	0.0	0.0
0.0	0.0	0.0	0.0	0.0	0.0	0.0	0.0
0.0	0.0	0.0	0.0	0.0	0.0	0.0	0.0
0.0	0.0	0.0	0.0	0.0	0.0	0.0	0.0
0.0	0.0	0.0	0.0	0.0	0.0	0.0	0.0
0.0	0.0	0.0	0.0	0.0	0.0	0.0	0.0
0.0	0.0	0.0	0.0	0.0	0.0	0.0	0.0
0.0	0.0	0.0	0.0	0.0	1.0	0.0	0.0
0.0	0.0	0.0	0.0	0.0	0.0	0.0	0.0
0.0	0.0	0.0	0.0	0.0	0.0	0.0	0.0
0.0	0.0	0.0	0.0	0.0	0.0	0.0	0.0
0.0	0.0	0.0	0.0	0.0	0.0	0.0	0.0
0.0	0.0	0.0	0.0	0.0	0.0	0.0	0.0
0.0	0.0	0.0	0.0	0.0	0.0	0.0	1.0
0.0	0.0	0.0	0.0	0.0	0.0	0.0	0.0
0.0	0.0	0.0	0.0	0.0	0.0	0.0	0.0
0.0	0.0	0.0	0.0	0.0	0.0	0.0	0.0
0.0	0.0	0.0	0.0	0.0	0.0	0.0	0.0
0.0	0.0	0.0	0.0	0.0	0.0	0.0	0.0
0.0	0.0	0.0	0.0	0.0	0.0	0.0	0.0
0.0	0.0	0.0	0.0	0.0	0.0	0.0	0.0
0.0	0.0	0.0	0.0	0.0	0.0	0.0	0.0
0.0	0.0	0.0	0.0	0.0	0.0	0.0	0.0
0.0	0.0	0.0	0.0	0.0	0.0	0.0	0.0
0.0	0.0	0.0	0.0	0.0	0.0	0.0	0.0
0.0	0.0	0.0	0.0	0.0	0.0	0.0	0.0
0.0	0.0	0.0	0.0	0.0	0.0	0.0	0.0
0.0	0.0	0.0	0.0	0.0	0.0	0.0	0.0
0.0	0.0	0.0	0.0	0.0	0.0	0.0	0.0
0.0	0.0	0.0	9.0	0.0	0.0	0.0	0.0

0.0	0.0	0.0	0.0	0.0	0.0	0.0	0.0
0.0	0.0	0.0	0.0	0.0	0.0	0.0	0.0
0.0	0.0	0.0	0.0	0.0	0.0	0.0	2.0
0.0	0.0	0.0	0.0	0.0	0.0	0.0	0.0
0.0	0.0	0.0	0.0	0.0	0.0	0.0	0.0
0.0	0.0	0.0	0.0	0.0	0.0	0.0	0.0
0.0	0.0	0.0	0.0	0.0	0.0	0.0	0.0
0.0	0.0	0.0	0.0	0.0	0.0	0.0	6.0
0.0	0.0	0.0	0.0	0.0	0.0	0.0	0.0
0.0	0.0	0.0	0.0	0.0	0.0	0.0	0.0
0.0	0.0	0.0	0.0	0.0	0.0	0.0	0.0
0.0	0.0	0.0	0.0	0.0	0.0	0.0	0.0
0.0	0.0	0.0	0.0	0.0	0.0	0.0	0.0
0.0	0.0	0.0	0.0	0.0	0.0	0.0	0.0
0.0	0.0	0.0	0.0	0.0	0.0	0.0	0.0
0.0	0.0	0.0	0.0	0.0	0.0	0.0	0.0
0.0	0.0	0.0	0.0	0.0	0.0	0.0	0.0
0.0	0.0	0.0	0.0	0.0	0.0	0.0	0.0
0.0	0.0	0.0	0.0	0.0	0.0	0.0	0.0
0.0	0.0	0.0	0.0	0.0	0.0	0.0	0.0
0.0	0.0	0.0	0.0	0.0	0.0	0.0	0.0
0.0	0.0	0.0	0.0	0.0	0.0	0.0	0.0
0.0	0.0	0.0	0.0	0.0	0.0	0.0	0.0
0.0	0.0	0.0	0.0	0.0	0.0	0.0	0.0
0.0	0.0	0.0	0.0	0.0	0.0	4.0	0.0
0.0	0.0	0.0	0.0	0.0	0.0	0.0	7.0
0.0	0.0	0.0	0.0	0.0	0.0	0.0	0.0
0.0	0.0	0.0	0.0	0.0	0.0	0.0	0.0
0.0	0.0	0.0	0.0	0.0	0.0	0.0	0.0
0.0	0.0	0.0	0.0	0.0	0.0	0.0	0.0
0.0	0.0	0.0	0.0	0.0	0.0	0.0	0.0
0.0	0.0	0.0	0.0	0.0	0.0	0.0	0.0
0.0	0.0	0.0	0.0	0.0	0.0	0.0	0.0
0.0	0.0	0.0	0.0	0.0	0.0	0.0	0.0
0.0	0.0	0.0	0.0	0.0	0.0	0.0	0.0
0.0	0.0	0.0	0.0	0.0	0.0	0.0	0.0
0.0	0.0	0.0	0.0	0.0	0.0	0.0	0.0
0.0	0.0	0.0	0.0	0.0	0.0	0.0	0.0
0.0	0.0	0.0	0.0	0.0	0.0	0.0	0.0
0.0	0.0	0.0	0.0	0.0	0.0	0.0	0.0
0.0	0.0	0.0	0.0	0.0	0.0	0.0	0.0
0.0	0.0	0.0	0.0	0.0	0.0	0.0	0.0
0.0	0.0	0.0	0.0	0.0	0.0	0.0	0.0
0.0	0.0	0.0	0.0	0.0	0.0	0.0	0.0
0.0	0.0	0.0	0.0	0.0	0.0	0.0	0.0
0.0	0.0	0.0	0.0	0.0	0.0	0.0	0.0
0.0	0.0	0.0	0.0	0.0	0.0	0.0	0.0

0.0	0.0	0.0	0.0	0.0	0.0	0.0	0.0
0.0	0.0	0.0	0.0	0.0	0.0	0.0	0.0
1.0	0.0	0.0	0.0	0.0	0.0	0.0	0.0
0.0	0.0	0.0	0.0	0.0	0.0	0.0	0.0
0.0	0.0	0.0	0.0	0.0	0.0	0.0	0.0
0.0	0.0	0.0	0.0	0.0	0.0	0.0	0.0
0.0	0.0	0.0	0.0	0.0	0.0	0.0	0.0
0.0	0.0	0.0	0.0	0.0	0.0	0.0	0.0
0.0	0.0	0.0	0.0	0.0	0.0	0.0	0.0
0.0	0.0	0.0	0.0	0.0	0.0	0.0	0.0
0.0	0.0	0.0	0.0	0.0	0.0	0.0	0.0
0.0	0.0	0.0	0.0	0.0	0.0	0.0	0.0
0.0	0.0	0.0	0.0	0.0	0.0	0.0	0.0
0.0	0.0	0.0	0.0	0.0	0.0	0.0	0.0
0.0	0.0	0.0	0.0	0.0	0.0	0.0	0.0
0.0	0.0	0.0	0.0	0.0	0.0	0.0	0.0
9.0	0.0	0.0	0.0	0.0	0.0	0.0	0.0
0.0	0.0	0.0	0.0	0.0	0.0	0.0	0.0
0.0	0.0	0.0	0.0	0.0	0.0	0.0	0.0
0.0	0.0	0.0	0.0	0.0	0.0	0.0	0.0
0.0	0.0	0.0	0.0	0.0	3.0	0.0	0.0
0.0	0.0	0.0	0.0	0.0	0.0	0.0	0.0
0.0	0.0	0.0	0.0	0.0	0.0	0.0	0.0
0.0	0.0	0.0	0.0	0.0	0.0	0.0	2.0
0.0	0.0	0.0	0.0	0.0	0.0	0.0	0.0
0.0	0.0	0.0	0.0	0.0	0.0	0.0	0.0
0.0	0.0	0.0	0.0	0.0	0.0	0.0	0.0
0.0	0.0	0.0	0.0	0.0	0.0	0.0	0.0
0.0	0.0	0.0	0.0	0.0	0.0	0.0	0.0
0.0	0.0	0.0	0.0	0.0	0.0	0.0	0.0
0.0	0.0	0.0	0.0	0.0	0.0	0.0	0.0
0.0	0.0	4.0	0.0	0.0	0.0	0.0	0.0
0.0	0.0	0.0	0.0	0.0	0.0	0.0	0.0
0.0	0.0	0.0	0.0	0.0	0.0	0.0	0.0
0.0	0.0	0.0	0.0	0.0	0.0	0.0	0.0
0.0	0.0	0.0	0.0	0.0	0.0	0.0	0.0
0.0	0.0	0.0	0.0	0.0	0.0	0.0	0.0
0.0	0.0	0.0	0.0	0.0	0.0	0.0	0.0
0.0	0.0	0.0	0.0	0.0	0.0	0.0	0.0
0.0	0.0	0.0	0.0	0.0	0.0	0.0	0.0
0.0	0.0	0.0	0.0	0.0	0.0	0.0	0.0
0.0	0.0	0.0	0.0	0.0	0.0	0.0	0.0
0.0	0.0	0.0	0.0	0.0	0.0	0.0	0.0
0.0	0.0	0.0	0.0	0.0	0.0	0.0	0.0
0.0	1.0	0.0	0.0	0.0	0.0	0.0	0.0
0.0	0.0	0.0	0.0	0.0	0.0	0.0	0.0
0.0	0.0	0.0	0.0	0.0	0.0	0.0	0.0

0.0	0.0	0.0	0.0	0.0	0.0	0.0	0.0
0.0	0.0	0.0	0.0	0.0	0.0	0.0	0.0
0.0	0.0	0.0	0.0	0.0	0.0	0.0	0.0
0.0	0.0	0.0	0.0	0.0	0.0	0.0	0.0
0.0	0.0	0.0	0.0	0.0	0.0	0.0	0.0
0.0	0.0	0.0	0.0	0.0	0.0	0.0	0.0
0.0	0.0	0.0	0.0	0.0	0.0	0.0	0.0
0.0	2.0	0.0	0.0	0.0	2.0	0.0	0.0
0.0	0.0	0.0	0.0	0.0	0.0	0.0	0.0
0.0	0.0	0.0	0.0	0.0	0.0	0.0	0.0
0.0	0.0	0.0	6.0	0.0	0.0	0.0	0.0
0.0	0.0	0.0	0.0	0.0	0.0	0.0	0.0
0.0	0.0	0.0	0.0	0.0	0.0	0.0	0.0
0.0	0.0	0.0	0.0	0.0	0.0	0.0	0.0
0.0	0.0	0.0	0.0	0.0	0.0	0.0	0.0
0.0	0.0	0.0	0.0	0.0	0.0	0.0	0.0
0.0	0.0	0.0	0.0	0.0	0.0	0.0	0.0
0.0	0.0	0.0	0.0	0.0	0.0	0.0	0.0
17.0	0.0	0.0	0.0	0.0	0.0	0.0	1.0
0.0	0.0	0.0	0.0	0.0	0.0	0.0	0.0
0.0	0.0	0.0	0.0	0.0	0.0	0.0	0.0
0.0	0.0	0.0	0.0	39.0	0.0	0.0	0.0
0.0	0.0	0.0	0.0	0.0	0.0	0.0	0.0
0.0	0.0	0.0	0.0	0.0	0.0	0.0	0.0
0.0	0.0	0.0	0.0	0.0	0.0	0.0	0.0
0.0	0.0	0.0	0.0	0.0	0.0	0.0	0.0
0.0	0.0	0.0	0.0	0.0	0.0	0.0	0.0
0.0	0.0	0.0	0.0	0.0	0.0	0.0	0.0
0.0	0.0	0.0	0.0	0.0	0.0	0.0	0.0
0.0	0.0	0.0	0.0	0.0	0.0	0.0	0.0
0.0	0.0	0.0	0.0	0.0	0.0	0.0	0.0
0.0	0.0	0.0	0.0	0.0	0.0	0.0	0.0
0.0	0.0	0.0	0.0	0.0	0.0	0.0	0.0
0.0	0.0	0.0	0.0	0.0	0.0	0.0	0.0
0.0	0.0	0.0	0.0	0.0	0.0	0.0	0.0
0.0	0.0	0.0	0.0	0.0	0.0	0.0	0.0
0.0	0.0	0.0	2.0	0.0	0.0	0.0	0.0
0.0	0.0	1.0	0.0	0.0	2.0	0.0	0.0
0.0	0.0	0.0	0.0	0.0	0.0	0.0	0.0
2.0	0.0	0.0	0.0	0.0	0.0	0.0	0.0
0.0	0.0	0.0	0.0	0.0	0.0	0.0	0.0
0.0	0.0	0.0	0.0	0.0	0.0	0.0	0.0
0.0	0.0	0.0	0.0	0.0	0.0	0.0	0.0
0.0	0.0	0.0	0.0	0.0	0.0	0.0	0.0
0.0	0.0	0.0	0.0	0.0	0.0	0.0	0.0
0.0	0.0	0.0	0.0	0.0	0.0	0.0	0.0
0.0	0.0	0.0	0.0	0.0	0.0	0.0	0.0

0.0	0.0	0.0	0.0	0.0	0.0	0.0	0.0
0.0	2.0	0.0	0.0	0.0	0.0	0.0	0.0
0.0	0.0	0.0	0.0	0.0	0.0	0.0	0.0
0.0	0.0	0.0	0.0	0.0	0.0	0.0	0.0
0.0	0.0	0.0	0.0	0.0	0.0	0.0	0.0
0.0	0.0	0.0	0.0	0.0	0.0	0.0	0.0
0.0	0.0	0.0	0.0	0.0	0.0	0.0	0.0
0.0	0.0	0.0	0.0	0.0	0.0	0.0	0.0
2.0	0.0	0.0	0.0	0.0	0.0	0.0	0.0
0.0	0.0	0.0	0.0	0.0	0.0	0.0	0.0
0.0	0.0	0.0	0.0	0.0	0.0	0.0	0.0
0.0	0.0	0.0	0.0	0.0	0.0	0.0	0.0
0.0	0.0	0.0	0.0	0.0	0.0	0.0	0.0
0.0	0.0	0.0	0.0	0.0	0.0	0.0	0.0
0.0	0.0	0.0	0.0	0.0	0.0	0.0	0.0
0.0	0.0	0.0	0.0	0.0	0.0	0.0	0.0
0.0	0.0	0.0	0.0	0.0	0.0	0.0	0.0
0.0	0.0	0.0	0.0	0.0	0.0	0.0	0.0
2.0	0.0	0.0	0.0	0.0	0.0	0.0	0.0
0.0	0.0	0.0	0.0	0.0	0.0	0.0	0.0
0.0	0.0	0.0	0.0	0.0	0.0	0.0	0.0
0.0	0.0	0.0	0.0	0.0	0.0	0.0	0.0
0.0	0.0	0.0	0.0	0.0	0.0	0.0	0.0
0.0	0.0	0.0	0.0	0.0	0.0	0.0	0.0
0.0	0.0	0.0	0.0	0.0	0.0	0.0	0.0
0.0	0.0	0.0	0.0	0.0	0.0	0.0	0.0
0.0	0.0	0.0	0.0	0.0	0.0	0.0	0.0
0.0	0.0	0.0	0.0	0.0	0.0	0.0	0.0
0.0	0.0	0.0	0.0	0.0	0.0	0.0	0.0
0.0	0.0	0.0	0.0	0.0	0.0	0.0	0.0
0.0	0.0	0.0	0.0	0.0	0.0	0.0	0.0
0.0	0.0	0.0	0.0	0.0	0.0	0.0	0.0
0.0	0.0	0.0	0.0	0.0	0.0	0.0	0.0
0.0	0.0	0.0	0.0	0.0	0.0	0.0	0.0
0.0	0.0	0.0	0.0	0.0	0.0	0.0	0.0
0.0	0.0	0.0	0.0	0.0	0.0	0.0	0.0
0.0	0.0	0.0	0.0	0.0	0.0	0.0	0.0
0.0	0.0	0.0	0.0	0.0	0.0	0.0	0.0
0.0	0.0	0.0	0.0	0.0	0.0	0.0	0.0
0.0	0.0	0.0	0.0	0.0	0.0	0.0	0.0
0.0	0.0	0.0	0.0	0.0	0.0	0.0	0.0
0.0	0.0	0.0	0.0	0.0	0.0	0.0	0.0
0.0	0.0	0.0	0.0	0.0	0.0	0.0	0.0
0.0	0.0	0.0	0.0	0.0	0.0	0.0	0.0
0.0	0.0	0.0	0.0	3.0	0.0	0.0	0.0
0.0	0.0	0.0	0.0	0.0	0.0	0.0	0.0
0.0	0.0	0.0	0.0	0.0	0.0	0.0	0.0

0.0	0.0	0.0	0.0	0.0	0.0	0.0	0.0
0.0	0.0	0.0	0.0	0.0	0.0	0.0	0.0
0.0	0.0	0.0	0.0	0.0	0.0	0.0	0.0
0.0	0.0	0.0	0.0	0.0	0.0	0.0	0.0
0.0	0.0	0.0	0.0	0.0	0.0	0.0	0.0
0.0	0.0	0.0	0.0	1.0	0.0	0.0	0.0
0.0	0.0	0.0	0.0	0.0	0.0	0.0	0.0
0.0	0.0	0.0	0.0	2.0	0.0	0.0	0.0
0.0	0.0	0.0	0.0	0.0	0.0	0.0	0.0
0.0	0.0	0.0	0.0	0.0	0.0	0.0	0.0
0.0	0.0	0.0	0.0	0.0	0.0	0.0	0.0
0.0	0.0	0.0	0.0	0.0	0.0	0.0	0.0
0.0	0.0	0.0	0.0	0.0	0.0	0.0	0.0
0.0	0.0	0.0	0.0	0.0	0.0	0.0	0.0
0.0	0.0	0.0	0.0	0.0	0.0	0.0	0.0
0.0	0.0	0.0	0.0	0.0	0.0	0.0	0.0
0.0	0.0	0.0	0.0	0.0	0.0	0.0	0.0
0.0	0.0	0.0	0.0	0.0	0.0	0.0	0.0
0.0	0.0	0.0	0.0	0.0	0.0	0.0	0.0
0.0	0.0	0.0	0.0	0.0	0.0	0.0	0.0
0.0	0.0	0.0	0.0	0.0	0.0	0.0	0.0
0.0	0.0	0.0	0.0	0.0	0.0	0.0	0.0
0.0	0.0	0.0	0.0	0.0	0.0	0.0	0.0
0.0	0.0	0.0	0.0	0.0	0.0	0.0	0.0
0.0	0.0	0.0	0.0	0.0	0.0	0.0	0.0
0.0	0.0	0.0	0.0	0.0	0.0	0.0	0.0
0.0	0.0	0.0	0.0	0.0	0.0	0.0	0.0
0.0	0.0	0.0	0.0	0.0	0.0	0.0	0.0
0.0	0.0	0.0	0.0	0.0	0.0	0.0	0.0
0.0	0.0	0.0	0.0	0.0	0.0	0.0	0.0
0.0	0.0	0.0	0.0	0.0	0.0	0.0	0.0
0.0	0.0	0.0	0.0	0.0	0.0	0.0	0.0
0.0	0.0	0.0	0.0	0.0	0.0	0.0	0.0
0.0	0.0	0.0	0.0	0.0	0.0	0.0	0.0
0.0	0.0	0.0	0.0	0.0	0.0	0.0	0.0
0.0	0.0	0.0	0.0	0.0	0.0	0.0	0.0
0.0	0.0	0.0	0.0	0.0	0.0	0.0	0.0
0.0	0.0	0.0	0.0	0.0	0.0	0.0	0.0
0.0	0.0	0.0	0.0	0.0	0.0	0.0	0.0
0.0	0.0	0.0	0.0	0.0	0.0	0.0	0.0
0.0	0.0	0.0	0.0	0.0	0.0	0.0	0.0
0.0	0.0	0.0	0.0	0.0	0.0	0.0	0.0
13.0	0.0	0.0	1.0	0.0	0.0	0.0	0.0
0.0	0.0	0.0	0.0	0.0	0.0	0.0	0.0
0.0	0.0	0.0	0.0	0.0	0.0	0.0	0.0
0.0	0.0	0.0	0.0	0.0	0.0	0.0	0.0
0.0	0.0	0.0	0.0	0.0	0.0	0.0	0.0

0.0	0.0	0.0	0.0	0.0	0.0	0.0	0.0
0.0	0.0	0.0	0.0	0.0	0.0	0.0	0.0
0.0	0.0	0.0	0.0	0.0	0.0	0.0	0.0
0.0	0.0	0.0	0.0	0.0	0.0	0.0	0.0
0.0	0.0	0.0	0.0	0.0	0.0	0.0	0.0
0.0	0.0	0.0	0.0	0.0	0.0	0.0	0.0
0.0	0.0	0.0	0.0	0.0	0.0	0.0	0.0
0.0	0.0	0.0	0.0	0.0	0.0	0.0	0.0
0.0	0.0	0.0	0.0	0.0	0.0	0.0	0.0
0.0	0.0	0.0	0.0	0.0	0.0	0.0	0.0
0.0	0.0	0.0	0.0	0.0	0.0	0.0	0.0
0.0	0.0	0.0	0.0	0.0	0.0	0.0	0.0
0.0	0.0	0.0	0.0	0.0	0.0	0.0	0.0
0.0	0.0	0.0	0.0	0.0	0.0	0.0	0.0
0.0	0.0	0.0	0.0	0.0	0.0	0.0	0.0
0.0	0.0	0.0	0.0	0.0	0.0	0.0	0.0
0.0	0.0	0.0	0.0	0.0	0.0	0.0	0.0
0.0	0.0	0.0	0.0	0.0	0.0	0.0	0.0
0.0	0.0	0.0	0.0	0.0	0.0	0.0	0.0
0.0	0.0	0.0	0.0	0.0	0.0	0.0	0.0
0.0	0.0	0.0	0.0	0.0	0.0	0.0	0.0
0.0	0.0	0.0	0.0	0.0	0.0	0.0	0.0
0.0	0.0	0.0	0.0	0.0	0.0	0.0	0.0
0.0	3.0	0.0	0.0	0.0	0.0	0.0	0.0
0.0	0.0	0.0	0.0	0.0	0.0	0.0	0.0
0.0	0.0	0.0	0.0	0.0	0.0	0.0	0.0
0.0	0.0	0.0	0.0	0.0	0.0	0.0	10.0
0.0	0.0	0.0	0.0	0.0	0.0	0.0	0.0
0.0	0.0	0.0	0.0	0.0	0.0	0.0	0.0
0.0	0.0	0.0	0.0	0.0	0.0	0.0	0.0
0.0	3.0	0.0	0.0	0.0	0.0	7.0	0.0
0.0	0.0	0.0	0.0	0.0	0.0	0.0	0.0
0.0	0.0	0.0	0.0	0.0	0.0	0.0	0.0
0.0	0.0	0.0	0.0	0.0	0.0	0.0	0.0
0.0	0.0	0.0	0.0	0.0	0.0	0.0	0.0
0.0	0.0	0.0	0.0	0.0	0.0	0.0	5.0
0.0	0.0	0.0	0.0	0.0	0.0	0.0	0.0
0.0	0.0	0.0	0.0	0.0	0.0	0.0	0.0
0.0	0.0	0.0	4.0	0.0	0.0	0.0	0.0
0.0	0.0	0.0	0.0	0.0	0.0	0.0	0.0
0.0	0.0	0.0	0.0	0.0	0.0	0.0	0.0
0.0	0.0	0.0	0.0	0.0	0.0	0.0	0.0
0.0	0.0	0.0	0.0	0.0	0.0	0.0	0.0
0.0	0.0	0.0	0.0	0.0	0.0	0.0	0.0
0.0	0.0	0.0	0.0	0.0	0.0	0.0	0.0
0.0	0.0	0.0	0.0	0.0	0.0	0.0	0.0
0.0	0.0	0.0	0.0	0.0	0.0	0.0	0.0

0.0	0.0	0.0	0.0	0.0	0.0	0.0	0.0
0.0	0.0	0.0	0.0	0.0	0.0	0.0	0.0
0.0	0.0	0.0	0.0	0.0	0.0	0.0	0.0
0.0	0.0	0.0	0.0	0.0	0.0	0.0	0.0
0.0	0.0	0.0	0.0	0.0	0.0	0.0	0.0
0.0	0.0	0.0	0.0	0.0	0.0	0.0	0.0
0.0	0.0	0.0	0.0	0.0	0.0	0.0	0.0
0.0	0.0	0.0	0.0	0.0	0.0	0.0	0.0
0.0	0.0	0.0	0.0	0.0	0.0	0.0	0.0
0.0	0.0	0.0	0.0	0.0	0.0	0.0	0.0
0.0	0.0	0.0	0.0	0.0	0.0	0.0	0.0
0.0	0.0	0.0	0.0	0.0	0.0	0.0	0.0
0.0	0.0	0.0	0.0	0.0	0.0	0.0	0.0
0.0	0.0	0.0	0.0	0.0	0.0	0.0	0.0
0.0	0.0	0.0	0.0	0.0	0.0	0.0	0.0
0.0	0.0	0.0	0.0	0.0	0.0	0.0	0.0
0.0	0.0	0.0	0.0	0.0	0.0	0.0	0.0
0.0	0.0	0.0	0.0	0.0	0.0	0.0	0.0
0.0	0.0	0.0	0.0	0.0	0.0	0.0	0.0
0.0	0.0	0.0	0.0	0.0	0.0	0.0	0.0
0.0	0.0	0.0	0.0	0.0	0.0	0.0	0.0
0.0	0.0	0.0	0.0	0.0	0.0	0.0	0.0
0.0	0.0	0.0	0.0	0.0	0.0	0.0	0.0
0.0	0.0	0.0	0.0	0.0	0.0	0.0	0.0
0.0	0.0	0.0	0.0	0.0	0.0	0.0	0.0
0.0	0.0	0.0	0.0	0.0	0.0	0.0	0.0
0.0	0.0	0.0	0.0	0.0	0.0	0.0	0.0
0.0	0.0	0.0	0.0	0.0	0.0	0.0	0.0
0.0	0.0	0.0	0.0	0.0	0.0	0.0	0.0
0.0	0.0	0.0	0.0	0.0	0.0	0.0	0.0
0.0	0.0	0.0	0.0	0.0	0.0	0.0	0.0
0.0	0.0	0.0	0.0	0.0	0.0	0.0	0.0
0.0	0.0	0.0	0.0	0.0	0.0	0.0	0.0
0.0	0.0	0.0	0.0	0.0	0.0	0.0	0.0
0.0	0.0	0.0	0.0	0.0	0.0	0.0	0.0
0.0	0.0	0.0	0.0	0.0	0.0	0.0	0.0
0.0	0.0	0.0	0.0	0.0	0.0	0.0	0.0
0.0	0.0	0.0	9.0	0.0	0.0	0.0	0.0
0.0	0.0	0.0	0.0	0.0	0.0	0.0	0.0
0.0	0.0	0.0	0.0	0.0	0.0	0.0	0.0
0.0	0.0	0.0	0.0	0.0	0.0	0.0	0.0
0.0	0.0	0.0	0.0	0.0	0.0	0.0	0.0
0.0	0.0	0.0	0.0	0.0	0.0	0.0	0.0
0.0	0.0	0.0	0.0	0.0	0.0	0.0	0.0
0.0	0.0	0.0	0.0	0.0	0.0	0.0	0.0
0.0	0.0	0.0	0.0	0.0	0.0	0.0	0.0
0.0	0.0	0.0	0.0	0.0	0.0	0.0	0.0

0.0	0.0	0.0	0.0	0.0	0.0	0.0	0.0
0.0	0.0	0.0	0.0	0.0	0.0	0.0	0.0
0.0	0.0	0.0	0.0	0.0	0.0	0.0	0.0
0.0	0.0	0.0	0.0	0.0	0.0	0.0	0.0
0.0	0.0	0.0	0.0	0.0	0.0	0.0	0.0
0.0	0.0	0.0	0.0	0.0	0.0	0.0	0.0
0.0	0.0	0.0	0.0	0.0	0.0	0.0	0.0
0.0	0.0	0.0	0.0	0.0	0.0	0.0	0.0
0.0	0.0	0.0	0.0	0.0	0.0	0.0	0.0
0.0	0.0	0.0	0.0	0.0	0.0	0.0	0.0
0.0	0.0	0.0	0.0	0.0	0.0	0.0	0.0
0.0	0.0	0.0	0.0	0.0	0.0	0.0	0.0
0.0	0.0	0.0	0.0	0.0	0.0	0.0	0.0
0.0	0.0	0.0	0.0	0.0	0.0	0.0	0.0
0.0	0.0	0.0	0.0	0.0	0.0	0.0	0.0
0.0	0.0	0.0	0.0	0.0	0.0	0.0	0.0
0.0	0.0	0.0	0.0	0.0	0.0	0.0	0.0
0.0	0.0	0.0	0.0	0.0	0.0	0.0	0.0
0.0	0.0	0.0	0.0	0.0	0.0	0.0	0.0
0.0	0.0	0.0	0.0	0.0	0.0	0.0	0.0
0.0	0.0	0.0	0.0	0.0	0.0	0.0	0.0
0.0	0.0	0.0	0.0	0.0	0.0	0.0	0.0
0.0	0.0	0.0	0.0	0.0	0.0	0.0	0.0
0.0	0.0	0.0	0.0	0.0	0.0	0.0	0.0
0.0	0.0	0.0	0.0	0.0	0.0	0.0	0.0
0.0	0.0	0.0	0.0	0.0	0.0	0.0	0.0
0.0	0.0	0.0	0.0	0.0	0.0	0.0	0.0
0.0	0.0	0.0	0.0	0.0	0.0	0.0	0.0
0.0	0.0	0.0	0.0	0.0	0.0	0.0	0.0
0.0	0.0	0.0	0.0	0.0	0.0	0.0	0.0
0.0	0.0	0.0	0.0	0.0	0.0	0.0	0.0
0.0	0.0	0.0	0.0	0.0	0.0	0.0	0.0
0.0	0.0	0.0	0.0	0.0	0.0	0.0	0.0
0.0	0.0	0.0	0.0	0.0	0.0	0.0	0.0
0.0	0.0	0.0	0.0	0.0	0.0	0.0	0.0
0.0	0.0	0.0	0.0	0.0	0.0	0.0	0.0
0.0	0.0	0.0	0.0	0.0	0.0	0.0	0.0
0.0	0.0	0.0	0.0	0.0	6.0	0.0	0.0
0.0	0.0	0.0	0.0	0.0	0.0	0.0	0.0
0.0	0.0	0.0	0.0	0.0	0.0	0.0	0.0
0.0	0.0	0.0	0.0	0.0	0.0	0.0	0.0
0.0	0.0	0.0	0.0	0.0	0.0	0.0	0.0
0.0	0.0	0.0	0.0	0.0	0.0	0.0	0.0
0.0	0.0	0.0	0.0	0.0	0.0	0.0	0.0
0.0	0.0	0.0	0.0	0.0	0.0	0.0	0.0
0.0	0.0	0.0	0.0	0.0	0.0	0.0	0.0
0.0	0.0	0.0	0.0	0.0	0.0	0.0	0.0

0.0	0.0	0.0	0.0	0.0	0.0	0.0	0.0
0.0	0.0	0.0	0.0	0.0	0.0	0.0	0.0
0.0	0.0	0.0	0.0	0.0	0.0	0.0	0.0
0.0	0.0	0.0	0.0	0.0	0.0	0.0	0.0
0.0	0.0	0.0	0.0	0.0	0.0	0.0	0.0
0.0	0.0	0.0	0.0	0.0	0.0	0.0	0.0
0.0	0.0	0.0	0.0	0.0	0.0	0.0	0.0
0.0	0.0	0.0	0.0	0.0	0.0	0.0	0.0
0.0	1.0	0.0	0.0	0.0	0.0	0.0	0.0
41.0	0.0	0.0	0.0	0.0	0.0	0.0	0.0
0.0	0.0	0.0	0.0	0.0	0.0	0.0	0.0
0.0	0.0	0.0	0.0	0.0	0.0	0.0	4.0
0.0	0.0	0.0	0.0	0.0	0.0	0.0	0.0
0.0	0.0	0.0	0.0	0.0	0.0	0.0	0.0
0.0	0.0	0.0	0.0	0.0	0.0	0.0	0.0
0.0	0.0	0.0	0.0	0.0	0.0	0.0	0.0
0.0	0.0	0.0	0.0	0.0	0.0	0.0	0.0
4.0	0.0	0.0	0.0	0.0	0.0	0.0	0.0
0.0	0.0	0.0	0.0	0.0	0.0	0.0	0.0
0.0	0.0	0.0	0.0	0.0	0.0	0.0	0.0
0.0	0.0	0.0	0.0	0.0	0.0	0.0	1.0
0.0	0.0	0.0	0.0	0.0	0.0	0.0	0.0
0.0	0.0	0.0	0.0	0.0	0.0	0.0	0.0
0.0	0.0	0.0	0.0	0.0	0.0	0.0	0.0
0.0	0.0	0.0	0.0	0.0	0.0	0.0	0.0
0.0	0.0	0.0	0.0	0.0	0.0	0.0	0.0
0.0	0.0	0.0	0.0	0.0	0.0	0.0	0.0
0.0	0.0	0.0	0.0	0.0	0.0	0.0	0.0
0.0	0.0	1.0	0.0	0.0	0.0	0.0	0.0
0.0	0.0	0.0	0.0	0.0	0.0	0.0	0.0
0.0	0.0	0.0	0.0	0.0	0.0	0.0	0.0
0.0	0.0	0.0	0.0	0.0	0.0	0.0	0.0
0.0	0.0	0.0	0.0	0.0	0.0	0.0	0.0
0.0	0.0	0.0	0.0	0.0	0.0	0.0	0.0
0.0	0.0	0.0	0.0	0.0	0.0	0.0	0.0
0.0	0.0	0.0	0.0	0.0	0.0	0.0	0.0
0.0	0.0	0.0	0.0	0.0	0.0	0.0	0.0
0.0	0.0	0.0	0.0	0.0	0.0	0.0	0.0
0.0	0.0	0.0	0.0	0.0	0.0	0.0	0.0
0.0	0.0	0.0	0.0	0.0	0.0	0.0	0.0
0.0	0.0	0.0	0.0	0.0	0.0	0.0	0.0
0.0	0.0	0.0	0.0	0.0	0.0	0.0	0.0
0.0	0.0	0.0	0.0	0.0	0.0	0.0	0.0
0.0	0.0	0.0	0.0	0.0	0.0	0.0	0.0
0.0	0.0	0.0	0.0	0.0	0.0	0.0	0.0
0.0	0.0	0.0	0.0	0.0	0.0	0.0	0.0
0.0	0.0	0.0	0.0	0.0	0.0	0.0	0.0

0.0	0.0	0.0	0.0	0.0	0.0	0.0	0.0
0.0	0.0	0.0	0.0	0.0	0.0	0.0	0.0
0.0	0.0	0.0	0.0	0.0	0.0	0.0	0.0
0.0	0.0	0.0	0.0	0.0	0.0	0.0	0.0
0.0	0.0	0.0	0.0	0.0	0.0	0.0	0.0
0.0	0.0	0.0	0.0	0.0	0.0	0.0	0.0
0.0	0.0	0.0	0.0	0.0	0.0	0.0	0.0
0.0	7.0	0.0	3.0	0.0	0.0	0.0	0.0
0.0	0.0	0.0	0.0	0.0	0.0	0.0	0.0
0.0	0.0	0.0	0.0	0.0	0.0	0.0	0.0
0.0	0.0	0.0	0.0	0.0	0.0	0.0	0.0
0.0	0.0	0.0	1.0	0.0	0.0	0.0	0.0
0.0	0.0	0.0	0.0	0.0	0.0	0.0	0.0
0.0	0.0	0.0	0.0	0.0	0.0	0.0	0.0
0.0	0.0	0.0	0.0	0.0	0.0	0.0	0.0
0.0	0.0	0.0	0.0	0.0	0.0	0.0	0.0
0.0	0.0	0.0	0.0	0.0	0.0	0.0	0.0
0.0	0.0	0.0	0.0	0.0	0.0	0.0	0.0
0.0	0.0	0.0	0.0	0.0	0.0	0.0	0.0
0.0	4.0	0.0	0.0	0.0	0.0	0.0	0.0
0.0	0.0	0.0	0.0	0.0	0.0	0.0	0.0
0.0	0.0	0.0	0.0	0.0	0.0	0.0	0.0
0.0	0.0	0.0	0.0	0.0	0.0	0.0	0.0
0.0	0.0	0.0	0.0	0.0	0.0	1.0	0.0
0.0	0.0	0.0	0.0	0.0	0.0	0.0	0.0
0.0	0.0	0.0	0.0	0.0	0.0	0.0	0.0
0.0	0.0	0.0	0.0	0.0	0.0	0.0	0.0
0.0	0.0	0.0	0.0	0.0	0.0	0.0	0.0
0.0	0.0	0.0	0.0	0.0	0.0	0.0	0.0
0.0	0.0	0.0	0.0	0.0	0.0	0.0	0.0
0.0	0.0	0.0	0.0	0.0	0.0	0.0	0.0
0.0	0.0	0.0	0.0	0.0	0.0	0.0	0.0
0.0	0.0	0.0	0.0	0.0	0.0	0.0	0.0
0.0	0.0	0.0	0.0	0.0	0.0	0.0	0.0
0.0	0.0	0.0	0.0	0.0	0.0	0.0	0.0
0.0	0.0	0.0	0.0	0.0	0.0	0.0	0.0
0.0	0.0	0.0	0.0	0.0	0.0	0.0	0.0
0.0	0.0	0.0	0.0	0.0	0.0	0.0	0.0
0.0	0.0	0.0	0.0	0.0	0.0	0.0	0.0
0.0	0.0	0.0	0.0	0.0	0.0	0.0	0.0
0.0	0.0	0.0	0.0	0.0	0.0	0.0	0.0
0.0	0.0	0.0	0.0	0.0	0.0	0.0	0.0
0.0	0.0	0.0	0.0	0.0	0.0	0.0	0.0
0.0	0.0	0.0	0.0	0.0	0.0	0.0	0.0
0.0	0.0	0.0	0.0	0.0	0.0	0.0	0.0
0.0	0.0	0.0	0.0	0.0	0.0	2.0	0.0
0.0	0.0	0.0	0.0	0.0	0.0	0.0	0.0

0.0	0.0	0.0	0.0	0.0	0.0	0.0	0.0
0.0	0.0	0.0	0.0	0.0	0.0	0.0	0.0
0.0	0.0	0.0	0.0	0.0	0.0	0.0	0.0
0.0	0.0	0.0	0.0	0.0	0.0	0.0	0.0
0.0	0.0	0.0	0.0	0.0	0.0	0.0	0.0
0.0	0.0	0.0	0.0	0.0	0.0	0.0	0.0
0.0	0.0	0.0	0.0	0.0	0.0	0.0	0.0
0.0	0.0	0.0	0.0	0.0	0.0	0.0	0.0
0.0	0.0	0.0	0.0	0.0	0.0	0.0	0.0
0.0	0.0	0.0	0.0	0.0	0.0	0.0	0.0
0.0	0.0	0.0	0.0	0.0	0.0	0.0	0.0
0.0	0.0	0.0	0.0	0.0	0.0	0.0	0.0
0.0	0.0	0.0	0.0	0.0	0.0	0.0	0.0
0.0	0.0	0.0	0.0	0.0	0.0	0.0	0.0
0.0	0.0	0.0	0.0	0.0	0.0	0.0	0.0
0.0	0.0	0.0	0.0	0.0	0.0	0.0	0.0
0.0	0.0	0.0	0.0	0.0	0.0	0.0	0.0
0.0	0.0	0.0	0.0	0.0	0.0	0.0	0.0
0.0	0.0	0.0	0.0	0.0	0.0	0.0	0.0
0.0	0.0	0.0	0.0	0.0	0.0	0.0	0.0
0.0	0.0	0.0	0.0	0.0	0.0	0.0	0.0
0.0	0.0	0.0	0.0	0.0	0.0	0.0	0.0
0.0	0.0	0.0	0.0	0.0	0.0	0.0	0.0
0.0	0.0	0.0	0.0	0.0	0.0	0.0	0.0
0.0	0.0	0.0	0.0	0.0	0.0	0.0	0.0
0.0	0.0	0.0	0.0	0.0	0.0	0.0	0.0
0.0	0.0	0.0	0.0	0.0	0.0	0.0	0.0
0.0	0.0	0.0	0.0	0.0	0.0	0.0	0.0
0.0	0.0	0.0	0.0	0.0	0.0	0.0	0.0
0.0	0.0	0.0	0.0	0.0	0.0	0.0	0.0
0.0	0.0	0.0	0.0	0.0	0.0	0.0	0.0
0.0	0.0	0.0	0.0	0.0	0.0	0.0	0.0
0.0	0.0	0.0	0.0	0.0	0.0	0.0	0.0
0.0	0.0	0.0	0.0	0.0	0.0	0.0	0.0
0.0	0.0	0.0	0.0	0.0	0.0	0.0	0.0
0.0	0.0	0.0	0.0	0.0	0.0	0.0	0.0
0.0	0.0	0.0	0.0	0.0	0.0	0.0	0.0
0.0	0.0	0.0	0.0	0.0	0.0	0.0	0.0
0.0	0.0	0.0	0.0	0.0	0.0	0.0	0.0
0.0	0.0	0.0	0.0	0.0	0.0	0.0	0.0
0.0	0.0	0.0	0.0	0.0	0.0	0.0	0.0
0.0	0.0	0.0	0.0	0.0	0.0	0.0	0.0
0.0	0.0	0.0	0.0	0.0	0.0	0.0	0.0
0.0	0.0	0.0	0.0	0.0	0.0	0.0	0.0
0.0	0.0	0.0	0.0	0.0	0.0	0.0	0.0
6.0	0.0	0.0	0.0	0.0	0.0	0.0	0.0
0.0	0.0	0.0	0.0	0.0	0.0	0.0	0.0

0.0	0.0	0.0	0.0	0.0	0.0	0.0	0.0
0.0	0.0	0.0	0.0	0.0	0.0	0.0	0.0
0.0	0.0	0.0	0.0	0.0	0.0	0.0	0.0
0.0	24.0	0.0	0.0	0.0	0.0	0.0	0.0
0.0	0.0	0.0	0.0	0.0	0.0	0.0	0.0
0.0	0.0	0.0	0.0	0.0	0.0	0.0	0.0
0.0	31.0	0.0	0.0	0.0	0.0	0.0	0.0
0.0	0.0	0.0	0.0	0.0	0.0	0.0	0.0
0.0	0.0	0.0	0.0	0.0	0.0	0.0	0.0
0.0	0.0	0.0	0.0	0.0	0.0	0.0	0.0
297.0	0.0	0.0	0.0	0.0	0.0	0.0	0.0
0.0	0.0	0.0	0.0	0.0	0.0	0.0	0.0
0.0	0.0	0.0	0.0	0.0	0.0	0.0	0.0
0.0	0.0	0.0	0.0	0.0	0.0	0.0	1.0
0.0	0.0	0.0	0.0	0.0	0.0	0.0	0.0
0.0	0.0	0.0	0.0	0.0	0.0	0.0	0.0
0.0	0.0	0.0	0.0	0.0	0.0	0.0	0.0
0.0	0.0	0.0	1.0	0.0	0.0	0.0	0.0
0.0	0.0	0.0	0.0	0.0	0.0	0.0	0.0
0.0	0.0	0.0	0.0	0.0	0.0	0.0	0.0
0.0	0.0	0.0	0.0	0.0	0.0	0.0	0.0
0.0	0.0	0.0	0.0	0.0	2.0	0.0	0.0
0.0	0.0	0.0	0.0	0.0	0.0	0.0	0.0
0.0	0.0	0.0	0.0	0.0	0.0	0.0	0.0
0.0	0.0	0.0	0.0	0.0	0.0	0.0	0.0
0.0	0.0	0.0	0.0	0.0	0.0	0.0	0.0
0.0	0.0	0.0	0.0	0.0	0.0	0.0	0.0
2.0	1.0	0.0	1.0	0.0	0.0	0.0	0.0
0.0	0.0	0.0	0.0	0.0	0.0	0.0	0.0
0.0	0.0	0.0	0.0	0.0	0.0	0.0	0.0
0.0	0.0	0.0	0.0	0.0	0.0	0.0	0.0
0.0	0.0	0.0	0.0	0.0	0.0	0.0	0.0
0.0	0.0	0.0	0.0	0.0	0.0	0.0	0.0
0.0	0.0	0.0	0.0	0.0	0.0	0.0	0.0
0.0	0.0	0.0	0.0	0.0	0.0	0.0	0.0
0.0	0.0	0.0	0.0	0.0	0.0	0.0	0.0
0.0	0.0	0.0	0.0	0.0	0.0	0.0	0.0
0.0	0.0	0.0	0.0	0.0	0.0	0.0	0.0
0.0	0.0	0.0	0.0	0.0	0.0	0.0	0.0
0.0	0.0	0.0	0.0	0.0	0.0	0.0	0.0
0.0	0.0	0.0	0.0	0.0	0.0	0.0	0.0
0.0	0.0	0.0	0.0	0.0	0.0	0.0	0.0
0.0	0.0	0.0	0.0	0.0	0.0	0.0	0.0
0.0	0.0	0.0	0.0	0.0	0.0	0.0	0.0
0.0	0.0	0.0	0.0	0.0	0.0	0.0	0.0
0.0	0.0	0.0	0.0	0.0	0.0	0.0	0.0
0.0	0.0	0.0	0.0	0.0	0.0	0.0	0.0

0.0	0.0	0.0	0.0	0.0	0.0	0.0	0.0
0.0	0.0	0.0	0.0	0.0	0.0	0.0	0.0
0.0	0.0	0.0	0.0	0.0	0.0	0.0	0.0
0.0	0.0	0.0	0.0	0.0	0.0	0.0	0.0
0.0	0.0	0.0	0.0	0.0	0.0	0.0	0.0
0.0	0.0	0.0	0.0	0.0	0.0	0.0	0.0
0.0	0.0	0.0	0.0	0.0	0.0	0.0	0.0
0.0	0.0	0.0	0.0	0.0	0.0	0.0	0.0
0.0	0.0	0.0	0.0	0.0	0.0	0.0	0.0
0.0	0.0	0.0	0.0	0.0	0.0	0.0	0.0
0.0	0.0	0.0	0.0	0.0	0.0	0.0	0.0
0.0	0.0	0.0	0.0	0.0	226.0	0.0	0.0
0.0	0.0	0.0	0.0	0.0	0.0	0.0	0.0
0.0	0.0	0.0	0.0	0.0	0.0	0.0	0.0
0.0	0.0	0.0	0.0	0.0	0.0	0.0	0.0
0.0	0.0	0.0	0.0	0.0	0.0	0.0	0.0
0.0	0.0	0.0	0.0	0.0	0.0	0.0	0.0
0.0	0.0	0.0	0.0	0.0	0.0	0.0	0.0
0.0	0.0	2.0	0.0	0.0	0.0	0.0	0.0
0.0	0.0	0.0	0.0	0.0	0.0	0.0	0.0
0.0	0.0	0.0	0.0	0.0	0.0	0.0	0.0
4.0	0.0	0.0	0.0	0.0	1.0	0.0	0.0
0.0	0.0	0.0	0.0	0.0	0.0	0.0	0.0
0.0	0.0	0.0	0.0	0.0	0.0	0.0	0.0
0.0	0.0	0.0	0.0	0.0	0.0	0.0	0.0
0.0	0.0	0.0	0.0	0.0	0.0	0.0	0.0
0.0	0.0	0.0	0.0	0.0	0.0	0.0	0.0
0.0	0.0	0.0	0.0	0.0	0.0	0.0	0.0
0.0	0.0	0.0	0.0	0.0	0.0	0.0	0.0
0.0	0.0	0.0	0.0	0.0	0.0	0.0	0.0
0.0	0.0	0.0	0.0	0.0	0.0	0.0	0.0
0.0	0.0	0.0	0.0	0.0	0.0	0.0	0.0
0.0	0.0	0.0	0.0	0.0	0.0	0.0	0.0
0.0	0.0	0.0	0.0	0.0	0.0	0.0	0.0
0.0	0.0	0.0	0.0	0.0	0.0	0.0	0.0
0.0	0.0	0.0	0.0	0.0	0.0	0.0	0.0
0.0	0.0	0.0	0.0	0.0	0.0	0.0	0.0
0.0	0.0	0.0	0.0	0.0	0.0	0.0	0.0
0.0	0.0	0.0	0.0	0.0	0.0	0.0	0.0
0.0	0.0	0.0	0.0	0.0	0.0	0.0	0.0
0.0	0.0	0.0	0.0	0.0	0.0	0.0	0.0
0.0	0.0	0.0	0.0	0.0	0.0	0.0	0.0
0.0	0.0	0.0	0.0	0.0	0.0	0.0	0.0
0.0	0.0	0.0	0.0	0.0	0.0	0.0	0.0
0.0	0.0	0.0	0.0	0.0	0.0	0.0	0.0
0.0	0.0	0.0	0.0	0.0	0.0	0.0	0.0
0.0	0.0	0.0	0.0	0.0	0.0	0.0	0.0
0.0	0.0	0.0	0.0	0.0	0.0	0.0	0.0

0.0	0.0	0.0	0.0	0.0	0.0	0.0	0.0
0.0	0.0	0.0	0.0	0.0	0.0	0.0	0.0
0.0	0.0	0.0	0.0	0.0	0.0	0.0	0.0
0.0	0.0	0.0	0.0	0.0	0.0	0.0	0.0
0.0	0.0	0.0	0.0	0.0	0.0	0.0	0.0
0.0	0.0	0.0	0.0	0.0	0.0	0.0	16.0
0.0	0.0	0.0	0.0	0.0	0.0	0.0	0.0
0.0	0.0	0.0	0.0	0.0	0.0	0.0	0.0
0.0	0.0	0.0	0.0	0.0	0.0	0.0	0.0
0.0	0.0	0.0	0.0	0.0	0.0	0.0	0.0
0.0	0.0	0.0	0.0	0.0	0.0	0.0	1.0
0.0	0.0	0.0	0.0	0.0	0.0	0.0	0.0
0.0	0.0	0.0	0.0	0.0	0.0	0.0	0.0
0.0	0.0	0.0	0.0	0.0	0.0	0.0	0.0
0.0	0.0	0.0	0.0	0.0	0.0	0.0	0.0
0.0	0.0	0.0	0.0	0.0	0.0	0.0	0.0
0.0	0.0	0.0	0.0	0.0	0.0	0.0	0.0
0.0	0.0	0.0	0.0	0.0	0.0	0.0	0.0
0.0	0.0	0.0	0.0	0.0	0.0	0.0	0.0
0.0	0.0	0.0	0.0	0.0	0.0	0.0	0.0
0.0	0.0	0.0	0.0	0.0	0.0	0.0	0.0
0.0	0.0	0.0	0.0	0.0	0.0	0.0	0.0
0.0	0.0	0.0	0.0	0.0	0.0	0.0	0.0
0.0	0.0	0.0	0.0	0.0	0.0	0.0	0.0
0.0	0.0	0.0	0.0	0.0	0.0	0.0	0.0
0.0	0.0	0.0	0.0	0.0	0.0	0.0	0.0
0.0	0.0	0.0	0.0	0.0	0.0	0.0	0.0
6.0	0.0	0.0	1.0	0.0	0.0	0.0	0.0
0.0	0.0	0.0	0.0	0.0	0.0	0.0	0.0
0.0	0.0	0.0	0.0	0.0	0.0	0.0	0.0
0.0	0.0	0.0	0.0	0.0	0.0	0.0	0.0
0.0	0.0	0.0	0.0	0.0	0.0	0.0	0.0
0.0	0.0	0.0	1.0	0.0	0.0	0.0	0.0
0.0	0.0	0.0	0.0	0.0	0.0	0.0	0.0
0.0	0.0	0.0	0.0	0.0	0.0	0.0	0.0
0.0	0.0	0.0	0.0	0.0	0.0	0.0	0.0
0.0	0.0	0.0	0.0	0.0	0.0	0.0	0.0
0.0	0.0	0.0	0.0	0.0	0.0	0.0	0.0
0.0	0.0	0.0	0.0	0.0	0.0	0.0	0.0
0.0	0.0	0.0	0.0	0.0	0.0	0.0	0.0
0.0	0.0	0.0	0.0	0.0	0.0	0.0	0.0
0.0	0.0	0.0	0.0	0.0	0.0	0.0	0.0
0.0	0.0	0.0	0.0	0.0	0.0	0.0	0.0
0.0	0.0	0.0	0.0	0.0	0.0	0.0	0.0
0.0	0.0	0.0	0.0	0.0	0.0	0.0	0.0
0.0	0.0	0.0	0.0	0.0	0.0	0.0	0.0
0.0	0.0	0.0	0.0	0.0	0.0	0.0	0.0

0.0	0.0	0.0	0.0	0.0	0.0	0.0	0.0
1.0	0.0	0.0	0.0	0.0	0.0	0.0	0.0
0.0	0.0	0.0	0.0	0.0	0.0	0.0	0.0
0.0	0.0	0.0	0.0	0.0	0.0	0.0	0.0
0.0	0.0	0.0	0.0	0.0	0.0	0.0	0.0
0.0	0.0	0.0	0.0	0.0	0.0	0.0	1.0
0.0	0.0	0.0	0.0	0.0	0.0	0.0	0.0
0.0	0.0	0.0	0.0	0.0	0.0	0.0	0.0
0.0	0.0	0.0	0.0	0.0	0.0	0.0	0.0
0.0	0.0	0.0	0.0	0.0	0.0	0.0	0.0
0.0	0.0	0.0	0.0	0.0	0.0	0.0	0.0
0.0	0.0	0.0	0.0	0.0	0.0	0.0	0.0
0.0	0.0	0.0	0.0	0.0	0.0	0.0	0.0
0.0	0.0	0.0	0.0	0.0	0.0	0.0	0.0
0.0	0.0	0.0	0.0	0.0	0.0	0.0	0.0
0.0	0.0	0.0	0.0	0.0	0.0	0.0	0.0
0.0	0.0	0.0	0.0	0.0	0.0	0.0	0.0
0.0	0.0	0.0	0.0	0.0	0.0	0.0	0.0
0.0	0.0	0.0	0.0	0.0	0.0	0.0	0.0
0.0	0.0	0.0	0.0	0.0	0.0	0.0	0.0
0.0	0.0	0.0	0.0	0.0	0.0	0.0	0.0
0.0	0.0	0.0	0.0	0.0	0.0	0.0	0.0
0.0	0.0	0.0	0.0	0.0	0.0	0.0	0.0
0.0	0.0	0.0	0.0	0.0	0.0	0.0	0.0
0.0	0.0	0.0	0.0	0.0	0.0	0.0	0.0
0.0	0.0	0.0	0.0	0.0	0.0	0.0	0.0
0.0	0.0	0.0	0.0	0.0	0.0	0.0	0.0
0.0	0.0	0.0	0.0	0.0	0.0	0.0	13.0
0.0	0.0	0.0	0.0	0.0	0.0	0.0	0.0
0.0	0.0	0.0	0.0	0.0	0.0	0.0	0.0
0.0	0.0	0.0	0.0	0.0	0.0	0.0	0.0
0.0	0.0	0.0	0.0	0.0	0.0	0.0	0.0
0.0	0.0	0.0	0.0	0.0	0.0	0.0	0.0
0.0	0.0	17.0	0.0	0.0	0.0	0.0	3.0
0.0	0.0	0.0	0.0	0.0	0.0	0.0	0.0
0.0	0.0	0.0	0.0	0.0	0.0	0.0	0.0
0.0	0.0	0.0	0.0	0.0	0.0	0.0	0.0
0.0	0.0	0.0	0.0	0.0	0.0	0.0	0.0
0.0	0.0	0.0	0.0	0.0	0.0	0.0	2.0
0.0	0.0	0.0	0.0	0.0	0.0	0.0	0.0
0.0	0.0	0.0	0.0	0.0	0.0	0.0	0.0
0.0	0.0	0.0	0.0	0.0	0.0	0.0	0.0
0.0	0.0	0.0	0.0	0.0	0.0	0.0	0.0
0.0	0.0	0.0	0.0	0.0	0.0	0.0	0.0
0.0	0.0	0.0	0.0	0.0	0.0	0.0	0.0
0.0	0.0	0.0	0.0	0.0	0.0	0.0	0.0
0.0	0.0	0.0	0.0	0.0	0.0	0.0	0.0

0.0	0.0	0.0	0.0	0.0	0.0	0.0	0.0
0.0	0.0	0.0	0.0	0.0	0.0	0.0	0.0
0.0	0.0	0.0	0.0	0.0	0.0	0.0	0.0
0.0	0.0	0.0	0.0	0.0	0.0	0.0	0.0
0.0	0.0	0.0	0.0	0.0	0.0	0.0	0.0
0.0	0.0	0.0	0.0	0.0	0.0	0.0	0.0
0.0	0.0	0.0	0.0	0.0	0.0	0.0	0.0
0.0	0.0	0.0	0.0	0.0	0.0	0.0	0.0
0.0	0.0	0.0	0.0	0.0	0.0	0.0	0.0
0.0	0.0	0.0	0.0	0.0	0.0	0.0	0.0
0.0	0.0	0.0	0.0	0.0	0.0	0.0	0.0
0.0	0.0	0.0	0.0	0.0	0.0	1.0	0.0
0.0	0.0	0.0	0.0	0.0	0.0	0.0	0.0
0.0	0.0	0.0	0.0	0.0	0.0	0.0	0.0
0.0	0.0	0.0	0.0	0.0	0.0	0.0	0.0
0.0	0.0	0.0	0.0	0.0	0.0	0.0	0.0
0.0	0.0	0.0	0.0	0.0	0.0	0.0	0.0
0.0	0.0	0.0	0.0	0.0	0.0	0.0	0.0
0.0	0.0	0.0	0.0	0.0	0.0	0.0	0.0
0.0	0.0	0.0	0.0	0.0	0.0	0.0	0.0
0.0	0.0	0.0	0.0	0.0	0.0	0.0	0.0
0.0	0.0	0.0	0.0	0.0	0.0	0.0	0.0
0.0	0.0	0.0	0.0	0.0	0.0	0.0	0.0
0.0	0.0	0.0	0.0	0.0	0.0	0.0	0.0
0.0	0.0	0.0	0.0	0.0	0.0	0.0	0.0
0.0	0.0	0.0	0.0	0.0	0.0	0.0	0.0
0.0	0.0	0.0	0.0	0.0	0.0	0.0	0.0
0.0	0.0	0.0	0.0	0.0	0.0	0.0	0.0
0.0	0.0	0.0	0.0	0.0	0.0	0.0	0.0
0.0	0.0	0.0	0.0	0.0	0.0	0.0	0.0
0.0	0.0	0.0	0.0	0.0	0.0	0.0	0.0
0.0	0.0	0.0	0.0	0.0	0.0	0.0	0.0
0.0	0.0	0.0	0.0	0.0	0.0	0.0	0.0
0.0	0.0	0.0	0.0	0.0	0.0	0.0	3.0
0.0	0.0	0.0	0.0	0.0	0.0	0.0	0.0
0.0	0.0	0.0	0.0	0.0	0.0	0.0	0.0
0.0	0.0	0.0	0.0	0.0	0.0	0.0	0.0
0.0	0.0	0.0	0.0	0.0	0.0	0.0	0.0
0.0	0.0	0.0	0.0	0.0	0.0	0.0	0.0
0.0	0.0	0.0	0.0	0.0	0.0	0.0	0.0
0.0	0.0	0.0	0.0	0.0	0.0	0.0	0.0
0.0	0.0	0.0	0.0	0.0	0.0	0.0	0.0
0.0	0.0	0.0	0.0	0.0	0.0	0.0	0.0
0.0	0.0	0.0	0.0	0.0	0.0	0.0	0.0
0.0	0.0	0.0	0.0	0.0	0.0	0.0	0.0
0.0	0.0	0.0	0.0	0.0	0.0	0.0	0.0
0.0	0.0	0.0	0.0	0.0	0.0	0.0	0.0

0.0	0.0	0.0	0.0	0.0	0.0	0.0	0.0
0.0	0.0	0.0	0.0	0.0	0.0	0.0	0.0
0.0	0.0	0.0	0.0	0.0	0.0	0.0	0.0
0.0	0.0	0.0	0.0	0.0	0.0	0.0	0.0
0.0	0.0	0.0	0.0	0.0	0.0	0.0	0.0
0.0	0.0	0.0	0.0	0.0	0.0	0.0	0.0
0.0	0.0	0.0	0.0	0.0	0.0	0.0	0.0
0.0	1.0	0.0	0.0	0.0	0.0	0.0	0.0
0.0	0.0	0.0	0.0	0.0	0.0	0.0	0.0
0.0	0.0	0.0	0.0	0.0	0.0	0.0	0.0
0.0	0.0	0.0	0.0	0.0	0.0	0.0	0.0
0.0	0.0	0.0	0.0	0.0	0.0	0.0	0.0
0.0	0.0	0.0	0.0	0.0	0.0	0.0	0.0
0.0	0.0	0.0	0.0	0.0	0.0	0.0	0.0
0.0	0.0	0.0	0.0	0.0	0.0	0.0	0.0
0.0	0.0	0.0	0.0	0.0	0.0	0.0	0.0
0.0	0.0	0.0	0.0	0.0	0.0	0.0	0.0
0.0	0.0	0.0	0.0	0.0	0.0	0.0	0.0
0.0	0.0	0.0	0.0	0.0	0.0	0.0	0.0
0.0	0.0	0.0	0.0	0.0	0.0	0.0	0.0
0.0	0.0	0.0	0.0	0.0	0.0	0.0	0.0
0.0	0.0	0.0	0.0	0.0	0.0	0.0	0.0
0.0	0.0	0.0	0.0	0.0	0.0	0.0	0.0
0.0	0.0	0.0	0.0	0.0	0.0	0.0	0.0
17.0	0.0	0.0	0.0	0.0	0.0	0.0	0.0
17.0	0.0	0.0	0.0	0.0	0.0	0.0	0.0
12.0	0.0	0.0	0.0	0.0	0.0	0.0	0.0
8.0	0.0	0.0	0.0	0.0	0.0	0.0	0.0
7.0	0.0	0.0	0.0	0.0	0.0	0.0	0.0
4.0	0.0	0.0	0.0	0.0	0.0	0.0	0.0
1.0	0.0	0.0	0.0	0.0	0.0	0.0	0.0
5.0	0.0	0.0	0.0	0.0	0.0	0.0	0.0
3.0	0.0	0.0	0.0	0.0	0.0	0.0	0.0
3.0	0.0	0.0	0.0	0.0	0.0	0.0	0.0
4.0	0.0	0.0	0.0	0.0	0.0	0.0	0.0
1.0	0.0	0.0	2.0	0.0	0.0	0.0	0.0
2.0	0.0	0.0	0.0	0.0	0.0	0.0	0.0
2.0	0.0	0.0	0.0	0.0	0.0	0.0	0.0
2.0	0.0	0.0	0.0	0.0	0.0	0.0	0.0
2.0	0.0	0.0	0.0	0.0	0.0	0.0	0.0
1.0	0.0	0.0	0.0	0.0	0.0	0.0	0.0
0.0	0.0	0.0	0.0	0.0	0.0	0.0	0.0
2.0	0.0	0.0	0.0	0.0	0.0	0.0	0.0
2.0	0.0	0.0	0.0	0.0	0.0	0.0	0.0
0.0	84.0	0.0	0.0	0.0	0.0	0.0	0.0
0.0	47.0	0.0	0.0	0.0	0.0	0.0	0.0
0.0	27.0	0.0	0.0	0.0	0.0	0.0	0.0

0.0	27.0	0.0	0.0	0.0	0.0	0.0	0.0
0.0	10.0	0.0	0.0	0.0	0.0	0.0	0.0
0.0	8.0	0.0	0.0	0.0	0.0	0.0	0.0
0.0	3.0	0.0	0.0	0.0	0.0	0.0	0.0
0.0	6.0	0.0	0.0	0.0	0.0	0.0	0.0
0.0	4.0	0.0	0.0	0.0	0.0	0.0	0.0
0.0	5.0	0.0	0.0	0.0	0.0	0.0	0.0
0.0	2.0	0.0	0.0	0.0	0.0	0.0	0.0
0.0	3.0	0.0	0.0	0.0	0.0	0.0	0.0
0.0	2.0	0.0	0.0	0.0	0.0	0.0	0.0
0.0	1.0	0.0	0.0	0.0	0.0	0.0	0.0
0.0	2.0	0.0	0.0	0.0	0.0	0.0	0.0
0.0	1.0	0.0	0.0	0.0	0.0	0.0	0.0
0.0	2.0	0.0	0.0	0.0	0.0	0.0	0.0
0.0	1.0	0.0	0.0	0.0	0.0	0.0	0.0
0.0	1.0	0.0	0.0	0.0	0.0	0.0	0.0
0.0	2.0	0.0	0.0	0.0	0.0	0.0	0.0
0.0	1.0	0.0	0.0	0.0	0.0	0.0	0.0
0.0	1.0	0.0	0.0	0.0	0.0	0.0	0.0
0.0	1.0	0.0	0.0	0.0	0.0	0.0	0.0
0.0	1.0	0.0	0.0	0.0	0.0	0.0	0.0
0.0	0.0	0.0	0.0	0.0	0.0	0.0	0.0
0.0	1.0	0.0	0.0	0.0	0.0	0.0	0.0
0.0	1.0	0.0	0.0	0.0	0.0	0.0	0.0
0.0	1.0	0.0	0.0	0.0	0.0	0.0	0.0
0.0	1.0	0.0	0.0	0.0	0.0	0.0	0.0
0.0	1.0	0.0	0.0	0.0	0.0	0.0	0.0
0.0	1.0	0.0	0.0	0.0	0.0	0.0	0.0
0.0	1.0	0.0	0.0	0.0	0.0	0.0	0.0
0.0	1.0	0.0	0.0	0.0	0.0	0.0	0.0
0.0	1.0	0.0	0.0	0.0	0.0	0.0	0.0
0.0	0.0	0.0	0.0	0.0	0.0	0.0	0.0
0.0	1.0	0.0	0.0	0.0	0.0	0.0	0.0
0.0	1.0	0.0	0.0	0.0	0.0	0.0	0.0
0.0	1.0	0.0	0.0	0.0	0.0	0.0	0.0
0.0	1.0	0.0	0.0	0.0	0.0	0.0	0.0
0.0	1.0	0.0	0.0	0.0	0.0	0.0	0.0
0.0	1.0	0.0	0.0	0.0	0.0	0.0	0.0
0.0	1.0	0.0	0.0	0.0	0.0	0.0	0.0
0.0	0.0	32.0	0.0	0.0	0.0	0.0	0.0
0.0	0.0	11.0	0.0	0.0	0.0	0.0	0.0
0.0	0.0	4.0	0.0	0.0	0.0	0.0	0.0
0.0	0.0	1.0	0.0	0.0	0.0	0.0	0.0
0.0	0.0	1.0	0.0	0.0	0.0	0.0	0.0
0.0	0.0	1.0	0.0	0.0	0.0	0.0	0.0
0.0	0.0	1.0	0.0	0.0	0.0	0.0	0.0
0.0	0.0	1.0	0.0	0.0	0.0	0.0	0.0

0.0	0.0	1.0	0.0	0.0	0.0	0.0	0.0
0.0	0.0	1.0	0.0	0.0	0.0	0.0	0.0
0.0	0.0	1.0	0.0	0.0	0.0	0.0	0.0
0.0	0.0	1.0	0.0	0.0	0.0	0.0	0.0
0.0	0.0	1.0	0.0	0.0	0.0	0.0	0.0
0.0	0.0	0.0	0.0	0.0	0.0	0.0	0.0
0.0	0.0	0.0	0.0	0.0	0.0	0.0	0.0
0.0	0.0	1.0	0.0	0.0	0.0	0.0	0.0
0.0	0.0	0.0	150.0	0.0	0.0	0.0	1.0
0.0	0.0	0.0	43.0	0.0	0.0	0.0	0.0
0.0	0.0	0.0	27.0	0.0	0.0	0.0	0.0
0.0	0.0	0.0	15.0	0.0	0.0	0.0	0.0
0.0	0.0	0.0	11.0	0.0	0.0	0.0	0.0
0.0	0.0	0.0	12.0	0.0	0.0	0.0	0.0
0.0	0.0	0.0	8.0	0.0	0.0	0.0	0.0
0.0	0.0	0.0	6.0	0.0	0.0	0.0	0.0
0.0	0.0	0.0	7.0	0.0	0.0	0.0	0.0
0.0	0.0	0.0	4.0	0.0	0.0	0.0	0.0
0.0	0.0	0.0	0.0	0.0	0.0	0.0	0.0
0.0	0.0	0.0	2.0	0.0	0.0	0.0	0.0
0.0	0.0	0.0	3.0	0.0	0.0	0.0	0.0
0.0	0.0	0.0	1.0	0.0	0.0	0.0	0.0
0.0	0.0	0.0	2.0	0.0	0.0	0.0	0.0
0.0	0.0	0.0	3.0	0.0	0.0	0.0	0.0
0.0	0.0	0.0	1.0	0.0	0.0	0.0	0.0
0.0	0.0	0.0	1.0	0.0	0.0	0.0	0.0
0.0	0.0	0.0	1.0	0.0	0.0	0.0	0.0
0.0	0.0	0.0	0.0	0.0	0.0	0.0	0.0
0.0	0.0	0.0	2.0	0.0	0.0	0.0	0.0
0.0	0.0	0.0	0.0	0.0	0.0	0.0	0.0
0.0	0.0	0.0	0.0	0.0	0.0	0.0	0.0
0.0	0.0	0.0	1.0	0.0	0.0	0.0	0.0
0.0	0.0	0.0	1.0	0.0	0.0	0.0	0.0
0.0	0.0	0.0	0.0	0.0	0.0	0.0	0.0
0.0	0.0	0.0	1.0	0.0	0.0	0.0	0.0
0.0	0.0	0.0	1.0	0.0	0.0	0.0	0.0
0.0	0.0	0.0	1.0	0.0	0.0	0.0	0.0
0.0	0.0	0.0	1.0	0.0	0.0	0.0	0.0
0.0	0.0	0.0	0.0	0.0	0.0	0.0	0.0
0.0	0.0	0.0	0.0	2.0	0.0	0.0	0.0
0.0	0.0	0.0	0.0	2.0	0.0	0.0	0.0
0.0	0.0	0.0	0.0	2.0	0.0	0.0	0.0
0.0	0.0	0.0	0.0	2.0	0.0	0.0	0.0
0.0	0.0	0.0	0.0	1.0	0.0	0.0	0.0
0.0	0.0	0.0	0.0	2.0	0.0	0.0	0.0
0.0	0.0	0.0	0.0	1.0	0.0	0.0	0.0
0.0	0.0	0.0	0.0	0.0	0.0	0.0	0.0

0.0	0.0	0.0	0.0	0.0	0.0	0.0	0.0
0.0	0.0	0.0	0.0	0.0	0.0	0.0	0.0
0.0	0.0	0.0	0.0	0.0	0.0	0.0	0.0
0.0	0.0	0.0	0.0	0.0	0.0	0.0	0.0
0.0	0.0	0.0	0.0	0.0	0.0	0.0	0.0
0.0	0.0	0.0	0.0	0.0	479.0	0.0	0.0
0.0	0.0	0.0	0.0	0.0	46.0	0.0	0.0
0.0	0.0	0.0	0.0	0.0	31.0	0.0	0.0
0.0	0.0	0.0	0.0	0.0	14.0	0.0	0.0
0.0	0.0	0.0	0.0	0.0	8.0	0.0	0.0
0.0	0.0	0.0	0.0	0.0	2.0	0.0	0.0
0.0	0.0	0.0	0.0	0.0	3.0	0.0	0.0
0.0	0.0	0.0	0.0	0.0	0.0	0.0	0.0
0.0	0.0	0.0	0.0	0.0	1.0	0.0	0.0
0.0	0.0	0.0	0.0	0.0	1.0	0.0	0.0
0.0	0.0	0.0	0.0	0.0	0.0	0.0	0.0
0.0	0.0	0.0	0.0	0.0	2.0	0.0	0.0
0.0	0.0	0.0	0.0	0.0	2.0	0.0	0.0
0.0	0.0	0.0	0.0	0.0	1.0	0.0	0.0
0.0	0.0	0.0	0.0	0.0	1.0	0.0	0.0
0.0	0.0	0.0	0.0	0.0	1.0	0.0	0.0
0.0	0.0	0.0	0.0	0.0	1.0	0.0	0.0
0.0	0.0	0.0	0.0	0.0	1.0	0.0	0.0
0.0	0.0	0.0	0.0	0.0	1.0	0.0	0.0
0.0	0.0	0.0	0.0	0.0	1.0	0.0	0.0
0.0	0.0	0.0	0.0	0.0	1.0	0.0	0.0
0.0	0.0	0.0	0.0	0.0	1.0	0.0	0.0
0.0	0.0	0.0	0.0	0.0	1.0	0.0	0.0
0.0	0.0	0.0	0.0	0.0	1.0	0.0	0.0
0.0	0.0	0.0	0.0	0.0	0.0	0.0	0.0
0.0	0.0	0.0	0.0	0.0	1.0	0.0	0.0
0.0	0.0	0.0	0.0	0.0	0.0	177.0	0.0
0.0	0.0	0.0	0.0	0.0	0.0	58.0	0.0
0.0	0.0	0.0	0.0	0.0	0.0	14.0	0.0
0.0	0.0	0.0	0.0	0.0	0.0	7.0	0.0
0.0	0.0	0.0	0.0	0.0	0.0	3.0	0.0
0.0	0.0	0.0	0.0	0.0	0.0	2.0	0.0
0.0	0.0	0.0	0.0	0.0	0.0	2.0	0.0
0.0	0.0	0.0	0.0	0.0	0.0	1.0	0.0
0.0	0.0	0.0	0.0	0.0	0.0	3.0	0.0
0.0	0.0	0.0	0.0	0.0	0.0	3.0	0.0
0.0	0.0	0.0	0.0	0.0	0.0	1.0	0.0
0.0	0.0	0.0	0.0	0.0	0.0	2.0	0.0
0.0	0.0	0.0	0.0	0.0	0.0	1.0	0.0
0.0	0.0	0.0	0.0	0.0	0.0	1.0	0.0
0.0	0.0	0.0	0.0	0.0	0.0	1.0	0.0
0.0	0.0	0.0	0.0	0.0	0.0	2.0	0.0

0.0	0.0	0.0	0.0	0.0	0.0	2.0	0.0
0.0	0.0	0.0	0.0	0.0	0.0	2.0	0.0
0.0	0.0	0.0	0.0	0.0	0.0	2.0	0.0
0.0	0.0	0.0	0.0	0.0	0.0	1.0	0.0
0.0	0.0	0.0	0.0	0.0	0.0	1.0	0.0
0.0	0.0	0.0	0.0	0.0	0.0	1.0	0.0
0.0	0.0	0.0	0.0	0.0	0.0	2.0	0.0
0.0	0.0	0.0	0.0	0.0	0.0	1.0	0.0
0.0	0.0	0.0	0.0	0.0	0.0	2.0	0.0
0.0	0.0	0.0	0.0	0.0	0.0	1.0	0.0
0.0	0.0	0.0	0.0	0.0	0.0	1.0	0.0
0.0	0.0	0.0	0.0	0.0	0.0	1.0	0.0
0.0	0.0	0.0	0.0	0.0	0.0	1.0	0.0
0.0	0.0	0.0	0.0	0.0	0.0	1.0	0.0
0.0	0.0	0.0	0.0	0.0	0.0	1.0	0.0
0.0	0.0	0.0	0.0	0.0	0.0	2.0	0.0
0.0	0.0	0.0	0.0	0.0	0.0	1.0	0.0
0.0	0.0	0.0	0.0	0.0	0.0	2.0	0.0
0.0	0.0	0.0	0.0	0.0	0.0	1.0	0.0
0.0	0.0	0.0	0.0	0.0	0.0	1.0	0.0
0.0	0.0	0.0	0.0	0.0	0.0	1.0	0.0
0.0	0.0	0.0	0.0	0.0	0.0	1.0	0.0
0.0	0.0	0.0	0.0	0.0	0.0	1.0	0.0
0.0	0.0	0.0	0.0	0.0	0.0	1.0	0.0
0.0	0.0	0.0	0.0	0.0	0.0	1.0	0.0
0.0	0.0	0.0	0.0	0.0	0.0	1.0	0.0
0.0	0.0	0.0	0.0	0.0	0.0	1.0	0.0
0.0	0.0	0.0	0.0	0.0	0.0	1.0	0.0
0.0	0.0	0.0	0.0	0.0	0.0	1.0	0.0
0.0	0.0	0.0	0.0	0.0	0.0	1.0	0.0
0.0	0.0	0.0	0.0	0.0	0.0	1.0	0.0
0.0	0.0	0.0	0.0	0.0	0.0	1.0	0.0
0.0	0.0	0.0	0.0	0.0	0.0	1.0	0.0
0.0	0.0	0.0	0.0	0.0	0.0	1.0	0.0
0.0	0.0	0.0	0.0	0.0	0.0	1.0	0.0
0.0	0.0	0.0	0.0	0.0	0.0	0.0	121.0
0.0	0.0	0.0	0.0	0.0	0.0	0.0	129.0
0.0	0.0	0.0	0.0	0.0	0.0	0.0	16.0
0.0	0.0	0.0	0.0	0.0	0.0	0.0	8.0
0.0	0.0	0.0	0.0	0.0	0.0	0.0	14.0
0.0	0.0	0.0	0.0	0.0	0.0	0.0	5.0
0.0	0.0	0.0	0.0	0.0	0.0	0.0	2.0
0.0	0.0	0.0	0.0	0.0	0.0	0.0	8.0
0.0	0.0	0.0	0.0	0.0	0.0	0.0	3.0
0.0	0.0	0.0	0.0	0.0	0.0	0.0	2.0
0.0	0.0	0.0	0.0	0.0	0.0	0.0	1.0
0.0	0.0	0.0	0.0	0.0	0.0	0.0	2.0

0.0	0.0	0.0	0.0	0.0	0.0	0.0	1.0
0.0	0.0	0.0	0.0	0.0	0.0	0.0	1.0
0.0	0.0	0.0	0.0	0.0	0.0	0.0	5.0
0.0	0.0	0.0	0.0	0.0	0.0	0.0	2.0
0.0	0.0	0.0	0.0	0.0	0.0	0.0	2.0
0.0	0.0	0.0	0.0	0.0	0.0	0.0	3.0
0.0	0.0	0.0	0.0	0.0	0.0	0.0	1.0
0.0	0.0	0.0	0.0	0.0	0.0	0.0	1.0
0.0	0.0	0.0	0.0	0.0	0.0	0.0	2.0
0.0	0.0	0.0	0.0	0.0	0.0	0.0	1.0
0.0	0.0	0.0	0.0	0.0	0.0	0.0	1.0
0.0	0.0	0.0	0.0	0.0	0.0	0.0	2.0
0.0	0.0	0.0	0.0	0.0	0.0	0.0	1.0
0.0	0.0	0.0	0.0	0.0	0.0	0.0	1.0
0.0	0.0	0.0	0.0	0.0	0.0	0.0	2.0
0.0	0.0	0.0	0.0	0.0	0.0	0.0	2.0
0.0	0.0	0.0	0.0	0.0	0.0	0.0	1.0
0.0	0.0	0.0	0.0	0.0	0.0	0.0	1.0
0.0	0.0	0.0	0.0	0.0	0.0	0.0	1.0
0.0	0.0	0.0	0.0	0.0	0.0	0.0	0.0
0.0	0.0	0.0	0.0	0.0	0.0	0.0	1.0
0.0	0.0	0.0	0.0	0.0	0.0	0.0	1.0
0.0	0.0	0.0	0.0	0.0	0.0	0.0	1.0
0.0	0.0	0.0	0.0	0.0	0.0	0.0	1.0
0.0	0.0	0.0	0.0	0.0	0.0	0.0	1.0
0.0	0.0	0.0	0.0	0.0	0.0	0.0	1.0
0.0	0.0	0.0	0.0	0.0	0.0	0.0	0.0
0.0	0.0	0.0	0.0	0.0	0.0	0.0	0.0
0.0	0.0	0.0	0.0	0.0	0.0	0.0	0.0
0.0	0.0	0.0	0.0	0.0	0.0	0.0	0.0
0.0	0.0	0.0	0.0	0.0	0.0	0.0	0.0
0.0	0.0	0.0	0.0	0.0	0.0	0.0	0.0
0.0	0.0	0.0	0.0	0.0	0.0	0.0	0.0
0.0	0.0	0.0	0.0	0.0	0.0	0.0	0.0
0.0	0.0	0.0	0.0	0.0	0.0	0.0	0.0
0.0	0.0	0.0	0.0	0.0	0.0	0.0	0.0
0.0	0.0	0.0	0.0	0.0	0.0	0.0	0.0
0.0	0.0	0.0	0.0	0.0	0.0	0.0	0.0
0.0	0.0	0.0	0.0	0.0	0.0	0.0	0.0
0.0	0.0	0.0	0.0	0.0	0.0	0.0	0.0
0.0	0.0	0.0	0.0	0.0	0.0	0.0	0.0
0.0	0.0	0.0	0.0	0.0	0.0	0.0	0.0
0.0	0.0	0.0	0.0	0.0	0.0	0.0	0.0
0.0	0.0	0.0	0.0	0.0	0.0	0.0	0.0
0.0	0.0	0.0	0.0	0.0	0.0	0.0	0.0
0.0	0.0	0.0	0.0	0.0	0.0	0.0	0.0
0.0	0.0	0.0	0.0	0.0	0.0	0.0	0.0

0.0	0.0	0.0	0.0	0.0	0.0	0.0	0.0
0.0	0.0	0.0	0.0	0.0	0.0	0.0	0.0
0.0	0.0	0.0	0.0	0.0	0.0	0.0	0.0
0.0	0.0	0.0	0.0	0.0	0.0	0.0	0.0
0.0	0.0	0.0	0.0	0.0	0.0	0.0	0.0
0.0	0.0	0.0	0.0	0.0	0.0	0.0	0.0
0.0	0.0	0.0	0.0	0.0	0.0	0.0	0.0
0.0	0.0	0.0	0.0	0.0	0.0	0.0	0.0
0.0	0.0	0.0	0.0	0.0	0.0	0.0	0.0
0.0	0.0	0.0	0.0	0.0	0.0	0.0	0.0
0.0	0.0	0.0	0.0	0.0	0.0	0.0	0.0
0.0	0.0	0.0	0.0	0.0	0.0	0.0	0.0
0.0	0.0	0.0	0.0	0.0	0.0	0.0	0.0
0.0	0.0	0.0	0.0	0.0	0.0	0.0	0.0
0.0	0.0	0.0	0.0	0.0	0.0	0.0	0.0
0.0	0.0	0.0	0.0	0.0	0.0	0.0	0.0
0.0	0.0	0.0	0.0	0.0	0.0	0.0	0.0
0.0	0.0	0.0	0.0	0.0	0.0	0.0	0.0
0.0	0.0	0.0	0.0	0.0	0.0	0.0	0.0
0.0	0.0	0.0	0.0	0.0	0.0	0.0	0.0
0.0	0.0	0.0	0.0	0.0	0.0	0.0	0.0
0.0	0.0	0.0	0.0	0.0	0.0	0.0	0.0
0.0	0.0	0.0	0.0	0.0	0.0	0.0	0.0
0.0	0.0	0.0	0.0	0.0	0.0	0.0	0.0
0.0	0.0	0.0	0.0	0.0	0.0	0.0	0.0
0.0	0.0	0.0	0.0	0.0	0.0	0.0	0.0
0.0	0.0	0.0	0.0	0.0	0.0	0.0	0.0
0.0	0.0	0.0	0.0	0.0	0.0	0.0	0.0
0.0	0.0	0.0	0.0	0.0	0.0	0.0	0.0
0.0	0.0	0.0	0.0	0.0	0.0	0.0	0.0
0.0	0.0	0.0	0.0	0.0	0.0	0.0	0.0
0.0	0.0	0.0	0.0	0.0	0.0	0.0	0.0
0.0	0.0	0.0	0.0	0.0	0.0	0.0	0.0
0.0	0.0	0.0	0.0	0.0	0.0	0.0	0.0
0.0	0.0	0.0	0.0	0.0	0.0	0.0	0.0
0.0	0.0	0.0	0.0	0.0	0.0	0.0	0.0
0.0	0.0	0.0	0.0	0.0	0.0	0.0	0.0
0.0	0.0	0.0	0.0	0.0	0.0	0.0	0.0
0.0	0.0	0.0	0.0	0.0	0.0	0.0	0.0
0.0	0.0	0.0	0.0	0.0	0.0	0.0	0.0
0.0	0.0	0.0	0.0	0.0	0.0	0.0	0.0
0.0	0.0	0.0	0.0	0.0	0.0	0.0	0.0
0.0	0.0	0.0	0.0	0.0	0.0	0.0	0.0
0.0	0.0	0.0	0.0	0.0	0.0	0.0	0.0
0.0	0.0	0.0	0.0	0.0	0.0	0.0	0.0
0.0	0.0	0.0	0.0	0.0	0.0	0.0	0.0
0.0	0.0	0.0	0.0	0.0	0.0	0.0	0.0
0.0	0.0	0.0	0.0	0.0	0.0	0.0	0.0

0.0	0.0	0.0	0.0	0.0	0.0	0.0	0.0
0.0	0.0	0.0	0.0	0.0	0.0	0.0	0.0
0.0	0.0	0.0	0.0	0.0	0.0	0.0	0.0
0.0	0.0	0.0	0.0	0.0	0.0	0.0	0.0
0.0	0.0	0.0	0.0	0.0	0.0	0.0	0.0
0.0	0.0	0.0	0.0	0.0	0.0	0.0	0.0
0.0	0.0	0.0	0.0	0.0	0.0	0.0	0.0
0.0	0.0	0.0	0.0	0.0	0.0	0.0	0.0
0.0	0.0	0.0	0.0	0.0	0.0	0.0	0.0
0.0	0.0	0.0	0.0	0.0	0.0	0.0	0.0
0.0	0.0	0.0	0.0	0.0	0.0	0.0	0.0
0.0	0.0	0.0	0.0	0.0	0.0	0.0	0.0
0.0	0.0	0.0	0.0	0.0	0.0	0.0	0.0
0.0	0.0	0.0	0.0	0.0	0.0	0.0	0.0
0.0	0.0	0.0	0.0	0.0	0.0	0.0	0.0
0.0	0.0	0.0	0.0	0.0	0.0	0.0	0.0
0.0	0.0	0.0	0.0	0.0	0.0	0.0	0.0
0.0	0.0	0.0	0.0	0.0	0.0	0.0	0.0
0.0	0.0	0.0	0.0	0.0	0.0	0.0	0.0
0.0	0.0	0.0	0.0	0.0	0.0	0.0	0.0
0.0	0.0	0.0	0.0	0.0	0.0	0.0	0.0
0.0	0.0	0.0	0.0	0.0	0.0	0.0	0.0
0.0	0.0	0.0	0.0	0.0	0.0	0.0	0.0
0.0	0.0	0.0	0.0	0.0	0.0	0.0	0.0
0.0	0.0	0.0	0.0	0.0	0.0	0.0	0.0
0.0	0.0	0.0	0.0	0.0	0.0	0.0	0.0
0.0	0.0	0.0	0.0	0.0	0.0	0.0	0.0
0.0	0.0	0.0	0.0	0.0	0.0	0.0	0.0
0.0	0.0	0.0	0.0	0.0	0.0	0.0	0.0
0.0	0.0	0.0	0.0	0.0	0.0	0.0	0.0
0.0	0.0	0.0	0.0	0.0	0.0	0.0	0.0
0.0	0.0	0.0	0.0	0.0	0.0	0.0	0.0
0.0	0.0	0.0	0.0	0.0	0.0	0.0	0.0
0.0	0.0	0.0	0.0	0.0	0.0	0.0	0.0
0.0	0.0	0.0	0.0	0.0	0.0	0.0	0.0
0.0	0.0	0.0	0.0	0.0	0.0	0.0	0.0
0.0	0.0	0.0	0.0	0.0	0.0	0.0	0.0
0.0	0.0	0.0	0.0	0.0	0.0	0.0	0.0
0.0	0.0	0.0	0.0	0.0	0.0	0.0	0.0
0.0	0.0	0.0	0.0	0.0	0.0	0.0	0.0
0.0	0.0	0.0	0.0	0.0	0.0	0.0	0.0
0.0	0.0	0.0	0.0	0.0	0.0	0.0	0.0
0.0	0.0	0.0	0.0	0.0	0.0	0.0	0.0
0.0	0.0	0.0	0.0	0.0	0.0	0.0	0.0
0.0	0.0	0.0	0.0	0.0	0.0	0.0	0.0
0.0	0.0	0.0	0.0	0.0	0.0	0.0	0.0
0.0	0.0	0.0	0.0	0.0	0.0	0.0	0.0

0.0	0.0	0.0	0.0	0.0	0.0	0.0	0.0
0.0	0.0	0.0	0.0	0.0	0.0	0.0	0.0
0.0	0.0	0.0	0.0	0.0	0.0	0.0	0.0
0.0	0.0	0.0	0.0	0.0	0.0	0.0	0.0
0.0	0.0	0.0	0.0	0.0	0.0	0.0	0.0
0.0	0.0	0.0	0.0	0.0	0.0	0.0	0.0
0.0	0.0	0.0	0.0	0.0	0.0	0.0	0.0
0.0	0.0	0.0	0.0	0.0	0.0	0.0	0.0
0.0	0.0	0.0	0.0	0.0	0.0	0.0	0.0
0.0	0.0	0.0	0.0	0.0	0.0	0.0	0.0
0.0	0.0	0.0	0.0	0.0	0.0	0.0	0.0
0.0	0.0	0.0	0.0	0.0	0.0	0.0	0.0
0.0	0.0	0.0	0.0	0.0	0.0	0.0	0.0
0.0	0.0	0.0	0.0	0.0	0.0	0.0	0.0
0.0	0.0	0.0	0.0	0.0	0.0	0.0	0.0
0.0	0.0	0.0	0.0	0.0	0.0	0.0	0.0
0.0	0.0	0.0	0.0	0.0	0.0	0.0	0.0
0.0	0.0	0.0	0.0	0.0	0.0	0.0	0.0
0.0	0.0	0.0	0.0	0.0	0.0	0.0	0.0
0.0	0.0	0.0	0.0	0.0	0.0	0.0	0.0
0.0	0.0	0.0	0.0	0.0	0.0	0.0	0.0
0.0	0.0	0.0	0.0	0.0	0.0	0.0	0.0
0.0	0.0	0.0	0.0	0.0	0.0	0.0	0.0
0.0	0.0	0.0	0.0	0.0	0.0	0.0	0.0
0.0	0.0	0.0	0.0	0.0	0.0	0.0	0.0
0.0	0.0	0.0	0.0	0.0	0.0	0.0	0.0
0.0	0.0	0.0	0.0	0.0	0.0	0.0	0.0
0.0	0.0	0.0	0.0	0.0	0.0	0.0	0.0
0.0	0.0	0.0	0.0	0.0	0.0	0.0	0.0
0.0	0.0	0.0	0.0	0.0	0.0	0.0	0.0
0.0	0.0	0.0	0.0	0.0	0.0	0.0	0.0
0.0	0.0	0.0	0.0	0.0	0.0	0.0	0.0
0.0	0.0	0.0	0.0	0.0	0.0	0.0	0.0
0.0	0.0	0.0	0.0	0.0	0.0	0.0	0.0
0.0	0.0	0.0	0.0	0.0	0.0	0.0	0.0
0.0	0.0	0.0	0.0	0.0	0.0	0.0	0.0
0.0	0.0	0.0	0.0	0.0	0.0	0.0	0.0
0.0	0.0	0.0	0.0	0.0	0.0	0.0	0.0
0.0	0.0	0.0	0.0	0.0	0.0	0.0	0.0
0.0	0.0	0.0	0.0	0.0	0.0	0.0	0.0
0.0	0.0	0.0	0.0	0.0	0.0	0.0	0.0
0.0	0.0	0.0	0.0	0.0	0.0	0.0	0.0
0.0	0.0	0.0	0.0	0.0	0.0	0.0	0.0
0.0	0.0	0.0	0.0	0.0	0.0	0.0	0.0
0.0	0.0	0.0	0.0	0.0	0.0	0.0	0.0
0.0	0.0	0.0	0.0	0.0	0.0	0.0	0.0
0.0	0.0	0.0	0.0	0.0	0.0	0.0	0.0

0.0	0.0	0.0	0.0	0.0	0.0	0.0	0.0
0.0	0.0	0.0	0.0	0.0	0.0	0.0	0.0
0.0	0.0	0.0	0.0	0.0	0.0	0.0	0.0
0.0	0.0	0.0	0.0	0.0	0.0	0.0	0.0
0.0	0.0	0.0	0.0	0.0	0.0	0.0	0.0
0.0	0.0	0.0	0.0	0.0	0.0	0.0	0.0
0.0	0.0	0.0	0.0	0.0	0.0	0.0	0.0
0.0	0.0	0.0	0.0	0.0	0.0	0.0	0.0
0.0	0.0	0.0	0.0	0.0	0.0	0.0	0.0
0.0	0.0	0.0	0.0	0.0	0.0	0.0	0.0
0.0	0.0	0.0	0.0	0.0	0.0	0.0	0.0
0.0	0.0	0.0	0.0	0.0	0.0	0.0	0.0
0.0	0.0	0.0	0.0	0.0	0.0	0.0	0.0
0.0	0.0	0.0	0.0	0.0	0.0	0.0	0.0
0.0	0.0	0.0	0.0	0.0	0.0	0.0	0.0
0.0	0.0	0.0	0.0	0.0	0.0	0.0	0.0
0.0	0.0	0.0	0.0	0.0	0.0	0.0	0.0
0.0	0.0	0.0	0.0	0.0	0.0	0.0	0.0
0.0	0.0	0.0	0.0	0.0	0.0	0.0	0.0
0.0	0.0	0.0	0.0	0.0	0.0	0.0	0.0
0.0	0.0	0.0	0.0	0.0	0.0	0.0	0.0
0.0	0.0	0.0	0.0	0.0	0.0	0.0	0.0
0.0	0.0	0.0	0.0	0.0	0.0	0.0	0.0
0.0	0.0	0.0	0.0	0.0	0.0	0.0	0.0
0.0	0.0	0.0	0.0	0.0	0.0	0.0	0.0
0.0	0.0	0.0	0.0	0.0	0.0	0.0	0.0
0.0	0.0	0.0	0.0	0.0	0.0	0.0	0.0
0.0	0.0	0.0	0.0	0.0	0.0	0.0	0.0
0.0	0.0	0.0	0.0	0.0	0.0	0.0	0.0
0.0	0.0	0.0	0.0	0.0	0.0	0.0	0.0
0.0	0.0	0.0	0.0	0.0	0.0	0.0	0.0
0.0	0.0	0.0	0.0	0.0	0.0	0.0	0.0
0.0	0.0	0.0	0.0	0.0	0.0	0.0	0.0
0.0	0.0	0.0	0.0	0.0	0.0	0.0	0.0
0.0	0.0	0.0	0.0	0.0	0.0	0.0	0.0
0.0	0.0	0.0	0.0	0.0	0.0	0.0	0.0
0.0	0.0	0.0	0.0	0.0	0.0	0.0	0.0
0.0	0.0	0.0	0.0	0.0	0.0	0.0	0.0
0.0	0.0	0.0	0.0	0.0	0.0	0.0	0.0
0.0	0.0	0.0	0.0	0.0	0.0	0.0	0.0
0.0	0.0	0.0	0.0	0.0	0.0	0.0	0.0
0.0	0.0	0.0	0.0	0.0	0.0	0.0	0.0
0.0	0.0	0.0	0.0	0.0	0.0	0.0	0.0
0.0	0.0	0.0	0.0	0.0	0.0	0.0	0.0
0.0	0.0	0.0	0.0	0.0	0.0	0.0	0.0
0.0	0.0	0.0	0.0	0.0	0.0	0.0	0.0
0.0	0.0	0.0	0.0	0.0	0.0	0.0	0.0

0.0	0.0	0.0	0.0	0.0	0.0	0.0	0.0
0.0	0.0	0.0	0.0	0.0	0.0	0.0	0.0
0.0	0.0	0.0	0.0	0.0	0.0	0.0	0.0
0.0	0.0	0.0	0.0	0.0	0.0	0.0	0.0
0.0	0.0	0.0	0.0	0.0	0.0	0.0	0.0
0.0	0.0	0.0	0.0	0.0	0.0	0.0	0.0
0.0	0.0	0.0	0.0	0.0	0.0	0.0	0.0
0.0	0.0	0.0	0.0	0.0	0.0	0.0	0.0
0.0	0.0	0.0	0.0	0.0	0.0	0.0	0.0
0.0	0.0	0.0	0.0	0.0	0.0	0.0	0.0
0.0	0.0	0.0	0.0	0.0	0.0	0.0	0.0
0.0	0.0	0.0	0.0	0.0	0.0	0.0	0.0
0.0	0.0	0.0	0.0	0.0	0.0	0.0	0.0
0.0	0.0	0.0	0.0	0.0	0.0	0.0	0.0
0.0	0.0	0.0	0.0	0.0	0.0	0.0	0.0
0.0	0.0	0.0	0.0	0.0	0.0	0.0	0.0
0.0	0.0	0.0	0.0	0.0	0.0	0.0	0.0
0.0	0.0	0.0	0.0	0.0	0.0	0.0	0.0
0.0	0.0	0.0	0.0	0.0	0.0	0.0	0.0
0.0	0.0	0.0	0.0	0.0	0.0	0.0	0.0
0.0	0.0	0.0	0.0	0.0	0.0	0.0	0.0
0.0	0.0	0.0	0.0	0.0	0.0	0.0	0.0
0.0	0.0	0.0	0.0	0.0	0.0	0.0	0.0
0.0	0.0	0.0	0.0	0.0	0.0	0.0	0.0
0.0	0.0	0.0	0.0	0.0	0.0	0.0	0.0
0.0	0.0	0.0	0.0	0.0	0.0	0.0	0.0
0.0	0.0	0.0	0.0	0.0	0.0	0.0	0.0
0.0	0.0	0.0	0.0	0.0	0.0	0.0	0.0
0.0	0.0	0.0	0.0	0.0	0.0	0.0	0.0
0.0	0.0	0.0	0.0	0.0	0.0	0.0	0.0
0.0	0.0	0.0	0.0	0.0	0.0	0.0	0.0
0.0	0.0	0.0	0.0	0.0	0.0	0.0	0.0
0.0	0.0	0.0	0.0	0.0	0.0	0.0	0.0
0.0	0.0	0.0	0.0	0.0	0.0	0.0	0.0
0.0	0.0	0.0	0.0	0.0	0.0	0.0	0.0
0.0	0.0	0.0	0.0	0.0	0.0	0.0	0.0
0.0	0.0	0.0	0.0	0.0	0.0	0.0	0.0
0.0	0.0	0.0	0.0	0.0	0.0	0.0	0.0
0.0	0.0	0.0	0.0	0.0	0.0	0.0	0.0
0.0	0.0	0.0	0.0	0.0	0.0	0.0	0.0
0.0	0.0	0.0	0.0	0.0	0.0	0.0	0.0
0.0	0.0	0.0	0.0	0.0	0.0	0.0	0.0
0.0	0.0	0.0	0.0	0.0	0.0	0.0	0.0
0.0	0.0	0.0	0.0	0.0	0.0	0.0	0.0
0.0	0.0	0.0	0.0	0.0	0.0	0.0	0.0
0.0	0.0	0.0	0.0	0.0	0.0	0.0	0.0
0.0	0.0	0.0	0.0	0.0	0.0	0.0	0.0

0.0	0.0	0.0	0.0	0.0	0.0	0.0	0.0
0.0	0.0	0.0	0.0	0.0	0.0	0.0	0.0
0.0	0.0	0.0	0.0	0.0	0.0	0.0	0.0
0.0	0.0	0.0	0.0	0.0	0.0	0.0	0.0
0.0	0.0	0.0	0.0	0.0	0.0	0.0	0.0
0.0	0.0	0.0	0.0	0.0	0.0	0.0	0.0
0.0	0.0	0.0	0.0	0.0	0.0	0.0	0.0
0.0	0.0	0.0	0.0	0.0	0.0	0.0	0.0
0.0	0.0	0.0	0.0	0.0	0.0	0.0	0.0
0.0	0.0	0.0	0.0	0.0	0.0	0.0	0.0
0.0	0.0	0.0	0.0	0.0	0.0	0.0	0.0
0.0	0.0	0.0	0.0	0.0	0.0	0.0	0.0
0.0	0.0	0.0	0.0	0.0	0.0	0.0	0.0
0.0	0.0	0.0	0.0	0.0	0.0	0.0	0.0
0.0	0.0	0.0	0.0	0.0	0.0	0.0	0.0
0.0	0.0	0.0	0.0	0.0	0.0	0.0	0.0
0.0	0.0	0.0	0.0	0.0	0.0	0.0	0.0
0.0	0.0	0.0	0.0	0.0	0.0	0.0	0.0
0.0	0.0	0.0	0.0	0.0	0.0	0.0	0.0
0.0	0.0	0.0	0.0	0.0	0.0	0.0	0.0
0.0	0.0	0.0	0.0	0.0	0.0	0.0	0.0
0.0	0.0	0.0	0.0	0.0	0.0	0.0	0.0
0.0	0.0	0.0	0.0	0.0	0.0	0.0	0.0
0.0	0.0	0.0	0.0	0.0	0.0	0.0	0.0
0.0	0.0	0.0	0.0	0.0	0.0	0.0	0.0
0.0	0.0	0.0	0.0	0.0	0.0	0.0	0.0
0.0	0.0	0.0	0.0	0.0	0.0	0.0	0.0
0.0	0.0	0.0	0.0	0.0	0.0	0.0	0.0
0.0	0.0	0.0	0.0	0.0	0.0	0.0	0.0
0.0	0.0	0.0	0.0	0.0	0.0	0.0	0.0
0.0	0.0	0.0	0.0	0.0	0.0	0.0	0.0
0.0	0.0	0.0	0.0	0.0	0.0	0.0	0.0
0.0	0.0	0.0	0.0	0.0	0.0	0.0	0.0
0.0	0.0	0.0	0.0	0.0	0.0	0.0	0.0
0.0	0.0	0.0	0.0	0.0	0.0	0.0	0.0
0.0	0.0	0.0	0.0	0.0	0.0	0.0	0.0
0.0	0.0	0.0	0.0	0.0	0.0	0.0	0.0
0.0	0.0	0.0	0.0	0.0	0.0	0.0	0.0
0.0	0.0	0.0	0.0	0.0	0.0	0.0	0.0
0.0	0.0	0.0	0.0	0.0	0.0	0.0	0.0
0.0	0.0	0.0	0.0	0.0	0.0	0.0	0.0
0.0	0.0	0.0	0.0	0.0	0.0	0.0	0.0
0.0	0.0	0.0	0.0	0.0	0.0	0.0	0.0
0.0	0.0	0.0	0.0	0.0	0.0	0.0	0.0
0.0	0.0	0.0	0.0	0.0	0.0	0.0	0.0
0.0	0.0	0.0	0.0	0.0	0.0	0.0	0.0
0.0	0.0	0.0	0.0	0.0	0.0	0.0	0.0
0.0	0.0	0.0	0.0	0.0	0.0	0.0	0.0

0.0	0.0	0.0	0.0	0.0	0.0	0.0	0.0
0.0	0.0	0.0	0.0	0.0	0.0	0.0	0.0
0.0	0.0	0.0	0.0	0.0	0.0	0.0	0.0
0.0	0.0	0.0	0.0	0.0	0.0	0.0	0.0
0.0	0.0	0.0	0.0	0.0	0.0	0.0	0.0
0.0	0.0	0.0	0.0	0.0	0.0	0.0	0.0
0.0	0.0	0.0	0.0	0.0	0.0	0.0	0.0
0.0	0.0	0.0	0.0	0.0	0.0	0.0	0.0
0.0	0.0	0.0	0.0	0.0	0.0	0.0	0.0
0.0	0.0	0.0	0.0	0.0	0.0	0.0	0.0
0.0	0.0	0.0	0.0	0.0	0.0	0.0	0.0
0.0	0.0	0.0	0.0	0.0	0.0	0.0	0.0
0.0	0.0	0.0	0.0	0.0	0.0	0.0	0.0
0.0	0.0	0.0	0.0	0.0	0.0	0.0	0.0
0.0	0.0	0.0	0.0	0.0	0.0	0.0	0.0
0.0	0.0	0.0	0.0	0.0	0.0	0.0	0.0
0.0	0.0	0.0	0.0	0.0	0.0	0.0	0.0
0.0	0.0	0.0	0.0	0.0	0.0	0.0	0.0
0.0	0.0	0.0	0.0	0.0	0.0	0.0	0.0
0.0	0.0	0.0	0.0	0.0	0.0	0.0	0.0
0.0	0.0	0.0	0.0	0.0	0.0	0.0	0.0
0.0	0.0	0.0	0.0	0.0	0.0	0.0	0.0
0.0	0.0	0.0	0.0	0.0	0.0	0.0	0.0
0.0	0.0	0.0	0.0	0.0	0.0	0.0	0.0
0.0	0.0	0.0	0.0	0.0	0.0	0.0	0.0
0.0	0.0	0.0	0.0	0.0	0.0	0.0	0.0
0.0	0.0	0.0	0.0	0.0	0.0	0.0	0.0
0.0	0.0	0.0	0.0	0.0	0.0	0.0	0.0
0.0	0.0	0.0	0.0	0.0	0.0	0.0	0.0
0.0	0.0	0.0	0.0	0.0	0.0	0.0	0.0
0.0	0.0	0.0	0.0	0.0	0.0	0.0	0.0
0.0	0.0	0.0	0.0	0.0	0.0	0.0	0.0
0.0	0.0	0.0	0.0	0.0	0.0	0.0	0.0
0.0	0.0	0.0	0.0	0.0	0.0	0.0	0.0
0.0	0.0	0.0	0.0	0.0	0.0	0.0	0.0
0.0	0.0	0.0	0.0	0.0	0.0	0.0	0.0
0.0	0.0	0.0	0.0	0.0	0.0	0.0	0.0
0.0	0.0	0.0	0.0	0.0	0.0	0.0	0.0
0.0	0.0	0.0	0.0	0.0	0.0	0.0	0.0
0.0	0.0	0.0	0.0	0.0	0.0	0.0	0.0
0.0	0.0	0.0	0.0	0.0	0.0	0.0	0.0
0.0	0.0	0.0	0.0	0.0	0.0	0.0	0.0
0.0	0.0	0.0	0.0	0.0	0.0	0.0	0.0
0.0	0.0	0.0	0.0	0.0	0.0	0.0	0.0
0.0	0.0	0.0	0.0	0.0	0.0	0.0	0.0
0.0	0.0	0.0	0.0	0.0	0.0	0.0	0.0
0.0	0.0	0.0	0.0	0.0	0.0	0.0	0.0
0.0	0.0	0.0	0.0	0.0	0.0	0.0	0.0

0.0	0.0	0.0	0.0	0.0	0.0	0.0	0.0
0.0	0.0	0.0	0.0	0.0	0.0	0.0	0.0
0.0	0.0	0.0	0.0	0.0	0.0	0.0	0.0
0.0	0.0	0.0	0.0	0.0	0.0	0.0	0.0
0.0	0.0	0.0	0.0	0.0	0.0	0.0	0.0
0.0	0.0	0.0	0.0	0.0	0.0	0.0	0.0
0.0	0.0	0.0	0.0	0.0	0.0	0.0	0.0
0.0	0.0	0.0	0.0	0.0	0.0	0.0	0.0
0.0	0.0	0.0	0.0	0.0	0.0	0.0	0.0
0.0	0.0	0.0	0.0	0.0	0.0	0.0	0.0
0.0	0.0	0.0	0.0	0.0	0.0	0.0	0.0
0.0	0.0	0.0	0.0	0.0	0.0	0.0	0.0
0.0	0.0	0.0	0.0	0.0	0.0	0.0	0.0
0.0	0.0	0.0	0.0	0.0	0.0	0.0	0.0
0.0	0.0	0.0	0.0	0.0	0.0	0.0	0.0
0.0	0.0	0.0	0.0	0.0	0.0	0.0	0.0
0.0	0.0	0.0	0.0	0.0	0.0	0.0	0.0
0.0	0.0	0.0	0.0	0.0	0.0	0.0	0.0
0.0	0.0	0.0	0.0	0.0	0.0	0.0	0.0
0.0	0.0	0.0	0.0	0.0	0.0	0.0	0.0
0.0	0.0	0.0	0.0	0.0	0.0	0.0	0.0
0.0	0.0	0.0	0.0	0.0	0.0	0.0	0.0
0.0	0.0	0.0	0.0	0.0	0.0	0.0	0.0
0.0	0.0	0.0	0.0	0.0	0.0	0.0	0.0
0.0	0.0	0.0	0.0	0.0	0.0	0.0	0.0
0.0	0.0	0.0	0.0	0.0	0.0	0.0	0.0
0.0	0.0	0.0	0.0	0.0	0.0	0.0	0.0
0.0	0.0	0.0	0.0	0.0	0.0	0.0	0.0
0.0	0.0	0.0	0.0	0.0	0.0	0.0	0.0
0.0	0.0	0.0	0.0	0.0	0.0	0.0	0.0
0.0	0.0	0.0	0.0	0.0	0.0	0.0	0.0
0.0	0.0	0.0	0.0	0.0	0.0	0.0	0.0
0.0	0.0	0.0	0.0	0.0	0.0	0.0	0.0
0.0	0.0	0.0	0.0	0.0	0.0	0.0	0.0
0.0	0.0	0.0	0.0	0.0	0.0	0.0	0.0
0.0	0.0	0.0	0.0	0.0	0.0	0.0	0.0
0.0	0.0	0.0	0.0	0.0	0.0	0.0	0.0
0.0	0.0	0.0	0.0	0.0	0.0	0.0	0.0
0.0	0.0	0.0	0.0	0.0	0.0	0.0	0.0
0.0	0.0	0.0	0.0	0.0	0.0	0.0	0.0
0.0	0.0	0.0	0.0	0.0	0.0	0.0	0.0
0.0	0.0	0.0	0.0	0.0	0.0	0.0	0.0
0.0	0.0	0.0	0.0	0.0	0.0	0.0	0.0
0.0	0.0	0.0	0.0	0.0	0.0	0.0	0.0
0.0	0.0	0.0	0.0	0.0	0.0	0.0	0.0
0.0	0.0	0.0	0.0	0.0	0.0	0.0	0.0
0.0	0.0	0.0	0.0	0.0	0.0	0.0	0.0

0.0	0.0	0.0	0.0	0.0	0.0	0.0	0.0
0.0	0.0	0.0	0.0	0.0	0.0	0.0	0.0
0.0	0.0	0.0	0.0	0.0	0.0	0.0	0.0
0.0	0.0	0.0	0.0	0.0	0.0	0.0	0.0
0.0	0.0	0.0	0.0	0.0	0.0	0.0	0.0
0.0	0.0	0.0	0.0	0.0	0.0	0.0	0.0
0.0	0.0	0.0	0.0	0.0	0.0	0.0	0.0
0.0	0.0	0.0	0.0	0.0	0.0	0.0	0.0
0.0	0.0	0.0	0.0	0.0	0.0	0.0	0.0
0.0	0.0	0.0	0.0	0.0	0.0	0.0	0.0
0.0	0.0	0.0	0.0	0.0	0.0	0.0	0.0
0.0	0.0	0.0	0.0	0.0	0.0	0.0	0.0
0.0	0.0	0.0	0.0	0.0	0.0	0.0	0.0
0.0	0.0	0.0	0.0	0.0	0.0	0.0	0.0
0.0	0.0	0.0	0.0	0.0	0.0	0.0	0.0
0.0	0.0	0.0	0.0	0.0	0.0	0.0	0.0
0.0	0.0	0.0	0.0	0.0	0.0	0.0	0.0
0.0	0.0	0.0	0.0	0.0	0.0	0.0	0.0
0.0	0.0	0.0	0.0	0.0	0.0	0.0	0.0
0.0	0.0	0.0	0.0	0.0	0.0	0.0	0.0
0.0	0.0	0.0	0.0	0.0	0.0	0.0	0.0
0.0	0.0	0.0	0.0	0.0	0.0	0.0	0.0
0.0	0.0	0.0	0.0	0.0	0.0	0.0	0.0
0.0	0.0	0.0	0.0	0.0	0.0	0.0	0.0
0.0	0.0	0.0	0.0	0.0	0.0	0.0	0.0
0.0	0.0	0.0	0.0	0.0	0.0	0.0	0.0
0.0	0.0	0.0	0.0	0.0	0.0	0.0	0.0
0.0	0.0	0.0	0.0	0.0	0.0	0.0	0.0
0.0	0.0	0.0	0.0	0.0	0.0	0.0	0.0
0.0	0.0	0.0	0.0	0.0	0.0	0.0	0.0
0.0	0.0	0.0	0.0	0.0	0.0	0.0	0.0
0.0	0.0	0.0	0.0	0.0	0.0	0.0	0.0
0.0	0.0	0.0	0.0	0.0	0.0	0.0	0.0
0.0	0.0	0.0	0.0	0.0	0.0	0.0	0.0
0.0	0.0	0.0	0.0	0.0	0.0	0.0	0.0
0.0	0.0	0.0	0.0	0.0	0.0	0.0	0.0
0.0	0.0	0.0	0.0	0.0	0.0	0.0	0.0
0.0	0.0	0.0	0.0	0.0	0.0	0.0	0.0
0.0	0.0	0.0	0.0	0.0	0.0	0.0	0.0
0.0	0.0	0.0	0.0	0.0	0.0	0.0	0.0
0.0	0.0	0.0	0.0	0.0	0.0	0.0	0.0
0.0	0.0	0.0	0.0	0.0	0.0	0.0	0.0
0.0	0.0	0.0	0.0	0.0	0.0	0.0	0.0
0.0	0.0	0.0	0.0	0.0	0.0	0.0	0.0
0.0	0.0	0.0	0.0	0.0	0.0	0.0	0.0
0.0	0.0	0.0	0.0	0.0	0.0	0.0	0.0
0.0	0.0	0.0	0.0	0.0	0.0	0.0	0.0
0.0	0.0	0.0	0.0	0.0	0.0	0.0	0.0

0.0	0.0	0.0	0.0	0.0	0.0	0.0	0.0
0.0	0.0	0.0	0.0	0.0	0.0	0.0	0.0
0.0	0.0	0.0	0.0	0.0	0.0	0.0	0.0
0.0	0.0	0.0	0.0	0.0	0.0	0.0	0.0
0.0	0.0	0.0	0.0	0.0	0.0	0.0	0.0
0.0	0.0	0.0	0.0	0.0	0.0	0.0	0.0
0.0	0.0	0.0	0.0	0.0	0.0	0.0	0.0
0.0	0.0	0.0	0.0	0.0	0.0	0.0	0.0
0.0	0.0	0.0	0.0	0.0	0.0	0.0	0.0
0.0	0.0	0.0	0.0	0.0	0.0	0.0	0.0
0.0	0.0	0.0	0.0	0.0	0.0	0.0	0.0
0.0	0.0	0.0	0.0	0.0	0.0	0.0	0.0
0.0	0.0	0.0	0.0	0.0	0.0	0.0	0.0
0.0	0.0	0.0	0.0	0.0	0.0	0.0	0.0
0.0	0.0	0.0	0.0	0.0	0.0	0.0	0.0
0.0	0.0	0.0	0.0	0.0	0.0	0.0	0.0
0.0	0.0	0.0	0.0	0.0	0.0	0.0	0.0
0.0	0.0	0.0	0.0	0.0	0.0	0.0	0.0
0.0	0.0	0.0	0.0	0.0	0.0	0.0	0.0
0.0	0.0	0.0	0.0	0.0	0.0	0.0	0.0
0.0	0.0	0.0	0.0	0.0	0.0	0.0	0.0
0.0	0.0	0.0	0.0	0.0	0.0	0.0	0.0
0.0	0.0	0.0	0.0	0.0	0.0	0.0	0.0
0.0	0.0	0.0	0.0	0.0	0.0	0.0	0.0
0.0	0.0	0.0	0.0	0.0	0.0	0.0	0.0
0.0	0.0	0.0	0.0	0.0	0.0	0.0	0.0
0.0	0.0	0.0	0.0	0.0	0.0	0.0	0.0
0.0	0.0	0.0	0.0	0.0	0.0	0.0	0.0
0.0	0.0	0.0	0.0	0.0	0.0	0.0	0.0
0.0	0.0	0.0	0.0	0.0	0.0	0.0	0.0
0.0	0.0	0.0	0.0	0.0	0.0	0.0	0.0
0.0	0.0	0.0	0.0	0.0	0.0	0.0	0.0
0.0	0.0	0.0	0.0	0.0	0.0	0.0	0.0
0.0	0.0	0.0	0.0	0.0	0.0	0.0	0.0
0.0	0.0	0.0	0.0	0.0	0.0	0.0	0.0
0.0	0.0	0.0	0.0	0.0	0.0	0.0	0.0
0.0	0.0	0.0	0.0	0.0	0.0	0.0	0.0
0.0	0.0	0.0	0.0	0.0	0.0	0.0	0.0
0.0	0.0	0.0	0.0	0.0	0.0	0.0	0.0
0.0	0.0	0.0	0.0	0.0	0.0	0.0	0.0
0.0	0.0	0.0	0.0	0.0	0.0	0.0	0.0
0.0	0.0	0.0	0.0	0.0	0.0	0.0	0.0
0.0	0.0	0.0	0.0	0.0	0.0	0.0	0.0
0.0	0.0	0.0	0.0	0.0	0.0	0.0	0.0
0.0	0.0	0.0	0.0	0.0	0.0	0.0	0.0
0.0	0.0	0.0	0.0	0.0	0.0	0.0	0.0
0.0	0.0	0.0	0.0	0.0	0.0	0.0	0.0

0.0	0.0	0.0	0.0	0.0	0.0	0.0	0.0
0.0	0.0	0.0	0.0	0.0	0.0	0.0	0.0
0.0	0.0	0.0	0.0	0.0	0.0	0.0	0.0
0.0	0.0	0.0	0.0	0.0	0.0	0.0	0.0
0.0	0.0	0.0	0.0	0.0	0.0	0.0	0.0
0.0	0.0	0.0	0.0	0.0	0.0	0.0	0.0
0.0	0.0	0.0	0.0	0.0	0.0	0.0	0.0
0.0	0.0	0.0	0.0	0.0	0.0	0.0	0.0
0.0	0.0	0.0	0.0	0.0	0.0	0.0	0.0
0.0	0.0	0.0	0.0	0.0	0.0	0.0	0.0
0.0	0.0	0.0	0.0	0.0	0.0	0.0	0.0
0.0	0.0	0.0	0.0	0.0	0.0	0.0	0.0
0.0	0.0	0.0	0.0	0.0	0.0	0.0	0.0
0.0	0.0	0.0	0.0	0.0	0.0	0.0	0.0
0.0	0.0	0.0	0.0	0.0	0.0	0.0	0.0
0.0	0.0	0.0	0.0	0.0	0.0	0.0	0.0
0.0	0.0	0.0	0.0	0.0	0.0	0.0	0.0
0.0	0.0	0.0	0.0	0.0	0.0	0.0	0.0
0.0	0.0	0.0	0.0	0.0	0.0	0.0	0.0
0.0	0.0	0.0	0.0	0.0	0.0	0.0	0.0
0.0	0.0	0.0	0.0	0.0	0.0	0.0	0.0
0.0	0.0	0.0	0.0	0.0	0.0	0.0	0.0
0.0	0.0	0.0	0.0	0.0	0.0	0.0	0.0
0.0	0.0	0.0	0.0	0.0	0.0	0.0	0.0
0.0	0.0	0.0	0.0	0.0	0.0	0.0	0.0
0.0	0.0	0.0	0.0	0.0	0.0	0.0	0.0
0.0	0.0	0.0	0.0	0.0	0.0	0.0	0.0
0.0	0.0	0.0	0.0	0.0	0.0	0.0	0.0
0.0	0.0	0.0	0.0	0.0	0.0	0.0	0.0
0.0	0.0	0.0	0.0	0.0	0.0	0.0	0.0
0.0	0.0	0.0	0.0	0.0	0.0	0.0	0.0
0.0	0.0	0.0	0.0	0.0	0.0	0.0	0.0
0.0	0.0	0.0	0.0	0.0	0.0	0.0	0.0
0.0	0.0	0.0	0.0	0.0	0.0	0.0	0.0
0.0	0.0	0.0	0.0	0.0	0.0	0.0	0.0
0.0	0.0	0.0	0.0	0.0	0.0	0.0	0.0
0.0	0.0	0.0	0.0	0.0	0.0	0.0	0.0
0.0	0.0	0.0	0.0	0.0	0.0	0.0	0.0
0.0	0.0	0.0	0.0	0.0	0.0	0.0	0.0
0.0	0.0	0.0	0.0	0.0	0.0	0.0	0.0
0.0	0.0	0.0	0.0	0.0	0.0	0.0	0.0
0.0	0.0	0.0	0.0	0.0	0.0	0.0	0.0
0.0	0.0	0.0	0.0	0.0	0.0	0.0	0.0
0.0	0.0	0.0	0.0	0.0	0.0	0.0	0.0
0.0	0.0	0.0	0.0	0.0	0.0	0.0	0.0
0.0	0.0	0.0	0.0	0.0	0.0	0.0	0.0
0.0	0.0	0.0	0.0	0.0	0.0	0.0	0.0
0.0	0.0	0.0	0.0	0.0	0.0	0.0	0.0

0.0	0.0	0.0	0.0	0.0	0.0	0.0	0.0
0.0	0.0	0.0	0.0	0.0	0.0	0.0	0.0
0.0	0.0	0.0	0.0	0.0	0.0	0.0	0.0
0.0	0.0	0.0	0.0	0.0	0.0	0.0	0.0
0.0	0.0	0.0	0.0	0.0	0.0	0.0	0.0
0.0	0.0	0.0	0.0	0.0	0.0	0.0	0.0
0.0	0.0	0.0	0.0	0.0	0.0	0.0	0.0
0.0	0.0	0.0	0.0	0.0	0.0	0.0	0.0
0.0	0.0	0.0	0.0	0.0	0.0	0.0	0.0
0.0	0.0	0.0	0.0	0.0	0.0	0.0	0.0
0.0	0.0	0.0	0.0	0.0	0.0	0.0	0.0
0.0	0.0	0.0	0.0	0.0	0.0	0.0	0.0
0.0	0.0	0.0	0.0	0.0	0.0	0.0	0.0
0.0	0.0	0.0	0.0	0.0	0.0	0.0	0.0
0.0	0.0	0.0	0.0	0.0	0.0	0.0	0.0
0.0	0.0	0.0	0.0	0.0	0.0	0.0	0.0
0.0	0.0	0.0	0.0	0.0	0.0	0.0	0.0
0.0	0.0	0.0	0.0	0.0	0.0	0.0	0.0
0.0	0.0	0.0	0.0	0.0	0.0	0.0	0.0
0.0	0.0	0.0	0.0	0.0	0.0	0.0	0.0
0.0	0.0	0.0	0.0	0.0	0.0	0.0	0.0
0.0	0.0	0.0	0.0	0.0	0.0	0.0	0.0
0.0	0.0	0.0	0.0	0.0	0.0	0.0	0.0
0.0	0.0	0.0	0.0	0.0	0.0	0.0	0.0
0.0	0.0	0.0	0.0	0.0	0.0	0.0	0.0
0.0	0.0	0.0	0.0	0.0	0.0	0.0	0.0
0.0	0.0	0.0	0.0	0.0	0.0	0.0	0.0
0.0	0.0	0.0	0.0	0.0	0.0	0.0	0.0
0.0	0.0	0.0	0.0	0.0	0.0	0.0	0.0
0.0	0.0	0.0	0.0	0.0	0.0	0.0	0.0
0.0	0.0	0.0	0.0	0.0	0.0	0.0	0.0
0.0	0.0	0.0	0.0	0.0	0.0	0.0	0.0
0.0	0.0	0.0	0.0	0.0	0.0	0.0	0.0
0.0	0.0	0.0	0.0	0.0	0.0	0.0	0.0
0.0	0.0	0.0	0.0	0.0	0.0	0.0	0.0
0.0	0.0	0.0	0.0	0.0	0.0	0.0	0.0
0.0	0.0	0.0	0.0	0.0	0.0	0.0	0.0
0.0	0.0	0.0	0.0	0.0	0.0	0.0	0.0
0.0	0.0	0.0	0.0	0.0	0.0	0.0	0.0
0.0	0.0	0.0	0.0	0.0	0.0	0.0	0.0
0.0	0.0	0.0	0.0	0.0	0.0	0.0	0.0
0.0	0.0	0.0	0.0	0.0	0.0	0.0	0.0
0.0	0.0	0.0	0.0	0.0	0.0	0.0	0.0
0.0	0.0	0.0	0.0	0.0	0.0	0.0	0.0
0.0	0.0	0.0	0.0	0.0	0.0	0.0	0.0
0.0	0.0	0.0	0.0	0.0	0.0	0.0	0.0
0.0	0.0	0.0	0.0	0.0	0.0	0.0	0.0
0.0	0.0	0.0	0.0	0.0	0.0	0.0	0.0

0.0	0.0	0.0	0.0	0.0	0.0	0.0	0.0
0.0	0.0	0.0	0.0	0.0	0.0	0.0	0.0
0.0	0.0	0.0	0.0	0.0	0.0	0.0	0.0
0.0	0.0	0.0	0.0	0.0	0.0	0.0	0.0
0.0	0.0	0.0	0.0	0.0	0.0	0.0	0.0
0.0	0.0	0.0	0.0	0.0	0.0	0.0	0.0
0.0	0.0	0.0	0.0	0.0	0.0	0.0	0.0
0.0	0.0	0.0	0.0	0.0	0.0	0.0	0.0
0.0	0.0	0.0	0.0	0.0	0.0	0.0	0.0
0.0	0.0	0.0	0.0	0.0	0.0	0.0	0.0
0.0	0.0	0.0	0.0	0.0	0.0	0.0	0.0
0.0	0.0	0.0	0.0	0.0	0.0	0.0	0.0
0.0	0.0	0.0	0.0	0.0	0.0	0.0	0.0
0.0	0.0	0.0	0.0	0.0	0.0	0.0	0.0
0.0	0.0	0.0	0.0	0.0	0.0	0.0	0.0
0.0	0.0	0.0	0.0	0.0	0.0	0.0	0.0
0.0	0.0	0.0	0.0	0.0	0.0	0.0	0.0
0.0	0.0	0.0	0.0	0.0	0.0	0.0	0.0
0.0	0.0	0.0	0.0	0.0	0.0	0.0	0.0
0.0	0.0	0.0	0.0	0.0	0.0	0.0	0.0
0.0	0.0	0.0	0.0	0.0	0.0	0.0	0.0
0.0	0.0	0.0	0.0	0.0	0.0	0.0	0.0
0.0	0.0	0.0	0.0	0.0	0.0	0.0	0.0
0.0	0.0	0.0	0.0	0.0	0.0	0.0	0.0
0.0	0.0	0.0	0.0	0.0	0.0	0.0	0.0
0.0	0.0	0.0	0.0	0.0	0.0	0.0	0.0
0.0	0.0	0.0	0.0	0.0	0.0	0.0	0.0
0.0	0.0	0.0	0.0	0.0	0.0	0.0	0.0
0.0	0.0	0.0	0.0	0.0	0.0	0.0	0.0
0.0	0.0	0.0	0.0	0.0	0.0	0.0	0.0
0.0	0.0	0.0	0.0	0.0	0.0	0.0	0.0
0.0	0.0	0.0	0.0	0.0	0.0	0.0	0.0
0.0	0.0	0.0	0.0	0.0	0.0	0.0	0.0
0.0	0.0	0.0	0.0	0.0	0.0	0.0	0.0
0.0	0.0	0.0	0.0	0.0	0.0	0.0	0.0
0.0	0.0	0.0	0.0	0.0	0.0	0.0	0.0
0.0	0.0	0.0	0.0	0.0	0.0	0.0	0.0
0.0	0.0	0.0	0.0	0.0	0.0	0.0	0.0
0.0	0.0	0.0	0.0	0.0	0.0	0.0	0.0
0.0	0.0	0.0	0.0	0.0	0.0	0.0	0.0
0.0	0.0	0.0	0.0	0.0	0.0	0.0	0.0
0.0	0.0	0.0	0.0	0.0	0.0	0.0	0.0
0.0	0.0	0.0	0.0	0.0	0.0	0.0	0.0
0.0	0.0	0.0	0.0	0.0	0.0	0.0	0.0
0.0	0.0	0.0	0.0	0.0	0.0	0.0	0.0
0.0	0.0	0.0	0.0	0.0	0.0	0.0	0.0
0.0	0.0	0.0	0.0	0.0	0.0	0.0	0.0

0.0	0.0	0.0	0.0	0.0	0.0	0.0	0.0
0.0	0.0	0.0	0.0	0.0	0.0	0.0	0.0
0.0	0.0	0.0	0.0	0.0	0.0	0.0	0.0
0.0	0.0	0.0	0.0	0.0	0.0	0.0	0.0
0.0	0.0	0.0	0.0	0.0	0.0	0.0	0.0
0.0	0.0	0.0	0.0	0.0	0.0	0.0	0.0
0.0	0.0	0.0	0.0	0.0	0.0	0.0	0.0
0.0	0.0	0.0	0.0	0.0	0.0	0.0	0.0
0.0	0.0	0.0	0.0	0.0	0.0	0.0	0.0
0.0	0.0	0.0	0.0	0.0	0.0	0.0	0.0
0.0	0.0	0.0	0.0	0.0	0.0	0.0	0.0
0.0	0.0	0.0	0.0	0.0	0.0	0.0	0.0
0.0	0.0	0.0	0.0	0.0	0.0	0.0	0.0
0.0	0.0	0.0	0.0	0.0	0.0	0.0	0.0
0.0	0.0	0.0	0.0	0.0	0.0	0.0	0.0
0.0	0.0	0.0	0.0	0.0	0.0	0.0	0.0
0.0	0.0	0.0	0.0	0.0	0.0	0.0	0.0
0.0	0.0	0.0	0.0	0.0	0.0	0.0	0.0
0.0	0.0	0.0	0.0	0.0	0.0	0.0	0.0
0.0	0.0	0.0	0.0	0.0	0.0	0.0	0.0
0.0	0.0	0.0	0.0	0.0	0.0	0.0	0.0
0.0	0.0	0.0	0.0	0.0	0.0	0.0	0.0
0.0	0.0	0.0	0.0	0.0	0.0	0.0	0.0
0.0	0.0	0.0	0.0	0.0	0.0	0.0	0.0
0.0	0.0	0.0	0.0	0.0	0.0	0.0	0.0
0.0	0.0	0.0	0.0	0.0	0.0	0.0	0.0
0.0	0.0	0.0	0.0	0.0	0.0	0.0	0.0
0.0	0.0	0.0	0.0	0.0	0.0	0.0	0.0
0.0	0.0	0.0	0.0	0.0	0.0	0.0	0.0
0.0	0.0	0.0	0.0	0.0	0.0	0.0	0.0
0.0	0.0	0.0	0.0	0.0	0.0	0.0	0.0
0.0	0.0	0.0	0.0	0.0	0.0	0.0	0.0
0.0	0.0	0.0	0.0	0.0	0.0	0.0	0.0
0.0	0.0	0.0	0.0	0.0	0.0	0.0	0.0
0.0	0.0	0.0	0.0	0.0	0.0	0.0	0.0
0.0	0.0	0.0	0.0	0.0	0.0	0.0	0.0
0.0	0.0	0.0	0.0	0.0	0.0	0.0	0.0
0.0	0.0	0.0	0.0	0.0	0.0	0.0	0.0
0.0	0.0	0.0	0.0	0.0	0.0	0.0	0.0
0.0	0.0	0.0	0.0	0.0	0.0	0.0	0.0
0.0	0.0	0.0	0.0	0.0	0.0	0.0	0.0
0.0	0.0	0.0	0.0	0.0	0.0	0.0	0.0
0.0	0.0	0.0	0.0	0.0	0.0	0.0	0.0
0.0	0.0	0.0	0.0	0.0	0.0	0.0	0.0
0.0	0.0	0.0	0.0	0.0	0.0	0.0	0.0
0.0	0.0	0.0	0.0	0.0	0.0	0.0	0.0
0.0	0.0	0.0	0.0	0.0	0.0	0.0	0.0

0.0	0.0	0.0	0.0	0.0	0.0	0.0	0.0
0.0	0.0	0.0	0.0	0.0	0.0	0.0	0.0
0.0	0.0	0.0	0.0	0.0	0.0	0.0	0.0
0.0	0.0	0.0	0.0	0.0	0.0	0.0	0.0
0.0	0.0	0.0	0.0	0.0	0.0	0.0	0.0
0.0	0.0	0.0	0.0	0.0	0.0	0.0	0.0
0.0	0.0	0.0	0.0	0.0	0.0	0.0	0.0
0.0	0.0	0.0	0.0	0.0	0.0	0.0	0.0
0.0	0.0	0.0	0.0	0.0	0.0	0.0	0.0
0.0	0.0	0.0	0.0	0.0	0.0	0.0	0.0
0.0	0.0	0.0	0.0	0.0	0.0	0.0	0.0
0.0	0.0	0.0	0.0	0.0	0.0	0.0	0.0
0.0	0.0	0.0	0.0	0.0	0.0	0.0	0.0
0.0	0.0	0.0	0.0	0.0	0.0	0.0	0.0
0.0	0.0	0.0	0.0	0.0	0.0	0.0	0.0
0.0	0.0	0.0	0.0	0.0	0.0	0.0	0.0
0.0	0.0	0.0	0.0	0.0	0.0	0.0	0.0
0.0	0.0	0.0	0.0	0.0	0.0	0.0	0.0
0.0	0.0	0.0	0.0	0.0	0.0	0.0	0.0
0.0	0.0	0.0	0.0	0.0	0.0	0.0	0.0
0.0	0.0	0.0	0.0	0.0	0.0	0.0	0.0
0.0	0.0	0.0	0.0	0.0	0.0	0.0	0.0
0.0	0.0	0.0	0.0	0.0	0.0	0.0	0.0
0.0	0.0	0.0	0.0	0.0	0.0	0.0	0.0
0.0	0.0	0.0	0.0	0.0	0.0	0.0	0.0
0.0	0.0	0.0	0.0	0.0	0.0	0.0	0.0
0.0	0.0	0.0	0.0	0.0	0.0	0.0	0.0
0.0	0.0	0.0	0.0	0.0	0.0	0.0	0.0
0.0	0.0	0.0	0.0	0.0	0.0	0.0	0.0
0.0	0.0	0.0	0.0	0.0	0.0	0.0	0.0
0.0	0.0	0.0	0.0	0.0	0.0	0.0	0.0
0.0	0.0	0.0	0.0	0.0	0.0	0.0	0.0
0.0	0.0	0.0	0.0	0.0	0.0	0.0	0.0
0.0	0.0	0.0	0.0	0.0	0.0	0.0	0.0
0.0	0.0	0.0	0.0	0.0	0.0	0.0	0.0
0.0	0.0	0.0	0.0	0.0	0.0	0.0	0.0
0.0	0.0	0.0	0.0	0.0	0.0	0.0	0.0
0.0	0.0	0.0	0.0	0.0	0.0	0.0	0.0
0.0	0.0	0.0	0.0	0.0	0.0	0.0	0.0
0.0	0.0	0.0	0.0	0.0	0.0	0.0	0.0
0.0	0.0	0.0	0.0	0.0	0.0	0.0	0.0
0.0	0.0	0.0	0.0	0.0	0.0	0.0	0.0
0.0	0.0	0.0	0.0	0.0	0.0	0.0	0.0
0.0	0.0	0.0	0.0	0.0	0.0	0.0	0.0
0.0	0.0	0.0	0.0	0.0	0.0	0.0	0.0
0.0	0.0	0.0	0.0	0.0	0.0	0.0	0.0
0.0	0.0	0.0	0.0	0.0	0.0	0.0	0.0

0.0	0.0	0.0	0.0	0.0	0.0	0.0	0.0
0.0	0.0	0.0	0.0	0.0	0.0	0.0	0.0
0.0	0.0	0.0	0.0	0.0	0.0	0.0	0.0
0.0	0.0	0.0	0.0	0.0	0.0	0.0	0.0
0.0	0.0	0.0	0.0	0.0	0.0	0.0	0.0
0.0	0.0	0.0	0.0	0.0	0.0	0.0	0.0
0.0	0.0	0.0	0.0	0.0	0.0	0.0	0.0
0.0	0.0	0.0	0.0	0.0	0.0	0.0	0.0
0.0	0.0	0.0	0.0	0.0	0.0	0.0	0.0
0.0	0.0	0.0	0.0	0.0	0.0	0.0	0.0
0.0	0.0	0.0	0.0	0.0	0.0	0.0	0.0
0.0	0.0	0.0	0.0	0.0	0.0	0.0	0.0
0.0	0.0	0.0	0.0	0.0	0.0	0.0	0.0
0.0	0.0	0.0	0.0	0.0	0.0	0.0	0.0
0.0	0.0	0.0	0.0	0.0	0.0	0.0	0.0
0.0	0.0	0.0	0.0	0.0	0.0	0.0	0.0
0.0	0.0	0.0	0.0	0.0	0.0	0.0	0.0
0.0	0.0	0.0	0.0	0.0	0.0	0.0	0.0
0.0	0.0	0.0	0.0	0.0	0.0	0.0	0.0
0.0	0.0	0.0	0.0	0.0	0.0	0.0	0.0
0.0	0.0	0.0	0.0	0.0	0.0	0.0	0.0
0.0	0.0	0.0	0.0	0.0	0.0	0.0	0.0
0.0	0.0	0.0	0.0	0.0	0.0	0.0	0.0
0.0	0.0	0.0	0.0	0.0	0.0	0.0	0.0
0.0	0.0	0.0	0.0	0.0	0.0	0.0	0.0
0.0	0.0	0.0	0.0	0.0	0.0	0.0	0.0
0.0	0.0	0.0	0.0	0.0	0.0	0.0	0.0
0.0	0.0	0.0	0.0	0.0	0.0	0.0	0.0
0.0	0.0	0.0	0.0	0.0	0.0	0.0	0.0
0.0	0.0	0.0	0.0	0.0	0.0	0.0	0.0
0.0	0.0	0.0	0.0	0.0	0.0	0.0	0.0
0.0	0.0	0.0	0.0	0.0	0.0	0.0	0.0
0.0	0.0	0.0	0.0	0.0	0.0	0.0	0.0
0.0	0.0	0.0	0.0	0.0	0.0	0.0	0.0
0.0	0.0	0.0	0.0	0.0	0.0	0.0	0.0
0.0	0.0	0.0	0.0	0.0	0.0	0.0	0.0
0.0	0.0	0.0	0.0	0.0	0.0	0.0	0.0
0.0	0.0	0.0	0.0	0.0	0.0	0.0	0.0
0.0	0.0	0.0	0.0	0.0	0.0	0.0	0.0
0.0	0.0	0.0	0.0	0.0	0.0	0.0	0.0
0.0	0.0	0.0	0.0	0.0	0.0	0.0	0.0
0.0	0.0	0.0	0.0	0.0	0.0	0.0	0.0
0.0	0.0	0.0	0.0	0.0	0.0	0.0	0.0
0.0	0.0	0.0	0.0	0.0	0.0	0.0	0.0
0.0	0.0	0.0	0.0	0.0	0.0	0.0	0.0
0.0	0.0	0.0	0.0	0.0	0.0	0.0	0.0
0.0	0.0	0.0	0.0	0.0	0.0	0.0	0.0

0.0	0.0	0.0	0.0	0.0	0.0	0.0	0.0
0.0	0.0	0.0	0.0	0.0	0.0	0.0	0.0
0.0	0.0	0.0	0.0	0.0	0.0	0.0	0.0
0.0	0.0	0.0	0.0	0.0	0.0	0.0	0.0
0.0	0.0	0.0	0.0	0.0	0.0	0.0	0.0
0.0	0.0	0.0	0.0	0.0	0.0	0.0	0.0
0.0	0.0	0.0	0.0	0.0	0.0	0.0	0.0
0.0	0.0	0.0	0.0	0.0	0.0	0.0	0.0
0.0	0.0	0.0	0.0	0.0	0.0	0.0	0.0
0.0	0.0	0.0	0.0	0.0	0.0	0.0	0.0
0.0	0.0	0.0	0.0	0.0	0.0	0.0	0.0
0.0	0.0	0.0	0.0	0.0	0.0	0.0	0.0
0.0	0.0	0.0	0.0	0.0	0.0	0.0	0.0
0.0	0.0	0.0	0.0	0.0	0.0	0.0	0.0
0.0	0.0	0.0	0.0	0.0	0.0	0.0	0.0
0.0	0.0	0.0	0.0	0.0	0.0	0.0	0.0
0.0	0.0	0.0	0.0	0.0	0.0	0.0	0.0
0.0	0.0	0.0	0.0	0.0	0.0	0.0	0.0
0.0	0.0	0.0	0.0	0.0	0.0	0.0	0.0
0.0	0.0	0.0	0.0	0.0	0.0	0.0	0.0
0.0	0.0	0.0	0.0	0.0	0.0	0.0	0.0
0.0	0.0	0.0	0.0	0.0	0.0	0.0	0.0
0.0	0.0	0.0	0.0	0.0	0.0	0.0	0.0
0.0	0.0	0.0	0.0	0.0	0.0	0.0	0.0
0.0	0.0	0.0	0.0	0.0	0.0	0.0	0.0
0.0	0.0	0.0	0.0	0.0	0.0	0.0	0.0
0.0	0.0	0.0	0.0	0.0	0.0	0.0	0.0
0.0	0.0	0.0	0.0	0.0	0.0	0.0	0.0
0.0	0.0	0.0	0.0	0.0	0.0	0.0	0.0
0.0	0.0	0.0	0.0	0.0	0.0	0.0	0.0
0.0	0.0	0.0	0.0	0.0	0.0	0.0	0.0
0.0	0.0	0.0	0.0	0.0	0.0	0.0	0.0
0.0	0.0	0.0	0.0	0.0	0.0	0.0	0.0
0.0	0.0	0.0	0.0	0.0	0.0	0.0	0.0
0.0	0.0	0.0	0.0	0.0	0.0	0.0	0.0
0.0	0.0	0.0	0.0	0.0	0.0	0.0	0.0
0.0	0.0	0.0	0.0	0.0	0.0	0.0	0.0
0.0	0.0	0.0	0.0	0.0	0.0	0.0	0.0
0.0	0.0	0.0	0.0	0.0	0.0	0.0	0.0
0.0	0.0	0.0	0.0	0.0	0.0	0.0	0.0
0.0	0.0	0.0	0.0	0.0	0.0	0.0	0.0
0.0	0.0	0.0	0.0	0.0	0.0	0.0	0.0
0.0	0.0	0.0	0.0	0.0	0.0	0.0	0.0
0.0	0.0	0.0	0.0	0.0	0.0	0.0	0.0
0.0	0.0	0.0	0.0	0.0	0.0	0.0	0.0
0.0	0.0	0.0	0.0	0.0	0.0	0.0	0.0
0.0	0.0	0.0	0.0	0.0	0.0	0.0	0.0

0.0	0.0	0.0	0.0	0.0	0.0	0.0	0.0
0.0	0.0	0.0	0.0	0.0	0.0	0.0	0.0
0.0	0.0	0.0	0.0	0.0	0.0	0.0	0.0
0.0	0.0	0.0	0.0	0.0	0.0	0.0	0.0
0.0	0.0	0.0	0.0	0.0	0.0	0.0	0.0
0.0	0.0	0.0	0.0	0.0	0.0	0.0	0.0
0.0	0.0	0.0	0.0	0.0	0.0	0.0	0.0
0.0	0.0	0.0	0.0	0.0	0.0	0.0	0.0
0.0	0.0	0.0	0.0	0.0	0.0	0.0	0.0
0.0	0.0	0.0	0.0	0.0	0.0	0.0	0.0
0.0	0.0	0.0	0.0	0.0	0.0	0.0	0.0
0.0	0.0	0.0	0.0	0.0	0.0	0.0	0.0
0.0	0.0	0.0	0.0	0.0	0.0	0.0	0.0
0.0	0.0	0.0	0.0	0.0	0.0	0.0	0.0
0.0	0.0	0.0	0.0	0.0	0.0	0.0	0.0
0.0	0.0	0.0	0.0	0.0	0.0	0.0	0.0
0.0	0.0	0.0	0.0	0.0	0.0	0.0	0.0
0.0	0.0	0.0	0.0	0.0	0.0	0.0	0.0
0.0	0.0	0.0	0.0	0.0	0.0	0.0	0.0
0.0	0.0	0.0	0.0	0.0	0.0	0.0	0.0
0.0	0.0	0.0	0.0	0.0	0.0	0.0	0.0
0.0	0.0	0.0	0.0	0.0	0.0	0.0	0.0
0.0	0.0	0.0	0.0	0.0	0.0	0.0	0.0
0.0	0.0	0.0	0.0	0.0	0.0	0.0	0.0
0.0	0.0	0.0	0.0	0.0	0.0	0.0	0.0
0.0	0.0	0.0	0.0	0.0	0.0	0.0	0.0
0.0	0.0	0.0	0.0	0.0	0.0	0.0	0.0
0.0	0.0	0.0	0.0	0.0	0.0	0.0	0.0
0.0	0.0	0.0	0.0	0.0	0.0	0.0	0.0
0.0	0.0	0.0	0.0	0.0	0.0	0.0	0.0
0.0	0.0	0.0	0.0	0.0	0.0	0.0	0.0
0.0	0.0	0.0	0.0	0.0	0.0	0.0	0.0
0.0	0.0	0.0	0.0	0.0	0.0	0.0	0.0
0.0	0.0	0.0	0.0	0.0	0.0	0.0	0.0
0.0	0.0	0.0	0.0	0.0	0.0	0.0	0.0
0.0	0.0	0.0	0.0	0.0	0.0	0.0	0.0
0.0	0.0	0.0	0.0	0.0	0.0	0.0	0.0
0.0	0.0	0.0	0.0	0.0	0.0	0.0	0.0
0.0	0.0	0.0	0.0	0.0	0.0	0.0	0.0
0.0	0.0	0.0	0.0	0.0	0.0	0.0	0.0
0.0	0.0	0.0	0.0	0.0	0.0	0.0	0.0
0.0	0.0	0.0	0.0	0.0	0.0	0.0	0.0
0.0	0.0	0.0	0.0	0.0	0.0	0.0	0.0
0.0	0.0	0.0	0.0	0.0	0.0	0.0	0.0
0.0	0.0	0.0	0.0	0.0	0.0	0.0	0.0
0.0	0.0	0.0	0.0	0.0	0.0	0.0	0.0
0.0	0.0	0.0	0.0	0.0	0.0	0.0	0.0

0.0	0.0	0.0	0.0	0.0	0.0	0.0	0.0
0.0	0.0	0.0	0.0	0.0	0.0	0.0	0.0
0.0	0.0	0.0	0.0	0.0	0.0	0.0	0.0
0.0	0.0	0.0	0.0	0.0	0.0	0.0	0.0
0.0	0.0	0.0	0.0	0.0	0.0	0.0	0.0
0.0	0.0	0.0	0.0	0.0	0.0	0.0	0.0
0.0	0.0	0.0	0.0	0.0	0.0	0.0	0.0
0.0	0.0	0.0	0.0	0.0	0.0	0.0	0.0
0.0	0.0	0.0	0.0	0.0	0.0	0.0	0.0
0.0	0.0	0.0	0.0	0.0	0.0	0.0	0.0
0.0	0.0	0.0	0.0	0.0	0.0	0.0	0.0
0.0	0.0	0.0	0.0	0.0	0.0	0.0	0.0
0.0	0.0	0.0	0.0	0.0	0.0	0.0	0.0
0.0	0.0	0.0	0.0	0.0	0.0	0.0	0.0
0.0	0.0	0.0	0.0	0.0	0.0	0.0	0.0
0.0	0.0	0.0	0.0	0.0	0.0	0.0	0.0
0.0	0.0	0.0	0.0	0.0	0.0	0.0	0.0
0.0	0.0	0.0	0.0	0.0	0.0	0.0	0.0
0.0	0.0	0.0	0.0	0.0	0.0	0.0	0.0
0.0	0.0	0.0	0.0	0.0	0.0	0.0	0.0
0.0	0.0	0.0	0.0	0.0	0.0	0.0	0.0
0.0	0.0	0.0	0.0	0.0	0.0	0.0	0.0
0.0	0.0	0.0	0.0	0.0	0.0	0.0	0.0
0.0	0.0	0.0	0.0	0.0	0.0	0.0	0.0
0.0	0.0	0.0	0.0	0.0	0.0	0.0	0.0
0.0	0.0	0.0	0.0	0.0	0.0	0.0	0.0
0.0	0.0	0.0	0.0	0.0	0.0	0.0	0.0
0.0	0.0	0.0	0.0	0.0	0.0	0.0	0.0
0.0	0.0	0.0	0.0	0.0	0.0	0.0	0.0
0.0	0.0	0.0	0.0	0.0	0.0	0.0	0.0
0.0	0.0	0.0	0.0	0.0	0.0	0.0	0.0
0.0	0.0	0.0	0.0	0.0	0.0	0.0	0.0
0.0	0.0	0.0	0.0	0.0	0.0	0.0	0.0
0.0	0.0	0.0	0.0	0.0	0.0	0.0	0.0
0.0	0.0	0.0	0.0	0.0	0.0	0.0	0.0
0.0	0.0	0.0	0.0	0.0	0.0	0.0	0.0
0.0	0.0	0.0	0.0	0.0	0.0	0.0	0.0
0.0	0.0	0.0	0.0	0.0	0.0	0.0	0.0
0.0	0.0	0.0	0.0	0.0	0.0	0.0	0.0
0.0	0.0	0.0	0.0	0.0	0.0	0.0	0.0
0.0	0.0	0.0	0.0	0.0	0.0	0.0	0.0
0.0	0.0	0.0	0.0	0.0	0.0	0.0	0.0
0.0	0.0	0.0	0.0	0.0	0.0	0.0	0.0
0.0	0.0	0.0	0.0	0.0	0.0	0.0	0.0
0.0	0.0	0.0	0.0	0.0	0.0	0.0	0.0
0.0	0.0	0.0	0.0	0.0	0.0	0.0	0.0
0.0	0.0	0.0	0.0	0.0	0.0	0.0	0.0

0.0	0.0	0.0	0.0	0.0	0.0	0.0	0.0
0.0	0.0	0.0	0.0	0.0	0.0	0.0	0.0
0.0	0.0	0.0	0.0	0.0	0.0	0.0	0.0
0.0	0.0	0.0	0.0	0.0	0.0	0.0	0.0
0.0	0.0	0.0	0.0	0.0	0.0	0.0	0.0
0.0	0.0	0.0	0.0	0.0	0.0	0.0	0.0
0.0	0.0	0.0	0.0	0.0	0.0	0.0	0.0
0.0	0.0	0.0	0.0	0.0	0.0	0.0	0.0
0.0	0.0	0.0	0.0	0.0	0.0	0.0	0.0
0.0	0.0	0.0	0.0	0.0	0.0	0.0	0.0
0.0	0.0	0.0	0.0	0.0	0.0	0.0	0.0
0.0	0.0	0.0	0.0	0.0	0.0	0.0	0.0
0.0	0.0	0.0	0.0	0.0	0.0	0.0	0.0
0.0	0.0	0.0	0.0	0.0	0.0	0.0	0.0
0.0	0.0	0.0	0.0	0.0	0.0	0.0	0.0
0.0	0.0	0.0	0.0	0.0	0.0	0.0	0.0
0.0	0.0	0.0	0.0	0.0	0.0	0.0	0.0
0.0	0.0	0.0	0.0	0.0	0.0	0.0	0.0
0.0	0.0	0.0	0.0	0.0	0.0	0.0	0.0
0.0	0.0	0.0	0.0	0.0	0.0	0.0	0.0
0.0	0.0	0.0	0.0	0.0	0.0	0.0	0.0
0.0	0.0	0.0	0.0	0.0	0.0	0.0	0.0
0.0	0.0	0.0	0.0	0.0	0.0	0.0	0.0
0.0	0.0	0.0	0.0	0.0	0.0	0.0	0.0
0.0	0.0	0.0	0.0	0.0	0.0	0.0	0.0
0.0	0.0	0.0	0.0	0.0	0.0	0.0	0.0
0.0	0.0	0.0	0.0	0.0	0.0	0.0	0.0
0.0	0.0	0.0	0.0	0.0	0.0	0.0	0.0
0.0	0.0	0.0	0.0	0.0	0.0	0.0	0.0
0.0	0.0	0.0	0.0	0.0	0.0	0.0	0.0
0.0	0.0	0.0	0.0	0.0	0.0	0.0	0.0
0.0	0.0	0.0	0.0	0.0	0.0	0.0	0.0
0.0	0.0	0.0	0.0	0.0	0.0	0.0	0.0
0.0	0.0	0.0	0.0	0.0	0.0	0.0	0.0
0.0	0.0	0.0	0.0	0.0	0.0	0.0	0.0
0.0	0.0	0.0	0.0	0.0	0.0	0.0	0.0
0.0	0.0	0.0	0.0	0.0	0.0	0.0	0.0
0.0	0.0	0.0	0.0	0.0	0.0	0.0	0.0
0.0	0.0	0.0	0.0	0.0	0.0	0.0	0.0
0.0	0.0	0.0	0.0	0.0	0.0	0.0	0.0
0.0	0.0	0.0	0.0	0.0	0.0	0.0	0.0
0.0	0.0	0.0	0.0	0.0	0.0	0.0	0.0
0.0	0.0	0.0	0.0	0.0	0.0	0.0	0.0
0.0	0.0	0.0	0.0	0.0	0.0	0.0	0.0
0.0	0.0	0.0	0.0	0.0	0.0	0.0	0.0
0.0	0.0	0.0	0.0	0.0	0.0	0.0	0.0
0.0	0.0	0.0	0.0	0.0	0.0	0.0	0.0

0.0	0.0	0.0	0.0	0.0	0.0	0.0	0.0
0.0	0.0	0.0	0.0	0.0	0.0	0.0	0.0
0.0	0.0	0.0	0.0	0.0	0.0	0.0	0.0
0.0	0.0	0.0	0.0	0.0	0.0	0.0	0.0
0.0	0.0	0.0	0.0	0.0	0.0	0.0	0.0
0.0	0.0	0.0	0.0	0.0	0.0	0.0	0.0
0.0	0.0	0.0	0.0	0.0	0.0	0.0	0.0
0.0	0.0	0.0	0.0	0.0	0.0	0.0	0.0
0.0	0.0	0.0	0.0	0.0	0.0	0.0	0.0
0.0	0.0	0.0	0.0	0.0	0.0	0.0	0.0
0.0	0.0	0.0	0.0	0.0	0.0	0.0	0.0
0.0	0.0	0.0	0.0	0.0	0.0	0.0	0.0
0.0	0.0	0.0	0.0	0.0	0.0	0.0	0.0
0.0	0.0	0.0	0.0	0.0	0.0	0.0	0.0
0.0	0.0	0.0	0.0	0.0	0.0	0.0	0.0
0.0	0.0	0.0	0.0	0.0	0.0	0.0	0.0
0.0	0.0	0.0	0.0	0.0	0.0	0.0	0.0
0.0	0.0	0.0	0.0	0.0	0.0	0.0	0.0
0.0	0.0	0.0	0.0	0.0	0.0	0.0	0.0
0.0	0.0	0.0	0.0	0.0	0.0	0.0	0.0
0.0	0.0	0.0	0.0	0.0	0.0	0.0	0.0
0.0	0.0	0.0	0.0	0.0	0.0	0.0	0.0
0.0	0.0	0.0	0.0	0.0	0.0	0.0	0.0
0.0	0.0	0.0	0.0	0.0	0.0	0.0	0.0
0.0	0.0	0.0	0.0	0.0	0.0	0.0	0.0
0.0	0.0	0.0	0.0	0.0	0.0	0.0	0.0
0.0	0.0	0.0	0.0	0.0	0.0	0.0	0.0
0.0	0.0	0.0	0.0	0.0	0.0	0.0	0.0
0.0	0.0	0.0	0.0	0.0	0.0	0.0	0.0
0.0	0.0	0.0	0.0	0.0	0.0	0.0	0.0
0.0	0.0	0.0	0.0	0.0	0.0	0.0	0.0
0.0	0.0	0.0	0.0	0.0	0.0	0.0	0.0
0.0	0.0	0.0	0.0	0.0	0.0	0.0	0.0
0.0	0.0	0.0	0.0	0.0	0.0	0.0	0.0
0.0	0.0	0.0	0.0	0.0	0.0	0.0	0.0
0.0	0.0	0.0	0.0	0.0	0.0	0.0	0.0
0.0	0.0	0.0	0.0	0.0	0.0	0.0	0.0
0.0	0.0	0.0	0.0	0.0	0.0	0.0	0.0
0.0	0.0	0.0	0.0	0.0	0.0	0.0	0.0
0.0	0.0	0.0	0.0	0.0	0.0	0.0	0.0
0.0	0.0	0.0	0.0	0.0	0.0	0.0	0.0
0.0	0.0	0.0	0.0	0.0	0.0	0.0	0.0
0.0	0.0	0.0	0.0	0.0	0.0	0.0	0.0
0.0	0.0	0.0	0.0	0.0	0.0	0.0	0.0
0.0	0.0	0.0	0.0	0.0	0.0	0.0	0.0
0.0	0.0	0.0	0.0	0.0	0.0	0.0	0.0
0.0	0.0	0.0	0.0	0.0	0.0	0.0	0.0

0.0	0.0	0.0	0.0	0.0	0.0	0.0	0.0
0.0	0.0	0.0	0.0	0.0	0.0	0.0	0.0
0.0	0.0	0.0	0.0	0.0	0.0	0.0	0.0
0.0	0.0	0.0	0.0	0.0	0.0	0.0	0.0
0.0	0.0	0.0	0.0	0.0	0.0	0.0	0.0
0.0	0.0	0.0	0.0	0.0	0.0	0.0	0.0
0.0	0.0	0.0	0.0	0.0	0.0	0.0	0.0
0.0	0.0	0.0	0.0	0.0	0.0	0.0	0.0
0.0	0.0	0.0	0.0	0.0	0.0	0.0	0.0
0.0	0.0	0.0	0.0	0.0	0.0	0.0	0.0
0.0	0.0	0.0	0.0	0.0	0.0	0.0	0.0
0.0	0.0	0.0	0.0	0.0	0.0	0.0	0.0
0.0	0.0	0.0	0.0	0.0	0.0	0.0	0.0
0.0	0.0	0.0	0.0	0.0	0.0	0.0	0.0
0.0	0.0	0.0	0.0	0.0	0.0	0.0	0.0
0.0	0.0	0.0	0.0	0.0	0.0	0.0	0.0
0.0	0.0	0.0	0.0	0.0	0.0	0.0	0.0
0.0	0.0	0.0	0.0	0.0	0.0	0.0	0.0
0.0	0.0	0.0	0.0	0.0	0.0	0.0	0.0
0.0	0.0	0.0	0.0	0.0	0.0	0.0	0.0
0.0	0.0	0.0	0.0	0.0	0.0	0.0	0.0
0.0	0.0	0.0	0.0	0.0	0.0	0.0	0.0
0.0	0.0	0.0	0.0	0.0	0.0	0.0	0.0
0.0	0.0	0.0	0.0	0.0	0.0	0.0	0.0
0.0	0.0	0.0	0.0	0.0	0.0	0.0	0.0
0.0	0.0	0.0	0.0	0.0	0.0	0.0	0.0
0.0	0.0	0.0	0.0	0.0	0.0	0.0	0.0
0.0	0.0	0.0	0.0	0.0	0.0	0.0	0.0
0.0	0.0	0.0	0.0	0.0	0.0	0.0	0.0
0.0	0.0	0.0	0.0	0.0	0.0	0.0	0.0
0.0	0.0	0.0	0.0	0.0	0.0	0.0	0.0
0.0	0.0	0.0	0.0	0.0	0.0	0.0	0.0
0.0	0.0	0.0	0.0	0.0	0.0	0.0	0.0
0.0	0.0	0.0	0.0	0.0	0.0	0.0	0.0
0.0	0.0	0.0	0.0	0.0	0.0	0.0	0.0
0.0	0.0	0.0	0.0	0.0	0.0	0.0	0.0
0.0	0.0	0.0	0.0	0.0	0.0	0.0	0.0
0.0	0.0	0.0	0.0	0.0	0.0	0.0	0.0
0.0	0.0	0.0	0.0	0.0	0.0	0.0	0.0
0.0	0.0	0.0	0.0	0.0	0.0	0.0	0.0
0.0	0.0	0.0	0.0	0.0	0.0	0.0	0.0
0.0	0.0	0.0	0.0	0.0	0.0	0.0	0.0
0.0	0.0	0.0	0.0	0.0	0.0	0.0	0.0
0.0	0.0	0.0	0.0	0.0	0.0	0.0	0.0
0.0	0.0	0.0	0.0	0.0	0.0	0.0	0.0
0.0	0.0	0.0	0.0	0.0	0.0	0.0	0.0
0.0	0.0	0.0	0.0	0.0	0.0	0.0	0.0

0.0	0.0	0.0	0.0	0.0	0.0	0.0	0.0
0.0	0.0	0.0	0.0	0.0	0.0	0.0	0.0
0.0	0.0	0.0	0.0	0.0	0.0	0.0	0.0
0.0	0.0	0.0	0.0	0.0	0.0	0.0	0.0
0.0	0.0	0.0	0.0	0.0	0.0	0.0	0.0
0.0	0.0	0.0	0.0	0.0	0.0	0.0	0.0
0.0	0.0	0.0	0.0	0.0	0.0	0.0	0.0
0.0	0.0	0.0	0.0	0.0	0.0	0.0	0.0
0.0	0.0	0.0	0.0	0.0	0.0	0.0	0.0
0.0	0.0	0.0	0.0	0.0	0.0	0.0	0.0
0.0	0.0	0.0	0.0	0.0	0.0	0.0	0.0
0.0	0.0	0.0	0.0	0.0	0.0	0.0	0.0
0.0	0.0	0.0	0.0	0.0	0.0	0.0	0.0
0.0	0.0	0.0	0.0	0.0	0.0	0.0	0.0
0.0	0.0	0.0	0.0	0.0	0.0	0.0	0.0
0.0	0.0	0.0	0.0	0.0	0.0	0.0	0.0
0.0	0.0	0.0	0.0	0.0	0.0	0.0	0.0
0.0	0.0	0.0	0.0	0.0	0.0	0.0	0.0
0.0	0.0	0.0	0.0	0.0	0.0	0.0	0.0
0.0	0.0	0.0	0.0	0.0	0.0	0.0	0.0
0.0	0.0	0.0	0.0	0.0	0.0	0.0	0.0
0.0	0.0	0.0	0.0	0.0	0.0	0.0	0.0
0.0	0.0	0.0	0.0	0.0	0.0	0.0	0.0
0.0	0.0	0.0	0.0	0.0	0.0	0.0	0.0
0.0	0.0	0.0	0.0	0.0	0.0	0.0	0.0
0.0	0.0	0.0	0.0	0.0	0.0	0.0	0.0
0.0	0.0	0.0	0.0	0.0	0.0	0.0	0.0
0.0	0.0	0.0	0.0	0.0	0.0	0.0	0.0
0.0	0.0	0.0	0.0	0.0	0.0	0.0	0.0
0.0	0.0	0.0	0.0	0.0	0.0	0.0	0.0
0.0	0.0	0.0	0.0	0.0	0.0	0.0	0.0
0.0	0.0	0.0	0.0	0.0	0.0	0.0	0.0
0.0	0.0	0.0	0.0	0.0	0.0	0.0	0.0
0.0	0.0	0.0	0.0	0.0	0.0	0.0	0.0
0.0	0.0	0.0	0.0	0.0	0.0	0.0	0.0
0.0	0.0	0.0	0.0	0.0	0.0	0.0	0.0
0.0	0.0	0.0	0.0	0.0	0.0	0.0	0.0
0.0	0.0	0.0	0.0	0.0	0.0	0.0	0.0
0.0	0.0	0.0	0.0	0.0	0.0	0.0	0.0
0.0	0.0	0.0	0.0	0.0	0.0	0.0	0.0
0.0	0.0	0.0	0.0	0.0	0.0	0.0	0.0
0.0	0.0	0.0	0.0	0.0	0.0	0.0	0.0
0.0	0.0	0.0	0.0	0.0	0.0	0.0	0.0
0.0	0.0	0.0	0.0	0.0	0.0	0.0	0.0
0.0	0.0	0.0	0.0	0.0	0.0	0.0	0.0
0.0	0.0	0.0	0.0	0.0	0.0	0.0	0.0
0.0	0.0	0.0	0.0	0.0	0.0	0.0	0.0

0.0	0.0	0.0	0.0	0.0	0.0	0.0	0.0
0.0	0.0	0.0	0.0	0.0	0.0	0.0	0.0
0.0	0.0	0.0	0.0	0.0	0.0	0.0	0.0
0.0	0.0	0.0	0.0	0.0	0.0	0.0	0.0
0.0	0.0	0.0	0.0	0.0	0.0	0.0	0.0
0.0	0.0	0.0	0.0	0.0	0.0	0.0	0.0
0.0	0.0	0.0	0.0	0.0	0.0	0.0	0.0
0.0	0.0	0.0	0.0	0.0	0.0	0.0	0.0
0.0	0.0	0.0	0.0	0.0	0.0	0.0	0.0
0.0	0.0	0.0	0.0	0.0	0.0	0.0	0.0
0.0	0.0	0.0	0.0	0.0	0.0	0.0	0.0
0.0	0.0	0.0	0.0	0.0	0.0	0.0	0.0
0.0	0.0	0.0	0.0	0.0	0.0	0.0	0.0
0.0	0.0	0.0	0.0	0.0	0.0	0.0	0.0
0.0	0.0	0.0	0.0	0.0	0.0	0.0	0.0
0.0	0.0	0.0	0.0	0.0	0.0	0.0	0.0
0.0	0.0	0.0	0.0	0.0	0.0	0.0	0.0
0.0	0.0	0.0	0.0	0.0	0.0	0.0	0.0
0.0	0.0	0.0	0.0	0.0	0.0	0.0	0.0
0.0	0.0	0.0	0.0	0.0	0.0	0.0	0.0
0.0	0.0	0.0	0.0	0.0	0.0	0.0	0.0
0.0	0.0	0.0	0.0	0.0	0.0	0.0	0.0
0.0	0.0	0.0	0.0	0.0	0.0	0.0	0.0
0.0	0.0	0.0	0.0	0.0	0.0	0.0	0.0
0.0	0.0	0.0	0.0	0.0	0.0	0.0	0.0
0.0	0.0	0.0	0.0	0.0	0.0	0.0	0.0
0.0	0.0	0.0	0.0	0.0	0.0	0.0	0.0
0.0	0.0	0.0	0.0	0.0	0.0	0.0	0.0
0.0	0.0	0.0	0.0	0.0	0.0	0.0	0.0
0.0	0.0	0.0	0.0	0.0	0.0	0.0	0.0
0.0	0.0	0.0	0.0	0.0	0.0	0.0	0.0
0.0	0.0	0.0	0.0	0.0	0.0	0.0	0.0
0.0	0.0	0.0	0.0	0.0	0.0	0.0	0.0
0.0	0.0	0.0	0.0	0.0	0.0	0.0	0.0
0.0	0.0	0.0	0.0	0.0	0.0	0.0	0.0
0.0	0.0	0.0	0.0	0.0	0.0	0.0	0.0
0.0	0.0	0.0	0.0	0.0	0.0	0.0	0.0
0.0	0.0	0.0	0.0	0.0	0.0	0.0	0.0
0.0	0.0	0.0	0.0	0.0	0.0	0.0	0.0
0.0	0.0	0.0	0.0	0.0	0.0	0.0	0.0
0.0	0.0	0.0	0.0	0.0	0.0	0.0	0.0
0.0	0.0	0.0	0.0	0.0	0.0	0.0	0.0
0.0	0.0	0.0	0.0	0.0	0.0	0.0	0.0
0.0	0.0	0.0	0.0	0.0	0.0	0.0	0.0
0.0	0.0	0.0	0.0	0.0	0.0	0.0	0.0
0.0	0.0	0.0	0.0	0.0	0.0	0.0	0.0
0.0	0.0	0.0	0.0	0.0	0.0	0.0	0.0

0.0	0.0	0.0	0.0	0.0	0.0	0.0	0.0
0.0	0.0	0.0	0.0	0.0	0.0	0.0	0.0
0.0	0.0	0.0	0.0	0.0	0.0	0.0	0.0
0.0	0.0	0.0	0.0	0.0	0.0	0.0	0.0
0.0	0.0	0.0	0.0	0.0	0.0	0.0	0.0
0.0	0.0	0.0	0.0	0.0	0.0	0.0	0.0
0.0	0.0	0.0	0.0	0.0	0.0	0.0	0.0
0.0	0.0	0.0	0.0	0.0	0.0	0.0	0.0
0.0	0.0	0.0	0.0	0.0	0.0	0.0	0.0
0.0	0.0	0.0	0.0	0.0	0.0	0.0	0.0
0.0	0.0	0.0	0.0	0.0	0.0	0.0	0.0
0.0	0.0	0.0	0.0	0.0	0.0	0.0	0.0
0.0	0.0	0.0	0.0	0.0	0.0	0.0	0.0
0.0	0.0	0.0	0.0	0.0	0.0	0.0	0.0
0.0	0.0	0.0	0.0	0.0	0.0	0.0	0.0
0.0	0.0	0.0	0.0	0.0	0.0	0.0	0.0
0.0	0.0	0.0	0.0	0.0	0.0	0.0	0.0
0.0	0.0	0.0	0.0	0.0	0.0	0.0	0.0
0.0	0.0	0.0	0.0	0.0	0.0	0.0	0.0
0.0	0.0	0.0	0.0	0.0	0.0	0.0	0.0
0.0	0.0	0.0	0.0	0.0	0.0	0.0	0.0
0.0	0.0	0.0	0.0	0.0	0.0	0.0	0.0
0.0	0.0	0.0	0.0	0.0	0.0	0.0	0.0
0.0	0.0	0.0	0.0	0.0	0.0	0.0	0.0
0.0	0.0	0.0	0.0	0.0	0.0	0.0	0.0
0.0	0.0	0.0	0.0	0.0	0.0	0.0	0.0
0.0	0.0	0.0	0.0	0.0	0.0	0.0	0.0
0.0	0.0	0.0	0.0	0.0	0.0	0.0	0.0
0.0	0.0	0.0	0.0	0.0	0.0	0.0	0.0
0.0	0.0	0.0	0.0	0.0	0.0	0.0	0.0
0.0	0.0	0.0	0.0	0.0	0.0	0.0	0.0
0.0	0.0	0.0	0.0	0.0	0.0	0.0	0.0
0.0	0.0	0.0	0.0	0.0	0.0	0.0	0.0
0.0	0.0	0.0	0.0	0.0	0.0	0.0	0.0
0.0	0.0	0.0	0.0	0.0	0.0	0.0	0.0
0.0	0.0	0.0	0.0	0.0	0.0	0.0	0.0
0.0	0.0	0.0	0.0	0.0	0.0	0.0	0.0
0.0	0.0	0.0	0.0	0.0	0.0	0.0	0.0
0.0	0.0	0.0	0.0	0.0	0.0	0.0	0.0
0.0	0.0	0.0	0.0	0.0	0.0	0.0	0.0
0.0	0.0	0.0	0.0	0.0	0.0	0.0	0.0
0.0	0.0	0.0	0.0	0.0	0.0	0.0	0.0
0.0	0.0	0.0	0.0	0.0	0.0	0.0	0.0
0.0	0.0	0.0	0.0	0.0	0.0	0.0	0.0
0.0	0.0	0.0	0.0	0.0	0.0	0.0	0.0
0.0	0.0	0.0	0.0	0.0	0.0	0.0	0.0
0.0	0.0	0.0	0.0	0.0	0.0	0.0	0.0
0.0	0.0	0.0	0.0	0.0	0.0	0.0	0.0

0.0	0.0	0.0	0.0	0.0	0.0	0.0	0.0
0.0	0.0	0.0	0.0	0.0	0.0	0.0	0.0
0.0	0.0	0.0	0.0	0.0	0.0	0.0	0.0
0.0	0.0	0.0	0.0	0.0	0.0	0.0	0.0
0.0	0.0	0.0	0.0	0.0	0.0	0.0	0.0
0.0	0.0	0.0	0.0	0.0	0.0	0.0	0.0
0.0	0.0	0.0	0.0	0.0	0.0	0.0	0.0
0.0	0.0	0.0	0.0	0.0	0.0	0.0	0.0
0.0	0.0	0.0	0.0	0.0	0.0	0.0	0.0
0.0	0.0	0.0	0.0	0.0	0.0	0.0	0.0
0.0	0.0	0.0	0.0	0.0	0.0	0.0	0.0
0.0	0.0	0.0	0.0	0.0	0.0	0.0	0.0
0.0	0.0	0.0	0.0	0.0	0.0	0.0	0.0
0.0	0.0	0.0	0.0	0.0	0.0	0.0	0.0
0.0	0.0	0.0	0.0	0.0	0.0	0.0	0.0
0.0	0.0	0.0	0.0	0.0	0.0	0.0	0.0
0.0	0.0	0.0	0.0	0.0	0.0	0.0	0.0
0.0	0.0	0.0	0.0	0.0	0.0	0.0	0.0
0.0	0.0	0.0	0.0	0.0	0.0	0.0	0.0
0.0	0.0	0.0	0.0	0.0	0.0	0.0	0.0
0.0	0.0	0.0	0.0	0.0	0.0	0.0	0.0
0.0	0.0	0.0	0.0	0.0	0.0	0.0	0.0
0.0	0.0	0.0	0.0	0.0	0.0	0.0	0.0
0.0	0.0	0.0	0.0	0.0	0.0	0.0	0.0
0.0	0.0	0.0	0.0	0.0	0.0	0.0	0.0
0.0	0.0	0.0	0.0	0.0	0.0	0.0	0.0
0.0	0.0	0.0	0.0	0.0	0.0	0.0	0.0
0.0	0.0	0.0	0.0	0.0	0.0	0.0	0.0
0.0	0.0	0.0	0.0	0.0	0.0	0.0	0.0
0.0	0.0	0.0	0.0	0.0	0.0	0.0	0.0
0.0	0.0	0.0	0.0	0.0	0.0	0.0	0.0
0.0	0.0	0.0	0.0	0.0	0.0	0.0	0.0
0.0	0.0	0.0	0.0	0.0	0.0	0.0	0.0
0.0	0.0	0.0	0.0	0.0	0.0	0.0	0.0
0.0	0.0	0.0	0.0	0.0	0.0	0.0	0.0
0.0	0.0	0.0	0.0	0.0	0.0	0.0	0.0
0.0	0.0	0.0	0.0	0.0	0.0	0.0	0.0
0.0	0.0	0.0	0.0	0.0	0.0	0.0	0.0
0.0	0.0	0.0	0.0	0.0	0.0	0.0	0.0
0.0	0.0	0.0	0.0	0.0	0.0	0.0	0.0
0.0	0.0	0.0	0.0	0.0	0.0	0.0	0.0
0.0	0.0	0.0	0.0	0.0	0.0	0.0	0.0
0.0	0.0	0.0	0.0	0.0	0.0	0.0	0.0
0.0	0.0	0.0	0.0	0.0	0.0	0.0	0.0
0.0	0.0	0.0	0.0	0.0	0.0	0.0	0.0
0.0	0.0	0.0	0.0	0.0	0.0	0.0	0.0
0.0	0.0	0.0	0.0	0.0	0.0	0.0	0.0
0.0	0.0	0.0	0.0	0.0	0.0	0.0	0.0

0.0	0.0	0.0	0.0	0.0	0.0	0.0	0.0
0.0	0.0	0.0	0.0	0.0	0.0	0.0	0.0
0.0	0.0	0.0	0.0	0.0	0.0	0.0	0.0
0.0	0.0	0.0	0.0	0.0	0.0	0.0	0.0
0.0	0.0	0.0	0.0	0.0	0.0	0.0	0.0
0.0	0.0	0.0	0.0	0.0	0.0	0.0	0.0
0.0	0.0	0.0	0.0	0.0	0.0	0.0	0.0
0.0	0.0	0.0	0.0	0.0	0.0	0.0	0.0
0.0	0.0	0.0	0.0	0.0	0.0	0.0	0.0
0.0	0.0	0.0	0.0	0.0	0.0	0.0	0.0
0.0	0.0	0.0	0.0	0.0	0.0	0.0	0.0
0.0	0.0	0.0	0.0	0.0	0.0	0.0	0.0
0.0	0.0	0.0	0.0	0.0	0.0	0.0	0.0
0.0	0.0	0.0	0.0	0.0	0.0	0.0	0.0
0.0	0.0	0.0	0.0	0.0	0.0	0.0	0.0
0.0	0.0	0.0	0.0	0.0	0.0	0.0	0.0
0.0	0.0	0.0	0.0	0.0	0.0	0.0	0.0
0.0	0.0	0.0	0.0	0.0	0.0	0.0	0.0
0.0	0.0	0.0	0.0	0.0	0.0	0.0	0.0
0.0	0.0	0.0	0.0	0.0	0.0	0.0	0.0
0.0	0.0	0.0	0.0	0.0	0.0	0.0	0.0
0.0	0.0	0.0	0.0	0.0	0.0	0.0	0.0
0.0	0.0	0.0	0.0	0.0	0.0	0.0	0.0
0.0	0.0	0.0	0.0	0.0	0.0	0.0	0.0
0.0	0.0	0.0	0.0	0.0	0.0	0.0	0.0
0.0	0.0	0.0	0.0	0.0	0.0	0.0	0.0
0.0	0.0	0.0	0.0	0.0	0.0	0.0	0.0
0.0	0.0	0.0	0.0	0.0	0.0	0.0	0.0
0.0	0.0	0.0	0.0	0.0	0.0	0.0	0.0
0.0	0.0	0.0	0.0	0.0	0.0	0.0	0.0
0.0	0.0	0.0	0.0	0.0	0.0	0.0	0.0
0.0	0.0	0.0	0.0	0.0	0.0	0.0	0.0
0.0	0.0	0.0	0.0	0.0	0.0	0.0	0.0
0.0	0.0	0.0	0.0	0.0	0.0	0.0	0.0
0.0	0.0	0.0	0.0	0.0	0.0	0.0	0.0
0.0	0.0	0.0	0.0	0.0	0.0	0.0	0.0
0.0	0.0	0.0	0.0	0.0	0.0	0.0	0.0
0.0	0.0	0.0	0.0	0.0	0.0	0.0	0.0
0.0	0.0	0.0	0.0	0.0	0.0	0.0	0.0
0.0	0.0	0.0	0.0	0.0	0.0	0.0	0.0
0.0	0.0	0.0	0.0	0.0	0.0	0.0	0.0
0.0	0.0	0.0	0.0	0.0	0.0	0.0	0.0
0.0	0.0	0.0	0.0	0.0	0.0	0.0	0.0
0.0	0.0	0.0	0.0	0.0	0.0	0.0	0.0
0.0	0.0	0.0	0.0	0.0	0.0	0.0	0.0
0.0	0.0	0.0	0.0	0.0	0.0	0.0	0.0
0.0	0.0	0.0	0.0	0.0	0.0	0.0	0.0
0.0	0.0	0.0	0.0	0.0	0.0	0.0	0.0

0.0	0.0	0.0	0.0	0.0	0.0	0.0	0.0
0.0	0.0	0.0	0.0	0.0	0.0	0.0	0.0
0.0	0.0	0.0	0.0	0.0	0.0	0.0	0.0
0.0	0.0	0.0	0.0	0.0	0.0	0.0	0.0
0.0	0.0	0.0	0.0	0.0	0.0	0.0	0.0
0.0	0.0	0.0	0.0	0.0	0.0	0.0	0.0
0.0	0.0	0.0	0.0	0.0	0.0	0.0	0.0
0.0	0.0	0.0	0.0	0.0	0.0	0.0	0.0
0.0	0.0	0.0	0.0	0.0	0.0	0.0	0.0
0.0	0.0	0.0	0.0	0.0	0.0	0.0	0.0
0.0	0.0	0.0	0.0	0.0	0.0	0.0	0.0
0.0	0.0	0.0	0.0	0.0	0.0	0.0	0.0
0.0	0.0	0.0	0.0	0.0	0.0	0.0	0.0
0.0	0.0	0.0	0.0	0.0	0.0	0.0	0.0
0.0	0.0	0.0	0.0	0.0	0.0	0.0	0.0
0.0	0.0	0.0	0.0	0.0	0.0	0.0	0.0
0.0	0.0	0.0	0.0	0.0	0.0	0.0	0.0
0.0	0.0	0.0	0.0	0.0	0.0	0.0	0.0
0.0	0.0	0.0	0.0	0.0	0.0	0.0	0.0
0.0	0.0	0.0	0.0	0.0	0.0	0.0	0.0
0.0	0.0	0.0	0.0	0.0	0.0	0.0	0.0
0.0	0.0	0.0	0.0	0.0	0.0	0.0	0.0
0.0	0.0	0.0	0.0	0.0	0.0	0.0	0.0
0.0	0.0	0.0	0.0	0.0	0.0	0.0	0.0
0.0	0.0	0.0	0.0	0.0	0.0	0.0	0.0
0.0	0.0	0.0	0.0	0.0	0.0	0.0	0.0
0.0	0.0	0.0	0.0	0.0	0.0	0.0	0.0
0.0	0.0	0.0	0.0	0.0	0.0	0.0	0.0
0.0	0.0	0.0	0.0	0.0	0.0	0.0	0.0
0.0	0.0	0.0	0.0	0.0	0.0	0.0	0.0
0.0	0.0	0.0	0.0	0.0	0.0	0.0	0.0
0.0	0.0	0.0	0.0	0.0	0.0	0.0	0.0
0.0	0.0	0.0	0.0	0.0	0.0	0.0	0.0
0.0	0.0	0.0	0.0	0.0	0.0	0.0	0.0
0.0	0.0	0.0	0.0	0.0	0.0	0.0	0.0
0.0	0.0	0.0	0.0	0.0	0.0	0.0	0.0
0.0	0.0	0.0	0.0	0.0	0.0	0.0	0.0
0.0	0.0	0.0	0.0	0.0	0.0	0.0	0.0
0.0	0.0	0.0	0.0	0.0	0.0	0.0	0.0
0.0	0.0	0.0	0.0	0.0	0.0	0.0	0.0
0.0	0.0	0.0	0.0	0.0	0.0	0.0	0.0
0.0	0.0	0.0	0.0	0.0	0.0	0.0	0.0
0.0	0.0	0.0	0.0	0.0	0.0	0.0	0.0
0.0	0.0	0.0	0.0	0.0	0.0	0.0	0.0
0.0	0.0	0.0	0.0	0.0	0.0	0.0	0.0
0.0	0.0	0.0	0.0	0.0	0.0	0.0	0.0
0.0	0.0	0.0	0.0	0.0	0.0	0.0	0.0
0.0	0.0	0.0	0.0	0.0	0.0	0.0	0.0

0.0	0.0	0.0	0.0	0.0	0.0	0.0	0.0
0.0	0.0	0.0	0.0	0.0	0.0	0.0	0.0
0.0	0.0	0.0	0.0	0.0	0.0	0.0	0.0
0.0	0.0	0.0	0.0	0.0	0.0	0.0	0.0
0.0	0.0	0.0	0.0	0.0	0.0	0.0	0.0
0.0	0.0	0.0	0.0	0.0	0.0	0.0	0.0
0.0	0.0	0.0	0.0	0.0	0.0	0.0	0.0
0.0	0.0	0.0	0.0	0.0	0.0	0.0	0.0
0.0	0.0	0.0	0.0	0.0	0.0	0.0	0.0
0.0	0.0	0.0	0.0	0.0	0.0	0.0	0.0
0.0	0.0	0.0	0.0	0.0	0.0	0.0	0.0
0.0	0.0	0.0	0.0	0.0	0.0	0.0	0.0
0.0	0.0	0.0	0.0	0.0	0.0	0.0	0.0
0.0	0.0	0.0	0.0	0.0	0.0	0.0	0.0
0.0	0.0	0.0	0.0	0.0	0.0	0.0	0.0
0.0	0.0	0.0	0.0	0.0	0.0	0.0	0.0
0.0	0.0	0.0	0.0	0.0	0.0	0.0	0.0
0.0	0.0	0.0	0.0	0.0	0.0	0.0	0.0
0.0	0.0	0.0	0.0	0.0	0.0	0.0	0.0
0.0	0.0	0.0	0.0	0.0	0.0	0.0	0.0
0.0	0.0	0.0	0.0	0.0	0.0	0.0	0.0
0.0	0.0	0.0	0.0	0.0	0.0	0.0	0.0
0.0	0.0	0.0	0.0	0.0	0.0	0.0	0.0
0.0	0.0	0.0	0.0	0.0	0.0	0.0	0.0
0.0	0.0	0.0	0.0	0.0	0.0	0.0	0.0
0.0	0.0	0.0	0.0	0.0	0.0	0.0	0.0
0.0	0.0	0.0	0.0	0.0	0.0	0.0	0.0
0.0	0.0	0.0	0.0	0.0	0.0	0.0	0.0
0.0	0.0	0.0	0.0	0.0	0.0	0.0	0.0
0.0	0.0	0.0	0.0	0.0	0.0	0.0	0.0
0.0	0.0	0.0	0.0	0.0	0.0	0.0	0.0
0.0	0.0	0.0	0.0	0.0	0.0	0.0	0.0
0.0	0.0	0.0	0.0	0.0	0.0	0.0	0.0
0.0	0.0	0.0	0.0	0.0	0.0	0.0	0.0
0.0	0.0	0.0	0.0	0.0	0.0	0.0	0.0
0.0	0.0	0.0	0.0	0.0	0.0	0.0	0.0
0.0	0.0	0.0	0.0	0.0	0.0	0.0	0.0
0.0	0.0	0.0	0.0	0.0	0.0	0.0	0.0
0.0	0.0	0.0	0.0	0.0	0.0	0.0	0.0
0.0	0.0	0.0	0.0	0.0	0.0	0.0	0.0
0.0	0.0	0.0	0.0	0.0	0.0	0.0	0.0
0.0	0.0	0.0	0.0	0.0	0.0	0.0	0.0
0.0	0.0	0.0	0.0	0.0	0.0	0.0	0.0
0.0	0.0	0.0	0.0	0.0	0.0	0.0	0.0
0.0	0.0	0.0	0.0	0.0	0.0	0.0	0.0
0.0	0.0	0.0	0.0	0.0	0.0	0.0	0.0
0.0	0.0	0.0	0.0	0.0	0.0	0.0	0.0

0.0	0.0	0.0	0.0	0.0	0.0	0.0	0.0
0.0	0.0	0.0	0.0	0.0	0.0	0.0	0.0
0.0	0.0	0.0	0.0	0.0	0.0	0.0	0.0
0.0	0.0	0.0	0.0	0.0	0.0	0.0	0.0
0.0	0.0	0.0	0.0	0.0	0.0	0.0	0.0
0.0	0.0	0.0	0.0	0.0	0.0	0.0	0.0
0.0	0.0	0.0	0.0	0.0	0.0	0.0	0.0
0.0	0.0	0.0	0.0	0.0	0.0	0.0	0.0
0.0	0.0	0.0	0.0	0.0	0.0	0.0	0.0
0.0	0.0	0.0	0.0	0.0	0.0	0.0	0.0
0.0	0.0	0.0	0.0	0.0	0.0	0.0	0.0
0.0	0.0	0.0	0.0	0.0	0.0	0.0	0.0
0.0	0.0	0.0	0.0	0.0	0.0	0.0	0.0
0.0	0.0	0.0	0.0	0.0	0.0	0.0	0.0
0.0	0.0	0.0	0.0	0.0	0.0	0.0	0.0
0.0	0.0	0.0	0.0	0.0	0.0	0.0	0.0
0.0	0.0	0.0	0.0	0.0	0.0	0.0	0.0
0.0	0.0	0.0	0.0	0.0	0.0	0.0	0.0
0.0	0.0	0.0	0.0	0.0	0.0	0.0	0.0
0.0	0.0	0.0	0.0	0.0	0.0	0.0	0.0
0.0	0.0	0.0	0.0	0.0	0.0	0.0	0.0
0.0	0.0	0.0	0.0	0.0	0.0	0.0	0.0
0.0	0.0	0.0	0.0	0.0	0.0	0.0	0.0
0.0	0.0	0.0	0.0	0.0	0.0	0.0	0.0
0.0	0.0	0.0	0.0	0.0	0.0	0.0	0.0
0.0	0.0	0.0	0.0	0.0	0.0	0.0	0.0
0.0	0.0	0.0	0.0	0.0	0.0	0.0	0.0
0.0	0.0	0.0	0.0	0.0	0.0	0.0	0.0
0.0	0.0	0.0	0.0	0.0	0.0	0.0	0.0
0.0	0.0	0.0	0.0	0.0	0.0	0.0	0.0
0.0	0.0	0.0	0.0	0.0	0.0	0.0	0.0
0.0	0.0	0.0	0.0	0.0	0.0	0.0	0.0
0.0	0.0	0.0	0.0	0.0	0.0	0.0	0.0
0.0	0.0	0.0	0.0	0.0	0.0	0.0	0.0
0.0	0.0	0.0	0.0	0.0	0.0	0.0	0.0
0.0	0.0	0.0	0.0	0.0	0.0	0.0	0.0
0.0	0.0	0.0	0.0	0.0	0.0	0.0	0.0
0.0	0.0	0.0	0.0	0.0	0.0	0.0	0.0
0.0	0.0	0.0	0.0	0.0	0.0	0.0	0.0
0.0	0.0	0.0	0.0	0.0	0.0	0.0	0.0
0.0	0.0	0.0	0.0	0.0	0.0	0.0	0.0
0.0	0.0	0.0	0.0	0.0	0.0	0.0	0.0
0.0	0.0	0.0	0.0	0.0	0.0	0.0	0.0
0.0	0.0	0.0	0.0	0.0	0.0	0.0	0.0
0.0	0.0	0.0	0.0	0.0	0.0	0.0	0.0
0.0	0.0	0.0	0.0	0.0	0.0	0.0	0.0
0.0	0.0	0.0	0.0	0.0	0.0	0.0	0.0

0.0	0.0	0.0	0.0	0.0	0.0	0.0	0.0
0.0	0.0	0.0	0.0	0.0	0.0	0.0	0.0
0.0	0.0	0.0	0.0	0.0	0.0	0.0	0.0
0.0	0.0	0.0	0.0	0.0	0.0	0.0	0.0
0.0	0.0	0.0	0.0	0.0	0.0	0.0	0.0
0.0	0.0	0.0	0.0	0.0	0.0	0.0	0.0
0.0	0.0	0.0	0.0	0.0	0.0	0.0	0.0
0.0	0.0	0.0	0.0	0.0	0.0	0.0	0.0
0.0	0.0	0.0	0.0	0.0	0.0	0.0	0.0
0.0	0.0	0.0	0.0	0.0	0.0	0.0	0.0
0.0	0.0	0.0	0.0	0.0	0.0	0.0	0.0
0.0	0.0	0.0	0.0	0.0	0.0	0.0	0.0
0.0	0.0	0.0	0.0	0.0	0.0	0.0	0.0
0.0	0.0	0.0	0.0	0.0	0.0	0.0	0.0
0.0	0.0	0.0	0.0	0.0	0.0	0.0	0.0
0.0	0.0	0.0	0.0	0.0	0.0	0.0	0.0
0.0	0.0	0.0	0.0	0.0	0.0	0.0	0.0
0.0	0.0	0.0	0.0	0.0	0.0	0.0	0.0
0.0	0.0	0.0	0.0	0.0	0.0	0.0	0.0
0.0	0.0	0.0	0.0	0.0	0.0	0.0	0.0
0.0	0.0	0.0	0.0	0.0	0.0	0.0	0.0
0.0	0.0	0.0	0.0	0.0	0.0	0.0	0.0
0.0	0.0	0.0	0.0	0.0	0.0	0.0	0.0
0.0	0.0	0.0	0.0	0.0	0.0	0.0	0.0
0.0	0.0	0.0	0.0	0.0	0.0	0.0	0.0
0.0	0.0	0.0	0.0	0.0	0.0	0.0	0.0
0.0	0.0	0.0	0.0	0.0	0.0	0.0	0.0
0.0	0.0	0.0	0.0	0.0	0.0	0.0	0.0
0.0	0.0	0.0	0.0	0.0	0.0	0.0	0.0
0.0	0.0	0.0	0.0	0.0	0.0	0.0	0.0
0.0	0.0	0.0	0.0	0.0	0.0	0.0	0.0
0.0	0.0	0.0	0.0	0.0	0.0	0.0	0.0
0.0	0.0	0.0	0.0	0.0	0.0	0.0	0.0
0.0	0.0	0.0	0.0	0.0	0.0	0.0	0.0
0.0	0.0	0.0	0.0	0.0	0.0	0.0	0.0
0.0	0.0	0.0	0.0	0.0	0.0	0.0	0.0
0.0	0.0	0.0	0.0	0.0	0.0	0.0	0.0
0.0	0.0	0.0	0.0	0.0	0.0	0.0	0.0
0.0	0.0	0.0	0.0	0.0	0.0	0.0	0.0
0.0	0.0	0.0	0.0	0.0	0.0	0.0	0.0
0.0	0.0	0.0	0.0	0.0	0.0	0.0	0.0
0.0	0.0	0.0	0.0	0.0	0.0	0.0	0.0
0.0	0.0	0.0	0.0	0.0	0.0	0.0	0.0
0.0	0.0	0.0	0.0	0.0	0.0	0.0	0.0
0.0	0.0	0.0	0.0	0.0	0.0	0.0	0.0
0.0	0.0	0.0	0.0	0.0	0.0	0.0	0.0
0.0	0.0	0.0	0.0	0.0	0.0	0.0	0.0

0.0	0.0	0.0	0.0	0.0	0.0	0.0	0.0
0.0	0.0	0.0	0.0	0.0	0.0	0.0	0.0
0.0	0.0	0.0	0.0	0.0	0.0	0.0	0.0
0.0	0.0	0.0	0.0	0.0	0.0	0.0	0.0
0.0	0.0	0.0	0.0	0.0	0.0	0.0	0.0
0.0	0.0	0.0	0.0	0.0	0.0	0.0	0.0
0.0	0.0	0.0	0.0	0.0	0.0	0.0	0.0
0.0	0.0	0.0	0.0	0.0	0.0	0.0	0.0
0.0	0.0	0.0	0.0	0.0	0.0	0.0	0.0
0.0	0.0	0.0	0.0	0.0	0.0	0.0	0.0
0.0	0.0	0.0	0.0	0.0	0.0	0.0	0.0
0.0	0.0	0.0	0.0	0.0	0.0	0.0	0.0
0.0	0.0	0.0	0.0	0.0	0.0	0.0	0.0
0.0	0.0	0.0	0.0	0.0	0.0	0.0	0.0
0.0	0.0	0.0	0.0	0.0	0.0	0.0	0.0
0.0	0.0	0.0	0.0	0.0	0.0	0.0	0.0
0.0	0.0	0.0	0.0	0.0	0.0	0.0	0.0
0.0	0.0	0.0	0.0	0.0	0.0	0.0	0.0
0.0	0.0	0.0	0.0	0.0	0.0	0.0	0.0
0.0	0.0	0.0	0.0	0.0	0.0	0.0	0.0
0.0	0.0	0.0	0.0	0.0	0.0	0.0	0.0
0.0	0.0	0.0	0.0	0.0	0.0	0.0	0.0
0.0	0.0	0.0	0.0	0.0	0.0	0.0	0.0
0.0	0.0	0.0	0.0	0.0	0.0	0.0	0.0
0.0	0.0	0.0	0.0	0.0	0.0	0.0	0.0
0.0	0.0	0.0	0.0	0.0	0.0	0.0	0.0
0.0	0.0	0.0	0.0	0.0	0.0	0.0	0.0
0.0	0.0	0.0	0.0	0.0	0.0	0.0	0.0
0.0	0.0	0.0	0.0	0.0	0.0	0.0	0.0
0.0	0.0	0.0	0.0	0.0	0.0	0.0	0.0
0.0	0.0	0.0	0.0	0.0	0.0	0.0	0.0
0.0	0.0	0.0	0.0	0.0	0.0	0.0	0.0
0.0	0.0	0.0	0.0	0.0	0.0	0.0	0.0
0.0	0.0	0.0	0.0	0.0	0.0	0.0	0.0
0.0	0.0	0.0	0.0	0.0	0.0	0.0	0.0
0.0	0.0	0.0	0.0	0.0	0.0	0.0	0.0
0.0	0.0	0.0	0.0	0.0	0.0	0.0	0.0
0.0	0.0	0.0	0.0	0.0	0.0	0.0	0.0
0.0	0.0	0.0	0.0	0.0	0.0	0.0	0.0
0.0	0.0	0.0	0.0	0.0	0.0	0.0	0.0
0.0	0.0	0.0	0.0	0.0	0.0	0.0	0.0
0.0	0.0	0.0	0.0	0.0	0.0	0.0	0.0
0.0	0.0	0.0	0.0	0.0	0.0	0.0	0.0
0.0	0.0	0.0	0.0	0.0	0.0	0.0	0.0
0.0	0.0	0.0	0.0	0.0	0.0	0.0	0.0
0.0	0.0	0.0	0.0	0.0	0.0	0.0	0.0
0.0	0.0	0.0	0.0	0.0	0.0	0.0	0.0

0.0	0.0	0.0	0.0	0.0	0.0	0.0	0.0
0.0	0.0	0.0	0.0	0.0	0.0	0.0	0.0
0.0	0.0	0.0	0.0	0.0	0.0	0.0	0.0
0.0	0.0	0.0	0.0	0.0	0.0	0.0	0.0
0.0	0.0	0.0	0.0	0.0	0.0	0.0	0.0
0.0	0.0	0.0	0.0	0.0	0.0	0.0	0.0
0.0	0.0	0.0	0.0	0.0	0.0	0.0	0.0
0.0	0.0	0.0	0.0	0.0	0.0	0.0	0.0
0.0	0.0	0.0	0.0	0.0	0.0	0.0	0.0
0.0	0.0	0.0	0.0	0.0	0.0	0.0	0.0
0.0	0.0	0.0	0.0	0.0	0.0	0.0	0.0
0.0	0.0	0.0	0.0	0.0	0.0	0.0	0.0
0.0	0.0	0.0	0.0	0.0	0.0	0.0	0.0
0.0	0.0	0.0	0.0	0.0	0.0	0.0	0.0
0.0	0.0	0.0	0.0	0.0	0.0	0.0	0.0
0.0	0.0	0.0	0.0	0.0	0.0	0.0	0.0
0.0	0.0	0.0	0.0	0.0	0.0	0.0	0.0
0.0	0.0	0.0	0.0	0.0	0.0	0.0	0.0
0.0	0.0	0.0	0.0	0.0	0.0	0.0	0.0
0.0	0.0	0.0	0.0	0.0	0.0	0.0	0.0
0.0	0.0	0.0	0.0	0.0	0.0	0.0	0.0
0.0	0.0	0.0	0.0	0.0	0.0	0.0	0.0
0.0	0.0	0.0	0.0	0.0	0.0	0.0	0.0
0.0	0.0	0.0	0.0	0.0	0.0	0.0	0.0
0.0	0.0	0.0	0.0	0.0	0.0	0.0	0.0
0.0	0.0	0.0	0.0	0.0	0.0	0.0	0.0
0.0	0.0	0.0	0.0	0.0	0.0	0.0	0.0
0.0	0.0	0.0	0.0	0.0	0.0	0.0	0.0
0.0	0.0	0.0	0.0	0.0	0.0	0.0	0.0
0.0	0.0	0.0	0.0	0.0	0.0	0.0	0.0
0.0	0.0	0.0	0.0	0.0	0.0	0.0	0.0
0.0	0.0	0.0	0.0	0.0	0.0	0.0	0.0
0.0	0.0	0.0	0.0	0.0	0.0	0.0	0.0
0.0	0.0	0.0	0.0	0.0	0.0	0.0	0.0
0.0	0.0	0.0	0.0	0.0	0.0	0.0	0.0
0.0	0.0	0.0	0.0	0.0	0.0	0.0	0.0
0.0	0.0	0.0	0.0	0.0	0.0	0.0	0.0
0.0	0.0	0.0	0.0	0.0	0.0	0.0	0.0
0.0	0.0	0.0	0.0	0.0	0.0	0.0	0.0
0.0	0.0	0.0	0.0	0.0	0.0	0.0	0.0
0.0	0.0	0.0	0.0	0.0	0.0	0.0	0.0
0.0	0.0	0.0	0.0	0.0	0.0	0.0	0.0
0.0	0.0	0.0	0.0	0.0	0.0	0.0	0.0
0.0	0.0	0.0	0.0	0.0	0.0	0.0	0.0
0.0	0.0	0.0	0.0	0.0	0.0	0.0	0.0
0.0	0.0	0.0	0.0	0.0	0.0	0.0	0.0
0.0	0.0	0.0	0.0	0.0	0.0	0.0	0.0

0.0	0.0	0.0	0.0	0.0	0.0	0.0	0.0
0.0	0.0	0.0	0.0	0.0	0.0	0.0	0.0
0.0	0.0	0.0	0.0	0.0	0.0	0.0	0.0
0.0	0.0	0.0	0.0	0.0	0.0	0.0	0.0
0.0	0.0	0.0	0.0	0.0	0.0	0.0	0.0
0.0	0.0	0.0	0.0	0.0	0.0	0.0	0.0
0.0	0.0	0.0	0.0	0.0	0.0	0.0	0.0
0.0	0.0	0.0	0.0	0.0	0.0	0.0	0.0
0.0	0.0	0.0	0.0	0.0	0.0	0.0	0.0
0.0	0.0	0.0	0.0	0.0	0.0	0.0	0.0
0.0	0.0	0.0	0.0	0.0	0.0	0.0	0.0
0.0	0.0	0.0	0.0	0.0	0.0	0.0	0.0
0.0	0.0	0.0	0.0	0.0	0.0	0.0	0.0
0.0	0.0	0.0	0.0	0.0	0.0	0.0	0.0
0.0	0.0	0.0	0.0	0.0	0.0	0.0	0.0
0.0	0.0	0.0	0.0	0.0	0.0	0.0	0.0
0.0	0.0	0.0	0.0	0.0	0.0	0.0	0.0
0.0	0.0	0.0	0.0	0.0	0.0	0.0	0.0
0.0	0.0	0.0	0.0	0.0	0.0	0.0	0.0
0.0	0.0	0.0	0.0	0.0	0.0	0.0	0.0
0.0	0.0	0.0	0.0	0.0	0.0	0.0	0.0
0.0	0.0	0.0	0.0	0.0	0.0	0.0	0.0
0.0	0.0	0.0	0.0	0.0	0.0	0.0	0.0
0.0	0.0	0.0	0.0	0.0	0.0	0.0	0.0
0.0	0.0	0.0	0.0	0.0	0.0	0.0	0.0
0.0	0.0	0.0	0.0	0.0	0.0	0.0	0.0
0.0	0.0	0.0	0.0	0.0	0.0	0.0	0.0
0.0	0.0	0.0	0.0	0.0	0.0	0.0	0.0
0.0	0.0	0.0	0.0	0.0	0.0	0.0	0.0
0.0	0.0	0.0	0.0	0.0	0.0	0.0	0.0
0.0	0.0	0.0	0.0	0.0	0.0	0.0	0.0
0.0	0.0	0.0	0.0	0.0	0.0	0.0	0.0
0.0	0.0	0.0	0.0	0.0	0.0	0.0	0.0
0.0	0.0	0.0	0.0	0.0	0.0	0.0	0.0
0.0	0.0	0.0	0.0	0.0	0.0	0.0	0.0
0.0	0.0	0.0	0.0	0.0	0.0	0.0	0.0
0.0	0.0	0.0	0.0	0.0	0.0	0.0	0.0
0.0	0.0	0.0	0.0	0.0	0.0	0.0	0.0
0.0	0.0	0.0	0.0	0.0	0.0	0.0	0.0
0.0	0.0	0.0	0.0	0.0	0.0	0.0	0.0
0.0	0.0	0.0	0.0	0.0	0.0	0.0	0.0
0.0	0.0	0.0	0.0	0.0	0.0	0.0	0.0
0.0	0.0	0.0	0.0	0.0	0.0	0.0	0.0
0.0	0.0	0.0	0.0	0.0	0.0	0.0	0.0
0.0	0.0	0.0	0.0	0.0	0.0	0.0	0.0
0.0	0.0	0.0	0.0	0.0	0.0	0.0	0.0
0.0	0.0	0.0	0.0	0.0	0.0	0.0	0.0

0.0	0.0	0.0	0.0	0.0	0.0	0.0	0.0
0.0	0.0	0.0	0.0	0.0	0.0	0.0	0.0
0.0	0.0	0.0	0.0	0.0	0.0	0.0	0.0
0.0	0.0	0.0	0.0	0.0	0.0	0.0	0.0
0.0	0.0	0.0	0.0	0.0	0.0	0.0	0.0
0.0	0.0	0.0	0.0	0.0	0.0	0.0	0.0
0.0	0.0	0.0	0.0	0.0	0.0	0.0	0.0
0.0	0.0	0.0	0.0	0.0	0.0	0.0	0.0
0.0	0.0	0.0	0.0	0.0	0.0	0.0	0.0
0.0	0.0	0.0	0.0	0.0	0.0	0.0	0.0
0.0	0.0	0.0	0.0	0.0	0.0	0.0	0.0
0.0	0.0	0.0	0.0	0.0	0.0	0.0	0.0
0.0	0.0	0.0	0.0	0.0	0.0	0.0	0.0
0.0	0.0	0.0	0.0	0.0	0.0	0.0	0.0
0.0	0.0	0.0	0.0	0.0	0.0	0.0	0.0
0.0	0.0	0.0	0.0	0.0	0.0	0.0	0.0
0.0	0.0	0.0	0.0	0.0	0.0	0.0	0.0
0.0	0.0	0.0	0.0	0.0	0.0	0.0	0.0
0.0	0.0	0.0	0.0	0.0	0.0	0.0	0.0
0.0	0.0	0.0	0.0	0.0	0.0	0.0	0.0
0.0	0.0	0.0	0.0	0.0	0.0	0.0	0.0
0.0	0.0	0.0	0.0	0.0	0.0	0.0	0.0
0.0	0.0	0.0	0.0	0.0	0.0	0.0	0.0
0.0	0.0	0.0	0.0	0.0	0.0	0.0	0.0
0.0	0.0	0.0	0.0	0.0	0.0	0.0	0.0
0.0	0.0	0.0	0.0	0.0	0.0	0.0	0.0
0.0	0.0	0.0	0.0	0.0	0.0	0.0	0.0
0.0	0.0	0.0	0.0	0.0	0.0	0.0	0.0
0.0	0.0	0.0	0.0	0.0	0.0	0.0	0.0
0.0	0.0	0.0	0.0	0.0	0.0	0.0	0.0
0.0	0.0	0.0	0.0	0.0	0.0	0.0	0.0
0.0	0.0	0.0	0.0	0.0	0.0	0.0	0.0
0.0	0.0	0.0	0.0	0.0	0.0	0.0	0.0
0.0	0.0	0.0	0.0	0.0	0.0	0.0	0.0
0.0	0.0	0.0	0.0	0.0	0.0	0.0	0.0
0.0	0.0	0.0	0.0	0.0	0.0	0.0	0.0
0.0	0.0	0.0	0.0	0.0	0.0	0.0	0.0
0.0	0.0	0.0	0.0	0.0	0.0	0.0	0.0
0.0	0.0	0.0	0.0	0.0	0.0	0.0	0.0
0.0	0.0	0.0	0.0	0.0	0.0	0.0	0.0
0.0	0.0	0.0	0.0	0.0	0.0	0.0	0.0
0.0	0.0	0.0	0.0	0.0	0.0	0.0	0.0
0.0	0.0	0.0	0.0	0.0	0.0	0.0	0.0
0.0	0.0	0.0	0.0	0.0	0.0	0.0	0.0
0.0	0.0	0.0	0.0	0.0	0.0	0.0	0.0
0.0	0.0	0.0	0.0	0.0	0.0	0.0	0.0
0.0	0.0	0.0	0.0	0.0	0.0	0.0	0.0

0.0	0.0	0.0	0.0	0.0	0.0	0.0	0.0
0.0	0.0	0.0	0.0	0.0	0.0	0.0	0.0
0.0	0.0	0.0	0.0	0.0	0.0	0.0	0.0
0.0	0.0	0.0	0.0	0.0	0.0	0.0	0.0
0.0	0.0	0.0	0.0	0.0	0.0	0.0	0.0
0.0	0.0	0.0	0.0	0.0	0.0	0.0	0.0
0.0	0.0	0.0	0.0	0.0	0.0	0.0	0.0
0.0	0.0	0.0	0.0	0.0	0.0	0.0	0.0
0.0	0.0	0.0	0.0	0.0	0.0	0.0	0.0
0.0	0.0	0.0	0.0	0.0	0.0	0.0	0.0
0.0	0.0	0.0	0.0	0.0	0.0	0.0	0.0
0.0	0.0	0.0	0.0	0.0	0.0	0.0	0.0
0.0	0.0	0.0	0.0	0.0	0.0	0.0	0.0
0.0	0.0	0.0	0.0	0.0	0.0	0.0	0.0
0.0	0.0	0.0	0.0	0.0	0.0	0.0	0.0
0.0	0.0	0.0	0.0	0.0	0.0	0.0	0.0
0.0	0.0	0.0	0.0	0.0	0.0	0.0	0.0
0.0	0.0	0.0	0.0	0.0	0.0	0.0	0.0
0.0	0.0	0.0	0.0	0.0	0.0	0.0	0.0
0.0	0.0	0.0	0.0	0.0	0.0	0.0	0.0
0.0	0.0	0.0	0.0	0.0	0.0	0.0	0.0
0.0	0.0	0.0	0.0	0.0	0.0	0.0	0.0
0.0	0.0	0.0	0.0	0.0	0.0	0.0	0.0
0.0	0.0	0.0	0.0	0.0	0.0	0.0	0.0
0.0	0.0	0.0	0.0	0.0	0.0	0.0	0.0
0.0	0.0	0.0	0.0	0.0	0.0	0.0	0.0
0.0	0.0	0.0	0.0	0.0	0.0	0.0	0.0
0.0	0.0	0.0	0.0	0.0	0.0	0.0	0.0
0.0	0.0	0.0	0.0	0.0	0.0	0.0	0.0
0.0	0.0	0.0	0.0	0.0	0.0	0.0	0.0
0.0	0.0	0.0	0.0	0.0	0.0	0.0	0.0
0.0	0.0	0.0	0.0	0.0	0.0	0.0	0.0
0.0	0.0	0.0	0.0	0.0	0.0	0.0	0.0
0.0	0.0	0.0	0.0	0.0	0.0	0.0	0.0
0.0	0.0	0.0	0.0	0.0	0.0	0.0	0.0
0.0	0.0	0.0	0.0	0.0	0.0	0.0	0.0
0.0	0.0	0.0	0.0	0.0	0.0	0.0	0.0
0.0	0.0	0.0	0.0	0.0	0.0	0.0	0.0
0.0	0.0	0.0	0.0	0.0	0.0	0.0	0.0
0.0	0.0	0.0	0.0	0.0	0.0	0.0	0.0
0.0	0.0	0.0	0.0	0.0	0.0	0.0	0.0
0.0	0.0	0.0	0.0	0.0	0.0	0.0	0.0
0.0	0.0	0.0	0.0	0.0	0.0	0.0	0.0
0.0	0.0	0.0	0.0	0.0	0.0	0.0	0.0
0.0	0.0	0.0	0.0	0.0	0.0	0.0	0.0
0.0	0.0	0.0	0.0	0.0	0.0	0.0	0.0
0.0	0.0	0.0	0.0	0.0	0.0	0.0	0.0

0.0	0.0	0.0	0.0	0.0	0.0	0.0	0.0
0.0	0.0	0.0	0.0	0.0	0.0	0.0	0.0
0.0	0.0	0.0	0.0	0.0	0.0	0.0	0.0
0.0	0.0	0.0	0.0	0.0	0.0	0.0	0.0
0.0	0.0	0.0	0.0	0.0	0.0	0.0	0.0
0.0	0.0	0.0	0.0	0.0	0.0	0.0	0.0
0.0	0.0	0.0	0.0	0.0	0.0	0.0	0.0
0.0	0.0	0.0	0.0	0.0	0.0	0.0	0.0
0.0	0.0	0.0	0.0	0.0	0.0	0.0	0.0
0.0	0.0	0.0	0.0	0.0	0.0	0.0	0.0
0.0	0.0	0.0	0.0	0.0	0.0	0.0	0.0
0.0	0.0	0.0	0.0	0.0	0.0	0.0	0.0
0.0	0.0	0.0	0.0	0.0	0.0	0.0	0.0
0.0	0.0	0.0	0.0	0.0	0.0	0.0	0.0
0.0	0.0	0.0	0.0	0.0	0.0	0.0	0.0
0.0	0.0	0.0	0.0	0.0	0.0	0.0	0.0
0.0	0.0	0.0	0.0	0.0	0.0	0.0	0.0
0.0	0.0	0.0	0.0	0.0	0.0	0.0	0.0
0.0	0.0	0.0	0.0	0.0	0.0	0.0	0.0
0.0	0.0	0.0	0.0	0.0	0.0	0.0	0.0
0.0	0.0	0.0	0.0	0.0	0.0	0.0	0.0
0.0	0.0	0.0	0.0	0.0	0.0	0.0	0.0
0.0	0.0	0.0	0.0	0.0	0.0	0.0	0.0
0.0	0.0	0.0	0.0	0.0	0.0	0.0	0.0
0.0	0.0	0.0	0.0	0.0	0.0	0.0	0.0
0.0	0.0	0.0	0.0	0.0	0.0	0.0	0.0
0.0	0.0	0.0	0.0	0.0	0.0	0.0	0.0
0.0	0.0	0.0	0.0	0.0	0.0	0.0	0.0
0.0	0.0	0.0	0.0	0.0	0.0	0.0	0.0
0.0	0.0	0.0	0.0	0.0	0.0	0.0	0.0
0.0	0.0	0.0	0.0	0.0	0.0	0.0	0.0
0.0	0.0	0.0	0.0	0.0	0.0	0.0	0.0
0.0	0.0	0.0	0.0	0.0	0.0	0.0	0.0
0.0	0.0	0.0	0.0	0.0	0.0	0.0	0.0
0.0	0.0	0.0	0.0	0.0	0.0	0.0	0.0
0.0	0.0	0.0	0.0	0.0	0.0	0.0	0.0
0.0	0.0	0.0	0.0	0.0	0.0	0.0	0.0
0.0	0.0	0.0	0.0	0.0	0.0	0.0	0.0
0.0	0.0	0.0	0.0	0.0	0.0	0.0	0.0
0.0	0.0	0.0	0.0	0.0	0.0	0.0	0.0
0.0	0.0	0.0	0.0	0.0	0.0	0.0	0.0
0.0	0.0	0.0	0.0	0.0	0.0	0.0	0.0
0.0	0.0	0.0	0.0	0.0	0.0	0.0	0.0
0.0	0.0	0.0	0.0	0.0	0.0	0.0	0.0
0.0	0.0	0.0	0.0	0.0	0.0	0.0	0.0
0.0	0.0	0.0	0.0	0.0	0.0	0.0	0.0
0.0	0.0	0.0	0.0	0.0	0.0	0.0	0.0

0.0	0.0	0.0	0.0	0.0	0.0	0.0	0.0
0.0	0.0	0.0	0.0	0.0	0.0	0.0	0.0
0.0	0.0	0.0	0.0	0.0	0.0	0.0	0.0
0.0	0.0	0.0	0.0	0.0	0.0	0.0	0.0
0.0	0.0	0.0	0.0	0.0	0.0	0.0	0.0
0.0	0.0	0.0	0.0	0.0	0.0	0.0	0.0
0.0	0.0	0.0	0.0	0.0	0.0	0.0	0.0
0.0	0.0	0.0	0.0	0.0	0.0	0.0	0.0
0.0	0.0	0.0	0.0	0.0	0.0	0.0	0.0
0.0	0.0	0.0	0.0	0.0	0.0	0.0	0.0
0.0	0.0	0.0	0.0	0.0	0.0	0.0	0.0
0.0	0.0	0.0	0.0	0.0	0.0	0.0	0.0
0.0	0.0	0.0	0.0	0.0	0.0	0.0	0.0
0.0	0.0	0.0	0.0	0.0	0.0	0.0	0.0
0.0	0.0	0.0	0.0	0.0	0.0	0.0	0.0
0.0	0.0	0.0	0.0	0.0	0.0	0.0	0.0
0.0	0.0	0.0	0.0	0.0	0.0	0.0	0.0
0.0	0.0	0.0	0.0	0.0	0.0	0.0	0.0
0.0	0.0	0.0	0.0	0.0	0.0	0.0	0.0
0.0	0.0	0.0	0.0	0.0	0.0	0.0	0.0
0.0	0.0	0.0	0.0	0.0	0.0	0.0	0.0
0.0	0.0	0.0	0.0	0.0	0.0	0.0	0.0
0.0	0.0	0.0	0.0	0.0	0.0	0.0	0.0
0.0	0.0	0.0	0.0	0.0	0.0	0.0	0.0
0.0	0.0	0.0	0.0	0.0	0.0	0.0	0.0
0.0	0.0	0.0	0.0	0.0	0.0	0.0	0.0
0.0	0.0	0.0	0.0	0.0	0.0	0.0	0.0
0.0	0.0	0.0	0.0	0.0	0.0	0.0	0.0
0.0	0.0	0.0	0.0	0.0	0.0	0.0	0.0
0.0	0.0	0.0	0.0	0.0	0.0	0.0	0.0
0.0	0.0	0.0	0.0	0.0	0.0	0.0	0.0
0.0	0.0	0.0	0.0	0.0	0.0	0.0	0.0
0.0	0.0	0.0	0.0	0.0	0.0	0.0	0.0
0.0	0.0	0.0	0.0	0.0	0.0	0.0	0.0
0.0	0.0	0.0	0.0	0.0	0.0	0.0	0.0
0.0	0.0	0.0	0.0	0.0	0.0	0.0	0.0
0.0	0.0	0.0	0.0	0.0	0.0	0.0	0.0
0.0	0.0	0.0	0.0	0.0	0.0	0.0	0.0
0.0	0.0	0.0	0.0	0.0	0.0	0.0	0.0
0.0	0.0	0.0	0.0	0.0	0.0	0.0	0.0
0.0	0.0	0.0	0.0	0.0	0.0	0.0	0.0
0.0	0.0	0.0	0.0	0.0	0.0	0.0	0.0
0.0	0.0	0.0	0.0	0.0	0.0	0.0	0.0
0.0	0.0	0.0	0.0	0.0	0.0	0.0	0.0
0.0	0.0	0.0	0.0	0.0	0.0	0.0	0.0
0.0	0.0	0.0	0.0	0.0	0.0	0.0	0.0
0.0	0.0	0.0	0.0	0.0	0.0	0.0	0.0
0.0	0.0	0.0	0.0	0.0	0.0	0.0	0.0

0.0	0.0	0.0	0.0	0.0	0.0	0.0	0.0
0.0	0.0	0.0	0.0	0.0	0.0	0.0	0.0
0.0	0.0	0.0	0.0	0.0	0.0	0.0	0.0
0.0	0.0	0.0	0.0	0.0	0.0	0.0	0.0
0.0	0.0	0.0	0.0	0.0	0.0	0.0	0.0
0.0	0.0	0.0	0.0	0.0	0.0	0.0	0.0
0.0	0.0	0.0	0.0	0.0	0.0	0.0	0.0
0.0	0.0	0.0	0.0	0.0	0.0	0.0	0.0
0.0	0.0	0.0	0.0	0.0	0.0	0.0	0.0
0.0	0.0	0.0	0.0	0.0	0.0	0.0	0.0
0.0	0.0	0.0	0.0	0.0	0.0	0.0	0.0
0.0	0.0	0.0	0.0	0.0	0.0	0.0	0.0
0.0	0.0	0.0	0.0	0.0	0.0	0.0	0.0
0.0	0.0	0.0	0.0	0.0	0.0	0.0	0.0
0.0	0.0	0.0	0.0	0.0	0.0	0.0	0.0
0.0	0.0	0.0	0.0	0.0	0.0	0.0	0.0
0.0	0.0	0.0	0.0	0.0	0.0	0.0	0.0
0.0	0.0	0.0	0.0	0.0	0.0	0.0	0.0
0.0	0.0	0.0	0.0	0.0	0.0	0.0	0.0
0.0	0.0	0.0	0.0	0.0	0.0	0.0	0.0
0.0	0.0	0.0	0.0	0.0	0.0	0.0	0.0
0.0	0.0	0.0	0.0	0.0	0.0	0.0	0.0
0.0	0.0	0.0	0.0	0.0	0.0	0.0	0.0
0.0	0.0	0.0	0.0	0.0	0.0	0.0	0.0
0.0	0.0	0.0	0.0	0.0	0.0	0.0	0.0
0.0	0.0	0.0	0.0	0.0	0.0	0.0	0.0
0.0	0.0	0.0	0.0	0.0	0.0	0.0	0.0
0.0	0.0	0.0	0.0	0.0	0.0	0.0	0.0
0.0	0.0	0.0	0.0	0.0	0.0	0.0	0.0
0.0	0.0	0.0	0.0	0.0	0.0	0.0	0.0
0.0	0.0	0.0	0.0	0.0	0.0	0.0	0.0
0.0	0.0	0.0	0.0	0.0	0.0	0.0	0.0
0.0	0.0	0.0	0.0	0.0	0.0	0.0	0.0
0.0	0.0	0.0	0.0	0.0	0.0	0.0	0.0
0.0	0.0	0.0	0.0	0.0	0.0	0.0	0.0
0.0	0.0	0.0	0.0	0.0	0.0	0.0	0.0
0.0	0.0	0.0	0.0	0.0	0.0	0.0	0.0
0.0	0.0	0.0	0.0	0.0	0.0	0.0	0.0
0.0	0.0	0.0	0.0	0.0	0.0	0.0	0.0
0.0	0.0	0.0	0.0	0.0	0.0	0.0	0.0
0.0	0.0	0.0	0.0	0.0	0.0	0.0	0.0
0.0	0.0	0.0	0.0	0.0	0.0	0.0	0.0
0.0	0.0	0.0	0.0	0.0	0.0	0.0	0.0
0.0	0.0	0.0	0.0	0.0	0.0	0.0	0.0
0.0	0.0	0.0	0.0	0.0	0.0	0.0	0.0
0.0	0.0	0.0	0.0	0.0	0.0	0.0	0.0
0.0	0.0	0.0	0.0	0.0	0.0	0.0	0.0
0.0	0.0	0.0	0.0	0.0	0.0	0.0	0.0

0.0	0.0	0.0	0.0	0.0	0.0	0.0	0.0
0.0	0.0	0.0	0.0	0.0	0.0	0.0	0.0
0.0	0.0	0.0	0.0	0.0	0.0	0.0	0.0
0.0	0.0	0.0	0.0	0.0	0.0	0.0	0.0
0.0	0.0	0.0	0.0	0.0	0.0	0.0	0.0
0.0	0.0	0.0	0.0	0.0	0.0	0.0	0.0
0.0	0.0	0.0	0.0	0.0	0.0	0.0	0.0
0.0	0.0	0.0	0.0	0.0	0.0	0.0	0.0
0.0	0.0	0.0	0.0	0.0	0.0	0.0	0.0
0.0	0.0	0.0	0.0	0.0	0.0	0.0	0.0
0.0	0.0	0.0	0.0	0.0	0.0	0.0	0.0
0.0	0.0	0.0	0.0	0.0	0.0	0.0	0.0
0.0	0.0	0.0	0.0	0.0	0.0	0.0	0.0
0.0	0.0	0.0	0.0	0.0	0.0	0.0	0.0
0.0	0.0	0.0	0.0	0.0	0.0	0.0	0.0
0.0	0.0	0.0	0.0	0.0	0.0	0.0	0.0
0.0	0.0	0.0	0.0	0.0	0.0	0.0	0.0
0.0	0.0	0.0	0.0	0.0	0.0	0.0	0.0
0.0	0.0	0.0	0.0	0.0	0.0	0.0	0.0
0.0	0.0	0.0	0.0	0.0	0.0	0.0	0.0
0.0	0.0	0.0	0.0	0.0	0.0	0.0	0.0
0.0	0.0	0.0	0.0	0.0	0.0	0.0	0.0
0.0	0.0	0.0	0.0	0.0	0.0	0.0	0.0
0.0	0.0	0.0	0.0	0.0	0.0	0.0	0.0
0.0	0.0	0.0	0.0	0.0	0.0	0.0	0.0
0.0	0.0	0.0	0.0	0.0	0.0	0.0	0.0
0.0	0.0	0.0	0.0	0.0	0.0	0.0	0.0
0.0	0.0	0.0	0.0	0.0	0.0	0.0	0.0
0.0	0.0	0.0	0.0	0.0	0.0	0.0	0.0
0.0	0.0	0.0	0.0	0.0	0.0	0.0	0.0
0.0	0.0	0.0	0.0	0.0	0.0	0.0	0.0
0.0	0.0	0.0	0.0	0.0	0.0	0.0	0.0
0.0	0.0	0.0	0.0	0.0	0.0	0.0	0.0
0.0	0.0	0.0	0.0	0.0	0.0	0.0	0.0
0.0	0.0	0.0	0.0	0.0	0.0	0.0	0.0
0.0	0.0	0.0	0.0	0.0	0.0	0.0	0.0
0.0	0.0	0.0	0.0	0.0	0.0	0.0	0.0
0.0	0.0	0.0	0.0	0.0	0.0	0.0	0.0
0.0	0.0	0.0	0.0	0.0	0.0	0.0	0.0
0.0	0.0	0.0	0.0	0.0	0.0	0.0	0.0
0.0	0.0	0.0	0.0	0.0	0.0	0.0	0.0
0.0	0.0	0.0	0.0	0.0	0.0	0.0	0.0
0.0	0.0	0.0	0.0	0.0	0.0	0.0	0.0
0.0	0.0	0.0	0.0	0.0	0.0	0.0	0.0
0.0	0.0	0.0	0.0	0.0	0.0	0.0	0.0
0.0	0.0	0.0	0.0	0.0	0.0	0.0	0.0
0.0	0.0	0.0	0.0	0.0	0.0	0.0	0.0

0.0	0.0	0.0	0.0	0.0	0.0	0.0	0.0
0.0	0.0	0.0	0.0	0.0	0.0	0.0	0.0
0.0	0.0	0.0	0.0	0.0	0.0	0.0	0.0
0.0	0.0	0.0	0.0	0.0	0.0	0.0	0.0
0.0	0.0	0.0	0.0	0.0	0.0	0.0	0.0
0.0	0.0	0.0	0.0	0.0	0.0	0.0	0.0
0.0	0.0	0.0	0.0	0.0	0.0	0.0	0.0
0.0	0.0	0.0	0.0	0.0	0.0	0.0	0.0
0.0	0.0	0.0	0.0	0.0	0.0	0.0	0.0
0.0	0.0	0.0	0.0	0.0	0.0	0.0	0.0
0.0	0.0	0.0	0.0	0.0	0.0	0.0	0.0
0.0	0.0	0.0	0.0	0.0	0.0	0.0	0.0
0.0	0.0	0.0	0.0	0.0	0.0	0.0	0.0
0.0	0.0	0.0	0.0	0.0	0.0	0.0	0.0
0.0	0.0	0.0	0.0	0.0	0.0	0.0	0.0
0.0	0.0	0.0	0.0	0.0	0.0	0.0	0.0
0.0	0.0	0.0	0.0	0.0	0.0	0.0	0.0
0.0	0.0	0.0	0.0	0.0	0.0	0.0	0.0
0.0	0.0	0.0	0.0	0.0	0.0	0.0	0.0
0.0	0.0	0.0	0.0	0.0	0.0	0.0	0.0
0.0	0.0	0.0	0.0	0.0	0.0	0.0	0.0
0.0	0.0	0.0	0.0	0.0	0.0	0.0	0.0
0.0	0.0	0.0	0.0	0.0	0.0	0.0	0.0
0.0	0.0	0.0	0.0	0.0	0.0	0.0	0.0
0.0	0.0	0.0	0.0	0.0	0.0	0.0	0.0
0.0	0.0	0.0	0.0	0.0	0.0	0.0	0.0
0.0	0.0	0.0	0.0	0.0	0.0	0.0	0.0
0.0	0.0	0.0	0.0	0.0	0.0	0.0	0.0
0.0	0.0	0.0	0.0	0.0	0.0	0.0	0.0
0.0	0.0	0.0	0.0	0.0	0.0	0.0	0.0
0.0	0.0	0.0	0.0	0.0	0.0	0.0	0.0
0.0	0.0	0.0	0.0	0.0	0.0	0.0	0.0
0.0	0.0	0.0	0.0	0.0	0.0	0.0	0.0
0.0	0.0	0.0	0.0	0.0	0.0	0.0	0.0
0.0	0.0	0.0	0.0	0.0	0.0	0.0	0.0
0.0	0.0	0.0	0.0	0.0	0.0	0.0	0.0
0.0	0.0	0.0	0.0	0.0	0.0	0.0	0.0
0.0	0.0	0.0	0.0	0.0	0.0	0.0	0.0
0.0	0.0	0.0	0.0	0.0	0.0	0.0	0.0
0.0	0.0	0.0	0.0	0.0	0.0	0.0	0.0
0.0	0.0	0.0	0.0	0.0	0.0	0.0	0.0
0.0	0.0	0.0	0.0	0.0	0.0	0.0	0.0
0.0	0.0	0.0	0.0	0.0	0.0	0.0	0.0
0.0	0.0	0.0	0.0	0.0	0.0	0.0	0.0
0.0	0.0	0.0	0.0	0.0	0.0	0.0	0.0
0.0	0.0	0.0	0.0	0.0	0.0	0.0	0.0
0.0	0.0	0.0	0.0	0.0	0.0	0.0	0.0

0.0	0.0	0.0	0.0	0.0	0.0	0.0	0.0
0.0	0.0	0.0	0.0	0.0	0.0	0.0	0.0
0.0	0.0	0.0	0.0	0.0	0.0	0.0	0.0
0.0	0.0	0.0	0.0	0.0	0.0	0.0	0.0
0.0	0.0	0.0	0.0	0.0	0.0	0.0	0.0
0.0	0.0	0.0	0.0	0.0	0.0	0.0	0.0
0.0	0.0	0.0	0.0	0.0	0.0	0.0	0.0
0.0	0.0	0.0	0.0	0.0	0.0	0.0	0.0
0.0	0.0	0.0	0.0	0.0	0.0	0.0	0.0
0.0	0.0	0.0	0.0	0.0	0.0	0.0	0.0
0.0	0.0	0.0	0.0	0.0	0.0	0.0	0.0
0.0	0.0	0.0	0.0	0.0	0.0	0.0	0.0
0.0	0.0	0.0	0.0	0.0	0.0	0.0	0.0
0.0	0.0	0.0	0.0	0.0	0.0	0.0	0.0
0.0	0.0	0.0	0.0	0.0	0.0	0.0	0.0
0.0	0.0	0.0	0.0	0.0	0.0	0.0	0.0
0.0	0.0	0.0	0.0	0.0	0.0	0.0	0.0
0.0	0.0	0.0	0.0	0.0	0.0	0.0	0.0
0.0	0.0	0.0	0.0	0.0	0.0	0.0	0.0
0.0	0.0	0.0	0.0	0.0	0.0	0.0	0.0
0.0	0.0	0.0	0.0	0.0	0.0	0.0	0.0
0.0	0.0	0.0	0.0	0.0	0.0	0.0	0.0
0.0	0.0	0.0	0.0	0.0	0.0	0.0	0.0
0.0	0.0	0.0	0.0	0.0	0.0	0.0	0.0
0.0	0.0	0.0	0.0	0.0	0.0	0.0	0.0
0.0	0.0	0.0	0.0	0.0	0.0	0.0	0.0
0.0	0.0	0.0	0.0	0.0	0.0	0.0	0.0
0.0	0.0	0.0	0.0	0.0	0.0	0.0	0.0
0.0	0.0	0.0	0.0	0.0	0.0	0.0	0.0
0.0	0.0	0.0	0.0	0.0	0.0	0.0	0.0
0.0	0.0	0.0	0.0	0.0	0.0	0.0	0.0
0.0	0.0	0.0	0.0	0.0	0.0	0.0	0.0
0.0	0.0	0.0	0.0	0.0	0.0	0.0	0.0
0.0	0.0	0.0	0.0	0.0	0.0	0.0	0.0
0.0	0.0	0.0	0.0	0.0	0.0	0.0	0.0
0.0	0.0	0.0	0.0	0.0	0.0	0.0	0.0
0.0	0.0	0.0	0.0	0.0	0.0	0.0	0.0
0.0	0.0	0.0	0.0	0.0	0.0	0.0	0.0
0.0	0.0	0.0	0.0	0.0	0.0	0.0	0.0
0.0	0.0	0.0	0.0	0.0	0.0	0.0	0.0
0.0	0.0	0.0	0.0	0.0	0.0	0.0	0.0
0.0	0.0	0.0	0.0	0.0	0.0	0.0	0.0
0.0	0.0	0.0	0.0	0.0	0.0	0.0	0.0
0.0	0.0	0.0	0.0	0.0	0.0	0.0	0.0
0.0	0.0	0.0	0.0	0.0	0.0	0.0	0.0
0.0	0.0	0.0	0.0	0.0	0.0	0.0	0.0
0.0	0.0	0.0	0.0	0.0	0.0	0.0	0.0

0.0	0.0	0.0	0.0	0.0	0.0	0.0	0.0
0.0	0.0	0.0	0.0	0.0	0.0	0.0	0.0
0.0	0.0	0.0	0.0	0.0	0.0	0.0	0.0
0.0	0.0	0.0	0.0	0.0	0.0	0.0	0.0
0.0	0.0	0.0	0.0	0.0	0.0	0.0	0.0
0.0	0.0	0.0	0.0	0.0	0.0	0.0	0.0
0.0	0.0	0.0	0.0	0.0	0.0	0.0	0.0
0.0	0.0	0.0	0.0	0.0	0.0	0.0	0.0
0.0	0.0	0.0	0.0	0.0	0.0	0.0	0.0
0.0	0.0	0.0	0.0	0.0	0.0	0.0	0.0
0.0	0.0	0.0	0.0	0.0	0.0	0.0	0.0
0.0	0.0	0.0	0.0	0.0	0.0	0.0	0.0
0.0	0.0	0.0	0.0	0.0	0.0	0.0	0.0
0.0	0.0	0.0	0.0	0.0	0.0	0.0	0.0
0.0	0.0	0.0	0.0	0.0	0.0	0.0	0.0
0.0	0.0	0.0	0.0	0.0	0.0	0.0	0.0
0.0	0.0	0.0	0.0	0.0	0.0	0.0	0.0
0.0	0.0	0.0	0.0	0.0	0.0	0.0	0.0
0.0	0.0	0.0	0.0	0.0	0.0	0.0	0.0
0.0	0.0	0.0	0.0	0.0	0.0	0.0	0.0
0.0	0.0	0.0	0.0	0.0	0.0	0.0	0.0
0.0	0.0	0.0	0.0	0.0	0.0	0.0	0.0
0.0	0.0	0.0	0.0	0.0	0.0	0.0	0.0
0.0	0.0	0.0	0.0	0.0	0.0	0.0	0.0
0.0	0.0	0.0	0.0	0.0	0.0	0.0	0.0
0.0	0.0	0.0	0.0	0.0	0.0	0.0	0.0
0.0	0.0	0.0	0.0	0.0	0.0	0.0	0.0
0.0	0.0	0.0	0.0	0.0	0.0	0.0	0.0
0.0	0.0	0.0	0.0	0.0	0.0	0.0	0.0
0.0	0.0	0.0	0.0	0.0	0.0	0.0	0.0
0.0	0.0	0.0	0.0	0.0	0.0	0.0	0.0
0.0	0.0	0.0	0.0	0.0	0.0	0.0	0.0
0.0	0.0	0.0	0.0	0.0	0.0	0.0	0.0
0.0	0.0	0.0	0.0	0.0	0.0	0.0	0.0
0.0	0.0	0.0	0.0	0.0	0.0	0.0	0.0
0.0	0.0	0.0	0.0	0.0	0.0	0.0	0.0
0.0	0.0	0.0	0.0	0.0	0.0	0.0	0.0
0.0	0.0	0.0	0.0	0.0	0.0	0.0	0.0
0.0	0.0	0.0	0.0	0.0	0.0	0.0	0.0
0.0	0.0	0.0	0.0	0.0	0.0	0.0	0.0
0.0	0.0	0.0	0.0	0.0	0.0	0.0	0.0
0.0	0.0	0.0	0.0	0.0	0.0	0.0	0.0
0.0	0.0	0.0	0.0	0.0	0.0	0.0	0.0
0.0	0.0	0.0	0.0	0.0	0.0	0.0	0.0
0.0	0.0	0.0	0.0	0.0	0.0	0.0	0.0
0.0	0.0	0.0	0.0	0.0	0.0	0.0	0.0
0.0	0.0	0.0	0.0	0.0	0.0	0.0	0.0

0.0	0.0	0.0	0.0	0.0	0.0	0.0	0.0
0.0	0.0	0.0	0.0	0.0	0.0	0.0	0.0
0.0	0.0	0.0	0.0	0.0	0.0	0.0	0.0
0.0	0.0	0.0	0.0	0.0	0.0	0.0	0.0
0.0	0.0	0.0	0.0	0.0	0.0	0.0	0.0
0.0	0.0	0.0	0.0	0.0	0.0	0.0	0.0
0.0	0.0	0.0	0.0	0.0	0.0	0.0	0.0
0.0	0.0	0.0	0.0	0.0	0.0	0.0	0.0
0.0	0.0	0.0	0.0	0.0	0.0	0.0	0.0
0.0	0.0	0.0	0.0	0.0	0.0	0.0	0.0
0.0	0.0	0.0	0.0	0.0	0.0	0.0	0.0
0.0	0.0	0.0	0.0	0.0	0.0	0.0	0.0
0.0	0.0	0.0	0.0	0.0	0.0	0.0	0.0
0.0	0.0	0.0	0.0	0.0	0.0	0.0	0.0
0.0	0.0	0.0	0.0	0.0	0.0	0.0	0.0
0.0	0.0	0.0	0.0	0.0	0.0	0.0	0.0
0.0	0.0	0.0	0.0	0.0	0.0	0.0	0.0
0.0	0.0	0.0	0.0	0.0	0.0	0.0	0.0
0.0	0.0	0.0	0.0	0.0	0.0	0.0	0.0
0.0	0.0	0.0	0.0	0.0	0.0	0.0	0.0
0.0	0.0	0.0	0.0	0.0	0.0	0.0	0.0
0.0	0.0	0.0	0.0	0.0	0.0	0.0	0.0
0.0	0.0	0.0	0.0	0.0	0.0	0.0	0.0
0.0	0.0	0.0	0.0	0.0	0.0	0.0	0.0
0.0	0.0	0.0	0.0	0.0	0.0	0.0	0.0
0.0	0.0	0.0	0.0	0.0	0.0	0.0	0.0
0.0	0.0	0.0	0.0	0.0	0.0	0.0	0.0
0.0	0.0	0.0	0.0	0.0	0.0	0.0	0.0
0.0	0.0	0.0	0.0	0.0	0.0	0.0	0.0
0.0	0.0	0.0	0.0	0.0	0.0	0.0	0.0
0.0	0.0	0.0	0.0	0.0	0.0	0.0	0.0
0.0	0.0	0.0	0.0	0.0	0.0	0.0	0.0
0.0	0.0	0.0	0.0	0.0	0.0	0.0	0.0
0.0	0.0	0.0	0.0	0.0	0.0	0.0	0.0
0.0	0.0	0.0	0.0	0.0	0.0	0.0	0.0
0.0	0.0	0.0	0.0	0.0	0.0	0.0	0.0
0.0	0.0	0.0	0.0	0.0	0.0	0.0	0.0
0.0	0.0	0.0	0.0	0.0	0.0	0.0	0.0
0.0	0.0	0.0	0.0	0.0	0.0	0.0	0.0
0.0	0.0	0.0	0.0	0.0	0.0	0.0	0.0
0.0	0.0	0.0	0.0	0.0	0.0	0.0	0.0
0.0	0.0	0.0	0.0	0.0	0.0	0.0	0.0
0.0	0.0	0.0	0.0	0.0	0.0	0.0	0.0
0.0	0.0	0.0	0.0	0.0	0.0	0.0	0.0
0.0	0.0	0.0	0.0	0.0	0.0	0.0	0.0
0.0	0.0	0.0	0.0	0.0	0.0	0.0	0.0
0.0	0.0	0.0	0.0	0.0	0.0	0.0	0.0

0.0	0.0	0.0	0.0	0.0	0.0	0.0	0.0
0.0	0.0	0.0	0.0	0.0	0.0	0.0	0.0
0.0	0.0	0.0	0.0	0.0	0.0	0.0	0.0
0.0	0.0	0.0	0.0	0.0	0.0	0.0	0.0
0.0	0.0	0.0	0.0	0.0	0.0	0.0	0.0
0.0	0.0	0.0	0.0	0.0	0.0	0.0	0.0
0.0	0.0	0.0	0.0	0.0	0.0	0.0	0.0
0.0	0.0	0.0	0.0	0.0	0.0	0.0	0.0
0.0	0.0	0.0	0.0	0.0	0.0	0.0	0.0
0.0	0.0	0.0	0.0	0.0	0.0	0.0	0.0
0.0	0.0	0.0	0.0	0.0	0.0	0.0	0.0
0.0	0.0	0.0	0.0	0.0	0.0	0.0	0.0
0.0	0.0	0.0	0.0	0.0	0.0	0.0	0.0
0.0	0.0	0.0	0.0	0.0	0.0	0.0	0.0
0.0	0.0	0.0	0.0	0.0	0.0	0.0	0.0
0.0	0.0	0.0	0.0	0.0	0.0	0.0	0.0
0.0	0.0	0.0	0.0	0.0	0.0	0.0	0.0
0.0	0.0	0.0	0.0	0.0	0.0	0.0	0.0
0.0	0.0	0.0	0.0	0.0	0.0	0.0	0.0
0.0	0.0	0.0	0.0	0.0	0.0	0.0	0.0
0.0	0.0	0.0	0.0	0.0	0.0	0.0	0.0
0.0	0.0	0.0	0.0	0.0	0.0	0.0	0.0
0.0	0.0	0.0	0.0	0.0	0.0	0.0	0.0
0.0	0.0	0.0	0.0	0.0	0.0	0.0	0.0
0.0	0.0	0.0	0.0	0.0	0.0	0.0	0.0
0.0	0.0	0.0	0.0	0.0	0.0	0.0	0.0
0.0	0.0	0.0	0.0	0.0	0.0	0.0	0.0
0.0	0.0	0.0	0.0	0.0	0.0	0.0	0.0
0.0	0.0	0.0	0.0	0.0	0.0	0.0	0.0
0.0	0.0	0.0	0.0	0.0	0.0	0.0	0.0
0.0	0.0	0.0	0.0	0.0	0.0	0.0	0.0
0.0	0.0	0.0	0.0	0.0	0.0	0.0	0.0
0.0	0.0	0.0	0.0	0.0	0.0	0.0	0.0
0.0	0.0	0.0	0.0	0.0	0.0	0.0	0.0
0.0	0.0	0.0	0.0	0.0	0.0	0.0	0.0
0.0	0.0	0.0	0.0	0.0	0.0	0.0	0.0
0.0	0.0	0.0	0.0	0.0	0.0	0.0	0.0
0.0	0.0	0.0	0.0	0.0	0.0	0.0	0.0
0.0	0.0	0.0	0.0	0.0	0.0	0.0	0.0
0.0	0.0	0.0	0.0	0.0	0.0	0.0	0.0
0.0	0.0	0.0	0.0	0.0	0.0	0.0	0.0
0.0	0.0	0.0	0.0	0.0	0.0	0.0	0.0
0.0	0.0	0.0	0.0	0.0	0.0	0.0	0.0
0.0	0.0	0.0	0.0	0.0	0.0	0.0	0.0
0.0	0.0	0.0	0.0	0.0	0.0	0.0	0.0
0.0	0.0	0.0	0.0	0.0	0.0	0.0	0.0
0.0	0.0	0.0	0.0	0.0	0.0	0.0	0.0

0.0	0.0	0.0	0.0	0.0	0.0	0.0	0.0
0.0	0.0	0.0	0.0	0.0	0.0	0.0	0.0
0.0	0.0	0.0	0.0	0.0	0.0	0.0	0.0
0.0	0.0	0.0	0.0	0.0	0.0	0.0	0.0
0.0	0.0	0.0	0.0	0.0	0.0	0.0	0.0
0.0	0.0	0.0	0.0	0.0	0.0	0.0	0.0
0.0	0.0	0.0	0.0	0.0	0.0	0.0	0.0
0.0	0.0	0.0	0.0	0.0	0.0	0.0	0.0
0.0	0.0	0.0	0.0	0.0	0.0	0.0	0.0
0.0	0.0	0.0	0.0	0.0	0.0	0.0	0.0
0.0	0.0	0.0	0.0	0.0	0.0	0.0	0.0
0.0	0.0	0.0	0.0	0.0	0.0	0.0	0.0
0.0	0.0	0.0	0.0	0.0	0.0	0.0	0.0
0.0	0.0	0.0	0.0	0.0	0.0	0.0	0.0
0.0	0.0	0.0	0.0	0.0	0.0	0.0	0.0
0.0	0.0	0.0	0.0	0.0	0.0	0.0	0.0
0.0	0.0	0.0	0.0	0.0	0.0	0.0	0.0
0.0	0.0	0.0	0.0	0.0	0.0	0.0	0.0
0.0	0.0	0.0	0.0	0.0	0.0	0.0	0.0
0.0	0.0	0.0	0.0	0.0	0.0	0.0	0.0
0.0	0.0	0.0	0.0	0.0	0.0	0.0	0.0
0.0	0.0	0.0	0.0	0.0	0.0	0.0	0.0
0.0	0.0	0.0	0.0	0.0	0.0	0.0	0.0
0.0	0.0	0.0	0.0	0.0	0.0	0.0	0.0
0.0	0.0	0.0	0.0	0.0	0.0	0.0	0.0
0.0	0.0	0.0	0.0	0.0	0.0	0.0	0.0
0.0	0.0	0.0	0.0	0.0	0.0	0.0	0.0
0.0	0.0	0.0	0.0	0.0	0.0	0.0	0.0
0.0	0.0	0.0	0.0	0.0	0.0	0.0	0.0
0.0	0.0	0.0	0.0	0.0	0.0	0.0	0.0
0.0	0.0	0.0	0.0	0.0	0.0	0.0	0.0
0.0	0.0	0.0	0.0	0.0	0.0	0.0	0.0
0.0	0.0	0.0	0.0	0.0	0.0	0.0	0.0
0.0	0.0	0.0	0.0	0.0	0.0	0.0	0.0
0.0	0.0	0.0	0.0	0.0	0.0	0.0	0.0
0.0	0.0	0.0	0.0	0.0	0.0	0.0	0.0
0.0	0.0	0.0	0.0	0.0	0.0	0.0	0.0
0.0	0.0	0.0	0.0	0.0	0.0	0.0	0.0
0.0	0.0	0.0	0.0	0.0	0.0	0.0	0.0
0.0	0.0	0.0	0.0	0.0	0.0	0.0	0.0
0.0	0.0	0.0	0.0	0.0	0.0	0.0	0.0
0.0	0.0	0.0	0.0	0.0	0.0	0.0	0.0
0.0	0.0	0.0	0.0	0.0	0.0	0.0	0.0
0.0	0.0	0.0	0.0	0.0	0.0	0.0	0.0
0.0	0.0	0.0	0.0	0.0	0.0	0.0	0.0
0.0	0.0	0.0	0.0	0.0	0.0	0.0	0.0
0.0	0.0	0.0	0.0	0.0	0.0	0.0	0.0

0.0	0.0	0.0	0.0	0.0	0.0	0.0	0.0
0.0	0.0	0.0	0.0	0.0	0.0	0.0	0.0
0.0	0.0	0.0	0.0	0.0	0.0	0.0	0.0
0.0	0.0	0.0	0.0	0.0	0.0	0.0	0.0
0.0	0.0	0.0	0.0	0.0	0.0	0.0	0.0
0.0	0.0	0.0	0.0	0.0	0.0	0.0	0.0
0.0	0.0	0.0	0.0	0.0	0.0	0.0	0.0
0.0	0.0	0.0	0.0	0.0	0.0	0.0	0.0
0.0	0.0	0.0	0.0	0.0	0.0	0.0	0.0
0.0	0.0	0.0	0.0	0.0	0.0	0.0	0.0
0.0	0.0	0.0	0.0	0.0	0.0	0.0	0.0
0.0	0.0	0.0	0.0	0.0	0.0	0.0	0.0
0.0	0.0	0.0	0.0	0.0	0.0	0.0	0.0
0.0	0.0	0.0	0.0	0.0	0.0	0.0	0.0
0.0	0.0	0.0	0.0	0.0	0.0	0.0	0.0
0.0	0.0	0.0	0.0	0.0	0.0	0.0	0.0
0.0	0.0	0.0	0.0	0.0	0.0	0.0	0.0
0.0	0.0	0.0	0.0	0.0	0.0	0.0	0.0
0.0	0.0	0.0	0.0	0.0	0.0	0.0	0.0
0.0	0.0	0.0	0.0	0.0	0.0	0.0	0.0
0.0	0.0	0.0	0.0	0.0	0.0	0.0	0.0
0.0	0.0	0.0	0.0	0.0	0.0	0.0	0.0
0.0	0.0	0.0	0.0	0.0	0.0	0.0	0.0
0.0	0.0	0.0	0.0	0.0	0.0	0.0	0.0
0.0	0.0	0.0	0.0	0.0	0.0	0.0	0.0
0.0	0.0	0.0	0.0	0.0	0.0	0.0	0.0
0.0	0.0	0.0	0.0	0.0	0.0	0.0	0.0
0.0	0.0	0.0	0.0	0.0	0.0	0.0	0.0
0.0	0.0	0.0	0.0	0.0	0.0	0.0	0.0
0.0	0.0	0.0	0.0	0.0	0.0	0.0	0.0
0.0	0.0	0.0	0.0	0.0	0.0	0.0	0.0
0.0	0.0	0.0	0.0	0.0	0.0	0.0	0.0
0.0	0.0	0.0	0.0	0.0	0.0	0.0	0.0
0.0	0.0	0.0	0.0	0.0	0.0	0.0	0.0
0.0	0.0	0.0	0.0	0.0	0.0	0.0	0.0
0.0	0.0	0.0	0.0	0.0	0.0	0.0	0.0
0.0	0.0	0.0	0.0	0.0	0.0	0.0	0.0
0.0	0.0	0.0	0.0	0.0	0.0	0.0	0.0
0.0	0.0	0.0	0.0	0.0	0.0	0.0	0.0
0.0	0.0	0.0	0.0	0.0	0.0	0.0	0.0
0.0	0.0	0.0	0.0	0.0	0.0	0.0	0.0
0.0	0.0	0.0	0.0	0.0	0.0	0.0	0.0
0.0	0.0	0.0	0.0	0.0	0.0	0.0	0.0
0.0	0.0	0.0	0.0	0.0	0.0	0.0	0.0
0.0	0.0	0.0	0.0	0.0	0.0	0.0	0.0
0.0	0.0	0.0	0.0	0.0	0.0	0.0	0.0
0.0	0.0	0.0	0.0	0.0	0.0	0.0	0.0

0.0	0.0	0.0	0.0	0.0	0.0	0.0	0.0
0.0	0.0	0.0	0.0	0.0	0.0	0.0	0.0
0.0	0.0	0.0	0.0	0.0	0.0	0.0	0.0
0.0	0.0	0.0	0.0	0.0	0.0	0.0	0.0
0.0	0.0	0.0	0.0	0.0	0.0	0.0	0.0
0.0	0.0	0.0	0.0	0.0	0.0	0.0	0.0
0.0	0.0	0.0	0.0	0.0	0.0	0.0	0.0
0.0	0.0	0.0	0.0	0.0	0.0	0.0	0.0
0.0	0.0	0.0	0.0	0.0	0.0	0.0	0.0
0.0	0.0	0.0	0.0	0.0	0.0	0.0	0.0
0.0	0.0	0.0	0.0	0.0	0.0	0.0	0.0
0.0	0.0	0.0	0.0	0.0	0.0	0.0	0.0
0.0	0.0	0.0	0.0	0.0	0.0	0.0	0.0
0.0	0.0	0.0	0.0	0.0	0.0	0.0	0.0
0.0	0.0	0.0	0.0	0.0	0.0	0.0	0.0
0.0	0.0	0.0	0.0	0.0	0.0	0.0	0.0
0.0	0.0	0.0	0.0	0.0	0.0	0.0	0.0
0.0	0.0	0.0	0.0	0.0	0.0	0.0	0.0
0.0	0.0	0.0	0.0	0.0	0.0	0.0	0.0
0.0	0.0	0.0	0.0	0.0	0.0	0.0	0.0
0.0	0.0	0.0	0.0	0.0	0.0	0.0	0.0
0.0	0.0	0.0	0.0	0.0	0.0	0.0	0.0
0.0	0.0	0.0	0.0	0.0	0.0	0.0	0.0
0.0	0.0	0.0	0.0	0.0	0.0	0.0	0.0
0.0	0.0	0.0	0.0	0.0	0.0	0.0	0.0
0.0	0.0	0.0	0.0	0.0	0.0	0.0	0.0
0.0	0.0	0.0	0.0	0.0	0.0	0.0	0.0
0.0	0.0	0.0	0.0	0.0	0.0	0.0	0.0
0.0	0.0	0.0	0.0	0.0	0.0	0.0	0.0
0.0	0.0	0.0	0.0	0.0	0.0	0.0	0.0
0.0	0.0	0.0	0.0	0.0	0.0	0.0	0.0
0.0	0.0	0.0	0.0	0.0	0.0	0.0	0.0
0.0	0.0	0.0	0.0	0.0	0.0	0.0	0.0
0.0	0.0	0.0	0.0	0.0	0.0	0.0	0.0
0.0	0.0	0.0	0.0	0.0	0.0	0.0	0.0
0.0	0.0	0.0	0.0	0.0	0.0	0.0	0.0
0.0	0.0	0.0	0.0	0.0	0.0	0.0	0.0
0.0	0.0	0.0	0.0	0.0	0.0	0.0	0.0
0.0	0.0	0.0	0.0	0.0	0.0	0.0	0.0
0.0	0.0	0.0	0.0	0.0	0.0	0.0	0.0
0.0	0.0	0.0	0.0	0.0	0.0	0.0	0.0
0.0	0.0	0.0	0.0	0.0	0.0	0.0	0.0
0.0	0.0	0.0	0.0	0.0	0.0	0.0	0.0
0.0	0.0	0.0	0.0	0.0	0.0	0.0	0.0
0.0	0.0	0.0	0.0	0.0	0.0	0.0	0.0
0.0	0.0	0.0	0.0	0.0	0.0	0.0	0.0
0.0	0.0	0.0	0.0	0.0	0.0	0.0	0.0

0.0	0.0	0.0	0.0	0.0	0.0	0.0	0.0
0.0	0.0	0.0	0.0	0.0	0.0	0.0	0.0
0.0	0.0	0.0	0.0	0.0	0.0	0.0	0.0
0.0	0.0	0.0	0.0	0.0	0.0	0.0	0.0
0.0	0.0	0.0	0.0	0.0	0.0	0.0	0.0
0.0	0.0	0.0	0.0	0.0	0.0	0.0	0.0
0.0	0.0	0.0	0.0	0.0	0.0	0.0	0.0
0.0	0.0	0.0	0.0	0.0	0.0	0.0	0.0
0.0	0.0	0.0	0.0	0.0	0.0	0.0	0.0
0.0	0.0	0.0	0.0	0.0	0.0	0.0	0.0
0.0	0.0	0.0	0.0	0.0	0.0	0.0	0.0
0.0	0.0	0.0	0.0	0.0	0.0	0.0	0.0
0.0	0.0	0.0	0.0	0.0	0.0	0.0	0.0
0.0	0.0	0.0	0.0	0.0	0.0	0.0	0.0
0.0	0.0	0.0	0.0	0.0	0.0	0.0	0.0
0.0	0.0	0.0	0.0	0.0	0.0	0.0	0.0
0.0	0.0	0.0	0.0	0.0	0.0	0.0	0.0
0.0	0.0	0.0	0.0	0.0	0.0	0.0	0.0
0.0	0.0	0.0	0.0	0.0	0.0	0.0	0.0
0.0	0.0	0.0	0.0	0.0	0.0	0.0	0.0
0.0	0.0	0.0	0.0	0.0	0.0	0.0	0.0
0.0	0.0	0.0	0.0	0.0	0.0	0.0	0.0
0.0	0.0	0.0	0.0	0.0	0.0	0.0	0.0
0.0	0.0	0.0	0.0	0.0	0.0	0.0	0.0
0.0	0.0	0.0	0.0	0.0	0.0	0.0	0.0
0.0	0.0	0.0	0.0	0.0	0.0	0.0	0.0
0.0	0.0	0.0	0.0	0.0	0.0	0.0	0.0
0.0	0.0	0.0	0.0	0.0	0.0	0.0	0.0
0.0	0.0	0.0	0.0	0.0	0.0	0.0	0.0
0.0	0.0	0.0	0.0	0.0	0.0	0.0	0.0
0.0	0.0	0.0	0.0	0.0	0.0	0.0	0.0
0.0	0.0	0.0	0.0	0.0	0.0	0.0	0.0
0.0	0.0	0.0	0.0	0.0	0.0	0.0	0.0
0.0	0.0	0.0	0.0	0.0	0.0	0.0	0.0
0.0	0.0	0.0	0.0	0.0	0.0	0.0	0.0
0.0	0.0	0.0	0.0	0.0	0.0	0.0	0.0
0.0	0.0	0.0	0.0	0.0	0.0	0.0	0.0
0.0	0.0	0.0	0.0	0.0	0.0	0.0	0.0
0.0	0.0	0.0	0.0	0.0	0.0	0.0	0.0
0.0	0.0	0.0	0.0	0.0	0.0	0.0	0.0
0.0	0.0	0.0	0.0	0.0	0.0	0.0	0.0
0.0	0.0	0.0	0.0	0.0	0.0	0.0	0.0
0.0	0.0	0.0	0.0	0.0	0.0	0.0	0.0
0.0	0.0	0.0	0.0	0.0	0.0	0.0	0.0
0.0	0.0	0.0	0.0	0.0	0.0	0.0	0.0
0.0	0.0	0.0	0.0	0.0	0.0	0.0	0.0
0.0	0.0	0.0	0.0	0.0	0.0	0.0	0.0

0.0	0.0	0.0	0.0	0.0	0.0	0.0	0.0
0.0	0.0	0.0	0.0	0.0	0.0	0.0	0.0
0.0	0.0	0.0	0.0	0.0	0.0	0.0	0.0
0.0	0.0	0.0	0.0	0.0	0.0	0.0	0.0
0.0	0.0	0.0	0.0	0.0	0.0	0.0	0.0
0.0	0.0	0.0	0.0	0.0	0.0	0.0	0.0
0.0	0.0	0.0	0.0	0.0	0.0	0.0	0.0
0.0	0.0	0.0	0.0	0.0	0.0	0.0	0.0
0.0	0.0	0.0	0.0	0.0	0.0	0.0	0.0
0.0	0.0	0.0	0.0	0.0	0.0	0.0	0.0
0.0	0.0	0.0	0.0	0.0	0.0	0.0	0.0
0.0	0.0	0.0	0.0	0.0	0.0	0.0	0.0
0.0	0.0	0.0	0.0	0.0	0.0	0.0	0.0
0.0	0.0	0.0	0.0	0.0	0.0	0.0	0.0
0.0	0.0	0.0	0.0	0.0	0.0	0.0	0.0
0.0	0.0	0.0	0.0	0.0	0.0	0.0	0.0
0.0	0.0	0.0	0.0	0.0	0.0	0.0	0.0
0.0	0.0	0.0	0.0	0.0	0.0	0.0	0.0
0.0	0.0	0.0	0.0	0.0	0.0	0.0	0.0
0.0	0.0	0.0	0.0	0.0	0.0	0.0	0.0
0.0	0.0	0.0	0.0	0.0	0.0	0.0	0.0
0.0	0.0	0.0	0.0	0.0	0.0	0.0	0.0
0.0	0.0	0.0	0.0	0.0	0.0	0.0	0.0
0.0	0.0	0.0	0.0	0.0	0.0	0.0	0.0
0.0	0.0	0.0	0.0	0.0	0.0	0.0	0.0
0.0	0.0	0.0	0.0	0.0	0.0	0.0	0.0
0.0	0.0	0.0	0.0	0.0	0.0	0.0	0.0
0.0	0.0	0.0	0.0	0.0	0.0	0.0	0.0
0.0	0.0	0.0	0.0	0.0	0.0	0.0	0.0
0.0	0.0	0.0	0.0	0.0	0.0	0.0	0.0
0.0	0.0	0.0	0.0	0.0	0.0	0.0	0.0
0.0	0.0	0.0	0.0	0.0	0.0	0.0	0.0
0.0	0.0	0.0	0.0	0.0	0.0	0.0	0.0
0.0	0.0	0.0	0.0	0.0	0.0	0.0	0.0
0.0	0.0	0.0	0.0	0.0	0.0	0.0	0.0
0.0	0.0	0.0	0.0	0.0	0.0	0.0	0.0
0.0	0.0	0.0	0.0	0.0	0.0	0.0	0.0
0.0	0.0	0.0	0.0	0.0	0.0	0.0	0.0
0.0	0.0	0.0	0.0	0.0	0.0	0.0	0.0
0.0	0.0	0.0	0.0	0.0	0.0	0.0	0.0
0.0	0.0	0.0	0.0	0.0	0.0	0.0	0.0
0.0	0.0	0.0	0.0	0.0	0.0	0.0	0.0
0.0	0.0	0.0	0.0	0.0	0.0	0.0	0.0
0.0	0.0	0.0	0.0	0.0	0.0	0.0	0.0
0.0	0.0	0.0	0.0	0.0	0.0	0.0	0.0
0.0	0.0	0.0	0.0	0.0	0.0	0.0	0.0
0.0	0.0	0.0	0.0	0.0	0.0	0.0	0.0

0.0	0.0	0.0	0.0	0.0	0.0	0.0	0.0
0.0	0.0	0.0	0.0	0.0	0.0	0.0	0.0
0.0	0.0	0.0	0.0	0.0	0.0	0.0	0.0
0.0	0.0	0.0	0.0	0.0	0.0	0.0	0.0
0.0	0.0	0.0	0.0	0.0	0.0	0.0	0.0
0.0	0.0	0.0	0.0	0.0	0.0	0.0	0.0
0.0	0.0	0.0	0.0	0.0	0.0	0.0	0.0
0.0	0.0	0.0	0.0	0.0	0.0	0.0	0.0
0.0	0.0	0.0	0.0	0.0	0.0	0.0	0.0
0.0	0.0	0.0	0.0	0.0	0.0	0.0	0.0
0.0	0.0	0.0	0.0	0.0	0.0	0.0	0.0
0.0	0.0	0.0	0.0	0.0	0.0	0.0	0.0
0.0	0.0	0.0	0.0	0.0	0.0	0.0	0.0
0.0	0.0	0.0	0.0	0.0	0.0	0.0	0.0
0.0	0.0	0.0	0.0	0.0	0.0	0.0	0.0
0.0	0.0	0.0	0.0	0.0	0.0	0.0	0.0
0.0	0.0	0.0	0.0	0.0	0.0	0.0	0.0
0.0	0.0	0.0	0.0	0.0	0.0	0.0	0.0
0.0	0.0	0.0	0.0	0.0	0.0	0.0	0.0
0.0	0.0	0.0	0.0	0.0	0.0	0.0	0.0
0.0	0.0	0.0	0.0	0.0	0.0	0.0	0.0
0.0	0.0	0.0	0.0	0.0	0.0	0.0	0.0
0.0	0.0	0.0	0.0	0.0	0.0	0.0	0.0
0.0	0.0	0.0	0.0	0.0	0.0	0.0	0.0
0.0	0.0	0.0	0.0	0.0	0.0	0.0	0.0
0.0	0.0	0.0	0.0	0.0	0.0	0.0	0.0
0.0	0.0	0.0	0.0	0.0	0.0	0.0	0.0
0.0	0.0	0.0	0.0	0.0	0.0	0.0	0.0
0.0	0.0	0.0	0.0	0.0	0.0	0.0	0.0
0.0	0.0	0.0	0.0	0.0	0.0	0.0	0.0
0.0	0.0	0.0	0.0	0.0	0.0	0.0	0.0
0.0	0.0	0.0	0.0	0.0	0.0	0.0	0.0
0.0	0.0	0.0	0.0	0.0	0.0	0.0	0.0
0.0	0.0	0.0	0.0	0.0	0.0	0.0	0.0
0.0	0.0	0.0	0.0	0.0	0.0	0.0	0.0
0.0	0.0	0.0	0.0	0.0	0.0	0.0	0.0
0.0	0.0	0.0	0.0	0.0	0.0	0.0	0.0
0.0	0.0	0.0	0.0	0.0	0.0	0.0	0.0
0.0	0.0	0.0	0.0	0.0	0.0	0.0	0.0
0.0	0.0	0.0	0.0	0.0	0.0	0.0	0.0
0.0	0.0	0.0	0.0	0.0	0.0	0.0	0.0
0.0	0.0	0.0	0.0	0.0	0.0	0.0	0.0
0.0	0.0	0.0	0.0	0.0	0.0	0.0	0.0
0.0	0.0	0.0	0.0	0.0	0.0	0.0	0.0
0.0	0.0	0.0	0.0	0.0	0.0	0.0	0.0
0.0	0.0	0.0	0.0	0.0	0.0	0.0	0.0
0.0	0.0	0.0	0.0	0.0	0.0	0.0	0.0

0.0	0.0	0.0	0.0	0.0	0.0	0.0	0.0
0.0	0.0	0.0	0.0	0.0	0.0	0.0	0.0
0.0	0.0	0.0	0.0	0.0	0.0	0.0	0.0
0.0	0.0	0.0	0.0	0.0	0.0	0.0	0.0
0.0	0.0	0.0	0.0	0.0	0.0	0.0	0.0
0.0	0.0	0.0	0.0	0.0	0.0	0.0	0.0
0.0	0.0	0.0	0.0	0.0	0.0	0.0	0.0
0.0	0.0	0.0	0.0	0.0	0.0	0.0	0.0
0.0	0.0	0.0	0.0	0.0	0.0	0.0	0.0
0.0	0.0	0.0	0.0	0.0	0.0	0.0	0.0
0.0	0.0	0.0	0.0	0.0	0.0	0.0	0.0
0.0	0.0	0.0	0.0	0.0	0.0	0.0	0.0
0.0	0.0	0.0	0.0	0.0	0.0	0.0	0.0
0.0	0.0	0.0	0.0	0.0	0.0	0.0	0.0
0.0	0.0	0.0	0.0	0.0	0.0	0.0	0.0
0.0	0.0	0.0	0.0	0.0	0.0	0.0	0.0
0.0	0.0	0.0	0.0	0.0	0.0	0.0	0.0
0.0	0.0	0.0	0.0	0.0	0.0	0.0	0.0
0.0	0.0	0.0	0.0	0.0	0.0	0.0	0.0
0.0	0.0	0.0	0.0	0.0	0.0	0.0	0.0
0.0	0.0	0.0	0.0	0.0	0.0	0.0	0.0
0.0	0.0	0.0	0.0	0.0	0.0	0.0	0.0
0.0	0.0	0.0	0.0	0.0	0.0	0.0	0.0
0.0	0.0	0.0	0.0	0.0	0.0	0.0	0.0
0.0	0.0	0.0	0.0	0.0	0.0	0.0	0.0
0.0	0.0	0.0	0.0	0.0	0.0	0.0	0.0
0.0	0.0	0.0	0.0	0.0	0.0	0.0	0.0
0.0	0.0	0.0	0.0	0.0	0.0	0.0	0.0
0.0	0.0	0.0	0.0	0.0	0.0	0.0	0.0
0.0	0.0	0.0	0.0	0.0	0.0	0.0	0.0
0.0	0.0	0.0	0.0	0.0	0.0	0.0	0.0
0.0	0.0	0.0	0.0	0.0	0.0	0.0	0.0
0.0	0.0	0.0	0.0	0.0	0.0	0.0	0.0
0.0	0.0	0.0	0.0	0.0	0.0	0.0	0.0
0.0	0.0	0.0	0.0	0.0	0.0	0.0	0.0
0.0	0.0	0.0	0.0	0.0	0.0	0.0	0.0
0.0	0.0	0.0	0.0	0.0	0.0	0.0	0.0
0.0	0.0	0.0	0.0	0.0	0.0	0.0	0.0
0.0	0.0	0.0	0.0	0.0	0.0	0.0	0.0
0.0	0.0	0.0	0.0	0.0	0.0	0.0	0.0
0.0	0.0	0.0	0.0	0.0	0.0	0.0	0.0
0.0	0.0	0.0	0.0	0.0	0.0	0.0	0.0
0.0	0.0	0.0	0.0	0.0	0.0	0.0	0.0
0.0	0.0	0.0	0.0	0.0	0.0	0.0	0.0
0.0	0.0	0.0	0.0	0.0	0.0	0.0	0.0
0.0	0.0	0.0	0.0	0.0	0.0	0.0	0.0
0.0	0.0	0.0	0.0	0.0	0.0	0.0	0.0

0.0	0.0	0.0	0.0	0.0	0.0	0.0	0.0
0.0	0.0	0.0	0.0	0.0	0.0	0.0	0.0
0.0	0.0	0.0	0.0	0.0	0.0	0.0	0.0
0.0	0.0	0.0	0.0	0.0	0.0	0.0	0.0
0.0	0.0	0.0	0.0	0.0	0.0	0.0	0.0
0.0	0.0	0.0	0.0	0.0	0.0	0.0	0.0
0.0	0.0	0.0	0.0	0.0	0.0	0.0	0.0
0.0	0.0	0.0	0.0	0.0	0.0	0.0	0.0
0.0	0.0	0.0	0.0	0.0	0.0	0.0	0.0
0.0	0.0	0.0	0.0	0.0	0.0	0.0	0.0
0.0	0.0	0.0	0.0	0.0	0.0	0.0	0.0
0.0	0.0	0.0	0.0	0.0	0.0	0.0	0.0
0.0	0.0	0.0	0.0	0.0	0.0	0.0	0.0
0.0	0.0	0.0	0.0	0.0	0.0	0.0	0.0
0.0	0.0	0.0	0.0	0.0	0.0	0.0	0.0
0.0	0.0	0.0	0.0	0.0	0.0	0.0	0.0
0.0	0.0	0.0	0.0	0.0	0.0	0.0	0.0
0.0	0.0	0.0	0.0	0.0	0.0	0.0	0.0
0.0	0.0	0.0	0.0	0.0	0.0	0.0	0.0
0.0	0.0	0.0	0.0	0.0	0.0	0.0	0.0
0.0	0.0	0.0	0.0	0.0	0.0	0.0	0.0
0.0	0.0	0.0	0.0	0.0	0.0	0.0	0.0
0.0	0.0	0.0	0.0	0.0	0.0	0.0	0.0
0.0	0.0	0.0	0.0	0.0	0.0	0.0	0.0
0.0	0.0	0.0	0.0	0.0	0.0	0.0	0.0
0.0	0.0	0.0	0.0	0.0	0.0	0.0	0.0
0.0	0.0	0.0	0.0	0.0	0.0	0.0	0.0
0.0	0.0	0.0	0.0	0.0	0.0	0.0	0.0
0.0	0.0	0.0	0.0	0.0	0.0	0.0	0.0
0.0	0.0	0.0	0.0	0.0	0.0	0.0	0.0
0.0	0.0	0.0	0.0	0.0	0.0	0.0	0.0
0.0	0.0	0.0	0.0	0.0	0.0	0.0	0.0
0.0	0.0	0.0	0.0	0.0	0.0	0.0	0.0
0.0	0.0	0.0	0.0	0.0	0.0	0.0	0.0
0.0	0.0	0.0	0.0	0.0	0.0	0.0	0.0
0.0	0.0	0.0	0.0	0.0	0.0	0.0	0.0
0.0	0.0	0.0	0.0	0.0	0.0	0.0	0.0
0.0	0.0	0.0	0.0	0.0	0.0	0.0	0.0
0.0	0.0	0.0	0.0	0.0	0.0	0.0	0.0
0.0	0.0	0.0	0.0	0.0	0.0	0.0	0.0
0.0	0.0	0.0	0.0	0.0	0.0	0.0	0.0
0.0	0.0	0.0	0.0	0.0	0.0	0.0	0.0
0.0	0.0	0.0	0.0	0.0	0.0	0.0	0.0
0.0	0.0	0.0	0.0	0.0	0.0	0.0	0.0
0.0	0.0	0.0	0.0	0.0	0.0	0.0	0.0
0.0	0.0	0.0	0.0	0.0	0.0	0.0	0.0
0.0	0.0	0.0	0.0	0.0	0.0	0.0	0.0

0.0	0.0	0.0	0.0	0.0	0.0	0.0	0.0
0.0	0.0	0.0	0.0	0.0	0.0	0.0	0.0
0.0	0.0	0.0	0.0	0.0	0.0	0.0	0.0
0.0	0.0	0.0	0.0	0.0	0.0	0.0	0.0
0.0	0.0	0.0	0.0	0.0	0.0	0.0	0.0
0.0	0.0	0.0	0.0	0.0	0.0	0.0	0.0
0.0	0.0	0.0	0.0	0.0	0.0	0.0	0.0
0.0	0.0	0.0	0.0	0.0	0.0	0.0	0.0
0.0	0.0	0.0	0.0	0.0	0.0	0.0	0.0
0.0	0.0	0.0	0.0	0.0	0.0	0.0	0.0
0.0	0.0	0.0	0.0	0.0	0.0	0.0	0.0
0.0	0.0	0.0	0.0	0.0	0.0	0.0	0.0
0.0	0.0	0.0	0.0	0.0	0.0	0.0	0.0
0.0	0.0	0.0	0.0	0.0	0.0	0.0	0.0
0.0	0.0	0.0	0.0	0.0	0.0	0.0	0.0
0.0	0.0	0.0	0.0	0.0	0.0	0.0	0.0
0.0	0.0	0.0	0.0	0.0	0.0	0.0	0.0
0.0	0.0	0.0	0.0	0.0	0.0	0.0	0.0
0.0	0.0	0.0	0.0	0.0	0.0	0.0	0.0
0.0	0.0	0.0	0.0	0.0	0.0	0.0	0.0
0.0	0.0	0.0	0.0	0.0	0.0	0.0	0.0
0.0	0.0	0.0	0.0	0.0	0.0	0.0	0.0
0.0	0.0	0.0	0.0	0.0	0.0	0.0	0.0
0.0	0.0	0.0	0.0	0.0	0.0	0.0	0.0
0.0	0.0	0.0	0.0	0.0	0.0	0.0	0.0
0.0	0.0	0.0	0.0	0.0	0.0	0.0	0.0
0.0	0.0	0.0	0.0	0.0	0.0	0.0	0.0
0.0	0.0	0.0	0.0	0.0	0.0	0.0	0.0
0.0	0.0	0.0	0.0	0.0	0.0	0.0	0.0
0.0	0.0	0.0	0.0	0.0	0.0	0.0	0.0
0.0	0.0	0.0	0.0	0.0	0.0	0.0	0.0
0.0	0.0	0.0	0.0	0.0	0.0	0.0	0.0
0.0	0.0	0.0	0.0	0.0	0.0	0.0	0.0
0.0	0.0	0.0	0.0	0.0	0.0	0.0	0.0
0.0	0.0	0.0	0.0	0.0	0.0	0.0	0.0
0.0	0.0	0.0	0.0	0.0	0.0	0.0	0.0
0.0	0.0	0.0	0.0	0.0	0.0	0.0	0.0
0.0	0.0	0.0	0.0	0.0	0.0	0.0	0.0
0.0	0.0	0.0	0.0	0.0	0.0	0.0	0.0
0.0	0.0	0.0	0.0	0.0	0.0	0.0	0.0
0.0	0.0	0.0	0.0	0.0	0.0	0.0	0.0
0.0	0.0	0.0	0.0	0.0	0.0	0.0	0.0
0.0	0.0	0.0	0.0	0.0	0.0	0.0	0.0
0.0	0.0	0.0	0.0	0.0	0.0	0.0	0.0
0.0	0.0	0.0	0.0	0.0	0.0	0.0	0.0
0.0	0.0	0.0	0.0	0.0	0.0	0.0	0.0
0.0	0.0	0.0	0.0	0.0	0.0	0.0	0.0

0.0	0.0	0.0	0.0	0.0	0.0	0.0	0.0
0.0	0.0	0.0	0.0	0.0	0.0	0.0	0.0
0.0	0.0	0.0	0.0	0.0	0.0	0.0	0.0
0.0	0.0	0.0	0.0	0.0	0.0	0.0	0.0
0.0	0.0	0.0	0.0	0.0	0.0	0.0	0.0
0.0	0.0	0.0	0.0	0.0	0.0	0.0	0.0
0.0	0.0	0.0	0.0	0.0	0.0	0.0	0.0
0.0	0.0	0.0	0.0	0.0	0.0	0.0	0.0
0.0	0.0	0.0	0.0	0.0	0.0	0.0	0.0
0.0	0.0	0.0	0.0	0.0	0.0	0.0	0.0
0.0	0.0	0.0	0.0	0.0	0.0	0.0	0.0
0.0	0.0	0.0	0.0	0.0	0.0	0.0	0.0
0.0	0.0	0.0	0.0	0.0	0.0	0.0	0.0
0.0	0.0	0.0	0.0	0.0	0.0	0.0	0.0
0.0	0.0	0.0	0.0	0.0	0.0	0.0	0.0
0.0	0.0	0.0	0.0	0.0	0.0	0.0	0.0
0.0	0.0	0.0	0.0	0.0	0.0	0.0	0.0
0.0	0.0	0.0	0.0	0.0	0.0	0.0	0.0
0.0	0.0	0.0	0.0	0.0	0.0	0.0	0.0
0.0	0.0	0.0	0.0	0.0	0.0	0.0	0.0
0.0	0.0	0.0	0.0	0.0	0.0	0.0	0.0
0.0	0.0	0.0	0.0	0.0	0.0	0.0	0.0
0.0	0.0	0.0	0.0	0.0	0.0	0.0	0.0
0.0	0.0	0.0	0.0	0.0	0.0	0.0	0.0
0.0	0.0	0.0	0.0	0.0	0.0	0.0	0.0
0.0	0.0	0.0	0.0	0.0	0.0	0.0	0.0
0.0	0.0	0.0	0.0	0.0	0.0	0.0	0.0
0.0	0.0	0.0	0.0	0.0	0.0	0.0	0.0
0.0	0.0	0.0	0.0	0.0	0.0	0.0	0.0
0.0	0.0	0.0	0.0	0.0	0.0	0.0	0.0
0.0	0.0	0.0	0.0	0.0	0.0	0.0	0.0
0.0	0.0	0.0	0.0	0.0	0.0	0.0	0.0
0.0	0.0	0.0	0.0	0.0	0.0	0.0	0.0
0.0	0.0	0.0	0.0	0.0	0.0	0.0	0.0
0.0	0.0	0.0	0.0	0.0	0.0	0.0	0.0
0.0	0.0	0.0	0.0	0.0	0.0	0.0	0.0
0.0	0.0	0.0	0.0	0.0	0.0	0.0	0.0
0.0	0.0	0.0	0.0	0.0	0.0	0.0	0.0
0.0	0.0	0.0	0.0	0.0	0.0	0.0	0.0
0.0	0.0	0.0	0.0	0.0	0.0	0.0	0.0
0.0	0.0	0.0	0.0	0.0	0.0	0.0	0.0
0.0	0.0	0.0	0.0	0.0	0.0	0.0	0.0
0.0	0.0	0.0	0.0	0.0	0.0	0.0	0.0
0.0	0.0	0.0	0.0	0.0	0.0	0.0	0.0
0.0	0.0	0.0	0.0	0.0	0.0	0.0	0.0
0.0	0.0	0.0	0.0	0.0	0.0	0.0	0.0
0.0	0.0	0.0	0.0	0.0	0.0	0.0	0.0

0.0	0.0	0.0	0.0	0.0	0.0	0.0	0.0
0.0	0.0	0.0	0.0	0.0	0.0	0.0	0.0
0.0	0.0	0.0	0.0	0.0	0.0	0.0	0.0
0.0	0.0	0.0	0.0	0.0	0.0	0.0	0.0
0.0	0.0	0.0	0.0	0.0	0.0	0.0	0.0
0.0	0.0	0.0	0.0	0.0	0.0	0.0	0.0
0.0	0.0	0.0	0.0	0.0	0.0	0.0	0.0
0.0	0.0	0.0	0.0	0.0	0.0	0.0	0.0
0.0	0.0	0.0	0.0	0.0	0.0	0.0	0.0
0.0	0.0	0.0	0.0	0.0	0.0	0.0	0.0
0.0	0.0	0.0	0.0	0.0	0.0	0.0	0.0
0.0	0.0	0.0	0.0	0.0	0.0	0.0	0.0
0.0	0.0	0.0	0.0	0.0	0.0	0.0	0.0
0.0	0.0	0.0	0.0	0.0	0.0	0.0	0.0
0.0	0.0	0.0	0.0	0.0	0.0	0.0	0.0
0.0	0.0	0.0	0.0	0.0	0.0	0.0	0.0
0.0	0.0	0.0	0.0	0.0	0.0	0.0	0.0
0.0	0.0	0.0	0.0	0.0	0.0	0.0	0.0
0.0	0.0	0.0	0.0	0.0	0.0	0.0	0.0
0.0	0.0	0.0	0.0	0.0	0.0	0.0	0.0
0.0	0.0	0.0	0.0	0.0	0.0	0.0	0.0
0.0	0.0	0.0	0.0	0.0	0.0	0.0	0.0
0.0	0.0	0.0	0.0	0.0	0.0	0.0	0.0
0.0	0.0	0.0	0.0	0.0	0.0	0.0	0.0
0.0	0.0	0.0	0.0	0.0	0.0	0.0	0.0
0.0	0.0	0.0	0.0	0.0	0.0	0.0	0.0
0.0	0.0	0.0	0.0	0.0	0.0	0.0	0.0
0.0	0.0	0.0	0.0	0.0	0.0	0.0	0.0
0.0	0.0	0.0	0.0	0.0	0.0	0.0	0.0
0.0	0.0	0.0	0.0	0.0	0.0	0.0	0.0
0.0	0.0	0.0	0.0	0.0	0.0	0.0	0.0
0.0	0.0	0.0	0.0	0.0	0.0	0.0	0.0
0.0	0.0	0.0	0.0	0.0	0.0	0.0	0.0
0.0	0.0	0.0	0.0	0.0	0.0	0.0	0.0
0.0	0.0	0.0	0.0	0.0	0.0	0.0	0.0
0.0	0.0	0.0	0.0	0.0	0.0	0.0	0.0
0.0	0.0	0.0	0.0	0.0	0.0	0.0	0.0
0.0	0.0	0.0	0.0	0.0	0.0	0.0	0.0
0.0	0.0	0.0	0.0	0.0	0.0	0.0	0.0
0.0	0.0	0.0	0.0	0.0	0.0	0.0	0.0
0.0	0.0	0.0	0.0	0.0	0.0	0.0	0.0
0.0	0.0	0.0	0.0	0.0	0.0	0.0	0.0
0.0	0.0	0.0	0.0	0.0	0.0	0.0	0.0
0.0	0.0	0.0	0.0	0.0	0.0	0.0	0.0
0.0	0.0	0.0	0.0	0.0	0.0	0.0	0.0
0.0	0.0	0.0	0.0	0.0	0.0	0.0	0.0
0.0	0.0	0.0	0.0	0.0	0.0	0.0	0.0
0.0	0.0	0.0	0.0	0.0	0.0	0.0	0.0

0.0	0.0	0.0	0.0	0.0	0.0	0.0	0.0
0.0	0.0	0.0	0.0	0.0	0.0	0.0	0.0
0.0	0.0	0.0	0.0	0.0	0.0	0.0	0.0
0.0	0.0	0.0	0.0	0.0	0.0	0.0	0.0
0.0	0.0	0.0	0.0	0.0	0.0	0.0	0.0
0.0	0.0	0.0	0.0	0.0	0.0	0.0	0.0
0.0	0.0	0.0	0.0	0.0	0.0	0.0	0.0
0.0	0.0	0.0	0.0	0.0	0.0	0.0	0.0
0.0	0.0	0.0	0.0	0.0	0.0	0.0	0.0
0.0	0.0	0.0	0.0	0.0	0.0	0.0	0.0
0.0	0.0	0.0	0.0	0.0	0.0	0.0	0.0
0.0	0.0	0.0	0.0	0.0	0.0	0.0	0.0
0.0	0.0	0.0	0.0	0.0	0.0	0.0	0.0
0.0	0.0	0.0	0.0	0.0	0.0	0.0	0.0
0.0	0.0	0.0	0.0	0.0	0.0	0.0	0.0
0.0	0.0	0.0	0.0	0.0	0.0	0.0	0.0
0.0	0.0	0.0	0.0	0.0	0.0	0.0	0.0
0.0	0.0	0.0	0.0	0.0	0.0	0.0	0.0
0.0	0.0	0.0	0.0	0.0	0.0	0.0	0.0
0.0	0.0	0.0	0.0	0.0	0.0	0.0	0.0
0.0	0.0	0.0	0.0	0.0	0.0	0.0	0.0
0.0	0.0	0.0	0.0	0.0	0.0	0.0	0.0
0.0	0.0	0.0	0.0	0.0	0.0	0.0	0.0
0.0	0.0	0.0	0.0	0.0	0.0	0.0	0.0
0.0	0.0	0.0	0.0	0.0	0.0	0.0	0.0
0.0	0.0	0.0	0.0	0.0	0.0	0.0	0.0
0.0	0.0	0.0	0.0	0.0	0.0	0.0	0.0
0.0	0.0	0.0	0.0	0.0	0.0	0.0	0.0
0.0	0.0	0.0	0.0	0.0	0.0	0.0	0.0
0.0	0.0	0.0	0.0	0.0	0.0	0.0	0.0
0.0	0.0	0.0	0.0	0.0	0.0	0.0	0.0
0.0	0.0	0.0	0.0	0.0	0.0	0.0	0.0
0.0	0.0	0.0	0.0	0.0	0.0	0.0	0.0
0.0	0.0	0.0	0.0	0.0	0.0	0.0	0.0
0.0	0.0	0.0	0.0	0.0	0.0	0.0	0.0
0.0	0.0	0.0	0.0	0.0	0.0	0.0	0.0
0.0	0.0	0.0	0.0	0.0	0.0	0.0	0.0
0.0	0.0	0.0	0.0	0.0	0.0	0.0	0.0
0.0	0.0	0.0	0.0	0.0	0.0	0.0	0.0
0.0	0.0	0.0	0.0	0.0	0.0	0.0	0.0
0.0	0.0	0.0	0.0	0.0	0.0	0.0	0.0
0.0	0.0	0.0	0.0	0.0	0.0	0.0	0.0
0.0	0.0	0.0	0.0	0.0	0.0	0.0	0.0
0.0	0.0	0.0	0.0	0.0	0.0	0.0	0.0
0.0	0.0	0.0	0.0	0.0	0.0	0.0	0.0
0.0	0.0	0.0	0.0	0.0	0.0	0.0	0.0
0.0	0.0	0.0	0.0	0.0	0.0	0.0	0.0
0.0	0.0	0.0	0.0	0.0	0.0	0.0	0.0

0.0	0.0	0.0	0.0	0.0	0.0	0.0	0.0
0.0	0.0	0.0	0.0	0.0	0.0	0.0	0.0
0.0	0.0	0.0	0.0	0.0	0.0	0.0	0.0
0.0	0.0	0.0	0.0	0.0	0.0	0.0	0.0
0.0	0.0	0.0	0.0	0.0	0.0	0.0	0.0
0.0	0.0	0.0	0.0	0.0	0.0	0.0	0.0
0.0	0.0	0.0	0.0	0.0	0.0	0.0	0.0
0.0	0.0	0.0	0.0	0.0	0.0	0.0	0.0
0.0	0.0	0.0	0.0	0.0	0.0	0.0	0.0
0.0	0.0	0.0	0.0	0.0	0.0	0.0	0.0
0.0	0.0	0.0	0.0	0.0	0.0	0.0	0.0
0.0	0.0	0.0	0.0	0.0	0.0	0.0	0.0
0.0	0.0	0.0	0.0	0.0	0.0	0.0	0.0
0.0	0.0	0.0	0.0	0.0	0.0	0.0	0.0
0.0	0.0	0.0	0.0	0.0	0.0	0.0	0.0
0.0	0.0	0.0	0.0	0.0	0.0	0.0	0.0
0.0	0.0	0.0	0.0	0.0	0.0	0.0	0.0
0.0	0.0	0.0	0.0	0.0	0.0	0.0	0.0
0.0	0.0	0.0	0.0	0.0	0.0	0.0	0.0
0.0	0.0	0.0	0.0	0.0	0.0	0.0	0.0
0.0	0.0	0.0	0.0	0.0	0.0	0.0	0.0
0.0	0.0	0.0	0.0	0.0	0.0	0.0	0.0
0.0	0.0	0.0	0.0	0.0	0.0	0.0	0.0
0.0	0.0	0.0	0.0	0.0	0.0	0.0	0.0
0.0	0.0	0.0	0.0	0.0	0.0	0.0	0.0
0.0	0.0	0.0	0.0	0.0	0.0	0.0	0.0
0.0	0.0	0.0	0.0	0.0	0.0	0.0	0.0
0.0	0.0	0.0	0.0	0.0	0.0	0.0	0.0
0.0	0.0	0.0	0.0	0.0	0.0	0.0	0.0
0.0	0.0	0.0	0.0	0.0	0.0	0.0	0.0
0.0	0.0	0.0	0.0	0.0	0.0	0.0	0.0
0.0	0.0	0.0	0.0	0.0	0.0	0.0	0.0
0.0	0.0	0.0	0.0	0.0	0.0	0.0	0.0
0.0	0.0	0.0	0.0	0.0	0.0	0.0	0.0
0.0	0.0	0.0	0.0	0.0	0.0	0.0	0.0
0.0	0.0	0.0	0.0	0.0	0.0	0.0	0.0
0.0	0.0	0.0	0.0	0.0	0.0	0.0	0.0
0.0	0.0	0.0	0.0	0.0	0.0	0.0	0.0
0.0	0.0	0.0	0.0	0.0	0.0	0.0	0.0
0.0	0.0	0.0	0.0	0.0	0.0	0.0	0.0
0.0	0.0	0.0	0.0	0.0	0.0	0.0	0.0
0.0	0.0	0.0	0.0	0.0	0.0	0.0	0.0
0.0	0.0	0.0	0.0	0.0	0.0	0.0	0.0
0.0	0.0	0.0	0.0	0.0	0.0	0.0	0.0
0.0	0.0	0.0	0.0	0.0	0.0	0.0	0.0
0.0	0.0	0.0	0.0	0.0	0.0	0.0	0.0
0.0	0.0	0.0	0.0	0.0	0.0	0.0	0.0

0.0	0.0	0.0	0.0	0.0	0.0	0.0	0.0
0.0	0.0	0.0	0.0	0.0	0.0	0.0	0.0
0.0	0.0	0.0	0.0	0.0	0.0	0.0	0.0
0.0	0.0	0.0	0.0	0.0	0.0	0.0	0.0
0.0	0.0	0.0	0.0	0.0	0.0	0.0	0.0
0.0	0.0	0.0	0.0	0.0	0.0	0.0	0.0
0.0	0.0	0.0	0.0	0.0	0.0	0.0	0.0
0.0	0.0	0.0	0.0	0.0	0.0	0.0	0.0
0.0	0.0	0.0	0.0	0.0	0.0	0.0	0.0
0.0	0.0	0.0	0.0	0.0	0.0	0.0	0.0
0.0	0.0	0.0	0.0	0.0	0.0	0.0	0.0
0.0	0.0	0.0	0.0	0.0	0.0	0.0	0.0
0.0	0.0	0.0	0.0	0.0	0.0	0.0	0.0
0.0	0.0	0.0	0.0	0.0	0.0	0.0	0.0
0.0	0.0	0.0	0.0	0.0	0.0	0.0	0.0
0.0	0.0	0.0	0.0	0.0	0.0	0.0	0.0
0.0	0.0	0.0	0.0	0.0	0.0	0.0	0.0
0.0	0.0	0.0	0.0	0.0	0.0	0.0	0.0
0.0	0.0	0.0	0.0	0.0	0.0	0.0	0.0
0.0	0.0	0.0	0.0	0.0	0.0	0.0	0.0
0.0	0.0	0.0	0.0	0.0	0.0	0.0	0.0
0.0	0.0	0.0	0.0	0.0	0.0	0.0	0.0
0.0	0.0	0.0	0.0	0.0	0.0	0.0	0.0
0.0	0.0	0.0	0.0	0.0	0.0	0.0	0.0
0.0	0.0	0.0	0.0	0.0	0.0	0.0	0.0
0.0	0.0	0.0	0.0	0.0	0.0	0.0	0.0
0.0	0.0	0.0	0.0	0.0	0.0	0.0	0.0
0.0	0.0	0.0	0.0	0.0	0.0	0.0	0.0
0.0	0.0	0.0	0.0	0.0	0.0	0.0	0.0
0.0	0.0	0.0	0.0	0.0	0.0	0.0	0.0
0.0	0.0	0.0	0.0	0.0	0.0	0.0	0.0
0.0	0.0	0.0	0.0	0.0	0.0	0.0	0.0
0.0	0.0	0.0	0.0	0.0	0.0	0.0	0.0
0.0	0.0	0.0	0.0	0.0	0.0	0.0	0.0
0.0	0.0	0.0	0.0	0.0	0.0	0.0	0.0
0.0	0.0	0.0	0.0	0.0	0.0	0.0	0.0
0.0	0.0	0.0	0.0	0.0	0.0	0.0	0.0
0.0	0.0	0.0	0.0	0.0	0.0	0.0	0.0
0.0	0.0	0.0	0.0	0.0	0.0	0.0	0.0
0.0	0.0	0.0	0.0	0.0	0.0	0.0	0.0
0.0	0.0	0.0	0.0	0.0	0.0	0.0	0.0
0.0	0.0	0.0	0.0	0.0	0.0	0.0	0.0
0.0	0.0	0.0	0.0	0.0	0.0	0.0	0.0
0.0	0.0	0.0	0.0	0.0	0.0	0.0	0.0
0.0	0.0	0.0	0.0	0.0	0.0	0.0	0.0
0.0	0.0	0.0	0.0	0.0	0.0	0.0	0.0
0.0	0.0	0.0	0.0	0.0	0.0	0.0	0.0

0.0	0.0	0.0	0.0	0.0	0.0	0.0	0.0
0.0	0.0	0.0	0.0	0.0	0.0	0.0	0.0
0.0	0.0	0.0	0.0	0.0	0.0	0.0	0.0
0.0	0.0	0.0	0.0	0.0	0.0	0.0	0.0
0.0	0.0	0.0	0.0	0.0	0.0	0.0	0.0
0.0	0.0	0.0	0.0	0.0	0.0	0.0	0.0
0.0	0.0	0.0	0.0	0.0	0.0	0.0	0.0
0.0	0.0	0.0	0.0	0.0	0.0	0.0	0.0
0.0	0.0	0.0	0.0	0.0	0.0	0.0	0.0
0.0	0.0	0.0	0.0	0.0	0.0	0.0	0.0
0.0	0.0	0.0	0.0	0.0	0.0	0.0	0.0
0.0	0.0	0.0	0.0	0.0	0.0	0.0	0.0
0.0	0.0	0.0	0.0	0.0	0.0	0.0	0.0
0.0	0.0	0.0	0.0	0.0	0.0	0.0	0.0
0.0	0.0	0.0	0.0	0.0	0.0	0.0	0.0
0.0	0.0	0.0	0.0	0.0	0.0	0.0	0.0
0.0	0.0	0.0	0.0	0.0	0.0	0.0	0.0
0.0	0.0	0.0	0.0	0.0	0.0	0.0	0.0
0.0	0.0	0.0	0.0	0.0	0.0	0.0	0.0
0.0	0.0	0.0	0.0	0.0	0.0	0.0	0.0
0.0	0.0	0.0	0.0	0.0	0.0	0.0	0.0
0.0	0.0	0.0	0.0	0.0	0.0	0.0	0.0
0.0	0.0	0.0	0.0	0.0	0.0	0.0	0.0
0.0	0.0	0.0	0.0	0.0	0.0	0.0	0.0
0.0	0.0	0.0	0.0	0.0	0.0	0.0	0.0
0.0	0.0	0.0	0.0	0.0	0.0	0.0	0.0
0.0	0.0	0.0	0.0	0.0	0.0	0.0	0.0
0.0	0.0	0.0	0.0	0.0	0.0	0.0	0.0
0.0	0.0	0.0	0.0	0.0	0.0	0.0	0.0
0.0	0.0	0.0	0.0	0.0	0.0	0.0	0.0
0.0	0.0	0.0	0.0	0.0	0.0	0.0	0.0
0.0	0.0	0.0	0.0	0.0	0.0	0.0	0.0
0.0	0.0	0.0	0.0	0.0	0.0	0.0	0.0
0.0	0.0	0.0	0.0	0.0	0.0	0.0	0.0
0.0	0.0	0.0	0.0	0.0	0.0	0.0	0.0
0.0	0.0	0.0	0.0	0.0	0.0	0.0	0.0
0.0	0.0	0.0	0.0	0.0	0.0	0.0	0.0
0.0	0.0	0.0	0.0	0.0	0.0	0.0	0.0
0.0	0.0	0.0	0.0	0.0	0.0	0.0	0.0
0.0	0.0	0.0	0.0	0.0	0.0	0.0	0.0
0.0	0.0	0.0	0.0	0.0	0.0	0.0	0.0
0.0	0.0	0.0	0.0	0.0	0.0	0.0	0.0
0.0	0.0	0.0	0.0	0.0	0.0	0.0	0.0
0.0	0.0	0.0	0.0	0.0	0.0	0.0	0.0
0.0	0.0	0.0	0.0	0.0	0.0	0.0	0.0
0.0	0.0	0.0	0.0	0.0	0.0	0.0	0.0
0.0	0.0	0.0	0.0	0.0	0.0	0.0	0.0
0.0	0.0	0.0	0.0	0.0	0.0	0.0	0.0

0.0	0.0	0.0	0.0	0.0	0.0	0.0	0.0
0.0	0.0	0.0	0.0	0.0	0.0	0.0	0.0
0.0	0.0	0.0	0.0	0.0	0.0	0.0	0.0
0.0	0.0	0.0	0.0	0.0	0.0	0.0	0.0
0.0	0.0	0.0	0.0	0.0	0.0	0.0	0.0
0.0	0.0	0.0	0.0	0.0	0.0	0.0	0.0
0.0	0.0	0.0	0.0	0.0	0.0	0.0	0.0
0.0	0.0	0.0	0.0	0.0	0.0	0.0	0.0
0.0	0.0	0.0	0.0	0.0	0.0	0.0	0.0
0.0	0.0	0.0	0.0	0.0	0.0	0.0	0.0
0.0	0.0	0.0	0.0	0.0	0.0	0.0	0.0
0.0	0.0	0.0	0.0	0.0	0.0	0.0	0.0
0.0	0.0	0.0	0.0	0.0	0.0	0.0	0.0
0.0	0.0	0.0	0.0	0.0	0.0	0.0	0.0
0.0	0.0	0.0	0.0	0.0	0.0	0.0	0.0
0.0	0.0	0.0	0.0	0.0	0.0	0.0	0.0
0.0	0.0	0.0	0.0	0.0	0.0	0.0	0.0
0.0	0.0	0.0	0.0	0.0	0.0	0.0	0.0
0.0	0.0	0.0	0.0	0.0	0.0	0.0	0.0
0.0	0.0	0.0	0.0	0.0	0.0	0.0	0.0
0.0	0.0	0.0	0.0	0.0	0.0	0.0	0.0
0.0	0.0	0.0	0.0	0.0	0.0	0.0	0.0
0.0	0.0	0.0	0.0	0.0	0.0	0.0	0.0
0.0	0.0	0.0	0.0	0.0	0.0	0.0	0.0
0.0	0.0	0.0	0.0	0.0	0.0	0.0	0.0
0.0	0.0	0.0	0.0	0.0	0.0	0.0	0.0
0.0	0.0	0.0	0.0	0.0	0.0	0.0	0.0
0.0	0.0	0.0	0.0	0.0	0.0	0.0	0.0
0.0	0.0	0.0	0.0	0.0	0.0	0.0	0.0
0.0	0.0	0.0	0.0	0.0	0.0	0.0	0.0
0.0	0.0	0.0	0.0	0.0	0.0	0.0	0.0
0.0	0.0	0.0	0.0	0.0	0.0	0.0	0.0
0.0	0.0	0.0	0.0	0.0	0.0	0.0	0.0
0.0	0.0	0.0	0.0	0.0	0.0	0.0	0.0
0.0	0.0	0.0	0.0	0.0	0.0	0.0	0.0
0.0	0.0	0.0	0.0	0.0	0.0	0.0	0.0
0.0	0.0	0.0	0.0	0.0	0.0	0.0	0.0
0.0	0.0	0.0	0.0	0.0	0.0	0.0	0.0
0.0	0.0	0.0	0.0	0.0	0.0	0.0	0.0
0.0	0.0	0.0	0.0	0.0	0.0	0.0	0.0
0.0	0.0	0.0	0.0	0.0	0.0	0.0	0.0
0.0	0.0	0.0	0.0	0.0	0.0	0.0	0.0
0.0	0.0	0.0	0.0	0.0	0.0	0.0	0.0
0.0	0.0	0.0	0.0	0.0	0.0	0.0	0.0
0.0	0.0	0.0	0.0	0.0	0.0	0.0	0.0
0.0	0.0	0.0	0.0	0.0	0.0	0.0	0.0
0.0	0.0	0.0	0.0	0.0	0.0	0.0	0.0

0.0	0.0	0.0	0.0	0.0	0.0	0.0	0.0
0.0	0.0	0.0	0.0	0.0	0.0	0.0	0.0
0.0	0.0	0.0	0.0	0.0	0.0	0.0	0.0
0.0	0.0	0.0	0.0	0.0	0.0	0.0	0.0
0.0	0.0	0.0	0.0	0.0	0.0	0.0	0.0
0.0	0.0	0.0	0.0	0.0	0.0	0.0	0.0
0.0	0.0	0.0	0.0	0.0	0.0	0.0	0.0
0.0	0.0	0.0	0.0	0.0	0.0	0.0	0.0
0.0	0.0	0.0	0.0	0.0	0.0	0.0	0.0
0.0	0.0	0.0	0.0	0.0	0.0	0.0	0.0
0.0	0.0	0.0	0.0	0.0	0.0	0.0	0.0
0.0	0.0	0.0	0.0	0.0	0.0	0.0	0.0
0.0	0.0	0.0	0.0	0.0	0.0	0.0	0.0
0.0	0.0	0.0	0.0	0.0	0.0	0.0	0.0
0.0	0.0	0.0	0.0	0.0	0.0	0.0	0.0
0.0	0.0	0.0	0.0	0.0	0.0	0.0	0.0
0.0	0.0	0.0	0.0	0.0	0.0	0.0	0.0
0.0	0.0	0.0	0.0	0.0	0.0	0.0	0.0
0.0	0.0	0.0	0.0	0.0	0.0	0.0	0.0
0.0	0.0	0.0	0.0	0.0	0.0	0.0	0.0
0.0	0.0	0.0	0.0	0.0	0.0	0.0	0.0
0.0	0.0	0.0	0.0	0.0	0.0	0.0	0.0
0.0	0.0	0.0	0.0	0.0	0.0	0.0	0.0
0.0	0.0	0.0	0.0	0.0	0.0	0.0	0.0
0.0	0.0	0.0	0.0	0.0	0.0	0.0	0.0
0.0	0.0	0.0	0.0	0.0	0.0	0.0	0.0
0.0	0.0	0.0	0.0	0.0	0.0	0.0	0.0
0.0	0.0	0.0	0.0	0.0	0.0	0.0	0.0
0.0	0.0	0.0	0.0	0.0	0.0	0.0	0.0
0.0	0.0	0.0	0.0	0.0	0.0	0.0	0.0
0.0	0.0	0.0	0.0	0.0	0.0	0.0	0.0
0.0	0.0	0.0	0.0	0.0	0.0	0.0	0.0
0.0	0.0	0.0	0.0	0.0	0.0	0.0	0.0
0.0	0.0	0.0	0.0	0.0	0.0	0.0	0.0
0.0	0.0	0.0	0.0	0.0	0.0	0.0	0.0
0.0	0.0	0.0	0.0	0.0	0.0	0.0	0.0
0.0	0.0	0.0	0.0	0.0	0.0	0.0	0.0
0.0	0.0	0.0	0.0	0.0	0.0	0.0	0.0
0.0	0.0	0.0	0.0	0.0	0.0	0.0	0.0
0.0	0.0	0.0	0.0	0.0	0.0	0.0	0.0
0.0	0.0	0.0	0.0	0.0	0.0	0.0	0.0
0.0	0.0	0.0	0.0	0.0	0.0	0.0	0.0
0.0	0.0	0.0	0.0	0.0	0.0	0.0	0.0
0.0	0.0	0.0	0.0	0.0	0.0	0.0	0.0
0.0	0.0	0.0	0.0	0.0	0.0	0.0	0.0
0.0	0.0	0.0	0.0	0.0	0.0	0.0	0.0
0.0	0.0	0.0	0.0	0.0	0.0	0.0	0.0
0.0	0.0	0.0	0.0	0.0	0.0	0.0	0.0

0.0	0.0	0.0	0.0	0.0	0.0	0.0	0.0
0.0	0.0	0.0	0.0	0.0	0.0	0.0	0.0
0.0	0.0	0.0	0.0	0.0	0.0	0.0	0.0
0.0	0.0	0.0	0.0	0.0	0.0	0.0	0.0
0.0	0.0	0.0	0.0	0.0	0.0	0.0	0.0
0.0	0.0	0.0	0.0	0.0	0.0	0.0	0.0
0.0	0.0	0.0	0.0	0.0	0.0	0.0	0.0
0.0	0.0	0.0	0.0	0.0	0.0	0.0	0.0
0.0	0.0	0.0	0.0	0.0	0.0	0.0	0.0
0.0	0.0	0.0	0.0	0.0	0.0	0.0	0.0
0.0	0.0	0.0	0.0	0.0	0.0	0.0	0.0
0.0	0.0	0.0	0.0	0.0	0.0	0.0	0.0
0.0	0.0	0.0	0.0	0.0	0.0	0.0	0.0
0.0	0.0	0.0	0.0	0.0	0.0	0.0	0.0
0.0	0.0	0.0	0.0	0.0	0.0	0.0	0.0
0.0	0.0	0.0	0.0	0.0	0.0	0.0	0.0
0.0	0.0	0.0	0.0	0.0	0.0	0.0	0.0
0.0	0.0	0.0	0.0	0.0	0.0	0.0	0.0
0.0	0.0	0.0	0.0	0.0	0.0	0.0	0.0
0.0	0.0	0.0	0.0	0.0	0.0	0.0	0.0
0.0	0.0	0.0	0.0	0.0	0.0	0.0	0.0
0.0	0.0	0.0	0.0	0.0	0.0	0.0	0.0
0.0	0.0	0.0	0.0	0.0	0.0	0.0	0.0
0.0	0.0	0.0	0.0	0.0	0.0	0.0	0.0
0.0	0.0	0.0	0.0	0.0	0.0	0.0	0.0
0.0	0.0	0.0	0.0	0.0	0.0	0.0	0.0
0.0	0.0	0.0	0.0	0.0	0.0	0.0	0.0
0.0	0.0	0.0	0.0	0.0	0.0	0.0	0.0
0.0	0.0	0.0	0.0	0.0	0.0	0.0	0.0
0.0	0.0	0.0	0.0	0.0	0.0	0.0	0.0
0.0	0.0	0.0	0.0	0.0	0.0	0.0	0.0
0.0	0.0	0.0	0.0	0.0	0.0	0.0	0.0
0.0	0.0	0.0	0.0	0.0	0.0	0.0	0.0
0.0	0.0	0.0	0.0	0.0	0.0	0.0	0.0
0.0	0.0	0.0	0.0	0.0	0.0	0.0	0.0
0.0	0.0	0.0	0.0	0.0	0.0	0.0	0.0
0.0	0.0	0.0	0.0	0.0	0.0	0.0	0.0
0.0	0.0	0.0	0.0	0.0	0.0	0.0	0.0
0.0	0.0	0.0	0.0	0.0	0.0	0.0	0.0
0.0	0.0	0.0	0.0	0.0	0.0	0.0	0.0
0.0	0.0	0.0	0.0	0.0	0.0	0.0	0.0
0.0	0.0	0.0	0.0	0.0	0.0	0.0	0.0
0.0	0.0	0.0	0.0	0.0	0.0	0.0	0.0
0.0	0.0	0.0	0.0	0.0	0.0	0.0	0.0
0.0	0.0	0.0	0.0	0.0	0.0	0.0	0.0
0.0	0.0	0.0	0.0	0.0	0.0	0.0	0.0
0.0	0.0	0.0	0.0	0.0	0.0	0.0	0.0

0.0	0.0	0.0	0.0	0.0	0.0	0.0	0.0
0.0	0.0	0.0	0.0	0.0	0.0	0.0	0.0
0.0	0.0	0.0	0.0	0.0	0.0	0.0	0.0
0.0	0.0	0.0	0.0	0.0	0.0	0.0	0.0
0.0	0.0	0.0	0.0	0.0	0.0	0.0	0.0
0.0	0.0	0.0	0.0	0.0	0.0	0.0	0.0
0.0	0.0	0.0	0.0	0.0	0.0	0.0	0.0
0.0	0.0	0.0	0.0	0.0	0.0	0.0	0.0
0.0	0.0	0.0	0.0	0.0	0.0	0.0	0.0
0.0	0.0	0.0	0.0	0.0	0.0	0.0	0.0
0.0	0.0	0.0	0.0	0.0	0.0	0.0	0.0
0.0	0.0	0.0	0.0	0.0	0.0	0.0	0.0
0.0	0.0	0.0	0.0	0.0	0.0	0.0	0.0
0.0	0.0	0.0	0.0	0.0	0.0	0.0	0.0
0.0	0.0	0.0	0.0	0.0	0.0	0.0	0.0
0.0	0.0	0.0	0.0	0.0	0.0	0.0	0.0
0.0	0.0	0.0	0.0	0.0	0.0	0.0	0.0
0.0	0.0	0.0	0.0	0.0	0.0	0.0	0.0
0.0	0.0	0.0	0.0	0.0	0.0	0.0	0.0
0.0	0.0	0.0	0.0	0.0	0.0	0.0	0.0
0.0	0.0	0.0	0.0	0.0	0.0	0.0	0.0
0.0	0.0	0.0	0.0	0.0	0.0	0.0	0.0
0.0	0.0	0.0	0.0	0.0	0.0	0.0	0.0
0.0	0.0	0.0	0.0	0.0	0.0	0.0	0.0
0.0	0.0	0.0	0.0	0.0	0.0	0.0	0.0
0.0	0.0	0.0	0.0	0.0	0.0	0.0	0.0
0.0	0.0	0.0	0.0	0.0	0.0	0.0	0.0
0.0	0.0	0.0	0.0	0.0	0.0	0.0	0.0
0.0	0.0	0.0	0.0	0.0	0.0	0.0	0.0
0.0	0.0	0.0	0.0	0.0	0.0	0.0	0.0
0.0	0.0	0.0	0.0	0.0	0.0	0.0	0.0
0.0	0.0	0.0	0.0	0.0	0.0	0.0	0.0
0.0	0.0	0.0	0.0	0.0	0.0	0.0	0.0
0.0	0.0	0.0	0.0	0.0	0.0	0.0	0.0
0.0	0.0	0.0	0.0	0.0	0.0	0.0	0.0
0.0	0.0	0.0	0.0	0.0	0.0	0.0	0.0
0.0	0.0	0.0	0.0	0.0	0.0	0.0	0.0
0.0	0.0	0.0	0.0	0.0	0.0	0.0	0.0
0.0	0.0	0.0	0.0	0.0	0.0	0.0	0.0
0.0	0.0	0.0	0.0	0.0	0.0	0.0	0.0
0.0	0.0	0.0	0.0	0.0	0.0	0.0	0.0
0.0	0.0	0.0	0.0	0.0	0.0	0.0	0.0
0.0	0.0	0.0	0.0	0.0	0.0	0.0	0.0
0.0	0.0	0.0	0.0	0.0	0.0	0.0	0.0
0.0	0.0	0.0	0.0	0.0	0.0	0.0	0.0
0.0	0.0	0.0	0.0	0.0	0.0	0.0	0.0
0.0	0.0	0.0	0.0	0.0	0.0	0.0	0.0
0.0	0.0	0.0	0.0	0.0	0.0	0.0	0.0

0.0	0.0	0.0	0.0	0.0	0.0	0.0	0.0
0.0	0.0	0.0	0.0	0.0	0.0	0.0	0.0
0.0	0.0	0.0	0.0	0.0	0.0	0.0	0.0
0.0	0.0	0.0	0.0	0.0	0.0	0.0	0.0
0.0	0.0	0.0	0.0	0.0	0.0	0.0	0.0
0.0	0.0	0.0	0.0	0.0	0.0	0.0	0.0
0.0	0.0	0.0	0.0	0.0	0.0	0.0	0.0
0.0	0.0	0.0	0.0	0.0	0.0	0.0	0.0
0.0	0.0	0.0	0.0	0.0	0.0	0.0	0.0
0.0	0.0	0.0	0.0	0.0	0.0	0.0	0.0
0.0	0.0	0.0	0.0	0.0	0.0	0.0	0.0
0.0	0.0	0.0	0.0	0.0	0.0	0.0	0.0
0.0	0.0	0.0	0.0	0.0	0.0	0.0	0.0
0.0	0.0	0.0	0.0	0.0	0.0	0.0	0.0
0.0	0.0	0.0	0.0	0.0	0.0	0.0	0.0
0.0	0.0	0.0	0.0	0.0	0.0	0.0	0.0
0.0	0.0	0.0	0.0	0.0	0.0	0.0	0.0
0.0	0.0	0.0	0.0	0.0	0.0	0.0	0.0
0.0	0.0	0.0	0.0	0.0	0.0	0.0	0.0
0.0	0.0	0.0	0.0	0.0	0.0	0.0	0.0
0.0	0.0	0.0	0.0	0.0	0.0	0.0	0.0
0.0	0.0	0.0	0.0	0.0	0.0	0.0	0.0
0.0	0.0	0.0	0.0	0.0	0.0	0.0	0.0
0.0	0.0	0.0	0.0	0.0	0.0	0.0	0.0
0.0	0.0	0.0	0.0	0.0	0.0	0.0	0.0
0.0	0.0	0.0	0.0	0.0	0.0	0.0	0.0
0.0	0.0	0.0	0.0	0.0	0.0	0.0	0.0
0.0	0.0	0.0	0.0	0.0	0.0	0.0	0.0
0.0	0.0	0.0	0.0	0.0	0.0	0.0	0.0
0.0	0.0	0.0	0.0	0.0	0.0	0.0	0.0
0.0	0.0	0.0	0.0	0.0	0.0	0.0	0.0
0.0	0.0	0.0	0.0	0.0	0.0	0.0	0.0
0.0	0.0	0.0	0.0	0.0	0.0	0.0	0.0
0.0	0.0	0.0	0.0	0.0	0.0	0.0	0.0
0.0	0.0	0.0	0.0	0.0	0.0	0.0	0.0
0.0	0.0	0.0	0.0	0.0	0.0	0.0	0.0
0.0	0.0	0.0	0.0	0.0	0.0	0.0	0.0
0.0	0.0	0.0	0.0	0.0	0.0	0.0	0.0
0.0	0.0	0.0	0.0	0.0	0.0	0.0	0.0
0.0	0.0	0.0	0.0	0.0	0.0	0.0	0.0
0.0	0.0	0.0	0.0	0.0	0.0	0.0	0.0
0.0	0.0	0.0	0.0	0.0	0.0	0.0	0.0
0.0	0.0	0.0	0.0	0.0	0.0	0.0	0.0
0.0	0.0	0.0	0.0	0.0	0.0	0.0	0.0
0.0	0.0	0.0	0.0	0.0	0.0	0.0	0.0
0.0	0.0	0.0	0.0	0.0	0.0	0.0	0.0
0.0	0.0	0.0	0.0	0.0	0.0	0.0	0.0

0.0	0.0	0.0	0.0	0.0	0.0	0.0	0.0
0.0	0.0	0.0	0.0	0.0	0.0	0.0	0.0
0.0	0.0	0.0	0.0	0.0	0.0	0.0	0.0
0.0	0.0	0.0	0.0	0.0	0.0	0.0	0.0
0.0	0.0	0.0	0.0	0.0	0.0	0.0	0.0
0.0	0.0	0.0	0.0	0.0	0.0	0.0	0.0
0.0	0.0	0.0	0.0	0.0	0.0	0.0	0.0
0.0	0.0	0.0	0.0	0.0	0.0	0.0	0.0
0.0	0.0	0.0	0.0	0.0	0.0	0.0	0.0
0.0	0.0	0.0	0.0	0.0	0.0	0.0	0.0
0.0	0.0	0.0	0.0	0.0	0.0	0.0	0.0
0.0	0.0	0.0	0.0	0.0	0.0	0.0	0.0
0.0	0.0	0.0	0.0	0.0	0.0	0.0	0.0
0.0	0.0	0.0	0.0	0.0	0.0	0.0	0.0
0.0	0.0	0.0	0.0	0.0	0.0	0.0	0.0
0.0	0.0	0.0	0.0	0.0	0.0	0.0	0.0
0.0	0.0	0.0	0.0	0.0	0.0	0.0	0.0
0.0	0.0	0.0	0.0	0.0	0.0	0.0	0.0
0.0	0.0	0.0	0.0	0.0	0.0	0.0	0.0
0.0	0.0	0.0	0.0	0.0	0.0	0.0	0.0
0.0	0.0	0.0	0.0	0.0	0.0	0.0	0.0
0.0	0.0	0.0	0.0	0.0	0.0	0.0	0.0
0.0	0.0	0.0	0.0	0.0	0.0	0.0	0.0
0.0	0.0	0.0	0.0	0.0	0.0	0.0	0.0
0.0	0.0	0.0	0.0	0.0	0.0	0.0	0.0
0.0	0.0	0.0	0.0	0.0	0.0	0.0	0.0
0.0	0.0	0.0	0.0	0.0	0.0	0.0	0.0
0.0	0.0	0.0	0.0	0.0	0.0	0.0	0.0
0.0	0.0	0.0	0.0	0.0	0.0	0.0	0.0
0.0	0.0	0.0	0.0	0.0	0.0	0.0	0.0
0.0	0.0	0.0	0.0	0.0	0.0	0.0	0.0
0.0	0.0	0.0	0.0	0.0	0.0	0.0	0.0
0.0	0.0	0.0	0.0	0.0	0.0	0.0	0.0
0.0	0.0	0.0	0.0	0.0	0.0	0.0	0.0
0.0	0.0	0.0	0.0	0.0	0.0	0.0	0.0
0.0	0.0	0.0	0.0	0.0	0.0	0.0	0.0
0.0	0.0	0.0	0.0	0.0	0.0	0.0	0.0
0.0	0.0	0.0	0.0	0.0	0.0	0.0	0.0
0.0	0.0	0.0	0.0	0.0	0.0	0.0	0.0
0.0	0.0	0.0	0.0	0.0	0.0	0.0	0.0
0.0	0.0	0.0	0.0	0.0	0.0	0.0	0.0
0.0	0.0	0.0	0.0	0.0	0.0	0.0	0.0
0.0	0.0	0.0	0.0	0.0	0.0	0.0	0.0
0.0	0.0	0.0	0.0	0.0	0.0	0.0	0.0
0.0	0.0	0.0	0.0	0.0	0.0	0.0	0.0
0.0	0.0	0.0	0.0	0.0	0.0	0.0	0.0
0.0	0.0	0.0	0.0	0.0	0.0	0.0	0.0

0.0	0.0	0.0	0.0	0.0	0.0	0.0	0.0
0.0	0.0	0.0	0.0	0.0	0.0	0.0	0.0
0.0	0.0	0.0	0.0	0.0	0.0	0.0	0.0
0.0	0.0	0.0	0.0	0.0	0.0	0.0	0.0
0.0	0.0	0.0	0.0	0.0	0.0	0.0	0.0
0.0	0.0	0.0	0.0	0.0	0.0	0.0	0.0
0.0	0.0	0.0	0.0	0.0	0.0	0.0	0.0
0.0	0.0	0.0	0.0	0.0	0.0	0.0	0.0
0.0	0.0	0.0	0.0	0.0	0.0	0.0	0.0
0.0	0.0	0.0	0.0	0.0	0.0	0.0	0.0
0.0	0.0	0.0	0.0	0.0	0.0	0.0	0.0
0.0	0.0	0.0	0.0	0.0	0.0	0.0	0.0
0.0	0.0	0.0	0.0	0.0	0.0	0.0	0.0
0.0	0.0	0.0	0.0	0.0	0.0	0.0	0.0
0.0	0.0	0.0	0.0	0.0	0.0	0.0	0.0
0.0	0.0	0.0	0.0	0.0	0.0	0.0	0.0
0.0	0.0	0.0	0.0	0.0	0.0	0.0	0.0
0.0	0.0	0.0	0.0	0.0	0.0	0.0	0.0
0.0	0.0	0.0	0.0	0.0	0.0	0.0	0.0
0.0	0.0	0.0	0.0	0.0	0.0	0.0	0.0
0.0	0.0	0.0	0.0	0.0	0.0	0.0	0.0
0.0	0.0	0.0	0.0	0.0	0.0	0.0	0.0
0.0	0.0	0.0	0.0	0.0	0.0	0.0	0.0
0.0	0.0	0.0	0.0	0.0	0.0	0.0	0.0
0.0	0.0	0.0	0.0	0.0	0.0	0.0	0.0
0.0	0.0	0.0	0.0	0.0	0.0	0.0	0.0
0.0	0.0	0.0	0.0	0.0	0.0	0.0	0.0
0.0	0.0	0.0	0.0	0.0	0.0	0.0	0.0
0.0	0.0	0.0	0.0	0.0	0.0	0.0	0.0
0.0	0.0	0.0	0.0	0.0	0.0	0.0	0.0
0.0	0.0	0.0	0.0	0.0	0.0	0.0	0.0
0.0	0.0	0.0	0.0	0.0	0.0	0.0	0.0
0.0	0.0	0.0	0.0	0.0	0.0	0.0	0.0
0.0	0.0	0.0	0.0	0.0	0.0	0.0	0.0
0.0	0.0	0.0	0.0	0.0	0.0	0.0	0.0
0.0	0.0	0.0	0.0	0.0	0.0	0.0	0.0
0.0	0.0	0.0	0.0	0.0	0.0	0.0	0.0
0.0	0.0	0.0	0.0	0.0	0.0	0.0	0.0
0.0	0.0	0.0	0.0	0.0	0.0	0.0	0.0
0.0	0.0	0.0	0.0	0.0	0.0	0.0	0.0
0.0	0.0	0.0	0.0	0.0	0.0	0.0	0.0
0.0	0.0	0.0	0.0	0.0	0.0	0.0	0.0
0.0	0.0	0.0	0.0	0.0	0.0	0.0	0.0
0.0	0.0	0.0	0.0	0.0	0.0	0.0	0.0
0.0	0.0	0.0	0.0	0.0	0.0	0.0	0.0
0.0	0.0	0.0	0.0	0.0	0.0	0.0	0.0
0.0	0.0	0.0	0.0	0.0	0.0	0.0	0.0

0.0	0.0	0.0	0.0	0.0	0.0	0.0	0.0
0.0	0.0	0.0	0.0	0.0	0.0	0.0	0.0
0.0	0.0	0.0	0.0	0.0	0.0	0.0	0.0
0.0	0.0	0.0	0.0	0.0	0.0	0.0	0.0
0.0	0.0	0.0	0.0	0.0	0.0	0.0	0.0
0.0	0.0	0.0	0.0	0.0	0.0	0.0	0.0
0.0	0.0	0.0	0.0	0.0	0.0	0.0	0.0
0.0	0.0	0.0	0.0	0.0	0.0	0.0	0.0
0.0	0.0	0.0	0.0	0.0	0.0	0.0	0.0
0.0	0.0	0.0	0.0	0.0	0.0	0.0	0.0
0.0	0.0	0.0	0.0	0.0	0.0	0.0	0.0
0.0	0.0	0.0	0.0	0.0	0.0	0.0	0.0
0.0	0.0	0.0	0.0	0.0	0.0	0.0	0.0
0.0	0.0	0.0	0.0	0.0	0.0	0.0	0.0
0.0	0.0	0.0	0.0	0.0	0.0	0.0	0.0
0.0	0.0	0.0	0.0	0.0	0.0	0.0	0.0
0.0	0.0	0.0	0.0	0.0	0.0	0.0	0.0
0.0	0.0	0.0	0.0	0.0	0.0	0.0	0.0
0.0	0.0	0.0	0.0	0.0	0.0	0.0	0.0
0.0	0.0	0.0	0.0	0.0	0.0	0.0	0.0
0.0	0.0	0.0	0.0	0.0	0.0	0.0	0.0
0.0	0.0	0.0	0.0	0.0	0.0	0.0	0.0
0.0	0.0	0.0	0.0	0.0	0.0	0.0	0.0
0.0	0.0	0.0	0.0	0.0	0.0	0.0	0.0
0.0	0.0	0.0	0.0	0.0	0.0	0.0	0.0
0.0	0.0	0.0	0.0	0.0	0.0	0.0	0.0
0.0	0.0	0.0	0.0	0.0	0.0	0.0	0.0
0.0	0.0	0.0	0.0	0.0	0.0	0.0	0.0
0.0	0.0	0.0	0.0	0.0	0.0	0.0	0.0
0.0	0.0	0.0	0.0	0.0	0.0	0.0	0.0
0.0	0.0	0.0	0.0	0.0	0.0	0.0	0.0
0.0	0.0	0.0	0.0	0.0	0.0	0.0	0.0
0.0	0.0	0.0	0.0	0.0	0.0	0.0	0.0
0.0	0.0	0.0	0.0	0.0	0.0	0.0	0.0
0.0	0.0	0.0	0.0	0.0	0.0	0.0	0.0
0.0	0.0	0.0	0.0	0.0	0.0	0.0	0.0
0.0	0.0	0.0	0.0	0.0	0.0	0.0	0.0
0.0	0.0	0.0	0.0	0.0	0.0	0.0	0.0
0.0	0.0	0.0	0.0	0.0	0.0	0.0	0.0
0.0	0.0	0.0	0.0	0.0	0.0	0.0	0.0
0.0	0.0	0.0	0.0	0.0	0.0	0.0	0.0
0.0	0.0	0.0	0.0	0.0	0.0	0.0	0.0
0.0	0.0	0.0	0.0	0.0	0.0	0.0	0.0
0.0	0.0	0.0	0.0	0.0	0.0	0.0	0.0
0.0	0.0	0.0	0.0	0.0	0.0	0.0	0.0
0.0	0.0	0.0	0.0	0.0	0.0	0.0	0.0
0.0	0.0	0.0	0.0	0.0	0.0	0.0	0.0

0.0	0.0	0.0	0.0	0.0	0.0	0.0	0.0
0.0	0.0	0.0	0.0	0.0	0.0	0.0	0.0
0.0	0.0	0.0	0.0	0.0	0.0	0.0	0.0
0.0	0.0	0.0	0.0	0.0	0.0	0.0	0.0
0.0	0.0	0.0	0.0	0.0	0.0	0.0	0.0
0.0	0.0	0.0	0.0	0.0	0.0	0.0	0.0
0.0	0.0	0.0	0.0	0.0	0.0	0.0	0.0
0.0	0.0	0.0	0.0	0.0	0.0	0.0	0.0
0.0	0.0	0.0	0.0	0.0	0.0	0.0	0.0
0.0	0.0	0.0	0.0	0.0	0.0	0.0	0.0
0.0	0.0	0.0	0.0	0.0	0.0	0.0	0.0
0.0	0.0	0.0	0.0	0.0	0.0	0.0	0.0
0.0	0.0	0.0	0.0	0.0	0.0	0.0	0.0
0.0	0.0	0.0	0.0	0.0	0.0	0.0	0.0
0.0	0.0	0.0	0.0	0.0	0.0	0.0	0.0
0.0	0.0	0.0	0.0	0.0	0.0	0.0	0.0
0.0	0.0	0.0	0.0	0.0	0.0	0.0	0.0
0.0	0.0	0.0	0.0	0.0	0.0	0.0	0.0
0.0	0.0	0.0	0.0	0.0	0.0	0.0	0.0
0.0	0.0	0.0	0.0	0.0	0.0	0.0	0.0
0.0	0.0	0.0	0.0	0.0	0.0	0.0	0.0
0.0	0.0	0.0	0.0	0.0	0.0	0.0	0.0
0.0	0.0	0.0	0.0	0.0	0.0	0.0	0.0
0.0	0.0	0.0	0.0	0.0	0.0	0.0	0.0
0.0	0.0	0.0	0.0	0.0	0.0	0.0	0.0
0.0	0.0	0.0	0.0	0.0	0.0	0.0	0.0
0.0	0.0	0.0	0.0	0.0	0.0	0.0	0.0
0.0	0.0	0.0	0.0	0.0	0.0	0.0	0.0
0.0	0.0	0.0	0.0	0.0	0.0	0.0	0.0
0.0	0.0	0.0	0.0	0.0	0.0	0.0	0.0
0.0	0.0	0.0	0.0	0.0	0.0	0.0	0.0
0.0	0.0	0.0	0.0	0.0	0.0	0.0	0.0
0.0	0.0	0.0	0.0	0.0	0.0	0.0	0.0
0.0	0.0	0.0	0.0	0.0	0.0	0.0	0.0
0.0	0.0	0.0	0.0	0.0	0.0	0.0	0.0
0.0	0.0	0.0	0.0	0.0	0.0	0.0	0.0
0.0	0.0	0.0	0.0	0.0	0.0	0.0	0.0
0.0	0.0	0.0	0.0	0.0	0.0	0.0	0.0
0.0	0.0	0.0	0.0	0.0	0.0	0.0	0.0
0.0	0.0	0.0	0.0	0.0	0.0	0.0	0.0
0.0	0.0	0.0	0.0	0.0	0.0	0.0	0.0
0.0	0.0	0.0	0.0	0.0	0.0	0.0	0.0
0.0	0.0	0.0	0.0	0.0	0.0	0.0	0.0
0.0	0.0	0.0	0.0	0.0	0.0	0.0	0.0
0.0	0.0	0.0	0.0	0.0	0.0	0.0	0.0
0.0	0.0	0.0	0.0	0.0	0.0	0.0	0.0
0.0	0.0	0.0	0.0	0.0	0.0	0.0	0.0

0.0	0.0	0.0	0.0	0.0	0.0	0.0	0.0
0.0	0.0	0.0	0.0	0.0	0.0	0.0	0.0
0.0	0.0	0.0	0.0	0.0	0.0	0.0	0.0
0.0	0.0	0.0	0.0	0.0	0.0	0.0	0.0
0.0	0.0	0.0	0.0	0.0	0.0	0.0	0.0
0.0	0.0	0.0	0.0	0.0	0.0	0.0	0.0
0.0	0.0	0.0	0.0	0.0	0.0	0.0	0.0
0.0	0.0	0.0	0.0	0.0	0.0	0.0	0.0
0.0	0.0	0.0	0.0	0.0	0.0	0.0	0.0
0.0	0.0	0.0	0.0	0.0	0.0	0.0	0.0
0.0	0.0	0.0	0.0	0.0	0.0	0.0	0.0
0.0	0.0	0.0	0.0	0.0	0.0	0.0	0.0
0.0	0.0	0.0	0.0	0.0	0.0	0.0	0.0
0.0	0.0	0.0	0.0	0.0	0.0	0.0	0.0
0.0	0.0	0.0	0.0	0.0	0.0	0.0	0.0
0.0	0.0	0.0	0.0	0.0	0.0	0.0	0.0
0.0	0.0	0.0	0.0	0.0	0.0	0.0	0.0
0.0	0.0	0.0	0.0	0.0	0.0	0.0	0.0
0.0	0.0	0.0	0.0	0.0	0.0	0.0	0.0
0.0	0.0	0.0	0.0	0.0	0.0	0.0	0.0
0.0	0.0	0.0	0.0	0.0	0.0	0.0	0.0
0.0	0.0	0.0	0.0	0.0	0.0	0.0	0.0
0.0	0.0	0.0	0.0	0.0	0.0	0.0	0.0
0.0	0.0	0.0	0.0	0.0	0.0	0.0	0.0
0.0	0.0	0.0	0.0	0.0	0.0	0.0	0.0
0.0	0.0	0.0	0.0	0.0	0.0	0.0	0.0
0.0	0.0	0.0	0.0	0.0	0.0	0.0	0.0
0.0	0.0	0.0	0.0	0.0	0.0	0.0	0.0
0.0	0.0	0.0	0.0	0.0	0.0	0.0	0.0
0.0	0.0	0.0	0.0	0.0	0.0	0.0	0.0
0.0	0.0	0.0	0.0	0.0	0.0	0.0	0.0
0.0	0.0	0.0	0.0	0.0	0.0	0.0	0.0
0.0	0.0	0.0	0.0	0.0	0.0	0.0	0.0
0.0	0.0	0.0	0.0	0.0	0.0	0.0	0.0
0.0	0.0	0.0	0.0	0.0	0.0	0.0	0.0
0.0	0.0	0.0	0.0	0.0	0.0	0.0	0.0
0.0	0.0	0.0	0.0	0.0	0.0	0.0	0.0
0.0	0.0	0.0	0.0	0.0	0.0	0.0	0.0
0.0	0.0	0.0	0.0	0.0	0.0	0.0	0.0
0.0	0.0	0.0	0.0	0.0	0.0	0.0	0.0
0.0	0.0	0.0	0.0	0.0	0.0	0.0	0.0
0.0	0.0	0.0	0.0	0.0	0.0	0.0	0.0
0.0	0.0	0.0	0.0	0.0	0.0	0.0	0.0
0.0	0.0	0.0	0.0	0.0	0.0	0.0	0.0
0.0	0.0	0.0	0.0	0.0	0.0	0.0	0.0
0.0	0.0	0.0	0.0	0.0	0.0	0.0	0.0
0.0	0.0	0.0	0.0	0.0	0.0	0.0	0.0

0.0	0.0	0.0	0.0	0.0	0.0	0.0	0.0
0.0	0.0	0.0	0.0	0.0	0.0	0.0	0.0
0.0	0.0	0.0	0.0	0.0	0.0	0.0	0.0
0.0	0.0	0.0	0.0	0.0	0.0	0.0	0.0
0.0	0.0	0.0	0.0	0.0	0.0	0.0	0.0
0.0	0.0	0.0	0.0	0.0	0.0	0.0	0.0
0.0	0.0	0.0	0.0	0.0	0.0	0.0	0.0
0.0	0.0	0.0	0.0	0.0	0.0	0.0	0.0
0.0	0.0	0.0	0.0	0.0	0.0	0.0	0.0
0.0	0.0	0.0	0.0	0.0	0.0	0.0	0.0
0.0	0.0	0.0	0.0	0.0	0.0	0.0	0.0
0.0	0.0	0.0	0.0	0.0	0.0	0.0	0.0
0.0	0.0	0.0	0.0	0.0	0.0	0.0	0.0
0.0	0.0	0.0	0.0	0.0	0.0	0.0	0.0
0.0	0.0	0.0	0.0	0.0	0.0	0.0	0.0
0.0	0.0	0.0	0.0	0.0	0.0	0.0	0.0
0.0	0.0	0.0	0.0	0.0	0.0	0.0	0.0
0.0	0.0	0.0	0.0	0.0	0.0	0.0	0.0
0.0	0.0	0.0	0.0	0.0	0.0	0.0	0.0
0.0	0.0	0.0	0.0	0.0	0.0	0.0	0.0
0.0	0.0	0.0	0.0	0.0	0.0	0.0	0.0
0.0	0.0	0.0	0.0	0.0	0.0	0.0	0.0
0.0	0.0	0.0	0.0	0.0	0.0	0.0	0.0
33	23	22	58	56	28	33	55
1	0	0	0	1	0	0	1
21.5	26.3	30.3	57.6	41.87327824	34.8026792	103.1381951	33.1

11635.TW.CK.	11635.CIR51.(	11635.CIR5.0(	11635.TW.BQ	11635.TW.DH	11635.TW.BA	11635.TW.AK	11635.CIR16.(
1.0	33.0	2.0	5.0	1039.0	8.0	1.0	0.0
0.0	0.0	553.0	40.0	0.0	0.0	0.0	17.0
4.0	0.0	0.0	0.0	0.0	1.0	1.0	1520.0
64.0	0.0	0.0	396.0	0.0	141.0	351.0	0.0
11.0	22.0	61.0	170.0	0.0	2.0	38.0	0.0
68.0	8.0	26.0	993.0	0.0	148.0	803.0	1273.0
0.0	0.0	0.0	0.0	0.0	0.0	0.0	0.0
179.0	0.0	0.0	0.0	0.0	0.0	0.0	0.0
731.0	14.0	0.0	0.0	0.0	66.0	321.0	0.0
16.0	0.0	0.0	209.0	0.0	15.0	250.0	802.0
1.0	0.0	212.0	76.0	0.0	1.0	0.0	1.0
27.0	0.0	0.0	0.0	0.0	0.0	0.0	0.0
0.0	2397.0	0.0	0.0	0.0	0.0	3.0	0.0
986.0	27.0	0.0	0.0	165.0	8.0	3.0	1.0
0.0	4.0	0.0	1.0	0.0	20.0	0.0	0.0
21.0	91.0	116.0	53.0	1.0	2.0	130.0	0.0
27.0	0.0	0.0	0.0	0.0	0.0	65.0	143.0
2424.0	6.0	0.0	0.0	257.0	0.0	0.0	5.0
41.0	47.0	1398.0	155.0	1.0	31.0	33.0	716.0
35.0	36.0	0.0	166.0	0.0	54.0	481.0	175.0
81.0	186.0	0.0	89.0	0.0	76.0	12.0	0.0
184.0	73.0	0.0	0.0	0.0	4009.0	228.0	0.0
29.0	0.0	0.0	19.0	0.0	8.0	30.0	61.0
0.0	0.0	2.0	138.0	0.0	0.0	0.0	0.0
0.0	34.0	0.0	0.0	0.0	0.0	0.0	0.0
29.0	12.0	77.0	20.0	0.0	8.0	36.0	156.0
0.0	0.0	0.0	0.0	0.0	1.0	0.0	0.0
1.0	0.0	0.0	0.0	0.0	0.0	345.0	0.0
7.0	0.0	0.0	0.0	0.0	3.0	0.0	0.0
8.0	22.0	0.0	0.0	0.0	0.0	12.0	0.0
28.0	4.0	0.0	32.0	1.0	9.0	11.0	243.0
249.0	5.0	0.0	6.0	0.0	21.0	11.0	455.0
10.0	0.0	674.0	0.0	0.0	0.0	0.0	0.0
1.0	0.0	0.0	0.0	0.0	4.0	11.0	0.0
145.0	27.0	0.0	0.0	11.0	18.0	54.0	88.0
0.0	0.0	0.0	0.0	0.0	0.0	0.0	0.0
7.0	0.0	0.0	116.0	0.0	0.0	0.0	0.0
16.0	21.0	0.0	13.0	0.0	42.0	47.0	209.0
8.0	23.0	0.0	0.0	0.0	93.0	0.0	0.0
21.0	8.0	0.0	45.0	0.0	45.0	7.0	89.0
7.0	0.0	0.0	0.0	0.0	0.0	4.0	2.0
12.0	0.0	0.0	0.0	0.0	9.0	38.0	0.0
20.0	11.0	0.0	9.0	0.0	4.0	0.0	216.0
13.0	3.0	0.0	165.0	2.0	14.0	21.0	385.0
0.0	187.0	21.0	1.0	0.0	2.0	1.0	37.0
192.0	12.0	0.0	2.0	35.0	69.0	47.0	272.0

346.0	0.0	2.0	6.0	5.0	23.0	2.0	747.0
0.0	0.0	0.0	226.0	0.0	0.0	0.0	0.0
0.0	0.0	0.0	0.0	0.0	2.0	0.0	0.0
0.0	0.0	0.0	0.0	0.0	0.0	0.0	0.0
0.0	0.0	0.0	0.0	0.0	0.0	0.0	5.0
2.0	4.0	0.0	14.0	0.0	5.0	1.0	0.0
5.0	0.0	0.0	182.0	0.0	9.0	33.0	3.0
0.0	0.0	0.0	0.0	0.0	0.0	0.0	0.0
0.0	0.0	0.0	0.0	0.0	45.0	0.0	0.0
0.0	0.0	0.0	0.0	0.0	0.0	0.0	0.0
0.0	0.0	0.0	0.0	0.0	0.0	0.0	0.0
2.0	0.0	0.0	0.0	1.0	2.0	0.0	0.0
5.0	0.0	0.0	0.0	0.0	0.0	0.0	0.0
0.0	0.0	0.0	0.0	0.0	0.0	0.0	0.0
13.0	0.0	0.0	143.0	0.0	49.0	25.0	0.0
0.0	0.0	0.0	0.0	0.0	0.0	0.0	0.0
0.0	0.0	0.0	0.0	0.0	0.0	0.0	0.0
0.0	0.0	0.0	0.0	0.0	0.0	0.0	0.0
11.0	39.0	0.0	0.0	0.0	11.0	10.0	52.0
0.0	0.0	0.0	0.0	0.0	2.0	0.0	0.0
0.0	0.0	0.0	0.0	0.0	0.0	0.0	0.0
0.0	0.0	0.0	0.0	0.0	0.0	0.0	0.0
0.0	0.0	0.0	0.0	0.0	4.0	0.0	0.0
22.0	18.0	0.0	99.0	0.0	17.0	58.0	27.0
4.0	0.0	0.0	0.0	0.0	0.0	0.0	0.0
7.0	0.0	0.0	92.0	0.0	6.0	5.0	1.0
0.0	0.0	0.0	0.0	0.0	0.0	0.0	0.0
0.0	0.0	0.0	0.0	0.0	0.0	0.0	0.0
0.0	0.0	0.0	5.0	0.0	0.0	0.0	0.0
0.0	0.0	0.0	0.0	0.0	0.0	0.0	0.0
0.0	0.0	0.0	0.0	0.0	0.0	0.0	0.0
0.0	0.0	0.0	0.0	0.0	0.0	0.0	0.0
0.0	0.0	0.0	0.0	0.0	0.0	0.0	0.0
0.0	0.0	0.0	0.0	0.0	0.0	0.0	0.0
23.0	0.0	0.0	96.0	131.0	7.0	14.0	1.0
7.0	0.0	0.0	0.0	0.0	1.0	0.0	0.0
2.0	16.0	0.0	181.0	0.0	0.0	0.0	0.0
0.0	0.0	0.0	0.0	0.0	0.0	0.0	0.0
52.0	0.0	0.0	0.0	13.0	21.0	15.0	0.0
0.0	289.0	3.0	0.0	0.0	3.0	1.0	0.0
1.0	0.0	0.0	0.0	0.0	1.0	1.0	0.0
0.0	0.0	0.0	0.0	0.0	0.0	0.0	0.0
3.0	38.0	18.0	0.0	0.0	0.0	41.0	0.0
9.0	0.0	0.0	0.0	0.0	0.0	9.0	0.0
15.0	7.0	0.0	0.0	0.0	31.0	1.0	0.0
0.0	0.0	0.0	0.0	0.0	0.0	0.0	0.0
0.0	0.0	0.0	7.0	0.0	4.0	0.0	4.0

3.0	34.0	0.0	21.0	0.0	16.0	13.0	63.0
2.0	0.0	0.0	52.0	0.0	8.0	6.0	0.0
189.0	49.0	18.0	4.0	54.0	0.0	121.0	84.0
0.0	0.0	0.0	0.0	0.0	0.0	0.0	0.0
17.0	0.0	8.0	0.0	0.0	0.0	1.0	1.0
0.0	0.0	0.0	0.0	0.0	0.0	0.0	0.0
8.0	3.0	0.0	79.0	0.0	0.0	18.0	0.0
0.0	0.0	0.0	0.0	0.0	0.0	0.0	0.0
7.0	899.0	0.0	146.0	0.0	11.0	16.0	2.0
0.0	0.0	0.0	62.0	0.0	0.0	0.0	0.0
7.0	0.0	0.0	0.0	0.0	11.0	1.0	0.0
0.0	0.0	0.0	232.0	0.0	0.0	0.0	0.0
2.0	0.0	0.0	0.0	0.0	3.0	0.0	0.0
4.0	0.0	0.0	0.0	0.0	0.0	2.0	0.0
7.0	23.0	13.0	434.0	0.0	1.0	10.0	0.0
0.0	0.0	0.0	0.0	0.0	0.0	0.0	0.0
0.0	0.0	0.0	0.0	0.0	0.0	0.0	0.0
0.0	0.0	0.0	0.0	0.0	0.0	0.0	0.0
0.0	0.0	0.0	0.0	0.0	32.0	0.0	0.0
0.0	0.0	0.0	0.0	0.0	0.0	0.0	0.0
6.0	2.0	0.0	0.0	0.0	63.0	29.0	81.0
4.0	0.0	0.0	37.0	0.0	0.0	21.0	6.0
0.0	0.0	0.0	0.0	0.0	0.0	8.0	0.0
72.0	0.0	0.0	0.0	0.0	10.0	0.0	0.0
0.0	0.0	0.0	0.0	0.0	0.0	62.0	0.0
0.0	0.0	0.0	0.0	0.0	0.0	0.0	0.0
0.0	0.0	14.0	0.0	0.0	22.0	10.0	0.0
0.0	0.0	0.0	0.0	0.0	82.0	221.0	0.0
0.0	1.0	0.0	200.0	1.0	154.0	4.0	0.0
32.0	0.0	1.0	40.0	1.0	0.0	0.0	0.0
0.0	0.0	0.0	0.0	0.0	0.0	1.0	0.0
0.0	0.0	0.0	0.0	0.0	0.0	0.0	0.0
7.0	0.0	0.0	0.0	0.0	0.0	0.0	0.0
0.0	0.0	0.0	0.0	0.0	0.0	0.0	0.0
0.0	0.0	0.0	0.0	0.0	0.0	0.0	0.0
2.0	0.0	0.0	0.0	0.0	32.0	4.0	0.0
0.0	0.0	0.0	0.0	0.0	0.0	0.0	0.0
1.0	4.0	0.0	0.0	0.0	2.0	6.0	17.0
1.0	39.0	4.0	0.0	0.0	79.0	71.0	0.0
0.0	0.0	0.0	0.0	0.0	0.0	0.0	0.0
0.0	0.0	0.0	0.0	0.0	0.0	0.0	0.0
0.0	0.0	7.0	11.0	0.0	1.0	31.0	0.0
1.0	0.0	0.0	87.0	0.0	0.0	0.0	0.0
0.0	0.0	0.0	1.0	0.0	0.0	0.0	0.0
0.0	0.0	0.0	0.0	0.0	0.0	3.0	0.0
0.0	0.0	0.0	0.0	0.0	0.0	0.0	0.0
0.0	4.0	40.0	46.0	0.0	1.0	8.0	0.0

0.0	0.0	0.0	0.0	0.0	0.0	0.0	0.0
1.0	0.0	0.0	0.0	0.0	5.0	0.0	0.0
0.0	0.0	0.0	0.0	0.0	0.0	0.0	0.0
0.0	0.0	0.0	0.0	0.0	0.0	0.0	0.0
2.0	25.0	0.0	0.0	0.0	3.0	3.0	0.0
0.0	0.0	0.0	0.0	0.0	0.0	0.0	0.0
0.0	0.0	0.0	0.0	0.0	0.0	0.0	0.0
0.0	3.0	0.0	0.0	0.0	0.0	0.0	0.0
1.0	13.0	3.0	6.0	0.0	0.0	3.0	7.0
0.0	0.0	0.0	0.0	0.0	0.0	0.0	0.0
2.0	11.0	0.0	0.0	0.0	4.0	9.0	0.0
0.0	49.0	0.0	25.0	0.0	13.0	2.0	0.0
0.0	0.0	0.0	2.0	0.0	6.0	8.0	0.0
0.0	0.0	0.0	0.0	0.0	0.0	0.0	0.0
13.0	0.0	0.0	0.0	0.0	0.0	0.0	126.0
8.0	0.0	0.0	0.0	0.0	0.0	6.0	0.0
0.0	0.0	0.0	0.0	0.0	0.0	0.0	0.0
0.0	14.0	0.0	12.0	0.0	0.0	0.0	0.0
12.0	0.0	0.0	0.0	0.0	0.0	10.0	0.0
0.0	8.0	0.0	0.0	0.0	1.0	0.0	16.0
0.0	0.0	0.0	0.0	0.0	0.0	0.0	0.0
2.0	0.0	0.0	51.0	0.0	7.0	0.0	0.0
0.0	4.0	2.0	0.0	0.0	2.0	1.0	0.0
2.0	0.0	0.0	0.0	0.0	3.0	3.0	0.0
0.0	0.0	0.0	0.0	0.0	0.0	0.0	0.0
0.0	8.0	0.0	0.0	0.0	52.0	0.0	0.0
0.0	0.0	0.0	0.0	0.0	0.0	0.0	0.0
0.0	0.0	0.0	5.0	0.0	3.0	21.0	0.0
0.0	0.0	0.0	0.0	0.0	2.0	1.0	0.0
0.0	0.0	0.0	0.0	0.0	0.0	0.0	0.0
0.0	0.0	1.0	4.0	0.0	0.0	15.0	0.0
0.0	0.0	0.0	0.0	0.0	2.0	0.0	0.0
0.0	0.0	0.0	0.0	0.0	0.0	0.0	32.0
4.0	0.0	0.0	0.0	0.0	0.0	0.0	0.0
0.0	0.0	0.0	22.0	0.0	2.0	8.0	0.0
1.0	0.0	0.0	0.0	0.0	0.0	0.0	0.0
0.0	4.0	0.0	49.0	2.0	0.0	0.0	0.0
0.0	0.0	0.0	22.0	0.0	0.0	0.0	0.0
0.0	35.0	0.0	12.0	0.0	1.0	1.0	0.0
5.0	1.0	0.0	0.0	0.0	6.0	160.0	0.0
0.0	0.0	0.0	2.0	0.0	0.0	35.0	15.0
10.0	0.0	78.0	0.0	0.0	15.0	20.0	0.0
3.0	0.0	23.0	12.0	0.0	4.0	9.0	0.0
0.0	0.0	0.0	0.0	0.0	0.0	0.0	0.0
4.0	68.0	74.0	55.0	0.0	0.0	0.0	13.0
0.0	0.0	0.0	0.0	0.0	1.0	2.0	0.0
0.0	0.0	0.0	0.0	0.0	0.0	0.0	0.0

0.0	0.0	0.0	0.0	0.0	0.0	0.0	0.0
0.0	0.0	0.0	6.0	0.0	0.0	0.0	0.0
0.0	0.0	0.0	19.0	0.0	0.0	0.0	0.0
0.0	0.0	0.0	7.0	0.0	5.0	16.0	0.0
0.0	0.0	0.0	7.0	0.0	4.0	0.0	0.0
0.0	0.0	0.0	0.0	0.0	0.0	0.0	0.0
2.0	3.0	0.0	0.0	0.0	1.0	0.0	6.0
0.0	25.0	0.0	12.0	0.0	0.0	0.0	0.0
2.0	58.0	4.0	4.0	0.0	0.0	0.0	3.0
0.0	0.0	0.0	0.0	0.0	0.0	0.0	0.0
0.0	14.0	0.0	0.0	0.0	4.0	3.0	0.0
2.0	0.0	0.0	4.0	0.0	0.0	6.0	0.0
0.0	0.0	0.0	0.0	0.0	0.0	0.0	0.0
0.0	30.0	247.0	3.0	0.0	0.0	0.0	4.0
0.0	0.0	0.0	7.0	0.0	0.0	0.0	0.0
2.0	0.0	0.0	0.0	0.0	1.0	0.0	0.0
0.0	0.0	0.0	0.0	0.0	0.0	0.0	0.0
0.0	0.0	0.0	0.0	0.0	0.0	0.0	0.0
0.0	0.0	0.0	0.0	0.0	0.0	0.0	0.0
0.0	4.0	0.0	1.0	0.0	0.0	0.0	0.0
13.0	0.0	0.0	20.0	0.0	13.0	0.0	0.0
0.0	0.0	0.0	0.0	0.0	7.0	0.0	1.0
0.0	0.0	0.0	0.0	0.0	0.0	0.0	0.0
1.0	2.0	0.0	1.0	0.0	2.0	6.0	2.0
0.0	0.0	0.0	0.0	0.0	0.0	0.0	0.0
0.0	0.0	0.0	0.0	0.0	0.0	0.0	0.0
4.0	0.0	0.0	13.0	0.0	0.0	0.0	0.0
0.0	0.0	0.0	0.0	0.0	0.0	0.0	0.0
0.0	0.0	0.0	3.0	0.0	0.0	0.0	0.0
0.0	0.0	0.0	0.0	0.0	0.0	0.0	0.0
0.0	0.0	0.0	0.0	0.0	0.0	0.0	0.0
0.0	0.0	0.0	2.0	0.0	0.0	0.0	0.0
0.0	0.0	0.0	0.0	0.0	0.0	0.0	0.0
0.0	0.0	0.0	0.0	0.0	0.0	0.0	8.0
0.0	0.0	0.0	0.0	0.0	0.0	0.0	0.0
0.0	0.0	0.0	0.0	0.0	3.0	0.0	0.0
0.0	0.0	0.0	0.0	0.0	0.0	0.0	0.0
0.0	0.0	0.0	0.0	0.0	0.0	0.0	0.0
0.0	0.0	0.0	0.0	0.0	0.0	0.0	0.0
0.0	0.0	0.0	0.0	0.0	4.0	0.0	0.0
0.0	27.0	3.0	4.0	0.0	0.0	3.0	0.0
0.0	0.0	0.0	4.0	0.0	0.0	0.0	0.0
0.0	0.0	0.0	0.0	0.0	0.0	0.0	0.0
0.0	0.0	0.0	0.0	0.0	0.0	0.0	0.0
0.0	0.0	0.0	0.0	0.0	0.0	0.0	0.0
0.0	0.0	0.0	0.0	0.0	0.0	0.0	0.0
0.0	0.0	0.0	0.0	0.0	0.0	0.0	0.0

0.0	1.0	0.0	0.0	0.0	0.0	0.0	0.0
1.0	0.0	0.0	0.0	0.0	0.0	3.0	0.0
13.0	0.0	0.0	0.0	0.0	0.0	0.0	0.0
0.0	0.0	0.0	7.0	0.0	0.0	0.0	0.0
0.0	0.0	0.0	0.0	0.0	0.0	0.0	0.0
0.0	0.0	0.0	0.0	0.0	0.0	0.0	0.0
0.0	0.0	0.0	0.0	0.0	0.0	1.0	0.0
0.0	0.0	0.0	4.0	0.0	0.0	0.0	0.0
0.0	0.0	0.0	0.0	0.0	0.0	0.0	0.0
0.0	0.0	0.0	0.0	0.0	0.0	0.0	0.0
0.0	0.0	0.0	63.0	0.0	0.0	0.0	0.0
0.0	0.0	0.0	0.0	0.0	0.0	0.0	0.0
0.0	0.0	1.0	18.0	0.0	0.0	0.0	0.0
0.0	0.0	0.0	27.0	0.0	0.0	0.0	0.0
0.0	0.0	0.0	0.0	0.0	1.0	0.0	0.0
0.0	0.0	0.0	234.0	0.0	0.0	0.0	0.0
0.0	0.0	0.0	2.0	0.0	0.0	0.0	0.0
0.0	0.0	0.0	0.0	0.0	0.0	0.0	0.0
0.0	2.0	0.0	0.0	0.0	0.0	0.0	0.0
0.0	0.0	0.0	0.0	0.0	0.0	0.0	0.0
3.0	0.0	0.0	0.0	0.0	6.0	5.0	0.0
0.0	0.0	0.0	14.0	0.0	0.0	0.0	0.0
0.0	0.0	0.0	3.0	0.0	0.0	0.0	0.0
0.0	0.0	0.0	1.0	0.0	0.0	0.0	0.0
0.0	0.0	0.0	0.0	0.0	0.0	0.0	0.0
0.0	0.0	0.0	0.0	0.0	0.0	0.0	0.0
0.0	0.0	3.0	2.0	0.0	1.0	1.0	0.0
0.0	0.0	0.0	0.0	0.0	0.0	0.0	0.0
0.0	3.0	0.0	0.0	0.0	0.0	0.0	0.0
0.0	0.0	0.0	12.0	0.0	0.0	0.0	0.0
0.0	0.0	0.0	0.0	0.0	0.0	0.0	0.0
0.0	0.0	0.0	0.0	0.0	0.0	0.0	7.0
0.0	0.0	0.0	0.0	0.0	0.0	0.0	0.0
0.0	0.0	0.0	3.0	0.0	0.0	0.0	0.0
0.0	0.0	0.0	1.0	0.0	0.0	0.0	0.0
0.0	0.0	0.0	0.0	0.0	0.0	24.0	0.0
0.0	0.0	0.0	16.0	0.0	0.0	0.0	0.0
0.0	0.0	0.0	29.0	0.0	0.0	0.0	0.0
0.0	0.0	0.0	0.0	0.0	0.0	0.0	0.0
0.0	0.0	0.0	0.0	0.0	0.0	0.0	0.0
0.0	0.0	0.0	0.0	0.0	0.0	0.0	0.0
0.0	0.0	3.0	0.0	0.0	0.0	0.0	0.0
0.0	13.0	0.0	0.0	0.0	0.0	0.0	0.0
0.0	114.0	145.0	8.0	0.0	0.0	0.0	0.0
0.0	0.0	0.0	0.0	0.0	0.0	0.0	0.0
0.0	0.0	0.0	4.0	0.0	0.0	0.0	0.0
0.0	0.0	0.0	0.0	0.0	0.0	0.0	0.0

0.0	0.0	0.0	0.0	0.0	0.0	0.0	0.0
0.0	0.0	0.0	78.0	0.0	0.0	0.0	0.0
0.0	0.0	0.0	3.0	0.0	0.0	0.0	0.0
0.0	0.0	0.0	0.0	0.0	0.0	0.0	0.0
0.0	0.0	0.0	0.0	0.0	0.0	0.0	0.0
0.0	0.0	0.0	0.0	0.0	0.0	0.0	0.0
0.0	5.0	0.0	7.0	0.0	0.0	0.0	0.0
0.0	0.0	0.0	0.0	0.0	0.0	0.0	0.0
0.0	0.0	0.0	0.0	0.0	0.0	0.0	0.0
0.0	0.0	0.0	0.0	0.0	0.0	0.0	0.0
6.0	0.0	0.0	3.0	0.0	0.0	0.0	2.0
1.0	0.0	0.0	0.0	0.0	0.0	0.0	0.0
0.0	0.0	0.0	0.0	0.0	0.0	0.0	0.0
0.0	0.0	0.0	0.0	0.0	0.0	0.0	0.0
0.0	0.0	0.0	0.0	0.0	5.0	12.0	0.0
74.0	7.0	0.0	0.0	0.0	0.0	11.0	18.0
0.0	0.0	0.0	0.0	0.0	0.0	0.0	0.0
0.0	0.0	2.0	73.0	0.0	0.0	0.0	0.0
0.0	0.0	0.0	0.0	0.0	0.0	0.0	0.0
0.0	9.0	3.0	0.0	0.0	0.0	0.0	0.0
0.0	0.0	0.0	0.0	0.0	0.0	0.0	0.0
0.0	0.0	0.0	3.0	0.0	0.0	0.0	0.0
0.0	0.0	0.0	0.0	0.0	0.0	0.0	0.0
0.0	0.0	0.0	0.0	0.0	0.0	0.0	0.0
1.0	0.0	0.0	18.0	0.0	2.0	1.0	3.0
0.0	0.0	0.0	0.0	0.0	0.0	0.0	0.0
0.0	0.0	2.0	2.0	1.0	1.0	2.0	317.0
0.0	0.0	0.0	26.0	0.0	0.0	0.0	0.0
0.0	0.0	0.0	1.0	0.0	0.0	0.0	0.0
0.0	0.0	0.0	0.0	0.0	0.0	0.0	0.0
0.0	0.0	0.0	0.0	0.0	0.0	0.0	0.0
0.0	0.0	0.0	0.0	0.0	0.0	0.0	0.0
0.0	0.0	0.0	0.0	0.0	0.0	0.0	0.0
0.0	0.0	0.0	6.0	0.0	0.0	0.0	0.0
0.0	2.0	0.0	0.0	0.0	0.0	0.0	0.0
0.0	0.0	0.0	70.0	0.0	0.0	0.0	0.0
0.0	0.0	0.0	0.0	0.0	0.0	0.0	0.0
0.0	0.0	0.0	0.0	0.0	0.0	0.0	0.0
0.0	0.0	0.0	0.0	0.0	0.0	0.0	0.0
0.0	0.0	0.0	0.0	0.0	0.0	0.0	0.0
0.0	1.0	0.0	1.0	0.0	0.0	0.0	0.0
0.0	0.0	0.0	1.0	0.0	0.0	0.0	0.0
0.0	0.0	0.0	0.0	0.0	0.0	0.0	0.0
0.0	0.0	1.0	2.0	0.0	0.0	0.0	0.0
0.0	0.0	0.0	0.0	0.0	0.0	0.0	0.0
0.0	0.0	0.0	0.0	0.0	0.0	0.0	0.0
0.0	9.0	0.0	0.0	0.0	0.0	0.0	0.0

0.0	0.0	0.0	0.0	0.0	0.0	0.0	0.0
0.0	0.0	0.0	0.0	0.0	0.0	0.0	0.0
0.0	0.0	0.0	0.0	0.0	0.0	0.0	0.0
0.0	0.0	0.0	0.0	0.0	0.0	0.0	0.0
0.0	11.0	0.0	0.0	0.0	0.0	0.0	0.0
0.0	0.0	0.0	0.0	0.0	0.0	0.0	0.0
0.0	0.0	0.0	0.0	0.0	0.0	0.0	0.0
0.0	0.0	0.0	0.0	0.0	0.0	0.0	0.0
0.0	0.0	4.0	0.0	0.0	0.0	0.0	1.0
0.0	0.0	0.0	0.0	0.0	0.0	0.0	0.0
0.0	0.0	0.0	0.0	0.0	0.0	0.0	0.0
0.0	0.0	0.0	0.0	0.0	0.0	0.0	0.0
0.0	0.0	0.0	0.0	0.0	0.0	0.0	0.0
0.0	0.0	0.0	0.0	0.0	0.0	0.0	0.0
0.0	2.0	0.0	0.0	0.0	0.0	0.0	0.0
0.0	0.0	0.0	0.0	0.0	0.0	0.0	0.0
0.0	6.0	11.0	27.0	0.0	0.0	0.0	3.0
0.0	0.0	0.0	0.0	0.0	0.0	0.0	0.0
0.0	0.0	0.0	0.0	0.0	0.0	0.0	0.0
0.0	0.0	0.0	0.0	0.0	0.0	0.0	0.0
0.0	0.0	0.0	2.0	0.0	0.0	2.0	0.0
0.0	0.0	0.0	0.0	0.0	0.0	0.0	0.0
0.0	0.0	0.0	0.0	0.0	0.0	0.0	0.0
0.0	0.0	0.0	0.0	0.0	0.0	0.0	0.0
0.0	0.0	0.0	0.0	0.0	0.0	0.0	0.0
0.0	0.0	0.0	0.0	0.0	0.0	0.0	0.0
0.0	0.0	0.0	0.0	0.0	0.0	0.0	0.0
0.0	0.0	78.0	0.0	0.0	0.0	0.0	0.0
0.0	0.0	0.0	0.0	0.0	0.0	0.0	0.0
0.0	0.0	0.0	0.0	0.0	0.0	0.0	0.0
0.0	0.0	0.0	0.0	0.0	0.0	0.0	0.0
0.0	0.0	0.0	0.0	0.0	0.0	0.0	0.0
0.0	0.0	0.0	0.0	0.0	0.0	0.0	0.0
0.0	0.0	0.0	2.0	0.0	0.0	0.0	0.0
0.0	0.0	12.0	0.0	0.0	0.0	0.0	6.0
0.0	0.0	0.0	0.0	0.0	0.0	0.0	0.0
0.0	0.0	0.0	0.0	0.0	1.0	0.0	0.0
0.0	0.0	0.0	8.0	0.0	0.0	0.0	0.0
0.0	0.0	0.0	2.0	0.0	0.0	0.0	0.0
0.0	0.0	0.0	0.0	0.0	0.0	0.0	0.0
0.0	0.0	0.0	0.0	0.0	0.0	0.0	0.0
0.0	0.0	0.0	0.0	0.0	0.0	0.0	0.0
0.0	0.0	0.0	0.0	0.0	0.0	0.0	0.0
0.0	0.0	0.0	0.0	0.0	0.0	0.0	0.0
0.0	0.0	0.0	0.0	0.0	0.0	0.0	3.0
0.0	0.0	0.0	4.0	0.0	0.0	0.0	0.0
0.0	0.0	0.0	0.0	0.0	0.0	0.0	0.0

0.0	1.0	0.0	0.0	0.0	0.0	0.0	0.0
4.0	11.0	0.0	0.0	0.0	0.0	1.0	0.0
0.0	0.0	0.0	0.0	0.0	0.0	0.0	0.0
0.0	0.0	1.0	0.0	0.0	0.0	0.0	3.0
0.0	0.0	0.0	0.0	0.0	0.0	0.0	0.0
6.0	0.0	29.0	0.0	0.0	1.0	0.0	0.0
0.0	0.0	0.0	0.0	0.0	0.0	0.0	0.0
0.0	0.0	0.0	0.0	0.0	0.0	0.0	0.0
0.0	0.0	0.0	1.0	0.0	0.0	0.0	0.0
0.0	0.0	0.0	4.0	0.0	0.0	0.0	0.0
0.0	0.0	0.0	5.0	0.0	0.0	0.0	0.0
0.0	0.0	3.0	5.0	0.0	0.0	0.0	0.0
0.0	0.0	0.0	86.0	0.0	0.0	0.0	0.0
2.0	0.0	0.0	0.0	0.0	0.0	0.0	0.0
0.0	0.0	0.0	0.0	0.0	0.0	0.0	0.0
0.0	0.0	0.0	0.0	0.0	3.0	0.0	0.0
0.0	0.0	0.0	0.0	0.0	2.0	3.0	0.0
0.0	0.0	0.0	0.0	0.0	0.0	0.0	0.0
0.0	0.0	0.0	0.0	0.0	0.0	0.0	0.0
0.0	0.0	0.0	0.0	0.0	0.0	0.0	0.0
0.0	0.0	0.0	0.0	0.0	0.0	0.0	0.0
0.0	0.0	0.0	0.0	0.0	0.0	0.0	0.0
0.0	0.0	0.0	0.0	0.0	0.0	0.0	0.0
2.0	853.0	1.0	0.0	0.0	0.0	0.0	0.0
0.0	0.0	1.0	0.0	0.0	0.0	0.0	2.0
0.0	0.0	0.0	0.0	0.0	0.0	0.0	0.0
0.0	0.0	0.0	0.0	0.0	2.0	0.0	0.0
0.0	0.0	0.0	0.0	0.0	0.0	0.0	0.0
0.0	0.0	0.0	0.0	0.0	0.0	0.0	0.0
1.0	0.0	0.0	0.0	0.0	2.0	17.0	0.0
0.0	0.0	0.0	0.0	0.0	0.0	0.0	0.0
0.0	0.0	0.0	0.0	0.0	0.0	0.0	0.0
0.0	0.0	1.0	0.0	0.0	0.0	0.0	0.0
0.0	0.0	0.0	0.0	0.0	0.0	0.0	0.0
0.0	0.0	0.0	0.0	0.0	0.0	0.0	0.0
0.0	0.0	0.0	1.0	0.0	0.0	2.0	0.0
0.0	0.0	0.0	121.0	0.0	0.0	0.0	0.0
0.0	0.0	0.0	0.0	0.0	0.0	0.0	0.0
2.0	0.0	0.0	0.0	0.0	0.0	0.0	0.0
0.0	0.0	0.0	0.0	0.0	0.0	0.0	0.0
0.0	0.0	1578.0	0.0	0.0	1.0	0.0	0.0
2.0	175.0	0.0	378.0	0.0	0.0	33.0	0.0
0.0	0.0	0.0	0.0	0.0	0.0	0.0	0.0
0.0	0.0	0.0	0.0	0.0	0.0	0.0	0.0
0.0	0.0	0.0	0.0	0.0	0.0	0.0	0.0
0.0	0.0	0.0	0.0	0.0	0.0	0.0	0.0
0.0	2.0	2.0	1.0	3.0	2.0	0.0	1.0

0.0	0.0	0.0	0.0	0.0	0.0	0.0	0.0
0.0	0.0	0.0	0.0	0.0	0.0	0.0	0.0
0.0	0.0	3.0	0.0	0.0	7.0	0.0	27.0
0.0	3.0	6.0	1.0	0.0	0.0	0.0	0.0
0.0	0.0	0.0	0.0	0.0	0.0	0.0	0.0
0.0	0.0	0.0	0.0	0.0	0.0	0.0	0.0
0.0	0.0	0.0	0.0	0.0	0.0	0.0	0.0
0.0	0.0	0.0	0.0	0.0	0.0	0.0	1.0
0.0	0.0	0.0	0.0	0.0	0.0	0.0	0.0
0.0	0.0	0.0	0.0	0.0	0.0	0.0	0.0
0.0	0.0	0.0	0.0	0.0	2.0	0.0	1.0
0.0	0.0	0.0	0.0	0.0	0.0	0.0	0.0
0.0	0.0	0.0	5.0	1.0	0.0	0.0	0.0
0.0	0.0	0.0	0.0	0.0	0.0	0.0	0.0
0.0	0.0	0.0	0.0	0.0	0.0	0.0	0.0
0.0	0.0	0.0	0.0	0.0	0.0	0.0	0.0
0.0	0.0	0.0	0.0	0.0	0.0	0.0	0.0
0.0	0.0	0.0	0.0	0.0	0.0	0.0	0.0
0.0	170.0	0.0	0.0	0.0	0.0	0.0	0.0
0.0	0.0	0.0	0.0	0.0	0.0	0.0	0.0
0.0	0.0	0.0	121.0	0.0	0.0	0.0	0.0
1.0	0.0	0.0	0.0	0.0	0.0	0.0	0.0
0.0	0.0	0.0	0.0	0.0	0.0	0.0	0.0
5.0	0.0	0.0	0.0	0.0	0.0	0.0	0.0
0.0	0.0	0.0	0.0	0.0	0.0	0.0	0.0
0.0	0.0	4.0	0.0	0.0	0.0	0.0	0.0
0.0	0.0	0.0	0.0	0.0	0.0	0.0	0.0
0.0	0.0	0.0	0.0	0.0	0.0	0.0	0.0
0.0	7.0	0.0	0.0	0.0	0.0	0.0	0.0
0.0	0.0	0.0	0.0	0.0	0.0	2.0	0.0
0.0	0.0	0.0	0.0	0.0	7.0	0.0	0.0
0.0	0.0	0.0	0.0	0.0	0.0	0.0	0.0
0.0	0.0	0.0	0.0	0.0	0.0	0.0	0.0
0.0	0.0	0.0	0.0	0.0	0.0	0.0	0.0
0.0	0.0	0.0	2.0	0.0	0.0	0.0	0.0
7.0	0.0	0.0	0.0	0.0	45.0	0.0	0.0
1.0	3.0	0.0	0.0	0.0	1.0	7.0	9.0
0.0	0.0	0.0	0.0	0.0	0.0	0.0	0.0
76.0	0.0	0.0	0.0	2.0	0.0	0.0	0.0
1.0	0.0	0.0	0.0	0.0	0.0	1.0	0.0
0.0	0.0	0.0	0.0	0.0	0.0	0.0	0.0
0.0	0.0	0.0	9.0	0.0	0.0	1.0	0.0
0.0	0.0	0.0	2.0	0.0	0.0	0.0	0.0
0.0	0.0	0.0	0.0	0.0	0.0	0.0	0.0
2.0	0.0	0.0	99.0	0.0	2.0	2.0	0.0
0.0	0.0	3.0	4.0	0.0	0.0	0.0	0.0
0.0	0.0	0.0	0.0	0.0	4.0	0.0	4.0

0.0	0.0	0.0	0.0	0.0	0.0	0.0	0.0
0.0	0.0	0.0	0.0	0.0	0.0	0.0	0.0
0.0	0.0	0.0	0.0	0.0	0.0	0.0	0.0
1.0	0.0	0.0	0.0	782.0	0.0	1.0	0.0
0.0	1.0	0.0	0.0	0.0	0.0	0.0	0.0
0.0	0.0	0.0	0.0	0.0	0.0	0.0	0.0
0.0	3.0	0.0	0.0	0.0	0.0	0.0	0.0
0.0	0.0	0.0	0.0	0.0	0.0	0.0	0.0
0.0	0.0	0.0	0.0	0.0	0.0	0.0	0.0
0.0	0.0	0.0	0.0	0.0	0.0	0.0	0.0
0.0	0.0	0.0	0.0	0.0	0.0	0.0	0.0
0.0	0.0	0.0	0.0	0.0	2.0	0.0	0.0
0.0	6.0	0.0	0.0	0.0	0.0	0.0	0.0
0.0	0.0	0.0	0.0	2.0	0.0	0.0	0.0
0.0	0.0	0.0	1.0	0.0	1.0	2.0	1.0
0.0	0.0	0.0	0.0	0.0	0.0	0.0	0.0
0.0	0.0	0.0	0.0	0.0	0.0	0.0	0.0
0.0	0.0	0.0	0.0	0.0	0.0	0.0	0.0
0.0	0.0	0.0	0.0	0.0	0.0	0.0	0.0
0.0	0.0	0.0	0.0	0.0	0.0	0.0	0.0
0.0	0.0	0.0	0.0	0.0	0.0	0.0	0.0
0.0	7.0	5.0	2.0	3.0	0.0	0.0	0.0
0.0	0.0	0.0	0.0	0.0	0.0	0.0	0.0
0.0	0.0	0.0	0.0	0.0	0.0	0.0	0.0
0.0	0.0	0.0	1.0	0.0	0.0	1.0	0.0
0.0	0.0	0.0	0.0	0.0	0.0	0.0	0.0
0.0	0.0	0.0	0.0	0.0	0.0	0.0	0.0
0.0	2.0	37.0	0.0	0.0	0.0	0.0	0.0
0.0	0.0	0.0	0.0	0.0	0.0	0.0	0.0
0.0	0.0	0.0	0.0	0.0	0.0	0.0	0.0
0.0	0.0	0.0	3.0	0.0	0.0	0.0	0.0
0.0	0.0	0.0	0.0	0.0	0.0	1.0	0.0
0.0	0.0	0.0	0.0	0.0	0.0	0.0	0.0
0.0	0.0	0.0	0.0	0.0	0.0	0.0	0.0
0.0	0.0	0.0	1.0	0.0	0.0	0.0	0.0
0.0	0.0	0.0	0.0	0.0	0.0	0.0	0.0
0.0	0.0	0.0	0.0	0.0	0.0	0.0	0.0
0.0	0.0	0.0	0.0	0.0	0.0	0.0	0.0
0.0	0.0	0.0	0.0	0.0	0.0	0.0	1.0
0.0	0.0	0.0	0.0	0.0	0.0	0.0	0.0
0.0	0.0	0.0	0.0	0.0	0.0	0.0	0.0
0.0	2.0	0.0	0.0	0.0	0.0	0.0	0.0
0.0	10.0	3.0	0.0	0.0	0.0	0.0	0.0
0.0	0.0	0.0	0.0	0.0	0.0	0.0	0.0
0.0	0.0	0.0	1.0	0.0	0.0	0.0	0.0
0.0	0.0	0.0	0.0	0.0	0.0	0.0	0.0
0.0	0.0	0.0	0.0	0.0	0.0	0.0	0.0

0.0	0.0	6.0	0.0	0.0	0.0	0.0	1.0
0.0	0.0	0.0	0.0	0.0	0.0	0.0	0.0
0.0	0.0	0.0	0.0	0.0	0.0	0.0	0.0
6.0	0.0	1.0	0.0	1.0	0.0	0.0	10.0
0.0	12.0	0.0	0.0	0.0	0.0	0.0	0.0
0.0	0.0	0.0	0.0	0.0	0.0	0.0	0.0
0.0	0.0	0.0	0.0	0.0	0.0	0.0	0.0
0.0	0.0	0.0	1.0	0.0	0.0	0.0	0.0
0.0	0.0	0.0	0.0	0.0	0.0	0.0	0.0
0.0	0.0	0.0	0.0	0.0	0.0	0.0	0.0
0.0	0.0	0.0	0.0	0.0	0.0	0.0	0.0
0.0	0.0	0.0	0.0	0.0	0.0	0.0	0.0
0.0	0.0	0.0	0.0	0.0	0.0	0.0	0.0
0.0	0.0	0.0	0.0	0.0	0.0	0.0	0.0
0.0	0.0	0.0	0.0	0.0	0.0	0.0	0.0
0.0	0.0	0.0	0.0	0.0	0.0	0.0	0.0
0.0	0.0	0.0	0.0	0.0	0.0	0.0	0.0
0.0	0.0	0.0	0.0	0.0	0.0	0.0	0.0
0.0	0.0	0.0	0.0	0.0	0.0	0.0	0.0
0.0	0.0	0.0	13.0	0.0	0.0	0.0	0.0
0.0	0.0	0.0	0.0	0.0	0.0	0.0	0.0
0.0	0.0	0.0	0.0	0.0	0.0	0.0	0.0
0.0	0.0	0.0	0.0	0.0	0.0	0.0	0.0
0.0	0.0	0.0	0.0	0.0	0.0	0.0	0.0
0.0	0.0	0.0	0.0	0.0	0.0	0.0	0.0
0.0	0.0	0.0	0.0	5109.0	0.0	0.0	0.0
0.0	0.0	0.0	0.0	0.0	0.0	0.0	0.0
0.0	0.0	0.0	0.0	0.0	0.0	0.0	0.0
0.0	0.0	0.0	0.0	0.0	0.0	0.0	0.0
0.0	0.0	0.0	0.0	0.0	0.0	0.0	0.0
0.0	0.0	0.0	0.0	0.0	0.0	0.0	0.0
0.0	0.0	0.0	0.0	0.0	0.0	0.0	0.0
0.0	0.0	0.0	0.0	0.0	0.0	0.0	0.0
0.0	0.0	0.0	0.0	0.0	0.0	0.0	0.0
0.0	0.0	0.0	0.0	0.0	0.0	0.0	0.0
0.0	0.0	0.0	0.0	0.0	0.0	0.0	0.0
0.0	0.0	0.0	0.0	0.0	0.0	0.0	0.0
0.0	0.0	0.0	0.0	0.0	0.0	0.0	0.0
0.0	0.0	0.0	0.0	0.0	0.0	0.0	0.0
0.0	2.0	2.0	4.0	0.0	0.0	0.0	0.0
0.0	0.0	0.0	0.0	0.0	0.0	0.0	0.0
0.0	0.0	0.0	0.0	0.0	0.0	0.0	0.0
0.0	0.0	0.0	3.0	0.0	0.0	0.0	0.0
0.0	0.0	0.0	0.0	0.0	0.0	0.0	0.0
0.0	0.0	0.0	0.0	0.0	0.0	0.0	0.0
0.0	0.0	0.0	0.0	0.0	0.0	0.0	0.0
0.0	0.0	0.0	0.0	0.0	0.0	0.0	0.0

0.0	0.0	0.0	0.0	0.0	0.0	0.0	0.0
0.0	0.0	0.0	0.0	0.0	0.0	0.0	0.0
0.0	0.0	0.0	0.0	0.0	0.0	0.0	0.0
0.0	0.0	0.0	0.0	0.0	0.0	0.0	0.0
0.0	0.0	0.0	0.0	0.0	0.0	0.0	0.0
0.0	0.0	0.0	0.0	0.0	0.0	0.0	0.0
0.0	0.0	49.0	0.0	0.0	0.0	0.0	0.0
0.0	1.0	3.0	1.0	0.0	0.0	0.0	6.0
0.0	0.0	43.0	0.0	0.0	0.0	0.0	0.0
0.0	0.0	0.0	0.0	0.0	0.0	0.0	0.0
0.0	1.0	0.0	0.0	0.0	0.0	0.0	2.0
0.0	0.0	0.0	0.0	0.0	0.0	0.0	0.0
0.0	0.0	0.0	0.0	0.0	0.0	0.0	0.0
0.0	3.0	27.0	1.0	0.0	0.0	0.0	4.0
0.0	0.0	0.0	0.0	0.0	0.0	0.0	0.0
0.0	0.0	1.0	0.0	0.0	0.0	0.0	0.0
0.0	0.0	0.0	0.0	0.0	1.0	0.0	0.0
0.0	0.0	0.0	0.0	0.0	0.0	0.0	0.0
0.0	0.0	0.0	0.0	0.0	0.0	0.0	5.0
0.0	0.0	0.0	0.0	0.0	0.0	0.0	0.0
0.0	0.0	2907.0	0.0	0.0	0.0	0.0	0.0
0.0	0.0	0.0	0.0	0.0	0.0	0.0	0.0
0.0	0.0	0.0	0.0	0.0	0.0	0.0	0.0
0.0	0.0	0.0	0.0	0.0	0.0	0.0	1.0
0.0	0.0	0.0	0.0	0.0	0.0	0.0	0.0
2.0	0.0	0.0	0.0	0.0	0.0	0.0	3.0
0.0	0.0	0.0	0.0	0.0	0.0	0.0	0.0
0.0	0.0	0.0	0.0	0.0	0.0	0.0	0.0
0.0	0.0	0.0	0.0	0.0	0.0	0.0	0.0
0.0	0.0	0.0	0.0	0.0	0.0	0.0	0.0
0.0	0.0	0.0	0.0	0.0	0.0	0.0	0.0
0.0	0.0	0.0	0.0	0.0	0.0	0.0	0.0
0.0	0.0	0.0	0.0	0.0	0.0	0.0	0.0
0.0	0.0	0.0	0.0	0.0	0.0	0.0	0.0
0.0	0.0	0.0	0.0	0.0	0.0	0.0	0.0
0.0	0.0	0.0	0.0	0.0	0.0	0.0	0.0
0.0	0.0	0.0	1.0	0.0	0.0	0.0	0.0
0.0	0.0	0.0	0.0	0.0	0.0	0.0	0.0
0.0	0.0	0.0	0.0	0.0	0.0	0.0	0.0
0.0	0.0	0.0	0.0	0.0	0.0	0.0	5.0
0.0	0.0	0.0	0.0	0.0	0.0	0.0	0.0
0.0	0.0	0.0	0.0	0.0	0.0	0.0	0.0
9.0	0.0	0.0	0.0	0.0	0.0	0.0	0.0
0.0	0.0	0.0	0.0	0.0	0.0	0.0	0.0
0.0	0.0	0.0	0.0	0.0	0.0	0.0	0.0
0.0	0.0	0.0	0.0	0.0	5.0	0.0	0.0
0.0	1.0	0.0	0.0	0.0	0.0	0.0	0.0

0.0	0.0	0.0	0.0	0.0	0.0	0.0	0.0
0.0	0.0	0.0	0.0	0.0	0.0	16.0	0.0
0.0	0.0	0.0	0.0	0.0	0.0	0.0	0.0
0.0	0.0	0.0	0.0	0.0	0.0	0.0	0.0
0.0	0.0	10.0	2.0	0.0	0.0	0.0	0.0
0.0	0.0	0.0	0.0	0.0	0.0	0.0	0.0
0.0	0.0	0.0	0.0	0.0	0.0	0.0	0.0
0.0	0.0	0.0	0.0	0.0	0.0	0.0	0.0
0.0	0.0	0.0	0.0	0.0	0.0	1.0	0.0
0.0	0.0	0.0	0.0	0.0	0.0	0.0	0.0
0.0	3.0	0.0	0.0	0.0	0.0	0.0	0.0
0.0	0.0	0.0	3.0	0.0	0.0	0.0	0.0
0.0	0.0	0.0	2.0	0.0	0.0	0.0	0.0
0.0	0.0	0.0	0.0	0.0	0.0	0.0	3.0
0.0	17.0	0.0	1.0	0.0	0.0	0.0	0.0
0.0	0.0	0.0	0.0	0.0	0.0	0.0	0.0
0.0	0.0	0.0	0.0	0.0	2.0	0.0	0.0
0.0	4.0	0.0	0.0	0.0	0.0	0.0	0.0
0.0	0.0	0.0	0.0	0.0	0.0	0.0	0.0
0.0	0.0	0.0	0.0	0.0	0.0	0.0	0.0
0.0	0.0	0.0	0.0	0.0	0.0	0.0	0.0
0.0	0.0	0.0	0.0	0.0	0.0	0.0	0.0
23.0	0.0	0.0	237.0	0.0	51.0	0.0	0.0
0.0	0.0	0.0	0.0	0.0	0.0	0.0	0.0
0.0	0.0	48.0	0.0	0.0	0.0	0.0	0.0
0.0	0.0	0.0	0.0	0.0	0.0	0.0	0.0
0.0	0.0	0.0	0.0	0.0	0.0	0.0	0.0
0.0	0.0	0.0	0.0	0.0	0.0	0.0	0.0
0.0	0.0	0.0	0.0	0.0	0.0	3.0	0.0
1.0	0.0	0.0	0.0	0.0	0.0	0.0	0.0
0.0	0.0	0.0	24.0	0.0	0.0	0.0	0.0
0.0	16.0	0.0	0.0	0.0	0.0	0.0	0.0
0.0	0.0	0.0	0.0	0.0	0.0	0.0	0.0
0.0	0.0	0.0	0.0	0.0	0.0	0.0	0.0
0.0	0.0	0.0	0.0	0.0	0.0	0.0	0.0
0.0	0.0	0.0	0.0	0.0	0.0	0.0	0.0
0.0	0.0	0.0	0.0	0.0	0.0	0.0	0.0
0.0	0.0	0.0	16.0	0.0	0.0	0.0	0.0
0.0	0.0	0.0	0.0	0.0	0.0	0.0	0.0
0.0	0.0	0.0	21.0	0.0	0.0	0.0	0.0
0.0	0.0	0.0	0.0	0.0	0.0	0.0	0.0
0.0	0.0	0.0	0.0	0.0	0.0	0.0	0.0
0.0	0.0	0.0	15.0	0.0	0.0	2.0	0.0
0.0	0.0	0.0	0.0	0.0	0.0	0.0	0.0
0.0	0.0	0.0	0.0	0.0	0.0	0.0	0.0
0.0	0.0	0.0	3.0	0.0	0.0	0.0	0.0
0.0	0.0	7.0	0.0	0.0	0.0	0.0	0.0

0.0	3.0	0.0	0.0	0.0	0.0	0.0	0.0
0.0	0.0	0.0	0.0	0.0	0.0	0.0	0.0
0.0	0.0	0.0	0.0	0.0	0.0	0.0	0.0
0.0	0.0	0.0	1.0	0.0	0.0	0.0	0.0
0.0	0.0	0.0	0.0	0.0	0.0	0.0	0.0
0.0	0.0	0.0	0.0	0.0	0.0	0.0	0.0
0.0	0.0	0.0	0.0	0.0	0.0	0.0	0.0
0.0	0.0	0.0	0.0	0.0	0.0	0.0	0.0
1.0	0.0	0.0	0.0	0.0	5.0	2.0	0.0
0.0	15.0	0.0	1.0	0.0	0.0	0.0	0.0
0.0	0.0	0.0	0.0	0.0	0.0	0.0	0.0
0.0	0.0	0.0	1.0	0.0	0.0	0.0	0.0
0.0	0.0	0.0	2.0	0.0	0.0	0.0	0.0
0.0	0.0	0.0	0.0	0.0	0.0	0.0	0.0
0.0	0.0	0.0	0.0	0.0	0.0	0.0	0.0
0.0	0.0	0.0	0.0	0.0	0.0	1.0	0.0
0.0	0.0	0.0	0.0	0.0	0.0	0.0	0.0
0.0	0.0	0.0	0.0	0.0	0.0	0.0	0.0
0.0	0.0	0.0	0.0	0.0	0.0	0.0	0.0
0.0	0.0	0.0	0.0	0.0	0.0	0.0	0.0
0.0	0.0	0.0	0.0	0.0	0.0	0.0	0.0
0.0	0.0	0.0	1.0	0.0	0.0	0.0	0.0
0.0	0.0	0.0	0.0	0.0	0.0	0.0	0.0
0.0	0.0	0.0	0.0	0.0	0.0	0.0	0.0
0.0	0.0	0.0	0.0	0.0	0.0	0.0	0.0
0.0	0.0	0.0	0.0	0.0	0.0	0.0	0.0
0.0	0.0	0.0	0.0	0.0	0.0	0.0	0.0
0.0	0.0	0.0	0.0	0.0	0.0	0.0	0.0
0.0	1.0	0.0	0.0	0.0	0.0	0.0	0.0
0.0	0.0	0.0	0.0	0.0	0.0	0.0	0.0
0.0	0.0	0.0	0.0	0.0	0.0	0.0	0.0
0.0	0.0	0.0	0.0	0.0	0.0	0.0	0.0
0.0	0.0	0.0	0.0	0.0	0.0	0.0	0.0
0.0	0.0	0.0	0.0	0.0	0.0	0.0	0.0
0.0	0.0	5.0	0.0	0.0	8.0	0.0	0.0
0.0	0.0	0.0	0.0	0.0	0.0	0.0	0.0
0.0	0.0	1.0	1.0	0.0	0.0	0.0	0.0
0.0	0.0	0.0	0.0	0.0	0.0	0.0	0.0
0.0	0.0	0.0	0.0	0.0	0.0	0.0	0.0
0.0	0.0	0.0	0.0	0.0	0.0	0.0	0.0
0.0	0.0	0.0	0.0	0.0	0.0	0.0	0.0
0.0	0.0	0.0	0.0	0.0	0.0	0.0	0.0
0.0	0.0	0.0	0.0	0.0	0.0	0.0	0.0
0.0	0.0	0.0	0.0	0.0	0.0	0.0	0.0
0.0	0.0	0.0	0.0	0.0	0.0	0.0	0.0
0.0	0.0	0.0	0.0	0.0	0.0	0.0	0.0
0.0	0.0	0.0	0.0	0.0	0.0	0.0	0.0

0.0	0.0	0.0	0.0	0.0	0.0	0.0	0.0
0.0	0.0	0.0	0.0	0.0	0.0	0.0	0.0
0.0	0.0	0.0	0.0	0.0	0.0	0.0	0.0
0.0	0.0	0.0	0.0	0.0	0.0	0.0	0.0
0.0	0.0	0.0	0.0	0.0	0.0	0.0	0.0
0.0	0.0	0.0	0.0	0.0	0.0	0.0	0.0
0.0	0.0	0.0	0.0	0.0	0.0	0.0	0.0
0.0	0.0	0.0	0.0	0.0	0.0	0.0	0.0
0.0	0.0	0.0	0.0	0.0	0.0	0.0	0.0
0.0	0.0	0.0	0.0	0.0	0.0	0.0	0.0
0.0	0.0	0.0	0.0	0.0	0.0	0.0	0.0
0.0	0.0	0.0	0.0	0.0	0.0	0.0	0.0
0.0	0.0	0.0	0.0	0.0	0.0	0.0	0.0
0.0	0.0	0.0	0.0	0.0	0.0	0.0	0.0
0.0	0.0	0.0	0.0	0.0	0.0	0.0	0.0
0.0	0.0	0.0	0.0	0.0	0.0	0.0	0.0
0.0	0.0	0.0	0.0	0.0	0.0	0.0	0.0
0.0	0.0	0.0	0.0	0.0	49.0	0.0	0.0
0.0	0.0	0.0	0.0	0.0	0.0	0.0	0.0
0.0	0.0	0.0	0.0	0.0	0.0	0.0	0.0
0.0	0.0	0.0	0.0	0.0	0.0	0.0	0.0
0.0	0.0	0.0	0.0	0.0	0.0	0.0	0.0
0.0	0.0	0.0	0.0	0.0	0.0	0.0	0.0
0.0	0.0	0.0	0.0	0.0	0.0	0.0	0.0
0.0	0.0	0.0	0.0	0.0	0.0	0.0	0.0
0.0	0.0	0.0	1.0	0.0	134.0	0.0	0.0
0.0	0.0	0.0	0.0	0.0	0.0	0.0	0.0
0.0	0.0	0.0	0.0	0.0	0.0	0.0	0.0
0.0	0.0	0.0	0.0	0.0	0.0	0.0	0.0
0.0	0.0	0.0	0.0	0.0	0.0	0.0	0.0
0.0	0.0	0.0	0.0	0.0	0.0	0.0	0.0
0.0	0.0	0.0	0.0	0.0	0.0	110.0	0.0
0.0	0.0	0.0	0.0	0.0	0.0	0.0	0.0
0.0	0.0	0.0	0.0	0.0	0.0	0.0	0.0
0.0	0.0	0.0	0.0	0.0	0.0	21.0	0.0
0.0	0.0	0.0	0.0	0.0	0.0	0.0	0.0
0.0	0.0	0.0	0.0	0.0	0.0	0.0	0.0
0.0	0.0	0.0	0.0	0.0	0.0	0.0	0.0
0.0	0.0	0.0	0.0	0.0	0.0	0.0	0.0
28.0	0.0	0.0	1.0	0.0	0.0	0.0	0.0
0.0	0.0	0.0	0.0	0.0	0.0	0.0	0.0
0.0	0.0	0.0	0.0	0.0	0.0	0.0	0.0
0.0	0.0	0.0	0.0	0.0	0.0	0.0	0.0
0.0	0.0	0.0	0.0	0.0	0.0	0.0	0.0
6.0	0.0	0.0	0.0	0.0	1.0	0.0	0.0
0.0	0.0	0.0	0.0	0.0	0.0	0.0	0.0
85.0	0.0	0.0	0.0	0.0	0.0	7.0	0.0

0.0	0.0	0.0	0.0	0.0	0.0	0.0	0.0
0.0	0.0	0.0	0.0	0.0	1.0	0.0	0.0
0.0	0.0	0.0	0.0	0.0	0.0	0.0	0.0
0.0	0.0	0.0	0.0	0.0	0.0	0.0	0.0
0.0	0.0	0.0	0.0	0.0	0.0	0.0	0.0
0.0	0.0	0.0	0.0	0.0	0.0	0.0	0.0
0.0	0.0	0.0	0.0	0.0	0.0	0.0	0.0
0.0	0.0	0.0	0.0	0.0	0.0	0.0	0.0
0.0	0.0	0.0	0.0	0.0	0.0	0.0	0.0
0.0	0.0	0.0	0.0	0.0	0.0	0.0	0.0
0.0	0.0	0.0	0.0	0.0	0.0	0.0	0.0
0.0	0.0	0.0	0.0	0.0	0.0	0.0	0.0
0.0	0.0	0.0	0.0	0.0	31.0	0.0	0.0
0.0	3.0	0.0	0.0	0.0	0.0	0.0	0.0
0.0	2.0	0.0	0.0	0.0	0.0	0.0	0.0
0.0	0.0	0.0	0.0	0.0	0.0	0.0	0.0
0.0	0.0	0.0	0.0	0.0	0.0	0.0	0.0
0.0	0.0	0.0	0.0	0.0	0.0	0.0	0.0
0.0	0.0	0.0	0.0	0.0	0.0	0.0	0.0
0.0	0.0	0.0	1.0	0.0	0.0	0.0	0.0
0.0	0.0	0.0	0.0	0.0	0.0	0.0	0.0
0.0	0.0	0.0	0.0	0.0	0.0	0.0	0.0
0.0	0.0	0.0	0.0	0.0	0.0	0.0	0.0
0.0	0.0	0.0	10.0	0.0	3.0	0.0	0.0
0.0	0.0	0.0	2.0	0.0	0.0	0.0	0.0
0.0	6.0	0.0	0.0	0.0	0.0	0.0	0.0
0.0	0.0	0.0	0.0	0.0	0.0	0.0	0.0
0.0	0.0	0.0	0.0	0.0	0.0	0.0	0.0
0.0	0.0	0.0	0.0	0.0	0.0	0.0	0.0
0.0	0.0	0.0	0.0	0.0	0.0	0.0	0.0
0.0	0.0	0.0	0.0	0.0	0.0	0.0	0.0
0.0	0.0	0.0	0.0	0.0	0.0	0.0	0.0
0.0	0.0	0.0	0.0	0.0	0.0	0.0	0.0
0.0	0.0	0.0	0.0	0.0	0.0	0.0	0.0
0.0	0.0	0.0	0.0	0.0	0.0	0.0	0.0
0.0	0.0	0.0	0.0	0.0	0.0	0.0	0.0
0.0	0.0	0.0	0.0	0.0	0.0	0.0	0.0
0.0	0.0	0.0	0.0	0.0	0.0	0.0	0.0
0.0	0.0	0.0	4.0	0.0	0.0	0.0	0.0
0.0	0.0	0.0	0.0	0.0	0.0	0.0	0.0
0.0	0.0	0.0	0.0	0.0	0.0	0.0	0.0
0.0	0.0	0.0	2.0	0.0	0.0	0.0	0.0
0.0	0.0	0.0	0.0	0.0	0.0	0.0	0.0
0.0	0.0	0.0	0.0	0.0	0.0	0.0	0.0
0.0	0.0	0.0	0.0	0.0	2.0	0.0	0.0
0.0	0.0	0.0	0.0	0.0	0.0	0.0	0.0
0.0	0.0	0.0	0.0	0.0	0.0	0.0	0.0

0.0	0.0	0.0	0.0	0.0	0.0	0.0	0.0
0.0	0.0	0.0	0.0	0.0	0.0	0.0	0.0
0.0	0.0	0.0	0.0	0.0	0.0	0.0	0.0
0.0	0.0	0.0	0.0	0.0	0.0	0.0	0.0
0.0	0.0	0.0	0.0	0.0	0.0	0.0	0.0
0.0	0.0	0.0	0.0	0.0	0.0	0.0	0.0
0.0	0.0	0.0	0.0	0.0	0.0	0.0	0.0
0.0	0.0	0.0	0.0	0.0	0.0	0.0	0.0
0.0	0.0	0.0	0.0	0.0	0.0	0.0	0.0
0.0	0.0	0.0	0.0	0.0	0.0	0.0	0.0
0.0	0.0	0.0	0.0	0.0	2.0	0.0	0.0
0.0	0.0	0.0	0.0	0.0	0.0	0.0	0.0
0.0	0.0	0.0	0.0	0.0	0.0	0.0	0.0
0.0	0.0	0.0	0.0	0.0	0.0	0.0	0.0
0.0	0.0	0.0	0.0	0.0	0.0	0.0	0.0
0.0	0.0	0.0	0.0	0.0	0.0	0.0	0.0
0.0	0.0	0.0	0.0	0.0	0.0	0.0	0.0
0.0	0.0	0.0	0.0	0.0	0.0	0.0	0.0
0.0	0.0	0.0	0.0	0.0	0.0	0.0	0.0
0.0	0.0	0.0	0.0	0.0	0.0	0.0	0.0
0.0	0.0	0.0	0.0	0.0	1.0	0.0	0.0
0.0	0.0	0.0	0.0	0.0	0.0	0.0	0.0
0.0	0.0	0.0	0.0	0.0	0.0	0.0	0.0
0.0	0.0	0.0	0.0	0.0	0.0	0.0	0.0
0.0	0.0	0.0	0.0	0.0	0.0	0.0	0.0
0.0	0.0	0.0	0.0	0.0	0.0	0.0	0.0
0.0	0.0	0.0	0.0	0.0	15.0	0.0	0.0
0.0	0.0	0.0	0.0	0.0	0.0	0.0	0.0
0.0	0.0	0.0	0.0	0.0	0.0	0.0	0.0
0.0	0.0	0.0	0.0	0.0	0.0	0.0	0.0
0.0	0.0	0.0	0.0	0.0	0.0	0.0	0.0
0.0	0.0	0.0	0.0	0.0	0.0	0.0	0.0
0.0	0.0	0.0	0.0	0.0	0.0	0.0	0.0
0.0	0.0	0.0	0.0	0.0	0.0	0.0	0.0
0.0	0.0	0.0	0.0	0.0	0.0	0.0	0.0
0.0	0.0	0.0	0.0	0.0	0.0	0.0	0.0
0.0	0.0	0.0	0.0	0.0	0.0	0.0	0.0
0.0	0.0	0.0	0.0	0.0	0.0	0.0	0.0
0.0	0.0	0.0	0.0	0.0	0.0	48.0	0.0
6.0	0.0	0.0	1.0	0.0	0.0	38.0	0.0
5.0	0.0	0.0	0.0	0.0	0.0	0.0	0.0
0.0	4.0	0.0	0.0	0.0	0.0	104.0	0.0
0.0	0.0	0.0	0.0	0.0	0.0	0.0	0.0
0.0	0.0	0.0	0.0	0.0	0.0	0.0	0.0
0.0	0.0	0.0	39.0	0.0	0.0	0.0	0.0
0.0	0.0	0.0	2.0	0.0	0.0	0.0	0.0
0.0	0.0	0.0	0.0	0.0	0.0	0.0	0.0

0.0	0.0	0.0	0.0	0.0	0.0	0.0	0.0
0.0	0.0	0.0	0.0	0.0	0.0	0.0	0.0
0.0	0.0	0.0	0.0	0.0	0.0	0.0	0.0
0.0	0.0	0.0	0.0	0.0	0.0	0.0	0.0
0.0	0.0	0.0	0.0	0.0	0.0	0.0	0.0
0.0	0.0	0.0	0.0	0.0	0.0	0.0	0.0
0.0	0.0	0.0	0.0	0.0	0.0	0.0	0.0
0.0	0.0	0.0	0.0	0.0	0.0	0.0	0.0
0.0	0.0	0.0	0.0	0.0	0.0	0.0	0.0
0.0	0.0	0.0	0.0	0.0	0.0	0.0	0.0
0.0	0.0	0.0	0.0	0.0	0.0	0.0	0.0
0.0	0.0	0.0	0.0	0.0	0.0	0.0	0.0
0.0	0.0	0.0	0.0	0.0	0.0	0.0	0.0
0.0	0.0	0.0	0.0	0.0	0.0	0.0	0.0
0.0	0.0	0.0	0.0	0.0	0.0	0.0	0.0
0.0	0.0	0.0	0.0	0.0	0.0	0.0	0.0
0.0	0.0	0.0	3.0	0.0	0.0	0.0	0.0
0.0	0.0	0.0	0.0	0.0	0.0	0.0	0.0
0.0	0.0	0.0	0.0	0.0	0.0	2.0	0.0
0.0	0.0	0.0	0.0	0.0	0.0	0.0	0.0
0.0	0.0	0.0	14.0	0.0	0.0	0.0	0.0
0.0	0.0	0.0	0.0	0.0	0.0	0.0	0.0
0.0	0.0	0.0	0.0	0.0	0.0	0.0	0.0
0.0	0.0	0.0	4.0	0.0	0.0	0.0	0.0
0.0	0.0	0.0	0.0	0.0	0.0	0.0	0.0
0.0	0.0	0.0	0.0	0.0	0.0	0.0	0.0
0.0	0.0	0.0	0.0	0.0	0.0	0.0	0.0
0.0	0.0	0.0	0.0	0.0	0.0	0.0	0.0
0.0	0.0	0.0	1.0	0.0	0.0	0.0	0.0
0.0	0.0	0.0	0.0	0.0	0.0	0.0	0.0
0.0	0.0	0.0	0.0	0.0	0.0	0.0	0.0
0.0	0.0	0.0	0.0	0.0	0.0	0.0	0.0
0.0	0.0	0.0	0.0	0.0	0.0	0.0	0.0
0.0	0.0	0.0	0.0	0.0	0.0	0.0	0.0
0.0	0.0	0.0	0.0	0.0	0.0	0.0	0.0
0.0	0.0	0.0	0.0	0.0	0.0	0.0	0.0
0.0	0.0	0.0	2.0	0.0	0.0	0.0	0.0
0.0	0.0	0.0	0.0	0.0	0.0	0.0	0.0
0.0	0.0	0.0	0.0	0.0	0.0	0.0	0.0
0.0	0.0	0.0	0.0	0.0	0.0	0.0	0.0
0.0	0.0	0.0	0.0	0.0	0.0	0.0	0.0
0.0	0.0	0.0	0.0	0.0	0.0	0.0	0.0
0.0	0.0	0.0	0.0	0.0	0.0	0.0	0.0
0.0	0.0	0.0	21.0	0.0	0.0	0.0	0.0
0.0	0.0	0.0	0.0	0.0	0.0	0.0	0.0
0.0	0.0	0.0	0.0	0.0	0.0	0.0	0.0
0.0	0.0	0.0	0.0	0.0	0.0	0.0	0.0

0.0	0.0	0.0	0.0	0.0	0.0	0.0	0.0
0.0	0.0	0.0	0.0	0.0	0.0	0.0	0.0
0.0	0.0	0.0	0.0	0.0	0.0	0.0	0.0
0.0	0.0	0.0	2.0	0.0	0.0	0.0	0.0
0.0	0.0	0.0	0.0	0.0	0.0	0.0	0.0
0.0	0.0	0.0	0.0	0.0	0.0	0.0	0.0
0.0	0.0	0.0	0.0	0.0	0.0	0.0	0.0
0.0	0.0	0.0	0.0	0.0	0.0	0.0	0.0
0.0	0.0	0.0	0.0	0.0	0.0	0.0	0.0
0.0	0.0	0.0	0.0	0.0	0.0	0.0	0.0
0.0	0.0	0.0	0.0	0.0	0.0	0.0	0.0
0.0	0.0	0.0	0.0	0.0	0.0	0.0	0.0
0.0	0.0	0.0	0.0	0.0	0.0	0.0	0.0
0.0	0.0	0.0	0.0	0.0	0.0	0.0	0.0
0.0	0.0	0.0	0.0	0.0	0.0	0.0	0.0
0.0	0.0	0.0	0.0	0.0	0.0	0.0	0.0
0.0	0.0	0.0	0.0	0.0	0.0	0.0	0.0
0.0	0.0	0.0	0.0	0.0	0.0	0.0	0.0
0.0	0.0	0.0	0.0	0.0	0.0	0.0	0.0
0.0	0.0	0.0	0.0	0.0	0.0	0.0	0.0
0.0	0.0	0.0	0.0	0.0	0.0	0.0	0.0
0.0	0.0	0.0	0.0	0.0	0.0	0.0	0.0
0.0	0.0	0.0	6.0	0.0	0.0	0.0	0.0
0.0	0.0	0.0	0.0	0.0	0.0	0.0	0.0
0.0	0.0	0.0	0.0	0.0	0.0	0.0	0.0
0.0	0.0	0.0	0.0	0.0	0.0	0.0	0.0
0.0	0.0	0.0	0.0	0.0	0.0	0.0	0.0
0.0	0.0	0.0	0.0	0.0	0.0	0.0	0.0
0.0	0.0	0.0	0.0	0.0	0.0	0.0	0.0
0.0	0.0	0.0	0.0	0.0	0.0	0.0	0.0
0.0	0.0	0.0	0.0	0.0	0.0	0.0	0.0
0.0	0.0	0.0	0.0	0.0	0.0	0.0	0.0
0.0	0.0	0.0	0.0	750.0	0.0	0.0	0.0
0.0	0.0	0.0	0.0	0.0	0.0	0.0	0.0
0.0	0.0	0.0	0.0	0.0	0.0	0.0	0.0
0.0	0.0	0.0	0.0	0.0	0.0	0.0	0.0
0.0	0.0	0.0	0.0	0.0	0.0	0.0	0.0
0.0	0.0	0.0	0.0	0.0	0.0	0.0	0.0
0.0	0.0	0.0	0.0	0.0	0.0	0.0	0.0
0.0	0.0	0.0	0.0	0.0	0.0	0.0	0.0
0.0	0.0	0.0	0.0	0.0	0.0	0.0	0.0
0.0	0.0	1.0	0.0	0.0	0.0	0.0	0.0
0.0	0.0	0.0	0.0	1.0	0.0	0.0	0.0
0.0	0.0	0.0	0.0	0.0	0.0	0.0	0.0
0.0	0.0	0.0	0.0	0.0	0.0	0.0	0.0
0.0	0.0	0.0	0.0	0.0	0.0	0.0	0.0
0.0	0.0	0.0	0.0	0.0	0.0	0.0	0.0

0.0	0.0	0.0	0.0	0.0	0.0	0.0	0.0
0.0	0.0	0.0	0.0	0.0	0.0	0.0	0.0
0.0	0.0	0.0	0.0	0.0	0.0	0.0	0.0
0.0	0.0	0.0	0.0	0.0	0.0	0.0	0.0
0.0	0.0	0.0	0.0	0.0	0.0	0.0	0.0
0.0	0.0	0.0	24.0	0.0	0.0	0.0	0.0
2.0	0.0	0.0	27.0	0.0	42.0	0.0	0.0
0.0	0.0	0.0	0.0	0.0	0.0	0.0	0.0
0.0	0.0	0.0	0.0	0.0	0.0	0.0	0.0
0.0	0.0	0.0	0.0	0.0	0.0	0.0	0.0
0.0	0.0	0.0	0.0	0.0	0.0	0.0	0.0
0.0	0.0	0.0	0.0	0.0	0.0	0.0	0.0
0.0	0.0	0.0	0.0	0.0	0.0	0.0	0.0
0.0	0.0	22.0	0.0	0.0	0.0	0.0	0.0
0.0	0.0	0.0	0.0	0.0	0.0	0.0	0.0
0.0	0.0	0.0	0.0	0.0	0.0	0.0	0.0
0.0	0.0	2.0	1.0	1.0	1.0	0.0	0.0
0.0	0.0	0.0	0.0	0.0	0.0	0.0	0.0
0.0	0.0	0.0	0.0	0.0	0.0	0.0	0.0
0.0	0.0	0.0	0.0	0.0	0.0	0.0	0.0
0.0	0.0	0.0	0.0	0.0	0.0	0.0	0.0
0.0	0.0	0.0	0.0	0.0	0.0	0.0	0.0
0.0	0.0	0.0	0.0	0.0	0.0	0.0	0.0
0.0	0.0	0.0	0.0	0.0	0.0	0.0	0.0
0.0	0.0	0.0	0.0	0.0	0.0	0.0	0.0
0.0	0.0	0.0	0.0	0.0	0.0	0.0	0.0
0.0	0.0	0.0	0.0	0.0	0.0	0.0	0.0
0.0	0.0	0.0	0.0	0.0	0.0	0.0	3.0
0.0	0.0	0.0	0.0	0.0	0.0	0.0	0.0
0.0	0.0	0.0	0.0	0.0	0.0	0.0	0.0
0.0	0.0	0.0	0.0	0.0	0.0	0.0	0.0
0.0	0.0	0.0	0.0	0.0	0.0	0.0	0.0
0.0	0.0	0.0	0.0	0.0	0.0	0.0	0.0
0.0	0.0	0.0	0.0	0.0	0.0	0.0	0.0
0.0	0.0	0.0	1.0	0.0	0.0	0.0	0.0
1.0	0.0	0.0	0.0	0.0	0.0	0.0	0.0
0.0	0.0	0.0	0.0	0.0	0.0	0.0	0.0
0.0	0.0	0.0	0.0	0.0	0.0	0.0	0.0
0.0	0.0	0.0	0.0	0.0	0.0	0.0	0.0
0.0	0.0	0.0	0.0	0.0	0.0	0.0	0.0
0.0	0.0	0.0	0.0	0.0	0.0	0.0	0.0
0.0	0.0	0.0	0.0	0.0	0.0	0.0	0.0
0.0	0.0	0.0	0.0	0.0	0.0	0.0	0.0
0.0	0.0	0.0	0.0	0.0	0.0	0.0	0.0
0.0	0.0	0.0	0.0	0.0	0.0	0.0	0.0
3.0	0.0	0.0	0.0	2.0	0.0	0.0	2.0
0.0	0.0	0.0	0.0	0.0	0.0	0.0	0.0

0.0	1.0	0.0	0.0	0.0	0.0	0.0	0.0
0.0	0.0	0.0	0.0	0.0	0.0	0.0	0.0
0.0	0.0	0.0	0.0	0.0	0.0	0.0	0.0
0.0	0.0	0.0	0.0	0.0	0.0	0.0	0.0
0.0	0.0	0.0	0.0	0.0	0.0	0.0	0.0
0.0	0.0	0.0	0.0	0.0	0.0	0.0	0.0
0.0	1.0	0.0	0.0	0.0	0.0	1.0	0.0
0.0	0.0	0.0	0.0	0.0	0.0	0.0	0.0
0.0	0.0	0.0	0.0	0.0	0.0	0.0	0.0
0.0	0.0	0.0	0.0	0.0	0.0	0.0	0.0
0.0	0.0	0.0	0.0	0.0	0.0	0.0	0.0
0.0	0.0	0.0	0.0	0.0	0.0	0.0	0.0
0.0	0.0	0.0	0.0	0.0	0.0	0.0	0.0
0.0	7.0	0.0	0.0	0.0	0.0	0.0	0.0
0.0	0.0	0.0	0.0	0.0	0.0	0.0	0.0
0.0	0.0	7.0	0.0	0.0	0.0	0.0	0.0
0.0	0.0	0.0	0.0	0.0	0.0	0.0	0.0
0.0	0.0	0.0	0.0	0.0	0.0	0.0	0.0
0.0	1.0	0.0	0.0	0.0	0.0	0.0	0.0
0.0	1.0	0.0	0.0	0.0	0.0	0.0	0.0
0.0	0.0	0.0	0.0	0.0	0.0	0.0	0.0
0.0	0.0	4.0	0.0	1.0	0.0	0.0	0.0
0.0	0.0	0.0	0.0	0.0	0.0	0.0	0.0
0.0	50.0	0.0	0.0	0.0	0.0	2102.0	0.0
0.0	7.0	0.0	0.0	0.0	0.0	0.0	0.0
0.0	0.0	0.0	0.0	0.0	0.0	0.0	0.0
0.0	0.0	0.0	0.0	0.0	0.0	0.0	0.0
0.0	0.0	0.0	0.0	0.0	0.0	0.0	0.0
0.0	0.0	0.0	0.0	0.0	0.0	0.0	0.0
0.0	0.0	0.0	0.0	0.0	0.0	0.0	0.0
0.0	0.0	0.0	0.0	0.0	0.0	0.0	0.0
0.0	0.0	0.0	0.0	0.0	0.0	0.0	0.0
0.0	0.0	0.0	0.0	0.0	0.0	0.0	0.0
0.0	0.0	0.0	0.0	0.0	0.0	0.0	0.0
0.0	0.0	0.0	0.0	0.0	0.0	0.0	0.0
0.0	0.0	0.0	0.0	0.0	0.0	0.0	0.0
0.0	0.0	0.0	0.0	0.0	0.0	0.0	0.0
0.0	0.0	0.0	0.0	0.0	0.0	0.0	0.0
0.0	0.0	0.0	0.0	0.0	0.0	0.0	0.0
0.0	0.0	0.0	0.0	0.0	0.0	0.0	0.0
0.0	0.0	0.0	0.0	0.0	0.0	0.0	0.0
0.0	0.0	0.0	0.0	0.0	0.0	0.0	0.0
0.0	0.0	0.0	0.0	0.0	0.0	0.0	0.0
0.0	0.0	0.0	0.0	0.0	0.0	0.0	0.0
0.0	0.0	0.0	0.0	0.0	0.0	0.0	0.0
0.0	0.0	0.0	0.0	0.0	0.0	0.0	0.0
0.0	0.0	0.0	0.0	0.0	0.0	0.0	0.0

0.0	0.0	0.0	0.0	0.0	0.0	0.0	0.0
0.0	4.0	0.0	0.0	0.0	0.0	0.0	0.0
0.0	0.0	0.0	0.0	0.0	0.0	0.0	0.0
0.0	0.0	0.0	4.0	0.0	0.0	0.0	0.0
0.0	0.0	0.0	0.0	0.0	0.0	0.0	0.0
0.0	0.0	0.0	0.0	0.0	0.0	0.0	3.0
0.0	0.0	0.0	0.0	0.0	0.0	0.0	0.0
0.0	0.0	0.0	0.0	0.0	0.0	0.0	0.0
0.0	0.0	0.0	0.0	0.0	0.0	0.0	0.0
0.0	0.0	0.0	0.0	0.0	0.0	0.0	0.0
0.0	0.0	0.0	0.0	0.0	0.0	0.0	0.0
0.0	0.0	0.0	0.0	0.0	0.0	0.0	0.0
0.0	0.0	0.0	0.0	0.0	0.0	0.0	0.0
0.0	3.0	0.0	4.0	2.0	0.0	0.0	0.0
0.0	0.0	0.0	0.0	0.0	0.0	0.0	0.0
2.0	0.0	0.0	0.0	0.0	0.0	0.0	0.0
2.0	0.0	0.0	0.0	0.0	0.0	0.0	0.0
1.0	0.0	0.0	0.0	0.0	0.0	9.0	0.0
0.0	0.0	0.0	0.0	0.0	0.0	0.0	0.0
0.0	0.0	0.0	0.0	0.0	0.0	0.0	0.0
1.0	0.0	0.0	0.0	0.0	0.0	0.0	0.0
0.0	0.0	0.0	0.0	0.0	0.0	0.0	0.0
0.0	0.0	0.0	0.0	0.0	0.0	0.0	0.0
0.0	1.0	5.0	0.0	0.0	0.0	0.0	0.0
0.0	0.0	0.0	0.0	0.0	0.0	0.0	0.0
0.0	0.0	0.0	0.0	0.0	0.0	0.0	0.0
0.0	0.0	0.0	0.0	0.0	0.0	0.0	0.0
0.0	0.0	0.0	0.0	0.0	0.0	0.0	0.0
0.0	0.0	0.0	0.0	0.0	0.0	0.0	0.0
0.0	0.0	0.0	0.0	0.0	0.0	0.0	0.0
0.0	0.0	0.0	0.0	0.0	0.0	0.0	0.0
0.0	0.0	0.0	0.0	0.0	0.0	0.0	0.0
0.0	0.0	0.0	0.0	0.0	0.0	0.0	0.0
0.0	0.0	0.0	0.0	0.0	0.0	0.0	0.0
0.0	0.0	0.0	0.0	0.0	0.0	0.0	0.0
0.0	4.0	0.0	0.0	0.0	0.0	0.0	0.0
0.0	0.0	0.0	0.0	0.0	0.0	0.0	0.0
2.0	0.0	0.0	0.0	1.0	0.0	0.0	10.0
0.0	0.0	0.0	0.0	0.0	0.0	0.0	0.0
0.0	0.0	0.0	0.0	0.0	0.0	0.0	0.0
0.0	0.0	0.0	0.0	0.0	0.0	0.0	0.0
0.0	0.0	0.0	0.0	0.0	0.0	0.0	0.0
0.0	0.0	0.0	0.0	0.0	0.0	0.0	0.0
0.0	0.0	0.0	0.0	0.0	0.0	0.0	0.0
0.0	0.0	0.0	0.0	0.0	0.0	0.0	0.0
0.0	0.0	0.0	0.0	0.0	0.0	0.0	0.0
0.0	0.0	0.0	0.0	0.0	0.0	0.0	0.0

0.0	0.0	0.0	0.0	0.0	0.0	0.0	0.0
0.0	0.0	0.0	0.0	0.0	0.0	0.0	0.0
0.0	0.0	0.0	0.0	0.0	0.0	0.0	0.0
0.0	0.0	0.0	0.0	0.0	0.0	0.0	0.0
0.0	0.0	0.0	0.0	0.0	0.0	0.0	0.0
0.0	0.0	0.0	0.0	0.0	0.0	0.0	0.0
0.0	0.0	0.0	0.0	0.0	0.0	0.0	0.0
0.0	0.0	0.0	0.0	0.0	0.0	0.0	0.0
0.0	0.0	0.0	0.0	0.0	0.0	0.0	0.0
0.0	0.0	0.0	0.0	0.0	0.0	0.0	0.0
0.0	0.0	0.0	0.0	0.0	0.0	0.0	0.0
0.0	0.0	0.0	0.0	0.0	0.0	0.0	0.0
0.0	0.0	0.0	0.0	0.0	0.0	0.0	0.0
0.0	0.0	0.0	0.0	0.0	0.0	0.0	0.0
0.0	0.0	0.0	0.0	0.0	0.0	0.0	0.0
0.0	0.0	0.0	0.0	0.0	0.0	0.0	0.0
0.0	0.0	0.0	0.0	0.0	0.0	0.0	0.0
0.0	0.0	0.0	0.0	0.0	0.0	0.0	0.0
0.0	0.0	0.0	0.0	0.0	0.0	0.0	0.0
0.0	0.0	0.0	0.0	0.0	0.0	0.0	0.0
0.0	0.0	0.0	0.0	0.0	0.0	0.0	0.0
0.0	0.0	0.0	0.0	0.0	0.0	0.0	0.0
0.0	0.0	0.0	0.0	0.0	0.0	0.0	0.0
0.0	0.0	0.0	0.0	0.0	0.0	0.0	0.0
0.0	0.0	0.0	0.0	0.0	0.0	0.0	0.0
0.0	0.0	0.0	0.0	0.0	0.0	0.0	0.0
0.0	0.0	0.0	0.0	0.0	0.0	0.0	0.0
0.0	0.0	0.0	0.0	0.0	0.0	0.0	0.0
0.0	0.0	0.0	0.0	0.0	0.0	0.0	0.0
0.0	0.0	0.0	0.0	0.0	0.0	0.0	0.0
0.0	0.0	0.0	0.0	0.0	0.0	0.0	0.0
0.0	0.0	0.0	0.0	0.0	0.0	0.0	0.0
0.0	0.0	0.0	0.0	0.0	0.0	0.0	0.0
0.0	0.0	0.0	0.0	0.0	0.0	0.0	0.0
0.0	0.0	0.0	0.0	0.0	0.0	0.0	0.0
0.0	0.0	0.0	0.0	0.0	0.0	0.0	0.0
0.0	0.0	0.0	0.0	0.0	0.0	0.0	0.0
0.0	0.0	0.0	0.0	0.0	0.0	0.0	0.0
0.0	0.0	0.0	9.0	0.0	0.0	0.0	0.0
0.0	0.0	0.0	0.0	0.0	0.0	0.0	0.0
0.0	0.0	0.0	0.0	0.0	0.0	0.0	0.0
0.0	0.0	0.0	0.0	0.0	0.0	0.0	0.0
0.0	9.0	0.0	2.0	0.0	0.0	0.0	0.0
1.0	0.0	0.0	0.0	0.0	0.0	0.0	0.0
0.0	0.0	0.0	0.0	0.0	0.0	0.0	0.0
0.0	0.0	0.0	0.0	0.0	0.0	0.0	0.0
0.0	0.0	0.0	0.0	0.0	0.0	0.0	0.0

0.0	0.0	0.0	0.0	0.0	0.0	0.0	0.0
0.0	0.0	0.0	0.0	0.0	0.0	0.0	0.0
0.0	0.0	0.0	0.0	0.0	0.0	0.0	0.0
0.0	0.0	0.0	0.0	0.0	0.0	0.0	0.0
0.0	0.0	0.0	0.0	0.0	0.0	0.0	0.0
0.0	0.0	0.0	8.0	0.0	0.0	0.0	0.0
0.0	0.0	0.0	0.0	0.0	0.0	0.0	0.0
0.0	0.0	0.0	0.0	0.0	0.0	0.0	0.0
0.0	0.0	0.0	0.0	0.0	0.0	0.0	0.0
0.0	0.0	0.0	0.0	0.0	0.0	0.0	0.0
0.0	0.0	0.0	0.0	0.0	0.0	0.0	0.0
0.0	0.0	0.0	0.0	0.0	0.0	0.0	0.0
0.0	0.0	0.0	0.0	0.0	0.0	0.0	0.0
0.0	0.0	0.0	0.0	0.0	0.0	0.0	0.0
0.0	0.0	0.0	0.0	0.0	0.0	0.0	0.0
0.0	0.0	0.0	0.0	0.0	0.0	0.0	0.0
0.0	0.0	0.0	0.0	0.0	0.0	0.0	0.0
0.0	0.0	0.0	0.0	0.0	0.0	0.0	0.0
0.0	0.0	0.0	0.0	0.0	0.0	0.0	0.0
0.0	0.0	0.0	0.0	0.0	0.0	0.0	0.0
0.0	0.0	0.0	0.0	0.0	0.0	0.0	0.0
0.0	0.0	0.0	0.0	0.0	0.0	0.0	0.0
0.0	0.0	0.0	0.0	0.0	0.0	0.0	0.0
0.0	0.0	0.0	0.0	0.0	0.0	0.0	0.0
0.0	0.0	0.0	0.0	0.0	0.0	0.0	0.0
0.0	0.0	0.0	0.0	0.0	0.0	0.0	0.0
0.0	0.0	0.0	2.0	0.0	0.0	0.0	0.0
0.0	0.0	0.0	0.0	0.0	0.0	0.0	0.0
0.0	0.0	0.0	0.0	0.0	0.0	0.0	0.0
0.0	0.0	0.0	0.0	0.0	0.0	0.0	0.0
0.0	0.0	0.0	0.0	0.0	0.0	0.0	0.0
0.0	0.0	0.0	0.0	0.0	0.0	0.0	0.0
0.0	0.0	0.0	0.0	0.0	0.0	0.0	0.0
0.0	0.0	0.0	0.0	0.0	0.0	0.0	0.0
0.0	0.0	0.0	7.0	0.0	0.0	0.0	0.0
0.0	0.0	0.0	0.0	0.0	0.0	0.0	0.0
0.0	0.0	0.0	0.0	0.0	0.0	0.0	0.0
0.0	0.0	0.0	0.0	0.0	0.0	0.0	0.0
0.0	0.0	0.0	0.0	0.0	0.0	0.0	0.0
0.0	0.0	0.0	0.0	0.0	0.0	0.0	0.0
0.0	0.0	0.0	0.0	0.0	0.0	0.0	0.0
0.0	0.0	0.0	0.0	0.0	0.0	0.0	0.0
0.0	0.0	0.0	0.0	0.0	0.0	0.0	0.0
0.0	0.0	0.0	0.0	0.0	0.0	0.0	0.0
0.0	0.0	0.0	0.0	0.0	0.0	0.0	0.0
0.0	0.0	0.0	0.0	0.0	0.0	0.0	0.0
0.0	0.0	0.0	0.0	0.0	0.0	0.0	0.0

0.0	0.0	0.0	0.0	0.0	0.0	0.0	0.0
0.0	0.0	0.0	0.0	0.0	0.0	0.0	0.0
0.0	0.0	0.0	0.0	0.0	0.0	0.0	0.0
0.0	0.0	0.0	0.0	0.0	0.0	0.0	0.0
0.0	0.0	0.0	0.0	0.0	0.0	0.0	0.0
0.0	0.0	0.0	0.0	0.0	0.0	0.0	0.0
0.0	0.0	0.0	0.0	0.0	0.0	0.0	0.0
0.0	0.0	0.0	0.0	0.0	0.0	0.0	0.0
0.0	0.0	0.0	0.0	0.0	0.0	0.0	0.0
0.0	0.0	0.0	0.0	0.0	0.0	0.0	0.0
0.0	0.0	0.0	0.0	0.0	0.0	0.0	0.0
0.0	0.0	0.0	0.0	0.0	0.0	0.0	0.0
0.0	0.0	0.0	0.0	0.0	0.0	0.0	0.0
0.0	0.0	0.0	0.0	0.0	0.0	0.0	0.0
0.0	0.0	0.0	0.0	0.0	0.0	0.0	0.0
0.0	0.0	0.0	0.0	0.0	0.0	0.0	0.0
0.0	0.0	0.0	0.0	0.0	0.0	0.0	0.0
0.0	0.0	0.0	0.0	0.0	0.0	0.0	0.0
0.0	0.0	0.0	0.0	0.0	0.0	0.0	0.0
0.0	0.0	0.0	0.0	0.0	0.0	0.0	0.0
0.0	0.0	0.0	0.0	0.0	0.0	0.0	0.0
0.0	0.0	0.0	0.0	0.0	0.0	0.0	0.0
0.0	0.0	0.0	0.0	0.0	0.0	0.0	0.0
0.0	0.0	0.0	0.0	0.0	0.0	0.0	0.0
0.0	0.0	0.0	0.0	0.0	0.0	0.0	0.0
0.0	0.0	0.0	0.0	0.0	0.0	0.0	0.0
0.0	0.0	0.0	0.0	0.0	0.0	0.0	0.0
0.0	0.0	0.0	0.0	0.0	0.0	0.0	0.0
2.0	0.0	0.0	0.0	0.0	0.0	0.0	0.0
0.0	0.0	0.0	0.0	0.0	0.0	0.0	0.0
0.0	0.0	0.0	0.0	0.0	0.0	0.0	0.0
0.0	0.0	0.0	0.0	0.0	0.0	0.0	0.0
0.0	0.0	0.0	0.0	0.0	0.0	0.0	0.0
0.0	0.0	0.0	0.0	0.0	0.0	0.0	0.0
0.0	0.0	0.0	0.0	0.0	0.0	0.0	0.0
0.0	0.0	0.0	0.0	0.0	0.0	0.0	0.0
0.0	0.0	0.0	0.0	0.0	0.0	0.0	0.0
0.0	0.0	0.0	0.0	0.0	0.0	0.0	0.0
0.0	0.0	0.0	0.0	0.0	0.0	0.0	0.0
0.0	0.0	0.0	0.0	0.0	0.0	0.0	0.0
0.0	0.0	0.0	0.0	0.0	0.0	0.0	0.0
0.0	0.0	0.0	0.0	0.0	0.0	0.0	0.0
0.0	0.0	0.0	0.0	0.0	0.0	0.0	0.0
0.0	0.0	0.0	0.0	0.0	0.0	0.0	0.0
0.0	0.0	0.0	0.0	0.0	0.0	0.0	0.0
0.0	0.0	0.0	0.0	0.0	0.0	0.0	0.0
0.0	0.0	0.0	0.0	0.0	0.0	0.0	0.0

0.0	0.0	0.0	0.0	0.0	0.0	0.0	0.0
0.0	0.0	0.0	0.0	0.0	0.0	0.0	0.0
0.0	0.0	0.0	0.0	0.0	0.0	0.0	0.0
0.0	0.0	0.0	0.0	0.0	0.0	1.0	0.0
0.0	0.0	1.0	0.0	1.0	0.0	0.0	0.0
0.0	0.0	0.0	0.0	0.0	0.0	0.0	0.0
0.0	0.0	0.0	0.0	0.0	0.0	0.0	0.0
0.0	0.0	0.0	3.0	0.0	0.0	0.0	0.0
0.0	0.0	0.0	0.0	0.0	0.0	0.0	0.0
0.0	0.0	0.0	0.0	0.0	0.0	0.0	0.0
0.0	0.0	0.0	0.0	0.0	0.0	0.0	0.0
0.0	0.0	0.0	0.0	0.0	0.0	0.0	0.0
0.0	0.0	0.0	0.0	0.0	0.0	0.0	0.0
0.0	0.0	0.0	0.0	0.0	0.0	0.0	0.0
0.0	0.0	0.0	0.0	0.0	0.0	0.0	0.0
0.0	0.0	0.0	0.0	0.0	0.0	0.0	0.0
0.0	0.0	0.0	0.0	0.0	0.0	0.0	0.0
0.0	0.0	0.0	0.0	0.0	0.0	0.0	0.0
0.0	0.0	0.0	0.0	0.0	0.0	0.0	0.0
0.0	0.0	0.0	0.0	0.0	0.0	0.0	0.0
0.0	0.0	0.0	0.0	0.0	0.0	0.0	0.0
0.0	0.0	0.0	0.0	0.0	0.0	0.0	0.0
0.0	0.0	0.0	0.0	0.0	0.0	0.0	0.0
0.0	0.0	0.0	0.0	0.0	0.0	0.0	0.0
0.0	0.0	0.0	0.0	0.0	0.0	0.0	0.0
0.0	3.0	0.0	0.0	0.0	0.0	0.0	0.0
0.0	0.0	0.0	0.0	0.0	0.0	0.0	0.0
0.0	0.0	0.0	0.0	0.0	0.0	0.0	0.0
0.0	0.0	0.0	0.0	0.0	0.0	0.0	0.0
0.0	0.0	0.0	0.0	0.0	0.0	0.0	0.0
0.0	0.0	0.0	0.0	0.0	0.0	0.0	0.0
0.0	0.0	0.0	0.0	0.0	0.0	0.0	0.0
0.0	0.0	0.0	0.0	0.0	0.0	0.0	0.0
0.0	0.0	0.0	0.0	0.0	0.0	0.0	0.0
0.0	0.0	0.0	0.0	0.0	0.0	0.0	0.0
0.0	0.0	0.0	0.0	0.0	0.0	0.0	0.0
0.0	0.0	0.0	3.0	0.0	0.0	0.0	0.0
0.0	0.0	0.0	0.0	0.0	0.0	0.0	0.0
0.0	0.0	0.0	0.0	0.0	0.0	0.0	0.0
0.0	0.0	0.0	0.0	0.0	0.0	0.0	0.0
0.0	0.0	0.0	0.0	0.0	0.0	0.0	0.0
0.0	0.0	0.0	0.0	0.0	0.0	0.0	0.0
0.0	0.0	0.0	0.0	0.0	0.0	0.0	0.0
0.0	0.0	0.0	0.0	0.0	0.0	0.0	0.0
0.0	0.0	0.0	0.0	0.0	0.0	0.0	0.0
0.0	0.0	0.0	0.0	0.0	0.0	0.0	0.0
0.0	0.0	0.0	0.0	0.0	0.0	0.0	0.0

0.0	0.0	0.0	0.0	0.0	0.0	0.0	0.0
0.0	0.0	0.0	3.0	0.0	0.0	0.0	0.0
0.0	0.0	0.0	0.0	0.0	0.0	0.0	0.0
0.0	0.0	0.0	0.0	0.0	0.0	0.0	0.0
0.0	0.0	0.0	0.0	0.0	0.0	0.0	0.0
0.0	0.0	0.0	0.0	0.0	0.0	0.0	0.0
0.0	0.0	0.0	0.0	0.0	0.0	0.0	0.0
0.0	0.0	0.0	0.0	0.0	0.0	0.0	0.0
0.0	0.0	0.0	0.0	0.0	0.0	0.0	0.0
0.0	0.0	0.0	0.0	0.0	0.0	0.0	0.0
0.0	0.0	0.0	0.0	0.0	0.0	0.0	0.0
0.0	0.0	0.0	0.0	0.0	0.0	0.0	0.0
0.0	0.0	0.0	0.0	0.0	0.0	0.0	0.0
0.0	0.0	0.0	0.0	0.0	0.0	0.0	0.0
0.0	0.0	0.0	0.0	0.0	0.0	0.0	0.0
0.0	0.0	0.0	0.0	0.0	0.0	0.0	0.0
0.0	0.0	0.0	0.0	0.0	0.0	0.0	0.0
0.0	0.0	0.0	0.0	0.0	0.0	0.0	0.0
0.0	0.0	0.0	0.0	0.0	3.0	0.0	0.0
0.0	0.0	0.0	0.0	0.0	0.0	0.0	0.0
0.0	0.0	0.0	0.0	0.0	0.0	0.0	0.0
0.0	0.0	0.0	0.0	0.0	0.0	0.0	0.0
0.0	1.0	3.0	0.0	0.0	0.0	0.0	0.0
0.0	0.0	0.0	0.0	0.0	0.0	0.0	0.0
0.0	0.0	0.0	0.0	0.0	0.0	0.0	0.0
0.0	0.0	0.0	0.0	0.0	0.0	0.0	0.0
0.0	0.0	0.0	0.0	0.0	0.0	0.0	0.0
0.0	0.0	0.0	0.0	0.0	0.0	0.0	0.0
0.0	0.0	0.0	0.0	0.0	0.0	0.0	0.0
0.0	0.0	0.0	0.0	0.0	0.0	0.0	0.0
0.0	0.0	1.0	0.0	0.0	0.0	0.0	0.0
0.0	0.0	0.0	0.0	0.0	0.0	0.0	0.0
0.0	0.0	0.0	0.0	0.0	0.0	0.0	0.0
0.0	0.0	0.0	1.0	0.0	0.0	0.0	3.0
0.0	0.0	0.0	0.0	6.0	0.0	0.0	0.0
0.0	0.0	0.0	0.0	0.0	0.0	0.0	0.0
0.0	0.0	0.0	0.0	0.0	0.0	0.0	0.0
0.0	0.0	0.0	0.0	0.0	0.0	0.0	0.0
0.0	0.0	0.0	0.0	0.0	0.0	0.0	0.0
0.0	0.0	0.0	0.0	0.0	0.0	0.0	0.0
0.0	0.0	0.0	0.0	0.0	0.0	0.0	0.0
0.0	0.0	0.0	0.0	0.0	0.0	0.0	0.0
0.0	0.0	0.0	0.0	0.0	0.0	0.0	0.0
0.0	0.0	0.0	0.0	0.0	0.0	0.0	0.0
0.0	0.0	0.0	0.0	0.0	0.0	0.0	0.0
0.0	0.0	0.0	0.0	0.0	0.0	0.0	0.0
0.0	0.0	0.0	0.0	0.0	0.0	0.0	0.0

0.0	0.0	0.0	0.0	0.0	0.0	0.0	0.0
0.0	0.0	0.0	0.0	0.0	0.0	0.0	0.0
0.0	0.0	0.0	0.0	0.0	0.0	0.0	0.0
0.0	0.0	0.0	0.0	0.0	0.0	0.0	0.0
0.0	0.0	0.0	0.0	0.0	0.0	0.0	0.0
0.0	0.0	0.0	0.0	0.0	0.0	0.0	0.0
0.0	0.0	0.0	0.0	0.0	0.0	0.0	0.0
0.0	0.0	0.0	0.0	0.0	0.0	0.0	0.0
0.0	0.0	0.0	0.0	0.0	0.0	0.0	0.0
0.0	0.0	0.0	0.0	0.0	0.0	0.0	0.0
0.0	0.0	0.0	0.0	0.0	0.0	0.0	0.0
0.0	0.0	0.0	0.0	0.0	0.0	0.0	0.0
0.0	0.0	0.0	0.0	0.0	0.0	2.0	0.0
0.0	0.0	0.0	0.0	0.0	0.0	0.0	0.0
0.0	0.0	0.0	0.0	0.0	0.0	0.0	0.0
0.0	0.0	0.0	0.0	0.0	0.0	0.0	0.0
1.0	0.0	0.0	0.0	0.0	0.0	0.0	0.0
0.0	0.0	0.0	0.0	0.0	0.0	0.0	0.0
0.0	0.0	0.0	0.0	0.0	0.0	0.0	0.0
0.0	0.0	0.0	8.0	0.0	0.0	0.0	0.0
0.0	0.0	0.0	0.0	0.0	0.0	0.0	0.0
0.0	0.0	0.0	0.0	0.0	0.0	0.0	0.0
0.0	0.0	0.0	0.0	0.0	0.0	0.0	0.0
0.0	0.0	0.0	0.0	0.0	0.0	0.0	0.0
0.0	3.0	0.0	0.0	0.0	0.0	0.0	0.0
0.0	0.0	0.0	0.0	0.0	0.0	0.0	0.0
0.0	0.0	0.0	0.0	0.0	0.0	0.0	0.0
0.0	0.0	0.0	0.0	0.0	0.0	0.0	0.0
0.0	1.0	0.0	2.0	0.0	0.0	0.0	0.0
0.0	0.0	0.0	0.0	0.0	0.0	0.0	0.0
0.0	0.0	0.0	0.0	0.0	0.0	3.0	0.0
0.0	0.0	0.0	0.0	0.0	0.0	0.0	0.0
0.0	0.0	0.0	0.0	0.0	0.0	0.0	0.0
0.0	0.0	0.0	0.0	0.0	0.0	0.0	0.0
0.0	0.0	0.0	0.0	0.0	0.0	0.0	0.0
0.0	1.0	0.0	0.0	0.0	0.0	0.0	0.0
0.0	0.0	0.0	0.0	0.0	0.0	0.0	0.0
0.0	0.0	0.0	0.0	0.0	0.0	1.0	0.0
0.0	0.0	0.0	0.0	0.0	0.0	0.0	0.0
0.0	0.0	0.0	0.0	0.0	0.0	0.0	0.0
0.0	0.0	0.0	0.0	0.0	0.0	0.0	0.0
0.0	0.0	0.0	6.0	0.0	0.0	0.0	0.0
0.0	0.0	0.0	0.0	0.0	0.0	0.0	0.0
0.0	0.0	0.0	0.0	0.0	0.0	0.0	0.0
0.0	0.0	0.0	0.0	0.0	0.0	0.0	0.0
0.0	0.0	0.0	0.0	0.0	0.0	0.0	0.0
0.0	0.0	0.0	0.0	0.0	0.0	0.0	0.0

0.0	0.0	0.0	0.0	0.0	0.0	0.0	0.0
0.0	3.0	0.0	0.0	0.0	0.0	0.0	0.0
0.0	0.0	0.0	0.0	0.0	0.0	0.0	0.0
0.0	0.0	0.0	0.0	0.0	0.0	0.0	0.0
0.0	0.0	0.0	0.0	0.0	0.0	0.0	0.0
0.0	0.0	0.0	0.0	0.0	0.0	0.0	0.0
0.0	0.0	0.0	0.0	0.0	0.0	0.0	0.0
0.0	0.0	0.0	0.0	0.0	0.0	0.0	0.0
0.0	0.0	0.0	0.0	0.0	0.0	0.0	0.0
0.0	0.0	0.0	0.0	0.0	0.0	0.0	0.0
0.0	0.0	0.0	0.0	0.0	0.0	0.0	0.0
0.0	0.0	0.0	0.0	0.0	0.0	0.0	0.0
0.0	0.0	0.0	0.0	0.0	0.0	0.0	0.0
0.0	1.0	0.0	1.0	0.0	0.0	0.0	0.0
0.0	0.0	0.0	0.0	0.0	0.0	0.0	0.0
0.0	0.0	0.0	0.0	0.0	0.0	0.0	0.0
0.0	0.0	0.0	0.0	0.0	0.0	0.0	0.0
0.0	0.0	0.0	0.0	0.0	0.0	0.0	0.0
0.0	0.0	0.0	0.0	0.0	0.0	0.0	0.0
0.0	0.0	0.0	0.0	0.0	0.0	0.0	0.0
0.0	0.0	0.0	0.0	0.0	0.0	0.0	0.0
0.0	0.0	0.0	0.0	0.0	0.0	0.0	0.0
0.0	2.0	0.0	1.0	0.0	0.0	0.0	0.0
0.0	0.0	0.0	0.0	0.0	0.0	0.0	0.0
0.0	0.0	0.0	0.0	0.0	0.0	0.0	0.0
0.0	6.0	0.0	0.0	0.0	0.0	0.0	0.0
0.0	0.0	0.0	0.0	0.0	0.0	0.0	0.0
0.0	0.0	0.0	0.0	0.0	0.0	0.0	0.0
0.0	0.0	0.0	0.0	0.0	0.0	0.0	0.0
0.0	0.0	0.0	0.0	0.0	0.0	0.0	0.0
0.0	0.0	0.0	0.0	0.0	0.0	0.0	0.0
0.0	0.0	0.0	0.0	0.0	0.0	0.0	0.0
0.0	0.0	0.0	0.0	0.0	0.0	0.0	0.0
0.0	0.0	0.0	0.0	0.0	0.0	0.0	0.0
0.0	0.0	0.0	0.0	0.0	0.0	0.0	0.0
0.0	0.0	0.0	0.0	0.0	1.0	0.0	0.0
0.0	0.0	0.0	0.0	0.0	0.0	0.0	0.0
0.0	0.0	0.0	0.0	0.0	0.0	0.0	0.0
0.0	0.0	0.0	0.0	0.0	0.0	0.0	0.0
0.0	0.0	0.0	1.0	0.0	0.0	0.0	0.0
0.0	0.0	0.0	0.0	0.0	0.0	0.0	0.0
0.0	0.0	0.0	0.0	0.0	0.0	0.0	0.0
0.0	0.0	0.0	0.0	0.0	0.0	0.0	0.0
0.0	0.0	0.0	0.0	0.0	0.0	0.0	0.0
0.0	0.0	0.0	0.0	0.0	0.0	0.0	0.0
0.0	0.0	0.0	0.0	0.0	0.0	0.0	0.0
0.0	0.0	0.0	0.0	0.0	0.0	0.0	0.0

0.0	0.0	0.0	0.0	0.0	0.0	0.0	0.0
0.0	0.0	0.0	0.0	0.0	0.0	0.0	0.0
0.0	0.0	44.0	0.0	0.0	0.0	0.0	0.0
0.0	0.0	0.0	0.0	0.0	0.0	0.0	0.0
0.0	0.0	0.0	0.0	0.0	0.0	0.0	0.0
0.0	0.0	0.0	0.0	0.0	0.0	0.0	0.0
0.0	0.0	0.0	0.0	0.0	0.0	0.0	0.0
0.0	0.0	0.0	0.0	0.0	0.0	0.0	0.0
0.0	0.0	0.0	0.0	0.0	0.0	0.0	0.0
0.0	0.0	0.0	0.0	0.0	0.0	0.0	0.0
0.0	0.0	0.0	0.0	0.0	0.0	0.0	0.0
0.0	0.0	0.0	0.0	0.0	0.0	0.0	0.0
0.0	0.0	0.0	0.0	0.0	0.0	0.0	0.0
0.0	0.0	0.0	0.0	0.0	0.0	0.0	0.0
0.0	0.0	0.0	0.0	0.0	0.0	0.0	0.0
0.0	0.0	0.0	0.0	0.0	0.0	0.0	0.0
0.0	0.0	0.0	0.0	0.0	0.0	0.0	0.0
0.0	0.0	0.0	0.0	0.0	0.0	0.0	0.0
0.0	0.0	0.0	0.0	0.0	0.0	0.0	1.0
0.0	0.0	0.0	0.0	0.0	0.0	0.0	2.0
0.0	0.0	0.0	6.0	0.0	0.0	0.0	0.0
0.0	0.0	0.0	0.0	0.0	0.0	0.0	0.0
0.0	0.0	0.0	2.0	0.0	0.0	0.0	0.0
0.0	0.0	0.0	0.0	0.0	0.0	0.0	0.0
0.0	0.0	0.0	0.0	0.0	0.0	0.0	0.0
0.0	0.0	0.0	1.0	0.0	0.0	0.0	0.0
0.0	0.0	0.0	0.0	0.0	0.0	0.0	0.0
0.0	0.0	0.0	0.0	0.0	0.0	0.0	0.0
0.0	0.0	0.0	0.0	0.0	0.0	0.0	0.0
0.0	0.0	0.0	0.0	0.0	0.0	0.0	0.0
0.0	0.0	0.0	0.0	0.0	0.0	0.0	0.0
0.0	0.0	0.0	0.0	0.0	0.0	0.0	0.0
0.0	0.0	0.0	0.0	0.0	0.0	0.0	0.0
0.0	0.0	0.0	0.0	0.0	0.0	0.0	0.0
0.0	0.0	0.0	0.0	0.0	0.0	0.0	0.0
0.0	0.0	0.0	0.0	0.0	0.0	0.0	0.0
0.0	0.0	0.0	0.0	0.0	0.0	0.0	0.0
0.0	0.0	0.0	0.0	0.0	0.0	0.0	0.0
0.0	0.0	0.0	0.0	0.0	0.0	0.0	0.0
0.0	0.0	0.0	0.0	0.0	0.0	0.0	0.0
0.0	0.0	0.0	0.0	0.0	0.0	0.0	0.0
0.0	0.0	0.0	0.0	0.0	0.0	0.0	0.0
0.0	0.0	0.0	0.0	0.0	0.0	0.0	0.0
0.0	0.0	0.0	0.0	0.0	0.0	0.0	0.0
0.0	0.0	0.0	0.0	0.0	0.0	0.0	0.0
0.0	0.0	0.0	0.0	0.0	0.0	0.0	0.0
0.0	0.0	0.0	0.0	0.0	0.0	0.0	0.0

0.0	0.0	0.0	0.0	0.0	0.0	1.0	0.0
0.0	0.0	0.0	0.0	0.0	0.0	0.0	0.0
0.0	0.0	0.0	0.0	0.0	0.0	0.0	0.0
0.0	0.0	0.0	0.0	0.0	0.0	0.0	0.0
0.0	0.0	0.0	0.0	0.0	0.0	0.0	0.0
0.0	0.0	0.0	0.0	0.0	0.0	0.0	0.0
0.0	0.0	0.0	0.0	0.0	0.0	0.0	0.0
0.0	0.0	0.0	0.0	0.0	0.0	0.0	0.0
0.0	0.0	0.0	0.0	0.0	0.0	0.0	0.0
0.0	0.0	0.0	0.0	0.0	0.0	0.0	0.0
0.0	0.0	0.0	0.0	0.0	0.0	0.0	0.0
0.0	0.0	0.0	0.0	0.0	0.0	0.0	0.0
0.0	0.0	0.0	0.0	0.0	0.0	0.0	0.0
0.0	0.0	0.0	0.0	0.0	0.0	0.0	0.0
0.0	0.0	0.0	0.0	0.0	0.0	0.0	0.0
0.0	0.0	0.0	0.0	0.0	0.0	0.0	0.0
0.0	1.0	0.0	0.0	0.0	0.0	0.0	0.0
0.0	0.0	0.0	0.0	0.0	0.0	0.0	0.0
0.0	0.0	0.0	0.0	0.0	10.0	0.0	0.0
0.0	0.0	0.0	0.0	0.0	0.0	0.0	0.0
1.0	0.0	0.0	0.0	0.0	0.0	0.0	0.0
0.0	0.0	0.0	0.0	0.0	0.0	0.0	0.0
0.0	38.0	0.0	0.0	0.0	0.0	0.0	0.0
0.0	0.0	0.0	0.0	0.0	0.0	0.0	0.0
0.0	0.0	0.0	0.0	0.0	0.0	0.0	0.0
0.0	0.0	0.0	0.0	0.0	0.0	0.0	0.0
0.0	0.0	0.0	0.0	0.0	0.0	0.0	0.0
0.0	0.0	0.0	0.0	0.0	0.0	0.0	0.0
0.0	0.0	0.0	0.0	0.0	0.0	0.0	0.0
0.0	0.0	0.0	0.0	0.0	0.0	0.0	0.0
0.0	0.0	0.0	0.0	0.0	0.0	0.0	0.0
0.0	0.0	0.0	0.0	0.0	0.0	0.0	0.0
0.0	0.0	0.0	0.0	0.0	0.0	0.0	8.0
0.0	0.0	0.0	0.0	0.0	0.0	0.0	0.0
0.0	0.0	0.0	0.0	0.0	0.0	0.0	0.0
0.0	0.0	0.0	0.0	0.0	0.0	0.0	0.0
0.0	0.0	0.0	0.0	0.0	0.0	0.0	0.0
0.0	0.0	0.0	0.0	0.0	0.0	0.0	0.0
0.0	0.0	0.0	0.0	0.0	0.0	0.0	0.0
0.0	0.0	0.0	0.0	0.0	0.0	0.0	0.0
0.0	0.0	0.0	0.0	0.0	0.0	0.0	0.0
0.0	0.0	0.0	0.0	0.0	0.0	0.0	0.0
0.0	0.0	0.0	0.0	0.0	0.0	0.0	0.0
1.0	0.0	0.0	0.0	0.0	0.0	0.0	0.0
0.0	0.0	0.0	0.0	0.0	0.0	0.0	0.0
0.0	0.0	0.0	0.0	0.0	0.0	0.0	0.0
0.0	0.0	0.0	0.0	0.0	0.0	0.0	0.0

0.0	0.0	0.0	0.0	0.0	0.0	0.0	0.0
0.0	0.0	0.0	0.0	0.0	0.0	0.0	0.0
0.0	0.0	0.0	0.0	0.0	0.0	0.0	0.0
0.0	0.0	0.0	0.0	0.0	0.0	0.0	0.0
0.0	0.0	0.0	0.0	0.0	0.0	0.0	0.0
0.0	0.0	0.0	0.0	0.0	0.0	0.0	0.0
0.0	0.0	0.0	0.0	0.0	0.0	0.0	0.0
0.0	0.0	0.0	0.0	0.0	0.0	0.0	0.0
0.0	0.0	0.0	0.0	0.0	0.0	0.0	0.0
0.0	0.0	0.0	0.0	0.0	0.0	0.0	0.0
0.0	0.0	0.0	0.0	0.0	0.0	0.0	0.0
0.0	0.0	0.0	0.0	0.0	0.0	0.0	0.0
0.0	0.0	0.0	0.0	0.0	0.0	0.0	0.0
0.0	0.0	0.0	0.0	0.0	0.0	0.0	0.0
0.0	0.0	0.0	0.0	0.0	0.0	0.0	0.0
0.0	0.0	0.0	0.0	0.0	0.0	0.0	0.0
0.0	0.0	0.0	0.0	0.0	0.0	0.0	0.0
0.0	0.0	0.0	0.0	0.0	0.0	0.0	0.0
0.0	0.0	0.0	0.0	0.0	0.0	0.0	0.0
0.0	0.0	0.0	0.0	0.0	0.0	0.0	0.0
0.0	0.0	0.0	0.0	0.0	0.0	0.0	0.0
0.0	0.0	0.0	0.0	0.0	0.0	0.0	0.0
0.0	0.0	0.0	0.0	0.0	0.0	0.0	0.0
0.0	0.0	0.0	0.0	0.0	0.0	0.0	0.0
0.0	0.0	0.0	0.0	0.0	0.0	0.0	0.0
0.0	0.0	0.0	0.0	0.0	0.0	0.0	0.0
0.0	0.0	0.0	0.0	0.0	0.0	0.0	0.0
0.0	0.0	0.0	0.0	0.0	0.0	0.0	0.0
0.0	0.0	0.0	0.0	0.0	0.0	0.0	0.0
0.0	0.0	0.0	0.0	0.0	0.0	0.0	0.0
0.0	0.0	0.0	0.0	0.0	0.0	0.0	0.0
0.0	0.0	0.0	0.0	0.0	0.0	0.0	0.0
0.0	0.0	0.0	0.0	0.0	0.0	0.0	0.0
0.0	0.0	0.0	0.0	0.0	0.0	0.0	0.0
0.0	0.0	0.0	0.0	0.0	0.0	0.0	0.0
0.0	0.0	0.0	0.0	0.0	0.0	0.0	0.0
154.0	0.0	0.0	0.0	0.0	1211.0	0.0	0.0
0.0	0.0	0.0	0.0	0.0	0.0	0.0	0.0
0.0	0.0	0.0	0.0	0.0	0.0	0.0	0.0
3.0	0.0	0.0	0.0	0.0	0.0	0.0	0.0
0.0	0.0	0.0	0.0	0.0	0.0	0.0	0.0
3.0	0.0	0.0	0.0	0.0	0.0	0.0	0.0
0.0	0.0	0.0	0.0	0.0	0.0	0.0	0.0
0.0	0.0	0.0	0.0	0.0	0.0	0.0	0.0
13.0	0.0	0.0	0.0	0.0	0.0	0.0	0.0
0.0	0.0	0.0	0.0	0.0	0.0	0.0	0.0
2.0	0.0	0.0	0.0	0.0	0.0	0.0	0.0

0.0	0.0	0.0	0.0	0.0	0.0	0.0	0.0
0.0	0.0	0.0	0.0	0.0	0.0	0.0	0.0
0.0	0.0	0.0	0.0	0.0	0.0	0.0	0.0
11.0	0.0	0.0	0.0	0.0	83.0	0.0	0.0
0.0	0.0	0.0	0.0	0.0	0.0	0.0	0.0
3.0	0.0	0.0	0.0	0.0	0.0	0.0	0.0
0.0	0.0	0.0	0.0	0.0	0.0	0.0	0.0
0.0	0.0	0.0	0.0	0.0	0.0	0.0	0.0
0.0	0.0	0.0	0.0	0.0	0.0	0.0	0.0
18.0	91.0	0.0	0.0	0.0	0.0	0.0	0.0
0.0	0.0	0.0	0.0	0.0	0.0	0.0	0.0
0.0	0.0	0.0	0.0	0.0	0.0	0.0	0.0
0.0	0.0	0.0	0.0	0.0	0.0	0.0	0.0
0.0	0.0	0.0	0.0	0.0	0.0	0.0	0.0
3.0	0.0	0.0	0.0	0.0	0.0	0.0	0.0
0.0	0.0	0.0	0.0	0.0	0.0	0.0	1.0
0.0	0.0	0.0	0.0	0.0	5.0	0.0	0.0
0.0	0.0	0.0	0.0	0.0	0.0	0.0	0.0
0.0	0.0	0.0	0.0	0.0	0.0	0.0	0.0
0.0	0.0	0.0	0.0	0.0	0.0	0.0	0.0
0.0	0.0	0.0	0.0	0.0	0.0	0.0	0.0
0.0	0.0	0.0	0.0	0.0	0.0	0.0	0.0
0.0	0.0	0.0	0.0	0.0	0.0	0.0	0.0
0.0	0.0	0.0	0.0	0.0	0.0	0.0	0.0
7.0	0.0	0.0	0.0	0.0	6.0	0.0	0.0
0.0	0.0	0.0	0.0	0.0	0.0	0.0	0.0
0.0	0.0	0.0	0.0	0.0	5.0	0.0	0.0
0.0	0.0	0.0	0.0	0.0	0.0	0.0	0.0
0.0	0.0	0.0	0.0	0.0	0.0	0.0	0.0
0.0	0.0	0.0	0.0	0.0	0.0	0.0	0.0
0.0	0.0	0.0	0.0	0.0	0.0	0.0	0.0
0.0	0.0	0.0	0.0	0.0	0.0	0.0	0.0
0.0	0.0	0.0	0.0	0.0	0.0	0.0	0.0
0.0	0.0	0.0	0.0	0.0	0.0	0.0	0.0
3.0	0.0	0.0	0.0	0.0	33.0	0.0	0.0
2.0	0.0	0.0	0.0	0.0	0.0	0.0	0.0
0.0	0.0	0.0	0.0	0.0	0.0	0.0	0.0
0.0	0.0	0.0	0.0	0.0	0.0	0.0	0.0
0.0	0.0	0.0	0.0	0.0	0.0	0.0	0.0
0.0	0.0	0.0	0.0	0.0	0.0	0.0	0.0
1.0	0.0	0.0	0.0	0.0	0.0	0.0	0.0
16.0	0.0	0.0	0.0	0.0	0.0	0.0	0.0
0.0	0.0	0.0	0.0	0.0	0.0	0.0	0.0
0.0	0.0	0.0	0.0	0.0	0.0	0.0	0.0
0.0	0.0	0.0	0.0	0.0	0.0	0.0	0.0
0.0	0.0	0.0	0.0	0.0	0.0	0.0	0.0
0.0	0.0	0.0	0.0	0.0	17.0	0.0	0.0

0.0	0.0	0.0	0.0	0.0	0.0	0.0	0.0
15.0	0.0	0.0	0.0	0.0	0.0	0.0	0.0
0.0	0.0	0.0	0.0	0.0	0.0	0.0	0.0
0.0	0.0	0.0	0.0	0.0	0.0	0.0	0.0
0.0	0.0	0.0	0.0	0.0	0.0	0.0	0.0
0.0	0.0	0.0	0.0	0.0	0.0	0.0	0.0
4.0	0.0	0.0	0.0	0.0	0.0	0.0	0.0
0.0	0.0	0.0	0.0	0.0	0.0	0.0	0.0
0.0	0.0	0.0	0.0	0.0	0.0	0.0	0.0
0.0	0.0	0.0	82.0	0.0	0.0	0.0	0.0
0.0	0.0	0.0	0.0	0.0	0.0	0.0	0.0
0.0	0.0	0.0	0.0	0.0	0.0	0.0	0.0
0.0	0.0	0.0	0.0	0.0	0.0	0.0	0.0
0.0	0.0	0.0	0.0	0.0	0.0	0.0	0.0
0.0	0.0	0.0	0.0	0.0	0.0	0.0	0.0
0.0	0.0	0.0	0.0	0.0	0.0	0.0	0.0
0.0	0.0	0.0	0.0	0.0	0.0	0.0	0.0
0.0	0.0	0.0	0.0	0.0	0.0	0.0	0.0
0.0	0.0	0.0	11.0	0.0	0.0	0.0	0.0
0.0	0.0	0.0	0.0	0.0	0.0	0.0	0.0
0.0	0.0	0.0	9.0	0.0	0.0	0.0	0.0
0.0	0.0	0.0	0.0	0.0	0.0	0.0	0.0
0.0	0.0	0.0	0.0	0.0	0.0	0.0	0.0
0.0	0.0	0.0	0.0	0.0	0.0	0.0	0.0
0.0	0.0	0.0	0.0	0.0	0.0	0.0	0.0
0.0	0.0	0.0	0.0	0.0	0.0	0.0	0.0
0.0	0.0	0.0	0.0	0.0	0.0	0.0	0.0
0.0	0.0	0.0	1.0	0.0	0.0	0.0	0.0
0.0	0.0	0.0	0.0	0.0	0.0	0.0	0.0
2.0	0.0	0.0	0.0	0.0	0.0	0.0	0.0
0.0	0.0	0.0	2.0	0.0	0.0	0.0	0.0
0.0	0.0	0.0	0.0	0.0	0.0	0.0	0.0
0.0	0.0	0.0	0.0	0.0	0.0	0.0	0.0
0.0	0.0	0.0	0.0	0.0	0.0	0.0	0.0
0.0	0.0	0.0	0.0	0.0	0.0	0.0	0.0
0.0	0.0	0.0	0.0	0.0	0.0	0.0	0.0
0.0	0.0	0.0	0.0	0.0	0.0	0.0	0.0
0.0	0.0	0.0	0.0	0.0	0.0	0.0	0.0
0.0	0.0	0.0	0.0	0.0	0.0	0.0	0.0
0.0	0.0	0.0	0.0	0.0	0.0	0.0	0.0
0.0	0.0	0.0	0.0	0.0	0.0	0.0	0.0
0.0	0.0	0.0	0.0	0.0	0.0	0.0	0.0
0.0	0.0	0.0	1.0	0.0	0.0	0.0	0.0
0.0	0.0	0.0	0.0	0.0	0.0	0.0	0.0
0.0	0.0	0.0	1.0	0.0	0.0	0.0	0.0
0.0	0.0	0.0	0.0	0.0	0.0	0.0	0.0
0.0	0.0	0.0	0.0	0.0	0.0	0.0	0.0

0.0	0.0	0.0	0.0	0.0	0.0	0.0	0.0
0.0	0.0	0.0	0.0	0.0	0.0	0.0	0.0
0.0	0.0	0.0	0.0	0.0	0.0	0.0	0.0
0.0	0.0	0.0	0.0	0.0	0.0	0.0	0.0
0.0	0.0	0.0	0.0	0.0	0.0	0.0	0.0
0.0	0.0	0.0	0.0	0.0	0.0	0.0	0.0
0.0	0.0	0.0	1.0	0.0	0.0	0.0	0.0
0.0	0.0	0.0	0.0	0.0	0.0	0.0	0.0
0.0	0.0	0.0	0.0	0.0	0.0	0.0	0.0
0.0	0.0	0.0	0.0	0.0	0.0	0.0	0.0
0.0	0.0	0.0	0.0	0.0	0.0	0.0	0.0
0.0	0.0	0.0	0.0	0.0	0.0	0.0	0.0
0.0	0.0	0.0	0.0	0.0	0.0	0.0	0.0
0.0	0.0	0.0	0.0	0.0	0.0	0.0	0.0
0.0	0.0	0.0	0.0	0.0	0.0	0.0	0.0
0.0	0.0	0.0	0.0	0.0	0.0	0.0	0.0
0.0	0.0	0.0	0.0	0.0	0.0	0.0	0.0
0.0	0.0	0.0	0.0	0.0	0.0	0.0	0.0
0.0	0.0	0.0	0.0	0.0	0.0	0.0	0.0
0.0	0.0	0.0	0.0	0.0	0.0	0.0	0.0
0.0	0.0	0.0	0.0	0.0	0.0	0.0	0.0
0.0	0.0	0.0	0.0	0.0	0.0	0.0	0.0
0.0	0.0	0.0	0.0	0.0	0.0	0.0	0.0
0.0	0.0	0.0	0.0	0.0	0.0	0.0	0.0
0.0	0.0	0.0	0.0	0.0	0.0	0.0	0.0
0.0	0.0	0.0	0.0	0.0	0.0	0.0	0.0
0.0	0.0	0.0	0.0	0.0	0.0	0.0	0.0
0.0	0.0	0.0	0.0	0.0	0.0	0.0	0.0
0.0	0.0	0.0	0.0	0.0	0.0	0.0	0.0
0.0	0.0	0.0	0.0	0.0	0.0	0.0	0.0
0.0	0.0	0.0	0.0	0.0	0.0	0.0	0.0
0.0	0.0	0.0	0.0	0.0	0.0	0.0	0.0
0.0	0.0	0.0	0.0	0.0	0.0	0.0	0.0
0.0	0.0	0.0	0.0	0.0	0.0	0.0	0.0
0.0	0.0	0.0	0.0	0.0	0.0	0.0	0.0
0.0	0.0	0.0	0.0	0.0	0.0	0.0	0.0
0.0	0.0	0.0	0.0	0.0	0.0	0.0	0.0
0.0	0.0	0.0	0.0	0.0	0.0	0.0	0.0
0.0	0.0	0.0	0.0	0.0	0.0	0.0	0.0
0.0	0.0	0.0	0.0	0.0	0.0	0.0	0.0
0.0	0.0	0.0	0.0	0.0	0.0	0.0	0.0
0.0	0.0	0.0	0.0	0.0	0.0	0.0	0.0
0.0	0.0	0.0	0.0	0.0	0.0	0.0	0.0
0.0	0.0	0.0	0.0	0.0	1.0	0.0	0.0
0.0	0.0	0.0	0.0	0.0	0.0	0.0	0.0
0.0	0.0	0.0	0.0	0.0	0.0	0.0	0.0
0.0	0.0	0.0	0.0	0.0	0.0	0.0	0.0

0.0	0.0	0.0	0.0	0.0	0.0	0.0	0.0
0.0	0.0	0.0	0.0	0.0	0.0	0.0	0.0
0.0	0.0	0.0	7.0	0.0	0.0	0.0	0.0
0.0	0.0	0.0	0.0	0.0	0.0	0.0	0.0
0.0	0.0	0.0	0.0	0.0	0.0	0.0	0.0
0.0	0.0	0.0	0.0	0.0	0.0	0.0	0.0
0.0	0.0	0.0	0.0	0.0	0.0	0.0	0.0
0.0	0.0	0.0	0.0	0.0	2.0	0.0	0.0
0.0	0.0	0.0	0.0	0.0	0.0	0.0	0.0
0.0	0.0	0.0	0.0	0.0	0.0	0.0	0.0
0.0	0.0	0.0	0.0	0.0	0.0	0.0	0.0
0.0	0.0	0.0	0.0	0.0	0.0	0.0	0.0
0.0	0.0	0.0	0.0	0.0	0.0	0.0	0.0
0.0	0.0	0.0	0.0	0.0	0.0	0.0	0.0
0.0	0.0	0.0	2.0	0.0	0.0	0.0	0.0
0.0	0.0	0.0	0.0	0.0	0.0	0.0	0.0
0.0	0.0	0.0	0.0	0.0	0.0	0.0	0.0
0.0	0.0	0.0	0.0	0.0	0.0	1.0	0.0
0.0	0.0	0.0	0.0	0.0	0.0	0.0	0.0
0.0	0.0	0.0	0.0	0.0	0.0	0.0	0.0
0.0	0.0	0.0	0.0	0.0	0.0	0.0	0.0
0.0	0.0	0.0	0.0	0.0	0.0	0.0	0.0
0.0	0.0	0.0	0.0	0.0	0.0	0.0	0.0
0.0	0.0	0.0	0.0	0.0	0.0	0.0	0.0
0.0	0.0	3.0	0.0	0.0	0.0	0.0	0.0
0.0	0.0	0.0	0.0	0.0	0.0	2.0	0.0
0.0	0.0	0.0	0.0	0.0	0.0	0.0	0.0
0.0	0.0	0.0	0.0	0.0	0.0	0.0	0.0
0.0	0.0	0.0	0.0	0.0	0.0	0.0	0.0
0.0	0.0	0.0	0.0	0.0	0.0	0.0	0.0
0.0	0.0	0.0	0.0	0.0	0.0	0.0	0.0
0.0	0.0	0.0	0.0	0.0	0.0	0.0	0.0
0.0	0.0	0.0	0.0	0.0	0.0	0.0	0.0
0.0	0.0	0.0	0.0	0.0	0.0	0.0	0.0
0.0	0.0	0.0	0.0	0.0	0.0	0.0	0.0
0.0	0.0	0.0	0.0	0.0	0.0	0.0	0.0
0.0	0.0	0.0	0.0	0.0	0.0	0.0	0.0
0.0	0.0	0.0	0.0	0.0	0.0	0.0	0.0
0.0	0.0	0.0	0.0	0.0	0.0	0.0	0.0
0.0	0.0	0.0	0.0	0.0	0.0	0.0	0.0
0.0	0.0	0.0	0.0	0.0	0.0	0.0	0.0
0.0	0.0	0.0	0.0	0.0	0.0	0.0	0.0
0.0	0.0	0.0	0.0	0.0	0.0	0.0	0.0
0.0	0.0	0.0	0.0	0.0	0.0	0.0	0.0
0.0	0.0	0.0	0.0	0.0	0.0	0.0	0.0
0.0	0.0	0.0	0.0	0.0	0.0	0.0	0.0
0.0	0.0	0.0	0.0	0.0	0.0	0.0	0.0

0.0	0.0	0.0	0.0	0.0	0.0	0.0	0.0
0.0	0.0	0.0	0.0	0.0	0.0	0.0	0.0
0.0	0.0	0.0	0.0	0.0	0.0	0.0	0.0
0.0	0.0	0.0	0.0	0.0	0.0	0.0	0.0
0.0	0.0	0.0	0.0	0.0	0.0	0.0	0.0
0.0	0.0	0.0	0.0	0.0	0.0	0.0	0.0
0.0	0.0	0.0	0.0	0.0	0.0	0.0	0.0
0.0	0.0	0.0	0.0	0.0	0.0	0.0	0.0
0.0	0.0	0.0	0.0	0.0	0.0	0.0	0.0
0.0	0.0	0.0	0.0	0.0	0.0	0.0	0.0
0.0	0.0	0.0	0.0	0.0	0.0	0.0	0.0
0.0	0.0	0.0	1.0	0.0	0.0	0.0	0.0
0.0	0.0	0.0	0.0	0.0	0.0	0.0	0.0
0.0	0.0	0.0	3.0	0.0	0.0	0.0	0.0
0.0	0.0	0.0	0.0	0.0	0.0	0.0	0.0
0.0	0.0	0.0	0.0	0.0	0.0	0.0	0.0
0.0	0.0	0.0	0.0	0.0	0.0	0.0	0.0
0.0	0.0	0.0	0.0	0.0	0.0	0.0	0.0
0.0	0.0	0.0	0.0	0.0	0.0	0.0	0.0
0.0	0.0	0.0	0.0	0.0	0.0	0.0	0.0
0.0	0.0	0.0	0.0	0.0	0.0	0.0	0.0
0.0	0.0	0.0	0.0	0.0	0.0	0.0	0.0
0.0	0.0	0.0	0.0	0.0	0.0	0.0	0.0
0.0	0.0	0.0	0.0	0.0	0.0	0.0	0.0
0.0	0.0	0.0	0.0	0.0	0.0	0.0	0.0
0.0	0.0	0.0	0.0	0.0	0.0	0.0	0.0
0.0	0.0	0.0	0.0	0.0	0.0	0.0	0.0
0.0	0.0	0.0	0.0	0.0	0.0	0.0	0.0
0.0	0.0	0.0	0.0	0.0	0.0	0.0	0.0
0.0	0.0	0.0	0.0	0.0	0.0	0.0	0.0
0.0	0.0	0.0	0.0	0.0	0.0	0.0	0.0
0.0	0.0	0.0	0.0	0.0	0.0	0.0	0.0
0.0	0.0	0.0	0.0	0.0	0.0	0.0	0.0
0.0	0.0	0.0	0.0	0.0	0.0	0.0	0.0
0.0	0.0	0.0	0.0	0.0	0.0	0.0	0.0
0.0	0.0	0.0	0.0	0.0	0.0	0.0	0.0
0.0	0.0	0.0	0.0	0.0	0.0	0.0	0.0
0.0	0.0	0.0	0.0	0.0	0.0	0.0	0.0
0.0	0.0	0.0	0.0	0.0	0.0	0.0	0.0
0.0	0.0	0.0	0.0	0.0	0.0	0.0	0.0
0.0	0.0	0.0	0.0	0.0	0.0	0.0	0.0
0.0	0.0	0.0	0.0	0.0	0.0	0.0	0.0
0.0	0.0	0.0	0.0	0.0	0.0	0.0	0.0
0.0	0.0	0.0	0.0	0.0	0.0	0.0	0.0
0.0	0.0	0.0	0.0	0.0	0.0	0.0	0.0
0.0	0.0	0.0	0.0	0.0	0.0	0.0	0.0
0.0	0.0	0.0	0.0	0.0	0.0	0.0	0.0

0.0	0.0	0.0	0.0	0.0	0.0	0.0	0.0
0.0	0.0	0.0	0.0	0.0	0.0	0.0	0.0
0.0	0.0	0.0	0.0	0.0	0.0	0.0	0.0
0.0	0.0	0.0	0.0	0.0	0.0	0.0	0.0
0.0	0.0	0.0	0.0	0.0	0.0	0.0	0.0
0.0	0.0	0.0	0.0	0.0	0.0	0.0	0.0
0.0	0.0	0.0	0.0	0.0	0.0	0.0	0.0
0.0	0.0	0.0	0.0	0.0	11.0	0.0	0.0
0.0	0.0	0.0	0.0	0.0	0.0	0.0	0.0
0.0	0.0	0.0	0.0	0.0	0.0	0.0	0.0
0.0	0.0	0.0	0.0	0.0	0.0	0.0	0.0
0.0	0.0	0.0	0.0	0.0	6.0	4.0	0.0
0.0	0.0	0.0	0.0	0.0	0.0	0.0	0.0
0.0	0.0	0.0	0.0	0.0	0.0	0.0	0.0
0.0	0.0	0.0	0.0	0.0	0.0	0.0	0.0
0.0	0.0	0.0	0.0	0.0	0.0	0.0	0.0
0.0	0.0	0.0	0.0	0.0	0.0	0.0	0.0
0.0	0.0	0.0	0.0	0.0	0.0	0.0	0.0
0.0	0.0	0.0	0.0	0.0	0.0	0.0	0.0
0.0	0.0	0.0	0.0	0.0	0.0	0.0	0.0
0.0	0.0	0.0	0.0	0.0	0.0	0.0	0.0
0.0	0.0	0.0	0.0	0.0	0.0	0.0	0.0
0.0	0.0	0.0	0.0	0.0	0.0	0.0	0.0
0.0	0.0	0.0	0.0	0.0	0.0	0.0	0.0
0.0	0.0	0.0	0.0	0.0	0.0	0.0	0.0
0.0	0.0	0.0	0.0	0.0	0.0	0.0	0.0
0.0	0.0	0.0	0.0	0.0	0.0	0.0	0.0
0.0	0.0	0.0	0.0	0.0	0.0	0.0	0.0
0.0	0.0	0.0	0.0	0.0	0.0	0.0	0.0
0.0	0.0	0.0	0.0	0.0	0.0	0.0	0.0
0.0	0.0	0.0	0.0	0.0	0.0	0.0	0.0
0.0	0.0	0.0	0.0	0.0	0.0	0.0	0.0
0.0	0.0	0.0	0.0	0.0	0.0	0.0	0.0
0.0	0.0	0.0	0.0	0.0	0.0	0.0	0.0
0.0	0.0	0.0	0.0	0.0	0.0	0.0	0.0
0.0	0.0	0.0	0.0	0.0	0.0	0.0	0.0
0.0	0.0	0.0	0.0	0.0	0.0	0.0	0.0
0.0	0.0	0.0	0.0	0.0	0.0	0.0	0.0
0.0	0.0	0.0	0.0	0.0	0.0	0.0	0.0
0.0	0.0	0.0	0.0	0.0	0.0	0.0	0.0
0.0	0.0	0.0	0.0	0.0	0.0	0.0	0.0
0.0	0.0	0.0	0.0	0.0	0.0	0.0	0.0
0.0	0.0	0.0	0.0	0.0	0.0	0.0	0.0
0.0	0.0	0.0	0.0	0.0	0.0	0.0	0.0
0.0	0.0	0.0	0.0	0.0	0.0	0.0	0.0
0.0	0.0	0.0	0.0	0.0	0.0	0.0	0.0
0.0	0.0	0.0	0.0	0.0	0.0	0.0	0.0
0.0	0.0	0.0	0.0	0.0	0.0	0.0	0.0

0.0	0.0	0.0	0.0	0.0	0.0	0.0	0.0
0.0	0.0	0.0	0.0	0.0	0.0	0.0	0.0
0.0	0.0	0.0	0.0	0.0	0.0	0.0	0.0
0.0	0.0	0.0	0.0	0.0	0.0	0.0	0.0
0.0	0.0	0.0	0.0	0.0	0.0	0.0	0.0
0.0	0.0	0.0	0.0	0.0	0.0	0.0	0.0
0.0	0.0	0.0	0.0	0.0	0.0	0.0	0.0
0.0	0.0	0.0	0.0	0.0	0.0	0.0	0.0
0.0	0.0	0.0	0.0	0.0	0.0	0.0	0.0
0.0	0.0	0.0	0.0	0.0	0.0	0.0	0.0
0.0	0.0	0.0	0.0	0.0	0.0	0.0	0.0
0.0	0.0	0.0	0.0	0.0	0.0	0.0	0.0
0.0	0.0	0.0	0.0	0.0	0.0	0.0	0.0
0.0	0.0	0.0	0.0	0.0	0.0	0.0	0.0
0.0	0.0	0.0	0.0	0.0	0.0	0.0	0.0
0.0	0.0	0.0	0.0	0.0	0.0	0.0	0.0
0.0	0.0	0.0	0.0	0.0	0.0	0.0	0.0
0.0	0.0	0.0	0.0	0.0	0.0	0.0	0.0
0.0	0.0	0.0	0.0	0.0	0.0	0.0	0.0
0.0	0.0	0.0	0.0	0.0	0.0	0.0	0.0
0.0	0.0	0.0	0.0	0.0	0.0	0.0	0.0
0.0	0.0	0.0	0.0	0.0	0.0	0.0	0.0
0.0	0.0	0.0	0.0	0.0	0.0	0.0	0.0
0.0	0.0	0.0	0.0	0.0	0.0	0.0	0.0
0.0	0.0	0.0	0.0	0.0	0.0	0.0	0.0
0.0	0.0	0.0	0.0	0.0	0.0	0.0	0.0
0.0	0.0	0.0	0.0	0.0	0.0	0.0	0.0
0.0	0.0	0.0	0.0	0.0	0.0	0.0	0.0
0.0	0.0	0.0	0.0	0.0	0.0	0.0	0.0
0.0	0.0	0.0	0.0	0.0	0.0	0.0	0.0
0.0	0.0	0.0	0.0	0.0	0.0	0.0	0.0
0.0	0.0	0.0	0.0	0.0	0.0	0.0	0.0
0.0	0.0	0.0	0.0	0.0	0.0	0.0	0.0
0.0	0.0	0.0	0.0	0.0	0.0	0.0	0.0
0.0	0.0	0.0	0.0	0.0	1.0	0.0	0.0
0.0	0.0	0.0	0.0	0.0	0.0	0.0	0.0
0.0	0.0	0.0	0.0	0.0	0.0	0.0	0.0
0.0	0.0	0.0	0.0	0.0	0.0	0.0	0.0
0.0	0.0	0.0	0.0	0.0	0.0	0.0	0.0
0.0	0.0	0.0	0.0	0.0	0.0	0.0	0.0
0.0	0.0	0.0	0.0	0.0	0.0	0.0	0.0
0.0	0.0	0.0	0.0	0.0	0.0	0.0	0.0
0.0	0.0	0.0	0.0	0.0	0.0	0.0	0.0
0.0	0.0	0.0	0.0	0.0	0.0	0.0	0.0
0.0	0.0	0.0	0.0	0.0	0.0	0.0	0.0
0.0	2.0	0.0	0.0	0.0	0.0	0.0	0.0
0.0	0.0	0.0	0.0	0.0	0.0	0.0	0.0

0.0	0.0	0.0	0.0	0.0	0.0	0.0	0.0
0.0	0.0	0.0	0.0	0.0	0.0	0.0	0.0
0.0	0.0	0.0	0.0	0.0	0.0	67.0	0.0
0.0	0.0	0.0	0.0	0.0	0.0	0.0	0.0
0.0	0.0	0.0	0.0	0.0	0.0	0.0	0.0
0.0	0.0	0.0	0.0	0.0	0.0	0.0	0.0
0.0	0.0	0.0	0.0	0.0	4.0	0.0	0.0
0.0	0.0	0.0	0.0	0.0	0.0	0.0	0.0
0.0	0.0	0.0	0.0	0.0	0.0	0.0	0.0
0.0	0.0	0.0	4.0	0.0	0.0	0.0	0.0
0.0	0.0	0.0	0.0	0.0	0.0	0.0	0.0
0.0	0.0	0.0	6.0	0.0	0.0	0.0	0.0
0.0	0.0	0.0	0.0	0.0	0.0	0.0	0.0
0.0	0.0	0.0	0.0	0.0	0.0	0.0	0.0
0.0	0.0	0.0	0.0	0.0	0.0	0.0	0.0
0.0	0.0	0.0	0.0	0.0	0.0	0.0	0.0
0.0	0.0	0.0	0.0	0.0	0.0	0.0	0.0
0.0	0.0	0.0	0.0	0.0	0.0	0.0	0.0
0.0	0.0	0.0	0.0	0.0	0.0	0.0	0.0
0.0	0.0	0.0	0.0	0.0	0.0	0.0	0.0
0.0	0.0	0.0	0.0	0.0	0.0	0.0	0.0
0.0	0.0	0.0	0.0	0.0	0.0	0.0	0.0
0.0	0.0	0.0	0.0	0.0	0.0	0.0	0.0
0.0	0.0	0.0	0.0	0.0	0.0	0.0	0.0
0.0	0.0	0.0	0.0	0.0	0.0	0.0	0.0
0.0	0.0	0.0	0.0	0.0	0.0	0.0	0.0
0.0	0.0	0.0	0.0	0.0	0.0	0.0	0.0
0.0	0.0	0.0	3.0	0.0	0.0	0.0	0.0
0.0	0.0	0.0	0.0	0.0	1.0	0.0	43.0
0.0	0.0	0.0	0.0	0.0	0.0	0.0	0.0
0.0	0.0	0.0	0.0	0.0	0.0	0.0	0.0
0.0	0.0	0.0	0.0	0.0	0.0	0.0	0.0
0.0	0.0	0.0	0.0	0.0	0.0	0.0	0.0
0.0	0.0	0.0	0.0	0.0	0.0	0.0	0.0
0.0	0.0	0.0	0.0	0.0	0.0	0.0	0.0
0.0	0.0	0.0	0.0	0.0	0.0	0.0	0.0
0.0	0.0	0.0	0.0	0.0	0.0	0.0	0.0
0.0	0.0	0.0	0.0	0.0	0.0	0.0	0.0
0.0	0.0	0.0	0.0	0.0	0.0	0.0	0.0
0.0	0.0	0.0	0.0	0.0	0.0	0.0	0.0
0.0	0.0	0.0	0.0	0.0	0.0	0.0	0.0
0.0	0.0	0.0	0.0	0.0	0.0	0.0	0.0
0.0	0.0	0.0	0.0	0.0	0.0	0.0	0.0
0.0	0.0	0.0	0.0	0.0	0.0	0.0	0.0
0.0	0.0	0.0	0.0	0.0	0.0	0.0	0.0
0.0	0.0	0.0	0.0	0.0	0.0	0.0	0.0

0.0	0.0	0.0	0.0	0.0	0.0	0.0	0.0
0.0	0.0	0.0	0.0	0.0	0.0	0.0	0.0
0.0	0.0	0.0	0.0	0.0	0.0	0.0	0.0
0.0	0.0	0.0	0.0	0.0	0.0	0.0	0.0
0.0	0.0	0.0	0.0	0.0	0.0	0.0	0.0
0.0	0.0	0.0	0.0	0.0	0.0	0.0	0.0
0.0	0.0	0.0	0.0	0.0	0.0	0.0	0.0
0.0	0.0	0.0	0.0	0.0	0.0	0.0	0.0
0.0	0.0	0.0	0.0	0.0	0.0	0.0	0.0
0.0	0.0	0.0	0.0	0.0	0.0	0.0	0.0
0.0	0.0	0.0	0.0	0.0	0.0	0.0	0.0
0.0	0.0	0.0	0.0	0.0	0.0	0.0	0.0
0.0	0.0	0.0	0.0	0.0	0.0	0.0	0.0
0.0	0.0	0.0	0.0	0.0	0.0	0.0	0.0
0.0	0.0	0.0	0.0	0.0	0.0	0.0	0.0
0.0	0.0	0.0	0.0	0.0	0.0	0.0	0.0
0.0	0.0	0.0	0.0	0.0	0.0	0.0	0.0
0.0	0.0	0.0	0.0	0.0	0.0	0.0	0.0
0.0	0.0	0.0	2.0	0.0	0.0	0.0	0.0
1.0	0.0	0.0	0.0	0.0	0.0	0.0	0.0
0.0	0.0	0.0	0.0	0.0	0.0	0.0	0.0
0.0	0.0	1.0	0.0	0.0	0.0	0.0	0.0
0.0	0.0	0.0	0.0	0.0	0.0	0.0	0.0
0.0	0.0	0.0	1.0	0.0	0.0	0.0	0.0
0.0	0.0	0.0	0.0	0.0	0.0	0.0	0.0
0.0	0.0	0.0	0.0	0.0	0.0	1.0	0.0
0.0	0.0	0.0	0.0	0.0	0.0	0.0	0.0
0.0	0.0	0.0	0.0	0.0	0.0	0.0	0.0
0.0	0.0	0.0	0.0	0.0	0.0	1.0	0.0
0.0	0.0	0.0	0.0	0.0	0.0	0.0	0.0
0.0	0.0	0.0	0.0	0.0	0.0	0.0	0.0
0.0	0.0	0.0	0.0	0.0	0.0	0.0	0.0
0.0	0.0	0.0	0.0	0.0	0.0	0.0	0.0
0.0	0.0	0.0	0.0	0.0	0.0	0.0	0.0
0.0	0.0	0.0	0.0	0.0	0.0	0.0	0.0
0.0	0.0	0.0	0.0	0.0	0.0	0.0	0.0
0.0	0.0	0.0	0.0	0.0	0.0	0.0	0.0
0.0	0.0	0.0	0.0	0.0	0.0	0.0	0.0
0.0	0.0	0.0	0.0	0.0	0.0	0.0	0.0
0.0	0.0	0.0	0.0	0.0	0.0	0.0	0.0
0.0	0.0	0.0	0.0	0.0	0.0	0.0	0.0
0.0	0.0	0.0	0.0	0.0	0.0	0.0	0.0
0.0	0.0	0.0	0.0	0.0	0.0	0.0	0.0
0.0	0.0	0.0	0.0	0.0	0.0	0.0	0.0
0.0	0.0	0.0	0.0	0.0	0.0	0.0	0.0
0.0	0.0	0.0	0.0	0.0	0.0	0.0	0.0
0.0	0.0	0.0	0.0	0.0	0.0	0.0	0.0

0.0	0.0	0.0	0.0	0.0	0.0	0.0	0.0
0.0	0.0	0.0	0.0	0.0	0.0	0.0	0.0
0.0	0.0	0.0	0.0	0.0	0.0	0.0	0.0
0.0	0.0	0.0	0.0	0.0	0.0	0.0	0.0
0.0	0.0	0.0	0.0	0.0	0.0	0.0	0.0
0.0	0.0	0.0	0.0	0.0	0.0	0.0	0.0
0.0	0.0	0.0	0.0	0.0	0.0	0.0	0.0
0.0	0.0	0.0	0.0	0.0	0.0	0.0	0.0
0.0	0.0	0.0	0.0	0.0	0.0	0.0	0.0
0.0	0.0	0.0	0.0	0.0	0.0	0.0	0.0
0.0	0.0	0.0	0.0	0.0	2.0	0.0	0.0
0.0	0.0	0.0	0.0	0.0	0.0	0.0	0.0
0.0	0.0	0.0	0.0	0.0	0.0	0.0	0.0
0.0	0.0	0.0	0.0	0.0	0.0	0.0	0.0
0.0	0.0	0.0	0.0	0.0	0.0	0.0	0.0
0.0	0.0	0.0	0.0	0.0	0.0	0.0	0.0
0.0	0.0	0.0	0.0	0.0	0.0	0.0	0.0
0.0	0.0	0.0	0.0	0.0	0.0	0.0	0.0
0.0	0.0	0.0	0.0	0.0	0.0	0.0	0.0
0.0	0.0	0.0	0.0	0.0	0.0	0.0	0.0
0.0	0.0	0.0	0.0	0.0	0.0	0.0	0.0
0.0	0.0	0.0	0.0	0.0	0.0	0.0	0.0
0.0	0.0	0.0	0.0	0.0	0.0	0.0	0.0
0.0	0.0	0.0	0.0	0.0	0.0	0.0	0.0
0.0	0.0	0.0	0.0	0.0	0.0	0.0	0.0
0.0	0.0	0.0	0.0	0.0	0.0	0.0	0.0
0.0	0.0	0.0	0.0	0.0	0.0	0.0	0.0
0.0	0.0	0.0	0.0	0.0	0.0	0.0	0.0
0.0	0.0	0.0	0.0	0.0	0.0	0.0	0.0
0.0	0.0	0.0	0.0	0.0	0.0	0.0	0.0
0.0	0.0	0.0	0.0	0.0	0.0	0.0	0.0
0.0	0.0	0.0	310.0	0.0	0.0	0.0	0.0
0.0	0.0	0.0	1.0	0.0	0.0	0.0	0.0
0.0	0.0	0.0	0.0	0.0	0.0	0.0	0.0
0.0	0.0	0.0	0.0	0.0	0.0	0.0	0.0
0.0	0.0	0.0	0.0	0.0	0.0	0.0	0.0
0.0	0.0	0.0	0.0	0.0	0.0	0.0	0.0
0.0	0.0	0.0	0.0	0.0	0.0	0.0	0.0
0.0	0.0	0.0	0.0	0.0	0.0	0.0	0.0
0.0	0.0	0.0	0.0	0.0	0.0	0.0	0.0
0.0	0.0	0.0	0.0	0.0	0.0	0.0	0.0
0.0	0.0	0.0	0.0	0.0	0.0	0.0	0.0
0.0	0.0	0.0	0.0	0.0	0.0	0.0	0.0
0.0	0.0	0.0	0.0	0.0	0.0	0.0	0.0
0.0	0.0	0.0	0.0	0.0	0.0	0.0	0.0
0.0	0.0	0.0	0.0	0.0	0.0	0.0	0.0
0.0	0.0	0.0	0.0	0.0	0.0	0.0	0.0

0.0	0.0	0.0	0.0	0.0	0.0	0.0	0.0
0.0	0.0	0.0	0.0	0.0	0.0	0.0	0.0
0.0	0.0	0.0	0.0	0.0	0.0	0.0	0.0
0.0	0.0	0.0	0.0	0.0	0.0	0.0	0.0
0.0	0.0	0.0	0.0	0.0	0.0	0.0	0.0
0.0	0.0	0.0	0.0	0.0	0.0	0.0	0.0
0.0	0.0	0.0	0.0	0.0	0.0	0.0	0.0
0.0	0.0	0.0	0.0	0.0	0.0	0.0	0.0
0.0	0.0	0.0	0.0	0.0	0.0	0.0	0.0
0.0	0.0	0.0	0.0	0.0	0.0	0.0	0.0
0.0	0.0	0.0	0.0	0.0	0.0	0.0	0.0
0.0	0.0	0.0	0.0	0.0	0.0	0.0	0.0
0.0	0.0	0.0	0.0	0.0	0.0	0.0	0.0
0.0	0.0	0.0	0.0	0.0	0.0	0.0	0.0
0.0	0.0	0.0	0.0	0.0	0.0	0.0	0.0
0.0	0.0	0.0	0.0	0.0	0.0	0.0	0.0
0.0	0.0	0.0	0.0	0.0	0.0	0.0	0.0
0.0	0.0	0.0	0.0	0.0	0.0	0.0	0.0
0.0	0.0	0.0	0.0	0.0	0.0	0.0	0.0
0.0	0.0	0.0	0.0	0.0	0.0	0.0	0.0
0.0	0.0	0.0	0.0	0.0	0.0	0.0	0.0
0.0	0.0	0.0	0.0	0.0	0.0	0.0	0.0
0.0	0.0	0.0	0.0	0.0	0.0	0.0	0.0
0.0	0.0	0.0	0.0	0.0	0.0	0.0	0.0
0.0	0.0	0.0	0.0	0.0	0.0	0.0	0.0
0.0	0.0	0.0	0.0	0.0	0.0	0.0	0.0
0.0	0.0	0.0	0.0	0.0	0.0	0.0	0.0
0.0	0.0	0.0	6.0	0.0	0.0	0.0	0.0
0.0	0.0	0.0	0.0	0.0	0.0	0.0	0.0
0.0	0.0	0.0	0.0	0.0	0.0	0.0	0.0
0.0	0.0	0.0	0.0	0.0	0.0	0.0	0.0
0.0	0.0	0.0	0.0	0.0	0.0	0.0	0.0
0.0	0.0	0.0	0.0	0.0	0.0	0.0	0.0
0.0	0.0	0.0	2.0	0.0	0.0	0.0	0.0
0.0	0.0	0.0	0.0	0.0	0.0	0.0	0.0
0.0	0.0	0.0	0.0	0.0	0.0	0.0	0.0
0.0	0.0	0.0	0.0	0.0	0.0	0.0	0.0
0.0	0.0	0.0	0.0	0.0	0.0	0.0	0.0
0.0	0.0	0.0	0.0	0.0	0.0	0.0	0.0
0.0	0.0	0.0	0.0	0.0	0.0	0.0	0.0
0.0	0.0	0.0	0.0	0.0	0.0	0.0	0.0
0.0	0.0	0.0	0.0	0.0	0.0	0.0	0.0
0.0	0.0	0.0	0.0	0.0	0.0	0.0	0.0
0.0	0.0	0.0	0.0	0.0	0.0	0.0	0.0
0.0	0.0	0.0	0.0	0.0	0.0	0.0	0.0
0.0	0.0	0.0	0.0	0.0	0.0	0.0	0.0
0.0	0.0	0.0	0.0	0.0	0.0	0.0	0.0

0.0	0.0	0.0	0.0	0.0	0.0	0.0	0.0
0.0	0.0	0.0	0.0	0.0	0.0	0.0	0.0
0.0	0.0	0.0	0.0	0.0	0.0	0.0	0.0
0.0	0.0	0.0	0.0	0.0	0.0	0.0	0.0
0.0	0.0	0.0	0.0	0.0	0.0	0.0	0.0
0.0	0.0	0.0	0.0	0.0	0.0	0.0	0.0
0.0	0.0	0.0	0.0	0.0	0.0	0.0	0.0
0.0	0.0	0.0	0.0	0.0	0.0	0.0	0.0
0.0	0.0	0.0	0.0	0.0	0.0	0.0	0.0
0.0	0.0	0.0	5.0	0.0	0.0	0.0	0.0
0.0	0.0	0.0	0.0	0.0	0.0	0.0	0.0
0.0	0.0	0.0	0.0	0.0	0.0	0.0	0.0
0.0	0.0	0.0	0.0	0.0	0.0	0.0	0.0
0.0	0.0	0.0	0.0	0.0	0.0	0.0	0.0
0.0	0.0	0.0	0.0	0.0	0.0	0.0	0.0
0.0	0.0	0.0	0.0	0.0	0.0	0.0	0.0
0.0	0.0	0.0	0.0	0.0	0.0	0.0	0.0
0.0	0.0	0.0	0.0	0.0	0.0	0.0	0.0
0.0	0.0	0.0	0.0	0.0	0.0	0.0	0.0
0.0	0.0	0.0	0.0	0.0	0.0	0.0	0.0
0.0	0.0	0.0	0.0	0.0	0.0	0.0	0.0
0.0	0.0	0.0	0.0	0.0	0.0	0.0	0.0
0.0	0.0	0.0	0.0	0.0	0.0	0.0	0.0
0.0	0.0	0.0	0.0	0.0	0.0	0.0	0.0
0.0	0.0	0.0	0.0	0.0	0.0	0.0	0.0
0.0	0.0	0.0	0.0	0.0	0.0	0.0	0.0
0.0	0.0	0.0	0.0	0.0	0.0	0.0	0.0
0.0	0.0	0.0	0.0	0.0	0.0	0.0	0.0
0.0	0.0	0.0	0.0	0.0	0.0	0.0	0.0
0.0	0.0	0.0	0.0	0.0	0.0	0.0	0.0
0.0	0.0	0.0	0.0	0.0	0.0	0.0	0.0
0.0	0.0	0.0	0.0	0.0	0.0	0.0	0.0
0.0	0.0	0.0	0.0	0.0	0.0	0.0	0.0
0.0	0.0	0.0	0.0	0.0	0.0	0.0	0.0
0.0	0.0	0.0	0.0	0.0	0.0	0.0	0.0
0.0	0.0	0.0	0.0	0.0	0.0	0.0	0.0
0.0	0.0	0.0	0.0	0.0	0.0	0.0	0.0
0.0	0.0	0.0	0.0	0.0	0.0	0.0	0.0
0.0	0.0	0.0	0.0	0.0	0.0	0.0	0.0
0.0	0.0	0.0	0.0	0.0	0.0	0.0	0.0
0.0	0.0	0.0	0.0	0.0	0.0	0.0	0.0
0.0	0.0	0.0	0.0	0.0	0.0	0.0	0.0
0.0	0.0	0.0	0.0	0.0	0.0	0.0	0.0
0.0	0.0	0.0	0.0	0.0	0.0	0.0	0.0
0.0	0.0	0.0	0.0	0.0	0.0	0.0	0.0
0.0	0.0	0.0	0.0	0.0	0.0	0.0	0.0
0.0	0.0	0.0	0.0	0.0	0.0	0.0	0.0

0.0	0.0	0.0	0.0	0.0	0.0	0.0	0.0
0.0	0.0	0.0	0.0	0.0	0.0	0.0	0.0
0.0	0.0	0.0	0.0	0.0	0.0	0.0	0.0
0.0	0.0	0.0	0.0	0.0	0.0	0.0	0.0
0.0	0.0	0.0	0.0	0.0	0.0	0.0	0.0
0.0	0.0	1.0	0.0	0.0	0.0	0.0	0.0
0.0	0.0	0.0	0.0	0.0	0.0	0.0	0.0
0.0	0.0	0.0	0.0	0.0	0.0	0.0	0.0
0.0	0.0	0.0	0.0	0.0	0.0	0.0	0.0
0.0	0.0	0.0	0.0	0.0	0.0	0.0	0.0
0.0	0.0	0.0	0.0	0.0	0.0	0.0	0.0
0.0	0.0	0.0	0.0	0.0	0.0	0.0	0.0
0.0	0.0	0.0	0.0	0.0	0.0	0.0	0.0
0.0	0.0	0.0	0.0	0.0	0.0	0.0	0.0
0.0	0.0	0.0	0.0	0.0	0.0	0.0	0.0
0.0	0.0	0.0	0.0	0.0	0.0	0.0	0.0
0.0	0.0	0.0	0.0	0.0	0.0	0.0	0.0
0.0	0.0	0.0	0.0	0.0	0.0	0.0	0.0
0.0	0.0	0.0	0.0	0.0	0.0	0.0	0.0
0.0	0.0	0.0	0.0	0.0	0.0	0.0	0.0
0.0	0.0	0.0	0.0	0.0	0.0	0.0	0.0
0.0	0.0	0.0	0.0	0.0	0.0	0.0	0.0
0.0	0.0	0.0	0.0	0.0	0.0	0.0	0.0
0.0	0.0	0.0	0.0	0.0	0.0	0.0	0.0
0.0	0.0	0.0	0.0	0.0	0.0	0.0	0.0
0.0	0.0	0.0	0.0	0.0	0.0	0.0	0.0
0.0	0.0	0.0	0.0	0.0	0.0	0.0	0.0
0.0	0.0	0.0	0.0	0.0	0.0	0.0	0.0
0.0	0.0	0.0	0.0	0.0	0.0	0.0	0.0
427.0	0.0	0.0	0.0	0.0	0.0	0.0	0.0
0.0	0.0	0.0	0.0	0.0	0.0	0.0	0.0
0.0	0.0	0.0	0.0	0.0	0.0	0.0	0.0
0.0	0.0	0.0	0.0	0.0	0.0	0.0	0.0
0.0	0.0	0.0	0.0	0.0	0.0	0.0	0.0
0.0	0.0	0.0	0.0	0.0	0.0	0.0	0.0
0.0	0.0	0.0	0.0	0.0	0.0	0.0	0.0
0.0	0.0	0.0	2.0	0.0	0.0	0.0	0.0
0.0	0.0	0.0	0.0	0.0	0.0	0.0	0.0
0.0	0.0	0.0	0.0	0.0	0.0	0.0	0.0
0.0	0.0	0.0	0.0	0.0	0.0	0.0	0.0
0.0	0.0	0.0	0.0	0.0	0.0	0.0	0.0
0.0	0.0	48.0	0.0	0.0	0.0	0.0	0.0
0.0	0.0	0.0	0.0	0.0	0.0	0.0	0.0
0.0	0.0	0.0	0.0	0.0	0.0	0.0	0.0
0.0	0.0	0.0	0.0	0.0	0.0	0.0	0.0
0.0	0.0	0.0	0.0	0.0	0.0	0.0	0.0
0.0	0.0	0.0	0.0	0.0	0.0	0.0	0.0

0.0	284.0	18.0	0.0	0.0	8.0	0.0	0.0
0.0	0.0	0.0	0.0	0.0	0.0	0.0	0.0
0.0	0.0	0.0	0.0	0.0	0.0	0.0	0.0
0.0	0.0	0.0	0.0	0.0	0.0	0.0	0.0
0.0	0.0	0.0	0.0	0.0	0.0	0.0	0.0
0.0	0.0	0.0	0.0	0.0	0.0	0.0	0.0
0.0	0.0	0.0	0.0	0.0	0.0	0.0	0.0
0.0	0.0	0.0	0.0	0.0	0.0	0.0	0.0
0.0	0.0	0.0	0.0	0.0	0.0	0.0	0.0
0.0	0.0	0.0	0.0	0.0	0.0	0.0	0.0
0.0	0.0	0.0	0.0	0.0	0.0	0.0	0.0
0.0	0.0	0.0	0.0	0.0	0.0	0.0	0.0
0.0	0.0	0.0	0.0	0.0	0.0	0.0	0.0
0.0	0.0	0.0	0.0	0.0	0.0	0.0	0.0
0.0	0.0	0.0	0.0	0.0	0.0	0.0	0.0
0.0	0.0	0.0	0.0	0.0	0.0	0.0	0.0
0.0	0.0	0.0	0.0	0.0	0.0	0.0	0.0
0.0	0.0	0.0	0.0	0.0	0.0	0.0	0.0
0.0	0.0	0.0	0.0	0.0	0.0	0.0	0.0
0.0	0.0	0.0	0.0	0.0	0.0	0.0	0.0
0.0	0.0	0.0	0.0	0.0	0.0	0.0	0.0
0.0	16.0	0.0	0.0	0.0	0.0	0.0	0.0
0.0	0.0	0.0	0.0	0.0	0.0	0.0	0.0
0.0	0.0	0.0	0.0	0.0	0.0	0.0	0.0
0.0	0.0	0.0	0.0	0.0	0.0	0.0	0.0
0.0	0.0	0.0	0.0	0.0	0.0	0.0	0.0
0.0	0.0	0.0	0.0	0.0	0.0	0.0	0.0
0.0	0.0	0.0	0.0	0.0	0.0	0.0	0.0
0.0	0.0	0.0	0.0	0.0	0.0	0.0	0.0
0.0	0.0	0.0	0.0	0.0	0.0	0.0	0.0
0.0	0.0	0.0	0.0	0.0	0.0	0.0	0.0
0.0	0.0	0.0	4.0	0.0	0.0	0.0	0.0
0.0	0.0	0.0	0.0	0.0	0.0	0.0	0.0
0.0	0.0	0.0	0.0	0.0	0.0	0.0	0.0
0.0	0.0	0.0	0.0	0.0	0.0	0.0	0.0
0.0	0.0	0.0	0.0	0.0	0.0	0.0	0.0
0.0	0.0	0.0	0.0	0.0	0.0	0.0	0.0
0.0	0.0	0.0	0.0	0.0	0.0	0.0	0.0
0.0	0.0	0.0	0.0	0.0	0.0	0.0	0.0
0.0	0.0	0.0	0.0	0.0	0.0	0.0	0.0
0.0	0.0	0.0	0.0	0.0	0.0	0.0	0.0
0.0	0.0	0.0	0.0	0.0	0.0	0.0	0.0
0.0	0.0	0.0	0.0	0.0	0.0	0.0	0.0
0.0	0.0	0.0	0.0	0.0	0.0	0.0	0.0
0.0	0.0	0.0	0.0	0.0	0.0	0.0	0.0
0.0	0.0	0.0	0.0	0.0	0.0	0.0	0.0
0.0	0.0	0.0	0.0	0.0	0.0	0.0	0.0

0.0	0.0	0.0	0.0	0.0	0.0	0.0	0.0
0.0	0.0	0.0	0.0	0.0	0.0	0.0	0.0
0.0	0.0	0.0	0.0	0.0	0.0	0.0	0.0
0.0	0.0	0.0	0.0	0.0	0.0	0.0	0.0
0.0	0.0	0.0	0.0	0.0	0.0	0.0	0.0
0.0	0.0	1.0	1.0	6.0	0.0	0.0	0.0
0.0	0.0	0.0	0.0	0.0	0.0	0.0	0.0
0.0	0.0	0.0	0.0	0.0	0.0	0.0	0.0
0.0	0.0	0.0	0.0	0.0	0.0	0.0	0.0
0.0	0.0	0.0	0.0	0.0	0.0	0.0	0.0
0.0	0.0	0.0	0.0	0.0	0.0	0.0	0.0
0.0	0.0	0.0	0.0	0.0	0.0	0.0	0.0
0.0	0.0	0.0	0.0	0.0	0.0	0.0	0.0
0.0	0.0	0.0	0.0	0.0	0.0	0.0	0.0
0.0	0.0	0.0	0.0	0.0	0.0	0.0	0.0
0.0	0.0	0.0	0.0	0.0	0.0	0.0	0.0
0.0	0.0	0.0	0.0	0.0	0.0	0.0	0.0
0.0	0.0	0.0	0.0	0.0	0.0	0.0	0.0
0.0	0.0	0.0	0.0	0.0	0.0	0.0	0.0
0.0	0.0	0.0	0.0	0.0	0.0	0.0	0.0
0.0	0.0	0.0	0.0	0.0	0.0	0.0	0.0
0.0	0.0	0.0	0.0	0.0	0.0	0.0	1.0
0.0	0.0	2.0	0.0	0.0	0.0	0.0	0.0
0.0	0.0	0.0	0.0	0.0	0.0	0.0	0.0
0.0	0.0	0.0	0.0	0.0	0.0	0.0	0.0
0.0	0.0	0.0	0.0	0.0	0.0	0.0	0.0
0.0	0.0	0.0	0.0	0.0	0.0	0.0	0.0
0.0	0.0	0.0	0.0	0.0	0.0	0.0	0.0
0.0	0.0	0.0	0.0	0.0	0.0	0.0	0.0
0.0	0.0	0.0	0.0	0.0	0.0	0.0	0.0
0.0	0.0	0.0	0.0	0.0	0.0	0.0	0.0
0.0	0.0	0.0	0.0	0.0	0.0	0.0	0.0
0.0	0.0	0.0	0.0	0.0	0.0	0.0	0.0
0.0	0.0	0.0	0.0	0.0	0.0	0.0	0.0
0.0	0.0	0.0	0.0	0.0	0.0	0.0	0.0
0.0	0.0	0.0	0.0	0.0	0.0	0.0	0.0
0.0	0.0	0.0	0.0	0.0	0.0	0.0	0.0
0.0	0.0	0.0	0.0	0.0	0.0	0.0	0.0
0.0	0.0	0.0	0.0	0.0	0.0	0.0	0.0
0.0	0.0	0.0	0.0	0.0	0.0	0.0	0.0
0.0	0.0	0.0	0.0	0.0	0.0	0.0	0.0
0.0	0.0	0.0	0.0	0.0	0.0	0.0	0.0
0.0	0.0	0.0	0.0	0.0	0.0	1.0	0.0
0.0	0.0	0.0	0.0	0.0	0.0	0.0	0.0
0.0	0.0	0.0	0.0	0.0	0.0	0.0	0.0
0.0	0.0	0.0	0.0	0.0	0.0	0.0	0.0
0.0	0.0	0.0	0.0	0.0	0.0	0.0	0.0

0.0	0.0	0.0	0.0	0.0	0.0	0.0	0.0
0.0	0.0	0.0	0.0	0.0	0.0	0.0	0.0
0.0	0.0	0.0	0.0	0.0	0.0	0.0	0.0
0.0	0.0	0.0	0.0	0.0	0.0	0.0	0.0
0.0	0.0	0.0	0.0	0.0	0.0	0.0	0.0
0.0	11.0	0.0	0.0	0.0	0.0	0.0	0.0
0.0	0.0	0.0	0.0	0.0	0.0	0.0	0.0
0.0	0.0	0.0	0.0	0.0	0.0	0.0	0.0
0.0	0.0	0.0	0.0	0.0	0.0	0.0	0.0
0.0	0.0	0.0	0.0	0.0	0.0	0.0	0.0
0.0	0.0	0.0	2.0	0.0	0.0	0.0	0.0
0.0	0.0	0.0	0.0	0.0	0.0	0.0	0.0
0.0	0.0	0.0	0.0	0.0	0.0	0.0	0.0
0.0	0.0	0.0	0.0	0.0	0.0	0.0	0.0
0.0	0.0	0.0	0.0	0.0	0.0	0.0	0.0
0.0	0.0	0.0	0.0	0.0	0.0	2.0	0.0
0.0	0.0	0.0	0.0	0.0	0.0	0.0	0.0
0.0	0.0	0.0	0.0	0.0	0.0	0.0	0.0
0.0	0.0	0.0	0.0	0.0	0.0	0.0	0.0
0.0	0.0	0.0	0.0	0.0	0.0	0.0	0.0
0.0	0.0	0.0	0.0	0.0	0.0	0.0	0.0
0.0	0.0	0.0	0.0	0.0	0.0	0.0	0.0
0.0	0.0	0.0	0.0	0.0	0.0	0.0	0.0
0.0	0.0	0.0	0.0	0.0	0.0	0.0	0.0
0.0	0.0	0.0	0.0	0.0	0.0	0.0	0.0
0.0	0.0	0.0	0.0	0.0	0.0	0.0	0.0
0.0	0.0	0.0	0.0	0.0	0.0	0.0	0.0
0.0	0.0	0.0	0.0	0.0	0.0	0.0	0.0
0.0	0.0	0.0	0.0	0.0	0.0	0.0	0.0
0.0	0.0	0.0	0.0	0.0	0.0	0.0	0.0
0.0	0.0	0.0	0.0	0.0	0.0	0.0	0.0
0.0	0.0	0.0	0.0	0.0	0.0	0.0	0.0
0.0	0.0	0.0	0.0	0.0	0.0	0.0	0.0
0.0	0.0	0.0	0.0	0.0	0.0	0.0	0.0
0.0	0.0	0.0	0.0	0.0	0.0	0.0	0.0
0.0	0.0	0.0	0.0	0.0	0.0	0.0	0.0
0.0	0.0	0.0	0.0	0.0	0.0	0.0	0.0
0.0	0.0	0.0	0.0	0.0	0.0	0.0	0.0
0.0	0.0	0.0	1.0	0.0	0.0	0.0	0.0
0.0	0.0	0.0	0.0	0.0	0.0	0.0	0.0
0.0	0.0	0.0	0.0	0.0	0.0	0.0	0.0
0.0	0.0	0.0	0.0	0.0	0.0	0.0	0.0
0.0	0.0	0.0	0.0	0.0	0.0	0.0	0.0
0.0	0.0	0.0	0.0	0.0	0.0	0.0	0.0
0.0	0.0	0.0	0.0	0.0	0.0	0.0	0.0
0.0	0.0	0.0	0.0	0.0	0.0	0.0	0.0
0.0	0.0	0.0	0.0	0.0	0.0	0.0	0.0

0.0	0.0	0.0	0.0	0.0	0.0	0.0	0.0
0.0	0.0	0.0	0.0	0.0	0.0	0.0	0.0
0.0	0.0	0.0	0.0	0.0	0.0	0.0	0.0
0.0	0.0	0.0	0.0	0.0	0.0	0.0	0.0
0.0	0.0	0.0	0.0	0.0	0.0	0.0	0.0
0.0	0.0	0.0	0.0	0.0	0.0	0.0	0.0
0.0	0.0	0.0	0.0	0.0	0.0	0.0	0.0
0.0	0.0	0.0	0.0	0.0	0.0	0.0	0.0
0.0	0.0	0.0	0.0	0.0	0.0	0.0	0.0
0.0	0.0	0.0	0.0	0.0	0.0	0.0	0.0
0.0	0.0	0.0	0.0	0.0	0.0	0.0	0.0
0.0	0.0	0.0	0.0	0.0	0.0	0.0	0.0
0.0	0.0	0.0	0.0	0.0	0.0	0.0	0.0
0.0	0.0	0.0	0.0	0.0	0.0	0.0	0.0
0.0	0.0	0.0	0.0	0.0	0.0	0.0	0.0
0.0	0.0	0.0	0.0	0.0	0.0	0.0	0.0
0.0	0.0	0.0	0.0	0.0	0.0	0.0	0.0
0.0	0.0	0.0	0.0	0.0	0.0	0.0	0.0
0.0	0.0	0.0	0.0	0.0	0.0	0.0	0.0
0.0	0.0	0.0	0.0	0.0	0.0	0.0	0.0
0.0	0.0	0.0	0.0	0.0	0.0	0.0	0.0
0.0	0.0	0.0	0.0	0.0	0.0	0.0	0.0
0.0	0.0	0.0	0.0	0.0	0.0	0.0	0.0
0.0	0.0	0.0	0.0	0.0	0.0	0.0	0.0
0.0	0.0	0.0	0.0	0.0	0.0	0.0	0.0
0.0	0.0	0.0	0.0	0.0	0.0	0.0	0.0
0.0	0.0	0.0	0.0	0.0	0.0	0.0	0.0
0.0	0.0	0.0	0.0	0.0	0.0	0.0	0.0
0.0	0.0	0.0	0.0	0.0	0.0	0.0	0.0
0.0	0.0	0.0	0.0	0.0	0.0	0.0	0.0
0.0	0.0	0.0	0.0	0.0	0.0	0.0	0.0
0.0	0.0	0.0	0.0	0.0	0.0	0.0	0.0
0.0	0.0	0.0	0.0	0.0	0.0	0.0	0.0
0.0	0.0	0.0	0.0	0.0	0.0	0.0	0.0
0.0	0.0	0.0	0.0	0.0	0.0	0.0	0.0
0.0	0.0	0.0	0.0	0.0	0.0	0.0	0.0
0.0	0.0	0.0	0.0	0.0	0.0	0.0	0.0
0.0	0.0	0.0	0.0	0.0	0.0	0.0	0.0
0.0	0.0	0.0	0.0	0.0	0.0	0.0	0.0
0.0	0.0	0.0	0.0	0.0	0.0	0.0	0.0
0.0	0.0	0.0	0.0	0.0	0.0	0.0	0.0
0.0	0.0	0.0	0.0	0.0	0.0	0.0	0.0
0.0	0.0	0.0	0.0	0.0	0.0	0.0	0.0
0.0	0.0	0.0	0.0	0.0	0.0	0.0	0.0
0.0	0.0	0.0	1.0	0.0	0.0	0.0	0.0
0.0	0.0	0.0	0.0	0.0	0.0	0.0	0.0
0.0	0.0	0.0	0.0	0.0	0.0	0.0	0.0

0.0	0.0	0.0	0.0	0.0	0.0	0.0	0.0
0.0	0.0	0.0	0.0	0.0	0.0	2.0	0.0
0.0	0.0	0.0	0.0	0.0	0.0	0.0	0.0
0.0	0.0	0.0	0.0	0.0	0.0	0.0	0.0
0.0	0.0	0.0	0.0	0.0	0.0	0.0	0.0
0.0	0.0	0.0	0.0	0.0	0.0	0.0	0.0
0.0	0.0	0.0	0.0	0.0	0.0	0.0	0.0
0.0	0.0	0.0	0.0	0.0	0.0	0.0	0.0
0.0	0.0	0.0	0.0	0.0	0.0	0.0	0.0
0.0	0.0	0.0	0.0	0.0	0.0	0.0	0.0
0.0	0.0	0.0	0.0	0.0	0.0	0.0	0.0
0.0	0.0	0.0	0.0	0.0	0.0	0.0	0.0
0.0	0.0	0.0	0.0	0.0	0.0	0.0	0.0
0.0	0.0	0.0	0.0	0.0	0.0	0.0	0.0
0.0	0.0	0.0	0.0	0.0	0.0	0.0	0.0
0.0	0.0	0.0	0.0	0.0	0.0	0.0	0.0
0.0	0.0	0.0	0.0	0.0	0.0	0.0	0.0
0.0	0.0	0.0	0.0	0.0	0.0	0.0	0.0
0.0	0.0	0.0	0.0	0.0	0.0	0.0	0.0
0.0	0.0	0.0	0.0	0.0	7.0	0.0	0.0
0.0	0.0	0.0	0.0	0.0	0.0	0.0	0.0
0.0	0.0	0.0	0.0	0.0	0.0	0.0	0.0
0.0	0.0	0.0	0.0	0.0	0.0	0.0	0.0
0.0	0.0	0.0	0.0	0.0	0.0	0.0	0.0
0.0	0.0	0.0	0.0	0.0	0.0	0.0	0.0
0.0	0.0	0.0	0.0	0.0	0.0	0.0	0.0
0.0	0.0	0.0	0.0	0.0	0.0	0.0	0.0
0.0	0.0	0.0	0.0	0.0	0.0	0.0	0.0
903.0	0.0	0.0	0.0	0.0	0.0	0.0	75.0
163.0	0.0	0.0	0.0	0.0	0.0	0.0	0.0
58.0	0.0	0.0	0.0	0.0	0.0	0.0	0.0
32.0	0.0	0.0	0.0	0.0	0.0	0.0	0.0
19.0	0.0	0.0	0.0	0.0	4.0	0.0	175.0
6.0	0.0	0.0	0.0	0.0	0.0	0.0	0.0
4.0	0.0	0.0	0.0	0.0	0.0	0.0	0.0
2.0	0.0	0.0	0.0	0.0	0.0	0.0	0.0
1.0	0.0	0.0	0.0	0.0	0.0	0.0	0.0
2.0	0.0	0.0	0.0	0.0	0.0	0.0	0.0
2.0	0.0	0.0	0.0	0.0	0.0	0.0	0.0
1.0	0.0	0.0	0.0	0.0	0.0	0.0	0.0
1.0	0.0	0.0	0.0	0.0	0.0	0.0	0.0
0.0	0.0	0.0	0.0	0.0	0.0	0.0	0.0
1.0	0.0	0.0	0.0	0.0	0.0	0.0	0.0
1.0	0.0	0.0	0.0	0.0	0.0	0.0	0.0
1.0	0.0	0.0	0.0	0.0	0.0	0.0	0.0
1.0	0.0	0.0	0.0	0.0	0.0	0.0	0.0
1.0	0.0	0.0	0.0	0.0	0.0	0.0	0.0

1.0	0.0	0.0	0.0	0.0	0.0	0.0	0.0
1.0	0.0	0.0	0.0	0.0	0.0	0.0	0.0
1.0	0.0	0.0	0.0	0.0	0.0	0.0	0.0
1.0	0.0	0.0	0.0	0.0	0.0	0.0	0.0
1.0	0.0	0.0	0.0	0.0	0.0	0.0	0.0
1.0	0.0	0.0	0.0	0.0	0.0	0.0	0.0
0.0	0.0	0.0	0.0	0.0	5.0	0.0	0.0
1.0	0.0	0.0	0.0	0.0	0.0	0.0	0.0
1.0	0.0	0.0	0.0	0.0	0.0	0.0	0.0
0.0	1589.0	0.0	0.0	0.0	0.0	0.0	0.0
0.0	53.0	0.0	0.0	0.0	0.0	0.0	0.0
0.0	32.0	0.0	0.0	0.0	0.0	0.0	0.0
0.0	7.0	0.0	0.0	0.0	0.0	0.0	0.0
0.0	5.0	0.0	0.0	0.0	0.0	0.0	0.0
0.0	3.0	0.0	0.0	0.0	0.0	0.0	0.0
0.0	1.0	0.0	2.0	0.0	0.0	0.0	0.0
0.0	1.0	0.0	0.0	0.0	0.0	0.0	0.0
0.0	3.0	0.0	0.0	0.0	0.0	0.0	0.0
0.0	3.0	0.0	0.0	0.0	0.0	0.0	0.0
0.0	1.0	0.0	0.0	0.0	0.0	0.0	0.0
0.0	1.0	0.0	0.0	0.0	0.0	0.0	0.0
0.0	2.0	0.0	0.0	0.0	0.0	0.0	0.0
0.0	2.0	0.0	0.0	0.0	0.0	0.0	0.0
0.0	1.0	0.0	0.0	0.0	0.0	0.0	2.0
0.0	1.0	0.0	0.0	0.0	0.0	0.0	0.0
0.0	1.0	0.0	0.0	0.0	0.0	0.0	0.0
0.0	2.0	0.0	0.0	0.0	0.0	0.0	0.0
0.0	1.0	0.0	0.0	0.0	0.0	0.0	0.0
0.0	2.0	0.0	0.0	0.0	0.0	0.0	0.0
0.0	1.0	0.0	0.0	0.0	0.0	0.0	0.0
0.0	1.0	0.0	0.0	0.0	0.0	0.0	0.0
0.0	2.0	0.0	0.0	0.0	0.0	0.0	0.0
0.0	1.0	0.0	0.0	0.0	0.0	0.0	0.0
0.0	1.0	0.0	0.0	0.0	0.0	0.0	0.0
0.0	1.0	0.0	0.0	0.0	0.0	0.0	0.0
0.0	1.0	0.0	0.0	0.0	0.0	0.0	0.0
0.0	1.0	0.0	0.0	0.0	0.0	0.0	0.0
0.0	1.0	0.0	0.0	0.0	0.0	0.0	0.0
0.0	1.0	0.0	0.0	0.0	0.0	0.0	0.0
0.0	1.0	0.0	0.0	0.0	0.0	0.0	0.0
0.0	1.0	0.0	0.0	0.0	0.0	0.0	0.0
0.0	0.0	36.0	0.0	0.0	0.0	0.0	0.0
0.0	0.0	13.0	0.0	0.0	0.0	0.0	0.0
0.0	0.0	5.0	0.0	0.0	0.0	0.0	0.0
0.0	0.0	5.0	0.0	0.0	0.0	0.0	0.0
0.0	0.0	6.0	0.0	0.0	0.0	0.0	0.0
0.0	0.0	2.0	1.0	0.0	0.0	0.0	0.0

0.0	0.0	3.0	0.0	0.0	0.0	0.0	0.0
0.0	0.0	2.0	0.0	0.0	0.0	0.0	0.0
0.0	0.0	2.0	0.0	0.0	0.0	0.0	0.0
0.0	0.0	1.0	0.0	0.0	0.0	0.0	0.0
0.0	0.0	0.0	0.0	0.0	0.0	0.0	0.0
0.0	0.0	1.0	0.0	0.0	0.0	0.0	0.0
0.0	0.0	1.0	0.0	0.0	0.0	0.0	0.0
0.0	0.0	1.0	0.0	0.0	0.0	0.0	0.0
0.0	0.0	1.0	0.0	0.0	0.0	0.0	0.0
0.0	0.0	0.0	269.0	0.0	0.0	9.0	3.0
0.0	0.0	0.0	24.0	0.0	0.0	0.0	0.0
0.0	0.0	0.0	23.0	0.0	0.0	0.0	0.0
0.0	0.0	0.0	18.0	0.0	0.0	0.0	0.0
0.0	0.0	0.0	8.0	0.0	0.0	0.0	0.0
0.0	0.0	0.0	4.0	0.0	0.0	0.0	0.0
0.0	0.0	0.0	6.0	0.0	0.0	0.0	0.0
0.0	0.0	0.0	6.0	0.0	0.0	0.0	0.0
0.0	0.0	0.0	5.0	0.0	0.0	0.0	0.0
0.0	0.0	0.0	2.0	0.0	0.0	0.0	0.0
0.0	0.0	0.0	3.0	0.0	0.0	0.0	0.0
0.0	0.0	0.0	4.0	0.0	0.0	0.0	0.0
0.0	0.0	0.0	2.0	0.0	0.0	0.0	0.0
0.0	0.0	0.0	2.0	0.0	0.0	0.0	0.0
0.0	0.0	0.0	2.0	0.0	0.0	0.0	0.0
0.0	0.0	0.0	1.0	0.0	0.0	0.0	0.0
0.0	0.0	0.0	1.0	0.0	0.0	0.0	0.0
0.0	0.0	0.0	3.0	0.0	0.0	0.0	0.0
0.0	0.0	0.0	1.0	0.0	0.0	0.0	0.0
0.0	0.0	0.0	4.0	0.0	0.0	0.0	0.0
0.0	0.0	0.0	2.0	0.0	0.0	0.0	0.0
0.0	0.0	0.0	4.0	0.0	0.0	0.0	0.0
0.0	0.0	0.0	1.0	0.0	0.0	0.0	0.0
0.0	0.0	0.0	3.0	0.0	0.0	0.0	0.0
0.0	0.0	0.0	1.0	0.0	0.0	0.0	0.0
0.0	0.0	0.0	1.0	0.0	0.0	0.0	0.0
0.0	0.0	0.0	1.0	0.0	0.0	0.0	0.0
0.0	0.0	0.0	1.0	0.0	0.0	0.0	0.0
0.0	0.0	0.0	1.0	0.0	0.0	0.0	0.0
0.0	0.0	0.0	2.0	0.0	0.0	0.0	0.0
0.0	0.0	0.0	0.0	0.0	0.0	0.0	0.0
0.0	0.0	0.0	1.0	0.0	0.0	0.0	0.0
0.0	0.0	0.0	3.0	0.0	0.0	0.0	0.0
0.0	0.0	0.0	2.0	0.0	0.0	0.0	0.0
0.0	0.0	0.0	1.0	0.0	0.0	0.0	0.0
0.0	0.0	0.0	1.0	0.0	0.0	0.0	0.0
0.0	0.0	0.0	1.0	0.0	0.0	0.0	0.0
0.0	0.0	0.0	1.0	0.0	0.0	0.0	0.0

0.0	0.0	0.0	3.0	0.0	0.0	0.0	0.0
0.0	0.0	0.0	1.0	0.0	0.0	0.0	0.0
0.0	0.0	0.0	2.0	0.0	0.0	0.0	0.0
0.0	0.0	0.0	1.0	0.0	0.0	0.0	0.0
0.0	0.0	0.0	1.0	0.0	0.0	0.0	0.0
0.0	0.0	0.0	1.0	0.0	0.0	0.0	0.0
0.0	0.0	0.0	0.0	0.0	0.0	0.0	0.0
0.0	0.0	0.0	1.0	0.0	0.0	0.0	0.0
0.0	0.0	0.0	1.0	0.0	0.0	0.0	0.0
0.0	0.0	0.0	1.0	0.0	0.0	0.0	0.0
0.0	0.0	0.0	1.0	0.0	0.0	0.0	0.0
0.0	0.0	0.0	1.0	0.0	0.0	0.0	0.0
0.0	0.0	0.0	0.0	0.0	0.0	0.0	0.0
0.0	0.0	0.0	1.0	0.0	0.0	0.0	0.0
0.0	0.0	0.0	1.0	0.0	0.0	0.0	0.0
0.0	0.0	0.0	1.0	0.0	0.0	0.0	0.0
0.0	0.0	0.0	2.0	0.0	0.0	0.0	0.0
0.0	0.0	0.0	1.0	0.0	0.0	0.0	0.0
0.0	0.0	0.0	1.0	0.0	0.0	0.0	0.0
0.0	0.0	0.0	1.0	0.0	0.0	0.0	0.0
0.0	0.0	0.0	1.0	0.0	0.0	0.0	0.0
0.0	0.0	0.0	1.0	0.0	0.0	0.0	0.0
0.0	0.0	0.0	1.0	0.0	0.0	0.0	0.0
0.0	0.0	0.0	1.0	0.0	0.0	0.0	0.0
0.0	0.0	0.0	1.0	0.0	0.0	0.0	0.0
0.0	0.0	0.0	0.0	0.0	0.0	0.0	0.0
0.0	0.0	0.0	2.0	0.0	0.0	0.0	0.0
0.0	0.0	0.0	2.0	0.0	0.0	0.0	0.0
0.0	0.0	0.0	1.0	0.0	0.0	0.0	0.0
0.0	0.0	0.0	2.0	0.0	0.0	0.0	0.0
0.0	0.0	0.0	2.0	0.0	0.0	0.0	0.0
0.0	0.0	0.0	1.0	0.0	0.0	0.0	0.0
0.0	0.0	0.0	1.0	0.0	0.0	0.0	0.0
0.0	0.0	0.0	1.0	0.0	0.0	0.0	0.0
0.0	0.0	0.0	1.0	0.0	0.0	0.0	0.0
0.0	0.0	0.0	1.0	0.0	0.0	0.0	0.0
0.0	0.0	0.0	1.0	0.0	0.0	0.0	0.0
0.0	0.0	0.0	2.0	0.0	0.0	0.0	0.0
0.0	0.0	0.0	1.0	0.0	0.0	0.0	0.0
0.0	0.0	0.0	1.0	0.0	0.0	0.0	0.0
0.0	0.0	0.0	1.0	0.0	0.0	0.0	0.0
0.0	0.0	0.0	1.0	0.0	0.0	0.0	0.0
0.0	0.0	0.0	1.0	0.0	0.0	0.0	0.0
0.0	0.0	0.0	1.0	0.0	0.0	0.0	0.0
0.0	0.0	0.0	1.0	0.0	0.0	0.0	0.0
0.0	0.0	0.0	1.0	0.0	0.0	0.0	0.0
0.0	0.0	0.0	1.0	0.0	0.0	0.0	0.0

0.0	0.0	0.0	1.0	0.0	0.0	0.0	0.0
0.0	0.0	0.0	1.0	0.0	0.0	0.0	0.0
0.0	0.0	0.0	1.0	0.0	0.0	0.0	0.0
0.0	0.0	0.0	0.0	395.0	0.0	0.0	0.0
0.0	0.0	0.0	0.0	105.0	0.0	0.0	0.0
0.0	0.0	0.0	0.0	38.0	0.0	0.0	0.0
0.0	0.0	0.0	0.0	34.0	0.0	0.0	0.0
0.0	0.0	0.0	0.0	18.0	0.0	0.0	0.0
0.0	0.0	0.0	0.0	2.0	0.0	0.0	0.0
0.0	0.0	0.0	0.0	3.0	0.0	0.0	0.0
0.0	0.0	0.0	0.0	1.0	0.0	0.0	0.0
0.0	0.0	0.0	0.0	2.0	0.0	0.0	0.0
0.0	0.0	0.0	0.0	2.0	0.0	0.0	0.0
0.0	0.0	0.0	0.0	2.0	0.0	0.0	0.0
0.0	0.0	0.0	0.0	1.0	0.0	0.0	0.0
0.0	0.0	0.0	0.0	2.0	0.0	0.0	0.0
0.0	0.0	0.0	0.0	0.0	373.0	0.0	0.0
0.0	0.0	0.0	0.0	0.0	218.0	0.0	0.0
0.0	0.0	0.0	0.0	0.0	169.0	0.0	0.0
0.0	0.0	0.0	0.0	0.0	125.0	0.0	0.0
0.0	0.0	0.0	0.0	0.0	87.0	0.0	0.0
0.0	0.0	0.0	0.0	0.0	38.0	0.0	0.0
0.0	0.0	0.0	0.0	0.0	38.0	0.0	0.0
0.0	0.0	0.0	0.0	0.0	29.0	0.0	0.0
0.0	0.0	0.0	0.0	0.0	29.0	0.0	0.0
0.0	0.0	0.0	0.0	0.0	23.0	0.0	0.0
0.0	0.0	0.0	0.0	0.0	21.0	0.0	0.0
0.0	0.0	0.0	0.0	0.0	18.0	0.0	0.0
0.0	0.0	0.0	0.0	0.0	11.0	0.0	0.0
0.0	0.0	0.0	0.0	0.0	7.0	0.0	0.0
0.0	0.0	0.0	0.0	0.0	10.0	0.0	0.0
0.0	0.0	0.0	0.0	0.0	5.0	0.0	0.0
0.0	0.0	0.0	0.0	0.0	7.0	0.0	0.0
0.0	0.0	0.0	0.0	0.0	3.0	0.0	0.0
0.0	0.0	0.0	0.0	0.0	4.0	0.0	0.0
0.0	0.0	0.0	0.0	0.0	3.0	0.0	0.0
0.0	0.0	0.0	0.0	0.0	2.0	0.0	0.0
0.0	0.0	0.0	0.0	0.0	2.0	0.0	0.0
0.0	0.0	0.0	0.0	0.0	1.0	0.0	0.0
0.0	0.0	0.0	0.0	0.0	2.0	0.0	0.0
0.0	0.0	0.0	0.0	0.0	1.0	1.0	0.0
0.0	0.0	0.0	0.0	0.0	1.0	0.0	0.0
0.0	0.0	0.0	0.0	0.0	2.0	0.0	0.0
0.0	0.0	0.0	0.0	0.0	0.0	0.0	0.0
0.0	0.0	0.0	0.0	0.0	1.0	0.0	0.0
0.0	0.0	0.0	0.0	0.0	1.0	0.0	0.0
0.0	0.0	0.0	0.0	0.0	1.0	0.0	0.0

0.0	0.0	0.0	0.0	0.0	2.0	0.0	0.0
0.0	0.0	0.0	0.0	0.0	1.0	0.0	0.0
0.0	0.0	0.0	0.0	0.0	0.0	0.0	0.0
0.0	0.0	0.0	0.0	0.0	1.0	0.0	0.0
0.0	0.0	0.0	0.0	0.0	1.0	0.0	0.0
0.0	0.0	0.0	0.0	0.0	0.0	0.0	0.0
0.0	0.0	0.0	0.0	0.0	1.0	0.0	0.0
0.0	0.0	0.0	0.0	0.0	1.0	0.0	0.0
0.0	0.0	0.0	0.0	0.0	1.0	0.0	0.0
0.0	0.0	0.0	0.0	0.0	0.0	1235.0	0.0
0.0	0.0	0.0	0.0	0.0	0.0	206.0	0.0
0.0	0.0	0.0	0.0	0.0	0.0	75.0	0.0
0.0	0.0	0.0	0.0	0.0	0.0	44.0	0.0
0.0	0.0	0.0	0.0	0.0	0.0	32.0	0.0
0.0	0.0	0.0	0.0	0.0	0.0	12.0	0.0
0.0	0.0	0.0	0.0	0.0	0.0	15.0	0.0
0.0	0.0	0.0	0.0	0.0	0.0	6.0	10.0
0.0	0.0	0.0	0.0	0.0	0.0	5.0	0.0
0.0	0.0	0.0	0.0	0.0	0.0	3.0	0.0
0.0	0.0	0.0	0.0	0.0	0.0	2.0	0.0
0.0	0.0	0.0	0.0	0.0	0.0	3.0	0.0
0.0	0.0	0.0	0.0	0.0	0.0	3.0	0.0
0.0	0.0	0.0	0.0	0.0	0.0	1.0	0.0
0.0	0.0	0.0	0.0	0.0	0.0	4.0	0.0
0.0	0.0	0.0	0.0	0.0	0.0	2.0	0.0
0.0	0.0	0.0	0.0	0.0	0.0	1.0	0.0
0.0	0.0	0.0	0.0	0.0	0.0	1.0	0.0
0.0	0.0	0.0	0.0	0.0	0.0	0.0	0.0
0.0	0.0	0.0	0.0	0.0	0.0	1.0	0.0
0.0	0.0	0.0	0.0	0.0	0.0	1.0	0.0
0.0	0.0	0.0	0.0	0.0	0.0	1.0	0.0
0.0	0.0	0.0	0.0	0.0	0.0	0.0	0.0
0.0	0.0	0.0	0.0	0.0	0.0	1.0	0.0
0.0	0.0	0.0	0.0	0.0	0.0	1.0	0.0
0.0	0.0	0.0	0.0	0.0	0.0	1.0	0.0
0.0	0.0	0.0	0.0	0.0	0.0	1.0	0.0
0.0	0.0	0.0	0.0	0.0	0.0	1.0	0.0
0.0	0.0	0.0	0.0	0.0	0.0	1.0	0.0
0.0	0.0	0.0	0.0	0.0	0.0	1.0	0.0
0.0	0.0	0.0	0.0	0.0	0.0	0.0	0.0
0.0	0.0	0.0	0.0	0.0	0.0	1.0	0.0
0.0	0.0	0.0	0.0	0.0	0.0	1.0	0.0
0.0	0.0	0.0	0.0	0.0	0.0	1.0	0.0
0.0	0.0	0.0	0.0	0.0	0.0	1.0	0.0
0.0	0.0	0.0	0.0	0.0	0.0	1.0	0.0
0.0	0.0	0.0	0.0	0.0	0.0	1.0	0.0
0.0	0.0	0.0	0.0	0.0	0.0	1.0	0.0

0.0	0.0	0.0	0.0	0.0	0.0	1.0	0.0
0.0	0.0	0.0	0.0	0.0	0.0	1.0	0.0
0.0	0.0	0.0	0.0	0.0	0.0	0.0	4.0
0.0	0.0	0.0	0.0	0.0	0.0	0.0	3.0
0.0	0.0	0.0	0.0	0.0	0.0	0.0	1.0
0.0	0.0	0.0	0.0	0.0	0.0	0.0	1.0
0.0	0.0	0.0	0.0	0.0	0.0	0.0	1.0
0.0	0.0	0.0	0.0	0.0	0.0	0.0	1.0
0.0	0.0	0.0	0.0	0.0	0.0	0.0	1.0
0.0	0.0	0.0	0.0	0.0	0.0	0.0	1.0
0.0	0.0	0.0	0.0	0.0	0.0	0.0	1.0
0.0	0.0	0.0	0.0	0.0	0.0	0.0	1.0
0.0	0.0	0.0	0.0	0.0	0.0	0.0	0.0
0.0	0.0	0.0	0.0	0.0	0.0	0.0	0.0
0.0	0.0	0.0	0.0	0.0	0.0	0.0	0.0
0.0	0.0	0.0	0.0	0.0	0.0	0.0	0.0
0.0	0.0	0.0	0.0	0.0	0.0	0.0	0.0
0.0	0.0	0.0	0.0	0.0	0.0	0.0	0.0
0.0	0.0	0.0	0.0	0.0	0.0	0.0	0.0
0.0	0.0	0.0	0.0	0.0	0.0	0.0	0.0
0.0	0.0	0.0	0.0	0.0	0.0	0.0	0.0
0.0	0.0	0.0	0.0	0.0	0.0	0.0	0.0
0.0	0.0	0.0	0.0	0.0	0.0	0.0	0.0
0.0	0.0	0.0	0.0	0.0	0.0	0.0	0.0
0.0	0.0	0.0	0.0	0.0	0.0	0.0	0.0
0.0	0.0	0.0	0.0	0.0	0.0	0.0	0.0
0.0	0.0	0.0	0.0	0.0	0.0	0.0	0.0
0.0	0.0	0.0	0.0	0.0	0.0	0.0	0.0
0.0	0.0	0.0	0.0	0.0	0.0	0.0	0.0
0.0	0.0	0.0	0.0	0.0	0.0	0.0	0.0
0.0	0.0	0.0	0.0	0.0	0.0	0.0	0.0
0.0	0.0	0.0	0.0	0.0	0.0	0.0	0.0
0.0	0.0	0.0	0.0	0.0	0.0	0.0	0.0
0.0	0.0	0.0	0.0	0.0	0.0	0.0	0.0
0.0	0.0	0.0	0.0	0.0	0.0	0.0	0.0
0.0	0.0	0.0	0.0	0.0	0.0	0.0	0.0
0.0	0.0	0.0	0.0	0.0	0.0	0.0	0.0
0.0	0.0	0.0	0.0	0.0	0.0	0.0	0.0
0.0	0.0	0.0	0.0	0.0	0.0	0.0	0.0
0.0	0.0	0.0	0.0	0.0	0.0	0.0	0.0
0.0	0.0	0.0	0.0	0.0	0.0	0.0	0.0
0.0	0.0	0.0	0.0	0.0	0.0	0.0	0.0
0.0	0.0	0.0	0.0	0.0	0.0	0.0	0.0
0.0	0.0	0.0	0.0	0.0	0.0	0.0	0.0
0.0	0.0	0.0	0.0	0.0	0.0	0.0	0.0
0.0	0.0	0.0	0.0	0.0	0.0	0.0	0.0
0.0	0.0	0.0	0.0	0.0	0.0	0.0	0.0

0.0	0.0	0.0	0.0	0.0	0.0	0.0	0.0
0.0	0.0	0.0	0.0	0.0	0.0	0.0	0.0
0.0	0.0	0.0	0.0	0.0	0.0	0.0	0.0
0.0	0.0	0.0	0.0	0.0	0.0	0.0	0.0
0.0	0.0	0.0	0.0	0.0	0.0	0.0	0.0
0.0	0.0	0.0	0.0	0.0	0.0	0.0	0.0
0.0	0.0	0.0	0.0	0.0	0.0	0.0	0.0
0.0	0.0	0.0	0.0	0.0	0.0	0.0	0.0
0.0	0.0	0.0	0.0	0.0	0.0	0.0	0.0
0.0	0.0	0.0	0.0	0.0	0.0	0.0	0.0
0.0	0.0	0.0	0.0	0.0	0.0	0.0	0.0
0.0	0.0	0.0	0.0	0.0	0.0	0.0	0.0
0.0	0.0	0.0	0.0	0.0	0.0	0.0	0.0
0.0	0.0	0.0	0.0	0.0	0.0	0.0	0.0
0.0	0.0	0.0	0.0	0.0	0.0	0.0	0.0
0.0	0.0	0.0	0.0	0.0	0.0	0.0	0.0
0.0	0.0	0.0	0.0	0.0	0.0	0.0	0.0
0.0	0.0	0.0	0.0	0.0	0.0	0.0	0.0
0.0	0.0	0.0	0.0	0.0	0.0	0.0	0.0
0.0	0.0	0.0	0.0	0.0	0.0	0.0	0.0
0.0	0.0	0.0	0.0	0.0	0.0	0.0	0.0
0.0	0.0	0.0	0.0	0.0	0.0	0.0	0.0
0.0	0.0	0.0	0.0	0.0	0.0	0.0	0.0
0.0	0.0	0.0	0.0	0.0	0.0	0.0	0.0
0.0	0.0	0.0	0.0	0.0	0.0	0.0	0.0
0.0	0.0	0.0	0.0	0.0	0.0	0.0	0.0
0.0	0.0	0.0	0.0	0.0	0.0	0.0	0.0
0.0	0.0	0.0	0.0	0.0	0.0	0.0	0.0
0.0	0.0	0.0	0.0	0.0	0.0	0.0	0.0
0.0	0.0	0.0	0.0	0.0	0.0	0.0	0.0
0.0	0.0	0.0	0.0	0.0	0.0	0.0	0.0
0.0	0.0	0.0	0.0	0.0	0.0	0.0	0.0
0.0	0.0	0.0	0.0	0.0	0.0	0.0	0.0
0.0	0.0	0.0	0.0	0.0	0.0	0.0	0.0
0.0	0.0	0.0	0.0	0.0	0.0	0.0	0.0
0.0	0.0	0.0	0.0	0.0	0.0	0.0	0.0
0.0	0.0	0.0	0.0	0.0	0.0	0.0	0.0
0.0	0.0	0.0	0.0	0.0	0.0	0.0	0.0
0.0	0.0	0.0	0.0	0.0	0.0	0.0	0.0
0.0	0.0	0.0	0.0	0.0	0.0	0.0	0.0
0.0	0.0	0.0	0.0	0.0	0.0	0.0	0.0
0.0	0.0	0.0	0.0	0.0	0.0	0.0	0.0
0.0	0.0	0.0	0.0	0.0	0.0	0.0	0.0
0.0	0.0	0.0	0.0	0.0	0.0	0.0	0.0
0.0	0.0	0.0	0.0	0.0	0.0	0.0	0.0
0.0	0.0	0.0	0.0	0.0	0.0	0.0	0.0
0.0	0.0	0.0	0.0	0.0	0.0	0.0	0.0

0.0	0.0	0.0	0.0	0.0	0.0	0.0	0.0
0.0	0.0	0.0	0.0	0.0	0.0	0.0	0.0
0.0	0.0	0.0	0.0	0.0	0.0	0.0	0.0
0.0	0.0	0.0	0.0	0.0	0.0	0.0	0.0
0.0	0.0	0.0	0.0	0.0	0.0	0.0	0.0
0.0	0.0	0.0	0.0	0.0	0.0	0.0	0.0
0.0	0.0	0.0	0.0	0.0	0.0	0.0	0.0
0.0	0.0	0.0	0.0	0.0	0.0	0.0	0.0
0.0	0.0	0.0	0.0	0.0	0.0	0.0	0.0
0.0	0.0	0.0	0.0	0.0	0.0	0.0	0.0
0.0	0.0	0.0	0.0	0.0	0.0	0.0	0.0
0.0	0.0	0.0	0.0	0.0	0.0	0.0	0.0
0.0	0.0	0.0	0.0	0.0	0.0	0.0	0.0
0.0	0.0	0.0	0.0	0.0	0.0	0.0	0.0
0.0	0.0	0.0	0.0	0.0	0.0	0.0	0.0
0.0	0.0	0.0	0.0	0.0	0.0	0.0	0.0
0.0	0.0	0.0	0.0	0.0	0.0	0.0	0.0
0.0	0.0	0.0	0.0	0.0	0.0	0.0	0.0
0.0	0.0	0.0	0.0	0.0	0.0	0.0	0.0
0.0	0.0	0.0	0.0	0.0	0.0	0.0	0.0
0.0	0.0	0.0	0.0	0.0	0.0	0.0	0.0
0.0	0.0	0.0	0.0	0.0	0.0	0.0	0.0
0.0	0.0	0.0	0.0	0.0	0.0	0.0	0.0
0.0	0.0	0.0	0.0	0.0	0.0	0.0	0.0
0.0	0.0	0.0	0.0	0.0	0.0	0.0	0.0
0.0	0.0	0.0	0.0	0.0	0.0	0.0	0.0
0.0	0.0	0.0	0.0	0.0	0.0	0.0	0.0
0.0	0.0	0.0	0.0	0.0	0.0	0.0	0.0
0.0	0.0	0.0	0.0	0.0	0.0	0.0	0.0
0.0	0.0	0.0	0.0	0.0	0.0	0.0	0.0
0.0	0.0	0.0	0.0	0.0	0.0	0.0	0.0
0.0	0.0	0.0	0.0	0.0	0.0	0.0	0.0
0.0	0.0	0.0	0.0	0.0	0.0	0.0	0.0
0.0	0.0	0.0	0.0	0.0	0.0	0.0	0.0
0.0	0.0	0.0	0.0	0.0	0.0	0.0	0.0
0.0	0.0	0.0	0.0	0.0	0.0	0.0	0.0
0.0	0.0	0.0	0.0	0.0	0.0	0.0	0.0
0.0	0.0	0.0	0.0	0.0	0.0	0.0	0.0
0.0	0.0	0.0	0.0	0.0	0.0	0.0	0.0
0.0	0.0	0.0	0.0	0.0	0.0	0.0	0.0
0.0	0.0	0.0	0.0	0.0	0.0	0.0	0.0
0.0	0.0	0.0	0.0	0.0	0.0	0.0	0.0
0.0	0.0	0.0	0.0	0.0	0.0	0.0	0.0
0.0	0.0	0.0	0.0	0.0	0.0	0.0	0.0
0.0	0.0	0.0	0.0	0.0	0.0	0.0	0.0
0.0	0.0	0.0	0.0	0.0	0.0	0.0	0.0
0.0	0.0	0.0	0.0	0.0	0.0	0.0	0.0

0.0	0.0	0.0	0.0	0.0	0.0	0.0	0.0
0.0	0.0	0.0	0.0	0.0	0.0	0.0	0.0
0.0	0.0	0.0	0.0	0.0	0.0	0.0	0.0
0.0	0.0	0.0	0.0	0.0	0.0	0.0	0.0
0.0	0.0	0.0	0.0	0.0	0.0	0.0	0.0
0.0	0.0	0.0	0.0	0.0	0.0	0.0	0.0
0.0	0.0	0.0	0.0	0.0	0.0	0.0	0.0
0.0	0.0	0.0	0.0	0.0	0.0	0.0	0.0
0.0	0.0	0.0	0.0	0.0	0.0	0.0	0.0
0.0	0.0	0.0	0.0	0.0	0.0	0.0	0.0
0.0	0.0	0.0	0.0	0.0	0.0	0.0	0.0
0.0	0.0	0.0	0.0	0.0	0.0	0.0	0.0
0.0	0.0	0.0	0.0	0.0	0.0	0.0	0.0
0.0	0.0	0.0	0.0	0.0	0.0	0.0	0.0
0.0	0.0	0.0	0.0	0.0	0.0	0.0	0.0
0.0	0.0	0.0	0.0	0.0	0.0	0.0	0.0
0.0	0.0	0.0	0.0	0.0	0.0	0.0	0.0
0.0	0.0	0.0	0.0	0.0	0.0	0.0	0.0
0.0	0.0	0.0	0.0	0.0	0.0	0.0	0.0
0.0	0.0	0.0	0.0	0.0	0.0	0.0	0.0
0.0	0.0	0.0	0.0	0.0	0.0	0.0	0.0
0.0	0.0	0.0	0.0	0.0	0.0	0.0	0.0
0.0	0.0	0.0	0.0	0.0	0.0	0.0	0.0
0.0	0.0	0.0	0.0	0.0	0.0	0.0	0.0
0.0	0.0	0.0	0.0	0.0	0.0	0.0	0.0
0.0	0.0	0.0	0.0	0.0	0.0	0.0	0.0
0.0	0.0	0.0	0.0	0.0	0.0	0.0	0.0
0.0	0.0	0.0	0.0	0.0	0.0	0.0	0.0
0.0	0.0	0.0	0.0	0.0	0.0	0.0	0.0
0.0	0.0	0.0	0.0	0.0	0.0	0.0	0.0
0.0	0.0	0.0	0.0	0.0	0.0	0.0	0.0
0.0	0.0	0.0	0.0	0.0	0.0	0.0	0.0
0.0	0.0	0.0	0.0	0.0	0.0	0.0	0.0
0.0	0.0	0.0	0.0	0.0	0.0	0.0	0.0
0.0	0.0	0.0	0.0	0.0	0.0	0.0	0.0
0.0	0.0	0.0	0.0	0.0	0.0	0.0	0.0
0.0	0.0	0.0	0.0	0.0	0.0	0.0	0.0
0.0	0.0	0.0	0.0	0.0	0.0	0.0	0.0
0.0	0.0	0.0	0.0	0.0	0.0	0.0	0.0
0.0	0.0	0.0	0.0	0.0	0.0	0.0	0.0
0.0	0.0	0.0	0.0	0.0	0.0	0.0	0.0
0.0	0.0	0.0	0.0	0.0	0.0	0.0	0.0
0.0	0.0	0.0	0.0	0.0	0.0	0.0	0.0
0.0	0.0	0.0	0.0	0.0	0.0	0.0	0.0
0.0	0.0	0.0	0.0	0.0	0.0	0.0	0.0
0.0	0.0	0.0	0.0	0.0	0.0	0.0	0.0
0.0	0.0	0.0	0.0	0.0	0.0	0.0	0.0

0.0	0.0	0.0	0.0	0.0	0.0	0.0	0.0
0.0	0.0	0.0	0.0	0.0	0.0	0.0	0.0
0.0	0.0	0.0	0.0	0.0	0.0	0.0	0.0
0.0	0.0	0.0	0.0	0.0	0.0	0.0	0.0
0.0	0.0	0.0	0.0	0.0	0.0	0.0	0.0
0.0	0.0	0.0	0.0	0.0	0.0	0.0	0.0
0.0	0.0	0.0	0.0	0.0	0.0	0.0	0.0
0.0	0.0	0.0	0.0	0.0	0.0	0.0	0.0
0.0	0.0	0.0	0.0	0.0	0.0	0.0	0.0
0.0	0.0	0.0	0.0	0.0	0.0	0.0	0.0
0.0	0.0	0.0	0.0	0.0	0.0	0.0	0.0
0.0	0.0	0.0	0.0	0.0	0.0	0.0	0.0
0.0	0.0	0.0	0.0	0.0	0.0	0.0	0.0
0.0	0.0	0.0	0.0	0.0	0.0	0.0	0.0
0.0	0.0	0.0	0.0	0.0	0.0	0.0	0.0
0.0	0.0	0.0	0.0	0.0	0.0	0.0	0.0
0.0	0.0	0.0	0.0	0.0	0.0	0.0	0.0
0.0	0.0	0.0	0.0	0.0	0.0	0.0	0.0
0.0	0.0	0.0	0.0	0.0	0.0	0.0	0.0
0.0	0.0	0.0	0.0	0.0	0.0	0.0	0.0
0.0	0.0	0.0	0.0	0.0	0.0	0.0	0.0
0.0	0.0	0.0	0.0	0.0	0.0	0.0	0.0
0.0	0.0	0.0	0.0	0.0	0.0	0.0	0.0
0.0	0.0	0.0	0.0	0.0	0.0	0.0	0.0
0.0	0.0	0.0	0.0	0.0	0.0	0.0	0.0
0.0	0.0	0.0	0.0	0.0	0.0	0.0	0.0
0.0	0.0	0.0	0.0	0.0	0.0	0.0	0.0
0.0	0.0	0.0	0.0	0.0	0.0	0.0	0.0
0.0	0.0	0.0	0.0	0.0	0.0	0.0	0.0
0.0	0.0	0.0	0.0	0.0	0.0	0.0	0.0
0.0	0.0	0.0	0.0	0.0	0.0	0.0	0.0
0.0	0.0	0.0	0.0	0.0	0.0	0.0	0.0
0.0	0.0	0.0	0.0	0.0	0.0	0.0	0.0
0.0	0.0	0.0	0.0	0.0	0.0	0.0	0.0
0.0	0.0	0.0	0.0	0.0	0.0	0.0	0.0
0.0	0.0	0.0	0.0	0.0	0.0	0.0	0.0
0.0	0.0	0.0	0.0	0.0	0.0	0.0	0.0
0.0	0.0	0.0	0.0	0.0	0.0	0.0	0.0
0.0	0.0	0.0	0.0	0.0	0.0	0.0	0.0
0.0	0.0	0.0	0.0	0.0	0.0	0.0	0.0
0.0	0.0	0.0	0.0	0.0	0.0	0.0	0.0
0.0	0.0	0.0	0.0	0.0	0.0	0.0	0.0
0.0	0.0	0.0	0.0	0.0	0.0	0.0	0.0
0.0	0.0	0.0	0.0	0.0	0.0	0.0	0.0
0.0	0.0	0.0	0.0	0.0	0.0	0.0	0.0
0.0	0.0	0.0	0.0	0.0	0.0	0.0	0.0
0.0	0.0	0.0	0.0	0.0	0.0	0.0	0.0

0.0	0.0	0.0	0.0	0.0	0.0	0.0	0.0
0.0	0.0	0.0	0.0	0.0	0.0	0.0	0.0
0.0	0.0	0.0	0.0	0.0	0.0	0.0	0.0
0.0	0.0	0.0	0.0	0.0	0.0	0.0	0.0
0.0	0.0	0.0	0.0	0.0	0.0	0.0	0.0
0.0	0.0	0.0	0.0	0.0	0.0	0.0	0.0
0.0	0.0	0.0	0.0	0.0	0.0	0.0	0.0
0.0	0.0	0.0	0.0	0.0	0.0	0.0	0.0
0.0	0.0	0.0	0.0	0.0	0.0	0.0	0.0
0.0	0.0	0.0	0.0	0.0	0.0	0.0	0.0
0.0	0.0	0.0	0.0	0.0	0.0	0.0	0.0
0.0	0.0	0.0	0.0	0.0	0.0	0.0	0.0
0.0	0.0	0.0	0.0	0.0	0.0	0.0	0.0
0.0	0.0	0.0	0.0	0.0	0.0	0.0	0.0
0.0	0.0	0.0	0.0	0.0	0.0	0.0	0.0
0.0	0.0	0.0	0.0	0.0	0.0	0.0	0.0
0.0	0.0	0.0	0.0	0.0	0.0	0.0	0.0
0.0	0.0	0.0	0.0	0.0	0.0	0.0	0.0
0.0	0.0	0.0	0.0	0.0	0.0	0.0	0.0
0.0	0.0	0.0	0.0	0.0	0.0	0.0	0.0
0.0	0.0	0.0	0.0	0.0	0.0	0.0	0.0
0.0	0.0	0.0	0.0	0.0	0.0	0.0	0.0
0.0	0.0	0.0	0.0	0.0	0.0	0.0	0.0
0.0	0.0	0.0	0.0	0.0	0.0	0.0	0.0
0.0	0.0	0.0	0.0	0.0	0.0	0.0	0.0
0.0	0.0	0.0	0.0	0.0	0.0	0.0	0.0
0.0	0.0	0.0	0.0	0.0	0.0	0.0	0.0
0.0	0.0	0.0	0.0	0.0	0.0	0.0	0.0
0.0	0.0	0.0	0.0	0.0	0.0	0.0	0.0
0.0	0.0	0.0	0.0	0.0	0.0	0.0	0.0
0.0	0.0	0.0	0.0	0.0	0.0	0.0	0.0
0.0	0.0	0.0	0.0	0.0	0.0	0.0	0.0
0.0	0.0	0.0	0.0	0.0	0.0	0.0	0.0
0.0	0.0	0.0	0.0	0.0	0.0	0.0	0.0
0.0	0.0	0.0	0.0	0.0	0.0	0.0	0.0
0.0	0.0	0.0	0.0	0.0	0.0	0.0	0.0
0.0	0.0	0.0	0.0	0.0	0.0	0.0	0.0
0.0	0.0	0.0	0.0	0.0	0.0	0.0	0.0
0.0	0.0	0.0	0.0	0.0	0.0	0.0	0.0
0.0	0.0	0.0	0.0	0.0	0.0	0.0	0.0
0.0	0.0	0.0	0.0	0.0	0.0	0.0	0.0
0.0	0.0	0.0	0.0	0.0	0.0	0.0	0.0
0.0	0.0	0.0	0.0	0.0	0.0	0.0	0.0
0.0	0.0	0.0	0.0	0.0	0.0	0.0	0.0
0.0	0.0	0.0	0.0	0.0	0.0	0.0	0.0
0.0	0.0	0.0	0.0	0.0	0.0	0.0	0.0
0.0	0.0	0.0	0.0	0.0	0.0	0.0	0.0
0.0	0.0	0.0	0.0	0.0	0.0	0.0	0.0

0.0	0.0	0.0	0.0	0.0	0.0	0.0	0.0
0.0	0.0	0.0	0.0	0.0	0.0	0.0	0.0
0.0	0.0	0.0	0.0	0.0	0.0	0.0	0.0
0.0	0.0	0.0	0.0	0.0	0.0	0.0	0.0
0.0	0.0	0.0	0.0	0.0	0.0	0.0	0.0
0.0	0.0	0.0	0.0	0.0	0.0	0.0	0.0
0.0	0.0	0.0	0.0	0.0	0.0	0.0	0.0
0.0	0.0	0.0	0.0	0.0	0.0	0.0	0.0
0.0	0.0	0.0	0.0	0.0	0.0	0.0	0.0
0.0	0.0	0.0	0.0	0.0	0.0	0.0	0.0
0.0	0.0	0.0	0.0	0.0	0.0	0.0	0.0
0.0	0.0	0.0	0.0	0.0	0.0	0.0	0.0
0.0	0.0	0.0	0.0	0.0	0.0	0.0	0.0
0.0	0.0	0.0	0.0	0.0	0.0	0.0	0.0
0.0	0.0	0.0	0.0	0.0	0.0	0.0	0.0
0.0	0.0	0.0	0.0	0.0	0.0	0.0	0.0
0.0	0.0	0.0	0.0	0.0	0.0	0.0	0.0
0.0	0.0	0.0	0.0	0.0	0.0	0.0	0.0
0.0	0.0	0.0	0.0	0.0	0.0	0.0	0.0
0.0	0.0	0.0	0.0	0.0	0.0	0.0	0.0
0.0	0.0	0.0	0.0	0.0	0.0	0.0	0.0
0.0	0.0	0.0	0.0	0.0	0.0	0.0	0.0
0.0	0.0	0.0	0.0	0.0	0.0	0.0	0.0
0.0	0.0	0.0	0.0	0.0	0.0	0.0	0.0
0.0	0.0	0.0	0.0	0.0	0.0	0.0	0.0
0.0	0.0	0.0	0.0	0.0	0.0	0.0	0.0
0.0	0.0	0.0	0.0	0.0	0.0	0.0	0.0
0.0	0.0	0.0	0.0	0.0	0.0	0.0	0.0
0.0	0.0	0.0	0.0	0.0	0.0	0.0	0.0
0.0	0.0	0.0	0.0	0.0	0.0	0.0	0.0
0.0	0.0	0.0	0.0	0.0	0.0	0.0	0.0
0.0	0.0	0.0	0.0	0.0	0.0	0.0	0.0
0.0	0.0	0.0	0.0	0.0	0.0	0.0	0.0
0.0	0.0	0.0	0.0	0.0	0.0	0.0	0.0
0.0	0.0	0.0	0.0	0.0	0.0	0.0	0.0
0.0	0.0	0.0	0.0	0.0	0.0	0.0	0.0
0.0	0.0	0.0	0.0	0.0	0.0	0.0	0.0
0.0	0.0	0.0	0.0	0.0	0.0	0.0	0.0
0.0	0.0	0.0	0.0	0.0	0.0	0.0	0.0
0.0	0.0	0.0	0.0	0.0	0.0	0.0	0.0
0.0	0.0	0.0	0.0	0.0	0.0	0.0	0.0
0.0	0.0	0.0	0.0	0.0	0.0	0.0	0.0
0.0	0.0	0.0	0.0	0.0	0.0	0.0	0.0
0.0	0.0	0.0	0.0	0.0	0.0	0.0	0.0
0.0	0.0	0.0	0.0	0.0	0.0	0.0	0.0
0.0	0.0	0.0	0.0	0.0	0.0	0.0	0.0
0.0	0.0	0.0	0.0	0.0	0.0	0.0	0.0
0.0	0.0	0.0	0.0	0.0	0.0	0.0	0.0

0.0	0.0	0.0	0.0	0.0	0.0	0.0	0.0
0.0	0.0	0.0	0.0	0.0	0.0	0.0	0.0
0.0	0.0	0.0	0.0	0.0	0.0	0.0	0.0
0.0	0.0	0.0	0.0	0.0	0.0	0.0	0.0
0.0	0.0	0.0	0.0	0.0	0.0	0.0	0.0
0.0	0.0	0.0	0.0	0.0	0.0	0.0	0.0
0.0	0.0	0.0	0.0	0.0	0.0	0.0	0.0
0.0	0.0	0.0	0.0	0.0	0.0	0.0	0.0
0.0	0.0	0.0	0.0	0.0	0.0	0.0	0.0
0.0	0.0	0.0	0.0	0.0	0.0	0.0	0.0
0.0	0.0	0.0	0.0	0.0	0.0	0.0	0.0
0.0	0.0	0.0	0.0	0.0	0.0	0.0	0.0
0.0	0.0	0.0	0.0	0.0	0.0	0.0	0.0
0.0	0.0	0.0	0.0	0.0	0.0	0.0	0.0
0.0	0.0	0.0	0.0	0.0	0.0	0.0	0.0
0.0	0.0	0.0	0.0	0.0	0.0	0.0	0.0
0.0	0.0	0.0	0.0	0.0	0.0	0.0	0.0
0.0	0.0	0.0	0.0	0.0	0.0	0.0	0.0
0.0	0.0	0.0	0.0	0.0	0.0	0.0	0.0
0.0	0.0	0.0	0.0	0.0	0.0	0.0	0.0
0.0	0.0	0.0	0.0	0.0	0.0	0.0	0.0
0.0	0.0	0.0	0.0	0.0	0.0	0.0	0.0
0.0	0.0	0.0	0.0	0.0	0.0	0.0	0.0
0.0	0.0	0.0	0.0	0.0	0.0	0.0	0.0
0.0	0.0	0.0	0.0	0.0	0.0	0.0	0.0
0.0	0.0	0.0	0.0	0.0	0.0	0.0	0.0
0.0	0.0	0.0	0.0	0.0	0.0	0.0	0.0
0.0	0.0	0.0	0.0	0.0	0.0	0.0	0.0
0.0	0.0	0.0	0.0	0.0	0.0	0.0	0.0
0.0	0.0	0.0	0.0	0.0	0.0	0.0	0.0
0.0	0.0	0.0	0.0	0.0	0.0	0.0	0.0
0.0	0.0	0.0	0.0	0.0	0.0	0.0	0.0
0.0	0.0	0.0	0.0	0.0	0.0	0.0	0.0
0.0	0.0	0.0	0.0	0.0	0.0	0.0	0.0
0.0	0.0	0.0	0.0	0.0	0.0	0.0	0.0
0.0	0.0	0.0	0.0	0.0	0.0	0.0	0.0
0.0	0.0	0.0	0.0	0.0	0.0	0.0	0.0
0.0	0.0	0.0	0.0	0.0	0.0	0.0	0.0
0.0	0.0	0.0	0.0	0.0	0.0	0.0	0.0
0.0	0.0	0.0	0.0	0.0	0.0	0.0	0.0
0.0	0.0	0.0	0.0	0.0	0.0	0.0	0.0
0.0	0.0	0.0	0.0	0.0	0.0	0.0	0.0
0.0	0.0	0.0	0.0	0.0	0.0	0.0	0.0
0.0	0.0	0.0	0.0	0.0	0.0	0.0	0.0
0.0	0.0	0.0	0.0	0.0	0.0	0.0	0.0
0.0	0.0	0.0	0.0	0.0	0.0	0.0	0.0
0.0	0.0	0.0	0.0	0.0	0.0	0.0	0.0

0.0	0.0	0.0	0.0	0.0	0.0	0.0	0.0
0.0	0.0	0.0	0.0	0.0	0.0	0.0	0.0
0.0	0.0	0.0	0.0	0.0	0.0	0.0	0.0
0.0	0.0	0.0	0.0	0.0	0.0	0.0	0.0
0.0	0.0	0.0	0.0	0.0	0.0	0.0	0.0
0.0	0.0	0.0	0.0	0.0	0.0	0.0	0.0
0.0	0.0	0.0	0.0	0.0	0.0	0.0	0.0
0.0	0.0	0.0	0.0	0.0	0.0	0.0	0.0
0.0	0.0	0.0	0.0	0.0	0.0	0.0	0.0
0.0	0.0	0.0	0.0	0.0	0.0	0.0	0.0
0.0	0.0	0.0	0.0	0.0	0.0	0.0	0.0
0.0	0.0	0.0	0.0	0.0	0.0	0.0	0.0
0.0	0.0	0.0	0.0	0.0	0.0	0.0	0.0
0.0	0.0	0.0	0.0	0.0	0.0	0.0	0.0
0.0	0.0	0.0	0.0	0.0	0.0	0.0	0.0
0.0	0.0	0.0	0.0	0.0	0.0	0.0	0.0
0.0	0.0	0.0	0.0	0.0	0.0	0.0	0.0
0.0	0.0	0.0	0.0	0.0	0.0	0.0	0.0
0.0	0.0	0.0	0.0	0.0	0.0	0.0	0.0
0.0	0.0	0.0	0.0	0.0	0.0	0.0	0.0
0.0	0.0	0.0	0.0	0.0	0.0	0.0	0.0
0.0	0.0	0.0	0.0	0.0	0.0	0.0	0.0
0.0	0.0	0.0	0.0	0.0	0.0	0.0	0.0
0.0	0.0	0.0	0.0	0.0	0.0	0.0	0.0
0.0	0.0	0.0	0.0	0.0	0.0	0.0	0.0
0.0	0.0	0.0	0.0	0.0	0.0	0.0	0.0
0.0	0.0	0.0	0.0	0.0	0.0	0.0	0.0
0.0	0.0	0.0	0.0	0.0	0.0	0.0	0.0
0.0	0.0	0.0	0.0	0.0	0.0	0.0	0.0
0.0	0.0	0.0	0.0	0.0	0.0	0.0	0.0
0.0	0.0	0.0	0.0	0.0	0.0	0.0	0.0
0.0	0.0	0.0	0.0	0.0	0.0	0.0	0.0
0.0	0.0	0.0	0.0	0.0	0.0	0.0	0.0
0.0	0.0	0.0	0.0	0.0	0.0	0.0	0.0
0.0	0.0	0.0	0.0	0.0	0.0	0.0	0.0
0.0	0.0	0.0	0.0	0.0	0.0	0.0	0.0
0.0	0.0	0.0	0.0	0.0	0.0	0.0	0.0
0.0	0.0	0.0	0.0	0.0	0.0	0.0	0.0
0.0	0.0	0.0	0.0	0.0	0.0	0.0	0.0
0.0	0.0	0.0	0.0	0.0	0.0	0.0	0.0
0.0	0.0	0.0	0.0	0.0	0.0	0.0	0.0
0.0	0.0	0.0	0.0	0.0	0.0	0.0	0.0
0.0	0.0	0.0	0.0	0.0	0.0	0.0	0.0
0.0	0.0	0.0	0.0	0.0	0.0	0.0	0.0
0.0	0.0	0.0	0.0	0.0	0.0	0.0	0.0
0.0	0.0	0.0	0.0	0.0	0.0	0.0	0.0
0.0	0.0	0.0	0.0	0.0	0.0	0.0	0.0

0.0	0.0	0.0	0.0	0.0	0.0	0.0	0.0
0.0	0.0	0.0	0.0	0.0	0.0	0.0	0.0
0.0	0.0	0.0	0.0	0.0	0.0	0.0	0.0
0.0	0.0	0.0	0.0	0.0	0.0	0.0	0.0
0.0	0.0	0.0	0.0	0.0	0.0	0.0	0.0
0.0	0.0	0.0	0.0	0.0	0.0	0.0	0.0
0.0	0.0	0.0	0.0	0.0	0.0	0.0	0.0
0.0	0.0	0.0	0.0	0.0	0.0	0.0	0.0
0.0	0.0	0.0	0.0	0.0	0.0	0.0	0.0
0.0	0.0	0.0	0.0	0.0	0.0	0.0	0.0
0.0	0.0	0.0	0.0	0.0	0.0	0.0	0.0
0.0	0.0	0.0	0.0	0.0	0.0	0.0	0.0
0.0	0.0	0.0	0.0	0.0	0.0	0.0	0.0
0.0	0.0	0.0	0.0	0.0	0.0	0.0	0.0
0.0	0.0	0.0	0.0	0.0	0.0	0.0	0.0
0.0	0.0	0.0	0.0	0.0	0.0	0.0	0.0
0.0	0.0	0.0	0.0	0.0	0.0	0.0	0.0
0.0	0.0	0.0	0.0	0.0	0.0	0.0	0.0
0.0	0.0	0.0	0.0	0.0	0.0	0.0	0.0
0.0	0.0	0.0	0.0	0.0	0.0	0.0	0.0
0.0	0.0	0.0	0.0	0.0	0.0	0.0	0.0
0.0	0.0	0.0	0.0	0.0	0.0	0.0	0.0
0.0	0.0	0.0	0.0	0.0	0.0	0.0	0.0
0.0	0.0	0.0	0.0	0.0	0.0	0.0	0.0
0.0	0.0	0.0	0.0	0.0	0.0	0.0	0.0
0.0	0.0	0.0	0.0	0.0	0.0	0.0	0.0
0.0	0.0	0.0	0.0	0.0	0.0	0.0	0.0
0.0	0.0	0.0	0.0	0.0	0.0	0.0	0.0
0.0	0.0	0.0	0.0	0.0	0.0	0.0	0.0
0.0	0.0	0.0	0.0	0.0	0.0	0.0	0.0
0.0	0.0	0.0	0.0	0.0	0.0	0.0	0.0
0.0	0.0	0.0	0.0	0.0	0.0	0.0	0.0
0.0	0.0	0.0	0.0	0.0	0.0	0.0	0.0
0.0	0.0	0.0	0.0	0.0	0.0	0.0	0.0
0.0	0.0	0.0	0.0	0.0	0.0	0.0	0.0
0.0	0.0	0.0	0.0	0.0	0.0	0.0	0.0
0.0	0.0	0.0	0.0	0.0	0.0	0.0	0.0
0.0	0.0	0.0	0.0	0.0	0.0	0.0	0.0
0.0	0.0	0.0	0.0	0.0	0.0	0.0	0.0
0.0	0.0	0.0	0.0	0.0	0.0	0.0	0.0
0.0	0.0	0.0	0.0	0.0	0.0	0.0	0.0
0.0	0.0	0.0	0.0	0.0	0.0	0.0	0.0
0.0	0.0	0.0	0.0	0.0	0.0	0.0	0.0
0.0	0.0	0.0	0.0	0.0	0.0	0.0	0.0
0.0	0.0	0.0	0.0	0.0	0.0	0.0	0.0
0.0	0.0	0.0	0.0	0.0	0.0	0.0	0.0
0.0	0.0	0.0	0.0	0.0	0.0	0.0	0.0

0.0	0.0	0.0	0.0	0.0	0.0	0.0	0.0
0.0	0.0	0.0	0.0	0.0	0.0	0.0	0.0
0.0	0.0	0.0	0.0	0.0	0.0	0.0	0.0
0.0	0.0	0.0	0.0	0.0	0.0	0.0	0.0
0.0	0.0	0.0	0.0	0.0	0.0	0.0	0.0
0.0	0.0	0.0	0.0	0.0	0.0	0.0	0.0
0.0	0.0	0.0	0.0	0.0	0.0	0.0	0.0
0.0	0.0	0.0	0.0	0.0	0.0	0.0	0.0
0.0	0.0	0.0	0.0	0.0	0.0	0.0	0.0
0.0	0.0	0.0	0.0	0.0	0.0	0.0	0.0
0.0	0.0	0.0	0.0	0.0	0.0	0.0	0.0
0.0	0.0	0.0	0.0	0.0	0.0	0.0	0.0
0.0	0.0	0.0	0.0	0.0	0.0	0.0	0.0
0.0	0.0	0.0	0.0	0.0	0.0	0.0	0.0
0.0	0.0	0.0	0.0	0.0	0.0	0.0	0.0
0.0	0.0	0.0	0.0	0.0	0.0	0.0	0.0
0.0	0.0	0.0	0.0	0.0	0.0	0.0	0.0
0.0	0.0	0.0	0.0	0.0	0.0	0.0	0.0
0.0	0.0	0.0	0.0	0.0	0.0	0.0	0.0
0.0	0.0	0.0	0.0	0.0	0.0	0.0	0.0
0.0	0.0	0.0	0.0	0.0	0.0	0.0	0.0
0.0	0.0	0.0	0.0	0.0	0.0	0.0	0.0
0.0	0.0	0.0	0.0	0.0	0.0	0.0	0.0
0.0	0.0	0.0	0.0	0.0	0.0	0.0	0.0
0.0	0.0	0.0	0.0	0.0	0.0	0.0	0.0
0.0	0.0	0.0	0.0	0.0	0.0	0.0	0.0
0.0	0.0	0.0	0.0	0.0	0.0	0.0	0.0
0.0	0.0	0.0	0.0	0.0	0.0	0.0	0.0
0.0	0.0	0.0	0.0	0.0	0.0	0.0	0.0
0.0	0.0	0.0	0.0	0.0	0.0	0.0	0.0
0.0	0.0	0.0	0.0	0.0	0.0	0.0	0.0
0.0	0.0	0.0	0.0	0.0	0.0	0.0	0.0
0.0	0.0	0.0	0.0	0.0	0.0	0.0	0.0
0.0	0.0	0.0	0.0	0.0	0.0	0.0	0.0
0.0	0.0	0.0	0.0	0.0	0.0	0.0	0.0
0.0	0.0	0.0	0.0	0.0	0.0	0.0	0.0
0.0	0.0	0.0	0.0	0.0	0.0	0.0	0.0
0.0	0.0	0.0	0.0	0.0	0.0	0.0	0.0
0.0	0.0	0.0	0.0	0.0	0.0	0.0	0.0
0.0	0.0	0.0	0.0	0.0	0.0	0.0	0.0
0.0	0.0	0.0	0.0	0.0	0.0	0.0	0.0
0.0	0.0	0.0	0.0	0.0	0.0	0.0	0.0
0.0	0.0	0.0	0.0	0.0	0.0	0.0	0.0
0.0	0.0	0.0	0.0	0.0	0.0	0.0	0.0
0.0	0.0	0.0	0.0	0.0	0.0	0.0	0.0
0.0	0.0	0.0	0.0	0.0	0.0	0.0	0.0
0.0	0.0	0.0	0.0	0.0	0.0	0.0	0.0

0.0	0.0	0.0	0.0	0.0	0.0	0.0	0.0
0.0	0.0	0.0	0.0	0.0	0.0	0.0	0.0
0.0	0.0	0.0	0.0	0.0	0.0	0.0	0.0
0.0	0.0	0.0	0.0	0.0	0.0	0.0	0.0
0.0	0.0	0.0	0.0	0.0	0.0	0.0	0.0
0.0	0.0	0.0	0.0	0.0	0.0	0.0	0.0
0.0	0.0	0.0	0.0	0.0	0.0	0.0	0.0
0.0	0.0	0.0	0.0	0.0	0.0	0.0	0.0
0.0	0.0	0.0	0.0	0.0	0.0	0.0	0.0
0.0	0.0	0.0	0.0	0.0	0.0	0.0	0.0
0.0	0.0	0.0	0.0	0.0	0.0	0.0	0.0
0.0	0.0	0.0	0.0	0.0	0.0	0.0	0.0
0.0	0.0	0.0	0.0	0.0	0.0	0.0	0.0
0.0	0.0	0.0	0.0	0.0	0.0	0.0	0.0
0.0	0.0	0.0	0.0	0.0	0.0	0.0	0.0
0.0	0.0	0.0	0.0	0.0	0.0	0.0	0.0
0.0	0.0	0.0	0.0	0.0	0.0	0.0	0.0
0.0	0.0	0.0	0.0	0.0	0.0	0.0	0.0
0.0	0.0	0.0	0.0	0.0	0.0	0.0	0.0
0.0	0.0	0.0	0.0	0.0	0.0	0.0	0.0
0.0	0.0	0.0	0.0	0.0	0.0	0.0	0.0
0.0	0.0	0.0	0.0	0.0	0.0	0.0	0.0
0.0	0.0	0.0	0.0	0.0	0.0	0.0	0.0
0.0	0.0	0.0	0.0	0.0	0.0	0.0	0.0
0.0	0.0	0.0	0.0	0.0	0.0	0.0	0.0
0.0	0.0	0.0	0.0	0.0	0.0	0.0	0.0
0.0	0.0	0.0	0.0	0.0	0.0	0.0	0.0
0.0	0.0	0.0	0.0	0.0	0.0	0.0	0.0
0.0	0.0	0.0	0.0	0.0	0.0	0.0	0.0
0.0	0.0	0.0	0.0	0.0	0.0	0.0	0.0
0.0	0.0	0.0	0.0	0.0	0.0	0.0	0.0
0.0	0.0	0.0	0.0	0.0	0.0	0.0	0.0
0.0	0.0	0.0	0.0	0.0	0.0	0.0	0.0
0.0	0.0	0.0	0.0	0.0	0.0	0.0	0.0
0.0	0.0	0.0	0.0	0.0	0.0	0.0	0.0
0.0	0.0	0.0	0.0	0.0	0.0	0.0	0.0
0.0	0.0	0.0	0.0	0.0	0.0	0.0	0.0
0.0	0.0	0.0	0.0	0.0	0.0	0.0	0.0
0.0	0.0	0.0	0.0	0.0	0.0	0.0	0.0
0.0	0.0	0.0	0.0	0.0	0.0	0.0	0.0
0.0	0.0	0.0	0.0	0.0	0.0	0.0	0.0
0.0	0.0	0.0	0.0	0.0	0.0	0.0	0.0
0.0	0.0	0.0	0.0	0.0	0.0	0.0	0.0
0.0	0.0	0.0	0.0	0.0	0.0	0.0	0.0
0.0	0.0	0.0	0.0	0.0	0.0	0.0	0.0
0.0	0.0	0.0	0.0	0.0	0.0	0.0	0.0
0.0	0.0	0.0	0.0	0.0	0.0	0.0	0.0

0.0	0.0	0.0	0.0	0.0	0.0	0.0	0.0
0.0	0.0	0.0	0.0	0.0	0.0	0.0	0.0
0.0	0.0	0.0	0.0	0.0	0.0	0.0	0.0
0.0	0.0	0.0	0.0	0.0	0.0	0.0	0.0
0.0	0.0	0.0	0.0	0.0	0.0	0.0	0.0
0.0	0.0	0.0	0.0	0.0	0.0	0.0	0.0
0.0	0.0	0.0	0.0	0.0	0.0	0.0	0.0
0.0	0.0	0.0	0.0	0.0	0.0	0.0	0.0
0.0	0.0	0.0	0.0	0.0	0.0	0.0	0.0
0.0	0.0	0.0	0.0	0.0	0.0	0.0	0.0
0.0	0.0	0.0	0.0	0.0	0.0	0.0	0.0
0.0	0.0	0.0	0.0	0.0	0.0	0.0	0.0
0.0	0.0	0.0	0.0	0.0	0.0	0.0	0.0
0.0	0.0	0.0	0.0	0.0	0.0	0.0	0.0
0.0	0.0	0.0	0.0	0.0	0.0	0.0	0.0
0.0	0.0	0.0	0.0	0.0	0.0	0.0	0.0
0.0	0.0	0.0	0.0	0.0	0.0	0.0	0.0
0.0	0.0	0.0	0.0	0.0	0.0	0.0	0.0
0.0	0.0	0.0	0.0	0.0	0.0	0.0	0.0
0.0	0.0	0.0	0.0	0.0	0.0	0.0	0.0
0.0	0.0	0.0	0.0	0.0	0.0	0.0	0.0
0.0	0.0	0.0	0.0	0.0	0.0	0.0	0.0
0.0	0.0	0.0	0.0	0.0	0.0	0.0	0.0
0.0	0.0	0.0	0.0	0.0	0.0	0.0	0.0
0.0	0.0	0.0	0.0	0.0	0.0	0.0	0.0
0.0	0.0	0.0	0.0	0.0	0.0	0.0	0.0
0.0	0.0	0.0	0.0	0.0	0.0	0.0	0.0
0.0	0.0	0.0	0.0	0.0	0.0	0.0	0.0
0.0	0.0	0.0	0.0	0.0	0.0	0.0	0.0
0.0	0.0	0.0	0.0	0.0	0.0	0.0	0.0
0.0	0.0	0.0	0.0	0.0	0.0	0.0	0.0
0.0	0.0	0.0	0.0	0.0	0.0	0.0	0.0
0.0	0.0	0.0	0.0	0.0	0.0	0.0	0.0
0.0	0.0	0.0	0.0	0.0	0.0	0.0	0.0
0.0	0.0	0.0	0.0	0.0	0.0	0.0	0.0
0.0	0.0	0.0	0.0	0.0	0.0	0.0	0.0
0.0	0.0	0.0	0.0	0.0	0.0	0.0	0.0
0.0	0.0	0.0	0.0	0.0	0.0	0.0	0.0
0.0	0.0	0.0	0.0	0.0	0.0	0.0	0.0
0.0	0.0	0.0	0.0	0.0	0.0	0.0	0.0
0.0	0.0	0.0	0.0	0.0	0.0	0.0	0.0
0.0	0.0	0.0	0.0	0.0	0.0	0.0	0.0
0.0	0.0	0.0	0.0	0.0	0.0	0.0	0.0
0.0	0.0	0.0	0.0	0.0	0.0	0.0	0.0
0.0	0.0	0.0	0.0	0.0	0.0	0.0	0.0
0.0	0.0	0.0	0.0	0.0	0.0	0.0	0.0
0.0	0.0	0.0	0.0	0.0	0.0	0.0	0.0

0.0	0.0	0.0	0.0	0.0	0.0	0.0	0.0
0.0	0.0	0.0	0.0	0.0	0.0	0.0	0.0
0.0	0.0	0.0	0.0	0.0	0.0	0.0	0.0
0.0	0.0	0.0	0.0	0.0	0.0	0.0	0.0
0.0	0.0	0.0	0.0	0.0	0.0	0.0	0.0
0.0	0.0	0.0	0.0	0.0	0.0	0.0	0.0
0.0	0.0	0.0	0.0	0.0	0.0	0.0	0.0
0.0	0.0	0.0	0.0	0.0	0.0	0.0	0.0
0.0	0.0	0.0	0.0	0.0	0.0	0.0	0.0
0.0	0.0	0.0	0.0	0.0	0.0	0.0	0.0
0.0	0.0	0.0	0.0	0.0	0.0	0.0	0.0
0.0	0.0	0.0	0.0	0.0	0.0	0.0	0.0
0.0	0.0	0.0	0.0	0.0	0.0	0.0	0.0
0.0	0.0	0.0	0.0	0.0	0.0	0.0	0.0
0.0	0.0	0.0	0.0	0.0	0.0	0.0	0.0
0.0	0.0	0.0	0.0	0.0	0.0	0.0	0.0
0.0	0.0	0.0	0.0	0.0	0.0	0.0	0.0
0.0	0.0	0.0	0.0	0.0	0.0	0.0	0.0
0.0	0.0	0.0	0.0	0.0	0.0	0.0	0.0
0.0	0.0	0.0	0.0	0.0	0.0	0.0	0.0
0.0	0.0	0.0	0.0	0.0	0.0	0.0	0.0
0.0	0.0	0.0	0.0	0.0	0.0	0.0	0.0
0.0	0.0	0.0	0.0	0.0	0.0	0.0	0.0
0.0	0.0	0.0	0.0	0.0	0.0	0.0	0.0
0.0	0.0	0.0	0.0	0.0	0.0	0.0	0.0
0.0	0.0	0.0	0.0	0.0	0.0	0.0	0.0
0.0	0.0	0.0	0.0	0.0	0.0	0.0	0.0
0.0	0.0	0.0	0.0	0.0	0.0	0.0	0.0
0.0	0.0	0.0	0.0	0.0	0.0	0.0	0.0
0.0	0.0	0.0	0.0	0.0	0.0	0.0	0.0
0.0	0.0	0.0	0.0	0.0	0.0	0.0	0.0
0.0	0.0	0.0	0.0	0.0	0.0	0.0	0.0
0.0	0.0	0.0	0.0	0.0	0.0	0.0	0.0
0.0	0.0	0.0	0.0	0.0	0.0	0.0	0.0
0.0	0.0	0.0	0.0	0.0	0.0	0.0	0.0
0.0	0.0	0.0	0.0	0.0	0.0	0.0	0.0
0.0	0.0	0.0	0.0	0.0	0.0	0.0	0.0
0.0	0.0	0.0	0.0	0.0	0.0	0.0	0.0
0.0	0.0	0.0	0.0	0.0	0.0	0.0	0.0
0.0	0.0	0.0	0.0	0.0	0.0	0.0	0.0
0.0	0.0	0.0	0.0	0.0	0.0	0.0	0.0
0.0	0.0	0.0	0.0	0.0	0.0	0.0	0.0
0.0	0.0	0.0	0.0	0.0	0.0	0.0	0.0
0.0	0.0	0.0	0.0	0.0	0.0	0.0	0.0
0.0	0.0	0.0	0.0	0.0	0.0	0.0	0.0
0.0	0.0	0.0	0.0	0.0	0.0	0.0	0.0
0.0	0.0	0.0	0.0	0.0	0.0	0.0	0.0

0.0	0.0	0.0	0.0	0.0	0.0	0.0	0.0
0.0	0.0	0.0	0.0	0.0	0.0	0.0	0.0
0.0	0.0	0.0	0.0	0.0	0.0	0.0	0.0
0.0	0.0	0.0	0.0	0.0	0.0	0.0	0.0
0.0	0.0	0.0	0.0	0.0	0.0	0.0	0.0
0.0	0.0	0.0	0.0	0.0	0.0	0.0	0.0
0.0	0.0	0.0	0.0	0.0	0.0	0.0	0.0
0.0	0.0	0.0	0.0	0.0	0.0	0.0	0.0
0.0	0.0	0.0	0.0	0.0	0.0	0.0	0.0
0.0	0.0	0.0	0.0	0.0	0.0	0.0	0.0
0.0	0.0	0.0	0.0	0.0	0.0	0.0	0.0
0.0	0.0	0.0	0.0	0.0	0.0	0.0	0.0
0.0	0.0	0.0	0.0	0.0	0.0	0.0	0.0
0.0	0.0	0.0	0.0	0.0	0.0	0.0	0.0
0.0	0.0	0.0	0.0	0.0	0.0	0.0	0.0
0.0	0.0	0.0	0.0	0.0	0.0	0.0	0.0
0.0	0.0	0.0	0.0	0.0	0.0	0.0	0.0
0.0	0.0	0.0	0.0	0.0	0.0	0.0	0.0
0.0	0.0	0.0	0.0	0.0	0.0	0.0	0.0
0.0	0.0	0.0	0.0	0.0	0.0	0.0	0.0
0.0	0.0	0.0	0.0	0.0	0.0	0.0	0.0
0.0	0.0	0.0	0.0	0.0	0.0	0.0	0.0
0.0	0.0	0.0	0.0	0.0	0.0	0.0	0.0
0.0	0.0	0.0	0.0	0.0	0.0	0.0	0.0
0.0	0.0	0.0	0.0	0.0	0.0	0.0	0.0
0.0	0.0	0.0	0.0	0.0	0.0	0.0	0.0
0.0	0.0	0.0	0.0	0.0	0.0	0.0	0.0
0.0	0.0	0.0	0.0	0.0	0.0	0.0	0.0
0.0	0.0	0.0	0.0	0.0	0.0	0.0	0.0
0.0	0.0	0.0	0.0	0.0	0.0	0.0	0.0
0.0	0.0	0.0	0.0	0.0	0.0	0.0	0.0
0.0	0.0	0.0	0.0	0.0	0.0	0.0	0.0
0.0	0.0	0.0	0.0	0.0	0.0	0.0	0.0
0.0	0.0	0.0	0.0	0.0	0.0	0.0	0.0
0.0	0.0	0.0	0.0	0.0	0.0	0.0	0.0
0.0	0.0	0.0	0.0	0.0	0.0	0.0	0.0
0.0	0.0	0.0	0.0	0.0	0.0	0.0	0.0
0.0	0.0	0.0	0.0	0.0	0.0	0.0	0.0
0.0	0.0	0.0	0.0	0.0	0.0	0.0	0.0
0.0	0.0	0.0	0.0	0.0	0.0	0.0	0.0
0.0	0.0	0.0	0.0	0.0	0.0	0.0	0.0
0.0	0.0	0.0	0.0	0.0	0.0	0.0	0.0
0.0	0.0	0.0	0.0	0.0	0.0	0.0	0.0
0.0	0.0	0.0	0.0	0.0	0.0	0.0	0.0
0.0	0.0	0.0	0.0	0.0	0.0	0.0	0.0
0.0	0.0	0.0	0.0	0.0	0.0	0.0	0.0
0.0	0.0	0.0	0.0	0.0	0.0	0.0	0.0

0.0	0.0	0.0	0.0	0.0	0.0	0.0	0.0
0.0	0.0	0.0	0.0	0.0	0.0	0.0	0.0
0.0	0.0	0.0	0.0	0.0	0.0	0.0	0.0
0.0	0.0	0.0	0.0	0.0	0.0	0.0	0.0
0.0	0.0	0.0	0.0	0.0	0.0	0.0	0.0
0.0	0.0	0.0	0.0	0.0	0.0	0.0	0.0
0.0	0.0	0.0	0.0	0.0	0.0	0.0	0.0
0.0	0.0	0.0	0.0	0.0	0.0	0.0	0.0
0.0	0.0	0.0	0.0	0.0	0.0	0.0	0.0
0.0	0.0	0.0	0.0	0.0	0.0	0.0	0.0
0.0	0.0	0.0	0.0	0.0	0.0	0.0	0.0
0.0	0.0	0.0	0.0	0.0	0.0	0.0	0.0
0.0	0.0	0.0	0.0	0.0	0.0	0.0	0.0
0.0	0.0	0.0	0.0	0.0	0.0	0.0	0.0
0.0	0.0	0.0	0.0	0.0	0.0	0.0	0.0
0.0	0.0	0.0	0.0	0.0	0.0	0.0	0.0
0.0	0.0	0.0	0.0	0.0	0.0	0.0	0.0
0.0	0.0	0.0	0.0	0.0	0.0	0.0	0.0
0.0	0.0	0.0	0.0	0.0	0.0	0.0	0.0
0.0	0.0	0.0	0.0	0.0	0.0	0.0	0.0
0.0	0.0	0.0	0.0	0.0	0.0	0.0	0.0
0.0	0.0	0.0	0.0	0.0	0.0	0.0	0.0
0.0	0.0	0.0	0.0	0.0	0.0	0.0	0.0
0.0	0.0	0.0	0.0	0.0	0.0	0.0	0.0
0.0	0.0	0.0	0.0	0.0	0.0	0.0	0.0
0.0	0.0	0.0	0.0	0.0	0.0	0.0	0.0
0.0	0.0	0.0	0.0	0.0	0.0	0.0	0.0
0.0	0.0	0.0	0.0	0.0	0.0	0.0	0.0
0.0	0.0	0.0	0.0	0.0	0.0	0.0	0.0
0.0	0.0	0.0	0.0	0.0	0.0	0.0	0.0
0.0	0.0	0.0	0.0	0.0	0.0	0.0	0.0
0.0	0.0	0.0	0.0	0.0	0.0	0.0	0.0
0.0	0.0	0.0	0.0	0.0	0.0	0.0	0.0
0.0	0.0	0.0	0.0	0.0	0.0	0.0	0.0
0.0	0.0	0.0	0.0	0.0	0.0	0.0	0.0
0.0	0.0	0.0	0.0	0.0	0.0	0.0	0.0
0.0	0.0	0.0	0.0	0.0	0.0	0.0	0.0
0.0	0.0	0.0	0.0	0.0	0.0	0.0	0.0
0.0	0.0	0.0	0.0	0.0	0.0	0.0	0.0
0.0	0.0	0.0	0.0	0.0	0.0	0.0	0.0
0.0	0.0	0.0	0.0	0.0	0.0	0.0	0.0
0.0	0.0	0.0	0.0	0.0	0.0	0.0	0.0
0.0	0.0	0.0	0.0	0.0	0.0	0.0	0.0
0.0	0.0	0.0	0.0	0.0	0.0	0.0	0.0
0.0	0.0	0.0	0.0	0.0	0.0	0.0	0.0
0.0	0.0	0.0	0.0	0.0	0.0	0.0	0.0
0.0	0.0	0.0	0.0	0.0	0.0	0.0	0.0
0.0	0.0	0.0	0.0	0.0	0.0	0.0	0.0

0.0	0.0	0.0	0.0	0.0	0.0	0.0	0.0
0.0	0.0	0.0	0.0	0.0	0.0	0.0	0.0
0.0	0.0	0.0	0.0	0.0	0.0	0.0	0.0
0.0	0.0	0.0	0.0	0.0	0.0	0.0	0.0
0.0	0.0	0.0	0.0	0.0	0.0	0.0	0.0
0.0	0.0	0.0	0.0	0.0	0.0	0.0	0.0
0.0	0.0	0.0	0.0	0.0	0.0	0.0	0.0
0.0	0.0	0.0	0.0	0.0	0.0	0.0	0.0
0.0	0.0	0.0	0.0	0.0	0.0	0.0	0.0
0.0	0.0	0.0	0.0	0.0	0.0	0.0	0.0
0.0	0.0	0.0	0.0	0.0	0.0	0.0	0.0
0.0	0.0	0.0	0.0	0.0	0.0	0.0	0.0
0.0	0.0	0.0	0.0	0.0	0.0	0.0	0.0
0.0	0.0	0.0	0.0	0.0	0.0	0.0	0.0
0.0	0.0	0.0	0.0	0.0	0.0	0.0	0.0
0.0	0.0	0.0	0.0	0.0	0.0	0.0	0.0
0.0	0.0	0.0	0.0	0.0	0.0	0.0	0.0
0.0	0.0	0.0	0.0	0.0	0.0	0.0	0.0
0.0	0.0	0.0	0.0	0.0	0.0	0.0	0.0
0.0	0.0	0.0	0.0	0.0	0.0	0.0	0.0
0.0	0.0	0.0	0.0	0.0	0.0	0.0	0.0
0.0	0.0	0.0	0.0	0.0	0.0	0.0	0.0
0.0	0.0	0.0	0.0	0.0	0.0	0.0	0.0
0.0	0.0	0.0	0.0	0.0	0.0	0.0	0.0
0.0	0.0	0.0	0.0	0.0	0.0	0.0	0.0
0.0	0.0	0.0	0.0	0.0	0.0	0.0	0.0
0.0	0.0	0.0	0.0	0.0	0.0	0.0	0.0
0.0	0.0	0.0	0.0	0.0	0.0	0.0	0.0
0.0	0.0	0.0	0.0	0.0	0.0	0.0	0.0
0.0	0.0	0.0	0.0	0.0	0.0	0.0	0.0
0.0	0.0	0.0	0.0	0.0	0.0	0.0	0.0
0.0	0.0	0.0	0.0	0.0	0.0	0.0	0.0
0.0	0.0	0.0	0.0	0.0	0.0	0.0	0.0
0.0	0.0	0.0	0.0	0.0	0.0	0.0	0.0
0.0	0.0	0.0	0.0	0.0	0.0	0.0	0.0
0.0	0.0	0.0	0.0	0.0	0.0	0.0	0.0
0.0	0.0	0.0	0.0	0.0	0.0	0.0	0.0
0.0	0.0	0.0	0.0	0.0	0.0	0.0	0.0
0.0	0.0	0.0	0.0	0.0	0.0	0.0	0.0
0.0	0.0	0.0	0.0	0.0	0.0	0.0	0.0
0.0	0.0	0.0	0.0	0.0	0.0	0.0	0.0
0.0	0.0	0.0	0.0	0.0	0.0	0.0	0.0
0.0	0.0	0.0	0.0	0.0	0.0	0.0	0.0
0.0	0.0	0.0	0.0	0.0	0.0	0.0	0.0
0.0	0.0	0.0	0.0	0.0	0.0	0.0	0.0
0.0	0.0	0.0	0.0	0.0	0.0	0.0	0.0
0.0	0.0	0.0	0.0	0.0	0.0	0.0	0.0
0.0	0.0	0.0	0.0	0.0	0.0	0.0	0.0

0.0	0.0	0.0	0.0	0.0	0.0	0.0	0.0
0.0	0.0	0.0	0.0	0.0	0.0	0.0	0.0
0.0	0.0	0.0	0.0	0.0	0.0	0.0	0.0
0.0	0.0	0.0	0.0	0.0	0.0	0.0	0.0
0.0	0.0	0.0	0.0	0.0	0.0	0.0	0.0
0.0	0.0	0.0	0.0	0.0	0.0	0.0	0.0
0.0	0.0	0.0	0.0	0.0	0.0	0.0	0.0
0.0	0.0	0.0	0.0	0.0	0.0	0.0	0.0
0.0	0.0	0.0	0.0	0.0	0.0	0.0	0.0
0.0	0.0	0.0	0.0	0.0	0.0	0.0	0.0
0.0	0.0	0.0	0.0	0.0	0.0	0.0	0.0
0.0	0.0	0.0	0.0	0.0	0.0	0.0	0.0
0.0	0.0	0.0	0.0	0.0	0.0	0.0	0.0
0.0	0.0	0.0	0.0	0.0	0.0	0.0	0.0
0.0	0.0	0.0	0.0	0.0	0.0	0.0	0.0
0.0	0.0	0.0	0.0	0.0	0.0	0.0	0.0
0.0	0.0	0.0	0.0	0.0	0.0	0.0	0.0
0.0	0.0	0.0	0.0	0.0	0.0	0.0	0.0
0.0	0.0	0.0	0.0	0.0	0.0	0.0	0.0
0.0	0.0	0.0	0.0	0.0	0.0	0.0	0.0
0.0	0.0	0.0	0.0	0.0	0.0	0.0	0.0
0.0	0.0	0.0	0.0	0.0	0.0	0.0	0.0
0.0	0.0	0.0	0.0	0.0	0.0	0.0	0.0
0.0	0.0	0.0	0.0	0.0	0.0	0.0	0.0
0.0	0.0	0.0	0.0	0.0	0.0	0.0	0.0
0.0	0.0	0.0	0.0	0.0	0.0	0.0	0.0
0.0	0.0	0.0	0.0	0.0	0.0	0.0	0.0
0.0	0.0	0.0	0.0	0.0	0.0	0.0	0.0
0.0	0.0	0.0	0.0	0.0	0.0	0.0	0.0
0.0	0.0	0.0	0.0	0.0	0.0	0.0	0.0
0.0	0.0	0.0	0.0	0.0	0.0	0.0	0.0
0.0	0.0	0.0	0.0	0.0	0.0	0.0	0.0
0.0	0.0	0.0	0.0	0.0	0.0	0.0	0.0
0.0	0.0	0.0	0.0	0.0	0.0	0.0	0.0
0.0	0.0	0.0	0.0	0.0	0.0	0.0	0.0
0.0	0.0	0.0	0.0	0.0	0.0	0.0	0.0
0.0	0.0	0.0	0.0	0.0	0.0	0.0	0.0
0.0	0.0	0.0	0.0	0.0	0.0	0.0	0.0
0.0	0.0	0.0	0.0	0.0	0.0	0.0	0.0
0.0	0.0	0.0	0.0	0.0	0.0	0.0	0.0
0.0	0.0	0.0	0.0	0.0	0.0	0.0	0.0
0.0	0.0	0.0	0.0	0.0	0.0	0.0	0.0
0.0	0.0	0.0	0.0	0.0	0.0	0.0	0.0
0.0	0.0	0.0	0.0	0.0	0.0	0.0	0.0
0.0	0.0	0.0	0.0	0.0	0.0	0.0	0.0
0.0	0.0	0.0	0.0	0.0	0.0	0.0	0.0
0.0	0.0	0.0	0.0	0.0	0.0	0.0	0.0

0.0	0.0	0.0	0.0	0.0	0.0	0.0	0.0
0.0	0.0	0.0	0.0	0.0	0.0	0.0	0.0
0.0	0.0	0.0	0.0	0.0	0.0	0.0	0.0
0.0	0.0	0.0	0.0	0.0	0.0	0.0	0.0
0.0	0.0	0.0	0.0	0.0	0.0	0.0	0.0
0.0	0.0	0.0	0.0	0.0	0.0	0.0	0.0
0.0	0.0	0.0	0.0	0.0	0.0	0.0	0.0
0.0	0.0	0.0	0.0	0.0	0.0	0.0	0.0
0.0	0.0	0.0	0.0	0.0	0.0	0.0	0.0
0.0	0.0	0.0	0.0	0.0	0.0	0.0	0.0
0.0	0.0	0.0	0.0	0.0	0.0	0.0	0.0
0.0	0.0	0.0	0.0	0.0	0.0	0.0	0.0
0.0	0.0	0.0	0.0	0.0	0.0	0.0	0.0
0.0	0.0	0.0	0.0	0.0	0.0	0.0	0.0
0.0	0.0	0.0	0.0	0.0	0.0	0.0	0.0
0.0	0.0	0.0	0.0	0.0	0.0	0.0	0.0
0.0	0.0	0.0	0.0	0.0	0.0	0.0	0.0
0.0	0.0	0.0	0.0	0.0	0.0	0.0	0.0
0.0	0.0	0.0	0.0	0.0	0.0	0.0	0.0
0.0	0.0	0.0	0.0	0.0	0.0	0.0	0.0
0.0	0.0	0.0	0.0	0.0	0.0	0.0	0.0
0.0	0.0	0.0	0.0	0.0	0.0	0.0	0.0
0.0	0.0	0.0	0.0	0.0	0.0	0.0	0.0
0.0	0.0	0.0	0.0	0.0	0.0	0.0	0.0
0.0	0.0	0.0	0.0	0.0	0.0	0.0	0.0
0.0	0.0	0.0	0.0	0.0	0.0	0.0	0.0
0.0	0.0	0.0	0.0	0.0	0.0	0.0	0.0
0.0	0.0	0.0	0.0	0.0	0.0	0.0	0.0
0.0	0.0	0.0	0.0	0.0	0.0	0.0	0.0
0.0	0.0	0.0	0.0	0.0	0.0	0.0	0.0
0.0	0.0	0.0	0.0	0.0	0.0	0.0	0.0
0.0	0.0	0.0	0.0	0.0	0.0	0.0	0.0
0.0	0.0	0.0	0.0	0.0	0.0	0.0	0.0
0.0	0.0	0.0	0.0	0.0	0.0	0.0	0.0
0.0	0.0	0.0	0.0	0.0	0.0	0.0	0.0
0.0	0.0	0.0	0.0	0.0	0.0	0.0	0.0
0.0	0.0	0.0	0.0	0.0	0.0	0.0	0.0
0.0	0.0	0.0	0.0	0.0	0.0	0.0	0.0
0.0	0.0	0.0	0.0	0.0	0.0	0.0	0.0
0.0	0.0	0.0	0.0	0.0	0.0	0.0	0.0
0.0	0.0	0.0	0.0	0.0	0.0	0.0	0.0
0.0	0.0	0.0	0.0	0.0	0.0	0.0	0.0
0.0	0.0	0.0	0.0	0.0	0.0	0.0	0.0
0.0	0.0	0.0	0.0	0.0	0.0	0.0	0.0
0.0	0.0	0.0	0.0	0.0	0.0	0.0	0.0
0.0	0.0	0.0	0.0	0.0	0.0	0.0	0.0
0.0	0.0	0.0	0.0	0.0	0.0	0.0	0.0

0.0	0.0	0.0	0.0	0.0	0.0	0.0	0.0
0.0	0.0	0.0	0.0	0.0	0.0	0.0	0.0
0.0	0.0	0.0	0.0	0.0	0.0	0.0	0.0
0.0	0.0	0.0	0.0	0.0	0.0	0.0	0.0
0.0	0.0	0.0	0.0	0.0	0.0	0.0	0.0
0.0	0.0	0.0	0.0	0.0	0.0	0.0	0.0
0.0	0.0	0.0	0.0	0.0	0.0	0.0	0.0
0.0	0.0	0.0	0.0	0.0	0.0	0.0	0.0
0.0	0.0	0.0	0.0	0.0	0.0	0.0	0.0
0.0	0.0	0.0	0.0	0.0	0.0	0.0	0.0
0.0	0.0	0.0	0.0	0.0	0.0	0.0	0.0
0.0	0.0	0.0	0.0	0.0	0.0	0.0	0.0
0.0	0.0	0.0	0.0	0.0	0.0	0.0	0.0
0.0	0.0	0.0	0.0	0.0	0.0	0.0	0.0
0.0	0.0	0.0	0.0	0.0	0.0	0.0	0.0
0.0	0.0	0.0	0.0	0.0	0.0	0.0	0.0
0.0	0.0	0.0	0.0	0.0	0.0	0.0	0.0
0.0	0.0	0.0	0.0	0.0	0.0	0.0	0.0
0.0	0.0	0.0	0.0	0.0	0.0	0.0	0.0
0.0	0.0	0.0	0.0	0.0	0.0	0.0	0.0
0.0	0.0	0.0	0.0	0.0	0.0	0.0	0.0
0.0	0.0	0.0	0.0	0.0	0.0	0.0	0.0
0.0	0.0	0.0	0.0	0.0	0.0	0.0	0.0
0.0	0.0	0.0	0.0	0.0	0.0	0.0	0.0
0.0	0.0	0.0	0.0	0.0	0.0	0.0	0.0
0.0	0.0	0.0	0.0	0.0	0.0	0.0	0.0
0.0	0.0	0.0	0.0	0.0	0.0	0.0	0.0
0.0	0.0	0.0	0.0	0.0	0.0	0.0	0.0
0.0	0.0	0.0	0.0	0.0	0.0	0.0	0.0
0.0	0.0	0.0	0.0	0.0	0.0	0.0	0.0
0.0	0.0	0.0	0.0	0.0	0.0	0.0	0.0
0.0	0.0	0.0	0.0	0.0	0.0	0.0	0.0
0.0	0.0	0.0	0.0	0.0	0.0	0.0	0.0
0.0	0.0	0.0	0.0	0.0	0.0	0.0	0.0
0.0	0.0	0.0	0.0	0.0	0.0	0.0	0.0
0.0	0.0	0.0	0.0	0.0	0.0	0.0	0.0
0.0	0.0	0.0	0.0	0.0	0.0	0.0	0.0
0.0	0.0	0.0	0.0	0.0	0.0	0.0	0.0
0.0	0.0	0.0	0.0	0.0	0.0	0.0	0.0
0.0	0.0	0.0	0.0	0.0	0.0	0.0	0.0
0.0	0.0	0.0	0.0	0.0	0.0	0.0	0.0
0.0	0.0	0.0	0.0	0.0	0.0	0.0	0.0
0.0	0.0	0.0	0.0	0.0	0.0	0.0	0.0
0.0	0.0	0.0	0.0	0.0	0.0	0.0	0.0
0.0	0.0	0.0	0.0	0.0	0.0	0.0	0.0
0.0	0.0	0.0	0.0	0.0	0.0	0.0	0.0
0.0	0.0	0.0	0.0	0.0	0.0	0.0	0.0
0.0	0.0	0.0	0.0	0.0	0.0	0.0	0.0

0.0	0.0	0.0	0.0	0.0	0.0	0.0	0.0
0.0	0.0	0.0	0.0	0.0	0.0	0.0	0.0
0.0	0.0	0.0	0.0	0.0	0.0	0.0	0.0
0.0	0.0	0.0	0.0	0.0	0.0	0.0	0.0
0.0	0.0	0.0	0.0	0.0	0.0	0.0	0.0
0.0	0.0	0.0	0.0	0.0	0.0	0.0	0.0
0.0	0.0	0.0	0.0	0.0	0.0	0.0	0.0
0.0	0.0	0.0	0.0	0.0	0.0	0.0	0.0
0.0	0.0	0.0	0.0	0.0	0.0	0.0	0.0
0.0	0.0	0.0	0.0	0.0	0.0	0.0	0.0
0.0	0.0	0.0	0.0	0.0	0.0	0.0	0.0
0.0	0.0	0.0	0.0	0.0	0.0	0.0	0.0
0.0	0.0	0.0	0.0	0.0	0.0	0.0	0.0
0.0	0.0	0.0	0.0	0.0	0.0	0.0	0.0
0.0	0.0	0.0	0.0	0.0	0.0	0.0	0.0
0.0	0.0	0.0	0.0	0.0	0.0	0.0	0.0
0.0	0.0	0.0	0.0	0.0	0.0	0.0	0.0
0.0	0.0	0.0	0.0	0.0	0.0	0.0	0.0
0.0	0.0	0.0	0.0	0.0	0.0	0.0	0.0
0.0	0.0	0.0	0.0	0.0	0.0	0.0	0.0
0.0	0.0	0.0	0.0	0.0	0.0	0.0	0.0
0.0	0.0	0.0	0.0	0.0	0.0	0.0	0.0
0.0	0.0	0.0	0.0	0.0	0.0	0.0	0.0
0.0	0.0	0.0	0.0	0.0	0.0	0.0	0.0
0.0	0.0	0.0	0.0	0.0	0.0	0.0	0.0
0.0	0.0	0.0	0.0	0.0	0.0	0.0	0.0
0.0	0.0	0.0	0.0	0.0	0.0	0.0	0.0
0.0	0.0	0.0	0.0	0.0	0.0	0.0	0.0
0.0	0.0	0.0	0.0	0.0	0.0	0.0	0.0
0.0	0.0	0.0	0.0	0.0	0.0	0.0	0.0
0.0	0.0	0.0	0.0	0.0	0.0	0.0	0.0
0.0	0.0	0.0	0.0	0.0	0.0	0.0	0.0
0.0	0.0	0.0	0.0	0.0	0.0	0.0	0.0
0.0	0.0	0.0	0.0	0.0	0.0	0.0	0.0
0.0	0.0	0.0	0.0	0.0	0.0	0.0	0.0
0.0	0.0	0.0	0.0	0.0	0.0	0.0	0.0
0.0	0.0	0.0	0.0	0.0	0.0	0.0	0.0
0.0	0.0	0.0	0.0	0.0	0.0	0.0	0.0
0.0	0.0	0.0	0.0	0.0	0.0	0.0	0.0
0.0	0.0	0.0	0.0	0.0	0.0	0.0	0.0
0.0	0.0	0.0	0.0	0.0	0.0	0.0	0.0
0.0	0.0	0.0	0.0	0.0	0.0	0.0	0.0
0.0	0.0	0.0	0.0	0.0	0.0	0.0	0.0
0.0	0.0	0.0	0.0	0.0	0.0	0.0	0.0
0.0	0.0	0.0	0.0	0.0	0.0	0.0	0.0
0.0	0.0	0.0	0.0	0.0	0.0	0.0	0.0
0.0	0.0	0.0	0.0	0.0	0.0	0.0	0.0

0.0	0.0	0.0	0.0	0.0	0.0	0.0	0.0
0.0	0.0	0.0	0.0	0.0	0.0	0.0	0.0
0.0	0.0	0.0	0.0	0.0	0.0	0.0	0.0
0.0	0.0	0.0	0.0	0.0	0.0	0.0	0.0
0.0	0.0	0.0	0.0	0.0	0.0	0.0	0.0
0.0	0.0	0.0	0.0	0.0	0.0	0.0	0.0
0.0	0.0	0.0	0.0	0.0	0.0	0.0	0.0
0.0	0.0	0.0	0.0	0.0	0.0	0.0	0.0
0.0	0.0	0.0	0.0	0.0	0.0	0.0	0.0
0.0	0.0	0.0	0.0	0.0	0.0	0.0	0.0
0.0	0.0	0.0	0.0	0.0	0.0	0.0	0.0
0.0	0.0	0.0	0.0	0.0	0.0	0.0	0.0
0.0	0.0	0.0	0.0	0.0	0.0	0.0	0.0
0.0	0.0	0.0	0.0	0.0	0.0	0.0	0.0
0.0	0.0	0.0	0.0	0.0	0.0	0.0	0.0
0.0	0.0	0.0	0.0	0.0	0.0	0.0	0.0
0.0	0.0	0.0	0.0	0.0	0.0	0.0	0.0
0.0	0.0	0.0	0.0	0.0	0.0	0.0	0.0
0.0	0.0	0.0	0.0	0.0	0.0	0.0	0.0
0.0	0.0	0.0	0.0	0.0	0.0	0.0	0.0
0.0	0.0	0.0	0.0	0.0	0.0	0.0	0.0
0.0	0.0	0.0	0.0	0.0	0.0	0.0	0.0
0.0	0.0	0.0	0.0	0.0	0.0	0.0	0.0
0.0	0.0	0.0	0.0	0.0	0.0	0.0	0.0
0.0	0.0	0.0	0.0	0.0	0.0	0.0	0.0
0.0	0.0	0.0	0.0	0.0	0.0	0.0	0.0
0.0	0.0	0.0	0.0	0.0	0.0	0.0	0.0
0.0	0.0	0.0	0.0	0.0	0.0	0.0	0.0
0.0	0.0	0.0	0.0	0.0	0.0	0.0	0.0
0.0	0.0	0.0	0.0	0.0	0.0	0.0	0.0
0.0	0.0	0.0	0.0	0.0	0.0	0.0	0.0
0.0	0.0	0.0	0.0	0.0	0.0	0.0	0.0
0.0	0.0	0.0	0.0	0.0	0.0	0.0	0.0
0.0	0.0	0.0	0.0	0.0	0.0	0.0	0.0
0.0	0.0	0.0	0.0	0.0	0.0	0.0	0.0
0.0	0.0	0.0	0.0	0.0	0.0	0.0	0.0
0.0	0.0	0.0	0.0	0.0	0.0	0.0	0.0
0.0	0.0	0.0	0.0	0.0	0.0	0.0	0.0
0.0	0.0	0.0	0.0	0.0	0.0	0.0	0.0
0.0	0.0	0.0	0.0	0.0	0.0	0.0	0.0
0.0	0.0	0.0	0.0	0.0	0.0	0.0	0.0
0.0	0.0	0.0	0.0	0.0	0.0	0.0	0.0
0.0	0.0	0.0	0.0	0.0	0.0	0.0	0.0
0.0	0.0	0.0	0.0	0.0	0.0	0.0	0.0
0.0	0.0	0.0	0.0	0.0	0.0	0.0	0.0
0.0	0.0	0.0	0.0	0.0	0.0	0.0	0.0
0.0	0.0	0.0	0.0	0.0	0.0	0.0	0.0

0.0	0.0	0.0	0.0	0.0	0.0	0.0	0.0
0.0	0.0	0.0	0.0	0.0	0.0	0.0	0.0
0.0	0.0	0.0	0.0	0.0	0.0	0.0	0.0
0.0	0.0	0.0	0.0	0.0	0.0	0.0	0.0
0.0	0.0	0.0	0.0	0.0	0.0	0.0	0.0
0.0	0.0	0.0	0.0	0.0	0.0	0.0	0.0
0.0	0.0	0.0	0.0	0.0	0.0	0.0	0.0
0.0	0.0	0.0	0.0	0.0	0.0	0.0	0.0
0.0	0.0	0.0	0.0	0.0	0.0	0.0	0.0
0.0	0.0	0.0	0.0	0.0	0.0	0.0	0.0
0.0	0.0	0.0	0.0	0.0	0.0	0.0	0.0
0.0	0.0	0.0	0.0	0.0	0.0	0.0	0.0
0.0	0.0	0.0	0.0	0.0	0.0	0.0	0.0
0.0	0.0	0.0	0.0	0.0	0.0	0.0	0.0
0.0	0.0	0.0	0.0	0.0	0.0	0.0	0.0
0.0	0.0	0.0	0.0	0.0	0.0	0.0	0.0
0.0	0.0	0.0	0.0	0.0	0.0	0.0	0.0
0.0	0.0	0.0	0.0	0.0	0.0	0.0	0.0
0.0	0.0	0.0	0.0	0.0	0.0	0.0	0.0
0.0	0.0	0.0	0.0	0.0	0.0	0.0	0.0
0.0	0.0	0.0	0.0	0.0	0.0	0.0	0.0
0.0	0.0	0.0	0.0	0.0	0.0	0.0	0.0
0.0	0.0	0.0	0.0	0.0	0.0	0.0	0.0
0.0	0.0	0.0	0.0	0.0	0.0	0.0	0.0
0.0	0.0	0.0	0.0	0.0	0.0	0.0	0.0
0.0	0.0	0.0	0.0	0.0	0.0	0.0	0.0
0.0	0.0	0.0	0.0	0.0	0.0	0.0	0.0
0.0	0.0	0.0	0.0	0.0	0.0	0.0	0.0
0.0	0.0	0.0	0.0	0.0	0.0	0.0	0.0
0.0	0.0	0.0	0.0	0.0	0.0	0.0	0.0
0.0	0.0	0.0	0.0	0.0	0.0	0.0	0.0
0.0	0.0	0.0	0.0	0.0	0.0	0.0	0.0
0.0	0.0	0.0	0.0	0.0	0.0	0.0	0.0
0.0	0.0	0.0	0.0	0.0	0.0	0.0	0.0
0.0	0.0	0.0	0.0	0.0	0.0	0.0	0.0
0.0	0.0	0.0	0.0	0.0	0.0	0.0	0.0
0.0	0.0	0.0	0.0	0.0	0.0	0.0	0.0
0.0	0.0	0.0	0.0	0.0	0.0	0.0	0.0
0.0	0.0	0.0	0.0	0.0	0.0	0.0	0.0
0.0	0.0	0.0	0.0	0.0	0.0	0.0	0.0
0.0	0.0	0.0	0.0	0.0	0.0	0.0	0.0
0.0	0.0	0.0	0.0	0.0	0.0	0.0	0.0
0.0	0.0	0.0	0.0	0.0	0.0	0.0	0.0
0.0	0.0	0.0	0.0	0.0	0.0	0.0	0.0
0.0	0.0	0.0	0.0	0.0	0.0	0.0	0.0
0.0	0.0	0.0	0.0	0.0	0.0	0.0	0.0
0.0	0.0	0.0	0.0	0.0	0.0	0.0	0.0

0.0	0.0	0.0	0.0	0.0	0.0	0.0	0.0
0.0	0.0	0.0	0.0	0.0	0.0	0.0	0.0
0.0	0.0	0.0	0.0	0.0	0.0	0.0	0.0
0.0	0.0	0.0	0.0	0.0	0.0	0.0	0.0
0.0	0.0	0.0	0.0	0.0	0.0	0.0	0.0
0.0	0.0	0.0	0.0	0.0	0.0	0.0	0.0
0.0	0.0	0.0	0.0	0.0	0.0	0.0	0.0
0.0	0.0	0.0	0.0	0.0	0.0	0.0	0.0
0.0	0.0	0.0	0.0	0.0	0.0	0.0	0.0
0.0	0.0	0.0	0.0	0.0	0.0	0.0	0.0
0.0	0.0	0.0	0.0	0.0	0.0	0.0	0.0
0.0	0.0	0.0	0.0	0.0	0.0	0.0	0.0
0.0	0.0	0.0	0.0	0.0	0.0	0.0	0.0
0.0	0.0	0.0	0.0	0.0	0.0	0.0	0.0
0.0	0.0	0.0	0.0	0.0	0.0	0.0	0.0
0.0	0.0	0.0	0.0	0.0	0.0	0.0	0.0
0.0	0.0	0.0	0.0	0.0	0.0	0.0	0.0
0.0	0.0	0.0	0.0	0.0	0.0	0.0	0.0
0.0	0.0	0.0	0.0	0.0	0.0	0.0	0.0
0.0	0.0	0.0	0.0	0.0	0.0	0.0	0.0
0.0	0.0	0.0	0.0	0.0	0.0	0.0	0.0
0.0	0.0	0.0	0.0	0.0	0.0	0.0	0.0
0.0	0.0	0.0	0.0	0.0	0.0	0.0	0.0
0.0	0.0	0.0	0.0	0.0	0.0	0.0	0.0
0.0	0.0	0.0	0.0	0.0	0.0	0.0	0.0
0.0	0.0	0.0	0.0	0.0	0.0	0.0	0.0
0.0	0.0	0.0	0.0	0.0	0.0	0.0	0.0
0.0	0.0	0.0	0.0	0.0	0.0	0.0	0.0
0.0	0.0	0.0	0.0	0.0	0.0	0.0	0.0
0.0	0.0	0.0	0.0	0.0	0.0	0.0	0.0
0.0	0.0	0.0	0.0	0.0	0.0	0.0	0.0
0.0	0.0	0.0	0.0	0.0	0.0	0.0	0.0
0.0	0.0	0.0	0.0	0.0	0.0	0.0	0.0
0.0	0.0	0.0	0.0	0.0	0.0	0.0	0.0
0.0	0.0	0.0	0.0	0.0	0.0	0.0	0.0
0.0	0.0	0.0	0.0	0.0	0.0	0.0	0.0
0.0	0.0	0.0	0.0	0.0	0.0	0.0	0.0
0.0	0.0	0.0	0.0	0.0	0.0	0.0	0.0
0.0	0.0	0.0	0.0	0.0	0.0	0.0	0.0
0.0	0.0	0.0	0.0	0.0	0.0	0.0	0.0
0.0	0.0	0.0	0.0	0.0	0.0	0.0	0.0
0.0	0.0	0.0	0.0	0.0	0.0	0.0	0.0
0.0	0.0	0.0	0.0	0.0	0.0	0.0	0.0
0.0	0.0	0.0	0.0	0.0	0.0	0.0	0.0
0.0	0.0	0.0	0.0	0.0	0.0	0.0	0.0
0.0	0.0	0.0	0.0	0.0	0.0	0.0	0.0
0.0	0.0	0.0	0.0	0.0	0.0	0.0	0.0

0.0	0.0	0.0	0.0	0.0	0.0	0.0	0.0
0.0	0.0	0.0	0.0	0.0	0.0	0.0	0.0
0.0	0.0	0.0	0.0	0.0	0.0	0.0	0.0
0.0	0.0	0.0	0.0	0.0	0.0	0.0	0.0
0.0	0.0	0.0	0.0	0.0	0.0	0.0	0.0
0.0	0.0	0.0	0.0	0.0	0.0	0.0	0.0
0.0	0.0	0.0	0.0	0.0	0.0	0.0	0.0
0.0	0.0	0.0	0.0	0.0	0.0	0.0	0.0
0.0	0.0	0.0	0.0	0.0	0.0	0.0	0.0
0.0	0.0	0.0	0.0	0.0	0.0	0.0	0.0
0.0	0.0	0.0	0.0	0.0	0.0	0.0	0.0
0.0	0.0	0.0	0.0	0.0	0.0	0.0	0.0
0.0	0.0	0.0	0.0	0.0	0.0	0.0	0.0
0.0	0.0	0.0	0.0	0.0	0.0	0.0	0.0
0.0	0.0	0.0	0.0	0.0	0.0	0.0	0.0
0.0	0.0	0.0	0.0	0.0	0.0	0.0	0.0
0.0	0.0	0.0	0.0	0.0	0.0	0.0	0.0
0.0	0.0	0.0	0.0	0.0	0.0	0.0	0.0
0.0	0.0	0.0	0.0	0.0	0.0	0.0	0.0
0.0	0.0	0.0	0.0	0.0	0.0	0.0	0.0
0.0	0.0	0.0	0.0	0.0	0.0	0.0	0.0
0.0	0.0	0.0	0.0	0.0	0.0	0.0	0.0
0.0	0.0	0.0	0.0	0.0	0.0	0.0	0.0
0.0	0.0	0.0	0.0	0.0	0.0	0.0	0.0
0.0	0.0	0.0	0.0	0.0	0.0	0.0	0.0
0.0	0.0	0.0	0.0	0.0	0.0	0.0	0.0
0.0	0.0	0.0	0.0	0.0	0.0	0.0	0.0
0.0	0.0	0.0	0.0	0.0	0.0	0.0	0.0
0.0	0.0	0.0	0.0	0.0	0.0	0.0	0.0
0.0	0.0	0.0	0.0	0.0	0.0	0.0	0.0
0.0	0.0	0.0	0.0	0.0	0.0	0.0	0.0
0.0	0.0	0.0	0.0	0.0	0.0	0.0	0.0
0.0	0.0	0.0	0.0	0.0	0.0	0.0	0.0
0.0	0.0	0.0	0.0	0.0	0.0	0.0	0.0
0.0	0.0	0.0	0.0	0.0	0.0	0.0	0.0
0.0	0.0	0.0	0.0	0.0	0.0	0.0	0.0
0.0	0.0	0.0	0.0	0.0	0.0	0.0	0.0
0.0	0.0	0.0	0.0	0.0	0.0	0.0	0.0
0.0	0.0	0.0	0.0	0.0	0.0	0.0	0.0
0.0	0.0	0.0	0.0	0.0	0.0	0.0	0.0
0.0	0.0	0.0	0.0	0.0	0.0	0.0	0.0
0.0	0.0	0.0	0.0	0.0	0.0	0.0	0.0
0.0	0.0	0.0	0.0	0.0	0.0	0.0	0.0
0.0	0.0	0.0	0.0	0.0	0.0	0.0	0.0
0.0	0.0	0.0	0.0	0.0	0.0	0.0	0.0
0.0	0.0	0.0	0.0	0.0	0.0	0.0	0.0
0.0	0.0	0.0	0.0	0.0	0.0	0.0	0.0
0.0	0.0	0.0	0.0	0.0	0.0	0.0	0.0

0.0	0.0	0.0	0.0	0.0	0.0	0.0	0.0
0.0	0.0	0.0	0.0	0.0	0.0	0.0	0.0
0.0	0.0	0.0	0.0	0.0	0.0	0.0	0.0
0.0	0.0	0.0	0.0	0.0	0.0	0.0	0.0
0.0	0.0	0.0	0.0	0.0	0.0	0.0	0.0
0.0	0.0	0.0	0.0	0.0	0.0	0.0	0.0
0.0	0.0	0.0	0.0	0.0	0.0	0.0	0.0
0.0	0.0	0.0	0.0	0.0	0.0	0.0	0.0
0.0	0.0	0.0	0.0	0.0	0.0	0.0	0.0
0.0	0.0	0.0	0.0	0.0	0.0	0.0	0.0
0.0	0.0	0.0	0.0	0.0	0.0	0.0	0.0
0.0	0.0	0.0	0.0	0.0	0.0	0.0	0.0
0.0	0.0	0.0	0.0	0.0	0.0	0.0	0.0
0.0	0.0	0.0	0.0	0.0	0.0	0.0	0.0
0.0	0.0	0.0	0.0	0.0	0.0	0.0	0.0
0.0	0.0	0.0	0.0	0.0	0.0	0.0	0.0
0.0	0.0	0.0	0.0	0.0	0.0	0.0	0.0
0.0	0.0	0.0	0.0	0.0	0.0	0.0	0.0
0.0	0.0	0.0	0.0	0.0	0.0	0.0	0.0
0.0	0.0	0.0	0.0	0.0	0.0	0.0	0.0
0.0	0.0	0.0	0.0	0.0	0.0	0.0	0.0
0.0	0.0	0.0	0.0	0.0	0.0	0.0	0.0
0.0	0.0	0.0	0.0	0.0	0.0	0.0	0.0
0.0	0.0	0.0	0.0	0.0	0.0	0.0	0.0
0.0	0.0	0.0	0.0	0.0	0.0	0.0	0.0
0.0	0.0	0.0	0.0	0.0	0.0	0.0	0.0
0.0	0.0	0.0	0.0	0.0	0.0	0.0	0.0
0.0	0.0	0.0	0.0	0.0	0.0	0.0	0.0
0.0	0.0	0.0	0.0	0.0	0.0	0.0	0.0
0.0	0.0	0.0	0.0	0.0	0.0	0.0	0.0
0.0	0.0	0.0	0.0	0.0	0.0	0.0	0.0
0.0	0.0	0.0	0.0	0.0	0.0	0.0	0.0
0.0	0.0	0.0	0.0	0.0	0.0	0.0	0.0
0.0	0.0	0.0	0.0	0.0	0.0	0.0	0.0
0.0	0.0	0.0	0.0	0.0	0.0	0.0	0.0
[truncated: 399,887 more chars]
